# Proteomic Studies of the Mechanism of Cytotoxicity, Induced by Palytoxin on HaCaT Cells

**DOI:** 10.3390/toxins14040269

**Published:** 2022-04-10

**Authors:** Dingyuan Cheng, Bowen Deng, Qiling Tong, Siyi Gao, Boyi Xiao, Mengxuan Zhu, Ziyu Ren, Lianghua Wang, Mingjuan Sun

**Affiliations:** Department of Biochemistry and Molecular Biology, College of Basic Medical Sciences, Naval Medical University, Shanghai 200433, China; dingyuancheng2022@163.com (D.C.); dengbwgsy9373@gmail.com (B.D.); tongql244790@163.com (Q.T.); nicecanny@sina.com (S.G.); cnshekinah@163.com (B.X.); zhumx0508@163.com (M.Z.); renziyup@163.com (Z.R.)

**Keywords:** palytoxin, proteomic analysis, MAPK, ferroptosis

## Abstract

Palytoxin (PLTX) is a polyether marine toxin isolated from sea anemones. It is one of the most toxic nonprotein substances, causing many people to be poisoned every year and to die in severe cases. Despite its known impact on Na^+^,K^+^-ATPase, much still remains unclear about PLTX’s mechanism of action. Here, we tested different concentrations of PLTX on HaCaT cells and studied its distributions in cells, its impact on gene expression, and the associated pathways via proteomics combined with bioinformatics tools. We found that PLTX could cause ferroptosis in HaCaT cells, a new type of programmed cell death, by up-regulating the expression of VDAC3, ACSL4 and NCOA4, which lead to the occurrence of ferroptosis. PLTX also acts on the MAPK pathway, which is related to cell apoptosis, proliferation, division and differentiation. Different from its effect on ferroptosis, PLTX down-regulates the expression of ERK, and, as a result, the expressions of MAPK1, MAP2K1 and MAP2K2 are also lower, affecting cell proliferation. The genes from these two mechanisms showed interactions, but we did not find overlap genes between the two. Both ferroptosis and MAPK pathways can be used as anticancer targets, so PLTX may become an anticancer drug with appropriate modification.

## 1. Introduction

Palytoxin (PLTX) is one of the most powerful natural nonprotein toxins; it was first characterized in 1971 from zoanthid corals belonging to the Palythoa genus [1]. Later, PLTX and analogues were isolated from dinoflagellates of the Ostreopsis genus [2], as well as from other zoanthid corals [3] and cyanobacteria [4]. The molecular weight of PLTX ranges from 2659 to 2680 Da, depending on the Palythoa species from which it is obtained [5,6]. It has a long polyhydroxyl and partially unsaturated aliphatic skeleton containing 64 chiral centers and asymmetric centers [7,8]. Its chemical structure was elucidated by two independent groups in 1981 and was first fully synthesized in 1994 [9,10].

Palytoxin-group toxins exert their powerful biological activity by altering the ion homeostasis mechanism in excitable and non-excitable tissues. The main target is the Na^+^,K^+^-ATPase [11]. For example, Marco Pelin’s work showed that PLTX can bind to Na^+^,K^+^-ATPase in the cell membrane [12]. This toxin has been reported as a potent tumor promoter and cytotoxic molecule that leads to actin filament distortion and triggers cell death or apoptosis [13]. In addition, PLTX has been shown to alter gene expression in mouse keratinocytes. PLTX stimulates an increase in mRNA for the metalloproteinase MMP-13, a carcinogenic enzyme. At the same time, it can stimulate c-FOS to bind to the active protein AP-1 site, thus promoting the initiation of the MMP-13 gene [14].

PLTX is highly toxic to mammals, and its acute toxicity depends on the route of administration, with paralytic toxins produced intravenously being the most toxic [15]. The LD50 of PLTX after intraperitoneal injection is 9 × 10^−10^ mol/kg for rabbits and 1.8 × 10^−9^ mol/kg for mice [16]. Because of the high toxicity of PLTX to animals and its reported involvement in human poisoning, EFSA recommends that PLTX be regulated with an upper limit of 1 × 10^−5^ mol/kg for shellfish meat [17]. Inhalation of PLTX aerosols by beachgoers in Italy can cause a runny nose, cough, fever and bronchial constriction, often requiring hospital treatment [18]. PLTX is also a carcinogen [19].

However, much remains unclear about its molecular mechanism [14]. PLTX is likely to function via other cellular receptors when associated with skin tumors [20]. PLTX seems to possess preferential toxicity for head and neck cancer cells and xenografts when the expression of c-Jun N-terminal kinase-3 (JNK3) is repressed [21]. In this study, we explored the mechanism of PLTX and its possible roles by checking its distribution in cells, its impact on cell morphology and biological behavior, and its involvement in pathways using a proteomics method and bioinformatics tools. Our study can provide insight for the rescue of PLTX poisoning and contribute new knowledge for drug development using PLTX.

## 2. Results

### 2.1. PLTX Causes Cell Death

**Figure 1:**
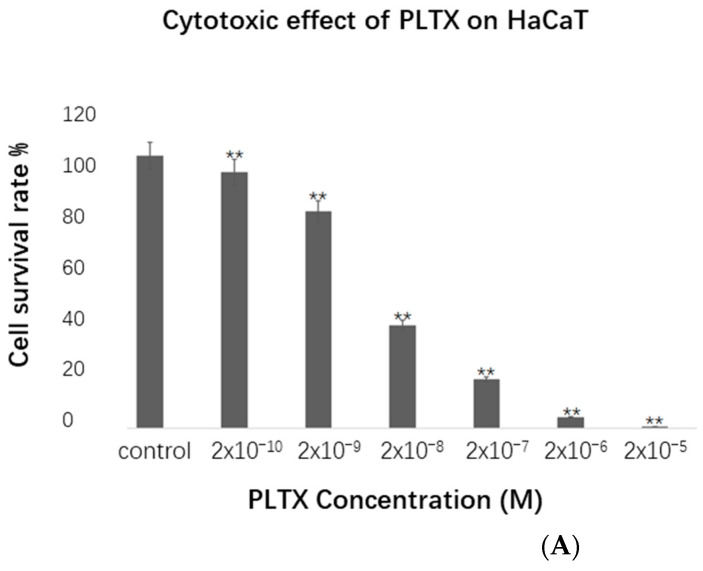

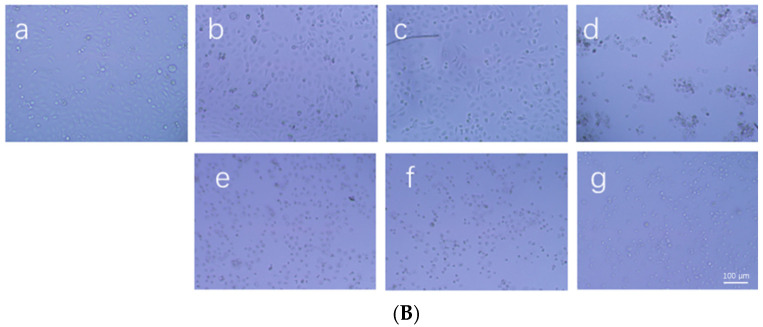
Cytotoxicity assay. (**A**) The histogram shows the cell survival rate at different PLTX concentrations, and the cell survival rate was detected by the CCK-8 method. All experiments were repeated at least three times, and the cell survival rate decreased with increasing PLTX concentration. The values are presented as the means ± SEM. ** *p* < 0.01 vs. the control group. (**B**) Cells were treated with different concentrations of PLTX (**a**–**g**: 0, 2 × 10^−^^10^, 2 × 10^−^^9^, 2 × 10^−^^8^, 2 × 10^−^^7^, 2 × 10^−^^6^, 2 × 10^−^^5^ M) for 24 h. A 20× magnification objective was used. Scale bar: 100 µm. Representative images of three independent experiments are presented.

PLTX has strong cytotoxicity. In order to evaluate the specific toxicity of PLTX on HaCaT cells, the CCK-8 method was used. Cell survival rate was assessed after 24 h of treatment with different concentrations of PLTX. As expected, the cell survival rate decreased significantly with increasing PLTX concentration (Figure 1A). The IC_50_ value calculated via SPSS was 1.3 × 10^−8^ M. After application of PLTX, the morphology of cells changed, and intercellular connections disappeared (Figure 1B). In order to study the difference in cell protein expression after PLTX treatment, the concentration of 2 × 10^−8^ M was selected as the treatment group for subsequent experiments.

### 2.2. PLTX can Enter Cells and Bind to Intracellular Molecules

**Figure 2:**
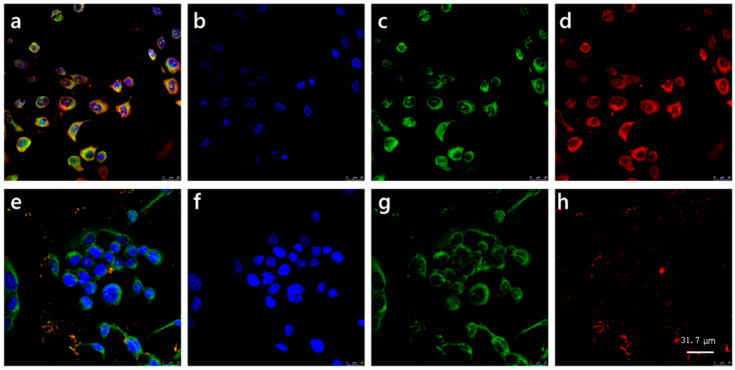
Immunolocalization of PLTX in HaCaT cells. Cells were exposed to 2 × 10^−8^ M PLTX. PLTX is shown in red by 5′-Cy5-labeled aptamer (red fluorescence). The nuclei are shown in blue by DAPI (blue fluorescence). The cell membrane is shown in green by DiO (green fluorescence). A 63× magnification objective was used. Scale bar: 31.7 µm. The top panel was treated with specific aptamer to show the subcellular distribution of PLTX. The bottom panel shows controls with random aptamer. (**a**,**e**) Co-localization of the nucleus, cell membrane and PLTX. (**b**,**f**) The nucleus only. (**c**,**g**) The cell membrane only. (**d**,**h**) Distribution of PLTX labeled by different aptamers in cells: PLTX-specific aptamer (**d**) and random aptamer (**h**). Representative images of three independent experiments are presented.

To verify the distribution of PLTX in cells, immunofluorescence chemistry combined with laser confocal microscopy was performed as a qualitative analysis. We chose the PLTX-13 aptamer with a 5′-Cy5 label to mark PLTX, which was firstly reported by Gao et al. and proved to have high affinity and specificity for PLTX [22]. More information about the aptamer is available in the Supplementary Materials (Supplementary Table S1). As shown in the top panel of Figure 2, the red-fluorescent aptamer specifically binding to PLTX was present on the cell membrane and in the nucleus, and the aptamer that entered the nucleus was superposed with the blue-fluorescent nucleus, presenting a purple color. The results showed that the PLTX could not only distribute on the cell membrane but also enter the nucleus. This specificity of PLTX-13 aptamer targeting to PLTX can be confirmed by comparing these results with those for the control group in the bottom panel of Figure 2. The 5′-Cy5-labeled control aptamer with random sequence was applied to exclude any background caused by random nucleic acids binding to PLTX. As shown in Figure 2e,h, the control aptamer with red fluorescence was scattered randomly and could not bind to the PLTX.

### 2.3. PLTX Can Cause Programmed Cell Death

**Figure 3:**
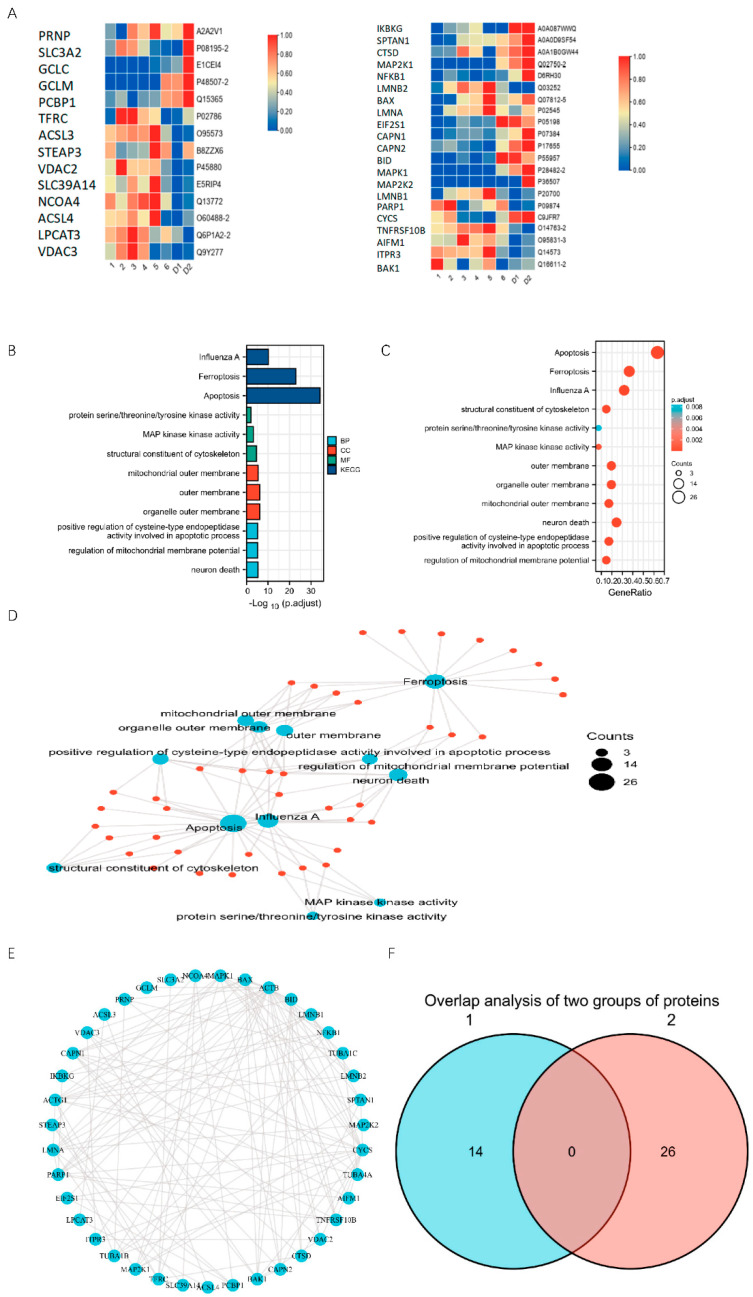
Effects of PLTX on HaCaT cells at the proteomic level. (**A**) Heat map presenting the results of cluster analysis using the Euclidean distance and hierarchical algorithm in each group; (**B**) KEGG and GO pathway analysis: the ordinate in the figure gives the name of the pathway with differentially expressed proteins involved, and the abscissa represents the number of differentially expressed proteins involved in this pathway; (**C**) The GO and KEGG pathways were enriched in differentially expressed proteins (DEPs). The X-axis gives the enrichment coefficient. The *Y*-axis labels are the pathway names. The color represents the p-value (high: blue; low: orange), where the lower the p-value, the more significant the enrichment. The dot size represents the number of DEPs. The larger the dot, the larger the number. The gene ratio refers to the value of the enrichment factor, that is, the ratio of the foreground value (number of DEPs) to the background value (total proteins). The higher the value, the more significant the enrichment; (**D**) Visualization of GO and KEGG enrichment pathways; (**E**) Network of protein–protein interactions; (**F**) Overlap analysis of the two groups of proteins. Group 1 corresponds to the left-hand side of (**A**), and Group 2 corresponds to the right-hand side of (**A**).

To further understand the mechanism of PLTX, we explored it through proteomics combined with bioinformatics. MaxQuant software (BGI, China) was used for qualitative analysis of proteins. DEPs were defined as those with a fold change of degree >1.2 and *p* < 0.05. Groups 1, 2, 3, 4, 5 and 6 were the experimental groups, with PLTX concentration from high to low, and D1 and D2 were the control groups. The concentration of PLTX in the sixth group was close to zero, so the protein expression was expected to be similar to that of the control group. We found that in the experimental groups, the expression of VDAC3, ACLS4 and NCO4 was significantly up-regulated, while the expression of MAPK1, MAP2K1 and MAP2K2 was down-regulated. The Euclidean distance was used for cluster analysis, and a hierarchical algorithm was used to display the expression of each group of proteins (Figure 3A). We also found changes in the expression of other proteins (Supplementary Figure S1). Please refer to the Supplementary Materials for all protein expression differences (Supplementary Table S2).

GO and KEGG enrichment analysis showed that proteins in “Ferroptosis” and “Apoptosis” were significantly altered in the PLTX-treated group compared with the control group (Figure 3B,C). Figure 3D shows the connection between each pathway. The larger the point is, the more proteins were enriched in this pathway.

These DEPs were exported into the STRING database and Cytoscape Version 3.9.0 software to create a landscape of protein–protein interactions. STRING analysis showed that many of these proteins are related to each other and may interact with each other (Figure 3E). However, there was no overlap between the two groups of proteins (Figure 3F).

### 2.4. PLTX Resulted in Increased Expression of VDAC3

**Figure 4:**
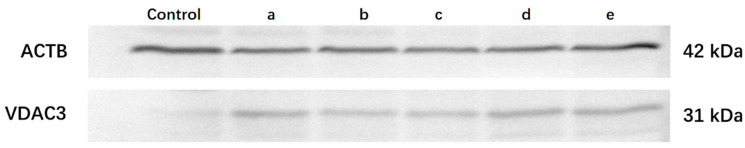
VDAC3 expression increased significantly after cells were treated with PLTX for 24 h. **a**, **b**, **c**, **d** and **e** were treated with PLTX at 2 × 10^−6^ M, 2 × 10^−7^ M, 2 × 10^−8^ M, 2 × 10^−9^ M and 2 × 10^−10^ M, respectively. There was almost no expression of VDAC3 in the control group, while expression was significantly increased in the experimental groups. For gray scale analysis, see the Supplementary Materials (Supplementary materials Figure S2). Representative images of three independent experiments are presented.

To verify whether PLTX really promotes ferroptosis in cells, we selected the expression products of VDAC3 for verification. VDVC3, a member of the eukaryotic mitochondrial pore protein family, forms a channel through the outer membrane of mitochondria that allows the diffusion of small hydrophilic molecules. VDAC3 regulates various cellular processes by regulating molecular exchange between cell compartments. It can regulate the permeability of the mitochondrial outer membrane and induce ferroptosis. After treatment with PLTX for 24 h, we extracted the protein and checked its expression level by western blot. Compared to the reference protein, ACTB, the expression level of VDAC3 in PLTX-treated cells was significantly increased (Figure 4). This is consistent with our proteomic results shown above.

### 2.5. PLTX Can Increase Intracellular ROS Concentration

**Figure 5:**
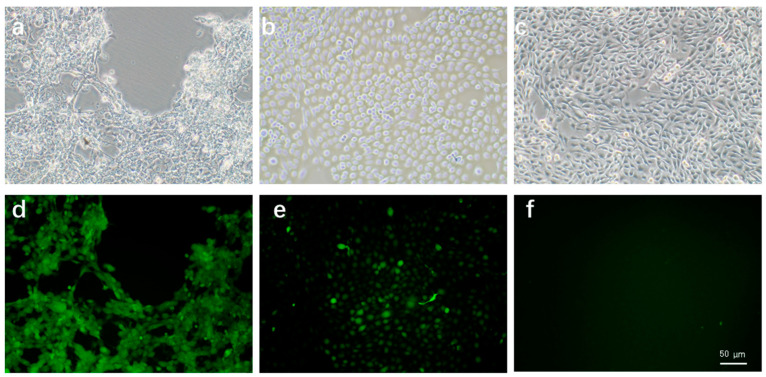
Effect of PLTX treatment on intracellular ROS levels. (**a**,**d**) Treated with 2 × 10^−8^ M PLTX. (**b**,**e**) Positive control treated with 50 μg/mL Rosup. (**c**,**f**) Negative controls untreated with Rosup. Here, (**a**–**c**) are bright field images and (**d**–**f**) are fluorescence images. A 40× magnification objective was used. Scale bar: 50 µm. Representative images of three independent experiments are presented.

Through the above experiments, we preliminarily concluded that PLTX can promote ferroptosis. Lipid peroxidation and the accumulation of intracellular reactive oxygen species lead to cell ferroptosis. To further determine the relationship between PLTX and ferroptosis, we qualitatively detected intracellular reactive oxygen species (ROS) levels after PLTX treatment. From the experimental results, the fluorescence intensity of cells in the experimental group was higher than that of cells in the negative control group, which was similar to that of cells in the positive control group, and the level of intracellular reactive oxygen species increased (Figure 5). This further demonstrates the role of PLTX in promoting cell ferroptosis.

## 3. Discussion

In this study, we used proteomics combined with bioinformatics analysis to study the mechanism of the toxin PLTX in cells. Our research demonstrates that: (1) PLTX promotes apoptosis in cells, (2) PLTX is likely to function as a promoter of ferroptosis, acting on the ferroptosis signaling pathway and causing cell apoptosis, and (3) PLTX affects the MAPK pathway, which is associated with ferroptosis.

Studies have shown that PLTX acts on Na^+^,K^+^-ATPase, as well as different cell signaling pathways, affecting cell survival, apoptosis and carcinogenesis [23]. PLTX has been shown to have the effect of inducing apoptosis. After PLTX treatment, the ion concentration in cells is changed, and some pathway proteins in cells are also changed [24]. The voltage-dependent anion channel (VDAC) is a hole located in the outer membrane of mitochondria. It allows for the entry and exit of a large number of ions and metabolites between the cytoplasm and mitochondria. VDAC2 and VDAC3 have been shown to jointly promote A375 cell induced ferroptosis [25]. VDAC3 is a marker of redox signaling in the mitochondrial intermembrane space [26].

We observed several genes of interest that were expressed differently in the experiment group as compared to the control group. The first one is ACSL4, with increased gene expression in the experimental group. ACSL4 is involved in the biosynthesis and remodeling of PUFA-PEs in cell membranes. The loss of these gene products depletes the substrate for lipid peroxidation and increases resistance to ferroptosis. Increased ACSL4 expression promotes cell ferroptosis [27]. Ferroptosis disease is a form of regulated cell death characterized by the accumulation of iron-dependent lipid hydroperoxides to lethal levels. Emerging evidence also suggests that ferroptosis might serve a tumor suppressor function in removing cells that lack access to critical nutrients in their environment, or that have been compromised by infection or environmental stress [28]. Increased ACLS4 expression triggered by PLTX in the environment may cause cells to undergo programmed death. Many drugs have been found not only to initiate apoptosis, but also to induce ferroptosis and prevent cancer growth [29,30]. Ames testing of PLTX was negative, indicating that PLTX does not have a mutagenic effect [15]. PLTX has been described as a skin tumor promoter that stimulates a variety of cellular responses [20]. However, the basis for classifying drugs as tumor promoters is conditional and only in the context of a two-stage (or multi-stage) model approach [31]. PLTX alone does not cause cancer but it kills cancer cells [21].

Another gene of interest with increased expression in the experimental group is NCOA4. NCOA4 is required for ferritin delivery to the lysosome, and the inability of NCOA4-deficient cells to degrade ferritin results in reduced intracellular bioavailability of iron [32]. Within cells, specific metabolic pathways of glutamine breakdown and components that mediate transferrin input are required for ferroptosis [33]. NCOA4 is a selective cargo receptor for selective autophagy turnover of ferritin in ferroptosis. Similarly, gene inhibition of NCOA4 inhibits ferritin degradation and inhibits ferroptosis. In contrast, overexpression of NCOA4 increases ferritin degradation and promotes ferroptosis [34]. In our study, the identification of two key molecules involved in ferroptosis indicates that PLTX is involved in, and a promoter of, ferroptosis.

Different from ACSL4 and NCOA4, our study showed that the expression levels of MAPK1, MAP2K1 and MAP2K2 decreased in the presence of PLTX. These genes are involved in the ERK pathway, which is one of three subfamilies for the mitogen-activated protein kinase (MAPK) pathway besides JNK and P38-MAPK. MAPK, commonly referred to as the Ras-Raf-MEK-ERK signaling cascade, functions to transmit upstream signals to its downstream effectors to regulate physiological processes such as cell proliferation, differentiation, survival and death [35]. Cells are constantly exposed to various environmental pressures and respond to the environment. Members of the MAPK family are critical for cell maintenance in many signaling pathways that respond to stress. In particular, ERK is important for cell survival [36]. ERK overexpression can induce the regulation of anti-apoptotic molecules, which is associated with tumor drug resistance [37]. Activated ERK phosphorylates a wide variety of substrates, from kinases to transcription factors, and because of its fairly broad substrate recognition properties, it has been targeted as a key kinase for controlling a large number of cellular processes and maintaining cellular homeostasis. Therefore, inhibitors that target the MAPK pathway are thought to have antitumor effects [35]. However, the development of selective inhibitors of ERK1 and ERK2 has been limited. RAF and MEK inhibitors have been commonly developed because ERK is the only known downstream target of MEK, and ERK inhibitors do not seem to confer additional benefits compared to MEK inhibitors. Nevertheless, there has been increased interest in ERK inhibition recently. One study has shown that tumor resistance to RAF and MEK inhibitors often involves the restoration of ERK signaling pathways, suggesting that ERK inhibitors may reduce tumor resistance [38]. PLTX may serve as an antitumor agent by inhibiting ERK signaling.

The ERK signaling pathway is related to cellular oxidative stress in that the pathway is inactivated after cell hypoxia and reoxygenation [39]. Interestingly, ferroptosis is also associated with oxidative stress in cells [33]. One related nutrient transporter to this process that is frequently overexpressed in human cancers is the cystine/glutamate antiporter solute carrier family 7 member 11 (SLC7A11). SLC7A11 promotes cystine uptake and glutathione biosynthesis, resulting in protection from oxidative stress and ferroptotic cell death [40]. ERK-dependent signaling is required for RAS-dependent ferroptosis in solid cancer cells [41]. PLTX has also been found to stimulate JNK activation through a pathway involving ion flux, and inhibition of the JNK pathway in acute myeloid leukemia (AML) reduces its sensitivity to ferroptosis [41,42]. The effect of PLTX on initiating keratinocytes is significantly different from its effect on other cell types, with PLTX leading to the activation of ERK expression in 308 cells [20] and the inhibition of ERK expression in epidermal cells.

We believe that our study is useful in understanding the molecular mechanisms of PLTX, but, inevitably, there are limitations. First, the study was limited to focusing on only one cell line’s response to PLTX; more cell lines are needed in order to have a complete understanding of PLTX’s effects. Second, we detected the pathway of PLTX through individual studies, but the crosstalk between these pathways is not clear. Third, further studies are needed for a better understanding of the molecular mechanism of PLTX-induced ferroptosis. Fourthly, some experiments were qualitative analyses without quantitative analysis.

## 4. Conclusions

In summary, we demonstrated that PLTX may act on the MAPK pathway and is involved in ferroptosis in cells. PLTX may act through the ERK pathway, a subfamily of MAPK, making PLTX a potential anticancer drug with a potential anticancer mechanism. However, other experimental data are needed to confirm this.

## 5. Materials and Methods

### 5.1. Cell Culture

HaCaT cells were purchased from China Center For Type Culture Collection (Wuhan, China, GDC0106) and cultured in DMEM (Gibco, New York, NY, USA) with 10% fetal bovine serum (Pan-Biotece, Adenbach, Germany) and 1% penicillin/streptomycin (Sigma-Aldrich, St. Louis, MO, USA). Cells were grown in 5% carbon dioxide humidified air at 37 °C. The cell culture medium was replaced every two days, and a generation was defined after transmission every three days.

### 5.2. Cell Viability Assay

The Cell Counting Kit-8 (CCK-8, Dojindo, Kyushu Island, Japan) assay was used to measure cell viability. A 100 µL (5000 cells/100 µL) cell suspension was configured in 96-well plates. The plates were cultured for 24 h at 37 °C and 5% CO_2_. Next, PLTX (Wako Pure Chemical Industries, Osaka, Japan) in different concentrations was added to the culture plates. The culture plates were incubated for 24 h. Then, the original culture medium was discarded, 100 μL of medium containing 10% CCK-8 reagent was added to each well, and the plates were incubated for 2 h at 37 °C. Finally, the absorbance at 450 nm was measured using a Thermo Scientific Microplate Reader (Thermo, Waltham, MA, USA).

### 5.3. Immunofluorescence Chemistry Combined with Laser Confocal Microscopy

Cells were seeded on coverslips in 12-well plates, and the density of planking cells was about 2 × 10^4^ cells/mL. After a period of culture for 24 h, cells were treated with 2 × 10^−8^ M PLTX for 10 min, followed by fixation for 20 min with 4% multi-local methanol (PFA). The cells were incubated with permeable solution of 0.3% TritonX-100 for 30 min. Then, cells were blocked with 5% BSA Quick Block (Beyotime, Shanghai, China) for 10 min. The fixed cells were covered with 1 µM red fluorescent 5′-Cy5-labeled specific aptamer (Sangon Biotech, Shanghai, China) or 1 µM random aptamer for 30 min. Then 5 µM DiO (Beyotime, Shanghai, China) was added to stain the cell membrane for 20 min. Next, 5 μg/mL DAPI (Beyotime, Shanghai, China) was added to counterstain the nucleus for 5 min. Finally, the coverslips were mounted on the glass slides with anti-fluorescence quenching sealing plate reagent. The cells were imaged on a single plane via laser confocal microscopy (Zeiss, Oberkochen, Germany). A 63× magnification objective was used.

### 5.4. Analysis of Protein Expression Profiles

The cells were treated with different concentrations of PLTX and divided into six groups from high to low concentration: 2 × 10^−5^, 2 × 10^−6^, 2 × 10^−7^, 2 × 10^−8^, 2 × 10^−9^, 2 × 10^−10^ M. After 24 h, SDT (4% SDS, 100 mM Tris-HCl, 1 mM DTT, pH 7.6) lysate was added and boiled at 98 °C for 10 min to achieve complete lysate and protein denaturation. The supernatant of the lysate sample was obtained after high-speed centrifugation, namely, protein lysate.

After protein quantification, 200 μg of protein solution was added to dithiothreitol (DTT) to produce a reduction reaction; the protein solution was placed in an ultrafiltration tube after reaction, all samples were added to the ultrafiltration tube, and the collection solution was discarded after centrifugation. Next, 200 μL urea (UA) was added into the ultrafiltration tube, and the collection solution after centrifugation was discarded. Then, 20 μL 1M iodoacetamide (IAA) solution was added. The samples were mixed well and reacted at room temperature for 1 h. A quantity of 100 μL UA was then added into the ultrafiltration tube, and the collection solution after centrifugation was discarded; this was repeated twice. Then, 100 μL of 50 mM ammonium bicarbonate (ABC) was added, and the collected solution was discarded after centrifugation; this was repeated twice. Then, the collection tube was updated, and 100 μL ABC was added to the ultrafiltration tube. Finally, trypsin was added at an enzyme-to-protein ratio of 1:50 (*W*/*W*), followed by enzymolysis at 37 °C for more than 16 h.

The C18 tip was slowly and repeatedly blown in 0.1% trifluoroacetic acid solution to fully bind the C18 to the polyhydrolysate. After binding, the C18 tip was washed with 0.1% trifluoroacetic acid. Finally, the polypeptide was eluted with 100 μL solution containing 50% acetonitrile and 0.1% trifluoroacetic acid. After desalting and purification, the sample was concentrated and dried in vacuum.

Protein analysis was performed via hybrid electric field track well cyclotron resonance liquid mass spectrometry (Orbitrap Elite LC-MS/MS, Thermo, Waltham, MA, USA) with a separation time of 203 min and a flow rate of 300 nL/min.

The mass spectrometry proteomics data were uploaded to the ProteomeXchange Consortium (http://proteomecentral.proteomexchange.org, accessed on 24 March 2022) via the iProX partner repository with the dataset identifier PXD032777. MaxQuant 1.5.2.8 was used for database identification and analysis. The relevant parameters are in the Supplementary material (Supplementary Table S3)

### 5.5. Differential Expression Analysis

GO-Term Finder was used to identify Gene Ontology (GO) terms that annotate lists of enriched proteins with P-value significantly less than 0.05. The Kyoto Encyclopedia of Genes and Genomes was used to analyze cell signaling pathways (http://en.wikipedia.org/wiki/KEGG, accessed on 17 August 2021). The Search Tool for the Retrieval of Interacting Genes (STRING) was used to analyze protein interaction networks (http://string-db.org/, accessed on 17 August 2021). Cytoscape version 3.9.0 was used for data visualization (http://www.cytoscape.org/, accessed on 18 August 2021).

### 5.6. Western Blot

Cells were treated with different concentrations of PLTX (2 × 10^−6^ M, 2 × 10^−7^ M, 2 × 10^−8^ M, 2 × 10^−9^ M, 2 × 10^−10^ M) and lysed in M-PER Lysis Buffer (Thermo Scientific, 78501) for Western blotting. After incubation on ice for 30 min, the supernatant protein concentration was determined using a BCA kit (Beyotime, Shanghai, China) after centrifugation at 14,000× *g* for 15 min. The total protein was boiled in SDS loading buffer (Beyotime, Shanghai, China) and separated using 5% concentrated gel and 10% separated gel, then it was transferred to PVDF membranes. The PVDF membranes were blocked with 5% BSA for 1 h and were incubated with antibody against VDAC3 (Proteintech, Chicago, IL, USA) at 4 °C overnight. Then, the membranes were washed three times in 1 × TBST (Sangon Biotech, Shanghai, China) and incubated with the corresponding HRP-conjugated anti-rabbit secondary antibodies (Beyotime, Shanghai, China). After 1 h, the membranes were washed three times in 1 × TBST and then visualized using an Immobilon Forte Western HRP substrate (Millipore, Burlington, MA, USA). The bands were quantified via Image Lab software (BIORAD, Hercules, CA, USA). The relative optical density was calculated by comparing the optical density of the target protein with that of ACTB (Proteintech, Chicago, IL, USA) proteins.

### 5.7. Reactive Oxygen Species Detection

A reactive oxygen species (ROS) detection kit (Beyotime, Shanghhai, China) was used to detect intracellular ROS. Cells were cultured in 6-well plates and treated with 2 × 10^−8^ M PLTX for 24 h. Next, the cell culture medium was replaced, and the cells were incubated in DMEM with 10 µM DCFH-DA. The plate was incubated in a 37 °C incubator for 20 min. The cells were then washed three times in serum-free cell culture medium. In the positive control group, cells were treated with 50 μg/mL Rosup, which is a kind of compound mixture that promotes the intracellular production of reactive oxygen species. Finally, a fluorescence microscope (Olympus IX71, Tokyo, Japan) was used for observation. A 40× magnification objective was used.

### 5.8. Statistical Analysis

Data are expressed as the mean ± SEM. Data between groups were compared with data of parameters (normality and equal variance passed) via one-way ANOVA (LSD) using SPSS.

## Data Availability

Data are contained within the article.

## Supplementary Materials

The following supporting information can be downloaded at: https://www.mdpi.com/article/10.3390/toxins14040269/s1, Figure S1: Cluster analysis of protein expression differences; Figure S2: Gray scale analysis of Western blot. All experiments were repeated at least 3 times. Values are expressed as average ± SEM. ** *p* < 0.01 vs. the control group. SPSS was used for one-way analysis of variance; Table S1: Kinetic parameters between PTX and selected aptamer; Table S2: Proteomic results; Table S3: Proteomics related parameters.
