# Supplementary material for: Proteomic Studies of the Mechanism of Cytotoxicity, Induced by Palytoxin on HaCaT Cells
Source: Toxins (Basel). 2022 Apr 10;14(4):269. doi: 10.3390/toxins14040269 (PMC9031313; doi:10.3390/toxins14040269)
Supplement: Supplementary file 1 [file toxins-14-00269-s001.zip › Supplementary Table S2 Proteomic results.pdf]

Protein IDs	Majority pr	Peptide co	Peptide co	Peptide co	Protein names	Gene name
P61604;B8ZZL8;B8ZZ54	P61604;B8	8;6;4;3	8;6;4;3	8;6;4;3	10 kDa heat shock protein,	HSPE1
Q15029-2;Q15029;Q15	Q15029-2;	10;10;9;4;3	9;9;8;3;2;1	9;9;8;3;2;1	116 kDa U5 small nuclear ri	EFTUD2
Q9NRX4-2;Q9NRX4	Q9NRX4-2;	1;1	1;1	1;1	14 kDa phosphohistidine ph	PHPT1
P31946-2;P31946;Q4VY	P31946-2;F	8;8;3;3;3;2	4;4;1;1;1;1	4;4;1;1;1;1	14-3-3 protein beta/alpha;1	YWHAB
P62258;P62258-2;B4DJI	P62258;P6	15;12;5;5;5	12;10;3;3;4	12;10;3;3;4	14-3-3 protein epsilon	YWHAE
Q04917;A2IDB2;F8WEB	Q04917	12;5;3;3	9;4;2;2	9;4;2;2	14-3-3 protein eta	YWHAH
P61981	P61981	10	7	7	14-3-3 protein gamma;14-3	YWHAG
P31947-2;P31947	P31947-2;F	17;17	17;17	14;14	14-3-3 protein sigma	SFN
P27348;E9PG15	P27348;E9I	12;8	8;6	8;6	14-3-3 protein theta	YWHAQ
P63104;E7EX29;E7ESK7	P63104;E7I	14;11;8;8;7	11;8;6;7;6;	10;7;6;6;5;	14-3-3 protein zeta/delta	YWHAZ
Q9C0C2;Q9C0C2-2	Q9C0C2;Q9	3;2	3;2	3;2	182 kDa tankyrase-1-bindin	TNKS1BP1
Q9NUQ2	Q9NUQ2	2	2	2	1-acyl-sn-glycerol-3-phosph	AGPAT5
P09543-2;P09543	P09543-2;F	1;1	1;1	1;1	2,3-cyclic-nucleotide 3-pho	CNP
Q16698-2;Q16698;E5RJ	Q16698-2;	7;7;3;3;3;1	7;7;3;3;3;1	7;7;3;3;3;1	2,4-dienoyl-CoA reductase,	DECR1
Q9Y6K5;F8VWK9;F8VS3	Q9Y6K5;F8	4;2;2	4;2;2	4;2;2	2-5-oligoadenylate synthase	OAS3
A0A087X2I1;P62333;H0	A0A087X2I	7;7;5;4;1;1	7;7;5;4;1;1	7;7;5;4;1;1	26S protease regulatory sub	PSMC6
P62191-2;P62191;G3V4	P62191-2;F	5;5;1	5;5;1	5;5;1	26S protease regulatory sub	PSMC1
E9PM69;R4GNH3;P1798	E9PM69;R4	5;5;5;3;3;3	5;5;5;3;3;3	5;5;5;3;3;3	26S protease regulatory sub	PSMC3
P43686-2;P43686	P43686-2;F	4;4	4;4	4;4	26S protease regulatory sub	PSMC4
P35998;A0A1W2PQS1;F	P35998;A0	10;9;5;4	10;9;5;4	10;9;5;4	26S protease regulatory sub	PSMC2
J3QQM1;P62195-2;P62	J3QQM1;P	5;5;5;4;4;4	5;5;5;4;4;4	4;4;4;3;3;3	26S protease regulatory sub	PSMC5
A0A087WW66;Q99460	A0A087WV	6;6;6;2;1;1	6;6;6;2;1;1	6;6;6;2;1;1	26S proteasome non-ATPas	PSMD1
B1AJY5;B1AJY7;O75832	B1AJY5;B1	2;2;2;1;1	2;2;2;1;1	2;2;2;1;1	26S proteasome non-ATPas	PSMD10
O00231;O00231-2;J3QF	O00231;O0	10;10;6;1	10;10;6;1	10;10;6;1	26S proteasome non-ATPas	PSMD11
O00232-2;O00232;J3KT	O00232-2;	6;6;1;1	6;6;1;1	6;6;1;1	26S proteasome non-ATPas	PSMD12
J3KNQ3;Q9UNM6;Q9UI	J3KNQ3;Q9	6;6;6;5;4;4	6;6;6;5;4;4	6;6;6;5;4;4	26S proteasome non-ATPas	PSMD13
O00487	O00487	1	1	1	26S proteasome non-ATPas	PSMD14
Q13200;Q13200-3;Q13	Q13200;Q1	12;10;8;3;2	12;10;8;3;2	12;10;8;3;2	26S proteasome non-ATPas	PSMD2
O43242-2;O43242	O43242-2;	5;5	5;5	5;5	26S proteasome non-ATPas	PSMD3
Q5VWC4;P55036;H0Y3	Q5VWC4;P	2;2;1;1;1	2;2;1;1;1	2;2;1;1;1	26S proteasome non-ATPas	PSMD4
Q15008-3;Q15008-2;Q1	Q15008-3;	4;4;4;4;3;2	4;4;4;4;3;2	4;4;4;4;3;2	26S proteasome non-ATPas	PSMD6
H3BNT7;H3BTM8;P516	H3BNT7;H3	3;3;3;1	3;3;3;1	3;3;3;1	26S proteasome non-ATPas	PSMD7
F5H5V4;F5GX23;J3KN2	F5H5V4;F5	2;2;2;2;2;1	2;2;2;2;2;1	2;2;2;2;2;1	26S proteasome non-ATPas	PSMD9
Q13442	Q13442	5	5	5	28 kDa heat- and acid-stabl	PDAP1
P82912-2;P82912	P82912-2;F	1;1	1;1	1;1	28S ribosomal protein S11,	MRPS11
A6ND22;Q9Y3D3-2;Q9Y	A6ND22;Q	1;1;1	1;1;1	1;1;1	28S ribosomal protein S16,	MRPS16
E9PE17;I3L0E3;Q9Y2R5	E9PE17;I3L	2;2;2	2;2;2	2;2;2	28S ribosomal protein S17,	MRPS17;h(
Q9Y676;A0A0G2JIC6	Q9Y676;A0	4;3	4;3	4;3	28S ribosomal protein S18b	MRPS18B
Q9Y399;Q5T8A0	Q9Y399;Q5	3;2	3;2	3;2	28S ribosomal protein S2, m	MRPS2
P82921	P82921	1	1	1	28S ribosomal protein S21,	MRPS21
G5E9W7;G5E9V5;P8265	G5E9W7;G	4;4;4;3;3;3	4;4;4;3;3;3	4;4;4;3;3;3	28S ribosomal protein S22,	MRPS22
J3QLR8;Q9Y3D9	J3QLR8;Q9	2;2	2;2	2;2	28S ribosomal protein S23,	MRPS23
P82663;E7EPW2;P8266	P82663	3;1;1;1	3;1;1;1	3;1;1;1	28S ribosomal protein S25,	MRPS25
D6RH20;G5EA06;Q925	D6RH20;G	2;2;2;2;1	2;2;2;2;1	2;2;2;2;1	28S ribosomal protein S27,	MRPS27
Q9Y2Q9;E5RFT8;E5RGC	Q9Y2Q9;E5	2;1;1;1;1;1	2;1;1;1;1;1	2;1;1;1;1;1	28S ribosomal protein S28,	MRPS28
P82909;D6R941	P82909;D6	2;1	2;1	2;1	28S ribosomal protein S36,	MRPS36
P82675	P82675	1	1	1	28S ribosomal protein S5, m	MRPS5
P82932	P82932	1	1	1	28S ribosomal protein S6, m	MRPS6

J3QQS1;J3QLS3;Q9Y2R9	J3QQS1;J3(	3;3;3;2;2	3;3;3;2;2	3;3;3;2;2	28S ribosomal protein S7, m	MRPS7
P82933	P82933	2	2	2	28S ribosomal protein S9, m	MRPS9
E9PDF2;E9PCR7;Q0221	E9PDF2;E9I	18;18;18;1	18;18;18;1	18;18;18;1	2-oxoglutarate dehydrogen	OGDH
H0Y8N7;Q9BYD6	H0Y8N7;Q9	1;1	1;1	1;1	39S ribosomal protein L1, m	MRPL1
Q9Y3B7-2;Q9Y3B7;Q9Y	Q9Y3B7-2;(	3;3;2;1;1	3;3;2;1;1	3;3;2;1;1	39S ribosomal protein L11,	MRPL11
B4DLN1;P52815	B4DLN1;P5	6;3	6;3	3;3	39S ribosomal protein L12,	MRPL12
H0YAX3;E5RJI7;Q9BYD1	H0YAX3;E5	2;2;2	2;2;2	2;2;2	39S ribosomal protein L13,	MRPL13
Q9H0U6	Q9H0U6	3	3	3	39S ribosomal protein L18,	MRPL18
C9IY40;Q5T653	C9IY40;Q5T	1;1	1;1	1;1	39S ribosomal protein L2, m	MRPL2
E7ESL0;J3KQY1;Q9NWU	E7ESL0;J3K	2;2;2;2;2	2;2;2;2;2	2;2;2;2;2	39S ribosomal protein L22,	MRPL22
Q96A35	Q96A35	2	2	2	39S ribosomal protein L24,	MRPL24
H0Y4J2;A6NHR2;S4R369	H0Y4J2;A6I	1;1;1;1	1;1;1;1	1;1;1;1	39S ribosomal protein L37,	MRPL37
Q96DV4;Q96DV4-2	Q96DV4;Q9	2;1	2;1	2;1	39S ribosomal protein L38,	MRPL38
C9JG87;Q9NYK5;Q9NYK	C9JG87;Q9	1;1;1	1;1;1	1;1;1	39S ribosomal protein L39,	MRPL39
K7EKI4;K7ELF1;K7ELQ0;	K7EKI4;K7E	1;1;1;1;1;1	1;1;1;1;1;1	1;1;1;1;1;1	39S ribosomal protein L4, m	MRPL4
H0YBU8;H0Y6Y8;B1AL0	H0YBU8;H(	2;2;2;2;2;2	2;2;2;2;2;2	2;2;2;2;2;2	39S ribosomal protein L43,	MRPL43
Q9H9J2	Q9H9J2	1	1	1	39S ribosomal protein L44,	MRPL44
E9PI78;E9PNF1;Q13405	E9PI78;E9P	1;1;1	1;1;1	1;1;1	39S ribosomal protein L49,	MRPL49
Q8N5N7-2;Q8N5N7	Q8N5N7-2;	2;2	2;2	2;2	39S ribosomal protein L50,	MRPL50
Q96EL3	Q96EL3	2	2	2	39S ribosomal protein L53,	MRPL53
Q9BYD2	Q9BYD2	1	1	1	39S ribosomal protein L9, m	MRPL9
Q15125;C9J719;C9JJ78	Q15125;C9	3;2;2	3;2;2	3;2;2	3-beta-hydroxysteroid-Delta	EBP
Q99714;Q99714-2;Q5H	Q99714;Q9	9;8;5	9;8;5	9;8;5	3-hydroxyacyl-CoA dehydro	HSD17B10
P31937;H7BZL2	P31937;H7	2;1	2;1	2;1	3-hydroxyisobutyrate dehyd	HIBADH
K7ERC8;Q06136	K7ERC8;Q0	1;1	1;1	1;1	3-ketodihydrosphingosine r	KDSR
P51857-2;P51857	P51857-2;P	1;1	1;1	1;1	3-oxo-5-beta-steroid 4-dehy	AKR1D1
P46783;Q9NQ39;F6U21	P46783;Q9	5;4;3;3;3	5;4;3;3;3	5;4;3;3;3	40S ribosomal protein S10;R	RPS10;RPS
P62280;M0QZC5;M0R1	P62280;M(	10;8;4;2	10;8;4;2	10;8;4;2	40S ribosomal protein S11	RPS11
P25398	P25398	7	7	7	40S ribosomal protein S12	RPS12
P62277;J3KMX5;E9PS50	P62277;J3K	6;5;3	6;5;3	6;5;3	40S ribosomal protein S13	RPS13
P62263;H0YB22;E5RH77	P62263;H0	7;5;5	7;5;5	7;5;5	40S ribosomal protein S14	RPS14
I3L3P7;P62244;H3BVC7	I3L3P7;P62	7;7;1	7;7;1	2;2;1	40S ribosomal protein S15a	RPS15A
P62249;M0R3H0;M0R2	P62249;M(	6;5;5;4;3;3	6;5;5;4;3;3	6;5;5;4;3;3	40S ribosomal protein S16	RPS16
P08708;H0YN88;A0A07	P08708;H0	7;6;6;3;1	7;6;6;3;1	7;6;6;3;1	40S ribosomal protein S17	RPS17
P62269;J3JS69;A0A0G2	P62269	6;2;1;1	6;2;1;1	6;2;1;1	40S ribosomal protein S18	RPS18
P39019;M0R2L9;M0QX	P39019;M(	8;4;4;4;4;3	8;4;4;4;4;3	8;4;4;4;4;3	40S ribosomal protein S19	RPS19
P15880;H0YEN5;E9PQD	P15880;H0	9;8;8;4;4;4	9;8;8;4;4;4	9;8;8;4;4;4	40S ribosomal protein S2	RPS2
P60866;P60866-2;E5RIF	P60866;P6(	3;3;2;2;1	3;3;2;2;1	3;3;2;2;1	40S ribosomal protein S20	RPS20
Q8WVC2;P63220;Q9BY	Q8WVC2;P	5;5;3	5;5;3	5;5;3	40S ribosomal protein S21	RPS21
P62266;D6RD47;D6RDJ	P62266;D6	4;3;2;2;2	4;3;2;2;2	4;3;2;2;2	40S ribosomal protein S23	RPS23
E7ETK0;A0A087WUS0;F	E7ETK0;A0	3;3;3;3;3;3	3;3;3;3;3;3	3;3;3;3;3;3	40S ribosomal protein S24	RPS24
P62851	P62851	3	3	3	40S ribosomal protein S25	RPS25
P62854;Q5JNZ5	P62854;Q5	3;2	3;2	3;2	40S ribosomal protein S26;R	RPS26;RPS
P42677;Q5T4L4;H0YMV	P42677;Q5	4;3;2;2;1;1	4;3;2;2;1;1	4;3;2;2;1;1	40S ribosomal protein S27;4	RPS27;RPS
P62857	P62857	2	2	2	40S ribosomal protein S28	RPS28
P62273;P62273-2;A0A0	P62273;P6	2;2;1	2;2;1	2;2;1	40S ribosomal protein S29	RPS29
P23396;P23396-2;E9PL(	P23396;P2	13;12;11;9	13;12;11;9	13;12;11;9	40S ribosomal protein S3	RPS3
E9PR30;P62861	E9PR30;P6	1;1	1;1	1;1	40S ribosomal protein S30	FAU
P61247;D6RG13;D6RAT	P61247;D6	12;11;10;9	12;11;10;9	12;11;10;9	40S ribosomal protein S3a	RPS3A
P62701;C9JEH7;Q8TD47	P62701;C9	12;6;6;6	12;6;6;6	12;6;6;6	40S ribosomal protein S4, X	RPS4X;RPS

M0R0F0;P46782;M0R0I	M0R0F0;P4	7;7;6;3	7;7;6;3	7;7;6;3	40S ribosomal protein S5;4(	RPS5
P62753;A2A3R5;A2A3R	P62753;A2	6;4;3	6;4;3	6;4;3	40S ribosomal protein S6	RPS6
P62081;B5MCP9	P62081;B5I	4;3	4;3	4;3	40S ribosomal protein S7	RPS7
P62241;Q5JR95	P62241;Q5	10;9	10;9	10;9	40S ribosomal protein S8	RPS8
A0A024R4M0;P46781;E	A0A024R4I	9;9;6;6;1;1	9;9;6;6;1;1	9;9;6;6;1;1	40S ribosomal protein S9	RPS9
C9J9K3;A0A0C4DG17;P(	C9J9K3;A0/	8;8;8;4	8;8;8;4	8;8;8;4	40S ribosomal protein SA	RPSA
P08195-2;F5GZS6;J3KPI	P08195-2;F	25;24;24;2	25;24;24;2	25;24;24;2	4F2 cell-surface antigen hea	SLC3A2
Q9H0D6-2;Q9H0D6	Q9H0D6-2;	2;2	2;2	2;2	5-3 exoribonuclease 2	XRN2
P21589-2;P21589;H0Y3	P21589-2;F	2;2;1;1;1	2;2;1;1;1	2;2;1;1;1	5-nucleotidase	NT5E
P10809;E7ESH4;E7EXB4	P10809	28;7;6;3;2;	28;7;6;3;2;	28;7;6;3;2;	60 kDa heat shock protein,	HSPD1
F8VU65;P05388;F8VPE8	F8VU65;P0	9;9;8;8;8;8	9;9;8;8;8;8	9;9;8;8;8;8	60S acidic ribosomal proteir	RPLP0;RPLI
P05386	P05386	2	1	1	60S acidic ribosomal proteir	RPLP1
P05387;H0YDD8;P0538	P05387	7;3;1	7;3;1	6;2;0	60S acidic ribosomal proteir	RPLP2
X1WI28;P27635;B8A6G	X1WI28;P2	8;8;3;3	8;8;3;3	2;2;2;2	60S ribosomal protein L10	RPL10
P62906	P62906	8	8	8	60S ribosomal protein L10a	RPL10A
P62913;Q5VVC8;Q5VV(	P62913;Q5	4;3;2	4;3;2	1;0;0	60S ribosomal protein L11	RPL11
P62913-2	P62913-2	4	1	1	60S ribosomal protein L11	RPL11
P30050;P30050-2	P30050;P3(	6;4	6;4	6;4	60S ribosomal protein L12	RPL12
P26373;P26373-2;J3QSI	P26373;P2(	7;6;4;1;1	7;6;4;1;1	7;6;4;1;1	60S ribosomal protein L13	RPL13
P40429;M0QYS1;Q8J01	P40429;M(	8;7;6;3;1;1	8;7;6;3;1;1	8;7;6;3;1;1	60S ribosomal protein L13a	RPL13A;RP
E7EPB3;P50914	E7EPB3;P5(	4;4	4;4	4;4	60S ribosomal protein L14	RPL14
P61313;E7EX53;E7EQV	P61313;E7I	6;5;5;4;4;2	6;5;5;4;4;2	6;5;5;4;4;2	60S ribosomal protein L15;F	RPL15
A0A087WXM6;J3QQT2;	A0A087WX	7;7;7;7;7;7	7;7;7;7;7;7	7;7;7;7;7;7	60S ribosomal protein L17	RPL17;RPL1
G3V203;H0YHA7;J3QQ6	G3V203;H(	4;4;4;4;4;3	4;4;4;4;4;3	4;4;4;4;4;3	60S ribosomal protein L18	RPL18
M0R3D6;M0R1A7;M0R	M0R3D6;M	5;5;5;5;4	5;5;5;5;4	5;5;5;5;4	60S ribosomal protein L18a	RPL18A
G3V1B3;M0R181;P4677	G3V1B3;M(	2;2;2	2;2;2	2;2;2	60S ribosomal protein L21	RPL21
K7EJT5;K7EP65;K7EKS7;	K7EJT5;K7E	2;2;2;2;2;2	2;2;2;2;2;2	2;2;2;2;2;2	60S ribosomal protein L22	RPL22
P62829;J3KTJ3;C9JD32;	P62829;J3K	4;2;2;2;2	4;2;2;2;2	4;2;2;2;2	60S ribosomal protein L23	RPL23
H7BY10;K7EJV9;K7ERT8	H7BY10;K7	4;4;4;4;4;3	4;4;4;4;4;3	4;4;4;4;4;3	60S ribosomal protein L23a	RPL23A
C9JXB8;C9JNW5;P8373	C9JXB8;C9J	6;6;6	6;6;6	6;6;6	60S ribosomal protein L24	RPL24
P61254;J3KTJ8;J3QRI7;J	P61254;J3K	6;5;5;5;5;5	6;5;5;5;5;5	6;5;5;5;5;5	60S ribosomal protein L26;6	RPL26;RPL2
P61353;K7ELC7;K7EQQ	P61353;K7I	4;3;2;1	4;3;2;1	4;3;2;1	60S ribosomal protein L27	RPL27
E9PLL6;P46776;E9PJD9;	E9PLL6;P46	4;4;3;1	4;4;3;1	4;4;3;1	60S ribosomal protein L27a	RPL27A
H0YMF4;H0YKD8;P4677	H0YMF4;H(	5;5;5;5;5;5	5;5;5;5;5;5	5;5;5;5;5;5	60S ribosomal protein L28	RPL28
P47914	P47914	2	2	2	60S ribosomal protein L29	RPL29
P39023;G5E9G0;H7C42	P39023;G5	16;13;9;8;7	16;13;9;8;7	16;13;9;8;7	60S ribosomal protein L3	RPL3
P62888;E5RI99;A0A0B4	P62888;E5I	5;4;3;2;1	5;4;3;2;1	5;4;3;2;1	60S ribosomal protein L30	RPL30
H7C2W9;C9JU56;B7Z4E	H7C2W9;C	4;4;4;4;4;4	4;4;4;4;4;4	4;4;4;4;4;4	60S ribosomal protein L31	RPL31
D3YTB1;F8W727;P6291	D3YTB1;F8	5;5;5	5;5;5	5;5;5	60S ribosomal protein L32	RPL32
P49207	P49207	6	6	6	60S ribosomal protein L34	RPL34
F2Z388;P42766	F2Z388;P4	2;2	2;2	2;2	60S ribosomal protein L35	RPL35
C9K025;P18077;F8WBS	C9K025;P1	5;5;3;3	5;5;3;3	5;5;3;3	60S ribosomal protein L35a	RPL35A
Q9Y3U8;J3QSB5;J3KTD	Q9Y3U8;J3	3;2;1	3;2;1	3;2;1	60S ribosomal protein L36	RPL36
H0Y5B4;H7BZ11;J3KQN	H0Y5B4;H7	2;2;2;2;2;1	2;2;2;2;2;1	2;2;2;2;2;1	60S ribosomal protein L36a	RPL36A;RP
C9J4Z3;P61513;M0R0A	C9J4Z3;P61	4;4;3;3;3;3	4;4;3;3;3;3	4;4;3;3;3;3	60S ribosomal protein L37a	RPL37A
J3KT73;J3QL01;P63173	J3KT73;J3C	1;1;1	1;1;1	1;1;1	60S ribosomal protein L38	RPL38
P36578;H3BM89;H3BTI	P36578;H3	15;9;7;7	15;9;7;7	15;9;7;7	60S ribosomal protein L4	RPL4
P46777;Q5T7N0	P46777	9;4	9;4	9;4	60S ribosomal protein L5	RPL5
Q02878;F8VZ45;U3KQR	Q02878	13;3;3;2;1;	13;3;3;2;1;	13;3;3;2;1;	60S ribosomal protein L6	RPL6
A8MUD9;P18124;C9JZ8	A8MUD9;P	6;6;2;2	6;6;2;2	6;6;2;2	60S ribosomal protein L7	RPL7

P62424;Q5T8U2;Q5T8U	P62424;Q5	14;8;8	14;8;8	14;8;8	60S ribosomal protein L7a	RPL7A
R4GMU7;Q6DKI1-2;Q6D	R4GMU7;C	1;1;1	1;1;1	1;1;1	60S ribosomal protein L7-lik	RPL7L1
P62917;E9PKZ0;E9PKU4	P62917;E9I	5;4;4;3;2	5;4;4;3;2	5;4;4;3;2	60S ribosomal protein L8	RPL8
P32969;E7ESE0;D6RAN4	P32969;E7I	5;4;4;4;2	5;4;4;4;2	5;4;4;4;2	60S ribosomal protein L9	RPL9
J3QLW7;Q9Y221-2;Q9Y	J3QLW7;Q9	2;2;2	2;2;2	2;2;2	60S ribosome subunit bioge	NIP7
P52209-2;P52209;K7EM	P52209-2;P	10;10;7;6;6	10;10;7;6;6	10;10;7;6;6	6-phosphogluconate dehyd	PGD
M0R0U3;M0R261;O953	M0R0U3;M	2;2;2;1	2;2;2;1	2;2;2;1	6-phosphogluconolactonase	PGLS
P36639-4;P36639-3;P36	P36639-4;P	2;2;2;2	2;2;2;2	2;2;2;2	7,8-dihydro-8-oxoguanine t	NUDT1
P11021	P11021	30	30	29	78 kDa glucose-regulated pı	HSPA5
Q9UBM7;E9PJ54;E9PM	Q9UBM7;E	6;4;4;3;3;3	6;4;4;3;3;3	6;4;4;3;3;3	7-dehydrocholesterol reduc	DHCR7
Q7L2J0	Q7L2J0	1	1	1	7SK snRNA methylphosphat	MEPCE
A0A0J9YWL0;Q9Y4K1;C	A0A0J9YW	3;3;1;1	3;3;1;1	3;3;1;1	Absent in melanoma 1 prot	AIM1
M0R026;A1L0T0	M0R026;A:	1;1	1;1	1;1	Acetolactate synthase-like p	ILVBL
Q9BWD1;Q9BWD1-2	Q9BWD1;C	2;2	2;2	2;2	Acetyl-CoA acetyltransferas	ACAT2
P24752;H0YEL7;P24752	P24752;H0	6;3;3;2;1	6;3;3;2;1	6;3;3;2;1	Acetyl-CoA acetyltransferas	ACAT1
O00763-3;Q13085-3;Q1	O00763-3;(	1;1;1;1;1;1	1;1;1;1;1;1	1;1;1;1;1;1	Acetyl-CoA carboxylase 2;Bi	ACACB;ACA
H0YN26;P39687;O4342	H0YN26;P3	4;4;2;1	4;4;2;1	3;3;2;1	Acidic leucine-rich nuclear p	ANP32A;AN
Q92688-2;Q92688	Q92688-2;(	2;2	1;1	1;1	Acidic leucine-rich nuclear p	ANP32B
A2A274;Q99798	A2A274;Q9	9;9	9;9	9;9	Aconitate hydratase, mitoch	ACO2
P60709;G5E9R0;E7EVS6	P60709	19;8;8;7;6;	1;1;1;1;1;0	1;1;1;1;1;0	Actin, cytoplasmic 1;Actin, c	ACTB
P63261;I3L3I0;I3L1U9;I3	P63261;I3L	19;11;11;9;	19;11;11;9;	1;1;1;1;1;1	Actin, cytoplasmic 2;Actin, c	ACTG1
P63267;P68133;P68032	P63267;P6	10;10;10;10	1;1;1;1;1;1	1;1;1;1;1;1	Actin, gamma-enteric smoo	ACTG2;ACT
O96019;O96019-2;H7C5	O96019;O9	6;5;3;1;1	6;5;3;1;1	6;5;3;1;1	Actin-like protein 6A	ACTL6A
P61160;P61160-2;F5H6	P61160;P6	5;4;3;1;1	5;4;3;1;1	5;4;3;1;1	Actin-related protein 2	ACTR2
O15143;C9J4Z7;F8VXW	O15143;C9	4;2;2;2;2;2	4;2;2;2;2;2	4;2;2;2;2;2	Actin-related protein 2/3 cc	ARPC1B
O15144;G5E9S7;C9JTV5	O15144	4;1;1;1	4;1;1;1	4;1;1;1	Actin-related protein 2/3 cc	ARPC2
F8VR50;C9JZD1;O15145	F8VR50;C9	1;1;1	1;1;1	1;1;1	Actin-related protein 2/3 cc	ARPC3
F8WDD7;F8WCF6;A0A0	F8WDD7;F8	4;4;4;4;4;3	4;4;4;4;4;3	4;4;4;4;4;3	Actin-related protein 2/3 cc	ARPC4;ARP
B1ALC0;O15511;O1551	B1ALC0;O1	1;1;1	1;1;1	1;1;1	Actin-related protein 2/3 cc	ARPC5
Q9BPX5	Q9BPX5	1	1	1	Actin-related protein 2/3 cc	ARPC5L
P61158;B4DXW1;F8WD	P61158;B4	7;6;3;1;1;1	7;6;3;1;1;1	7;6;3;1;1;1	Actin-related protein 3	ACTR3
P53999	P53999	3	3	3	Activated RNA polymerase I	SUB1
O95433-2;O95433;H0YJ	O95433-2;(	8;8;6;4;3;3	8;8;6;4;3;3	8;8;6;4;3;3	Activator of 90 kDa heat sh	AHSA1
Q86WX3	Q86WX3	1	1	1	Active regulator of SIRT1	RPS19BP1
O14561;I3L505;H3BNK3	O14561;I3L	2;1;1	2;1;1	2;1;1	Acyl carrier protein, mitoch	NDUFAB1
C9JIF9;P13798	C9JIF9;P13	2;2	2;2	2;2	Acylamino-acid-releasing er	APEH
Q9H845;H0Y8Z9	Q9H845	5;2	5;2	5;2	Acyl-CoA dehydrogenase fa	ACAD9
O00767	O00767	2	2	2	Acyl-CoA desaturase	SCD
B8ZWD1;A0A0A0MTI5;I	B8ZWD1;A	3;3;3;3;3;3	3;3;3;3;3;3	3;3;3;3;3;3	Acyl-CoA-binding protein	DBI
Q9NPJ3-2;Q9NPJ3	Q9NPJ3-2;(	1;1	1;1	1;1	Acyl-coenzyme A thioestera	ACOT13
H7C5A7;O14734;E9PRD	H7C5A7;O1	2;2;1;1	2;2;1;1	2;2;1;1	Acyl-coenzyme A thioestera	ACOT8
Q9Y305-3;Q9Y305-2;Q9	Q9Y305-3;(	5;5;5;5;3	5;5;5;5;3	5;5;5;5;3	Acyl-coenzyme A thioestera	ACOT9
A0A087X1K9;E5RGR0;O	A0A087X1K	3;3;3;3;2;1	3;3;3;3;2;1	3;3;3;3;2;1	Acyl-protein thioesterase 1	LYPLA1
Q5QPQ0;O95372	Q5QPQ0;O	2;2	2;2	2;2	Acyl-protein thioesterase 2	LYPLA2
Q6P587;Q6P587-2;Q6P	Q6P587;Q6	1;1;1	1;1;1	1;1;1	Acylpyruvase FAHD1, mitoc	FAHD1
H3BSW3;H3BQF1;H3BC	H3BSW3;H	3;3;3;3;3;2	3;3;3;3;3;2	3;3;3;3;3;2	Adenine phosphoribosyltran	APRT
Q8TB61-5;Q8TB61-4;Q8	Q8TB61-5;(	2;2;2;2;2	2;2;2;2;2	2;2;2;2;2	Adenosine 3-phospho 5-pho	SLC35B2
P55263-3;P55263-4;P55	P55263-3;P	1;1;1;1	1;1;1;1	1;1;1;1	Adenosine kinase	ADK
P23526;P23526-2	P23526;P2	15;13	15;13	15;13	Adenosylhomocysteinase	AHCY
F8W1A4;P54819-5;P548	F8W1A4;P5	6;6;6;6;5;5	6;6;6;6;5;5	6;6;6;6;5;5	Adenylate kinase 2, mitocho	AK2

P27144;D3DQ64	P27144;D3	2;1	2;1	2;1	Adenylate kinase 4, mitocho	AK4
Q5T9B7;P00568;H0Y4J6	Q5T9B7;P0	5;5;3;1	5;5;3;1	5;5;3;1	Adenylate kinase isoenzyme	AK1
A0A096LNY6;A0A1B0GV	A0A096LNY	2;2;2;2;2;1	2;2;2;2;2;1	2;2;2;2;2;1	Adenylosuccinate lyase	ADSL
P30520	P30520	5	5	5	Adenylosuccinate synthetas	ADSS
Q01518-2;Q01518;Q5T	Q01518-2;	11;11;4;4;4	11;11;4;4;4	11;11;4;4;4	Adenylyl cyclase-associated	CAP1
Q9HDC9-2;Q9HDC9;H0Y	Q9HDC9-2;	6;6;5	6;6;5	6;6;5	Adipocyte plasma membrar	APMAP
Q15847	Q15847	1	1	1	Adipogenesis regulatory fac	ADIRF
P05141;Q9H0C2	P05141	15;3	15;3	6;0	ADP/ATP translocase 2;ADP	SLC25A5
P12236	P12236	13	4	2	ADP/ATP translocase 3;ADP	SLC25A6
Q9BRR6-2;Q9BRR6;Q9B	Q9BRR6-2;	3;3;2;2;1;1	3;3;2;2;1;1	3;3;2;2;1;1	ADP-dependent glucokinase	ADPGK
P84077;P61204;F5H423	P84077;P6	6;6;5;4;3;1	6;6;5;4;3;1	3;3;2;2;1;1	ADP-ribosylation factor 1;A	ARF1;ARF3
C9JPM4;P18085;C9JAK5	C9JPM4;P1	3;3;1	1;1;1	1;1;1	ADP-ribosylation factor 4	ARF4
C9J1Z8;P84085;F5H1V1	C9J1Z8;P84	5;5;1;1;1	2;2;0;0;0	2;2;0;0;0	ADP-ribosylation factor 5	ARF5
P62330	P62330	3	3	3	ADP-ribosylation factor 6	ARF6
Q9NVJ2	Q9NVJ2	2	2	1	ADP-ribosylation factor-like	ARL8B
A6NCQ0;A6NJU6;A6NF	A6NCQ0;A	3;3;3;3;2;1	3;3;3;3;2;1	3;3;3;3;2;1	ADP-sugar pyrophosphatase	NUDT5
Q9Y4W6	Q9Y4W6	2	2	2	AFG3-like protein 2	AFG3L2
A0A087X208;O00468-2	A0A087X2	5;5;5;5;5;5	5;5;5;5;5;5	5;5;5;5;5;5	Agrin;Agrin N-terminal 110	AGRN
O00170	O00170	1	1	1	AH receptor-interacting pro	AIP
H0YK84;H0YMW2;Q128	H0YK84;H0	1;1;1;1;1	1;1;1;1;1	1;1;1;1;1	A-kinase anchor protein 13	AKAP13
H3BU82;F8VZ44;Q9NR	H3BU82;F8	2;2;2;2;1	2;2;2;2;1	2;2;2;2;1	Aladin	AAAS
P49588;P49588-2;H3BP	P49588;P4	7;6;1	7;6;1	7;6;1	Alanine--tRNA ligase, cytopl	AARS
P14550;V9GYG2;V9GYP	P14550;V9	4;2;2;2	4;2;2;2	4;2;2;2	Alcohol dehydrogenase [NA	AKR1A1
P11766;D6RFE4;H0YAG	P11766	4;1;1	4;1;1	4;1;1	Alcohol dehydrogenase clas	ADH5
P47895;H0Y2X5;H0YKF	P47895;H0	18;16;7;6;2	18;16;7;6;2	16;15;6;6;1	Aldehyde dehydrogenase fa	ALDH1A3
P30837	P30837	4	2	2	Aldehyde dehydrogenase X,	ALDH1B1
Q04828;H0Y804	Q04828;H0	11;6	11;6	0;0	Aldo-keto reductase family	AKR1C1
B4DK69;P52895;S4R3P	B4DK69;P5	10;10;5;2	2;2;2;0	2;2;2;0	Aldo-keto reductase family	AKR1C2
A0A0A0MSS8;P42330;S	A0A0A0MS	8;8;7;4;4;4	2;2;1;0;1;0	2;2;1;0;1;0	Aldo-keto reductase family	AKR1C3;AK
P15121;E9PEF9;E9PCX2	P15121;E9	5;3;3;1;1	5;3;3;1;1	5;3;3;1;1	Aldose reductase	AKR1B1
O00116;A0A1B0GWA2;	O00116;A0	10;7;4	10;7;4	10;7;4	Alkyldihydroxyacetonephos	AGPS
Q9H553-2;Q9H553	Q9H553-2;	1;1	1;1	1;1	Alpha-1,3/1,6-mannosyltrar	ALG2
E9PK47;P11217-2;P067	E9PK47;P1	1;1;1;1;1;1	1;1;1;1;1;1	1;1;1;1;1;1	Alpha-1,4 glucan phosphory	PYGL;PYGM
P30533	P30533	2	2	2	Alpha-2-macroglobulin rece	LRPAP1
P12814;P12814-3;P128	P12814;P1	36;35;34;3	36;35;34;3	23;22;21;1	Alpha-actinin-1	ACTN1
O43707;O43707-2;F5G	O43707;O4	35;24;19;1	22;15;13;1	22;15;13;1	Alpha-actinin-4	ACTN4
A0A1B0GTY9;A0A1B0G	A0A1B0GT	5;5;5;5;5;5	5;5;5;5;5;5	5;5;5;5;5;5	Alpha-aminoadipic semiald	ALDH7A1
R4GMT0;P61163	R4GMT0;P	1;1	1;1	1;1	Alpha-centractin	ACTR1A
A0A1W2PRU0;A6NMQ	A0A1W2PR	1;1;1;1;1;1	1;1;1;1;1;1	1;1;1;1;1;1	Alpha-endosulfine	ENSA
P06733;P06733-2;K7EM	P06733;P0	23;14;11;2	23;14;11;2	23;14;11;2	Alpha-enolase	ENO1
Q16706	Q16706	2	2	2	Alpha-mannosidase 2	MAN2A1
H0YKM7;H0YLB9;A0A0	H0YKM7;H	1;1;1;1;1;1	1;1;1;1;1;1	1;1;1;1;1;1	Alpha-mannosidase;Alpha-r	MAN2A2
M0R0Y2;P54920;M0R2	M0R0Y2;P5	6;6;5;2;1;1	6;6;5;2;1;1	6;6;5;2;1;1	Alpha-soluble NSF attachme	NAPA
P40222	P40222	1	1	1	Alpha-taxilin	TXLNA
P21397;P21397-2;P273	P21397;P2	4;3;1;1	4;3;1;1	4;3;1;1	Amine oxidase [flavin-conta	MAOA
Q12904;Q12904-2;D6R	Q12904;Q1	4;4;1	4;4;1	4;4;1	Aminoacyl tRNA synthase c	AIMP1
Q13155;A8MU58;F8W	Q13155;A8	3;2;2	3;2;2	3;2;2	Aminoacyl tRNA synthase c	AIMP2
A6NKB8;Q9H4A4;A0A0	A6NKB8;Q	6;6;4;3;1	6;6;4;3;1	6;6;4;3;1	Aminopeptidase B	RNPEP
Q86SJ2	Q86SJ2	2	2	2	Amphoterin-induced protei	AMIGO2
E9PG40;P05067-7;P050	E9PG40;P0	8;8;8;8;8;8	8;8;8;8;8;8	7;7;7;7;7;7	Amyloid beta A4 protein;N-	APP

Q06481-3;Q06481-6;Q0	Q06481-3;(	7;7;7;5;5;5	6;6;6;4;4;4	6;6;6;4;4;4	Amyloid-like protein 2	APLP2
Q9Y679-3;Q9Y679-2;Q9	Q9Y679-3;(	1;1;1	1;1;1	1;1;1	Ancient ubiquitous protein	AUP1
A0A1W2PQ15;E9PDP5;I	A0A1W2PC	1;1;1;1;1;1	1;1;1;1;1;1	1;1;1;1;1;1	Ankyrin repeat and KH dom	ANKHD1;AI
P04083;Q5T3N1;Q5T3N	P04083	23;9;6	23;9;6	23;9;6	Annexin A1	ANXA1
Q9UJ72	Q9UJ72	1	1	1	Annexin A10	ANXA10
P50995-2;P50995;H0Y6	P50995-2;F	7;7;2;1;1;1	7;7;2;1;1;1	7;7;2;1;1;1	Annexin A11	ANXA11
P07355;P07355-2;H0YN	P07355;P0	25;24;23;2	25;24;23;2	25;24;23;2	Annexin A2;Annexin;Putativ	ANXA2;AN
P12429;D6RA82;D6RFG	P12429;D6	12;10;5;2;2	12;10;5;2;2	12;10;5;2;2	Annexin A3;Annexin	ANXA3
P09525;Q6P452;P0952	P09525;Q6	11;10;9;4	11;10;9;4	11;10;9;4	Annexin A4;Annexin	ANXA4
P08758;D6RBL5;D6RBE	P08758;D6	11;8;7;5;1	11;8;7;5;1	11;8;7;5;1	Annexin A5;Annexin	ANXA5
P20073-2;P20073	P20073-2;F	11;11	11;11	11;11	Annexin A7	ANXA7
P13928;A0A087WTN9;(	P13928;A0	10;9;8;7;7;	10;9;8;7;7;	10;9;8;7;7;	Annexin A8;Annexin;Annexi	ANXA8;AN
P46013-2;P46013	P46013-2;F	15;15	15;15	15;15	Antigen KI-67	MKI67
A0A140T9T7;Q03518	A0A140T91	2;2	2;2	2;2	Antigen peptide transporte	TAP1
Q10567-4;Q10567-3;Q1	Q10567-4;(	7;7;7;7;5	7;7;7;7;5	3;3;3;3;1	AP-1 complex subunit beta-	AP1B1
H7C1E4;P61966;P6196	H7C1E4;P6	2;2;1	2;2;1	2;2;1	AP-1 complex subunit sigma	AP1S1
O95782-2;O95782;A0A	O95782-2;(	4;4;2;2;2;2	4;4;2;2;2;2	4;4;2;2;2;2	AP-2 complex subunit alpha	AP2A1;AP2
A0A087WU93;A0A087V	A0A087WL	6;6;6;6;6;4	2;2;2;2;2;2	2;2;2;2;2;2	AP-2 complex subunit beta	AP2B1
A0A087WY71;E9PFW3;	A0A087WY	3;3;3;3;2;1	3;3;3;3;2;1	3;3;3;3;2;1	AP-2 complex subunit mu	AP2M1
O14617-3;O14617-4;O1	O14617-3;(	1;1;1;1;1	1;1;1;1;1	1;1;1;1;1	AP-3 complex subunit delta	AP3D1
Q9Y2T2	Q9Y2T2	1	1	1	AP-3 complex subunit mu-1	AP3M1
Q92572;F5H459	Q92572;F5	2;1	2;1	2;1	AP-3 complex subunit sigma	AP3S1
P04114	P04114	7	5	5	Apolipoprotein B-100;Apoli	APOB
B0YIW2;P02656	B0YIW2;P0	1;1	1;1	1;1	Apolipoprotein C-III	APOC3
J3KQL8;Q9BQE5;E9PM	J3KQL8;Q9	2;2;1	2;2;1	2;2;1	Apolipoprotein L2	APOL2
G3V1B6;H7C1U8;Q9BU	G3V1B6;H7	2;2;2;2	2;2;2;2	2;2;2;2	Apolipoprotein O	APOO
Q9BZZ5-2;G3V1C3;Q9B	Q9BZZ5-2;(	4;3;3;3;3;2	4;3;3;3;3;2	4;3;3;3;3;2	Apoptosis inhibitor 5	API5
Q07812-5;Q07812-7;Q0	Q07812-5;(	3;3;3;3;3;2	3;3;3;3;3;2	3;3;3;3;3;2	Apoptosis regulator BAX	BAX
O95831-3;O95831;E9PI	O95831-3;(	9;9;5;5;5;4	9;9;5;5;5;4	9;9;5;5;5;4	Apoptosis-inducing factor 1	AIFM1
S4R3H4;E7EQT4;Q9UK	S4R3H4;E7	8;7;7;7;5;5	8;7;7;7;5;5	8;7;7;7;5;5	Apoptotic chromatin conde	ACIN1
A0A087X1E4;P53365-2;	A0A087X1E	1;1;1;1	1;1;1;1	1;1;1;1	Arfaptin-2	ARFIP2
P54136;P54136-2;E5RH	P54136;P5	8;7;1;1	8;7;1;1	8;7;1;1	Arginine--tRNA ligase, cytop	RARS
P08243-2;P08243;P082	P08243-2;F	5;5;4;3;2;2	5;5;4;3;2;2	5;5;4;3;2;2	Asparagine synthetase [glut	ASNS
O43776;K7EIU7;K7EPK2	O43776	6;2;2;2;2;1	6;2;2;2;2;1	6;2;2;2;2;1	Asparagine--tRNA ligase, cy	NARS
P17174;P17174-2	P17174;P1	4;3	4;3	4;3	Aspartate aminotransferase	GOT1
P00505;P00505-2	P00505;P0	13;10	13;10	13;10	Aspartate aminotransferase	GOT2
P14868;P14868-2;C9J7	P14868;P1	20;17;9;9;6	20;17;9;9;6	20;17;9;9;6	Aspartate--tRNA ligase, cyto	DARS
Q6PI48	Q6PI48	2	2	2	Aspartate--tRNA ligase, mito	DARS2
Q12797;Q12797-10;E5F	Q12797;Q1	4;3;2;2;2;2	4;3;2;2;2;2	4;3;2;2;2;2	Aspartyl/asparaginyl beta-h	ASPH
B1AKZ5;Q15121;Q1512	B1AKZ5;Q1	1;1;1	1;1;1	1;1;1	Astrocytic phosphoprotein I	PEA15
Q8WWM7-6;Q8WWM7	Q8WWM7-	2;2;2;2;2;2	2;2;2;2;2;2	2;2;2;2;2;2	Ataxin-2-like protein	ATXN2L
B5MCN0;Q8NHH9-3;Q8	B5MCN0;Q	3;3;3;3;3;3	3;3;3;3;3;3	3;3;3;3;3;3	Atlastin-2	ATL2
F5H6I7;Q6DD88;F5GWI	F5H6I7;Q6I	2;2;1	2;2;1	2;2;1	Atlastin-3	ATL3
Q5QNZ2;P24539	Q5QNZ2;P2	7;7	7;7	7;7	ATP synthase F(0) complex	ATP5F1
P00846	P00846	1	1	1	ATP synthase subunit a	MT-ATP6
P25705;P25705-2;P257	P25705;P2	24;23;22;1	24;23;22;1	24;23;22;1	ATP synthase subunit alpha	ATP5A1
P06576;H0YH81;F8W07	P06576;H0	22;18;11;6;	22;18;11;6;	22;18;11;6;	ATP synthase subunit beta,	ATP5B
O75947;O75947-2;F5H	O75947;O7	8;6;3	8;6;3	8;6;3	ATP synthase subunit d, mit	ATP5H
P30049	P30049	2	2	2	ATP synthase subunit delta,	ATP5D
P56385	P56385	2	2	2	ATP synthase subunit e, mit	ATP5I

P56134-3;P56134;C9JJT	P56134-3;F	2;2;1;1;1;1	2;2;1;1;1;1	2;2;1;1;1;1	ATP synthase subunit f, mit	ATP5J2;ATI
E9PN17;O75964	E9PN17;O7	3;3	3;3	3;3	ATP synthase subunit g, mit	ATP5L
P36542-2;P36542	P36542-2;F	10;10	10;10	10;10	ATP synthase subunit gamn	ATP5C1
P48047;H7C0C1;H7C06	P48047;H7	7;4;3;2	7;4;3;2	7;4;3;2	ATP synthase subunit O, mi	ATP5O
P18859;P18859-2;A8M	P18859;P1	4;4;3	4;4;3	4;4;3	ATP synthase-coupling fact	ATP5J
Q9NVI7-2;Q9NVI7;H0Y	Q9NVI7-2;	6;6;5;4;4;3	6;6;5;4;4;3	6;6;5;4;4;3	ATPase family AAA domain-	ATAD3A;AT
E9PJR8;B4DGL8;A0A08	E9PJR8;B4	1;1;1;1;1;1	1;1;1;1;1;1	1;1;1;1;1;1	ATP-binding cassette sub-fa	ABCB7
P28288;P28288-3;P282	P28288;P2	2;1;1	2;1;1	2;1;1	ATP-binding cassette sub-fa	ABCD3
P61221;D6R9I9;H0Y990	P61221;D6	4;2;1;1	4;2;1;1	4;2;1;1	ATP-binding cassette sub-fa	ABCE1
H0YGW7;Q8NE71-2;Q8	H0YGW7;Q	2;2;2	2;2;2	2;2;2	ATP-binding cassette sub-fa	ABCF1
Q9UG63;Q9UG63-2	Q9UG63;Q	1;1	1;1	1;1	ATP-binding cassette sub-fa	ABCF2
Q9NUQ8-2;Q9NUQ8	Q9NUQ8-2	1;1	1;1	1;1	ATP-binding cassette sub-fa	ABCF3
P53396-2;P53396;P533	P53396-2;F	22;22;15;5	22;22;15;5	22;22;15;5	ATP-citrate synthase	ACLY
P17858;P17858-2	P17858;P1	4;3	2;2	2;2	ATP-dependent 6-phosphof	PFKL
P08237-2;P08237;P082	P08237-2;F	4;4;4;1	3;3;3;1	3;3;3;1	ATP-dependent 6-phosphof	PFKM
Q01813;Q01813-2;B1A	Q01813;Q	13;12;4;3;3	13;12;4;3;3	11;11;4;3;2	ATP-dependent 6-phosphof	PFKP
O76031;H0YM48;H0YK	O76031	5;2;1	5;2;1	5;2;1	ATP-dependent Clp proteas	CLPX
M0R208;Q16740;M0QY	M0R208;Q	3;3;1	3;3;1	3;3;1	ATP-dependent Clp proteas	CLPP
P46063	P46063	3	3	3	ATP-dependent DNA helica	RECQL
Q08211;Q08211-2	Q08211	26;6	26;6	26;6	ATP-dependent RNA helicas	DHX9
F1T0B3;A0A087X2G1;Q	F1T0B3;A0	10;10;10;9	10;10;10;9	10;10;10;9	ATP-dependent RNA helicas	DDX1
Q9NVP1;H7C452	Q9NVP1	9;3	9;3	9;3	ATP-dependent RNA helicas	DDX18
I3L0H8;F6QDS0;H3BQK	I3L0H8;F6	2;2;2;2;2;2	2;2;2;2;2;2	2;2;2;2;2;2	ATP-dependent RNA helicas	DDX19A;DI
G3V529;Q9GZR7-2;Q9	G3V529;Q	6;6;6;4;4;1	6;6;6;4;4;1	6;6;6;4;4;1	ATP-dependent RNA helicas	DDX24
O00148;K7EPJ3;O0014	O00148;K7	8;5;5;5;4;3	1;1;1;1;1;0	1;1;1;1;1;0	ATP-dependent RNA helicas	DDX39A
A0A0D9SFB3;A0A0D9S	A0A0D9SF	14;14;14;1	13;13;13;1	13;13;13;1	ATP-dependent RNA helicas	DDX3X;DD
A0A0A0MSJ0;Q86XP3-2	A0A0A0MS	1;1;1	1;1;1	1;1;1	ATP-dependent RNA helicas	DDX42
Q8TDD1;Q8TDD1-2;H0	Q8TDD1;Q	6;6;1	6;6;1	6;6;1	ATP-dependent RNA helicas	DDX54
Q8IYB8	Q8IYB8	2	2	2	ATP-dependent RNA helicas	SUPV3L1
J3KRJ2;J3KRF8;J3QR41;	J3KRJ2;J3K	1;1;1;1;1;1	1;1;1;1;1;1	1;1;1;1;1;1	Aurora kinase B	AURKB
O95816	O95816	1	1	1	BAG family molecular chap	BAG2
O95817	O95817	3	3	3	BAG family molecular chap	BAG3
A0A0C4DH22;Q9H4G0-	A0A0C4DH	2;2;2;2;2;1	2;2;2;2;2;1	2;2;2;2;2;1	Band 4.1-like protein 1	EPB41L1
O75531	O75531	1	1	1	Barrier-to-autointegration f	BANF1
P98160	P98160	1	1	1	Basement membrane-speci	HSPG2
Q7L1Q6-2;Q7L1Q6;Q7L	Q7L1Q6-2;	4;4;4;4;3;2	4;4;4;4;3;2	4;4;4;4;3;2	Basic leucine zipper and W2	BZW1
Q75MG1;E7ETZ4;Q9Y6	Q75MG1;E	3;3;3;2;2;2	3;3;3;2;2;2	3;3;3;2;2;2	Basic leucine zipper and W2	BZW2
P35613-4;P35613-2;P3	P35613-4;F	8;8;8;7;7;6	8;8;8;7;7;6	8;8;8;7;7;6	Basigin	BSG
P51572;P51572-2;C9JS	P51572;P5	6;6;2;2;2;1	6;6;2;2;2;1	6;6;2;2;2;1	B-cell receptor-associated p	BCAP31
Q16611-2;Q16611	Q16611-2;	1;1	1;1	1;1	Bcl-2 homologous antagoni	BAK1
E9PJA7;A0A1W2PQ43;E	E9PJA7;A0	4;4;4;4;4;4	4;4;4;4;4;4	4;4;4;4;4;4	Bcl-2-associated transcripti	BCLAF1
A0A087WTL4;A0A087W	A0A087WT	3;3;3;3	3;3;3;3	3;3;3;3	Bcl-2-like protein 13	BCL2L13
P61769;H0YLF3;F5H6I0	P61769;H0	3;2;2	3;2;2	3;2;2	Beta-2-microglobulin;Beta-	B2M
Q13425	Q13425	1	1	1	Beta-2-syntrophin	SNTB2
P42025	P42025	1	1	1	Beta-centractin	ACTR1B
Q53H82	Q53H82	3	3	3	Beta-lactamase-like protein	LACTB2
P55957;P55957-2;B2ZP	P55957;P5	3;3;1;1	3;3;1;1	3;3;1;1	BH3-interacting domain de	BID
Q13057;Q13057-2;K7E	Q13057;Q1	3;3;1;1;1;1	3;3;1;1;1;1	3;3;1;1;1;1	Bifunctional coenzyme A sy	COASY
P07814;V9GYZ6;V9GZ7	P07814;V9	22;13;1	22;13;1	22;13;1	Bifunctional glutamate/prol	EPRS
P13995-2;P13995;B9A0	P13995-2;F	2;2;1	2;2;1	2;2;1	Bifunctional methylenetetr	MTHFD2

P31939-2;P31939;H7C1	P31939-2;F	13;13;5;3;2	13;13;5;3;2	13;13;5;3;2	Bifunctional purine biosynth	ATIC
Q8NFC6	Q8NFC6	1	1	1	Biorientation of chromosom	BOD1L1
A0A087WZT3;Q9H3K6;I	A0A087WZ	2;2;1;1;1;1	2;2;1;1;1;1	2;2;1;1;1;1	BolA-like protein 2	BOLA2;BOL
F5GXW6;O95415	F5GXW6;O	1;1	1;1	1;1	Brain protein I3	BRI3
Q9UHR4	Q9UHR4	4	4	4	Brain-specific angiogenesis	BAIAP2L1
P54687-2;P54687-3;P54	P54687-2;F	1;1;1;1;1	1;1;1;1;1	1;1;1;1;1	Branched-chain-amino-acid	BCAT1
Q9P287-4;Q9P287-3;Q9	Q9P287-4;(	2;2;2;2	2;2;2;2	2;2;2;2	BRCA2 and CDKN1A-interac	BCCIP
Q8WY22	Q8WY22	2	2	2	BRI3-binding protein	BRI3BP
Q9NRL2-2;Q9NRL2	Q9NRL2-2;(	2;2	2;2	2;2	Bromodomain adjacent to z	BAZ1A
Q96CX2	Q96CX2	4	4	4	BTB/POZ domain-containing	KCTD12
H3BSS5;Q9NXV2	H3BSS5;Q9	1;1	1;1	1;1	BTB/POZ domain-containing	KCTD5
J3QRS9;X6R4W8;O4367	J3QRS9;X6I	4;4;4;4;4;3	4;4;4;4;4;3	4;4;4;4;4;3	BUB3-interacting and GLEBS	ZNF207
F5H2F4;P11586;V9GYY3	F5H2F4;P1	15;15;3	15;15;3	15;15;3	C-1-tetrahydrofolate syntha	MTHFD1
O75844	O75844	4	4	4	CAAX prenyl protease 1 hor	ZMPSTE24
E9PKA7;E9PI08;Q9Y256	E9PKA7;E9	1;1;1	1;1;1	1;1;1	CAAX prenyl protease 2	RCE1
F8VPD4;P27708;H7C2E4	F8VPD4;P2	20;20;3;2;1	20;20;3;2;1	20;20;3;2;1	CAD protein;Glutamine-dep	CAD
A0A087WXI5;P12830-2	A0A087WX	2;2;2	2;2;2	2;2;2	Cadherin-1;E-Cad/CTF1;E-Ca	CDH1
P22223-2;P22223	P22223-2;F	4;4	4;4	4;4	Cadherin-3	CDH3
Q99653;H0YLY7;H0YKE7	Q99653;H0	3;2;2;2;2	3;2;2;2;2	3;2;2;2;2	Calcineurin B homologous p	CHP1
Q8IU85-2;Q8IU85	Q8IU85-2;(	1;1	1;1	1;1	Calcium/calmodulin-depend	CAMK1D
Q9UJS0;Q9UJS0-2;O757	Q9UJS0;Q9	17;17;4;4;2	17;17;4;4;2	17;17;4;4;2	Calcium-binding mitochond	SLC25A13
Q6NUK1-2;Q6NUK1	Q6NUK1-2;	6;6	6;6	6;6	Calcium-binding mitochond	SLC25A24
B4E2Q0;P98194-4;P981	B4E2Q0;P9	7;7;7;6;6;6	7;7;7;6;6;6	7;7;7;6;6;6	Calcium-transporting ATPas	ATP2C1
Q9HB71-3;Q9HB71	Q9HB71-3;	6;6	6;6	6;6	Calcyclin-binding protein	CACYBP
P27824;P27824-2;P278	P27824;P27	21;21;14;1(	21;21;14;1(	21;21;14;1(	Calnexin	CANX
K7EM73;K7EIV0;A0A075	K7EM73;K7	4;4;4;4;4;3	4;4;4;4;4;3	4;4;4;4;4;3	Calpain small subunit 1	CAPNS1
P07384;E9PLX0;E9PJJ3;I	P07384	8;2;2;2;2;2	8;2;2;2;2;2	8;2;2;2;2;2	Calpain-1 catalytic subunit	CAPN1
P17655;P17655-2	P17655;P1	6;4	6;4	6;4	Calpain-2 catalytic subunit	CAPN2
E7EQ12;E7EQA0;H0Y7F	E7EQ12;E7	5;5;5;5;5;5	5;5;5;5;5;5	5;5;5;5;5;5	Calpastatin	CAST
B4DDF4;B4DUT8;Q9943	B4DDF4;B4	8;8;8;7;6;5	8;8;8;7;6;5	8;8;8;7;6;5	Calponin;Calponin-2	CNN2
P27797;K7EJB9;K7EL50	P27797;K7I	15;10;3	15;10;3	15;10;3	Calreticulin	CALR
O43852-10;O43852-6;C	O43852-10	3;3;3;3;2;2	3;3;3;3;2;2	3;3;3;3;2;2	Calumenin	CALU
P10644;K7EM13;P1064	P10644;K7I	3;2;2;1	3;2;2;1	3;2;2;1	cAMP-dependent protein ki	PRKAR1A
H7C1L0;P13861-2;P138	H7C1L0;P1	1;1;1	1;1;1	1;1;1	cAMP-dependent protein ki	PRKAR2A
G3V153;Q14444-2;Q144	G3V153;Q1	4;4;4;3	4;4;4;3	4;4;4;3	Caprin-1	CAPRIN1
P00918;E5RID5;E5RK37	P00918	4;1;1	4;1;1	4;1;1	Carbonic anhydrase 2	CA2
P16152;E9PQ63;A8MTN	P16152;E9I	12;6;6;6;2	12;6;6;6;2	12;6;6;6;2	Carbonyl reductase [NADPH	CBR1
O75976;O75976-2;J3QC	O75976;O7	8;5;2	8;5;2	8;5;2	Carboxypeptidase D	CPD
P50416-2;P50416;H3BP	P50416-2;F	9;9;2;2;1;1	9;9;2;2;1;1	9;9;2;2;1;1	Carnitine O-palmitoyltransfe	CPT1A
P23786;A0A1B0GTB8;A	P23786;A0	6;5;5;5;4;1	6;5;5;5;4;1	6;5;5;5;4;1	Carnitine O-palmitoyltransfe	CPT2
D6REM4;E7ETM0;P4877	D6REM4;E7	3;3;3;3;3;2	3;3;3;3;3;2	3;3;3;3;3;2	Casein kinase I isoform alph	CSNK1A1
E7EU96;Q8NEV1;P6840	E7EU96;Q8	2;2;2;1;1	2;2;2;1;1	2;2;2;1;1	Casein kinase II subunit alph	CSNK2A1;C
Q5SRQ3;Q5SRQ6;N0E47	Q5SRQ3;Q5	2;2;2;2;2;1	2;2;2;2;2;1	2;2;2;2;2;1	Casein kinase II subunit beta	CSNK2B-LY
F5GX99;H0YGM0;Q9H0	F5GX99;H0	2;2;2;2;2;2	2;2;2;2;2;2	2;2;2;2;2;2	Caseinolytic peptidase B pro	CLPB
Q9BXW7-2;Q9BXW7	Q9BXW7-2	2;2	2;2	2;2	Cat eye syndrome critical re	CECR5
P04040	P04040	9	9	9	Catalase	CAT
P21964-2;P21964;E7EM	P21964-2;F	6;6;4;3;2;1	6;6;4;3;2;1	6;6;4;3;2;1	Catechol O-methyltransfera	COMT
P35221;P35221-2;G3XA	P35221;P35	21;21;18;1	21;21;18;1	21;21;18;1	Catenin alpha-1	CTNNA1
B4DGU4;P35222	B4DGU4;P3	7;7	5;5	5;5	Catenin beta-1	CTNNB1
C9JZR2;O60716-21;O60	C9JZR2;O6(	14;14;14;14	14;14;14;14	14;14;14;14	Catenin delta-1	CTNND1

A0A1B0GW44;A0A1B0(	A0A1B0GW	6;6;6;6;6;6	6;6;6;6;6;6	6;6;6;6;6;6	Cathepsin D;Cathepsin D lig	CTSD
P20645;H0YGT2;F5GX3	P20645;H0	3;2;2;1;1;1	3;2;2;1;1;1	3;2;2;1;1;1	Cation-dependent mannose	M6PR
P11717	P11717	8	8	8	Cation-independent manno	IGF2R
Q03135;E9PCT5;C9JKI3;	Q03135;E9	8;6;5;5;1	8;6;5;5;1	8;6;5;5;1	Caveolin-1;Caveolin	CAV1
Q03701	Q03701	2	2	2	CCAAT/enhancer-binding pi	CEBPZ
Q6YHK3-2;Q6YHK3;Q6Y	Q6YHK3-2;	3;3;2;2	3;3;2;2	3;3;2;2	CD109 antigen	CD109
H7C543;F5GXJ9;Q1374	H7C543;F5	3;3;3;3;3	3;3;3;3;3	3;3;3;3;3	CD166 antigen	ALCAM
A0A0C4DGH0;Q5ZPR3-	A0A0C4DG	2;2;2;2;1;1	2;2;2;2;1;1	2;2;2;2;1;1	CD276 antigen	CD276
H0YD13;H0Y2P0;P1607	H0YD13;H	6;6;6;6;6;6	6;6;6;6;6;6	6;6;6;6;6;6	CD44 antigen	CD44
E9PNW4;E9PR17;P1398	E9PNW4;E	3;3;3;1	3;3;3;1	3;3;3;1	CD59 glycoprotein	CD59
P27701-2;P27701;E9PJ	P27701-2;F	3;3;1	3;3;1	3;3;1	CD82 antigen	CD82
P48960-2;P48960-3;P48	P48960-2;F	3;3;3	3;3;3	3;3;3	CD97 antigen;CD97 antigen	CD97
I3L1N9;Q8N5K1	I3L1N9;Q8I	2;2	2;2	2;2	CDGSH iron-sulfur domain-	CISD2
B3KY94;O14735-3;O14	B3KY94;O1	2;2;2;1	2;2;2;1	2;2;2;1	CDP-diacylglycerol--inositol	CDIPT
Q8N163-2;Q8N163;G3	Q8N163-2;	4;4;3;3;2;1	4;4;3;3;2;1	4;4;3;3;2;1	Cell cycle and apoptosis reg	CCAR2
P60953;Q5JYX0;P60953	P60953;Q5	5;4;4	4;3;3	4;3;3	Cell division control protein	CDC42
Q99459	Q99459	4	4	4	Cell division cycle 5-like pro	CDC5L
Q9NX58;D6RDJ1	Q9NX58;D	2;1	2;1	2;1	Cell growth-regulating nucl	LYAR
P62633-2;P62633;P626	P62633-2;F	5;5;4;4;4;4	5;5;4;4;4;4	1;1;1;1;0;0	Cellular nucleic acid-binding	CNBP
Q5JVD3;Q7Z7A1-5;Q7Z	Q5JVD3;Q7	1;1;1	1;1;1	1;1;1	Centriolin	CNTRL
Q7Z7K6-2;Q7Z7K6-3;Q7	Q7Z7K6-2;	2;2;2	2;2;2	2;2;2	Centromere protein V	CENPV
A0A1B0GW73;H3BNF1;	A0A1B0GW	2;2;2;2;2;2	2;2;2;2;2;2	2;2;2;2;2;2	Ceroid-lipofuscinosis neuro	CLN6
M0R1L7;M0R1T5;O436	M0R1L7;M	1;1;1	1;1;1	1;1;1	Charged multivesicular bod	CHMP2A
Q9H444;Q96CF2	Q9H444	5;1	5;1	5;1	Charged multivesicular bod	CHMP4B
O00299	O00299	9	9	9	Chloride intracellular chann	CLIC1
Q8WWI5-3;Q8WWI5-2;	Q8WWI5-3	3;3;3	3;3;3	3;3;3	Choline transporter-like pro	SLC44A1
Q8IWA5-3;Q8IWA5;Q8I	Q8IWA5-3;	1;1;1	1;1;1	1;1;1	Choline transporter-like pro	SLC44A2
A0A0C4DGS9;Q9Y6K0	A0A0C4DG	1;1	1;1	1;1	Choline/ethanolaminephos	CEPT1
H7BZN1;H7C1T3;C9J05	H7BZN1;H7	1;1;1;1;1;1	1;1;1;1;1;1	1;1;1;1;1;1	Choline-phosphate cytidylyl	PCYT1A;PC
A0A087X1B7;X6R700;Q	A0A087X1E	1;1;1;1;1	1;1;1;1;1	1;1;1;1;1	Chromatin target of PRMT1	CHTOP
Q13185;S4R2Y4;C9JMN	Q13185;S4	6;3;2;2;1;1	6;3;2;2;1;1	6;3;2;2;1;1	Chromobox protein homolo	CBX3
F5GWX5;A0A0C4DGG9;	F5GWX5;A	2;2;2;2;1;1	2;2;2;2;1;1	2;2;2;2;1;1	Chromodomain-helicase-DN	CHD4;CHD
B4DJV2;O75390;A0A0C	B4DJV2;O7	9;9;8;5;3;3	9;9;8;5;3;3	8;8;8;5;2;2	Citrate synthase;Citrate syn	CS
A0A087WVQ6;Q00610-	A0A087WV	46;46;46;1	46;46;46;1	35;35;35;1	Clathrin heavy chain;Clathri	CLTC
P09496-2;F8WF69;P094	P09496-2;F	3;2;2;2;2;2	3;2;2;2;2;2	3;2;2;2;2;2	Clathrin light chain A	CLTA
P09497-2;P09497	P09497-2;F	2;2	2;2	2;2	Clathrin light chain B	CLTB
K7EP40;F5H496;I3L3X1;	K7EP40;F5I	2;2;2;2;2;1	2;2;2;2;2;1	2;2;2;2;2;1	Claudin;Claudin-7	CLDN7
O95832	O95832	3	3	3	Claudin-1	CLDN1
P56749	P56749	1	1	1	Claudin-12	CLDN12
O14493;O95484;P5674	O14493;O9	2;1;1;1	2;1;1;1	2;1;1;1	Claudin-4;Claudin-9;Claudin	CLDN4;CLD
O43809;H3BV41;H3BN	O43809;H3	3;2;2	3;2;2	3;2;2	Cleavage and polyadenylati	NUDT21
F8WJN3;Q16630-3;Q16	F8WJN3;Q	4;4;4;4;1	4;4;4;4;1	4;4;4;4;1	Cleavage and polyadenylati	CPSF6
J3QT54;F5H6M0;F5H04	J3QT54;F5	1;1;1;1;1;1	1;1;1;1;1;1	1;1;1;1;1;1	Cleavage and polyadenylati	CPSF7
A0A0A0MT56;E9PID8;E	A0A0A0MT	1;1;1;1;1	1;1;1;1;1	1;1;1;1;1	Cleavage stimulation factor	CSTF2
O96005;O96005-4;K7E	O96005;O9	5;4;3;2;1	5;4;3;2;1	5;4;3;2;1	Cleft lip and palate transme	CLPTM1
G5E9Z2;Q96KA5-2;Q96	G5E9Z2;Q9	3;3;3	3;3;3	3;3;3	Cleft lip and palate transme	CLPTM1L
I3L2B0;K7EIG1;O75153	I3L2B0;K7E	3;2;2	3;2;2	3;2;2	Clustered mitochondria pro	CLUH
Q14019;H3BT58	Q14019	5;2	5;2	5;2	Coactosin-like protein	COTL1
Q9NQP5;A6PVK5;P004	Q9NQP5;A	1;1;1	1;1;1	1;1;1	Coagulation factor XIII A ch	F13A1
P53621;P53621-2	P53621;P5	10;10	10;10	10;10	Coatomer subunit alpha;Xe	COPA

P53618;E9PP73	P53618	5;2	5;2	5;2	Coatomer subunit beta	COPB1
P35606-2;P35606;D6R9	P35606-2;F	10;10;3;2;2	10;10;3;2;2	10;10;3;2;2	Coatomer subunit beta	COPB2
P48444;B0YIW6;Q6P1Q	P48444;B0	7;6;4;4;2	7;6;4;4;2	7;6;4;4;2	Coatomer subunit delta	ARCN1
M0QXB4;O14579;O145	M0QXB4;O	3;3;2;2;1	3;3;2;2;1	3;3;2;2;1	Coatomer subunit epsilon	COPE
Q9Y678;H0Y8X7	Q9Y678	4;1	4;1	4;1	Coatomer subunit gamma-1	COPG1
P23528;G3V1A4;E9PP5(	P23528;G3	14;13;13;1:	14;13;13;1:	13;12;12;1:	Cofilin-1	CFL1
Q68DW7;Q8WVM7-2;C	Q68DW7;C	1;1;1	1;1;1	1;1;1	Cohesin subunit SA-1	DKFZp781D
Q96A33-2;Q96A33	Q96A33-2;(	5;5	5;5	5;5	Coiled-coil domain-containi	CCDC47
Q96ER9-2;Q96ER9	Q96ER9-2;(	1;1	1;1	1;1	Coiled-coil domain-containi	CCDC51
C9JQ41;H7C525;Q4VC3	C9JQ41;H7	1;1;1	1;1;1	1;1;1	Coiled-coil domain-containi	CCDC58
H0YG79;Q9H6F5	H0YG79;Q9	1;1	1;1	1;1	Coiled-coil domain-containi	CCDC86
Q9Y6H1;Q5T1J5	Q9Y6H1;Q5	3;2	3;2	3;2	Coiled-coil-helix-coiled-coil-	CHCHD2;CH
O75534-2;O75534;O75!	O75534-2;(	5;5;5;5;4;1	5;5;5;5;4;1	5;5;5;5;4;1	Cold shock domain-containi	CSDE1
Q9UMD9-2;Q9UMD9;A	Q9UMD9-2	11;11;8	11;11;8	11;11;8	Collagen alpha-1(XVII) chair	COL17A1
Q07021;I3L3Q7;I3L3B0	Q07021;I3L	8;6;6	8;6;6	8;6;6	Complement component 1	C1QBP
Q15021	Q15021	1	1	1	Condensin complex subunit	NCAPD2
E9PHA2;Q15003;C9J47(	E9PHA2;Q1	4;4;3;3;2;1	4;4;3;3;2;1	4;4;3;3;2;1	Condensin complex subunit	NCAPH
Q9BPX3;H0Y9Z8	Q9BPX3	3;1	3;1	3;1	Condensin complex subunit	NCAPG
Q96JB2-2;Q96JB2	Q96JB2-2;(	1;1	1;1	1;1	Conserved oligomeric Golgi	COG3
Q9NZB2;Q9NZB2-6;Q9N	Q9NZB2;Q9	8;8;6;4;2;2	8;8;6;4;2;2	8;8;6;4;2;2	Constitutive coactivator of I	FAM120A
C9JFE4;A0A096LP07;A0	C9JFE4;A0/	2;2;2;2;2;2	2;2;2;2;2;2	2;2;2;2;2;2	COP9 signalosome complex	GPS1
D6RAX7;Q9BT78-2;Q9B	D6RAX7;Q9	2;2;2;1;1	2;2;2;1;1	2;2;2;1;1	COP9 signalosome complex	COPS4
E9PGT6;Q99627-2;Q99(	E9PGT6;Q9	2;2;2;1	2;2;2;1	2;2;2;1	COP9 signalosome complex	COPS8
O75131;A0A087WYQ3;.	O75131;A0	10;5;3;3;3;:	10;5;3;3;3;:	9;5;3;3;3;3	Copine-3	CPNE3
E7ENV7;Q8IYJ1;Q86YQ8	E7ENV7;Q8	2;2;2;2;1;1	1;1;1;1;1;1	1;1;1;1;1;1	Copine-9;Copine-8;Copine-!	CPNE8;CPN
O75367-2;O75367-3;O7	O75367-2;(	10;10;10;8;	10;10;10;8;	9;9;9;7;1	Core histone macro-H2A.1;I	H2AFY
I3L1U7;I3L0X9;I3L3T0;P	I3L1U7;I3L(	1;1;1;1;1;1	1;1;1;1;1;1	1;1;1;1;1;1	Coronin;Mitochondrial imp(	PAM16;CO
Q9BR76	Q9BR76	2	2	2	Coronin-1B	CORO1B
Q9ULV4;Q9ULV4-2;Q9L	Q9ULV4;Q9	6;6;6;3;3;3	6;6;6;3;3;3	6;6;6;3;3;3	Coronin-1C;Coronin	CORO1C
Q92828	Q92828	1	1	1	Coronin-2A	CORO2A
Q5SZC9;Q9P1F3	Q5SZC9;Q9	1;1	1;1	1;1	Costars family protein ABRA	ABRACL
P78310-2;P78310-6;P78	P78310-2;F	4;4;4;3;2	4;4;4;3;2	4;4;4;3;2	Coxsackievirus and adenovi	CXADR
P12532;P12532-2;C9JJX	P12532;P1:	3;3;1;1;1;1	3;3;1;1;1;1	3;3;1;1;1;1	Creatine kinase U-type, mit(	CKMT1A
P46109	P46109	2	2	2	Crk-like protein	CRKL
X6RI56;O94886	X6RI56;O9	1;1	1;1	1;1	CSC1-like protein 1	TMEM63A
Q5SQP8;P56545;P5654!	Q5SQP8;P5	2;2;2;2	2;2;2;2	2;2;2;2	C-terminal-binding protein :	CTBP2
P17812;P17812-2	P17812	5;2	5;2	5;2	CTP synthase 1	CTPS1
Q9H5V8;Q9H5V8-2;Q9H	Q9H5V8;Q9	3;2;1	3;2;1	3;2;1	CUB domain-containing pro	CDCP1
G5EA30;Q92879-5;Q92!	G5EA30;Q9	1;1;1;1;1;1	1;1;1;1;1;1	1;1;1;1;1;1	CUGBP Elav-like family men	CELF1
K4DI93;Q13620-3;Q136	K4DI93;Q1:	1;1;1;1	1;1;1;1	1;1;1;1	Cullin-4B	CUL4B
Q86VP6;Q86VP6-2;A0A	Q86VP6;Q8	7;6;5;1;1	7;6;5;1;1	7;6;5;1;1	Cullin-associated NEDD8-di:	CAND1
A0A024QZP7;P06493;E!	A0A024QZI	5;5;4;4;4;1	4;4;3;3;4;0	4;4;3;3;4;0	Cyclin-dependent kinase 1	CDC2;CDK1
Q00534	Q00534	6	6	5	Cyclin-dependent kinase 6	CDK6
P01040	P01040	1	1	1	Cystatin-A;Cystatin-A, N-ter	CSTA
P04080;A0A1W2PS52	P04080;A0.	3;2	3;2	3;2	Cystatin-B	CSTB
P01034	P01034	1	1	1	Cystatin-C	CST3
P21291;E9PS42;E9PP21	P21291;E9I	6;4;4;3	6;4;4;3	6;4;4;3	Cysteine and glycine-rich pr	CSRP1
Q9UHD1;E9PIZ4;E9PSD!	Q9UHD1;E!	2;1;1;1;1;1	2;1;1;1;1;1	2;1;1;1;1;1	Cysteine and histidine-rich (	CHORDC1
Q9H1C7	Q9H1C7	1	1	1	Cysteine-rich and transmen	CYSTM1
Q9NZV1;H7C1Z0;H7C2T	Q9NZV1	3;1;1	3;1;1	3;1;1	Cysteine-rich motor neuron	CRIM1

H0YFA4;P52943;P52943	H0YFA4;P5	1;1;1	1;1;1	1;1;1	Cysteine-rich protein 2	CRIP2
Q6UXH1-4;Q6UXH1;Q6	Q6UXH1-4;	1;1;1;1	1;1;1;1	1;1;1;1	Cysteine-rich with EGF-like	CRELD2
P00167-2;P00167-3;P0(	P00167-2;F	1;1;1	1;1;1	1;1;1	Cytochrome b5	CYB5A
H3BUX2;J3KNF8;O4316	H3BUX2;J3	2;2;2;1	2;2;2;1	2;2;2;1	Cytochrome b5 type B	CYB5B
P31930	P31930	14	14	13	Cytochrome b-c1 complex s	UQCRC1
P22695;H3BRG4;H3BSJ!	P22695;H3	13;12;11;9;	13;12;11;9;	13;12;11;9;	Cytochrome b-c1 complex s	UQCRC2
P07919;A0A096LP55	P07919	3;1	3;1	3;1	Cytochrome b-c1 complex s	UQCRH
P14927;E5RHG9;B7Z2R:	P14927;E5I	2;1;1;1	2;1;1;1	2;1;1;1	Cytochrome b-c1 complex s	UQCRB
Q9UDW1	Q9UDW1	1	1	1	Cytochrome b-c1 complex s	UQCR10
P47985;P0C7P4	P47985;P0(	5;4	5;4	5;4	Cytochrome b-c1 complex s	UQCRFS1;U
C9JFR7;P99999;CON__F	C9JFR7;P9!	2;2;1	2;2;1	2;2;1	Cytochrome c	CYCS
Q9Y2R0;K7EPV0	Q9Y2R0;K7	3;2	3;2	3;2	Cytochrome c oxidase asser	COA3
Q7KZN9-2;Q7KZN9	Q7KZN9-2;'	1;1	1;1	1;1	Cytochrome c oxidase asser	COX15
C9J8T6;Q14061	C9J8T6;Q1·	1;1	1;1	1;1	Cytochrome c oxidase copp	COX17
P00403	P00403	3	3	3	Cytochrome c oxidase subu	MT-CO2
P13073;Q86WV2;H3BN	P13073;Q8	4;3;3;3;2;1	4;3;3;3;2;1	4;3;3;3;2;1	Cytochrome c oxidase subu	COX4I1
H3BNX8;P20674;H3BRN	H3BNX8;P2	2;2;1;1	2;2;1;1	2;2;1;1	Cytochrome c oxidase subu	COX5A
P10606	P10606	5	5	5	Cytochrome c oxidase subu	COX5B
P14854	P14854	4	4	4	Cytochrome c oxidase subu	COX6B1
P09669	P09669	3	3	3	Cytochrome c oxidase subu	COX6C
D6RIE3;D6RGV5;H0UI0(	D6RIE3;D6I	2;2;2;2;1	2;2;2;2;1	2;2;2;2;1	Cytochrome c oxidase subu	COX7A2
O14548;E5RJZ1;H0YBD2	O14548;E5	2;1;1	2;1;1	2;1;1	Cytochrome c oxidase subu	COX7A2L
D6R9Z7;P15954	D6R9Z7;P1	2;2	2;2	2;2	Cytochrome c oxidase subu	COX7C
O00483	O00483	2	2	2	Cytochrome c oxidase subu	NDUFA4
P08574	P08574	2	2	2	Cytochrome c1, heme prote	CYC1
P53701	P53701	4	4	4	Cytochrome c-type heme ly	HCCS
Q14204;H0YJ21	Q14204	41;2	41;2	41;2	Cytoplasmic dynein 1 heavy	DYNC1H1
Q13409-6;Q13409-3;Q1	Q13409-6;(	3;3;3;3;3;3	3;3;3;3;3;3	3;3;3;3;3;3	Cytoplasmic dynein 1 interr	DYNC1I2
J3KRI4;O43237-2;O432:	J3KRI4;O43	3;3;3;2	3;3;3;2	3;3;3;2	Cytoplasmic dynein 1 light i	DYNC1LI2
Q7L576;A0A0G2JR96;A(	Q7L576;A0	4;3;3;2;2;2	4;3;3;2;2;2	4;3;3;2;2;2	Cytoplasmic FMR1-interacti	CYFIP1;CYF
Q07065	Q07065	22	22	22	Cytoskeleton-associated pr(	CKAP4
P28838-2;P28838;H0Y9	P28838-2;F	3;3;2	3;3;2	3;3;2	Cytosol aminopeptidase	LAP3
O00154-2;O00154-3;O(	O00154-2;(	2;2;2;1;1;1	2;2;2;1;1;1	2;2;2;1;1;1	Cytosolic acyl coenzyme A t	ACOT7
Q96KP4;Q96KP4-2;J3QF	Q96KP4;Q!	5;4;1;1	5;4;1;1	5;4;1;1	Cytosolic non-specific dipep	CNDP2
Q5JUV6;P49902-2;P499	Q5JUV6;P4	2;2;2	2;2;2	2;2;2	Cytosolic purine 5-nucleotic	NT5C2
A0A286YF22;A0A2C9F2	A0A286YF2	10;10;10;7;	10;10;10;7;	10;10;10;7;	D-3-phosphoglycerate dehy	PHGDH
K7EQ02;K7EQ55;K7EK3:	K7EQ02;K7	3;3;3;3;3	3;3;3;3;3	3;3;3;3;3	DAZ-associated protein 1	DAZAP1
Q5BKZ1-2;Q5BKZ1;E2QI	Q5BKZ1-2;(	2;2;1;1	2;2;1;1	2;2;1;1	DBIRD complex subunit ZNF	ZNF326
Q9H773	Q9H773	2	2	2	dCTP pyrophosphatase 1	DCTPP1
A0A087WT20;Q9NV06;	A0A087WT	2;2;1	2;2;1	2;2;1	DDB1- and CUL4-associated	DCAF13
P30046;J3KQ18;B5MC8	P30046;J3k	4;3;3;3;3;1	4;3;3;3;3;1	4;3;3;3;3;1	D-dopachrome decarboxyla	DDT;DDTL
A0A0A0MRX2;Q96HY6-	A0A0A0MF	2;2;2	2;2;2	2;2;2	DDRGK domain-containing		DDRGK1
Q9Y394-2;Q9Y394;H0YJ	Q9Y394-2;(	3;3;2;2;2	3;3;2;2;2	3;3;2;2;2	Dehydrogenase/reductase !	DHRS7
Q3LIE7;Q15392-2;Q153	Q3LIE7;Q1!	5;5;5;2	5;5;5;2	5;5;5;2	Delta(24)-sterol reductase	Nbla03646
Q13011;M0R248;M0QZ	Q13011;M(	4;3;2	4;3;2	3;2;1	Delta(3,5)-Delta(2,4)-dieno	ECH1
P30038-3;P30038;Q5TF	P30038-3;F	2;2;1;1	2;2;1;1	2;2;1;1	Delta-1-pyrroline-5-carboxy	ALDH4A1
P54886-2;P54886	P54886-2;F	15;15	15;15	15;15	Delta-1-pyrroline-5-carboxy	ALDH18A1
O43583	O43583	1	1	1	Density-regulated protein	DENR
J3KP30;Q5QJE6;E9PRB3	J3KP30;Q5(	3;3;1	3;3;1	3;3;1	Deoxynucleotidyltransferas	DNTTIP2
G3V158;E9PPM8;Q9Y3:	G3V158;E9	1;1;1	1;1;1	1;1;1	Deoxyribose-phosphate ald	DERA

A0A0C4DGL3;H0YNW5;	A0A0C4DG	5;5;5;5;4;3	5;5;5;5;4;3	5;5;5;5;4;3	Deoxyuridine 5-triphosphat	DUT
E5RGY0;B4E1G1;Q9BUI	E5RGY0;B4	1;1;1;1	1;1;1;1	1;1;1;1	Derlin-1	DERL1
I3L1T3;Q9GZP9	I3L1T3;Q9(	1;1	1;1	1;1	Derlin-2	DERL2
Q02487-2;Q02487	Q02487-2;(	1;1	1;1	1;1	Desmocollin-2	DSC2
Q14574-2;Q14574;J3QI	Q14574-2;(	4;4;1	4;4;1	4;4;1	Desmocollin-3	DSC3
Q14126	Q14126	12	12	12	Desmoglein-2	DSG2
P32926	P32926	12	12	12	Desmoglein-3	DSG3
P15924;P15924-3;P159	P15924;P1!	46;40;39	46;40;39	46;40;39	Desmoplakin	DSP
P60981;F6RFD5;P60981	P60981;F6I	8;7;7	7;6;6	7;6;6	Destrin	DSTN
Q9Y295	Q9Y295	2	2	2	Developmentally-regulated	DRG1
P09622;E9PEX6;P09622	P09622;E9I	5;4;4;4;1;1	5;4;4;4;1;1	5;4;4;4;1;1	Dihydrolipoyl dehydrogenas	DLD
P10515;H0YDD4;E9PEJ4	P10515;H0	5;4;4	5;4;4	5;4;4	Dihydrolipoyllysine-residue	DLAT
P36957;P36957-2;Q86S	P36957;P3(	8;7;5;1;1;1	8;7;5;1;1;1	8;7;5;1;1;1	Dihydrolipoyllysine-residue	DLST
G3V1D3;G3V180;Q9NY:	G3V1D3;G:	1;1;1;1	1;1;1;1	1;1;1;1	Dipeptidyl peptidase 3	DPP3
O14672	O14672	2	2	2	Disintegrin and metalloprot	ADAM10
F8WC54;A0AVL1;Q1344	F8WC54;A(	5;5;5;5	5;5;5;5	5;5;5;5	Disintegrin and metalloprot	ADAM9
Q05BS2;Q96SL1-2;Q96S	Q05BS2;Q(	1;1;1	1;1;1	1;1;1	Disrupted in renal carcinom	DIRC2
Q16531;F5GY55;F5H2L:	Q16531;F5	9;8;3;3;2;2	9;8;3;3;2;2	9;8;3;3;2;2	DNA damage-binding prote	DDB1
Q6UX65	Q6UX65	1	1	1	DNA damage-regulated aut	DRAM2
B0QYD3;Q9UH17-3;Q9I	B0QYD3;Q(	5;5;5;3;2;2	5;5;5;3;2;2	5;5;5;3;2;2	DNA dC->dU-editing enzym	APOBEC3B
B1AHB1;P33992;B1AHB	B1AHB1;P3	5;5;1;1	5;5;1;1	5;5;1;1	DNA helicase;DNA replicati	MCM5
E9PHA6;P43246-2;P432	E9PHA6;P4	3;3;3;1	3;3;3;1	3;3;3;1	DNA mismatch repair prote	MSH2
P52701-4;P52701-3;P52	P52701-4;F	4;4;4;3;3;1	4;4;4;3;3;1	4;4;4;3;3;1	DNA mismatch repair prote	MSH6
E5RFF9;Q9BRT9	E5RFF9;Q9	1;1	1;1	1;1	DNA replication complex GI	GINS4
H0Y8E6;P49736	H0Y8E6;P4	12;12	12;12	12;12	DNA replication licensing fa	MCM2
P25205;P25205-2;J3KQ	P25205;P2!	8;7;6;1	8;7;6;1	8;7;6;1	DNA replication licensing fa	MCM3
P33991;E5RG31;E5RFJ8	P33991	11;3;2	11;3;2	11;3;2	DNA replication licensing fa	MCM4
Q14566	Q14566	8	8	8	DNA replication licensing fa	MCM6
P33993;P33993-3;P339	P33993;P3:	16;14;8;4	16;14;8;4	16;14;8;4	DNA replication licensing fa	MCM7
P11387;E5RIC7;Q969P6	P11387	14;1;1;1	14;1;1;1	14;1;1;1	DNA topoisomerase 1	TOP1
P11388;P11388-2;P113	P11388;P1	14;14;14;1	14;14;14;1	14;14;14;1	DNA topoisomerase 2-alpha	TOP2A
G3V5Q1;G3V3M6;P276	G3V5Q1;G:	5;5;5;4;4;4	5;5;5;4;4;4	5;5;5;4;4;4	DNA-(apurinic or apyrimidir	APEX1
P78527;P78527-2	P78527;P7	56;55	56;55	56;55	DNA-dependent protein kin	PRKDC
O95602;B9ZVN9	O95602;B9	2;1	2;1	2;1	DNA-directed RNA polymer	POLR1A
O15446;O15446-2	O15446;O1	2;2	2;2	2;2	DNA-directed RNA polymer	CD3EAP
P24928-2;P24928	P24928-2;F	1;1	1;1	1;1	DNA-directed RNA polymer	POLR2A
K7EMH3;O00411	K7EMH3;O	1;1	1;1	1;1	DNA-directed RNA polymer	POLRMT
C9J2Y9;P30876;C9J4M6	C9J2Y9;P3(	2;2;1;1	2;2;1;1	2;2;1;1	DNA-directed RNA polymer	POLR2B
O15160-2;O15160;D6RI	O15160-2;(	2;2;1;1;1	2;2;1;1;1	2;2;1;1;1	DNA-directed RNA polymer	POLR1C
A0A087WVZ9;A0A0A0N	A0A087WV	1;1;1	1;1;1	1;1;1	DNA-directed RNA polymer	POLR2E
P31689-2;P31689	P31689-2;F	3;3	3;3	3;3	DnaJ homolog subfamily A r	DNAJA1
O60884;A0A087WT48;I	O60884;A0	4;3;1	4;3;1	4;3;1	DnaJ homolog subfamily A r	DNAJA2
Q96EY1-2;Q96EY1;Q96I	Q96EY1-2;(	3;3;2	3;3;2	3;3;2	DnaJ homolog subfamily A r	DNAJA3
P25685;M0R080;P2568	P25685;M(	4;3;3;1;1;1	4;3;3;1;1;1	4;3;3;1;1;1	DnaJ homolog subfamily B r	DNAJB1
Q9UBS4;H7C2Y5	Q9UBS4;H7	4;2	4;2	4;2	DnaJ homolog subfamily B r	DNAJB11
V9GY70;V9GYN7;J3KPS(	V9GY70;V9	1;1;1;1;1	1;1;1;1;1	1;1;1;1;1	DnaJ homolog subfamily B r	DNAJB12
Q9NVH1-3;Q9NVH1;Q9	Q9NVH1-3;	4;4;2	4;4;2	4;4;2	DnaJ homolog subfamily C r	DNAJC11
H0YA63;O75165	H0YA63;O7	1;1	1;1	1;1	DnaJ homolog subfamily C r	DNAJC13
X6R9L0;Q13217	X6R9L0;Q1	3;3	3;3	3;3	DnaJ homolog subfamily C r	DNAJC3
Q9H3Z4	Q9H3Z4	1	1	1	DnaJ homolog subfamily C r	DNAJC5

Q99615-2;Q99615;K7E(	Q99615-2;(	4;4;1;1;1;1	4;4;1;1;1;1	4;4;1;1;1;1	DnaJ homolog subfamily C r	DNAJC7
Q8WXX5	Q8WXX5	1	1	1	DnaJ homolog subfamily C r	DNAJC9
Q5QPK2;H0Y368;O6076	Q5QPK2;H(	3;3;3;1	3;3;3;1	3;3;3;1	Dolichol-phosphate manno:	DPM1
Q9P2X0;Q9P2X0-2	Q9P2X0;Q9	2;2	2;2	2;2	Dolichol-phosphate manno:	DPM3
A0A0C4DGS1;P39656;P	A0A0C4DG	8;8;7;6;5	8;8;7;6;5	8;8;7;6;5	Dolichyl-diphosphooligosac	DDOST
P04843;B7Z4L4;F8WF3:	P04843;B7.	24;18;5	24;18;5	24;18;5	Dolichyl-diphosphooligosac	RPN1
P04844;P04844-2;Q5JYI	P04844;P0	11;10;3;2;2	11;10;3;2;2	11;10;3;2;2	Dolichyl-diphosphooligosac	RPN2
F5GXX5;P61803;F5H89!	F5GXX5;P6	4;4;2;2	4;4;2;2	4;4;2;2	Dolichyl-diphosphooligosac	DAD1
P46977;P46977-2;A0A0	P46977;P4(	5;4;1;1;1	5;4;1;1;1	5;4;1;1;1	Dolichyl-diphosphooligosac	STT3A
Q8TCJ2	Q8TCJ2	2	2	2	Dolichyl-diphosphooligosac	STT3B
Q9Y673-2;Q9Y673	Q9Y673-2;(	1;1	1;1	1;1	Dolichyl-phosphate beta-glu	ALG5
A0A087X1A5;Q5JW30;(	A0A087X1/	2;2;2;2;2	2;2;2;2;2	2;2;2;2;2	Double-stranded RNA-bindi	STAU1
H0YCK3;P55265-5;P552	H0YCK3;P5	3;3;3;3;3;3	3;3;3;3;3;3	3;3;3;3;3;3	Double-stranded RNA-speci	ADAR
A8MY26;A8MTY9;O149	A8MY26;A8	1;1;1;1	1;1;1;1	1;1;1;1	Down syndrome critical reg	DSCR3
H0Y5J4;B4DDD6;Q9UJU	H0Y5J4;B4I	3;3;3;3;3;3	3;3;3;3;3;3	3;3;3;3;3;3	Drebrin-like protein	DBNL
Q02750-2;Q02750	Q02750-2;(	1;1	1;1	1;1	Dual specificity mitogen-act	MAP2K1
P36507	P36507	1	1	1	Dual specificity mitogen-act	MAP2K2
P46734-2;P46734;P467.	P46734-2;F	3;3;3;2;1;1	3;3;3;2;1;1	3;3;3;2;1;1	Dual specificity mitogen-act	MAP2K3
E7EX90;Q14203-5;Q142	E7EX90;Q1	2;2;2;2;2;2	2;2;2;2;2;2	2;2;2;2;2;2	Dynactin subunit 1	DCTN1;DKF
G8JLD5;O00429-4;O004	G8JLD5;O0	3;3;3;3;3;3	3;3;3;3;3;3	3;3;3;3;3;3	Dynamin-1-like protein	DNM1L
E5KLJ9;O60313-13;O60.	E5KLJ9;O6(	3;3;3;3;3;3	3;3;3;3;3;3	3;3;3;3;3;3	Dynamin-like 120 kDa prote	OPA1
A0A075B6F3;Q96JB1-2;	A0A075B6F	1;1;1	1;1;1	1;1;1	Dynein heavy chain 8, axon	DNAH8
P63167;F8VXI7;F8VXL2;	P63167	3;1;1;1	1;1;1;1	1;1;1;1	Dynein light chain 1, cytopla	DYNLL1
Q96FJ2	Q96FJ2	3	3	1	Dynein light chain 2, cytopla	DYNLL2
Q9NP97;B1AKR6;Q8TF(	Q9NP97;B1	3;2;2;1;1;1	3;2;2;1;1;1	3;2;2;1;1;1	Dynein light chain roadbloc	DYNLRB1;D
Q6P0N6;E7ETB9;E9PHN	Q6P0N6;E7	1;1;1;1;1;1	1;1;1;1;1;1	1;1;1;1;1;1	Dystonin	DST
Q14118	Q14118	2	2	2	Dystroglycan;Alpha-dystrog	DAG1
P49792;J3KNE0;Q7Z3J3	P49792	22;5;5;5;4;	22;5;5;5;4;	21;5;5;5;4;	E3 SUMO-protein ligase Rar	RANBP2
Q14258	Q14258	7	7	7	E3 ubiquitin/ISG15 ligase TF	TRIM25
H3BUD0;H3BS86;Q9UN	H3BUD0;H:	1;1;1;1	1;1;1;1	1;1;1;1	E3 ubiquitin-protein ligase (	STUB1
Q7Z6Z7-2;Q7Z6Z7-3;Q7	Q7Z6Z7-2;(	5;5;5;1	5;5;5;1	5;5;5;1	E3 ubiquitin-protein ligase I	HUWE1
F8WCD0;Q8NC42	F8WCD0;Q	3;3	3;3	3;3	E3 ubiquitin-protein ligase F	RNF149
Q5JVY0;Q13049	Q5JVY0;Q1	1;1	1;1	1;1	E3 ubiquitin-protein ligase T	TRIM32
Q9C037-3;Q9C037-2;Q9	Q9C037-3;(	2;2;2	2;2;2	2;2;2	E3 ubiquitin-protein ligase T	TRIM4
A0A087WVR3;A0A087V	A0A087WV	3;3;3;3;2	3;3;3;3;2	3;3;3;3;2	E3 ubiquitin-protein ligase U	UHRF1
Q96C19;H0Y4Y4;A0A08	Q96C19;H(	7;4;1;1;1	7;4;1;1;1	7;4;1;1;1	EF-hand domain-containing	EFHD2
O43854-2;O43854	O43854-2;(	2;2	2;2	2;2	EGF-like repeat and discoidi	EDIL3
C9J2Z4;A0A024R571;Q9	C9J2Z4;A0/	2;2;2	2;2;2	1;1;1	EH domain-containing prote	EHD1
Q9H223;Q9NZN3-2	Q9H223	6;1	6;1	5;0	EH domain-containing prote	EHD4
Q15717;Q15717-2;M0(	Q15717;Q1	7;7;4;2	7;7;4;2	7;7;4;2	ELAV-like protein 1	ELAVL1
H0YKF0;H0YLU7;P1380	H0YKF0;H0	6;6;6;5;5;4	6;6;6;5;5;4	6;6;6;5;5;4	Electron transfer flavoprote	ETFA
P38117;P38117-2;M0Q	P38117;P3	10;10;5	10;10;5	10;10;5	Electron transfer flavoprote	ETFB
Q05639	Q05639	9	1	1	Elongation factor 1-alpha 2	EEF1A2
P24534;F2Z2G2;C9JZW:	P24534	5;2;2;1	5;2;2;1	5;2;2;1	Elongation factor 1-beta	EEF1B2
E9PK01;P29692;A0A08	E9PK01;P2	8;8;7;7;7;6	8;8;7;7;7;6	1;1;1;1;1;1	Elongation factor 1-delta	EEF1D
P29692-3;E9PL71;E9PN	P29692-3;E	8;6;5	1;1;1	1;1;1	Elongation factor 1-delta	EEF1D
P26641;P26641-2	P26641;P2(	16;15	16;15	16;15	Elongation factor 1-gamma	EEF1G
P13639	P13639	34	34	33	Elongation factor 2	EEF2
C9IZ01;Q96RP9;Q96RP9	C9IZ01;Q9(	5;5;5;4	5;5;5;4	5;5;5;4	Elongation factor G, mitoch	GFM1
P43897;P43897-2;C9JG:	P43897;P4:	3;2;1;1;1;1	3;2;1;1;1;1	3;2;1;1;1;1	Elongation factor Ts, mitoch	TSFM

P49411;H3BNU3	P49411	19;1	19;1	19;1	Elongation factor Tu, mitocl	TUFM
Q9BW60	Q9BW60	1	1	1	Elongation of very long chai	ELOVL1
A0A0A0MTI6;Q9NYP7-3	A0A0A0MT	1;1;1;1	1;1;1;1	1;1;1;1	Elongation of very long chai	ELOVL5
F5H2T0;O95163	F5H2T0;O9	1;1	1;1	1;1	Elongator complex protein 1	IKBKAP
P50402;Q5HY57	P50402;Q5	5;3	5;3	5;3	Emerin	EMD
O94919	O94919	3	3	3	Endonuclease domain-conta	ENDOD1
M0QYE0;M0R0I3;Q999(	M0QYE0;M	1;1;1;1	1;1;1;1	1;1;1;1	Endophilin-A2	SH3GL1
Q9NZ08;Q9NZ08-2;D6R	Q9NZ08;Q9	7;7;2;1	7;7;2;1	7;7;2;1	Endoplasmic reticulum amii	ERAP1
E7ER77;Q7Z2K6;A0A0C	E7ER77;Q7	4;4;1;1	4;4;1;1	4;4;1;1	Endoplasmic reticulum met	ERMP1
P30040;F8VY02;P30040	P30040;F8	7;5;1	7;5;1	6;5;1	Endoplasmic reticulum resic	ERP29
Q9BS26	Q9BS26	6	6	6	Endoplasmic reticulum resic	ERP44
H0Y621;H0Y5K5;Q9Y28	H0Y621;H0	3;3;3;3;2;2	3;3;3;3;2;2	3;3;3;3;2;2	Endoplasmic reticulum-Golg	ERGIC3
P14625;Q96GW1;A0A1	P14625	28;10;10;5;	26;8;8;5;5;	26;8;8;5;5;	Endoplasmin	HSP90B1
O60869-2;O60869-3;O6	O60869-2;(	2;2;2	2;2;2	2;2;2	Endothelial differentiation-r	EDF1
P84090;G3V279	P84090;G3	3;2	3;2	3;2	Enhancer of rudimentary hc	ERH
P42126-2;P42126;Q96D	P42126-2;F	5;5;4;3	5;5;4;3	5;5;4;3	Enoyl-CoA delta isomerase 1	ECI1;DCI
A0A0C4DGA2;O75521-2	A0A0C4DG	4;4;4;3;3;3	4;4;4;3;3;3	4;4;4;3;3;3	Enoyl-CoA delta isomerase 2	ECI2
P30084	P30084	8	8	8	Enoyl-CoA hydratase, mitoc	ECHS1
P98172	P98172	1	1	1	Ephrin-B1	EFNB1
P00533;E9PFD7;Q504U	P00533;E9I	30;29;25;1	30;29;25;1	30;29;25;1	Epidermal growth factor rec	EGFR
G3V2V8;J3KMY5;G3V3E	G3V2V8;J3	3;3;3;3;3;3	3;3;3;3;3;3	3;3;3;3;3;3	Epididymal secretory protei	NPC2
B5MCA4;P16422;C9JKY	B5MCA4;P1	6;6;3	6;6;3	6;6;3	Epithelial cell adhesion mol	EPCAM
H0YBR2;H0YBB3;Q6NX(	H0YBR2;H0	2;2;2;2;2;2	2;2;2;2;2;2	2;2;2;2;2;2	Epithelial splicing regulator	ESRP1
P07099	P07099	6	6	6	Epoxide hydrolase 1	EPHX1
Q99808;Q99808-2	Q99808;Q9	1;1	1;1	1;1	Equilibrative nucleoside tra	SLC29A1
M0R1Y2;P33947-2;P339	M0R1Y2;P3	2;2;2;2;1	2;2;2;2;1	2;2;2;2;1	ER lumen protein-retaining	KDELR1;KD
Q8N766-3;Q8N766-2;Q	Q8N766-3;	3;3;3;2	3;3;3;2	3;3;3;2	ER membrane protein comp	EMC1
Q15006;H0YAS9	Q15006;H0	3;2	3;2	3;2	ER membrane protein comp	EMC2
S4R3U9;Q9P0I2-2;Q9P0	S4R3U9;Q9	1;1;1	1;1;1	1;1;1	ER membrane protein comp	EMC3
O43402;M0R1B0;O434(	O43402;M(	2;1;1	2;1;1	2;1;1	ER membrane protein comp	EMC8
O75477;B0QZ43;O9490	O75477;B0	5;4;1;1	2;1;0;0	2;1;0;0	Erlin-1	ERLIN1
E5RHW4;O94905;E5RJ0	E5RHW4;O	6;6;2	6;6;2	3;3;1	Erlin-2	ERLIN2
Q96HE7;G3V3E6;G3V5(	Q96HE7;G3	12;7;5;5;2;	12;7;5;5;2;	12;7;5;5;2;	ERO1-like protein alpha	ERO1L
A0A096LP73;A0A096LN	A0A096LP7	2;2;2;2;2;2	2;2;2;2;2;2	2;2;2;2;2;2	ES1 protein homolog, mitoc	C21orf33
Q9H501	Q9H501	1	1	1	ESF1 homolog	ESF1
Q92506	Q92506	1	1	1	Estradiol 17-beta-dehydrog	HSD17B8
P60842;J3KT12;P60842	P60842;J3K	20;17;17;1	20;17;17;1	16;13;13;1	Eukaryotic initiation factor 4	EIF4A1;EIF4
P38919;I3L3H2	P38919	8;3	4;1	4;1	Eukaryotic initiation factor 4	EIF4A3
P15170-2;P15170-3;H3I	P15170-2;F	7;7;5;5;2;1	7;7;5;5;2;1	7;7;5;5;2;1	Eukaryotic peptide chain re	GSPT1
B7Z7P8;P62495-2;P624	B7Z7P8;P6	6;6;6;2;1;1	6;6;6;2;1;1	6;6;6;2;1;1	Eukaryotic peptide chain re	ETF1
D6RBD7;C9J1V9;O4332	D6RBD7;C9	2;2;2;2;1;1	2;2;2;2;1;1	2;2;2;2;1;1	Eukaryotic translation elong	EEF1E1;EEF
K7EM18;P41567;O6073	K7EM18;P4	2;2;2;1	2;2;2;1	2;2;2;1	Eukaryotic translation initia	EIF1;EIF1B
P47813;X6RAC9;A6NJH	P47813;X6I	3;2;2;2	3;2;2;2	3;2;2;2	Eukaryotic translation initia	EIF1AX;EIF1
P05198;H0YJS4;G3V4T5	P05198;H0	5;3;3	5;3;3	5;3;3	Eukaryotic translation initia	EIF2S1
P20042	P20042	7	7	7	Eukaryotic translation initia	EIF2S2
P41091;Q2VIR3;F8W81	P41091;Q2	8;6;4;4;1	8;6;4;4;1	8;6;4;4;1	Eukaryotic translation initia	EIF2S3;EIF2
C9IZE1;F8WAE5;Q9BY4	C9IZE1;F8V	2;2;2;2;2;1	2;2;2;2;2;1	2;2;2;2;2;1	Eukaryotic translation initia	EIF2A
Q14152;Q14152-2	Q14152;Q1	11;10	11;10	11;10	Eukaryotic translation initia	EIF3A
P55884;P55884-2;C9JQ	P55884;P5	8;8;3;3	8;8;3;3	8;8;3;3	Eukaryotic translation initia	EIF3B
Q99613-2;Q99613;B5M	Q99613-2;(	7;7;6;1;1	7;7;6;1;1	7;7;6;1;1	Eukaryotic translation initia	EIF3C;EIF3C

O15371-2;O15371-3;O1	O15371-2;(	9;9;9;3;1;1	9;9;9;3;1;1	9;9;9;3;1;1	Eukaryotic translation initia	EIF3D
P60228;E5RGA2;H0YAW	P60228;E5I	7;6;3;2;2;1	7;6;3;2;2;1	7;6;3;2;2;1	Eukaryotic translation initia	EIF3E
O00303;H0YDT6	O00303	3;1	3;1	3;1	Eukaryotic translation initia	EIF3F
K7EL20;O75821;K7ER9(	K7EL20;O7	4;4;3;2;1	4;4;3;2;1	4;4;3;2;1	Eukaryotic translation initia	EIF3G
A0A087WZK9;B3KS98;C	A0A087WZ	4;4;4;2;1;1	4;4;4;2;1;1	4;4;4;2;1;1	Eukaryotic translation initia	EIF3H
Q13347;Q5TFK1	Q13347	10;3	10;3	10;3	Eukaryotic translation initia	EIF3I
H0YGJ7;H0YLP3;O7582	H0YGJ7;H0	2;2;2;2;1	2;2;2;2;1	2;2;2;2;1	Eukaryotic translation initia	EIF3J
K7ES31;K7ERF1;Q9UBQ	K7ES31;K7I	1;1;1;1	1;1;1;1	1;1;1;1	Eukaryotic translation initia	EIF3K
B0QY89;Q9Y262;Q9Y26	B0QY89;Q9	7;7;6;5;3;3	7;7;6;5;3;3	7;7;6;5;3;3	Eukaryotic translation initia	EIF3L
E9PRY0;J3KNJ2;H0YCQ8	E9PRY0;J3K	1;1;1;1;1	1;1;1;1;1	1;1;1;1;1	Eukaryotic translation initia	EIF3M
Q04637-5;Q04637-4;Q(	Q04637-5;(	16;16;16;1(	16;16;16;1(	16;16;16;1(	Eukaryotic translation initia	EIF4G1
H0YCH5;H0Y3P2;D3DQ\	H0YCH5;H(	2;2;2;2;2;1	2;2;2;2;2;1	2;2;2;2;2;1	Eukaryotic translation initia	EIF4G2
E7EX17;P23588-2;P235	E7EX17;P2	4;4;4;1	4;4;4;1	4;4;4;1	Eukaryotic translation initia	EIF4B
Q15056-2;Q15056	Q15056-2;(	6;6	6;6	6;6	Eukaryotic translation initia	EIF4H
P55010;H0YMJ8;H0YM5	P55010;H0	2;1;1;1;1	2;1;1;1;1	2;1;1;1;1	Eukaryotic translation initia	EIF5
I3L397;I3L504;P63241;F	I3L397;I3L5	8;8;8;8;5;4	8;8;8;8;5;4	8;8;8;8;5;4	Eukaryotic translation initia	EIF5A;EIF5A
A0A087WUT6;O60841	A0A087WU	5;5	5;5	5;5	Eukaryotic translation initia	EIF5B
P56537;B7ZBH1;P5653	P56537;B7	5;3;3;1;1;1	5;3;3;1;1;1	5;3;3;1;1;1	Eukaryotic translation initia	EIF6
R4GNH9;R4GMQ7;B1AI	R4GNH9;R4	1;1;1;1;1;1	1;1;1;1;1;1	1;1;1;1;1;1	Exosome complex compone	EXOSC1
Q5RKV6	Q5RKV6	4	4	4	Exosome complex compone	EXOSC6
Q13868-3;Q13868	Q13868-3;(	1;1	1;1	1;1	Exosome complex compone	EXOSC2
Q15024	Q15024	2	2	2	Exosome complex compone	EXOSC7
F2Z2C0;G3V1J5;Q9Y2L1	F2Z2C0;G3	2;2;2;2	2;2;2;2	2;2;2;2	Exosome complex exonucle	DIS3
O14980;C9J673;C9JKM9	O14980	9;3;3;2;2;2	9;3;3;2;2;2	9;3;3;2;2;2	Exportin-1	XPO1
P55060-3;P55060;P550	P55060-3;F	12;12;11;2	12;12;11;2	12;12;11;2	Exportin-2	CSE1L
Q9HAV4;H0Y3Q8;E2QR	Q9HAV4;H(	2;1;1	2;1;1	2;1;1	Exportin-5	XPO5
Q9BSJ8;Q9BSJ8-2	Q9BSJ8;Q9	11;11	11;11	11;11	Extended synaptotagmin-1	ESYT1
E7EQR4;P15311;P3524	E7EQR4;P1	14;14;5;5;3	9;9;0;0;0;0	9;9;0;0;0;0	Ezrin	EZR
Q9Y5B9;G3V5A4;G3V4(	Q9Y5B9	10;1;1;1	10;1;1;1	10;1;1;1	FACT complex subunit SPT1	SUPT16H
Q08945;E9PMD4;E9PPZ	Q08945	10;4;1;1	10;4;1;1	10;4;1;1	FACT complex subunit SSRP	SSRP1
P52907	P52907	7	7	6	F-actin-capping protein sub	CAPZA1
P47755;C9JUG7;F8W9N	P47755;C9	6;5;5;5;4	5;4;4;4;3	5;4;4;4;3	F-actin-capping protein sub	CAPZA2
P47756-2;B1AK87;B1AK	P47756-2;E	10;9;9;9;7	10;9;9;9;7	10;9;9;9;7	F-actin-capping protein sub	CAPZB
Q96AE4-2;E9PEB5;Q96	Q96AE4-2;I	12;11;11;6	11;10;10;6	11;10;10;6	Far upstream element-bind	FUBP1
A0A087WTP3;Q92945;I	A0A087WT	14;14;7;5;3	14;14;7;5;3	13;13;6;5;3	Far upstream element-bind	KHSRP
Q96I24;Q96I24-2	Q96I24;Q9	8;5	7;4	7;4	Far upstream element-bind	FUBP3
P14324-2;P14324;A0A0	P14324-2;F	3;3;2;2;2;1	3;3;2;2;2;1	3;3;2;2;2;1	Farnesyl pyrophosphate syr	FDPS
Q96CS3	Q96CS3	6	6	6	FAS-associated factor 2	FAF2
Q16658;C9JFC0;A0A0A(	Q16658	19;4;4;2	19;4;4;2	19;4;4;2	Fascin	FSCN1
A0A0U1RQF0;P49327;A	A0A0U1RQ	49;49;5;1;1	49;49;5;1;1	49;49;5;1;1	Fatty acid synthase;[Acyl-ca	FASN
Q01469;I6L8B7;A8MUU	Q01469;I6I	4;3;1	4;3;1	4;3;1	Fatty acid-binding protein, e	FABP5
E9PNW8;Q8WVX9	E9PNW8;Q	3;3	3;3	3;3	Fatty acyl-CoA reductase 1	FAR1
J3QRD1;P51648;P51648	J3QRD1;P5	7;7;7;4;3;1	7;7;7;4;3;1	7;7;7;4;3;1	Fatty aldehyde dehydrogen	ALDH3A2
G3V1L6;Q9BQL6;Q9BQI	G3V1L6;Q9	4;4;3;3;1	4;4;3;3;1	4;4;3;3;1	Fermitin family homolog 1	FERMT1
P02794;G3V1D1;G3V19	P02794;G3	6;4;4;3;3;2	6;4;4;3;3;2	6;4;4;3;3;2	Ferritin heavy chain;Ferritin	FTH1
Q4ZHG4-2;Q4ZHG4	Q4ZHG4-2;	1;1	1;1	1;1	Fibronectin type III domain-	FNDC1
P21333-2;P21333;Q5H\	P21333-2;F	88;88;87;87	88;88;87;87	80;80;79;79	Filamin-A	FLNA
O75369-2;O75369-9;O7	O75369-2;(	48;48;48;47	43;43;43;42	41;41;41;4(	Filamin-B	FLNB
Q8WUP2-3;Q8WUP2;Q	Q8WUP2-3	2;2;2	2;2;2	2;2;2	Filamin-binding LIM protein	FBLIM1
P39748;P39748-2;F5H1	P39748;P39	6;4;3;2	6;4;3;2	6;4;3;2	Flap endonuclease 1	FEN1

P30043;M0R192;M0QZ	P30043;M(	5;4;3	5;4;3	5;4;3	Flavin reductase (NADPH)	BLVRB
O75955-2;O75955;A0A:	O75955-2;(	5;5;4;4;2;2	5;5;4;4;2;2	5;5;4;4;2;2	Flotillin-1	FLOT1
J3QLD9;E7EMK3;Q1425	J3QLD9;E7I	6;6;6;3	6;6;6;3	6;6;6;3	Flotillin-2	FLOT2
J3KNW4;A0A0A0MSG2;	J3KNW4;A(	2;2;2	2;2;2	2;2;2	Four and a half LIM domain	FHL2
B4DXZ6;P51114-2;P511	B4DXZ6;P5	6;6;6;5;4;4	6;6;6;5;4;4	6;6;6;5;4;4	Fragile X mental retardatior	FXR1
H7C585;C9JAX1;Q1659!	H7C585;C9	2;2;2;1;1	2;2;2;1;1	2;2;2;1;1	Frataxin, mitochondrial;Frat	FXN
O60353-2;O60353	O60353-2;(	1;1	1;1	1;1	Frizzled-6	FZD6
P04075;P04075-2;H3BC	P04075;P0	23;22;21;2	23;22;21;2	17;16;15;1	Fructose-bisphosphate aldo	ALDOA
P09972;K7EKH5;C9J8F3	P09972;K7I	8;4;4;4;4;4	2;2;2;2;2;2	2;2;2;2;2;2	Fructose-bisphosphate aldo	ALDOC
P07954-2;P07954	P07954-2;F	11;11	11;11	11;11	Fumarate hydratase, mitocl	FH
S4R445;Q14802;Q1480:	S4R445;Q1	1;1;1;1	1;1;1;1	1;1;1;1	FXYD domain-containing ior	FXYD3
E9PN19;O00214;O0021	E9PN19;O(	5;5;5;4;3;2	5;5;5;4;3;2	5;5;5;4;3;2	Galectin;Galectin-8	LGALS8
P09382;F8WEI7	P09382	8;2	8;2	8;2	Galectin-1	LGALS1
P17931;G3V3R6	P17931;G3	2;1	2;1	2;1	Galectin-3;Galectin	LGALS3
Q08380;K7EJY8;K7ESM:	Q08380	4;1;1;1;1	4;1;1;1;1	4;1;1;1;1	Galectin-3-binding protein	LGALS3BP
P60520;H3BQ50;F5GZY	P60520;H3	2;1;1;1;1;1	2;1;1;1;1;1	2;1;1;1;1;1	Gamma-aminobutyric acid r	GABARAPL
O75223-3;O75223;M0C	O75223-3;(	3;3;2;1;1;1	3;3;2;1;1;1	3;3;2;1;1;1	Gamma-glutamylcyclotrans	GGCT
Q16666-3;Q16666-2;Q1	Q16666-3;(	9;9;9;8;4;3	9;9;9;8;4;3	9;9;9;8;4;3	Gamma-interferon-inducibl	IFI16
Q9H488;Q9H488-2	Q9H488;Q!	2;1	2;1	2;1	GDP-fucose protein O-fucos	POFUT1
CON__Q3SX14;A0A0A0	CON__Q3S	12;9;9;9;9;	12;9;9;9;9;	12;9;9;9;9;	Gelsolin	GSN
Q12789-3;Q12789	Q12789-3;(	1;1	1;1	1;1	General transcription factor	GTF3C1
P78347-2;P78347-4;P78	P78347-2;F	8;8;8;8;2	8;8;8;8;2	8;8;8;8;2	General transcription factor	GTF2I
O60763;O60763-2	O60763;O6	2;2	2;2	2;2	General vesicular transport	USO1
G3V4P8;P60983	G3V4P8;P6	1;1	1;1	1;1	Glia maturation factor beta	GMFB
Q9NZM5	Q9NZM5	2	2	2	Glioma tumor suppressor ca	GLTSCR2
G3V5E4;G3V4W4;Q96E	G3V5E4;G3	1;1;1	1;1;1	1;1;1	Glucosamine 6-phosphate N	GNPNAT1
D6RAY7;D6R9P4;P4692	D6RAY7;D6	5;5;5;4;4;3	5;5;5;4;4;3	5;5;5;4;4;3	Glucosamine-6-phosphate i	GNPDA1
P11413;P11413-3;P114	P11413;P1	15;15;15;9;	15;15;15;9;	15;15;15;9;	Glucose-6-phosphate 1-deh	G6PD
A0A0A0MTS2;P06744;P	A0A0A0MT	10;10;10;9;	10;10;10;9;	10;10;10;9;	Glucose-6-phosphate isome	GPI
K7ELL7;P14314-2;P1431	K7ELL7;P14	16;15;15;6;	16;15;15;6;	16;15;15;6;	Glucosidase 2 subunit beta	PRKCSH
P00367;P00367-3;P003	P00367;P0(	18;16;14;1	18;16;14;1	18;16;14;1	Glutamate dehydrogenase :	GLUD1;GLU
E1CEI4;P48506;H0Y9I7;	E1CEI4;P48	2;2;1;1	2;2;1;1	2;2;1;1	Glutamate--cysteine ligase (	GCLC
P48507-2;P48507	P48507-2;F	1;1	1;1	1;1	Glutamate--cysteine ligase r	GCLM
Q9BQ67;M0QX71	Q9BQ67;M	3;2	3;2	3;2	Glutamate-rich WD repeat-	GRWD1
O94925-3;O94925;B8Z	O94925-3;(	10;8;3;2;2;	10;8;3;2;2;	10;8;3;2;2;	Glutaminase kidney isoform	GLS
Q06210-2;Q06210	Q06210-2;(	4;4	4;4	4;4	Glutamine--fructose-6-phos	GFPT1
P47897-2;P47897;A0A0	P47897-2;F	6;6;3;3;3;3	6;6;3;3;3;3	2;2;2;2;2;2	Glutamine--tRNA ligase	QARS
O76003	O76003	2	2	2	Glutaredoxin-3	GLRX3
Q86SX6	Q86SX6	1	1	1	Glutaredoxin-related protei	GLRX5
P00390-4;P00390-2;P0(	P00390-4;F	4;4;4;3;3;1	4;4;4;3;3;1	4;4;4;3;3;1	Glutathione reductase, mitc	GSR
E9PFN5;Q9Y2Q3-4;Q9Y	E9PFN5;Q9	3;3;3;2;2	3;3;3;2;2	3;3;3;2;2	Glutathione S-transferase k	GSTK1
P78417;Q5TA02;P7841	P78417;Q5	6;5;5;5;4	6;5;5;5;4	6;5;5;5;4	Glutathione S-transferase o	GSTO1
P09211;A8MX94;A0A0	P09211;A8	9;7;4;1	9;7;4;1	9;7;4;1	Glutathione S-transferase P	GSTP1
P04406-2;P04406;E7EU	P04406-2;F	15;15;13;1	15;15;13;1	15;15;13;1	Glyceraldehyde-3-phosphat	GAPDH
Q53EU6	Q53EU6	1	1	1	Glycerol-3-phosphate acyltr	AGPAT9
P43304;P43304-2;F5GY	P43304;P4:	8;7;2	8;7;2	8;7;2	Glycerol-3-phosphate dehy(	GPD2
A0A1W2PQV2;H3BUH9	A0A1W2PC	2;2;2	2;2;2	2;2;2	Glycine cleavage system H p	GCSH
P41250;H7C443;F8WCK	P41250	5;1;1	5;1;1	5;1;1	Glycine--tRNA ligase	GARS
P49841;P49841-2	P49841;P4!	1;1	1;1	1;1	Glycogen synthase kinase-3	GSK3B
G5E9W8;C9JQ42;P4697	G5E9W8;C!	1;1;1;1;1	1;1;1;1;1	1;1;1;1;1	Glycogenin-1	GYG1

F6TLX2;Q9HC38-2;Q9H(	F6TLX2;Q9I	3;3;3;1;1;1	3;3;3;1;1;1	3;3;3;1;1;1	Glyoxalase domain-containi	GLOD4
U3KQ56;Q9UBQ7	U3KQ56;Q9	1;1	1;1	1;1	Glyoxylate reductase/hydro	GRHPR
P35052;H7C410;H7C02	P35052;H7	7;6;4;4;3;2	7;6;4;4;3;2	7;6;4;4;3;2	Glypican-1;Secreted glypica	GPC1
P49915-2;P49915	P49915-2;F	7;7	7;7	7;7	GMP synthase [glutamine-h	GMPS
Q92896;Q92896-3;Q92	Q92896;Q9	6;6;6;4;1;1	6;6;6;4;1;1	6;6;6;4;1;1	Golgi apparatus protein 1	GLG1
F8W785;O00461	F8W785;O(	3;3	3;3	3;3	Golgi integral membrane pr	GOLIM4
Q9H4A6;Q9H4A5	Q9H4A6;Q9	2;1	2;1	2;1	Golgi phosphoprotein 3;Gol	GOLPH3;G(
Q9H8Y8-2;Q9H8Y8;Q9H	Q9H8Y8-2;	4;4;4	4;4;4	4;4;4	Golgi reassembly-stacking p	GORASP2
Q9H3P7	Q9H3P7	1	1	1	Golgi resident protein GCP6	ACBD3
F6RU00;B4DQA8;K7EJC	F6RU00;B4	1;1;1;1;1	1;1;1;1;1	1;1;1;1;1	Golgi SNAP receptor comple	GOSR1
Q08379-2;Q08379;A0A	Q08379-2;(	4;4;2	4;4;2	4;4;2	Golgin subfamily A member	GOLGA2
Q7Z5G4-3;Q7Z5G4	Q7Z5G4-3;	1;1	1;1	1;1	Golgin subfamily A member	GOLGA7
D6R9J9;E7EM50;D6RFE	D6R9J9;E7I	1;1;1;1;1;1	1;1;1;1;1;1	1;1;1;1;1;1	GPI ethanolamine phosphat	PIGG
Q9Y653-5;Q9Y653-2;Q9	Q9Y653-5;(	2;2;2;2;1;1	2;2;2;2;1;1	2;2;2;2;1;1	G-protein coupled receptor	GPR56;AD(
H0Y8R1;F5H5I6;H0YAK1	H0Y8R1;F5	2;2;2;2;2	2;2;2;2;2	2;2;2;2;2	G-rich sequence factor 1	GRSF1
Q9H3K2	Q9H3K2	2	2	2	Growth hormone-inducible	GHITM
Q9HAV7	Q9HAV7	5	5	5	GrpE protein homolog 1, mi	GRPEL1
Q9UIJ7-2;Q9UIJ7-3;Q9U	Q9UIJ7-2;C	1;1;1	1;1;1	1;1;1	GTP:AMP phosphotransfera	AK3
P01116-2;P01116;P011	P01116-2;F	2;2;2;1;1;1	2;2;2;1;1;1	2;2;2;1;1;1	GTPase KRas;GTPase KRas, I	KRAS;NRAS
B5MDF5;P62826;J3KQE	B5MDF5;P(	7;7;6;5;3;3	7;7;6;5;3;3	7;7;6;5;3;3	GTP-binding nuclear protein	RAN
C9J931;Q15382	C9J931;Q1!	1;1	1;1	1;1	GTP-binding protein Rheb	RHEB
H0Y5E8;Q9NR31-2;Q9N	H0Y5E8;Q9	1;1;1	1;1;1	1;1;1	GTP-binding protein SAR1a	SAR1A
P52735-3;P52735-2;P52	P52735-3;F	2;2;2	2;2;2	2;2;2	Guanine nucleotide exchan	VAV2
P63096;P63096-2;C9JPI	P63096;P6	8;7;4;1	4;4;4;1	4;4;4;1	Guanine nucleotide-binding	GNAI1
P04899;P04899-4;P048	P04899;P0	8;8;7;7;7;7	4;4;4;4;4;4	4;4;4;4;4;4	Guanine nucleotide-binding	GNAI2
Q9UBI6	Q9UBI6	5	5	5	Guanine nucleotide-binding	GNG12
P62873;P62873-2;B1AK	P62873;P6	7;6;5;5;3;2	4;4;3;3;1;1	4;4;3;3;1;1	Guanine nucleotide-binding	GNB1
P62879;C9JIS1;C9JXA5;(	P62879;C9	9;8;8;7;5;5	9;8;8;7;5;5	6;6;6;5;2;3	Guanine nucleotide-binding	GNB2;GNB
P08754;P11488;P09471	P08754	8;2;2;2;2;1	8;2;2;2;2;1	4;0;0;0;0;0	Guanine nucleotide-binding	GNAI3
P50148;B1AM21	P50148;B1	2;1	2;1	1;1	Guanine nucleotide-binding	GNAQ
P63092-3;P63092-2;P63	P63092-3;F	4;4;4;4;4;4	3;3;3;3;3;3	3;3;3;3;3;3	Guanine nucleotide-binding	GNAS
P29992;O95837	P29992;O9	2;1	1;0	1;0	Guanine nucleotide-binding	GNA11;GN
Q14344-2;Q14344	Q14344-2;(	1;1	1;1	1;1	Guanine nucleotide-binding	GNA13
P63244;J3KPE3;H0YAF8	P63244;J3I	14;13;10;1(	14;13;10;1(	14;13;10;1(	Guanine nucleotide-binding	GNB2L1
Q9BVP2-2;Q9BVP2	Q9BVP2-2;	2;2	2;2	2;2	Guanine nucleotide-binding	GNL3
H0Y2M6;P51798-2;P51	H0Y2M6;P!	1;1;1	1;1;1	1;1;1	H(+)/Cl(-) exchange transpo	CLCN7
Q9NY12-2;Q9NY12	Q9NY12-2;	2;2	2;2	2;2	H/ACA ribonucleoprotein cc	GAR1
Q9NX24;J3QSY4;D6RC5	Q9NX24;J3	4;3;3;3	4;3;3;3	4;3;3;3	H/ACA ribonucleoprotein cc	NHP2
O60832;O60832-2;H7C	O60832;O6	6;4;2;2;2;1	6;4;2;2;2;1	6;4;2;2;2;1	H/ACA ribonucleoprotein cc	DKC1
Q5T3Q7;Q9H583;Q6P6(	Q5T3Q7;Q9	10;10;1	10;10;1	10;10;1	HEAT repeat-containing pro	HEATR1
Q0VDF9	Q0VDF9	1	1	1	Heat shock 70 kDa protein 1	HSPA14
A0A0G2JIW1;P0DMV9;I	A0A0G2JIW	29;29;29;2!	29;29;29;2!	15;15;15;1	Heat shock 70 kDa protein 1	HSPA1B;HS
P34932;A0A087WYC1;A	P34932;A0	19;16;11;4	18;15;10;4	18;15;10;4	Heat shock 70 kDa protein 4	HSPA4
P17066;P48741	P17066;P4	10;6	1;1	1;1	Heat shock 70 kDa protein 6	HSPA6;HSP
P11142;E9PKE3;P11142	P11142;E9I	28;27;22;1!	24;23;19;1(	18;17;13;1	Heat shock cognate 71 kDa	HSPA8
O75506	O75506	1	1	1	Heat shock factor-binding p	HSBP1
Q92598-2;Q92598;Q92	Q92598-2;(	21;21;20;2(	21;21;20;2(	20;20;19;1!	Heat shock protein 105 kDa	HSPH1
Q12931-2;Q12931;I3L0I	Q12931-2;(	13;13;9;3	13;13;9;3	13;13;9;3	Heat shock protein 75 kDa,	TRAP1
P04792;F8WE04;C9J3N	P04792	14;6;3	14;6;3	14;6;3	Heat shock protein beta-1	HSPB1
P07900;P07900-2;G3V2	P07900;P0	34;34;10;7;	19;19;5;2;2	19;19;5;2;2	Heat shock protein HSP 90-a	HSP90AA1

P08238;Q58FF7	P08238	39;16	39;16	19;6	Heat shock protein HSP 90-l	HSP90AB1
P54652	P54652	11	1	1	Heat shock-related 70 kDa p	HSPA2
J3KT51;Q9UK76;Q9UK7	J3KT51;Q9	4;4;4;3;3	4;4;4;3;3	4;4;4;3;3	Hematological and neurolog	HN1
H3BMT0;H3BTV5;H3BU	H3BMT0;H	2;2;2;2;2;2	2;2;2;2;2;2	2;2;2;2;2;2	Hematological and neurolog	HN1L
P30519;A0A087WT44;P	P30519;A0	4;3;3;2;2;1	4;3;3;2;2;1	4;3;3;2;2;1	Heme oxygenase 2	HMOX2
Q5THN1;Q9Y5Z4	Q5THN1;Q	1;1	1;1	1;1	Heme-binding protein 2	HEBP2
CON__P01966;G3V1N2	CON__P01	5;3;3;1	5;3;3;1	5;3;3;1	Hemoglobin subunit alpha	HBA2;HBA1
P68871;F8W6P5	P68871;F8	3;2	2;2	2;2	Hemoglobin subunit beta;L	HBB
CON__ENSEMBL:ENSBT	CON__ENS	3;2;2	3;2;2	3;2;2	Hepatocyte growth factor a	HGFAC
I3L1P5;O14964-2;O149	I3L1P5;O14	1;1;1	1;1;1	1;1;1	Hepatocyte growth factor-r	HGS
P51858;P51858-2;P518	P51858;P5	5;3;3	5;3;3	5;3;3	Hepatoma-derived growth f	HDGF
B0QZK4;Q5SSJ5-2;Q5SS	B0QZK4;Q5	6;6;6;5;5;5	6;6;6;5;5;5	6;6;6;5;5;5	Heterochromatin protein 1-	HP1BP3
D6R9P3;D6RD18;D6RBZ	D6R9P3;D6	4;4;4;4;4;3	4;4;4;4;4;3	4;4;4;4;4;3	Heterogeneous nuclear ribo	HNRNPAB
Q13151	Q13151	5	5	5	Heterogeneous nuclear ribo	HNRNPA0
F8W6I7;P09651-3;P096	F8W6I7;P0	11;11;11;1	10;10;10;10	10;10;10;10	Heterogeneous nuclear ribo	HNRNPA1;I
P51991-2;P51991;H7C1	P51991-2;P	8;7;1	8;7;1	8;7;1	Heterogeneous nuclear ribo	HNRNPA3
H0YA96;Q14103-4;Q14	H0YA96;Q1	7;7;7;6;6;6	7;7;7;6;6;6	7;7;7;6;6;6	Heterogeneous nuclear ribo	HNRNPD
A0A087WUK2;O14979-	A0A087WU	3;3;3;3	3;3;3;3	3;3;3;3	Heterogeneous nuclear ribo	HNRNPDL
P52597;A0A1B0GW42	P52597	11;2	11;2	9;2	Heterogeneous nuclear ribo	HNRNPF
E9PCY7;G8JLB6;P31943	E9PCY7;G8	10;10;10;7	8;8;8;5;4;5	4;4;4;2;2;2	Heterogeneous nuclear ribo	HNRNPH1
P55795	P55795	9	4	4	Heterogeneous nuclear ribo	HNRNPH2
P31942-2;P31942;P319	P31942-2;P	7;7;6;4;3;3	7;7;6;4;3;3	7;7;6;4;3;3	Heterogeneous nuclear ribo	HNRNPH3
P61978-3;P61978;P619	P61978-3;P	17;16;16;1	17;16;16;1	17;16;16;1	Heterogeneous nuclear ribo	HNRNPK
P14866;M0QXS5;P1486	P14866;M0	14;11;11;3	14;11;11;3	8;5;5;0;0;1	Heterogeneous nuclear ribo	HNRNPL
P52272-2;P52272;A0A0	P52272-2;P	23;23;22;1	23;23;22;1	23;23;22;1	Heterogeneous nuclear ribo	HNRNPM
O60506-4;O60506-3;O6	O60506-4;(	16;16;16;1	16;16;16;1	11;11;11;1	Heterogeneous nuclear ribo	SYNCRIP
O43390;O43390-2;O43	O43390;O4	14;13;11;1	9;8;7;6;5	9;8;7;6;5	Heterogeneous nuclear ribo	HNRNPR
Q00839;A0A1W2PPS1;(	Q00839;A0	24;23;23;2	24;23;23;2	24;23;23;2	Heterogeneous nuclear ribo	HNRNPU
M0R3F1;A0A0A0MRA5;	M0R3F1;A(	3;3;3;3;3;3	3;3;3;3;3;3	3;3;3;3;3;3	Heterogeneous nuclear ribo	HNRNPUL1
Q1KMD3;H3BQZ7	Q1KMD3;H	15;14	15;14	15;14	Heterogeneous nuclear ribo	HNRNPUL2
P22626;P22626-2;A0A0	P22626;P2	16;15;10	16;15;10	15;14;9	Heterogeneous nuclear ribo	HNRNPA2B
G3V4W0;B4DY08;B2R5	G3V4W0;B	15;15;15;1	15;15;15;1	15;15;15;1	Heterogeneous nuclear ribo	HNRNPC
E9PB90;P52789	E9PB90;P5	9;9	8;8	8;8	Hexokinase;Hexokinase-2	HK2
P19367-4;P19367-2;P19	P19367-4;P	14;14;14;1	14;14;14;1	13;13;13;1	Hexokinase-1	HK1
P30825	P30825	2	2	2	High affinity cationic amino	SLC7A1
Q5T7C4;P09429;B2RPK(	Q5T7C4;P0	5;5;2	5;5;2	4;4;1	High mobility group protein	HMGB1
D6R9A6;P26583	D6R9A6;P2	2;2	1;1	1;1	High mobility group protein	HMGB2
E9PES6;E7ES08;E7EQU1	E9PES6;E7E	1;1;1;1	1;1;1;1	1;1;1;1	High mobility group protein	HMGB3
P17096;P17096-3;P170	P17096;P1	4;4;2;1	4;4;2;1	4;4;2;1	High mobility group protein	HMGA1
F5H2U8;F5H2A4;F5H6H	F5H2U8;F5	1;1;1;1;1;1	1;1;1;1;1;1	1;1;1;1;1;1	High mobility group protein	HMGA2
P49773;D6REP8;D6RE9	P49773;D6	4;3;3;3;2	4;3;3;3;2	4;3;3;3;2	Histidine triad nucleotide-b	HINT1
D6RJE6;B3KWE1;P1208	D6RJE6;B3I	2;2;2;2;2;2	2;2;2;2;2;2	2;2;2;2;2;2	Histidine--tRNA ligase, cyto	HARS2;HAR
O14929-2;O14929	O14929-2;(	1;1	1;1	1;1	Histone acetyltransferase ty	HAT1
Q92769-3;Q92769	Q92769-3;(	4;4	4;4	2;2	Histone deacetylase 2	HDAC2
P07305;P07305-2	P07305;P0	4;2	4;2	4;2	Histone H1.0;Histone H1.0,	H1F0
P16403	P16403	10	10	3	Histone H1.2	HIST1H1C
P10412;P16402;P22492	P10412;P1	9;7;3;3	2;0;0;0	2;0;0;0	Histone H1.4;Histone H1.3	HIST1H1E;H
P16401	P16401	7	6	6	Histone H1.5	HIST1H1B
Q92522	Q92522	2	2	2	Histone H1x	H1FX
A0A0U1RR32;A0A0U1R	A0A0U1RR	5;5;5;5;5;5	5;5;5;5;5;5	1;1;1;1;1;1	Histone H2A type 1-J;Histor	HIST1H2AJ;

Q71UI9;P0C0S5;C9J0D1	Q71UI9;P0	4;4;3;3;3;3	2;2;1;2;2;1	2;2;1;2;2;1	Histone H2A.V;Histone H2A	H2AFV;H2A
Q5SQT3;Q9P0M6	Q5SQT3;Q9	2;2	1;1	1;1	Histone H2A;Core histone m	H2AFY2
P16104;Q96QV6;REV__	P16104;Q9	4;3;1;1;1;1	1;0;0;0;0;0	1;0;0;0;0;0	Histone H2AX;Histone H2A	H2AFX;HIST
Q99880;Q96A08	Q99880;Q9	8;5	1;1	0;0	Histone H2B type 1-L;Histor	HIST1H2BL
Q16778;P33778;P23527	Q16778;P3	7;7;7;7;2;2	1;1;1;1;1;1	0;0;0;0;0;0	Histone H2B type 2-E;Histor	HIST2H2BE
Q8N257	Q8N257	7	1	1	Histone H2B type 3-B	HIST3H2BB
U3KQK0;Q99879;Q9987	U3KQK0;Q9	8;8;8;8;8;8	8;8;8;8;8;8	0;0;0;0;0;0	Histone H2B;Histone H2B ty	HIST1H2BN
Q5TEC6	Q5TEC6	5	5	1	Histone H3	HIST2H3PS
P62805	P62805	8	8	8	Histone H4	HIST1H4A
Q09028-3;Q09028;Q09	Q09028-3;(	9;9;7;6;3;3	9;9;7;6;3;3	5;5;3;3;2;2	Histone-binding protein RBI	RBBP4
Q16576;Q16576-2;E9P(	Q16576;Q1	6;6;5;3	2;2;1;2	2;2;1;2	Histone-binding protein RBI	RBBP7
P16189;Q9GJ45;B0UXQ	P16189;Q9	12;10;10;1	2;0;1;0;0;1	0;0;0;0;0;0	HLA class I histocompatibili	HLA-A
P30453;A0A140T8Y4;P3	P30453;A0	12;11;11;1	12;11;11;1	0;0;0;0;0;0	HLA class I histocompatibili	HLA-A
Q8MH48;Q5RJ85;A0A0	Q8MH48;C	2;2;2;2;2;1	1;1;1;1;1;1	1;1;1;1;1;1	HLA class I histocompatibili	HLA-G
P30479;P30481;A0A14(	P30479;P3	8;6;5;3;3;3	2;2;1;1;1;1	0;0;0;0;0;0	HLA class I histocompatibili	HLA-B
P30508;A0A1W2PRZ0;A	P30508;A0	9;8;8;8;8;8	5;4;4;4;4;5	0;0;0;0;0;0	HLA class I histocompatibili	HLA-C
Q95604	Q95604	8	2	0	HLA class I histocompatibili	HLA-C
P50502;Q3KNR6;Q8NFI	P50502;Q3	7;6;6;5;4;4	7;6;6;5;4;4	7;6;6;5;4;4	Hsc70-interacting protein;P	ST13;ST13P
Q16543;K7EQA9;K7EKC	Q16543;K7	8;6;5;3;2	8;6;5;3;2	8;6;5;3;2	Hsp90 co-chaperone Cdc37	CDC37
H3BRH5;O00219-2;O00	H3BRH5;O(	1;1;1	1;1;1	1;1;1	Hyaluronan synthase 3	HAS3
A0A0D9SFP2;E9PF18;Q	A0A0D9SFI	5;5;5;5;5;4	5;5;5;5;5;4	5;5;5;5;5;4	Hydroxyacyl-coenzyme A de	HADH
P35914	P35914	2	2	2	Hydroxymethylglutaryl-CoA	HMGCL
Q01581;D6RIW1	Q01581	8;2	8;2	8;2	Hydroxymethylglutaryl-CoA	HMGCS1
Q6YN16-2;Q6YN16	Q6YN16-2;	5;5	5;5	5;5	Hydroxysteroid dehydroger	HSDL2
P00492	P00492	5	5	5	Hypoxanthine-guanine pho:	HPRT1
A0A087X054;Q9Y4L1;E9	A0A087X05	8;8;6;6;6;4	8;8;6;6;6;4	8;8;6;6;6;4	Hypoxia up-regulated prote	HYOU1
A0A1W2PNR9;Q9Y5U9	A0A1W2PN	1;1	1;1	1;1	Immediate early response 3	IER3IP1
P52292;J3QLL0;J3KS65;	P52292	9;4;4;1	9;4;4;1	9;4;4;1	Importin subunit alpha-1	KPNA2
O00629;H7C4F6	O00629	5;2	5;2	4;2	Importin subunit alpha-3	KPNA4
O00505	O00505	2	1	1	Importin subunit alpha-4	KPNA3
P52294;C9J352;C9JWD9	P52294;C9	2;1;1;1;1;1	2;1;1;1;1;1	2;1;1;1;1;1	Importin subunit alpha-5;Im	KPNA1;KPN
Q14974;J3KTM9;Q1497	Q14974;J3I	15;12;12;3	15;12;12;3	15;12;12;3	Importin subunit beta-1	KPNB1
H0Y8C6;O00410-2;O004	H0Y8C6;O0	10;10;10;1	10;10;10;1	10;10;10;1	Importin-5	IPO5
O95373	O95373	4	4	4	Importin-7	IPO7
Q96P70	Q96P70	2	2	2	Importin-9	IPO9
Q13308-3;Q13308-4;Q1	Q13308-3;(	2;2;2;2;2;1	2;2;2;2;2;1	2;2;2;2;2;1	Inactive tyrosine-protein kir	PTK7
Q70UQ0;Q70UQ0-2	Q70UQ0;Q	2;1	1;1	1;1	Inhibitor of nuclear factor k	IKBIP
Q70UQ0-4	Q70UQ0-4	2	2	1	Inhibitor of nuclear factor k	IKBIP
Q9NQS7-2;Q9NQS7	Q9NQS7-2;	2;2	2;2	2;2	Inner centromere protein	INCENP
Q9Y2U8	Q9Y2U8	2	2	2	Inner nuclear membrane pr	LEMD3
Q15181;Q5SQT6	Q15181;Q5	9;6	9;6	9;6	Inorganic pyrophosphatase	PPA1
Q9BY32-2;Q9BY32;Q9B	Q9BY32-2;(	2;2;1	2;2;1	2;2;1	Inosine triphosphate pyropl	ITPA
H0Y4R1;P12268;E7ETK5	H0Y4R1;P1	12;12;4;1	12;12;4;1	11;11;3;0	Inosine-5-monophosphate (	IMPDH2
Q14573;B7ZMI3;Q1464	Q14573	6;1;1;1;1;1	6;1;1;1;1;1	6;1;1;1;1;1	Inositol 1,4,5-trisphosphate	ITPR3
O00425;O00425-2;F8W	O00425;O0	4;2;1;1;1;1	4;2;1;1;1;1	4;2;1;1;1;1	Insulin-like growth factor 2	IGF2BP3
Q9Y287;Q9Y287-2	Q9Y287;Q9	4;3	4;3	4;3	Integral membrane protein	ITM2B
J3QRY6;C9IYS7;A0A087	J3QRY6;C9I	1;1;1;1;1;1	1;1;1;1;1;1	1;1;1;1;1;1	Integrator complex subunit	CPSF3L
P17301;E7ESP4;E7EMF1	P17301;E7I	15;12;11;7	15;12;11;7	15;12;11;7	Integrin alpha-2	ITGA2
P26006;P26006-1;H0YA	P26006;P2(	5;5;2;1	5;5;2;1	5;5;2;1	Integrin alpha-3;Integrin alp	ITGA3
P23229-4;P23229-2;P23	P23229-4;F	22;22;22;2	22;22;22;2	22;22;22;2	Integrin alpha-6;Integrin alp	ITGA6

P06756-3;P06756-2;P06	P06756-3;F	5;5;5;1	5;5;5;1	5;5;5;1	Integrin alpha-V;Integrin alp	ITGAV
A0A087WXP3;E9PEE8;P	A0A087WX	2;2;2;1	2;2;2;1	2;2;2;1	Integrin beta;Integrin beta-(	ITGB6
P05556;P05556-2;P055	P05556;P0	14;13;13;1	14;13;13;1	14;13;13;1	Integrin beta-1	ITGB1
P16144-4;P16144-2;P16	P16144-4;F	36;36;36;3	36;36;36;3	36;36;36;3	Integrin beta-4	ITGB4
P05362;E7ESS4	P05362;E7I	2;1	2;1	2;1	Intercellular adhesion mole	ICAM1
P15260;Q5TFD1;Q5TFC	P15260	3;1;1;1	3;1;1;1	3;1;1;1	Interferon gamma receptor	IFNGR1
B1AJU4;O14896;O1489	B1AJU4;O1	3;3;2	3;3;2	3;3;2	Interferon regulatory factor	IRF6
H7BYV1;E9PS44;E9PQN	H7BYV1;E9	1;1;1;1;1;1	1;1;1;1;1;1	1;1;1;1;1;1	Interferon-induced transme	IFITM2;IFIT
P19525;P19525-2;F8WE	P19525;P1	5;4;1;1	5;4;1;1	5;4;1;1	Interferon-induced, double-	EIF2AK2
B4DY09;Q12905;X6R6Z	B4DY09;Q1	8;8;3;2	8;8;3;2	8;8;3;2	Interleukin enhancer-bindir	ILF2
Q12906;Q12906-7;Q12	Q12906;Q1	20;20;18;1	20;20;18;1	20;20;18;1	Interleukin enhancer-bindir	ILF3
Q27J81-2;Q27J81	Q27J81-2;C	1;1	1;1	1;1	Inverted formin-2	INF2
D6RGE2;Q96CN7	D6RGE2;Q	1;1	1;1	1;1	Isochorismatase domain-co	ISOC1
H0YL72;P50213;H0YLI6;	H0YL72;P5	8;8;5;5;5;4	8;8;5;5;5;4	8;8;5;5;5;4	Isocitrate dehydrogenase [N	IDH3A
P51553;H7C1W2;E9PF8	P51553;H7	3;2;2;2;2;2	3;2;2;2;2;2	3;2;2;2;2;2	Isocitrate dehydrogenase [N	IDH3G
O75874;C9J4N6;C9JJE5;	O75874	13;6;3;3;1;	13;6;3;3;1;	13;6;3;3;1;	Isocitrate dehydrogenase [N	IDH1
P41252;J3KR24;A0A0AC	P41252;J3K	12;11;11;3	12;11;11;3	12;11;11;3	Isoleucine--tRNA ligase, cyto	IARS
Q9NSE4	Q9NSE4	7	7	7	Isoleucine--tRNA ligase, mit	IARS2
C9JD53;C9JKM8;Q1390	C9JD53;C9J	1;1;1;1	1;1;1;1	1;1;1;1	Isopentenyl-diphosphate De	IDI1
P14923;C9JTX4;C9J826;	P14923	20;8;8;8;8;	20;8;8;8;8;	18;8;8;8;8;	Junction plakoglobin	JUP
Q9Y624;A0A087WY82;(	Q9Y624;A0	9;6;6	9;6;6	9;6;6	Junctional adhesion molecu	F11R
K7EM02;K7EIJ8;Q8IYT4	K7EM02;K7	1;1;1;1	1;1;1;1	1;1;1;1	Katanin p60 ATPase-contair	KATNAL2
CON__P13645;P13645;	CON__P13	7;7;1;1;1;1	4;4;1;1;1;1	4;4;1;1;1;1	Keratin, type I cytoskeletal 1	KRT10
CON__P13646-1;P1364	CON__P13	27;26;25;2	25;24;23;2	11;10;9;9;9	Keratin, type I cytoskeletal 1	KRT13
CON__P19012;P19012;	CON__P19	16;16;10;1	7;7;5;5;4	5;5;3;3;2	Keratin, type I cytoskeletal 1	KRT15
P05783;F8VZY9;CON__	P05783;F8	25;22;2;1;1	24;21;2;1;1	18;15;2;1;1	Keratin, type I cytoskeletal 1	KRT18
CON__P08727;P08727;	CON__P08	33;32;15;1	33;32;15;1	22;21;9;7;2	Keratin, type I cytoskeletal 1	KRT19
P35527;CON__P35527;	P35527;CO	5;4;2	4;3;2	4;3;2	Keratin, type I cytoskeletal 9	KRT9
CON__P04264;P04264	CON__P04	8;8	4;4	2;2	Keratin, type II cytoskeletal	KRT1
CON__P35908;P35908;	CON__P35	7;7;4	1;1;1	1;1;1	Keratin, type II cytoskeletal	KRT2
CON__P13647;P13647;	CON__P13	28;28;10;9	24;24;6;9;7	5;5;0;2;1;2	Keratin, type II cytoskeletal	KRT5
CON__P02538;P02538;	CON__P02	23;23;21;2	12;12;10;1	2;2;1;0;1;0	Keratin, type II cytoskeletal	KRT6A;KRT
P04259	P04259	20	1	0	Keratin, type II cytoskeletal	KRT6B
CON__Q3KNV1;P08729	CON__Q3K	37;37	37;37	3;3	Keratin, type II cytoskeletal	KRT7
CON__P05787;P05787;	CON__P05	36;36;36;2	33;33;33;2	29;29;29;1	Keratin, type II cytoskeletal	KRT8
Q07666-2;Q07666;Q07	Q07666-2;(	3;3;2	3;3;2	3;3;2	KH domain-containing, RNA	KHDRBS1
Q86UP2-2;Q86UP2-3;Q	Q86UP2-2;	5;5;5;5;2;2	5;5;5;5;2;2	5;5;5;5;2;2	Kinectin	KTN1
P33176;A0A0G2JMZ6;J	P33176	6;1;1;1;1	6;1;1;1;1	6;1;1;1;1	Kinesin-1 heavy chain	KIF5B
Q15058	Q15058	1	1	1	Kinesin-like protein KIF14	KIF14
H7BYN4;Q02241-2;Q02	H7BYN4;Q(	5;5;5;4;2;1	5;5;5;4;2;1	5;5;5;4;2;1	Kinesin-like protein;Kinesin-	KIF23
Q13601-2;Q13601	Q13601-2;(	1;1	1;1	1;1	KRR1 small subunit process	KRR1
K7EM91;O43291-2;O43	K7EM91;O	4;4;4;2;2;1	4;4;4;2;2;1	3;3;3;1;1;1	Kunitz-type protease inhibit	SPINT2
Q16719	Q16719	1	1	1	Kynureninase	KYNU
Q04760-2;Q04760	Q04760-2;(	2;2	2;2	2;2	Lactoylglutathione lyase	GLO1
E9PDI4;O00515;A0A0J9	E9PDI4;O0	7;7;4;4;3;1	7;7;4;4;3;1	7;7;4;4;3;1	Ladinin-1	LAD1
P42166;P42167-3	P42166;P4	11;7	4;0	4;0	Lamina-associated polypept	TMPO
P42167;G5E972;P42167	P42167;G5	13;11;10;6	13;11;10;6	6;4;3;4	Lamina-associated polypept	TMPO
Q14739;C9JXK0;C9JES9	Q14739;C9	4;2;1	4;2;1	4;2;1	Lamin-B receptor	LBR
P20700;E9PBF6;A0A0D	P20700;E9	32;25;22;1	31;24;22;1	30;24;22;1	Lamin-B1	LMNB1
Q03252	Q03252	27	25	25	Lamin-B2	LMNB2

K7EIP4;A0A0A0MTS5;A(	K7EIP4;A0/	1;1;1;1;1;1	1;1;1;1;1;1	1;1;1;1;1;1	Laminin subunit alpha-3	LAMA3
O15230;O15230-2	O15230	3;1	3;1	3;1	Laminin subunit alpha-5	LAMA5
Q13751;Q5THA1	Q13751	7;2	7;2	7;2	Laminin subunit beta-3	LAMB3
P11047	P11047	5	5	5	Laminin subunit gamma-1	LAMC1
Q13753-2;Q13753	Q13753-2;(	7;7	7;7	7;7	Laminin subunit gamma-2	LAMC2
F8WDS9;E9PHS0;O4381	F8WDS9;E9	1;1;1	1;1;1	1;1;1	LanC-like protein 1	LANCL1
A0A0C4DFL7;Q16850;Q	A0A0C4DFI	6;6;5;2	6;6;5;2	6;6;5;2	Lanosterol 14-alpha demetl	CYP51A1
P48449-3;P48449;C9J31	P48449-3;F	3;3;2;2;1	3;3;2;2;1	3;3;2;2;1	Lanosterol synthase	LSS
Q01650	Q01650	5	5	5	Large neutral amino acids tı	SLC7A5
A0A0G2JL47;A0A024RC	A0A0G2JL4	3;3;3;3;3;3	3;3;3;3;3;3	3;3;3;3;3;3	Large proline-rich protein B	BAT3;BAG6
Q14696	Q14696	2	2	2	LDLR chaperone MESD	MESDC2
Q8NC56-2;Q8NC56;D6F	Q8NC56-2;	2;2;1;1;1	2;2;1;1;1	2;2;1;1;1	LEM domain-containing pro	LEMD2
O95202	O95202	5	5	5	LETM1 and EF-hand domair	LETM1
P42704;B8ZZ38;A0A0C4	P42704	33;11;11;9;	33;11;11;9;	33;11;11;9;	Leucine-rich PPR motif-cont	LRPPRC
Q32MZ4-3;Q32MZ4-2;C	Q32MZ4-3;	5;5;5;1;1;1	5;5;5;1;1;1	5;5;5;1;1;1	Leucine-rich repeat flightles	LRRFIP1
O75473-3;O75473-2;O7	O75473-3;(	1;1;1	1;1;1	1;1;1	Leucine-rich repeat-contain	LGR5
Q8N1G4	Q8N1G4	3	3	3	Leucine-rich repeat-contain	LRRC47
Q96AG4;I3L223	Q96AG4	8;1	8;1	8;1	Leucine-rich repeat-contain	LRRC59
Q9P2J5-2;Q9P2J5;Q9P2	Q9P2J5-2;C	5;5;3	5;5;3	5;5;3	Leucine--tRNA ligase, cytop	LARS
Q9UIQ6-3;Q9UIQ6-2;Q9	Q9UIQ6-3;(	3;3;3	3;3;3	3;3;3	Leucyl-cystinyl aminopeptic	LNPEP
P30740;P30740-2	P30740;P3(	4;3	4;3	4;3	Leukocyte elastase inhibitoı	SERPINB1
Q08722-2;Q08722-3;Q(	Q08722-2;(	3;3;3;3	3;3;3;3	3;3;3;3	Leukocyte surface antigen (	CD47
P09960;P09960-2;P099	P09960;P0!	6;5;5;4;1	6;5;5;4;1	6;5;5;4;1	Leukotriene A-4 hydrolase	LTA4H
Q14847;Q14847-2;C9J9	Q14847;Q1	8;8;7;5;2;1	8;8;7;5;2;1	8;8;7;5;2;1	LIM and SH3 domain protei	LASP1
F8VQE1;Q9UHB6-2;Q9U	F8VQE1;Q9	12;12;12;1	12;12;12;1	12;12;12;1	LIM domain and actin-bindi	LIMA1
S4R3V8;Q86X29-6;Q86)	S4R3V8;Q8	3;3;3;3;3;2	3;3;3;3;3;2	3;3;3;3;3;2	Lipolysis-stimulated lipopro	LSR
P00338;P00338-3;P003	P00338;P0(	13;13;10;9;	13;13;10;9;	12;12;9;8;8	L-lactate dehydrogenase A (	LDHA
P07195;A8MW50;C9J7I	P07195;A8	9;6;5;4	8;5;5;4	8;5;5;4	L-lactate dehydrogenase B (	LDHB
K7EJE8;K7EKE6;P36776	K7EJE8;K7E	6;6;6;6;6;3	6;6;6;6;6;3	6;6;6;6;6;3	Lon protease homolog, mit(	LONP1
Q6P1M0;Q6P1M0-2	Q6P1M0	4;1	4;1	4;1	Long-chain fatty acid transp	SLC27A4
O95573;F5GWH2	O95573	12;1	12;1	10;1	Long-chain-fatty-acid--CoA	ACSL3
O60488-2;O60488;H0Y9	O60488-2;(	5;5;2	3;3;2	3;3;2	Long-chain-fatty-acid--CoA	ACSL4
P24666;F2Z2Q9;G5E9R!	P24666;F2	3;2;2;2;2;2	3;2;2;2;2;2	3;2;2;2;2;2	Low molecular weight phos	ACP1
J3KMZ9;P01130-2;P011	J3KMZ9;P0	5;5;5;5;5;5	5;5;5;5;5;5	5;5;5;5;5;5	Low-density lipoprotein rec	LDLR
Q7Z4F1	Q7Z4F1	1	1	1	Low-density lipoprotein rec	LRP10
J3KPP4;O95232;D6RDI2	J3KPP4;O9!	3;3;2;1;1	3;3;2;1;1	3;3;2;1;1	Luc7-like protein 3	LUC7L3
P05455;E9PGX9;E7ERC4	P05455	4;1;1	4;1;1	4;1;1	Lupus La protein	SSB
C9JY28;H7C4Q5;C9JRX8	C9JY28;H7(	1;1;1;1;1	1;1;1;1;1	1;1;1;1;1	LYR motif-containing protei	LYRM4
Q15046;Q15046-2;J3KR	Q15046;Q1	8;8;1;1;1	8;8;1;1;1	8;8;1;1;1	Lysine--tRNA ligase	KARS
Q6UWP7-3;Q6UWP7;C9	Q6UWP7-3	3;3;2;2;1;1	3;3;2;2;1;1	3;3;2;2;1;1	Lysocardiolipin acyltransfer	LCLAT1
A0A0G2JRI7;A0A0G2JQ	A0A0G2JRI	2;2;2	2;2;2	2;2;2	Lysophosphatidylcholine ac	LPCAT1
Q7L5N7;Q7L5N7-2;H3B	Q7L5N7;Q7	3;2;1	3;2;1	3;2;1	Lysophosphatidylcholine ac	LPCAT2
Q6P1A2-2;Q6P1A2	Q6P1A2-2;(	1;1	1;1	1;1	Lysophospholipid acyltransf	LPCAT3
Q96N66-2;Q96N66;Q96	Q96N66-2;	4;4;3	4;4;3	4;4;3	Lysophospholipid acyltransf	MBOAT7
D6RDG0;A0A1W2PS70;	D6RDG0;A(	1;1;1;1;1;1	1;1;1;1;1;1	1;1;1;1;1;1	Lysosome membrane prote	SCARB2
P11279;P11279-2	P11279;P1	4;3	4;3	4;3	Lysosome-associated meml	LAMP1
P13473-2;P13473;P134	P13473-2;F	2;2;2	2;2;2	2;2;2	Lysosome-associated meml	LAMP2
H0YAR1;Q9Y4K0	H0YAR1;Q9	2;2	2;2	2;2	Lysyl oxidase homolog 2	LOXL2
P14174	P14174	2	2	2	Macrophage migration inhil	MIF
P40121-2;P40121;H7C0	P40121-2;F	4;4;2;2	4;4;2;2	4;4;2;2	Macrophage-capping protei	CAPG

A0A087WU53;Q9H0U3;	A0A087WU	3;3;2	3;3;2	3;3;2	Magnesium transporter pro	MAGT1
P07199	P07199	4	4	4	Major centromere autoanti	CENPB
D6RIZ4;D6RA47;D6RE79	D6RIZ4;D6I	1;1;1;1	1;1;1;1	1;1;1;1	Major facilitator superfamil	MFSD10
Q95460-3;Q95460-2;Q9	Q95460-3;(	1;1;1	1;1;1	1;1;1	Major histocompatibility co	MR1
A2A2V1;P04156-2;P041	A2A2V1;P0	2;2;2	2;2;2	2;2;2	Major prion protein	PRNP
Q14764;H3BP76;H3BUK	Q14764	11;2;2;2;2;:	11;2;2;2;2;:	11;2;2;2;2;:	Major vault protein	MVP
B9A041;P40925-2;P409	B9A041;P4	6;6;6;6;4;4.	6;6;6;6;4;4.	6;6;6;6;4;4.	Malate dehydrogenase, cyt	MDH1
P40926;P40926-2;G3XA	P40926;P4	18;15;13	18;15;13	18;15;13	Malate dehydrogenase, mit	MDH2
Q14165;F5H1S8;H0YG0	Q14165;F5	4;3;2;2	4;3;2;2	4;3;2;2	Malectin	MLEC
Q9ULC4-2;Q9ULC4;Q9L	Q9ULC4-2;	1;1;1	1;1;1	1;1;1	Malignant T-cell-amplified s	MCTS1
Q9Y5P6;Q9Y5P6-2	Q9Y5P6;Q9	1;1	1;1	1;1	Mannose-1-phosphate guar	GMPPB
J3KSI4;I3L1D2;I3L4E0;J3	J3KSI4;I3L1	2;2;2;2;2;2.	2;2;2;2;2;2.	2;2;2;2;2;2.	Mannose-P-dolichol utilizat	MPDU1
Q13724-2;Q13724;C9J8	Q13724-2;(	6;6;4;2	6;6;4;2	6;6;4;2	Mannosyl-oligosaccharide g	MOGS
Q8NDC0	Q8NDC0	1	1	1	MAPK-interacting and spinc	MAPK1IP1L
P49006	P49006	2	2	2	MARCKS-related protein	MARCKSL1
A0A0R4J2E8;A8MXP9;P	A0A0R4J2E	17;17;17;1	17;17;17;1	17;17;17;1	Matrin-3	MATR3
P50281;F8W1B7	P50281	4;1	4;1	4;1	Matrix metalloproteinase-1	MMP14
Q14676-2;Q14676;Q14	Q14676-2;(	8;8;6;1;1	8;8;6;1;1	8;8;6;1;1	Mediator of DNA damage cl	MDC1
E9PGW7;Q5T8T7;E7EN9	E9PGW7;Q	1;1;1;1;1;1	1;1;1;1;1;1	1;1;1;1;1;1	Mediator of RNA polymeras	MED22
B7Z9I1;Q5T4U5;P11310	B7Z9I1;Q5T	2;2;2;2;1;1	2;2;2;2;1;1	2;2;2;2;1;1	Medium-chain specific acyl-	ACADM
P08582	P08582	3	3	3	Melanotransferrin	MFI2
Q8N4V1;Q8N4V1-2	Q8N4V1;Q	1;1	1;1	1;1	Membrane magnesium trar	MMGT1
O00264;O00264-2	O00264;OC	4;2	3;2	3;2	Membrane-associated prog	PGRMC1
O15173;O15173-2;U3K	O15173;O1	4;4;1	4;4;1	3;3;0	Membrane-associated prog	PGRMC2
I3L1H7;I3L4K3;I3L3P0;I3	I3L1H7;I3L	1;1;1;1;1;1.	1;1;1;1;1;1.	1;1;1;1;1;1.	Membrane-associated tyros	PKMYT1
A8K878;P55145;H7C2D	A8K878;P5	4;4;1	4;4;1	4;4;1	Mesencephalic astrocyte-de	MANF
B8ZZX6;Q658P3-4;Q658	B8ZZX6;Q6	2;2;2;2;2	2;2;2;2;2	2;2;2;2;2	Metalloreductase STEAP3	STEAP3
P80297;P13640-2;P027	P80297;P1	2;2;2;2;1;1.	2;2;2;2;1;1.	2;2;2;2;1;1.	Metallothionein-1X;Metallo	MT1X;MT1
O94776;O94776-2	O94776;O9	4;2	4;2	4;2	Metastasis-associated prote	MTA2
Q13505-3;Q13505;A0A	Q13505-3;(	4;4;3;3	4;4;3;3	4;4;3;3	Metaxin-1	MTX1
C9JAZ1;O75431-2;O754	C9JAZ1;O7	2;2;2;1	2;2;2;1	2;2;2;1	Metaxin-2	MTX2
H7C0X7;Q9NZL9-3;Q9N	H7C0X7;Q9	1;1;1;1;1	1;1;1;1;1	1;1;1;1;1	Methionine adenosyltransfe	MAT2B
P53582;H0YAI3;H0Y955	P53582	3;1;1	3;1;1	3;1;1	Methionine aminopeptidas	METAP1
G3V1U3;P50579-3;P505	G3V1U3;P5	2;2;2;1;1;1.	2;2;2;1;1;1.	2;2;2;1;1;1.	Methionine aminopeptidas	METAP2
P56192;P56192-2;H0YI	P56192;P5	6;5;1;1;1;1.	6;5;1;1;1;1.	6;5;1;1;1;1.	Methionine--tRNA ligase, cy	MARS
X6RBL6;Q9UBB5-3;Q9U	X6RBL6;Q9	1;1;1	1;1;1	1;1;1	Methyl-CpG-binding domai	MBD2
Q9HCC0-2;Q9HCC0;D6F	Q9HCC0-2;	3;3;2;1;1	3;3;2;1;1	3;3;2;1;1	Methylcrotonoyl-CoA carbo	MCCC2
G5E9X5;F5GYT8;E9PG3	G5E9X5;F5	1;1;1;1;1	1;1;1;1;1	1;1;1;1;1	Methylcrotonoyl-CoA carbo	MCCC1
Q9BQA1-2;Q9BQA1	Q9BQA1-2;	1;1	1;1	1;1	Methylosome protein 50	WDR77
E9PMI6;J3KN38;P54105	E9PMI6;J3K	1;1;1	1;1;1	1;1;1	Methylosome subunit pICln	CLNS1A
F5H8H2;A0A1B0GWC2;	F5H8H2;A0	2;2;2;1	2;2;2;1	2;2;2;1	Mevalonate kinase	MVK
A0A087WU07;Q5TGZ0-	A0A087WU	2;2;2;1;1	2;2;2;1;1	2;2;2;1;1	MICOS complex subunit MI	MINOS1;M
C9JRZ6;Q9NX63;F8WAF	C9JRZ6;Q9	6;6;5;3	6;6;5;3	6;6;5;3	MICOS complex subunit MI	CHCHD3
J3QTB2;J3QTA6;Q9BRQ	J3QTB2;J3C	1;1;1	1;1;1	1;1;1	MICOS complex subunit MI	CHCHD6
Q16891-2;Q16891;B9A	Q16891-2;(	16;16;15;1	16;16;15;1	16;16;15;1	MICOS complex subunit MI	IMMT
Q99735-2;Q99735	Q99735-2;(	1;1	1;1	1;1	Microsomal glutathione S-t	MGST2
Q5VV89;O14880;Q5VV	Q5VV89;O1	2;2;1	2;2;1	2;2;1	Microsomal glutathione S-t	MGST3
P11137-3;P11137	P11137-3;F	1;1	1;1	1;1	Microtubule-associated pro	MAP2
Q15691	Q15691	8	8	8	Microtubule-associated pro	MAPRE1
H0Y2V1;H7C4C5;B5ME	H0Y2V1;H7	3;3;3;3;3;3.	3;3;3;3;3;3.	3;3;3;3;3;3.	Microtubule-associated pro	MAP4

E9PPJ5;E9PLM6;P21741	E9PPJ5;E9F	2;2;2;1	2;2;2;1	2;2;2;1	Midkine	MDK
Q9BTE3-2;Q9BTE3	Q9BTE3-2;(	1;1	1;1	1;1	Mini-chromosome mainten	MCMBP
Q8TCT9-5;Q8TCT9;A0A(	Q8TCT9-5;(	3;3;2;2;2;1	3;3;2;2;2;1	3;3;2;2;2;1	Minor histocompatibility an	HM13
Q8N4C8-5;Q8N4C8-2;Q	Q8N4C8-5;	1;1;1;1;1	1;1;1;1;1	1;1;1;1;1	Misshapen-like kinase 1	MINK1
I3L1P8;Q02978-2;Q029	I3L1P8;Q02	5;5;5	5;5;5	5;5;5	Mitochondrial 2-oxoglutara	SLC25A11
Q7Z434-4;Q7Z434	Q7Z434-4;(	3;3	3;3	3;3	Mitochondrial antiviral-sign	MAVS
H0Y8C3;Q9NZJ7-2;Q9N	H0Y8C3;Q9	4;4;4;1	4;4;4;1	4;4;4;1	Mitochondrial carrier homo	MTCH1
Q9Y6C9;E9PIE4	Q9Y6C9;E9	6;5	6;5	6;5	Mitochondrial carrier homo	MTCH2
Q9UBX3;Q9UBX3-2;F6R	Q9UBX3;Q9	4;4;2	1;1;1	1;1;1	Mitochondrial dicarboxylate	SLC25A10
Q9Y3D6	Q9Y3D6	2	2	2	Mitochondrial fission 1 prot	FIS1
E9PJH7;Q9H936;A0A0D	E9PJH7;Q9	2;2;1;1;1;1	2;2;1;1;1;1	2;2;1;1;1;1	Mitochondrial glutamate ca	SLC25A22;S
Q9Y5L4	Q9Y5L4	2	2	2	Mitochondrial import inner	TIMM13
O43615;M0QXU7;M0Q	O43615;M(	6;4;1	6;4;1	6;4;1	Mitochondrial import inner	TIMM44
M0R047;Q3ZCQ8;Q3ZC	M0R047;Q	1;1;1	1;1;1	1;1;1	Mitochondrial import inner	TIMM50
O60220	O60220	2	2	2	Mitochondrial import inner	TIMM8A
G3V2F3;A0A1W2PRH9;	G3V2F3;A0	1;1;1;1;1	1;1;1;1;1	1;1;1;1;1	Mitochondrial import inner	TIMM9
Q9NS69	Q9NS69	5	5	5	Mitochondrial import recep	TOMM22
Q15785	Q15785	4	4	4	Mitochondrial import recep	TOMM34
O96008;O96008-2;K7EJ	O96008;O9	9;8;6;4	9;8;6;4	9;8;6;4	Mitochondrial import recep	TOMM40
O94826	O94826	8	8	8	Mitochondrial import recep	TOMM70A
C9JC63;E7EVY0;S4R3Q9	C9JC63;E7E	1;1;1;1;1;1	1;1;1;1;1;1	1;1;1;1;1;1	Mitochondrial inner membr	OXA1L
Q99797	Q99797	1	1	1	Mitochondrial intermediate	MIPEP
C9JVB6;Q7L0Y3	C9JVB6;Q7	3;3	3;3	3;3	Mitochondrial ribonuclease	TRMT10C
Q10713;Q10713-2;Q5S	Q10713;Q1	6;3;1	6;3;1	6;3;1	Mitochondrial-processing p	PMPCA
O75439;G3V0E4;F8WE	O75439;G3	4;3;1;1;1	3;2;1;1;1	3;2;1;1;1	Mitochondrial-processing p	PMPCB
P28482-2;P28482	P28482-2;F	2;2	2;2	2;2	Mitogen-activated protein k	MAPK1
J3QT28;O43684-2;O436	J3QT28;O4	5;5;5;3	5;5;5;3	5;5;5;3	Mitotic checkpoint protein	BUB3
Q8IVT2	Q8IVT2	2	2	2	Mitotic interactor and subs	MISP
Q9BYG3;H7BZL0;C9J808	Q9BYG3;H7	3;2;2;1	3;2;2;1	3;2;2;1	MKI67 FHA domain-interact	NIFK
P26038;V9GZ54	P26038	21;4	21;4	16;2	Moesin	MSN
P53985	P53985	2	2	2	Monocarboxylate transport	SLC16A1
A0A087WVM4;B7ZM99	A0A087WV	3;3;3;1;1;1	3;3;3;1;1;1	3;3;3;1;1;1	Monofunctional C1-tetrahy	MTHFD1L
H3BVD1;B7Z5N5;P8402	H3BVD1;B7	2;2;2;2;2;2	2;2;2;2;2;2	2;2;2;2;2;2	Mothers against decapenta	SMAD3;SM
E9PPG9;E9PQ57;P7840	E9PPG9;E9	5;5;5;2	5;5;5;2	5;5;5;2	mRNA export factor	RAE1
Q9UKD2	Q9UKD2	3	3	3	mRNA turnover protein 4 h	MRTO4
F8WCY8;O15440-5;O15	F8WCY8;O	1;1;1	1;1;1	1;1;1	Multidrug resistance-associ	ABCC5
F5GYQ2;F5GX77;Q9UI3	F5GYQ2;F5	1;1;1;1	1;1;1;1	1;1;1;1	Multifunctional methyltrans	TRMT112
E9PBS1;P22234;P22234	E9PBS1;P2	8;8;8;6	8;8;8;6	8;8;8;6	Multifunctional protein ADE	PAICS
C9JP00;A0A0A0MQX8;C	C9JP00;A0	2;2;2;1;1;1	2;2;2;1;1;1	2;2;2;1;1;1	Muscleblind-like protein 1;M	MBNL1;ME
Q9BQG0;Q9BQG0-2;I3L	Q9BQG0;Q	15;15;13;2	15;15;13;2	15;15;13;2	Myb-binding protein 1A	MYBBP1A
Q9NUJ1;Q9NUJ1-2;Q9N	Q9NUJ1;Q9	2;1;1	2;1;1	2;1;1	Mycophenolic acid acyl-gluc	ABHD10
O95297-4;O95297-2;O9	O95297-4;(	4;4;4;1;1;1	4;4;4;1;1;1	4;4;4;1;1;1	Myelin protein zero-like pro	MPZL1
O60487	O60487	1	1	1	Myelin protein zero-like pro	MPZL2
C9JJV6;C9JZL8;C9J5M0;	C9JJV6;C9J	2;2;2;2;2	2;2;2;2;2	2;2;2;2;2	Myeloid-associated differer	MYADM
Q9NZM1-6;Q9NZM1;Q9	Q9NZM1-6	49;49;48;4	49;49;48;4	49;49;48;4	Myoferlin	MYOF
P05976-2;P05976;P085	P05976-2;F	1;1;1	1;1;1	1;1;1	Myosin light chain 1/3, skel	MYL1;MYL3
F8W1R7;G3V1V0;P6066	F8W1R7;G3	9;9;9;9;8;8	9;9;9;9;8;8	7;7;7;7;6;6	Myosin light polypeptide 6	MYL6
J3KSW8;H0Y2S9;Q6WC	J3KSW8;H0	2;2;2;2;2;1	2;2;2;2;2;1	2;2;2;2;2;1	Myosin phosphatase Rho-in	MPRIP
J3QRS3;P19105;O1495(	J3QRS3;P1	8;8;8;5;4;3	8;8;8;5;4;3	8;8;8;5;4;3	Myosin regulatory light chai	MYL12A;M
P35580;P35580-5;P355	P35580;P3	13;13;13;1	1;1;1;1;1	1;1;1;1;1	Myosin-10	MYH10

P35749-4;P35749-3;P35	P35749-4;F	11;11;11;1	1;1;1;1	1;1;1;1	Myosin-11	MYH11
Q7Z406;Q7Z406-6;Q7Z4	Q7Z406;Q7	9;9;9;7;6;5	2;2;2;2;1;1	2;2;2;2;1;1	Myosin-14	MYH14
P35579;P35579-2;Q5BK	P35579;P3	104;67;9;5	104;67;9;5	88;53;7;4;C	Myosin-9	MYH9
C9JL85;P58546	C9JL85;P58	2;2	2;2	2;2	Myotrophin	MTPN
P29966	P29966	8	8	8	Myristoylated alanine-rich (	MARCKS
O14745;J3QRP6;O1474	O14745;J3	2;1;1	2;1;1	2;1;1	Na(+)/H(+) exchange regula	SLC9A3R1
Q86SF2;H0YAK0;E9PBY	Q86SF2;H0	2;1;1;1	2;1;1;1	2;1;1;1	N-acetylgalactosaminyltran	GALNT7
Q9H0A0;Q9H0A0-2;A0A	Q9H0A0;Q	13;12;11;3	13;12;11;3	13;12;11;3	N-acetyltransferase 10	NAT10
A0A0G2JPB6;A0A0G2JL	A0A0G2JPE	4;4;4;4;4;4	4;4;4;4;4;4	4;4;4;4;4;4	NACHT, LRR and PYD domai	NLRP2;NLR
Q13423;D6RHU2;D6RAI	Q13423;D6	4;2;2;2	4;2;2;2	4;2;2;2	NAD(P) transhydrogenase, r	NNT
B4DLR8;H3BRK3;P1555	B4DLR8;H3	4;4;4;4;4;3	4;4;4;4;4;3	4;4;4;4;4;3	NAD(P)H dehydrogenase [q	NQO1
A0A1W2PQF8;A0A1W2	A0A1W2PC	4;4;4;4;4;4	4;4;4;4;4;4	4;4;4;4;4;4	NAD-dependent malic enzy	ME2
Q9BU61	Q9BU61	1	1	1	NADH dehydrogenase [ubiq	NDUFAF3
F8WAS3;C9IZN5;A0A08	F8WAS3;C	1;1;1;1;1;1	1;1;1;1;1;1	1;1;1;1;1;1	NADH dehydrogenase [ubiq	NDUFA5
F5H0J3;Q16795	F5H0J3;Q1	1;1	1;1	1;1	NADH dehydrogenase [ubiq	NDUFA9
H3BPJ9;O96000;H3BV1	H3BPJ9;O9	2;2;1;1	2;2;1;1	2;2;1;1	NADH dehydrogenase [ubiq	NDUFB10
E7EPT4;P19404	E7EPT4;P1	2;2	2;2	2;2	NADH dehydrogenase [ubiq	NDUFV2
O75306-2;O75306	O75306-2;	2;2	2;2	2;2	NADH dehydrogenase [ubiq	NDUFS2
O75489	O75489	1	1	1	NADH dehydrogenase [ubiq	NDUFS3
H7C0R7;Q9UHQ9	H7C0R7;Q	1;1	1;1	1;1	NADH-cytochrome b5 redu	CYB5R1
P00387-2;P00387;P003	P00387-2;F	6;6;6;3	6;6;6;3	6;6;6;3	NADH-cytochrome b5 redu	CYB5R3
P48163-2;P48163	P48163-2;F	1;1	1;1	1;1	NADP-dependent malic enz	ME1
A0A0A0MTN9;J3QQX3;	A0A0A0MT	3;3;3;3;3;3	3;3;3;3;3;3	3;3;3;3;3;3	NADPH:adrenodoxin oxidor	FDXR
P16435;E7EMD0;H0Y4R	P16435;E7I	4;3;3	4;3;3	4;3;3	NADPH--cytochrome P450 r	POR
E7EQ69;Q9GZZ1;F8WCH	E7EQ69;Q	2;2;1;1;1;1	2;2;1;1;1;1	2;2;1;1;1;1	N-alpha-acetyltransferase 5	NAA50
F8VZJ2;F8W0W4;H0YH	F8VZJ2;F8V	2;2;2;2;2;2	2;2;2;2;2;2	2;2;2;2;2;2	Nascent polypeptide-associ	NACA
Q9Y2A7;Q9Y2A7-2	Q9Y2A7;Q	1;1	1;1	1;1	Nck-associated protein 1	NCKAP1
Q9BT67	Q9BT67	1	1	1	NEDD4 family-interacting p	NDFIP1
H7C5F7;B4DGY6;A0A0C	H7C5F7;B4	2;2;2;2	2;2;2;2	2;2;2;2	NEDD4 family-interacting p	NDFIP2
P61081;M0QX69;M0QY	P61081;M	3;2;1	3;2;1	3;2;1	NEDD8-conjugating enzyme	UBE2M
Q9UMX5	Q9UMX5	2	2	2	Neudesin	NENF
Q09666;E9PJZ0;E9PKR9	Q09666	138;3;3;3;3	138;3;3;3;3	138;3;3;3;3	Neuroblast differentiation-a	AHNAK
H0YAK4;E9PCB6;Q9BYT	H0YAK4;E9	1;1;1	1;1;1	1;1;1	Neurolysin, mitochondrial	NLN
Q9Y639-1;Q9Y639;H3B	Q9Y639-1;	3;3;2;2;2;2	3;3;2;2;2;2	3;3;2;2;2;2	Neuroplastin	NPTN;DKFZ
Q14697;F5H6X6	Q14697;F5	26;23	26;23	1;1	Neutral alpha-glucosidase A	GANAB
Q14697-2;E9PKU7;E9P	Q14697-2;	26;20;5;1	1;1;1;0	1;1;1;0	Neutral alpha-glucosidase A	GANAB
P43007;P43007-2	P43007;P4	2;1	2;1	2;1	Neutral amino acid transpo	SLC1A4
Q15758;M0QXM4;Q15	Q15758;M	9;8;6;6;4;1	9;8;6;6;4;1	9;8;6;6;4;1	Neutral amino acid transpo	SLC1A5
A0A0R4J2G3;A0A0A0M	A0A0R4J2C	4;4;4;4;3;2	4;4;4;4;3;2	4;4;4;4;3;2	Neutral cholesterol ester hy	NCEH1
X6R8F3;P80188;P80188	X6R8F3;P8	4;4;3	4;4;3	4;4;3	Neutrophil gelatinase-assoc	LCN2
B7Z5R6;Q14596-2;Q14	B7Z5R6;Q1	1;1;1	1;1;1	1;1;1	Next to BRCA1 gene 1 prote	NBR1
B1AHD1;P55769	B1AHD1;P5	3;3	3;3	3;3	NHP2-like protein 1;NHP2-li	NHP2L1
Q96TA1-2;Q96TA1	Q96TA1-2;	3;3	3;3	3;3	Niban-like protein 1	FAM129B
Q969V3-2;Q969V3;K7E	Q969V3-2;	4;4;1;1	4;4;1;1	4;4;1;1	Nicalin	NCLN
P43490;A0A0C4DFS8;C	P43490;A0	8;6;1;1	8;6;1;1	8;6;1;1	Nicotinamide phosphoribos	NAMPT
Q6XQN6-3;Q6XQN6	Q6XQN6-3;	1;1	1;1	1;1	Nicotinate phosphoribosylt	NAPRT
K7EQ23;O15118-2;O15	K7EQ23;O1	1;1;1	1;1;1	1;1;1	Niemann-Pick C1 protein	NPC1
J3KN36;A0A0G2JN29;A	J3KN36;A0	6;6;6;6;6;6	6;6;6;6;6;6	6;6;6;6;6;6	Nodal modulator 2;Nodal m	NOMO3;N
P05204;A0A087WZE9;C	P05204	3;1;1;1	3;1;1;1	3;1;1;1	Non-histone chromosomal	HMGN2
Q15233;Q15233-2;H7C	Q15233;Q1	13;12;9;7;5	12;11;8;7;4	12;11;8;7;4	Non-POU domain-containin	NONO

P22307-6;P22307-2;P22	P22307-6;F	4;4;4;4;4;4	4;4;4;4;4;4	4;4;4;4;4;4	Non-specific lipid-transfer p	SCP2
J3QRU1;P07947;P06241	J3QRU1;P0	3;3;1;1;1;1	3;3;1;1;1;1	3;3;1;1;1;1	Non-specific protein-tyrosir	YES1
Q9UNZ2;Q9UNZ2-5;F2Z	Q9UNZ2;Q	6;6;5;5;3;2	6;6;5;5;3;2	6;6;5;5;3;2	NSFL1 cofactor p47	NSFL1C
Q9BV86-2;Q9BV86	Q9BV86-2;	1;1	1;1	1;1	N-terminal Xaa-Pro-Lys N-m	NTMT1
H0YDS9;Q5T624;P4932	H0YDS9;Q5	3;3;3;3;3;3	3;3;3;3;3;3	3;3;3;3;3;3	Nuclear autoantigenic sperr	NASP
Q09161	Q09161	4	4	4	Nuclear cap-binding proteir	NCBP1
F8WE41;P52298-3;P522	F8WE41;P5	1;1;1	1;1;1	1;1;1	Nuclear cap-binding proteir	NCBP2
D6RH30;P19838;P1983	D6RH30;P1	1;1;1	1;1;1	1;1;1	Nuclear factor NF-kappa-B	NFKB1
Q7Z417	Q7Z417	3	3	3	Nuclear fragile X mental ret	NUFIP2
Q9Y266;A0A0A0MSS4;A	Q9Y266	5;1;1	5;1;1	5;1;1	Nuclear migration protein n	NUDC
Q14980-2;A0A087WY6	Q14980-2;	46;45;45;3	46;45;45;3	46;45;45;3	Nuclear mitotic apparatus p	NUMA1
P57740;P57740-3;P577	P57740;P5	6;4;4;2;1	6;4;4;2;1	6;4;4;2;1	Nuclear pore complex prote	NUP107
Q8WUM0	Q8WUM0	6	6	6	Nuclear pore complex prote	NUP133
P49790-2;P49790;P497	P49790-2;F	6;6;6	6;6;6	6;6;6	Nuclear pore complex prote	NUP153
E9PF10;O75694;O7569	E9PF10;O7	13;13;12;1	13;13;12;1	13;13;12;1	Nuclear pore complex prote	NUP155
G3V198;Q12769;E9PR1	G3V198;Q1	7;7;6;2;2;1	7;7;6;2;2;1	7;7;6;2;2;1	Nuclear pore complex prote	NUP160
Q92621;U3KPX2	Q92621	9;4	9;4	9;4	Nuclear pore complex prote	NUP205
A0A0A0MSW3;P35658-	A0A0A0MS	5;5;5;5;5;5	5;5;5;5;5;5	5;5;5;5;5;5	Nuclear pore complex prote	NUP214
Q9UKX7-2;Q9UKX7	Q9UKX7-2;	1;1	1;1	1;1	Nuclear pore complex prote	NUP50
J3QL54;Q9BW27-2;Q9B	J3QL54;Q9	2;2;2;2;1;1	2;2;2;2;1;1	2;2;2;2;1;1	Nuclear pore complex prote	NUP85
J3KMX1;Q99567;I3L4K7	J3KMX1;Q9	5;5;1;1	5;5;1;1	5;5;1;1	Nuclear pore complex prote	NUP88
H3BVG0;Q8N1F7;Q8N1	H3BVG0;Q8	7;7;5;3;2;2	7;7;5;3;2;2	7;7;5;3;2;2	Nuclear pore complex prote	NUP93
P52948-6;P52948-5;P52	P52948-6;F	6;6;6;5;5;2	6;6;6;5;5;2	6;6;6;5;5;2	Nuclear pore complex prote	NUP98
P37198;M0QXN5	P37198;M0	3;2	3;2	3;2	Nuclear pore glycoprotein p	NUP62
Q8TEM1;Q8TEM1-2	Q8TEM1;Q	4;2	4;2	4;2	Nuclear pore membrane gly	NUP210
Q8TAT6;Q8TAT6-2	Q8TAT6;Q8	1;1	1;1	1;1	Nuclear protein localization	NPLOC4
Q13772;Q13772-3;Q13	Q13772;Q1	6;6;6;4;4;2	6;6;6;4;4;2	6;6;6;4;4;2	Nuclear receptor coactivatc	NCOA4
P61970;H3BRV9	P61970;H3	5;3	5;3	5;3	Nuclear transport factor 2	NUTF2
Q9H1E3;Q9H1E3-2	Q9H1E3	3;1	3;1	3;1	Nuclear ubiquitous casein a	NUCKS1
O15381-3;O15381-4;O1	O15381-3;	1;1;1;1;1	1;1;1;1;1	1;1;1;1;1	Nuclear valosin-containing	NVL
P67809;H0Y449;C9J5V9	P67809;H0	10;9;5;3	10;9;5;3	7;7;4;0	Nuclease-sensitive element	YBX1
Q02818;C9JKZ2	Q02818	3;1	3;1	3;1	Nucleobindin-1	NUCB1
A0A0A0MRM9;Q14978	A0A0A0MF	5;5;5;5;1;1	5;5;5;5;1;1	5;5;5;5;1;1	Nucleolar and coiled-body p	NOLC1
Q9Y3T9	Q9Y3T9	4	4	4	Nucleolar complex protein 2	NOC2L
Q8WTT2	Q8WTT2	1	1	1	Nucleolar complex protein 3	NOC3L
Q9BZE4;Q9BZE4-2;Q9B	Q9BZE4;Q9	10;9;7;5	10;9;7;5	10;9;7;5	Nucleolar GTP-binding prote	GTPBP4
O60287	O60287	1	1	1	Nucleolar pre-ribosomal-as	URB1
Q9H8H0;J3QLQ6;J3QR2	Q9H8H0;J3	2;1;1;1	2;1;1;1	2;1;1;1	Nucleolar protein 11	NOL11
E9PFK5;P78316-2;P783	E9PFK5;P7	1;1;1	1;1;1	1;1;1	Nucleolar protein 14	NOP14
D6RC60;D6RIC3;A0A0C	D6RC60;D6	1;1;1;1;1;1	1;1;1;1;1;1	1;1;1;1;1;1	Nucleolar protein 16	NOP16
O00567;H0Y653;H0YDL	O00567;H0	10;5;5;4	10;5;5;4	10;5;5;4	Nucleolar protein 56	NOP56
Q9Y2X3;H7BZ72;F8WED	Q9Y2X3	10;3;1	10;3;1	10;3;1	Nucleolar protein 58	NOP58
Q9H6R4-4;Q9H6R4;A0A	Q9H6R4-4;	2;2;1;1;1;1	2;2;1;1;1;1	2;2;1;1;1;1	Nucleolar protein 6	NOL6
Q9NR30;Q9NR30-2;A0A	Q9NR30;Q9	18;16;1	18;16;1	17;15;0	Nucleolar RNA helicase 2	DDX21
E9PKP7;P17480-2;P174	E9PKP7;P1	3;3;3;1	3;3;3;1	3;3;3;1	Nucleolar transcription fact	UBTF
P19338;H7BY16;C9JYW	P19338;H7	23;15;1;1;1	23;15;1;1;1	23;15;1;1;1	Nucleolin	NCL
F8WE16;C9JTN7;F8W8I	F8WE16;C9	1;1;1;1;1;1	1;1;1;1;1;1	1;1;1;1;1;1	Nucleolysin TIAR;Nucleolysi	TIA1;TIAL1
P06748;P06748-3;P067	P06748;P0	12;11;11;3	12;11;11;3	12;11;11;3	Nucleophosmin	NPM1
F8VTY2;Q8NFH4	F8VTY2;Q8	1;1	1;1	1;1	Nucleoporin Nup37	NUP37
Q8NFH3;Q8NFH3-2	Q8NFH3	3;1	3;1	3;1	Nucleoporin Nup43	NUP43

Q8NFH5-2;Q8NFH5;C9I	Q8NFH5-2;	5;5;3;3;3;2	5;5;3;3;3;2	5;5;3;3;3;2	Nucleoporin NUP53	NUP35
Q7Z3B4-2;Q7Z3B4-3;Q7	Q7Z3B4-2;(	3;3;3	3;3;3	3;3;3	Nucleoporin p54	NUP54
Q96EE3;Q96EE3-1;K7EL	Q96EE3;Q9	5;5;4;3;3;1	5;5;4;3;3;1	5;5;4;3;3;1	Nucleoporin SEH1	SEH1L
P12270;P12270-2	P12270	14;4	14;4	14;4	Nucleoprotein TPR	TPR
A0A0G2JQK5;Q6DKJ4	A0A0G2JQI	1;1	1;1	1;1	Nucleoredoxin	NXN
P15531;P15531-2;E5RH	P15531;P1!	5;5;2;1	1;1;0;0	1;1;0;0	Nucleoside diphosphate kin	NME1
Q32Q12;P22392-2;P22:	Q32Q12;P2	8;8;7;6;5;4	8;8;7;6;5;4	4;4;4;4;1;0	Nucleoside diphosphate kin	NME1-NMI
H0YHC3;F8W020;F8W1	H0YHC3;F8	3;3;3;3;3;3	3;3;3;3;3;3	3;3;3;3;3;3	Nucleosome assembly prot	NAP1L1
C9JZI7;Q99733;Q99733	C9JZI7;Q99	4;4;4;3;3;3	4;4;4;3;3;3	4;4;4;3;3;3	Nucleosome assembly prot	NAP1L4
Q96RS6-3;Q96RS6-2;Q9	Q96RS6-3;(	2;2;2	2;2;2	2;2;2	NudC domain-containing pr	NUDCD1
E5RFP0;Q8WVJ2	E5RFP0;Q8	1;1	1;1	1;1	NudC domain-containing pr	NUDCD2
C9JTK6;J3KQ32;Q9NTK	C9JTK6;J3K	1;1;1;1	1;1;1;1	1;1;1;1	Obg-like ATPase 1	OLA1
Q56VL3;D6RD77;J3KPI9	Q56VL3;D6	3;2;2;2	3;2;2;2	3;2;2;2	OCIA domain-containing pr	OCIAD2
A0A087WUD3;Q9NRP0	A0A087WU	1;1;1	1;1;1	1;1;1	Oligosaccharyltransferase c	OSTC
Q9NQR4;H7C579;F8WF	Q9NQR4;H	7;6;1	7;6;1	7;6;1	Omega-amidase NIT2	NIT2
Q99650-2;Q99650	Q99650-2;(	1;1	1;1	1;1	Oncostatin-M-specific recep	OSMR
F6WST4;A0A1B0GXE7;(	F6WST4;A0	1;1;1;1	1;1;1;1	1;1;1;1	ORM1-like protein 1;ORM1-	ORMDL1;O
J3QRM9;Q8N138-4;Q8I	J3QRM9;Q	1;1;1	1;1;1	1;1;1	ORM1-like protein 3	ORMDL3
P04181;P04181-2	P04181;P0	10;5	10;5	10;5	Ornithine aminotransferase	OAT
Q92882	Q92882	1	1	1	Osteoclast-stimulating fact	OSTF1
P36551;H0YA22;P3655	P36551	5;1;1	5;1;1	5;1;1	Oxygen-dependent copropo	CPOX
Q9NWT1	Q9NWT1	1	1	1	p21-activated protein kinas	PAK1IP1
Q15645;H0YAL2;Q1564	Q15645;H0	3;2;1	3;2;1	3;2;1	Pachytene checkpoint prote	TRIP13
E9PSE5;E9PIA8;A0A2C9	E9PSE5;E9I	1;1;1;1;1;1	1;1;1;1;1;1	1;1;1;1;1;1	Palmitoyl-protein thioester	PPT1
B4DRN8;Q5W0Z9	B4DRN8;Q	1;1	1;1	1;1	Palmitoyltransferase;Probal	ZDHHC20
Q8WXF1-2;Q8WXF1;X6	Q8WXF1-2	3;3;2	3;3;2	3;3;2	Paraspeckle component 1	PSPC1
O00151	O00151	10	10	10	PDZ and LIM domain protei	PDLIM1
O14908;K7ELJ2;K7EM1	O14908;K7	2;1;1;1;1	2;1;1;1;1	2;1;1;1;1	PDZ domain-containing pro	GIPC1
Q96EY7;Q96EY7-2	Q96EY7;Q9	2;1	2;1	2;1	Pentatricopeptide repeat d	PTCD3
P62937-2	P62937-2	8	1	1	Peptidyl-prolyl cis-trans isor	PPIA
P62937;F8WE65;C9J5S7	P62937;F8	11;7;7;2;2;	11;7;7;2;2;	4;4;4;1;1;1	Peptidyl-prolyl cis-trans isor	PPIA
P23284	P23284	10	10	10	Peptidyl-prolyl cis-trans isor	PPIB
P45877	P45877	1	1	1	Peptidyl-prolyl cis-trans isor	PPIC
P30405;R4GN99;P3040	P30405;R4	4;3;3;2;1;1	3;2;2;1;1;1	3;2;2;1;1;1	Peptidyl-prolyl cis-trans isor	PPIF
P62942;Q5W0X3;A0A0	P62942;Q5	3;2;2;1;1;1	3;2;2;1;1;1	3;2;2;1;1;1	Peptidyl-prolyl cis-trans isor	FKBP1A
P26885;F5H0N4	P26885;F5I	2;1	2;1	2;1	Peptidyl-prolyl cis-trans isor	FKBP2
Q00688	Q00688	3	3	3	Peptidyl-prolyl cis-trans isor	FKBP3
Q02790;H0YFG2;F5H1U	Q02790	12;3;3;1	12;3;3;1	12;3;3;1	Peptidyl-prolyl cis-trans isor	FKBP4
M0R2K9;Q14318;Q143	M0R2K9;Q	1;1;1	1;1;1	1;1;1	Peptidyl-prolyl cis-trans isor	FKBP8
Q9Y3C6	Q9Y3C6	2	2	2	Peptidyl-prolyl cis-trans isor	PPIL1
Q8WUA2	Q8WUA2	1	1	1	Peptidyl-prolyl cis-trans isor	PPIL4
J3KQ48;Q9Y3E5	J3KQ48;Q9	2;2	2;2	2;2	Peptidyl-tRNA hydrolase 2,	PTRH2
E5RGQ4;E9PGW9;E7EV	E5RGQ4;E9	2;2;2;2;2;2	2;2;2;2;2;2	2;2;2;2;2;2	Pericentriolar material 1 pr	PCM1
O60664-4;O60664-3;O6	O60664-4;(	8;8;8;6;4;4	8;8;8;6;4;4	8;8;8;6;4;4	Perilipin-3	PLIN3
Q15269;A0A0B4J2E5;C	Q15269;A0	3;2;1	3;2;1	3;2;1	Periodic tryptophan protein	PWP2
Q06830;A0A0A0MSI0;A	Q06830;A0	15;13;7	15;13;7	11;10;3	Peroxiredoxin-1	PRDX1
P32119;A6NIW5;P3211	P32119	9;4;1	8;4;1	8;4;1	Peroxiredoxin-2	PRDX2
Q13162;H7C3T4;A6NG	Q13162;H7	12;7;4;2	9;4;4;2	9;4;4;2	Peroxiredoxin-4	PRDX4
P30044-2;P30044;P300	P30044-2;F	6;6;5;2	6;6;5;2	6;6;5;2	Peroxiredoxin-5, mitochonc	PRDX5
P30041	P30041	9	9	9	Peroxiredoxin-6	PRDX6

I3L2U4;K7ELT1;I3L0T4;(	I3L2U4;K7E	1;1;1;1;1	1;1;1;1;1	1;1;1;1;1	Peroxisomal acyl-coenzyme	ACOX1
Q92968	Q92968	1	1	1	Peroxisomal membrane prc	PEX13
O75381-2;O75381	O75381-2;(	2;2	2;2	2;2	Peroxisomal membrane prc	PEX14
P51659;E7EWE5;P5165	P51659;E7I	17;14;14;1	17;14;14;1	17;14;14;1	Peroxisomal multifunctiona	HSD17B4
M0QXB5;O95571	M0QXB5;O	1;1	1;1	1;1	Persulfide dioxygenase ETH	ETHE1
B5MCF9;O00541;B3KXD	B5MCF9;O(	8;8;7;7;2	8;8;7;7;2	8;8;7;7;2	Pescadillo homolog	PES1
C9JZE2;C9IZ09;Q8IYB4-8	C9JZE2;C9I	1;1;1;1;1;1	1;1;1;1;1;1	1;1;1;1;1;1	PEX5-related protein	PEX5L
Q7RTV0	Q7RTV0	2	2	2	PHD finger-like domain-con	PHF5A
K7ER00;Q9Y285;K7ER16	K7ER00;Q9	3;3;2;2;1	3;3;2;2;1	3;3;2;2;1	Phenylalanine--tRNA ligase	FARSA
Q9NSD9;Q9NSD9-2	Q9NSD9;Q	10;8	10;8	10;8	Phenylalanine--tRNA ligase	FARSB
Q8WWQ0	Q8WWQ0	2	2	2	PH-interacting protein	PHIP
Q00325-2;Q00325;F8V\	Q00325-2;(	8;8;7;2;2;2	8;8;7;2;2;2	8;8;7;2;2;2	Phosphate carrier protein, r	SLC25A3
O95674	O95674	1	1	1	Phosphatidate cytidylyltran	CDS2
P30086	P30086	6	6	6	Phosphatidylethanolamine-	PEBP1
C9J3E3;F8WDN7;E9PGZ	C9J3E3;F8V	1;1;1;1;1	1;1;1;1;1	1;1;1;1;1	Phosphatidylinositide phos	SACM1L
A0A0A0MSW4;P48739;	A0A0A0MS	4;4;4;4;3;2	4;4;4;4;3;2	4;4;4;4;3;2	Phosphatidylinositol transfe	PITPNB;PIT
Q13492-4;Q13492-3;Q1	Q13492-4;(	4;4;4;4;4;2	4;4;4;4;4;2	4;4;4;4;4;2	Phosphatidylinositol-bindin	PICALM
P48651-3;P48651-2;P48	P48651-3;F	1;1;1	1;1;1	1;1;1	Phosphatidylserine synthas	PTDSS1
Q16822;A0A0A0MS74;I	Q16822;A0	4;3;3;3;2;2	4;3;3;3;2;2	4;3;3;3;2;2	Phosphoenolpyruvate carbo	PCK2
P36871;P36871-2;P368	P36871;P3	8;6;5	8;6;5	8;6;5	Phosphoglucomutase-1	PGM1
Q96G03-2;Q96G03	Q96G03-2;	1;1	1;1	1;1	Phosphoglucomutase-2	PGM2
P00558-2;P00558;P072	P00558-2;F	15;15;3	15;15;3	15;15;3	Phosphoglycerate kinase 1	PGK1
P18669;P15259;Q8N0Y	P18669	11;3;3	11;3;3	11;3;3	Phosphoglycerate mutase 1	PGAM1
Q8IV08;M0R3G9	Q8IV08;M(	2;1	2;1	2;1	Phospholipase D3	PLD3
C9J7K9;O15162-2;O151	C9J7K9;O1	2;2;2;1;1;1	2;2;2;1;1;1	2;2;2;1;1;1	Phospholipid scramblase 1	PLSCR1
C9JDU5;C9JJS3;C9K0K7;	C9JDU5;C9	1;1;1;1;1;1	1;1;1;1;1;1	1;1;1;1;1;1	Phosphoribosyl pyrophosph	PRPSAP2
Q9Y617;Q9Y617-2	Q9Y617;Q	8;7	8;7	8;7	Phosphoserine aminotransf	PSAT1
C9JBI3;P78330	C9JBI3;P78	1;1	1;1	1;1	Phosphoserine phosphatase	PSPH
Q9H307	Q9H307	3	3	3	Pinin	PNN
O00625	O00625	1	1	1	Pirin	PIR
Q13835-2;Q13835	Q13835-2;(	2;2	2;2	2;2	Plakophilin-1	PKP1
Q99959-2;Q99959;A0A(	Q99959-2;(	2;2;1	2;2;1	2;2;1	Plakophilin-2	PKP2
Q9Y446-2;Q9Y446;E9P(	Q9Y446-2;(	9;8;4;3;3;3	9;8;4;3;3;3	9;8;4;3;3;3	Plakophilin-3	PKP3
P20020-5;P20020-2;P2(	P20020-5;F	3;3;3;3;3;3	3;3;3;3;3;3	3;3;3;3;3;3	Plasma membrane calcium-	ATP2B1;AT
Q8NC51-3;Q8NC51;Q8N	Q8NC51-3;	11;11;10;1(	11;11;10;1(	11;11;10;1(	Plasminogen activator inhib	SERBP1
P05120;H7C004;H7BYS	P05120;H7	6;4;4;1;1;1	6;4;4;1;1;1	6;4;4;1;1;1	Plasminogen activator inhib	SERPINB2;S
P13796;Q5TBN3;P1379	P13796	19;8;3;2	19;8;3;2	13;5;3;2	Plastin-2	LCP1
A0A0A0MSQ0;P13797-3	A0A0A0MS	16;16;16;1(	10;10;10;1(	10;10;10;1(	Plastin-3	PLS3
P43034;I3L3N5;I3L495	P43034;I3L	5;3;1	5;3;1	5;3;1	Platelet-activating factor ac	PAFAH1B1
P68402-3;P68402-2;P68	P68402-3;F	1;1;1;1	1;1;1;1	1;1;1;1	Platelet-activating factor ac	PAFAH1B2
R4GND3;Q8WV24	R4GND3;Q	1;1	1;1	1;1	Pleckstrin homology-like do	PHLDA1
Q15149-4;Q15149-7;Q1	Q15149-4;(	102;101;10	102;101;10	102;101;10	Plectin	PLEC
O43660-2;O43660;H0Y/	O43660-2;(	2;2;1	2;2;1	2;2;1	Pleiotropic regulator 1	PLRG1
O75051	O75051	1	1	1	Plexin-A2	PLXNA2
O15031;A6QRG9;A6QR	O15031	6;2;1;1	6;2;1;1	6;2;1;1	Plexin-B2	PLXNB2
P09874;Q5VX84;Q5VX8	P09874	17;1;1	17;1;1	17;1;1	Poly [ADP-ribose] polymera	PARP1
Q9NVV4;Q9NVV4-2	Q9NVV4;Q	1;1	1;1	1;1	Poly(A) RNA polymerase, m	MTPAP
Q15365;F8VTZ0	Q15365	9;1	9;1	7;0	Poly(rC)-binding protein 1	PCBP1
Q15366-6;Q15366-3;Q1	Q15366-6;(	8;8;7;7;7;7	6;6;5;5;5;5	6;6;5;5;5;5	Poly(rC)-binding protein 2;P	PCBP2;PCB
A0A0J9YYL3;A0A0J9YVF	A0A0J9YYL	8;8;8;8;8;7	8;8;8;8;8;7	8;8;8;8;8;7	Poly(U)-binding-splicing fac	PUF60

P11940;A0A087WTT1;E	P11940;A0.	18;17;17;1	18;17;17;1	14;13;13;1	Polyadenylate-binding prot	PABPC1;PA
Q86U42-2;Q86U42;H0Y	Q86U42-2;	2;2;1;1;1;1	2;2;1;1;1;1	2;2;1;1;1;1	Polyadenylate-binding prot	PABPN1
B1ANR0;Q13310-2;Q13	B1ANR0;Q	7;7;7;7;4;3	3;3;3;3;2;1	3;3;3;3;2;1	Polyadenylate-binding prot	PABPC4
D6REB4;Q9H074-3;Q9H	D6REB4;Q	2;2;2;2	2;2;2;2	2;2;2;2	Polyadenylate-binding prot	PAIP1
B4DEM9;Q9Y2S7	B4DEM9;Q	1;1	1;1	1;1	Polymerase delta-interactin	POLDIP2
F6VRR5;Q9BY77-2;Q9B	F6VRR5;Q9	2;2;2;1;1;1	2;2;2;1;1;1	2;2;2;1;1;1	Polymerase delta-interactin	POLDIP3;PI
Q6NZI2;Q6NZI2-2;Q6N	Q6NZI2;Q6	10;6;4	10;6;4	10;6;4	Polymerase I and transcript	PTRF
Q10471;Q10471-2;F8V	Q10471	9;2;1;1;1	9;2;1;1;1	9;2;1;1;1	Polypeptide N-acetylgalacto	GALNT2
E7EUL0;Q14435;C9J2C3	E7EUL0;Q1	3;3;1	3;3;1	3;3;1	Polypeptide N-acetylgalacto	GALNT3
A6NLN1;A0A0U1RRM4;	A6NLN1;A	10;10;10;1	10;10;10;1	8;8;8;8;8;4	Polypyrimidine tract-bindin	PTBP1
O95758-7;O95758-1;O	O95758-7;	8;8;8;8;8;8	6;6;6;6;6;6	6;6;6;6;6;6	Polypyrimidine tract-bindin	PTBP3
Q8TCS8;H7BXF6;F8WBI	Q8TCS8;H7	3;2;1;1	3;2;1;1	3;2;1;1	Polyribonucleotide nucleoti	PNPT1
A6NP52;O60831	A6NP52;O6	1;1	1;1	1;1	PRA1 family protein 2	PRAF2
E5RGS4;O60925	E5RGS4;O6	1;1	1;1	1;1	Prefoldin subunit 1	PFDN1
Q9UHV9	Q9UHV9	1	1	1	Prefoldin subunit 2	PFDN2
E9PQY2;Q9NQP4	E9PQY2;Q	1;1	1;1	1;1	Prefoldin subunit 4	PFDN4
P02545;P02545-3;P025	P02545;P0	39;38;37;3	39;38;37;3	38;37;36;3	Prelamin-A/C;Lamin-A/C	LMNA
Q9UMS4;F5GY56;F5H2I	Q9UMS4	10;3;2;2;1	10;3;2;2;1	10;3;2;2;1	Pre-mRNA-processing facto	PRPF19
O75400-2;O75400-3;O7	O75400-2;	2;2;2	2;2;2	2;2;2	Pre-mRNA-processing facto	PRPF40A
O94906;O94906-2	O94906;O	9;8	9;8	9;8	Pre-mRNA-processing facto	PRPF6
Q6P2Q9;I3L0J9;I3L3Z8	Q6P2Q9	16;4;2	16;4;2	16;4;2	Pre-mRNA-processing-splici	PRPF8
Q8NAV1	Q8NAV1	1	1	1	Pre-mRNA-splicing factor 3	PRPF38A
O43143	O43143	6	6	6	Pre-mRNA-splicing factor A	DHX15
Q9ULR0;Q9ULR0-2;Q9	Q9ULR0;Q	1;1;1	1;1;1	1;1;1	Pre-mRNA-splicing factor IS	ISY1
Q9UHG3-2;Q9UHG3;C9	Q9UHG3-2	2;2;1;1;1	2;2;1;1;1	2;2;1;1;1	Prenylcysteine oxidase 1	PCYOX1
Q8IY81	Q8IY81	7	7	7	pre-rRNA processing protei	FTSJ3
Q2NL82;I3L1Q5	Q2NL82;I3	4;3	4;3	4;3	Pre-rRNA-processing protei	TSR1
Q5JRX3-3;Q5JRX3;Q5JR	Q5JRX3-3;	6;6;6;2;1;1	6;6;6;2;1;1	6;6;6;2;1;1	Presequence protease, mito	PITRM1
Q96IZ0	Q96IZ0	1	1	1	PRKC apoptosis WT1 regula	PAWR
P46087;P46087-4;P460	P46087;P4	14;14;13;1	14;14;13;1	14;14;13;1	Probable 28S rRNA (cytosin	NOP2
F5GYY1;F5GXL6;F5GXJ8	F5GYY1;F5	1;1;1;1;1;1	1;1;1;1;1;1	1;1;1;1;1;1	Probable ATP-dependent R	DDX11;DD
A0A1X7SBZ2;A0A1W2P	A0A1X7SB	18;18;18;1	18;18;18;1	11;11;11;1	Probable ATP-dependent R	DDX17
B7Z6D5;Q96GQ7;A0A0	B7Z6D5;Q	7;7;4;1	7;7;4;1	7;7;4;1	Probable ATP-dependent R	DDX27
F8WAJ0;Q9H8H2-4;Q9	F8WAJ0;Q	2;2;2;2	2;2;2;2	2;2;2;2	Probable ATP-dependent R	DDX31
A0A0C4DG89;Q7L014;D	A0A0C4DG	7;7;5	7;7;5	7;7;5	Probable ATP-dependent R	DDX46
Q9H0S4-2;Q9H0S4	Q9H0S4-2;	2;2	2;2	2;2	Probable ATP-dependent R	DDX47
J3KTA4;P17844-2;P178	J3KTA4;P1	17;17;17;7	10;10;10;2	10;10;10;2	Probable ATP-dependent R	DDX5
F8WDT8;G3V0G3;H7C3	F8WDT8;G	1;1;1;1;1	1;1;1;1;1	1;1;1;1;1	Probable ATP-dependent R	DDX56
P26196;Q8IV96	P26196;Q8	2;1	2;1	2;1	Probable ATP-dependent R	DDX6
Q9HA77;H0YFV1;F5H62	Q9HA77;H	2;1;1	2;1;1	2;1;1	Probable cysteine--tRNA lig	CARS2
A0A0A0MRP6;B7ZLQ5;	A0A0A0M	3;3;3;3	2;2;2;2	2;2;2;2	Probable global transcriptio	SMARCA1
Q96K78	Q96K78	1	1	1	Probable G-protein coupled	GPR128
H3BMQ2;Q9UHA3	H3BMQ2;C	1;1	1;1	1;1	Probable ribosome biogene	RSL24D1
Q9Y4C8	Q9Y4C8	2	2	2	Probable RNA-binding prote	RBM19
H7C2Q8;Q99848	H7C2Q8;Q	7;7	7;7	7;7	Probable rRNA-processing	EBNA1BP2
Q8NBJ5;M0QX72;M0Q	Q8NBJ5	9;3;2	9;3;2	9;3;2	Procollagen galactosyltrans	COLGALT1
Q02809;Q02809-2	Q02809;Q	4;4	4;4	4;4	Procollagen-lysine,2-oxoglu	PLOD1
E7ETU9;O00469;O0046	E7ETU9;O0	2;2;2;1	2;2;2;1	2;2;2;1	Procollagen-lysine,2-oxoglu	PLOD2
O60568;H7C2V1;H7C2S	O60568;H7	7;4;2;1	7;4;2;1	7;4;2;1	Procollagen-lysine,2-oxoglu	PLOD3
P07737;K7EJ44;CON__	P07737;K7	10;8;4;3	10;8;4;3	10;8;4;3	Profilin-1	PFN1

Q8WUM4;Q8WUM4-2;	Q8WUM4;	7;7;4;2;2;2	7;7;4;2;2;2	7;7;4;2;2;2	Programmed cell death 6-in	PDCD6IP
Q53EL6-2;Q53EL6	Q53EL6-2;	1;1	1;1	1;1	Programmed cell death pro	PDCD4
A0A024QZ42;O75340-2	A0A024QZ	2;2;2	2;2;2	2;2;2	Programmed cell death pro	PDCD6
P35232;C9JW96;C9JZ20	P35232;C9	13;12;10;1	13;12;10;1	13;12;10;1	Prohibitin	PHB
Q99623;J3KPX7;F5GY37	Q99623;J3	17;16;15;1	17;16;15;1	17;16;15;1	Prohibitin-2	PHB2
P12004	P12004	7	7	7	Proliferating cell nuclear an	PCNA
Q9UQ80;Q9UQ80-2;F8	Q9UQ80;Q	16;13;9;6;5	16;13;9;6;5	16;13;9;6;5	Proliferation-associated prc	PA2G4
P13674-3;P13674-2;P1	P13674-3;F	6;6;6	6;6;6	6;6;6	Prolyl 4-hydroxylase subuni	P4HA1
O15460-2;O15460	O15460-2;	3;3	3;3	3;3	Prolyl 4-hydroxylase subuni	P4HA2
P48147	P48147	3	3	3	Prolyl endopeptidase	PREP
C9JIZ6;P07602;P07602-	C9JIZ6;P07	8;8;8;8;7;1	8;8;8;8;7;1	8;8;8;8;7;1	Prosaposin;Saposin-A;Sapos	PSAP
O14684	O14684	2	2	2	Prostaglandin E synthase	PTGES
X6RJ95;Q9H7Z7;A6NHH	X6RJ95;Q9	3;3;2	3;3;2	3;3;2	Prostaglandin E synthase 2;	PTGES2
A0A087WYT3;Q15185-	A0A087WY	5;5;5;4;3;3	5;5;5;4;3;3	5;5;5;4;3;3	Prostaglandin E synthase 3	PTGES3
Q9P2B2	Q9P2B2	2	2	2	Prostaglandin F2 receptor n	PTGFRN
Q14914-2;Q14914;F2Z3	Q14914-2;	6;6;3;1	6;6;3;1	6;6;3;1	Prostaglandin reductase 1	PTGR1
Q16186;A0A087WX59	Q16186;A0	2;1	2;1	2;1	Proteasomal ubiquitin recep	ADRM1
Q06323-3;Q06323;Q06	Q06323-3;	5;5;5;1	5;5;5;1	5;5;5;1	Proteasome activator comp	PSME1
H0YM70;A0A087X1Z3;C	H0YM70;A	2;2;2;1	2;2;2;1	2;2;2;1	Proteasome activator comp	PSME2
K7ENH2;K9J957;P61289	K7ENH2;K9	3;3;3;3;3;2	3;3;3;3;3;2	3;3;3;3;3;2	Proteasome activator comp	PSME3
H0Y555;F5H4Z3;Q5QPN	H0Y555;F5	1;1;1;1	1;1;1;1	1;1;1;1	Proteasome inhibitor PI31 s	PSMF1
C9JCK5;A0A024RA52;P2	C9JCK5;A0	2;2;2	2;2;2	2;2;2	Proteasome subunit alpha t	PSMA2
H0YMZ1;H0YN18;H0YL6	H0YMZ1;H	5;5;5;5;4;4	5;5;5;5;4;4	5;5;5;5;4;4	Proteasome subunit alpha t	PSMA4
G3V295;G3V5Z7;P6090	G3V295;G3	7;7;7;6;5;3	7;7;7;6;5;3	7;7;7;6;5;3	Proteasome subunit alpha t	PSMA6
P25786;P25786-2;F5GX	P25786;P2	11;11;9;5	11;11;9;5	11;11;9;5	Proteasome subunit alpha t	PSMA1
P25788-2;P25788;G3V3	P25788-2;F	3;3;1;1;1	3;3;1;1;1	3;3;1;1;1	Proteasome subunit alpha t	PSMA3
P28066;P28066-2	P28066;P2	8;5	8;5	8;5	Proteasome subunit alpha t	PSMA5
O14818;O14818-2;H0Y	O14818;O1	9;7;6;4;4;4	9;7;6;4;4;4	9;7;6;4;4;4	Proteasome subunit alpha t	PSMA7
X5D2R7;P28062-2;P280	X5D2R7;P2	3;3;3;2;2	3;3;3;2;2	3;3;3;2;2	Proteasome subunit beta ty	PSM8;PSM
A2ACR0;A0A0G2JJQ0;A	A2ACR0;A0	1;1;1;1;1;1	1;1;1;1;1;1	1;1;1;1;1;1	Proteasome subunit beta ty	PSMB9
P20618	P20618	5	5	5	Proteasome subunit beta ty	PSMB1
P49721;A0A087WVV1	P49721;A0	2;1	2;1	2;1	Proteasome subunit beta ty	PSMB2
A0A087WXQ8;A0A087V	A0A087WX	2;2;2;2	2;2;2;2	2;2;2;2	Proteasome subunit beta ty	PSMB3
P28070	P28070	2	2	2	Proteasome subunit beta ty	PSMB4
P28074;P28074-3;H0YJ	P28074;P2	7;5;3;3	7;5;3;3	7;5;3;3	Proteasome subunit beta ty	PSMB5
P28072;I3L3X7;A0A087	P28072	3;1;1	3;1;1	3;1;1	Proteasome subunit beta ty	PSMB6
Q99436;Q5TBG5;Q994	Q99436;Q5	2;1;1	2;1;1	2;1;1	Proteasome subunit beta ty	PSMB7
J3KN16;Q5VYK3;R4GM	J3KN16;Q5	2;2;1;1	2;2;1;1	2;2;1;1	Proteasome-associated pro	KIAA0368;E
Q9NY61;A0A087WW41	Q9NY61	3;1;1	3;1;1	3;1;1	Protein AATF	AATF
Q99873-3;E9PKG1;Q99	Q99873-3;	7;6;6;6;5;3	7;6;6;6;5;3	7;6;6;6;5;3	Protein arginine N-methyltr	PRMT1
H0YJX6;O14744-3;O147	H0YJX6;O1	2;2;2;2;2;1	2;2;2;2;2;1	2;2;2;2;2;1	Protein arginine N-methyltr	PRMT5
Q8TD16;Q8TD16-2	Q8TD16;Q	1;1	1;1	1;1	Protein bicaudal D homolog	BICD2
P41223;P41223-2;C9JC	P41223;P4	2;2;1	2;2;1	2;2;1	Protein BUD31 homolog	BUD31
F8W031;F8VXJ7;Q9Y2B	F8W031;F8	5;4;4;3;2;1	4;4;4;3;1;1	4;4;4;3;1;1	Protein canopy homolog 2	CNPY2
A0A0J9YYJ0;Q8N129	A0A0J9YYJ	2;2	2;2	2;2	Protein canopy homolog 4	CNPY4
Q9UKY7-2;Q9UKY7;D6R	Q9UKY7-2;	3;3;2;2;2;2	3;3;2;2;2;2	3;3;2;2;2;2	Protein CDV3 homolog	CDV3
A6NLH6;Q9P003	A6NLH6;Q	1;1	1;1	1;1	Protein cornichon homolog	CNIH4
C9IZG4;O60888-3;O608	C9IZG4;O6	1;1;1;1	1;1;1;1	1;1;1;1	Protein CutA	CUTA
Q99497;K7ELW0;K7EN2	Q99497;K7	6;5;3	6;5;3	6;5;3	Protein deglycase DJ-1	PARK7
P35659;B4DFG0;P3565	P35659;B4	5;4;4;2;2;2	5;4;4;2;2;2	5;4;4;2;2;2	Protein DEK	DEK

A0A140T8Z0;A0A0G2JH	A0A140T8Z	9;9;9;9;9;8	9;9;9;9;9;8	9;9;9;9;9;8	Protein diaphanous homolo	DIAPH1
P07237;H7BZ94;H0Y3Z3	P07237;H7	22;20;12;1	22;20;12;1	22;20;12;1	Protein disulfide-isomerase	P4HB
P30101	P30101	22	22	17	Protein disulfide-isomerase	PDIA3
P13667	P13667	14	14	14	Protein disulfide-isomerase	PDIA4
Q15084-3;Q15084;Q15	Q15084-3;	13;13;13;1	13;13;13;1	13;13;13;1	Protein disulfide-isomerase	PDIA6
H3BPB3;B4DIE3;Q96JJ7	H3BPB3;B4	1;1;1;1	1;1;1;1	1;1;1;1	Protein disulfide-isomerase	TMX3
Q8WYP5;Q8WYP5-3;Q8	Q8WYP5;Q	4;4;4;1	4;4;4;1	4;4;4;1	Protein ELYS	AHCTF1
P49257	P49257	9	9	9	Protein ERGIC-53	LMAN1
H7C3D5;Q8NC44	H7C3D5;Q8	1;1	1;1	1;1	Protein FAM134A	FAM134A
Q49AJ0-3;Q49AJ0-4;Q4	Q49AJ0-3;	2;2;2;2;1	2;2;2;2;1	2;2;2;2;1	Protein FAM135B	FAM135B
F8W7Q4;Q96A26;E9PH	F8W7Q4;Q	3;3;2	3;3;2	3;3;2	Protein FAM162A	FAM162A
K7ELZ8;K7EIY1;K7ENN5	K7ELZ8;K7E	1;1;1;1;1	1;1;1;1;1	1;1;1;1;1	Protein FAM32A	FAM32A
Q92520;C9JP35;C9JMN	Q92520	6;2;2	6;2;2	6;2;2	Protein FAM3C	FAM3C
Q9NUQ9;Q9NUQ9-2;E5	Q9NUQ9;Q	3;2;1;1;1;1	3;2;1;1;1;1	3;2;1;1;1;1	Protein FAM49B	FAM49B
Q6ZRV2;J3KPS2	Q6ZRV2;J3	22;19	22;19	22;19	Protein FAM83H	FAM83H
Q8NCA5-2;Q8NCA5	Q8NCA5-2;	1;1	1;1	1;1	Protein FAM98A	FAM98A
Q52LJ0;Q52LJ0-2	Q52LJ0;Q5	3;3	3;3	3;3	Protein FAM98B	FAM98B
Q13045-2;Q13045-3;Q1	Q13045-2;	2;2;2;1;1;1	2;2;2;1;1;1	2;2;2;1;1;1	Protein flightless-1 homolog	FLII
G5E994;Q5VW38-2;Q5	G5E994;Q5	1;1;1	1;1;1	1;1;1	Protein GPR107	GPR107
M0R2E1;M0QZW9;M0F	M0R2E1;M	1;1;1;1;1	1;1;1;1;1	1;1;1;1;1	Protein GPR108	GPR108
P78504-2;P78504;Q9Y2	P78504-2;F	2;2;1;1	2;2;1;1	2;2;1;1	Protein jagged-1;Protein jag	JAG1;JAG2
A0A0U1RR22;Q9UNF0-	A0A0U1RR	2;2;2;1;1;1	2;2;2;1;1;1	2;2;2;1;1;1	Protein kinase C and casein	PACSIN2
E9PIE3;Q969G5	E9PIE3;Q96	2;2	2;2	2;2	Protein kinase C delta-bindi	PRKCDBP
P41743	P41743	1	1	1	Protein kinase C iota type	PRKCI
C9JEN3;C9IYT2;F8WDY4	C9JEN3;C9I	2;2;2;2;2;2	2;2;2;2;2;2	2;2;2;2;2;2	Protein lifeguard 3	TMBIM1
G3V1D4;Q9NUP9;H0YI	G3V1D4;Q	2;2;1;1;1;1	2;2;1;1;1;1	2;2;1;1;1;1	Protein lin-7 homolog C;Pro	LIN7C;LIN7
Q9C0E8-3;Q9C0E8-2;Q	Q9C0E8-3;	2;2;2;2	2;2;2;2	2;2;2;2	Protein lunapark	LNP
Q86UE4;E5RJU9;H0YB5	Q86UE4;E5	5;4;2	5;4;2	5;4;2	Protein LYRIC	MTDH
F5H6P7;P61326;Q96A7	F5H6P7;P6	2;2;2;1;1;1	2;2;2;1;1;1	2;2;2;1;1;1	Protein mago nashi homolo	MAGOHB;
H0YBV6;Q9BXY0	H0YBV6;Q	1;1	1;1	1;1	Protein MAK16 homolog	MAK16
Q969L2	Q969L2	1	1	1	Protein MAL2	MAL2
Q92597;Q92597-3;Q92	Q92597;Q	9;8;8;4;3;2	9;8;8;4;3;2	9;8;8;4;3;2	Protein NDRG1	NDRG1
Q9BZQ8	Q9BZQ8	4	4	4	Protein Niban	FAM129A
Q9BPW8;F8WCR5;H7C2	Q9BPW8;F	2;1;1	2;1;1	2;1;1	Protein NipSnap homolog 1	NIPSNAP1
Q9UFN0	Q9UFN0	1	1	1	Protein NipSnap homolog 3	NIPSNAP3A
Q96C90	Q96C90	2	2	2	Protein phosphatase 1 regu	PPP1R14B
B5MBZ8;C9JD73;C9J17	B5MBZ8;C	1;1;1;1;1;1	1;1;1;1;1;1	1;1;1;1;1;1	Protein phosphatase 1 regu	PPP1R7
O15355	O15355	5	5	5	Protein phosphatase 1G	PPM1G
P29590-4;P29590-2;P2	P29590-4;F	12;12;12;1	12;12;12;1	12;12;12;1	Protein PML	PML
E7EPN9;Q9Y520-2;Q9Y	E7EPN9;Q	2;2;2;2;2;2	2;2;2;2;2;2	2;2;2;2;2;2	Protein PRRC2C	PRRC2C
Q9P258	Q9P258	7	7	7	Protein RCC2	RCC2
Q5T092;A0A0A0MR06;	Q5T092;A0	1;1;1;1	1;1;1;1	1;1;1;1	Protein RER1	RER1
Q14690	Q14690	6	6	6	Protein RRP5 homolog	PDCD11
R4GN98;P06703	R4GN98;PC	2;2	2;2	2;2	Protein S100;Protein S100-A	S100A6
P60903	P60903	3	3	3	Protein S100-A10	S100A10
P31949	P31949	4	4	4	Protein S100-A11;Protein S	S100A11
Q99584	Q99584	1	1	1	Protein S100-A13	S100A13
Q9HCY8	Q9HCY8	5	5	5	Protein S100-A14	S100A14
Q96FQ6	Q96FQ6	2	2	2	Protein S100-A16	S100A16
P29034;R4GN49;Q5RH	P29034;R4	5;3;2	5;3;2	5;3;2	Protein S100-A2	S100A2

O43819	O43819	2	2	2	Protein SCO2 homolog, mit	SCO2
P55735;P55735-2;P557	P55735;P5	4;3;3;3;2	4;3;3;3;2	4;3;3;3;2	Protein SEC13 homolog	SEC13
Q01105-2;A0A0C4DFV9	Q01105-2;/	8;7;7;7;7;4	8;7;7;7;7;4	8;7;7;7;7;4	Protein SET;Protein SETSIP	SET;SETSIP
P18583-6;P18583-2;P18	P18583-6;F	4;4;4;4;4;4	4;4;4;4;4;4	4;4;4;4;4;4	Protein SON	SON
H3BT44;H3BPQ9;H3BR8	H3BT44;H3	1;1;1;1;1;1	1;1;1;1;1;1	1;1;1;1;1;1	Protein spinster homolog 1	SPNS1
H7C4R5;Q969Z0-2;Q969	H7C4R5;Q9	2;2;2	2;2;2	2;2;2	Protein TBRG4	TBRG4
Q92734-4;Q92734-3;Q9	Q92734-4;(	4;4;4;4;3;3	4;4;4;4;3;3	4;4;4;4;3;3	Protein TFG	TFG
G5EA31;P53992-2;P539	G5EA31;P5	2;2;2	2;2;2	2;2;2	Protein transport protein Se	SEC24C
D6RHZ5;H7BXG7;D6REX	D6RHZ5;H7	1;1;1;1;1;1	1;1;1;1;1;1	1;1;1;1;1;1	Protein transport protein Se	SEC31A
B4DR61;P61619;P61619	B4DR61;P6	4;4;3;2;2;2	4;4;3;2;2;2	4;4;3;2;2;2	Protein transport protein Se	SEC61A1;SI
P60468;S4R3B5	P60468	3;1	3;1	3;1	Protein transport protein Se	SEC61B
P60059	P60059	1	1	1	Protein transport protein Se	SEC61G
A0A1W2PNX8;Q9H3U1	A0A1W2PN	1;1;1	1;1;1	1;1;1	Protein unc-45 homolog A	UNC45A
E9PS11;A6NGW1;O950	E9PS11;A6I	1;1;1	1;1;1	1;1;1	Protein YIF1A	YIF1A
Q969M3;Q969M3-3	Q969M3;Q	1;1	1;1	1;1	Protein YIPF5	YIPF5
A0A0A0MRJ6;H7BY58;F	A0A0A0MF	5;5;5;5;4;2	5;5;5;5;4;2	5;5;5;5;4;2	Protein-L-isoaspartate O-m	PCMT1
Q5TG12;E9PGC5;Q1526	Q5TG12;E9	3;3;3;3;3;3	3;3;3;3;3;3	3;3;3;3;3;3	Protein-tyrosine-phosphata	PTPRK
B8ZZQ6;P06454-2;P064	B8ZZQ6;P0	5;5;5;4;4;4	5;5;5;4;4;4	5;5;5;4;4;4	Prothymosin alpha;Prothym	PTMA
Q08174;Q08174-2;D6R	Q08174;Q0	7;7;2;1;1	7;7;2;1;1	7;7;2;1;1	Protocadherin-1	PCDH1
O60245;O60245-2;H0Y	O60245;O6	3;3;2;1	3;3;2;1	3;3;2;1	Protocadherin-7	PCDH7
P00491;G3V5M2;G3V2I	P00491;G3	7;5;3;1	7;5;3;1	7;5;3;1	Purine nucleoside phospho	PNP
J3QSB7;B4DUC8;Q1312	J3QSB7;B4I	4;4;4;4;4;4	4;4;4;4;4;4	4;4;4;4;4;4	Purine nucleoside phospho	MTAP
E9PLK3;P55786;P55786	E9PLK3;P5	8;8;6;4;2;1	8;8;6;4;2;1	8;8;6;4;2;1	Puromycin-sensitive amino	NPEPPS;NP
O43865-2;O43865;H0Y8	O43865-2;(	2;2;1;1;1;1	2;2;1;1;1;1	2;2;1;1;1;1	Putative adenosylhomocyst	AHCYL1;AH
Q4W5N1	Q4W5N1	1	1	1	Putative ATP-binding casset	ABCA11P
H7BXY3;Q7L2E3-3;Q7L2	H7BXY3;Q7	1;1;1;1	1;1;1;1	1;1;1;1	Putative ATP-dependent RN	DHX30
Q5VTE0;P68104;A0A08	Q5VTE0;P6	19;19;16;1	19;19;16;1	11;11;10;1	Putative elongation factor 1	EEF1A1P5;I
Q58FF8	Q58FF8	10	1	1	Putative heat shock protein	HSP90AB2I
Q58FF6	Q58FF6	6	1	1	Putative heat shock protein	HSP90AB4I
Q5JR04;Q9HCE1;Q9HCE	Q5JR04;Q9	3;3;1	3;3;1	3;3;1	Putative helicase MOV-10	MOV10
Q5T280;R4GNG4	Q5T280;R4	2;1	2;1	2;1	Putative methyltransferase	C9orf114
K7EMM8;Q49A26-5;Q4	K7EMM8;C	1;1;1;1;1;1	1;1;1;1;1;1	1;1;1;1;1;1	Putative oxidoreductase GL	GLYR1
A0A087WWP4;Q96T37	A0A087WV	2;2;2;2;2	2;2;2;2;2	2;2;2;2;2	Putative RNA-binding prote	RBM15
Q96HJ9-2;A0A0A6YYJ8;	Q96HJ9-2;/	3;3;3;3;3;1	3;3;3;3;3;1	3;3;3;3;3;1	Putative RNA-binding prote	LUC7L2
Q5T440	Q5T440	1	1	1	Putative transferase CAF17,	IBA57
A6NIL9	A6NIL9	1	1	1	Putative uncharacterized pr	CRYM-AS1
A0A087WTV6;J3KR12;C	A0A087WT	2;2;2;1;1;1	2;2;2;1;1;1	2;2;2;1;1;1	Pyrroline-5-carboxylate red	PYCR2;PYC
P11498;E9PS68;P11498	P11498;E9I	8;4;3;2	8;4;3;2	8;4;3;2	Pyruvate carboxylase, mito	PC
P08559-3;P08559;P085	P08559-3;F	3;3;3;3;2;2	3;3;3;3;2;2	3;3;3;3;2;2	Pyruvate dehydrogenase E1	PDHA1
P11177-3;P11177-2;P11	P11177-3;F	5;5;5;3;2	5;5;5;3;2	5;5;5;3;2	Pyruvate dehydrogenase E1	PDHB
O00330-3;O00330	O00330-3;(	1;1	1;1	1;1	Pyruvate dehydrogenase pr	PDHX
P14618;P14618-3;B4DN	P14618;P1	39;34;32;1	39;34;32;1	4;4;4;0;0;0	Pyruvate kinase PKM;Pyruv	PKM
P14618-2;H3BTN5;H3B	P14618-2;H	36;33;22;2	1;1;0;1	1;1;0;1	Pyruvate kinase PKM;Pyruv	PKM
P50395;P50395-2;Q5SX	P50395;P5	13;11;4;3;3	13;11;4;3;3	13;11;4;3;3	Rab GDP dissociation inhibi	GDI2
Q9H0H5;H0YIK5;F8VZ4	Q9H0H5;H	3;2;1;1	3;2;1;1	3;2;1;1	Rac GTPase-activating prote	RACGAP1
Q6IAA8;H0YFI1;F5H479	Q6IAA8;H0	4;2;2;2;2	4;2;2;2;2	4;2;2;2;2	Ragulator complex protein I	LAMTOR1
R4GMU8;E9PLX3;A0A0	R4GMU8;E	1;1;1;1	1;1;1;1	1;1;1;1	Ragulator complex protein I	LAMTOR5
P46060;H0Y4Q3;B0QYT	P46060;H0	12;6;3;3;1	12;6;3;3;1	12;6;3;3;1	Ran GTPase-activating prot	RANGAP1
P43487-2;P43487;C9JJ3	P43487-2;F	3;3;2;2;1;1	3;3;2;2;1;1	3;3;2;2;1;1	Ran-specific GTPase-activat	RANBP1
Q13283;E5RIZ6;E5RI46;	Q13283	9;3;2;2;2;2	9;3;2;2;2;2	9;3;2;2;2;2	Ras GTPase-activating prote	G3BP1

P46940;A0A0J9YXZ5;H(	P46940;A0,	36;28;22;2;	36;28;22;2;	35;27;21;2;	Ras GTPase-activating-like p	IQGAP1
Q15404-2;Q15404	Q15404-2;(	2;2	2;2	2;2	Ras suppressor protein 1	RSU1
P63000;P63000-2;P607	P63000;P6	9;9;5	9;9;5	3;3;1	Ras-related C3 botulinum to	RAC1;RAC3
P15153;B1AH77;B1AH8	P15153;B1,	8;7;7;6;3;3,	2;2;2;1;0;0,	2;2;2;1;0;0,	Ras-related C3 botulinum to	RAC2
Q9HB90;Q9NQL2;Q9NC	Q9HB90;Q	2;2;1	2;2;1	2;2;1	Ras-related GTP-binding pro	RRAGC;RR/
P61026;P51153;A0A087	P61026	6;2;1	5;1;1	5;1;1	Ras-related protein Rab-10	RAB10
Q15907;Q15907-2;H3BI	Q15907;Q1	11;10;9;9;9	11;10;9;9;9	10;9;8;8;8;	Ras-related protein Rab-11B	RAB11B;RA
P61106;X6RFL8	P61106;X6I	13;10	13;10	13;10	Ras-related protein Rab-14	RAB14
Q9NP72;Q9NP72-2;Q5\	Q9NP72;Q	7;6;5;5;4;4	7;6;5;5;4;4	7;6;5;5;4;4	Ras-related protein Rab-18	RAB18
P62820;E7END7;P6282(	P62820;E7I	11;9;8;6;2;	11;9;8;6;2;	4;4;4;3;0;0	Ras-related protein Rab-1A	RAB1A
Q9H0U4;E9PLD0;Q9292	Q9H0U4;E9	11;10;5	4;4;2	3;3;1	Ras-related protein Rab-1B	RAB1B
Q9UL25	Q9UL25	4	4	4	Ras-related protein Rab-21	RAB21
P57735	P57735	2	1	1	Ras-related protein Rab-25	RAB25
H3BU81;H3BUD9;H3BS4	H3BU81;H3	1;1;1;1;1;1,	1;1;1;1;1;1,	1;1;1;1;1;1,	Ras-related protein Rab-27A	RAB27A
P61019;E9PKL7;P61019	P61019;E9I	7;5;5;5;2;2,	7;5;5;5;2;2,	7;5;5;5;2;2,	Ras-related protein Rab-2A;	RAB2A;RAE
Q13637;P57729;H0YEA4	Q13637	5;2;1	5;2;1	4;1;1	Ras-related protein Rab-32	RAB32
F5H157;Q15286;Q1528	F5H157;Q1	2;2;1	2;2;1	2;2;1	Ras-related protein Rab-35	RAB35
Q86YS6	Q86YS6	1	1	1	Ras-related protein Rab-43	RAB43
P51148;P51148-2;K7ER	P51148;P5	7;7;4;4;3;3,	7;7;4;4;3;3,	7;7;4;4;3;3,	Ras-related protein Rab-5C	RAB5C
P20340-2;P20340;F5H3	P20340-2;F	5;5;3;3;3;2,	5;5;3;3;3;2,	5;5;3;3;3;2,	Ras-related protein Rab-6A	RAB6A
P51149;C9J8S3;C9J4V0;	P51149;C9,	16;12;10;1(	16;12;10;1(	16;12;10;1(	Ras-related protein Rab-7a	RAB7A
O14966;O14966-2;E9PL	O14966;O1	3;2;1;1	2;2;1;1	2;2;1;1	Ras-related protein Rab-7L1	RAB29
P61006-2;P61006	P61006-2;F	3;3	1;1	1;1	Ras-related protein Rab-8A	RAB8A
P51151;A0A1B0GUI0;Q	P51151	3;1;1	3;1;1	3;1;1	Ras-related protein Rab-9A	RAB9A
H7C3P7;P11233	H7C3P7;P1	4;4	4;4	3;3	Ras-related protein Ral-A	RALA
P61224-3;P61224;E7ES\	P61224-3;F	6;6;5;5;5;4,	6;6;5;5;5;4,	2;2;2;2;1;1,	Ras-related protein Rap-1b;	RAP1B;RAP
P61225	P61225	4	4	2	Ras-related protein Rap-2b	RAP2B
P10301	P10301	3	1	1	Ras-related protein R-Ras	RRAS
P62070;P62070-4;E9PK	P62070;P6	4;4;3;3;2;1,	4;4;3;3;2;1,	2;2;2;2;2;0,	Ras-related protein R-Ras2	RRAS2
P60602-2;P60602	P60602-2;F	1;1	1;1	1;1	Reactive oxygen species mo	ROMO1
Q00765;B7Z332;B7Z51(	Q00765	3;1;1;1;1;1	3;1;1;1;1;1	3;1;1;1;1;1	Receptor expression-enhan	REEP5
Q96L35;P54760;P54753	Q96L35;P5	2;2;2	2;2;2	2;2;2	Receptor protein-tyrosine k	EPHB4;EPH
P10586-2;P10586;H0Y4	P10586-2;F	4;4;3;3;3;2,	4;4;3;3;3;2,	4;4;3;3;3;2,	Receptor-type tyrosine-prot	PTPRF
Q9BRX8-2;Q9BRX8	Q9BRX8-2;	4;4	4;4	4;4	Redox-regulatory protein F/	FAM213A
P18754;P18754-2;C9JW	P18754;P1	8;8;7;6;6;6,	8;8;7;6;6;6,	8;8;7;6;6;6,	Regulator of chromosome c	RCC1
Q96DB5-2;Q96DB5;H0Y	Q96DB5-2;	3;3;2;2;2;2,	3;3;2;2;2;2,	3;3;2;2;2;2,	Regulator of microtubule dy	RMDN1
H0YNE5;H0YLG5;Q96TC	H0YNE5;H(	1;1;1	1;1;1	1;1;1	Regulator of microtubule dy	RMDN3
Q92900-2;Q92900	Q92900-2;(	1;1	1;1	1;1	Regulator of nonsense trans	UPF1
Q8WUF5;K7EN03	Q8WUF5;K	2;1	2;1	2;1	RelA-associated inhibitor	PPP1R13L
P35250;P35250-2;H7C5	P35250;P3	3;2;1;1;1;1,	3;2;1;1;1;1,	3;2;1;1;1;1,	Replication factor C subunit	RFC2
P40938-2;P40938;A0A0	P40938-2;F	5;5;1	5;5;1	5;5;1	Replication factor C subunit	RFC3
C9JZI1;P35249-2;P3524	C9JZI1;P35	2;2;2;1;1;1,	2;2;2;1;1;1,	2;2;2;1;1;1,	Replication factor C subunit	RFC4
P40937-2;P40937;F5H3	P40937-2;F	2;2;1;1;1	2;2;1;1;1	2;2;1;1;1	Replication factor C subunit	RFC5
B5MC59;P35244	B5MC59;P3	3;3	3;3	3;3	Replication protein A 14 kD	RPA3
P15927;P15927-2;P159	P15927;P1	2;2;2;1;1	2;2;2;1;1	2;2;2;1;1	Replication protein A 32 kD	RPA2
P27694;I3L4R8	P27694;I3L	2;1	2;1	2;1	Replication protein A 70 kD	RPA1
Q15293;Q15293-2	Q15293;Q1	9;7	9;7	9;7	Reticulocalbin-1	RCN1
H0YL43;Q14257;Q1425	H0YL43;Q1	3;3;3	3;3;3	3;3;3	Reticulocalbin-2	RCN2
Q9NQC3-2;Q9NQC3-5;(	Q9NQC3-2;	7;7;7;6;5;4,	7;7;7;6;5;4,	7;7;7;6;5;4,	Reticulon-4;Reticulon	RTN4
P06400	P06400	1	1	1	Retinoblastoma-associated	RB1

Q8NFJ5;F5GWG3	Q8NFJ5;F5(	2;1	2;1	2;1	Retinoic acid-induced prote	GPRC5A
Q8IZV5;E5RK48	Q8IZV5	3;1	3;1	3;1	Retinol dehydrogenase 10	RDH10
Q8TC12;G3V2G6;Q8TC1	Q8TC12;G3	5;4;4;3;3;2	5;4;4;3;3;2	5;4;4;3;3;2	Retinol dehydrogenase 11	RDH11
Q9HBH5	Q9HBH5	2	2	2	Retinol dehydrogenase 14	RDH14
J3KRE2;J3KTF8;J3QQX2;	J3KRE2;J3K	3;3;3;3;2;2	3;3;3;3;2;2	3;3;3;3;2;2	Rho GDP-dissociation inhibi	ARHGDIA
F5H2R5;F5H6Q0;F5H3P	F5H2R5;F5	2;2;2;2;1	2;2;2;2;1	2;2;2;2;1	Rho GDP-dissociation inhibi	ARHGDIB
Q07960;H0YE29;E9PNR	Q07960;H0	5;4;1	5;4;1	5;4;1	Rho GTPase-activating prot	ARHGAP1
R4GNI4;R4GMS4;Q14CI	R4GNI4;R4	1;1;1;1;1;1	1;1;1;1;1;1	1;1;1;1;1;1	Rho GTPase-activating prot	ARHGAP19
Q5JR08;P08134;E9PQH	Q5JR08;P0	5;5;4;3;3;3	5;5;4;3;3;3	1;1;1;0;0;0	Rho-related GTP-binding pr	RHOC;RHO
P84095	P84095	6	5	5	Rho-related GTP-binding pr	RHOG
P13489;E9PIM9;E9PMJ	P13489;E9I	10;6;5;5;4;	10;6;5;5;4;	10;6;5;5;4;	Ribonuclease inhibitor	RNH1
P23921;E9PL69;E9PP77	P23921;E9I	3;2;1;1	3;2;1;1	3;2;1;1	Ribonucleoside-diphosphat	RRM1
C9JXC1;A0A286YFD6;P3	C9JXC1;A0	4;4;4;4;1;1	4;4;4;4;1;1	4;4;4;4;1;1	Ribonucleoside-diphosphat	RRM2
P60891;B1ALA9;P60891	P60891;B1	3;2;2;1	2;1;1;1	2;1;1;1	Ribose-phosphate pyropho	PRPS1
P11908;P11908-2;D3YT	P11908;P1	4;4;3;2;1;1	4;4;3;2;1;1	3;3;3;2;0;1	Ribose-phosphate pyropho	PRPS2
Q9Y4W2-4;Q9Y4W2-2;(	Q9Y4W2-4	1;1;1	1;1;1	1;1;1	Ribosomal biogenesis prote	LAS1L
O76021;J3QSV6;O7602	O76021;J3	16;15;8;6;6	16;15;8;6;6	16;15;8;6;6	Ribosomal L1 domain-conta	RSL1D1
J3QR09;J3KTE4;P84098	J3QR09;J3K	4;4;4;3	4;4;4;3	4;4;4;3	Ribosomal protein L19;60S	RPL19
P56182;D6RE82	P56182	5;2	5;2	5;2	Ribosomal RNA processing	RRP1
Q92979;A0A087WVM7	Q92979;A0	3;2;1;1	3;2;1;1	3;2;1;1	Ribosomal RNA small subur	EMG1
Q9Y3A4	Q9Y3A4	1	1	1	Ribosomal RNA-processing	RRP7A
Q14692	Q14692	5	5	5	Ribosome biogenesis protei	BMS1
Q14137;Q14137-2;A0A	Q14137;Q1	7;6;1	7;6;1	7;6;1	Ribosome biogenesis protei	BOP1
Q8TDN6	Q8TDN6	6	6	6	Ribosome biogenesis protei	BRIX1
A0A0A0MQZ6;O95478;	A0A0A0MC	3;3;1	3;3;1	3;3;1	Ribosome biogenesis protei	NSA2
Q9GZL7	Q9GZL7	4	4	4	Ribosome biogenesis protei	WDR12
Q15050	Q15050	2	2	2	Ribosome biogenesis regula	RRS1
F8WE72;A0A087X020;C	F8WE72;A0	2;2;2	2;2;2	2;2;2	Ribosome maturation prote	SBDS
Q5VXN0;Q9H7B2;U3KQ	Q5VXN0;Q	4;4;1;1	4;4;1;1	4;4;1;1	Ribosome production facto	RPF2
A0A0A0MRV0;Q9P2E9-	A0A0A0MF	17;17;17;1	17;17;17;1	17;17;17;1	Ribosome-binding protein 1	RRBP1
Q96E11-8;Q96E11-3;Q	Q96E11-8;(	5;5;5;3;2;2	5;5;5;3;2;2	5;5;5;3;2;2	Ribosome-recycling factor,	MRRF
D6RAL1;Q969S9-5;Q96	D6RAL1;Q	1;1;1;1;1;1	1;1;1;1;1;1	1;1;1;1;1;1	Ribosome-releasing factor 2	GFM2
P38159;P38159-2;H3BT	P38159;P3	11;10;8;7;7	11;10;8;7;7	11;10;8;7;7	RNA-binding motif protein,	RBMX;RBM
Q96PK6;Q96PK6-2	Q96PK6	10;3	10;3	8;1	RNA-binding protein 14	RBM14
Q96PK6-5;A0A0A6YYI9;	Q96PK6-5;	4;2;2;2;2;2	2;0;0;0;0;0	0;0;0;0;0;0	RNA-binding protein 14	RBM14;RBI
P49756;E9PQU5;P4975	P49756;E9I	3;2;2;2;1;1	3;2;2;2;1;1	3;2;2;2;1;1	RNA-binding protein 25	RBM25
U3KPZ7;Q9P2N5	U3KPZ7;Q	1;1	1;1	1;1	RNA-binding protein 27	RBM27
Q9NW13;Q9NW13-2;C	Q9NW13;C	5;4;2;2	5;4;2;2	5;4;2;2	RNA-binding protein 28	RBM28
Q5TCT4;A2A2V2;P4269	Q5TCT4;A2	2;2;2;2	2;2;2;2	2;2;2;2	RNA-binding protein 34	RBM34
A0A0U1RQH7;G3XAC6;	A0A0U1RQ	7;7;7;7;7;6	7;7;7;7;7;6	7;7;7;7;7;6	RNA-binding protein 39	RBM39
Q9Y5S9;Q9Y5S9-2;A0A	Q9Y5S9;Q	3;2;1	3;2;1	3;2;1	RNA-binding protein 8A	RBM8A
B0QYK0;Q01844-6;Q01	B0QYK0;Q	6;6;6;6;6;5	6;6;6;6;6;5	6;6;6;6;6;5	RNA-binding protein EWS	EWSR1
H3BPE7;P35637-2;P356	H3BPE7;P3	5;5;5	3;3;3	3;3;3	RNA-binding protein FUS	FUS
H3BUR4;Q9ULX3	H3BUR4;Q	1;1	1;1	1;1	RNA-binding protein NOB1	NOB1
Q9NRX1;F8WBJ6	Q9NRX1;F8	2;1	2;1	2;1	RNA-binding protein PNO1	PNO1
Q5QPL9;Q9UKM9-2;Q9	Q5QPL9;Q	5;5;5;4;3;2	5;5;5;4;3;2	5;5;5;4;3;2	RNA-binding protein Raly	RALY
H3BV80;H3BMS0;H3BM	H3BV80;H3	2;2;2;2;2;2	2;2;2;2;2;2	2;2;2;2;2;2	RNA-binding protein with s	RNPS1
Q5TZA2-2;Q5TZA2;B1AI	Q5TZA2-2;(	2;2;1	2;2;1	2;2;1	Rootletin	CROCC
M0R299;M0QXL5;P220	M0R299;M	5;5;5;4;2;2	5;5;5;4;2;2	5;5;5;4;2;2	rRNA 2-O-methyltransferas	FBL
Q6IN84	Q6IN84	1	1	1	rRNA methyltransferase 1,	MRM1

A6NHQ2	A6NHQ2	1	1	1	rRNA/tRNA 2-O-methyltran	FBLL1
G3V2M5;G3V5S9;G3V1	G3V2M5;G	1;1;1;1	1;1;1;1	1;1;1;1	rRNA-processing protein FC	FCF1
Q5JTH9-2;Q5JTH9-3;Q5	Q5JTH9-2;(	4;4;4;1	4;4;4;1	4;4;4;1	RRP12-like protein	RRP12
Q9Y3B9	Q9Y3B9	3	3	3	RRP15-like protein	RRP15
Q9Y265;Q9Y265-2;E7ET	Q9Y265;Q9	12;9;7;6;3;	12;9;7;6;3;	12;9;7;6;3;	RuvB-like 1	RUVBL1
Q9Y230-2;Q9Y230;M0R	Q9Y230-2;(	13;13;9;4;3	13;13;9;4;3	13;13;9;4;3	RuvB-like 2	RUVBL2
P31153;P31153-2	P31153;P3:	5;4	5;4	5;4	S-adenosylmethionine syntl	MAT2A
F8VZQ9;H0YHG0;P8297	F8VZQ9;H0	2;2;2	2;2;2	2;2;2	SAP domain-containing ribo	SARNP
P16615;P16615-5;P166	P16615;P1(	22;21;21;2	22;21;21;2	22;21;21;2	Sarcoplasmic/endoplasmic	ATP2A2
Q15424-2;Q15424;Q15	Q15424-2;(	9;9;9;9;1	9;9;9;9;1	4;4;4;4;1	Scaffold attachment factor	SAFB
Q14151;Q14151-2	Q14151	8;1	3;1	3;1	Scaffold attachment factor	SAFB2
B7ZKQ9;F5H4X0;Q8WT	B7ZKQ9;F5	1;1;1;1;1;1	1;1;1;1;1;1	1;1;1;1;1;1	Scavenger receptor class B	SCARB1
Q08AF3	Q08AF3	1	1	1	Schlafen family member 5	SLFN5
Q8WVM8;Q8WVM8-2;(	Q8WVM8;(	4;3;3;1;1;1	4;3;3;1;1;1	4;3;3;1;1;1	Sec1 family domain-contain	SCFD1
Q12765;Q12765-2;Q12	Q12765;Q1	3;3;2;1;1;1	3;3;2;1;1;1	3;3;2;1;1;1	Secernin-1	SCRN1
A0A087WZA6;O15126-:	A0A087WZ	1;1;1	1;1;1	1;1;1	Secretory carrier-associatec	SCAMP1
O15127	O15127	1	1	1	Secretory carrier-associatec	SCAMP2
O14828;O14828-2	O14828;O1	4;3	4;3	4;3	Secretory carrier-associatec	SCAMP3
Q969E2-2;Q969E2	Q969E2-2;(	1;1	1;1	1;1	Secretory carrier-associatec	SCAMP4
P35270	P35270	1	1	1	Sepiapterin reductase	SPR
D6RDP1;D6R9Y6;D6RDI	D6RDP1;D6	1;1;1;1;1;1	1;1;1;1;1;1	1;1;1;1;1;1	Septin-6;Septin-11	SEPT11;SEF
Q13501;Q13501-2;E7EN	Q13501;Q1	14;11;10;6;	14;11;10;6;	14;11;10;6;	Sequestosome-1	SQSTM1
P83111-2;P83111	P83111-2;F	2;2	2;2	2;2	Serine beta-lactamase-like	LACTB
P34897-3;P34897;P348	P34897-3;F	15;15;14;8;	15;15;14;8;	14;14;13;7;	Serine hydroxymethyltransf	SHMT2
Q9NRX5	Q9NRX5	4	4	4	Serine incorporator 1	SERINC1
O15269;O15269-2	O15269	7;3	7;3	7;3	Serine palmitoyltransferase	SPTLC1
O15270;H0YJV2	O15270;H0	2;1	2;1	2;1	Serine palmitoyltransferase	SPTLC2
M0R088;E9PCT1;A9Z1X	M0R088;E9	1;1;1;1;1	1;1;1;1;1	1;1;1;1;1	Serine/arginine repetitive m	SRRM1
Q9UQ35;Q9UQ35-2;I3L	Q9UQ35	13;4;2;2;2;	13;4;2;2;2;	13;4;2;2;2;	Serine/arginine repetitive m	SRRM2
J3KTL2;Q07955;Q07955	J3KTL2;Q07	7;7;6;6;4;1	7;7;6;6;4;1	7;7;6;6;4;1	Serine/arginine-rich splicing	SRSF1
Q5JRI1;O75494-5;O754	Q5JRI1;O75	2;2;2;2;2;2	2;2;2;2;2;2	2;2;2;2;2;2	Serine/arginine-rich splicing	SRSF10;SRS
Q5T760;Q05519-2;Q05	Q5T760;Q0	1;1;1	1;1;1	1;1;1	Serine/arginine-rich splicing	SRSF11
J3KP15;Q01130;J3QL05	J3KP15;Q0	5;5;4;4;2;2	5;5;4;4;2;2	5;5;4;4;2;2	Serine/arginine-rich splicing	SRSF2
A0A087X2D0;P84103-2	A0A087X2D	4;4;4	3;3;3	3;3;3	Serine/arginine-rich splicing	SRSF3
Q13243-3;Q13243;B4D	Q13243-3;(	3;3;2;2;1;1	2;2;1;1;1;1	2;2;1;1;1;1	Serine/arginine-rich splicing	SRSF5
Q13247-3;Q13247;A0A(	Q13247-3;(	5;5;3;3;1	5;5;3;3;1	4;4;2;2;1	Serine/arginine-rich splicing	SRSF6;SRSF
A0A0B4J1Z1;C9JAB2;Q1	A0A0B4J1Z	6;6;6;6;6;6	6;6;6;6;6;6	5;5;5;5;5;5	Serine/arginine-rich splicing	SRSF7
Q13242;H0YIB4;S4R3G(	Q13242;H0	7;5;3	7;5;3	7;5;3	Serine/arginine-rich splicing	SRSF9
H0Y630;B4DR80;Q9Y6E	H0Y630;B4	2;2;2;2;1;1	2;2;2;2;1;1	1;1;1;1;1;1	Serine/threonine-protein ki	STK24
E7ETY4;E9PC69;Q7KZI7	E7ETY4;E9F	2;2;2;2;2;2	2;2;2;2;2;2	2;2;2;2;2;2	Serine/threonine-protein ki	MARK2
C9JIG9;O95747	C9JIG9;O95	1;1	1;1	1;1	Serine/threonine-protein ki	OXSR1
Q13177;B1AKS5;H7C1X	Q13177	5;1;1;1;1;1	5;1;1;1;1;1	5;1;1;1;1;1	Serine/threonine-protein ki	PAK2
H0YJF7;Q99986	H0YJF7;Q9	1;1	1;1	1;1	Serine/threonine-protein ki	VRK1
P63151;P63151-2;E5RF	P63151;P6	2;2;1;1;1;1	2;2;1;1;1;1	2;2;1;1;1;1	Serine/threonine-protein pl	PPP2R2A;P
P30153;C9J9C1;B3KQV6	P30153;C9	11;7;7;3;3;	11;7;7;3;3;	11;7;7;3;3;	Serine/threonine-protein pl	PPP2R1A
A6PVN9;F6WIT2;A6PVN	A6PVN9;F6	3;3;3;3;3;3	3;3;3;3;3;3	3;3;3;3;3;3	Serine/threonine-protein pl	PPP2R4
P67775;P67775-2;P627	P67775;P6	7;6;6;3;3;2	7;6;6;3;3;2	6;5;5;3;2;2	Serine/threonine-protein pl	PPP2CA;PP
Q96HS1-2;Q96HS1;F5G	Q96HS1-2;	5;5;1	5;5;1	5;5;1	Serine/threonine-protein pl	PGAM5
P62136-3;P62136;P621	P62136-3;F	8;8;8;5	2;2;2;1	2;2;2;1	Serine/threonine-protein pl	PPP1CA
P62140;E7ETD8;C9JP48	P62140;E7I	9;5;4;3;1;1	9;5;4;3;1;1	3;1;2;2;0;0	Serine/threonine-protein pl	PPP1CB

E7ETC2;Q08209-5;Q082	E7ETC2;Q0	2;2;2;2;2;1	2;2;2;2;2;1	2;2;2;2;2;1	Serine/threonine-protein pl	PPP3CA;PP
H3BV22;H3BTA2;P6051	H3BV22;H3	4;4;4;2;1;1	3;3;3;2;1;1	3;3;3;2;1;1	Serine/threonine-protein pl	PPP4C
F8VYE8;P36873;P36873	F8VYE8;P3	8;8;8;7;7;4	2;2;2;2;1;2	2;2;2;2;1;2	Serine/threonine-protein pl	PPP1CC
Q9Y3F4;Q9Y3F4-2;H0YH	Q9Y3F4;Q9	5;5;2	5;5;2	5;5;2	Serine-threonine kinase rec	STRAP
Q5T5C7;P49591	Q5T5C7;P4	4;4	4;4	4;4	Serine--tRNA ligase, cytopla	SARS
M0QWZ7;M0R2C6;Q9N	M0QWZ7;M	2;2;2;2;1;1	2;2;2;2;1;1	2;2;2;2;1;1	Serine--tRNA ligase, mitoch	SARS2
F8WE70;A0A0A0MQW3	F8WE70;A0	4;4;4;4;2	4;4;4;4;2	4;4;4;4;2	Serpin B13	SERPINB13
P36952;P36952-2;C9JLN	P36952;P3	14;8;6	14;8;6	14;8;6	Serpin B5	SERPINB5
A0A024QZX5;A0A087X1	A0A024QZ	6;6;6;1;1;1	6;6;6;1;1;1	6;6;6;1;1;1	Serpin B6	SERPINB6
P50454;E9PR70;E9PPV6	P50454;E9	13;10;10;8	13;10;10;8	13;10;10;8	Serpin H1	SERPINH1
Q9BXP5-5;Q9BXP5-4;Q9	Q9BXP5-5;	3;3;3;3;3;2	3;3;3;3;3;2	3;3;3;3;3;2	Serrate RNA effector molec	SRRT
J3QT77;A0A0J9YYG4;A0	J3QT77;A0	1;1;1;1;1;1	1;1;1;1;1;1	1;1;1;1;1;1	Serum paraoxonase/arylest	PON2
U3KQT1;H7BZT7;X6RA1	U3KQT1;H7	1;1;1;1	1;1;1;1	1;1;1;1	S-formylglutathione hydrola	ESD
Q5T123;Q9H299	Q5T123;Q9	2;2	2;2	2;2	SH3 domain-binding glutam	SH3BGRL3
Q9NR45	Q9NR45	2	2	2	Sialic acid synthase	NANS
A0A0A0MS41;A0A1P0A	A0A0A0MS	6;6;6;5	6;6;6;5	6;6;6;5	Sideroflexin;Sideroflexin-3	SFXN3
Q9H9B4;D6RDG7;S4R2X	Q9H9B4;D6	6;3;3;3;1	6;3;3;3;1	6;3;3;3;1	Sideroflexin-1	SFXN1
H0YNG3;P67812-4;P678	H0YNG3;P6	2;2;2;2;1;1	2;2;2;2;1;1	2;2;2;2;1;1	Signal peptidase complex ca	SEC11A
Q9Y6A9;C9JBL1;X6R2S6	Q9Y6A9;C9	3;2;2	3;2;2	3;2;2	Signal peptidase complex su	SPCS1
E9PL01;A0A087WUC6;E	E9PL01;A0	4;4;4;4;3	4;4;4;4;3	4;4;4;4;3	Signal peptidase complex su	SPCS2
P61009	P61009	3	3	3	Signal peptidase complex su	SPCS3
H0YLA2;P37108	H0YLA2;P3	4;4	4;4	4;4	Signal recognition particle 1	SRP14
A0A087WYR0;P09132;A	A0A087WY	2;2;1	2;2;1	2;2;1	Signal recognition particle 1	SRP19
P49458;E9PE20;P49458	P49458;E9	4;3;3	4;3;3	4;3;3	Signal recognition particle 9	SRP9
Q9Y5M8;H7C4H2	Q9Y5M8;H	6;5	6;5	6;5	Signal recognition particle r	SRPRB
Q9UHB9-4;Q9UHB9-2;C	Q9UHB9-4;	3;3;3;2	3;3;3;2	3;3;3;2	Signal recognition particle s	SRP68
O76094;D6RDY6;O7609	O76094;D6	3;2;2	3;2;2	3;2;2	Signal recognition particle s	SRP72
J3KPM9;P42224-2;P422	J3KPM9;P4	5;5;5;2;2;1	5;5;5;2;2;1	5;5;5;2;2;1	Signal transducer and activa	STAT1
Q92783-2;Q92783	Q92783-2;	1;1	1;1	1;1	Signal transducing adapter r	STAM
O75886-2;O75886	O75886-2;	1;1	1;1	1;1	Signal transducing adapter r	STAM2
Q04837;A0A0G2JLD8;E	Q04837;A0	4;3;3;2	4;3;3;2	4;3;3;2	Single-stranded DNA-bindin	SSBP1
G3V1B8;H0YEB6;O6023	G3V1B8;H0	1;1;1	1;1;1	1;1;1	Sjoegren syndrome/sclerod	SSSCA1
K7EMD6;O43765	K7EMD6;O	2;2	2;2	2;2	Small glutamine-rich tetratr	SGTA
P62306	P62306	2	2	2	Small nuclear ribonucleopro	SNRPF
P62314;J3QLR7;J3QLI9	P62314;J3Q	2;1;1	2;1;1	2;1;1	Small nuclear ribonucleopro	SNRPD1
P62316-2;P62316;K7ER	P62316-2;P	3;3;2	3;3;2	3;3;2	Small nuclear ribonucleopro	SNRPD2
P62318-2;P62318;H3BT	P62318-2;P	4;4;2	4;4;2	4;4;2	Small nuclear ribonucleopro	SNRPD3
J3QLE5;P14678-2;P6316	J3QLE5;P14	5;5;5;5;5;5	5;5;5;5;5;5	5;5;5;5;5;5	Small nuclear ribonucleopro	SNRPN;SNF
O75691	O75691	3	3	3	Small subunit processome c	UTP20
B8ZZN6;P63165;B8ZZJ0	B8ZZN6;P6	3;3;2;2;1;1	3;3;2;2;1;1	3;3;2;2;1;1	Small ubiquitin-related moc	SUMO1
P61956-2;P61956;A8MU	P61956-2;P	2;2;1;1;1;1	2;2;1;1;1;1	2;2;1;1;1;1	Small ubiquitin-related moc	SUMO2;SU
G3V5R3;G3V3A4;Q1357	G3V5R3;G3	1;1;1	1;1;1	1;1;1	SNW domain-containing pro	SNW1
P05023-4;P05023;P050	P05023-4;P	28;28;25;19	28;28;25;19	27;27;24;19	Sodium/potassium-transpo	ATP1A1
P05026-2;P05026;V9GY	P05026-2;P	4;4;3	4;4;3	4;4;3	Sodium/potassium-transpo	ATP1B1
P54709;C9JXZ1;C9JA36;	P54709;C9	3;2;2;2;1	3;2;2;2;1	3;2;2;2;1	Sodium/potassium-transpo	ATP1B3
F8VX04;Q9H2H9	F8VX04;Q9	2;2	2;2	2;2	Sodium-coupled neutral am	SLC38A1
Q96QD8;Q96QD8-2;F8V	Q96QD8;Q	3;2;1;1	3;2;1;1	3;2;1;1	Sodium-coupled neutral am	SLC38A2
Q8WUX1;C9JJU1;C9JMY	Q8WUX1;C	4;2;2;2;2;2	4;2;2;2;2;2	4;2;2;2;2;2	Sodium-coupled neutral am	SLC38A5
H7BZK4;Q8WUM9	H7BZK4;Q8	1;1	1;1	1;1	Sodium-dependent phospha	SLC20A1
G3XAL9;P55011-3;P550	G3XAL9;P5	2;2;2	2;2;2	2;2;2	Solute carrier family 12 mer	SLC12A2

P11166	P11166	3	3	3	Solute carrier family 2, facili	SLC2A1
Q9NQZ2	Q9NQZ2	1	1	1	Something about silencing p	UTP3
Q00796;H0YLA4;H0YKB	Q00796;H0	8;6;3;3	8;6;3;3	8;6;3;3	Sorbitol dehydrogenase	SORD
C9J0K6;P30626-3;P3062	C9J0K6;P30	5;5;5;5;3	5;5;5;5;3	5;5;5;5;3	Sorcin	SRI
Q9Y512	Q9Y512	2	2	2	Sorting and assembly machi	SAMM50
Q9UMY4;A0A087X0R6;(	Q9UMY4;A	2;1;1;1	2;1;1;1	2;1;1;1	Sorting nexin-12	SNX12
O60493-2;O60493;O604	O60493-2;(	2;2;1;1	2;2;1;1	2;2;1;1	Sorting nexin-3	SNX3
Q9NUQ6-2;Q9NUQ6;B8	Q9NUQ6-2	10;10;9;9;9	10;10;9;9;9	10;10;9;9;9	SPATS2-like protein	SPATS2L
Q9BW04-2;Q9BW04	Q9BW04-2	2;2	2;2	2;2	Specifically androgen-regula	SARG
A0A0D9SF54;A0A0D9SC	A0A0D9SF5	29;29;29;2!	29;29;29;2!	29;29;29;2!	Spectrin alpha chain, non-er	SPTAN1
A0A087WUZ3;Q01082;(	A0A087WU	17;17;14;1	17;17;14;1	17;17;14;1	Spectrin beta chain, non-ery	SPTBN1
E5RJR5;P63208;E5RGM	E5RJR5;P63	3;3;1;1;1;1	3;3;1;1;1;1	3;3;1;1;1;1	S-phase kinase-associated p	SKP1
H7BXF4;Q9NXE4-3;Q9N	H7BXF4;Q9	2;2;2;2;2;1	2;2;2;2;2;1	2;2;2;2;2;1	Sphingomyelin phosphodies	SMPD4
H0Y3V8;H7BXL7;O9547	H0Y3V8;H7	2;2;2	2;2;2	2;2;2	Sphingosine-1-phosphate ly	SGPL1
Q13838;Q13838-2;A0A(	Q13838;Q1	9;9;7;7;5;5	9;9;7;7;5;5	2;2;2;2;1;1	Spliceosome RNA helicase D	DDX39B
Q15637-4;Q15637-6;Q1	Q15637-4;(	4;4;4;4;4;4	4;4;4;4;4;4	4;4;4;4;4;4	Splicing factor 1	SF1
Q15459;Q15459-2;H7C	Q15459;Q1	3;2;1	3;2;1	3;2;1	Splicing factor 3A subunit 1	SF3A1
Q12874	Q12874	4	4	4	Splicing factor 3A subunit 3	SF3A3
O75533;F8WC19;B4DG	O75533	9;1;1;1;1	9;1;1;1;1	9;1;1;1;1	Splicing factor 3B subunit 1	SF3B1
Q13435;E9PJT3;E9PJ04;	Q13435;E9	6;5;5;5;4;1	6;5;5;5;4;1	6;5;5;5;4;1	Splicing factor 3B subunit 2	SF3B2
Q15393;Q15393-3;Q15	Q15393	10;3;2;1;1;	10;3;2;1;1;	10;3;2;1;1;	Splicing factor 3B subunit 3	SF3B3
Q9Y3B4	Q9Y3B4	2	2	2	Splicing factor 3B subunit 6	SF3B6
P0DN76;Q01081;Q0108	P0DN76;Q(	4;4;3;2;2;2	4;4;3;2;2;2	4;4;3;2;2;2	Splicing factor U2AF 35 kDa	U2AF1;U2A
K7ENG2;P26368-2;P263	K7ENG2;P2	6;6;6	6;6;6	6;6;6	Splicing factor U2AF 65 kDa	U2AF2
P23246;P23246-2;H0Y9	P23246;P2	17;15;4;1	17;15;4;1	16;14;4;1	Splicing factor, proline- and	SFPQ
Q8WW59	Q8WW59	3	3	3	SPRY domain-containing pro	SPRYD4
E9PNM1;P37268;A0A1\	E9PNM1;P	11;11;10;1	11;11;10;1	11;11;10;1	Squalene synthase	FDFT1
F8VV04;Q15020-4;Q15(	F8VV04;Q1	1;1;1	1;1;1	1;1;1	Squamous cell carcinoma ar	SART3
A0A087WUN7;H0YJ40;(	A0A087WU	3;3;3;3;3;3	3;3;3;3;3;3	3;3;3;3;3;3	SRA stem-loop-interacting R	SLIRP
Q14247;Q14247-3;Q14	Q14247;Q1	17;16;15;5;	17;16;15;5;	17;16;15;5;	Src substrate cortactin	CTTN
Q7KZF4;H7C597	Q7KZF4	16;5	16;5	16;5	Staphylococcal nuclease do	SND1
P16949;A2A2D0;P1694!	P16949;A2	5;4;4	5;4;4	5;4;4	Stathmin	STMN1
E5RGX5;Q93045;Q9304	E5RGX5;Q9	1;1;1	1;1;1	1;1;1	Stathmin;Stathmin-2	STMN2
Q5K651;C9JKF1	Q5K651;C9	2;1	2;1	2;1	Sterile alpha motif domain-	SAMD9
A0A087X1T2;G3V0I8;Q	A0A087X1T	1;1;1;1	1;1;1;1	1;1;1;1	Sterol regulatory element-b	SREBF2
Q15738;C9JDR0	Q15738;C9	6;5	6;5	6;5	Sterol-4-alpha-carboxylate 3	NSDHL
A0A087WYB4;Q9UJZ1;(	A0A087WY	8;8;7;3	8;8;7;3	8;8;7;3	Stomatin-like protein 2, mit	STOML2
Q8WXE9;Q8WXE9-3	Q8WXE9;Q	1;1	1;1	1;1	Stonin-2	STON2
P38646;D6RJI2;H0YBG6	P38646	33;6;5;3;3	33;6;5;3;3	32;6;5;3;3	Stress-70 protein, mitochon	HSPA9
P31948;P31948-2;P319	P31948;P3	16;16;14;7;	16;16;14;7;	16;16;14;7;	Stress-induced-phosphopro	STIP1
Q9HCN8	Q9HCN8	2	2	2	Stromal cell-derived factor 2	SDF2L1
H0YDB2;E9PNJ4;G0XQ3	H0YDB2;E9	1;1;1;1;1	1;1;1;1;1	1;1;1;1;1	Stromal interaction molecul	STIM1
O95347;O95347-2;Q5T	O95347;O9	6;5;1;1	6;5;1;1	6;5;1;1	Structural maintenance of c	SMC2
Q9UQE7	Q9UQE7	5	5	5	Structural maintenance of c	SMC3
G8JLG1;Q14683;H0Y7K	G8JLG1;Q1	4;4;1;1	4;4;1;1	4;4;1;1	Structural maintenance of c	SMC1A
E9PD53;Q9NTJ3-2;Q9N	E9PD53;Q9	6;6;6;3;2;2	6;6;6;3;2;2	6;6;6;3;2;2	Structural maintenance of c	SMC4
P31040-2;P31040;D6RF	P31040-2;F	8;8;7;6;6;1	8;8;7;6;6;1	8;8;7;6;6;1	Succinate dehydrogenase [u	SDHA
P53597;H7C233	P53597	4;1	4;1	4;1	Succinyl-CoA ligase [ADP/GI	SUCLG1
Q9P2R7-2;Q9P2R7;A0A	Q9P2R7-2;(	5;5;3;3;1;1	5;5;3;3;1;1	5;5;3;3;1;1	Succinyl-CoA ligase [ADP-fo	SUCLA2
Q96I99;H0Y852;E9PDQ	Q96I99;H0	4;2;2;2	4;2;2;2	4;2;2;2	Succinyl-CoA ligase [GDP-fo	SUCLG2

P55809;E9PDW2;P5580	P55809;E9I	6;4;2	6;4;2	6;4;2	Succinyl-CoA:3-ketoacid co	OXCT1
A8MXB9;J3KQJ1;Q8NBJ	A8MXB9;J3	3;3;3;2;2;2	3;3;3;2;2;2	3;3;3;2;2;2	Sulfatase-modifying factor	SUMF2
Q9Y6N5;H3BNX3;H3BM	Q9Y6N5;H3	12;7;6;4;4;	12;7;6;4;4;	12;7;6;4;4;	Sulfide:quinone oxidoreduc	SQRDL
M0QX65;M0QZS6;B3KN	M0QX65;M	1;1;1;1;1;1	1;1;1;1;1;1	1;1;1;1;1;1	SUMO-activating enzyme su	SAE1
H3BPC4;H3BQQ9;B0QY	H3BPC4;H3	3;3;3;3	3;3;3;3	3;3;3;3	SUMO-conjugating enzyme	UBE2I
H7C2K3;H0Y6N5;H0Y74	H7C2K3;H0	1;1;1;1;1;1	1;1;1;1;1;1	1;1;1;1;1;1	SUN domain-containing pro	SUN1
P42285;H0Y8U3	P42285;H0	2;1	2;1	2;1	Superkiller viralicidic activit	SKIV2L2
P00441;H7BYH4	P00441;H7	3;2	3;2	3;2	Superoxide dismutase [Cu-Z	SOD1
P04179-4;P04179;F5H3	P04179-4;F	5;5;4;4;4;4	5;5;4;4;4;4	5;5;4;4;4;4	Superoxide dismutase [Mn]	SOD2
Q9Y2Z0-2;Q9Y2Z0	Q9Y2Z0-2;(	4;4	4;4	4;4	Suppressor of G2 allele of S	SUGT1
Q9BRV8;Q9BRV8-2	Q9BRV8;Q	1;1	1;1	1;1	Suppressor of IKBKE 1	SIKE1
A8MV53;A0A0A6YYI3;A	A8MV53;A(	4;4;4;4;4;4	4;4;4;4;4;4	4;4;4;4;4;4	Suppressor of SWI4 1 homo	PPAN;PPAN
Q15526-2;Q15526	Q15526-2;(	1;1	1;1	1;1	Surfeit locus protein 1	SURF1
Q5T8U5;O15260-2;O15	Q5T8U5;O	2;2;2;1;1	2;2;2;1;1	2;2;2;1;1	Surfeit locus protein 4	SURF4
O75683	O75683	3	3	3	Surfeit locus protein 6	SURF6
O75940	O75940	2	2	2	Survival of motor neuron-re	SMNDC1
F8VXC8;Q8TAQ2-2;Q8T	F8VXC8;Q8	3;3;3;3;1	3;3;3;3;1	3;3;3;3;1	SWI/SNF complex subunit S	SMARCC2
O60264	O60264	9	9	8	SWI/SNF-related matrix-ass	SMARCA5
J3KMX2;B9EGA3;Q9292	J3KMX2;B9	2;2;2;2;2;1	2;2;2;2;2;1	2;2;2;2;2;1	SWI/SNF-related matrix-ass	SMARCD2
K7EMQ8;J3QKS7;B4DGI	K7EMQ8;J3	2;2;2;2;2;2	2;2;2;2;2;2	2;2;2;2;2;2	SWI/SNF-related matrix-ass	SMARCE1
E7EMB1;Q9UH65	E7EMB1;Q	2;2	2;2	2;2	Switch-associated protein 7	SWAP70
Q99536;K7ERT7;Q9953	Q99536;K7	9;6;6;3;2;2	9;6;6;3;2;2	9;6;6;3;2;2	Synaptic vesicle membrane	VAT1
O15498-2;O15498;H7C	O15498-2;(	2;2;1	2;2;1	2;2;1	Synaptobrevin homolog YK	YKT6
Q16563-2;Q16563	Q16563-2;(	1;1	1;1	1;1	Synaptophysin-like protein	SYPL1
Q9H987-2;Q9H987	Q9H987-2;	1;1	1;1	1;1	Synaptopodin 2-like protein	SYNPO2L
O95721	O95721	1	1	1	Synaptosomal-associated p	SNAP29
H3BV99;H3BP15;H3BQ	H3BV99;H3	2;2;2;2;2;1	2;2;2;2;2;1	2;2;2;2;2;1	Synaptosomal-associated p	SNAP23
P18827;E9PHH3;H7C1K	P18827;E9I	2;1;1	2;1;1	2;1;1	Syndecan-1	SDC1
P31431-2;P31431	P31431-2;F	2;2	2;2	2;2	Syndecan-4	SDC4
O43752	O43752	2	2	2	Syntaxin-6	STX6
O15400-2;O15400	O15400-2;(	2;2	2;2	2;2	Syntaxin-7	STX7
O00560;O00560-3;B4DI	O00560;O(	10;8;7;7	10;8;7;7	1;1;0;0	Syntenin-1	SDCBP
O00560-2;G5EA09	O00560-2;(	10;9	1;1	1;1	Syntenin-1	SDCBP
Q9Y490;H0YMT1;A0A1	Q9Y490	10;1;1;1	10;1;1;1	10;1;1;1	Talin-1	TLN1
A0A0G2JKZ1;A2AB90;A	A0A0G2JKZ	2;2;2;2;2;2	2;2;2;2;2;2	2;2;2;2;2;2	Tapasin	TAPBP
Q13148;A0A087WX29;	Q13148;A0	6;5;5;5;5;5	6;5;5;5;5;5	6;5;5;5;5;5	TAR DNA-binding protein 4	TARDBP
Q92804-2;Q92804;A0A	Q92804-2;(	6;6;5;3;1	6;6;5;3;1	4;4;3;3;1	TATA-binding protein-assoc	TAF15
B8ZZD4;Q86VP1-3;Q86	B8ZZD4;Q8	6;6;6;6;6;3	6;6;6;6;6;3	6;6;6;6;6;3	Tax1-binding protein 1	TAX1BP1
O14907	O14907	1	1	1	Tax1-binding protein 3	TAX1BP3
Q8TC07-2;Q8TC07-3;Q	Q8TC07-2;(	3;3;3	3;3;3	3;3;3	TBC1 domain family membe	TBC1D15
Q9BYX2-6;Q9BYX2-5;Q	Q9BYX2-6;(	1;1;1;1;1;1	1;1;1;1;1;1	1;1;1;1;1;1	TBC1 domain family membe	TBC1D2
P17987;E7EQR6;F5H28	P17987;E7I	17;12;11;1	17;12;11;1	17;12;11;1	T-complex protein 1 subuni	TCP1
P78371;F5GWF6;P7837	P78371;F5	17;16;16;1	17;16;16;1	17;16;16;1	T-complex protein 1 subuni	CCT2
P50991-2;P50991	P50991-2;F	15;15	15;15	15;15	T-complex protein 1 subuni	CCT4
E7ENZ3;P48643;B7ZAR	E7ENZ3;P4	11;11;10;1	11;11;10;1	11;11;10;1	T-complex protein 1 subuni	CCT5
Q99832-3;Q99832;Q99	Q99832-3;(	10;10;9;8;1	10;10;9;8;1	10;10;9;8;1	T-complex protein 1 subuni	CCT7
P49368;B4DUR8;P4936	P49368;B4I	15;14;14;5;	15;14;14;5;	15;14;14;5;	T-complex protein 1 subuni	CCT3
P50990;P50990-2;P509	P50990;P5	20;19;16;8;	20;19;16;8;	20;19;16;8;	T-complex protein 1 subuni	CCT8
P40227;P40227-2;Q925	P40227;P4	10;9;2;2;1;	10;9;2;2;1;	10;9;2;2;1;	T-complex protein 1 subuni	CCT6A
Q5UIP0-2;Q5UIP0;H7C2	Q5UIP0-2;(	3;3;1;1	3;3;1;1	3;3;1;1	Telomere-associated protei	RIF1

Q68CZ2-2;Q68CZ2;E9P(	Q68CZ2-2;(	2;2;1	2;2;1	2;2;1	Tensin-3	TNS3
Q9UGI8-2;Q9UGI8;F8W	Q9UGI8-2;(	2;2;1;1	2;2;1;1	2;2;1;1	Testin	TES
E9PRJ3;E9PMR4;P4850(	E9PRJ3;E9F	3;3;3	3;3;3	3;3;3	Tetraspanin;CD151 antigen	CD151
F8VV56;F8W022;F8VWI	F8VV56;F8'	3;3;3;3;3;3	3;3;3;3;3;3	3;3;3;3;3;3	Tetraspanin;CD63 antigen	CD63
E9PJK1;E9PRJ8;H0YDL9;	E9PJK1;E9F	3;3;3;3;3;3	3;3;3;3;3;3	3;3;3;3;3;3	Tetraspanin;CD81 antigen	CD81
A6NNI4;G8JLH6;P21926	A6NNI4;G8	2;2;2	2;2;2	2;2;2	Tetraspanin;CD9 antigen	CD9
O60635	O60635	1	1	1	Tetraspanin-1	TSPAN1
O95857	O95857	1	1	1	Tetraspanin-13	TSPAN13
P52888;K7EKB6;K7EP46	P52888;K7I	2;1;1	2;1;1	2;1;1	Thimet oligopeptidase	THOP1
P10599;P10599-2	P10599;P1(	5;4	5;4	5;4	Thioredoxin	TXN
O95881	O95881	2	2	2	Thioredoxin domain-contair	TXNDC12
Q9BRA2;I3L0K2;I3L3M7	Q9BRA2;I3	3;2;2;1	3;2;2;1	3;2;2;1	Thioredoxin domain-contair	TXNDC17
Q8NBS9;Q8NBS9-2	Q8NBS9;Q8	8;7	8;7	8;7	Thioredoxin domain-contair	TXNDC5
E9PIR7;F8W809;A0A08	E9PIR7;F8V	6;6;6;6;6;6	6;6;6;6;6;6	5;5;5;5;5;5	Thioredoxin reductase 1, cy	TXNRD1
P30048-2;P30048	P30048-2;F	7;7	7;7	7;7	Thioredoxin-dependent per	PRDX3
O43396;K7ER96;K7EML	O43396;K7	6;5;2;1;1	6;5;2;1;1	6;5;2;1;1	Thioredoxin-like protein 1	TXNL1
Q9H3N1;G3V448	Q9H3N1;G	4;2	4;2	4;2	Thioredoxin-related transm	TMX1
Q9Y320-2;Q9Y320;E9PS	Q9Y320-2;(	7;7;2;2;2;2	7;7;2;2;2;2	7;7;2;2;2;2	Thioredoxin-related transm	TMX2
Q16762;B1AH48	Q16762;B1	2;1	2;1	2;1	Thiosulfate sulfurtransferas	TST
A0A0C4DG98;Q8NI27	A0A0C4DG	1;1	1;1	1;1	THO complex subunit 2	THOC2
Q86V81;E9PB61	Q86V81;E9	6;5	6;5	6;5	THO complex subunit 4	ALYREF
Q6I9Y2	Q6I9Y2	1	1	1	THO complex subunit 7 hon	THOC7
P26639;P26639-2;D6RC	P26639;P2(	17;17;3;3;3	17;17;3;3;3	17;17;3;3;3	Threonine--tRNA ligase, cyt	TARS
P07996;P07996-2;CON_	P07996;P0	11;9;8;2	11;9;8;2	11;9;8;2	Thrombospondin-1	THBS1
H3BNW0;Q9NXG2	H3BNW0;C	1;1	1;1	1;1	THUMP domain-containing	THUMPD1
K7ES52;K7ERJ1;K7ERV3	K7ES52;K7I	1;1;1;1	1;1;1;1	1;1;1;1	Thymidine kinase;Thymidin	TK1
P62328	P62328	2	2	2	Thymosin beta-4;Hematopc	TMSB4X
Q9Y2W1	Q9Y2W1	11	11	11	Thyroid hormone receptor-	THRAP3
Q15654;H7BZE2	Q15654	12;5	12;5	12;5	Thyroid receptor-interactin	TRIP6
A0A1B0GTW1;Q9UDY2-	A0A1B0GT	10;10;10;1(	10;10;10;1(	10;10;10;1(	Tight junction protein ZO-2	TJP2
O75663-2;O75663	O75663-2;(	1;1	1;1	1;1	TIP41-like protein	TIPRL
Q86XR7-2;A0A0A6YYA0	Q86XR7-2;	7;6;6;6;1;1	7;6;6;6;1;1	7;6;6;6;1;1	TIR domain-containing adap	TICAM2;TM
P13726;P13726-2	P13726;P1	3;2	3;2	3;2	Tissue factor	F3
A0A0A0MRA3;A0A0A0N	A0A0A0MF	1;1;1;1;1;1	1;1;1;1;1;1	1;1;1;1;1;1	Titin	TTN
E7EMV7;A0A0A0MRZ4;	E7EMV7;A(	5;5;5;5;5;5	5;5;5;5;5;5	5;5;5;5;5;5	TNFAIP3-interacting proteir	TNIP1
E9PNS3;F2Z2Y8;Q9H0E	E9PNS3;F2	1;1;1	1;1;1	1;1;1	Toll-interacting protein	TOLLIP
J3KN66;Q5JTV8-3;A0A0	J3KN66;Q5	6;6;5;5;3;2	6;6;5;5;3;2	6;6;5;5;3;2	Torsin-1A-interacting protei	TOR1AIP1
J3KPT4;Q9H4I3	J3KPT4;Q9I	1;1	1;1	1;1	TraB domain-containing prc	TRABD
P37837;F2Z393	P37837;F2	7;5	7;5	7;5	Transaldolase	TALDO1
Q9HBD4;A0A0A0MT49;	Q9HBD4;A(	1;1;1;1;1;1	1;1;1;1;1;1	1;1;1;1;1;1	Transcription activator BRG	SMARCA4
A0A1W2PPZ5;P23193;E	A0A1W2PF	5;5;4;4;4;2	5;5;4;4;4;2	5;5;4;4;4;2	Transcription elongation fac	TCEA1
E5RHG8;Q15369;Q1536	E5RHG8;Q	3;3;2;1	3;3;2;1	3;3;2;1	Transcription elongation fac	TCEB1
B8ZZU8;I3L0M9;Q1537(	B8ZZU8;I3L	4;4;4;4;1	4;4;4;4;1	4;4;4;4;1	Transcription elongation fac	TCEB2
O14776-2;O14776	O14776-2;(	2;2	2;2	2;2	Transcription elongation reg	TCERG1
Q00059;H7BYN3;Q0005	Q00059;H7	4;3;3	4;3;3	4;3;3	Transcription factor A, mito	TFAM
P20290-2;P20290	P20290-2;F	1;1	1;1	1;1	Transcription factor BTF3	BTF3
Q13263;Q13263-2;M0R	Q13263;Q1	11;10;6;3;2	11;10;6;3;2	11;10;6;3;2	Transcription intermediary	TRIM28
P49711	P49711	3	3	3	Transcriptional repressor CT	CTCF
A0A0U1RR30;A0A0U1R	A0A0U1RR	1;1;1;1;1;1	1;1;1;1;1;1	1;1;1;1;1;1	Transcriptional repressor p6	GATAD2B;(
E9PF19;Q9Y4P3;Q96E4	E9PF19;Q9	3;3;2;1;1	3;3;2;1;1	3;3;2;1;1	Transducin beta-like proteir	TBL2

Q12788;J3KNP2;A0A08	Q12788;J3I	8;6;2	8;6;2	8;6;2	Transducin beta-like protein	TBL3
P02786;G3V0E5;F8WBE	P02786;G3	23;20;3;3	23;20;3;3	23;20;3;3	Transferrin receptor protein	TFRC
Q13595-2;Q13595-4;Q1	Q13595-2;(	1;1;1;1	1;1;1;1	1;1;1;1	Transformer-2 protein hom	TRA2A
P62995;P62995-3;H7C2	P62995;P6.	4;3;2;1;1	4;3;2;1;1	4;3;2;1;1	Transformer-2 protein hom	TRA2B
Q15582;H0Y8M8;H0Y8I	Q15582;H0	2;1;1	2;1;1	2;1;1	Transforming growth factor	TGFBI
P61586	P61586	5	1	1	Transforming protein RhoA	RHOA
P37802;P37802-2;X6RJI	P37802;P3	11;11;10;1	11;11;10;1	11;11;10;1	Transgelin-2	TAGLN2
O43493-6;O43493-4;O4	O43493-6;(	2;2;2;2;2;2	2;2;2;2;2;2	2;2;2;2;2;2	Trans-Golgi network integra	TGOLN2
P55072;C9JUP7;C9IZA5	P55072	22;2;2	22;2;2	22;2;2	Transitional endoplasmic re	VCP
P29401;P29401-2;A0A0	P29401;P2!	18;18;15;1(	18;18;15;1(	18;18;15;1(	Transketolase	TKT
Q92616	Q92616	9	9	9	Translational activator GCN	GCN1L1
Q9BSH4	Q9BSH4	2	2	2	Translational activator of cy	TACO1
Q5W0H4;A0A0B4J2C3;I	Q5W0H4;A	6;6;6;5;4;3	6;6;6;5;4;3	6;6;6;5;4;3	Translationally-controlled tu	TPT1
G3XAN4;Q15629-2;Q15	G3XAN4;Q	3;3;3	3;3;3	3;3;3	Translocating chain-associa	TRAM1
F8WF48;Q99442;F8WC	F8WF48;Q!	2;2;1	2;2;1	2;2;1	Translocation protein SEC62	SEC62
Q9UGP8	Q9UGP8	4	4	4	Translocation protein SEC63	SEC63
B1AH87;P30536	B1AH87;P3	2;2	2;2	2;2	Translocator protein	TSPO
C9J3L8;C9J5W0;E9PAL7	C9J3L8;C9J	3;3;3;3;3;2	3;3;3;3;3;2	3;3;3;3;3;2	Translocon-associated prote	SSR1
P51571;A6NLM8	P51571	3;1	3;1	3;1	Translocon-associated prote	SSR4
C9JA28;Q9UNL2;Q9UNI	C9JA28;Q9	2;2;2;1	2;2;2;1	2;2;2;1	Translocon-associated prote	SSR3
E9PMQ9;E9PSI1;O1532	E9PMQ9;E!	3;3;3;3;2;2	3;3;3;3;2;2	3;3;3;3;2;2	Transmembrane 9 superfan	TM9SF1
Q99805	Q99805	6	6	6	Transmembrane 9 superfan	TM9SF2
Q9HD45;Q5TB53	Q9HD45;Q	3;2	3;2	3;2	Transmembrane 9 superfan	TM9SF3
A0A0C4DFM1;Q92544	A0A0C4DFI	5;5	5;5	5;5	Transmembrane 9 superfan	TM9SF4
J9JIE6;Q9UM00-2;Q9UI	J9JIE6;Q9U	2;2;2;1;1;1	2;2;2;1;1;1	2;2;2;1;1;1	Transmembrane and coiled	TMCO1
Q13445	Q13445	1	1	1	Transmembrane emp24 do	TMED1
G3V2K7;P49755	G3V2K7;P4	2;2	2;2	2;2	Transmembrane emp24 do	TMED10
F5GX39;E7EQ72;Q1536	F5GX39;E7	1;1;1	1;1;1	1;1;1	Transmembrane emp24 do	TMED2
Q7Z7H5-2;Q7Z7H5;F8W	Q7Z7H5-2;	4;4;3;3	4;4;3;3	3;3;2;2	Transmembrane emp24 do	TMED4
Q9Y3A6;M0R072;B1AK	Q9Y3A6;M	2;1;1;1	2;1;1;1	2;1;1;1	Transmembrane emp24 do	TMED5
Q9BVK6	Q9BVK6	3	2	2	Transmembrane emp24 do	TMED9
Q14956-2;Q14956	Q14956-2;(	3;3	3;3	3;3	Transmembrane glycoprote	GPNMB
Q9BVC6	Q9BVC6	2	2	2	Transmembrane protein 10	TMEM109
Q9HC07;V9GY93;Q9HC	Q9HC07;V!	3;2;2;1;1	3;2;2;1;1	3;2;2;1;1	Transmembrane protein 16	TMEM165
Q8IY95-2;Q8IY95	Q8IY95-2;C	2;2	2;2	2;2	Transmembrane protein 19	TMEM192
J3KRW7;J3KS81;Q8N51	J3KRW7;J3	1;1;1	1;1;1	1;1;1	Transmembrane protein 19	TMEM199
K7EM09;K7EPR0;K7ELQ	K7EM09;K7	1;1;1;1	1;1;1;1	1;1;1;1	Transmembrane protein 20	TMEM205
Q6NUQ4-2;Q6NUQ4;H7	Q6NUQ4-2	4;4;3;2	4;4;3;2	4;4;3;2	Transmembrane protein 21	TMEM214
K7ENI6;K7ERE1;Q8N2U	K7ENI6;K7E	1;1;1	1;1;1	1;1;1	Transmembrane protein 25	TMEM256
D6RAA6;P57088;H0Y8N	D6RAA6;P5	2;2;1	2;2;1	2;2;1	Transmembrane protein 33	TMEM33
Q9BTV4	Q9BTV4	4	4	4	Transmembrane protein 43	TMEM43
Q9BXS4;Q5T6Z8;Q5T70	Q9BXS4;Q5	5;3;2;1;1	5;3;2;1;1	5;3;2;1;1	Transmembrane protein 59	TMEM59
Q8NBN3-3;Q8NBN3;H3	Q8NBN3-3;	3;3;1;1	3;3;1;1	3;3;1;1	Transmembrane protein 87	TMEM87A
B1ALM5;Q9P0T7;B1ALI	B1ALM5;Q!	2;2;1;1;1	2;2;1;1;1	2;2;1;1;1	Transmembrane protein 9	TMEM9
Q92973-2;Q92973;Q92	Q92973-2;(	4;4;3;1;1	4;4;3;1;1	4;4;3;1;1	Transportin-1	TNPO1
J3KQ96;E7ETY2;Q1342	J3KQ96;E7I	15;15;15;1!	15;15;15;1!	15;15;15;1!	Treacle protein	TCOF1
P53007;B4DP62	P53007	3;1	3;1	3;1	Tricarboxylate transport pro	SLC25A1
P40939;H0YFD6;P4093	P40939	20;8;1	20;8;1	20;8;1	Trifunctional enzyme subun	HADHA
P55084-2;P55084;F5GZ	P55084-2;F	14;14;12;8	14;14;12;8	14;14;12;8	Trifunctional enzyme subun	HADHB
P22102;P22102-2;C9JZ(	P22102;P2	4;3;2;2;2;2	4;3;2;2;2;2	4;3;2;2;2;2	Trifunctional purine biosynt	GART

P60174-1;P60174;P601	P60174-1;F	15;15;10;7;	15;15;10;7;	15;15;10;7;	Triosephosphate isomerase	TPI1
Q14134-2;Q14134	Q14134-2;(	2;2	2;2	2;2	Tripartite motif-containing	TRIM29
Q96LD4-2;Q96LD4	Q96LD4-2;(	2;2	2;2	2;2	Tripartite motif-containing	TRIM47
Q08J23-2;Q08J23;Q08J	Q08J23-2;C	14;14;11;2	14;14;11;2	14;14;11;2	tRNA (cytosine(34)-C(5))-me	NSUN2
F5H1S9;G8JLB3;Q9Y606	F5H1S9;G8	3;3;3;3;1;1	3;3;3;3;1;1	3;3;3;3;1;1	tRNA pseudouridine syntha	PUS1
Q9Y3I0	Q9Y3I0	8	8	8	tRNA-splicing ligase RtcB hc	RTCB
Q9NYL9;H0YKU1;H0YNJ	Q9NYL9;H0	4;3;3;1	4;3;3;1	4;3;3;1	Tropomodulin-3	TMOD3
P09493-3;P09493-4;P09	P09493-3;F	10;9;9;9;8;	4;3;3;3;2;2	1;1;1;1;1;1	Tropomyosin alpha-1 chain	TPM1
P06753-2;A0A087WWU	P06753-2;A	18;17;17;1	18;17;17;1	12;11;12;1	Tropomyosin alpha-3 chain	TPM3;DKFZ
P67936;K7EPB9;U3KQK	P67936	14;6;1;1;1	2;0;0;0;0	2;0;0;0;0	Tropomyosin alpha-4 chain	TPM4
P67936-2;K7ENT6;K7ER	P67936-2;K	15;11;7;6;6	9;5;1;0;1;4	2;1;1;0;0;0	Tropomyosin alpha-4 chain	TPM4
P23381-2;P23381;H0YJI	P23381-2;F	6;6;3;3;3;2	6;6;3;3;3;2	6;6;3;3;3;2	Tryptophan--tRNA ligase, cy	WARS
Q71U36-2;Q71U36;Q13	Q71U36-2;	18;18;14;1	1;1;1;1;1;1	0;0;0;0;0;0	Tubulin alpha-1A chain;Tub	TUBA1A;TU
P68363;P68363-2;C9JD	P68363;P6	18;15;5	18;15;5	0;0;0	Tubulin alpha-1B chain	TUBA1B
F5H5D3;Q9BQE3;A0A1	F5H5D3;Q9	18;18;12;8;	2;2;2;0;0;0	2;2;2;0;0;0	Tubulin alpha-1C chain	TUBA1C
P68366-2;P68366;C9JE	P68366-2;F	18;18;6;6;6	4;4;3;3;3;2	4;4;3;3;3;2	Tubulin alpha-4A chain	TUBA4A
Q5JP53;P07437;Q5ST81	Q5JP53;P0	16;16;13;6;	4;4;1;0;0;0	3;3;1;0;0;0	Tubulin beta chain	TUBB
Q13885;Q9BVA1	Q13885;Q9	15;14	3;2	3;2	Tubulin beta-2A chain;Tubu	TUBB2A;TU
A0A0B4J269;Q13509;Q	A0A0B4J26	11;11;9;2;2	2;2;1;1;1;1	1;1;0;1;1;1	Tubulin beta-3 chain	TUBB3
P68371;P04350;M0R2D	P68371;P0	17;12;5;2;2	17;12;5;2;2	2;1;1;1;1;1	Tubulin beta-4B chain;Tubu	TUBB4B;TU
Q9BUF5;K7ESM5;K7ESC	Q9BUF5;K7	9;8;2;2;2;2	3;2;2;2;2;2	3;2;2;2;2;2	Tubulin beta-6 chain	TUBB6
Q3ZCM7;A0A075B736;(	Q3ZCM7;A	9;8;8	2;2;2	2;2;2	Tubulin beta-8 chain	TUBB8
K7EKE5;Q9NRH3;P2325	K7EKE5;Q9	1;1;1	1;1;1	1;1;1	Tubulin gamma-2 chain;Tub	TUBG1;TUE
E5RIW3;E5RJD8;O7534	E5RIW3;E5	3;3;3;2;2	3;3;3;2;2	3;3;3;2;2	Tubulin-specific chaperone	TBCA
Q14166	Q14166	4	4	4	Tubulin--tyrosine ligase-like	TTLL12
Q03169	Q03169	1	1	1	Tumor necrosis factor alpha	TNFAIP2
O14763-2;O14763	O14763-2;(	1;1	1;1	1;1	Tumor necrosis factor recep	TNFRSF10E
Q9H3D4-10;Q9H3D4-12	Q9H3D4-1(	2;2;2;2;2;2	2;2;2;2;2;2	2;2;2;2;2;2	Tumor protein 63	TP63
P55327-2;P55327-4;P55	P55327-2;F	4;4;4;4;4;4	4;4;4;4;4;4	4;4;4;4;4;4	Tumor protein D52	TPD52
A0A087WYR3;O43399-2	A0A087WY	5;5;5;5;5;5	5;5;5;5;5;5	5;5;5;5;5;5	Tumor protein D54	TPD52L2
Q5T0D9;Q5T0D9-2	Q5T0D9;Q5	3;2	3;2	3;2	Tumor protein p63-regulate	TPRG1L
Q12888;Q12888-3;Q12	Q12888;Q1	1;1;1	1;1;1	1;1;1	Tumor suppressor p53-bind	TP53BP1
P09758	P09758	8	8	8	Tumor-associated calcium s	TACSTD2
Q12792;F8VRG3;F8VS8	Q12792;F8	2;1;1;1;1	2;1;1;1;1	2;1;1;1;1	Twinfilin-1	TWF1
D6RG15;H0Y858;Q6IBS	D6RG15;H0	1;1;1	1;1;1	1;1;1	Twinfilin-2	TWF2
Q6RW13-2;Q6RW13	Q6RW13-2	1;1	1;1	1;1	Type-1 angiotensin II recept	AGTRAP
Q9UIG0-2;Q9UIG0	Q9UIG0-2;(	11;11	11;11	11;11	Tyrosine-protein kinase BAZ	BAZ1B
P07948-2;P07948;H0Y3	P07948-2;F	5;4;1;1;1;1	5;4;1;1;1;1	5;4;1;1;1;1	Tyrosine-protein kinase Lyn	LYN
P18031;B4DSN5	P18031;B4	8;5	8;5	8;5	Tyrosine-protein phosphata	PTPN1
P54577;A0A0C4DGZ5	P54577;A0	3;2	3;2	3;2	Tyrosine--tRNA ligase, cytop	YARS
H0YHS6;Q9Y2Z4	H0YHS6;Q9	3;3	3;3	3;3	Tyrosine--tRNA ligase;Tyros	YARS2
P08621;P08621-2;P086	P08621;P0	6;5;3;3;2;1	6;5;3;3;2;1	6;5;3;3;2;1	U1 small nuclear ribonuclec	SNRNP70
M0R221;M0R2B8;M0Q	M0R221;M	2;2;2;2	1;1;1;1	1;1;1;1	U1 small nuclear ribonuclec	SNRPA
A0A0A0MRR7;P09234	A0A0A0MF	2;2	2;2	2;2	U1 small nuclear ribonuclec	SNRPC
P09661;H0YLR3;H0YMA	P09661;H0	4;3;3;1	4;3;3;1	4;3;3;1	U2 small nuclear ribonuclec	SNRPA1
P08579;M0R0G9;M0QX	P08579;M0	2;1;1;1	2;1;1;1	1;0;0;0	U2 small nuclear ribonuclec	SNRPB2;SN
O15042-2;O15042;E7ET	O15042-2;(	4;4;3;2;2;2	4;4;3;2;2;2	4;4;3;2;2;2	U2 snRNP-associated SURP	U2SURP
Q9NV31	Q9NV31	2	2	2	U3 small nucleolar ribonucl	IMP3
O00566	O00566	1	1	1	U3 small nucleolar ribonucl	MPHOSPH1
Q9BVJ6-3;Q9BVJ6;Q9B	Q9BVJ6-3;(	5;5;3;2	5;5;3;2	5;5;3;2	U3 small nucleolar RNA-ass	UTP14A

Q9Y5J1;J3QR85;J3KSR7	Q9Y5J1;J3C	2;1;1	2;1;1	2;1;1	U3 small nucleolar RNA-ass	UTP18
Q9NYH9	Q9NYH9	2	2	2	U3 small nucleolar RNA-ass	UTP6
O43818	O43818	3	3	3	U3 small nucleolar RNA-inte	RRP9
O43172-2;O43172	O43172-2;(	1;1	1;1	1;1	U4/U6 small nuclear ribonu	PRPF4
A0A087X1B2;B9A018;Q	A0A087X1E	2;2;2;2;1	2;2;2;2;1	2;2;2;2;1	U4/U6.U5 tri-snRNP-associa	USP39
O75643;O75643-2;B4E(	O75643	17;6;2	17;6;2	17;6;2	U5 small nuclear ribonuclec	SNRNP200
Q96DI7;Q96DI7-2;Q9NS	Q96DI7;Q9	3;3;1	3;3;1	3;3;1	U5 small nuclear ribonuclec	SNRNP40
Q9Y333	Q9Y333	1	1	1	U6 snRNA-associated Sm-lik	LSM2
F2Z2Y6;C9JIZ0;C9JNV3;(	F2Z2Y6;C9J	1;1;1;1	1;1;1;1	1;1;1;1	U6 snRNA-associated Sm-lik	LSM8
Q9UMX0-2;Q9UMX0;Q(	Q9UMX0-2	5;5;2;1;1;1	5;5;2;1;1;1	3;3;2;1;0;1	Ubiquilin-1	UBQLN1
H3BPW3;H3BVA5;H3BP	H3BPW3;H	1;1;1;1;1;1	1;1;1;1;1;1	1;1;1;1;1;1	Ubiquinone biosynthesis pr	COQ9
H3BQP1;Q14694;Q146(	H3BQP1;Q	1;1;1;1	1;1;1;1	1;1;1;1	Ubiquitin carboxyl-terminal	USP10
P45974-2;P45974;F5H5	P45974-2;F	2;2;1	2;2;1	2;2;1	Ubiquitin carboxyl-terminal	USP5
P15374;A0A087WTB8;(	P15374;A0	6;5;4	6;5;4	6;5;4	Ubiquitin carboxyl-terminal	UCHL3
A6NJA2;P54578-2;P545	A6NJA2;P5	8;8;8;8;1;1	8;8;8;8;1;1	8;8;8;8;1;1	Ubiquitin carboxyl-terminal	USP14
H0Y6Y4;Q5LJB0;Q5LJA5	H0Y6Y4;Q5	2;2;2;2;2;2	2;2;2;2;2;2	2;2;2;2;2;2	Ubiquitin carboxyl-terminal	UCHL5
Q92890;Q92890-1;C9J6	Q92890;Q(	2;2;1;1	2;2;1;1	2;2;1;1	Ubiquitin fusion degradatio	UFD1L
F5GYN4;J3KR44;Q96FW	F5GYN4;J3	4;4;4;3;3;3	4;4;4;3;3;3	4;4;4;3;3;3	Ubiquitin thioesterase OTU	OTUB1
P62979;J3QS39;J3QTR3	P62979;J3(	7;5;5;5;5;5	7;5;5;5;5;5	7;5;5;5;5;5	Ubiquitin-40S ribosomal prc	RPS27A;UB
F8W726;Q14157-4;Q14	F8W726;Q	4;4;4;4;4;4	4;4;4;4;4;4	4;4;4;4;4;4	Ubiquitin-associated protei	UBAP2L
O00762-2;O00762-3;O(	O00762-2;(	2;2;2	2;2;2	2;2;2	Ubiquitin-conjugating enzyr	UBE2C
P61086;D6RDM7;P610(	P61086;D6	3;2;2;1	3;2;2;1	3;2;2;1	Ubiquitin-conjugating enzyr	UBE2K
A0A1B0GUS4;P68036-2	A0A1B0GU	1;1;1;1	1;1;1;1	1;1;1;1	Ubiquitin-conjugating enzyr	UBE2L3
P61088;F8VZ29;F8VSD4	P61088;F8	4;3;3;3;3;1	4;3;3;3;3;1	4;3;3;3;3;1	Ubiquitin-conjugating enzyr	UBE2N;UBI
I3L0A0;Q13404;Q13404	I3L0A0;Q1	4;3;3;3;3;2	4;3;3;3;3;2	2;1;1;1;1;0	Ubiquitin-conjugating enzyr	TMEM189-
Q15819	Q15819	4	2	2	Ubiquitin-conjugating enzyr	UBE2V2
H0Y614;P61960	H0Y614;P6	1;1	1;1	1;1	Ubiquitin-fold modifier 1	UFM1
P22314-2;P22314;Q5JR	P22314-2;F	21;21;8;5;5	21;21;8;5;5	21;21;8;5;5	Ubiquitin-like modifier-acti	UBA1
Q9BZL1;K7EQ43	Q9BZL1;K7	2;1	2;1	2;1	Ubiquitin-like protein 5	UBL5
A0A087WTZ5;E9PJ81;E(	A0A087WT	1;1;1;1;1	1;1;1;1;1	1;1;1;1;1	UBX domain-containing pro	UBXN1
Q92575	Q92575	3	3	3	UBX domain-containing pro	UBXN4
O60701;O60701-2;O60	O60701;O6	9;8;6;3;3;3	9;8;6;3;3;3	9;8;6;3;3;3	UDP-glucose 6-dehydrogen	UGDH
Q9NYU2-2;Q9NYU2;H7I	Q9NYU2-2;	10;10;2;1	10;10;2;1	10;10;2;1	UDP-glucose:glycoprotein g	UGGT1
P19224-3;P19224;C9JM	P19224-3;F	4;4;2;1;1;1	4;4;2;1;1;1	4;4;2;1;1;1	UDP-glucuronosyltransferas	UGT1A6
P30085;Q5T0D2;P3008	P30085;Q5	5;4;3	5;4;3	5;4;3	UMP-CMP kinase	CMPK1
Q8N999-3;Q8N999	Q8N999-3;	1;1	1;1	1;1	Uncharacterized protein C1	C12orf29
Q8WWC4	Q8WWC4	1	1	1	Uncharacterized protein C2	C2orf47
C9JQV0;Q9BRJ6;H7C0T	C9JQV0;Q9	2;2;1;1	2;2;1;1	2;2;1;1	Uncharacterized protein C7	C7orf50
E9PDF6;O43795-2;O43	E9PDF6;O4	20;20;20;9	20;20;20;9	20;20;20;9	Unconventional myosin-Ib	MYO1B
F5H6E2;O00159-3;O00	F5H6E2;O0	12;12;12;1	12;12;12;1	12;12;12;1	Unconventional myosin-Ic	MYO1C
Q12965;H0YLE5;M0QX(	Q12965;H(	3;2;1;1	3;2;1;1	3;2;1;1	Unconventional myosin-Ie	MYO1E
A0A0A0MRM8;A0A0D9	A0A0A0MF	2;2;2;2;2;2	2;2;2;2;2;2	2;2;2;2;2;2	Unconventional myosin-VI	MYO6
Q9H7E9;Q9H7E9-2	Q9H7E9;Q(	1;1	1;1	1;1	UPF0488 protein C8orf33	C8orf33
H0YJB9;G3V4C6;Q9Y22	H0YJB9;G3	1;1;1	1;1;1	1;1;1	UPF0568 protein C14orf166	C14orf166
H0YF29;Q6P1X6-2;Q6P	H0YF29;Q6	1;1;1	1;1;1	1;1;1	UPF0598 protein C8orf82	C8orf82
Q96IX5	Q96IX5	2	2	2	Up-regulated during skeleta	USMG5
P11172-3;P11172;E9PF	P11172-3;F	3;3;2;2;1	3;3;2;2;1	3;3;2;2;1	Uridine 5-monophosphate s	UMPS
C9J486;C9JIP2;B4DND0	C9J486;C9J	3;3;3;3;1	3;3;3;3;1	3;3;3;3;1	Uridine phosphorylase 1	UPP1
G3V170;Q9HA47-2;Q9H	G3V170;Q(	1;1;1;1;1	1;1;1;1;1	1;1;1;1;1	Uridine-cytidine kinase 1	UCK1
Q5T446;H0Y5R6;A0A1B	Q5T446;H(	1;1;1;1;1	1;1;1;1;1	1;1;1;1;1	Uroporphyrinogen decarbo	UROD

Q5H8X8;O95399;O9539	Q5H8X8;O9	1;1;1	1;1;1	1;1;1	Urotensin-2	UTS2
A0A087WYS1;E7EUC7;(	A0A087WY	3;3;3;3;2;2	3;3;3;3;2;2	3;3;3;3;2;2	UTP--glucose-1-phosphate u	UGP2
P54727;P54727-2;H0Y5	P54727;P5	8;5;3;3;1;1	8;5;3;3;1;1	8;5;3;3;1;1	UV excision repair protein R	RAD23B
Q3ZAQ7	Q3ZAQ7	1	1	1	Vacuolar ATPase assembly i	VMA21
O75436;S4R3Q6;O7543	O75436;S4	3;2;2	3;2;2	3;2;2	Vacuolar protein sorting-as	VPS26A
E9PLM9;E9PR04;E9PQR	E9PLM9;E9	1;1;1;1;1;1	1;1;1;1;1;1	1;1;1;1;1;1	Vacuolar protein sorting-as	VPS28
Q96QK1;I3L4S0;A0A1W	Q96QK1	6;1;1;1	6;1;1;1	6;1;1;1	Vacuolar protein sorting-as	VPS35
O75351;I3L4J1;Q9UN37	O75351;I3L	2;1;1;1;1	2;1;1;1;1	2;1;1;1;1	Vacuolar protein sorting-as	VPS4B;VPS
A0A087WY55;Q9NP79;	A0A087WY	2;2;1;1	2;2;1;1	2;2;1;1	Vacuolar protein sorting-as	VTA1
A0A140T936;P26640;P2	A0A140T93	5;5;2;1;1;1	5;5;2;1;1;1	5;5;2;1;1;1	Valine--tRNA ligase	VARS
Q8TAA9-2;Q8TAA9	Q8TAA9-2;	1;1	1;1	1;1	Vang-like protein 1	VANGL1
P49748;P49748-3;P497	P49748;P4	21;21;20;7	21;21;20;7	21;21;20;7	Very long-chain specific acy	ACADVL
Q6Y1H2	Q6Y1H2	1	1	1	Very-long-chain (3R)-3-hydr	HACD2
Q9P035;H3BS72;H3BPZ	Q9P035;H3	7;6;5;5;3;2	7;6;5;5;3;2	7;6;5;5;3;2	Very-long-chain (3R)-3-hydr	HACD3
A0A1B0GV93;Q53GQ0;	A0A1B0GV	3;3;1;1	3;3;1;1	3;3;1;1	Very-long-chain 3-oxoacyl-C	HSD17B12
Q9NZ01;M0QXM3;M0R	Q9NZ01;M	3;2;1;1	3;2;1;1	3;2;1;1	Very-long-chain enoyl-CoA r	TECR
Q5TIH2;O95562	Q5TIH2;O9	1;1	1;1	1;1	Vesicle transport protein SF	SFT2D2
Q15836;K7ENK9;K7EKX	Q15836;K7	4;2;2;2;2;2	4;2;2;2;2;2	4;2;2;2;2;2	Vesicle-associated membra	VAMP3;VA
Q9BV40;B8ZZT4;C9JXZ5	Q9BV40;B8	3;2;1	3;2;1	3;2;1	Vesicle-associated membra	VAMP8
Q9P0L0	Q9P0L0	2	1	1	Vesicle-associated membra	VAPA
O95292;E5RK64;O9529	O95292	5;2;2;1	5;2;2;1	4;1;1;0	Vesicle-associated membra	VAPB
I3L0N3;P46459-2;P4645	I3L0N3;P46	1;1;1	1;1;1	1;1;1	Vesicle-fusing ATPase	NSF
F8WAU7;C9JR77;C9J46	F8WAU7;C	1;1;1;1;1;1	1;1;1;1;1;1	1;1;1;1;1;1	Vesicle-trafficking protein S	SEC22A
O75396;A0A087X1A9	O75396	3;1	3;1	3;1	Vesicle-trafficking protein S	SEC22B
Q12907;D6RIU4;D6RBV	Q12907;D6	5;4;4;3;1	5;4;4;3;1	5;4;4;3;1	Vesicular integral-membran	LMAN2
A0A024R4E5;Q00341;Q	A0A024R4E	8;8;7;4;4;2	8;8;7;4;4;2	8;8;7;4;4;2	Vigilin	HDLBP
P18206-2;P18206;P182	P18206-2;P	22;22;3	22;22;3	22;22;3	Vinculin	VCL
CON__Q3MHN5;CON__	CON__Q3M	2;2;1;1;1;1	2;2;1;1;1;1	2;2;1;1;1;1	Vitamin D-binding protein	GC
Q8N0U8;Q8N0U8-2	Q8N0U8;Q	1;1	1;1	1;1	Vitamin K epoxide reductas	VKORC1L1
P21796;C9JI87	P21796;C9	17;10	17;10	17;10	Voltage-dependent anion-s	VDAC1
P45880;A0A0A0MR02;F	P45880;A0	17;16;15;1	17;16;15;1	17;16;15;1	Voltage-dependent anion-s	VDAC2
Q9Y277;Q9Y277-2;E5RH	Q9Y277;Q9	8;7;4;4;4;3	8;7;4;4;4;3	8;7;4;4;4;3	Voltage-dependent anion-s	VDAC3
Q8IWT6	Q8IWT6	2	2	2	Volume-regulated anion ch	LRRC8A
P27449	P27449	1	1	1	V-type proton ATPase 16 kD	ATP6V0C
P38606-2;P38606;C9JA	P38606-2;P	7;7;1;1	7;7;1;1	7;7;1;1	V-type proton ATPase catal	ATP6V1A
K7EQW2;K7ELZ6;K7EM	K7EQW2;K	1;1;1;1;1;1	1;1;1;1;1;1	1;1;1;1;1;1	V-type proton ATPase subu	ATP6V0A1
P21281;C9JL73;P15313;	P21281;C9	8;4;4;3;2;2	8;4;4;3;2;2	8;4;4;3;2;2	V-type proton ATPase subu	ATP6V1B2;
H0YJ55;H0YJS0;G3V559	H0YJ55;H0	1;1;1;1;1	1;1;1;1;1	1;1;1;1;1	V-type proton ATPase subu	ATP6V1D
F5GYQ1;P61421;J3QL1	F5GYQ1;P6	3;3;2;2;1;1	3;3;2;2;1;1	3;3;2;2;1;1	V-type proton ATPase subu	ATP6V0D1
P36543;P36543-3;P365	P36543;P3	4;3;3;2;1	4;3;3;2;1	4;3;3;2;1	V-type proton ATPase subu	ATP6V1E1
O75348;F2Z307;O9567	O75348	3;1;1;1	3;1;1;1	3;1;1;1	V-type proton ATPase subu	ATP6V1G1
G3V126;Q9UI12-2;Q9U	G3V126;Q9	5;5;5;1;1	5;5;5;1;1	5;5;5;1;1	V-type proton ATPase subu	ATP6V1H
O75083;D6RD66;O7508	O75083;D6	7;4;4	7;4;4	7;4;4	WD repeat-containing prote	WDR1
U3KQC1;A0A0A0MQU0	U3KQC1;A0	5;5;5;3;2;1	5;5;5;3;2;1	5;5;5;3;2;1	WD repeat-containing prote	WDR18
A0A0A0MTB8;Q8NI36	A0A0A0MT	3;3	3;3	3;3	WD repeat-containing prote	WDR36
Q15061;C9JEE7;C9IZK7	Q15061	3;1;1	3;1;1	3;1;1	WD repeat-containing prote	WDR43
O15213;H0Y6G3	O15213;H0	4;3	4;3	1;0	WD repeat-containing prote	WDR46
P61964	P61964	1	1	1	WD repeat-containing prote	WDR5
H0YM76;H0YMF9;H0YN	H0YM76;H0	1;1;1;1	1;1;1;1	1;1;1;1	WD repeat-containing prote	WDR61
E9PS41;Q6RFH5;H0YFP	E9PS41;Q6	2;2;1;1	2;2;1;1	2;2;1;1	WD repeat-containing prote	WDR74

Q8IWA0;F8WE20;F8WC	Q8IWA0;F8	2;1;1	2;1;1	2;1;1	WD repeat-containing prot	WDR75
Q9UBH6-2;Q9UBH6	Q9UBH6-2;	1;1	1;1	1;1	Xenotropic and polytropic r	XPR1
P13010;C9JZ81;H7C0H9	P13010	16;2;1	16;2;1	16;2;1	X-ray repair cross-complem	XRCC5
P12956;B1AHC9;P1295	P12956;B1	13;12;12	13;12;12	13;12;12	X-ray repair cross-complem	XRCC6
P16989-2;P16989-3;P16	P16989-2;F	6;6;6;3	3;3;3;1	3;3;3;1	Y-box-binding protein 3	YBX3
Q9Y5A9-2;Q9Y5A9;A0A	Q9Y5A9-2;	2;2;1;1;1;1	2;2;1;1;1;1	2;2;1;1;1;1	YTH domain-containing fam	YTHDF2;YT
E9PQ61;O75152	E9PQ61;O7	2;2	2;2	2;2	Zinc finger CCCH domain-co	ZC3H11A
Q8WU90-2;Q8WU90	Q8WU90-2	1;1	1;1	1;1	Zinc finger CCCH domain-co	ZC3H15
C9J6P4;Q7Z2W4-3;Q7Z	C9J6P4;Q7	5;5;5;5	5;5;5;5	5;5;5;5	Zinc finger CCCH-type antivi	ZC3HAV1
O95159;H0YCU8;E9PM	O95159;H0	2;1;1;1;1;1	2;1;1;1;1;1	2;1;1;1;1;1	Zinc finger protein-like 1	ZFPL1
Q96KR1	Q96KR1	2	2	2	Zinc finger RNA-binding pro	ZFR
Q9BQ52-4;Q9BQ52;V9	Q9BQ52-4;	3;3;2;2;1;1	3;3;2;2;1;1	3;3;2;2;1;1	Zinc phosphodiesterase ELA	ELAC2
H0Y362;Q8NEW0	H0Y362;Q8	2;2	2;2	2;2	Zinc transporter 7	SLC30A7
Q5T4K2;Q5T4K4;Q5T4K	Q5T4K2;Q5	1;1;1;1;1	1;1;1;1;1	1;1;1;1;1	Zinc transporter ZIP1	SLC39A1
E5RIP4;Q15043-2;Q150	E5RIP4;Q1	1;1;1;1	1;1;1;1	1;1;1;1	Zinc transporter ZIP14	SLC39A14
Q8N4Q0	Q8N4Q0	2	2	2	Zinc-binding alcohol dehydr	ZADH2
H0Y2Y8;Q15942;Q1594	H0Y2Y8;Q1	3;3;2;1	3;3;2;1	3;3;2;1	Zyxin	ZYX

Fasta head	Number of	Peptides	Razor + uni	Unique pe	Peptides 1_	Peptides 2	Peptides 3	Peptides 4
sp|P61604	4	8	8	8	8	8	7	7
sp|Q15029	6	10	9	9	7	8	8	5
sp|Q9NRX4	2	1	1	1	1	1	1	1
sp|P31946	6	8	4	4	8	8	7	7
sp|P62258	7	15	12	12	12	11	12	12
sp|Q04917	4	12	9	9	8	8	10	9
sp|P61981	1	10	7	7	7	6	8	7
sp|P31947	2	17	17	14	14	15	14	14
sp|P27348	2	12	8	8	11	11	10	10
sp|P63104	11	14	11	10	11	10	10	12
sp|Q9C0C2	2	3	3	3	1	0	1	1
sp|Q9NUQ	1	2	2	2	2	2	2	2
sp|P09543	2	1	1	1	1	1	0	0
sp|Q16698	7	7	7	7	6	7	7	7
sp|Q9Y6K5	3	4	4	4	3	2	0	0
tr|A0A087	6	7	7	7	7	7	5	4
sp|P62191	3	5	5	5	4	4	2	4
tr|E9PM69	6	5	5	5	4	4	4	3
sp|P43686	2	4	4	4	4	4	2	2
sp|P35998	4	10	10	10	8	7	6	7
tr|J3QQM1	9	5	5	4	4	3	2	4
tr|A0A087	6	6	6	6	3	4	1	2
tr|B1AJY5|	5	2	2	2	1	1	0	1
sp|O00231	4	10	10	10	6	4	3	3
sp|O00232	4	6	6	6	2	4	1	1
tr|J3KNQ3	9	6	6	6	2	2	3	2
sp|O00487	1	1	1	1	1	1	1	1
sp|Q13200	7	12	12	12	8	12	6	7
sp|O43242	2	5	5	5	5	4	5	4
tr|Q5VWC	5	2	2	2	0	0	0	1
sp|Q15008	7	4	4	4	0	0	0	0
tr|H3BNT7	4	3	3	3	1	2	1	2
tr|F5H5V4	7	2	2	2	1	1	1	1
sp|Q13442	1	5	5	5	0	0	2	1
sp|P82912	2	1	1	1	1	1	1	1
tr|A6ND22	3	1	1	1	1	1	1	1
tr|E9PE17|	3	2	2	2	1	2	2	2
sp|Q9Y676	2	4	4	4	3	4	4	4
sp|Q9Y399	2	3	3	3	3	3	3	3
sp|P82921	1	1	1	1	1	1	1	0
tr|G5E9W7	7	4	4	4	3	2	3	2
tr|J3QLR8|	2	2	2	2	1	1	2	1
sp|P82663	4	3	3	3	3	3	1	1
tr|D6RH20	5	2	2	2	2	2	2	2
sp|Q9Y2Q9	7	2	2	2	2	0	2	2
sp|P82909	2	2	2	2	2	2	2	2
sp|P82675	1	1	1	1	1	1	1	1
sp|P82932	1	1	1	1	1	1	1	1

tr|J3QQS1	5	3	3	3	3	3	3	3
sp|P82933	1	2	2	2	1	2	1	1
tr|E9PDF2|	11	18	18	18	14	16	17	16
tr|H0Y8N7	2	1	1	1	1	1	1	1
sp|Q9Y3B7	5	3	3	3	2	2	3	3
tr|B4DLN1	2	6	6	3	4	3	4	3
tr|H0YAX3	3	2	2	2	2	2	2	1
sp|Q9H0U	1	3	3	3	2	2	2	3
tr|C9IY40|	2	1	1	1	1	0	1	1
tr|E7ESL0|	5	2	2	2	2	2	2	2
sp|Q96A35	1	2	2	2	1	2	1	1
tr|H0Y4J2|	4	1	1	1	1	1	1	1
sp|Q96DV4	2	2	2	2	2	1	2	2
tr|C9JG87|	3	1	1	1	0	1	0	1
tr|K7EKI4|	7	1	1	1	1	1	1	1
tr|H0YBU8	9	2	2	2	2	2	2	2
sp|Q9H9J2	1	1	1	1	1	1	1	1
tr|E9PI78|	3	1	1	1	1	1	1	1
sp|Q8N5N	2	2	2	2	1	2	1	1
sp|Q96EL3	1	2	2	2	1	1	1	1
sp|Q9BYD2	1	1	1	1	1	1	1	1
sp|Q15125	3	3	3	3	3	3	2	1
sp|Q99714	3	9	9	9	5	6	6	8
sp|P31937	2	2	2	2	2	2	2	2
tr|K7ERC8|	2	1	1	1	1	1	1	1
sp|P51857	2	1	1	1	1	0	1	1
sp|P46783	5	5	5	5	2	2	1	3
sp|P62280	4	10	10	10	8	8	9	8
sp|P25398	1	7	7	7	7	7	7	6
sp|P62277	3	6	6	6	6	5	5	3
sp|P62263	3	7	7	7	6	7	6	5
tr|I3L3P7|	3	7	7	2	5	4	4	4
sp|P62249	6	6	6	6	5	4	4	4
sp|P08708	5	7	7	7	7	5	5	4
sp|P62269	4	6	6	6	6	6	6	6
sp|P39019	6	8	8	8	7	5	6	6
sp|P15880	7	9	9	9	7	8	8	7
sp|P60866	5	3	3	3	3	3	3	3
tr|Q8WVC	3	5	5	5	3	2	3	2
sp|P62266	5	4	4	4	4	4	3	4
tr|E7ETK0|	6	3	3	3	2	2	2	2
sp|P62851	1	3	3	3	2	2	2	2
sp|P62854	2	3	3	3	3	3	3	2
sp|P42677	6	4	4	4	4	4	3	3
sp|P62857	1	2	2	2	1	2	2	2
sp|P62273	3	2	2	2	1	1	1	1
sp|P23396	15	13	13	13	12	12	11	9
tr|E9PR30|	2	1	1	1	1	1	1	1
sp|P61247	12	12	12	12	10	12	10	9
sp|P62701	4	12	12	12	8	10	6	6

tr|M0R0F0	4	7	7	7	7	7	7	4
sp|P62753	3	6	6	6	5	6	4	5
sp|P62081	2	4	4	4	3	4	3	2
sp|P62241	2	10	10	10	10	10	10	9
tr|A0A024I	6	9	9	9	8	8	7	7
tr|C9J9K3|	4	8	8	8	8	8	7	7
sp|P08195	12	25	25	25	24	22	23	22
sp|Q9H0D(	2	2	2	2	2	2	2	2
sp|P21589	5	2	2	2	2	1	2	2
sp|P10809	7	28	28	28	25	26	26	25
tr|F8VU65	13	9	9	9	5	8	8	9
sp|P05386	1	2	1	1	2	2	2	2
sp|P05387	3	7	7	6	7	7	6	6
tr|X1WI28	4	8	8	2	7	8	6	5
sp|P62906	1	8	8	8	7	7	6	7
sp|P62913	3	4	4	1	4	4	3	4
sp|P62913	1	4	1	1	4	4	3	4
sp|P30050	2	6	6	6	5	6	5	5
sp|P26373	5	7	7	7	5	6	5	5
sp|P40429	6	8	8	8	6	7	6	6
tr|E7EPB3|	2	4	4	4	4	4	4	4
sp|P61313	6	6	6	6	6	6	6	6
tr|A0A087'	14	7	7	7	4	4	4	2
tr|G3V203	8	4	4	4	4	4	4	4
tr|M0R3D(	5	5	5	5	5	4	5	5
tr|G3V1B3	3	2	2	2	2	1	2	1
tr|K7EJT5|	7	2	2	2	2	2	2	2
sp|P62829	5	4	4	4	4	4	4	4
tr|H7BY10	6	4	4	4	3	4	3	4
tr|C9JXB8|	3	6	6	6	4	5	5	5
sp|P61254	11	6	6	6	4	6	4	3
sp|P61353	4	4	4	4	3	4	3	2
tr|E9PLL6|	4	4	4	4	3	3	3	3
tr|H0YMF4	8	5	5	5	4	4	4	4
sp|P47914	1	2	2	2	0	2	2	2
sp|P39023	7	16	16	16	15	16	12	11
sp|P62888	5	5	5	5	4	5	5	4
tr|H7C2W(	8	4	4	4	4	4	3	3
tr|D3YTB1	3	5	5	5	4	4	4	4
sp|P49207	1	6	6	6	5	6	6	6
tr|F2Z388|	2	2	2	2	2	2	2	1
tr|C9K025|	4	5	5	5	4	4	4	4
sp|Q9Y3U8	3	3	3	3	3	3	3	3
tr|H0Y5B4	6	2	2	2	2	2	2	2
tr|C9J4Z3|	8	4	4	4	2	3	3	1
tr|J3KT73|	3	1	1	1	1	1	1	1
sp|P36578	4	15	15	15	14	14	13	11
sp|P46777	2	9	9	9	7	9	8	6
sp|Q02878	7	13	13	13	12	12	12	10
tr|A8MUD(	4	6	6	6	6	6	6	6

sp|P62424	3	14	14	14	12	13	13	13
tr|R4GMU1	3	1	1	1	1	1	1	1
sp|P62917	5	5	5	5	3	3	3	3
sp|P32969	5	5	5	5	5	4	4	3
tr|J3QLW7	3	2	2	2	0	1	0	0
sp|P52209	5	10	10	10	3	4	5	4
tr|M0R0U3	4	2	2	2	0	0	0	1
sp|P36639	4	2	2	2	0	1	1	2
sp|P11021	1	30	30	29	27	28	25	24
sp|Q9UBM	8	6	6	6	5	5	5	6
sp|Q7L2J0	1	1	1	1	1	0	0	0
tr|A0A0J9Y	4	3	3	3	1	1	3	2
tr|M0R026	2	1	1	1	0	1	1	0
sp|Q9BWD	2	2	2	2	1	1	1	0
sp|P24752	5	6	6	6	6	6	6	6
sp|O00763	7	1	1	1	1	1	1	0
tr|H0YN26	4	4	4	3	1	0	2	1
sp|Q92688	2	2	1	1	1	0	1	1
tr|A2A274	2	9	9	9	6	8	6	6
sp|P60709	14	19	1	1	19	19	19	18
sp|P63261	7	19	19	1	19	19	19	18
sp|P63267	10	10	1	1	10	10	10	10
sp|O96019	5	6	6	6	4	4	4	4
sp|P61160	5	5	5	5	0	2	1	2
sp|O15143	16	4	4	4	3	2	2	2
sp|O15144	4	4	4	4	1	2	2	1
tr|F8VR50|	3	1	1	1	0	0	0	0
tr|F8WDD7	11	4	4	4	3	3	2	2
tr|B1ALC0|	3	1	1	1	1	1	1	1
sp|Q9BPX5	1	1	1	1	0	1	1	1
sp|P61158	7	7	7	7	6	4	6	6
sp|P53999	1	3	3	3	3	3	3	3
sp|O95433	7	8	8	8	1	2	2	3
sp|Q86WX	1	1	1	1	0	0	0	0
sp|O14561	3	2	2	2	2	2	1	1
tr|C9JIF9|C	2	2	2	2	1	1	2	2
sp|Q9H845	2	5	5	5	5	5	5	5
sp|O00767	1	2	2	2	2	2	2	2
tr|B8ZWD1	8	3	3	3	1	2	2	2
sp|Q9NPJ3	2	1	1	1	1	1	1	1
tr|H7C5A7	4	2	2	2	2	2	2	2
sp|Q9Y305	5	5	5	5	5	5	5	5
tr|A0A087	7	3	3	3	1	2	3	3
tr|Q5QPQ0	2	2	2	2	2	2	2	1
sp|Q6P587	3	1	1	1	1	1	1	0
tr|H3BSW3	6	3	3	3	2	3	1	0
sp|Q8TB61	5	2	2	2	1	1	1	0
sp|P55263	4	1	1	1	1	0	0	0
sp|P23526	2	15	15	15	10	11	11	9
tr|F8W1A4	11	6	6	6	5	3	3	3

sp|P27144	2	2	2	2	1	1	2	2
tr|Q5T9B7	4	5	5	5	2	3	3	2
tr|A0A096I	9	2	2	2	0	0	0	0
sp|P30520	1	5	5	5	0	0	2	3
sp|Q01518	18	11	11	11	6	7	9	9
sp|Q9HDC9	3	6	6	6	5	6	5	5
sp|Q15847	1	1	1	1	0	0	0	1
sp|P05141	2	15	15	6	14	14	13	15
sp|P12236	1	13	4	2	13	13	13	13
sp|Q9BRR6	8	3	3	3	2	3	3	3
sp|P84077	6	6	6	3	4	4	5	4
tr|C9JPM4	3	3	1	1	3	3	3	3
tr|C9J1Z8|	5	5	2	2	3	3	2	3
sp|P62330	1	3	3	3	3	3	3	3
sp|Q9NVJ2	1	2	2	1	2	2	2	2
tr|A6NCQ0	6	3	3	3	0	0	2	1
sp|Q9Y4W	1	2	2	2	1	2	2	2
tr|A0A087Z	9	5	5	5	4	4	4	4
sp|O00170	1	1	1	1	0	1	1	1
tr|H0YK84	5	1	1	1	0	0	1	0
tr|H3BU82	5	2	2	2	2	2	2	2
sp|P49588	3	7	7	7	6	6	5	3
sp|P14550	4	4	4	4	0	0	1	3
sp|P11766	3	4	4	4	1	2	2	2
sp|P47895	10	18	18	16	14	13	12	9
sp|P30837	1	4	2	2	2	2	2	3
sp|Q04828	2	11	11	0	7	9	6	5
tr|B4DK69	4	10	2	2	6	8	6	5
tr|A0A0A0	7	8	2	2	4	6	3	2
sp|P15121	5	5	5	5	2	2	2	3
sp|O00116	3	10	10	10	10	10	10	9
sp|Q9H553	2	1	1	1	1	1	1	1
tr|E9PK47|	6	1	1	1	0	0	0	1
sp|P30533	1	2	2	2	2	1	1	1
sp|P12814	18	36	36	23	13	16	19	19
sp|O43707	14	35	22	22	11	15	18	18
tr|A0A1B0	16	5	5	5	4	2	4	3
tr|R4GMT0	2	1	1	1	1	1	0	0
tr|A0A1W2	11	1	1	1	0	0	1	0
sp|P06733	15	23	23	23	12	14	16	14
sp|Q16706	1	2	2	2	2	1	2	2
tr|H0YKM7	7	1	1	1	0	0	0	0
tr|M0R0Y2	10	6	6	6	3	4	5	4
sp|P40222	1	1	1	1	1	1	0	1
sp|P21397	4	4	4	4	4	4	4	4
sp|Q12904	3	4	4	4	4	4	2	2
sp|Q13155	3	3	3	3	2	3	2	2
tr|A6NKB8	5	6	6	6	3	2	4	4
sp|Q86SJ2	1	2	2	2	2	2	2	2
tr|E9PG40	14	8	8	7	6	7	7	8

sp|Q06481	8	7	6	6	5	6	7	6
sp|Q9Y679	3	1	1	1	1	0	1	1
tr|A0A1W2	15	1	1	1	1	1	1	0
sp|P04083	3	23	23	23	22	21	22	22
sp|Q9UJ72	1	1	1	1	1	1	1	0
sp|P50995	6	7	7	7	5	5	5	4
sp|P07355	25	25	25	25	25	25	24	24
sp|P12429	6	12	12	12	8	7	11	10
sp|P09525	4	11	11	11	6	11	10	10
sp|P08758	5	11	11	11	9	8	8	8
sp|P20073	2	11	11	11	7	10	6	8
sp|P13928	10	10	10	10	8	8	7	8
sp|P46013	2	15	15	15	5	9	12	11
tr|A0A140	2	2	2	2	1	1	1	2
sp|Q10567	5	7	7	3	2	2	4	4
tr|H7C1E4	3	2	2	2	1	0	1	1
sp|O95782	7	4	4	4	0	3	2	3
tr|A0A087	9	6	2	2	2	3	3	3
tr|A0A087	10	3	3	3	2	2	3	2
sp|O14617	5	1	1	1	0	1	0	0
sp|Q9Y2T2	1	1	1	1	0	1	0	0
sp|Q92572	2	2	2	2	1	2	1	1
sp|P04114	1	7	5	5	6	6	5	7
tr|B0YIW2	2	1	1	1	1	1	1	1
tr|J3KQL8|	3	2	2	2	2	2	2	2
tr|G3V1B6	4	2	2	2	2	0	2	2
sp|Q9BZZ5	9	4	4	4	0	0	1	0
sp|Q07812	8	3	3	3	1	1	1	1
sp|O95831	7	9	9	9	9	9	9	8
tr|S4R3H4	9	8	8	8	6	7	7	6
tr|A0A087	4	1	1	1	0	0	0	1
sp|P54136	4	8	8	8	5	5	4	3
sp|P08243	8	5	5	5	4	5	3	4
sp|O43776	7	6	6	6	3	4	4	4
sp|P17174	2	4	4	4	0	0	1	1
sp|P00505	2	13	13	13	11	12	12	11
sp|P14868	7	20	20	20	15	12	11	13
sp|Q6PI48	1	2	2	2	2	2	2	0
sp|Q12797	17	4	4	4	4	4	4	4
tr|B1AKZ5	3	1	1	1	1	1	1	1
sp|Q8WWI	9	2	2	2	1	0	1	2
tr|B5MCN(	7	3	3	3	3	2	2	3
tr|F5H6I7|	3	2	2	2	2	2	0	1
tr|Q5QNZ2	2	7	7	7	6	6	6	5
sp|P00846	1	1	1	1	1	1	1	1
sp|P25705	9	24	24	24	22	21	16	18
sp|P06576	5	22	22	22	21	21	16	17
sp|O75947	3	8	8	8	8	6	7	6
sp|P30049	1	2	2	2	2	2	2	2
sp|P56385	1	2	2	2	2	2	2	2

sp|P56134	7	2	2	2	1	2	2	1
tr|E9PN17	2	3	3	3	2	3	2	3
sp|P36542	2	10	10	10	10	10	8	9
sp|P48047	4	7	7	7	5	7	6	4
sp|P18859	3	4	4	4	4	4	4	4
sp|Q9NVI7	10	6	6	6	5	5	5	5
tr|E9PJR8|	6	1	1	1	1	1	1	1
sp|P28288	3	2	2	2	2	2	2	2
sp|P61221	4	4	4	4	2	1	1	1
tr|H0YGW7	3	2	2	2	2	2	0	1
sp|Q9UG63	2	1	1	1	1	1	1	1
sp|Q9NUQ	2	1	1	1	1	1	0	0
sp|P53396	5	22	22	22	7	9	13	13
sp|P17858	2	4	2	2	4	4	3	3
sp|P08237	4	4	3	3	4	3	0	0
sp|Q01813	8	13	13	11	12	11	10	9
sp|O76031	3	5	5	5	5	4	4	3
tr|M0R208	3	3	3	3	3	3	3	3
sp|P46063	1	3	3	3	1	2	1	2
sp|Q08211	2	26	26	26	19	19	18	16
tr|F1T0B3|	5	10	10	10	2	5	4	3
sp|Q9NVP1	2	9	9	9	5	6	6	7
tr|I3L0H8|	13	2	2	2	1	1	0	1
tr|G3V529	6	6	6	6	2	3	5	4
sp|O00148	19	8	1	1	6	7	3	3
tr|A0A0D9	21	14	13	13	10	12	7	9
tr|A0A0A0	3	1	1	1	0	0	0	0
sp|Q8TDD1	3	6	6	6	4	3	4	4
sp|Q8IYB8	1	2	2	2	2	2	2	2
tr|J3KRJ2|J	10	1	1	1	0	0	1	1
sp|O95816	1	1	1	1	1	0	1	1
sp|O95817	1	3	3	3	2	2	2	1
tr|A0A0C4I	11	2	2	2	1	1	0	0
sp|O75531	1	1	1	1	1	1	1	1
sp|P98160	1	1	1	1	0	1	0	0
sp|Q7L1Q6	6	4	4	4	1	1	1	1
tr|Q75MG1	11	3	3	3	0	0	1	1
sp|P35613	9	8	8	8	7	7	7	7
sp|P51572	6	6	6	6	3	3	4	4
sp|Q16611	2	1	1	1	1	1	0	1
tr|E9PJA7|	11	4	4	4	4	4	4	4
tr|A0A087Y	4	3	3	3	3	2	3	3
sp|P61769	3	3	3	3	3	3	2	2
sp|Q13425	1	1	1	1	1	1	1	1
sp|P42025	1	1	1	1	0	1	0	0
sp|Q53H82	1	3	3	3	2	2	2	1
sp|P55957	4	3	3	3	0	0	2	0
sp|Q13057	6	3	3	3	2	3	2	1
sp|P07814	3	22	22	22	20	17	17	11
sp|P13995	3	2	2	2	2	1	1	0

sp|P31939	5	13	13	13	6	8	7	7
sp|Q8NFC6	1	1	1	1	1	1	1	1
tr|A0A087\	7	2	2	2	1	0	1	2
tr|F5GXW6	2	1	1	1	1	1	1	1
sp|Q9UHR4	1	4	4	4	3	3	4	4
sp|P54687	5	1	1	1	0	0	0	0
sp|Q9P287	4	2	2	2	1	2	1	0
sp|Q8WY2	1	2	2	2	1	2	2	2
sp|Q9NRL2	2	2	2	2	0	1	0	0
sp|Q96CX2	1	4	4	4	1	0	0	1
tr|H3BSS5|	2	1	1	1	1	1	0	1
tr|J3QRS9|	12	4	4	4	1	1	1	3
tr|F5H2F4|	3	15	15	15	14	13	12	11
sp|O75844	1	4	4	4	4	4	4	4
tr|E9PKA7|	3	1	1	1	1	1	1	1
tr|F8VPD4	5	20	20	20	14	18	12	11
tr|A0A087\	3	2	2	2	1	0	1	2
sp|P22223	2	4	4	4	4	4	4	4
sp|Q99653	5	3	3	3	2	2	2	3
sp|Q8IU85	2	1	1	1	1	0	0	1
sp|Q9UJS0	5	17	17	17	14	15	14	13
sp|Q6NUK1	2	6	6	6	5	6	5	5
tr|B4E2Q0	14	7	7	7	6	6	7	6
sp|Q9HB71	2	6	6	6	5	5	4	3
sp|P27824	13	21	21	21	21	20	16	17
tr|K7EM73	9	4	4	4	3	3	4	3
sp|P07384	11	8	8	8	2	5	4	5
sp|P17655	2	6	6	6	5	6	4	3
tr|E7EQ12	24	5	5	5	0	0	0	1
tr|B4DDF4	13	8	8	8	2	1	3	3
sp|P27797	3	15	15	15	15	15	13	14
sp|O43852	16	3	3	3	1	0	2	2
sp|P10644	4	3	3	3	3	1	2	3
tr|H7C1L0|	3	1	1	1	1	1	1	1
tr|G3V153	4	4	4	4	2	2	2	3
sp|P00918	3	4	4	4	2	3	3	4
sp|P16152	5	12	12	12	2	2	5	5
sp|O75976	3	8	8	8	6	5	7	4
sp|P50416	6	9	9	9	8	9	9	9
sp|P23786	6	6	6	6	5	4	5	4
tr|D6REM4	10	3	3	3	2	3	2	1
tr|E7EU96	5	2	2	2	1	1	1	0
tr|Q5SRQ3	7	2	2	2	2	2	1	1
tr|F5GX99	9	2	2	2	2	1	1	1
sp|Q9BXW	2	2	2	2	1	1	0	1
sp|P04040	1	9	9	9	5	6	5	7
sp|P21964	6	6	6	6	3	3	3	1
sp|P35221	30	21	21	21	13	12	12	15
tr|B4DGU4	2	7	5	5	4	5	6	6
tr|C9JZR2|	36	14	14	14	11	13	11	11

tr|A0A1B0	13	6	6	6	3	3	5	4
sp|P20645	7	3	3	3	3	3	3	2
sp|P11717	1	8	8	8	5	4	6	6
sp|Q03135	5	8	8	8	8	8	8	8
sp|Q03701	1	2	2	2	1	1	2	2
sp|Q6YHK3	4	3	3	3	3	3	3	3
tr|H7C543	5	3	3	3	3	2	3	2
tr|A0A0C4	10	2	2	2	2	2	2	2
tr|H0YD13	29	6	6	6	5	6	4	4
tr|E9PNW4	4	3	3	3	3	3	2	3
sp|P27701	3	3	3	3	3	2	3	3
sp|P48960	3	3	3	3	3	2	3	3
tr|I3L1N9|	2	2	2	2	2	2	2	2
tr|B3KY94|	4	2	2	2	1	1	1	1
sp|Q8N163	6	4	4	4	3	3	2	1
sp|P60953	3	5	4	4	2	4	4	4
sp|Q99459	1	4	4	4	2	1	0	2
sp|Q9NX58	2	2	2	2	1	1	2	2
sp|P62633	6	5	5	1	1	2	3	2
tr|Q5JVD3	3	1	1	1	0	0	0	0
sp|Q7Z7K6	3	2	2	2	1	2	1	2
tr|A0A1B0	13	2	2	2	2	2	2	2
tr|M0R1L7	3	1	1	1	1	1	1	1
sp|Q9H444	2	5	5	5	5	5	5	5
sp|O00299	1	9	9	9	4	7	6	6
sp|Q8WWI	3	3	3	3	2	2	3	3
sp|Q8IWA5	3	1	1	1	0	0	0	1
tr|A0A0C4	2	1	1	1	1	1	1	1
tr|H7BZN1	9	1	1	1	1	1	1	1
tr|A0A087	5	1	1	1	1	1	1	1
sp|Q13185	8	6	6	6	3	3	3	4
tr|F5GWX5	7	2	2	2	2	2	1	1
tr|B4DJV2|	20	9	9	8	8	9	9	9
tr|A0A087	9	46	46	35	30	32	30	26
sp|P09496	7	3	3	3	2	1	2	2
sp|P09497	2	2	2	2	1	1	2	2
tr|K7EP40|	7	2	2	2	2	2	2	1
sp|O95832	1	3	3	3	3	3	2	3
sp|P56749	1	1	1	1	1	1	1	1
sp|O14493	4	2	2	2	2	2	2	2
sp|O43809	3	3	3	3	1	3	3	2
tr|F8WJN3	5	4	4	4	4	3	2	2
tr|J3QT54|	7	1	1	1	0	1	0	0
tr|A0A0A0	5	1	1	1	0	1	1	1
sp|O96005	5	5	5	5	4	1	3	5
tr|G5E9Z2|	3	3	3	3	2	3	3	3
tr|I3L2B0|	3	3	3	3	0	1	0	1
sp|Q14019	2	5	5	5	2	2	3	4
tr|Q9NQP5	3	1	1	1	1	1	1	1
sp|P53621	2	10	10	10	7	5	7	4

sp|P53618	2	5	5	5	2	2	2	2
sp|P35606	7	10	10	10	9	7	6	6
sp|P48444	5	7	7	7	4	5	4	4
tr|M0QXB4	5	3	3	3	3	3	1	1
sp|Q9Y678	2	4	4	4	1	1	1	0
sp|P23528	9	14	14	13	11	10	8	8
tr|Q68DW	3	1	1	1	0	0	1	0
sp|Q96A33	2	5	5	5	5	5	3	3
sp|Q96ER9	2	1	1	1	1	1	1	1
tr|C9JQ41|	3	1	1	1	1	1	1	1
tr|H0YG79	2	1	1	1	0	1	0	1
sp|Q9Y6H1	2	3	3	3	3	2	3	3
sp|O75534	7	5	5	5	3	3	3	3
sp|Q9UMD	3	11	11	11	6	9	9	8
sp|Q07021	3	8	8	8	7	6	6	4
sp|Q15021	1	1	1	1	0	0	0	0
tr|E9PHA2	6	4	4	4	1	2	1	2
sp|Q9BPX3	2	3	3	3	1	1	1	1
sp|Q96JB2	2	1	1	1	1	0	0	1
sp|Q9NZB2	7	8	8	8	5	7	6	6
tr|C9JFE4|	17	2	2	2	1	0	1	1
tr|D6RAX7	5	2	2	2	0	0	0	1
tr|E9PGT6	4	2	2	2	1	1	1	1
sp|O75131	7	10	10	9	10	10	8	8
tr|E7ENV7	8	2	1	1	2	2	2	2
sp|O75367	5	10	10	9	7	7	8	8
tr|I3L1U7|	6	1	1	1	1	1	1	1
sp|Q9BR76	1	2	2	2	1	0	1	2
sp|Q9ULV4	11	6	6	6	3	4	5	4
sp|Q92828	1	1	1	1	1	1	1	1
tr|Q5SZC9	2	1	1	1	0	0	0	0
sp|P78310	5	4	4	4	3	3	3	3
sp|P12532	10	3	3	3	0	2	1	0
sp|P46109	1	2	2	2	0	0	1	2
tr|X6RI56|	2	1	1	1	0	0	1	0
tr|Q5SQP8	4	2	2	2	2	2	1	0
sp|P17812	2	5	5	5	4	5	3	3
sp|Q9H5V8	3	3	3	3	2	2	1	2
tr|G5EA30	7	1	1	1	0	1	0	0
tr|K4DI93|	4	1	1	1	0	0	0	0
sp|Q86VP6	5	7	7	7	0	0	2	3
tr|A0A024	52	5	4	4	3	4	3	3
sp|Q00534	1	6	6	5	4	3	4	3
sp|P01040	1	1	1	1	0	0	0	0
sp|P04080	2	3	3	3	2	1	2	2
sp|P01034	1	1	1	1	1	1	1	0
sp|P21291	4	6	6	6	2	3	3	4
sp|Q9UHD	6	2	2	2	2	1	2	2
sp|Q9H1C7	1	1	1	1	1	1	1	1
sp|Q9NZV1	3	3	3	3	3	2	2	3

tr|H0YFA4	3	1	1	1	0	0	1	0
sp|Q6UXH1	4	1	1	1	1	1	1	1
sp|P00167	3	1	1	1	1	1	1	1
tr|H3BUX2	4	2	2	2	2	2	2	1
sp|P31930	1	14	14	13	12	13	10	8
sp|P22695	7	13	13	13	10	9	10	10
sp|P07919	2	3	3	3	3	1	2	2
sp|P14927	4	2	2	2	2	2	2	2
sp|Q9UDW	1	1	1	1	1	1	1	1
sp|P47985	2	5	5	5	3	4	3	4
tr|C9JFR7|	3	2	2	2	2	2	1	1
sp|Q9Y2R0	2	3	3	3	3	3	3	2
sp|Q7KZN9	2	1	1	1	1	1	1	1
tr|C9J8T6|	2	1	1	1	1	1	1	1
sp|P00403	1	3	3	3	3	3	3	3
sp|P13073	6	4	4	4	4	3	4	4
tr|H3BNX8	4	2	2	2	2	2	2	2
sp|P10606	1	5	5	5	5	5	4	5
sp|P14854	1	4	4	4	4	3	3	2
sp|P09669	1	3	3	3	3	3	1	1
tr|D6RIE3|	5	2	2	2	2	2	2	2
sp|O14548	3	2	2	2	2	1	1	2
tr|D6R9Z7	2	2	2	2	2	2	2	2
sp|O00483	1	2	2	2	2	2	2	2
sp|P08574	1	2	2	2	2	2	1	1
sp|P53701	1	4	4	4	4	4	4	3
sp|Q14204	2	41	41	41	28	27	22	19
sp|Q13409	18	3	3	3	0	0	0	0
tr|J3KRI4|J	4	3	3	3	2	2	2	1
sp|Q7L576	14	4	4	4	2	1	1	2
sp|Q07065	1	22	22	22	19	19	19	19
sp|P28838	3	3	3	3	1	1	1	1
sp|O00154	8	2	2	2	0	1	0	0
sp|Q96KP4	4	5	5	5	2	1	4	3
tr|Q5JUV6	3	2	2	2	0	0	0	0
tr|A0A286Y	14	10	10	10	9	9	7	8
tr|K7EQ02	5	3	3	3	2	2	2	1
sp|Q5BKZ1	4	2	2	2	2	2	2	2
sp|Q9H773	1	2	2	2	0	0	0	1
tr|A0A087Y	3	2	2	2	1	1	2	2
sp|P30046	6	4	4	4	1	4	3	3
tr|A0A0A0	3	2	2	2	1	1	2	2
sp|Q9Y394	5	3	3	3	3	3	2	2
tr|Q3LIE7|	4	5	5	5	5	5	5	5
sp|Q13011	3	4	4	3	4	3	2	3
sp|P30038	4	2	2	2	2	1	1	1
sp|P54886	2	15	15	15	13	12	13	12
sp|O43583	1	1	1	1	0	0	0	0
tr|J3KP30|	3	3	3	3	1	3	3	3
tr|G3V158	3	1	1	1	0	0	0	0

tr|A0A0C4I	9	5	5	5	1	0	2	2
tr|E5RGY0	4	1	1	1	1	1	1	1
tr|I3L1T3|I	2	1	1	1	1	0	1	0
sp|Q02487	2	1	1	1	0	0	0	1
sp|Q14574	3	4	4	4	2	4	3	3
sp|Q14126	1	12	12	12	9	11	11	10
sp|P32926	1	12	12	12	11	10	11	11
sp|P15924	3	46	46	46	37	38	35	37
sp|P60981	3	8	7	7	3	3	3	3
sp|Q9Y295	1	2	2	2	1	1	2	2
sp|P09622	7	5	5	5	5	3	3	5
sp|P10515	3	5	5	5	4	4	4	4
sp|P36957	6	8	8	8	6	8	8	8
tr|G3V1D3	4	1	1	1	0	0	0	0
sp|O14672	1	2	2	2	1	1	2	2
tr|F8WC54	4	5	5	5	4	4	4	4
tr|Q05BS2	3	1	1	1	1	1	1	1
sp|Q16531	7	9	9	9	6	5	4	4
sp|Q6UX65	1	1	1	1	1	0	0	0
tr|B0QYD3	6	5	5	5	3	3	1	1
tr|B1AHB1	4	5	5	5	2	4	2	0
tr|E9PHA6	4	3	3	3	3	3	2	0
sp|P52701	6	4	4	4	3	3	2	1
tr|E5RFF9|	2	1	1	1	0	0	1	0
tr|H0Y8E6|	2	12	12	12	5	3	1	1
sp|P25205	4	8	8	8	6	5	3	4
sp|P33991	3	11	11	11	8	10	6	4
sp|Q14566	1	8	8	8	5	5	5	4
sp|P33993	4	16	16	16	12	14	9	4
sp|P11387	4	14	14	14	8	12	12	12
sp|P11388	8	14	14	14	11	11	12	12
tr|G3V5Q1	9	5	5	5	0	2	1	2
sp|P78527	2	56	56	56	37	39	30	32
sp|O95602	2	2	2	2	1	1	2	2
sp|O15446	2	2	2	2	0	2	2	2
sp|P24928	2	1	1	1	0	0	0	0
tr|K7EMH3	2	1	1	1	1	1	1	1
tr|C9J2Y9|	4	2	2	2	2	2	2	1
sp|O15160	5	2	2	2	0	0	1	1
tr|A0A087Y	3	1	1	1	0	0	1	1
sp|P31689	2	3	3	3	1	2	2	1
sp|O60884	3	4	4	4	4	3	3	3
sp|Q96EY1	3	3	3	3	2	3	2	3
sp|P25685	9	4	4	4	1	3	2	2
sp|Q9UBS4	2	4	4	4	4	3	4	4
tr|V9GY70	5	1	1	1	1	0	1	1
sp|Q9NVH	3	4	4	4	2	2	2	2
tr|H0YA63	2	1	1	1	0	0	0	0
tr|X6R9L0|	2	3	3	3	1	3	3	3
sp|Q9H3Z4	1	1	1	1	1	1	1	1

sp|Q99615	13	4	4	4	2	2	3	2
sp|Q8WXX	1	1	1	1	0	0	0	0
tr|Q5QPK2	4	3	3	3	3	3	3	3
sp|Q9P2X0	2	2	2	2	2	1	2	2
tr|A0A0C4I	5	8	8	8	7	7	5	5
sp|P04843	3	24	24	24	20	22	19	19
sp|P04844	6	11	11	11	10	10	10	8
tr|F5GXX5	4	4	4	4	4	3	4	4
sp|P46977	5	5	5	5	4	5	4	4
sp|Q8TCJ2	1	2	2	2	2	2	1	2
sp|Q9Y673	2	1	1	1	1	0	0	1
tr|A0A087	5	2	2	2	1	2	2	2
tr|H0YCK3	6	3	3	3	2	2	1	1
tr|A8MY26	4	1	1	1	1	1	1	1
tr|H0Y5J4|	8	3	3	3	3	3	3	3
sp|Q02750	2	1	1	1	0	0	0	0
sp|P36507	1	1	1	1	0	0	0	0
sp|P46734	6	3	3	3	1	3	1	1
tr|E7EX90|	9	2	2	2	2	1	2	2
tr|G8JLD5|	13	3	3	3	0	0	1	1
tr|E5KLJ9|	8	3	3	3	3	3	2	0
tr|A0A075I	3	1	1	1	1	1	1	1
sp|P63167	4	3	1	1	2	2	1	1
sp|Q96FJ2	1	3	3	1	2	2	1	1
sp|Q9NP97	8	3	3	3	0	0	0	0
tr|Q6P0N6	11	1	1	1	0	0	0	0
sp|Q14118	1	2	2	2	2	2	2	2
sp|P49792	16	22	22	21	19	21	20	20
sp|Q14258	1	7	7	7	2	2	2	2
tr|H3BUD0	4	1	1	1	1	1	1	1
sp|Q7Z6Z7	4	5	5	5	1	2	3	2
tr|F8WCD0	2	3	3	3	1	3	3	3
tr|Q5JVY0|	2	1	1	1	1	1	1	1
sp|Q9C037	3	2	2	2	2	2	1	1
tr|A0A087	5	3	3	3	2	2	1	1
sp|Q96C19	5	7	7	7	3	6	6	6
sp|O43854	2	2	2	2	2	1	2	2
tr|C9J2Z4|	3	2	2	1	0	1	2	2
sp|Q9H223	2	6	6	5	5	3	4	2
sp|Q15717	4	7	7	7	6	3	5	4
tr|H0YKF0|	8	6	6	6	5	5	5	6
sp|P38117	3	10	10	10	7	8	9	8
sp|Q05639	1	9	1	1	8	8	8	7
sp|P24534	4	5	5	5	5	4	5	4
tr|E9PK01|	22	8	8	1	6	6	4	7
sp|P29692	3	8	1	1	5	5	3	7
sp|P26641	2	16	16	16	10	12	11	11
sp|P13639	1	34	34	33	30	28	23	22
tr|C9IZ01|	4	5	5	5	5	4	4	3
sp|P43897	7	3	3	3	3	2	2	3

sp|P49411	2	19	19	19	15	16	14	15
sp|Q9BW6	1	1	1	1	1	1	1	1
tr|A0A0A0	4	1	1	1	1	1	1	1
tr|F5H2T0|	2	1	1	1	1	1	1	1
sp|P50402	2	5	5	5	5	5	3	4
sp|O94919	1	3	3	3	3	3	3	3
tr|M0QYEC	4	1	1	1	0	0	0	0
sp|Q9NZ08	4	7	7	7	6	5	7	7
tr|E7ER77|	4	4	4	4	4	4	4	4
sp|P30040	3	7	7	6	6	6	6	6
sp|Q9BS26	1	6	6	6	5	5	5	6
tr|H0Y621	8	3	3	3	3	3	3	2
sp|P14625	7	28	26	26	26	25	23	22
sp|O60869	3	2	2	2	0	0	0	0
sp|P84090	2	3	3	3	1	3	3	3
sp|P42126	4	5	5	5	4	5	5	5
tr|A0A0C4I	7	4	4	4	3	3	3	2
sp|P30084	1	8	8	8	7	8	7	7
sp|P98172	1	1	1	1	1	1	1	1
sp|P00533	23	30	30	30	26	25	27	25
tr|G3V2V8	8	3	3	3	2	3	3	2
tr|B5MCA4	3	6	6	6	5	5	5	5
tr|H0YBR2	8	2	2	2	2	1	0	1
sp|P07099	1	6	6	6	5	5	5	4
sp|Q99808	2	1	1	1	1	0	1	1
tr|M0R1Y2	5	2	2	2	2	2	1	2
sp|Q8N766	4	3	3	3	2	0	1	0
sp|Q15006	2	3	3	3	2	2	3	2
tr|S4R3U9	3	1	1	1	0	0	1	0
sp|O43402	3	2	2	2	2	1	1	1
sp|O75477	4	5	2	2	5	5	4	5
tr|E5RHW4	3	6	6	3	5	6	5	6
sp|Q96HE7	7	12	12	12	6	11	11	11
tr|A0A096I	12	2	2	2	2	2	2	0
sp|Q9H501	1	1	1	1	0	1	1	1
sp|Q92506	1	1	1	1	1	1	1	1
sp|P60842	24	20	20	16	10	19	13	11
sp|P38919	2	8	4	4	4	5	4	6
sp|P15170	6	7	7	7	3	5	4	3
tr|B7Z7P8|	6	6	6	6	0	0	1	2
tr|D6RBD7	6	2	2	2	2	2	1	1
tr|K7EM18	4	2	2	2	0	0	0	0
sp|P47813	4	3	3	3	0	0	1	2
sp|P05198	3	5	5	5	2	2	4	3
sp|P20042	1	7	7	7	3	5	4	4
sp|P41091	5	8	8	8	3	4	4	4
tr|C9IZE1|(	7	2	2	2	0	0	1	0
sp|Q14152	2	11	11	11	2	3	2	4
sp|P55884	4	8	8	8	4	4	3	2
sp|Q99613	5	7	7	7	4	2	3	3

sp|O15371	7	9	9	9	5	5	4	3
sp|P60228	6	7	7	7	6	6	6	5
sp|O00303	2	3	3	3	2	2	2	2
tr|K7EL20|	5	4	4	4	2	0	0	1
tr|A0A087'	9	4	4	4	3	3	3	2
sp|Q13347	2	10	10	10	6	6	6	5
tr|H0YGJ7|	5	2	2	2	0	1	2	2
tr|K7ES31|	4	1	1	1	0	1	1	1
tr|B0QY89	7	7	7	7	2	1	4	4
tr|E9PRY0|	5	1	1	1	0	0	1	0
sp|Q04637	28	16	16	16	9	10	7	10
tr|H0YCH5	6	2	2	2	1	0	1	0
tr|E7EX17|	4	4	4	4	0	0	2	3
sp|Q15056	2	6	6	6	1	3	3	4
sp|P55010	5	2	2	2	1	0	0	1
tr|I3L397|I	9	8	8	8	6	5	5	4
tr|A0A087'	2	5	5	5	2	3	2	1
sp|P56537	7	5	5	5	4	4	2	2
tr|R4GNH9	6	1	1	1	1	1	1	1
sp|Q5RKV6	1	4	4	4	3	3	1	3
sp|Q13868	2	1	1	1	0	0	0	1
sp|Q15024	1	2	2	2	1	2	1	1
tr|F2Z2C0|	4	2	2	2	2	1	0	0
sp|O14980	10	9	9	9	2	1	3	1
sp|P55060	4	12	12	12	5	4	6	7
sp|Q9HAV4	3	2	2	2	2	2	1	1
sp|Q9BSJ8	2	11	11	11	8	10	9	8
tr|E7EQR4	8	14	9	9	6	6	9	7
sp|Q9Y5B9	4	10	10	10	5	6	5	5
sp|Q08945	4	10	10	10	9	8	8	7
sp|P52907	1	7	7	6	6	7	7	6
sp|P47755	5	6	5	5	3	3	3	3
sp|P47756	5	10	10	10	6	5	7	7
sp|Q96AE4	4	12	11	11	0	1	3	4
tr|A0A087'	8	14	14	13	5	5	4	6
sp|Q96I24	2	8	7	7	5	6	4	2
sp|P14324	6	3	3	3	1	1	1	2
sp|Q96CS3	1	6	6	6	6	5	6	4
sp|Q16658	4	19	19	19	13	13	13	12
tr|A0A0U1	5	49	49	49	36	31	31	28
sp|Q01469	3	4	4	4	0	0	2	2
tr|E9PNW8	2	3	3	3	3	3	3	3
tr|J3QRD1	22	7	7	7	4	5	6	4
tr|G3V1L6	5	4	4	4	3	2	2	3
sp|P02794	9	6	6	6	6	5	6	6
sp|Q4ZHG4	2	1	1	1	0	0	0	0
sp|P21333	8	88	88	80	69	69	66	69
sp|O75369	13	48	43	41	30	32	35	37
sp|Q8WUF	3	2	2	2	1	0	1	0
sp|P39748	4	6	6	6	4	5	5	3

sp|P30043	3	5	5	5	0	1	2	2
sp|O75955	13	5	5	5	5	5	5	5
tr|J3QLD9|	4	6	6	6	5	5	5	5
tr|J3KNW4	3	2	2	2	2	0	2	2
tr|B4DXZ6	20	6	6	6	4	4	4	3
tr|H7C585	5	2	2	2	2	2	2	2
sp|O60353	2	1	1	1	1	1	1	1
sp|P04075	10	23	23	17	13	14	17	15
sp|P09972	6	8	2	2	4	3	7	4
sp|P07954	2	11	11	11	9	9	9	9
tr|S4R445|	4	1	1	1	1	1	1	1
tr|E9PN19	9	5	5	5	4	4	4	4
sp|P09382	2	8	8	8	7	8	8	7
sp|P17931	2	2	2	2	0	1	0	0
sp|Q08380	5	4	4	4	4	3	4	2
sp|P60520	8	2	2	2	2	2	2	2
sp|O75223	7	3	3	3	0	1	2	3
sp|Q16666	16	9	9	9	7	7	8	7
sp|Q9H488	2	2	2	2	2	2	2	2
;tr|A0A0A0	10	12	12	12	11	12	9	5
sp|Q12789	2	1	1	1	1	1	0	0
sp|P78347	5	8	8	8	7	7	4	5
sp|O60763	2	2	2	2	0	0	1	2
tr|G3V4P8	2	1	1	1	0	0	0	0
sp|Q9NZM	1	2	2	2	0	0	1	1
tr|G3V5E4	3	1	1	1	0	0	0	0
tr|D6RAY7	10	5	5	5	0	1	1	2
sp|P11413	6	15	15	15	8	8	6	6
tr|A0A0A0	17	10	10	10	4	4	5	5
tr|K7ELL7|	7	16	16	16	16	15	15	15
sp|P00367	4	18	18	18	18	18	15	16
tr|E1CEI4|	4	2	2	2	0	0	0	0
sp|P48507	2	1	1	1	0	0	0	0
sp|Q9BQ67	2	3	3	3	0	0	0	0
sp|O94925	7	10	10	10	9	10	8	8
sp|Q06210	2	4	4	4	4	4	3	4
sp|P47897	10	6	6	2	5	5	4	3
sp|O76003	1	2	2	2	0	0	1	1
sp|Q86SX6	1	1	1	1	0	1	1	1
sp|P00390	7	4	4	4	1	2	3	3
tr|E9PFN5	5	3	3	3	1	1	1	1
sp|P78417	5	6	6	6	0	1	3	2
sp|P09211	4	9	9	9	3	4	5	5
sp|P04406	4	15	15	15	12	12	12	11
sp|Q53EU6	1	1	1	1	1	1	1	1
sp|P43304	3	8	8	8	7	8	7	7
tr|A0A1W2	3	2	2	2	2	1	2	2
sp|P41250	3	5	5	5	3	2	2	4
sp|P49841	2	1	1	1	1	1	1	1
tr|G5E9W8	5	1	1	1	0	0	0	1

tr|F6TLX2|	6	3	3	3	0	0	3	2
tr|U3KQ56	2	1	1	1	0	1	1	1
sp|P35052	7	7	7	7	5	6	7	6
sp|P49915	2	7	7	7	4	4	3	4
sp|Q92896	6	6	6	6	5	5	4	4
tr|F8W785	2	3	3	3	3	2	1	1
sp|Q9H4A(	2	2	2	2	1	1	1	1
sp|Q9H8Y{	3	4	4	4	2	2	3	4
sp|Q9H3P7	1	1	1	1	1	1	1	0
tr|F6RU00	5	1	1	1	1	1	1	1
sp|Q08379	3	4	4	4	2	3	4	3
sp|Q7Z5G4	2	1	1	1	1	1	1	1
tr|D6R9J9|	9	1	1	1	0	0	0	1
sp|Q9Y653	7	2	2	2	2	1	0	1
tr|H0Y8R1	5	2	2	2	2	2	2	2
sp|Q9H3K2	1	2	2	2	1	1	0	0
sp|Q9HAV7	1	5	5	5	4	5	3	3
sp|Q9UIJ7-	3	1	1	1	1	1	1	1
sp|P01116	7	2	2	2	2	1	2	2
tr|B5MDF5	7	7	7	7	5	4	6	6
tr|C9J931|	2	1	1	1	1	1	1	0
tr|H0Y5E8|	3	1	1	1	1	1	1	1
sp|P52735	3	2	2	2	1	2	1	1
sp|P63096	4	8	4	4	5	6	5	3
sp|P04899	6	8	4	4	6	6	6	5
sp|Q9UBI6	1	5	5	5	5	4	3	4
sp|P62873	12	7	4	4	6	5	5	6
sp|P62879	9	9	9	6	7	6	7	7
sp|P08754	18	8	8	4	6	6	6	4
sp|P50148	2	2	2	1	2	2	2	2
sp|P63092	10	4	3	3	4	4	4	4
sp|P29992	2	2	1	1	2	2	2	2
sp|Q14344	2	1	1	1	1	0	0	1
sp|P63244	22	14	14	14	10	12	11	7
sp|Q9BVP2	2	2	2	2	1	2	2	2
tr|H0Y2M6	3	1	1	1	0	0	0	1
sp|Q9NY12	2	2	2	2	2	2	2	2
sp|Q9NX24	4	4	4	4	3	3	3	2
sp|O60832	7	6	6	6	4	6	5	5
tr|Q5T3Q7	3	10	10	10	8	9	9	7
sp|Q0VDF9	1	1	1	1	1	1	1	1
tr|A0A0G2	5	29	29	15	18	18	19	18
sp|P34932	4	19	18	18	6	10	11	13
sp|P17066	2	10	1	1	9	9	9	9
sp|P11142	15	28	24	18	22	21	20	22
sp|O75506	1	1	1	1	1	1	1	1
sp|Q92598	10	21	21	20	9	13	13	12
sp|Q12931	4	13	13	13	11	10	9	9
sp|P04792	3	14	14	14	12	13	13	13
sp|P07900	6	34	19	19	23	25	27	25

sp|P08238	2	39	39	19	31	30	34	31
sp|P54652	1	11	1	1	8	9	9	10
tr|J3KT51|.	5	4	4	4	0	0	2	2
tr|H3BMT(	10	2	2	2	0	0	0	0
sp|P30519	9	4	4	4	3	3	1	2
tr|Q5THN1	2	1	1	1	0	0	1	1
;tr|G3V1N2	4	5	5	5	5	5	3	3
sp|P68871	2	3	2	2	1	1	1	3
;tr|D6RAR4	3	3	3	3	3	3	2	2
tr|I3L1P5|I	3	1	1	1	1	1	1	1
sp|P51858	3	5	5	5	0	0	1	2
tr|B0QZK4	6	6	6	6	3	2	4	3
tr|D6R9P3	7	4	4	4	1	2	3	3
sp|Q13151	1	5	5	5	5	4	4	3
tr|F8W6I7	10	11	10	10	2	4	4	5
sp|P51991	3	8	8	8	6	4	3	4
tr|H0YA96	10	7	7	7	2	1	2	3
tr|A0A087\	4	3	3	3	1	2	1	1
sp|P52597	2	11	11	9	9	9	8	8
tr|E9PCY7|	23	10	8	4	10	10	9	9
sp|P55795	1	9	4	4	8	8	8	8
sp|P31942	6	7	7	7	7	7	4	4
sp|P61978	6	17	17	17	10	12	7	6
sp|P14866	14	14	14	8	10	8	3	4
sp|P52272	12	23	23	23	19	19	18	15
sp|O60506	7	16	16	11	10	10	9	8
sp|O43390	5	14	9	9	12	13	10	12
sp|Q00839	16	24	24	24	18	21	16	16
tr|M0R3F1	9	3	3	3	2	2	2	3
sp|Q1KMD	2	15	15	15	12	10	8	10
sp|P22626	3	16	16	15	7	7	10	12
tr|G3V4W(	30	15	15	15	10	12	14	12
tr|E9PB90|	2	9	8	8	7	7	7	8
sp|P19367	8	14	14	13	12	14	14	12
sp|P30825	1	2	2	2	2	2	2	2
tr|Q5T7C4	3	5	5	4	3	3	3	1
tr|D6R9A6	2	2	1	1	0	1	0	1
tr|E9PES6|	4	1	1	1	0	0	0	0
sp|P17096	4	4	4	4	2	3	3	3
tr|F5H2U8	9	1	1	1	0	0	1	0
sp|P49773	5	4	4	4	2	2	1	2
tr|D6RJE6|	8	2	2	2	1	1	1	1
sp|O14929	2	1	1	1	0	0	1	0
sp|Q92769	2	4	4	2	2	3	3	3
sp|P07305	2	4	4	4	2	3	4	4
sp|P16403	1	10	10	3	7	9	9	9
sp|P10412	4	9	2	2	8	8	8	8
sp|P16401	1	7	6	6	6	7	7	7
sp|Q92522	1	2	2	2	1	1	1	1
tr|A0A0U1	11	5	5	1	5	5	5	5

sp|Q71UI9	7	4	2	2	4	4	4	4
tr|Q5SQT3	2	2	1	1	1	1	1	1
sp|P16104	11	4	1	1	4	4	4	4
sp|Q99880	2	8	1	0	8	7	8	7
sp|Q16778	6	7	1	0	6	7	7	6
sp|Q8N257	1	7	1	1	7	5	7	5
tr|U3KQK0	10	8	8	0	7	8	8	7
tr|Q5TEC6	1	5	5	1	5	4	5	5
sp|P62805	1	8	8	8	8	8	8	8
sp|Q09028	15	9	9	5	4	3	5	5
sp|Q16576	4	6	2	2	3	3	3	2
sp|P16189	19	12	2	0	12	10	10	10
sp|P30453	10	12	12	0	12	10	10	10
tr|Q8MH48	6	2	1	1	2	2	2	2
sp|P30479	7	8	2	0	8	8	7	7
sp|P30508	40	9	5	0	9	8	7	7
sp|Q95604	1	8	2	0	7	7	6	6
sp|P50502	7	7	7	7	5	5	6	6
sp|Q16543	5	8	8	8	4	4	4	5
tr|H3BRH5	3	1	1	1	1	1	1	1
tr|A0A0D9	13	5	5	5	5	5	5	4
sp|P35914	1	2	2	2	1	2	2	2
sp|Q01581	2	8	8	8	4	2	4	7
sp|Q6YN16	2	5	5	5	5	5	5	5
sp|P00492	1	5	5	5	1	0	2	5
tr|A0A087	13	8	8	8	7	7	8	6
tr|A0A1W2	2	1	1	1	1	1	1	1
sp|P52292	4	9	9	9	6	7	6	6
sp|O00629	2	5	5	4	4	3	4	3
sp|O00505	1	2	1	1	1	1	2	1
sp|P52294	8	2	2	2	1	2	1	1
sp|Q14974	7	15	15	15	10	12	10	10
tr|H0Y8C6	12	10	10	10	1	0	3	1
sp|O95373	1	4	4	4	1	1	1	1
sp|Q96P70	1	2	2	2	1	0	0	0
sp|Q13308	9	2	2	2	1	2	2	2
sp|Q70UQ	2	2	1	1	2	2	2	2
sp|Q70UQ	1	2	2	1	2	2	2	2
sp|Q9NQS	2	2	2	2	1	1	2	2
sp|Q9Y2U8	1	2	2	2	0	1	2	2
sp|Q15181	2	9	9	9	5	5	4	6
sp|Q9BY32	3	2	2	2	1	0	0	1
tr|H0Y4R1	4	12	12	11	10	12	10	8
sp|Q14573	11	6	6	6	6	6	5	5
sp|O00425	11	4	4	4	4	4	3	1
sp|Q9Y287	2	4	4	4	3	3	3	3
tr|J3QRY6|	8	1	1	1	1	1	1	1
sp|P17301	5	15	15	15	13	13	13	12
sp|P26006	4	5	5	5	5	4	4	4
sp|P23229	10	22	22	22	20	20	19	15

sp|P06756	4	5	5	5	5	5	4	5
tr|A0A087\	4	2	2	2	2	2	2	2
sp|P05556	15	14	14	14	13	14	13	14
sp|P16144	8	36	36	36	29	32	31	33
sp|P05362	2	2	2	2	2	2	2	2
sp|P15260	4	3	3	3	2	2	2	2
tr|B1AJU4|	3	3	3	3	0	0	1	1
tr|H7BYV1	6	1	1	1	0	0	1	1
sp|P19525	4	5	5	5	4	5	5	3
tr|B4DY09	4	8	8	8	3	6	6	5
sp|Q12906	20	20	20	20	7	9	11	11
sp|Q27J81	2	1	1	1	0	1	1	0
tr|D6RGE2	2	1	1	1	0	0	0	0
tr|H0YL72|	9	8	8	8	6	6	5	6
sp|P51553	7	3	3	3	2	2	3	2
sp|O75874	6	13	13	13	6	6	6	8
sp|P41252	5	12	12	12	9	9	6	5
sp|Q9NSE4	1	7	7	7	6	7	7	7
tr|C9JD53|	4	1	1	1	1	1	1	1
sp|P14923	7	20	20	18	15	17	17	17
sp|Q9Y624	3	9	9	9	8	5	6	9
tr|K7EM02	4	1	1	1	1	1	1	1
;sp|P13645	9	7	4	4	4	6	5	4
;sp|P13646	10	27	25	11	27	27	27	24
;sp|P19012	5	16	7	5	16	15	14	15
sp|P05783	7	25	24	18	23	22	21	21
;sp|P08727	6	33	33	22	30	32	31	30
sp|P35527	3	5	4	4	1	1	3	3
;sp|P04264	2	8	4	2	4	5	5	6
;sp|P35908	3	7	1	1	5	5	5	5
;sp|P13647	21	28	24	5	24	25	24	23
;sp|P02538	9	23	12	2	16	21	20	20
sp|P04259	1	20	1	0	13	17	17	17
;sp|P08729	2	37	37	3	34	32	32	31
;sp|P05787	52	36	33	29	33	34	33	31
sp|Q07666	3	3	3	3	2	2	1	1
sp|Q86UP2	7	5	5	5	0	0	1	1
sp|P33176	5	6	6	6	5	6	3	3
sp|Q15058	1	1	1	1	1	1	1	1
tr|H7BYN4	6	5	5	5	3	5	5	5
sp|Q13601	2	1	1	1	1	1	0	1
tr|K7EM91	6	4	4	3	4	4	4	3
sp|Q16719	1	1	1	1	0	0	0	0
sp|Q04760	2	2	2	2	1	1	2	0
tr|E9PDI4|	6	7	7	7	4	6	6	6
sp|P42166	2	11	4	4	10	10	10	8
sp|P42167	4	13	13	6	11	11	11	11
sp|Q14739	3	4	4	4	2	4	4	3
sp|P20700	4	32	31	30	25	29	28	27
sp|Q03252	1	27	25	25	26	25	24	24

tr|K7EIP4|	8	1	1	1	1	1	1	1
sp|O15230	2	3	3	3	2	3	1	1
sp|Q13751	2	7	7	7	6	6	6	6
sp|P11047	1	5	5	5	5	5	5	4
sp|Q13753	2	7	7	7	7	5	7	7
tr|F8WDS9	3	1	1	1	0	0	1	1
tr|A0A0C4I	4	6	6	6	3	6	4	3
sp|P48449	5	3	3	3	3	3	3	3
sp|Q01650	1	5	5	5	4	4	4	3
tr|A0A0G2	13	3	3	3	0	0	0	0
sp|Q14696	1	2	2	2	2	2	2	1
sp|Q8NC56	5	2	2	2	2	2	2	1
sp|O95202	1	5	5	5	5	5	5	4
sp|P42704	9	33	33	33	25	27	27	25
sp|Q32MZ4	9	5	5	5	2	1	1	2
sp|O75473	3	1	1	1	1	1	1	1
sp|Q8N1G4	1	3	3	3	1	1	1	1
sp|Q96AG4	2	8	8	8	7	7	7	7
sp|Q9P2J5	3	5	5	5	4	3	2	3
sp|Q9UIQ6	3	3	3	3	1	2	3	2
sp|P30740	2	4	4	4	1	0	2	2
sp|Q08722	4	3	3	3	3	3	3	3
sp|P09960	5	6	6	6	1	1	4	5
sp|Q14847	7	8	8	8	4	4	5	5
tr|F8VQE1	8	12	12	12	5	3	7	9
tr|S4R3V8|	9	3	3	3	2	2	2	2
sp|P00338	22	13	13	12	7	9	8	7
sp|P07195	4	9	8	8	4	2	5	6
tr|K7EJE8|	7	6	6	6	6	5	6	6
sp|Q6P1M	2	4	4	4	4	4	4	2
sp|O95573	2	12	12	10	11	11	10	10
sp|O60488	3	5	3	3	5	4	4	4
sp|P24666	7	3	3	3	1	2	0	1
tr|J3KMZ9	9	5	5	5	4	3	4	5
sp|Q7Z4F1	1	1	1	1	1	0	1	1
tr|J3KPP4|	5	3	3	3	1	2	1	1
sp|P05455	3	4	4	4	0	0	1	1
tr|C9JY28|	5	1	1	1	0	1	1	1
sp|Q15046	5	8	8	8	5	5	6	6
sp|Q6UWP	10	3	3	3	3	3	3	3
tr|A0A0G2	3	2	2	2	1	2	2	2
sp|Q7L5N7	3	3	3	3	1	3	1	1
sp|Q6P1A2	2	1	1	1	1	1	1	1
sp|Q96N66	3	4	4	4	3	4	3	3
tr|D6RDG0	12	1	1	1	1	1	1	1
sp|P11279	2	4	4	4	4	3	4	4
sp|P13473	3	2	2	2	2	2	2	2
tr|H0YAR1	2	2	2	2	2	2	2	1
sp|P14174	1	2	2	2	2	1	2	2
sp|P40121	4	4	4	4	1	1	2	4

tr|A0A087\	3	3	3	3	3	3	3	3
sp|P07199	1	4	4	4	3	2	2	2
tr|D6RIZ4|	4	1	1	1	0	1	1	1
sp|Q95460	3	1	1	1	1	1	1	1
tr|A2A2V1	3	2	2	2	2	1	2	2
sp|Q14764	10	11	11	11	8	7	10	11
tr|B9A041	7	6	6	6	1	2	3	4
sp|P40926	3	18	18	18	18	18	17	17
sp|Q14165	4	4	4	4	4	4	3	4
sp|Q9ULC4	3	1	1	1	0	0	0	0
sp|Q9Y5P6	2	1	1	1	0	0	0	0
tr|J3KSI4|J	14	2	2	2	1	1	1	1
sp|Q13724	4	6	6	6	4	4	4	2
sp|Q8NDC(	1	1	1	1	0	0	0	0
sp|P49006	1	2	2	2	0	0	0	1
tr|A0A0R4.	21	17	17	17	14	15	11	9
sp|P50281	2	4	4	4	4	2	3	3
sp|Q14676	5	8	8	8	3	5	6	7
tr|E9PGW7	6	1	1	1	0	0	0	0
tr|B7Z9I1|	6	2	2	2	1	2	2	1
sp|P08582	1	3	3	3	0	1	2	1
sp|Q8N4V1	2	1	1	1	1	1	1	1
sp|O00264	2	4	3	3	4	4	4	4
sp|O15173	3	4	4	3	4	4	4	4
tr|I3L1H7|	10	1	1	1	1	1	1	0
tr|A8K878	3	4	4	4	3	4	4	4
tr|B8ZZX6|	5	2	2	2	2	1	1	1
sp|P80297	10	2	2	2	1	1	1	1
sp|O94776	2	4	4	4	3	2	4	4
sp|Q13505	4	4	4	4	4	3	3	4
tr|C9JAZ1|	4	2	2	2	2	2	2	2
tr|H7C0X7	5	1	1	1	0	0	1	1
sp|P53582	3	3	3	3	3	3	3	3
tr|G3V1U3	9	2	2	2	0	0	1	1
sp|P56192	8	6	6	6	3	4	3	3
tr|X6RBL6|	3	1	1	1	0	1	1	0
sp|Q9HCC(	5	3	3	3	3	2	3	3
tr|G5E9X5	5	1	1	1	1	1	1	1
sp|Q9BQA1	2	1	1	1	0	1	1	1
tr|E9PMI6	3	1	1	1	1	1	1	1
tr|F5H8H2	4	2	2	2	1	2	1	2
tr|A0A087\	5	2	2	2	1	2	2	2
tr|C9JRZ6|	4	6	6	6	5	4	4	4
tr|J3QTB2|	3	1	1	1	1	1	1	1
sp|Q16891	9	16	16	16	15	15	13	13
sp|Q99735	2	1	1	1	1	1	1	1
tr|Q5VV89	3	2	2	2	1	2	1	2
sp|P11137	2	1	1	1	0	0	0	0
sp|Q15691	1	8	8	8	0	1	2	2
tr|H0Y2V1	10	3	3	3	0	1	1	1

tr|E9PPJ5|	4	2	2	2	2	2	2	2
sp|Q9BTE3	2	1	1	1	1	0	0	1
sp|Q8TCT9	6	3	3	3	2	2	2	2
sp|Q8N4C8	5	1	1	1	1	1	1	1
tr|I3L1P8|I	3	5	5	5	5	5	5	5
sp|Q7Z434	2	3	3	3	2	2	2	2
tr|H0Y8C3	4	4	4	4	4	4	4	4
sp|Q9Y6C9	2	6	6	6	2	4	4	3
sp|Q9UBX3	3	4	1	1	3	3	3	2
sp|Q9Y3D6	1	2	2	2	1	2	1	1
tr|E9PJH7|	16	2	2	2	1	2	2	2
sp|Q9Y5L4	1	2	2	2	2	2	0	0
sp|O43615	3	6	6	6	5	4	6	6
tr|M0R047	3	1	1	1	1	1	1	1
sp|O60220	1	2	2	2	2	2	2	2
tr|G3V2F3	5	1	1	1	1	1	1	1
sp|Q9NS69	1	5	5	5	5	5	5	5
sp|Q15785	1	4	4	4	3	2	1	3
sp|O96008	4	9	9	9	9	7	7	9
sp|O94826	1	8	8	8	7	8	7	7
tr|C9JC63|	7	1	1	1	0	1	0	0
sp|Q99797	1	1	1	1	1	1	1	1
tr|C9JVB6|	2	3	3	3	3	3	3	3
sp|Q10713	3	6	6	6	3	3	5	5
sp|O75439	5	4	3	3	3	3	3	1
sp|P28482	2	2	2	2	0	0	0	0
tr|J3QT28|	4	5	5	5	5	4	5	5
sp|Q8IVT2	1	2	2	2	1	1	1	1
sp|Q9BYG3	4	3	3	3	3	3	3	3
sp|P26038	2	21	21	16	7	5	10	11
sp|P53985	1	2	2	2	2	2	2	2
tr|A0A087Y	7	3	3	3	3	3	3	2
tr|H3BVD1	19	2	2	2	1	1	1	1
tr|E9PPG9	4	5	5	5	4	4	4	5
sp|Q9UKD1	1	3	3	3	2	2	3	3
tr|F8WCY8	3	1	1	1	0	1	1	1
tr|F5GYQ2	4	1	1	1	1	1	1	1
tr|E9PBS1|	4	8	8	8	5	3	3	4
tr|C9JP00|	15	2	2	2	2	2	0	1
sp|Q9BQG	5	15	15	15	9	13	13	12
sp|Q9NUJ1	3	2	2	2	2	2	2	1
sp|O95297	6	4	4	4	3	3	4	2
sp|O60487	1	1	1	1	1	1	1	1
tr|C9JJV6|	5	2	2	2	2	2	2	2
sp|Q9NZM	24	49	49	49	44	46	44	43
sp|P05976	3	1	1	1	1	1	0	1
tr|F8W1R7	14	9	9	7	8	7	6	7
tr|J3KSW8	6	2	2	2	1	2	2	1
tr|J3QRS3|	6	8	8	8	7	8	6	6
sp|P35580	5	13	1	1	13	13	10	8

sp|P35749	4	11	1	1	10	11	9	9
sp|Q7Z406	7	9	2	2	9	9	6	6
sp|P35579	20	104	104	88	99	94	75	68
tr|C9JL85|	2	2	2	2	0	0	1	2
sp|P29966	1	8	8	8	5	5	6	7
sp|O14745	3	2	2	2	0	0	0	0
sp|Q86SF2	4	2	2	2	2	2	2	2
sp|Q9H0A(	5	13	13	13	12	11	10	12
tr|A0A0G2	23	4	4	4	4	3	2	1
sp|Q13423	4	4	4	4	2	2	1	2
tr|B4DLR8	6	4	4	4	3	2	2	3
tr|A0A1W2	13	4	4	4	4	4	4	3
sp|Q9BU61	1	1	1	1	1	1	1	1
tr|F8WAS3	6	1	1	1	1	1	1	1
tr|F5H0J3|	2	1	1	1	1	1	0	0
tr|H3BPJ9|	4	2	2	2	1	1	1	1
tr|E7EPT4|	2	2	2	2	2	2	2	2
sp|O75306	2	2	2	2	0	1	2	2
sp|O75489	1	1	1	1	1	1	1	1
tr|H7C0R7	2	1	1	1	1	1	1	1
sp|P00387	4	6	6	6	5	5	5	5
sp|P48163	2	1	1	1	0	0	0	0
tr|A0A0A0	14	3	3	3	2	1	3	3
sp|P16435	3	4	4	4	4	3	3	3
tr|E7EQ69	9	2	2	2	1	0	1	1
tr|F8VZJ2|	8	2	2	2	2	2	2	2
sp|Q9Y2A7	2	1	1	1	0	0	0	1
sp|Q9BT67	1	1	1	1	1	0	0	0
tr|H7C5F7	4	2	2	2	2	2	1	2
sp|P61081	3	3	3	3	0	0	0	0
sp|Q9UMX	1	2	2	2	2	1	2	2
sp|Q09666	7	138	138	138	101	107	120	120
tr|H0YAK4	3	1	1	1	1	0	1	0
sp|Q9Y639	8	3	3	3	2	3	3	2
sp|Q14697	2	26	26	1	25	25	20	17
sp|Q14697	4	26	1	1	25	25	20	17
sp|P43007	2	2	2	2	2	2	2	2
sp|Q15758	6	9	9	9	8	8	8	8
tr|A0A0R4	6	4	4	4	4	4	4	3
tr|X6R8F3|	3	4	4	4	4	4	4	4
tr|B7Z5R6|	3	1	1	1	1	1	1	1
tr|B1AHD1	2	3	3	3	2	3	3	3
sp|Q96TA1	2	3	3	3	1	0	0	2
sp|Q969V3	4	4	4	4	3	3	3	2
sp|P43490	4	8	8	8	3	3	4	4
sp|Q6XQN	2	1	1	1	0	0	0	1
tr|K7EQ23	3	1	1	1	1	1	1	1
tr|J3KN36|	11	6	6	6	6	6	6	6
sp|P05204	4	3	3	3	0	0	0	0
sp|Q15233	6	13	12	12	8	10	9	6

sp|P22307	7	4	4	4	4	3	4	4
tr|J3QRU1	7	3	3	3	1	1	1	1
sp|Q9UNZ2	8	6	6	6	0	0	3	4
sp|Q9BV86	2	1	1	1	1	1	1	1
tr|H0YDS9	8	3	3	3	0	0	0	1
sp|Q09161	1	4	4	4	2	2	1	2
tr|F8WE41	3	1	1	1	0	0	1	1
tr|D6RH30	3	1	1	1	0	1	1	0
sp|Q7Z417	1	3	3	3	2	3	3	1
sp|Q9Y266	3	5	5	5	2	3	3	3
sp|Q14980	16	46	46	46	33	30	38	36
sp|P57740	5	6	6	6	5	4	5	5
sp|Q8WUM	1	6	6	6	5	4	4	4
sp|P49790	3	6	6	6	3	4	4	4
tr|E9PF10|	4	13	13	13	10	9	10	10
tr|G3V198	6	7	7	7	5	5	5	5
sp|Q92621	2	9	9	9	7	5	7	5
tr|A0A0A0	10	5	5	5	2	4	4	4
sp|Q9UKX7	2	1	1	1	1	1	1	1
tr|J3QL54|	9	2	2	2	2	2	2	2
tr|J3KMX1	4	5	5	5	5	5	5	5
tr|H3BVG0	12	7	7	7	7	7	5	6
sp|P52948	10	6	6	6	5	4	5	4
sp|P37198	2	3	3	3	2	2	2	3
sp|Q8TEM	2	4	4	4	3	4	3	2
sp|Q8TAT6	2	1	1	1	0	0	0	0
sp|Q13772	6	6	6	6	6	4	4	5
sp|P61970	2	5	5	5	1	3	3	2
sp|Q9H1E3	2	3	3	3	0	0	1	0
sp|O15381	5	1	1	1	1	1	1	1
sp|P67809	4	10	10	7	5	6	9	8
sp|Q02818	2	3	3	3	2	2	0	1
tr|A0A0A0	7	5	5	5	2	1	4	2
sp|Q9Y3T9	1	4	4	4	1	2	3	4
sp|Q8WTT	1	1	1	1	0	0	0	0
sp|Q9BZE4	4	10	10	10	6	8	8	7
sp|O60287	1	1	1	1	0	0	1	1
sp|Q9H8H0	4	2	2	2	2	2	2	2
tr|E9PFK5|	3	1	1	1	0	1	1	1
tr|D6RC60	7	1	1	1	0	1	1	1
sp|O00567	4	10	10	10	8	9	10	10
sp|Q9Y2X3	3	10	10	10	9	10	10	9
sp|Q9H6R4	6	2	2	2	2	2	1	2
sp|Q9NR30	3	18	18	17	15	15	15	16
tr|E9PKP7|	4	3	3	3	2	2	3	3
sp|P19338	6	23	23	23	17	17	16	17
tr|F8WE16	7	1	1	1	0	0	0	0
sp|P06748	5	12	12	12	9	10	10	10
tr|F8VTY2|	2	1	1	1	1	1	1	1
sp|Q8NFH3	2	3	3	3	3	3	2	3

sp|Q8NFH!	8	5	5	5	4	4	5	4
sp|Q7Z3B4	3	3	3	3	2	2	3	3
sp|Q96EE3	6	5	5	5	4	4	2	3
sp|P12270	2	14	14	14	9	8	9	8
tr|A0A0G2	2	1	1	1	0	1	0	1
sp|P15531	4	5	1	1	1	3	2	2
tr|Q32Q12	7	8	8	4	3	5	3	4
tr|H0YHC3	18	3	3	3	3	3	3	2
tr|C9JZI7|(	14	4	4	4	3	1	3	4
sp|Q96RS6	3	2	2	2	0	0	0	0
tr|E5RFP0|	2	1	1	1	1	1	1	1
tr|C9JTK6|	4	1	1	1	1	0	1	1
sp|Q56VL3	4	3	3	3	3	3	3	3
tr|A0A087'	3	1	1	1	1	1	1	1
sp|Q9NQR	3	7	7	7	6	6	6	6
sp|Q99650	2	1	1	1	1	1	1	1
tr|F6WST4	4	1	1	1	1	1	0	0
tr|J3QRM9	3	1	1	1	1	1	1	1
sp|P04181	2	10	10	10	10	9	9	9
sp|Q92882	1	1	1	1	0	0	1	0
sp|P36551	3	5	5	5	5	5	4	3
sp|Q9NWT	1	1	1	1	0	0	1	1
sp|Q15645	3	3	3	3	3	3	3	1
tr|E9PSE5|	6	1	1	1	1	1	1	1
tr|B4DRN8	2	1	1	1	1	1	1	1
sp|Q8WXF	3	3	3	3	1	0	0	1
sp|O00151	1	10	10	10	2	2	6	7
sp|O14908	5	2	2	2	1	1	1	1
sp|Q96EY7	2	2	2	2	2	1	2	2
sp|P62937	1	8	1	1	8	8	8	7
sp|P62937	12	11	11	4	10	10	9	8
sp|P23284	1	10	10	10	9	9	7	9
sp|P45877	1	1	1	1	1	1	1	1
sp|P30405	9	4	3	3	0	0	1	1
sp|P62942	7	3	3	3	0	1	1	1
sp|P26885	2	2	2	2	1	1	2	2
sp|Q00688	1	3	3	3	0	0	1	1
sp|Q02790	4	12	12	12	5	8	6	9
tr|M0R2K9	3	1	1	1	1	1	1	1
sp|Q9Y3C6	1	2	2	2	1	0	0	1
sp|Q8WUA	1	1	1	1	0	0	0	0
tr|J3KQ48|	2	2	2	2	2	2	2	2
tr|E5RGQ4	8	2	2	2	1	0	0	1
sp|O60664	7	8	8	8	2	3	7	6
sp|Q15269	3	3	3	3	3	2	2	1
sp|Q06830	3	15	15	11	11	11	10	11
sp|P32119	3	9	8	8	4	4	5	4
sp|Q13162	4	12	9	9	10	10	8	5
sp|P30044	4	6	6	6	6	6	6	6
sp|P30041	1	9	9	9	6	7	8	7

tr|I3L2U4|	5	1	1	1	1	1	1	1
sp|Q92968	1	1	1	1	0	1	1	0
sp|O75381	2	2	2	2	1	2	1	2
sp|P51659	8	17	17	17	15	14	16	15
tr|M0QXB5	2	1	1	1	1	1	1	1
tr|B5MCF9	5	8	8	8	1	3	3	3
tr|C9JZE2|	10	1	1	1	1	1	1	1
sp|Q7RTV0	1	2	2	2	2	2	2	1
tr|K7ER00|	5	3	3	3	1	2	2	1
sp|Q9NSD9	2	10	10	10	3	3	2	2
sp|Q8WW(	1	2	2	2	0	1	2	2
sp|Q00325	6	8	8	8	7	7	6	6
sp|O95674	1	1	1	1	1	1	1	1
sp|P30086	1	6	6	6	0	0	2	1
tr|C9J3E3|	5	1	1	1	1	1	1	0
tr|A0A0A0	15	4	4	4	1	1	1	1
sp|Q13492	24	4	4	4	2	2	2	3
sp|P48651	3	1	1	1	1	1	0	1
sp|Q16822	9	4	4	4	3	3	3	3
sp|P36871	3	8	8	8	0	0	2	2
sp|Q96G03	2	1	1	1	0	0	1	1
sp|P00558	3	15	15	15	10	13	13	12
sp|P18669	3	11	11	11	5	4	5	5
sp|Q8IV08	2	2	2	2	1	1	1	1
tr|C9J7K9|	7	2	2	2	1	1	2	2
tr|C9JDU5	9	1	1	1	0	1	1	0
sp|Q9Y617	2	8	8	8	2	2	3	4
tr|C9JBI3|(	2	1	1	1	0	0	0	1
sp|Q9H307	1	3	3	3	3	3	3	3
sp|O00625	1	1	1	1	0	0	0	0
sp|Q13835	2	2	2	2	1	2	2	1
sp|Q99959	3	2	2	2	1	1	1	1
sp|Q9Y446	7	9	9	9	4	8	8	8
sp|P20020	32	3	3	3	3	3	3	3
sp|Q8NC51	4	11	11	11	3	3	6	7
sp|P05120	6	6	6	6	3	3	2	4
sp|P13796	4	19	19	13	7	7	10	12
tr|A0A0A0	8	16	10	10	5	5	5	7
sp|P43034	3	5	5	5	2	3	4	5
sp|P68402	4	1	1	1	0	0	0	0
tr|R4GND3	2	1	1	1	1	1	1	1
sp|Q15149	14	102	102	102	86	88	87	79
sp|O43660	3	2	2	2	1	0	2	2
sp|O75051	1	1	1	1	1	1	1	1
sp|O15031	4	6	6	6	6	5	6	5
sp|P09874	3	17	17	17	13	14	12	11
sp|Q9NVV4	2	1	1	1	1	1	1	1
sp|Q15365	2	9	9	7	6	7	5	6
sp|Q15366	23	8	6	6	5	6	5	6
tr|A0A0J9Y	18	8	8	8	2	2	7	7

sp|P11940	26	18	18	14	14	16	12	12
sp|Q86U42	6	2	2	2	1	1	1	2
tr|B1ANR0	8	7	3	3	4	5	3	4
tr|D6REB4	4	2	2	2	0	0	0	0
tr|B4DEM9	2	1	1	1	1	1	1	1
tr|F6VRR5	7	2	2	2	2	1	1	1
sp|Q6NZI2	3	10	10	10	7	8	8	7
sp|Q10471	5	9	9	9	8	8	8	8
tr|E7EUL0|	3	3	3	3	3	2	3	3
tr|A6NLN1	12	10	10	8	6	7	7	6
sp|O95758	8	8	6	6	7	7	5	3
sp|Q8TCS8	4	3	3	3	3	2	2	1
tr|A6NP52	2	1	1	1	1	1	1	1
tr|E5RGS4	2	1	1	1	0	0	0	1
sp|Q9UHV9	1	1	1	1	0	0	0	0
tr|E9PQY2	2	1	1	1	0	0	0	0
sp|P02545	10	39	39	38	32	35	33	33
sp|Q9UMS	5	10	10	10	6	7	7	7
sp|O75400	3	2	2	2	2	2	1	1
sp|O94906	2	9	9	9	4	7	4	5
sp|Q6P2Q9	3	16	16	16	9	10	9	8
sp|Q8NAV1	1	1	1	1	0	1	1	0
sp|O43143	1	6	6	6	5	5	5	5
sp|Q9ULR0	3	1	1	1	1	0	0	0
sp|Q9UHG	5	2	2	2	1	1	1	1
sp|Q8IY81	1	7	7	7	4	5	6	7
sp|Q2NL82	2	4	4	4	4	4	1	0
sp|Q5JRX3	6	6	6	6	6	5	5	5
sp|Q96IZ0	1	1	1	1	1	0	1	1
sp|P46087	10	14	14	14	10	8	13	14
tr|F5GYY1|	14	1	1	1	1	1	1	1
tr|A0A1X79	6	18	18	11	15	16	13	12
tr|B7Z6D5	4	7	7	7	3	4	7	6
tr|F8WAJ0	4	2	2	2	1	2	1	2
tr|A0A0C4I	3	7	7	7	7	6	4	2
sp|Q9H0S4	2	2	2	2	2	2	2	2
tr|J3KTA4|	10	17	10	10	15	14	10	12
tr|F8WDT8	5	1	1	1	0	1	1	1
sp|P26196	2	2	2	2	2	1	2	2
sp|Q9HA77	3	2	2	2	1	1	2	1
tr|A0A0A0	4	3	2	2	2	3	2	1
sp|Q96K78	1	1	1	1	0	0	0	0
tr|H3BMQ1	2	1	1	1	1	1	1	1
sp|Q9Y4C8	1	2	2	2	2	1	2	2
tr|H7C2Q8	2	7	7	7	3	4	4	5
sp|Q8NBJ5	3	9	9	9	8	8	7	8
sp|Q02809	2	4	4	4	3	2	3	4
tr|E7ETU9|	4	2	2	2	2	2	2	2
sp|O60568	4	7	7	7	7	6	6	6
sp|P07737	4	10	10	10	7	7	7	8

sp|Q8WUN	7	7	7	7	4	5	5	5
sp|Q53EL6	2	1	1	1	0	0	0	0
tr|A0A024	3	2	2	2	0	2	1	0
sp|P35232	7	13	13	13	12	11	11	12
sp|Q99623	9	17	17	17	12	14	13	13
sp|P12004	1	7	7	7	4	2	7	5
sp|Q9UQ8	6	16	16	16	7	7	6	7
sp|P13674	3	6	6	6	6	5	5	5
sp|O15460	2	3	3	3	2	2	1	1
sp|P48147	1	3	3	3	2	1	2	1
tr|C9JIZ6|	6	8	8	8	4	4	4	3
sp|O14684	1	2	2	2	2	2	2	2
tr|X6RJ95|	3	3	3	3	3	3	3	3
tr|A0A087	6	5	5	5	4	3	0	1
sp|Q9P2B2	1	2	2	2	1	2	2	1
sp|Q14914	4	6	6	6	1	0	4	1
sp|Q16186	2	2	2	2	2	2	2	1
sp|Q06323	4	5	5	5	0	0	4	3
tr|H0YM70	4	2	2	2	1	0	1	2
tr|K7ENH2	12	3	3	3	0	0	0	0
tr|H0Y555	4	1	1	1	0	1	1	1
tr|C9JCK5|	3	2	2	2	0	1	2	0
tr|H0YMZ1	12	5	5	5	4	3	3	3
tr|G3V295	9	7	7	7	2	4	6	5
sp|P25786	4	11	11	11	3	5	5	4
sp|P25788	5	3	3	3	0	0	1	1
sp|P28066	2	8	8	8	4	5	3	2
sp|O14818	9	9	9	9	6	5	6	5
tr|X5D2R7	5	3	3	3	0	0	1	1
tr|A2ACR0	6	1	1	1	0	0	1	1
sp|P20618	1	5	5	5	4	4	5	4
sp|P49721	2	2	2	2	0	0	0	0
tr|A0A087	4	2	2	2	1	2	2	2
sp|P28070	1	2	2	2	0	0	1	1
sp|P28074	4	7	7	7	3	2	2	3
sp|P28072	3	3	3	3	3	2	3	3
sp|Q99436	3	2	2	2	1	1	1	1
tr|J3KN16|	4	2	2	2	0	0	0	0
sp|Q9NY61	3	3	3	3	1	0	2	1
sp|Q99873	10	7	7	7	4	5	5	4
tr|H0YJX6|	10	2	2	2	2	2	2	2
sp|Q8TD16	2	1	1	1	0	0	0	0
sp|P41223	3	2	2	2	1	0	1	1
tr|F8W031	9	5	4	4	4	3	3	4
tr|A0A0J9Y	2	2	2	2	2	1	2	2
sp|Q9UKY7	8	3	3	3	0	0	2	3
tr|A6NLH6	2	1	1	1	1	1	1	1
tr|C9IZG4|	4	1	1	1	0	0	1	1
sp|Q99497	3	6	6	6	3	3	3	4
sp|P35659	7	5	5	5	4	4	5	5

tr|A0A140	7	9	9	9	0	1	1	1
sp|P07237	12	22	22	22	21	21	18	19
sp|P30101	1	22	22	17	21	21	19	19
sp|P13667	1	14	14	14	12	12	12	13
sp|Q15084	5	13	13	13	12	9	11	9
tr|H3BPB3	4	1	1	1	1	0	1	1
sp|Q8WYP	4	4	4	4	2	2	4	4
sp|P49257	1	9	9	9	8	8	7	8
tr|H7C3D5	2	1	1	1	1	0	1	1
sp|Q49AJ0	5	2	2	2	1	1	1	2
tr|F8W7Q4	3	3	3	3	3	3	2	2
tr|K7ELZ8|	5	1	1	1	1	1	0	1
sp|Q92520	3	6	6	6	3	5	4	3
sp|Q9NUQ	12	3	3	3	0	1	3	0
sp|Q6ZRV2	2	22	22	22	19	20	18	18
sp|Q8NCA5	2	1	1	1	1	1	0	0
sp|Q52LJ0	2	3	3	3	2	2	1	2
sp|Q13045	6	2	2	2	2	1	2	1
tr|G5E994	3	1	1	1	0	0	1	1
tr|M0R2E1	5	1	1	1	1	0	0	0
sp|P78504	4	2	2	2	2	2	2	2
tr|A0A0U1	6	2	2	2	1	0	0	0
tr|E9PIE3|	2	2	2	2	1	2	2	2
sp|P41743	1	1	1	1	1	1	1	1
tr|C9JEN3|	8	2	2	2	2	2	2	2
tr|G3V1D4	6	2	2	2	0	0	0	1
sp|Q9C0E8	4	2	2	2	1	2	2	2
sp|Q86UE4	3	5	5	5	3	4	5	5
tr|F5H6P7|	6	2	2	2	2	2	1	1
tr|H0YBV6	2	1	1	1	1	0	0	0
sp|Q969L2	1	1	1	1	1	1	1	1
sp|Q92597	14	9	9	9	3	4	4	4
sp|Q9BZQ8	1	4	4	4	0	1	1	2
sp|Q9BPW	3	2	2	2	0	2	1	1
sp|Q9UFN0	1	1	1	1	1	1	1	1
sp|Q96C90	1	2	2	2	1	0	2	2
tr|B5MBZ8	9	1	1	1	0	0	0	0
sp|O15355	1	5	5	5	1	1	1	1
sp|P29590	16	12	12	12	10	10	9	10
tr|E7EPN9	9	2	2	2	1	0	1	0
sp|Q9P258	1	7	7	7	5	4	4	4
tr|Q5T092	4	1	1	1	1	1	1	1
sp|Q14690	1	6	6	6	4	4	3	5
tr|R4GN98	2	2	2	2	1	1	1	2
sp|P60903	1	3	3	3	2	3	2	2
sp|P31949	1	4	4	4	4	4	4	4
sp|Q99584	1	1	1	1	0	0	0	1
sp|Q9HCY8	1	5	5	5	4	5	5	3
sp|Q96FQ6	1	2	2	2	1	2	2	2
sp|P29034	3	5	5	5	5	4	3	3

sp|O43819	1	2	2	2	2	2	2	2
sp|P55735	5	4	4	4	4	3	3	4
sp|Q01105	7	8	8	8	1	0	2	4
sp|P18583	11	4	4	4	3	3	3	4
tr|H3BT44	8	1	1	1	1	1	1	1
tr|H7C4R5	3	2	2	2	1	2	2	1
sp|Q92734	7	4	4	4	3	2	3	3
tr|G5EA31	3	2	2	2	2	1	2	2
tr|D6RHZ5	13	1	1	1	0	1	1	0
tr|B4DR61	15	4	4	4	4	4	4	4
sp|P60468	2	3	3	3	3	3	3	3
sp|P60059	1	1	1	1	1	1	1	1
tr|A0A1W2	3	1	1	1	1	1	0	0
tr|E9PS11|	3	1	1	1	1	1	1	1
sp|Q969M	2	1	1	1	0	1	0	0
tr|A0A0A0	6	5	5	5	3	3	3	4
tr|Q5TG12	9	3	3	3	3	3	2	2
tr|B8ZZQ6	6	5	5	5	0	0	2	4
sp|Q08174	5	7	7	7	7	4	4	7
sp|O60245	4	3	3	3	3	3	3	2
sp|P00491	4	7	7	7	0	0	1	2
tr|J3QSB7|	11	4	4	4	2	1	2	1
tr|E9PLK3|	11	8	8	8	2	2	5	5
sp|O43865	7	2	2	2	1	1	1	0
sp|Q4W5N	1	1	1	1	0	0	0	0
tr|H7BXY3	4	1	1	1	1	1	1	1
sp|Q5VTE0	7	19	19	11	18	18	16	16
sp|Q58FF8	1	10	1	1	7	9	9	9
sp|Q58FF6	1	6	1	1	3	4	4	4
tr|Q5JR04|	3	3	3	3	2	2	1	1
sp|Q5T280	2	2	2	2	2	2	2	1
tr|K7EMM	6	1	1	1	0	0	1	1
tr|A0A087	5	2	2	2	1	0	0	0
sp|Q96HJ9	10	3	3	3	1	2	2	2
sp|Q5T440	1	1	1	1	1	1	1	1
sp|A6NIL9	1	1	1	1	1	1	0	0
tr|A0A087	9	2	2	2	2	2	1	1
sp|P11498	4	8	8	8	7	5	7	6
sp|P08559	8	3	3	3	3	3	3	3
sp|P11177	5	5	5	5	5	3	4	5
sp|O00330	2	1	1	1	0	0	1	1
sp|P14618	11	39	39	4	29	32	29	28
sp|P14618	4	36	1	1	26	29	26	25
sp|P50395	10	13	13	13	6	5	9	8
sp|Q9H0H	4	3	3	3	3	3	3	3
sp|Q6IAA8	5	4	4	4	4	4	4	3
tr|R4GMU	4	1	1	1	1	1	1	1
sp|P46060	5	12	12	12	6	6	7	8
sp|P43487	7	3	3	3	2	2	2	3
sp|Q13283	8	9	9	9	0	4	3	4

sp|P46940	9	36	36	35	19	19	21	24
sp|Q15404	2	2	2	2	0	0	1	1
sp|P63000	3	9	9	3	5	7	5	7
sp|P15153	13	8	2	2	5	7	3	7
sp|Q9HB9(	3	2	2	2	1	1	0	0
sp|P61026	3	6	5	5	5	5	6	6
sp|Q15907	8	11	11	10	9	9	10	9
sp|P61106	2	13	13	13	13	12	12	5
sp|Q9NP72	6	7	7	7	6	6	7	5
sp|P62820	11	11	11	4	11	10	11	11
sp|Q9H0U4	3	11	4	3	9	9	9	9
sp|Q9UL25	1	4	4	4	4	4	4	3
sp|P57735	1	2	1	1	2	2	2	2
tr|H3BU81	7	1	1	1	1	1	1	1
sp|P61019	10	7	7	7	7	7	6	5
sp|Q13637	3	5	5	4	4	5	5	5
tr|F5H157|	3	2	2	2	2	2	1	1
sp|Q86YS6	1	1	1	1	1	0	1	1
sp|P51148	19	7	7	7	7	7	7	6
sp|P20340	14	5	5	5	3	5	4	4
sp|P51149	7	16	16	16	12	16	13	12
sp|O14966	4	3	2	2	2	2	2	2
sp|P61006	2	3	1	1	2	2	3	2
sp|P51151	3	3	3	3	2	3	3	3
tr|H7C3P7	2	4	4	3	3	3	4	3
sp|P61224	23	6	6	2	5	5	5	5
sp|P61225	1	4	4	2	3	4	3	3
sp|P10301	1	3	1	1	3	3	3	3
sp|P62070	7	4	4	2	4	4	4	4
sp|P60602	2	1	1	1	1	1	1	1
sp|Q00765	6	3	3	3	3	3	3	3
tr|Q96L35	3	2	2	2	2	1	2	2
sp|P10586	7	4	4	4	3	2	2	2
sp|Q9BRX8	2	4	4	4	4	4	4	4
sp|P18754	7	8	8	8	5	7	7	6
sp|Q96DB5	10	3	3	3	3	2	2	2
tr|H0YNE5	3	1	1	1	1	1	1	1
sp|Q92900	2	1	1	1	1	1	1	1
sp|Q8WUF	2	2	2	2	2	2	2	2
sp|P35250	9	3	3	3	2	2	2	2
sp|P40938	3	5	5	5	5	5	4	3
tr|C9JZI1|(	8	2	2	2	2	2	0	1
sp|P40937	5	2	2	2	1	1	1	1
tr|B5MC59	2	3	3	3	0	2	1	1
sp|P15927	5	2	2	2	0	0	0	1
sp|P27694	2	2	2	2	1	0	1	1
sp|Q15293	2	9	9	9	6	8	5	5
tr|H0YL43|	3	3	3	3	2	2	2	2
sp|Q9NQC	9	7	7	7	4	6	5	6
sp|P06400	1	1	1	1	1	1	0	0

sp|Q8NFJ5	2	2	2	2	2	2	2	2
sp|Q8IZV5	2	3	3	3	3	2	2	3
sp|Q8TC12	8	5	5	5	5	5	5	5
sp|Q9HBH!	1	2	2	2	1	2	2	1
tr|J3KRE2|	7	3	3	3	2	3	2	2
tr|F5H2R5	5	2	2	2	1	1	2	2
sp|Q07960	3	5	5	5	1	2	2	3
tr|R4GNI4|	7	1	1	1	0	0	0	0
tr|Q5JR08|	13	5	5	1	5	5	5	4
sp|P84095	1	6	5	5	4	5	5	5
sp|P13489	10	10	10	10	6	5	6	6
sp|P23921	4	3	3	3	0	0	1	0
tr|C9JXC1|	9	4	4	4	1	2	2	1
sp|P60891	4	3	2	2	1	2	1	1
sp|P11908	8	4	4	3	2	2	1	1
sp|Q9Y4W	3	1	1	1	0	0	0	0
sp|O76021	6	16	16	16	10	10	12	13
tr|J3QR09|	4	4	4	4	3	3	4	3
sp|P56182	2	5	5	5	4	4	4	4
sp|Q92979	4	3	3	3	2	2	3	3
sp|Q9Y3A4	1	1	1	1	0	1	1	1
sp|Q14692	1	5	5	5	4	2	4	4
sp|Q14137	3	7	7	7	5	5	5	4
sp|Q8TDN(	1	6	6	6	1	4	1	1
tr|A0A0A0	3	3	3	3	3	3	3	2
sp|Q9GZL7	1	4	4	4	4	3	3	4
sp|Q15050	1	2	2	2	2	2	2	2
tr|F8WE72	3	2	2	2	0	0	1	2
tr|Q5VXN0	4	4	4	4	2	3	2	2
tr|A0A0A0	21	17	17	17	3	5	12	13
sp|Q96E11	9	5	5	5	4	5	5	5
tr|D6RAL1	6	1	1	1	1	1	0	0
sp|P38159	12	11	11	11	4	4	9	9
sp|Q96PK6	2	10	10	8	9	9	9	9
sp|Q96PK6	7	4	2	0	4	4	4	4
sp|P49756	6	3	3	3	3	3	2	3
tr|U3KPZ7	2	1	1	1	0	0	0	1
sp|Q9NW1	4	5	5	5	4	5	5	5
tr|Q5TCT4	4	2	2	2	0	1	2	2
tr|A0A0U1	15	7	7	7	5	6	5	4
sp|Q9Y5S9	3	3	3	3	1	2	3	3
tr|B0QYK0	10	6	6	6	1	1	3	2
tr|H3BPE7	3	5	3	3	1	2	5	5
tr|H3BUR4	2	1	1	1	1	1	1	1
sp|Q9NRX:	2	2	2	2	1	2	2	2
tr|Q5QPL9	6	5	5	5	3	4	4	4
tr|H3BV80	8	2	2	2	2	2	1	1
sp|Q5TZA2	3	2	2	2	1	2	2	2
tr|M0R299	8	5	5	5	5	5	5	5
sp|Q6IN84	1	1	1	1	1	1	1	1

sp|A6NHQ	1	1	1	1	1	1	1	1
tr|G3V2M5	4	1	1	1	1	1	1	1
sp|Q5JTH9	4	4	4	4	0	2	2	2
sp|Q9Y3B9	1	3	3	3	1	2	2	2
sp|Q9Y265	6	12	12	12	8	8	8	8
sp|Q9Y230	5	13	13	13	7	10	8	6
sp|P31153	2	5	5	5	2	4	2	3
tr|F8VZQ9	3	2	2	2	0	0	0	0
sp|P16615	18	22	22	22	21	20	20	20
sp|Q15424	5	9	9	4	5	8	8	7
sp|Q14151	2	8	3	3	4	5	7	6
tr|B7ZKQ9	7	1	1	1	1	1	1	1
sp|Q08AF3	1	1	1	1	1	1	1	0
sp|Q8WVN	9	4	4	4	0	0	4	4
sp|Q12765	6	3	3	3	0	1	1	3
tr|A0A087Y	3	1	1	1	1	1	1	1
sp|O15127	1	1	1	1	1	1	1	1
sp|O14828	2	4	4	4	4	3	3	4
sp|Q969E2	2	1	1	1	0	0	0	0
sp|P35270	1	1	1	1	0	0	0	0
tr|D6RDP1	12	1	1	1	0	0	1	1
sp|Q13501	8	14	14	14	13	14	14	12
sp|P83111	2	2	2	2	1	2	1	1
sp|P34897	17	15	15	14	15	14	13	14
sp|Q9NRX5	1	4	4	4	2	1	3	2
sp|O15269	2	7	7	7	7	6	7	5
sp|O15270	2	2	2	2	1	2	2	2
tr|M0R088	5	1	1	1	0	0	0	0
sp|Q9UQ3	9	13	13	13	9	8	10	9
tr|J3KTL2|	7	7	7	7	6	7	7	7
tr|Q5JRI1|	8	2	2	2	2	2	1	1
tr|Q5T760	3	1	1	1	1	1	1	1
tr|J3KP15|	6	5	5	5	5	4	5	5
tr|A0A087X	3	4	3	3	3	3	3	3
sp|Q13243	6	3	2	2	3	3	3	3
sp|Q13247	5	5	5	4	4	4	4	5
tr|A0A0B4	7	6	6	5	4	6	5	4
sp|Q13242	3	7	7	7	5	6	5	7
tr|H0Y630	6	2	2	1	1	2	2	1
tr|E7ETY4|	18	2	2	2	1	2	2	2
tr|C9JIG9|	2	1	1	1	1	0	0	0
sp|Q13177	12	5	5	5	0	1	2	4
tr|H0YJF7|	2	1	1	1	1	0	1	1
sp|P63151	7	2	2	2	2	2	2	2
sp|P30153	13	11	11	11	5	6	7	8
tr|A6PVN9	19	3	3	3	0	0	0	0
sp|P67775	10	7	7	6	5	6	4	5
sp|Q96HS1	3	5	5	5	4	5	4	4
sp|P62136	4	8	2	2	4	5	5	3
sp|P62140	6	9	9	3	7	8	6	4

tr|E7ETC2|	13	2	2	2	1	1	1	1
tr|H3BV22	6	4	3	3	2	1	1	1
tr|F8VYE8|	6	8	2	2	5	6	6	4
sp|Q9Y3F4	3	5	5	5	3	4	2	3
tr|Q5T5C7	2	4	4	4	2	2	2	1
tr|M0QWZ	7	2	2	2	2	2	2	2
tr|F8WE70	5	4	4	4	1	1	1	1
sp|P36952	3	14	14	14	5	8	8	8
tr|A0A024	9	6	6	6	0	1	3	3
sp|P50454	13	13	13	13	12	12	12	11
sp|Q9BXP5	6	3	3	3	2	1	2	1
tr|J3QT77|	6	1	1	1	1	1	1	1
tr|U3KQT1	4	1	1	1	0	0	1	0
tr|Q5T123	2	2	2	2	0	1	2	2
sp|Q9NR4	1	2	2	2	0	0	2	2
tr|A0A0A0	4	6	6	6	3	3	5	5
sp|Q9H9B	5	6	6	6	6	5	5	4
tr|H0YNG3	8	2	2	2	1	2	2	1
sp|Q9Y6A	3	3	3	3	2	2	2	1
tr|E9PL01|	5	4	4	4	4	2	4	4
sp|P61009	1	3	3	3	3	3	2	2
tr|H0YLA2	2	4	4	4	1	2	1	2
tr|A0A087	3	2	2	2	0	0	0	1
sp|P49458	3	4	4	4	1	1	3	2
sp|Q9Y5M	2	6	6	6	5	5	5	5
sp|Q9UHB	4	3	3	3	3	2	1	1
sp|O76094	3	3	3	3	2	1	1	1
tr|J3KPM9	8	5	5	5	3	3	4	4
sp|Q92783	2	1	1	1	0	1	1	0
sp|O75886	2	1	1	1	0	1	1	1
sp|Q04837	4	4	4	4	4	4	4	4
tr|G3V1B8	3	1	1	1	1	1	1	1
tr|K7EMD6	2	2	2	2	0	1	1	1
sp|P62306	1	2	2	2	1	1	1	1
sp|P62314	3	2	2	2	1	1	0	0
sp|P62316	3	3	3	3	0	1	1	1
sp|P62318	3	4	4	4	4	4	3	3
tr|J3QLE5|	8	5	5	5	4	3	2	3
sp|O75691	1	3	3	3	1	2	2	1
tr|B8ZZN6	6	3	3	3	2	2	3	2
sp|P61956	7	2	2	2	0	0	2	2
tr|G3V5R3	3	1	1	1	0	0	0	0
sp|P05023	19	28	28	27	25	28	27	23
sp|P05026	3	4	4	4	4	4	4	4
sp|P54709	5	3	3	3	3	2	2	2
tr|F8VX04|	2	2	2	2	1	1	1	2
sp|Q96QD	4	3	3	3	2	2	3	2
sp|Q8WUX	8	4	4	4	3	3	3	3
tr|H7BZK4	2	1	1	1	1	1	1	1
tr|G3XAL9	3	2	2	2	0	2	2	2

sp|P11166	1	3	3	3	3	3	3	3
sp|Q9NQZ	1	1	1	1	1	1	1	1
sp|Q00796	4	8	8	8	3	3	3	5
tr|C9J0K6|	5	5	5	5	2	5	2	3
sp|Q9Y512	1	2	2	2	1	1	1	1
sp|Q9UMY	4	2	2	2	0	0	1	1
sp|O60493	4	2	2	2	2	1	1	1
sp|Q9NUQ	16	10	10	10	6	10	9	8
sp|Q9BW0	2	2	2	2	0	1	2	2
tr|A0A0D9	8	29	29	29	7	8	19	21
tr|A0A087	5	17	17	17	3	4	12	9
tr|E5RJR5|	6	3	3	3	2	3	3	2
tr|H7BXF4	7	2	2	2	0	1	1	2
tr|H0Y3V8	3	2	2	2	2	2	2	2
sp|Q13838	17	9	9	2	5	6	2	3
sp|Q15637	9	4	4	4	2	1	2	2
sp|Q15459	3	3	3	3	0	0	2	1
sp|Q12874	1	4	4	4	0	0	4	2
sp|O75533	5	9	9	9	3	8	7	9
sp|Q13435	7	6	6	6	1	3	4	4
sp|Q15393	6	10	10	10	5	5	5	6
sp|Q9Y3B4	1	2	2	2	1	2	2	2
sp|P0DN76	11	4	4	4	4	4	3	3
tr|K7ENG2	3	6	6	6	4	4	4	3
sp|P23246	4	17	17	16	7	4	9	7
sp|Q8WW	1	3	3	3	3	3	3	3
tr|E9PNM1	12	11	11	11	6	9	6	5
tr|F8VV04|	3	1	1	1	0	0	0	0
tr|A0A087	8	3	3	3	2	3	2	3
sp|Q14247	5	17	17	17	9	8	11	11
sp|Q7KZF4	2	16	16	16	11	11	9	9
sp|P16949	3	5	5	5	0	0	0	1
tr|E5RGX5	3	1	1	1	0	0	0	0
sp|Q5K651	2	2	2	2	1	2	1	0
tr|A0A087	4	1	1	1	1	1	1	1
sp|Q15738	2	6	6	6	5	5	4	6
tr|A0A087	4	8	8	8	6	6	5	7
sp|Q8WXE	2	1	1	1	1	1	1	1
sp|P38646	5	33	33	32	29	31	32	30
sp|P31948	6	16	16	16	9	9	10	10
sp|Q9HCN	1	2	2	2	1	2	2	1
tr|H0YDB2	5	1	1	1	1	1	1	1
sp|O95347	4	6	6	6	3	3	3	3
sp|Q9UQE	1	5	5	5	1	0	2	2
tr|G8JLG1|	4	4	4	4	1	0	2	2
tr|E9PD53	7	6	6	6	6	4	3	3
sp|P31040	6	8	8	8	8	8	7	7
sp|P53597	2	4	4	4	3	2	2	3
sp|Q9P2R7	7	5	5	5	4	4	5	5
sp|Q96I99	4	4	4	4	4	4	4	4

sp|P55809	3	6	6	6	5	6	6	5
tr|A8MXB9	17	3	3	3	3	2	2	3
sp|Q9Y6N5	6	12	12	12	12	11	12	9
tr|M0QX65	6	1	1	1	0	0	0	0
tr|H3BPC4	4	3	3	3	2	1	1	1
tr|H7C2K3	9	1	1	1	1	1	1	1
sp|P42285	2	2	2	2	1	1	1	1
sp|P00441	2	3	3	3	1	1	1	2
sp|P04179	10	5	5	5	4	5	4	4
sp|Q9Y2Z0	2	4	4	4	2	1	3	1
sp|Q9BRV8	2	1	1	1	0	0	0	0
tr|A8MV53	9	4	4	4	1	1	3	3
sp|Q15526	2	1	1	1	1	1	1	1
tr|Q5T8U5	5	2	2	2	2	1	1	2
sp|O75683	1	3	3	3	1	2	1	1
sp|O75940	1	2	2	2	0	1	0	1
tr|F8VXC8|	5	3	3	3	1	1	2	3
sp|O60264	1	9	9	8	6	8	9	7
tr|J3KMX2	6	2	2	2	1	0	1	1
tr|K7EMQ8	10	2	2	2	1	0	1	1
tr|E7EMB1	2	2	2	2	0	0	0	0
sp|Q99536	9	9	9	9	4	3	7	7
sp|O15498	3	2	2	2	0	1	1	2
sp|Q16563	2	1	1	1	1	1	1	1
sp|Q9H987	2	1	1	1	1	1	1	1
sp|O95721	1	1	1	1	0	0	0	0
tr|H3BV99	10	2	2	2	2	2	2	2
sp|P18827	3	2	2	2	1	2	2	2
sp|P31431	2	2	2	2	2	2	1	2
sp|O43752	1	2	2	2	0	1	1	1
sp|O15400	2	2	2	2	2	2	2	2
sp|O00560	4	10	10	1	8	9	9	8
sp|O00560	2	10	1	1	8	9	9	8
sp|Q9Y490	4	10	10	10	4	4	6	4
tr|A0A0G2	11	2	2	2	2	2	2	2
sp|Q13148	19	6	6	6	5	6	4	2
sp|Q92804	5	6	6	4	1	2	5	4
tr|B8ZZD4|	6	6	6	6	6	6	6	6
sp|O14907	1	1	1	1	1	1	1	1
sp|Q8TC07	3	3	3	3	0	0	3	1
sp|Q9BYX2	6	1	1	1	1	0	0	0
sp|P17987	11	17	17	17	12	11	13	12
sp|P78371	4	17	17	17	9	6	6	7
sp|P50991	2	15	15	15	10	12	8	8
tr|E7ENZ3|	7	11	11	11	7	6	5	4
sp|Q99832	5	10	10	10	6	5	6	7
sp|P49368	9	15	15	15	9	9	6	8
sp|P50990	5	20	20	20	13	9	12	13
sp|P40227	6	10	10	10	3	5	6	5
sp|Q5UIP0	4	3	3	3	1	0	3	3

sp|Q68CZ2	3	2	2	2	1	2	2	1
sp|Q9UGI8	4	2	2	2	0	1	1	1
tr|E9PRJ3|	3	3	3	3	2	3	1	2
tr|F8VV56|	7	3	3	3	3	3	2	2
tr|E9PJK1|	9	3	3	3	3	3	3	3
tr|A6NNI4	3	2	2	2	0	0	0	1
sp|O60635	1	1	1	1	1	1	1	1
sp|O95857	1	1	1	1	1	1	1	1
sp|P52888	3	2	2	2	1	0	0	0
sp|P10599	2	5	5	5	2	2	3	2
sp|O95881	1	2	2	2	1	2	2	2
sp|Q9BRA2	4	3	3	3	1	1	2	2
sp|Q8NBS9	2	8	8	8	8	6	7	6
tr|E9PIR7|	22	6	6	5	1	1	2	1
sp|P30048	2	7	7	7	7	7	6	6
sp|O43396	5	6	6	6	3	5	6	6
sp|Q9H3N1	2	4	4	4	2	4	4	3
sp|Q9Y320	7	7	7	7	5	7	6	5
sp|Q16762	2	2	2	2	2	2	2	1
tr|A0A0C4I	2	1	1	1	1	1	1	1
sp|Q86V81	2	6	6	6	0	0	3	2
sp|Q6I9Y2	1	1	1	1	0	0	0	0
sp|P26639	9	17	17	17	7	7	8	8
sp|P07996	4	11	11	11	9	10	10	11
tr|H3BNW(	2	1	1	1	0	0	0	0
tr|K7ES52|	4	1	1	1	1	1	0	0
sp|P62328	1	2	2	2	0	0	1	1
sp|Q9Y2W	1	11	11	11	5	6	9	7
sp|Q15654	2	12	12	12	8	9	5	5
tr|A0A1B0(	10	10	10	10	5	7	8	7
sp|O75663	2	1	1	1	0	0	0	1
sp|Q86XR7	6	7	7	7	7	6	6	3
sp|P13726	2	3	3	3	3	3	3	3
tr|A0A0A0	14	1	1	1	1	1	1	0
tr|E7EMV7	12	5	5	5	3	2	3	4
tr|E9PNS3	3	1	1	1	1	0	1	1
tr|J3KN66|	8	6	6	6	5	5	5	5
tr|J3KPT4|	2	1	1	1	0	0	1	1
sp|P37837	2	7	7	7	2	1	3	4
tr|Q9HBD4	7	1	1	1	1	1	1	1
tr|A0A1W2	15	5	5	5	0	0	1	3
tr|E5RHG8	4	3	3	3	2	2	2	2
tr|B8ZZU8	5	4	4	4	2	2	2	2
sp|O14776	2	2	2	2	0	0	0	0
sp|Q00059	3	4	4	4	4	4	4	4
sp|P20290	2	1	1	1	0	0	0	0
sp|Q13263	5	11	11	11	3	2	3	3
sp|P49711	1	3	3	3	1	1	2	3
tr|A0A0U1	6	1	1	1	1	1	1	1
tr|E9PF19|	5	3	3	3	2	2	1	3

sp|Q12788	3	8	8	8	6	8	6	6
sp|P02786	4	23	23	23	19	20	19	19
sp|Q13595	4	1	1	1	1	1	1	1
sp|P62995	5	4	4	4	3	4	4	4
sp|Q15582	3	2	2	2	2	2	2	1
sp|P61586	1	5	1	1	5	5	5	4
sp|P37802	5	11	11	11	7	8	8	9
sp|O43493	6	2	2	2	2	2	2	2
sp|P55072	3	22	22	22	16	15	18	18
sp|P29401	6	18	18	18	9	12	9	12
sp|Q92616	1	9	9	9	5	7	4	4
sp|Q9BSH4	1	2	2	2	2	2	2	2
tr|Q5W0H4	8	6	6	6	2	4	4	3
tr|G3XAN4	3	3	3	3	2	3	2	1
tr|F8WF48	3	2	2	2	1	2	1	2
sp|Q9UGP8	1	4	4	4	4	4	4	4
tr|B1AH87	2	2	2	2	1	1	2	1
tr|C9J3L8|	6	3	3	3	2	2	1	1
sp|P51571	2	3	3	3	3	3	3	2
tr|C9JA28|	4	2	2	2	1	1	2	1
tr|E9PMQ9	10	3	3	3	3	2	3	3
sp|Q99805	1	6	6	6	6	6	5	4
sp|Q9HD45	2	3	3	3	3	3	3	3
tr|A0A0C4	2	5	5	5	5	5	4	4
tr|J9JIE6|J	6	2	2	2	2	2	2	2
sp|Q13445	1	1	1	1	0	1	1	1
tr|G3V2K7	2	2	2	2	2	2	2	2
tr|F5GX39	3	1	1	1	1	1	1	1
sp|Q7Z7H5	4	4	4	3	4	3	3	2
sp|Q9Y3A6	4	2	2	2	2	2	2	2
sp|Q9BVK6	1	3	2	2	3	3	2	2
sp|Q14956	2	3	3	3	3	3	3	3
sp|Q9BVC6	1	2	2	2	2	2	2	2
sp|Q9HC07	5	3	3	3	2	3	2	2
sp|Q8IY95-	2	2	2	2	0	0	2	1
tr|J3KRW7	3	1	1	1	1	1	1	1
tr|K7EM09	4	1	1	1	0	0	1	1
sp|Q6NUQ	4	4	4	4	3	3	4	4
tr|K7ENI6|	3	1	1	1	1	1	1	0
tr|D6RAA6	3	2	2	2	2	2	2	2
sp|Q9BTV4	1	4	4	4	4	4	4	4
sp|Q9BXS4	5	5	5	5	4	4	5	4
sp|Q8NBN	4	3	3	3	2	3	2	3
tr|B1ALM5	5	2	2	2	1	1	1	1
sp|Q92973	5	4	4	4	4	4	4	4
tr|J3KQ96|	11	15	15	15	9	11	9	11
sp|P53007	2	3	3	3	3	3	3	3
sp|P40939	3	20	20	20	18	18	20	17
sp|P55084	7	14	14	14	13	12	13	12
sp|P22102	7	4	4	4	3	2	3	3

sp|P60174	6	15	15	15	6	5	6	6
sp|Q14134	2	2	2	2	1	1	1	1
sp|Q96LD4	2	2	2	2	2	2	2	2
sp|Q08J23	4	14	14	14	14	12	10	9
tr|F5H1S9|	9	3	3	3	2	2	1	1
sp|Q9Y3I0	1	8	8	8	4	6	4	5
sp|Q9NYL9	4	4	4	4	3	3	0	1
sp|P09493	15	10	4	1	8	9	6	7
sp|P06753	16	18	18	12	14	13	13	15
sp|P67936	5	14	2	2	13	10	10	12
sp|P67936	9	15	9	2	12	10	10	11
sp|P23381	22	6	6	6	1	0	2	1
sp|Q71U36	9	18	1	0	15	16	16	13
sp|P68363	3	18	18	0	15	16	16	14
tr|F5H5D3	15	18	2	2	15	16	16	12
sp|P68366	6	18	4	4	15	15	16	15
tr|Q5JP53|	7	16	4	3	16	15	14	13
sp|Q13885	2	15	3	3	14	15	13	11
tr|A0A0B4J	7	11	2	1	11	11	9	9
sp|P68371	13	17	17	2	16	17	15	15
sp|Q9BUF5	10	9	3	3	8	8	6	6
sp|Q3ZCM	3	9	2	2	9	9	8	7
tr|K7EKE5|	3	1	1	1	1	1	1	0
tr|E5RIW3	5	3	3	3	0	0	1	1
sp|Q14166	1	4	4	4	3	3	1	1
sp|Q03169	1	1	1	1	0	0	0	0
sp|O14763	2	1	1	1	1	1	1	1
sp|Q9H3D4	12	2	2	2	2	2	1	1
sp|P55327	12	4	4	4	0	0	1	2
tr|A0A087Y	9	5	5	5	0	1	4	4
sp|Q5T0D9	2	3	3	3	2	2	2	2
sp|Q12888	3	1	1	1	1	0	1	1
sp|P09758	1	8	8	8	8	8	8	8
sp|Q12792	5	2	2	2	1	0	1	2
tr|D6RG15	3	1	1	1	0	0	1	1
sp|Q6RW1	2	1	1	1	1	1	1	1
sp|Q9UIG0	2	11	11	11	8	6	9	8
sp|P07948	9	5	5	5	5	4	5	5
sp|P18031	2	8	8	8	5	4	4	6
sp|P54577	2	3	3	3	3	3	2	2
tr|H0YHS6	2	3	3	3	3	3	3	2
sp|P08621	6	6	6	6	3	2	3	4
tr|M0R221	4	2	1	1	0	1	0	0
tr|A0A0A0	2	2	2	2	0	0	1	2
sp|P09661	4	4	4	4	0	2	3	2
sp|P08579	4	2	2	1	0	1	0	0
sp|O15042	7	4	4	4	2	2	1	2
sp|Q9NV31	1	2	2	2	2	2	1	2
sp|O00566	1	1	1	1	0	1	1	1
sp|Q9BVJ6	4	5	5	5	0	2	3	4

sp|Q9Y5J1	3	2	2	2	2	2	1	1
sp|Q9NYH!	1	2	2	2	2	2	2	2
sp|O43818	1	3	3	3	2	3	3	2
sp|O43172	2	1	1	1	1	1	1	1
tr|A0A087	5	2	2	2	0	0	1	2
sp|O75643	3	17	17	17	11	12	9	10
sp|Q96DI7	3	3	3	3	2	1	2	2
sp|Q9Y333	1	1	1	1	0	0	0	1
tr|F2Z2Y6|	4	1	1	1	0	0	0	0
sp|Q9UMX	6	5	5	3	3	3	1	2
tr|H3BPW3	8	1	1	1	1	0	1	1
tr|H3BQP1	4	1	1	1	1	1	0	0
sp|P45974	3	2	2	2	0	0	1	0
sp|P15374	3	6	6	6	2	2	4	5
tr|A6NJA2	6	8	8	8	0	1	2	1
tr|H0Y6Y4|	9	2	2	2	1	0	1	1
sp|Q92890	4	2	2	2	0	0	2	1
tr|F5GYN4	7	4	4	4	3	3	4	3
sp|P62979	23	7	7	7	7	7	7	7
tr|F8W726	13	4	4	4	1	0	1	2
sp|O00762	3	2	2	2	1	1	1	0
sp|P61086	4	3	3	3	0	0	1	1
sp|A0A1B0	4	1	1	1	0	1	1	1
sp|P61088	6	4	4	4	2	2	1	3
tr|I3L0A0|	16	4	4	2	1	1	3	3
sp|Q15819	1	4	2	2	2	1	3	3
tr|H0Y614	2	1	1	1	1	1	1	1
sp|P22314	8	21	21	21	9	12	13	12
sp|Q9BZL1	2	2	2	2	0	1	2	0
tr|A0A087	5	1	1	1	1	1	1	1
sp|Q92575	1	3	3	3	3	3	3	3
sp|O60701	9	9	9	9	7	7	5	6
sp|Q9NYU	4	10	10	10	9	9	10	9
sp|P19224	21	4	4	4	3	4	3	2
sp|P30085	3	5	5	5	0	1	0	1
sp|Q8N99	2	1	1	1	0	1	1	1
sp|Q8WW	1	1	1	1	1	1	1	1
tr|C9JQV0|	4	2	2	2	2	2	2	2
tr|E9PDF6|	11	20	20	20	16	16	13	14
tr|F5H6E2|	10	12	12	12	11	11	10	9
sp|Q12965	4	3	3	3	2	2	0	0
tr|A0A0A0	9	2	2	2	2	2	2	2
sp|Q9H7E!	2	1	1	1	0	1	0	1
tr|H0YJB9|	3	1	1	1	0	1	0	1
tr|H0YF29|	3	1	1	1	1	1	1	1
sp|Q96IX5	1	2	2	2	2	2	2	2
sp|P11172	5	3	3	3	2	1	0	0
tr|C9J486|	5	3	3	3	1	2	0	1
tr|G3V170	5	1	1	1	1	1	1	1
tr|Q5T446	5	1	1	1	0	0	0	0

tr|Q5H8X8	3	1	1	1	1	1	1	1
tr|A0A087'	10	3	3	3	0	0	0	1
sp|P54727	10	8	8	8	4	3	4	6
sp|Q3ZAQ	1	1	1	1	1	1	1	1
sp|O75436	3	3	3	3	1	3	3	3
tr|E9PLM9	6	1	1	1	0	1	1	1
sp|Q96QK	4	6	6	6	5	4	5	6
sp|O75351	5	2	2	2	2	2	2	2
tr|A0A087'	4	2	2	2	0	1	1	0
tr|A0A140	7	5	5	5	4	3	3	3
sp|Q8TAA	2	1	1	1	1	1	1	1
sp|P49748	11	21	21	21	18	19	18	17
sp|Q6Y1H2	1	1	1	1	0	1	0	0
sp|Q9P035	6	7	7	7	6	6	5	5
tr|A0A1B0	4	3	3	3	1	2	2	1
sp|Q9NZ01	4	3	3	3	2	2	2	2
tr|Q5TIH2|	2	1	1	1	1	1	1	1
sp|Q15836	7	4	4	4	4	4	4	4
sp|Q9BV4	3	3	3	3	2	3	3	3
sp|Q9P0L0	1	2	1	1	2	2	2	2
sp|O95292	4	5	5	4	5	4	4	4
tr|I3L0N3|	3	1	1	1	1	1	1	0
tr|F8WAU	8	1	1	1	1	1	1	1
sp|O75396	2	3	3	3	3	3	3	3
sp|Q12907	5	5	5	5	5	2	3	3
tr|A0A024	14	8	8	8	5	7	4	3
sp|P18206	3	22	22	22	4	3	5	8
;;tr|D6RBJ	7	2	2	2	2	2	1	1
sp|Q8N0U	2	1	1	1	1	1	1	1
sp|P21796	2	17	17	17	16	16	16	12
sp|P45880	7	17	17	17	17	17	16	17
sp|Q9Y277	8	8	8	8	6	6	6	6
sp|Q8IWT	1	2	2	2	2	2	2	2
sp|P27449	1	1	1	1	1	1	1	1
sp|P38606	4	7	7	7	3	4	5	5
tr|K7EQW	8	1	1	1	1	1	1	1
sp|P21281	7	8	8	8	3	3	4	5
tr|H0YJ55|	5	1	1	1	0	1	1	1
tr|F5GYQ1	6	3	3	3	1	2	2	2
sp|P36543	5	4	4	4	1	3	1	3
sp|O75348	4	3	3	3	1	2	3	3
tr|G3V126	5	5	5	5	4	4	5	5
sp|O75083	3	7	7	7	3	4	2	5
tr|U3KQC1	7	5	5	5	4	3	3	3
tr|A0A0A0	2	3	3	3	1	2	3	3
sp|Q15061	3	3	3	3	3	3	3	3
sp|O15213	2	4	4	1	3	3	3	3
sp|P61964	1	1	1	1	0	1	1	1
tr|H0YM7	4	1	1	1	0	0	0	0
tr|E9PS41|	4	2	2	2	2	2	1	1

sp|Q8IWA(	3	2	2	2	1	1	1	1
sp|Q9UBH(	2	1	1	1	0	1	1	0
sp|P13010	3	16	16	16	11	12	10	7
sp|P12956	3	13	13	13	6	7	7	9
sp|P16989	4	6	3	3	4	3	6	5
sp|Q9Y5A9	7	2	2	2	0	0	0	0
tr|E9PQ61	2	2	2	2	1	1	2	2
sp|Q8WU9	2	1	1	1	0	0	0	0
tr|C9J6P4|	4	5	5	5	4	4	4	4
sp|O95159	6	2	2	2	2	1	2	2
sp|Q96KR1	1	2	2	2	0	0	0	0
sp|Q9BQ52	12	3	3	3	3	2	3	1
tr|H0Y362	2	2	2	2	2	2	2	2
tr|Q5T4K2	5	1	1	1	1	1	1	1
tr|E5RIP4|	4	1	1	1	1	1	1	1
sp|Q8N4Q(	1	2	2	2	0	1	1	2
tr|H0Y2Y8|	4	3	3	3	0	0	1	3

Peptides 5	Peptides 6	Peptides D	Peptides D	Razor + uni	Razor + uni	Razor + uni	Razor + uni	Razor + uni
7	7	7	8	8	8	7	7	7
7	8	8	8	6	7	7	4	6
1	1	1	1	1	1	1	1	1
8	7	8	8	4	4	4	4	4
13	14	15	14	9	8	10	10	10
10	10	10	10	5	5	8	7	7
9	8	8	7	4	3	6	5	6
15	14	16	17	14	15	14	14	15
11	10	11	12	7	7	7	7	7
11	12	11	11	8	7	8	10	8
1	2	2	2	1	0	1	1	1
2	2	2	0	2	2	2	2	2
1	0	0	0	1	1	0	0	1
7	7	5	6	6	7	7	7	7
1	2	3	3	3	2	0	0	1
2	6	6	6	7	7	5	4	2
1	5	5	5	4	4	2	4	1
3	4	5	4	4	4	4	3	3
3	4	2	3	4	4	2	2	3
5	9	10	10	8	7	6	7	5
2	4	5	4	4	3	2	4	2
2	4	4	6	3	4	1	2	2
0	2	2	2	1	1	0	1	0
2	7	10	9	6	4	3	3	2
2	6	6	4	2	4	1	1	2
3	4	5	6	2	2	3	2	3
0	1	1	1	1	1	1	1	0
4	6	9	9	8	12	6	7	4
2	5	5	5	5	4	5	4	2
0	1	0	2	0	0	0	1	0
1	1	3	3	0	0	0	0	1
2	3	3	3	1	2	1	2	2
1	1	1	2	1	1	1	1	1
2	3	5	4	0	0	2	1	2
1	1	1	1	1	1	1	1	1
1	1	1	1	1	1	1	1	1
1	2	1	2	1	2	2	2	1
4	4	4	2	3	4	4	4	4
1	3	3	3	3	3	3	3	1
1	1	1	0	1	1	1	0	1
2	2	2	1	3	2	3	2	2
1	1	1	1	1	1	2	1	1
2	3	2	1	3	3	1	1	2
1	1	0	1	2	2	2	2	1
2	1	1	0	2	0	2	2	2
2	2	1	0	2	2	2	2	2
1	1	1	1	1	1	1	1	1
0	1	1	1	1	1	1	1	0

2	3	2	1	3	3	3	3	2
0	2	1	2	1	2	1	1	0
11	12	12	9	14	16	17	16	11
1	1	1	1	1	1	1	1	1
1	2	1	3	2	2	3	3	1
3	4	4	4	4	3	4	3	3
2	2	0	0	2	2	2	1	2
3	2	0	1	2	2	2	3	3
1	1	1	1	1	0	1	1	1
1	2	1	1	2	2	2	2	1
1	0	0	0	1	2	1	1	1
1	1	1	1	1	1	1	1	1
2	2	1	1	2	1	2	2	2
0	0	0	0	0	1	0	1	0
1	1	1	0	1	1	1	1	1
2	2	1	1	2	2	2	2	2
1	1	0	0	1	1	1	1	1
1	1	1	1	1	1	1	1	1
0	1	1	0	1	2	1	1	0
2	1	1	1	1	1	1	1	2
0	1	1	1	1	1	1	1	0
2	2	2	3	3	3	2	1	2
7	8	9	5	5	6	6	8	7
2	1	1	2	2	2	2	2	2
1	1	0	1	1	1	1	1	1
1	1	1	1	1	0	1	1	1
0	3	3	5	2	2	1	3	0
7	8	9	8	8	8	9	8	7
7	6	7	7	7	7	7	6	7
3	4	5	5	6	5	5	3	3
5	5	5	7	6	7	6	5	5
3	5	5	5	5	4	4	4	3
3	6	6	5	5	4	4	4	3
4	5	5	4	7	5	5	4	4
5	6	6	6	6	6	6	6	5
3	6	6	7	7	5	6	6	3
8	8	9	9	7	8	8	7	8
3	2	3	3	3	3	3	3	3
1	4	4	4	3	2	3	2	1
3	4	4	4	4	4	3	4	3
2	2	3	3	2	2	2	2	2
1	3	2	3	2	2	2	2	1
2	3	3	2	3	3	3	2	2
3	4	4	4	4	4	3	3	3
1	2	2	2	1	2	2	2	1
1	1	2	0	1	1	1	1	1
8	10	11	11	12	12	11	9	8
1	1	1	1	1	1	1	1	1
8	10	11	12	10	12	10	9	8
6	8	11	10	8	10	6	6	6

5	5	6	6	7	7	7	4	5
4	5	4	6	5	6	4	5	4
2	2	4	3	3	4	3	2	2
8	9	9	10	10	10	10	9	8
5	8	8	9	8	8	7	7	5
6	8	8	7	8	8	7	7	6
20	21	23	23	24	22	23	22	20
2	2	2	1	2	2	2	2	2
1	1	1	1	2	1	2	2	1
26	25	27	26	25	26	26	25	26
5	9	9	8	5	8	8	9	5
1	2	2	2	1	1	1	1	0
4	6	7	6	7	7	6	6	4
5	5	7	6	7	8	6	5	5
6	7	7	7	7	7	6	7	6
3	3	4	3	4	4	3	4	3
3	3	4	3	1	1	1	1	1
5	5	6	6	5	6	5	5	5
4	6	5	6	5	6	5	5	4
6	6	7	6	6	7	6	6	6
4	3	4	4	4	4	4	4	4
6	6	6	6	6	6	6	6	6
2	1	5	6	4	4	4	2	2
4	4	4	4	4	4	4	4	4
5	5	5	5	5	4	5	5	5
2	1	2	2	2	1	2	1	2
2	2	2	2	2	2	2	2	2
3	4	4	4	4	4	4	4	3
2	3	4	4	3	4	3	4	2
5	3	5	4	4	5	5	5	5
2	4	5	6	4	6	4	3	2
1	4	4	4	3	4	3	2	1
2	3	3	4	3	3	3	3	2
4	4	4	5	4	4	4	4	4
2	2	2	2	0	2	2	2	2
12	12	15	14	15	16	12	11	12
5	4	5	4	4	5	5	4	5
3	2	3	4	4	4	3	3	3
4	4	4	5	4	4	4	4	4
5	5	6	5	5	6	6	6	5
1	2	2	2	2	2	2	1	1
5	5	5	4	4	4	4	4	5
3	3	3	3	3	3	3	3	3
2	2	2	2	2	2	2	2	2
1	2	3	3	2	3	3	1	1
1	1	1	1	1	1	1	1	1
13	11	13	13	14	14	13	11	13
4	9	9	9	7	9	8	6	4
9	12	13	12	12	12	12	10	9
4	6	5	6	6	6	6	6	4

6	13	12	12	12	13	13	13	6
1	1	1	1	1	1	1	1	1
3	4	5	4	3	3	3	3	3
2	4	5	5	5	4	4	3	2
0	1	1	2	0	1	0	0	0
3	7	9	10	3	4	5	4	3
1	1	1	1	0	0	0	1	1
1	1	1	2	0	1	1	2	1
25	26	28	28	27	28	25	24	25
4	5	4	3	5	5	5	6	4
0	1	1	1	1	0	0	0	0
2	2	3	2	1	1	3	2	2
1	0	1	0	0	1	1	0	1
1	2	2	1	1	1	1	0	1
6	6	6	6	6	6	6	6	6
1	1	1	1	1	1	1	0	1
0	4	4	4	1	0	2	1	0
0	2	2	2	0	0	0	0	0
7	7	8	9	6	8	6	6	7
18	19	19	19	1	1	1	1	1
18	19	19	19	19	19	19	18	18
10	10	10	10	1	1	1	1	1
4	4	5	5	4	4	4	4	4
2	3	4	4	0	2	1	2	2
2	2	4	3	3	2	2	2	2
2	4	4	3	1	2	2	1	2
0	0	1	0	0	0	0	0	0
3	3	4	4	3	3	2	2	3
1	1	1	1	1	1	1	1	1
0	1	1	1	0	1	1	1	0
4	7	7	7	6	4	6	6	4
3	3	3	3	3	3	3	3	3
2	5	7	6	1	2	2	3	2
1	0	0	0	0	0	0	0	1
1	2	2	1	2	2	1	1	1
2	2	2	2	1	1	2	2	2
4	5	5	5	5	5	5	5	4
2	2	2	2	2	2	2	2	2
2	2	3	2	1	2	2	2	2
1	1	0	0	1	1	1	1	1
2	2	1	1	2	2	2	2	2
5	5	5	5	5	5	5	5	5
1	2	2	2	1	2	3	3	1
2	2	2	2	2	2	2	1	2
0	1	0	1	1	1	1	0	0
1	3	3	3	2	3	1	0	1
1	2	1	1	1	1	1	0	1
0	1	1	1	1	0	0	0	0
6	12	14	14	10	11	11	9	6
4	3	4	4	5	3	3	3	4

2	2	2	0	1	1	2	2	2
3	5	5	5	2	3	3	2	3
1	1	1	2	0	0	0	0	1
0	5	5	4	0	0	2	3	0
7	10	10	11	6	7	9	9	7
3	6	4	5	5	6	5	5	3
0	1	1	1	0	0	0	1	0
11	14	13	13	14	14	13	15	11
10	13	12	13	4	4	4	4	4
2	2	3	2	2	3	3	3	2
2	5	6	6	4	4	5	4	2
2	3	3	3	1	1	1	1	1
2	4	4	4	1	1	0	1	1
2	3	2	3	3	3	3	3	2
2	2	2	2	2	2	2	2	2
2	2	2	3	0	0	2	1	2
2	2	1	2	1	2	2	2	2
4	3	4	4	4	4	4	4	4
1	1	1	1	0	1	1	1	1
0	1	0	1	0	0	1	0	0
0	2	2	2	2	2	2	2	0
4	6	5	5	6	6	5	3	4
2	3	3	3	0	0	1	3	2
1	2	3	4	1	2	2	2	1
11	15	17	17	14	13	12	9	11
3	3	2	2	1	1	1	2	2
5	10	9	8	7	9	6	5	5
4	10	8	7	0	2	1	1	0
2	5	7	5	1	1	1	0	0
3	3	5	5	2	2	2	3	3
5	9	8	7	10	10	10	9	5
0	0	0	0	1	1	1	1	0
1	1	1	1	0	0	0	1	1
1	2	1	0	2	1	1	1	1
12	28	33	31	13	16	19	19	12
11	27	33	33	7	9	13	12	8
1	5	3	5	4	2	4	3	1
1	1	1	1	1	1	0	0	1
0	0	0	1	0	0	1	0	0
17	18	19	18	12	14	16	14	17
2	2	0	1	2	1	2	2	2
1	0	0	1	0	0	0	0	1
4	5	3	3	3	4	5	4	4
1	1	1	1	1	1	0	1	1
3	4	4	4	4	4	4	4	3
1	4	4	4	4	4	2	2	1
2	2	3	3	2	3	2	2	2
3	4	5	4	3	2	4	4	3
2	1	2	2	2	2	2	2	2
8	7	8	6	6	7	7	8	8

6	6	7	6	4	5	6	5	5
1	0	0	1	1	0	1	1	1
0	0	1	1	1	1	1	0	0
17	22	23	20	22	21	22	22	17
0	1	1	1	1	1	1	0	0
3	5	6	7	5	5	5	4	3
23	23	25	24	25	25	24	24	23
9	11	11	12	8	7	11	10	9
9	11	11	10	6	11	10	10	9
5	10	10	9	9	8	8	8	5
3	6	7	9	7	10	6	8	3
4	8	6	7	8	8	7	8	4
13	8	6	7	5	9	12	11	13
1	2	2	1	1	1	1	2	1
3	6	6	6	2	2	4	4	3
1	2	2	2	1	0	1	1	1
1	1	1	2	0	3	2	3	1
1	4	6	5	0	1	1	1	0
2	3	2	1	2	2	3	2	2
0	1	0	1	0	1	0	0	0
0	1	1	1	0	1	0	0	0
0	1	1	1	1	2	1	1	0
5	5	4	5	4	4	4	5	4
0	1	1	1	1	1	1	1	0
2	2	2	1	2	2	2	2	2
2	0	1	0	2	0	2	2	2
0	4	4	4	0	0	1	0	0
1	1	2	3	1	1	1	1	1
8	7	5	5	9	9	9	8	8
8	8	6	7	6	7	7	6	8
1	1	1	1	0	0	0	1	1
2	5	7	7	5	5	4	3	2
1	5	3	5	4	5	3	4	1
5	5	4	6	3	4	4	4	5
1	4	3	3	0	0	1	1	1
9	12	12	11	11	12	12	11	9
7	15	19	18	15	12	11	13	7
1	0	1	1	2	2	2	0	1
3	4	3	4	4	4	4	4	3
1	1	1	1	1	1	1	1	1
2	2	1	0	1	0	1	2	2
3	2	2	2	3	2	2	3	3
1	1	2	2	2	2	0	1	1
5	5	4	4	6	6	6	5	5
1	1	1	1	1	1	1	1	1
18	14	17	17	22	21	16	18	18
17	17	19	17	21	21	16	17	17
6	5	4	5	8	6	7	6	6
2	2	2	2	2	2	2	2	2
2	2	2	2	2	2	2	2	2

0	1	2	0	1	2	2	1	0
1	2	2	2	2	3	2	3	1
5	8	7	9	10	10	8	9	5
2	5	6	6	5	7	6	4	2
3	3	4	4	4	4	4	4	3
4	4	3	4	5	5	5	5	4
1	0	0	1	1	1	1	1	1
2	1	1	1	2	2	2	2	2
1	4	4	3	2	1	1	1	1
0	2	2	2	2	2	0	1	0
0	1	1	1	1	1	1	1	0
1	1	1	1	1	1	0	0	1
11	17	18	22	7	9	13	13	11
3	3	4	4	2	2	2	2	2
2	2	3	3	3	2	0	0	2
7	10	12	11	12	11	10	9	7
5	3	3	3	5	4	4	3	5
2	1	1	0	3	3	3	3	2
0	3	2	2	1	2	1	2	0
13	19	22	22	19	19	18	16	13
3	8	9	6	2	5	4	3	3
4	7	7	7	5	6	6	7	4
1	1	2	2	1	1	0	1	1
5	3	3	2	2	3	5	4	5
0	7	7	6	1	1	1	0	0
7	10	12	9	9	11	7	8	6
0	0	1	1	0	0	0	0	0
3	3	3	5	4	3	4	4	3
2	1	1	1	2	2	2	2	2
1	0	1	0	0	0	1	1	1
0	1	1	1	1	0	1	1	0
2	2	3	3	2	2	2	1	2
0	1	2	1	1	1	0	0	0
1	1	1	1	1	1	1	1	1
0	0	0	0	0	1	0	0	0
0	4	4	4	1	1	1	1	0
1	3	2	2	0	0	1	1	1
7	7	4	5	7	7	7	7	7
5	5	3	3	3	3	4	4	5
1	0	1	1	1	1	0	1	1
4	4	4	3	4	4	4	4	4
3	2	3	2	3	2	3	3	3
2	2	3	3	3	3	2	2	2
1	1	1	1	1	1	1	1	1
0	0	1	1	0	1	0	0	0
1	1	2	2	2	2	2	1	1
1	3	3	2	0	0	2	0	1
1	3	3	2	2	3	2	1	1
15	17	21	21	20	17	17	11	15
1	1	1	0	2	1	1	0	1

5	13	13	13	6	8	7	7	5
1	1	0	1	1	1	1	1	1
2	2	2	1	1	0	1	2	2
1	1	1	1	1	1	1	1	1
3	4	4	3	3	3	4	4	3
0	0	1	1	0	0	0	0	0
0	2	1	2	1	2	1	0	0
1	2	2	1	1	2	2	2	1
0	0	1	2	0	1	0	0	0
3	4	4	4	1	0	0	1	3
0	1	1	0	1	1	0	1	0
2	3	4	4	1	1	1	3	2
9	14	14	12	14	13	12	11	9
4	4	4	3	4	4	4	4	4
1	1	1	1	1	1	1	1	1
9	13	17	17	14	18	12	11	9
2	2	1	1	1	0	1	2	2
4	3	3	4	4	4	4	4	4
1	2	2	1	2	2	2	3	1
1	1	1	0	1	0	0	1	1
9	15	15	12	14	15	14	13	9
3	5	3	4	5	6	5	5	3
6	7	6	6	6	6	7	6	6
4	6	6	6	5	5	4	3	4
13	18	17	17	21	20	16	17	13
2	4	4	4	3	3	4	3	2
3	5	7	7	2	5	4	5	3
1	6	6	6	5	6	4	3	1
3	3	3	5	0	0	0	1	3
2	4	7	6	2	1	3	3	2
13	13	14	15	15	15	13	14	13
1	2	1	2	1	0	2	2	1
2	2	2	1	3	1	2	3	2
1	1	1	1	1	1	1	1	1
2	3	4	4	2	2	2	3	2
2	4	4	3	2	3	3	4	2
5	10	10	11	2	2	5	5	5
7	6	4	4	6	5	7	4	7
7	8	7	6	8	9	9	9	7
3	4	1	4	5	4	5	4	3
1	1	2	3	2	3	2	1	1
0	0	0	2	1	1	1	0	0
0	2	2	1	2	2	1	1	0
1	1	2	1	2	1	1	1	1
1	1	1	0	1	1	0	1	1
7	6	4	6	5	6	5	7	7
0	1	5	5	3	3	3	1	0
10	14	16	18	13	12	12	15	10
5	7	3	7	3	4	5	5	4
12	11	14	12	11	13	11	11	12

2	5	3	5	3	3	5	4	2
2	2	2	3	3	3	3	2	2
6	5	5	5	5	4	6	6	6
6	7	7	8	8	8	8	8	6
1	2	2	1	1	1	2	2	1
1	1	0	1	3	3	3	3	1
2	2	2	2	3	2	3	2	2
1	2	2	2	2	2	2	2	1
5	5	6	6	5	6	4	4	5
3	3	3	3	3	3	2	3	3
1	3	2	3	3	2	3	3	1
1	2	1	2	3	2	3	3	1
0	2	2	2	2	2	2	2	0
2	1	1	1	1	1	1	1	2
0	2	2	4	3	3	2	1	0
2	3	5	4	2	3	3	3	1
2	3	3	4	2	1	0	2	2
1	2	2	2	1	1	2	2	1
2	4	4	5	1	2	3	2	2
0	0	1	0	0	0	0	0	0
2	1	1	1	1	2	1	2	2
1	1	2	1	2	2	2	2	1
0	1	1	1	1	1	1	1	0
4	5	5	5	5	5	5	5	4
2	8	9	9	4	7	6	6	2
2	2	2	2	2	2	3	3	2
1	0	1	1	0	0	0	1	1
0	1	1	0	1	1	1	1	0
0	1	0	1	1	1	1	1	0
1	1	1	1	1	1	1	1	1
6	4	5	6	3	3	3	4	6
2	2	2	2	2	2	1	1	2
6	8	6	7	8	9	9	9	6
15	38	44	42	30	32	30	26	15
2	2	2	2	2	1	2	2	2
2	2	2	1	1	1	2	2	2
1	1	1	1	2	2	2	1	1
2	3	3	3	3	3	2	3	2
1	1	1	1	1	1	1	1	1
2	2	2	2	2	2	2	2	2
0	3	3	3	1	3	3	2	0
2	4	3	2	4	3	2	2	2
0	0	1	1	0	1	0	0	0
1	1	1	1	0	1	1	1	1
4	4	2	2	4	1	3	5	4
3	3	2	1	2	3	3	3	3
0	0	1	3	0	1	0	1	0
3	4	4	5	2	2	3	4	3
1	0	0	0	1	1	1	1	1
4	8	9	8	7	5	7	4	4

2	3	2	2	2	2	2	2	2
7	7	8	9	9	7	6	6	7
4	4	5	5	4	5	4	4	4
1	3	3	3	3	3	1	1	1
2	2	2	4	1	1	1	0	2
6	12	12	11	11	10	8	8	6
1	0	0	0	0	0	1	0	1
3	3	4	5	5	5	3	3	3
1	1	1	1	1	1	1	1	1
0	1	1	0	1	1	1	1	0
1	0	1	1	0	1	0	1	1
3	3	2	3	3	2	3	3	3
1	4	5	4	3	3	3	3	1
11	7	9	5	6	9	9	8	11
6	7	7	6	7	6	6	4	6
0	0	0	1	0	0	0	0	0
1	3	3	4	1	2	1	2	1
1	2	2	2	1	1	1	1	1
0	1	0	0	1	0	0	1	0
6	7	7	8	5	7	6	6	6
1	0	2	1	1	0	1	1	1
0	1	1	2	0	0	0	1	0
1	1	2	1	1	1	1	1	1
4	7	9	9	10	10	8	8	4
0	1	1	1	1	1	1	1	0
8	9	8	7	7	7	8	8	8
1	1	1	0	1	1	1	1	1
1	2	2	1	1	0	1	2	1
4	5	6	5	3	4	5	4	4
1	1	0	1	1	1	1	1	1
0	0	1	1	0	0	0	0	0
3	3	3	3	3	3	3	3	3
1	3	3	2	0	2	1	0	1
2	1	1	2	0	0	1	2	2
0	1	1	1	0	0	1	0	0
2	2	2	2	2	2	1	0	2
3	5	5	5	4	5	3	3	3
2	1	1	2	2	2	1	2	2
0	1	1	1	0	1	0	0	0
1	1	1	1	0	0	0	0	1
2	7	6	6	0	0	2	3	2
4	5	4	5	2	3	2	2	3
4	5	6	6	4	3	4	3	4
1	1	1	1	0	0	0	0	1
2	2	3	3	2	1	2	2	2
1	0	1	0	1	1	1	0	1
3	5	5	6	2	3	3	4	3
2	2	2	2	2	1	2	2	2
1	1	1	0	1	1	1	1	1
3	3	2	2	3	2	2	3	3

1	1	1	1	0	0	1	0	1
1	1	1	1	1	1	1	1	1
1	0	0	0	1	1	1	1	1
1	1	2	2	2	2	2	1	1
10	10	10	14	12	13	10	8	10
8	11	12	11	10	9	10	10	8
2	2	3	2	3	1	2	2	2
1	2	2	1	2	2	2	2	1
1	1	1	1	1	1	1	1	1
3	2	2	2	3	4	3	4	3
1	1	2	2	2	2	1	1	1
3	3	2	3	3	3	3	2	3
1	0	0	0	1	1	1	1	1
1	1	1	1	1	1	1	1	1
3	2	2	3	3	3	3	3	3
2	4	4	4	4	3	4	4	2
1	2	2	2	2	2	2	2	1
5	3	3	2	5	5	4	5	5
1	3	3	3	4	3	3	2	1
1	2	3	2	3	3	1	1	1
1	2	2	2	2	2	2	2	1
1	1	1	1	2	1	1	2	1
2	2	2	2	2	2	2	2	2
2	2	2	2	2	2	2	2	2
0	1	1	2	2	2	1	1	0
3	3	3	3	4	4	4	3	3
14	29	38	36	28	27	22	19	14
0	2	2	2	0	0	0	0	0
3	3	3	3	2	2	2	1	3
1	3	3	4	2	1	1	2	1
19	18	16	17	19	19	19	19	19
1	2	1	2	1	1	1	1	1
1	0	1	2	0	1	0	0	1
4	4	5	4	2	1	4	3	4
0	1	1	2	0	0	0	0	0
2	8	9	8	9	9	7	8	2
1	2	3	3	2	2	2	1	1
2	2	1	2	2	2	2	2	2
0	1	2	2	0	0	0	1	0
2	1	1	1	1	1	2	2	2
0	4	4	4	1	4	3	3	0
2	2	2	2	1	1	2	2	2
2	2	1	2	3	3	2	2	2
3	5	4	4	5	5	5	5	3
2	4	3	4	4	3	2	3	2
2	2	1	1	2	1	1	1	2
9	13	9	11	13	12	13	12	9
0	1	0	1	0	0	0	0	0
2	3	2	3	1	3	3	3	2
0	0	0	1	0	0	0	0	0

1	2	4	5	1	0	2	2	1
0	1	1	1	1	1	1	1	0
0	1	1	1	1	0	1	0	0
0	1	1	0	0	0	0	1	0
3	3	2	2	2	4	3	3	3
12	9	10	12	9	11	11	10	12
10	8	4	7	11	10	11	11	10
33	30	34	30	37	38	35	37	33
2	5	7	7	2	3	3	3	2
2	2	2	2	1	1	2	2	2
4	3	4	3	5	3	3	5	4
4	4	3	4	4	4	4	4	4
7	8	8	4	6	8	8	8	7
0	1	1	1	0	0	0	0	0
2	2	2	1	1	1	2	2	2
5	4	4	3	4	4	4	4	5
1	1	1	1	1	1	1	1	1
3	7	8	9	6	5	4	4	3
0	0	1	1	1	0	0	0	0
2	1	5	5	3	3	1	1	2
1	4	4	5	2	4	2	0	1
0	3	3	3	3	3	2	0	0
1	2	2	2	3	3	2	1	1
0	1	0	0	0	0	1	0	0
2	8	11	11	5	3	1	1	2
2	7	6	7	6	5	3	4	2
4	10	11	9	8	10	6	4	4
3	6	7	6	5	5	5	4	3
5	12	13	12	12	14	9	4	5
9	11	11	9	8	12	12	12	9
12	12	13	11	11	11	12	12	12
2	4	5	5	0	2	1	2	2
27	48	50	51	37	39	30	32	27
2	1	1	0	1	1	2	2	2
2	1	1	1	0	2	2	2	2
0	0	0	1	0	0	0	0	0
1	1	1	1	1	1	1	1	1
2	1	2	1	2	2	2	1	2
2	1	1	1	0	0	1	1	2
0	0	1	0	0	0	1	1	0
1	0	2	2	1	2	2	1	1
4	1	1	1	4	3	3	3	4
3	2	2	1	2	3	2	3	3
2	4	3	2	1	3	2	2	2
4	4	3	4	4	3	4	4	4
1	0	0	0	1	0	1	1	1
3	2	0	1	2	2	2	2	3
0	1	0	1	0	0	0	0	0
3	2	0	1	1	3	3	3	3
1	1	1	1	1	1	1	1	1

2	2	3	3	2	2	3	2	2
0	0	0	1	0	0	0	0	0
3	2	2	3	3	3	3	3	3
2	2	2	2	2	1	2	2	2
4	6	6	6	7	7	5	5	4
18	16	22	19	20	22	19	19	18
7	9	9	11	10	10	10	8	7
4	4	3	3	4	3	4	4	4
4	4	2	4	4	5	4	4	4
1	1	0	0	2	2	1	2	1
0	1	0	1	1	0	0	1	0
1	2	2	2	1	2	2	2	1
1	2	1	2	2	2	1	1	1
1	1	0	0	1	1	1	1	1
3	2	2	1	3	3	3	3	3
0	1	1	1	0	0	0	0	0
0	0	0	1	0	0	0	0	0
2	2	2	1	1	3	1	1	2
0	2	2	1	2	1	2	2	0
1	2	3	3	0	0	1	1	1
1	2	2	2	3	3	2	0	1
0	1	0	0	1	1	1	1	0
1	2	2	3	1	1	1	1	1
1	2	2	3	2	2	1	1	1
1	1	3	2	0	0	0	0	1
0	0	0	1	0	0	0	0	0
2	2	1	0	2	2	2	2	2
19	16	14	15	19	21	20	20	19
2	3	7	7	2	2	2	2	2
1	1	1	1	1	1	1	1	1
4	5	4	3	1	2	3	2	4
2	2	2	2	1	3	3	3	2
1	1	0	1	1	1	1	1	1
1	2	2	2	2	2	1	1	1
1	1	2	1	2	2	1	1	1
3	7	7	7	3	6	6	6	3
2	2	2	2	2	1	2	2	2
1	2	1	2	0	1	2	2	1
3	5	5	6	5	3	4	2	3
3	6	6	7	6	3	5	4	3
5	4	3	4	5	5	5	6	5
9	8	8	9	7	8	9	8	9
6	9	9	9	0	0	0	0	0
4	5	5	5	5	4	5	4	4
5	6	7	8	6	6	4	7	5
5	6	7	8	0	0	0	1	1
9	13	14	14	10	12	11	11	9
20	32	32	32	30	28	23	22	20
2	3	3	3	5	4	4	3	2
2	2	3	2	3	2	2	3	2

13	15	17	15	15	16	14	15	13
1	1	1	1	1	1	1	1	1
1	1	1	1	1	1	1	1	1
1	1	1	1	1	1	1	1	1
3	5	4	4	5	5	3	4	3
3	2	2	2	3	3	3	3	3
1	1	0	1	0	0	0	0	1
4	7	5	5	6	5	7	7	4
3	3	3	3	4	4	4	4	3
6	6	7	6	6	6	6	6	6
5	6	3	4	5	5	5	6	5
3	1	2	2	3	3	3	2	3
21	25	24	23	24	23	21	20	19
0	2	2	2	0	0	0	0	0
2	3	3	2	1	3	3	3	2
3	4	4	4	4	5	5	5	3
1	1	2	2	3	3	3	2	1
6	7	8	6	7	8	7	7	6
1	0	0	0	1	1	1	1	1
20	24	27	22	26	25	27	25	20
2	2	2	3	2	3	3	2	2
4	6	5	5	5	5	5	5	4
1	1	1	1	2	1	0	1	1
4	5	4	5	5	5	5	4	4
1	1	1	1	1	0	1	1	1
0	1	1	2	2	2	1	2	0
1	1	0	1	2	0	1	0	1
2	3	3	3	2	2	3	2	2
1	0	1	1	0	0	1	0	1
0	2	1	1	2	1	1	1	0
5	5	4	3	2	2	2	2	2
5	6	4	2	5	6	5	6	5
6	10	10	10	6	11	11	11	6
2	1	1	1	2	2	2	0	2
0	0	0	1	0	1	1	1	0
1	1	0	0	1	1	1	1	1
7	16	18	19	10	19	13	11	7
4	5	7	7	2	2	2	3	3
2	5	6	6	3	5	4	3	2
0	4	5	5	0	0	1	2	0
1	2	2	0	2	2	1	1	1
0	2	1	2	0	0	0	0	0
1	3	3	2	0	0	1	2	1
2	5	5	3	2	2	4	3	2
4	6	7	7	3	5	4	4	4
3	7	7	6	3	4	4	4	3
1	2	2	2	0	0	1	0	1
5	9	9	9	2	3	2	4	5
1	7	8	8	4	4	3	2	1
2	6	6	6	4	2	3	3	2

3	4	7	6	5	5	4	3	3
3	7	7	6	6	6	6	5	3
2	3	3	3	2	2	2	2	2
1	1	3	2	2	0	0	1	1
0	3	4	2	3	3	3	2	0
6	7	8	8	6	6	6	5	6
0	2	2	2	0	1	2	2	0
1	1	1	1	0	1	1	1	1
3	6	7	6	2	1	4	4	3
0	1	1	0	0	0	1	0	0
11	14	15	13	9	10	7	10	11
0	2	1	2	1	0	1	0	0
2	4	3	3	0	0	2	3	2
3	5	6	6	1	3	3	4	3
0	2	2	2	1	0	0	1	0
6	7	7	7	6	5	5	4	6
1	3	4	4	2	3	2	1	1
2	4	5	5	4	4	2	2	2
1	1	1	1	1	1	1	1	1
2	3	3	4	3	3	1	3	2
0	1	1	1	0	0	0	1	0
1	1	1	2	1	2	1	1	1
1	2	2	2	2	1	0	0	1
4	6	6	5	2	1	3	1	4
6	10	10	10	5	4	6	7	6
1	2	1	2	2	2	1	1	1
5	8	7	8	8	10	9	8	5
8	10	12	13	5	5	6	4	5
5	8	8	10	5	6	5	5	5
5	9	9	9	9	8	8	7	5
5	6	5	6	6	7	7	6	5
3	2	4	6	2	2	2	2	3
5	9	9	9	6	5	7	7	5
2	8	11	9	0	0	3	4	2
4	12	13	13	5	5	4	6	4
1	5	7	7	5	5	4	2	1
1	2	3	3	1	1	1	2	1
5	5	4	4	6	5	6	4	5
13	14	19	16	13	13	13	12	13
24	40	41	40	36	31	31	28	24
3	3	4	4	0	0	2	2	3
2	2	2	2	3	3	3	3	2
5	4	4	6	4	5	6	4	5
2	2	3	3	3	2	2	3	2
6	6	5	5	6	5	6	6	6
1	0	0	0	0	0	0	0	1
64	75	79	77	69	69	66	69	64
36	42	46	41	27	29	32	34	32
1	1	2	2	1	0	1	0	1
3	6	6	5	4	5	5	3	3

1	4	4	5	0	1	2	2	1
4	5	5	5	5	5	5	5	4
4	4	4	5	5	5	5	5	4
2	1	2	2	2	0	2	2	2
4	4	4	4	4	4	4	3	4
2	2	1	2	2	2	2	2	2
1	1	0	0	1	1	1	1	1
14	19	21	22	13	14	17	15	14
5	6	6	7	2	1	2	2	2
6	9	7	7	9	9	9	9	6
1	1	1	1	1	1	1	1	1
5	4	4	4	4	4	4	4	5
6	8	6	8	7	8	8	7	6
0	2	2	2	0	1	0	0	0
4	4	3	4	4	3	4	2	4
2	2	2	1	2	2	2	2	2
2	3	3	2	0	1	2	3	2
6	7	6	6	7	7	8	7	6
2	1	1	1	2	2	2	2	2
6	6	6	5	11	12	9	5	6
0	0	1	1	1	1	0	0	0
5	6	5	6	7	7	4	5	5
1	2	2	1	0	0	1	2	1
0	0	1	1	0	0	0	0	0
1	1	1	1	0	0	1	1	1
0	1	1	1	0	0	0	0	0
2	4	2	5	0	1	1	2	2
4	11	13	14	8	8	6	6	4
3	9	10	7	4	4	5	5	3
13	14	13	12	16	15	15	15	13
16	17	15	15	18	18	15	16	16
1	1	1	2	0	0	0	0	1
0	1	1	1	0	0	0	0	0
0	1	3	1	0	0	0	0	0
6	9	9	10	9	10	8	8	6
3	4	3	4	4	4	3	4	3
3	6	5	4	5	5	4	3	3
1	2	2	2	0	0	1	1	1
1	1	1	1	0	1	1	1	1
1	3	4	4	1	2	3	3	1
3	2	1	1	1	1	1	1	3
2	5	5	5	0	1	3	2	2
3	8	8	8	3	4	5	5	3
9	10	13	14	12	12	12	11	9
0	1	1	0	1	1	1	1	0
7	6	6	6	7	8	7	7	7
2	2	2	1	2	1	2	2	2
2	3	2	4	3	2	2	4	2
1	1	1	0	1	1	1	1	1
0	1	1	1	0	0	0	1	0

2	3	3	3	0	0	3	2	2
1	1	1	1	0	1	1	1	1
4	4	4	5	5	6	7	6	4
3	6	6	7	4	4	3	4	3
5	4	5	2	5	5	4	4	5
2	1	1	1	3	2	1	1	2
1	2	1	1	1	1	1	1	1
1	3	3	2	2	2	3	4	1
1	1	1	1	1	1	1	0	1
1	1	1	0	1	1	1	1	1
3	1	1	2	2	3	4	3	3
1	1	1	1	1	1	1	1	1
0	0	0	0	0	0	0	1	0
2	0	1	0	2	1	0	1	2
1	0	0	0	2	2	2	2	1
0	0	0	1	1	1	0	0	0
3	3	5	2	4	5	3	3	3
1	0	1	1	1	1	1	1	1
2	2	2	2	2	1	2	2	2
6	6	5	4	5	4	6	6	6
1	1	1	0	1	1	1	0	1
0	1	1	0	1	1	1	1	0
2	2	2	2	1	2	1	1	2
3	8	7	6	3	4	3	2	1
5	8	8	5	4	4	4	4	3
3	4	3	1	5	4	3	4	3
6	4	5	6	4	3	3	4	4
8	7	8	7	7	6	7	7	8
5	7	7	6	6	6	6	4	5
2	2	2	2	2	2	2	2	2
4	3	4	3	3	3	3	3	3
2	2	1	2	1	1	1	1	1
0	0	0	0	1	0	0	1	0
6	11	14	13	10	12	11	7	6
2	1	2	2	1	2	2	2	2
1	0	0	0	0	0	0	1	1
1	2	2	2	2	2	2	2	1
4	3	3	2	3	3	3	2	4
4	6	3	5	4	6	5	5	4
5	7	4	6	8	9	9	7	5
1	1	0	1	1	1	1	1	1
15	21	29	23	18	18	19	18	15
7	18	14	16	6	10	11	12	6
8	9	10	10	1	1	1	1	1
18	25	26	25	18	17	16	18	14
1	1	1	1	1	1	1	1	1
11	17	18	20	9	13	13	12	11
8	10	9	9	11	10	9	9	8
11	13	13	12	12	13	13	13	11
18	26	34	33	11	13	14	12	9

25	34	37	36	31	30	34	31	25
9	10	10	10	0	1	1	1	1
2	4	4	4	0	0	2	2	2
1	2	2	1	0	0	0	0	1
1	2	1	3	3	3	1	2	1
1	1	1	1	0	0	1	1	1
3	3	3	4	5	5	3	3	3
0	1	1	1	0	0	0	2	0
2	3	3	2	3	3	2	2	2
1	1	1	1	1	1	1	1	1
2	4	4	5	0	0	1	2	2
5	4	4	4	3	2	4	3	5
2	4	3	4	1	2	3	3	2
2	4	5	5	5	4	4	3	2
3	6	10	8	1	3	4	4	3
3	6	7	6	6	4	3	4	3
2	2	5	7	2	1	2	3	2
1	2	3	2	1	2	1	1	1
8	9	11	9	9	9	8	8	8
9	9	9	10	8	8	7	7	7
8	8	6	9	3	3	3	3	3
3	6	7	4	7	7	4	4	3
9	12	15	16	10	12	7	6	9
9	8	11	10	10	8	3	4	9
9	19	22	22	19	19	18	15	9
10	15	15	11	10	10	9	8	10
10	12	14	14	7	8	6	8	6
15	20	22	21	18	21	16	16	15
3	3	2	3	2	2	2	3	3
6	10	11	10	12	10	8	10	6
9	14	16	12	7	7	10	12	9
8	12	13	11	10	12	14	12	8
4	5	4	6	6	6	6	7	3
8	13	10	11	12	14	14	12	8
2	1	1	2	2	2	2	2	2
0	4	5	4	3	3	3	1	0
0	0	2	1	0	1	0	1	0
0	1	1	1	0	0	0	0	0
3	3	3	2	2	3	3	3	3
1	0	0	1	0	0	1	0	1
2	3	4	4	2	2	1	2	2
2	1	0	0	1	1	1	1	2
0	1	1	1	0	0	1	0	0
2	3	4	2	2	3	3	3	2
3	3	2	3	2	3	4	4	3
9	7	8	9	7	9	9	9	9
8	8	8	9	1	1	1	1	1
6	7	5	7	5	6	6	6	5
1	1	2	2	1	1	1	1	1
5	5	4	5	5	5	5	5	5

4	4	4	4	2	2	2	2	2
2	2	0	2	0	0	0	0	1
4	4	3	4	1	1	1	1	1
5	8	7	8	1	0	1	1	0
5	7	5	7	1	1	1	1	1
5	7	5	6	1	0	1	0	1
6	8	6	8	7	8	8	7	6
4	4	4	4	5	4	5	5	4
8	7	7	8	8	8	8	8	8
4	7	7	7	4	3	5	5	4
2	4	5	4	1	1	0	0	1
10	11	11	10	2	2	2	2	1
11	11	11	11	12	10	10	10	11
1	2	2	2	1	1	1	1	0
7	8	8	7	2	2	2	2	2
8	8	9	9	5	5	5	5	5
7	7	8	8	1	2	1	1	1
4	7	7	7	5	5	6	6	4
4	8	6	5	4	4	4	5	4
1	1	1	1	1	1	1	1	1
4	4	4	3	5	5	5	4	4
0	2	1	1	1	2	2	2	0
4	6	7	8	4	2	4	7	4
5	5	4	3	5	5	5	5	5
3	5	5	5	1	0	2	5	3
5	6	6	6	7	7	8	6	5
0	1	1	1	1	1	1	1	0
5	8	8	8	6	7	6	6	5
3	3	5	4	4	3	4	3	3
1	1	2	2	1	1	1	1	1
0	2	1	1	1	2	1	1	0
7	12	13	12	10	12	10	10	7
1	7	8	9	1	0	3	1	1
1	2	3	4	1	1	1	1	1
1	2	1	0	1	0	0	0	1
2	2	2	1	1	2	2	2	2
1	1	2	2	1	1	1	1	1
1	2	1	2	2	2	2	2	1
2	0	1	1	1	1	2	2	2
1	2	1	0	0	1	2	2	1
4	8	9	8	5	5	4	6	4
1	1	2	2	1	0	0	1	1
10	10	12	11	10	12	10	8	10
6	5	2	3	6	6	5	5	6
4	4	4	4	4	4	3	1	4
3	2	2	3	3	3	3	3	3
1	1	1	0	1	1	1	1	1
11	12	13	10	13	13	13	12	11
4	3	4	3	5	4	4	4	4
16	17	16	15	20	20	19	15	16

3	3	2	3	5	5	4	5	3
2	2	2	2	2	2	2	2	2
13	12	12	11	13	14	13	14	13
31	30	32	31	29	32	31	33	31
1	2	2	2	2	2	2	2	1
3	2	1	2	2	2	2	2	3
0	2	2	3	0	0	1	1	0
0	1	1	1	0	0	1	1	0
3	5	4	5	4	5	5	3	3
3	7	6	8	3	6	6	5	3
11	15	18	18	7	9	11	11	11
0	0	0	1	0	1	1	0	0
0	1	1	1	0	0	0	0	0
6	4	5	5	6	6	5	6	6
2	3	2	3	2	2	3	2	2
5	8	12	11	6	6	6	8	5
4	7	9	9	9	9	6	5	4
6	7	6	6	6	7	7	7	6
1	1	1	1	1	1	1	1	1
14	16	19	19	15	17	17	17	14
9	6	7	6	8	5	6	9	9
1	1	1	1	1	1	1	1	1
5	6	5	4	1	3	2	2	3
24	21	21	19	25	25	25	22	22
13	13	15	12	7	6	5	6	6
22	21	23	23	22	21	20	20	21
30	29	32	25	30	32	31	30	30
3	1	4	3	0	0	2	2	3
4	6	5	5	2	3	2	3	1
4	6	6	5	0	0	0	0	0
21	24	25	25	21	22	21	20	19
18	19	18	15	7	11	11	11	10
15	16	14	12	1	0	1	1	1
33	33	31	34	34	32	32	31	33
29	32	29	29	30	31	30	28	27
0	3	3	3	2	2	1	1	0
1	3	2	4	0	0	1	1	1
5	5	6	6	5	6	3	3	5
1	1	0	0	1	1	1	1	1
4	3	4	4	3	5	5	5	4
1	0	0	0	1	1	0	1	1
4	4	3	2	4	4	4	3	4
0	1	1	0	0	0	0	0	0
1	2	2	2	1	1	2	0	1
6	6	6	4	4	6	6	6	6
8	10	8	8	4	4	4	2	2
11	11	11	12	11	11	11	11	11
4	3	4	2	2	4	4	3	4
31	26	26	23	24	28	27	26	30
23	21	21	17	24	23	22	22	21

0	0	0	1	1	1	1	1	0
1	0	0	0	2	3	1	1	1
4	5	5	3	6	6	6	6	4
5	4	3	2	5	5	5	4	5
6	4	5	4	7	5	7	7	6
0	1	1	1	0	0	1	1	0
3	5	6	5	3	6	4	3	3
3	2	0	2	3	3	3	3	3
4	3	3	5	4	4	4	3	4
0	2	1	3	0	0	0	0	0
1	1	1	2	2	2	2	1	1
1	1	1	1	2	2	2	1	1
4	4	3	4	5	5	5	4	4
19	27	26	27	25	27	27	25	19
2	5	4	3	2	1	1	2	2
1	1	1	1	1	1	1	1	1
2	2	0	3	1	1	1	1	2
4	7	7	8	7	7	7	7	4
3	4	4	5	4	3	2	3	3
2	1	0	0	1	2	3	2	2
2	4	4	4	1	0	2	2	2
3	3	2	3	3	3	3	3	3
3	6	6	5	1	1	4	5	3
4	6	6	7	4	4	5	5	4
8	10	12	9	5	3	7	9	8
3	1	0	1	2	2	2	2	3
6	13	13	13	7	9	8	7	6
4	8	8	9	4	2	5	6	4
6	4	2	3	6	5	6	6	6
2	3	3	2	4	4	4	2	2
10	10	10	11	11	11	10	10	10
4	3	4	4	3	2	2	2	2
0	3	3	3	1	2	0	1	0
5	4	2	3	4	3	4	5	5
1	0	0	0	1	0	1	1	1
1	2	3	2	1	2	1	1	1
0	2	4	3	0	0	1	1	0
1	1	1	1	0	1	1	1	1
6	7	8	8	5	5	6	6	6
1	3	3	3	3	3	3	3	1
1	2	0	1	1	2	2	2	1
1	0	0	0	1	3	1	1	1
1	1	1	0	1	1	1	1	1
4	3	4	3	3	4	3	3	4
1	1	0	0	1	1	1	1	1
4	4	4	4	4	3	4	4	4
0	2	2	2	2	2	2	2	0
1	2	2	1	2	2	2	1	1
2	2	2	2	2	1	2	2	2
3	4	4	4	1	1	2	4	3

2	2	2	2	3	3	3	3	2
4	2	0	1	3	2	2	2	4
0	1	0	1	0	1	1	1	0
1	1	1	1	1	1	1	1	1
2	2	2	2	2	1	2	2	2
9	10	8	11	8	7	10	11	9
4	4	5	6	1	2	3	4	4
16	17	16	17	18	18	17	17	16
3	3	3	1	4	4	3	4	3
0	1	1	1	0	0	0	0	0
0	0	0	1	0	0	0	0	0
2	1	1	1	1	1	1	1	2
4	4	1	3	4	4	4	2	4
0	1	1	0	0	0	0	0	0
0	1	1	2	0	0	0	1	0
7	12	15	15	14	15	11	9	7
3	2	2	4	4	2	3	3	3
8	5	4	3	3	5	6	7	8
1	0	0	0	0	0	0	0	1
1	2	1	1	1	2	2	1	1
2	0	0	0	0	1	2	1	2
1	1	1	1	1	1	1	1	1
4	4	4	4	3	3	3	3	3
4	4	4	4	4	4	4	4	4
1	0	0	0	1	1	1	0	1
2	4	4	3	3	4	4	4	2
2	2	2	2	2	1	1	1	2
1	1	2	1	1	1	1	1	1
2	3	2	2	3	2	4	4	2
4	3	3	2	4	3	3	4	4
2	2	2	1	2	2	2	2	2
0	1	1	1	0	0	1	1	0
3	3	3	2	3	3	3	3	3
1	2	2	2	0	0	1	1	1
2	4	5	6	3	4	3	3	2
1	1	1	1	0	1	1	0	1
2	3	2	1	3	2	3	3	2
1	1	1	1	1	1	1	1	1
1	0	1	1	0	1	1	1	1
1	1	0	0	1	1	1	1	1
0	2	2	2	1	2	1	2	0
2	2	0	1	1	2	2	2	2
3	3	4	2	5	4	4	4	3
1	1	0	1	1	1	1	1	1
14	14	10	10	15	15	13	13	14
1	1	1	1	1	1	1	1	1
1	2	1	1	1	2	1	2	1
0	1	1	0	0	0	0	0	0
3	5	6	7	0	1	2	2	3
1	2	3	3	0	1	1	1	1

2	1	2	0	2	2	2	2	2
0	1	1	1	1	0	0	1	0
2	3	3	3	2	2	2	2	2
1	1	1	1	1	1	1	1	1
1	5	5	5	5	5	5	5	1
1	3	3	1	2	2	2	2	1
3	4	2	3	4	4	4	4	3
3	4	4	5	2	4	4	3	3
1	2	3	2	1	1	0	0	0
1	1	2	2	1	2	1	1	1
2	1	1	1	1	2	2	2	2
0	1	1	1	2	2	0	0	0
5	5	4	4	5	4	6	6	5
1	1	1	1	1	1	1	1	1
1	2	2	2	2	2	2	2	1
0	1	1	1	1	1	1	1	0
5	5	5	5	5	5	5	5	5
2	4	2	3	3	2	1	3	2
6	8	7	7	9	7	7	9	6
5	8	6	6	7	8	7	7	5
0	0	1	1	0	1	0	0	0
1	0	0	1	1	1	1	1	1
1	3	3	3	3	3	3	3	1
5	2	2	2	3	3	5	5	5
2	2	1	4	3	2	3	1	1
0	1	2	2	0	0	0	0	0
4	5	5	5	5	4	5	5	4
1	1	2	1	1	1	1	1	1
3	3	3	3	3	3	3	3	3
9	15	19	18	7	5	10	11	9
2	2	2	2	2	2	2	2	2
2	2	1	2	3	3	3	2	2
1	1	1	2	1	1	1	1	1
5	3	4	4	4	4	4	5	5
2	3	2	2	2	2	3	3	2
1	1	0	0	0	1	1	1	1
1	1	1	0	1	1	1	1	1
3	6	7	5	5	3	3	4	3
1	2	2	1	2	2	0	1	1
10	14	12	13	9	13	13	12	10
2	2	1	1	2	2	2	1	2
2	3	2	3	3	3	4	2	2
1	0	0	1	1	1	1	1	1
1	2	2	2	2	2	2	2	1
35	43	46	44	44	46	44	43	35
1	1	0	1	1	1	0	1	1
6	7	8	7	8	7	6	7	6
2	1	0	1	1	2	2	1	2
4	7	8	8	7	8	6	6	4
7	11	13	12	1	1	1	1	1

9	10	10	9	0	1	0	0	0
7	9	8	8	2	2	0	0	1
70	87	88	88	99	94	75	68	70
2	2	2	2	0	0	1	2	2
5	7	7	7	5	5	6	7	5
0	2	2	2	0	0	0	0	0
2	0	1	1	2	2	2	2	2
5	10	11	8	12	11	10	12	5
0	2	3	2	4	3	2	1	0
3	2	1	1	2	2	1	2	3
2	4	4	4	3	2	2	3	2
3	4	3	3	4	4	4	3	3
1	1	1	1	1	1	1	1	1
1	1	0	0	1	1	1	1	1
0	0	1	0	1	1	0	0	0
1	1	1	2	1	1	1	1	1
2	1	0	1	2	2	2	2	2
0	0	0	2	0	1	2	2	0
1	1	0	0	1	1	1	1	1
0	1	1	1	1	1	1	1	0
4	4	4	5	5	5	5	5	4
0	1	1	0	0	0	0	0	0
3	2	1	1	2	1	3	3	3
3	1	2	3	4	3	3	3	3
0	1	2	2	1	0	1	1	0
2	2	2	2	2	2	2	2	2
1	0	0	1	0	0	0	1	1
0	1	0	0	1	0	0	0	0
2	2	2	1	2	2	1	2	2
2	3	3	2	0	0	0	0	2
2	0	1	0	2	1	2	2	2
109	113	113	120	101	107	120	120	109
0	0	1	1	1	0	1	0	0
3	2	2	2	2	3	3	2	3
12	21	24	23	25	25	20	17	12
12	21	24	23	1	1	1	1	1
2	2	1	1	2	2	2	2	2
4	7	6	6	8	8	8	8	4
4	4	4	3	4	4	4	3	4
3	4	4	4	4	4	4	4	3
1	1	1	0	1	1	1	1	1
2	3	3	3	2	3	3	3	2
0	3	3	3	1	0	0	2	0
3	2	0	1	3	3	3	2	3
2	7	6	7	3	3	4	4	2
0	1	1	1	0	0	0	1	0
1	0	0	0	1	1	1	1	1
6	5	6	5	6	6	6	6	6
2	0	1	2	0	0	0	0	2
8	11	12	12	7	10	9	6	7

2	4	4	4	4	3	4	4	2
1	2	1	1	1	1	1	1	1
4	5	6	6	0	0	3	4	4
1	1	1	0	1	1	1	1	1
1	3	3	3	0	0	0	1	1
2	2	3	4	2	2	1	2	2
1	1	1	1	0	0	1	1	1
1	1	1	1	0	1	1	0	1
2	2	3	3	2	3	3	1	2
3	5	4	3	2	3	3	3	3
38	31	28	35	33	30	38	36	38
5	4	2	3	5	4	5	5	5
4	5	4	3	5	4	4	4	4
4	4	2	3	3	4	4	4	4
8	10	10	10	10	9	10	10	8
4	6	4	3	5	5	5	5	4
5	8	5	7	7	5	7	5	5
5	4	4	3	2	4	4	4	5
1	1	1	1	1	1	1	1	1
2	1	0	2	2	2	2	2	2
5	5	4	4	5	5	5	5	5
4	7	7	7	7	7	5	6	4
5	3	3	2	5	4	5	4	5
1	2	2	2	2	2	2	3	1
3	3	0	0	3	4	3	2	3
0	0	1	1	0	0	0	0	0
5	6	2	3	6	4	4	5	5
2	3	5	3	1	3	3	2	2
2	3	3	3	0	0	1	0	2
1	1	0	1	1	1	1	1	1
9	9	8	7	5	6	9	8	9
2	0	1	1	2	2	0	1	2
3	2	2	3	2	1	4	2	3
3	3	1	1	1	2	3	4	3
1	0	0	0	0	0	0	0	1
6	8	4	9	6	8	8	7	6
0	0	0	0	0	0	1	1	0
2	2	2	2	2	2	2	2	2
0	1	1	1	0	1	1	1	0
0	1	0	0	0	1	1	1	0
7	9	10	8	8	9	10	10	7
7	9	10	10	9	10	10	9	7
2	1	1	1	2	2	1	2	2
14	14	15	17	15	15	15	16	14
3	3	1	2	2	2	3	3	3
19	19	23	20	17	17	16	17	19
0	0	1	0	0	0	0	0	0
11	10	11	11	9	10	10	10	11
1	1	0	0	1	1	1	1	1
1	3	3	1	3	3	2	3	1

5	4	5	3	4	4	5	4	5
3	3	2	1	2	2	3	3	3
4	3	3	4	4	4	2	3	4
11	10	9	12	9	8	9	8	11
0	1	1	1	0	1	0	1	0
2	4	5	4	0	0	0	0	0
4	6	8	6	3	5	3	4	4
2	3	3	3	3	3	3	2	2
3	4	4	3	3	1	3	4	3
1	2	2	1	0	0	0	0	1
1	1	1	1	1	1	1	1	1
0	1	1	1	1	0	1	1	0
3	2	2	2	3	3	3	3	3
1	1	1	1	1	1	1	1	1
7	6	6	7	6	6	6	6	7
1	1	1	1	1	1	1	1	1
0	0	0	1	1	1	0	0	0
0	0	0	0	1	1	1	1	0
7	8	9	9	10	9	9	9	7
1	1	0	1	0	0	1	0	1
3	4	5	4	5	5	4	3	3
1	0	0	0	0	0	1	1	1
1	2	2	3	3	3	3	1	1
1	1	1	1	1	1	1	1	1
0	1	1	1	1	1	1	1	0
1	3	3	3	1	0	0	1	1
5	6	9	8	2	2	6	7	5
1	2	1	1	1	1	1	1	1
2	2	1	1	2	1	2	2	2
6	8	8	8	1	1	1	1	0
8	9	10	11	10	10	9	8	8
4	8	9	9	9	9	7	9	4
1	1	1	1	1	1	1	1	1
3	0	0	1	0	0	1	1	2
2	2	3	3	0	1	1	1	2
2	2	2	2	1	1	2	2	2
0	3	3	3	0	0	1	1	0
9	10	11	9	5	8	6	9	9
1	1	1	1	1	1	1	1	1
1	1	1	1	1	0	0	1	1
0	0	0	1	0	0	0	0	0
1	2	2	2	2	2	2	2	1
1	1	0	1	1	0	0	1	1
5	6	7	5	2	3	7	6	5
1	1	2	1	3	2	2	1	1
12	12	13	13	11	11	10	11	12
6	7	8	7	3	3	4	3	5
7	9	9	10	8	8	7	3	5
5	6	6	6	6	6	6	6	5
8	8	9	8	6	7	8	7	8

1	1	1	0	1	1	1	1	1
0	0	0	0	0	1	1	0	0
2	1	0	1	1	2	1	2	2
13	14	12	13	15	14	16	15	13
0	1	0	1	1	1	1	1	0
5	4	3	2	1	3	3	3	5
1	1	0	0	1	1	1	1	1
1	2	2	2	2	2	2	1	1
2	1	2	3	1	2	2	1	2
4	8	9	9	3	3	2	2	4
2	1	1	2	0	1	2	2	2
6	8	8	7	7	7	6	6	6
1	1	0	1	1	1	1	1	1
3	3	6	6	0	0	2	1	3
1	0	0	1	1	1	1	0	1
2	2	3	3	1	1	1	1	2
2	3	4	4	2	2	2	3	2
0	1	1	1	1	1	0	1	0
3	3	3	2	3	3	3	3	3
2	5	7	6	0	0	2	2	2
1	1	1	1	0	0	1	1	1
11	14	14	13	10	13	13	12	11
5	8	8	11	5	4	5	5	5
1	1	0	1	1	1	1	1	1
1	1	1	1	1	1	2	2	1
0	1	1	1	0	1	1	0	0
2	6	8	7	2	2	3	4	2
1	1	1	1	0	0	0	1	1
3	3	3	2	3	3	3	3	3
0	0	0	1	0	0	0	0	0
0	0	2	2	1	2	2	1	0
2	1	2	1	1	1	1	1	2
7	6	7	6	4	8	8	8	7
3	2	2	1	3	3	3	3	3
7	8	9	11	3	3	6	7	7
1	6	6	5	3	3	2	4	1
8	17	17	19	7	7	10	12	8
7	15	15	14	2	2	2	3	3
3	5	5	5	2	3	4	5	3
0	0	1	1	0	0	0	0	0
1	1	1	1	1	1	1	1	1
69	73	75	76	86	88	87	79	69
2	1	2	2	1	0	2	2	2
1	1	0	0	1	1	1	1	1
4	5	5	5	6	5	6	5	4
12	13	15	14	13	14	12	11	12
1	0	0	0	1	1	1	1	1
5	8	8	8	6	7	5	6	5
4	6	8	7	3	4	4	4	2
6	7	7	6	2	2	7	7	6

9	13	17	16	14	16	12	12	9
2	2	2	2	1	1	1	2	2
3	6	7	6	2	2	1	1	1
0	2	1	2	0	0	0	0	0
1	1	1	1	1	1	1	1	1
1	2	2	2	2	1	1	1	1
5	7	7	7	7	8	8	7	5
6	7	6	5	8	8	8	8	6
2	3	1	2	3	2	3	3	2
4	9	10	10	6	7	7	6	4
3	5	7	6	5	5	3	1	2
2	1	1	2	3	2	2	1	2
1	1	0	0	1	1	1	1	1
1	1	1	1	0	0	0	1	1
0	0	1	0	0	0	0	0	0
0	1	1	0	0	0	0	0	0
35	34	34	36	32	35	33	33	35
7	7	8	10	6	7	7	7	7
2	2	2	2	2	2	1	1	2
5	4	5	6	4	7	4	5	5
6	12	12	12	9	10	9	8	6
1	1	0	1	0	1	1	0	1
3	5	6	4	5	5	5	5	3
0	1	1	1	1	0	0	0	0
2	1	1	1	1	1	1	1	2
7	5	5	4	4	5	6	7	7
0	4	4	4	4	4	1	0	0
4	6	4	4	6	5	5	5	4
0	1	1	1	1	0	1	1	0
11	11	11	9	10	8	13	14	11
1	1	1	1	1	1	1	1	1
11	16	17	18	15	16	13	12	11
7	6	7	5	3	4	7	6	7
1	0	0	0	1	2	1	2	1
2	6	6	5	7	6	4	2	2
2	2	2	2	2	2	2	2	2
9	15	16	15	9	7	5	7	4
1	1	1	1	0	1	1	1	1
1	2	2	2	2	1	2	2	1
2	1	0	0	1	1	2	1	2
3	2	1	2	1	2	1	0	2
1	0	0	0	0	0	0	0	1
1	1	1	1	1	1	1	1	1
2	2	0	1	2	1	2	2	2
4	3	4	4	3	4	4	5	4
7	7	5	5	8	8	7	8	7
3	3	1	2	3	2	3	4	3
2	2	2	2	2	2	2	2	2
4	5	6	5	7	6	6	6	4
7	9	9	9	7	7	7	8	7

4	6	5	6	4	5	5	5	4
0	0	0	1	0	0	0	0	0
0	2	2	2	0	2	1	0	0
9	12	12	11	12	11	11	12	9
10	9	15	13	12	14	13	13	10
6	6	6	7	4	2	7	5	6
4	12	14	14	7	7	6	7	4
4	3	3	3	6	5	5	5	4
1	1	0	2	2	2	1	1	1
1	2	3	2	2	1	2	1	1
3	3	6	6	4	4	4	3	3
1	2	1	2	2	2	2	2	1
1	3	3	3	3	3	3	3	1
1	3	4	2	4	3	0	1	1
1	2	2	1	1	2	2	1	1
2	5	6	4	1	0	4	1	2
1	1	1	2	2	2	2	1	1
3	4	5	4	0	0	4	3	3
1	2	1	1	1	0	1	2	1
0	3	2	3	0	0	0	0	0
0	1	1	1	0	1	1	1	0
0	2	2	1	0	1	2	0	0
3	4	5	5	4	3	3	3	3
5	6	7	6	2	4	6	5	5
3	6	10	10	3	5	5	4	3
2	3	2	3	0	0	1	1	2
5	5	8	7	4	5	3	2	5
3	6	9	8	6	5	6	5	3
0	2	3	1	0	0	1	1	0
0	1	1	1	0	0	1	1	0
3	5	5	4	4	4	5	4	3
0	2	2	2	0	0	0	0	0
1	2	1	2	1	2	2	2	1
1	1	1	1	0	0	1	1	1
3	7	7	6	3	2	2	3	3
2	3	3	3	3	2	3	3	2
1	2	2	1	1	1	1	1	1
0	1	1	2	0	0	0	0	0
3	2	2	1	1	0	2	1	3
5	7	5	6	4	5	5	4	5
1	2	2	1	2	2	2	2	1
1	1	1	1	0	0	0	0	1
1	1	2	1	1	0	1	1	1
3	3	4	5	3	2	2	3	3
1	2	1	2	2	1	2	2	1
3	3	3	3	0	0	2	3	3
0	1	1	1	1	1	1	1	0
1	1	1	1	0	0	1	1	1
3	5	6	5	3	3	3	4	3
3	5	5	5	4	4	5	5	3

4	5	5	5	0	1	1	1	4
12	19	17	17	21	21	18	19	12
17	19	20	20	21	21	19	19	17
11	8	11	8	12	12	12	13	11
9	9	9	8	12	9	11	9	9
1	0	0	0	1	0	1	1	1
3	1	1	1	2	2	4	4	3
7	6	7	4	8	8	7	8	7
1	0	0	0	1	0	1	1	1
1	1	1	1	1	1	1	2	1
2	2	2	3	3	3	2	2	2
1	0	1	1	1	1	0	1	1
4	5	4	4	3	5	4	3	4
1	3	3	3	0	1	3	0	1
16	18	15	14	19	20	18	18	16
0	1	1	1	1	1	0	0	0
2	2	3	3	2	2	1	2	2
1	1	1	1	2	1	2	1	1
0	0	0	0	0	0	1	1	0
1	1	1	0	1	0	0	0	1
2	2	2	2	2	2	2	2	2
1	2	2	2	1	0	0	0	1
1	2	2	2	1	2	2	2	1
1	0	0	0	1	1	1	1	1
2	1	1	1	2	2	2	2	2
2	0	1	2	0	0	0	1	2
2	2	2	2	1	2	2	2	2
4	5	4	4	3	4	5	5	4
2	2	2	1	2	2	1	1	2
1	0	0	1	1	0	0	0	1
1	1	0	1	1	1	1	1	1
3	6	8	9	3	4	4	4	3
1	4	4	4	0	1	1	2	1
0	1	2	0	0	2	1	1	0
1	1	1	1	1	1	1	1	1
1	2	2	2	1	0	2	2	1
0	0	1	1	0	0	0	0	0
2	4	5	3	1	1	1	1	2
12	9	9	8	10	10	9	10	12
0	2	2	1	1	0	1	0	0
5	6	6	7	5	4	4	4	5
1	1	1	1	1	1	1	1	1
6	3	3	3	4	4	3	5	6
2	2	2	2	1	1	1	2	2
1	2	3	3	2	3	2	2	1
3	4	4	4	4	4	4	4	3
0	1	1	1	0	0	0	1	0
1	4	5	4	4	5	5	3	1
1	2	2	2	1	2	2	2	1
2	3	3	5	5	4	3	3	2

1	2	2	2	2	2	2	2	1
4	4	3	4	4	3	3	4	4
4	6	7	7	1	0	2	4	4
3	1	1	3	3	3	3	4	3
1	1	1	1	1	1	1	1	1
1	1	1	1	1	2	2	1	1
3	3	4	4	3	2	3	3	3
2	2	2	1	2	1	2	2	2
1	1	1	1	0	1	1	0	1
4	3	4	4	4	4	4	4	4
3	2	2	2	3	3	3	3	3
1	1	0	0	1	1	1	1	1
0	0	1	1	1	1	0	0	0
1	1	1	1	1	1	1	1	1
1	1	1	1	0	1	0	0	1
5	5	5	5	3	3	3	4	5
2	2	2	1	3	3	2	2	2
3	5	5	3	0	0	2	4	3
6	6	2	5	7	4	4	7	6
1	2	1	1	3	3	3	2	1
1	3	7	6	0	0	1	2	1
2	4	4	4	2	1	2	1	2
2	7	7	8	2	2	5	5	2
0	2	2	2	1	1	1	0	0
0	1	1	1	0	0	0	0	0
1	1	0	1	1	1	1	1	1
14	19	17	17	18	18	16	16	14
6	9	10	10	0	0	0	0	0
2	4	6	6	0	0	0	0	0
3	2	2	3	2	2	1	1	3
2	2	2	1	2	2	2	1	2
1	1	0	0	0	0	1	1	1
1	0	0	1	1	0	0	0	1
3	3	2	2	1	2	2	2	3
1	0	0	0	1	1	1	1	1
1	1	0	0	1	1	0	0	1
1	2	2	2	2	2	1	1	1
6	5	5	4	7	5	7	6	6
2	3	3	3	3	3	3	3	2
5	4	2	4	5	3	4	5	5
1	0	1	1	0	0	1	1	1
31	33	34	37	29	32	29	28	31
28	31	31	34	0	0	0	0	0
5	13	12	12	6	5	9	8	5
3	2	2	3	3	3	3	3	3
2	4	4	4	4	4	4	3	2
1	1	1	1	1	1	1	1	1
6	9	8	8	6	6	7	8	6
2	3	3	3	2	2	2	3	2
3	5	9	8	0	4	3	4	3

16	32	33	33	19	19	21	24	16
0	1	1	2	0	0	1	1	0
6	5	8	9	5	7	5	7	6
6	5	7	8	2	2	0	2	2
1	1	0	1	1	1	0	0	1
6	6	6	6	4	4	5	5	5
7	9	10	9	9	9	10	9	7
8	10	12	12	13	12	12	5	8
4	6	5	7	6	6	7	5	4
10	10	11	11	11	10	11	11	10
10	8	9	8	2	2	2	2	3
4	3	3	4	4	4	4	3	4
2	1	2	1	1	1	1	1	1
1	1	1	1	1	1	1	1	1
5	6	6	5	7	7	6	5	5
5	5	4	3	4	5	5	5	5
0	2	1	2	2	2	1	1	0
1	1	0	1	1	0	1	1	1
5	6	7	7	7	7	7	6	5
4	4	4	4	3	5	4	4	4
11	14	14	15	12	16	13	12	11
2	2	2	2	1	1	1	1	1
3	2	3	3	0	0	1	0	1
3	2	1	0	2	3	3	3	3
3	3	3	3	3	3	4	3	3
5	5	6	3	5	5	5	5	5
3	3	3	1	3	4	3	3	3
2	3	3	3	1	1	1	1	0
3	4	4	4	4	4	4	4	3
1	1	1	1	1	1	1	1	1
2	2	2	2	3	3	3	3	2
2	2	2	1	2	1	2	2	2
3	2	2	3	3	2	2	2	3
2	3	3	3	4	4	4	4	2
5	8	8	7	5	7	7	6	5
3	3	2	3	3	2	2	2	3
1	1	0	1	1	1	1	1	1
1	1	1	1	1	1	1	1	1
1	2	2	2	2	2	2	2	1
2	2	2	3	2	2	2	2	2
2	4	2	5	5	5	4	3	2
1	1	2	2	2	2	0	1	1
0	1	1	2	1	1	1	1	0
1	2	3	2	0	2	1	1	1
1	2	2	2	0	0	0	1	1
0	1	1	2	1	0	1	1	0
2	5	7	7	6	8	5	5	2
2	2	2	3	2	2	2	2	2
5	5	7	7	4	6	5	6	5
0	1	1	1	1	1	0	0	0

1	2	2	2	2	2	2	2	1
2	2	1	2	3	2	2	3	2
3	5	4	5	5	5	5	5	3
2	2	1	2	1	2	2	1	2
2	2	3	3	2	3	2	2	2
1	2	2	2	1	1	2	2	1
1	4	4	4	1	2	2	3	1
0	0	0	1	0	0	0	0	0
4	5	5	5	5	5	5	4	4
5	5	5	5	4	4	4	4	4
6	9	8	10	6	5	6	6	6
0	2	2	3	0	0	1	0	0
2	3	4	4	1	2	2	1	2
1	2	2	3	1	2	1	1	1
1	2	3	4	2	2	1	1	1
0	1	1	1	0	0	0	0	0
14	11	10	10	10	10	12	13	14
3	3	4	4	3	3	4	3	3
5	3	1	3	4	4	4	4	5
3	3	3	3	2	2	3	3	3
1	1	0	1	0	1	1	1	1
4	2	3	2	4	2	4	4	4
6	4	6	4	5	5	5	4	6
1	2	5	3	1	4	1	1	1
3	3	3	2	3	3	3	2	3
3	3	2	2	4	3	3	4	3
2	2	2	1	2	2	2	2	2
1	2	2	1	0	0	1	2	1
2	2	3	0	2	3	2	2	2
13	13	10	13	3	5	12	13	13
4	4	3	2	4	5	5	5	4
0	0	0	0	1	1	0	0	0
9	10	11	10	4	4	9	9	9
9	8	9	7	9	9	9	9	9
4	4	4	4	2	2	2	2	2
3	3	3	3	3	3	2	3	3
0	0	0	0	0	0	0	1	0
4	5	4	4	4	5	5	5	4
2	2	2	1	0	1	2	2	2
5	6	7	6	5	6	5	4	5
3	3	3	2	1	2	3	3	3
3	5	6	5	1	1	3	2	3
5	5	5	5	0	0	3	3	3
1	1	1	1	1	1	1	1	1
2	2	1	2	1	2	2	2	2
1	5	5	4	3	4	4	4	1
1	1	1	1	2	2	1	1	1
2	2	1	2	1	2	2	2	2
5	3	4	4	5	5	5	5	5
0	1	1	1	1	1	1	1	0

1	1	0	0	1	1	1	1	1
1	1	0	1	1	1	1	1	1
3	2	2	1	0	2	2	2	3
2	1	1	1	1	2	2	2	2
5	11	10	10	8	8	8	8	5
9	7	11	12	7	10	8	6	9
2	5	5	5	2	4	2	3	2
0	2	1	2	0	0	0	0	0
19	17	18	16	21	20	20	20	19
8	8	4	7	5	8	8	7	8
8	6	2	6	1	0	2	2	3
1	1	1	1	1	1	1	1	1
0	1	0	1	1	1	1	0	0
2	4	3	2	0	0	4	4	2
3	3	3	3	0	1	1	3	3
1	1	1	0	1	1	1	1	1
1	1	1	1	1	1	1	1	1
4	4	2	2	4	3	3	4	4
0	0	0	1	0	0	0	0	0
0	1	1	1	0	0	0	0	0
1	1	0	0	0	0	1	1	1
12	11	14	13	13	14	14	12	12
1	1	1	2	1	2	1	1	1
12	13	15	13	15	14	13	14	12
2	3	2	2	2	1	3	2	2
7	6	5	7	7	6	7	5	7
1	2	2	1	1	2	2	2	1
1	0	0	0	0	0	0	0	1
8	10	6	7	9	8	10	9	8
4	7	6	6	6	7	7	7	4
1	0	1	0	2	2	1	1	1
1	1	1	1	1	1	1	1	1
4	5	5	5	5	4	5	5	4
3	2	4	4	2	2	2	2	2
2	3	3	2	2	2	2	2	1
5	4	5	3	4	4	4	5	5
4	2	5	6	4	6	5	4	4
5	4	7	5	5	6	5	7	5
1	2	2	1	1	2	2	1	1
2	1	1	2	1	2	2	2	2
0	1	1	1	1	0	0	0	0
2	5	4	4	0	1	2	4	2
1	1	1	1	1	0	1	1	1
2	2	2	2	2	2	2	2	2
5	9	11	9	5	6	7	8	5
0	2	3	3	0	0	0	0	0
4	7	7	7	5	6	4	5	4
2	4	4	4	4	5	4	4	2
1	6	8	8	0	0	1	1	0
2	7	9	9	7	8	6	4	2

0	2	1	2	1	1	1	1	0
1	2	4	1	1	0	0	1	0
2	6	8	8	1	1	2	2	1
3	4	5	5	3	4	2	3	3
2	4	3	4	2	2	2	1	2
1	2	1	1	2	2	2	2	1
0	2	4	4	1	1	1	1	0
6	10	13	13	5	8	8	8	6
1	6	6	5	0	1	3	3	1
8	12	11	11	12	12	12	11	8
1	3	2	3	2	1	2	1	1
1	1	0	1	1	1	1	1	1
0	0	1	1	0	0	1	0	0
2	2	2	2	0	1	2	2	2
1	2	2	2	0	0	2	2	1
6	2	2	1	3	3	5	5	6
4	4	4	5	6	5	5	4	4
0	1	1	2	1	2	2	1	0
2	2	1	3	2	2	2	1	2
4	3	3	4	4	2	4	4	4
1	2	3	1	3	3	2	2	1
2	2	2	4	1	2	1	2	2
1	2	2	2	0	0	0	1	1
3	3	3	4	1	1	3	2	3
5	6	4	3	5	5	5	5	5
1	3	3	3	3	2	1	1	1
1	2	3	3	2	1	1	1	1
2	4	5	4	3	3	4	4	2
0	1	1	1	0	1	1	0	0
1	1	1	0	0	1	1	1	1
4	3	3	4	4	4	4	4	4
1	1	1	1	1	1	1	1	1
2	2	2	2	0	1	1	1	2
1	2	2	2	1	1	1	1	1
1	2	2	2	1	1	0	0	1
2	3	2	2	0	1	1	1	2
3	4	4	4	4	4	3	3	3
0	4	5	4	4	3	2	3	0
1	1	0	1	1	2	2	1	1
3	2	2	2	2	2	3	2	3
2	2	2	2	0	0	2	2	2
0	0	1	0	0	0	0	0	0
25	23	25	25	25	28	27	23	25
3	4	4	3	4	4	4	4	3
1	1	3	3	3	2	2	2	1
0	2	2	2	1	1	1	2	0
1	2	2	2	2	2	3	2	1
3	3	3	2	3	3	3	3	3
1	1	0	1	1	1	1	1	1
2	2	1	1	0	2	2	2	2

3	3	3	3	3	3	3	3	3
1	1	1	1	1	1	1	1	1
3	5	8	6	3	3	3	5	3
2	3	3	3	2	5	2	3	2
0	0	1	0	1	1	1	1	0
1	1	0	2	0	0	1	1	1
0	2	2	2	2	1	1	1	0
8	9	8	8	6	10	9	8	8
2	2	2	2	0	1	2	2	2
20	24	26	25	7	8	19	21	20
10	14	15	14	3	4	12	9	10
2	3	3	3	2	3	3	2	2
0	0	0	0	0	1	1	2	0
1	2	1	1	2	2	2	2	1
0	7	8	7	5	6	2	3	0
2	4	3	3	2	1	2	2	2
0	3	2	2	0	0	2	1	0
3	4	3	3	0	0	4	2	3
8	8	9	7	3	8	7	9	8
2	6	5	6	1	3	4	4	2
6	6	7	6	5	5	5	6	6
0	2	2	2	1	2	2	2	0
2	3	4	3	4	4	3	3	2
3	4	4	4	4	4	4	3	3
8	13	14	14	7	4	9	7	8
2	2	3	1	3	3	3	3	2
3	9	9	9	6	9	6	5	3
0	0	0	1	0	0	0	0	0
0	2	1	1	2	3	2	3	0
14	12	14	14	9	8	11	11	14
11	15	15	13	11	11	9	9	11
2	4	5	3	0	0	0	1	2
0	1	1	1	0	0	0	0	0
0	2	2	2	1	2	1	0	0
1	1	1	0	1	1	1	1	1
4	4	6	6	5	5	4	6	4
6	7	4	6	6	6	5	7	6
1	1	1	1	1	1	1	1	1
25	28	26	25	29	31	32	30	25
9	14	15	14	9	9	10	10	9
1	1	1	1	1	2	2	1	1
1	1	1	0	1	1	1	1	1
2	4	4	6	3	3	3	3	2
2	4	3	2	1	0	2	2	2
2	2	4	3	1	0	2	2	2
4	4	5	5	6	4	3	3	4
7	8	7	7	8	8	7	7	7
4	2	1	1	3	2	2	3	4
4	4	4	2	4	4	5	5	4
4	4	2	1	4	4	4	4	4

6	6	5	5	5	6	6	5	6
3	2	2	2	3	2	2	3	3
6	10	10	9	12	11	12	9	6
0	0	1	1	0	0	0	0	0
2	2	3	3	2	1	1	1	2
1	1	0	0	1	1	1	1	1
1	1	2	1	1	1	1	1	1
1	3	3	3	1	1	1	2	1
3	4	5	5	4	5	4	4	3
2	4	4	4	2	1	3	1	2
0	0	1	0	0	0	0	0	0
4	2	1	1	1	1	3	3	4
1	1	0	0	1	1	1	1	1
1	1	1	1	2	1	1	2	1
3	1	1	1	1	2	1	1	3
0	1	0	2	0	1	0	1	0
2	2	2	0	1	1	2	3	2
7	9	8	8	6	8	9	7	7
2	1	1	1	1	0	1	1	2
1	0	1	2	1	0	1	1	1
1	2	1	1	0	0	0	0	1
6	9	9	7	4	3	7	7	6
1	2	2	1	0	1	1	2	1
1	1	1	0	1	1	1	1	1
1	1	1	1	1	1	1	1	1
0	0	1	1	0	0	0	0	0
2	1	0	2	2	2	2	2	2
1	2	1	1	1	2	2	2	1
2	2	2	2	2	2	1	2	2
0	1	0	1	0	1	1	1	0
2	2	2	2	2	2	2	2	2
8	8	8	7	8	9	9	8	8
7	8	8	7	1	1	1	1	0
4	9	8	8	4	4	6	4	4
1	2	1	1	2	2	2	2	1
5	6	6	5	5	6	4	2	5
5	6	5	5	1	2	5	4	5
5	6	6	4	6	6	6	6	5
1	1	1	1	1	1	1	1	1
1	1	1	2	0	0	3	1	1
0	0	1	1	1	0	0	0	0
14	14	17	17	12	11	13	12	14
4	9	16	14	9	6	6	7	4
7	13	14	15	10	12	8	8	7
4	10	10	11	7	6	5	4	4
7	7	8	8	6	5	6	7	7
8	13	14	14	9	9	6	8	8
13	16	17	16	13	9	12	13	13
3	8	9	10	3	5	6	5	3
3	1	2	3	1	0	3	3	3

1	1	2	1	1	2	2	1	1
1	2	2	2	0	1	1	1	1
3	2	3	1	2	3	1	2	3
2	2	2	2	3	3	2	2	2
2	3	2	3	3	3	3	3	2
1	0	0	1	0	0	0	1	1
0	1	1	1	1	1	1	1	0
1	1	1	1	1	1	1	1	1
0	0	1	1	1	0	0	0	0
3	3	5	5	2	2	3	2	3
2	2	1	1	1	2	2	2	2
2	2	3	3	1	1	2	2	2
7	6	6	6	8	6	7	6	7
0	4	6	6	1	1	2	1	0
5	5	6	5	7	7	6	6	5
5	5	5	5	3	5	6	6	5
3	4	2	3	2	4	4	3	3
3	6	6	5	5	7	6	5	3
0	2	1	1	2	2	2	1	0
0	1	0	1	1	1	1	1	0
3	6	5	6	0	0	3	2	3
0	0	1	0	0	0	0	0	0
7	15	15	16	7	7	8	8	7
9	10	9	11	9	10	10	11	9
0	0	1	1	0	0	0	0	0
0	0	0	0	1	1	0	0	0
1	2	2	1	0	0	1	1	1
11	6	6	7	5	6	9	7	11
5	9	12	10	8	9	5	5	5
7	9	9	9	5	7	8	7	7
1	1	1	0	0	0	0	1	1
4	5	5	6	7	6	6	3	4
3	2	2	3	3	3	3	3	3
0	1	1	1	1	1	1	0	0
4	3	1	3	3	2	3	4	4
1	1	0	0	1	0	1	1	1
6	5	6	5	5	5	5	5	6
1	1	0	0	0	0	1	1	1
2	6	7	6	2	1	3	4	2
1	1	0	1	1	1	1	1	1
1	5	5	4	0	0	1	3	1
2	3	2	3	2	2	2	2	2
1	3	3	3	2	2	2	2	1
0	1	2	1	0	0	0	0	0
2	4	3	2	4	4	4	4	2
0	1	1	1	0	0	0	0	0
3	9	11	10	3	2	3	3	3
3	3	0	1	1	1	2	3	3
1	1	1	1	1	1	1	1	1
1	2	2	2	2	2	1	3	1

5	6	6	5	6	8	6	6	5
13	20	22	22	19	20	19	19	13
1	1	1	1	1	1	1	1	1
4	3	3	2	3	4	4	4	4
1	1	1	1	2	2	2	1	1
4	5	5	5	1	1	1	1	1
7	9	10	10	7	8	8	9	7
2	2	2	0	2	2	2	2	2
16	18	20	20	16	15	18	18	16
10	16	17	18	9	12	9	12	10
4	5	7	8	5	7	4	4	4
2	1	2	2	2	2	2	2	2
2	5	6	6	2	4	4	3	2
0	2	3	2	2	3	2	1	0
1	2	2	1	1	2	1	2	1
2	4	4	4	4	4	4	4	2
1	1	1	2	1	1	2	1	1
0	0	1	1	2	2	1	1	0
3	3	3	3	3	3	3	2	3
2	1	1	1	1	1	2	1	2
2	3	3	2	3	2	3	3	2
5	5	5	5	6	6	5	4	5
2	3	2	3	3	3	3	3	2
4	5	5	2	5	5	4	4	4
1	2	1	1	2	2	2	2	1
1	1	1	1	0	1	1	1	1
2	2	1	2	2	2	2	2	2
1	1	1	1	1	1	1	1	1
3	3	1	1	4	3	3	2	3
1	2	1	1	2	2	2	2	1
3	3	3	2	2	2	2	2	2
3	3	3	2	3	3	3	3	3
2	2	2	2	2	2	2	2	2
1	2	2	2	2	3	2	2	1
2	0	0	1	0	0	2	1	2
1	0	0	1	1	1	1	1	1
0	0	0	0	0	0	1	1	0
3	2	1	1	3	3	4	4	3
0	0	0	0	1	1	1	0	0
0	2	2	2	2	2	2	2	0
3	4	3	3	4	4	4	4	3
5	5	2	3	4	4	5	4	5
1	3	2	2	2	3	2	3	1
0	2	1	0	1	1	1	1	0
1	3	3	3	4	4	4	4	1
13	12	13	9	9	11	9	11	13
1	3	3	3	3	3	3	3	1
14	17	17	15	18	18	20	17	14
11	11	11	12	13	12	13	12	11
1	4	4	4	3	2	3	3	1

6	13	15	15	6	5	6	6	6
1	2	2	1	1	1	1	1	1
2	1	1	1	2	2	2	2	2
7	11	12	11	14	12	10	9	7
2	3	2	2	2	2	1	1	2
3	7	5	6	4	6	4	5	3
3	2	2	4	3	3	0	1	3
7	7	10	10	4	4	2	3	3
14	12	15	15	14	13	13	15	14
12	11	14	10	2	2	2	2	2
11	11	14	12	7	6	6	6	6
1	5	5	5	1	0	2	1	1
14	16	18	17	1	1	1	0	1
14	16	18	17	15	16	16	14	14
13	16	17	17	2	2	2	1	0
14	15	18	16	3	3	3	3	3
11	14	15	16	4	3	3	2	3
9	11	13	14	3	3	3	2	1
8	10	11	11	2	2	1	2	1
12	15	17	17	16	17	15	15	12
6	6	7	7	2	2	1	1	2
7	8	9	9	2	2	2	1	1
0	1	1	1	1	1	1	0	0
2	3	3	2	0	0	1	1	2
1	3	4	3	3	3	1	1	1
0	0	1	1	0	0	0	0	0
1	1	1	0	1	1	1	1	1
0	1	2	2	2	2	1	1	0
0	3	3	3	0	0	1	2	0
1	5	5	5	0	1	4	4	1
1	3	3	3	2	2	2	2	1
1	1	1	1	1	0	1	1	1
8	8	8	7	8	8	8	8	8
2	2	2	2	1	0	1	2	2
1	1	1	1	0	0	1	1	1
1	1	1	1	1	1	1	1	1
11	9	4	6	8	6	9	8	11
4	4	3	4	5	4	5	5	4
5	8	7	8	5	4	4	6	5
2	3	3	3	3	3	2	2	2
1	3	3	3	3	3	3	2	1
3	5	5	6	3	2	3	4	3
0	0	2	2	0	0	0	0	0
1	2	2	2	0	0	1	2	1
0	3	2	4	0	2	3	2	0
1	1	2	2	0	1	0	0	1
1	2	3	3	2	2	1	2	1
2	1	2	0	2	2	1	2	2
1	0	0	0	0	1	1	1	1
5	2	3	4	0	2	3	4	5

1	2	2	0	2	2	1	1	1
1	2	2	2	2	2	2	2	1
2	2	2	1	2	3	3	2	2
0	1	1	1	1	1	1	1	0
1	2	0	2	0	0	1	2	1
6	13	15	14	11	12	9	10	6
1	2	3	3	2	1	2	2	1
1	1	1	1	0	0	0	1	1
0	0	1	1	0	0	0	0	0
2	5	5	4	3	3	1	2	2
0	1	0	1	1	0	1	1	0
0	1	0	1	1	1	0	0	0
0	2	1	2	0	0	1	0	0
5	6	6	6	2	2	4	5	5
0	6	8	7	0	1	2	1	0
0	2	2	2	1	0	1	1	0
1	2	2	1	0	0	2	1	1
3	4	4	4	3	3	4	3	3
7	7	7	7	7	7	7	7	7
3	2	4	2	1	0	1	2	3
0	0	2	2	1	1	1	0	0
0	2	3	3	0	0	1	1	0
0	1	1	1	0	1	1	1	0
3	3	3	4	2	2	1	3	3
3	4	3	3	1	1	3	3	3
2	3	4	3	2	1	2	2	1
0	1	1	1	1	1	1	1	0
9	18	20	19	9	12	13	12	9
1	2	2	1	0	1	2	0	1
0	1	0	1	1	1	1	1	0
3	3	3	2	3	3	3	3	3
6	7	7	9	7	7	5	6	6
8	8	6	5	9	9	10	9	8
2	2	3	2	3	4	3	2	2
1	2	4	4	0	1	0	1	1
0	1	1	1	0	1	1	1	0
1	1	1	1	1	1	1	1	1
1	2	2	2	2	2	2	2	1
10	13	16	16	16	16	13	14	10
8	10	11	11	11	11	10	9	8
0	3	3	3	2	2	0	0	0
2	1	1	1	2	2	2	2	2
0	0	1	0	0	1	0	1	0
0	0	1	1	0	1	0	1	0
1	1	1	1	1	1	1	1	1
1	2	1	2	2	2	2	2	1
0	1	2	3	2	1	0	0	0
0	2	3	2	1	2	0	1	0
1	1	1	1	1	1	1	1	1
0	1	1	1	0	0	0	0	0

1	1	1	1	1	1	1	1	1
1	2	3	2	0	0	0	1	1
4	7	8	8	4	3	4	6	4
1	1	0	0	1	1	1	1	1
3	3	2	2	1	3	3	3	3
1	1	1	1	0	1	1	1	1
2	6	5	5	5	4	5	6	2
1	2	1	0	2	2	2	2	1
0	1	2	1	0	1	1	0	0
2	5	5	5	4	3	3	3	2
1	1	0	1	1	1	1	1	1
10	17	18	16	18	19	18	17	10
1	0	0	0	0	1	0	0	1
4	5	5	5	6	6	5	5	4
1	2	3	2	1	2	2	1	1
0	2	2	3	2	2	2	2	0
1	1	1	0	1	1	1	1	1
4	3	3	3	4	4	4	4	4
3	3	1	1	2	3	3	3	3
2	2	1	2	1	1	1	1	1
5	4	3	5	5	4	4	4	5
1	0	1	0	1	1	1	0	1
1	1	0	1	1	1	1	1	1
3	3	3	3	3	3	3	3	3
2	3	1	1	5	2	3	3	2
5	6	7	7	5	7	4	3	5
9	14	15	17	4	3	5	8	9
1	1	0	0	2	2	1	1	1
1	1	1	1	1	1	1	1	1
12	15	15	15	16	16	16	12	12
14	15	14	15	17	17	16	17	14
4	6	6	5	6	6	6	6	4
1	2	1	1	2	2	2	2	1
0	1	0	1	1	1	1	1	0
5	4	4	5	3	4	5	5	5
1	1	0	1	1	1	1	1	1
5	7	6	7	3	3	4	5	5
0	1	1	0	0	1	1	1	0
2	3	3	1	1	2	2	2	2
1	3	3	4	1	3	1	3	1
3	2	2	2	1	2	3	3	3
4	5	4	5	4	4	5	5	4
4	7	6	6	3	4	2	5	4
3	2	4	4	4	3	3	3	3
2	1	1	2	1	2	3	3	2
3	3	2	1	3	3	3	3	3
4	2	2	3	3	3	3	3	4
1	1	1	1	0	1	1	1	1
0	0	1	1	0	0	0	0	0
1	1	1	1	2	2	1	1	1

1	2	0	0	1	1	1	1	1
0	0	1	0	0	1	1	0	0
7	13	16	15	11	12	10	7	7
8	10	13	12	6	7	7	9	8
6	6	6	5	3	2	3	3	3
0	0	2	2	0	0	0	0	0
1	2	2	2	1	1	2	2	1
1	1	1	1	0	0	0	0	1
4	5	4	3	4	4	4	4	4
2	2	1	1	2	1	2	2	2
0	1	2	2	0	0	0	0	0
1	3	2	2	3	2	3	1	1
2	2	2	1	2	2	2	2	2
0	1	1	1	1	1	1	1	0
1	1	1	1	1	1	1	1	1
2	1	0	0	0	1	1	2	2
3	3	3	3	0	0	1	3	3

Razor + uni	Razor + uni	Razor + uni	Unique pe	Unique pe	Unique pe	Unique pe	Unique pe	Unique pe
7	7	8	8	8	7	7	7	7
7	7	7	6	7	7	4	6	7
1	1	1	1	1	1	1	1	1
4	4	4	4	4	4	4	4	4
12	12	11	9	8	10	10	10	12
8	7	7	5	5	8	7	7	8
6	5	4	4	3	6	5	6	6
14	16	17	11	12	12	12	12	12
7	7	8	7	7	7	7	7	7
10	8	8	7	6	7	9	7	9
2	2	2	1	0	1	1	1	2
2	2	0	2	2	2	2	2	2
0	0	0	1	1	0	0	1	0
7	5	6	6	7	7	7	7	7
2	3	3	3	2	0	0	1	2
6	6	6	7	7	5	4	2	6
5	5	5	4	4	2	4	1	5
4	5	4	4	4	4	3	3	4
4	2	3	4	4	2	2	3	4
9	10	10	8	7	6	7	5	9
4	5	4	4	3	2	3	2	3
4	4	6	3	4	1	2	2	4
2	2	2	1	1	0	1	0	2
7	10	9	6	4	3	3	2	7
6	6	4	2	4	1	1	2	6
4	5	6	2	2	3	2	3	4
1	1	1	1	1	1	1	0	1
6	9	9	8	12	6	7	4	6
5	5	5	5	4	5	4	2	5
1	0	2	0	0	0	1	0	1
1	3	3	0	0	0	0	1	1
3	3	3	1	2	1	2	2	3
1	1	2	1	1	1	1	1	1
3	5	4	0	0	2	1	2	3
1	1	1	1	1	1	1	1	1
1	1	1	1	1	1	1	1	1
2	1	2	1	2	2	2	1	2
4	4	2	3	4	4	4	4	4
3	3	3	3	3	3	3	1	3
1	1	0	1	1	1	0	1	1
2	2	1	3	2	3	2	2	2
1	1	1	1	1	2	1	1	1
3	2	1	3	3	1	1	2	3
1	0	1	2	2	2	2	1	1
1	1	0	2	0	2	2	2	1
2	1	0	2	2	2	2	2	2
1	1	1	1	1	1	1	1	1
1	1	1	1	1	1	1	0	1

3	2	1	3	3	3	3	2	3
2	1	2	1	2	1	1	0	2
12	12	9	14	16	17	16	11	12
1	1	1	1	1	1	1	1	1
2	1	3	2	2	3	3	1	2
4	4	4	2	1	1	1	2	2
2	0	0	2	2	2	1	2	2
2	0	1	2	2	2	3	3	2
1	1	1	1	0	1	1	1	1
2	1	1	2	2	2	2	1	2
0	0	0	1	2	1	1	1	0
1	1	1	1	1	1	1	1	1
2	1	1	2	1	2	2	2	2
0	0	0	0	1	0	1	0	0
1	1	0	1	1	1	1	1	1
2	1	1	2	2	2	2	2	2
1	0	0	1	1	1	1	1	1
1	1	1	1	1	1	1	1	1
1	1	0	1	2	1	1	0	1
1	1	1	1	1	1	1	2	1
1	1	1	1	1	1	1	0	1
2	2	3	3	3	2	1	2	2
8	9	5	5	6	6	8	7	8
1	1	2	2	2	2	2	2	1
1	0	1	1	1	1	1	1	1
1	1	1	1	0	1	1	1	1
3	3	5	2	2	1	3	0	3
8	9	8	8	8	9	8	7	8
6	7	7	7	7	7	6	7	6
4	5	5	6	5	5	3	3	4
5	5	7	6	7	6	5	5	5
5	5	5	1	1	1	1	1	1
6	6	5	5	4	4	4	3	6
5	5	4	7	5	5	4	4	5
6	6	6	6	6	6	6	5	6
6	6	7	7	5	6	6	3	6
8	9	9	7	8	8	7	8	8
2	3	3	3	3	3	3	3	2
4	4	4	3	2	3	2	1	4
4	4	4	4	4	3	4	3	4
2	3	3	2	2	2	2	2	2
3	2	3	2	2	2	2	1	3
3	3	2	3	3	3	2	2	3
4	4	4	4	4	3	3	3	4
2	2	2	1	2	2	2	1	2
1	2	0	1	1	1	1	1	1
10	11	11	12	12	11	9	8	10
1	1	1	1	1	1	1	1	1
10	11	12	10	12	10	9	8	10
8	11	10	8	10	6	6	6	8

5	6	6	7	7	7	4	5	5
5	4	6	5	6	4	5	4	5
2	4	3	3	4	3	2	2	2
9	9	10	10	10	10	9	8	9
8	8	9	8	8	7	7	5	8
8	8	7	8	8	7	7	6	8
21	23	23	24	22	23	22	20	21
2	2	1	2	2	2	2	2	2
1	1	1	2	1	2	2	1	1
25	27	26	25	26	26	25	26	25
9	9	8	5	8	8	9	5	9
1	1	1	1	1	1	1	0	1
6	7	6	6	6	5	5	3	5
5	7	6	2	2	1	1	1	1
7	7	7	7	7	6	7	6	7
3	4	3	1	1	1	1	1	1
1	1	0	1	1	1	1	1	1
5	6	6	5	6	5	5	5	5
6	5	6	5	6	5	5	4	6
6	7	6	6	7	6	6	6	6
3	4	4	4	4	4	4	4	3
6	6	6	6	6	6	6	6	6
1	5	6	4	4	4	2	2	1
4	4	4	4	4	4	4	4	4
5	5	5	5	4	5	5	5	5
1	2	2	2	1	2	1	2	1
2	2	2	2	2	2	2	2	2
4	4	4	4	4	4	4	3	4
3	4	4	3	4	3	4	2	3
3	5	4	4	5	5	5	5	3
4	5	6	4	6	4	3	2	4
4	4	4	3	4	3	2	1	4
3	3	4	3	3	3	3	2	3
4	4	5	4	4	4	4	4	4
2	2	2	0	2	2	2	2	2
12	15	14	15	16	12	11	12	12
4	5	4	4	5	5	4	5	4
2	3	4	4	4	3	3	3	2
4	4	5	4	4	4	4	4	4
5	6	5	5	6	6	6	5	5
2	2	2	2	2	2	1	1	2
5	5	4	4	4	4	4	5	5
3	3	3	3	3	3	3	3	3
2	2	2	2	2	2	2	2	2
2	3	3	2	3	3	1	1	2
1	1	1	1	1	1	1	1	1
11	13	13	14	14	13	11	13	11
9	9	9	7	9	8	6	4	9
12	13	12	12	12	12	10	9	12
6	5	6	6	6	6	6	4	6

13	12	12	12	13	13	13	6	13
1	1	1	1	1	1	1	1	1
4	5	4	3	3	3	3	3	4
4	5	5	5	4	4	3	2	4
1	1	2	0	1	0	0	0	1
7	9	10	3	4	5	4	3	7
1	1	1	0	0	0	1	1	1
1	1	2	0	1	1	2	1	1
26	28	28	26	27	24	23	24	25
5	4	3	5	5	5	6	4	5
1	1	1	1	0	0	0	0	1
2	3	2	1	1	3	2	2	2
0	1	0	0	1	1	0	1	0
2	2	1	1	1	1	0	1	2
6	6	6	6	6	6	6	6	6
1	1	1	1	1	1	0	1	1
4	4	4	0	0	1	0	0	3
1	1	1	0	0	0	0	0	1
7	8	9	6	8	6	6	7	7
1	1	1	1	1	1	1	1	1
19	19	19	1	1	1	1	1	1
1	1	1	1	1	1	1	1	1
4	5	5	4	4	4	4	4	4
3	4	4	0	2	1	2	2	3
2	4	3	3	2	2	2	2	2
4	4	3	1	2	2	1	2	4
0	1	0	0	0	0	0	0	0
3	4	4	3	3	2	2	3	3
1	1	1	1	1	1	1	1	1
1	1	1	0	1	1	1	0	1
7	7	7	6	4	6	6	4	7
3	3	3	3	3	3	3	3	3
5	7	6	1	2	2	3	2	5
0	0	0	0	0	0	0	1	0
2	2	1	2	2	1	1	1	2
2	2	2	1	1	2	2	2	2
5	5	5	5	5	5	5	4	5
2	2	2	2	2	2	2	2	2
2	3	2	1	2	2	2	2	2
1	0	0	1	1	1	1	1	1
2	1	1	2	2	2	2	2	2
5	5	5	5	5	5	5	5	5
2	2	2	1	2	3	3	1	2
2	2	2	2	2	2	1	2	2
1	0	1	1	1	1	0	0	1
3	3	3	2	3	1	0	1	3
2	1	1	1	1	1	0	1	2
1	1	1	1	0	0	0	0	1
12	14	14	10	11	11	9	6	12
3	4	4	5	3	3	3	4	3

2	2	0	1	1	2	2	2	2
5	5	5	2	3	3	2	3	5
1	1	2	0	0	0	0	1	1
5	5	4	0	0	2	3	0	5
10	10	11	6	7	9	9	7	10
6	4	5	5	6	5	5	3	6
1	1	1	0	0	0	1	0	1
14	13	13	5	5	4	6	5	5
4	4	4	2	2	2	2	2	2
2	3	2	2	3	3	3	2	2
5	6	6	2	2	3	2	1	3
1	1	1	1	1	1	1	1	1
2	1	1	1	1	0	1	1	2
3	2	3	3	3	3	3	2	3
2	2	2	1	1	1	1	1	1
2	2	3	0	0	2	1	2	2
2	1	2	1	2	2	2	2	2
3	4	4	4	4	4	4	4	3
1	1	1	0	1	1	1	1	1
1	0	1	0	0	1	0	0	1
2	2	2	2	2	2	2	0	2
6	5	5	6	6	5	3	4	6
3	3	3	0	0	1	3	2	3
2	3	4	1	2	2	2	1	2
15	17	17	13	12	11	8	10	13
1	0	1	1	1	1	2	2	1
10	9	8	0	0	0	0	0	0
2	2	2	0	2	1	1	0	2
0	1	1	1	1	1	0	0	0
3	5	5	2	2	2	3	3	3
9	8	7	10	10	10	9	5	9
0	0	0	1	1	1	1	0	0
1	1	1	0	0	0	1	1	1
2	1	0	2	1	1	1	1	2
28	33	31	9	10	14	13	9	18
17	20	20	7	9	13	12	8	17
5	3	5	4	2	4	3	1	5
1	1	1	1	1	0	0	1	1
0	0	1	0	0	1	0	0	0
18	19	18	12	14	16	14	17	18
2	0	1	2	1	2	2	2	2
0	0	1	0	0	0	0	1	0
5	3	3	3	4	5	4	4	5
1	1	1	1	1	0	1	1	1
4	4	4	4	4	4	4	3	4
4	4	4	4	4	2	2	1	4
2	3	3	2	3	2	2	2	2
4	5	4	3	2	4	4	3	4
1	2	2	2	2	2	2	2	1
7	8	6	5	6	6	7	7	6

5	6	5	4	5	6	5	5	5
0	0	1	1	0	1	1	1	0
0	1	1	1	1	1	0	0	0
22	23	20	22	21	22	22	17	22
1	1	1	1	1	1	0	0	1
5	6	7	5	5	5	4	3	5
23	25	24	25	25	24	24	23	23
11	11	12	8	7	11	10	9	11
11	11	10	6	11	10	10	9	11
10	10	9	9	8	8	8	5	10
6	7	9	7	10	6	8	3	6
8	6	7	8	8	7	8	4	8
8	6	7	5	9	12	11	13	8
2	2	1	1	1	1	2	1	2
6	6	6	0	0	2	2	2	2
2	2	2	1	0	1	1	1	2
1	1	2	0	3	2	3	1	1
0	2	2	0	1	1	1	0	0
3	2	1	2	2	3	2	2	3
1	0	1	0	1	0	0	0	1
1	1	1	0	1	0	0	0	1
1	1	1	1	2	1	1	0	1
4	3	4	4	4	4	5	4	4
1	1	1	1	1	1	1	0	1
2	2	1	2	2	2	2	2	2
0	1	0	2	0	2	2	2	0
4	4	4	0	0	1	0	0	4
1	2	3	1	1	1	1	1	1
7	5	5	9	9	9	8	8	7
8	6	7	6	7	7	6	8	8
1	1	1	0	0	0	1	1	1
5	7	7	5	5	4	3	2	5
5	3	5	4	5	3	4	1	5
5	4	6	3	4	4	4	5	5
4	3	3	0	0	1	1	1	4
12	12	11	11	12	12	11	9	12
15	19	18	15	12	11	13	7	15
0	1	1	2	2	2	0	1	0
4	3	4	4	4	4	4	3	4
1	1	1	1	1	1	1	1	1
2	1	0	1	0	1	2	2	2
2	2	2	3	2	2	3	3	2
1	2	2	2	2	0	1	1	1
5	4	4	6	6	6	5	5	5
1	1	1	1	1	1	1	1	1
14	17	17	22	21	16	18	18	14
17	19	17	21	21	16	17	17	17
5	4	5	8	6	7	6	6	5
2	2	2	2	2	2	2	2	2
2	2	2	2	2	2	2	2	2

1	2	0	1	2	2	1	0	1
2	2	2	2	3	2	3	1	2
8	7	9	10	10	8	9	5	8
5	6	6	5	7	6	4	2	5
3	4	4	4	4	4	4	3	3
4	3	4	5	5	5	5	4	4
0	0	1	1	1	1	1	1	0
1	1	1	2	2	2	2	2	1
4	4	3	2	1	1	1	1	4
2	2	2	2	2	0	1	0	2
1	1	1	1	1	1	1	0	1
1	1	1	1	1	0	0	1	1
17	18	22	7	9	13	13	11	17
2	2	2	2	2	2	2	2	2
2	2	2	3	2	0	0	2	2
10	12	11	10	9	9	8	6	9
3	3	3	5	4	4	3	5	3
1	1	0	3	3	3	3	2	1
3	2	2	1	2	1	2	0	3
19	22	22	19	19	18	16	13	19
8	9	6	2	5	4	3	3	8
7	7	7	5	6	6	7	4	7
1	2	2	1	1	0	1	1	1
3	3	2	2	3	5	4	5	3
1	1	1	1	1	1	0	0	1
9	11	8	9	11	7	8	6	9
0	1	1	0	0	0	0	0	0
3	3	5	4	3	4	4	3	3
1	1	1	2	2	2	2	2	1
0	1	0	0	0	1	1	1	0
1	1	1	1	0	1	1	0	1
2	3	3	2	2	2	1	2	2
1	2	1	1	1	0	0	0	1
1	1	1	1	1	1	1	1	1
0	0	0	0	1	0	0	0	0
4	4	4	1	1	1	1	0	4
3	2	2	0	0	1	1	1	3
7	4	5	7	7	7	7	7	7
5	3	3	3	3	4	4	5	5
0	1	1	1	1	0	1	1	0
4	4	3	4	4	4	4	4	4
2	3	2	3	2	3	3	3	2
2	3	3	3	3	2	2	2	2
1	1	1	1	1	1	1	1	1
0	1	1	0	1	0	0	0	0
1	2	2	2	2	2	1	1	1
3	3	2	0	0	2	0	1	3
3	3	2	2	3	2	1	1	3
17	21	21	20	17	17	11	15	17
1	1	0	2	1	1	0	1	1

13	13	13	6	8	7	7	5	13
1	0	1	1	1	1	1	1	1
2	2	1	1	0	1	2	2	2
1	1	1	1	1	1	1	1	1
4	4	3	3	3	4	4	3	4
0	1	1	0	0	0	0	0	0
2	1	2	1	2	1	0	0	2
2	2	1	1	2	2	2	1	2
0	1	2	0	1	0	0	0	0
4	4	4	1	0	0	1	3	4
1	1	0	1	1	0	1	0	1
3	4	4	1	1	1	3	2	3
14	14	12	14	13	12	11	9	14
4	4	3	4	4	4	4	4	4
1	1	1	1	1	1	1	1	1
13	17	17	14	18	12	11	9	13
2	1	1	1	0	1	2	2	2
3	3	4	4	4	4	4	4	3
2	2	1	2	2	2	3	1	2
1	1	0	1	0	0	1	1	1
15	15	12	14	15	14	13	9	15
5	3	4	5	6	5	5	3	5
7	6	6	6	6	7	6	6	7
6	6	6	5	5	4	3	4	6
18	17	17	21	20	16	17	13	18
4	4	4	3	3	4	3	2	4
5	7	7	2	5	4	5	3	5
6	6	6	5	6	4	3	1	6
3	3	5	0	0	0	1	3	3
4	7	6	2	1	3	3	2	4
13	14	15	15	15	13	14	13	13
2	1	2	1	0	2	2	1	2
2	2	1	3	1	2	3	2	2
1	1	1	1	1	1	1	1	1
3	4	4	2	2	2	3	2	3
4	4	3	2	3	3	4	2	4
10	10	11	2	2	5	5	5	10
6	4	4	6	5	7	4	7	6
8	7	6	8	9	9	9	7	8
4	1	4	5	4	5	4	3	4
1	2	3	2	3	2	1	1	1
0	0	2	1	1	1	0	0	0
2	2	1	2	2	1	1	0	2
1	2	1	2	1	1	1	1	1
1	1	0	1	1	0	1	1	1
6	4	6	5	6	5	7	7	6
1	5	5	3	3	3	1	0	1
14	16	18	13	12	12	15	10	14
5	2	5	3	4	5	5	4	5
11	14	12	11	13	11	11	12	11

5	3	5	3	3	5	4	2	5
2	2	3	3	3	3	2	2	2
5	5	5	5	4	6	6	6	5
7	7	8	8	8	8	8	6	7
2	2	1	1	1	2	2	1	2
1	0	1	3	3	3	3	1	1
2	2	2	3	2	3	2	2	2
2	2	2	2	2	2	2	1	2
5	6	6	5	6	4	4	5	5
3	3	3	3	3	2	3	3	3
3	2	3	3	2	3	3	1	3
2	1	2	3	2	3	3	1	2
2	2	2	2	2	2	2	0	2
1	1	1	1	1	1	1	2	1
2	2	4	3	3	2	1	0	2
3	4	3	2	3	3	3	1	3
3	3	4	2	1	0	2	2	3
2	2	2	1	1	2	2	1	2
4	4	5	1	1	1	1	1	1
0	1	0	0	0	0	0	0	0
1	1	1	1	2	1	2	2	1
1	2	1	2	2	2	2	1	1
1	1	1	1	1	1	1	0	1
5	5	5	5	5	5	5	4	5
8	9	9	4	7	6	6	2	8
2	2	2	2	2	3	3	2	2
0	1	1	0	0	0	1	1	0
1	1	0	1	1	1	1	0	1
1	0	1	1	1	1	1	0	1
1	1	1	1	1	1	1	1	1
4	5	6	3	3	3	4	6	4
2	2	2	2	2	1	1	2	2
8	6	7	7	8	8	8	6	7
38	44	42	23	26	24	21	13	31
2	2	2	2	1	2	2	2	2
2	2	1	1	1	2	2	2	2
1	1	1	2	2	2	1	1	1
3	3	3	3	3	2	3	2	3
1	1	1	1	1	1	1	1	1
2	2	2	2	2	2	2	2	2
3	3	3	1	3	3	2	0	3
4	3	2	4	3	2	2	2	4
0	1	1	0	1	0	0	0	0
1	1	1	0	1	1	1	1	1
4	2	2	4	1	3	5	4	4
3	2	1	2	3	3	3	3	3
0	1	3	0	1	0	1	0	0
4	4	5	2	2	3	4	3	4
0	0	0	1	1	1	1	1	0
8	9	8	7	5	7	4	4	8

3	2	2	2	2	2	2	2	3
7	8	9	9	7	6	6	7	7
4	5	5	4	5	4	4	4	4
3	3	3	3	3	1	1	1	3
2	2	4	1	1	1	0	2	2
12	12	11	10	10	8	8	6	12
0	0	0	0	0	1	0	1	0
3	4	5	5	5	3	3	3	3
1	1	1	1	1	1	1	1	1
1	1	0	1	1	1	1	0	1
0	1	1	0	1	0	1	1	0
3	2	3	3	2	3	3	3	3
4	5	4	3	3	3	3	1	4
7	9	5	6	9	9	8	11	7
7	7	6	7	6	6	4	6	7
0	0	1	0	0	0	0	0	0
3	3	4	1	2	1	2	1	3
2	2	2	1	1	1	1	1	2
1	0	0	1	0	0	1	0	1
7	7	8	5	7	6	6	6	7
0	2	1	1	0	1	1	1	0
1	1	2	0	0	0	1	0	1
1	2	1	1	1	1	1	1	1
7	9	9	9	9	7	7	4	6
0	0	0	1	1	1	1	0	0
9	8	7	6	6	7	7	7	8
1	1	0	1	1	1	1	1	1
2	2	1	1	0	1	2	1	2
5	6	5	3	4	5	4	4	5
1	0	1	1	1	1	1	1	1
0	1	1	0	0	0	0	0	0
3	3	3	3	3	3	3	3	3
3	3	2	0	2	1	0	1	3
1	1	2	0	0	1	2	2	1
1	1	1	0	0	1	0	0	1
2	2	2	2	2	1	0	2	2
5	5	5	4	5	3	3	3	5
1	1	2	2	2	1	2	2	1
1	1	1	0	1	0	0	0	1
1	1	1	0	0	0	0	1	1
7	6	6	0	0	2	3	2	7
4	3	4	2	3	2	2	3	4
5	6	6	3	2	3	2	3	4
1	1	1	0	0	0	0	1	1
2	3	3	2	1	2	2	2	2
0	1	0	1	1	1	0	1	0
5	5	6	2	3	3	4	3	5
2	2	2	2	1	2	2	2	2
1	1	0	1	1	1	1	1	1
3	2	2	3	2	2	3	3	3

1	1	1	0	0	1	0	1	1
1	1	1	1	1	1	1	1	1
0	0	0	1	1	1	1	1	0
1	2	2	2	2	2	1	1	1
10	10	14	12	12	10	8	9	9
11	12	11	10	9	10	10	8	11
2	3	2	3	1	2	2	2	2
2	2	1	2	2	2	2	1	2
1	1	1	1	1	1	1	1	1
2	2	2	3	4	3	4	3	2
1	2	2	2	2	1	1	1	1
3	2	3	3	3	3	2	3	3
0	0	0	1	1	1	1	1	0
1	1	1	1	1	1	1	1	1
2	2	3	3	3	3	3	3	2
4	4	4	4	3	4	4	2	4
2	2	2	2	2	2	2	1	2
3	3	2	5	5	4	5	5	3
3	3	3	4	3	3	2	1	3
2	3	2	3	3	1	1	1	2
2	2	2	2	2	2	2	1	2
1	1	1	2	1	1	2	1	1
2	2	2	2	2	2	2	2	2
2	2	2	2	2	2	2	2	2
1	1	2	2	2	1	1	0	1
3	3	3	4	4	4	3	3	3
29	38	36	28	27	22	19	14	29
2	2	2	0	0	0	0	0	2
3	3	3	2	2	2	1	3	3
3	3	4	2	1	1	2	1	3
18	16	17	19	19	19	19	19	18
2	1	2	1	1	1	1	1	2
0	1	2	0	1	0	0	1	0
4	5	4	2	1	4	3	4	4
1	1	2	0	0	0	0	0	1
8	9	8	9	9	7	8	2	8
2	3	3	2	2	2	1	1	2
2	1	2	2	2	2	2	2	2
1	2	2	0	0	0	1	0	1
1	1	1	1	1	2	2	2	1
4	4	4	1	4	3	3	0	4
2	2	2	1	1	2	2	2	2
2	1	2	3	3	2	2	2	2
5	4	4	5	5	5	5	3	5
4	3	4	3	2	2	3	2	3
2	1	1	2	1	1	1	2	2
13	9	11	13	12	13	12	9	13
1	0	1	0	0	0	0	0	1
3	2	3	1	3	3	3	2	3
0	0	1	0	0	0	0	0	0

2	4	5	1	0	2	2	1	2
1	1	1	1	1	1	1	0	1
1	1	1	1	0	1	0	0	1
1	1	0	0	0	0	1	0	1
3	2	2	2	4	3	3	3	3
9	10	12	9	11	11	10	12	9
8	4	7	11	10	11	11	10	8
30	34	30	37	38	35	37	33	30
5	6	7	2	3	3	3	2	5
2	2	2	1	1	2	2	2	2
3	4	3	5	3	3	5	4	3
4	3	4	4	4	4	4	4	4
8	8	4	6	8	8	8	7	8
1	1	1	0	0	0	0	0	1
2	2	1	1	1	2	2	2	2
4	4	3	4	4	4	4	5	4
1	1	1	1	1	1	1	1	1
7	8	9	6	5	4	4	3	7
0	1	1	1	0	0	0	0	0
1	5	5	3	3	1	1	2	1
4	4	5	2	4	2	0	1	4
3	3	3	3	3	2	0	0	3
2	2	2	3	3	2	1	1	2
1	0	0	0	0	1	0	0	1
8	11	11	5	3	1	1	2	8
7	6	7	6	5	3	4	2	7
10	11	9	8	10	6	4	4	10
6	7	6	5	5	5	4	3	6
12	13	12	12	14	9	4	5	12
11	11	9	8	12	12	12	9	11
12	13	11	11	11	12	12	12	12
4	5	5	0	2	1	2	2	4
48	50	51	37	39	30	32	27	48
1	1	0	1	1	2	2	2	1
1	1	1	0	2	2	2	2	1
0	0	1	0	0	0	0	0	0
1	1	1	1	1	1	1	1	1
1	2	1	2	2	2	1	2	1
1	1	1	0	0	1	1	2	1
0	1	0	0	0	1	1	0	0
0	2	2	1	2	2	1	1	0
1	1	1	4	3	3	3	4	1
2	2	1	2	3	2	3	3	2
4	3	2	1	3	2	2	2	4
4	3	4	4	3	4	4	4	4
0	0	0	1	0	1	1	1	0
2	0	1	2	2	2	2	3	2
1	0	1	0	0	0	0	0	1
2	0	1	1	3	3	3	3	2
1	1	1	1	1	1	1	1	1

2	3	3	2	2	3	2	2	2
0	0	1	0	0	0	0	0	0
2	2	3	3	3	3	3	3	2
2	2	2	2	1	2	2	2	2
6	6	6	7	7	5	5	4	6
16	22	19	20	22	19	19	18	16
9	9	11	10	10	10	8	7	9
4	3	3	4	3	4	4	4	4
4	2	4	4	5	4	4	4	4
1	0	0	2	2	1	2	1	1
1	0	1	1	0	0	1	0	1
2	2	2	1	2	2	2	1	2
2	1	2	2	2	1	1	1	2
1	0	0	1	1	1	1	1	1
2	2	1	3	3	3	3	3	2
1	1	1	0	0	0	0	0	1
0	0	1	0	0	0	0	0	0
2	2	1	1	3	1	1	2	2
2	2	1	2	1	2	2	0	2
2	3	3	0	0	1	1	1	2
2	2	2	3	3	2	0	1	2
1	0	0	1	1	1	1	0	1
1	1	1	1	1	1	1	1	1
2	2	3	1	1	1	1	1	1
1	3	2	0	0	0	0	1	1
0	0	1	0	0	0	0	0	0
2	1	0	2	2	2	2	2	2
16	14	15	19	21	20	20	18	16
3	7	7	2	2	2	2	2	3
1	1	1	1	1	1	1	1	1
5	4	3	1	2	3	2	4	5
2	2	2	1	3	3	3	2	2
1	0	1	1	1	1	1	1	1
2	2	2	2	2	1	1	1	2
1	2	1	2	2	1	1	1	1
7	7	7	3	6	6	6	3	7
2	2	2	2	1	2	2	2	2
2	1	2	0	1	1	1	1	1
5	5	6	4	2	3	2	2	4
6	6	7	6	3	5	4	3	6
4	3	4	5	5	5	6	5	4
8	8	9	7	8	9	8	9	8
1	1	1	0	0	0	0	0	1
5	5	5	5	4	5	4	4	5
6	7	8	1	1	1	1	1	1
1	1	1	0	0	0	1	1	1
13	14	14	10	12	11	11	9	13
32	32	32	29	27	22	21	19	31
3	3	3	5	4	4	3	2	3
2	3	2	3	2	2	3	2	2

15	17	15	15	16	14	15	13	15
1	1	1	1	1	1	1	1	1
1	1	1	1	1	1	1	1	1
1	1	1	1	1	1	1	1	1
5	4	4	5	5	3	4	3	5
2	2	2	3	3	3	3	3	2
1	0	1	0	0	0	0	1	1
7	5	5	6	5	7	7	4	7
3	3	3	4	4	4	4	3	3
6	7	6	5	5	5	5	5	5
6	3	4	5	5	5	6	5	6
1	2	2	3	3	3	2	3	1
23	22	21	24	23	21	20	19	23
2	2	2	0	0	0	0	0	2
3	3	2	1	3	3	3	2	3
4	4	4	4	5	5	5	3	4
1	2	2	3	3	3	2	1	1
7	8	6	7	8	7	7	6	7
0	0	0	1	1	1	1	1	0
24	27	22	26	25	27	25	20	24
2	2	3	2	3	3	2	2	2
6	5	5	5	5	5	5	4	6
1	1	1	2	1	0	1	1	1
5	4	5	5	5	5	4	4	5
1	1	1	1	0	1	1	1	1
1	1	2	2	2	1	2	0	1
1	0	1	2	0	1	0	1	1
3	3	3	2	2	3	2	2	3
0	1	1	0	0	1	0	1	0
2	1	1	2	1	1	1	0	2
2	2	2	2	2	2	2	2	2
6	4	2	2	3	3	3	2	3
10	10	10	6	11	11	11	6	10
1	1	1	2	2	2	0	2	1
0	0	1	0	1	1	1	0	0
1	0	0	1	1	1	1	1	1
16	18	19	8	16	11	8	6	14
3	4	4	2	2	2	3	3	3
5	6	6	3	5	4	3	2	5
4	5	5	0	0	1	2	0	4
2	2	0	2	2	1	1	1	2
2	1	2	0	0	0	0	0	2
3	3	2	0	0	1	2	1	3
5	5	3	2	2	4	3	2	5
6	7	7	3	5	4	4	4	6
7	7	6	3	4	4	4	3	7
2	2	2	0	0	1	0	1	2
9	9	9	2	3	2	4	5	9
7	8	8	4	4	3	2	1	7
6	6	6	4	2	3	3	2	6

4	7	6	5	5	4	3	3	4
7	7	6	6	6	6	5	3	7
3	3	3	2	2	2	2	2	3
1	3	2	2	0	0	1	1	1
3	4	2	3	3	3	2	0	3
7	8	8	6	6	6	5	6	7
2	2	2	0	1	2	2	0	2
1	1	1	0	1	1	1	1	1
6	7	6	2	1	4	4	3	6
1	1	0	0	0	1	0	0	1
14	15	13	9	10	7	10	11	14
2	1	2	1	0	1	0	0	2
4	3	3	0	0	2	3	2	4
5	6	6	1	3	3	4	3	5
2	2	2	1	0	0	1	0	2
7	7	7	6	5	5	4	6	7
3	4	4	2	3	2	1	1	3
4	5	5	4	4	2	2	2	4
1	1	1	1	1	1	1	1	1
3	3	4	3	3	1	3	2	3
1	1	1	0	0	0	1	0	1
1	1	2	1	2	1	1	1	1
2	2	2	2	1	0	0	1	2
6	6	5	2	1	3	1	4	6
10	10	10	5	4	6	7	6	10
2	1	2	2	2	1	1	1	2
8	7	8	8	10	9	8	5	8
6	7	9	5	5	6	4	5	6
8	8	10	5	6	5	5	5	8
9	9	9	9	8	8	7	5	9
6	5	6	5	6	6	5	5	6
2	4	5	2	2	2	2	3	2
9	9	9	6	5	7	7	5	9
8	10	9	0	0	3	4	2	8
12	13	13	5	4	4	6	4	12
5	6	7	5	5	4	2	1	5
2	3	3	1	1	1	2	1	2
5	4	4	6	5	6	4	5	5
14	19	16	13	13	13	12	13	14
40	41	40	36	31	31	28	24	40
3	4	4	0	0	2	2	3	3
2	2	2	3	3	3	3	2	2
4	4	6	4	5	6	4	5	4
2	3	3	3	2	2	3	2	2
6	5	5	6	5	6	6	6	6
0	0	0	0	0	0	0	1	0
75	79	77	63	64	62	64	59	69
39	41	37	25	27	30	32	30	37
1	2	2	1	0	1	0	1	1
6	6	5	4	5	5	3	3	6

4	4	5	0	1	2	2	1	4
5	5	5	5	5	5	5	4	5
4	4	5	5	5	5	5	4	4
1	2	2	2	0	2	2	2	1
4	4	4	4	4	4	3	4	4
2	1	2	2	2	2	2	2	2
1	0	0	1	1	1	1	1	1
19	21	22	11	12	12	13	11	15
2	2	2	2	1	2	2	2	2
9	7	7	9	9	9	9	6	9
1	1	1	1	1	1	1	1	1
4	4	4	4	4	4	4	5	4
8	6	8	7	8	8	7	6	8
2	2	2	0	1	0	0	0	2
4	3	4	4	3	4	2	4	4
2	2	1	2	2	2	2	2	2
3	3	2	0	1	2	3	2	3
7	6	6	7	7	8	7	6	7
1	1	1	2	2	2	2	2	1
6	6	5	11	12	9	5	6	6
0	1	1	1	1	0	0	0	0
6	5	6	7	7	4	5	5	6
2	2	1	0	0	1	2	1	2
0	1	1	0	0	0	0	0	0
1	1	1	0	0	1	1	1	1
1	1	1	0	0	0	0	0	1
4	2	5	0	1	1	2	2	4
11	13	14	8	8	6	6	4	11
9	10	7	4	4	5	5	3	9
14	13	12	16	15	15	15	13	14
17	15	15	18	18	15	16	16	17
1	1	2	0	0	0	0	1	1
1	1	1	0	0	0	0	0	1
1	3	1	0	0	0	0	0	1
9	9	10	9	10	8	8	6	9
4	3	4	4	4	3	4	3	4
6	5	4	2	2	2	2	1	2
2	2	2	0	0	1	1	1	2
1	1	1	0	1	1	1	1	1
3	4	4	1	2	3	3	1	3
2	1	1	1	1	1	1	3	2
5	5	5	0	1	3	2	2	5
8	8	8	3	4	5	5	3	8
10	13	14	12	12	12	11	9	10
1	1	0	1	1	1	1	0	1
6	6	6	7	8	7	7	7	6
2	2	1	2	1	2	2	2	2
3	2	4	3	2	2	4	2	3
1	1	0	1	1	1	1	1	1
1	1	1	0	0	0	1	0	1

3	3	3	0	0	3	2	2	3
1	1	1	0	1	1	1	1	1
4	4	5	5	6	7	6	4	4
6	6	7	4	4	3	4	3	6
4	5	2	5	5	4	4	5	4
1	1	1	3	2	1	1	2	1
2	1	1	1	1	1	1	1	2
3	3	2	2	2	3	4	1	3
1	1	1	1	1	1	0	1	1
1	1	0	1	1	1	1	1	1
1	1	2	2	3	4	3	3	1
1	1	1	1	1	1	1	1	1
0	0	0	0	0	0	1	0	0
0	1	0	2	1	0	1	2	0
0	0	0	2	2	2	2	1	0
0	0	1	1	1	0	0	0	0
3	5	2	4	5	3	3	3	3
0	1	1	1	1	1	1	1	0
2	2	2	2	1	2	2	2	2
6	5	4	5	4	6	6	6	6
1	1	0	1	1	1	0	1	1
1	1	0	1	1	1	1	0	1
2	2	2	1	2	1	1	2	2
4	3	3	3	4	3	2	1	4
4	4	2	4	4	4	4	3	4
4	3	1	5	4	3	4	3	4
2	2	4	4	3	3	4	4	2
7	8	7	5	4	5	5	6	5
7	7	6	4	4	4	3	3	3
2	2	2	1	1	1	1	1	1
2	3	2	3	3	3	3	3	2
1	0	1	1	1	1	1	1	1
0	0	0	1	0	0	1	0	0
11	14	13	10	12	11	7	6	11
1	2	2	1	2	2	2	2	1
0	0	0	0	0	0	1	1	0
2	2	2	2	2	2	2	1	2
3	3	2	3	3	3	2	4	3
6	3	5	4	6	5	5	4	6
7	4	6	8	9	9	7	5	7
1	0	1	1	1	1	1	1	1
21	29	23	8	6	8	7	5	11
17	13	15	6	10	11	12	6	17
1	1	1	1	1	1	1	1	1
21	23	22	14	13	12	13	10	16
1	1	1	1	1	1	1	1	1
17	18	20	9	13	13	11	10	16
10	9	9	11	10	9	9	8	10
13	13	12	12	13	13	13	11	13
13	19	19	11	13	14	12	9	13

34	37	36	15	13	16	13	11	16
1	1	1	0	1	1	1	1	1
4	4	4	0	0	2	2	2	4
2	2	1	0	0	0	0	1	2
2	1	3	3	3	1	2	1	2
1	1	1	0	0	1	1	1	1
3	3	4	5	5	3	3	3	3
0	0	0	0	0	0	2	0	0
3	3	2	3	3	2	2	2	3
1	1	1	1	1	1	1	1	1
4	4	5	0	0	1	2	2	4
4	4	4	3	2	4	3	5	4
4	3	4	1	2	3	3	2	4
4	5	5	5	4	4	3	2	4
5	9	7	1	3	4	4	3	5
6	7	6	6	4	3	4	3	6
2	5	7	2	1	2	3	2	2
2	3	2	1	2	1	1	1	2
9	11	9	7	7	6	6	6	7
7	7	8	4	4	3	3	3	3
3	2	4	3	3	3	3	3	3
6	7	4	7	7	4	4	3	6
12	15	16	10	12	7	6	9	12
8	11	10	4	4	2	3	6	3
19	22	22	19	19	18	15	9	19
15	15	11	5	5	5	4	6	10
7	9	9	7	8	6	8	6	7
20	22	21	18	21	16	16	15	20
3	2	3	2	2	2	3	3	3
10	11	10	12	10	8	10	6	10
14	16	12	6	6	10	11	9	13
12	13	11	10	12	14	12	8	12
4	3	5	6	6	6	7	3	4
13	10	11	11	13	13	11	7	12
1	1	2	2	2	2	2	2	1
4	5	4	3	3	3	1	0	4
0	1	0	0	1	0	1	0	0
1	1	1	0	0	0	0	0	1
3	3	2	2	3	3	3	3	3
0	0	1	0	0	1	0	1	0
3	4	4	2	2	1	2	2	3
1	0	0	1	1	1	1	2	1
1	1	1	0	0	1	0	0	1
3	4	2	0	1	1	1	0	1
3	2	3	2	3	4	4	3	3
7	8	9	0	2	2	2	2	1
2	2	2	1	1	1	1	1	2
6	5	6	5	6	6	6	5	6
1	2	2	1	1	1	1	1	1
5	4	5	1	1	1	1	1	1

2	2	2	2	2	2	2	2	2
1	0	1	0	0	0	0	1	1
1	1	1	1	1	1	1	1	1
1	1	1	0	0	0	0	0	0
1	1	1	0	0	0	0	0	0
1	0	0	1	0	1	0	1	1
8	6	8	0	0	0	0	0	0
4	4	4	1	1	1	1	1	1
7	7	8	8	8	8	8	8	7
7	7	7	2	1	2	3	3	4
1	2	1	1	1	0	0	1	1
2	2	1	0	0	0	0	0	0
11	11	11	0	0	0	0	0	0
1	1	1	1	1	1	1	0	1
2	2	1	0	0	0	0	0	0
5	5	5	0	0	0	0	0	0
2	2	2	0	0	0	0	0	0
7	7	7	5	5	6	6	4	7
8	6	5	4	4	4	5	4	8
1	1	1	1	1	1	1	1	1
4	4	3	5	5	5	4	4	4
2	1	1	1	2	2	2	0	2
6	7	8	4	2	4	7	4	6
5	4	3	5	5	5	5	5	5
5	5	5	1	0	2	5	3	5
6	6	6	7	7	8	6	5	6
1	1	1	1	1	1	1	0	1
8	8	8	6	7	6	6	5	8
3	5	4	4	3	3	3	3	3
1	1	1	1	1	1	1	1	1
2	1	1	1	2	1	1	0	2
12	13	12	10	12	10	10	7	12
7	8	9	1	0	3	1	1	7
2	3	4	1	1	1	1	1	2
2	1	0	1	0	0	0	1	2
2	2	1	1	2	2	2	2	2
0	1	1	1	1	1	1	1	0
2	1	2	1	1	1	1	1	1
0	1	1	1	1	2	2	2	0
2	1	0	0	1	2	2	1	2
8	9	8	5	5	4	6	4	8
1	2	2	1	0	0	1	1	1
10	12	11	9	11	9	7	9	9
5	2	3	6	6	5	5	6	5
4	4	4	4	4	3	1	4	4
2	2	3	3	3	3	3	3	2
1	1	0	1	1	1	1	1	1
12	13	10	13	13	13	12	11	12
3	4	3	5	4	4	4	4	3
17	16	15	20	20	19	15	16	17

3	2	3	5	5	4	5	3	3
2	2	2	2	2	2	2	2	2
12	12	11	13	14	13	14	13	12
30	32	31	29	32	31	33	31	30
2	2	2	2	2	2	2	1	2
2	1	2	2	2	2	2	3	2
2	2	3	0	0	1	1	0	2
1	1	1	0	0	1	1	0	1
5	4	5	4	5	5	3	3	5
7	6	8	3	6	6	5	3	7
15	18	18	7	9	11	11	11	15
0	0	1	0	1	1	0	0	0
1	1	1	0	0	0	0	0	1
4	5	5	6	6	5	6	6	4
3	2	3	2	2	3	2	2	3
8	12	11	6	6	6	8	5	8
7	9	9	9	9	6	5	4	7
7	6	6	6	7	7	7	6	7
1	1	1	1	1	1	1	1	1
16	19	19	14	16	16	16	13	14
6	7	6	8	5	6	9	9	6
1	1	1	1	1	1	1	1	1
4	3	2	1	3	2	2	3	4
19	19	17	11	11	11	11	11	10
5	6	4	5	5	4	5	4	4
20	22	22	18	16	16	16	17	16
29	32	25	20	21	20	20	21	19
0	3	2	0	0	2	2	3	0
2	3	2	0	1	0	1	0	0
1	1	1	0	0	0	0	0	1
20	22	22	5	5	5	5	4	5
10	9	6	2	2	2	2	2	2
1	0	1	0	0	0	0	0	0
33	31	34	2	2	2	2	2	2
29	26	26	27	28	27	25	24	25
3	3	3	2	2	1	1	0	3
3	2	4	0	0	1	1	1	3
5	6	6	5	6	3	3	5	5
1	0	0	1	1	1	1	1	1
3	4	4	3	5	5	5	4	3
0	0	0	1	1	0	1	1	0
4	3	2	3	3	3	2	3	3
1	1	0	0	0	0	0	0	1
2	2	2	1	1	2	0	1	2
6	6	4	4	6	6	6	6	6
3	2	2	4	4	4	2	2	3
11	11	12	5	5	5	5	5	4
3	4	2	2	4	4	3	4	3
25	25	22	23	27	26	25	29	24
19	20	16	24	23	22	22	21	19

0	0	1	1	1	1	1	0	0
0	0	0	2	3	1	1	1	0
5	5	3	6	6	6	6	4	5
4	3	2	5	5	5	4	5	4
4	5	4	7	5	7	7	6	4
1	1	1	0	0	1	1	0	1
5	6	5	3	6	4	3	3	5
2	0	2	3	3	3	3	3	2
3	3	5	4	4	4	3	4	3
2	1	3	0	0	0	0	0	2
1	1	2	2	2	2	1	1	1
1	1	1	2	2	2	1	1	1
4	3	4	5	5	5	4	4	4
27	26	27	25	27	27	25	19	27
5	4	3	2	1	1	2	2	5
1	1	1	1	1	1	1	1	1
2	0	3	1	1	1	1	2	2
7	7	8	7	7	7	7	4	7
4	4	5	4	3	2	3	3	4
1	0	0	1	2	3	2	2	1
4	4	4	1	0	2	2	2	4
3	2	3	3	3	3	3	3	3
6	6	5	1	1	4	5	3	6
6	6	7	4	4	5	5	4	6
10	12	9	5	3	7	9	8	10
1	0	1	2	2	2	2	3	1
13	13	13	7	9	8	7	6	12
7	7	8	4	2	5	6	4	7
4	2	3	6	5	6	6	6	4
3	3	2	4	4	4	2	2	3
10	10	11	9	9	8	8	8	8
1	2	2	3	2	2	2	2	1
3	3	3	1	2	0	1	0	3
4	2	3	4	3	4	5	5	4
0	0	0	1	0	1	1	1	0
2	3	2	1	2	1	1	1	2
2	4	3	0	0	1	1	0	2
1	1	1	0	1	1	1	1	1
7	8	8	5	5	6	6	6	7
3	3	3	3	3	3	3	1	3
2	0	1	1	2	2	2	1	2
0	0	0	1	3	1	1	1	0
1	1	0	1	1	1	1	1	1
3	4	3	3	4	3	3	4	3
1	0	0	1	1	1	1	1	1
4	4	4	4	3	4	4	4	4
2	2	2	2	2	2	2	0	2
2	2	1	2	2	2	1	1	2
2	2	2	2	1	2	2	2	2
4	4	4	1	1	2	4	3	4

2	2	2	3	3	3	3	2	2
2	0	1	3	2	2	2	4	2
1	0	1	0	1	1	1	0	1
1	1	1	1	1	1	1	1	1
2	2	2	2	1	2	2	2	2
10	8	11	8	7	10	11	9	10
4	5	6	1	2	3	4	4	4
17	16	17	18	18	17	17	16	17
3	3	1	4	4	3	4	3	3
1	1	1	0	0	0	0	0	1
0	0	1	0	0	0	0	0	0
1	1	1	1	1	1	1	2	1
4	1	3	4	4	4	2	4	4
1	1	0	0	0	0	0	0	1
1	1	2	0	0	0	1	0	1
12	15	15	14	15	11	9	7	12
2	2	4	4	2	3	3	3	2
5	4	3	3	5	6	7	8	5
0	0	0	0	0	0	0	1	0
2	1	1	1	2	2	1	1	2
0	0	0	0	1	2	1	2	0
1	1	1	1	1	1	1	1	1
3	3	3	3	3	3	3	3	3
4	4	4	3	3	3	3	3	3
0	0	0	1	1	1	0	1	0
4	4	3	3	4	4	4	2	4
2	2	2	2	1	1	1	2	2
1	2	1	1	1	1	1	1	1
3	2	2	3	2	4	4	2	3
3	3	2	4	3	3	4	4	3
2	2	1	2	2	2	2	2	2
1	1	1	0	0	1	1	0	1
3	3	2	3	3	3	3	3	3
2	2	2	0	0	1	1	1	2
4	5	6	3	4	3	3	2	4
1	1	1	0	1	1	0	1	1
3	2	1	3	2	3	3	2	3
1	1	1	1	1	1	1	1	1
0	1	1	0	1	1	1	1	0
1	0	0	1	1	1	1	1	1
2	2	2	1	2	1	2	0	2
2	0	1	1	2	2	2	2	2
3	4	2	5	4	4	4	3	3
1	0	1	1	1	1	1	1	1
14	10	10	15	15	13	13	14	14
1	1	1	1	1	1	1	1	1
2	1	1	1	2	1	2	1	2
1	1	0	0	0	0	0	0	1
5	6	7	0	1	2	2	3	5
2	3	3	0	1	1	1	1	2

1	2	0	2	2	2	2	2	1
1	1	1	1	0	0	1	0	1
3	3	3	2	2	2	2	2	3
1	1	1	1	1	1	1	1	1
5	5	5	5	5	5	5	1	5
3	3	1	2	2	2	2	1	3
4	2	3	4	4	4	4	3	4
4	4	5	2	4	4	3	3	4
0	1	0	1	1	0	0	0	0
1	2	2	1	2	1	1	1	1
1	1	1	1	2	2	2	2	1
1	1	1	2	2	0	0	0	1
5	4	4	5	4	6	6	5	5
1	1	1	1	1	1	1	1	1
2	2	2	2	2	2	2	1	2
1	1	1	1	1	1	1	0	1
5	5	5	5	5	5	5	5	5
4	2	3	3	2	1	3	2	4
8	7	7	9	7	7	9	6	8
8	6	6	7	8	7	7	5	8
0	1	1	0	1	0	0	0	0
0	0	1	1	1	1	1	1	0
3	3	3	3	3	3	3	1	3
2	2	2	3	3	5	5	5	2
1	1	3	3	2	3	1	1	1
1	2	2	0	0	0	0	0	1
5	5	5	5	4	5	5	4	5
1	2	1	1	1	1	1	1	1
3	3	3	3	3	3	3	3	3
15	19	18	6	4	7	8	6	11
2	2	2	2	2	2	2	2	2
2	1	2	3	3	3	2	2	2
1	1	2	1	1	1	1	1	1
3	4	4	4	4	4	5	5	3
3	2	2	2	2	3	3	2	3
1	0	0	0	1	1	1	1	1
1	1	0	1	1	1	1	1	1
6	7	5	5	3	3	4	3	6
2	2	1	2	2	0	1	1	2
14	12	13	9	13	13	12	10	14
2	1	1	2	2	2	1	2	2
3	2	3	3	3	4	2	2	3
0	0	1	1	1	1	1	1	0
2	2	2	2	2	2	2	1	2
43	46	44	44	46	44	43	35	43
1	0	1	1	1	0	1	1	1
7	8	7	6	5	4	5	5	5
1	0	1	1	2	2	1	2	1
7	8	8	7	8	6	6	4	7
1	1	1	1	1	1	1	1	1

0	0	0	0	1	0	0	0	0
2	1	1	2	2	0	0	1	2
87	88	88	83	78	62	57	60	73
2	2	2	0	0	1	2	2	2
7	7	7	5	5	6	7	5	7
2	2	2	0	0	0	0	0	2
0	1	1	2	2	2	2	2	0
10	11	8	12	11	10	12	5	10
2	3	2	4	3	2	1	0	2
2	1	1	2	2	1	2	3	2
4	4	4	3	2	2	3	2	4
4	3	3	4	4	4	3	3	4
1	1	1	1	1	1	1	1	1
1	0	0	1	1	1	1	1	1
0	1	0	1	1	0	0	0	0
1	1	2	1	1	1	1	1	1
1	0	1	2	2	2	2	2	1
0	0	2	0	1	2	2	0	0
1	0	0	1	1	1	1	1	1
1	1	1	1	1	1	1	0	1
4	4	5	5	5	5	5	4	4
1	1	0	0	0	0	0	0	1
2	1	1	2	1	3	3	3	2
1	2	3	4	3	3	3	3	1
1	2	2	1	0	1	1	0	1
2	2	2	2	2	2	2	2	2
0	0	1	0	0	0	1	1	0
1	0	0	1	0	0	0	0	1
2	2	1	2	2	1	2	2	2
3	3	2	0	0	0	0	2	3
0	1	0	2	1	2	2	2	0
113	113	120	101	107	120	120	109	113
0	1	1	1	0	1	0	0	0
2	2	2	2	3	3	2	3	2
21	24	23	1	1	1	1	1	1
1	1	1	1	1	1	1	1	1
2	1	1	2	2	2	2	2	2
7	6	6	8	8	8	8	4	7
4	4	3	4	4	4	3	4	4
4	4	4	4	4	4	4	3	4
1	1	0	1	1	1	1	1	1
3	3	3	2	3	3	3	2	3
3	3	3	1	0	0	2	0	3
2	0	1	3	3	3	2	3	2
7	6	7	3	3	4	4	2	7
1	1	1	0	0	0	1	0	1
0	0	0	1	1	1	1	1	0
5	6	5	6	6	6	6	6	5
0	1	2	0	0	0	0	2	0
10	11	12	7	10	9	6	7	10

4	4	4	4	3	4	4	2	4
2	1	1	1	1	1	1	1	2
5	6	6	0	0	3	4	4	5
1	1	0	1	1	1	1	1	1
3	3	3	0	0	0	1	1	3
2	3	4	2	2	1	2	2	2
1	1	1	0	0	1	1	1	1
1	1	1	0	1	1	0	1	1
2	3	3	2	3	3	1	2	2
5	4	3	2	3	3	3	3	5
31	28	35	33	30	38	36	38	31
4	2	3	5	4	5	5	5	4
5	4	3	5	4	4	4	4	5
4	2	3	3	4	4	4	4	4
10	10	10	10	9	10	10	8	10
6	4	3	5	5	5	5	4	6
8	5	7	7	5	7	5	5	8
4	4	3	2	4	4	4	5	4
1	1	1	1	1	1	1	1	1
1	0	2	2	2	2	2	2	1
5	4	4	5	5	5	5	5	5
7	7	7	7	7	5	6	4	7
3	3	2	5	4	5	4	5	3
2	2	2	2	2	2	3	1	2
3	0	0	3	4	3	2	3	3
0	1	1	0	0	0	0	0	0
6	2	3	6	4	4	5	5	6
3	5	3	1	3	3	2	2	3
3	3	3	0	0	1	0	2	3
1	0	1	1	1	1	1	1	1
9	8	7	4	5	6	6	6	6
0	1	1	2	2	0	1	2	0
2	2	3	2	1	4	2	3	2
3	1	1	1	2	3	4	3	3
0	0	0	0	0	0	0	1	0
8	4	9	6	8	8	7	6	8
0	0	0	0	0	1	1	0	0
2	2	2	2	2	2	2	2	2
1	1	1	0	1	1	1	0	1
1	0	0	0	1	1	1	0	1
9	10	8	8	9	10	10	7	9
9	10	10	9	10	10	9	7	9
1	1	1	2	2	1	2	2	1
14	15	17	14	14	14	15	14	13
3	1	2	2	2	3	3	3	3
19	23	20	17	17	16	17	19	19
0	1	0	0	0	0	0	0	0
10	11	11	9	10	10	10	11	10
1	0	0	1	1	1	1	1	1
3	3	1	3	3	2	3	1	3

4	5	3	4	4	5	4	5	4
3	2	1	2	2	3	3	3	3
3	3	4	4	4	2	3	4	3
10	9	12	9	8	9	8	11	10
1	1	1	0	1	0	1	0	1
1	1	0	0	0	0	0	0	1
6	8	6	2	2	1	2	2	3
3	3	3	3	3	3	2	2	3
4	4	3	3	1	3	4	3	4
2	2	1	0	0	0	0	1	2
1	1	1	1	1	1	1	1	1
1	1	1	1	0	1	1	0	1
2	2	2	3	3	3	3	3	2
1	1	1	1	1	1	1	1	1
6	6	7	6	6	6	6	7	6
1	1	1	1	1	1	1	1	1
0	0	1	1	1	0	0	0	0
0	0	0	1	1	1	1	0	0
8	9	9	10	9	9	9	7	8
1	0	1	0	0	1	0	1	1
4	5	4	5	5	4	3	3	4
0	0	0	0	0	1	1	1	0
2	2	3	3	3	3	1	1	2
1	1	1	1	1	1	1	1	1
1	1	1	1	1	1	1	0	1
3	3	3	1	0	0	1	1	3
6	9	8	2	2	6	7	5	6
2	1	1	1	1	1	1	1	2
2	1	1	2	1	2	2	2	2
1	1	1	1	1	1	1	0	1
9	10	11	3	3	2	2	2	2
8	9	9	9	9	7	9	4	8
1	1	1	1	1	1	1	1	1
0	0	1	0	0	1	1	2	0
2	3	3	0	1	1	1	2	2
2	2	2	1	1	2	2	2	2
3	3	3	0	0	1	1	0	3
10	11	9	5	8	6	9	9	10
1	1	1	1	1	1	1	1	1
1	1	1	1	0	0	1	1	1
0	0	1	0	0	0	0	0	0
2	2	2	2	2	2	2	1	2
1	0	1	1	0	0	1	1	1
6	7	5	2	3	7	6	5	6
1	2	1	3	2	2	1	1	1
12	13	13	8	8	8	8	9	9
6	7	6	3	3	4	3	5	6
7	7	7	8	8	7	3	5	7
6	6	6	6	6	6	6	5	6
8	9	8	6	7	8	7	8	8

1	1	0	1	1	1	1	1	1
0	0	0	0	1	1	0	0	0
1	0	1	1	2	1	2	2	1
14	12	13	15	14	16	15	13	14
1	0	1	1	1	1	1	0	1
4	3	2	1	3	3	3	5	4
1	0	0	1	1	1	1	1	1
2	2	2	2	2	2	1	1	2
1	2	3	1	2	2	1	2	1
8	9	9	3	3	2	2	4	8
1	1	2	0	1	2	2	2	1
8	8	7	7	7	6	6	6	8
1	0	1	1	1	1	1	1	1
3	6	6	0	0	2	1	3	3
0	0	1	1	1	1	0	1	0
2	3	3	1	1	1	1	2	2
3	4	4	2	2	2	3	2	3
1	1	1	1	1	0	1	0	1
3	3	2	3	3	3	3	3	3
5	7	6	0	0	2	2	2	5
1	1	1	0	0	1	1	1	1
14	14	13	10	13	13	12	11	14
8	8	11	5	4	5	5	5	8
1	0	1	1	1	1	1	1	1
1	1	1	1	1	2	2	1	1
1	1	1	0	1	1	0	0	1
6	8	7	2	2	3	4	2	6
1	1	1	0	0	0	1	1	1
3	3	2	3	3	3	3	3	3
0	0	1	0	0	0	0	0	0
0	2	2	1	2	2	1	0	0
1	2	1	1	1	1	1	2	1
6	7	6	4	8	8	8	7	6
2	2	1	3	3	3	3	3	2
8	9	11	3	3	6	7	7	8
6	6	5	3	3	2	4	1	6
17	17	19	4	4	7	8	4	11
9	9	8	2	2	2	3	3	9
5	5	5	2	3	4	5	3	5
0	1	1	0	0	0	0	0	0
1	1	1	1	1	1	1	1	1
73	75	76	86	88	87	79	69	73
1	2	2	1	0	2	2	2	1
1	0	0	1	1	1	1	1	1
5	5	5	6	5	6	5	4	5
13	15	14	13	14	12	11	12	13
0	0	0	1	1	1	1	1	0
8	8	8	4	5	4	4	3	6
4	6	5	3	4	4	4	2	4
7	7	6	2	2	7	7	6	7

13	17	16	12	13	10	9	7	9
2	2	2	1	1	1	2	2	2
2	3	2	2	2	1	1	1	2
2	1	2	0	0	0	0	0	2
1	1	1	1	1	1	1	1	1
2	2	2	2	1	1	1	1	2
7	7	7	7	8	8	7	5	7
7	6	5	8	8	8	8	6	7
3	1	2	3	2	3	3	2	3
9	10	10	4	5	5	4	3	7
3	5	4	5	5	3	1	2	3
1	1	2	3	2	2	1	2	1
1	0	0	1	1	1	1	1	1
1	1	1	0	0	0	1	1	1
0	1	0	0	0	0	0	0	0
1	1	0	0	0	0	0	0	1
34	34	36	31	34	32	32	34	33
7	8	10	6	7	7	7	7	7
2	2	2	2	2	1	1	2	2
4	5	6	4	7	4	5	5	4
12	12	12	9	10	9	8	6	12
1	0	1	0	1	1	0	1	1
5	6	4	5	5	5	5	3	5
1	1	1	1	0	0	0	0	1
1	1	1	1	1	1	1	2	1
5	5	4	4	5	6	7	7	5
4	4	4	4	4	1	0	0	4
6	4	4	6	5	5	5	4	6
1	1	1	1	0	1	1	0	1
11	11	9	10	8	13	14	11	11
1	1	1	1	1	1	1	1	1
16	17	18	9	9	8	7	6	9
6	7	5	3	4	7	6	7	6
0	0	0	1	2	1	2	1	0
6	6	5	7	6	4	2	2	6
2	2	2	2	2	2	2	2	2
8	9	8	9	7	5	7	4	8
1	1	1	0	1	1	1	1	1
2	2	2	2	1	2	2	1	2
1	0	0	1	1	2	1	2	1
1	0	1	1	2	1	0	2	1
0	0	0	0	0	0	0	1	0
1	1	1	1	1	1	1	1	1
2	0	1	2	1	2	2	2	2
3	4	4	3	4	4	5	4	3
7	5	5	8	8	7	8	7	7
3	1	2	3	2	3	4	3	3
2	2	2	2	2	2	2	2	2
5	6	5	7	6	6	6	4	5
9	9	9	7	7	7	8	7	9

6	5	6	4	5	5	5	4	6
0	0	1	0	0	0	0	0	0
2	2	2	0	2	1	0	0	2
12	12	11	12	11	11	12	9	12
9	15	13	12	14	13	13	10	9
6	6	7	4	2	7	5	6	6
12	14	14	7	7	6	7	4	12
3	3	3	6	5	5	5	4	3
1	0	2	2	2	1	1	1	1
2	3	2	2	1	2	1	1	2
3	6	6	4	4	4	3	3	3
2	1	2	2	2	2	2	1	2
3	3	3	3	3	3	3	1	3
3	4	2	4	3	0	1	1	3
2	2	1	1	2	2	1	1	2
5	6	4	1	0	4	1	2	5
1	1	2	2	2	2	1	1	1
4	5	4	0	0	4	3	3	4
2	1	1	1	0	1	2	1	2
3	2	3	0	0	0	0	0	3
1	1	1	0	1	1	1	0	1
2	2	1	0	1	2	0	0	2
4	5	5	4	3	3	3	3	4
6	7	6	2	4	6	5	5	6
6	10	10	3	5	5	4	3	6
3	2	3	0	0	1	1	2	3
5	8	7	4	5	3	2	5	5
6	9	8	6	5	6	5	3	6
2	3	1	0	0	1	1	0	2
1	1	1	0	0	1	1	0	1
5	5	4	4	4	5	4	3	5
2	2	2	0	0	0	0	0	2
2	1	2	1	2	2	2	1	2
1	1	1	0	0	1	1	1	1
7	7	6	3	2	2	3	3	7
3	3	3	3	2	3	3	2	3
2	2	1	1	1	1	1	1	2
1	1	2	0	0	0	0	0	1
2	2	1	1	0	2	1	3	2
7	5	6	4	5	5	4	5	7
2	2	1	2	2	2	2	1	2
1	1	1	0	0	0	0	1	1
1	2	1	1	0	1	1	1	1
2	3	4	3	2	2	3	3	2
2	1	2	2	1	2	2	1	2
3	3	3	0	0	2	3	3	3
1	1	1	1	1	1	1	0	1
1	1	1	0	0	1	1	1	1
5	6	5	3	3	3	4	3	5
5	5	5	4	4	5	5	3	5

5	5	5	0	1	1	1	4	5
19	17	17	21	21	18	19	12	19
19	20	20	16	16	14	14	13	14
8	11	8	12	12	12	13	11	8
9	9	8	12	9	11	9	9	9
0	0	0	1	0	1	1	1	0
1	1	1	2	2	4	4	3	1
6	7	4	8	8	7	8	7	6
0	0	0	1	0	1	1	1	0
1	1	1	1	1	1	2	1	1
2	2	3	3	3	2	2	2	2
0	1	1	1	1	0	1	1	0
5	4	4	3	5	4	3	4	5
3	3	3	0	1	3	0	1	3
18	15	14	19	20	18	18	16	18
1	1	1	1	1	0	0	0	1
2	3	3	2	2	1	2	2	2
1	1	1	2	1	2	1	1	1
0	0	0	0	0	1	1	0	0
1	1	0	1	0	0	0	1	1
2	2	2	2	2	2	2	2	2
2	2	2	1	0	0	0	1	2
2	2	2	1	2	2	2	1	2
0	0	0	1	1	1	1	1	0
1	1	1	2	2	2	2	2	1
0	1	2	0	0	0	1	2	0
2	2	2	1	2	2	2	2	2
5	4	4	3	4	5	5	4	5
2	2	1	2	2	1	1	2	2
0	0	1	1	0	0	0	1	0
1	0	1	1	1	1	1	1	1
6	8	9	3	4	4	4	3	6
4	4	4	0	1	1	2	1	4
1	2	0	0	2	1	1	0	1
1	1	1	1	1	1	1	1	1
2	2	2	1	0	2	2	1	2
0	1	1	0	0	0	0	0	0
4	5	3	1	1	1	1	2	4
9	9	8	10	10	9	10	12	9
2	2	1	1	0	1	0	0	2
6	6	7	5	4	4	4	5	6
1	1	1	1	1	1	1	1	1
3	3	3	4	4	3	5	6	3
2	2	2	1	1	1	2	2	2
2	3	3	2	3	2	2	1	2
4	4	4	4	4	4	4	3	4
1	1	1	0	0	0	1	0	1
4	5	4	4	5	5	3	1	4
2	2	2	1	2	2	2	1	2
3	3	5	5	4	3	3	2	3

2	2	2	2	2	2	2	1	2
4	3	4	4	3	3	4	4	4
6	7	7	1	0	2	4	4	6
1	1	3	3	3	3	4	3	1
1	1	1	1	1	1	1	1	1
1	1	1	1	2	2	1	1	1
3	4	4	3	2	3	3	3	3
2	2	1	2	1	2	2	2	2
1	1	1	0	1	1	0	1	1
3	4	4	4	4	4	4	4	3
2	2	2	3	3	3	3	3	2
1	0	0	1	1	1	1	1	1
0	1	1	1	1	0	0	0	0
1	1	1	1	1	1	1	1	1
1	1	1	0	1	0	0	1	1
5	5	5	3	3	3	4	5	5
2	2	1	3	3	2	2	2	2
5	5	3	0	0	2	4	3	5
6	2	5	7	4	4	7	6	6
2	1	1	3	3	3	2	1	2
3	7	6	0	0	1	2	1	3
4	4	4	2	1	2	1	2	4
7	7	8	2	2	5	5	2	7
2	2	2	1	1	1	0	0	2
1	1	1	0	0	0	0	0	1
1	0	1	1	1	1	1	1	1
19	17	17	10	10	8	9	8	11
0	1	1	0	0	0	0	0	0
0	1	1	0	0	0	0	0	0
2	2	3	2	2	1	1	3	2
2	2	1	2	2	2	1	2	2
1	0	0	0	0	1	1	1	1
0	0	1	1	0	0	0	1	0
3	2	2	1	2	2	2	3	3
0	0	0	1	1	1	1	1	0
1	0	0	1	1	0	0	1	1
2	2	2	2	2	1	1	1	2
5	5	4	7	5	7	6	6	5
3	3	3	3	3	3	3	2	3
4	2	4	5	3	4	5	5	4
0	1	1	0	0	1	1	1	0
33	34	37	3	3	3	3	3	3
1	1	1	0	0	0	0	0	1
13	12	12	6	5	9	8	5	13
2	2	3	3	3	3	3	3	2
4	4	4	4	4	4	3	2	4
1	1	1	1	1	1	1	1	1
9	8	8	6	6	7	8	6	9
3	3	3	2	2	2	3	2	3
5	9	8	0	4	3	4	3	5

32	33	33	19	19	21	24	16	31
1	1	2	0	0	1	1	0	1
5	8	9	2	2	2	2	2	2
2	2	2	2	2	0	2	2	2
1	0	1	1	1	0	0	1	1
5	5	5	4	4	5	5	5	5
9	10	9	8	8	9	8	6	9
10	12	12	13	12	12	5	8	10
6	5	7	6	6	7	5	4	6
10	11	11	4	3	4	4	3	4
2	2	1	2	2	2	2	3	2
3	3	4	4	4	4	3	4	3
1	1	1	1	1	1	1	1	1
1	1	1	1	1	1	1	1	1
6	6	5	7	7	6	5	5	6
5	4	3	3	4	4	4	4	4
2	1	2	2	2	1	1	0	2
1	0	1	1	0	1	1	1	1
6	7	7	7	7	7	6	5	6
4	4	4	3	5	4	4	4	4
14	14	15	12	16	13	12	11	14
1	1	1	1	1	1	1	1	1
0	1	1	0	0	1	0	1	0
2	1	0	2	3	3	3	3	2
3	3	3	2	2	3	2	2	2
5	6	3	2	2	2	2	2	2
3	3	1	1	2	1	1	1	1
1	1	1	1	1	1	1	0	1
4	4	4	2	2	2	2	1	2
1	1	1	1	1	1	1	1	1
2	2	2	3	3	3	3	2	2
2	2	1	2	1	2	2	2	2
2	2	3	3	2	2	2	3	2
3	3	3	4	4	4	4	2	3
8	8	7	5	7	7	6	5	8
3	2	3	3	2	2	2	3	3
1	0	1	1	1	1	1	1	1
1	1	1	1	1	1	1	1	1
2	2	2	2	2	2	2	1	2
2	2	3	2	2	2	2	2	2
4	2	5	5	5	4	3	2	4
1	2	2	2	2	0	1	1	1
1	1	2	1	1	1	1	0	1
2	3	2	0	2	1	1	1	2
2	2	2	0	0	0	1	1	2
1	1	2	1	0	1	1	0	1
5	7	7	6	8	5	5	2	5
2	2	3	2	2	2	2	2	2
5	7	7	4	6	5	6	5	5
1	1	1	1	1	0	0	0	1

2	2	2	2	2	2	2	1	2
2	1	2	3	2	2	3	2	2
5	4	5	5	5	5	5	3	5
2	1	2	1	2	2	1	2	2
2	3	3	2	3	2	2	2	2
2	2	2	1	1	2	2	1	2
4	4	4	1	2	2	3	1	4
0	0	1	0	0	0	0	0	0
5	5	5	1	1	1	1	1	1
5	4	4	4	4	4	4	4	5
9	8	10	6	5	6	6	6	9
2	2	3	0	0	1	0	0	2
3	4	4	1	2	2	1	2	3
2	2	2	1	2	1	1	1	2
2	3	4	2	2	1	1	1	2
1	1	1	0	0	0	0	0	1
11	10	10	10	10	12	13	14	11
3	4	4	3	3	4	3	3	3
3	1	3	4	4	4	4	5	3
3	3	3	2	2	3	3	3	3
1	0	1	0	1	1	1	1	1
2	3	2	4	2	4	4	4	2
4	6	4	5	5	5	4	6	4
2	5	3	1	4	1	1	1	2
3	3	2	3	3	3	2	3	3
3	2	2	4	3	3	4	3	3
2	2	1	2	2	2	2	2	2
2	2	1	0	0	1	2	1	2
2	3	0	2	3	2	2	2	2
13	10	13	3	5	12	13	13	13
4	3	2	4	5	5	5	4	4
0	0	0	1	1	0	0	0	0
10	11	10	4	4	9	9	9	10
8	9	7	7	7	7	7	7	6
2	2	2	0	0	0	0	0	0
3	3	3	3	3	2	3	3	3
0	0	0	0	0	0	1	0	0
5	4	4	4	5	5	5	4	5
2	2	1	0	1	2	2	2	2
6	7	6	5	6	5	4	5	6
3	3	2	1	2	3	3	3	3
5	6	5	1	1	3	2	3	5
3	3	3	0	0	3	3	3	3
1	1	1	1	1	1	1	1	1
2	1	2	1	2	2	2	2	2
5	5	4	3	4	4	4	1	5
1	1	1	2	2	1	1	1	1
2	1	2	1	2	2	2	2	2
3	4	4	5	5	5	5	5	3
1	1	1	1	1	1	1	0	1

1	0	0	1	1	1	1	1	1
1	0	1	1	1	1	1	1	1
2	2	1	0	2	2	2	3	2
1	1	1	1	2	2	2	2	1
11	10	10	8	8	8	8	5	11
7	11	12	7	10	8	6	9	7
5	5	5	2	4	2	3	2	5
2	1	2	0	0	0	0	0	2
17	18	16	21	20	20	20	19	17
8	4	7	2	3	3	3	3	4
2	1	1	1	0	2	2	3	2
1	1	1	1	1	1	1	1	1
1	0	1	1	1	1	0	0	1
4	3	2	0	0	4	4	2	4
3	3	3	0	1	1	3	3	3
1	1	0	1	1	1	1	1	1
1	1	1	1	1	1	1	1	1
4	2	2	4	3	3	4	4	4
0	0	1	0	0	0	0	0	0
1	1	1	0	0	0	0	0	1
1	0	0	0	0	1	1	1	1
11	14	13	13	14	14	12	12	11
1	1	2	1	2	1	1	1	1
13	15	13	14	13	12	13	11	12
3	2	2	2	1	3	2	2	3
6	5	7	7	6	7	5	7	6
2	2	1	1	2	2	2	1	2
0	0	0	0	0	0	0	1	0
10	6	7	9	8	10	9	8	10
7	6	6	6	7	7	7	4	7
0	1	0	2	2	1	1	1	0
1	1	1	1	1	1	1	1	1
5	5	5	5	4	5	5	4	5
2	3	3	2	2	2	2	2	2
2	2	2	2	2	2	2	1	2
4	5	3	3	3	3	4	4	3
2	5	6	3	5	4	3	3	2
4	7	5	5	6	5	7	5	4
2	2	1	0	1	1	1	0	1
1	1	2	1	2	2	2	2	1
1	1	1	1	0	0	0	0	1
5	4	4	0	1	2	4	2	5
1	1	1	1	0	1	1	1	1
2	2	2	2	2	2	2	2	2
9	11	9	5	6	7	8	5	9
2	3	3	0	0	0	0	0	2
7	7	7	4	5	3	5	3	6
4	4	4	4	5	4	4	2	4
2	2	2	0	0	1	1	0	2
7	9	9	3	3	2	2	1	3

2	1	2	1	1	1	1	0	2
1	3	0	1	0	0	1	0	1
2	2	2	1	1	2	2	1	2
4	5	5	3	4	2	3	3	4
4	3	4	2	2	2	1	2	4
2	1	1	2	2	2	2	1	2
2	4	4	1	1	1	1	0	2
10	13	13	5	8	8	8	6	10
6	6	5	0	1	3	3	1	6
12	11	11	12	12	12	11	8	12
3	2	3	2	1	2	1	1	3
1	0	1	1	1	1	1	1	1
0	1	1	0	0	1	0	0	0
2	2	2	0	1	2	2	2	2
2	2	2	0	0	2	2	1	2
2	2	1	3	3	5	5	6	2
4	4	5	6	5	5	4	4	4
1	1	2	1	2	2	1	0	1
2	1	3	2	2	2	1	2	2
3	3	4	4	2	4	4	4	3
2	3	1	3	3	2	2	1	2
2	2	4	1	2	1	2	2	2
2	2	2	0	0	0	1	1	2
3	3	4	1	1	3	2	3	3
6	4	3	5	5	5	5	5	6
3	3	3	3	2	1	1	1	3
2	3	3	2	1	1	1	1	2
4	5	4	3	3	4	4	2	4
1	1	1	0	1	1	0	0	1
1	1	0	0	1	1	1	1	1
3	3	4	4	4	4	4	4	3
1	1	1	1	1	1	1	1	1
2	2	2	0	1	1	1	2	2
2	2	2	1	1	1	1	1	2
2	2	2	1	1	0	0	1	2
3	2	2	0	1	1	1	2	3
4	4	4	4	4	3	3	3	4
4	5	4	4	3	2	3	0	4
1	0	1	1	2	2	1	1	1
2	2	2	2	2	3	2	3	2
2	2	2	0	0	2	2	2	2
0	1	0	0	0	0	0	0	0
23	25	25	24	27	26	22	24	22
4	4	3	4	4	4	4	3	4
1	3	3	3	2	2	2	1	1
2	2	2	1	1	1	2	0	2
2	2	2	2	2	3	2	1	2
3	3	2	3	3	3	3	3	3
1	0	1	1	1	1	1	1	1
2	1	1	0	2	2	2	2	2

3	3	3	3	3	3	3	3	3
1	1	1	1	1	1	1	1	1
5	8	6	3	3	3	5	3	5
3	3	3	2	5	2	3	2	3
0	1	0	1	1	1	1	0	0
1	0	2	0	0	1	1	1	1
2	2	2	2	1	1	1	0	2
9	8	8	6	10	9	8	8	9
2	2	2	0	1	2	2	2	2
24	26	25	7	8	19	21	20	24
14	15	14	3	4	12	9	10	14
3	3	3	2	3	3	2	2	3
0	0	0	0	1	1	2	0	0
2	1	1	2	2	2	2	1	2
7	8	7	0	0	0	0	0	1
4	3	3	2	1	2	2	2	4
3	2	2	0	0	2	1	0	3
4	3	3	0	0	4	2	3	4
8	9	7	3	8	7	9	8	8
6	5	6	1	3	4	4	2	6
6	7	6	5	5	5	6	6	6
2	2	2	1	2	2	2	0	2
3	4	3	4	4	3	3	2	3
4	4	4	4	4	4	3	3	4
13	14	14	6	4	9	7	7	12
2	3	1	3	3	3	3	2	2
9	9	9	6	9	6	5	3	9
0	0	1	0	0	0	0	0	0
2	1	1	2	3	2	3	0	2
12	14	14	9	8	11	11	14	12
15	15	13	11	11	9	9	11	15
4	5	3	0	0	0	1	2	4
1	1	1	0	0	0	0	0	1
2	2	2	1	2	1	0	0	2
1	1	0	1	1	1	1	1	1
4	6	6	5	5	4	6	4	4
7	4	6	6	6	5	7	6	7
1	1	1	1	1	1	1	1	1
28	26	25	28	30	31	29	24	27
14	15	14	9	9	10	10	9	14
1	1	1	1	2	2	1	1	1
1	1	0	1	1	1	1	1	1
4	4	6	3	3	3	3	2	4
4	3	2	1	0	2	2	2	4
2	4	3	1	0	2	2	2	2
4	5	5	6	4	3	3	4	4
8	7	7	8	8	7	7	7	8
2	1	1	3	2	2	3	4	2
4	4	2	4	4	5	5	4	4
4	2	1	4	4	4	4	4	4

6	5	5	5	6	6	5	6	6
2	2	2	3	2	2	3	3	2
10	10	9	12	11	12	9	6	10
0	1	1	0	0	0	0	0	0
2	3	3	2	1	1	1	2	2
1	0	0	1	1	1	1	1	1
1	2	1	1	1	1	1	1	1
3	3	3	1	1	1	2	1	3
4	5	5	4	5	4	4	3	4
4	4	4	2	1	3	1	2	4
0	1	0	0	0	0	0	0	0
2	1	1	1	1	3	3	4	2
1	0	0	1	1	1	1	1	1
1	1	1	2	1	1	2	1	1
1	1	1	1	2	1	1	3	1
1	0	2	0	1	0	1	0	1
2	2	0	1	1	2	3	2	2
9	8	8	5	7	8	6	6	8
1	1	1	1	0	1	1	2	1
0	1	2	1	0	1	1	1	0
2	1	1	0	0	0	0	1	2
9	9	7	4	3	7	7	6	9
2	2	1	0	1	1	2	1	2
1	1	0	1	1	1	1	1	1
1	1	1	1	1	1	1	1	1
0	1	1	0	0	0	0	0	0
1	0	2	2	2	2	2	2	1
2	1	1	1	2	2	2	1	2
2	2	2	2	2	1	2	2	2
1	0	1	0	1	1	1	0	1
2	2	2	2	2	2	2	2	2
8	8	7	1	1	1	1	1	1
1	1	1	1	1	1	1	0	1
9	8	8	4	4	6	4	4	9
2	1	1	2	2	2	2	1	2
6	6	5	5	6	4	2	5	6
6	5	5	0	0	3	2	3	4
6	6	4	6	6	6	6	5	6
1	1	1	1	1	1	1	1	1
1	1	2	0	0	3	1	1	1
0	1	1	1	0	0	0	0	0
14	17	17	12	11	13	12	14	14
9	16	14	9	6	6	7	4	9
13	14	15	10	12	8	8	7	13
10	10	11	7	6	5	4	4	10
7	8	8	6	5	6	7	7	7
13	14	14	9	9	6	8	8	13
16	17	16	13	9	12	13	13	16
8	9	10	3	5	6	5	3	8
1	2	3	1	0	3	3	3	1

1	2	1	1	2	2	1	1	1
2	2	2	0	1	1	1	1	2
2	3	1	2	3	1	2	3	2
2	2	2	3	3	2	2	2	2
3	2	3	3	3	3	3	2	3
0	0	1	0	0	0	1	1	0
1	1	1	1	1	1	1	0	1
1	1	1	1	1	1	1	1	1
0	1	1	1	0	0	0	0	0
3	5	5	2	2	3	2	3	3
2	1	1	1	2	2	2	2	2
2	3	3	1	1	2	2	2	2
6	6	6	8	6	7	6	7	6
4	6	6	0	0	1	1	0	3
5	6	5	7	7	6	6	5	5
5	5	5	3	5	6	6	5	5
4	2	3	2	4	4	3	3	4
6	6	5	5	7	6	5	3	6
2	1	1	2	2	2	1	0	2
1	0	1	1	1	1	1	0	1
6	5	6	0	0	3	2	3	6
0	1	0	0	0	0	0	0	0
15	15	16	7	7	8	8	7	15
10	9	11	9	10	10	11	9	10
0	1	1	0	0	0	0	0	0
0	0	0	1	1	0	0	0	0
2	2	1	0	0	1	1	1	2
6	6	7	5	6	9	7	11	6
9	12	10	8	9	5	5	5	9
9	9	9	5	7	8	7	7	9
1	1	0	0	0	0	1	1	1
5	5	6	7	6	6	3	4	5
2	2	3	3	3	3	3	3	2
1	1	1	1	1	1	0	0	1
3	1	3	3	2	3	4	4	3
1	0	0	1	0	1	1	1	1
5	6	5	5	5	5	5	6	5
1	0	0	0	0	1	1	1	1
6	7	6	2	1	3	4	2	6
1	0	1	1	1	1	1	1	1
5	5	4	0	0	1	3	1	5
3	2	3	2	2	2	2	2	3
3	3	3	2	2	2	2	1	3
1	2	1	0	0	0	0	0	1
4	3	2	4	4	4	4	2	4
1	1	1	0	0	0	0	0	1
9	11	10	3	2	3	3	3	9
3	0	1	1	1	2	3	3	3
1	1	1	1	1	1	1	1	1
2	2	2	2	2	1	3	1	2

6	6	5	6	8	6	6	5	6
20	22	22	19	20	19	19	13	20
1	1	1	1	1	1	1	1	1
3	3	2	3	4	4	4	4	3
1	1	1	2	2	2	1	1	1
1	1	1	1	1	1	1	1	1
9	10	10	7	8	8	9	7	9
2	2	0	2	2	2	2	2	2
18	20	20	16	15	18	18	16	18
16	17	18	9	12	9	12	10	16
5	7	8	5	7	4	4	4	5
1	2	2	2	2	2	2	2	1
5	6	6	2	4	4	3	2	5
2	3	2	2	3	2	1	0	2
2	2	1	1	2	1	2	1	2
4	4	4	4	4	4	4	2	4
1	1	2	1	1	2	1	1	1
0	1	1	2	2	1	1	0	0
3	3	3	3	3	3	2	3	3
1	1	1	1	1	2	1	2	1
3	3	2	3	2	3	3	2	3
5	5	5	6	6	5	4	5	5
3	2	3	3	3	3	3	2	3
5	5	2	5	5	4	4	4	5
2	1	1	2	2	2	2	1	2
1	1	1	0	1	1	1	1	1
2	1	2	2	2	2	2	2	2
1	1	1	1	1	1	1	1	1
3	1	1	3	2	3	2	2	2
2	1	1	2	2	2	2	1	2
2	2	1	2	2	2	2	2	2
3	3	2	3	3	3	3	3	3
2	2	2	2	2	2	2	2	2
2	2	2	2	3	2	2	1	2
0	0	1	0	0	2	1	2	0
0	0	1	1	1	1	1	1	0
0	0	0	0	0	1	1	0	0
2	1	1	3	3	4	4	3	2
0	0	0	1	1	1	0	0	0
2	2	2	2	2	2	2	0	2
4	3	3	4	4	4	4	3	4
5	2	3	4	4	5	4	5	5
3	2	2	2	3	2	3	1	3
2	1	0	1	1	1	1	0	2
3	3	3	4	4	4	4	1	3
12	13	9	9	11	9	11	13	12
3	3	3	3	3	3	3	1	3
17	17	15	18	18	20	17	14	17
11	11	12	13	12	13	12	11	11
4	4	4	3	2	3	3	1	4

13	15	15	6	5	6	6	6	13
2	2	1	1	1	1	1	1	2
1	1	1	2	2	2	2	2	1
11	12	11	14	12	10	9	7	11
3	2	2	2	2	1	1	2	3
7	5	6	4	6	4	5	3	7
2	2	4	3	3	0	1	3	2
4	4	4	1	1	1	1	1	1
12	15	15	9	9	9	10	9	9
2	2	1	2	2	2	2	2	2
8	8	7	1	1	1	1	1	1
5	5	5	1	0	2	1	1	5
1	1	1	0	0	0	0	0	0
16	18	17	0	0	0	0	0	0
2	1	2	2	2	2	1	0	2
3	4	3	3	3	3	3	3	3
3	3	4	3	2	2	2	2	2
1	1	2	3	3	3	2	1	1
2	2	2	1	1	0	1	1	1
15	17	17	2	2	2	2	2	2
1	1	1	2	2	1	1	2	1
2	2	2	2	2	2	1	1	2
1	1	1	1	1	1	0	0	1
3	3	2	0	0	1	1	2	3
3	4	3	3	3	1	1	1	3
0	1	1	0	0	0	0	0	0
1	1	0	1	1	1	1	1	1
1	2	2	2	2	1	1	0	1
3	3	3	0	0	1	2	0	3
5	5	5	0	1	4	4	1	5
3	3	3	2	2	2	2	1	3
1	1	1	1	0	1	1	1	1
8	8	7	8	8	8	8	8	8
2	2	2	1	0	1	2	2	2
1	1	1	0	0	1	1	1	1
1	1	1	1	1	1	1	1	1
9	4	6	8	6	9	8	11	9
4	3	4	5	4	5	5	4	4
8	7	8	5	4	4	6	5	8
3	3	3	3	3	2	2	2	3
3	3	3	3	3	3	2	1	3
5	5	6	3	2	3	4	3	5
0	1	1	0	0	0	0	0	0
2	2	2	0	0	1	2	1	2
3	2	4	0	2	3	2	0	3
1	2	2	0	0	0	0	1	1
2	3	3	2	2	1	2	1	2
1	2	0	2	2	1	2	2	1
0	0	0	0	1	1	1	1	0
2	3	4	0	2	3	4	5	2

2	2	0	2	2	1	1	1	2
2	2	2	2	2	2	2	1	2
2	2	1	2	3	3	2	2	2
1	1	1	1	1	1	1	0	1
2	0	2	0	0	1	2	1	2
13	15	14	11	12	9	10	6	13
2	3	3	2	1	2	2	1	2
1	1	1	0	0	0	1	1	1
0	1	1	0	0	0	0	0	0
5	5	4	2	2	1	1	1	3
1	0	1	1	0	1	1	0	1
1	0	1	1	1	0	0	0	1
2	1	2	0	0	1	0	0	2
6	6	6	2	2	4	5	5	6
6	8	7	0	1	2	1	0	6
2	2	2	1	0	1	1	0	2
2	2	1	0	0	2	1	1	2
4	4	4	3	3	4	3	3	4
7	7	7	7	7	7	7	7	7
2	4	2	1	0	1	2	3	2
0	2	2	1	1	1	0	0	0
2	3	3	0	0	1	1	0	2
1	1	1	0	1	1	1	0	1
3	3	4	2	2	1	3	3	3
4	3	3	1	1	2	2	2	2
1	2	1	2	1	2	2	1	1
1	1	1	1	1	1	1	0	1
18	20	19	9	12	13	12	9	18
2	2	1	0	1	2	0	1	2
1	0	1	1	1	1	1	0	1
3	3	2	3	3	3	3	3	3
7	7	9	7	7	5	6	6	7
8	6	5	9	9	10	9	8	8
2	3	2	3	4	3	2	2	2
2	4	4	0	1	0	1	1	2
1	1	1	0	1	1	1	0	1
1	1	1	1	1	1	1	1	1
2	2	2	2	2	2	2	1	2
13	16	16	16	16	13	14	10	13
10	11	11	11	11	10	9	8	10
3	3	3	2	2	0	0	0	3
1	1	1	2	2	2	2	2	1
0	1	0	0	1	0	1	0	0
0	1	1	0	1	0	1	0	0
1	1	1	1	1	1	1	1	1
2	1	2	2	2	2	2	1	2
1	2	3	2	1	0	0	0	1
2	3	2	1	2	0	1	0	2
1	1	1	1	1	1	1	1	1
1	1	1	0	0	0	0	0	1

1	1	1	1	1	1	1	1	1
2	3	2	0	0	0	1	1	2
7	8	8	4	3	4	6	4	7
1	0	0	1	1	1	1	1	1
3	2	2	1	3	3	3	3	3
1	1	1	0	1	1	1	1	1
6	5	5	5	4	5	6	2	6
2	1	0	2	2	2	2	1	2
1	2	1	0	1	1	0	0	1
5	5	5	4	3	3	3	2	5
1	0	1	1	1	1	1	1	1
17	18	16	18	19	18	17	10	17
0	0	0	0	1	0	0	1	0
5	5	5	6	6	5	5	4	5
2	3	2	1	2	2	1	1	2
2	2	3	2	2	2	2	0	2
1	1	0	1	1	1	1	1	1
3	3	3	4	4	4	4	4	3
3	1	1	2	3	3	3	3	3
1	1	1	1	1	1	1	1	1
4	3	5	4	3	3	3	4	3
0	1	0	1	1	1	0	1	0
1	0	1	1	1	1	1	1	1
3	3	3	3	3	3	3	3	3
3	1	1	5	2	3	3	2	3
6	7	7	5	7	4	3	5	6
14	15	17	4	3	5	8	9	14
1	0	0	2	2	1	1	1	1
1	1	1	1	1	1	1	1	1
15	15	15	16	16	16	12	12	15
15	14	15	17	17	16	17	14	15
6	6	5	6	6	6	6	4	6
2	1	1	2	2	2	2	1	2
1	0	1	1	1	1	1	0	1
4	4	5	3	4	5	5	5	4
1	0	1	1	1	1	1	1	1
7	6	7	3	3	4	5	5	7
1	1	0	0	1	1	1	0	1
3	3	1	1	2	2	2	2	3
3	3	4	1	3	1	3	1	3
2	2	2	1	2	3	3	3	2
5	4	5	4	4	5	5	4	5
7	6	6	3	4	2	5	4	7
2	4	4	4	3	3	3	3	2
1	1	2	1	2	3	3	2	1
3	2	1	3	3	3	3	3	3
2	2	3	1	1	1	1	1	1
1	1	1	0	1	1	1	1	1
0	1	1	0	0	0	0	0	0
1	1	1	2	2	1	1	1	1

2	0	0	1	1	1	1	1	2
0	1	0	0	1	1	0	0	0
13	16	15	11	12	10	7	7	13
10	13	12	6	7	7	9	8	10
3	3	3	3	2	3	3	3	3
0	2	2	0	0	0	0	0	0
2	2	2	1	1	2	2	1	2
1	1	1	0	0	0	0	1	1
5	4	3	4	4	4	4	4	5
2	1	1	2	1	2	2	2	2
1	2	2	0	0	0	0	0	1
3	2	2	3	2	3	1	1	3
2	2	1	2	2	2	2	2	2
1	1	1	1	1	1	1	0	1
1	1	1	1	1	1	1	1	1
1	0	0	0	1	1	2	2	1
3	3	3	0	0	1	3	3	3

Unique pep	Unique pep	Sequence c	Unique + ra	Unique seq	Mol. weigh	Sequence l	Sequence l	Q-value
7	8	68.6	68.6	68.6	10.932	102	102;101;47	0
7	7	13.7	12.6	12.6	105.38	937	937;972;96	0
1	1	9.7	9.7	9.7	13.671	124	124;125	0
4	4	36.9	23	23	27.85	244	244;246;74	0
12	11	61.2	51.4	51.4	29.174	255	255;233;94	0
7	7	48	41.1	41.1	28.218	246	246;162;39	0
5	4	35.6	25.5	25.5	28.302	247	247	0
13	14	71.3	71.3	59.7	24.336	216	216;248	0
7	8	45.7	31.8	31.8	27.764	245	245;149	0
7	7	52.7	42.4	38.8	27.745	245	245;246;13	0
2	2	3.1	3.1	3.1	181.79	1729	1729;778	0
2	0	6.3	6.3	6.3	42.072	364	364	0
0	0	6.5	6.5	6.5	45.098	401	401;421	0
5	6	26.4	26.4	26.4	34.994	326	326;335;15	0
3	3	5.1	5.1	5.1	121.17	1087	1087;177;2	0
6	6	26.8	26.8	26.8	45.796	403	403;389;26	0
5	5	20.2	20.2	20.2	41.167	367	367;440;84	0
5	4	18.4	18.4	18.4	44.323	397	397;423;43	0
2	3	18.9	18.9	18.9	43.507	387	387;418	0
10	10	31.6	31.6	31.6	48.633	433	433;400;29	0
4	4	23.6	23.6	19	29.347	263	263;398;40	0
4	6	10.7	10.7	10.7	105.85	953	953;922;95	0
2	2	17.3	17.3	17.3	20.217	185	185;193;22	0
10	9	28.9	28.9	28.9	47.463	422	422;423;18	0
6	4	14	14	14	50.578	436	436;456;38	0
5	6	22.3	22.3	22.3	39.829	349	349;376;37	0
1	1	4.2	4.2	4.2	34.577	310	310	0.001542
9	9	15.5	15.5	15.5	100.2	908	908;778;74	0
5	5	14.9	14.9	14.9	41.183	356	356;534	0
0	2	4.7	4.7	4.7	41.079	380	380;377;19	0
3	3	13.1	13.1	13.1	41.181	350	350;351;38	0
3	3	23.2	23.2	23.2	20.064	177	177;198;32	0
1	2	17.6	17.6	17.6	16.913	153	153;179;22	0
5	4	22.1	22.1	22.1	20.63	181	181	0
1	1	15	15	15	20.459	193	193;194	0
1	1	18.2	18.2	18.2	11.15	99	99;123;137	0
1	2	29.5	29.5	29.5	14.374	129	129;225;13	0
4	2	23.6	23.6	23.6	29.395	258	258;215	0
3	3	9.8	9.8	9.8	33.249	296	296;270	0
1	0	16.1	16.1	16.1	10.688	87	87	0.001534
2	1	15.4	15.4	15.4	36.805	319	319;359;36	0
1	1	13.2	13.2	13.2	17.517	152	152;190	0
2	1	24.3	24.3	24.3	20.116	173	173;110;12	0
0	1	12	12	12	34.458	299	299;358;41	0
1	0	10.2	10.2	10.2	20.843	187	187;86;103	0
1	0	29.1	29.1	29.1	11.466	103	103;38	0
1	1	3.3	3.3	3.3	48.006	430	430	0
1	1	10.4	10.4	10.4	14.226	125	125	0.004801

2	1	19.8	19.8	19.8	19.903	172	172;271;24	0
1	2	6.8	6.8	6.8	45.834	396	396	0
12	9	22.5	22.5	22.5	117.24	1034	1034;1038;	0
1	1	5.2	5.2	5.2	26.614	231	231;325	0.007601
1	3	23.5	23.5	23.5	18.205	166	166;192;18	0
2	2	20.6	20.6	12.7	48.099	442	442;198	0
0	0	53.2	53.2	53.2	5.7685	47	47;147;178	0
0	1	15	15	15	20.576	180	180	0
1	1	11.1	11.1	11.1	24.43	225	225;305	0
1	1	11.8	11.8	11.8	24.325	212	212;232;12	0
0	0	17.1	17.1	17.1	24.915	216	216	0
1	1	6.1	6.1	6.1	29.485	264	264;292;48	0.001525
1	1	8.4	8.4	8.4	44.596	380	380;196	0
0	0	5.1	5.1	5.1	34.032	297	297;338;35	0.007608
1	0	15	15	15	14.056	127	127;163;22	0
1	1	14.9	14.9	14.9	16.567	148	148;169;18	0
0	0	5.7	5.7	5.7	37.535	332	332	0
1	1	13.1	13.1	13.1	11.348	99	99;140;166	0
1	0	23	23	23	15.524	135	135;158	0
1	1	20.5	20.5	20.5	12.107	112	112	0
1	1	3.7	3.7	3.7	30.243	267	267	0.00294
2	3	16.5	16.5	16.5	26.352	230	230;145;16	0
9	5	54	54	54	26.923	261	261;252;16	0
1	2	8.6	8.6	8.6	35.329	336	336;206	0
0	1	5.4	5.4	5.4	22.62	203	203;332	0
1	1	3.1	3.1	3.1	32.747	290	290;326	0.00892
3	5	27.9	27.9	27.9	18.898	165	165;176;17	0
9	8	53.2	53.2	53.2	18.431	158	158;118;79	0
7	7	53.8	53.8	53.8	14.515	132	132	0
5	5	43	43	43	17.222	151	151;148;11	0
5	7	38.4	38.4	38.4	16.273	151	151;120;13	0
1	1	61	61	16	11.477	100	100;130;58	0
6	5	39	39	39	16.445	146	146;100;12	0
5	4	51.9	51.9	51.9	15.55	135	135;136;19	0
6	6	32.9	32.9	32.9	17.718	152	152;82;33;[illegible]	0
6	7	43.4	43.4	43.4	16.06	145	145;71;76;[illegible]	0
9	9	31.7	31.7	31.7	31.324	293	293;195;23	0
3	3	22.7	22.7	22.7	13.373	119	119;142;46	0
4	4	46.9	46.9	46.9	8.85	81	81;83;90	0
4	4	28.7	28.7	28.7	15.807	143	143;134;55	0
3	3	26.7	26.7	26.7	15.197	131	131;132;13	0
2	3	22.4	22.4	22.4	13.742	125	125	0
3	2	31.3	31.3	31.3	13.015	115	115;115	0
4	4	39.3	39.3	39.3	9.461	84	84;66;100;[illegible]	0
2	2	33.3	33.3	33.3	7.8409	69	69	0
2	0	32.1	32.1	32.1	6.6767	56	56;67;53	0
11	11	54.7	54.7	54.7	26.688	243	243;259;23	0
1	1	10.2	10.2	10.2	10.905	98	98;59	0.000531
11	12	47.3	47.3	47.3	29.945	264	264;223;22	0
11	10	44.1	44.1	44.1	29.597	263	263;262;26	0

6	6	35	35	35	22.391	200	200;204;22	0
4	6	26.1	26.1	26.1	28.68	249	249;218;91	0
4	3	24.2	24.2	24.2	22.127	194	194;187	0
9	10	50	50	50	24.205	208	208;188	0
8	9	38.7	38.7	38.7	22.591	194	194;194;13	0
8	7	40.7	40.7	40.7	29.404	263	263;300;29	0
23	23	50.5	50.5	50.5	57.944	529	529;599;63	0
2	1	2.7	2.7	2.7	99.96	874	874;950	0
1	1	4.4	4.4	4.4	57.948	524	524;574;17	0
27	26	59.7	59.7	59.7	61.054	573	573;234;22	0
9	8	41.7	41.7	41.7	27.187	247	247;317;15	0
1	1	28.9	14	14	11.514	114	114	0
6	5	79.1	79.1	64.3	11.665	115	115;92;89	0
2	2	40.5	40.5	14.5	22.975	200	200;214;10	0
7	7	37.3	37.3	37.3	24.831	217	217	0
1	0	23	23	6.7	20.252	178	178;174;13	0
1	0	22.6	6.2	6.2	20.124	177	177	0.009786
6	6	54.5	54.5	54.5	17.818	165	165;132	0
5	6	34.1	34.1	34.1	24.261	211	211;164;12	0
7	6	37.4	37.4	37.4	23.577	203	203;210;14	0
4	4	36.3	36.3	36.3	14.558	124	124;215	0
6	6	25	25	25	24.146	204	204;133;17	0
5	6	50.3	50.3	50.3	19.586	169	169;171;17	0
4	4	27.4	27.4	27.4	18.756	164	164;167;19	0
5	5	37.6	37.6	37.6	16.714	141	141;147;15	0
2	2	29.9	29.9	29.9	9.8864	87	87;122;160	0
2	2	51.1	51.1	51.1	5.0827	47	47;48;53;7	0
4	4	37.9	37.9	37.9	14.865	140	140;81;91;	0
4	4	26.6	26.6	26.6	17.692	158	158;170;17	0
5	4	36.4	36.4	36.4	14.369	121	121;150;15	0
5	6	29	29	29	17.258	145	145;96;107	0
4	4	36	36	36	15.798	136	136;144;80	0
3	4	38	38	38	12.201	108	108;148;91	0
4	5	42	42	42	12.39	112	112;170;69	0
2	2	14.5	14.5	14.5	17.752	159	159	0
15	14	39.7	39.7	39.7	46.108	403	403;351;30	0
5	4	59.1	59.1	59.1	12.784	115	115;114;51	0
3	4	44.4	44.4	44.4	12.701	108	108;115;12	0
4	5	38.3	38.3	38.3	15.616	133	133;153;13	0
6	5	36.8	36.8	36.8	13.293	117	117	0
2	2	24	24	24	10.645	96	96;123	0
5	4	44.7	44.7	44.7	10.645	94	94;110;55;	0
3	3	28.6	28.6	28.6	12.254	105	105;94;68	0
2	2	15.2	15.2	15.2	13.228	112	112;118;14	0
3	3	69.1	69.1	69.1	7.624	68	68;92;60;7	0
1	1	15.6	15.6	15.6	7.5649	64	64;67;70	0.009772
13	13	35.1	35.1	35.1	47.697	427	427;333;17	0
9	9	38	38	38	34.362	297	297;130	0
13	12	29.5	29.5	29.5	32.728	288	288;159;16	0
5	6	33.2	33.2	33.2	24.433	208	208;248;88	0

12	12	44.4	44.4	44.4	29.995	266	266;151;19	0
1	1	5.1	5.1	5.1	22.874	198	198;153;24	0.003887
5	4	25.3	25.3	25.3	28.024	257	257;205;23	0
5	5	35.4	35.4	35.4	21.863	192	192;166;18	0
1	2	17.3	17.3	17.3	12.453	110	110;133;18	0.00155
9	10	30.9	30.9	30.9	51.872	470	470;483;20	0
1	1	17.3	17.3	17.3	18.912	179	179;216;25	0.001549
1	2	16	16	16	17.951	156	156;171;17	0
27	27	47.7	47.7	47.7	72.332	654	654	0
4	3	14.3	14.3	14.3	54.489	475	475;164;25	0
1	1	3.5	3.5	3.5	74.354	689	689	0.001535
3	2	1.9	1.9	1.9	231.71	2131	2131;1723;	0
1	0	4	4	4	56.732	525	525;632	0.001563
2	1	11.8	11.8	11.8	41.35	397	397;426	0
6	6	21.1	21.1	21.1	45.199	427	427;136;16	0
1	1	1	1	1	255.09	2256	2256;2268;	0
3	3	27.7	27.7	18.6	19.997	177	177;249;23	0
1	1	13.8	5.6	5.6	22.276	195	195;251	0.000536
8	9	15	15	15	87.819	805	805;780	0
1	1	69.6	4.5	4.5	41.736	375	375;125;16	0.008523
1	1	69.6	69.6	4.5	41.792	375	375;214;21	0
1	1	26.6	4.3	4.3	41.876	376	376;377;37	0
5	5	22.1	22.1	22.1	47.46	429	429;387;16	0
4	4	17.5	17.5	17.5	44.76	394	394;399;33	0
4	3	16.4	16.4	16.4	40.949	372	372;128;13	0
4	3	14	14	14	34.333	300	300;38;89;	0
1	0	13.1	13.1	13.1	9.7261	84	84;99;178	0.002956
4	4	37.2	37.2	37.2	13.057	113	113;181;62	0
1	1	25.9	25.9	25.9	14.807	135	135;151;15	0
1	1	7.8	7.8	7.8	16.941	153	153	0
7	7	23.2	23.2	23.2	47.371	418	418;367;84	0
3	3	26	26	26	14.395	127	127	0
7	6	35.8	35.8	35.8	32.339	288	288;338;21	0
0	0	9.6	9.6	9.6	15.434	136	136	0.001557
2	1	14.7	14.7	14.7	17.417	156	156;70;111	0
2	2	2.8	2.8	2.8	81.673	737	737;732	0
5	5	11.3	11.3	11.3	68.76	621	621;384	0
2	2	10.6	10.6	10.6	41.522	359	359	0
3	2	45.4	45.4	45.4	11.15	97	97;143;87;	0
0	0	10.3	10.3	10.3	12.366	117	117;140	0
1	1	18.7	18.7	18.7	23.333	209	209;319;17	0
5	5	19	19	19	43.206	379	379;406;43	0
2	2	19.3	19.3	19.3	17.981	166	166;197;21	0
2	2	15.9	15.9	15.9	17.659	164	164;231	0
0	1	13.8	13.8	13.8	24.843	224	224;226;24	0.002006
3	3	58.7	58.7	58.7	6.9189	63	63;148;153	0
1	1	7.7	7.7	7.7	32.663	299	299;339;38	0
1	1	9.8	9.8	9.8	34.084	305	305;327;34	0
14	14	39.6	39.6	39.6	47.716	432	432;404	0
4	4	30.2	30.2	30.2	25.63	232	232;224;23	0

2	0	9	9	9	25.268	223	223;171	0
5	5	32.4	32.4	32.4	23.411	210	210;194;13	0
1	2	8.9	8.9	8.9	40.826	360	360;480;48	0.002947
5	4	15.4	15.4	15.4	50.097	456	456	0
10	11	26.4	26.4	26.4	51.83	474	474;475;17	0
4	5	29.4	29.4	29.4	32.161	289	289;416;40	0
1	1	18.4	18.4	18.4	7.8547	76	76	0
5	4	43	43	16.4	32.852	298	298;315	0
2	2	42.3	15.8	7.4	32.866	298	298	0
3	2	10.9	10.9	10.9	53.96	496	496;497;22	0
3	3	43.6	43.6	20.4	20.697	181	181;181;21	0
1	1	33.1	7.9	7.9	14.553	127	127;180;15	0.001532
1	1	44.7	16.7	16.7	17.107	150	150;180;40	0.000539
2	3	24.6	24.6	24.6	20.082	175	175	0
1	1	10.2	10.2	4.8	21.539	186	186	0
2	3	20.6	20.6	20.6	19.961	180	180;186;23	0
1	2	2.3	2.3	2.3	88.583	797	797	0
4	4	4.2	4.2	4.2	202.29	1930	1930;1963;	0
1	1	4.2	4.2	4.2	37.636	330	330	0
0	1	6.8	6.8	6.8	16.008	146	146;1745;2	1
2	2	6.4	6.4	6.4	38.518	358	358;422;51	0
5	5	10.4	10.4	10.4	106.81	968	968;992;28	0
3	3	13.8	13.8	13.8	36.573	325	325;119;14	0
3	4	14.2	14.2	14.2	39.724	374	374;115;26	0
15	16	35.2	35.2	32	56.108	512	512;405;14	0
0	1	9.7	6.6	6.6	57.206	517	517	0
0	0	45.8	45.8	0	36.788	323	323;248	0
2	2	44.1	7.4	7.4	33.834	297	297;323;20	0
1	1	31.3	11.8	11.8	36.867	323	323;323;30	0
5	5	18.7	18.7	18.7	35.853	316	316;102;26	0
8	7	24	24	24	72.911	658	658;543;18	0
0	0	3.7	3.7	3.7	37.017	323	323;416	0.002021
1	1	1.5	1.5	1.5	94.069	819	819;754;81	0
1	0	7.3	7.3	7.3	41.465	357	357	0
20	18	50.8	50.8	34.6	103.06	892	892;914;88	0
20	20	46	30.2	30.2	104.85	911	911;692;52	0
3	5	19.8	19.8	19.8	51.853	479	479;521;53	0
1	1	5.4	5.4	5.4	37.433	332	332;376	0
0	1	14.3	14.3	14.3	13.133	119	119;140;18	0.002004
19	18	65.2	65.2	65.2	47.168	434	434;341;19	0
0	1	2.1	2.1	2.1	131.14	1144	1144	0
0	1	3	3	3	35.66	329	329;793;79	0.005731
3	3	28.5	28.5	28.5	29.163	256	256;295;26	0
1	1	3.5	3.5	3.5	61.89	546	546	0
4	4	9.9	9.9	9.9	59.681	527	527;394;41	0
4	4	18.6	18.6	18.6	34.352	312	312;336;15	0
3	3	17.2	17.2	17.2	35.348	320	320;242;25	0
5	4	12.8	12.8	12.8	68.168	611	611;650;17	0
2	2	5.7	5.7	5.7	57.933	522	522	0
7	5	14.7	14.7	13.6	80.832	714	714;733;74	0

6	5	11.7	10.7	10.7	85.486	751	751;761;76	0
0	1	3.2	3.2	3.2	41.385	373	373;410;47	0
1	1	1.1	1.1	1.1	118.01	1123	1123;1565;	0.001526
23	20	64.5	64.5	64.5	38.714	346	346;204;11	0
1	1	4.9	4.9	4.9	37.277	324	324	0
6	7	17.6	17.6	17.6	51.242	472	472;505;14	0
25	24	58.4	58.4	58.4	38.604	339	339;357;25	0
11	12	38.4	38.4	38.4	36.375	323	323;284;15	0
11	10	40.4	40.4	40.4	35.882	319	319;299;23	0
10	9	35.6	35.6	35.6	35.936	320	320;260;22	0
7	9	26.6	26.6	26.6	50.315	466	466;488	0
6	7	41.6	41.6	41.6	36.881	327	327;365;32	0
6	7	8.5	8.5	8.5	319.44	2896	2896;3256	0
2	1	3.1	3.1	3.1	87.175	808	808;808	0
2	3	8.5	8.5	3.8	101.35	919	919;939;94	0
2	2	13.1	13.1	13.1	22.065	191	191;158;13	0
1	2	4.5	4.5	4.5	105.36	955	955;977;63	0
2	2	13.3	6.8	6.8	74.136	663	663;677;88	0
2	1	9	9	9	49.526	434	434;460;43	0
0	1	1.5	1.5	1.5	111.24	984	984;1021;1	0.002005
1	1	5.5	5.5	5.5	46.939	418	418	0.000529
1	1	13.5	13.5	13.5	21.732	193	193;162	0.00293
3	4	1.7	1.1	1.1	515.6	4563	4563	0
1	1	13.7	13.7	13.7	12.815	117	117;99	0
2	1	4.9	4.9	4.9	48.915	449	449;337;12	0
1	0	22.8	22.8	22.8	13.101	114	114;178;18	0
4	4	10.3	10.3	10.3	56.769	504	504;510;44	0
2	3	22.6	22.6	22.6	18.129	164	164;173;17	0
5	5	18.6	18.6	18.6	66.294	609	609;613;27	0
6	7	9.9	9.9	9.9	145.44	1283	1283;1301;	0
1	1	2.9	2.9	2.9	41.595	374	374;256;30	0.009341
7	7	17.7	17.7	17.7	75.378	660	660;588;11	0
3	5	13.3	13.3	13.3	62.168	540	540;561;47	0
4	6	14.6	14.6	14.6	62.942	548	548;162;16	0
3	3	14.3	14.3	14.3	46.247	413	413;392	0
12	11	33.5	33.5	33.5	47.517	430	430;387	0
19	18	42.5	42.5	42.5	57.136	501	501;401;17	0
1	1	2.8	2.8	2.8	73.562	645	645	0
3	4	8.8	8.8	8.8	85.862	758	758;729;11	0
1	1	9.3	9.3	9.3	12.53	108	108;130;15	0.001531
1	0	3.3	3.3	3.3	102.89	968	968;1044;1	0.001564
2	2	13.1	13.1	13.1	47.4	413	413;412;56	0
2	2	9	9	9	58.772	523	523;541;16	0
4	4	36.4	36.4	36.4	22.275	195	195;256	0
1	1	4.4	4.4	4.4	24.817	226	226	0.001565
17	17	45	45	45	59.75	553	553;503;53	0
19	17	52.6	52.6	52.6	56.559	529	529;362;28	0
4	5	60.2	60.2	60.2	18.491	161	161;137;81	0
2	2	13.7	13.7	13.7	17.49	168	168	0
2	2	30.4	30.4	30.4	7.9331	69	69	0

2	0	47.3	47.3	47.3	6.2953	55	55;94;54;9	0
2	2	51.3	51.3	51.3	8.4518	76	76;103	0
7	9	33.3	33.3	33.3	32.881	297	297;298	0
6	6	46.5	46.5	46.5	23.277	213	213;242;86	0
4	4	45.4	45.4	45.4	12.587	108	108;116;11	0
3	4	13	13	13	66.217	586	586;634;57	0
0	1	5.3	5.3	5.3	20.616	188	188;702;71	0.008527
1	1	4.4	4.4	4.4	75.475	659	659;236;54	0
4	3	9.5	9.5	9.5	67.314	599	599;421;14	0
2	2	4.7	4.7	4.7	67.55	596	596;807;84	0
1	1	2.2	2.2	2.2	71.289	623	623;634	0
1	1	1.6	1.6	1.6	78.983	703	703;709	0
18	22	25.1	25.1	25.1	119.77	1091	1091;1101;	0
2	2	7.8	4.2	4.2	85.018	780	780;827	0
2	2	6.5	5.3	5.3	81.775	749	749;780;85	0
10	9	24.9	24.9	21.3	85.595	784	784;776;20	0
3	3	9.8	9.8	9.8	69.223	633	633;268;13	0
1	0	22.1	22.1	22.1	20.568	190	190;277;16	0
2	2	5.1	5.1	5.1	73.457	649	649	0
22	22	27.2	27.2	27.2	140.96	1270	1270;235	0
9	6	20.2	20.2	20.2	73.915	659	659;659;74	0
7	7	17.5	17.5	17.5	75.406	670	670;214	0
2	2	7.2	7.2	7.2	50.519	447	447;479;48	0
3	2	14.3	14.3	14.3	91.48	816	816;804;85	0
1	1	23.7	2.1	2.1	49.129	427	427;189;26	0
11	8	25.8	24.1	24.1	70.839	640	640;647;73	0
1	1	3	3	3	77.813	708	708;819;93	0.001577
3	5	11.4	11.4	11.4	98.594	881	881;882;82	0
1	1	4.1	4.1	4.1	87.99	786	786	0
1	0	20.3	20.3	20.3	7.6758	69	69;123;172	0
1	1	5.2	5.2	5.2	23.772	211	211	0
3	3	7.5	7.5	7.5	61.594	575	575	0
2	1	3.1	3.1	3.1	98.374	880	880;701;77	0
1	1	27	27	27	10.058	89	89	0
0	0	0.3	0.3	0.3	468.83	4391	4391	0.001995
4	4	11.3	11.3	11.3	40.538	353	353;419;42	0
2	2	13.9	13.9	13.9	31.247	274	274;408;41	0
4	5	51.2	51.2	51.2	22.764	205	205;269;38	0
3	3	23.2	23.2	23.2	27.991	246	246;313;18	0
1	1	22.2	22.2	22.2	16.872	153	153;211	0
4	3	13.3	13.3	13.3	52.917	467	467;703;72	0
3	2	11.1	11.1	11.1	48.871	461	461;509;32	0
3	3	35.3	35.3	35.3	13.714	119	119;71;101	0
1	1	2.4	2.4	2.4	57.949	540	540	0
1	1	4	4	4	42.293	376	376	0
2	2	13.9	13.9	13.9	32.805	288	288	0
3	2	26.7	26.7	26.7	21.994	195	195;241;12	0
3	2	6.7	6.7	6.7	62.328	564	564;593;96	0
21	21	20	20	20	170.59	1512	1512;968;1	0
1	0	14.1	14.1	14.1	26.849	248	248;350;22	0

13	13	31.6	31.6	31.6	64.523	591	591;592;23	0
0	1	0.3	0.3	0.3	330.46	3051	3051	0
2	1	16.4	16.4	16.4	16.932	152	152;86;88;8	0
1	1	16.3	16.3	16.3	10.863	98	98;125	0
4	3	12.9	12.9	12.9	56.882	511	511	0
1	1	4.6	4.6	4.6	36.297	325	325;349;38	0
1	2	13.4	13.4	13.4	33.242	292	292;292;31	0
2	1	14.3	14.3	14.3	27.835	251	251	0
1	2	1.8	1.8	1.8	175.38	1524	1524;1556	0
4	4	17.8	17.8	17.8	35.7	325	325	0
1	0	4.9	4.9	4.9	27.126	246	246;234	0.008065
4	4	9.3	9.3	9.3	52.541	493	493;497;46	0
14	12	20	20	20	110.61	1020	1020;935;1	0
4	3	10.5	10.5	10.5	54.812	475	475	0
1	1	6.8	6.8	6.8	22.712	206	206;308;32	0.008929
17	17	13.1	13.1	13.1	236.02	2162	2162;2225;	0
1	1	3.1	3.1	3.1	100.03	903	903;821;88	0
3	4	8	8	8	86.551	784	784;829	0
2	1	24.6	24.6	24.6	22.456	195	195;91;123	0
1	0	2.2	2.2	2.2	40.189	357	357;385	0.008945
15	12	28.6	28.6	28.6	74.175	675	675;676;57	0
3	4	15.7	15.7	15.7	51.354	458	458;477	0
6	6	10.8	10.8	10.8	104.71	953	953;903;91	0
6	6	45.4	45.4	45.4	21.228	185	185;228	0
17	17	31.2	31.2	31.2	67.567	592	592;627;48	0
4	4	38	38	38	15.958	163	163;196;19	0
7	7	12.9	12.9	12.9	81.889	714	714;122;13	0
6	6	11.4	11.4	11.4	79.994	700	700;622	0
3	5	17.3	17.3	17.3	46.133	423	423;431;43	0
7	6	35.6	35.6	35.6	32.616	298	298;330;30	0
14	15	47	47	47	48.141	417	417;247;16	0
1	2	18.8	18.8	18.8	26.326	224	224;265;31	0
2	1	10	10	10	42.981	381	381;153;33	0
1	1	10	10	10	22.873	200	200;382;40	0
4	4	7.3	7.3	7.3	70.353	628	628;694;70	0
4	3	19.2	19.2	19.2	29.246	260	260;97;101	0
10	11	62.8	62.8	62.8	30.375	277	277;178;22	0
4	4	9.6	9.6	9.6	152.93	1380	1380;1133;	0
7	6	14	14	14	86.238	756	756;773;11	0
1	4	9.6	9.6	9.6	73.776	658	658;635;64	0
2	3	13.6	13.6	13.6	27.741	236	236;264;32	0
0	2	9.9	9.9	9.9	45.31	385	385;391;39	0
2	1	11	11	11	26.673	228	228;234;30	0
2	1	6.2	6.2	6.2	54.822	501	501;712;50	0.002973
1	0	8.4	8.4	8.4	43.588	393	393;423	0.002928
4	6	20.7	20.7	20.7	59.755	527	527	0
5	5	51.6	51.6	51.6	24.449	221	221;271;22	0
16	18	31.8	31.8	31.8	100.07	906	906;930;84	0
2	5	11.4	8.4	8.4	84.765	774	774;781	0
14	12	21.5	21.5	21.5	104.85	938	938;832;83	0

3	5	20.7	20.7	20.7	43.688	405	405;406;40	0
2	3	11.2	11.2	11.2	30.993	277	277;122;19	0
5	5	4.7	4.7	4.7	274.37	2491	2491	0
7	8	51.1	51.1	51.1	20.471	178	178;167;13	0
2	1	2.3	2.3	2.3	120.97	1054	1054	0
0	1	3.1	3.1	3.1	152.66	1368	1368;1445;	0
2	2	15.7	15.7	15.7	38.138	344	344;532;30	0
2	2	10.3	10.3	10.3	41.767	388	388;493;53	0
6	6	31.6	31.6	31.6	22.683	206	206;287;34	0
3	3	29.6	29.6	29.6	11.985	108	108;130;12	0
2	3	17.4	17.4	17.4	26.818	242	242;267;15	0
1	2	6.3	6.3	6.3	81.742	742	742;786;83	0
2	2	42.6	42.6	42.6	5.1441	47	47;135	0
1	1	8.9	8.9	8.9	25.946	237	237;168;21	0
2	4	6.9	6.9	6.9	103.14	923	923;923;59	0
4	3	26.7	20.9	20.9	21.258	191	191;136;19	0
3	4	7.4	7.4	7.4	92.25	802	802	0
2	2	7.1	7.1	7.1	43.614	379	379;79	0
1	1	36.5	36.5	8.2	18.742	170	170;177;16	0
1	0	2.6	2.6	2.6	53.082	460	460;2319;2	1
1	1	28	28	28	13.415	118	118;272;27	0
2	1	41.6	41.6	41.6	8.6227	77	77;105;108	0
1	1	6.3	6.3	6.3	18.739	159	159;227;22	0.002937
5	5	25.9	25.9	25.9	24.95	224	224;233	0
9	9	52.3	52.3	52.3	26.922	241	241	0
2	2	6	6	6	73.011	652	652;654;65	0
1	1	1.7	1.7	1.7	79.845	704	704;706;71	0
1	0	3.9	3.9	3.9	31.293	280	280;416	0
0	1	15.3	15.3	15.3	15.769	137	137;201;29	0
1	1	17.1	17.1	17.1	8.5199	76	76;223;202	0
5	6	36.1	36.1	36.1	20.811	183	183;107;62	0
2	2	1.7	1.7	1.7	217.1	1905	1905;1937;	0
5	6	20.5	20.5	18.5	50.431	453	453;466;40	0
34	32	34.5	34.5	27.6	192.06	1679	1679;1639;	0
2	2	17.4	17.4	17.4	23.662	218	218;260;16	0
2	1	7.1	7.1	7.1	23.181	211	211;229	0.001575
1	1	25.6	25.6	25.6	13.649	129	129;145;19	0
3	3	19	19	19	22.744	211	211	0
1	1	6.1	6.1	6.1	27.11	244	244	0
2	2	12.9	12.9	12.9	22.077	209	209;217;22	0
3	3	14.5	14.5	14.5	26.227	227	227;71;149	0
3	2	11.9	11.9	11.9	52.269	478	478;478;55	0
1	1	5.4	5.4	5.4	22.62	204	204;219;28	0.001545
1	1	4.9	4.9	4.9	24.717	225	225;438;59	0
2	2	10	10	10	76.096	669	669;655;17	0
2	1	10.8	10.8	10.8	43.28	369	369;502;53	0
1	3	3.1	3.1	3.1	138.23	1236	1236;1251;	0
4	5	43.7	43.7	43.7	15.945	142	142;73	0
0	0	11.6	11.6	11.6	15.951	138	138;185;73	0.002948
9	8	10.4	10.4	10.4	138.34	1224	1224;1233	0

2	2	7	7	7	107.14	953	953;485	0
8	9	15.8	15.8	15.8	99.045	877	877;906;15	0
5	5	14.9	14.9	14.9	57.21	511	511;552;18	0
3	3	13	13	13	36.923	331	331;308;25	0
2	4	5.7	5.7	5.7	97.717	874	874;188	0
11	11	64.5	64.5	60.2	18.502	166	166;149;15	0
0	0	2.2	2.2	2.2	115.01	998	998;1221;1	0.001539
4	5	13.5	13.5	13.5	55.317	480	480;483	0
1	1	7.3	7.3	7.3	33.657	302	302;411	0
1	0	8.5	8.5	8.5	15.296	130	130;141;14	0.008515
1	1	26.1	26.1	26.1	9.1349	88	88;360	0.002944
2	3	31.1	31.1	31.1	15.512	151	151;151	0
5	4	7.8	7.8	7.8	85.746	767	767;798;81	0
9	5	10	10	10	142.34	1415	1415;1497;	0
7	6	33.7	33.7	33.7	31.362	282	282;177;17	0
0	1	1	1	1	157.18	1401	1401	0
3	4	7.1	7.1	7.1	81.572	730	730;741;60	0
2	2	3.6	3.6	3.6	114.33	1015	1015;269	0
0	0	7.2	7.2	7.2	50.539	444	444;828	0.000532
7	8	11.6	11.6	11.6	121.89	1118	1118;1146;	0
2	1	5.7	5.7	5.7	53.371	471	471;486;49	0
1	2	6.7	6.7	6.7	47.726	420	420;352;40	0
2	1	20.8	20.8	20.8	19.344	173	173;160;20	0.004799
8	8	24.4	24.4	22.7	60.13	537	537;234;13	0
0	0	4.2	2.5	2.5	62.233	552	552;553;56	0.004831
8	6	33.3	33.3	30.9	39.183	369	369;371;37	0
1	0	17.8	17.8	17.8	14.013	129	129;137;14	0
2	1	7.6	7.6	7.6	54.234	489	489	0
6	5	15.2	15.2	15.2	53.248	474	474;480;52	0
0	1	2.3	2.3	2.3	59.763	525	525	0.007594
1	1	23.9	23.9	23.9	7.5164	67	67;81	0
3	3	14	14	14	38.123	344	344;352;36	0
3	2	14.9	14.9	14.9	47.036	417	417;448;10	0
1	2	9.9	9.9	9.9	33.777	303	303	0
1	1	10.2	10.2	10.2	13.328	118	118;807	0.002975
2	2	7.6	7.6	7.6	56.101	513	513;445;51	0
5	5	12.4	12.4	12.4	66.69	591	591;360	0
1	2	4.9	4.9	4.9	92.931	836	836;649;34	0
1	1	2.5	2.5	2.5	55.142	514	514;468;48	0
1	1	1.3	1.3	1.3	102.75	900	900;717;89	0.001571
6	6	7.6	7.6	7.6	136.37	1230	1230;1062;	0
3	4	14.1	11.4	11.4	34.081	297	297;297;18	0
5	5	22.4	22.4	19.9	36.938	326	326	0
1	1	18.4	18.4	18.4	11.006	98	98	0
3	3	49	49	49	11.139	98	98;83	0
1	0	11.6	11.6	11.6	15.799	146	146	0.001527
5	6	46.6	46.6	46.6	20.567	193	193;153;16	0
2	2	9	9	9	37.489	332	332;116;12	0
1	0	10.3	10.3	10.3	10.631	97	97	0.004847
2	2	4.8	4.8	4.8	113.74	1036	1036;175;2	0

1	1	16.7	16.7	16.7	20.6	192	192;208;28	0
1	1	4.9	4.9	4.9	35.288	325	325;353;37	0.008941
0	0	21.4	21.4	21.4	11.268	98	98;124;134	0.001559
2	2	37.9	37.9	37.9	15.716	140	140;150;14	0
10	13	43.5	43.5	42.1	52.645	480	480	0
12	11	38.2	38.2	38.2	48.442	453	453;412;33	0
3	2	36.3	36.3	36.3	10.739	91	91;91	0
2	1	25.2	25.2	25.2	13.53	111	111;76;161	0
1	1	27	27	27	7.3084	63	63	0
2	2	25.5	25.5	25.5	29.668	274	274;283	0
2	2	24.8	24.8	24.8	11.333	101	101;105;10	0.001054
2	3	26.4	26.4	26.4	11.731	106	106;71	0
0	0	4.4	4.4	4.4	43.835	388	388;410	0.00054
1	1	16.3	16.3	16.3	10.853	98	98;63	0
2	3	14.5	14.5	14.5	25.565	227	227	0
4	4	26	26	26	19.576	169	169;83;126	0
2	2	15.7	15.7	15.7	17.235	153	153;150;69	0
3	2	25.6	25.6	25.6	13.696	129	129	0
3	3	55.8	55.8	55.8	10.192	86	86	0
3	2	28	28	28	8.7813	75	75	0
2	2	25.3	25.3	25.3	10.264	91	91;103;115	0
1	1	36.8	36.8	36.8	12.615	114	114;79;95	0
2	2	32.1	32.1	32.1	6.3824	56	56;63	0
2	2	27.2	27.2	27.2	9.3697	81	81	0
1	2	12.3	12.3	12.3	35.422	325	325	0
3	3	26.5	26.5	26.5	30.601	268	268	0
38	36	11.7	11.7	11.7	532.4	4646	4646;180	0
2	2	7.5	7.5	7.5	68.297	611	611;612;63	0
3	3	26.9	26.9	26.9	17.778	167	167;415;49	0
3	4	4.1	4.1	4.1	145.18	1253	1253;862;8	0
16	17	43.4	43.4	43.4	66.022	602	602	0
1	2	7.4	7.4	7.4	52.771	488	488;519;20	0
1	2	12.6	12.6	12.6	27.041	246	246;283;33	0.001994
5	4	14.9	14.9	14.9	52.878	475	475;391;20	0
1	2	31.5	31.5	31.5	16.474	146	146;532;56	0
9	8	24.3	24.3	24.3	55.938	526	526;532;53	0
3	3	14.4	14.4	14.4	35.02	327	327;406;40	0
1	2	51.4	51.4	51.4	7.6679	70	70;582;53;4	0
2	2	15.3	15.3	15.3	18.681	170	170	0
1	1	4	4	4	67.55	597	597;445;22	0
4	4	35.6	35.6	35.6	12.712	118	118;132;16	0
2	2	10.7	10.7	10.7	33.939	298	298;313;31	0
1	2	14.2	14.2	14.2	32.267	289	289;339;16	0
4	4	10.5	10.5	10.5	49.436	427	427;475;51	0
3	3	17.4	17.4	9.1	35.816	328	328;267;18	0
1	1	6.1	6.1	6.1	56.042	512	512;563;21	0
9	11	20.4	20.4	20.4	87.088	793	793;795	0
0	1	8.6	8.6	8.6	22.092	198	198	0
2	3	6.8	6.8	6.8	66.174	601	601;756;16	0
0	1	5.2	5.2	5.2	25.113	230	230;275;31	0

4	5	43.3	43.3	43.3	15.395	141	141;167;16	0
1	1	7.3	7.3	7.3	17.221	151	151;151;23	0.001572
1	1	19	19	19	18.038	158	158;239	0
1	0	1.2	1.2	1.2	93.768	847	847;901	0.003899
2	2	7.2	7.2	7.2	93.453	839	839;896;21	0
10	12	19.5	19.5	19.5	122.29	1118	1118	0
4	7	18.5	18.5	18.5	107.53	999	999	0
34	30	20.3	20.3	20.3	331.77	2871	2871;2428;	0
6	7	44.2	40	40	18.506	165	165;135;14	0
2	2	6.3	6.3	6.3	40.542	367	367	0
4	3	12.2	12.2	12.2	54.177	509	509;486;41	0
3	4	10.5	10.5	10.5	68.996	647	647;479;54	0
8	4	23.8	23.8	23.8	48.755	453	453;367;27	0
1	1	3.3	3.3	3.3	84.304	756	756;757;70	0
2	1	3.1	3.1	3.1	84.141	748	748	0
4	3	11.9	11.9	11.9	69.323	628	628;712;65	0
1	1	13.9	13.9	13.9	12.672	115	115;411;47	0
8	9	11.4	11.4	11.4	126.97	1140	1140;1092;	0
1	1	8.3	8.3	8.3	29.766	266	266	0.008891
5	5	13.9	13.9	13.9	57.208	490	490;357;38	0
4	5	10.9	10.9	10.9	77.592	691	691;734;15	0
3	3	3.5	3.5	3.5	103.19	921	921;868;93	0
2	2	4.2	4.2	4.2	119.79	1058	1058;1230;	0
0	0	9.9	9.9	9.9	22.653	192	192;223	0.003895
11	11	18.4	18.4	18.4	93.998	836	836;904	0
6	7	12.3	12.3	12.3	90.98	808	808;853;81	0
11	9	18.9	18.9	18.9	96.557	863	863;278;21	0
7	6	11.3	11.3	11.3	92.888	821	821	0
13	12	29.3	29.3	29.3	81.307	719	719;543;38	0
11	9	21.6	21.6	21.6	90.725	765	765;376;50	0
13	11	11.8	11.8	11.8	174.38	1531	1531;1557;	0
5	5	29.3	29.3	29.3	27.125	242	242;263;31	0
50	51	16.3	16.3	16.3	469.08	4128	4128;4097	0
1	0	2.3	2.3	2.3	194.81	1720	1720;1659	0
1	1	7.5	7.5	7.5	54.985	510	510;512	0
0	1	3.2	3.2	3.2	63.64	566	566;1970	0.008908
1	1	6.9	6.9	6.9	38.883	347	347;1230	0
2	1	3.7	3.7	3.7	133.06	1167	1167;1174;	0
1	1	8.2	8.2	8.2	38.646	342	342;346;12	0.001524
1	0	9.2	9.2	9.2	21.459	184	184;204;21	0.00157
2	2	14.8	14.8	14.8	37.044	331	331;397	0
1	1	17	17	17	45.745	412	412;134;47	0
2	1	8.8	8.8	8.8	49.611	453	453;480;30	0
3	2	13.5	13.5	13.5	38.044	340	340;183;24	0
3	4	18.4	18.4	18.4	40.513	358	358;167	0
0	0	6.4	6.4	6.4	19.777	172	172;276;40	0.001558
0	1	9.1	9.1	9.1	57.196	507	507;559;52	0
0	1	17.7	17.7	17.7	10.302	96	96;2243	0
0	1	11.5	11.5	11.5	51.911	453	453;504	0
1	1	17.2	17.2	17.2	22.149	198	198	0

3	3	9.1	9.1	9.1	50.096	438	438;494;55	0
0	1	4.2	4.2	4.2	29.909	260	260	0.004789
2	3	13.2	13.2	13.2	32.426	287	287;295;26	0
2	2	23.9	23.9	23.9	10.094	92	92;122	0
6	6	20.3	20.3	20.3	48.799	439	439;456;43	0
22	19	42.8	42.8	42.8	68.569	607	607;435;12	0
9	11	27.9	27.9	27.9	69.283	631	631;615;34	0
3	3	47.1	47.1	47.1	9.5541	85	85;113;65;	0
2	4	7.1	7.1	7.1	80.529	705	705;613;10	0
0	0	2.7	2.7	2.7	93.673	826	826	0
0	1	4.1	4.1	4.1	33.525	294	294;324	0.000539
2	2	6.1	6.1	6.1	54.945	493	493;494;49	0
1	2	2.8	2.8	2.8	132.65	1195	1195;931;1	0
0	0	4.7	4.7	4.7	19.15	172	172;249;27	1
2	1	11.1	11.1	11.1	40.16	359	359;406;32	0
1	1	3.3	3.3	3.3	40.763	367	367;393	0
0	1	8.8	8.8	8.8	44.424	400	400	0
2	1	12.3	12.3	12.3	36.172	318	318;347;35	0
2	1	2.9	2.9	2.9	139.09	1256	1256;1139;	0
3	3	5.6	5.6	5.6	79.621	712	712;699;71	0
2	2	4.4	4.4	4.4	113.68	979	979;924;96	0
0	0	0.3	0.3	0.3	538.59	4707	4707;4454;	1
1	1	49.4	24.7	24.7	10.366	89	89;42;47;6	0.002003
1	1	49.4	49.4	24.7	10.35	89	89	0
3	2	38.5	38.5	38.5	10.921	96	96;148;96;	0
0	1	1.5	1.5	1.5	132.29	1143	1143;1479;	0
1	0	4.5	4.5	4.5	97.44	895	895	0
14	15	11.4	11.4	11.1	358.2	3224	3224;1760;	0
7	7	14.1	14.1	14.1	70.973	630	630	0
1	1	6.5	6.5	6.5	21.392	185	185;213;23	0.004808
4	3	1.5	1.5	1.5	480.19	4358	4358;4365;	0
2	2	12.8	12.8	12.8	43.061	398	398;400	0
0	1	8.1	8.1	8.1	19.188	172	172;653	0
2	2	10.2	10.2	10.2	34.193	294	294;474;50	0
2	1	6.3	6.3	6.3	91.099	806	806;857;79	0
7	7	33.8	33.8	33.8	26.697	240	240;179;14	0
2	2	8.7	8.7	8.7	52.747	470	470;480	0.001521
0	1	8.5	8.5	4.9	34.721	306	306;548;53	0
4	5	13.3	13.3	11.3	61.174	541	541;175	0
6	7	25.8	25.8	25.8	36.091	326	326;353;15	0
3	4	28.4	28.4	28.4	30.22	289	289;303;33	0
8	9	44.7	44.7	44.7	27.843	255	255;346;17	0
1	1	20.7	5.2	5.2	50.47	463	463	0.002931
5	5	38.7	38.7	38.7	24.763	225	225;68;123	0
1	1	46.4	46.4	9.2	28.821	261	261;281;63	0
1	1	45.9	8.2	8.2	28.558	257	257;187;10	0
14	14	34.3	34.3	34.3	50.118	437	437;487	0
31	31	43	43	41.8	95.337	858	858	0
3	3	10.7	10.7	10.7	66.034	591	591;751;77	0
3	2	17.5	17.5	17.5	35.39	325	325;346;14	0

17	15	46	46	46	49.541	452	452;101	0
1	1	5	5	5	32.662	279	279	0
1	1	5	5	5	30.742	262	262;88;299	0
1	1	2.2	2.2	2.2	111.47	983	983;1332	0
4	4	22.8	22.8	22.8	28.994	254	254;219	0
2	2	9.8	9.8	9.8	55.016	500	500	0
0	1	10.2	10.2	10.2	14.245	128	128;139;32	0.002013
5	5	9.5	9.5	9.5	107.23	941	941;948;12	0
3	3	5.1	5.1	5.1	93.159	841	841;904;35	0
6	5	31.4	31.4	27.6	28.993	261	261;160;53	0
3	4	16.7	16.7	16.7	46.971	406	406	0
2	2	15.3	15.3	15.3	36.851	326	326;397;38	0
22	21	38.2	36.5	36.5	92.468	803	803;315;31	0
2	2	12.9	12.9	12.9	15.48	139	139;141;14	0.008971
3	2	37.5	37.5	37.5	12.259	104	104;71	0
4	4	18.6	18.6	18.6	30.895	285	285;302;24	0
2	2	18.7	18.7	18.7	40.183	364	364;359;39	0
8	6	34.5	34.5	34.5	31.387	290	290	0
0	0	5.2	5.2	5.2	38.006	346	346	0
27	22	35.2	35.2	35.2	134.28	1210	1210;1165;	0
2	3	40.2	40.2	40.2	13.078	122	122;148;17	0
5	5	26.3	26.3	26.3	37.893	342	342;314;19	0
1	1	5.2	5.2	5.2	56.035	502	502;522;60	0
4	5	18	18	18	52.948	455	455	0
1	1	2.2	2.2	2.2	50.219	456	456;535	0
1	2	8.6	8.6	8.6	24.844	220	220;186;21	0
0	1	3.8	3.8	3.8	111.69	992	992;992;99	0
3	3	11.8	11.8	11.8	34.833	297	297;135	0
1	1	17.9	17.9	17.9	9.1037	78	78;219;261	0.000533
1	1	8.1	8.1	8.1	23.773	210	210;100;12	0.004836
2	2	15.9	6.9	6.9	38.925	346	346;275;15	0
2	1	21.6	21.6	12.4	37.725	338	338;339;19	0
10	10	31.2	31.2	31.2	54.392	468	468;257;12	0
1	1	25.5	25.5	25.5	14.417	137	137;182;19	0
0	1	1.5	1.5	1.5	98.795	851	851	0.001999
0	0	5.7	5.7	5.7	26.973	261	261	0.001522
15	16	53.7	53.7	47.8	46.153	406	406;341;34	0
4	4	16.8	10.9	10.9	46.871	411	411;125	0
6	6	14.5	14.5	14.5	68.6	636	636;637;47	0
5	5	16.5	16.5	16.5	47.475	423	423;404;43	0
2	0	14	14	14	16.686	150	150;151;13	0
1	2	33.1	33.1	33.1	13.607	121	121;113;11	0
3	2	21.5	21.5	21.5	16.46	144	144;116;12	0
5	3	19.7	19.7	19.7	36.112	315	315;252;27	0
7	7	26.7	26.7	26.7	38.388	333	333	0
7	6	22.2	22.2	22.2	51.109	472	472;472;46	0
2	2	11.4	11.4	11.4	26.008	236	236;580;52	0.001553
9	9	9.7	9.7	9.7	166.57	1382	1382;1348	0
8	8	11.2	11.2	11.2	92.48	814	814;873;15	0
6	6	9.3	9.3	9.3	104.1	903	903;913;91	0

7	6	26.5	26.5	26.5	58.14	499	499;533;54	0
7	6	19.6	19.6	19.6	52.22	445	445;352;15	0
3	3	8.4	8.4	8.4	37.563	357	357;100	0
3	2	19.8	19.8	19.8	29.283	262	262;320;22	0
4	2	13.2	13.2	13.2	39.589	349	349;366;35	0
8	8	39.4	39.4	39.4	36.501	325	325;125	0
2	2	18.9	18.9	18.9	11.959	106	106;117;20	0
1	1	10.2	10.2	10.2	15.875	137	137;192;21	0.001567
7	6	15	15	15	70.901	607	607;564;51	0
1	0	13.5	13.5	13.5	8.4675	74	74;191;220	0.001567
15	13	15.3	15.3	15.3	158.52	1435	1435;1512;	0
1	2	5.4	5.4	5.4	49.652	444	444;869;90	0.002008
3	3	9.6	9.6	9.6	69.697	616	616;572;61	0
6	6	34.6	34.6	34.6	25.2	228	228;248	0
2	2	6.5	6.5	6.5	49.222	431	431;101;10	0
7	7	37.7	37.7	37.7	16.019	146	146;186;15	0
4	4	8.1	8.1	8.1	138.68	1220	1220;1220	0
5	5	33.1	33.1	33.1	26.599	245	245;108;22	0
1	1	11.5	11.5	11.5	15.105	139	139;143;16	0
3	4	23.5	23.5	23.5	28.235	272	272	0
1	1	5.7	5.7	5.7	29.408	263	263;293	0
1	2	12.4	12.4	12.4	31.821	291	291	0
2	2	7.9	7.9	7.9	63.616	559	559;796;92	0.001535
6	5	10.1	10.1	10.1	123.38	1071	1071;132;1	0
10	10	14	14	14	107.78	945	945;971;91	0
1	2	2.2	2.2	2.2	136.31	1204	1204;24;76	0
7	8	14.9	14.9	14.9	122.85	1104	1104;1114	0
7	9	23.5	15.7	15.7	69.371	586	586;586;58	0
8	10	12.8	12.8	12.8	119.91	1047	1047;66;81	0
9	9	19	19	19	81.074	709	709;173;89	0
5	5	39.5	39.5	36	32.922	286	286	0
4	5	32.9	29.4	29.4	32.949	286	286;146;17	0
9	9	35.3	35.3	35.3	30.628	272	272;260;30	0
10	9	24.3	23.1	23.1	68.604	653	653;655;64	0
12	13	28.3	28.3	27.1	73.027	711	711;711;27	0
6	7	25.5	24.1	24.1	61.64	572	572;261	0
3	3	11.3	11.3	11.3	40.532	353	353;419;80	0
4	4	22.2	22.2	22.2	52.623	445	445	0
19	16	44.4	44.4	44.4	54.529	493	493;132;13	0
41	40	27.2	27.2	27.2	273.2	2509	2509;2511;	0
4	4	47.4	47.4	47.4	15.164	135	135;101;10	0
2	2	11.3	11.3	11.3	37.481	335	335;515	0
4	6	21.9	21.9	21.9	44.648	393	393;485;50	0
3	3	11.9	11.9	11.9	48.295	420	420;677;49	0
5	5	35.5	35.5	35.5	21.225	183	183;113;15	0
0	0	0.5	0.5	0.5	198.69	1831	1831;1894	1
71	70	48.2	48.2	45	280.01	2639	2639;2647;	0
39	35	27.4	25.9	24.6	275.66	2578	2578;2591;	0
2	2	12.3	12.3	12.3	30.77	276	276;373;37	0
6	5	26.8	26.8	26.8	42.592	380	380;316;16	0

4	5	32.5	32.5	32.5	22.119	206	206;163;15	0
5	5	15.3	15.3	15.3	42.08	379	379;427;18	0
4	5	18	18	18	47.142	428	428;483;42	0
2	2	6.2	6.2	6.2	44.205	389	389;395;27	0.000533
4	4	13	13	13	68.326	608	608;539;62	0
1	2	22.2	22.2	22.2	11.901	108	108;135;21	0
0	0	2.7	2.7	2.7	75.579	674	674;706	0
17	17	63.2	63.2	51.4	39.42	364	364;418;36	0
2	2	19	7.1	7.1	39.455	364	364;135;14	0
7	7	31.7	31.7	31.7	50.212	467	467;510	0
1	1	16.4	16.4	16.4	12.43	116	116;87;113	0
4	4	18.6	18.6	18.6	32.632	290	290;317;35	0
6	8	71.9	71.9	71.9	14.716	135	135;38	0
2	2	9.6	9.6	9.6	26.152	250	250;233	0
3	4	8.5	8.5	8.5	65.33	585	585;144;20	0
2	1	18.8	18.8	18.8	13.667	117	117;57;70;[illegible]	0
3	2	33	33	33	10.504	94	94;188;103	0
6	6	16.6	16.6	16.6	75.935	673	673;729;78	0
1	1	7.5	7.5	7.5	43.955	388	388;194	0
6	5	21.3	21.3	21.3	85.686	781	781;748;76	0
1	1	1.2	1.2	1.2	236.28	2084	2084;2109	0.00295
5	6	12.5	12.5	12.5	107.97	957	957;977;97	0
2	1	2.9	2.9	2.9	107.89	962	962;973	0.001053
1	1	15.3	15.3	15.3	17.512	150	150;142	0
1	1	6.9	6.9	6.9	54.389	478	478	0
1	1	9.7	9.7	9.7	12.976	113	113;163;18	0.000538
2	5	24.9	24.9	24.9	26.987	237	237;274;28	0
13	14	35.9	35.9	35.9	59.256	515	515;545;56	0
10	7	20.2	20.2	20.2	64.824	573	573;558;56	0
13	12	37.9	37.9	37.9	60.192	535	535;525;52	0
15	15	37.3	37.3	37.3	61.397	558	558;425;39	0
1	2	3.3	3.3	3.3	68.629	599	599;637;17	0
1	1	5.2	5.2	5.2	28.135	252	252;274	0.009767
3	1	8.7	8.7	8.7	49.419	446	446;228	0
9	10	22.6	22.6	22.6	65.459	598	598;669;16	0
3	4	9	9	9	76.758	681	681;699	0
2	2	10.7	10.7	3.7	86.578	764	764;775;17	0
2	2	7.8	7.8	7.8	37.432	335	335	0.001554
1	1	8.9	8.9	8.9	16.628	157	157	0
4	4	14.1	14.1	14.1	50.496	469	469;479;52	0
1	1	20	20	20	21.707	190	190;183;22	0
5	5	24.1	24.1	24.1	27.566	241	241;200;20	0
8	8	57.6	57.6	57.6	23.356	210	210;174;79	0
13	14	56	56	56	31.548	293	293;335;26	0
1	0	2.8	2.8	2.8	48.705	434	434	0.004845
6	6	14.4	14.4	14.4	80.852	727	727;601;37	0
2	1	30	30	30	10.999	100	100;118;17	0
2	4	9.2	9.2	9.2	83.165	739	739;77;79	0
1	0	5.7	5.7	5.7	46.744	420	420;433	0
1	1	7.3	7.3	7.3	21.51	193	193;265;27	0

3	3	6.6	6.6	6.6	54.719	502	502;298;31	0
1	1	6.7	6.7	6.7	38.695	358	358;328	0
4	5	18.3	18.3	18.3	61.68	558	558;486;29	0
6	7	14.1	14.1	14.1	65.928	594	594;693	0
5	2	7.5	7.5	7.5	134.55	1179	1179;1192;	0
1	1	4.9	4.9	4.9	78.759	668	668;696	0
1	1	10.4	10.4	10.4	33.81	298	298;285	0
3	2	14.8	14.8	14.8	39.768	384	384;452;46	0
1	1	2.5	2.5	2.5	60.593	528	528	0.008916
1	0	16	16	16	9.4134	81	81;174;236	0
1	2	8.7	8.7	8.7	70.472	620	620;1002;3	0
1	1	11.2	11.2	11.2	15.578	134	134;137	0
0	0	7.3	7.3	7.3	20.969	193	193;463;48	0.002967
1	0	5.6	5.6	5.6	58.041	518	518;687;69	0
0	0	7.2	7.2	7.2	47.27	417	417;424;45	0
0	1	6.4	6.4	6.4	37.205	345	345	0.001996
5	2	28.6	28.6	28.6	24.279	217	217	0
1	1	9.6	9.6	9.6	18.192	157	157;187;22	0
2	2	12.2	12.2	12.2	21.424	188	188;189;18	0
5	4	30.5	30.5	30.5	26.224	233	233;216;23	0
1	0	20.3	20.3	20.3	8.6739	79	79;184	0
1	0	10.4	10.4	10.4	12.964	115	115;155;19	0
2	2	4.3	4.3	4.3	97.031	839	839;868;87	0.001529
3	3	27.1	15.5	15.5	40.361	354	354;302;14	0
4	2	28.2	16.6	16.6	40.45	355	355;366;30	0
3	1	69.4	69.4	69.4	8.0061	72	72	0
2	4	26.5	13.5	13.5	37.377	340	340;332;10	0
5	5	30.3	30.3	17.4	37.331	340	340;232;25	0
3	3	26.6	26.6	15	40.532	354	354;350;35	0
1	1	7.2	7.2	4.2	42.142	359	359;170	0
3	2	11.3	8.4	8.4	44.179	379	379;380;39	0
0	1	7	3.9	3.9	42.123	359	359;355	0.00153
0	0	4.3	4.3	4.3	33.287	282	282;377	0.004824
14	13	68.1	68.1	68.1	35.076	317	317;273;19	0
2	2	6.3	6.3	6.3	60.54	537	537;549	0
0	0	1.2	1.2	1.2	97.23	901	901;781;80	0.001519
2	2	14.6	14.6	14.6	20.834	199	199;217	0
3	2	31.4	31.4	31.4	17.201	153	153;90;132	0
3	5	13.4	13.4	13.4	57.673	514	514;420;20	0
4	6	6.4	6.4	6.4	233.27	2063	2063;2144;	0
0	1	2.2	2.2	2.2	54.794	509	509	0.007605
15	11	52.5	52.5	29.4	70.108	642	642;641;64	0
13	15	32	30.4	30.4	94.33	840	840;699;47	0
1	1	15.4	1.7	1.7	71.027	643	643;367	0
17	16	50.5	46.3	36.4	70.897	646	646;627;49	0
1	1	32.9	32.9	32.9	8.5435	76	76	0
17	19	35.6	35.6	33.9	92.115	814	814;858;81	0
9	9	24.9	24.9	24.9	74.267	651	651;704;49	0
13	12	75.6	75.6	75.6	22.782	205	205;186;37	0
19	19	45.2	27.2	27.2	84.659	732	732;854;17	0

17	17	56.5	56.5	31.8	83.263	724	724;597	0
1	1	16.1	1.9	1.9	70.02	639	639	0
4	4	54.8	54.8	54.8	11.012	104	104;154;18	0
2	1	27	27	27	12.23	115	115;116;16	0
1	3	15.5	15.5	15.5	36.032	316	316;370;28	0
1	1	12.9	12.9	12.9	12.649	116	116;205	0.008887
3	4	48.6	48.6	48.6	15.184	142	142;110;14	0
0	0	27.9	21.8	21.8	15.998	147	147;90	0
3	2	7.7	7.7	7.7	67.703	634	634;662;65	0
1	1	4.2	4.2	4.2	30.004	263	263;690;77	0.002015
4	5	34.2	34.2	34.2	26.788	240	240;233;25	0
4	4	22.1	22.1	22.1	28.515	253	253;515;55	0
3	4	16.4	16.4	16.4	30.302	280	280;283;32	0
5	5	23.6	23.6	23.6	30.84	305	305	0
9	7	40.7	37.8	37.8	33.155	307	307;267;32	0
7	6	28.1	28.1	28.1	37.029	356	356;378;11	0
5	7	38.6	38.6	38.6	23.811	210	210;287;30	0
3	2	10.7	10.7	10.7	40.04	363	363;244;30	0
9	7	37.3	37.3	30.8	45.671	415	415;98	0
4	4	33.6	27.3	14.9	47.087	429	429;472;44	0
2	4	27.8	13.8	13.8	49.263	449	449	0
7	4	29	29	29	35.238	331	331;346;29	0
15	16	43.2	43.2	43.2	48.562	440	440;463;46	0
6	6	40.6	40.6	27.2	64.132	589	589;530;45	0
22	22	41.5	41.5	41.5	73.62	691	691;730;73	0
10	6	35.7	35.7	26.6	58.735	527	527;562;58	0
9	9	25.6	18	18	70.942	633	633;636;59	0
22	21	34.1	34.1	34.1	90.583	825	825;804;80	0
2	3	5.6	5.6	5.6	71.611	641	641;766;76	0
11	10	23.3	23.3	23.3	85.104	747	747;746	0
15	11	56.4	56.4	53.8	37.429	353	353;341;26	0
13	11	47.7	47.7	47.7	28.916	262	262;288;29	0
3	5	13.7	12.5	12.5	98.972	889	889;917	0
9	10	19.4	19.4	18.2	101.08	905	905;916;91	0
1	2	2.9	2.9	2.9	67.638	629	629	0
4	3	37.3	37.3	29.7	18.311	158	158;215;21	0
1	0	19.4	10.4	10.4	15.404	134	134;209	0
1	1	8.5	8.5	8.5	17.522	153	153;188;19	0
3	2	30.8	30.8	30.8	11.676	107	107;179;96	0
0	1	23.2	23.2	23.2	10.767	99	99;118;147	0
4	4	35.7	35.7	35.7	13.802	126	126;78;78;[illegible]	0
0	0	10.5	10.5	10.5	20.598	181	181;440;44	0.002
1	1	3.9	3.9	3.9	39.786	334	334;419	0.001517
2	1	11.4	11.4	6.1	51.998	458	458;488	0
2	3	17	17	17	20.863	194	194;177	0
2	2	36.2	36.2	16	21.364	213	213	0
2	2	31.1	11.4	11.4	21.865	219	219;221;20	0
5	6	26.1	22.1	22.1	22.58	226	226	0
2	2	10.3	10.3	10.3	22.487	213	213	0
1	1	27.2	27.2	6.5	18.481	169	169;170;12	0

2	2	31.2	18.8	18.8	13.509	128	128;128;12	0
0	1	14.5	10.7	10.7	26.153	242	242;372	0.006667
1	1	21.7	5.6	5.6	15.144	143	143;131;14	0.002001
0	0	53.2	5.6	0	13.952	126	126;127	1
0	0	52.4	7.1	0	13.92	126	126;126;12	1
0	0	52.4	5.6	5.6	13.908	126	126	1
0	0	40.4	40.4	0	18.804	166	166;126;12	0
0	0	26.5	26.5	8.1	15.43	136	136	0
7	8	52.4	52.4	52.4	11.367	103	103	0
4	4	22	22	13.2	46.158	410	410;425;42	0
2	1	19.5	11.1	11.1	47.82	425	425;469;41	0
0	0	43	6.8	0	41.003	365	365;326;36	0
0	0	43	43	0	41.054	365	365;371;36	0
1	1	6.8	3	3	38.254	338	338;343;34	0.004852
0	0	25.7	8	0	40.539	362	362;362;24	0
0	0	30.3	16.7	0	40.885	366	366;325;33	0
0	0	26.3	7	0	41.238	372	372	0
7	7	20.9	20.9	20.9	41.331	369	369;211;36	0
6	5	26.7	26.7	26.7	44.468	378	378;230;35	0
1	1	8	8	8	29.578	264	264;281;55	0
4	3	14.8	14.8	14.8	35.236	318	318;390;31	0
1	1	8	8	8	34.36	325	325	0
7	8	18.5	18.5	18.5	57.293	520	520;95	0
4	3	20.6	20.6	20.6	37.32	345	345;418	0
5	5	28	28	28	24.579	218	218	0
6	6	13.6	13.6	13.6	104.78	937	937;999;65	0
1	1	29	29	29	7.6079	69	69;82	0
8	8	24.6	24.6	24.6	57.861	529	529;134;14	0
4	3	22.1	22.1	19.4	57.886	521	521;170	0
1	1	4.6	1.9	1.9	57.81	521	521	0.001548
1	1	4.6	4.6	4.6	60.221	538	538;169;17	0
13	12	23.1	23.1	23.1	97.169	876	876;690;73	0
8	9	14.6	14.6	14.6	123.75	1099	1099;1037;	0
3	4	6.5	6.5	6.5	119.52	1038	1038	0
1	0	1.9	1.9	1.9	115.96	1041	1041	0
2	1	2.2	2.2	2.2	103.58	940	940;1014;1	0
1	1	6.9	3.4	3.4	39.309	350	350;244	0
0	1	6.6	6.6	3.4	43.083	377	377	0
1	1	3.1	3.1	3.1	104.99	914	914;918	0
1	0	4.5	4.5	4.5	99.996	911	911	0
9	8	47.4	47.4	47.4	32.66	289	289;178	0
2	2	18.6	18.6	18.6	19.603	177	177;194;15	0
11	10	31.9	31.9	30.2	50.957	470	470;514;27	0
2	3	3.8	3.8	3.8	304.1	2671	2671;721;2	0
4	4	10	10	10	63.704	579	579;198;60	0
2	3	18.8	18.8	18.8	30.338	266	266;160	0
1	0	2.6	2.6	2.6	38.463	342	342;502;50	0.002488
13	10	18.8	18.8	18.8	129.29	1181	1181;942;8	0
4	3	5	5	5	116.61	1051	1051;1066;	0
16	15	30.4	30.4	30.4	119.09	1068	1068;1073;	0

2	3	8.2	8.2	8.2	111.13	1002	1002;1012;	0
2	2	3.5	3.5	3.5	75.849	693	693;746;78	0.00054
12	11	20.8	20.8	20.8	88.414	798	798;789;80	0
32	31	28.1	28.1	28.1	194.46	1745	1745;1752;	0
2	2	5.3	5.3	5.3	57.825	532	532;310	0
1	2	8.8	8.8	8.8	54.404	489	489;161;21	0
2	3	10.5	10.5	10.5	31.356	276	276;467;37	0
1	1	21.6	21.6	21.6	8.0866	74	74;112;112	0
4	5	10.3	10.3	10.3	62.094	551	551;510;10	0
6	8	32.7	32.7	32.7	38.91	352	352;390;11	0
18	18	32.3	32.3	32.3	95.337	894	894;898;69	0
0	1	0.9	0.9	0.9	134.62	1240	1240;1249	0.001521
1	1	5.9	5.9	5.9	20.239	188	188;298	0.00152
5	5	27.2	27.2	27.2	35.786	331	331;366;13	0
2	3	16.5	16.5	16.5	42.794	393	393;199;28	0
12	11	37.4	37.4	37.4	46.659	414	414;157;62	0
9	9	12.3	12.3	12.3	144.5	1262	1262;1152;	0
6	6	8.7	8.7	8.7	113.79	1012	1012	0
1	1	25.8	25.8	25.8	13.651	120	120;122;22	0
18	17	37.2	37.2	34.1	81.744	745	745;235;27	0
7	6	35.8	35.8	35.8	32.583	299	299;279;25	0
1	1	9.4	9.4	9.4	13.999	128	128;341;46	0
3	2	12.1	8.9	8.9	59.51	593	593;584;45	0
9	9	60.3	56.8	26.4	49.586	458	458;458;41	0
5	3	31.6	16.9	12.7	49.167	456	456;456;29	0
18	18	57.2	55.1	45.8	48.057	430	430;391;29	0
21	15	80.2	80.2	60	44.091	400	400;400;18	0
3	2	14	12.8	12.8	62.064	623	623;623;39	0
1	0	12.7	7.8	4.5	66.017	644	644;644	0
1	1	10.9	2.2	2.2	65.865	645	645;639;70	0.001528
5	5	48.3	44.2	10.5	62.378	590	590;590;17	0
2	2	42.7	25.7	4.8	60.044	564	564;564;56	0
0	0	36.3	1.8	0	60.066	564	564	0.004805
2	3	64.8	64.8	6.4	51.385	469	469;469	0
23	23	62.1	57.6	51.8	53.704	483	483;483;51	0
3	3	9.1	9.1	9.1	45.86	418	418;443;40	0
2	4	5.2	5.2	5.2	149.61	1300	1300;1306;	0
6	6	8.3	8.3	8.3	109.68	963	963;860;94	0
0	0	1.1	1.1	1.1	186.49	1648	1648	0.001518
4	4	7.7	7.7	7.7	109.13	952	952;856;96	0
0	0	3.7	3.7	3.7	36.788	324	324;381	0.00201
2	1	32.7	32.7	26.7	22.828	202	202;195;25	0
1	0	2.4	2.4	2.4	52.351	465	465	0.003893
2	2	16	16	16	19.043	169	169;184	0
6	4	20.3	20.3	20.3	58.677	531	531;517;18	0
2	2	23.6	9.7	9.7	75.491	694	694;248	0
5	6	38.8	38.8	17.4	50.67	454	454;414;34	0
4	2	7.5	7.5	7.5	70.702	615	615;213;51	0
25	22	53.6	52.2	50.9	66.408	586	586;387;32	0
20	16	46.1	43.5	43.5	69.948	620	620	0

0	1	1.9	1.9	1.9	128.17	1147	1147;1668;	0
0	0	1.4	1.4	1.4	399.73	3695	3695;561	0
5	3	7.9	7.9	7.9	129.57	1172	1172;188	0
3	2	5.2	5.2	5.2	177.6	1609	1609	0
5	4	9.8	9.8	9.8	121.6	1111	1111;1193	0
1	1	14.6	14.6	14.6	9.2824	82	82;196;399	0.001569
6	5	14.1	14.1	14.1	57.278	509	509;503;40	0
0	2	5.4	5.4	5.4	82.198	721	721;732;26	0
3	5	9.9	9.9	9.9	55.01	507	507	0
1	3	3.7	3.7	3.7	113.46	1077	1077;1126;	0
1	2	14.5	14.5	14.5	26.076	234	234	0
1	1	11.9	11.9	11.9	23.52	201	201;503;53	0
3	4	11.1	11.1	11.1	83.353	739	739	0
26	27	29.1	29.1	29.1	157.9	1394	1394;504;5	0
4	3	8.4	8.4	8.4	82.688	752	752;784;80	0
1	1	1.1	1.1	1.1	92.005	835	835;883;90	0.008895
0	3	8.7	8.7	8.7	63.472	583	583	0
7	8	32.2	32.2	32.2	34.93	307	307;62	0
4	5	6.1	6.1	6.1	128.04	1122	1122;1176;	0
0	0	4.4	4.4	4.4	115.06	1006	1006;1011;	0
4	4	13.2	13.2	13.2	42.741	379	379;228	0
2	3	9.6	9.6	9.6	31.742	292	292;305;31	0
6	5	12.6	12.6	12.6	69.284	611	611;532;58	0
6	7	41	41	41	29.717	261	261;323;16	0
12	9	25.6	25.6	25.6	66.99	598	598;599;60	0
0	1	6.7	6.7	6.7	66.175	601	601;493;58	0
12	12	42.2	42.2	38.6	36.688	332	332;361;27	0
7	8	32.9	29.3	29.3	36.638	334	334;232;13	0
2	3	11	11	11	93.296	829	829;845;76	0
3	2	9.5	9.5	9.5	72.063	643	643;237	0
8	9	24.4	24.4	20.4	80.419	720	720;141	0
2	2	10.1	5.8	5.8	74.435	670	670;711;15	0
3	3	20.3	20.3	20.3	18.042	158	158;70;80;:	0
2	3	6.8	6.8	6.8	104.56	945	945;682;69	0
0	0	2.9	2.9	2.9	76.193	713	713	0
3	2	8.8	8.8	8.8	58.22	489	489;432;18	0
4	3	10.8	10.8	10.8	46.836	408	408;120;18	0
1	1	20	20	20	8.2474	70	70;74;96;1:	0.004822
8	8	14.9	14.9	14.9	68.047	597	597;625;10	0
3	3	8.8	8.8	8.8	44.561	376	376;414;15	0
0	1	9.4	9.4	9.4	25.74	234	234;489;53	0
0	0	5.3	5.3	5.3	60.207	544	544;274;18	0
1	0	2.5	2.5	2.5	46.765	407	407;487	0.004803
4	3	13.3	13.3	13.3	44.732	399	399;472;34	0
0	0	9.4	9.4	9.4	12.06	106	106;141;14	0.00485
4	4	8.6	8.6	8.6	44.882	417	417;364	0
2	2	4.1	4.1	4.1	44.955	410	410;410;41	0
2	1	17.9	17.9	17.9	22.279	207	207;774	0
2	2	17.4	17.4	17.4	12.476	115	115	0
4	4	14.1	14.1	14.1	36.856	333	333;348;10	0

2	2	9	9	9	41.531	367	367;335;13	0
0	1	9.8	9.8	9.8	65.171	599	599	0
0	1	2.5	2.5	2.5	38.247	356	356;366;48	0.004787
1	1	5.2	5.2	5.2	28.973	249	249;296;34	0.001555
2	2	8	8	8	27.278	249	249;246;25	0
8	11	16	16	16	99.326	893	893;94;107	0
5	6	39	39	39	23.038	210	210;245;33	0
16	17	65.7	65.7	65.7	35.503	338	338;296;23	0
3	1	19.2	19.2	19.2	32.233	292	292;146;17	0
1	1	11.2	11.2	11.2	19.228	169	169;181;18	0.000536
0	1	3.3	3.3	3.3	39.834	360	360;387	0.004843
1	1	38.5	38.5	38.5	5.7856	52	52;61;75;1	0
1	3	10.7	10.7	10.7	80.702	731	731;837;49	0
1	0	6.9	6.9	6.9	24.269	245	245	0
1	2	14.4	14.4	14.4	19.529	195	195	0
15	15	25	25	25	94.622	847	847;895;84	0
2	4	8.4	8.4	8.4	65.893	582	582;194	0
4	3	11	11	11	197.6	1825	1825;2089;	0
0	0	6.3	6.3	6.3	12.853	111	111;134;13	1
1	1	6.5	6.5	6.5	42.426	385	385;454;42	0
0	0	4.9	4.9	4.9	80.214	738	738	0
1	1	18.3	18.3	18.3	14.686	131	131;196	0
3	3	26.2	21.5	21.5	21.671	195	195;143	0
3	3	16.6	16.6	12.6	23.818	223	223;247;91	0
0	0	18.4	18.4	18.4	16.389	147	147;169;21	0.00572
4	3	26.5	26.5	26.5	21.143	185	185;182;71	0
2	2	5	5	5	50.628	457	457;456;48	0
2	1	32.8	32.8	32.8	6.0683	61	61;61;61;6	0
2	2	7.8	7.8	7.8	75.022	668	668;495	0
3	2	15.5	15.5	15.5	35.777	317	317;466;43	0
2	1	13.1	13.1	13.1	25.717	229	229;253;26	0
1	1	6.6	6.6	6.6	22.063	198	198;259;30	0.001998
3	2	11.1	11.1	11.1	43.215	386	386;38;170	0
2	2	11.3	11.3	11.3	35.991	328	328;455;47	0
5	6	9.7	9.7	9.7	101.11	900	900;546;58	0
1	1	5.8	5.8	5.8	24.618	241	241;302;41	0
2	1	9.7	9.7	9.7	57.518	525	525;563;28	0
1	1	3	3	3	48.216	434	434;575;59	0
1	1	5.4	5.4	5.4	29.651	278	278;342	0.002979
0	0	18.6	18.6	18.6	18.259	167	167;203;23	0.001568
2	2	11.3	11.3	11.3	37.171	344	344;346;39	0.002954
0	1	24.2	24.2	24.2	7.036	62	62;24;78;4	0.002963
4	2	28.9	28.9	28.9	26.753	232	232;227;24	0
0	1	17.1	17.1	17.1	15.303	140	140;256;23	0.003891
10	10	27.3	27.3	27.3	82.624	747	747;758;71	0
1	1	18.2	18.2	18.2	8.3587	77	77;147	0
1	1	18.1	18.1	18.1	18.416	166	166;152;12	0
1	0	0.6	0.6	0.6	199.08	1823	1823;1827	1
6	7	48.9	48.9	48.9	29.999	268	268	0
3	3	9.3	9.3	9.3	48.646	463	463;493;76	0

2	0	23.7	23.7	23.7	14.374	131	131;156;14	0
1	1	3	3	3	72.748	640	640;642	0.005723
3	3	13.4	13.4	13.4	36.813	335	335;377;27	0
1	1	2	2	2	143.64	1275	1275;1295;	0
5	5	21.3	21.3	21.3	32.182	296	296;263;31	0
3	1	12.5	12.5	12.5	40.467	399	399;540	0
2	3	10.2	10.2	10.2	43.121	394	394;372;38	0
4	5	29	29	29	33.331	303	303;262	0
1	0	15.7	3.5	3.5	31.282	287	287;296;40	0.00053
2	2	16.4	16.4	16.4	16.937	152	152	0
1	1	7.3	7.3	7.3	33.281	313	313;323;97	0
1	1	26.3	26.3	26.3	10.5	95	95	0
4	4	16.2	16.2	16.2	51.355	452	452;273;13	0
1	1	7	7	7	25.028	227	227;353;45	0
2	2	22.7	22.7	22.7	10.998	97	97	0
1	1	25	25	25	5.1438	44	44;60;61;6	0
5	5	65.5	65.5	65.5	15.521	142	142	0
2	3	17.2	17.2	17.2	34.559	309	309	0
7	7	39.6	39.6	39.6	37.893	361	361;329;21	0
6	6	17.9	17.9	17.9	67.454	608	608	0
1	1	10.7	10.7	10.7	12.728	112	112;408;43	0
0	1	1.3	1.3	1.3	80.64	713	713	0.008962
3	3	16	16	16	36.828	312	312;403	0
2	2	11.6	11.6	11.6	58.252	525	525;394;27	0
1	3	10.8	9.4	9.4	54.366	489	489;490;11	0
2	2	8.5	8.5	8.5	36.432	316	316;360	0
5	5	20.9	20.9	20.9	31.703	278	278;326;32	0
2	1	4.6	4.6	4.6	75.356	679	679	0
3	3	14.3	14.3	14.3	34.222	293	293;102;16	0
14	14	38.1	38.1	30.2	67.819	577	577;103	0
2	2	4.2	4.2	4.2	53.944	500	500	0
1	2	4.6	4.6	4.6	99.31	913	913;979;97	0
1	2	11.3	11.3	11.3	23.844	213	213;431;32	0
4	4	33.6	33.6	33.6	24.061	217	217;437;36	0
2	2	16.3	16.3	16.3	27.56	239	239	0
0	0	3.2	3.2	3.2	53.004	473	473;1394;1	0.002976
1	0	17.3	17.3	17.3	9.2555	81	81;106;120	0
7	5	24.9	24.9	24.9	45.651	413	413;425;43	0
2	1	8.6	8.6	8.6	37.898	348	348;400;38	0.001562
12	13	16.7	16.7	16.7	148.85	1328	1328;1332;	0
1	1	7.8	7.8	7.8	33.932	306	306;149;20	0
2	3	33.8	33.8	33.8	15.947	145	145;268;26	0
0	1	6	6	6	24.484	215	215	0
2	2	22.6	22.6	22.6	15.864	146	146;188;24	0
46	44	29.7	29.7	29.7	233.47	2048	2048;2061;	0
0	1	8	8	8	16.684	150	150;194;19	0.002934
6	5	62.8	62.8	46.2	16.29	145	145;161;15	0
0	1	3	3	3	95.928	844	844;1794;1	0
8	8	60.5	60.5	60.5	20.457	177	177;171;17	0
1	1	6.7	0.4	0.4	229	1976	1976;1985;	0.002969

0	0	6	0.5	0.5	223.57	1938	1938;1945;	0.004838
1	1	4.7	1.3	1.3	227.87	1995	1995;2003;	0
72	74	52.1	52.1	44	226.53	1960	1960;1382;	0
2	2	51.9	51.9	51.9	5.7044	52	52;118	0
7	7	38.6	38.6	38.6	31.554	332	332	0
2	2	7.8	7.8	7.8	38.868	358	358;215;20	0.002007
1	1	4.6	4.6	4.6	75.388	657	657;199;38	0
11	8	15.2	15.2	15.2	115.73	1025	1025;953;8	0
3	2	5.4	5.4	5.4	96.352	846	846;879;88	0
1	1	4.4	4.4	4.4	113.89	1086	1086;199;2	0
4	4	23.3	23.3	23.3	22.793	202	202;269;23	0
3	3	8.8	8.8	8.8	53.662	480	480;530;53	0
1	1	6	6	6	20.35	184	184	0
0	0	12.9	12.9	12.9	7.8423	70	70;93;101;	0.008904
1	0	14.7	14.7	14.7	15.733	136	136;377	0.00156
1	2	13.7	13.7	13.7	19.258	161	161;172;14	0
0	1	13.1	13.1	13.1	27.907	252	252;249	0
0	2	4.4	4.4	4.4	51.851	457	457;463	0
0	0	4.9	4.9	4.9	30.241	264	264	0
1	1	4.9	4.9	4.9	24.793	226	226;305	0.004854
4	5	27.7	27.7	27.7	31.628	278	278;301;33	0
1	0	2.2	2.2	2.2	55.165	497	497;572	0.009337
1	1	7.1	7.1	7.1	48.027	439	439;463;48	0
2	3	8.3	8.3	8.3	76.689	677	677;418;72	0
2	2	16.7	16.7	16.7	19.311	168	168;169;48	0
2	2	20.6	20.6	20.6	15.016	136	136;198;21	0
0	1	1.2	1.2	1.2	128.79	1128	1128;1134	0.008912
0	0	8.1	8.1	8.1	24.899	221	221	0.004833
2	1	22.6	22.6	22.6	15.727	146	146;222;24	0
3	2	15.8	15.8	15.8	20.9	183	183;69;114	0
1	0	15.1	15.1	15.1	18.856	172	172	0
113	120	46.4	46.4	46.4	629.09	5890	5890;121;1	0
1	1	2.7	2.7	2.7	46.91	414	414;600;70	0.000538
2	2	14.9	14.9	14.9	31.291	282	282;398;18	0
1	1	29.1	29.1	2.2	106.87	944	944;847	0
1	1	28.3	2	2	109.44	966	966;852;11	0
1	1	7.1	7.1	7.1	55.722	532	532;234	0
6	6	21.8	21.8	21.8	56.598	541	541;365;31	0
4	3	11.8	11.8	11.8	49.062	440	440;448;40	0
4	4	32	32	32	22.788	200	200;198;19	0
1	0	2.4	2.4	2.4	92.948	837	837;937;96	0
3	3	25.8	25.8	25.8	14.627	132	132;128	0
3	3	4.6	4.6	4.6	82.682	733	733;746	0
0	1	8.7	8.7	8.7	62.846	562	562;563;32	0
6	7	21.8	21.8	21.8	55.52	491	491;368;60	0
1	1	2.3	2.3	2.3	56.099	525	525;538	0.000539
0	0	1.2	1.2	1.2	108.06	971	971;960;12	0
6	5	5.5	5.5	5.5	139.38	1267	1267;1267;	0
1	2	38.9	38.9	38.9	9.3926	90	90;130;77;	0
11	12	33.8	32.1	32.1	54.231	471	471;382;23	0

4	4	29.3	29.3	29.3	15.079	140	140;143;46	0
1	1	9.7	9.7	9.7	61.386	548	548;543;48	0
6	6	24.3	24.3	24.3	40.572	370	370;372;27	0
1	0	10.3	10.3	10.3	15.896	145	145;223	0
3	3	21.1	21.1	21.1	18.76	175	175;422;44	0
3	4	7	7	7	91.838	790	790	0
1	1	20.4	20.4	20.4	6.199	54	54;103;156	0
1	1	11.6	11.6	11.6	21.87	198	198;968;96	0
3	3	6.5	6.5	6.5	76.12	695	695	0
4	3	16.9	16.9	16.9	38.242	331	331;133;18	0
28	35	29.7	29.7	29.7	236.51	2101	2101;2099;	0
2	3	11	11	11	106.37	925	925;686;89	0
4	3	8.4	8.4	8.4	128.98	1156	1156	0
2	3	5.9	5.9	5.9	149.39	1433	1433;1475;	0
10	10	15.6	15.6	15.6	148.09	1327	1327;1391;	0
4	3	6.2	6.2	6.2	148.86	1314	1314;1436;	0
5	7	6	6	6	227.92	2012	2012;273	0
4	3	5.7	5.7	5.7	152.57	1519	1519;2079;	0
1	1	3.4	3.4	3.4	46.862	440	440;468	0
0	2	4.4	4.4	4.4	62.101	544	544;462;61	0
4	4	12.4	12.4	12.4	78.77	696	696;741;11	0
7	7	13.4	13.4	13.4	99.554	880	880;819;69	0
3	2	4.2	4.2	4.2	186.27	1714	1714;1800;	0
2	2	9.2	9.2	9.2	53.254	522	522;446	0
0	0	3.3	3.3	3.3	205.11	1887	1887;967	0
1	1	1.6	1.6	1.6	68.119	608	608;617	0.003885
2	3	15.3	15.3	15.3	69.725	614	614;630;65	0
5	3	56.7	56.7	56.7	14.478	127	127;107	0
3	3	11.9	11.9	11.9	27.296	243	243;203	0
0	1	2.3	2.3	2.3	72.732	659	659;667;75	0.001539
5	5	42.6	42.6	31.5	35.924	324	324;374;21	0
1	1	8.2	8.2	8.2	53.879	461	461;267	0
2	3	7.8	7.8	7.8	74.543	708	708;699;70	0
1	1	5.6	5.6	5.6	84.918	749	749	0
0	0	1.9	1.9	1.9	92.547	800	800	0.002959
4	9	15	15	15	73.964	634	634;587;51	0
0	0	0.7	0.7	0.7	254.39	2271	2271	0.004794
2	2	3.9	3.9	3.9	81.123	719	719;189;27	0
1	1	1.8	1.8	1.8	88.865	788	788;806;85	0
0	0	14.7	14.7	14.7	10.701	95	95;150;231	0.001552
10	8	19.4	19.4	19.4	66.049	594	594;219;25	0
10	10	23.8	23.8	23.8	59.578	529	529;187;68	0
1	1	2.4	2.4	2.4	127.32	1143	1143;1146;	0
14	16	30.8	30.8	28.9	87.343	783	783;715;73	0
1	2	5.2	5.2	5.2	87.435	745	745;727;76	0
23	20	30.7	30.7	30.7	76.613	710	710;296;14	0
1	0	11.4	11.4	11.4	13.052	114	114;285;38	0
11	11	39.5	39.5	39.5	32.575	294	294;259;26	0
0	0	5.3	5.3	5.3	23.266	208	208;326	0.001554
3	1	10.5	10.5	10.5	42.15	380	380;284	0

5	3	24.3	24.3	24.3	33.12	309	309;326;11	0
2	1	14.1	14.1	14.1	33.179	291	291;459;50	0
3	4	24.4	24.4	24.4	39.648	360	360;421;26	0
9	12	9.1	9.1	9.1	267.29	2363	2363;726	0
1	1	20	20	20	12.832	120	120;435	0
1	0	40.8	9.2	9.2	17.149	152	152;177;82	0
4	2	45.9	45.9	16.4	32.642	292	292;267;15	0
3	3	23.2	23.2	23.2	23.417	198	198;207;20	0
4	3	23.7	23.7	23.7	31.839	278	278;375;38	0
2	1	4.4	4.4	4.4	56.613	496	496;554;58	0
1	1	8.3	8.3	8.3	14.745	132	132;157	0.002018
1	1	9.5	9.5	9.5	12.302	116	116;416;27	0
2	2	17.5	17.5	17.5	16.953	154	154;61;88;	0
1	1	14.5	14.5	14.5	9.369	83	83;149;171	0
6	7	37.3	37.3	37.3	30.608	276	276;265;92	0
1	1	5.3	5.3	5.3	39.503	342	342;979	0
0	1	13.9	13.9	13.9	12.157	108	108;109;15	0
0	0	11.6	11.6	11.6	14.788	129	129;137;15	0.001057
9	9	26.7	26.7	26.7	48.534	439	439;301	0
0	1	11.7	11.7	11.7	23.787	214	214	0.002982
5	4	13	13	13	50.151	454	454;70;287	0
0	0	2.3	2.3	2.3	43.963	392	392	0.00979
2	3	7.4	7.4	7.4	48.55	432	432;249;19	0
1	1	17.2	17.2	17.2	10.016	87	87;208;305	0.001055
1	1	5.8	5.8	5.8	33.743	292	292;365	0.007612
3	3	10.7	10.7	10.7	45.57	393	393;523;24	0
9	8	48	48	48	36.071	329	329	0
1	1	7.5	7.5	7.5	36.049	333	333;40;128	0
1	1	4.6	4.6	4.6	78.549	689	689;280	0
1	1	78.1	8.6	8.6	11.418	105	105	0.008979
3	4	70.9	70.9	26.7	18.012	165	165;120;12	0
9	9	44.9	44.9	44.9	23.742	216	216	0
1	1	6.1	6.1	6.1	22.763	212	212	0
0	1	25.1	21.7	21.7	22.04	207	207;145;15	0
3	3	40.7	40.7	40.7	11.951	108	108;103;12	0
2	2	18.3	18.3	18.3	15.649	142	142;102	0
3	3	14.7	14.7	14.7	25.177	224	224	0
11	9	32.2	32.2	32.2	51.804	459	459;84;86;	0
1	1	6.2	6.2	6.2	27.527	257	257;412;41	0
1	1	18.7	18.7	18.7	18.237	166	166	0.001577
0	1	2.8	2.8	2.8	57.224	492	492	0.001528
2	2	23.3	23.3	23.3	19.325	180	180;179	0
0	1	4.4	4.4	4.4	46.348	408	408;530;74	0.004829
7	5	30.6	30.6	30.6	45.803	422	422;433;43	0
2	1	6.5	6.5	6.5	102.45	919	919;919;18	0
10	9	72.9	72.9	59.8	22.11	199	199;171;97	0
7	6	53.5	48	48	21.892	198	198;136;14	0
7	7	50.9	45.4	45.4	30.54	271	271;161;18	0
6	6	48.1	48.1	48.1	17.031	162	162;214;17	0
9	8	40.2	40.2	40.2	25.035	224	224	0

1	0	28.2	28.2	28.2	7.8376	71	71;156;223	0
0	0	2.5	2.5	2.5	44.129	403	403	0.001543
0	1	6.3	6.3	6.3	36.701	334	334;377	0.008899
12	13	33.8	33.8	33.8	79.685	736	736;712;71	0
0	1	4.6	4.6	4.6	28.444	260	260;254	0.002014
3	2	20	20	20	66.077	571	571;588;44	0
0	0	38.2	38.2	38.2	3.692	34	34;70;434;!	0.008061
2	2	26.4	26.4	26.4	12.405	110	110	0
2	3	6.2	6.2	6.2	62.395	548	548;508;23	0
9	9	17.1	17.1	17.1	66.115	589	589;490	0
1	2	1.4	1.4	1.4	206.69	1821	1821	0
8	7	22.4	22.4	22.4	39.958	361	361;362;32	0
0	1	4.3	4.3	4.3	51.417	445	445	0
6	6	54.5	54.5	54.5	21.057	187	187	0
0	1	14.3	14.3	14.3	13.552	112	112;287;48	0
3	3	19.6	19.6	19.6	31.581	271	271;271;27	0
4	4	12.5	12.5	12.5	59.918	551	551;610;63	0
1	1	4	4	4	34.579	301	301;327;47	0.001055
3	2	10	10	10	70.698	640	640;441;50	0
7	6	18.3	18.3	18.3	61.448	562	562;580;36	0
1	1	8.2	8.2	8.2	32.724	291	291;612	0.00481
14	13	49.9	49.9	49.9	41.428	389	389;417;41	0
8	11	49.2	49.2	49.2	28.804	254	254;253;25	0
0	1	6.9	6.9	6.9	54.705	490	490;71	0
1	1	7.4	7.4	7.4	34.217	311	311;237;31	0
1	1	8	8	8	19.878	176	176;194;24	0.001544
8	7	26.5	26.5	26.5	40.422	370	370;324	0
1	1	7	7	7	20.745	187	187;225	0
3	2	6.1	6.1	6.1	81.627	717	717	0
0	1	3.1	3.1	3.1	32.113	290	290	0.002953
2	2	5.2	5.2	5.2	80.496	726	726;747	0
2	1	3.7	3.7	3.7	92.755	837	837;881;36	0
7	6	16	16	16	88.654	812	812;797;24	0
2	1	3.8	3.8	3.8	129.15	1171	1171;1176;	0
9	11	37.4	37.4	37.4	43.135	393	393;408;38	0
6	5	18.8	18.8	18.8	46.596	415	415;253;28	0
11	13	36.7	36.7	28.2	70.288	627	627;246;19	0
9	8	28.7	20.1	20.1	69.334	617	617;585;61	0
5	5	16.6	16.6	16.6	46.637	410	410;208;82	0
1	1	14.4	14.4	14.4	14.888	132	132;155;20	0.001997
1	1	3.5	3.5	3.5	29.652	260	260;401	0.002019
75	76	27.8	27.8	27.8	516.19	4547	4547;4515;	0
2	2	7.5	7.5	7.5	56.289	505	505;514;15	0
0	0	0.8	0.8	0.8	211.1	1894	1894	0.001536
5	5	4.6	4.6	4.6	205.12	1838	1838;234;1	0
15	14	20.9	20.9	20.9	113.08	1014	1014;108;1	0
0	0	2.1	2.1	2.1	66.171	582	582;712	0
6	6	45.2	45.2	36.5	37.497	356	356;64	0
6	5	30.7	22.2	22.2	38.15	361	361;362;31	0
7	6	22.2	22.2	22.2	54.626	505	505;534;49	0

13	12	33.8	33.8	25.6	70.67	636	636;522;59	0
2	2	9.1	9.1	9.1	31.496	296	296;306;96	0
3	2	13.7	5.2	5.2	67.97	615	615;631;64	0
1	2	6.6	6.6	6.6	39.908	351	351;367;40	0
1	1	3.4	3.4	3.4	39.871	350	350;368	0
2	2	6.2	6.2	6.2	48.103	438	438;392;42	0
7	7	26.4	26.4	26.4	43.476	390	390;300;19	0
6	5	19.6	19.6	19.6	64.732	571	571;265;57	0
1	2	12.4	12.4	12.4	42.533	371	371;633;46	0
8	8	28.8	28.8	25.6	56.51	527	527;588;53	0
5	4	23.9	20.1	20.1	49.539	457	457;521;52	0
1	2	4.5	4.5	4.5	85.95	783	783;179;71	0
0	0	6.9	6.9	6.9	17.188	160	160;178	0.003902
1	1	9.4	9.4	9.4	13.466	117	117;122	0
1	0	9.1	9.1	9.1	16.648	154	154	0
1	0	8.8	8.8	8.8	15.598	136	136;134	0
33	35	60.2	60.2	59	74.139	664	664;634;61	0
8	10	29.6	29.6	29.6	55.18	504	504;252;10	0
2	2	2	2	2	105.93	930	930;939;95	0
5	6	10.7	10.7	10.7	106.92	941	941;901	0
12	12	9.3	9.3	9.3	273.6	2335	2335;1012;	0
0	1	3.8	3.8	3.8	37.476	312	312	0
6	4	8.7	8.7	8.7	90.932	795	795	0
1	1	4.9	4.9	4.9	32.992	285	285;307;33	0.001533
1	1	6.3	6.3	6.3	48.506	428	428;505;15	0
5	4	14.6	14.6	14.6	96.557	847	847	0
4	4	7.7	7.7	7.7	91.809	804	804;368	0
4	4	10	10	10	106.06	939	939;1037;1	0
1	1	3.8	3.8	3.8	36.567	340	340	0
11	9	20.8	20.8	20.8	89.301	812	812;845;80	0
1	1	7	7	7	19.492	171	171;191;22	0.002011
10	11	33.1	33.1	22.6	80.253	729	729;731;72	0
7	5	10.5	10.5	10.5	86.604	765	765;796;27	0
0	0	5.5	5.5	5.5	83.515	746	746;585;77	0.000535
6	5	8.2	8.2	8.2	117.46	1032	1032;1031;	0
2	2	7.6	7.6	7.6	45.169	406	406;455	0
9	8	30.3	17.9	17.9	69.086	614	614;535;61	0
1	1	7.6	7.6	7.6	20.342	185	185;375;39	0.009346
2	2	7.2	7.2	7.2	54.416	483	483;187	0
0	0	4.6	4.6	4.6	62.223	564	564;119;15	0
0	1	3.3	2.4	2.4	122.86	1058	1058;1070;	0
0	0	2.8	2.8	2.8	88.907	797	797	0.001531
1	1	18.2	18.2	18.2	8.1336	66	66;163	0.004785
0	1	3	3	3	107.33	960	960	0
4	4	22.7	22.7	22.7	40.684	361	361;306	0
5	5	14	14	14	71.635	622	622;218;13	0
1	2	7.6	7.6	7.6	83.549	727	727;774	0
2	2	3.7	3.7	3.7	81.169	703	703;737;75	0
6	5	13	13	13	84.784	738	738;270;26	0
9	9	70	70	70	15.054	140	140;104;14	0

5	6	10.4	10.4	10.4	96.022	868	868;873;27	0
0	1	4.6	4.6	4.6	50.576	458	458;469	0
2	2	19	19	19	14.45	121	121;189;19	0.000534
12	11	51.1	51.1	51.1	29.804	272	272;246;20	0
15	13	57.2	57.2	57.2	33.296	299	299;298;26	0
6	7	38.3	38.3	38.3	28.768	261	261	0
14	14	49.5	49.5	49.5	43.786	394	394;340;28	0
3	3	14.3	14.3	14.3	58.957	516	516;534;53	0
0	2	7.1	7.1	7.1	60.632	533	533;535	0
3	2	5.9	5.9	5.9	80.699	710	710	0
6	6	17.8	17.8	17.8	58.44	527	527;524;52	0
1	2	11.8	11.8	11.8	17.102	152	152	0
3	3	19.2	19.2	19.2	29.13	265	265;377;18	0
4	2	38.4	38.4	38.4	19.155	164	164;139;16	0
2	1	2.7	2.7	2.7	98.555	879	879	0.000534
6	4	28.9	28.9	28.9	32.895	301	301;329;11	0
1	2	8.8	8.8	8.8	42.153	407	407;368	0
5	4	23.2	23.2	23.2	26.87	233	233;249;25	0
1	1	7.9	7.9	7.9	26.011	228	228;254;23	0.000529
2	3	22.9	22.9	22.9	20.593	179	179;231;25	0
1	1	11.2	11.2	11.2	11.487	107	107;127;26	0.001547
2	1	17.1	17.1	17.1	18.607	164	164;234;23	0
5	5	35	35	35	24.526	220	220;230;23	0
7	6	41.9	41.9	41.9	22.842	203	203;252;24	0
10	10	42.6	42.6	42.6	29.555	263	263;269;23	0
2	3	12.5	12.5	12.5	27.647	248	248;255;36	0
8	7	43.6	43.6	43.6	26.411	241	241;183	0
9	8	51.6	51.6	51.6	27.887	248	248;178;18	0
3	1	12.7	12.7	12.7	30.354	276	276;272;27	0
1	1	7.6	7.6	7.6	13.919	131	131;131;19	0.00053
5	4	30.7	30.7	30.7	26.489	241	241	0
2	2	9	9	9	22.836	201	201;84	0
1	2	21.5	21.5	21.5	12.082	107	107;112;14	0
1	1	15.9	15.9	15.9	29.204	264	264	0
7	6	28.9	28.9	28.9	28.48	263	263;160;11	0
3	3	13	13	13	25.357	239	239;102;21	0
2	1	6.9	6.9	6.9	29.965	277	277;210;14	0.000541
1	2	1.4	1.4	1.4	223.69	2017	2017;1845;	0
2	1	11.4	11.4	11.4	63.132	560	560;55;154	0
5	6	31.7	31.7	31.7	40.548	353	353;325;34	0
2	1	10.4	10.4	10.4	32.311	279	279;576;59	0
1	1	1.2	1.2	1.2	93.532	824	824;855	0.003897
2	1	13.9	13.9	13.9	17	144	144;145;10	0
3	4	23.2	19.8	19.8	29.224	263	263;168;18	0
1	2	19.5	19.5	19.5	13.171	118	118;248	0
3	3	48.8	48.8	48.8	22.079	213	213;258;10	0
1	1	14.6	14.6	14.6	15.771	137	137;139	0
1	1	10.4	10.4	10.4	14.401	135	135;156;17	0
6	5	38.1	38.1	38.1	19.891	189	189;169;16	0
5	5	15.5	15.5	15.5	42.674	375	375;347;34	0

5	5	9.6	9.6	9.6	140.3	1263	1263;1272;	0
17	17	38.4	38.4	38.4	57.116	508	508;464;27	0
15	15	46.7	46.7	37.6	56.782	505	505	0
11	8	24.7	24.7	24.7	72.932	645	645	0
9	8	31.4	31.4	31.4	47.837	437	437;440;44	0
0	0	12.5	12.5	12.5	14.364	128	128;197;19	0
1	1	2.9	2.9	2.9	252.5	2266	2266;2275;	0
7	4	19.8	19.8	19.8	57.548	510	510	0
0	0	12.3	12.3	12.3	21.491	203	203;543	0
1	1	2.8	2.8	2.8	125.56	1141	1141;1213;	0
2	3	21.5	21.5	21.5	16.515	144	144;154;14	0
1	1	11	11	11	9.404	82	82;94;107;	0.008967
4	4	31.7	31.7	31.7	24.68	227	227;109;15	0
3	3	13	13	13	36.748	324	324;178;63	0
15	14	29	29	29	127.12	1179	1179;906	0
1	1	4.2	4.2	4.2	55.272	518	518;519	0
3	3	11.8	11.8	11.8	37.19	330	330;433	0
1	1	2.1	2.1	2.1	138.49	1214	1214;1258;	0
0	0	1.9	1.9	1.9	63.984	571	571;552;60	0
1	0	6.6	6.6	6.6	18.874	167	167;177;19	0.000541
2	2	2.8	2.8	2.8	116.58	1059	1059;1218;	0
2	2	6	6	6	51.828	451	451;445;48	0
2	2	6.5	6.5	6.5	31.088	293	293;261	0
0	0	2.3	2.3	2.3	68.262	596	596	0
1	1	14.2	14.2	14.2	11.535	106	106;107;14	0
1	2	13.3	13.3	13.3	19.186	173	173;197;13	0
2	2	10.8	10.8	10.8	33	305	305;423;42	0.001572
4	4	11.7	11.7	11.7	63.836	582	582;526;22	0
2	1	30.4	30.4	30.4	11.799	102	102;146;14	0
0	1	8	8	8	16.552	137	137;300	0.008057
0	1	6.2	6.2	6.2	19.125	176	176	0
8	9	39.6	39.6	39.6	42.835	394	394;313;32	0
4	4	5.2	5.2	5.2	103.13	928	928	0
2	0	7.7	7.7	7.7	33.31	284	284;128;22	0
1	1	4	4	4	28.466	247	247	0.000534
2	2	17	17	17	15.911	147	147	0
1	1	6.9	6.9	6.9	31.357	274	274;282;28	0
5	3	14.3	14.3	14.3	59.271	546	546	0
9	8	24.5	24.5	24.5	62.006	560	560;611;63	0
2	1	1.3	1.3	1.3	308.77	2819	2819;2653;	0.004815
6	7	17.8	17.8	17.8	56.084	522	522	0
1	1	11.4	11.4	11.4	18.388	158	158;177;18	0
3	3	4.8	4.8	4.8	208.7	1871	1871	0
2	2	17.6	17.6	17.6	9.681	85	85;90	0
3	3	35.1	35.1	35.1	11.203	97	97	0
4	4	34.3	34.3	34.3	11.74	105	105	0
1	1	11.2	11.2	11.2	11.471	98	98	0.000537
5	4	65.4	65.4	65.4	11.662	104	104	0
2	2	22.3	22.3	22.3	11.801	103	103	0
3	5	27.6	27.6	27.6	11.117	98	98;64;95	0

2	2	10.5	10.5	10.5	29.81	266	266	0
3	4	23.3	23.3	23.3	35.54	322	322;308;32	0
7	7	30.7	30.7	30.7	32.103	277	277;266;26	0
1	3	3.7	3.7	3.7	228.18	2108	2108;2140;	0
1	1	7.9	7.9	7.9	18.967	178	178;274;53	0.00154
1	1	10.6	10.6	10.6	29.497	274	274;521;63	0
4	4	25.4	25.4	25.4	30.921	280	280;284;39	0
2	1	2.7	2.7	2.7	111.98	1042	1042;342;1	0
1	1	1.5	1.5	1.5	96.502	877	877;969;12	0.008984
4	4	8.5	8.5	8.5	52.949	482	482;476;35	0
2	2	37.5	37.5	37.5	9.9743	96	96;42	0
0	0	17.6	17.6	17.6	7.7412	68	68	0
1	1	2.7	2.7	2.7	118.39	1084	1084;929;9	0.00202
1	1	19	19	19	8.7513	79	79;241;293	0
1	1	4.7	4.7	4.7	27.989	257	257;265	0
5	5	23.5	23.5	23.5	30.315	285	285;286;22	0
2	1	3	3	3	164.2	1458	1458;1472;	0
5	3	36.4	36.4	36.4	11.758	107	107;110;11	0
2	5	11.4	11.4	11.4	114.74	1060	1060;1237;	0
1	1	3.6	3.6	3.6	116.07	1069	1069;1072;	0
7	6	34.3	34.3	34.3	32.118	289	289;221;93	0
4	4	25.6	25.6	25.6	26.777	242	242;300;23	0
7	8	10.5	10.5	10.5	102.99	915	915;919;83	0
2	2	6.8	6.8	6.8	53.753	483	483;530;51	0
1	1	6.4	6.4	6.4	17.479	156	156	1
0	1	2.1	2.1	2.1	130.55	1166	1166;1155;	0.004796
9	9	42.9	42.9	27.3	50.184	462	462;462;44	0
1	1	22.8	3.9	3.9	44.348	381	381	0.000534
1	1	9.9	2.2	2.2	58.264	505	505	0
2	3	3.9	3.9	3.9	107.21	947	947;1003;9	0
2	1	7.2	7.2	7.2	42.008	376	376;87	0
0	0	2.7	2.7	2.7	57.215	524	524;472;48	0
0	1	2.5	2.5	2.5	102.13	933	933;913;95	0.00053
2	2	9.2	9.2	9.2	54.223	458	458;458;38	0
0	0	4.2	4.2	4.2	38.155	356	356	0.008975
0	0	8.3	8.3	8.3	12.311	109	109	1
2	2	12.2	12.2	12.2	25.868	246	246;359;32	0
5	4	8.8	8.8	8.8	129.63	1178	1178;298;5	0
3	3	8.9	8.9	8.9	40.188	359	359;390;39	0
2	4	17	17	17	37.514	341	341;341;35	0
1	1	3.9	3.9	3.9	51.459	486	486;501	0.002489
4	4	71.6	71.6	9.4	57.936	531	531;516;45	0
1	1	66.3	4.1	4.1	58.061	531	531;485;28	0.008924
12	12	33.5	33.5	33.5	50.663	445	445;400;26	0
2	3	7.9	7.9	7.9	71.026	632	632;280;11	0
4	4	42.2	42.2	42.2	17.745	161	161;79;99;1	0
1	1	25.3	25.3	25.3	8.2153	79	79;90;173;9	0.001538
8	8	24.5	24.5	24.5	63.541	587	587;253;10	0
3	3	27	27	27	23.239	200	200;201;16	0
9	8	26	26	26	52.164	466	466;148;10	0

32	32	30.9	30.9	30.4	189.25	1657	1657;1191;	0
1	2	11.2	11.2	11.2	25.545	224	224;277	0.002491
3	3	49.5	49.5	19.3	21.45	192	192;211;19	0
2	2	42.2	12	12	21.429	192	192;148;18	0
0	1	7	7	7	44.223	399	399;400;24	0.000541
5	5	30.5	25	25	22.541	200	200;203;12	0
9	9	54.1	54.1	50.5	24.488	218	218;179;15	0
12	12	72.6	72.6	72.6	23.897	215	215;181	0
5	7	46.6	46.6	46.6	22.977	206	206;235;16	0
4	4	59.5	59.5	22	22.677	205	205;173;14	0
2	1	63.2	32.8	24.4	22.171	201	201;169;20	0
3	4	23.6	23.6	23.6	24.347	225	225	0
1	1	8.9	5.2	5.2	23.495	213	213	0.008519
1	1	15.5	15.5	15.5	7.8818	71	71;95;136;	0.001556
6	5	37.7	37.7	37.7	23.545	212	212;181;18	0
3	2	29.3	29.3	24.4	24.997	225	225;211;89	0
1	2	15.7	15.7	15.7	21.214	185	185;201;15	0
0	1	5.2	5.2	5.2	23.339	212	212	0.009333
7	7	38.9	38.9	38.9	23.482	216	216;249;16	0
4	4	27.4	27.4	27.4	23.548	208	208;208;71	0
14	15	76.3	76.3	76.3	23.489	207	207;160;13	0
1	1	15.8	10.3	10.3	23.155	203	203;179;42	0.00297
1	1	14.5	5.8	5.8	23.564	207	207;207	0
1	0	18.4	18.4	18.4	22.837	201	201;23;201	0
2	2	29.3	29.3	22.6	18.26	164	164;206	0
2	2	37.6	37.6	17	18.778	165	165;184;15	0
1	1	26.8	26.8	15.3	20.504	183	183	0
1	1	17.9	5.5	5.5	23.48	218	218	0
2	2	23.5	23.5	10.3	23.399	204	204;210;16	0
1	1	28.8	28.8	28.8	5.8998	59	59;79	0.000536
2	2	15.9	15.9	15.9	21.493	189	189;99;122	0
2	1	3.3	3.3	3.3	102.56	935	935;987;99	0
2	3	4.5	4.5	4.5	211.68	1898	1898;1907;	0
3	3	21.1	21.1	21.1	24.462	218	218;229	0
8	7	31.1	31.1	31.1	44.969	421	421;452;37	0
2	3	12	12	12	32.365	284	284;314;10	0
0	1	6.8	6.8	6.8	27.423	251	251;287;47	0.000536
1	1	1.5	1.5	1.5	123.03	1118	1118;1129	0
2	2	5.8	5.8	5.8	89.09	828	828;128	0
2	3	13.8	13.8	13.8	39.157	354	354;320;28	0
2	5	24.6	24.6	24.6	34.756	305	305;356;10	0
2	2	9.2	9.2	9.2	36.877	336	336;303;36	0
1	2	11.3	11.3	11.3	36.104	319	319;340;63	0.001578
3	2	52.4	52.4	52.4	9.1703	82	82;121	0
2	2	9.6	9.6	9.6	29.247	270	270;278;35	0
1	2	3.6	3.6	3.6	68.137	616	616;313	0
7	7	31.1	31.1	31.1	38.89	331	331;280	0
2	3	22.6	22.6	22.6	17.609	155	155;317;33	0
7	7	30.8	30.8	30.8	40.317	373	373;392;11	0
1	1	1.4	1.4	1.4	106.16	928	928	0.001523

2	2	8.7	8.7	8.7	40.251	357	357;273	0
1	2	12.3	12.3	12.3	38.087	341	341;176	0
4	5	23.6	23.6	23.6	35.386	318	318;178;30	0
1	2	7.4	7.4	7.4	36.864	336	336	0.000532
3	3	24.8	24.8	24.8	14.814	129	129;193;23	0
2	2	34.1	34.1	34.1	9.792	88	88;112;158	0
4	4	15.7	15.7	15.7	50.435	439	439;334;19	0
0	1	8.1	8.1	8.1	22.187	197	197;300;30	0.002946
1	1	24.5	24.5	6.4	21.523	188	188;193;16	0
4	4	41.4	35.6	35.6	21.308	191	191	0
8	10	30.4	30.4	30.4	49.973	461	461;199;18	0
2	3	4.7	4.7	4.7	90.069	792	792;570;75	0
4	4	18.3	18.3	18.3	33.789	289	289;374;38	0
2	2	11.3	8.8	8.8	34.834	318	318;218;25	0
3	3	16.7	16.7	14.2	34.769	318	318;321;20	0
1	1	4.8	4.8	4.8	33.108	291	291;717;73	0
10	10	33.7	33.7	33.7	54.972	490	490;430;27	0
4	4	17.6	17.6	17.6	23.134	193	193;194;19	0
1	3	16.7	16.7	16.7	52.839	461	461;45	0
3	3	11.9	11.9	11.9	26.72	244	244;149;21	0
0	1	8.6	8.6	8.6	32.334	280	280	0
3	2	6.3	6.3	6.3	145.81	1282	1282	0
6	4	13.7	13.7	13.7	83.629	746	746;634;21	0
5	3	18.1	18.1	18.1	41.401	353	353	0
3	2	17.5	17.5	17.5	22.653	189	189;260;98	0
2	2	15.8	15.8	15.8	47.707	423	423	0
2	1	7.4	7.4	7.4	41.193	365	365	0
2	1	20.9	20.9	20.9	9.7691	86	86;250;250	0.002002
3	0	24.3	24.3	24.3	24.605	214	214;306;59	0
10	13	16.9	16.9	16.9	152.45	1410	1410;1407;	0
3	2	30	30	30	23.51	210	210;218;26	0
0	0	4.2	4.2	4.2	31.886	285	285;513;60	0.003901
11	10	30.7	30.7	30.7	42.331	391	391;378;29	0
7	5	18.8	18.8	15.2	69.491	669	669;156	0
0	0	19.2	12.1	0	37.034	339	339;118;12	0
3	3	6.4	6.4	6.4	100.18	843	843;243;29	0
0	0	0.9	0.9	0.9	114.54	1027	1027;1060	1
4	4	8.4	8.4	8.4	85.737	759	759;618;20	0
2	1	10.4	10.4	10.4	24.079	211	211;408;22	0
7	6	34.7	34.7	34.7	26.698	245	245;423;50	0
3	2	20.7	20.7	20.7	19.889	174	174;173;10	0
6	5	14.1	14.1	14.1	64.929	618	618;600;65	0
3	3	13.9	9.5	9.5	53.496	527	527;525;52	0
1	1	13.6	13.6	13.6	7.5737	66	66;412	0.005725
1	2	9.5	9.5	9.5	27.924	252	252;136	0
5	4	24.9	24.9	24.9	24.665	237	237;290;30	0
1	1	12.8	12.8	12.8	24.561	211	211;257;28	0
1	2	1.4	1.4	1.4	148.25	1313	1313;2017;	0.001054
4	4	27.9	27.9	27.9	25.308	229	229;244;32	0
1	1	3.1	3.1	3.1	38.638	353	353	0.001518

0	0	3.3	3.3	3.3	34.803	334	334	0
0	1	18	18	18	7.0253	61	61;183;186	0.001541
2	1	3.7	3.7	3.7	132.71	1197	1197;1236;	0
1	1	12.4	12.4	12.4	31.484	282	282	0
10	10	37.9	37.9	37.9	50.227	456	456;386;31	0
11	12	37.3	37.3	37.3	46.306	418	418;463;35	0
5	5	21.5	21.5	21.5	43.66	395	395;299	0
1	2	9.4	9.4	9.4	24.105	213	213;523;21	0
18	16	24.9	24.9	24.9	114.76	1042	1042;997;9	0
3	2	16.4	16.4	7.2	95.18	848	848;915;91	0
1	1	13.2	5	5	107.47	953	953;115	0
1	1	2.5	2.5	2.5	53.847	481	481;499;40	0
0	1	1.6	1.6	1.6	101.05	891	891	0
3	2	9.3	9.3	9.3	72.379	642	642;550;57	0
3	3	9.7	9.7	9.7	46.382	414	414;434;34	0
1	0	13.4	13.4	13.4	13.322	119	119;157;33	0
1	1	6.1	6.1	6.1	36.648	329	329	0
2	2	19	19	19	38.287	347	347;321	0
0	1	5.6	5.6	5.6	22.047	195	195;229	0.008937
1	1	5.7	5.7	5.7	28.048	261	261	0.000533
0	0	8.7	8.7	8.7	14.133	126	126;136;37	0.009777
14	13	53.2	53.2	53.2	47.687	440	440;356;37	0
1	2	9.9	9.9	9.9	41.239	373	373;547	0
14	12	37.5	37.5	34.6	53.454	483	483;504;49	0
2	2	11.3	11.3	11.3	50.494	453	453	0
5	7	21.6	21.6	21.6	52.743	473	473;143	0
2	1	4.1	4.1	4.1	62.924	562	562;407	0
0	0	1.5	1.5	1.5	78.139	681	681;820;91	0.008954
6	7	7	7	7	299.61	2752	2752;2334;	0
6	6	30.4	30.4	30.4	28.329	253	253;248;20	0
1	0	13.4	13.4	13.4	20.913	172	172;165;17	0.002966
1	1	4.4	4.4	4.4	42.316	389	389;483;48	0.004792
5	5	42.1	42.1	42.1	15.371	133	133;221;13	0
3	3	40	40	40	10.32	95	95;124;164	0
2	2	13.4	10	10	30.858	269	269;272;12	0
4	3	15.2	15.2	12.5	38.418	335	335;344;37	0
4	5	42.3	42.3	42.3	15.763	137	137;235;13	0
7	5	33	33	33	25.542	221	221;109;11	0
1	1	8.4	8.4	4.1	40.781	369	369;412;43	0
1	2	4.6	4.6	4.6	78.452	699	699;778;68	0
1	1	3	3	3	51.856	468	468;527	0.000539
4	4	15.8	15.8	15.8	58.042	524	524;181;22	0
1	1	41.5	41.5	41.5	7.3568	65	65;396	0.007615
2	2	5.1	5.1	5.1	51.691	447	447;457;19	0
11	9	22.9	22.9	22.9	65.308	589	589;395;41	0
3	3	17.1	17.1	17.1	30.158	263	263;288;32	0
6	6	33.3	33.3	28.8	35.594	309	309;255;30	0
4	4	21.2	21.2	21.2	28.02	255	255;289;14	0
2	2	31.5	7.3	7.3	32.595	286	286;330;34	0
3	3	31.5	31.5	10.4	37.186	327	327;169;13	0

1	2	6.9	6.9	6.9	47.815	423	423;454;46	0
3	0	26.1	19.2	19.2	23.145	203	203;267;30	0
2	2	30.6	7.9	7.9	34.95	304	304;323;33	0
5	5	22.9	22.9	22.9	38.438	350	350;363;95	0
3	4	9.5	9.5	9.5	61.312	536	536;514	0
1	1	6.9	6.9	6.9	58.182	518	518;588;51	0
4	4	27.9	27.9	27.9	18.885	165	165;400;33	0
13	13	51.2	51.2	51.2	42.1	375	375;231;12	0
6	5	20.8	20.8	20.8	43.024	380	380;395;37	0
11	11	35.2	35.2	35.2	46.44	418	418;295;32	0
2	3	3.7	3.7	3.7	96.221	839	839;871;87	0
0	1	4.7	4.7	4.7	37.98	342	342;354;37	0
1	1	15	15	15	12.999	120	120;230;25	0.00296
2	2	20.5	20.5	20.5	9.3804	88	88;93	0
2	2	6.7	6.7	6.7	40.307	359	359	0
2	1	23.4	23.4	23.4	35.503	321	321;325;32	0
4	5	23.6	23.6	23.6	35.619	322	322;144;17	0
1	2	11.7	11.7	11.7	18.651	163	163;164;17	0.000529
1	3	31.4	31.4	31.4	11.805	102	102;80;169	0
3	4	31.8	31.8	31.8	17.027	157	157;227;25	0
3	1	17.8	17.8	17.8	20.313	180	180	0
2	4	39.1	39.1	39.1	13.058	115	115;136	0
2	2	24.2	24.2	24.2	13.654	120	120;144;10	0
3	4	45.3	45.3	45.3	10.112	86	86;66;82	0
4	3	28	28	28	29.702	271	271;160	0
3	3	6.1	6.1	6.1	66.107	589	589;596;62	0
3	3	6.4	6.4	6.4	74.605	671	671;357;61	0
5	4	9.8	9.8	9.8	83.359	714	714;712;75	0
1	1	3	3	3	44.971	403	403;540	0.002978
1	0	4.1	4.1	4.1	38.37	342	342;525	0.001573
3	4	36.5	36.5	36.5	17.259	148	148;134;13	0
1	1	18.5	18.5	18.5	17.176	162	162;193;19	0
2	2	16	16	16	17.792	162	162;313	0
2	2	24.4	24.4	24.4	9.7251	86	86	0
2	2	20.2	20.2	20.2	13.281	119	119;44;75	0
2	2	36.1	36.1	36.1	12.398	108	108;118;78	0
4	4	50	50	50	13.291	120	120;126;62	0
5	4	30.2	30.2	30.2	17.546	169	169;231;24	0
0	1	1.1	1.1	1.1	318.38	2785	2785	0
2	2	25.3	25.3	25.3	16.645	146	146;101;58	0
2	2	31	31	31	8.1111	71	71;95;135;	0
1	0	13.5	13.5	13.5	20.974	185	185;571;53	0.002981
24	24	31.6	31.6	30.7	113	1023	1023;1023;	0
4	3	15.3	15.3	15.3	34.893	301	301;303;13	0
3	3	12.2	12.2	12.2	31.512	279	279;119;13	0
2	2	4	4	4	56.195	503	503;487	0.002957
2	2	9.5	9.5	9.5	56.025	506	506;406;88	0
3	2	11.4	11.4	11.4	51.457	472	472;163;19	0
0	1	4.7	4.7	4.7	23.531	213	213;679	0.001057
1	1	3.4	3.4	3.4	124.59	1150	1150;1196;	0

3	3	10.4	10.4	10.4	54.083	492	492	0
1	1	4.6	4.6	4.6	54.557	479	479	0.00155
8	6	34.2	34.2	34.2	38.324	357	357;336;11	0
3	3	42.6	42.6	42.6	17.605	155	155;180;18	0
1	0	5.8	5.8	5.8	51.976	469	469	0.000532
0	2	15.7	15.7	15.7	19.73	172	172;172;15	0
2	2	36.2	36.2	36.2	14.766	130	130;162;11	0
8	8	24.9	24.9	24.9	54.178	489	489;558;49	0
2	2	12.1	12.1	12.1	37.523	355	355;601	0
26	25	15.5	15.5	15.5	282.83	2457	2457;2498;	0
15	14	9.9	9.9	9.9	274.83	2366	2366;2364;	0
3	3	20.9	20.9	20.9	18.72	163	163;163;10	0
0	0	3.6	3.6	3.6	94.584	837	837;735;83	0
1	1	19.3	19.3	19.3	14.864	135	135;202;56	0
2	2	28.7	28.7	7.2	48.991	428	428;443;32	0
3	3	7.3	7.3	7.3	59.711	548	548;571;61	0
2	2	5.5	5.5	5.5	88.885	793	793;728;25	0
3	3	12.4	12.4	12.4	58.848	501	501	0
9	7	9.7	9.7	9.7	145.83	1304	1304;37;15	0
5	6	10.6	10.6	10.6	100.23	895	895;273;35	0
7	6	11.3	11.3	11.3	135.58	1217	1217;399;2	0
2	2	20.8	20.8	20.8	14.585	125	125	0
4	3	20	20	20	27.872	240	240;240;24	0
4	4	30.9	30.9	30.9	33.901	307	307;471;47	0
13	14	27.7	27.7	26.6	76.149	707	707;669;22	0
3	1	15	15	15	23.128	207	207	0
9	9	30.7	30.7	30.7	47.285	410	410;417;47	0
0	1	3.1	3.1	3.1	89.04	771	771;927;96	0.009781
1	1	41.3	41.3	41.3	10.353	92	92;96;98;1	0
14	14	37.3	37.3	37.3	61.585	550	550;513;63	0
15	13	23.5	23.5	23.5	102	910	910;231	0
5	3	31.5	31.5	31.5	17.302	149	149;85;174	0
1	1	6	6	6	19.574	168	168;179;18	0.005261
2	2	1.4	1.4	1.4	184.28	1589	1589;1283	0
1	0	2.3	2.3	2.3	70.256	654	654;684;68	0
6	6	22.3	22.3	22.3	41.9	373	373;254	0
4	6	33.2	33.2	33.2	33.409	310	310;356;31	0
1	1	1	1	1	101.16	905	905;920	0.000541
26	25	54.8	54.8	54.8	73.68	679	679;95;163	0
15	14	34.1	34.1	34.1	62.639	543	543;590;51	0
1	1	14.9	14.9	14.9	23.598	221	221	0
1	0	2.5	2.5	2.5	45.263	405	405;512;79	0.002941
4	6	5.4	5.4	5.4	135.65	1197	1197;1099;	0
3	2	5.8	5.8	5.8	141.54	1217	1217	0
4	3	3.8	3.8	3.8	140.86	1211	1211;1233;	0
5	5	6	6	6	144.45	1263	1263;1230;	0
7	7	21.4	21.4	21.4	67.299	616	616;664;58	0
1	1	14.2	14.2	14.2	36.249	346	346;105	0
4	2	11.6	11.6	11.6	48.039	441	441;463;19	0
2	1	13	13	13	46.51	432	432;190;37	0

5	5	15.4	15.4	15.4	56.157	520	520;334;12	0
2	2	12.5	12.5	12.5	33.995	305	305;320;30	0
10	9	33.8	33.8	33.8	49.96	450	450;289;18	0
1	1	10.7	10.7	10.7	22.622	205	205;265;29	0
3	3	41.4	41.4	41.4	7.8891	70	70;137;184	0
0	0	2.8	2.8	2.8	46.772	427	427;634;71	0
2	1	2	2	2	117.8	1042	1042;88	0.002964
3	3	21.4	21.4	21.4	15.936	154	154;135	0
5	5	32.4	32.4	32.4	19.73	176	176;222;11	0
4	4	16.2	16.2	16.2	37.804	333	333;365	0
1	0	6.3	6.3	6.3	23.721	207	207;211	0.002962
1	1	15.7	15.7	15.7	47.144	420	420;520;79	0
0	0	5.6	5.6	5.6	31.978	288	288;300	0.001574
1	1	15.1	15.1	15.1	21.127	186	186;159;26	0
1	1	12.7	12.7	12.7	41.45	361	361	0
0	2	13	13	13	26.711	238	238	0
2	0	5.5	5.5	5.5	136.18	1245	1245;1130;	0
7	7	9	9	8.1	121.9	1052	1052	0
1	1	7.2	7.2	7.2	52.238	456	456;494;48	0.001566
1	2	15.8	15.8	15.8	20.549	177	177;288;39	0
1	1	3.6	3.6	3.6	62.032	527	527;585	0
9	7	37.9	37.9	37.9	41.92	393	393;146;32	0
2	1	10.4	10.4	10.4	18.58	164	164;198;29	0
1	0	10.4	10.4	10.4	26.411	241	241;259	0
1	1	1.5	1.5	1.5	79.145	753	753;977	0.000532
1	1	7.8	7.8	7.8	28.97	258	258	0
0	2	19.7	19.7	19.7	15.62	142	142;149;15	0
1	1	11.6	11.6	11.6	32.461	310	310;169;21	0
2	2	16.3	16.3	16.3	16.545	153	153;198	0
0	1	9.4	9.4	9.4	29.176	255	255	0
2	2	11.7	11.7	11.7	27.4	239	239;261	0
1	1	63.1	63.1	6	32.444	298	298;292;23	0
1	1	63	5.7	5.7	32.316	297	297;318	0
8	8	4.8	4.8	4.8	269.76	2541	2541;1471;	0
1	1	5.1	5.1	5.1	47.606	448	448;466;46	0
6	5	17.1	17.1	17.1	44.739	414	414;243;29	0
3	3	21.4	21.4	17.5	61.557	589	589;592;44	0
6	4	11.2	11.2	11.2	93.609	813	813;563;59	0
1	1	13.7	13.7	13.7	13.735	124	124	0
1	2	5.2	5.2	5.2	77.394	674	674;682;69	0
1	1	3.6	3.6	3.6	54.418	468	468;502;71	0.001558
17	17	40.1	40.1	40.1	60.343	556	556;401;33	0
16	14	36.3	36.3	36.3	57.488	535	535;530;48	0
14	15	35.6	35.6	35.6	54.719	509	509;539	0
10	11	33.5	33.5	33.5	53.848	486	486;541;50	0
8	8	26.3	26.3	26.3	54.804	499	499;543;45	0
14	14	38	38	38	60.533	545	545;500;50	0
17	16	43.4	43.4	43.4	59.62	548	548;529;47	0
9	10	20.5	20.5	20.5	58.024	531	531;486;48	0
2	3	1.8	1.8	1.8	271.69	2446	2446;2472;	0

2	1	2.1	2.1	2.1	129.06	1205	1205;1445;	0
2	2	5.1	5.1	5.1	46.91	412	412;421;79	0.001561
3	1	14	14	14	19.792	178	178;251;25	0
2	2	27.6	27.6	27.6	16.021	145	145;215;21	0
2	3	35.8	35.8	35.8	17.963	165	165;209;22	0
0	1	22	22	22	17.764	159	159;228;22	0
1	1	5.4	5.4	5.4	26.301	241	241	0
1	1	10.3	10.3	10.3	22.147	204	204	0
1	1	3	3	3	78.839	689	689;176;56	0
5	5	51.4	51.4	51.4	11.737	105	105;85	0
1	1	14	14	14	19.206	172	172	0
3	3	26.8	26.8	26.8	13.941	123	123;75;80;!	0
6	6	25.7	25.7	25.7	47.628	432	432;324	0
5	5	19.3	19.3	16.2	53.166	482	482;498;54	0
6	5	39.1	39.1	39.1	25.838	238	238;256	0
5	5	32.9	32.9	32.9	32.251	289	289;281;13	0
2	3	13.9	13.9	13.9	31.791	280	280;105	0
6	5	34.1	34.1	34.1	29.642	258	258;296;84	0
1	1	8.1	8.1	8.1	33.429	297	297;124	0
0	1	0.9	0.9	0.9	169.58	1478	1478;1593	0.00249
5	6	30.4	30.4	30.4	26.888	257	257;264	0
1	0	5.4	5.4	5.4	23.743	204	204	1
15	16	28.6	28.6	28.6	83.434	723	723;756;11	0
9	11	12.7	12.7	12.7	129.38	1170	1170;1085;	0
1	1	10.3	10.3	10.3	15.761	145	145;353	0.000537
0	0	6.7	6.7	6.7	19.786	180	180;187;26	0
2	1	31.8	31.8	31.8	5.0526	44	44	0
6	7	12.4	12.4	12.4	108.66	955	955	0
12	10	46	46	46	50.287	476	476;215	0
9	9	10.5	10.5	10.5	140.73	1249	1249;1157;	0
1	0	6.2	6.2	6.2	20.214	178	178;272	0.002972
5	6	20.3	20.3	20.3	46.172	404	404;188;18	0
2	3	11.2	11.2	11.2	33.067	295	295;238	0
1	1	0	0	0	3014	27118	27118;359!	1
1	3	12.6	12.6	12.6	55.697	492	492;619;54	0
0	0	17.5	17.5	17.5	8.6579	80	80;213;274	0
6	5	14	14	14	67.821	599	599;584;46	0
0	0	3.2	3.2	3.2	42.692	379	379;376	0
7	6	23.4	23.4	23.4	37.54	337	337;318	0
0	1	0.7	0.7	0.7	188.15	1679	1679;1681;	0
5	4	20.9	20.9	20.9	33.935	301	301;301;11	0
2	3	48.3	48.3	48.3	9.9601	89	89;112;96;(	0
3	3	28.3	28.3	28.3	12.527	113	113;140;11	0
2	1	2.2	2.2	2.2	121.69	1077	1077;1098	0.001563
3	2	14.6	14.6	14.6	29.096	246	246;219;21	0
1	1	17.3	17.3	17.3	17.699	162	162;206	0.008958
11	10	22.4	22.4	22.4	88.549	835	835;753;46	0
0	1	7.3	7.3	7.3	82.785	727	727	0
1	1	4.2	4.2	4.2	28.673	263	263;577;59	0.002016
2	2	8.3	8.3	8.3	45.935	411	411;447;44	0

6	5	15.5	15.5	15.5	89.034	808	808;697;51	0
22	22	36.3	36.3	36.3	84.87	760	760;679;81	0
1	1	12.4	12.4	12.4	12.951	113	113;180;18	0
3	2	16.3	16.3	16.3	33.665	288	288;188;10	0
1	1	4.2	4.2	4.2	74.68	683	683;128;36	0
1	1	23.8	6.2	6.2	21.768	193	193	0
10	10	66.3	66.3	66.3	22.391	199	199;220;18	0
2	0	11.7	11.7	11.7	29.541	281	281;379;43	0
20	20	34.4	34.4	34.4	89.321	806	806;115;16	0
17	18	36.8	36.8	36.8	67.877	623	623;631;45	0
7	8	4.1	4.1	4.1	292.75	2671	2671	0
2	2	12.8	12.8	12.8	32.477	297	297	0
6	6	33.5	33.5	33.5	21.525	188	188;197;17	0
3	2	11.1	11.1	11.1	33.435	288	288;343;37	0
2	1	21.2	21.2	21.2	9.8703	85	85;399;101	0
4	4	7	7	7	87.996	760	760	0
1	2	15	15	15	11.896	107	107;169	0.000535
1	1	12.8	12.8	12.8	29.573	265	265;266;29	0
3	3	24.3	24.3	24.3	18.998	173	173;148	0
1	1	12.6	12.6	12.6	20.161	174	174;185;19	0
3	2	5.8	5.8	5.8	66.911	589	589;815;48	0
5	5	15.2	15.2	15.2	75.775	663	663	0
2	3	6.6	6.6	6.6	67.887	589	589;253	0
5	2	11.4	11.4	11.4	72.54	625	625;642	0
1	1	7.1	7.1	7.1	27.079	239	239;169;18	0.002009
1	1	5.7	5.7	5.7	25.206	227	227	0
1	2	15	15	15	16.904	153	153;219	0
1	1	7.8	7.8	7.8	13.631	116	116;166;20	0
0	0	21.3	21.3	13.3	24.069	211	211;227;17	0
1	1	9.6	9.6	9.6	26.005	229	229;120;16	0
2	1	21.3	14	14	27.277	235	235	0
3	2	5.9	5.9	5.9	62.642	560	560;572	0
2	2	9.1	9.1	9.1	26.21	243	243	0
2	2	21.3	21.3	21.3	34.905	324	324;158;26	0
0	1	9.7	9.7	9.7	30.549	267	267;271	0
0	1	11.2	11.2	11.2	9.743	89	89;125;208	1
0	0	10.8	10.8	10.8	13.389	120	120;128;17	0
1	1	7.8	7.8	7.8	72.039	644	644;689;30	0
0	0	68.3	68.3	68.3	3.9694	41	41;68;113	0
2	2	9.9	9.9	9.9	25.223	222	222;247;15	0
3	3	14	14	14	44.875	400	400	0
2	3	17.6	17.6	17.6	36.223	323	323;192;28	0
2	2	8.5	8.5	8.5	56.773	494	494;555;19	0
1	0	14	14	14	20.958	186	186;183;13	0.001053
3	3	6	6	6	101.31	890	890;898;84	0
13	9	12.7	12.7	12.7	144.12	1414	1414;1488;	0
3	3	10	10	10	34.012	311	311;208	0
17	15	32.4	32.4	32.4	82.999	763	763;250;82	0
11	12	31.6	31.6	31.6	48.879	452	452;474;45	0
4	4	5.6	5.6	5.6	107.77	1010	1010;433;1	0

15	15	78.7	78.7	78.7	26.669	249	249;286;16	0
2	1	4.7	4.7	4.7	63.843	570	570;588	0
1	1	12.5	12.5	12.5	44.41	400	400;638	0
12	11	28.4	28.4	28.4	82.392	732	732;767;53	0
2	2	11	11	11	41.729	374	374;384;39	0
5	6	18.8	18.8	18.8	55.21	505	505	0
2	4	15.3	15.3	15.3	39.594	352	352;187;21	0
1	1	29.9	14.4	3.9	32.876	284	284;284;28	0
9	10	56	56	38.7	29.032	248	248;227;23	0
2	1	47.2	6	6	28.521	248	248;110;11	0
1	2	47.5	32.4	8.5	32.722	284	284;179;17	0
5	5	20	20	20	48.851	430	430;471;18	0
0	0	59.9	3.1	0	46.297	416	416;451;45	0
0	0	55.2	55.2	0	50.151	451	451;335;15	0
1	2	48	7.9	7.9	57.73	519	519;449;32	0
4	3	57.5	12.5	12.5	48.328	433	433;448;15	0
2	3	48.4	16.2	12.7	47.766	426	426;444;37	0
1	2	46.5	13.3	13.3	49.906	445	445;445	0
1	1	15.7	2.6	1.5	88.381	797	797;450;37	0
2	2	47.9	47.9	6.5	49.83	445	445;444;15	0
1	1	21.7	9.9	9.9	49.857	446	446;338;96	0
2	2	21.4	7.4	7.4	49.775	444	444;407;41	0
1	1	21.5	21.5	21.5	15.173	135	135;451;45	0
3	2	41.7	41.7	41.7	10.08	84	84;119;108	0
4	3	8.9	8.9	8.9	74.403	644	644	0
1	1	1.4	1.4	1.4	72.66	654	654	0.009795
1	0	3.6	3.6	3.6	45.083	411	411;440	0
2	2	6.2	6.2	6.2	56.749	501	501;582;58	0
3	3	39.1	39.1	39.1	19.863	184	184;207;22	0
5	5	30.6	30.6	30.6	23.764	222	222;186;20	0
3	3	22.8	22.8	22.8	30.211	272	272;213	0
1	1	1.1	1.1	1.1	213.57	1972	1972;1975;	0
8	7	27.2	27.2	27.2	35.709	323	323	0
2	2	7.4	7.4	7.4	40.282	350	350;59;291	0
1	1	7.5	7.5	7.5	28.882	254	254;1186;3	0
1	1	14.5	14.5	14.5	16.669	152	152;159	0
4	6	10.6	10.6	10.6	170.45	1479	1479;1483	0
3	4	13.4	13.4	13.4	56.033	491	491;512;52	0
7	8	21.1	21.1	21.1	49.966	435	435;362	0
3	3	6.1	6.1	6.1	59.143	528	528;388	0
3	3	12.7	12.7	12.7	32.184	291	291;477	0
5	6	17.6	17.6	17.6	51.556	437	437;428;16	0
1	1	14.8	9.2	9.2	15.945	142	142;148;18	0.003889
2	2	16.7	16.7	16.7	19.687	180	180;159	0
2	4	21.2	21.2	21.2	28.415	255	255;89;142	0
1	1	8.4	8.4	4.9	25.486	225	225;87;128	0.004817
3	3	5.3	5.3	5.3	118.23	1028	1028;1029;	0
2	0	15.2	15.2	15.2	21.85	184	184	0
0	0	4.7	4.7	4.7	78.863	681	681	0
3	4	10	10	10	82.004	719	719;771;60	0

2	0	5.2	5.2	5.2	62.003	556	556;143;17	0
2	2	4.4	4.4	4.4	70.193	597	597	0
2	1	8	8	8	51.84	475	475	0
1	1	3.1	3.1	3.1	58.32	521	521;522	0
0	2	5.5	5.5	5.5	56.399	487	487;536;46	0
15	14	9.6	9.6	9.6	244.5	2136	2136;625;5	0
3	3	14.6	14.6	14.6	39.31	357	357;409;12	0
1	1	20	20	20	10.834	95	95	0
1	1	23.9	23.9	23.9	7.3713	67	67;75;77;9	0.002984
3	2	14.1	14.1	8.4	59.219	561	561;589;41	0
0	1	20.6	20.6	20.6	11.422	102	102;172;18	0
0	1	10.9	10.9	10.9	12.383	110	110;798;80	0.002935
1	2	3	3	3	93.307	835	835;858;13	0.005717
6	6	35.7	35.7	35.7	26.182	230	230;194;22	0
8	7	25	25	25	51.086	448	448;459;48	0
2	2	16.1	16.1	16.1	25.335	218	218;258;35	0.000538
2	1	8.1	8.1	8.1	34.5	307	307;343;13	0
4	4	22.4	22.4	22.4	28.05	241	241;272;27	0
7	7	50.6	50.6	50.6	17.965	156	156;93;106	0
4	2	6.5	6.5	6.5	113.63	1079	1079;976;9	0
2	2	27.9	27.9	27.9	15.765	140	140;150;17	0
3	3	21.5	21.5	21.5	22.406	200	200;123;14	0
1	1	11.7	11.7	11.7	17.875	154	154;122;15	0
3	4	32.9	32.9	32.9	17.138	152	152;87;105	0
1	1	10.5	10.5	5.1	42.208	370	370;147;17	0
2	1	20.7	6.9	6.9	16.363	145	145	0.005728
1	1	18.5	18.5	18.5	8.5507	81	81;85	0
20	19	28.8	28.8	28.8	113.8	1018	1018;1058;	0
2	1	24.7	24.7	24.7	8.5468	73	73;36	0.000531
0	1	5	5	5	27.015	238	238;290;29	0.001542
3	2	13.6	13.6	13.6	56.777	508	508	0
7	9	29.4	29.4	29.4	55.023	494	494;427;39	0
6	5	9.3	9.3	9.3	174.97	1531	1531;1555;	0
3	2	9	9	9	50.527	443	443;532;18	0
4	4	32.1	32.1	32.1	22.222	196	196;169;14	0
1	1	3.3	3.3	3.3	34.723	302	302;325	0.004819
1	1	3.8	3.8	3.8	32.544	291	291	0.008933
2	2	17.2	17.2	17.2	21.883	192	192;194;16	0
16	16	20.3	20.3	20.3	128.48	1107	1107;1078;	0
11	11	14.8	14.8	14.8	118.99	1039	1039;1044;	0
3	3	3.4	3.4	3.4	127.06	1108	1108;187;1	0
1	1	2.4	2.4	2.4	145.01	1253	1253;1294;	0
1	0	7	7	7	24.992	229	229;242	0.001537
1	1	12.4	12.4	12.4	14.726	129	129;193;24	0
1	1	6.9	6.9	6.9	28.487	261	261;208;21	0
1	2	43.1	43.1	43.1	6.4575	58	58	0
2	3	14.2	14.2	14.2	42.495	388	388;480;34	0
3	2	24.3	24.3	24.3	15.621	144	144;146;17	0
1	1	6.4	6.4	6.4	24.884	218	218;201;26	0
1	1	8.3	8.3	8.3	21.458	193	193;228;29	0.001551

1	1	5.8	5.8	5.8	16.276	139	139;124;13	0.00895
3	2	8.3	8.3	8.3	56.967	508	508;517;49	0
8	8	23	23	23	43.171	409	409;337;11	0
0	0	11.9	11.9	11.9	11.354	101	101	0
2	2	14.4	14.4	14.4	38.169	327	327;226;25	0
1	1	71.4	71.4	71.4	3.9415	35	35;53;161;[illegible]	0
5	5	11.8	11.8	11.8	91.706	796	796;41;46;[illegible]	0
1	0	5.6	5.6	5.6	49.301	444	444;428;43	0
2	1	6.8	6.8	6.8	31.102	280	280;307;24	0.001576
5	5	6	6	6	131.83	1183	1183;1264;	0
0	1	3.1	3.1	3.1	59.747	522	522;524	0
18	16	42.4	42.4	42.4	70.389	655	655;678;63	0
0	0	3.9	3.9	3.9	28.368	254	254	0.000537
5	5	21.3	21.3	21.3	43.159	362	362;400;33	0
3	2	12.3	12.3	12.3	30.361	276	276;312;16	0
2	3	10.4	10.4	10.4	36.034	308	308;131;10	0
1	0	9.3	9.3	9.3	11.756	108	108;160	0.001056
3	3	52	52	52	11.309	100	100;68;72;[illegible]	0
1	1	33	33	33	11.438	100	100;99;74	0
1	1	12.4	7.6	7.6	27.893	249	249	0
3	4	25.5	25.5	20.6	27.228	243	243;71;99;[illegible]	0
1	0	1.6	1.6	1.6	82.091	739	739;644;74	0.007597
0	1	32.4	32.4	32.4	8.1425	71	71;99;101;[illegible]	0
3	3	20	20	20	24.593	215	215;79	0
1	1	15.7	15.7	15.7	40.228	356	356;191;32	0
7	7	8.8	8.8	8.8	141.44	1268	1268;1268;	0
15	17	25.5	25.5	25.5	116.72	1066	1066;1134;	0
0	0	9.3	9.3	9.3	53.341	474	474;475;34	0
1	1	5.7	5.7	5.7	19.835	176	176;177	0.005736
15	15	71.4	71.4	71.4	30.772	283	283;183	0
14	15	70.4	70.4	70.4	31.566	294	294;282;28	0
6	5	36.7	36.7	36.7	30.658	283	283;284;10	0
1	1	3.3	3.3	3.3	94.198	810	810	0.000531
0	1	11.6	11.6	11.6	15.736	155	155	0.004826
4	5	16.6	16.6	16.6	64.735	584	584;617;18	0
0	1	16.2	16.2	16.2	8.0792	68	68;93;130;[illegible]	0.001546
6	7	20	20	20	56.5	511	511;496;51	0
1	0	36.4	36.4	36.4	4.7434	44	44;119;179	0
3	1	11.7	11.7	11.7	44.659	392	392;351;23	0
3	4	20.8	20.8	20.8	26.145	226	226;196;20	0
2	2	33.9	33.9	33.9	13.757	118	118;99;78;[illegible]	0
4	5	17.2	17.2	17.2	51.57	443	443;465;48	0
6	6	22.4	22.4	22.4	66.193	606	606;245;46	0
4	4	18	18	18	43.237	394	394;429;43	0
1	2	3.9	3.9	3.9	99.365	895	895;951	0
2	1	6.6	6.6	6.6	74.89	677	677;147;18	0
1	1	11.6	11.6	1.8	68.07	610	610;322	0
1	1	4.5	4.5	4.5	36.588	334	334	0
1	1	33.3	33.3	33.3	5.4821	48	48;189;294	0.00484
1	1	7.6	7.6	7.6	36.405	328	328;385;38	0.00054

0	0	2.2	2.2	2.2	94.498	830	830;89;97	0.000531
1	0	3.6	3.6	3.6	73.918	631	631;696	0.002012
16	15	29.8	29.8	29.8	82.704	732	732;131;19	0
13	12	25.8	25.8	25.8	69.842	609	609;559;56	0
3	3	43.9	32	32	31.947	303	303;342;37	0
2	2	4.3	4.3	4.3	56.877	529	529;579;53	0.002951
2	2	6	6	6	72.531	652	652;810	0
1	1	13	13	13	18.947	162	162;426	0
4	3	7.2	7.2	7.2	114.08	1024	1024;624;6	0
1	1	8.7	8.7	8.7	34.114	310	310;53;109	0
2	2	3.1	3.1	3.1	117.01	1074	1074	0
2	2	4.3	4.3	4.3	87.563	786	786;826;15	0
2	1	28.7	28.7	28.7	9.8382	87	87;376	0
1	1	5.8	5.8	5.8	21.844	206	206;240;32	0
1	1	12.9	12.9	12.9	17	155	155;481;49	0
0	0	8	8	8	40.14	377	377	0
3	3	9.8	9.8	9.8	57.643	540	540;572;41	0

Table S2 Proteomic results

Score	Identificati	Identificati	Identificati	Identificati	Identificati	Identificati	Identificati	Identificati
38.42	By MS/MS	By MS/MS	By MS/MS	By MS/MS	By MS/MS	By MS/MS	By MS/MS	By MS/MS
23.623	By MS/MS	By MS/MS	By MS/MS	By matchin	By matchin	By MS/MS	By MS/MS	By MS/MS
6.8435	By matchin	By matchin	By matchin	By matchin	By matchin	By MS/MS	By MS/MS	By MS/MS
48.221	By MS/MS	By MS/MS	By MS/MS	By MS/MS	By MS/MS	By MS/MS	By MS/MS	By MS/MS
114.83	By MS/MS	By MS/MS	By MS/MS	By MS/MS	By MS/MS	By MS/MS	By MS/MS	By MS/MS
29.681	By matchin	By matchin	By MS/MS	By MS/MS	By MS/MS	By MS/MS	By MS/MS	By MS/MS
30.744	By MS/MS	By MS/MS	By MS/MS	By MS/MS	By MS/MS	By MS/MS	By MS/MS	By MS/MS
138.41	By MS/MS	By MS/MS	By MS/MS	By MS/MS	By MS/MS	By MS/MS	By MS/MS	By MS/MS
38.927	By MS/MS	By MS/MS	By MS/MS	By MS/MS	By MS/MS	By MS/MS	By MS/MS	By MS/MS
130.44	By MS/MS	By MS/MS	By MS/MS	By MS/MS	By MS/MS	By MS/MS	By MS/MS	By MS/MS
2.8474	By matching		By matchin	By matchin	By matchin	By matchin	By MS/MS	By MS/MS
5.2437	By matchin	By matchin	By MS/MS	By MS/MS	By MS/MS	By MS/MS	By matching	
4.3317	By matchin	By MS/MS			By matching			
70.802	By MS/MS	By MS/MS	By MS/MS	By MS/MS	By MS/MS	By MS/MS	By MS/MS	By MS/MS
7.7474	By MS/MS	By matching			By matchin	By MS/MS	By MS/MS	By MS/MS
30.939	By MS/MS	By MS/MS	By matchin	By matchin	By matchin	By MS/MS	By MS/MS	By MS/MS
15.057	By MS/MS	By MS/MS	By matchin	By MS/MS	By matchin	By MS/MS	By MS/MS	By MS/MS
16.383	By MS/MS	By MS/MS	By MS/MS	By matchin	By MS/MS	By MS/MS	By MS/MS	By MS/MS
4.6271	By matchin	By MS/MS	By matchin	By matchin	By matchin	By MS/MS	By matchin	By MS/MS
40.616	By MS/MS	By MS/MS	By MS/MS	By MS/MS	By MS/MS	By MS/MS	By MS/MS	By MS/MS
26.327	By MS/MS	By MS/MS	By MS/MS	By MS/MS	By matchin	By MS/MS	By MS/MS	By MS/MS
10.253	By matchin	By matchin	By matchin	By matchin	By matchin	By MS/MS	By MS/MS	By MS/MS
2.4303	By matchin	By matching		By matching		By matchin	By MS/MS	By MS/MS
16.444	By matchin	By MS/MS	By MS/MS	By MS/MS	By MS/MS	By MS/MS	By MS/MS	By MS/MS
11.065	By matchin	By MS/MS	By MS/MS	By MS/MS	By matchin	By MS/MS	By MS/MS	By MS/MS
16.988	By matchin	By matchin	By matchin	By matchin	By matchin	By MS/MS	By MS/MS	By MS/MS
2.0492	By MS/MS	By matchin	By matchin	By matching		By matchin	By MS/MS	By MS/MS
33.961	By MS/MS	By MS/MS	By MS/MS	By MS/MS	By MS/MS	By MS/MS	By MS/MS	By MS/MS
8.2739	By MS/MS	By MS/MS	By MS/MS	By matchin	By MS/MS	By MS/MS	By MS/MS	By MS/MS
3.047				By matching		By MS/MS		By MS/MS
9.4403					By matchin	By matchin	By MS/MS	By MS/MS
3.3453	By matchin	By matchin	By matchin	By matchin	By matchin	By matchin	By MS/MS	By MS/MS
5.3949	By matchin	By matchin	By matchin	By matchin	By MS/MS	By MS/MS	By MS/MS	By MS/MS
8.9226			By matchin	By matchin	By matchin	By matchin	By MS/MS	By MS/MS
3.9098	By MS/MS	By MS/MS	By matchin	By matchin	By MS/MS	By matchin	By matchin	By matchin
6.4657	By matchin	By MS/MS	By matchin	By matchin	By MS/MS	By matchin	By matchin	By matchin
10.205	By MS/MS	By MS/MS	By MS/MS	By MS/MS	By MS/MS	By MS/MS	By MS/MS	By MS/MS
18.459	By MS/MS	By MS/MS	By MS/MS	By MS/MS	By MS/MS	By MS/MS	By MS/MS	By MS/MS
4.5992	By MS/MS	By matchin	By MS/MS	By MS/MS	By MS/MS	By MS/MS	By matchin	By MS/MS
2.0157	By MS/MS	By MS/MS	By matching		By matchin	By matchin	By matching	
7.2971	By MS/MS	By MS/MS	By MS/MS	By MS/MS	By MS/MS	By matchin	By MS/MS	By matchin
3.3786	By matchin	By MS/MS	By MS/MS	By MS/MS	By matchin	By MS/MS	By MS/MS	By matchin
4.9234	By MS/MS	By MS/MS	By matchin	By matchin	By MS/MS	By matchin	By matchin	By matchin
7.4621	By MS/MS	By MS/MS	By MS/MS	By MS/MS	By matchin	By MS/MS		By matchin
2.353	By MS/MS		By MS/MS	By MS/MS	By matchin	By matchin	By matching	
4.4329	By matchin	By matchin	By matchin	By MS/MS	By MS/MS	By MS/MS	By matching	
2.3893	By matchin	By MS/MS	By MS/MS	By MS/MS	By matchin	By matchin	By matchin	By matchin
1.679	By matchin	By MS/MS	By matchin	By matching		By MS/MS	By matchin	By matchin

5.2893	By MS/MS	By MS/MS	By MS/MS	By MS/MS	By MS/MS	By MS/MS	By matchin	By matchin
3.5068	By matchin	By MS/MS	By matchin	By matching		By MS/MS	By matchin	By matchin
39.672	By MS/MS	By MS/MS	By MS/MS	By MS/MS	By MS/MS	By MS/MS	By MS/MS	By MS/MS
1.6032	By MS/MS	By matchin	By MS/MS	By matchin	By MS/MS	By matchin	By matchin	By matchin
8.1001	By matchin	By matchin	By MS/MS	By MS/MS	By MS/MS	By matchin	By matchin	By matchin
14.532	By MS/MS	By MS/MS	By MS/MS	By MS/MS	By MS/MS	By MS/MS	By MS/MS	By MS/MS
4.6085	By MS/MS	By MS/MS	By matchin	By matchin	By MS/MS	By MS/MS		
4.8001	By matchin	By MS/MS	By MS/MS	By MS/MS	By MS/MS	By matching		By matchin
17.582	By MS/MS		By MS/MS	By MS/MS	By MS/MS	By MS/MS	By matchin	By MS/MS
2.7637	By MS/MS	By MS/MS	By MS/MS	By matchin	By matchin	By matchin	By matchin	By matchin
4.8256	By matchin	By MS/MS	By matchin	By matchin	By MS/MS			
1.9918	By MS/MS	By matchin	By matchin	By MS/MS	By MS/MS	By matchin	By MS/MS	By MS/MS
3.7219	By MS/MS	By matchin	By matchin	By MS/MS	By matchin	By matchin	By matchin	By matchin
1.6085		By MS/MS		By matching				
4.9141	By MS/MS	By MS/MS	By MS/MS	By MS/MS	By MS/MS	By matchin	By matching	
3.696	By MS/MS	By MS/MS	By matchin	By matchin	By MS/MS	By MS/MS	By matchin	By matchin
16.076	By MS/MS	By matchin	By matchin	By matchin	By MS/MS	By matching		
3.943	By MS/MS	By MS/MS	By matchin	By matchin	By MS/MS	By matchin	By MS/MS	By matchin
3.4486	By MS/MS	By MS/MS	By MS/MS	By matching		By matchin	By matching	
3.6271	By MS/MS	By MS/MS	By MS/MS	By MS/MS	By MS/MS	By MS/MS	By matchin	By matchin
1.7948	By MS/MS	By MS/MS	By MS/MS	By matching		By matchin	By matchin	By matchin
8.8327	By MS/MS	By MS/MS	By matchin	By matchin	By MS/MS	By MS/MS	By MS/MS	By MS/MS
37.221	By MS/MS	By MS/MS	By MS/MS	By MS/MS	By MS/MS	By MS/MS	By MS/MS	By MS/MS
3.7107	By MS/MS	By MS/MS	By MS/MS	By MS/MS	By MS/MS	By matchin	By matchin	By matchin
3.2468	By MS/MS	By matchin	By MS/MS	By MS/MS	By MS/MS	By MS/MS		By MS/MS
1.5272	By matching		By MS/MS	By MS/MS	By MS/MS	By MS/MS	By matchin	By MS/MS
16.674	By MS/MS	By MS/MS	By matchin	By MS/MS		By MS/MS	By MS/MS	By MS/MS
51.215	By MS/MS	By MS/MS	By MS/MS	By MS/MS	By MS/MS	By MS/MS	By MS/MS	By MS/MS
29.813	By MS/MS	By MS/MS	By MS/MS	By MS/MS	By MS/MS	By MS/MS	By MS/MS	By MS/MS
12.258	By MS/MS	By MS/MS	By MS/MS	By MS/MS	By MS/MS	By MS/MS	By MS/MS	By MS/MS
48.667	By MS/MS	By MS/MS	By MS/MS	By MS/MS	By MS/MS	By MS/MS	By MS/MS	By MS/MS
8.7874	By MS/MS	By MS/MS	By MS/MS	By MS/MS	By MS/MS	By MS/MS	By MS/MS	By MS/MS
8.8125	By MS/MS	By MS/MS	By MS/MS	By MS/MS	By MS/MS	By MS/MS	By MS/MS	By MS/MS
29.615	By MS/MS	By MS/MS	By MS/MS	By MS/MS	By MS/MS	By MS/MS	By MS/MS	By MS/MS
13.042	By MS/MS	By MS/MS	By MS/MS	By MS/MS	By matchin	By MS/MS	By MS/MS	By MS/MS
16.204	By MS/MS	By MS/MS	By MS/MS	By MS/MS	By MS/MS	By MS/MS	By MS/MS	By MS/MS
28.609	By MS/MS	By MS/MS	By MS/MS	By MS/MS	By MS/MS	By matching	By MS/MS	By MS/MS
10.469	By MS/MS	By MS/MS	By MS/MS	By MS/MS	By MS/MS	By MS/MS	By MS/MS	By MS/MS
12.616	By matchin	By matchin	By MS/MS	By matchin	By matchin	By MS/MS	By MS/MS	By MS/MS
5.6077	By MS/MS	By MS/MS	By MS/MS	By MS/MS	By matchin	By MS/MS	By MS/MS	By MS/MS
10.52	By MS/MS	By MS/MS	By MS/MS	By MS/MS	By MS/MS	By MS/MS	By MS/MS	By MS/MS
5.585	By MS/MS	By MS/MS	By MS/MS	By MS/MS	By MS/MS	By MS/MS	By MS/MS	By MS/MS
4.8962	By MS/MS	By MS/MS	By MS/MS	By matchin	By matchin	By MS/MS	By MS/MS	By MS/MS
11.325	By MS/MS	By MS/MS	By MS/MS	By MS/MS	By MS/MS	By MS/MS	By MS/MS	By MS/MS
36.183	By MS/MS	By MS/MS	By MS/MS	By MS/MS	By MS/MS	By MS/MS	By MS/MS	By MS/MS
2.8134	By MS/MS	By matchin	By matchin	By MS/MS	By matchin	By MS/MS	By MS/MS	
34.572	By MS/MS	By MS/MS	By MS/MS	By MS/MS	By MS/MS	By MS/MS	By MS/MS	By MS/MS
2.2138	By MS/MS	By MS/MS	By MS/MS	By MS/MS	By MS/MS	By MS/MS	By MS/MS	By MS/MS
25.885	By MS/MS	By MS/MS	By MS/MS	By MS/MS	By MS/MS	By MS/MS	By MS/MS	By MS/MS
36.196	By MS/MS	By MS/MS	By MS/MS	By MS/MS	By MS/MS	By MS/MS	By MS/MS	By MS/MS

12.415	By MS/MS	By MS/MS	By MS/MS	By MS/MS	By MS/MS	By MS/MS	By MS/MS	By MS/MS
60.829	By MS/MS	By MS/MS	By MS/MS	By MS/MS	By MS/MS	By MS/MS	By MS/MS	By MS/MS
26.998	By matchin	By MS/MS	By MS/MS	By matchin	By matchin	By MS/MS	By MS/MS	By MS/MS
44.966	By MS/MS	By MS/MS	By MS/MS	By MS/MS	By MS/MS	By MS/MS	By MS/MS	By MS/MS
27.008	By MS/MS	By MS/MS	By MS/MS	By MS/MS	By MS/MS	By MS/MS	By MS/MS	By MS/MS
157.63	By MS/MS	By MS/MS	By MS/MS	By MS/MS	By MS/MS	By MS/MS	By MS/MS	By MS/MS
323.31	By MS/MS	By MS/MS	By MS/MS	By MS/MS	By MS/MS	By MS/MS	By MS/MS	By MS/MS
4.3995	By MS/MS	By matchin	By MS/MS	By matchin	By matchin	By matchin	By MS/MS	By MS/MS
3.4565	By MS/MS	By MS/MS	By MS/MS	By MS/MS	By matchin	By matchin	By MS/MS	By MS/MS
323.31	By MS/MS	By MS/MS	By MS/MS	By MS/MS	By MS/MS	By MS/MS	By MS/MS	By MS/MS
37.793	By MS/MS	By MS/MS	By MS/MS	By MS/MS	By matchin	By MS/MS	By MS/MS	By MS/MS
3.2937	By matchin	By matchin	By matchin	By MS/MS	By matchin	By matchin	By MS/MS	By matchin
24.411	By MS/MS	By MS/MS	By MS/MS	By MS/MS	By MS/MS	By MS/MS	By MS/MS	By MS/MS
95.356	By MS/MS	By MS/MS	By MS/MS	By MS/MS	By MS/MS	By MS/MS	By MS/MS	By MS/MS
13.258	By MS/MS	By MS/MS	By MS/MS	By MS/MS	By MS/MS	By MS/MS	By MS/MS	By MS/MS
14.764	By MS/MS	By MS/MS	By MS/MS	By MS/MS	By MS/MS	By MS/MS	By MS/MS	By MS/MS
1.4935	By matchin	By MS/MS	By matchin	By matchin	By MS/MS	By matchin	By matchin	By matchin
43.123	By MS/MS	By MS/MS	By MS/MS	By MS/MS	By MS/MS	By MS/MS	By MS/MS	By MS/MS
81.705	By MS/MS	By MS/MS	By MS/MS	By MS/MS	By MS/MS	By MS/MS	By MS/MS	By MS/MS
12.27	By MS/MS	By MS/MS	By MS/MS	By MS/MS	By MS/MS	By MS/MS	By MS/MS	By MS/MS
10.363	By MS/MS	By MS/MS	By MS/MS	By MS/MS	By MS/MS	By MS/MS	By MS/MS	By MS/MS
10.649	By MS/MS	By MS/MS	By MS/MS	By MS/MS	By MS/MS	By MS/MS	By MS/MS	By MS/MS
17.425	By MS/MS	By MS/MS	By MS/MS	By matchin	By matchin	By matchin	By MS/MS	By MS/MS
43.358	By MS/MS	By MS/MS	By MS/MS	By MS/MS	By MS/MS	By MS/MS	By MS/MS	By MS/MS
19.494	By MS/MS	By MS/MS	By MS/MS	By MS/MS	By MS/MS	By MS/MS	By MS/MS	By MS/MS
6.6834	By MS/MS	By MS/MS	By MS/MS	By matchin	By MS/MS	By MS/MS	By MS/MS	By MS/MS
11.46	By MS/MS	By MS/MS	By MS/MS	By MS/MS	By MS/MS	By MS/MS	By MS/MS	By MS/MS
52.004	By MS/MS	By MS/MS	By MS/MS	By MS/MS	By MS/MS	By MS/MS	By MS/MS	By MS/MS
13.034	By MS/MS	By MS/MS	By matchin	By MS/MS	By matchin	By MS/MS	By MS/MS	By MS/MS
17.908	By MS/MS	By MS/MS	By MS/MS	By MS/MS	By MS/MS	By MS/MS	By MS/MS	By MS/MS
12.66	By MS/MS	By MS/MS	By matchin	By MS/MS	By MS/MS	By MS/MS	By MS/MS	By MS/MS
20.33	By MS/MS	By MS/MS	By matchin	By MS/MS	By matchin	By MS/MS	By MS/MS	By MS/MS
8.5451	By MS/MS	By MS/MS	By MS/MS	By MS/MS	By MS/MS	By MS/MS	By MS/MS	By MS/MS
17.085	By MS/MS	By MS/MS	By MS/MS	By MS/MS	By MS/MS	By MS/MS	By MS/MS	By MS/MS
4.2955		By matchin	By MS/MS	By MS/MS	By MS/MS	By MS/MS	By MS/MS	By MS/MS
53.124	By MS/MS	By MS/MS	By MS/MS	By MS/MS	By MS/MS	By MS/MS	By MS/MS	By MS/MS
40.004	By MS/MS	By MS/MS	By MS/MS	By MS/MS	By MS/MS	By MS/MS	By MS/MS	By MS/MS
7.2025	By MS/MS	By MS/MS	By MS/MS	By matchin	By MS/MS	By MS/MS	By matchin	By MS/MS
14.247	By MS/MS	By MS/MS	By MS/MS	By MS/MS	By MS/MS	By MS/MS	By MS/MS	By MS/MS
8.5165	By MS/MS	By MS/MS	By MS/MS	By MS/MS	By MS/MS	By MS/MS	By MS/MS	By MS/MS
6.8127	By MS/MS	By MS/MS	By MS/MS	By MS/MS	By MS/MS	By matchin	By MS/MS	By MS/MS
14.164	By MS/MS	By MS/MS	By MS/MS	By MS/MS	By MS/MS	By MS/MS	By MS/MS	By MS/MS
11.263	By MS/MS	By MS/MS	By MS/MS	By MS/MS	By MS/MS	By MS/MS	By MS/MS	By MS/MS
4.672	By MS/MS	By MS/MS	By MS/MS	By MS/MS	By matchin	By MS/MS	By MS/MS	By MS/MS
6.9301	By matchin	By MS/MS	By MS/MS	By MS/MS	By MS/MS	By MS/MS	By MS/MS	By MS/MS
1.487	By matchin	By matchin	By matchin	By matchin	By matchin	By matchin	By MS/MS	By MS/MS
48.454	By MS/MS	By MS/MS	By MS/MS	By MS/MS	By MS/MS	By MS/MS	By MS/MS	By MS/MS
25.191	By MS/MS	By MS/MS	By MS/MS	By MS/MS	By MS/MS	By MS/MS	By MS/MS	By MS/MS
38.679	By MS/MS	By MS/MS	By MS/MS	By MS/MS	By MS/MS	By MS/MS	By MS/MS	By MS/MS
65.189	By MS/MS	By MS/MS	By MS/MS	By MS/MS	By MS/MS	By MS/MS	By MS/MS	By MS/MS

50.31	By MS/MS	By MS/MS	By MS/MS	By MS/MS	By MS/MS	By MS/MS	By MS/MS	By MS/MS
1.7519	By MS/MS	By MS/MS	By MS/MS	By MS/MS	By MS/MS	By matchin	By MS/MS	By matchin
60.251	By MS/MS	By MS/MS	By MS/MS	By MS/MS	By MS/MS	By MS/MS	By MS/MS	By MS/MS
20.285	By MS/MS	By MS/MS	By MS/MS	By MS/MS	By MS/MS	By MS/MS	By MS/MS	By MS/MS
2.0816		By matching				By matchin	By MS/MS	By MS/MS
40.875	By matchin	By MS/MS	By MS/MS	By MS/MS	By MS/MS	By MS/MS	By MS/MS	By MS/MS
2.0764				By matchin	By matchin	By MS/MS	By MS/MS	By MS/MS
4.6498		By matchin	By matchin	By matchin	By matchin	By matchin	By matchin	By MS/MS
192.33	By MS/MS	By MS/MS	By MS/MS	By MS/MS	By MS/MS	By MS/MS	By MS/MS	By MS/MS
39.341	By MS/MS	By MS/MS	By MS/MS	By MS/MS	By MS/MS	By MS/MS	By MS/MS	By MS/MS
2.0215	By matching					By MS/MS	By matchin	By MS/MS
2.9689	By MS/MS	By matchin	By MS/MS	By matchin	By matchin	By matchin	By matchin	By MS/MS
2.1036		By matchin	By matching		By MS/MS		By matching	
3.7378	By matchin	By matchin	By matching		By matchin	By MS/MS	By MS/MS	By matchin
18.896	By MS/MS	By MS/MS	By MS/MS	By MS/MS	By MS/MS	By MS/MS	By MS/MS	By MS/MS
2.5668	By MS/MS	By MS/MS	By matching		By MS/MS	By MS/MS	By matchin	By MS/MS
20.158	By matching		By matchin	By matching		By MS/MS	By MS/MS	By MS/MS
2.29	By matching		By matchin	By matching		By matchin	By matchin	By MS/MS
25.916	By MS/MS	By MS/MS	By MS/MS	By MS/MS	By MS/MS	By MS/MS	By MS/MS	By MS/MS
1.5821	By matchin	By MS/MS	By matchin	By matchin	By matchin	By matchin	By MS/MS	By matchin
323.31	By MS/MS	By MS/MS	By MS/MS	By MS/MS	By MS/MS	By MS/MS	By MS/MS	By MS/MS
21.441	By MS/MS	By MS/MS	By MS/MS	By MS/MS	By MS/MS	By MS/MS	By MS/MS	By MS/MS
22.281	By MS/MS	By MS/MS	By MS/MS	By MS/MS	By MS/MS	By MS/MS	By MS/MS	By MS/MS
13.148		By MS/MS	By MS/MS	By MS/MS	By MS/MS	By MS/MS	By MS/MS	By MS/MS
11.475	By matchin	By matchin	By MS/MS	By MS/MS	By MS/MS	By MS/MS	By MS/MS	By MS/MS
6.8047	By matchin	By matchin	By matchin	By matchin	By matchin	By MS/MS	By MS/MS	By MS/MS
1.824							By MS/MS	
6.4786	By MS/MS	By matchin	By matchin	By MS/MS	By MS/MS	By MS/MS	By MS/MS	By MS/MS
4.7582	By matchin	By matchin	By matchin	By matchin	By matchin	By MS/MS	By matchin	By MS/MS
3.4492		By matchin	By matchin	By matching		By matchin	By matchin	By MS/MS
27.113	By MS/MS	By MS/MS	By MS/MS	By MS/MS	By MS/MS	By MS/MS	By MS/MS	By MS/MS
10.266	By MS/MS	By MS/MS	By MS/MS	By MS/MS	By MS/MS	By MS/MS	By MS/MS	By MS/MS
121.87	By matchin	By matchin	By MS/MS	By MS/MS	By matchin	By MS/MS	By MS/MS	By MS/MS
2.0913					By MS/MS			
2.7622	By matchin	By MS/MS	By MS/MS	By matchin	By matchin	By matchin	By matchin	By matchin
2.4428	By matchin	By matchin	By matchin	By matchin	By matchin	By matchin	By MS/MS	By matchin
20.064	By MS/MS	By MS/MS	By MS/MS	By MS/MS	By MS/MS	By MS/MS	By MS/MS	By MS/MS
7.3967	By MS/MS	By MS/MS	By MS/MS	By MS/MS	By MS/MS	By MS/MS	By MS/MS	By MS/MS
25.322	By matchin	By matchin	By MS/MS	By matchin	By matchin	By MS/MS	By MS/MS	By MS/MS
2.5579	By MS/MS	By MS/MS	By MS/MS	By MS/MS	By MS/MS	By MS/MS		
3.6049	By MS/MS	By matchin	By MS/MS	By MS/MS	By MS/MS	By matchin	By matchin	By matchin
17.72	By MS/MS	By MS/MS	By MS/MS	By MS/MS	By MS/MS	By matchin	By MS/MS	By MS/MS
4.0095	By MS/MS	By MS/MS	By matchin	By MS/MS	By matchin	By matchin	By MS/MS	By MS/MS
2.4257	By matchin	By matchin	By matchin	By matchin	By matchin	By MS/MS	By MS/MS	By MS/MS
1.9235	By matchin	By MS/MS	By matching			By matching		By matchin
6.5567	By matchin	By MS/MS	By matching		By matchin	By MS/MS	By MS/MS	By MS/MS
5.2895	By MS/MS	By MS/MS	By MS/MS		By MS/MS	By MS/MS	By MS/MS	By matchin
6.2686	By matching					By MS/MS	By MS/MS	By MS/MS
41.902	By MS/MS	By MS/MS	By MS/MS	By MS/MS	By matchin	By MS/MS	By MS/MS	By MS/MS
12.748	By MS/MS	By MS/MS	By MS/MS	By matchin	By MS/MS	By MS/MS	By MS/MS	By MS/MS

3.6602	By matchin	By matchin	By MS/MS	By MS/MS	By MS/MS	By matchin	By MS/MS	
8.7763	By matchin	By matchin	By matchin	By MS/MS	By MS/MS	By MS/MS	By MS/MS	By MS/MS
1.8129					By matchin	By matchin	By matchin	By MS/MS
13.265			By matchin	By matching		By MS/MS	By MS/MS	By MS/MS
39.587	By matchin	By MS/MS	By MS/MS	By MS/MS	By MS/MS	By MS/MS	By MS/MS	By MS/MS
20.255	By MS/MS	By MS/MS	By MS/MS	By MS/MS	By MS/MS	By MS/MS	By MS/MS	By MS/MS
2.6228				By matching		By MS/MS	By matchin	By MS/MS
40.15	By MS/MS	By MS/MS	By MS/MS	By MS/MS	By MS/MS	By MS/MS	By MS/MS	By MS/MS
12.677	By MS/MS	By MS/MS	By MS/MS	By MS/MS	By MS/MS	By MS/MS	By MS/MS	By MS/MS
8.2524	By MS/MS	By MS/MS	By MS/MS	By MS/MS	By MS/MS	By MS/MS	By matchin	By MS/MS
27.271	By MS/MS	By MS/MS	By MS/MS	By MS/MS	By MS/MS	By MS/MS	By MS/MS	By MS/MS
2.0108	By matchin	By MS/MS	By MS/MS	By matchin	By matchin	By MS/MS	By MS/MS	By MS/MS
2.3135	By matchin	By matchin	By matchin	By matchin	By MS/MS	By MS/MS	By matchin	By MS/MS
6.4727	By MS/MS	By matchin	By matchin	By matchin	By matchin	By matchin	By MS/MS	By MS/MS
2.799	By MS/MS	By MS/MS	By MS/MS	By MS/MS	By MS/MS	By matchin	By MS/MS	By MS/MS
9.6776			By matchin	By matchin	By MS/MS	By MS/MS	By MS/MS	By MS/MS
2.5071	By matchin	By MS/MS	By matchin	By MS/MS	By MS/MS	By matchin	By matchin	By matchin
8.3743	By MS/MS	By MS/MS	By MS/MS	By MS/MS	By MS/MS	By matchin	By matchin	By matchin
5.3882		By matchin	By matchin	By matchin	By MS/MS	By MS/MS	By MS/MS	By MS/MS
-2			By MS/MS			By MS/MS		By MS/MS
4.2614	By MS/MS	By MS/MS	By MS/MS	By MS/MS		By matchin	By matchin	By matchin
22.492	By MS/MS	By MS/MS	By MS/MS	By MS/MS	By MS/MS	By MS/MS	By MS/MS	By MS/MS
8.0075			By matchin	By matchin	By MS/MS	By MS/MS	By MS/MS	By MS/MS
6.9989	By matchin	By matchin	By matchin	By MS/MS	By matchin	By MS/MS	By MS/MS	By MS/MS
61.435	By MS/MS	By MS/MS	By MS/MS	By MS/MS	By MS/MS	By MS/MS	By MS/MS	By MS/MS
5.8168	By MS/MS	By MS/MS	By matchin	By MS/MS	By matchin	By MS/MS	By matchin	By matchin
34.828	By MS/MS	By MS/MS	By MS/MS	By MS/MS	By matchin	By MS/MS	By MS/MS	By MS/MS
6.7473	By matchin	By matchin	By MS/MS	By matchin	By matchin	By MS/MS	By MS/MS	By MS/MS
9.756	By matchin	By matchin	By matchin	By matchin	By matchin	By matchin	By MS/MS	By MS/MS
7.1727	By matchin	By matchin	By matchin	By matchin	By matchin	By MS/MS	By MS/MS	By MS/MS
32.071	By MS/MS	By MS/MS	By MS/MS	By MS/MS	By MS/MS	By MS/MS	By MS/MS	By MS/MS
1.9546	By MS/MS	By matchin	By matchin	By matching				
2.8036				By matchin	By matchin	By MS/MS	By MS/MS	By matchin
3.6276	By MS/MS	By MS/MS	By matchin	By matchin	By matchin	By MS/MS	By matching	
153.22	By MS/MS	By MS/MS	By MS/MS	By MS/MS	By MS/MS	By MS/MS	By MS/MS	By MS/MS
84.772	By MS/MS	By MS/MS	By MS/MS	By MS/MS	By MS/MS	By MS/MS	By MS/MS	By MS/MS
14.192	By MS/MS	By MS/MS	By MS/MS	By MS/MS	By MS/MS	By MS/MS	By MS/MS	By MS/MS
4.0822	By matchin	By MS/MS			By MS/MS	By MS/MS	By MS/MS	By matchin
1.9213			By matching					By MS/MS
226.05	By MS/MS	By MS/MS	By MS/MS	By MS/MS	By MS/MS	By MS/MS	By MS/MS	By MS/MS
5.1307	By MS/MS	By MS/MS	By MS/MS	By MS/MS	By MS/MS	By matching		By matchin
1.6454					By MS/MS			By matchin
12.541	By MS/MS	By MS/MS	By MS/MS	By MS/MS	By MS/MS	By MS/MS	By MS/MS	By matchin
2.7093	By matchin	By matching		By matchin	By matchin	By MS/MS	By MS/MS	By MS/MS
12.25	By MS/MS	By MS/MS	By MS/MS	By MS/MS	By MS/MS	By MS/MS	By matchin	By MS/MS
12.212	By MS/MS	By MS/MS	By MS/MS	By MS/MS	By MS/MS	By MS/MS	By MS/MS	By MS/MS
29.252	By MS/MS	By MS/MS	By MS/MS	By MS/MS	By MS/MS	By MS/MS	By MS/MS	By MS/MS
11.996	By matchin	By MS/MS	By matchin	By matchin	By MS/MS	By MS/MS	By MS/MS	By MS/MS
5.74	By MS/MS	By MS/MS	By MS/MS	By MS/MS	By MS/MS	By MS/MS	By matchin	By MS/MS
26.192	By MS/MS	By MS/MS	By MS/MS	By MS/MS	By MS/MS	By MS/MS	By MS/MS	By MS/MS

21.252	By MS/MS	By MS/MS	By MS/MS	By MS/MS	By MS/MS	By MS/MS	By MS/MS	By MS/MS
2.8507	By MS/MS		By matchin	By MS/MS	By MS/MS			By matchin
1.9953	By matchin	By matchin	By matching				By matchin	By MS/MS
176.4	By MS/MS	By MS/MS	By MS/MS	By MS/MS	By MS/MS	By MS/MS	By MS/MS	By MS/MS
11.854	By matchin	By matchin	By matching			By matchin	By MS/MS	By MS/MS
27.818	By MS/MS	By matchin	By matchin	By MS/MS	By MS/MS	By MS/MS	By MS/MS	By MS/MS
323.31	By MS/MS	By MS/MS	By MS/MS	By MS/MS	By MS/MS	By MS/MS	By MS/MS	By MS/MS
28.223	By MS/MS	By MS/MS	By MS/MS	By MS/MS	By MS/MS	By MS/MS	By MS/MS	By MS/MS
69.753	By MS/MS	By MS/MS	By MS/MS	By MS/MS	By MS/MS	By MS/MS	By MS/MS	By MS/MS
108.65	By MS/MS	By MS/MS	By MS/MS	By MS/MS	By MS/MS	By MS/MS	By MS/MS	By MS/MS
34.689	By MS/MS	By MS/MS	By MS/MS	By MS/MS	By MS/MS	By MS/MS	By MS/MS	By MS/MS
49.662	By MS/MS	By MS/MS	By MS/MS	By MS/MS	By MS/MS	By MS/MS	By MS/MS	By MS/MS
35.827	By matchin	By MS/MS	By MS/MS	By MS/MS	By MS/MS	By MS/MS	By MS/MS	By MS/MS
4.6592	By MS/MS	By MS/MS	By MS/MS	By MS/MS	By MS/MS	By MS/MS	By matchin	By MS/MS
11.894	By matchin	By matchin	By MS/MS	By matchin	By matchin	By MS/MS	By MS/MS	By MS/MS
2.9295	By matching		By matchin	By matchin	By matchin	By MS/MS	By MS/MS	By matchin
5.5914		By matchin	By matchin	By MS/MS	By matchin	By matchin	By matchin	By MS/MS
37.334	By matchin	By matchin	By matchin	By matchin	By matchin	By matchin	By MS/MS	By MS/MS
6.8503	By matchin	By matchin	By MS/MS	By matchin	By MS/MS	By MS/MS	By matchin	By matchin
1.9225		By matching				By matching		By MS/MS
2.2008		By matching				By matchin	By matchin	By MS/MS
1.7812	By matchin	By MS/MS	By matchin	By matching		By matchin	By MS/MS	By matchin
9.7386	By matchin	By MS/MS	By MS/MS	By MS/MS	By MS/MS	By MS/MS	By MS/MS	By MS/MS
3.7777	By MS/MS	By MS/MS	By matchin	By matching		By MS/MS	By matchin	By MS/MS
4.8593	By MS/MS	By matchin	By MS/MS	By MS/MS	By MS/MS	By MS/MS	By MS/MS	By MS/MS
2.94	By matching		By matchin	By matchin	By MS/MS		By matching	
7.4365			By matching			By MS/MS	By MS/MS	By MS/MS
6.0598	By matchin	By MS/MS	By MS/MS	By matchin	By MS/MS	By MS/MS	By MS/MS	By MS/MS
38.759	By MS/MS	By MS/MS	By MS/MS	By MS/MS	By MS/MS	By MS/MS	By MS/MS	By MS/MS
30.138	By matchin	By MS/MS	By MS/MS	By MS/MS	By MS/MS	By MS/MS	By MS/MS	By MS/MS
1.5034				By matchin	By MS/MS	By matchin	By matchin	By matchin
12.911	By MS/MS	By MS/MS	By matchin	By matchin	By matchin	By MS/MS	By MS/MS	By MS/MS
9.5108	By MS/MS	By MS/MS	By MS/MS	By MS/MS	By MS/MS	By MS/MS	By MS/MS	By MS/MS
20.195	By MS/MS	By matchin	By MS/MS	By MS/MS	By MS/MS	By MS/MS	By MS/MS	By MS/MS
13.159			By matchin	By matchin	By matchin	By MS/MS	By MS/MS	By MS/MS
46.884	By MS/MS	By MS/MS	By MS/MS	By MS/MS	By MS/MS	By MS/MS	By MS/MS	By MS/MS
49.775	By MS/MS	By MS/MS	By MS/MS	By MS/MS	By MS/MS	By MS/MS	By MS/MS	By MS/MS
3.5746	By MS/MS	By MS/MS	By MS/MS		By matching		By matchin	By matchin
177.61	By MS/MS	By MS/MS	By MS/MS	By MS/MS	By MS/MS	By MS/MS	By MS/MS	By MS/MS
2.0104	By matchin	By matchin	By matchin	By matchin	By matchin	By MS/MS	By MS/MS	By MS/MS
2.1051	By matching		By matchin	By matchin	By matchin	By MS/MS	By MS/MS	
7.1346	By MS/MS	By MS/MS	By MS/MS	By MS/MS	By MS/MS	By MS/MS	By matchin	By MS/MS
4.0736	By MS/MS	By MS/MS		By matchin	By matchin	By matchin	By matchin	By matchin
163.31	By MS/MS	By MS/MS	By MS/MS	By MS/MS	By MS/MS	By MS/MS	By MS/MS	By MS/MS
2.1057	By MS/MS	By MS/MS	By MS/MS	By MS/MS	By matchin	By matchin	By MS/MS	By MS/MS
177.32	By MS/MS	By MS/MS	By MS/MS	By MS/MS	By MS/MS	By MS/MS	By MS/MS	By MS/MS
259.5	By MS/MS	By MS/MS	By MS/MS	By MS/MS	By MS/MS	By MS/MS	By MS/MS	By MS/MS
25.319	By MS/MS	By MS/MS	By MS/MS	By MS/MS	By MS/MS	By MS/MS	By MS/MS	By MS/MS
10.096	By MS/MS	By MS/MS	By MS/MS	By MS/MS	By MS/MS	By MS/MS	By MS/MS	By MS/MS
3.916	By MS/MS	By MS/MS	By MS/MS	By MS/MS	By MS/MS	By matchin	By matchin	By MS/MS

3.6097	By matchin	By MS/MS	By MS/MS	By MS/MS		By MS/MS	By MS/MS	
7.0971	By MS/MS	By MS/MS	By MS/MS	By MS/MS	By MS/MS	By MS/MS	By matchin	By MS/MS
26.352	By MS/MS	By MS/MS	By MS/MS	By MS/MS	By MS/MS	By MS/MS	By MS/MS	By MS/MS
16.847	By MS/MS	By MS/MS	By MS/MS	By MS/MS	By MS/MS	By MS/MS	By MS/MS	By MS/MS
52.891	By MS/MS	By MS/MS	By MS/MS	By MS/MS	By MS/MS	By MS/MS	By MS/MS	By matchin
10.322	By MS/MS	By MS/MS	By MS/MS	By MS/MS	By MS/MS	By MS/MS	By matchin	By matchin
1.5923	By matchin	By MS/MS	By matchin	By MS/MS	By matching			By matchin
5.1374	By MS/MS	By MS/MS	By MS/MS	By MS/MS	By MS/MS	By matchin	By MS/MS	By matchin
9.4189	By matchin	By matchin	By matchin	By matchin	By matchin	By MS/MS	By MS/MS	By MS/MS
5.9619	By matchin	By MS/MS		By matching		By MS/MS	By MS/MS	By MS/MS
2.4845	By matchin	By MS/MS	By matchin	By matching		By MS/MS	By matchin	By matchin
2.6799	By MS/MS	By matching			By matchin	By matchin	By matchin	By matchin
54.604	By matchin	By matchin	By MS/MS	By MS/MS	By MS/MS	By MS/MS	By MS/MS	By MS/MS
10.994	By MS/MS	By MS/MS	By MS/MS	By MS/MS	By matchin	By MS/MS	By MS/MS	By MS/MS
7.9002	By MS/MS	By MS/MS			By MS/MS	By matchin	By MS/MS	By MS/MS
59.063	By MS/MS	By MS/MS	By MS/MS	By MS/MS	By MS/MS	By MS/MS	By MS/MS	By MS/MS
7.883	By MS/MS	By MS/MS	By MS/MS	By MS/MS	By MS/MS	By matchin	By matchin	By matchin
6.9265	By MS/MS	By matchin	By MS/MS	By matchin	By MS/MS	By matchin	By matching	
4.6663	By matchin	By matchin	By matchin	By matching		By MS/MS	By MS/MS	By MS/MS
120.28	By MS/MS	By MS/MS	By MS/MS	By MS/MS	By MS/MS	By MS/MS	By MS/MS	By MS/MS
21.553	By matchin	By MS/MS	By MS/MS	By matchin	By MS/MS	By MS/MS	By MS/MS	By MS/MS
21.357	By matchin	By MS/MS	By MS/MS	By MS/MS	By MS/MS	By MS/MS	By MS/MS	By MS/MS
3.5902	By MS/MS	By MS/MS		By MS/MS	By MS/MS	By MS/MS	By MS/MS	By matchin
20.083	By matchin	By matchin	By MS/MS	By MS/MS	By MS/MS	By MS/MS	By MS/MS	By matchin
3.6875	By MS/MS	By MS/MS	By matchin	By matching		By matchin	By MS/MS	By MS/MS
47.597	By MS/MS	By MS/MS	By MS/MS	By MS/MS	By MS/MS	By MS/MS	By MS/MS	By MS/MS
2.1569							By matchin	By MS/MS
25.374	By matchin	By MS/MS	By MS/MS	By MS/MS	By MS/MS	By matchin	By MS/MS	By MS/MS
3.5951	By matchin	By matchin	By MS/MS	By matchin	By MS/MS	By matchin	By matchin	By matchin
2.428			By matchin	By matchin	By MS/MS		By matching	
3.6014	By matching		By matchin	By MS/MS		By matchin	By matchin	By MS/MS
7.4522	By MS/MS	By matchin	By MS/MS	By matchin	By MS/MS	By MS/MS	By MS/MS	By MS/MS
2.8262	By matchin	By matching				By MS/MS	By MS/MS	By matchin
49.334	By matchin	By MS/MS	By MS/MS	By MS/MS	By matchin	By MS/MS	By MS/MS	By MS/MS
1.8929		By MS/MS						
4.8177	By matchin	By matchin	By matchin	By matching		By MS/MS	By MS/MS	By MS/MS
4.2881			By matchin	By matchin	By matchin	By MS/MS	By MS/MS	By MS/MS
27.505	By MS/MS	By MS/MS	By MS/MS	By MS/MS	By MS/MS	By MS/MS	By MS/MS	By MS/MS
11.213	By MS/MS	By MS/MS	By MS/MS	By MS/MS	By MS/MS	By MS/MS	By MS/MS	By MS/MS
4.4679	By MS/MS	By matching		By matchin	By matching		By matchin	By matchin
7.6	By MS/MS	By MS/MS	By MS/MS	By MS/MS	By MS/MS	By MS/MS	By matchin	By MS/MS
7.8651	By matchin	By matchin	By MS/MS	By MS/MS	By MS/MS	By MS/MS	By matchin	By MS/MS
20.666	By MS/MS	By MS/MS	By MS/MS	By MS/MS	By MS/MS	By MS/MS	By MS/MS	By MS/MS
2.9351	By matchin	By matchin	By MS/MS	By MS/MS	By MS/MS	By matchin	By matchin	By matchin
2.9716		By matching					By MS/MS	By matchin
2.6686	By matchin	By MS/MS	By matchin	By MS/MS	By matchin	By matchin	By matchin	By MS/MS
6.0796			By MS/MS		By MS/MS	By MS/MS	By MS/MS	By MS/MS
4.423	By matchin	By matchin	By matchin	By matchin	By matchin	By matchin	By MS/MS	By matchin
65.183	By MS/MS	By MS/MS	By MS/MS	By MS/MS	By MS/MS	By MS/MS	By MS/MS	By MS/MS
2.7269	By MS/MS	By matchin	By matching		By MS/MS	By matchin	By matching	

39.604	By MS/MS	By matchin	By matchin	By matchin	By matchin	By MS/MS	By MS/MS	By MS/MS
3.0564	By matchin	By matchin	By matchin	By MS/MS	By matchin	By matching		By matchin
5.9187	By matching		By matchin	By MS/MS	By MS/MS	By MS/MS	By MS/MS	By matchin
2.9735	By matchin	By MS/MS	By MS/MS	By MS/MS	By MS/MS	By MS/MS	By MS/MS	By MS/MS
10.017	By MS/MS	By MS/MS	By MS/MS	By MS/MS	By MS/MS	By MS/MS	By MS/MS	By MS/MS
6.2915							By matchin	By MS/MS
10.953	By matchin	By matchin	By matching			By matchin	By MS/MS	By MS/MS
15.138	By matchin	By MS/MS	By MS/MS	By MS/MS	By matchin	By MS/MS	By matchin	By MS/MS
2.4503		By matching					By MS/MS	By MS/MS
5.5275	By matching			By matchin	By matchin	By MS/MS	By MS/MS	By MS/MS
1.6015	By matchin	By MS/MS		By matching		By matchin	By matching	
18.562	By matchin	By matchin	By MS/MS	By matchin	By matchin	By MS/MS	By MS/MS	By MS/MS
36.305	By MS/MS	By MS/MS	By MS/MS	By MS/MS	By MS/MS	By MS/MS	By MS/MS	By MS/MS
25.164	By MS/MS	By MS/MS	By MS/MS	By MS/MS	By MS/MS	By MS/MS	By MS/MS	By matchin
1.533	By matchin	By matchin	By matchin	By matchin	By MS/MS	By matchin	By matchin	By matchin
78.709	By MS/MS	By MS/MS	By MS/MS	By MS/MS	By MS/MS	By MS/MS	By MS/MS	By MS/MS
4.7801	By matching		By MS/MS	By matchin	By MS/MS	By MS/MS	By MS/MS	By MS/MS
20.006	By MS/MS	By MS/MS	By MS/MS	By MS/MS	By MS/MS	By MS/MS	By matchin	By MS/MS
8.5808	By MS/MS	By matchin	By MS/MS	By MS/MS	By MS/MS	By MS/MS	By MS/MS	By MS/MS
1.5402	By matching			By MS/MS	By matchin	By matchin	By matching	
68.819	By MS/MS	By MS/MS	By MS/MS	By MS/MS	By MS/MS	By MS/MS	By MS/MS	By MS/MS
47.782	By MS/MS	By MS/MS	By MS/MS	By MS/MS	By MS/MS	By MS/MS	By MS/MS	By MS/MS
33.781	By MS/MS	By MS/MS	By MS/MS	By MS/MS	By MS/MS	By MS/MS	By MS/MS	By MS/MS
21.538	By matchin	By matchin	By MS/MS	By MS/MS	By matchin	By MS/MS	By MS/MS	By MS/MS
139.18	By MS/MS	By MS/MS	By MS/MS	By MS/MS	By MS/MS	By MS/MS	By MS/MS	By MS/MS
27.984	By MS/MS	By MS/MS	By MS/MS	By MS/MS	By MS/MS	By MS/MS	By MS/MS	By MS/MS
27.071	By MS/MS	By matchin	By MS/MS	By MS/MS	By MS/MS	By MS/MS	By MS/MS	By MS/MS
13.861	By MS/MS	By matchin	By matchin	By matchin	By matchin	By MS/MS	By MS/MS	By MS/MS
13.497				By matchin	By matchin	By MS/MS	By MS/MS	By MS/MS
22.246	By matchin	By matchin	By matchin	By matchin	By MS/MS	By MS/MS	By MS/MS	By MS/MS
200.66	By MS/MS	By MS/MS	By MS/MS	By MS/MS	By MS/MS	By MS/MS	By MS/MS	By MS/MS
5.1198	By matching		By matchin	By MS/MS	By MS/MS	By MS/MS	By matchin	By MS/MS
25.547	By MS/MS	By matchin	By MS/MS	By MS/MS	By MS/MS	By MS/MS	By MS/MS	By MS/MS
3.0634	By matchin	By MS/MS	By matchin	By matchin	By matchin	By matchin	By MS/MS	By matchin
5.3555	By matchin	By matchin	By MS/MS	By MS/MS	By matchin	By MS/MS	By MS/MS	By MS/MS
11.508	By matchin	By matchin	By matchin	By matchin	By MS/MS	By MS/MS	By MS/MS	By MS/MS
48.785	By matchin	By matchin	By matchin	By MS/MS	By MS/MS	By MS/MS	By MS/MS	By MS/MS
24.419	By MS/MS	By MS/MS	By MS/MS	By MS/MS	By MS/MS	By MS/MS	By matchin	By MS/MS
22.866	By MS/MS	By MS/MS	By MS/MS	By MS/MS	By MS/MS	By MS/MS	By MS/MS	By MS/MS
8.4681	By MS/MS	By MS/MS	By MS/MS	By MS/MS	By MS/MS	By MS/MS	By matchin	By MS/MS
4.6479	By MS/MS	By MS/MS	By MS/MS	By MS/MS	By matchin	By MS/MS	By matchin	By MS/MS
9.7178	By MS/MS	By MS/MS	By matching					By MS/MS
3.6583	By MS/MS	By MS/MS	By matchin	By MS/MS		By matchin	By MS/MS	By MS/MS
1.8506	By matchin	By matchin	By MS/MS	By matchin	By matchin	By matchin	By MS/MS	By matchin
1.7798	By MS/MS	By matching		By matchin	By matchin	By matchin	By MS/MS	
26.269	By MS/MS	By MS/MS	By MS/MS	By MS/MS	By MS/MS	By MS/MS	By MS/MS	By MS/MS
21.323	By MS/MS	By MS/MS	By matchin	By matching		By MS/MS	By MS/MS	By MS/MS
91.263	By MS/MS	By MS/MS	By MS/MS	By MS/MS	By MS/MS	By MS/MS	By MS/MS	By MS/MS
13.572	By matchin	By MS/MS	By MS/MS	By MS/MS	By MS/MS	By MS/MS	By MS/MS	By MS/MS
37.282	By MS/MS	By MS/MS	By MS/MS	By MS/MS	By MS/MS	By MS/MS	By MS/MS	By MS/MS

11.27	By matchin	By MS/MS	By MS/MS	By MS/MS	By MS/MS	By MS/MS	By MS/MS	By MS/MS
8.3743	By MS/MS	By MS/MS	By MS/MS	By MS/MS	By MS/MS	By MS/MS	By MS/MS	By MS/MS
16.749	By MS/MS	By matchin	By MS/MS	By MS/MS	By MS/MS	By MS/MS	By MS/MS	By MS/MS
29.569	By MS/MS	By MS/MS	By MS/MS	By MS/MS	By MS/MS	By MS/MS	By MS/MS	By MS/MS
3.9229	By matchin	By MS/MS	By MS/MS	By MS/MS	By MS/MS	By matchin	By matchin	By matchin
5.4776	By MS/MS	By MS/MS	By MS/MS	By MS/MS	By MS/MS	By matching		By matchin
28.565	By MS/MS	By MS/MS	By MS/MS	By MS/MS	By MS/MS	By MS/MS	By MS/MS	By MS/MS
15.954	By matchin	By MS/MS	By MS/MS	By MS/MS	By MS/MS	By MS/MS	By MS/MS	By matchin
13.955	By matchin	By MS/MS	By MS/MS	By MS/MS	By MS/MS	By MS/MS	By MS/MS	By MS/MS
7.0153	By MS/MS	By MS/MS	By MS/MS	By MS/MS	By matchin	By MS/MS	By MS/MS	By MS/MS
4.2297	By matchin	By matchin	By matchin	By matchin	By MS/MS	By MS/MS	By matchin	By MS/MS
11.353	By MS/MS	By MS/MS	By MS/MS	By matchin	By matchin	By MS/MS	By matchin	By MS/MS
4.1608	By MS/MS	By MS/MS	By MS/MS	By MS/MS		By MS/MS	By matchin	By MS/MS
3.7407	By MS/MS	By matchin	By matchin	By matchin	By MS/MS	By matchin	By matchin	By matchin
8.4256	By MS/MS	By MS/MS	By matchin	By matching		By MS/MS	By matchin	By MS/MS
7.0463	By matchin	By MS/MS	By MS/MS	By MS/MS	By matchin	By MS/MS	By MS/MS	By MS/MS
8.0887	By matchin	By matching		By matchin	By matchin	By MS/MS	By MS/MS	By MS/MS
7.555	By matchin	By matchin	By MS/MS	By matchin	By matchin	By matchin	By matchin	By MS/MS
17.035	By matchin	By matchin	By MS/MS	By MS/MS	By MS/MS	By MS/MS	By MS/MS	By MS/MS
-2							By MS/MS	
4.0142	By MS/MS	By MS/MS	By MS/MS	By MS/MS	By MS/MS	By matchin	By matchin	By matchin
11.951	By MS/MS	By MS/MS	By MS/MS	By MS/MS	By MS/MS	By matchin	By MS/MS	By matchin
1.7936	By matchin	By MS/MS	By matchin	By matching		By matchin	By matchin	By matchin
20.824	By MS/MS	By MS/MS	By MS/MS	By MS/MS	By MS/MS	By MS/MS	By MS/MS	By MS/MS
43.737	By MS/MS	By MS/MS	By MS/MS	By MS/MS	By MS/MS	By MS/MS	By MS/MS	By MS/MS
7.666	By MS/MS	By MS/MS	By MS/MS	By MS/MS	By MS/MS	By MS/MS	By MS/MS	By MS/MS
2.6418				By MS/MS	By MS/MS		By matchin	By matchin
2.3914	By matchin	By MS/MS	By MS/MS	By MS/MS		By MS/MS	By MS/MS	
2.427	By MS/MS	By MS/MS	By matchin	By matching		By matching		By matchin
3.0718	By matchin	By MS/MS	By MS/MS	By MS/MS	By MS/MS	By MS/MS	By MS/MS	By MS/MS
22.781	By MS/MS	By MS/MS	By MS/MS	By MS/MS	By MS/MS	By MS/MS	By MS/MS	By MS/MS
4.4048	By matchin	By matchin	By matchin	By matchin	By MS/MS	By matchin	By matchin	By MS/MS
29.349	By MS/MS	By MS/MS	By MS/MS	By MS/MS	By MS/MS	By MS/MS	By MS/MS	By MS/MS
235.77	By MS/MS	By MS/MS	By MS/MS	By MS/MS	By MS/MS	By MS/MS	By MS/MS	By MS/MS
28.302	By MS/MS	By matchin	By MS/MS	By MS/MS	By MS/MS	By MS/MS	By MS/MS	By MS/MS
2.154	By matchin	By matchin	By matchin	By matchin	By matchin	By MS/MS	By MS/MS	By MS/MS
10.2	By MS/MS	By MS/MS	By MS/MS	By matchin	By matchin	By matchin	By matchin	By matchin
35.945	By MS/MS	By MS/MS	By MS/MS	By MS/MS	By matchin	By MS/MS	By MS/MS	By MS/MS
6.8205	By matchin	By matchin	By MS/MS	By MS/MS	By MS/MS	By MS/MS	By matchin	By matchin
6.9121	By matchin	By MS/MS	By MS/MS	By MS/MS	By MS/MS	By MS/MS	By MS/MS	By MS/MS
12.158	By matchin	By MS/MS	By MS/MS	By MS/MS		By MS/MS	By MS/MS	By MS/MS
12.174	By matchin	By matchin	By MS/MS	By matchin	By matchin	By MS/MS	By MS/MS	By MS/MS
2.0557		By MS/MS					By matchin	By MS/MS
2.4262		By matchin	By matchin	By matchin	By matchin	By MS/MS	By MS/MS	By MS/MS
13.3	By MS/MS	By MS/MS	By MS/MS	By MS/MS	By MS/MS	By MS/MS	By MS/MS	By MS/MS
3.4556	By matchin	By MS/MS	By MS/MS	By matchin	By MS/MS	By MS/MS	By matchin	By matchin
3.4421		By matching		By matching			By matchin	By MS/MS
43.071	By MS/MS	By matchin	By MS/MS	By MS/MS	By MS/MS	By MS/MS	By MS/MS	By MS/MS
1.815	By matchin	By MS/MS	By matchin	By matchin	By matching			
20.96	By MS/MS	By MS/MS	By matchin	By MS/MS	By matchin	By MS/MS	By MS/MS	By MS/MS

6.0634	By MS/MS	By matchin	By matchin	By matchin	By MS/MS	By MS/MS	By MS/MS	By MS/MS
30.05	By MS/MS	By MS/MS	By MS/MS	By MS/MS	By MS/MS	By MS/MS	By MS/MS	By MS/MS
9.7524	By MS/MS	By matchin	By MS/MS	By matchin	By MS/MS	By MS/MS	By MS/MS	By MS/MS
3.1198	By MS/MS	By matchin	By matchin	By matchin	By matchin	By MS/MS	By MS/MS	By MS/MS
5.4364	By matchin	By matchin	By matching		By matchin	By MS/MS	By MS/MS	By MS/MS
69.216	By MS/MS	By MS/MS	By MS/MS	By MS/MS	By MS/MS	By MS/MS	By MS/MS	By MS/MS
2.0325			By matching		By MS/MS			
25.386	By MS/MS	By MS/MS	By MS/MS	By MS/MS	By MS/MS	By MS/MS	By MS/MS	By MS/MS
3.198	By matchin	By matchin	By MS/MS	By MS/MS	By matchin	By matchin	By matchin	By matchin
1.5755	By matchin	By MS/MS	By matchin	By matching		By matchin	By matching	
1.8016		By matching		By matchin	By matching		By matchin	By MS/MS
7.4147	By MS/MS	By matchin	By MS/MS	By MS/MS	By MS/MS	By MS/MS	By matchin	By MS/MS
24.202	By matchin	By matchin	By matchin	By matchin	By matchin	By MS/MS	By MS/MS	By MS/MS
23.569	By MS/MS	By MS/MS	By MS/MS	By MS/MS	By MS/MS	By MS/MS	By MS/MS	By MS/MS
78.59	By MS/MS	By MS/MS	By MS/MS	By MS/MS	By MS/MS	By MS/MS	By MS/MS	By MS/MS
2.9502								By MS/MS
8.4648	By matchin	By matchin	By matchin	By matchin	By matchin	By MS/MS	By MS/MS	By MS/MS
4.5761	By MS/MS	By matchin	By matchin	By matchin	By matchin	By MS/MS	By matchin	By MS/MS
2.218	By MS/MS			By matching		By matching		
24.005	By MS/MS	By MS/MS	By MS/MS	By matchin	By MS/MS	By MS/MS	By MS/MS	By MS/MS
5.1828	By matching		By matchin	By matchin	By matching		By MS/MS	By MS/MS
4.5629				By matching		By matchin	By matchin	By MS/MS
1.6767	By matchin	By matchin	By matchin	By matchin	By matchin	By matchin	By MS/MS	By MS/MS
26.414	By MS/MS	By MS/MS	By MS/MS	By MS/MS	By MS/MS	By MS/MS	By MS/MS	By MS/MS
1.7091	By matchin	By matchin	By MS/MS	By matching		By matchin	By matchin	By matchin
43.956	By MS/MS	By MS/MS	By MS/MS	By MS/MS	By MS/MS	By MS/MS	By MS/MS	By MS/MS
2.6399	By matchin	By matchin	By matchin	By MS/MS	By MS/MS	By matchin	By matching	
3.5649	By matching		By matchin	By matchin	By matchin	By MS/MS	By MS/MS	By matchin
14.695	By matchin	By matchin	By MS/MS	By MS/MS	By MS/MS	By MS/MS	By MS/MS	By MS/MS
1.6032	By matchin	By matchin	By matchin	By matchin	By MS/MS	By matching		By matchin
16.673							By MS/MS	By matchin
6.988	By matchin	By matchin	By MS/MS	By MS/MS	By MS/MS	By MS/MS	By matchin	By MS/MS
6.7142		By MS/MS	By MS/MS		By matchin	By MS/MS	By MS/MS	By MS/MS
6.3041			By matchin	By matchin	By matchin	By MS/MS	By MS/MS	By MS/MS
1.8533			By MS/MS			By MS/MS	By MS/MS	By MS/MS
2.5756	By MS/MS	By matchin	By matching		By matchin	By matchin	By matchin	By matchin
19.893	By MS/MS	By MS/MS	By matchin	By matchin	By MS/MS	By MS/MS	By MS/MS	By MS/MS
5.5092	By MS/MS	By MS/MS	By MS/MS	By matchin	By MS/MS	By matchin	By matchin	By matchin
4.684		By MS/MS				By MS/MS	By MS/MS	By MS/MS
2.1347					By matchin	By matchin	By MS/MS	By matchin
16.37			By matchin	By matchin	By MS/MS	By MS/MS	By MS/MS	By MS/MS
5.7237	By matchin	By MS/MS	By matchin	By MS/MS	By MS/MS	By MS/MS	By MS/MS	By MS/MS
13.715	By matchin	By MS/MS	By MS/MS	By MS/MS	By MS/MS	By MS/MS	By MS/MS	By MS/MS
3.5681					By matchin	By MS/MS	By MS/MS	By MS/MS
10.224	By matchin	By matchin	By MS/MS	By matchin	By MS/MS	By MS/MS	By MS/MS	By MS/MS
1.9954	By matchin	By MS/MS	By matching		By matching		By matching	
27.998	By matchin	By matchin	By matchin	By MS/MS	By MS/MS	By MS/MS	By MS/MS	By MS/MS
10.894	By matchin	By matchin	By MS/MS	By MS/MS	By MS/MS	By MS/MS	By MS/MS	By MS/MS
1.729	By matchin	By MS/MS	By MS/MS	By matchin	By matchin	By matchin	By matching	
3.7628	By matchin	By MS/MS	By MS/MS	By MS/MS	By MS/MS	By MS/MS	By MS/MS	By MS/MS

36.319			By matching		By matchin	By MS/MS	By MS/MS	By MS/MS
1.5394	By matchin	By matchin	By MS/MS	By matchin	By matchin	By matchin	By matchin	By matchin
2.0949	By MS/MS	By matchin	By MS/MS	By MS/MS	By matching			
8.7097	By MS/MS	By MS/MS	By matchin	By matchin	By matchin	By matchin	By matchin	By matchin
82.508	By MS/MS	By MS/MS	By MS/MS	By MS/MS	By MS/MS	By MS/MS	By MS/MS	By MS/MS
27.719	By MS/MS	By MS/MS	By MS/MS	By MS/MS	By MS/MS	By MS/MS	By MS/MS	By MS/MS
42.572	By MS/MS	By MS/MS	By MS/MS	By MS/MS	By MS/MS	By matchin	By matchin	By MS/MS
7.315	By MS/MS	By MS/MS	By MS/MS	By MS/MS	By MS/MS	By MS/MS	By MS/MS	By MS/MS
5.5879	By MS/MS	By MS/MS	By MS/MS	By MS/MS	By matchin	By MS/MS	By matchin	By matchin
9.82	By MS/MS	By MS/MS	By MS/MS	By MS/MS	By MS/MS	By matchin	By matchin	By MS/MS
2.1812	By MS/MS	By MS/MS	By matchin	By matchin	By matchin	By matchin	By MS/MS	By MS/MS
7.1825	By matchin	By matchin	By MS/MS	By matchin	By MS/MS	By matchin	By matchin	By MS/MS
2.3326	By MS/MS	By MS/MS	By matchin	By matchin	By matching			
3.5376	By MS/MS	By MS/MS	By matchin	By matchin	By MS/MS	By matchin	By MS/MS	By MS/MS
13.461	By MS/MS	By MS/MS	By MS/MS	By MS/MS	By MS/MS	By MS/MS	By matchin	By MS/MS
13.396	By MS/MS	By MS/MS	By MS/MS	By MS/MS	By MS/MS	By MS/MS	By MS/MS	By MS/MS
6.3738	By MS/MS	By matchin	By MS/MS	By MS/MS	By MS/MS	By MS/MS	By matchin	By MS/MS
9.2923	By MS/MS	By MS/MS	By MS/MS	By MS/MS	By MS/MS	By MS/MS	By MS/MS	By MS/MS
9.205	By MS/MS	By MS/MS	By matchin	By MS/MS	By MS/MS	By MS/MS	By MS/MS	By MS/MS
4.5119	By MS/MS	By MS/MS	By MS/MS	By MS/MS	By MS/MS	By MS/MS	By MS/MS	By MS/MS
5.2819	By MS/MS	By MS/MS	By MS/MS	By MS/MS	By MS/MS	By MS/MS	By MS/MS	By MS/MS
2.6383	By MS/MS	By MS/MS	By matchin	By MS/MS	By matchin	By matchin	By matchin	By matchin
43.967	By MS/MS	By MS/MS	By matchin	By MS/MS	By MS/MS	By MS/MS	By MS/MS	By MS/MS
4.6607	By MS/MS	By MS/MS	By MS/MS	By MS/MS	By MS/MS	By MS/MS	By MS/MS	By MS/MS
2.9922	By MS/MS	By MS/MS	By matchin	By matching		By matchin	By MS/MS	By MS/MS
12.639	By MS/MS	By MS/MS	By MS/MS	By MS/MS	By MS/MS	By MS/MS	By MS/MS	By MS/MS
104.56	By MS/MS	By MS/MS	By MS/MS	By MS/MS	By MS/MS	By MS/MS	By MS/MS	By MS/MS
4.5186						By MS/MS	By MS/MS	By MS/MS
5.9804	By matchin	By matchin	By matchin	By matchin	By matchin	By MS/MS	By MS/MS	By MS/MS
6.7932	By matchin	By matchin	By matchin	By MS/MS	By matchin	By MS/MS	By MS/MS	By MS/MS
158.42	By MS/MS	By MS/MS	By MS/MS	By MS/MS	By MS/MS	By MS/MS	By MS/MS	By MS/MS
5.4867	By MS/MS	By MS/MS	By MS/MS	By MS/MS	By MS/MS	By MS/MS	By MS/MS	By MS/MS
1.891		By matching			By matching		By matchin	By MS/MS
9.7322	By matchin	By matchin	By matchin	By MS/MS	By MS/MS	By MS/MS	By MS/MS	By MS/MS
3.3816						By matchin	By MS/MS	By MS/MS
23.61	By MS/MS	By MS/MS	By MS/MS	By MS/MS	By MS/MS	By MS/MS	By MS/MS	By MS/MS
6.9396	By matchin	By matchin	By matchin	By matchin	By matchin	By MS/MS	By MS/MS	By MS/MS
4.6853	By matchin	By matchin	By matchin	By MS/MS	By matchin	By matchin	By MS/MS	By MS/MS
3.7266				By matching		By MS/MS	By MS/MS	By MS/MS
3.7857	By matchin	By MS/MS	By MS/MS	By MS/MS	By MS/MS	By matchin	By matchin	By matchin
17.822	By matchin	By matchin	By matchin	By MS/MS		By MS/MS	By MS/MS	By MS/MS
4.1711	By matchin	By MS/MS	By matchin	By MS/MS	By MS/MS	By MS/MS	By matchin	By MS/MS
17.049	By MS/MS	By MS/MS	By MS/MS	By MS/MS	By MS/MS	By MS/MS	By matchin	By MS/MS
6.8151	By MS/MS	By MS/MS	By MS/MS	By MS/MS	By MS/MS	By MS/MS	By MS/MS	By MS/MS
4.6483	By MS/MS	By MS/MS	By MS/MS	By MS/MS	By matchin	By MS/MS	By MS/MS	By matchin
9.8433	By matchin	By MS/MS	By MS/MS	By matchin	By MS/MS	By matchin	By matchin	By matchin
78.808	By MS/MS	By MS/MS	By MS/MS	By MS/MS	By MS/MS	By MS/MS	By MS/MS	By MS/MS
3.4632						By MS/MS		By matchin
4.5965	By matchin	By MS/MS	By MS/MS	By MS/MS	By MS/MS	By matchin	By matchin	By matchin
8.5732								By MS/MS

19.144	By matching		By MS/MS	By MS/MS	By matchin	By matchin	By MS/MS	By MS/MS
2.1351	By MS/MS	By MS/MS	By MS/MS	By matching		By MS/MS	By MS/MS	By MS/MS
14.857	By MS/MS		By matching			By matchin	By matchin	By matchin
1.7635				By MS/MS		By matchin	By matching	
8.7503	By MS/MS	By MS/MS	By matchin	By MS/MS	By MS/MS	By matchin	By matchin	By matchin
75.568	By MS/MS	By MS/MS	By MS/MS	By MS/MS	By MS/MS	By MS/MS	By MS/MS	By MS/MS
30.513	By MS/MS	By MS/MS	By MS/MS	By MS/MS	By MS/MS	By MS/MS	By MS/MS	By MS/MS
102.49	By MS/MS	By MS/MS	By MS/MS	By MS/MS	By MS/MS	By MS/MS	By MS/MS	By MS/MS
24.639	By MS/MS	By MS/MS	By MS/MS	By MS/MS	By MS/MS	By MS/MS	By MS/MS	By MS/MS
3.4402	By matchin	By matchin	By matchin	By matchin	By MS/MS	By matchin	By MS/MS	By matchin
10.536	By MS/MS	By MS/MS	By MS/MS	By MS/MS	By MS/MS	By MS/MS	By MS/MS	By MS/MS
16.416	By MS/MS	By MS/MS	By MS/MS	By MS/MS	By MS/MS	By matchin	By matchin	By MS/MS
34.127	By MS/MS	By MS/MS	By MS/MS	By MS/MS	By MS/MS	By MS/MS	By MS/MS	By MS/MS
25.101						By matchin	By matchin	By MS/MS
11.712	By matchin	By matchin	By MS/MS	By MS/MS	By matchin	By MS/MS	By matchin	By matchin
10.983	By MS/MS	By MS/MS	By MS/MS	By MS/MS	By MS/MS	By MS/MS	By MS/MS	By MS/MS
4.7883	By matchin	By matchin	By MS/MS	By MS/MS	By matchin	By matchin	By matchin	By matchin
16.927	By matchin	By matchin	By MS/MS	By MS/MS	By MS/MS	By MS/MS	By MS/MS	By MS/MS
1.5152	By MS/MS						By matchin	By matchin
31.344	By MS/MS	By MS/MS	By MS/MS	By matchin	By MS/MS	By matchin	By MS/MS	By MS/MS
14.662	By matchin	By MS/MS	By matching		By matchin	By MS/MS	By MS/MS	By MS/MS
3.7092	By matchin	By MS/MS	By matching			By matchin	By MS/MS	By MS/MS
3.7702	By MS/MS	By MS/MS	By matchin	By matchin	By matchin	By matchin	By matchin	By MS/MS
1.7591			By matching			By MS/MS		
41.8	By matchin	By matchin	By MS/MS	By MS/MS	By MS/MS	By MS/MS	By MS/MS	By MS/MS
28.089	By MS/MS	By MS/MS	By matchin	By matchin	By matchin	By MS/MS	By MS/MS	By MS/MS
28.131	By MS/MS	By MS/MS	By MS/MS	By MS/MS	By MS/MS	By MS/MS	By MS/MS	By MS/MS
10.518	By MS/MS	By MS/MS	By matchin	By matchin	By matchin	By MS/MS	By MS/MS	By MS/MS
56.819	By MS/MS	By MS/MS	By MS/MS	By MS/MS	By MS/MS	By MS/MS	By MS/MS	By MS/MS
43.524	By MS/MS	By MS/MS	By MS/MS	By MS/MS	By MS/MS	By MS/MS	By MS/MS	By MS/MS
35.863	By MS/MS	By MS/MS	By MS/MS	By MS/MS	By MS/MS	By MS/MS	By MS/MS	By MS/MS
20.487		By matchin	By matchin	By matchin	By matchin	By MS/MS	By MS/MS	By MS/MS
186.36	By MS/MS	By MS/MS	By MS/MS	By MS/MS	By MS/MS	By MS/MS	By MS/MS	By MS/MS
3.5022	By matchin	By matchin	By matchin	By MS/MS	By MS/MS	By matchin	By matching	
8.1471		By matchin	By MS/MS	By MS/MS	By MS/MS	By MS/MS	By matchin	By MS/MS
1.5224								By MS/MS
4.1495	By matchin	By MS/MS	By MS/MS	By MS/MS	By matchin	By matchin	By matchin	By matchin
3.1185	By matchin	By MS/MS	By matchin	By matchin	By MS/MS	By matchin	By matchin	By matchin
1.9859			By MS/MS	By matchin	By MS/MS	By matchin	By matchin	By matchin
2.125			By matchin	By matching			By MS/MS	
17.221	By MS/MS	By MS/MS	By MS/MS	By MS/MS	By matching		By MS/MS	By MS/MS
14.899	By MS/MS	By MS/MS	By MS/MS	By matchin	By MS/MS	By matchin	By matchin	By MS/MS
10.862	By MS/MS	By MS/MS	By MS/MS	By MS/MS	By MS/MS	By MS/MS	By matchin	By MS/MS
9.7386	By matchin	By MS/MS	By MS/MS	By MS/MS	By matchin	By MS/MS	By MS/MS	By MS/MS
22.74	By MS/MS	By MS/MS	By MS/MS	By MS/MS	By MS/MS	By MS/MS	By matchin	By MS/MS
2.0928	By matching		By matchin	By matchin	By MS/MS			
5.3296	By MS/MS	By MS/MS	By MS/MS	By matchin	By MS/MS	By matching		By matchin
3.1913						By matching		By MS/MS
3.9363	By matchin	By matchin	By matchin	By MS/MS	By MS/MS	By matching		By matchin
4.4316	By MS/MS	By matchin	By MS/MS	By MS/MS	By matchin	By matchin	By matchin	By matchin

4.923	By MS/MS	By MS/MS	By matchin	By MS/MS	By matchin	By MS/MS	By MS/MS	By MS/MS
1.6597								By MS/MS
7.2937	By MS/MS	By MS/MS	By MS/MS	By MS/MS	By MS/MS	By MS/MS	By matchin	By MS/MS
3.2357	By MS/MS	By matchin	By matchin	By MS/MS	By MS/MS	By matchin	By matchin	By matchin
23.722	By MS/MS	By MS/MS	By MS/MS	By MS/MS	By MS/MS	By MS/MS	By MS/MS	By MS/MS
81.224	By MS/MS	By MS/MS	By MS/MS	By MS/MS	By MS/MS	By MS/MS	By MS/MS	By MS/MS
95.902	By MS/MS	By MS/MS	By MS/MS	By MS/MS	By MS/MS	By MS/MS	By MS/MS	By MS/MS
10.269	By MS/MS	By MS/MS	By MS/MS	By MS/MS	By MS/MS	By MS/MS	By MS/MS	By MS/MS
11.59	By MS/MS	By MS/MS	By MS/MS	By MS/MS	By MS/MS	By MS/MS	By MS/MS	By MS/MS
2.7461	By MS/MS	By MS/MS	By matchin	By matchin	By matchin	By matching		
2.3207	By MS/MS			By MS/MS		By MS/MS		By matchin
7.2379	By MS/MS	By MS/MS	By MS/MS	By MS/MS	By MS/MS	By MS/MS	By MS/MS	By MS/MS
4.2778	By MS/MS	By MS/MS	By MS/MS	By MS/MS	By matchin	By matchin	By MS/MS	By MS/MS
-2	By matchin	By matchin	By matchin	By matchin	By MS/MS	By matching		
4.3778	By matchin	By matchin	By MS/MS	By MS/MS	By MS/MS	By matchin	By matchin	By MS/MS
2.8175						By matchin	By matchin	By MS/MS
7.5474								By MS/MS
5.3028	By matchin	By MS/MS	By MS/MS	By matchin	By matchin	By MS/MS	By MS/MS	By matchin
5.0422	By matchin	By matchin	By matchin	By matching		By MS/MS	By MS/MS	By matchin
6.9432			By matchin	By matchin	By matchin	By matchin	By MS/MS	By MS/MS
4.7008	By MS/MS	By MS/MS	By matching		By matchin	By matchin	By matchin	By MS/MS
-2	By matchin	By MS/MS	By matchin	By matching		By matching		
1.9176	By matchin	By matchin	By matchin	By matchin	By matchin	By MS/MS	By matchin	By MS/MS
12.156	By matchin	By MS/MS	By MS/MS	By MS/MS	By MS/MS	By MS/MS	By MS/MS	By MS/MS
9.4366					By MS/MS	By matchin	By MS/MS	By MS/MS
5.5287								By MS/MS
3.3951	By MS/MS	By MS/MS	By MS/MS	By matchin	By matchin	By MS/MS	By matching	
74.904	By MS/MS	By MS/MS	By MS/MS	By MS/MS	By MS/MS	By MS/MS	By MS/MS	By MS/MS
10.482	By matchin	By matchin	By matchin	By matchin	By matchin	By MS/MS	By MS/MS	By MS/MS
1.6895	By matchin	By matchin	By MS/MS	By MS/MS	By matchin	By MS/MS	By matchin	By matchin
7.966	By matchin	By matchin	By matchin	By matchin	By MS/MS	By MS/MS	By MS/MS	By MS/MS
28.418	By MS/MS	By MS/MS	By MS/MS	By matchin	By MS/MS	By MS/MS	By MS/MS	By matchin
4.5104	By matchin	By matchin	By matchin	By MS/MS	By MS/MS	By matching		By matchin
3.0881	By MS/MS	By MS/MS	By matchin	By matchin	By matchin	By matchin	By matchin	By matchin
3.3399	By MS/MS	By MS/MS	By matchin	By matchin	By matchin	By MS/MS	By MS/MS	By matchin
12.538	By MS/MS	By matchin	By MS/MS	By MS/MS	By MS/MS	By MS/MS	By MS/MS	By MS/MS
1.9719	By matchin	By matchin	By matchin	By matchin	By MS/MS	By MS/MS	By matchin	By matchin
2.7429		By matchin	By matchin	By matchin	By MS/MS	By MS/MS	By matchin	By matchin
20.722	By MS/MS	By MS/MS	By MS/MS	By MS/MS	By MS/MS	By MS/MS	By MS/MS	By MS/MS
27.251	By MS/MS	By MS/MS	By MS/MS	By MS/MS	By MS/MS	By MS/MS	By MS/MS	By MS/MS
42.301	By MS/MS	By MS/MS	By MS/MS	By MS/MS	By MS/MS	By MS/MS	By MS/MS	By MS/MS
22.524	By MS/MS	By MS/MS	By MS/MS	By MS/MS	By MS/MS	By MS/MS	By MS/MS	By MS/MS
1.7823	By matchin	By matchin	By matchin	By matchin	By matchin	By matchin	By matchin	By MS/MS
46.015	By MS/MS	By MS/MS	By MS/MS	By MS/MS	By MS/MS	By MS/MS	By MS/MS	By MS/MS
77.398	By MS/MS	By MS/MS	By MS/MS	By MS/MS	By MS/MS	By MS/MS	By MS/MS	By MS/MS
9.9199	By matchin	By matchin	By matchin	By matchin	By matchin	By matchin	By MS/MS	By MS/MS
58.993	By MS/MS	By MS/MS	By MS/MS	By MS/MS	By MS/MS	By MS/MS	By MS/MS	By MS/MS
178.65	By MS/MS	By MS/MS	By MS/MS	By MS/MS	By MS/MS	By MS/MS	By MS/MS	By MS/MS
7.1008	By MS/MS	By MS/MS	By MS/MS	By MS/MS	By MS/MS	By matchin	By matchin	By MS/MS
29.486	By MS/MS	By MS/MS	By MS/MS	By MS/MS	By MS/MS	By MS/MS	By MS/MS	By MS/MS

184.21	By MS/MS	By MS/MS	By MS/MS	By MS/MS	By MS/MS	By MS/MS	By MS/MS	By MS/MS
2.4529	By MS/MS	By matchin	By MS/MS	By matchin	By MS/MS	By MS/MS	By MS/MS	By MS/MS
3.2587	By matchin	By MS/MS	By matchin	By MS/MS	By matchin	By MS/MS	By matchin	By matchin
3.6634	By MS/MS	By matchin	By matchin	By matchin	By matchin	By matchin	By matchin	By matchin
13.369	By MS/MS	By MS/MS	By MS/MS	By MS/MS	By MS/MS	By MS/MS	By MS/MS	By MS/MS
11.843	By MS/MS	By MS/MS	By MS/MS	By MS/MS	By MS/MS	By MS/MS	By matchin	By MS/MS
1.9353					By matchin	By MS/MS		By MS/MS
13.926	By MS/MS	By MS/MS	By MS/MS	By MS/MS	By MS/MS	By MS/MS	By MS/MS	By MS/MS
7.3545	By MS/MS	By MS/MS	By MS/MS	By MS/MS	By MS/MS	By MS/MS	By matchin	By matchin
20.181	By MS/MS	By MS/MS	By MS/MS	By MS/MS	By MS/MS	By MS/MS	By MS/MS	By MS/MS
54.38	By MS/MS	By MS/MS	By MS/MS	By MS/MS	By MS/MS	By MS/MS	By MS/MS	By MS/MS
3.8324	By MS/MS	By MS/MS	By matchin	By MS/MS	By matchin	By matchin	By matchin	By matchin
100.04	By MS/MS	By MS/MS	By MS/MS	By MS/MS	By MS/MS	By MS/MS	By MS/MS	By MS/MS
1.5601						By matchin	By MS/MS	By MS/MS
14.763	By MS/MS	By MS/MS	By MS/MS	By MS/MS	By MS/MS	By MS/MS	By MS/MS	By MS/MS
12.747	By MS/MS	By MS/MS	By MS/MS	By MS/MS	By MS/MS	By MS/MS	By MS/MS	By MS/MS
10.635	By MS/MS	By MS/MS	By MS/MS	By MS/MS	By MS/MS	By matchin	By MS/MS	By MS/MS
39.811	By MS/MS	By MS/MS	By MS/MS	By MS/MS	By MS/MS	By MS/MS	By MS/MS	By MS/MS
10.963	By matchin	By matchin	By MS/MS	By MS/MS	By MS/MS			
152.57	By MS/MS	By MS/MS	By MS/MS	By MS/MS	By MS/MS	By MS/MS	By MS/MS	By MS/MS
12.758	By matchin	By MS/MS	By matchin	By MS/MS	By matchin	By matchin	By MS/MS	By MS/MS
20.34	By MS/MS	By MS/MS	By MS/MS	By MS/MS	By MS/MS	By MS/MS	By MS/MS	By MS/MS
3.7461	By MS/MS	By MS/MS		By matchin	By MS/MS	By matchin	By matchin	By MS/MS
15.821	By MS/MS	By MS/MS	By MS/MS	By MS/MS	By matchin	By MS/MS	By MS/MS	By MS/MS
2.7524	By MS/MS		By MS/MS	By MS/MS	By MS/MS	By MS/MS	By MS/MS	By matchin
2.6883	By MS/MS	By MS/MS	By matchin	By MS/MS		By MS/MS	By MS/MS	By matchin
5.6474	By MS/MS		By matching		By MS/MS	By matching		By matchin
9.0374	By matchin	By MS/MS	By MS/MS	By MS/MS	By MS/MS	By MS/MS	By matchin	By MS/MS
2.2272			By MS/MS		By MS/MS		By matchin	By matchin
1.7113	By matchin	By matchin	By matchin	By MS/MS		By matchin	By MS/MS	By matchin
3.1151	By MS/MS	By matchin	By MS/MS	By matchin	By matchin	By matchin	By matchin	By MS/MS
19.94	By MS/MS	By MS/MS	By MS/MS	By MS/MS	By MS/MS	By MS/MS	By MS/MS	By MS/MS
47.77	By MS/MS	By MS/MS	By MS/MS	By MS/MS	By MS/MS	By MS/MS	By MS/MS	By MS/MS
6.0523	By MS/MS	By matchin	By matching		By MS/MS	By matchin	By matchin	By MS/MS
1.9091		By MS/MS	By MS/MS	By MS/MS				By matchin
1.9804	By matchin	By MS/MS	By matchin	By MS/MS	By matchin	By matching		
99.1	By MS/MS	By MS/MS	By MS/MS	By MS/MS	By MS/MS	By MS/MS	By MS/MS	By MS/MS
8.6028	By matchin	By MS/MS	By MS/MS	By MS/MS	By MS/MS	By MS/MS	By MS/MS	By MS/MS
13.861	By matchin	By matchin	By MS/MS	By MS/MS	By MS/MS	By MS/MS	By MS/MS	By MS/MS
16.03			By matchin	By matching		By MS/MS	By MS/MS	By MS/MS
4.1541	By matchin	By matchin	By matchin	By matchin	By MS/MS	By matchin	By MS/MS	
6.5947						By matchin	By MS/MS	By MS/MS
3.2776			By matchin	By MS/MS	By matchin	By MS/MS	By MS/MS	By matchin
9.9732	By matchin	By MS/MS	By MS/MS	By MS/MS	By MS/MS	By MS/MS	By MS/MS	By MS/MS
24.39	By matchin	By MS/MS	By MS/MS	By MS/MS	By matchin	By MS/MS	By MS/MS	By MS/MS
22.217	By MS/MS	By MS/MS	By MS/MS	By MS/MS	By MS/MS	By MS/MS	By MS/MS	By MS/MS
2.0837			By matching		By matchin	By MS/MS	By matchin	By MS/MS
26.761	By MS/MS	By MS/MS	By MS/MS	By MS/MS	By MS/MS	By MS/MS	By MS/MS	By MS/MS
19.209	By MS/MS	By matchin	By matchin	By matchin	By matchin	By MS/MS	By MS/MS	By MS/MS
14.058	By MS/MS	By matchin	By MS/MS	By MS/MS	By matchin	By MS/MS	By MS/MS	By MS/MS

24.477	By MS/MS	By MS/MS	By matchin	By matchin	By matchin	By MS/MS	By MS/MS	By MS/MS
15.827	By MS/MS	By MS/MS	By MS/MS	By matchin	By matchin	By MS/MS	By MS/MS	By MS/MS
5.3587	By matchin	By matchin	By matchin	By matchin	By matchin	By MS/MS	By MS/MS	By MS/MS
6.6413	By matching			By matchin	By matchin	By matchin	By MS/MS	By MS/MS
11.416	By matchin	By MS/MS	By matchin	By matching		By MS/MS	By MS/MS	By MS/MS
21.442	By MS/MS	By MS/MS	By MS/MS	By MS/MS	By MS/MS	By MS/MS	By MS/MS	By MS/MS
5.1041		By matchin	By MS/MS	By MS/MS		By MS/MS	By MS/MS	By MS/MS
2.1126		By matchin	By matchin	By matchin	By matchin	By MS/MS	By matchin	By MS/MS
20.858	By matchin	By matchin	By matchin	By MS/MS	By MS/MS	By MS/MS	By MS/MS	By MS/MS
2.1166			By matching			By MS/MS	By MS/MS	
46.973	By MS/MS	By MS/MS	By MS/MS	By MS/MS	By MS/MS	By MS/MS	By MS/MS	By MS/MS
1.9278	By matching		By matching			By MS/MS	By matchin	By MS/MS
56.473			By MS/MS	By MS/MS	By MS/MS	By MS/MS	By MS/MS	By MS/MS
31.773	By MS/MS	By matchin	By matchin	By MS/MS	By MS/MS	By MS/MS	By MS/MS	By MS/MS
3.0254	By matching			By matching		By MS/MS	By MS/MS	By MS/MS
50.794	By MS/MS	By MS/MS	By MS/MS	By MS/MS	By MS/MS	By MS/MS	By MS/MS	By MS/MS
30.444	By matchin	By matchin	By matchin	By matchin	By matchin	By MS/MS	By MS/MS	By MS/MS
14.32	By MS/MS	By MS/MS	By matchin	By MS/MS	By MS/MS	By MS/MS	By MS/MS	By MS/MS
2.8456	By matchin	By matchin	By matchin	By MS/MS	By MS/MS	By MS/MS	By MS/MS	By MS/MS
16.662	By MS/MS	By matchin	By MS/MS	By matchin	By MS/MS	By MS/MS	By MS/MS	By MS/MS
2.6505				By matching		By matchin	By matchin	By MS/MS
5.2584	By MS/MS	By matchin	By MS/MS	By matchin	By MS/MS	By MS/MS	By matchin	By MS/MS
2.025	By matchin	By matching			By matchin	By matchin	By MS/MS	By MS/MS
13.069	By matchin	By matchin	By matchin	By matchin	By MS/MS	By MS/MS	By MS/MS	By MS/MS
25.633	By matchin	By matchin	By MS/MS	By MS/MS	By MS/MS	By MS/MS	By MS/MS	By MS/MS
2.7923	By matchin	By matchin	By matchin	By matchin	By matchin	By MS/MS	By matchin	By MS/MS
44.559	By MS/MS	By MS/MS	By MS/MS	By MS/MS	By MS/MS	By MS/MS	By MS/MS	By MS/MS
19.007	By matchin	By MS/MS	By MS/MS	By MS/MS	By MS/MS	By MS/MS	By MS/MS	By MS/MS
50.396	By MS/MS	By MS/MS	By MS/MS	By MS/MS	By MS/MS	By MS/MS	By MS/MS	By MS/MS
22.15	By MS/MS	By MS/MS	By MS/MS	By MS/MS	By MS/MS	By MS/MS	By MS/MS	By MS/MS
33.301	By MS/MS	By MS/MS	By MS/MS	By MS/MS	By MS/MS	By MS/MS	By MS/MS	By MS/MS
21.149	By matchin	By matchin	By matchin	By matchin	By matchin	By MS/MS	By MS/MS	By MS/MS
29.046	By MS/MS	By matchin	By MS/MS	By MS/MS	By MS/MS	By MS/MS	By MS/MS	By MS/MS
23.184		By matchin	By matchin	By MS/MS	By MS/MS	By MS/MS	By MS/MS	By MS/MS
46.525	By matchin	By matchin	By matchin	By MS/MS	By MS/MS	By MS/MS	By MS/MS	By MS/MS
27.622	By MS/MS	By MS/MS	By MS/MS	By MS/MS	By matchin	By MS/MS	By MS/MS	By MS/MS
8.0749	By MS/MS	By matchin	By matchin	By MS/MS	By matchin	By MS/MS	By MS/MS	By MS/MS
21.308	By MS/MS	By MS/MS	By MS/MS	By MS/MS	By MS/MS	By MS/MS	By matchin	By MS/MS
116.96	By MS/MS	By MS/MS	By MS/MS	By MS/MS	By MS/MS	By MS/MS	By MS/MS	By MS/MS
158.73	By MS/MS	By MS/MS	By MS/MS	By MS/MS	By MS/MS	By MS/MS	By MS/MS	By MS/MS
27.393			By matchin	By matchin	By matchin	By MS/MS	By MS/MS	By MS/MS
3.7803	By MS/MS	By MS/MS	By matchin	By MS/MS	By MS/MS	By matchin	By matchin	By matchin
19.737	By MS/MS	By MS/MS	By MS/MS	By MS/MS	By MS/MS	By MS/MS	By MS/MS	By MS/MS
4.1103	By MS/MS	By matchin	By matchin	By matchin	By matchin	By MS/MS	By MS/MS	By matchin
13.24	By MS/MS	By MS/MS	By MS/MS	By MS/MS	By MS/MS	By MS/MS	By MS/MS	By MS/MS
-2					By MS/MS			
323.31	By MS/MS	By MS/MS	By MS/MS	By MS/MS	By MS/MS	By MS/MS	By MS/MS	By MS/MS
155.02	By MS/MS	By MS/MS	By MS/MS	By MS/MS	By MS/MS	By MS/MS	By MS/MS	By MS/MS
3.6485	By matching		By matching		By matchin	By matchin	By MS/MS	By MS/MS
46.046	By MS/MS	By MS/MS	By MS/MS	By MS/MS	By MS/MS	By MS/MS	By MS/MS	By MS/MS

13.87		By matchin	By matchin	By matchin	By matchin	By MS/MS	By MS/MS	By MS/MS
11.562	By MS/MS	By MS/MS	By MS/MS	By MS/MS	By MS/MS	By MS/MS	By MS/MS	By MS/MS
11.276	By matchin	By matchin	By matchin	By MS/MS	By MS/MS	By matchin	By MS/MS	By MS/MS
2.2374	By matching		By matchin	By matchin	By MS/MS	By matchin	By MS/MS	By MS/MS
20.79	By MS/MS	By MS/MS	By MS/MS	By MS/MS	By matchin	By MS/MS	By MS/MS	By MS/MS
5.2857	By matchin	By MS/MS	By MS/MS	By matchin	By matchin	By MS/MS	By matchin	By matchin
2.3722	By matchin	By matchin	By matchin	By matchin	By MS/MS	By matching		
198.23	By MS/MS	By MS/MS	By MS/MS	By MS/MS	By MS/MS	By MS/MS	By MS/MS	By MS/MS
51.271	By matchin	By matchin	By MS/MS	By MS/MS	By MS/MS	By MS/MS	By MS/MS	By MS/MS
59.453	By MS/MS	By MS/MS	By MS/MS	By MS/MS	By MS/MS	By MS/MS	By MS/MS	By MS/MS
5.8019	By MS/MS	By MS/MS	By MS/MS	By MS/MS	By MS/MS	By matchin	By MS/MS	By MS/MS
7.8937	By MS/MS	By matchin	By MS/MS	By MS/MS	By MS/MS	By MS/MS	By MS/MS	By MS/MS
50.022	By MS/MS	By MS/MS	By MS/MS	By MS/MS	By MS/MS	By MS/MS	By MS/MS	By MS/MS
3.8539		By matching				By matchin	By MS/MS	By MS/MS
10.848	By MS/MS	By MS/MS	By MS/MS	By MS/MS	By MS/MS	By matchin	By MS/MS	By MS/MS
2.56	By MS/MS	By MS/MS	By MS/MS	By MS/MS	By matchin	By MS/MS	By MS/MS	By matchin
5.698		By matchin	By matchin	By matchin	By matchin	By MS/MS	By MS/MS	By MS/MS
24.006	By MS/MS	By MS/MS	By MS/MS	By MS/MS	By MS/MS	By MS/MS	By MS/MS	By MS/MS
4.1294	By matchin	By matchin	By MS/MS	By matchin	By matchin	By MS/MS	By matchin	By matchin
57.955	By MS/MS	By MS/MS	By MS/MS	By MS/MS	By MS/MS	By matchin	By matchin	By matchin
1.8152	By MS/MS	By matching					By matchin	By matchin
16.651	By matchin	By MS/MS	By MS/MS	By MS/MS	By matchin	By MS/MS	By MS/MS	By MS/MS
2.1722			By matchin	By matchin	By matchin	By MS/MS	By MS/MS	By MS/MS
3.4822							By matchin	By MS/MS
6.0681			By MS/MS	By matchin	By matchin	By matchin	By matchin	By MS/MS
2.3068						By MS/MS	By MS/MS	By MS/MS
10.484		By matchin	By matchin	By matchin	By matchin	By MS/MS	By MS/MS	By MS/MS
35.886	By MS/MS	By MS/MS	By MS/MS	By MS/MS	By matchin	By MS/MS	By MS/MS	By MS/MS
132.75	By MS/MS	By MS/MS	By MS/MS	By MS/MS	By MS/MS	By MS/MS	By MS/MS	By MS/MS
76.837	By MS/MS	By MS/MS	By MS/MS	By MS/MS	By MS/MS	By MS/MS	By MS/MS	By MS/MS
75.197	By MS/MS	By MS/MS	By MS/MS	By MS/MS	By MS/MS	By MS/MS	By MS/MS	By MS/MS
2.5722					By matchin	By matchin	By matchin	By MS/MS
1.4868						By matchin	By MS/MS	By MS/MS
5.3962						By matchin	By MS/MS	By MS/MS
38.885	By MS/MS	By MS/MS	By MS/MS	By MS/MS	By MS/MS	By MS/MS	By MS/MS	By MS/MS
7.8702	By MS/MS	By MS/MS	By MS/MS	By matchin	By matchin	By MS/MS	By MS/MS	By MS/MS
12.681	By matchin	By matchin	By matchin	By matchin	By MS/MS	By MS/MS	By MS/MS	By MS/MS
2.0848			By matchin	By matchin	By matchin	By MS/MS	By matchin	By MS/MS
6.0093		By MS/MS	By MS/MS	By MS/MS	By matchin	By MS/MS	By MS/MS	By matchin
6.8293	By matchin	By matchin	By matchin	By MS/MS	By matchin	By MS/MS	By MS/MS	By MS/MS
6.621	By matchin	By matchin	By matchin	By matchin	By MS/MS	By MS/MS	By MS/MS	By MS/MS
7.2366		By matchin	By matchin	By matchin	By matchin	By MS/MS	By MS/MS	By MS/MS
95.921	By MS/MS	By MS/MS	By MS/MS	By MS/MS	By MS/MS	By MS/MS	By MS/MS	By MS/MS
118.2	By MS/MS	By MS/MS	By MS/MS	By MS/MS	By MS/MS	By MS/MS	By MS/MS	By MS/MS
1.7186	By MS/MS	By MS/MS	By MS/MS	By MS/MS		By matchin	By matching	
40.767	By MS/MS	By MS/MS	By MS/MS	By MS/MS	By MS/MS	By MS/MS	By MS/MS	By MS/MS
9.754	By MS/MS	By matchin	By MS/MS	By MS/MS	By MS/MS	By MS/MS	By matchin	By matchin
9.9903	By matchin	By matchin	By matchin	By MS/MS	By MS/MS	By MS/MS	By matchin	By MS/MS
3.0126	By matchin	By MS/MS	By matchin	By matchin	By matchin	By MS/MS	By MS/MS	
2.5421				By matching		By MS/MS	By MS/MS	By MS/MS

6.2083			By matchin	By matchin	By matchin	By matchin	By matchin	By MS/MS
24.031		By matchin	By matchin	By matchin	By matchin	By matchin	By matchin	By MS/MS
13.714	By MS/MS	By MS/MS	By MS/MS	By MS/MS	By MS/MS	By matchin	By matchin	By matchin
14.05	By matchin	By matchin	By matchin	By matchin	By matchin	By MS/MS	By MS/MS	By MS/MS
22.706	By MS/MS	By MS/MS	By MS/MS	By MS/MS	By MS/MS	By MS/MS	By MS/MS	By matchin
4.24	By MS/MS	By MS/MS	By matchin	By matchin	By MS/MS	By matchin	By matchin	By matchin
5.4725	By matchin	By matchin	By matchin	By matchin	By MS/MS	By matchin	By MS/MS	By MS/MS
8.6561	By matchin	By MS/MS	By MS/MS	By MS/MS	By MS/MS	By MS/MS	By MS/MS	By MS/MS
1.525	By matchin	By matchin	By matching		By matchin	By matchin	By MS/MS	By matchin
4.2752	By MS/MS	By MS/MS	By MS/MS	By MS/MS	By MS/MS	By MS/MS	By MS/MS	
10.617	By MS/MS	By MS/MS	By MS/MS	By MS/MS	By MS/MS	By matchin	By MS/MS	By MS/MS
2.9168	By matchin	By matchin	By matchin	By MS/MS	By MS/MS	By matchin	By matchin	By matchin
1.8386				By MS/MS				
2.8955	By matchin	By matching		By MS/MS	By MS/MS		By matching	
2.9033	By matchin	By MS/MS	By MS/MS	By MS/MS	By MS/MS			
1.8946	By MS/MS	By MS/MS						By MS/MS
9.0223	By MS/MS	By MS/MS	By MS/MS	By MS/MS	By MS/MS	By MS/MS	By matchin	By matchin
3.6865	By matchin	By matchin	By MS/MS	By MS/MS	By MS/MS		By matchin	By MS/MS
4.0102	By MS/MS	By matchin	By MS/MS	By MS/MS	By MS/MS	By MS/MS	By matchin	By MS/MS
17.953	By MS/MS	By MS/MS	By MS/MS	By MS/MS	By MS/MS	By MS/MS	By MS/MS	By MS/MS
4.8463	By MS/MS	By MS/MS	By matching		By MS/MS	By MS/MS	By MS/MS	
2.6959	By MS/MS	By MS/MS	By MS/MS	By matching		By MS/MS	By MS/MS	
1.9975	By matchin	By matchin	By matchin	By matchin	By matchin	By MS/MS	By MS/MS	By matchin
5.0611	By matchin	By MS/MS	By MS/MS	By MS/MS	By matchin	By matchin	By MS/MS	By matchin
15.973	By MS/MS	By MS/MS	By MS/MS	By MS/MS	By MS/MS	By MS/MS	By MS/MS	By matchin
9.3185	By MS/MS	By MS/MS	By MS/MS	By MS/MS	By MS/MS	By MS/MS	By MS/MS	By matchin
36.738	By MS/MS	By MS/MS	By MS/MS	By MS/MS	By MS/MS	By MS/MS	By MS/MS	By MS/MS
110.27	By MS/MS	By MS/MS	By MS/MS	By MS/MS	By MS/MS	By MS/MS	By MS/MS	By MS/MS
31.404	By MS/MS	By MS/MS	By MS/MS	By MS/MS	By MS/MS	By MS/MS	By MS/MS	By MS/MS
4.9454	By matchin	By matchin	By MS/MS	By MS/MS	By MS/MS	By matchin	By matchin	By MS/MS
6.485	By matchin	By MS/MS	By MS/MS	By MS/MS	By MS/MS	By MS/MS	By MS/MS	By MS/MS
2.0003	By matchin	By matchin	By MS/MS	By matchin	By MS/MS	By matchin	By matchin	By matchin
1.705	By matching			By MS/MS				
72.106	By MS/MS	By MS/MS	By MS/MS	By MS/MS	By MS/MS	By MS/MS	By MS/MS	By MS/MS
4.5438	By matchin	By MS/MS	By matchin	By MS/MS	By MS/MS	By matchin	By matchin	By MS/MS
1.9662				By matchin	By MS/MS			
9.3678	By MS/MS	By MS/MS	By MS/MS	By MS/MS	By matchin	By MS/MS	By MS/MS	By MS/MS
11.618	By MS/MS	By MS/MS	By MS/MS	By MS/MS	By MS/MS	By MS/MS	By MS/MS	By MS/MS
10.926	By MS/MS	By MS/MS	By MS/MS	By MS/MS	By matchin	By MS/MS	By MS/MS	By MS/MS
24.54	By MS/MS	By MS/MS	By MS/MS	By MS/MS	By MS/MS	By MS/MS	By MS/MS	By MS/MS
1.6084	By matchin	By matchin	By matchin	By matchin	By MS/MS	By matching		By MS/MS
164.23	By MS/MS	By MS/MS	By MS/MS	By MS/MS	By MS/MS	By MS/MS	By MS/MS	By MS/MS
61.739	By MS/MS	By MS/MS	By MS/MS	By MS/MS	By MS/MS	By MS/MS	By MS/MS	By MS/MS
2.7693	By matchin	By MS/MS	By MS/MS	By MS/MS	By MS/MS	By matchin	By MS/MS	By MS/MS
198.7	By MS/MS	By MS/MS	By MS/MS	By MS/MS	By MS/MS	By MS/MS	By MS/MS	By MS/MS
2.4423	By matchin	By matchin	By matchin	By matchin	By MS/MS	By matchin	By matchin	By MS/MS
97.039	By MS/MS	By MS/MS	By MS/MS	By MS/MS	By MS/MS	By MS/MS	By MS/MS	By MS/MS
68.651	By MS/MS	By MS/MS	By MS/MS	By MS/MS	By MS/MS	By MS/MS	By MS/MS	By MS/MS
123	By MS/MS	By MS/MS	By MS/MS	By MS/MS	By MS/MS	By MS/MS	By MS/MS	By MS/MS
119.94	By MS/MS	By MS/MS	By MS/MS	By MS/MS	By MS/MS	By MS/MS	By MS/MS	By MS/MS

323.31	By MS/MS	By MS/MS	By MS/MS	By MS/MS	By MS/MS	By MS/MS	By MS/MS	By MS/MS
2.8507	By matchin	By matchin	By matchin	By matchin	By MS/MS	By matchin	By matchin	By matchin
71.758			By matchin	By matchin	By matchin	By MS/MS	By MS/MS	By MS/MS
2.6865					By matchin	By MS/MS	By MS/MS	By matchin
10.832	By MS/MS	By MS/MS	By MS/MS	By MS/MS	By MS/MS	By MS/MS	By MS/MS	By MS/MS
1.5104			By matchin	By matchin	By matchin	By MS/MS	By matchin	By MS/MS
17.325	By MS/MS	By MS/MS	By MS/MS	By MS/MS	By MS/MS	By MS/MS	By MS/MS	By MS/MS
4.4377	By matchin	By matchin	By matchin	By MS/MS		By matchin	By matchin	By matchin
4.9953	By matchin	By MS/MS	By MS/MS	By MS/MS	By MS/MS	By matchin	By matchin	By MS/MS
1.938	By matchin	By matchin	By MS/MS	By MS/MS	By MS/MS	By MS/MS	By MS/MS	By MS/MS
14.083			By matchin	By matchin	By matchin	By MS/MS	By MS/MS	By MS/MS
21.759	By MS/MS	By MS/MS	By MS/MS	By MS/MS	By MS/MS	By MS/MS	By MS/MS	By MS/MS
11.006	By matchin	By matchin	By MS/MS	By MS/MS	By MS/MS	By MS/MS	By MS/MS	By MS/MS
143.45	By MS/MS	By MS/MS	By MS/MS	By MS/MS	By matchin	By MS/MS	By MS/MS	By MS/MS
97.828	By matchin	By matchin	By MS/MS	By MS/MS	By MS/MS	By MS/MS	By MS/MS	By MS/MS
73.895	By MS/MS	By MS/MS	By MS/MS	By MS/MS	By MS/MS	By MS/MS	By MS/MS	By MS/MS
14.283	By matchin	By MS/MS	By matchin	By matchin	By MS/MS	By MS/MS	By MS/MS	By MS/MS
4.6428	By matchin	By matchin	By matchin	By matchin	By matchin	By MS/MS	By MS/MS	By matchin
60.083	By MS/MS	By MS/MS	By MS/MS	By MS/MS	By MS/MS	By MS/MS	By MS/MS	By MS/MS
51.285	By MS/MS	By MS/MS	By MS/MS	By MS/MS	By MS/MS	By MS/MS	By MS/MS	By MS/MS
8.7	By MS/MS	By MS/MS	By MS/MS	By MS/MS	By MS/MS	By MS/MS	By matchin	By MS/MS
30.362	By MS/MS	By MS/MS	By MS/MS	By MS/MS	By matchin	By MS/MS	By MS/MS	By MS/MS
82.762	By MS/MS	By MS/MS	By MS/MS	By MS/MS	By MS/MS	By MS/MS	By MS/MS	By MS/MS
62.883	By MS/MS	By MS/MS	By MS/MS	By MS/MS	By MS/MS	By MS/MS	By MS/MS	By MS/MS
74.778	By MS/MS	By MS/MS	By MS/MS	By MS/MS	By MS/MS	By MS/MS	By MS/MS	By MS/MS
42.572	By MS/MS	By MS/MS	By MS/MS	By MS/MS	By MS/MS	By MS/MS	By MS/MS	By MS/MS
41.383	By MS/MS	By MS/MS	By MS/MS	By MS/MS	By MS/MS	By MS/MS	By MS/MS	By MS/MS
143.7	By MS/MS	By MS/MS	By MS/MS	By MS/MS	By MS/MS	By MS/MS	By MS/MS	By MS/MS
5.718	By matchin	By MS/MS	By matchin	By MS/MS	By MS/MS	By MS/MS	By MS/MS	By MS/MS
42.916	By MS/MS	By MS/MS	By MS/MS	By MS/MS	By MS/MS	By MS/MS	By MS/MS	By MS/MS
165.57	By MS/MS	By MS/MS	By MS/MS	By MS/MS	By MS/MS	By MS/MS	By MS/MS	By MS/MS
192.02	By MS/MS	By MS/MS	By MS/MS	By MS/MS	By MS/MS	By MS/MS	By MS/MS	By MS/MS
12.412	By MS/MS	By MS/MS	By MS/MS	By MS/MS	By MS/MS	By MS/MS	By matchin	By MS/MS
44.225	By MS/MS	By MS/MS	By MS/MS	By MS/MS	By MS/MS	By MS/MS	By MS/MS	By MS/MS
3.8203	By MS/MS	By MS/MS	By MS/MS	By MS/MS	By MS/MS	By MS/MS	By MS/MS	By matchin
28.151	By matchin	By matchin	By matchin	By matching		By MS/MS	By MS/MS	By MS/MS
3.0273		By matching		By matching			By MS/MS	By matchin
2.4288						By matchin	By MS/MS	By matchin
10.125	By matchin	By MS/MS	By MS/MS	By MS/MS	By MS/MS	By MS/MS	By MS/MS	By MS/MS
2.4598			By matching		By MS/MS			By matchin
5.5404	By matchin	By matchin	By matchin	By MS/MS	By matchin	By MS/MS	By MS/MS	By MS/MS
1.9103	By matchin	By matchin	By matchin	By MS/MS	By MS/MS	By MS/MS		
1.9581			By matching			By MS/MS	By MS/MS	By matchin
6.5742	By MS/MS	By MS/MS	By MS/MS	By MS/MS	By MS/MS	By MS/MS	By MS/MS	By MS/MS
9.3628	By MS/MS	By MS/MS	By MS/MS	By MS/MS	By MS/MS	By MS/MS	By MS/MS	By MS/MS
49.97	By MS/MS	By MS/MS	By MS/MS	By MS/MS	By MS/MS	By MS/MS	By MS/MS	By MS/MS
7.5468	By MS/MS	By MS/MS	By MS/MS	By MS/MS	By MS/MS	By MS/MS	By MS/MS	By MS/MS
22.084	By MS/MS	By MS/MS	By MS/MS	By MS/MS	By MS/MS	By MS/MS	By MS/MS	By MS/MS
4.7124	By matchin	By MS/MS	By MS/MS	By MS/MS	By matchin	By matchin	By matchin	By MS/MS
27.378	By MS/MS	By MS/MS	By MS/MS	By MS/MS	By MS/MS	By MS/MS	By MS/MS	By MS/MS

3.5646	By matchin	By MS/MS	By MS/MS	By matchin	By matchin	By matchin	By matchin	By matchin
1.627	By matchin	By matchin	By matchin	By matchin	By MS/MS	By matching		By matchin
1.9119	By MS/MS	By MS/MS	By MS/MS	By MS/MS	By MS/MS	By matchin	By matchin	By matchin
-2	By matchin	By matchin	By matchin	By matchin	By matchin	By matchin	By matchin	By MS/MS
-2	By MS/MS	By matchin	By matchin	By matchin	By matchin	By matchin	By matchin	By matchin
-2	By matchin	By matchin	By matchin	By matchin	By MS/MS	By matchin	By matchin	By matchin
57.238	By MS/MS	By MS/MS	By MS/MS	By MS/MS	By MS/MS	By MS/MS	By MS/MS	By MS/MS
6.6266	By MS/MS	By MS/MS	By MS/MS	By MS/MS	By MS/MS	By MS/MS	By MS/MS	By MS/MS
40.277	By MS/MS	By MS/MS	By MS/MS	By MS/MS	By MS/MS	By MS/MS	By MS/MS	By MS/MS
24.948	By MS/MS	By MS/MS	By MS/MS	By MS/MS	By MS/MS	By MS/MS	By MS/MS	By MS/MS
18.06	By matchin	By matchin	By matchin	By matchin	By matchin	By MS/MS	By MS/MS	By MS/MS
22.038	By MS/MS	By MS/MS	By MS/MS	By matchin	By matchin	By MS/MS	By MS/MS	By MS/MS
143.43	By MS/MS	By MS/MS	By MS/MS	By MS/MS	By MS/MS	By MS/MS	By MS/MS	By MS/MS
1.729	By MS/MS	By MS/MS	By MS/MS	By MS/MS	By matchin	By MS/MS	By MS/MS	By MS/MS
18.584	By MS/MS	By MS/MS	By MS/MS	By MS/MS	By MS/MS	By MS/MS	By MS/MS	By MS/MS
30.266	By MS/MS	By MS/MS	By MS/MS	By MS/MS	By MS/MS	By MS/MS	By MS/MS	By MS/MS
4.232	By MS/MS	By matchin	By MS/MS	By MS/MS	By MS/MS	By MS/MS	By MS/MS	By matchin
13.781	By MS/MS	By MS/MS	By MS/MS	By MS/MS	By MS/MS	By MS/MS	By MS/MS	By MS/MS
12.277	By matchin	By matchin	By matchin	By matchin	By MS/MS	By MS/MS	By MS/MS	By MS/MS
3.2884	By MS/MS	By MS/MS	By matchin	By MS/MS	By MS/MS	By matchin	By matchin	By matchin
6.0481	By MS/MS	By MS/MS	By MS/MS	By MS/MS	By MS/MS	By MS/MS	By MS/MS	By matchin
4.4099	By matchin	By MS/MS	By MS/MS	By MS/MS		By MS/MS	By matchin	By matchin
23.284	By MS/MS	By matchin	By matchin	By MS/MS	By matchin	By MS/MS	By MS/MS	By MS/MS
75.013	By MS/MS	By MS/MS	By MS/MS	By MS/MS	By MS/MS	By MS/MS	By MS/MS	By MS/MS
8.2558	By matching		By matchin	By matchin	By MS/MS	By MS/MS	By MS/MS	By MS/MS
29.671	By MS/MS	By MS/MS	By MS/MS	By MS/MS	By MS/MS	By MS/MS	By MS/MS	By MS/MS
11.88	By MS/MS	By MS/MS	By MS/MS	By MS/MS		By MS/MS	By MS/MS	By MS/MS
42.991	By MS/MS	By MS/MS	By MS/MS	By MS/MS	By MS/MS	By MS/MS	By MS/MS	By MS/MS
12.142	By MS/MS	By matchin	By MS/MS	By MS/MS	By matchin	By MS/MS	By MS/MS	By MS/MS
2.075	By matchin	By matchin	By MS/MS	By MS/MS	By MS/MS	By MS/MS	By matchin	By MS/MS
9.6458	By matchin	By MS/MS	By matchin	By MS/MS		By MS/MS	By MS/MS	By MS/MS
62.241	By MS/MS	By MS/MS	By MS/MS	By MS/MS	By MS/MS	By MS/MS	By MS/MS	By MS/MS
55.118	By matching		By MS/MS	By matchin	By matchin	By MS/MS	By MS/MS	By MS/MS
12.169	By matchin	By matchin	By matchin	By matchin	By matchin	By MS/MS	By MS/MS	By MS/MS
4.0109	By matching				By matchin	By MS/MS	By MS/MS	
2.4281	By matchin	By MS/MS	By matchin	By matchin	By MS/MS	By matchin	By matchin	By matchin
2.6418	By matchin	By matchin	By MS/MS	By MS/MS	By MS/MS	By matchin	By matchin	By matchin
3.8858	By MS/MS	By matchin	By MS/MS	By matchin	By MS/MS	By matchin	By matchin	By MS/MS
3.9889	By matchin	By matchin	By matchin	By matchin	By MS/MS		By matchin	By matchin
5.6105		By matchin	By MS/MS	By matchin	By matchin	By MS/MS	By matching	
24.57	By MS/MS	By matchin	By MS/MS	By MS/MS	By MS/MS	By MS/MS	By MS/MS	By MS/MS
3.3942	By matching			By matchin	By matchin	By matchin	By matchin	By MS/MS
46.405	By MS/MS	By MS/MS	By MS/MS	By MS/MS	By MS/MS	By MS/MS	By MS/MS	By MS/MS
20.997	By MS/MS	By MS/MS	By MS/MS	By MS/MS	By MS/MS	By matchin	By matchin	By MS/MS
13.718	By MS/MS	By MS/MS	By matchin	By matchin	By matchin	By MS/MS	By matchin	By matchin
53.558	By MS/MS	By MS/MS	By MS/MS	By MS/MS	By MS/MS	By MS/MS	By MS/MS	By MS/MS
1.8764	By MS/MS	By matchin	By MS/MS	By MS/MS	By MS/MS	By MS/MS	By matching	
57.445	By MS/MS	By MS/MS	By MS/MS	By MS/MS	By MS/MS	By MS/MS	By MS/MS	By MS/MS
8.1894	By MS/MS	By MS/MS	By MS/MS	By MS/MS	By MS/MS	By MS/MS	By MS/MS	By MS/MS
141.18	By MS/MS	By MS/MS	By MS/MS	By MS/MS	By MS/MS	By MS/MS	By MS/MS	By MS/MS

11.505	By MS/MS	By MS/MS	By MS/MS	By MS/MS	By MS/MS	By matchin	By matchin	By matchin
2.3268	By MS/MS	By MS/MS	By MS/MS	By MS/MS	By matchin	By matchin	By MS/MS	By MS/MS
39.119	By MS/MS	By MS/MS	By MS/MS	By MS/MS	By MS/MS	By MS/MS	By MS/MS	By MS/MS
136.42	By MS/MS	By MS/MS	By MS/MS	By MS/MS	By MS/MS	By MS/MS	By MS/MS	By MS/MS
2.36	By MS/MS	By MS/MS	By MS/MS	By MS/MS	By MS/MS	By matchin	By matchin	By matchin
4.9855	By matchin	By MS/MS	By matchin	By matchin	By MS/MS	By matchin	By matchin	By matchin
3.1272			By matchin	By matching		By MS/MS	By MS/MS	By MS/MS
7.6287			By MS/MS	By MS/MS		By MS/MS	By MS/MS	By MS/MS
9.1703	By MS/MS	By MS/MS	By matchin	By matchin	By matchin	By MS/MS	By MS/MS	By MS/MS
39.652	By matchin	By matchin	By MS/MS	By MS/MS	By MS/MS	By MS/MS	By MS/MS	By MS/MS
95.436	By matchin	By matchin	By MS/MS	By MS/MS	By MS/MS	By MS/MS	By MS/MS	By MS/MS
1.9752		By MS/MS	By matching					By MS/MS
1.9681						By matchin	By MS/MS	By matchin
38.908	By MS/MS	By MS/MS	By MS/MS	By MS/MS	By MS/MS	By MS/MS	By MS/MS	By MS/MS
19.295	By MS/MS	By MS/MS	By MS/MS	By matchin	By MS/MS	By matchin	By MS/MS	By MS/MS
23.549	By matchin	By matchin	By MS/MS	By MS/MS	By MS/MS	By MS/MS	By MS/MS	By MS/MS
62.518	By MS/MS	By MS/MS	By MS/MS	By MS/MS	By matchin	By MS/MS	By MS/MS	By MS/MS
15.189	By MS/MS	By MS/MS	By MS/MS	By MS/MS	By MS/MS	By MS/MS	By MS/MS	By MS/MS
10.303	By matchin	By matchin	By matchin	By matchin	By matchin	By matchin	By MS/MS	By MS/MS
111.32	By MS/MS	By MS/MS	By MS/MS	By MS/MS	By MS/MS	By MS/MS	By MS/MS	By MS/MS
19.971	By MS/MS	By MS/MS	By MS/MS	By MS/MS	By MS/MS	By MS/MS	By MS/MS	By MS/MS
3.3442	By matchin	By MS/MS	By MS/MS	By MS/MS	By MS/MS	By MS/MS	By MS/MS	By matchin
7.0358	By matchin	By MS/MS	By MS/MS	By MS/MS	By MS/MS	By MS/MS	By MS/MS	By matchin
156.57	By MS/MS	By MS/MS	By MS/MS	By MS/MS	By MS/MS	By MS/MS	By MS/MS	By MS/MS
17.922	By MS/MS	By MS/MS	By MS/MS	By MS/MS	By MS/MS	By MS/MS	By MS/MS	By MS/MS
285.94	By MS/MS	By MS/MS	By MS/MS	By MS/MS	By MS/MS	By MS/MS	By MS/MS	By MS/MS
215.79	By MS/MS	By MS/MS	By MS/MS	By MS/MS	By MS/MS	By MS/MS	By MS/MS	By MS/MS
17.703	By matchin	By matchin	By matchin	By matchin	By MS/MS	By matchin	By MS/MS	By MS/MS
34.822	By matchin	By MS/MS	By MS/MS	By MS/MS	By matchin	By matchin	By MS/MS	By MS/MS
1.9963	By matchin	By matchin	By matchin	By matchin	By matchin	By MS/MS	By matchin	By MS/MS
101.62	By MS/MS	By MS/MS	By MS/MS	By MS/MS	By MS/MS	By MS/MS	By MS/MS	By MS/MS
50.703	By MS/MS	By MS/MS	By MS/MS	By MS/MS	By MS/MS	By MS/MS	By MS/MS	By MS/MS
1.6831	By matchin	By matchin	By MS/MS	By matchin	By MS/MS	By MS/MS	By matchin	By MS/MS
276.01	By MS/MS	By MS/MS	By MS/MS	By MS/MS	By MS/MS	By MS/MS	By MS/MS	By MS/MS
162.87	By MS/MS	By MS/MS	By MS/MS	By MS/MS	By MS/MS	By MS/MS	By MS/MS	By MS/MS
5.2579	By MS/MS	By MS/MS	By matchin	By matching		By MS/MS	By MS/MS	By MS/MS
9.0435			By matchin	By matchin	By MS/MS	By MS/MS	By matchin	By MS/MS
22.173	By MS/MS	By MS/MS	By matchin	By matchin	By MS/MS	By MS/MS	By MS/MS	By MS/MS
1.962	By matchin	By MS/MS	By MS/MS	By matchin	By MS/MS	By matching		
12.743	By matchin	By MS/MS	By MS/MS	By MS/MS	By MS/MS	By matchin	By matchin	By matchin
1.9317	By matchin	By matching		By MS/MS	By MS/MS			
58.1	By MS/MS	By MS/MS	By MS/MS	By MS/MS	By MS/MS	By MS/MS	By MS/MS	By MS/MS
1.7574						By MS/MS	By MS/MS	
9.0563	By matchin	By matchin	By matching		By matchin	By MS/MS	By MS/MS	By MS/MS
22.117	By matchin	By MS/MS	By MS/MS	By MS/MS	By MS/MS	By MS/MS	By MS/MS	By MS/MS
7.041	By MS/MS	By MS/MS	By MS/MS	By MS/MS	By matchin	By matchin	By matchin	By matchin
66.115	By MS/MS	By MS/MS	By MS/MS	By MS/MS	By MS/MS	By MS/MS	By MS/MS	By MS/MS
33.239	By MS/MS	By MS/MS	By MS/MS	By MS/MS	By MS/MS	By MS/MS	By MS/MS	By MS/MS
205.13	By MS/MS	By MS/MS	By MS/MS	By MS/MS	By MS/MS	By MS/MS	By MS/MS	By MS/MS
115.19	By MS/MS	By MS/MS	By MS/MS	By MS/MS	By MS/MS	By MS/MS	By MS/MS	By MS/MS

5.2888	By matchin	By MS/MS	By MS/MS	By MS/MS				By matchin
8.9105	By MS/MS	By MS/MS	By matchin	By matchin	By matching			
31.995	By MS/MS	By MS/MS	By MS/MS	By MS/MS	By MS/MS	By matchin	By MS/MS	By MS/MS
7.8649	By MS/MS	By MS/MS	By MS/MS	By MS/MS	By MS/MS	By matchin	By matchin	By matchin
13.958	By MS/MS	By MS/MS	By MS/MS	By MS/MS	By MS/MS	By matchin	By MS/MS	By matchin
2.1197			By matchin	By matching		By MS/MS	By MS/MS	By MS/MS
9.9724	By MS/MS	By MS/MS	By MS/MS	By MS/MS	By MS/MS	By MS/MS	By MS/MS	By MS/MS
4.0763	By MS/MS	By matchin	By MS/MS	By matchin	By matchin	By matching		By matchin
60.936	By MS/MS	By MS/MS	By MS/MS	By MS/MS	By MS/MS	By MS/MS	By MS/MS	By MS/MS
7.0754						By matchin	By matchin	By MS/MS
4.2931	By MS/MS	By MS/MS	By MS/MS	By MS/MS	By matchin	By matchin	By matchin	By MS/MS
3.1286	By matchin	By MS/MS	By matchin	By matchin	By matchin	By matchin	By matchin	By matchin
19.911	By MS/MS	By MS/MS	By MS/MS	By MS/MS	By MS/MS	By MS/MS	By matchin	By MS/MS
98.959	By MS/MS	By MS/MS	By MS/MS	By MS/MS	By MS/MS	By MS/MS	By MS/MS	By MS/MS
8.9542	By matchin	By matchin	By matchin	By matchin	By matchin	By MS/MS	By MS/MS	By MS/MS
1.5162	By MS/MS	By MS/MS	By MS/MS	By MS/MS	By MS/MS	By MS/MS	By matchin	By matchin
14.775	By matchin	By matchin	By matchin	By matchin	By matchin	By MS/MS		By MS/MS
82.259	By MS/MS	By MS/MS	By MS/MS	By MS/MS	By MS/MS	By MS/MS	By MS/MS	By MS/MS
7.5927	By MS/MS	By matchin	By matchin	By MS/MS	By matchin	By MS/MS	By MS/MS	By MS/MS
7.2245	By matchin	By matchin	By MS/MS	By MS/MS	By matchin	By matching		
4.797	By matching		By matchin	By matchin	By matchin	By MS/MS	By MS/MS	By MS/MS
5.0567	By MS/MS	By MS/MS	By MS/MS	By MS/MS	By MS/MS	By MS/MS	By MS/MS	By MS/MS
11.254	By matchin	By matchin	By matchin	By MS/MS	By MS/MS	By MS/MS	By MS/MS	By MS/MS
20.647	By MS/MS	By MS/MS	By MS/MS	By MS/MS	By MS/MS	By MS/MS	By MS/MS	By MS/MS
29.03	By matchin	By MS/MS	By MS/MS	By MS/MS	By MS/MS	By MS/MS	By MS/MS	By MS/MS
4.2657	By matchin	By MS/MS	By matchin	By matchin	By MS/MS	By matching		By MS/MS
101.76	By MS/MS	By MS/MS	By MS/MS	By MS/MS	By MS/MS	By MS/MS	By MS/MS	By MS/MS
18.961	By matchin	By matchin	By MS/MS	By MS/MS	By MS/MS	By MS/MS	By MS/MS	By MS/MS
14.918	By MS/MS	By MS/MS	By MS/MS	By MS/MS	By MS/MS	By MS/MS	By matchin	By MS/MS
5.9565	By MS/MS	By MS/MS	By MS/MS	By MS/MS	By MS/MS	By MS/MS	By matchin	By matchin
33.58	By MS/MS	By MS/MS	By MS/MS	By MS/MS	By MS/MS	By MS/MS	By MS/MS	By MS/MS
3.3706	By MS/MS	By matchin	By matchin	By matchin	By MS/MS	By matchin	By matchin	By matchin
5.5449	By matchin	By MS/MS		By matching		By MS/MS	By MS/MS	By MS/MS
12.495	By MS/MS	By MS/MS	By MS/MS	By MS/MS	By MS/MS	By MS/MS	By MS/MS	By MS/MS
8.9756	By MS/MS		By matchin	By MS/MS	By MS/MS			
5.7777	By matchin	By MS/MS	By MS/MS	By matchin	By matchin	By matchin	By MS/MS	By MS/MS
11.255			By MS/MS	By MS/MS		By MS/MS	By MS/MS	By MS/MS
1.7031		By matchin	By matchin	By MS/MS	By matchin	By matchin	By matchin	By matchin
38.582	By matchin	By matchin	By matchin	By MS/MS	By MS/MS	By MS/MS	By MS/MS	By MS/MS
4.027	By MS/MS	By MS/MS	By MS/MS	By MS/MS	By MS/MS	By MS/MS	By MS/MS	By MS/MS
4.8211	By matchin	By MS/MS	By MS/MS	By MS/MS	By MS/MS	By MS/MS		By matchin
5.5046	By matchin	By MS/MS	By matchin	By MS/MS	By MS/MS			
1.6831	By MS/MS	By MS/MS	By MS/MS	By MS/MS	By matchin	By matchin	By matching	
10.42	By MS/MS	By MS/MS	By MS/MS	By MS/MS	By MS/MS	By MS/MS	By MS/MS	By MS/MS
1.729	By matchin	By matchin	By MS/MS	By matchin	By MS/MS	By matching		
7.6818	By MS/MS	By MS/MS	By MS/MS	By MS/MS	By MS/MS	By MS/MS	By MS/MS	By MS/MS
3.0369	By MS/MS	By MS/MS	By MS/MS	By MS/MS		By MS/MS	By MS/MS	By MS/MS
2.8924	By MS/MS	By MS/MS	By matchin	By matchin	By matchin	By matchin	By matchin	By matchin
4.2656	By matchin	By matchin	By matchin	By MS/MS	By MS/MS	By MS/MS	By MS/MS	By MS/MS
10.783	By matchin	By matchin	By matchin	By matchin	By matchin	By MS/MS	By MS/MS	By MS/MS

4.469	By MS/MS	By matchin	By MS/MS	By MS/MS	By MS/MS	By MS/MS	By matchin	By MS/MS
6.3307	By matchin	By matchin	By matchin	By MS/MS	By MS/MS	By MS/MS		By matchin
1.659		By matchin	By matchin	By MS/MS		By MS/MS		By matchin
2.0856	By matchin	By MS/MS	By matchin	By MS/MS	By matchin	By MS/MS	By MS/MS	By MS/MS
3.5161	By MS/MS	By matchin	By MS/MS	By MS/MS	By MS/MS	By MS/MS	By matchin	By MS/MS
24.939	By MS/MS	By MS/MS	By MS/MS	By MS/MS	By MS/MS	By MS/MS	By MS/MS	By MS/MS
16.851	By MS/MS	By MS/MS	By matchin	By MS/MS	By MS/MS	By MS/MS	By MS/MS	By MS/MS
148.15	By MS/MS	By MS/MS	By MS/MS	By MS/MS	By MS/MS	By MS/MS	By MS/MS	By MS/MS
14.527	By MS/MS	By MS/MS	By MS/MS	By MS/MS	By MS/MS	By MS/MS	By MS/MS	By matchin
2.2884						By MS/MS	By MS/MS	By matchin
1.7186								By MS/MS
3.0338	By matchin	By matchin	By MS/MS	By MS/MS	By MS/MS	By MS/MS	By matchin	By MS/MS
13.697	By MS/MS	By MS/MS	By MS/MS	By MS/MS	By MS/MS	By MS/MS	By matchin	By MS/MS
2.9588						By MS/MS	By MS/MS	
5.9972				By MS/MS		By MS/MS	By MS/MS	By MS/MS
33.997	By MS/MS	By MS/MS	By MS/MS	By MS/MS	By MS/MS	By MS/MS	By MS/MS	By MS/MS
24.049	By MS/MS	By matchin	By MS/MS	By MS/MS	By matchin	By matchin	By matchin	By MS/MS
28.089	By matchin	By matchin	By matchin	By MS/MS	By MS/MS	By matchin	By matchin	By matchin
-2					By MS/MS			
4.1741	By MS/MS	By MS/MS	By MS/MS	By matchin	By MS/MS	By MS/MS	By MS/MS	By MS/MS
4.601		By matchin	By MS/MS	By matchin	By MS/MS			
12.225	By MS/MS	By MS/MS	By MS/MS	By MS/MS	By MS/MS	By MS/MS	By matchin	By MS/MS
8.0955	By MS/MS	By MS/MS	By MS/MS	By MS/MS	By matchin	By MS/MS	By MS/MS	By matchin
11.426	By MS/MS	By MS/MS	By MS/MS	By MS/MS	By MS/MS	By MS/MS	By MS/MS	By matchin
1.634	By matchin	By MS/MS	By matching		By matching			
9.5257	By MS/MS	By MS/MS	By MS/MS	By MS/MS	By MS/MS	By MS/MS	By MS/MS	By MS/MS
2.7928	By MS/MS	By matchin	By matchin	By matchin	By MS/MS	By MS/MS	By matchin	By matchin
14.626	By matchin	By matchin	By matchin	By matchin	By MS/MS	By MS/MS	By MS/MS	By MS/MS
8.353	By MS/MS	By MS/MS	By MS/MS	By MS/MS	By MS/MS	By MS/MS	By MS/MS	By MS/MS
5.5939	By MS/MS	By MS/MS	By MS/MS	By MS/MS	By MS/MS	By MS/MS	By matchin	By matchin
4.8351	By MS/MS	By MS/MS	By matchin	By matchin	By matchin	By matchin	By matchin	By matchin
1.9029			By matchin	By matching		By matchin	By MS/MS	By MS/MS
4.8581	By MS/MS	By MS/MS	By MS/MS	By MS/MS	By matchin	By matchin	By matchin	By MS/MS
29.34			By matchin	By matchin	By MS/MS	By MS/MS	By MS/MS	By MS/MS
13.41	By MS/MS	By MS/MS	By matchin	By MS/MS	By matchin	By MS/MS	By MS/MS	By MS/MS
2.4845		By matchin	By matching		By matchin	By matchin	By matchin	By MS/MS
5.7255	By MS/MS	By MS/MS	By MS/MS	By MS/MS	By MS/MS	By matchin	By matchin	By matchin
4.4417	By MS/MS	By MS/MS	By MS/MS	By MS/MS	By MS/MS	By matchin	By matchin	By matchin
1.8671		By matchin	By matchin	By matchin	By matching		By MS/MS	By matchin
2.1173	By MS/MS	By matchin	By matchin	By matchin	By matchin	By matching		
1.8236	By matchin	By matchin	By matchin	By matching		By MS/MS	By matchin	By MS/MS
1.8329	By matchin	By MS/MS	By MS/MS	By MS/MS	By matchin	By MS/MS		By matchin
9.6447	By MS/MS	By MS/MS	By MS/MS	By MS/MS	By MS/MS	By MS/MS	By MS/MS	By MS/MS
1.7557	By matchin	By MS/MS	By MS/MS	By matchin	By matchin	By matching		By matchin
41.798	By MS/MS	By MS/MS	By MS/MS	By MS/MS	By MS/MS	By MS/MS	By MS/MS	By MS/MS
2.747	By MS/MS	By MS/MS	By matchin	By MS/MS	By MS/MS	By matchin	By MS/MS	By matchin
6.8411	By MS/MS	By MS/MS	By matchin	By MS/MS	By matchin	By MS/MS	By MS/MS	By MS/MS
-2						By matchin	By MS/MS	
33.911		By matchin	By matchin	By matchin	By matchin	By MS/MS	By MS/MS	By MS/MS
5.6768		By matchin	By matchin	By matchin	By matchin	By matchin	By MS/MS	By MS/MS

2.3803	By MS/MS	By matchin	By matchin	By MS/MS	By matchin	By matchin	By matching	
1.6365	By matching			By matching		By matchin	By MS/MS	By matchin
26.398	By MS/MS	By MS/MS	By MS/MS	By MS/MS	By MS/MS	By MS/MS	By MS/MS	By MS/MS
2.8151	By matchin	By matchin	By MS/MS	By matchin	By matchin	By MS/MS	By matchin	By matchin
23.814	By MS/MS	By MS/MS	By MS/MS	By MS/MS	By MS/MS	By MS/MS	By MS/MS	By MS/MS
5.4005	By matchin	By matchin	By MS/MS	By MS/MS	By matchin	By MS/MS	By matchin	By matchin
7.9352	By MS/MS	By matchin	By MS/MS	By MS/MS	By MS/MS	By MS/MS	By matchin	By MS/MS
10.586	By MS/MS	By MS/MS	By MS/MS	By matchin	By MS/MS	By MS/MS	By matchin	By MS/MS
2.2048	By matchin	By MS/MS	By matchin	By matchin	By matchin	By matchin	By matchin	By matchin
4.1205	By matchin	By matchin	By MS/MS	By matchin	By matchin	By matchin	By MS/MS	By matchin
8.096	By MS/MS	By MS/MS	By MS/MS	By MS/MS	By matchin	By MS/MS	By matchin	By MS/MS
7.7816	By MS/MS	By MS/MS				By MS/MS	By MS/MS	By MS/MS
17.374	By MS/MS	By MS/MS	By MS/MS	By MS/MS	By MS/MS	By MS/MS	By MS/MS	By MS/MS
4.0978	By matchin	By matchin	By MS/MS	By matchin	By matchin	By MS/MS	By matchin	By matchin
7.0546	By MS/MS	By MS/MS	By MS/MS	By MS/MS	By MS/MS	By MS/MS	By matchin	By MS/MS
2.5864	By MS/MS	By MS/MS	By MS/MS	By MS/MS		By MS/MS	By MS/MS	By MS/MS
86.415	By MS/MS	By MS/MS	By MS/MS	By MS/MS	By MS/MS	By MS/MS	By MS/MS	By MS/MS
49.92	By MS/MS	By matchin	By matchin	By MS/MS	By matchin	By MS/MS	By matchin	By MS/MS
145.11	By MS/MS	By MS/MS	By MS/MS	By MS/MS	By MS/MS	By MS/MS	By MS/MS	By MS/MS
14.52	By MS/MS	By MS/MS	By MS/MS	By MS/MS	By MS/MS	By MS/MS	By MS/MS	By MS/MS
2.4508		By MS/MS					By matchin	By matchin
1.555	By matchin	By MS/MS	By MS/MS	By matchin	By MS/MS			By matchin
33.061	By MS/MS	By MS/MS	By MS/MS	By MS/MS	By matchin	By MS/MS	By MS/MS	By MS/MS
9.7049	By MS/MS	By MS/MS	By MS/MS	By MS/MS	By MS/MS	By MS/MS	By MS/MS	By MS/MS
14.343	By MS/MS	By MS/MS	By MS/MS	By matchin	By MS/MS	By MS/MS	By MS/MS	By MS/MS
3.5817						By matchin	By MS/MS	By MS/MS
7.9929	By MS/MS	By MS/MS	By MS/MS	By MS/MS	By matchin	By MS/MS	By MS/MS	By MS/MS
3.9052	By matchin	By matchin	By matchin	By matchin	By matchin	By matchin	By MS/MS	By MS/MS
28.343	By MS/MS	By MS/MS	By MS/MS	By MS/MS	By MS/MS	By MS/MS	By MS/MS	By MS/MS
89.453	By matchin	By matchin	By MS/MS	By MS/MS	By MS/MS	By MS/MS	By MS/MS	By MS/MS
9.6415	By matchin	By MS/MS	By matchin	By matchin	By MS/MS	By MS/MS	By matchin	By MS/MS
3.777	By MS/MS	By MS/MS	By MS/MS	By MS/MS	By MS/MS	By matchin	By matchin	By matchin
2.4347	By matchin	By matchin	By matchin	By matchin	By MS/MS	By matchin	By matchin	By MS/MS
24.162	By MS/MS	By MS/MS	By MS/MS	By MS/MS	By MS/MS	By MS/MS	By MS/MS	By MS/MS
6.7383	By matchin	By matchin	By MS/MS	By matchin	By matchin	By matchin	By MS/MS	By MS/MS
1.8545		By matchin	By MS/MS	By MS/MS	By MS/MS	By matching		
2.9816	By matchin	By matchin	By MS/MS	By matchin	By matchin	By MS/MS	By matching	
16.147	By matchin	By matchin	By matchin	By MS/MS	By matchin	By MS/MS	By MS/MS	By MS/MS
2.0991	By matchin	By matching		By matchin	By matchin	By matchin	By MS/MS	By MS/MS
117.78	By MS/MS	By MS/MS	By MS/MS	By MS/MS	By MS/MS	By MS/MS	By MS/MS	By MS/MS
3.584	By MS/MS	By matchin	By MS/MS	By matchin	By MS/MS	By MS/MS	By matchin	By matchin
7.5913	By MS/MS	By MS/MS	By MS/MS	By MS/MS	By MS/MS	By MS/MS	By matchin	By matchin
4.0153	By matchin	By MS/MS	By MS/MS	By MS/MS	By MS/MS			By MS/MS
2.6219	By matchin	By matchin	By MS/MS	By matchin	By matchin	By matchin	By matchin	By MS/MS
261.06	By MS/MS	By MS/MS	By MS/MS	By MS/MS	By MS/MS	By MS/MS	By MS/MS	By MS/MS
1.7908	By matchin	By MS/MS		By matchin	By matchin	By MS/MS		By matchin
26.579	By MS/MS	By MS/MS	By MS/MS	By MS/MS	By MS/MS	By MS/MS	By MS/MS	By MS/MS
4.036	By matchin	By MS/MS	By MS/MS	By matchin	By matchin	By matching		By matchin
38.589	By MS/MS	By MS/MS	By MS/MS	By MS/MS	By MS/MS	By MS/MS	By MS/MS	By MS/MS
1.8436	By MS/MS	By MS/MS	By MS/MS	By MS/MS	By matchin	By MS/MS	By matchin	By MS/MS

1.7167	By matchin	By MS/MS	By matchin	By matchin	By matchin	By matchin	By matchin	By matchin
4.7583	By matchin	By MS/MS	By matchin	By matchin	By MS/MS	By MS/MS	By MS/MS	By MS/MS
323.31	By MS/MS	By MS/MS	By MS/MS	By MS/MS	By MS/MS	By MS/MS	By MS/MS	By MS/MS
5.1821			By matchin	By matchin	By matchin	By matchin	By MS/MS	By MS/MS
123.94	By MS/MS	By MS/MS	By MS/MS	By MS/MS	By MS/MS	By MS/MS	By MS/MS	By MS/MS
1.926						By MS/MS	By MS/MS	By MS/MS
3.5151	By MS/MS	By MS/MS	By MS/MS	By MS/MS	By MS/MS		By matchin	By matchin
35.019	By MS/MS	By MS/MS	By MS/MS	By MS/MS	By MS/MS	By MS/MS	By MS/MS	By MS/MS
4.1992	By MS/MS	By MS/MS	By matchin	By matching		By matchin	By matchin	By MS/MS
8.2756	By matchin	By MS/MS	By matchin	By MS/MS	By MS/MS	By MS/MS	By matchin	By matchin
8.4033	By MS/MS	By matchin	By matchin	By MS/MS	By MS/MS	By MS/MS	By MS/MS	By MS/MS
6.5027	By MS/MS	By MS/MS	By MS/MS	By MS/MS	By matchin	By MS/MS	By MS/MS	By matchin
2.3603	By matchin	By MS/MS	By MS/MS	By matchin	By matchin	By matchin	By matchin	By matchin
1.5192	By matchin	By MS/MS	By matchin	By matchin	By matchin	By matching		
2.0961	By MS/MS	By MS/MS					By matching	
3.0172	By MS/MS	By MS/MS	By matchin	By MS/MS	By MS/MS	By matchin	By matchin	By MS/MS
3.888	By MS/MS	By MS/MS	By matchin	By matchin	By MS/MS	By matching		By matchin
6.7958		By MS/MS	By matchin	By matching				By MS/MS
3.2038	By MS/MS	By matchin	By MS/MS	By MS/MS	By MS/MS	By matching		
1.7305	By MS/MS	By MS/MS	By matchin	By matching		By matchin	By matchin	By matchin
54.419	By MS/MS	By MS/MS	By MS/MS	By MS/MS	By MS/MS	By MS/MS	By MS/MS	By MS/MS
1.5034						By MS/MS	By matching	
3.4987	By matchin	By matchin	By MS/MS	By MS/MS	By MS/MS	By matchin	By matchin	By matchin
9.998	By MS/MS	By MS/MS	By MS/MS	By MS/MS	By MS/MS	By MS/MS	By MS/MS	By MS/MS
3.3085	By matching		By matchin	By matching		By matchin	By MS/MS	By matchin
8.3504	By matchin	By matchin	By matchin	By MS/MS	By matchin	By MS/MS	By MS/MS	By MS/MS
1.525				By matchin	By matching			By MS/MS
1.711	By MS/MS					By MS/MS		
6.8879	By MS/MS	By MS/MS	By MS/MS	By matchin	By MS/MS	By MS/MS	By MS/MS	By MS/MS
5.7975					By matchin	By MS/MS	By MS/MS	By MS/MS
3.5166	By matchin	By matchin	By MS/MS	By matchin	By MS/MS		By matching	
323.31	By MS/MS	By MS/MS	By MS/MS	By MS/MS	By MS/MS	By MS/MS	By MS/MS	By MS/MS
2.3068	By matching		By matching				By MS/MS	By MS/MS
6.2507	By MS/MS	By MS/MS	By MS/MS	By matchin	By matchin	By matchin	By matchin	By MS/MS
154.11	By MS/MS	By MS/MS	By MS/MS	By MS/MS	By MS/MS	By MS/MS	By MS/MS	By MS/MS
16.05	By MS/MS	By MS/MS	By MS/MS	By MS/MS	By MS/MS	By MS/MS	By MS/MS	By matchin
3.3653	By matchin	By MS/MS	By matchin	By MS/MS	By MS/MS	By matchin	By matchin	By matchin
47.375	By MS/MS	By MS/MS	By MS/MS	By MS/MS	By MS/MS	By MS/MS	By MS/MS	By MS/MS
14.845	By MS/MS	By MS/MS	By matchin	By MS/MS	By MS/MS	By matchin	By MS/MS	By MS/MS
11.88	By MS/MS	By MS/MS	By MS/MS	By MS/MS	By MS/MS	By MS/MS	By MS/MS	By MS/MS
2.5322	By matchin	By MS/MS	By MS/MS	By matchin	By matchin	By MS/MS	By matching	
8.5308	By MS/MS	By MS/MS	By MS/MS	By MS/MS	By MS/MS	By MS/MS	By MS/MS	By MS/MS
2.9937	By matching			By matching		By MS/MS	By MS/MS	By MS/MS
7.4316	By MS/MS	By MS/MS	By MS/MS	By MS/MS	By MS/MS	By MS/MS		By matchin
15.194	By matchin	By matchin	By matchin	By matchin	By matchin	By MS/MS	By MS/MS	By MS/MS
2.3207				By matching		By MS/MS	By matchin	By MS/MS
3.0343	By MS/MS	By MS/MS	By MS/MS	By MS/MS	By MS/MS			
10.703	By MS/MS	By MS/MS	By MS/MS	By MS/MS	By MS/MS	By MS/MS	By matchin	By MS/MS
6.0681					By MS/MS		By MS/MS	By MS/MS
67.447	By MS/MS	By MS/MS	By MS/MS	By MS/MS	By MS/MS	By MS/MS	By MS/MS	By MS/MS

8.1261	By MS/MS	By MS/MS	By MS/MS	By MS/MS	By matchin	By MS/MS	By MS/MS	By MS/MS
3.8562	By MS/MS	By matchin	By matchin	By matchin	By MS/MS	By matchin	By matchin	By MS/MS
35.728			By MS/MS	By MS/MS	By MS/MS	By MS/MS	By MS/MS	By MS/MS
3.1142	By matchin	By MS/MS	By MS/MS	By matchin	By MS/MS	By MS/MS	By MS/MS	
3.2415				By matchin	By matchin	By MS/MS	By MS/MS	By MS/MS
8.6071	By MS/MS	By MS/MS	By matchin	By matchin	By MS/MS	By MS/MS	By MS/MS	By MS/MS
2.4984			By matchin	By matchin	By MS/MS	By MS/MS	By matchin	By MS/MS
14.199		By matchin	By matching		By matchin	By matchin	By MS/MS	By MS/MS
4.7584	By matchin	By MS/MS	By MS/MS	By matchin	By matchin	By matchin	By matchin	By MS/MS
9.8431	By matchin	By matchin	By matchin	By matchin	By MS/MS	By MS/MS	By MS/MS	By MS/MS
145.3	By MS/MS	By MS/MS	By MS/MS	By MS/MS	By MS/MS	By MS/MS	By MS/MS	By MS/MS
10.391	By MS/MS	By MS/MS	By MS/MS	By MS/MS	By MS/MS	By matchin	By matchin	By MS/MS
21.125	By MS/MS	By MS/MS	By MS/MS	By MS/MS	By MS/MS	By matchin	By matchin	By MS/MS
13.662	By matchin	By matchin	By MS/MS	By MS/MS	By MS/MS	By matchin	By matchin	By MS/MS
131.93	By MS/MS	By MS/MS	By MS/MS	By MS/MS	By MS/MS	By MS/MS	By MS/MS	By MS/MS
9.5599	By MS/MS	By MS/MS	By MS/MS	By MS/MS	By matchin	By MS/MS	By matchin	By matchin
21.695	By MS/MS	By MS/MS	By MS/MS	By MS/MS	By MS/MS	By MS/MS	By MS/MS	By MS/MS
5.7353	By matchin	By matchin	By matchin	By MS/MS	By MS/MS	By matchin	By matchin	By MS/MS
3.6193	By MS/MS	By MS/MS	By MS/MS	By MS/MS	By matchin	By MS/MS	By matchin	By matchin
5.7094	By MS/MS	By MS/MS	By MS/MS	By MS/MS	By MS/MS	By matching		By MS/MS
16.309	By MS/MS	By MS/MS	By MS/MS	By MS/MS	By MS/MS	By MS/MS	By MS/MS	By MS/MS
29.912	By MS/MS	By MS/MS	By MS/MS	By MS/MS	By MS/MS	By MS/MS	By MS/MS	By MS/MS
14.88	By MS/MS	By MS/MS	By MS/MS	By MS/MS	By MS/MS	By MS/MS	By matchin	By matchin
34.006	By MS/MS	By MS/MS	By MS/MS	By MS/MS	By matchin	By MS/MS	By MS/MS	By MS/MS
6.5707	By MS/MS	By MS/MS	By matchin	By MS/MS	By MS/MS	By matching		
1.7519							By matchin	By MS/MS
14.133	By MS/MS	By MS/MS	By MS/MS	By MS/MS	By MS/MS	By MS/MS	By matchin	By MS/MS
19.12	By matchin	By matchin	By matchin	By matchin	By matchin	By MS/MS	By MS/MS	By MS/MS
15.252			By matching		By matchin	By MS/MS	By MS/MS	By MS/MS
2.0315	By matchin	By MS/MS	By MS/MS	By matchin	By matchin	By matching		By matchin
318	By MS/MS	By MS/MS	By MS/MS	By MS/MS	By MS/MS	By MS/MS	By MS/MS	By MS/MS
5.4294	By MS/MS	By MS/MS		By matchin	By MS/MS		By matchin	By matchin
7.0623	By matchin	By matchin	By MS/MS	By MS/MS	By MS/MS	By MS/MS	By matchin	By MS/MS
6.7514	By MS/MS	By MS/MS	By MS/MS	By MS/MS	By MS/MS	By MS/MS	By matchin	By MS/MS
1.8248					By MS/MS			
12.736	By MS/MS	By MS/MS	By MS/MS	By MS/MS	By MS/MS	By MS/MS	By MS/MS	By MS/MS
1.6738			By MS/MS	By matching				
2.8552	By matchin	By matchin	By matchin	By MS/MS	By matchin	By matchin	By matchin	By MS/MS
5.1795		By matchin	By MS/MS	By MS/MS		By matchin	By matchin	By matchin
2.0835		By matchin	By MS/MS	By matching		By matching		
27.058	By MS/MS	By MS/MS	By MS/MS	By MS/MS	By MS/MS	By MS/MS	By MS/MS	By MS/MS
33.332	By MS/MS	By MS/MS	By MS/MS	By MS/MS	By MS/MS	By MS/MS	By MS/MS	By MS/MS
5.6909	By matchin	By matchin	By matchin	By MS/MS	By MS/MS	By matchin	By matchin	By MS/MS
75.74	By MS/MS	By MS/MS	By MS/MS	By MS/MS	By MS/MS	By MS/MS	By MS/MS	By MS/MS
7.4967	By matchin	By MS/MS	By MS/MS	By MS/MS	By MS/MS	By MS/MS	By matchin	By matchin
127.01	By MS/MS	By MS/MS	By MS/MS	By MS/MS	By MS/MS	By MS/MS	By MS/MS	By MS/MS
2.6123							By MS/MS	
232.42	By MS/MS	By MS/MS	By MS/MS	By MS/MS	By MS/MS	By MS/MS	By MS/MS	By MS/MS
2.085	By matchin	By MS/MS	By MS/MS	By MS/MS	By MS/MS	By MS/MS		
3.1982	By matchin	By matchin	By matchin	By MS/MS	By matchin	By matchin	By matchin	By matchin

13.435	By MS/MS	By MS/MS	By MS/MS	By MS/MS	By MS/MS	By MS/MS	By MS/MS	By MS/MS
5.4561	By matchin	By MS/MS	By matchin	By matchin	By MS/MS	By matchin	By matchin	By matchin
22.285	By MS/MS	By MS/MS	By matchin	By MS/MS	By MS/MS	By matchin	By matchin	By MS/MS
30.272	By MS/MS	By MS/MS	By MS/MS	By MS/MS	By MS/MS	By MS/MS	By MS/MS	By MS/MS
10.938		By MS/MS		By matching		By MS/MS	By MS/MS	By MS/MS
2.5	By matchin	By matchin	By matchin	By matchin	By matchin	By MS/MS	By MS/MS	By matchin
19.014	By MS/MS	By MS/MS	By MS/MS	By MS/MS	By MS/MS	By MS/MS	By MS/MS	By MS/MS
26.277	By MS/MS	By MS/MS	By MS/MS	By MS/MS	By MS/MS	By MS/MS	By MS/MS	By MS/MS
8.4989	By matchin	By matchin	By MS/MS	By matchin	By MS/MS	By MS/MS	By MS/MS	By MS/MS
3.1351					By matchin	By MS/MS	By MS/MS	By matchin
1.9417	By matchin	By matchin	By matchin	By matchin	By matchin	By matchin	By matchin	By MS/MS
2.6032	By matching		By matchin	By matching		By MS/MS	By matchin	By matchin
25.81	By MS/MS	By MS/MS	By matchin	By MS/MS	By MS/MS	By MS/MS	By MS/MS	By matchin
4.2099	By MS/MS	By MS/MS	By MS/MS	By MS/MS	By MS/MS	By MS/MS	By MS/MS	By MS/MS
32.9	By MS/MS	By MS/MS	By MS/MS	By MS/MS	By MS/MS	By MS/MS	By MS/MS	By MS/MS
6.376	By matchin	By MS/MS	By matchin	By matchin	By matchin	By matchin	By matchin	By matchin
2.9272	By MS/MS	By MS/MS						By MS/MS
2.1905	By MS/MS	By MS/MS	By MS/MS	By MS/MS				
34.149	By MS/MS	By MS/MS	By MS/MS	By MS/MS	By MS/MS	By MS/MS	By MS/MS	By MS/MS
1.8721			By matching		By matchin	By MS/MS		By MS/MS
7.7748	By MS/MS	By MS/MS	By matchin	By matchin	By matchin	By MS/MS	By MS/MS	By MS/MS
1.4939			By MS/MS	By MS/MS	By MS/MS			
3.5703	By MS/MS	By MS/MS	By matchin	By matchin	By matchin	By matchin	By MS/MS	By MS/MS
2.1845	By matchin	By matchin	By MS/MS	By MS/MS	By MS/MS	By MS/MS	By matchin	By MS/MS
1.6089	By matchin	By matchin	By matchin	By MS/MS		By matchin	By matchin	By matchin
9.0704	By matching			By matchin	By matchin	By matchin	By MS/MS	By MS/MS
52.093	By MS/MS	By matchin	By MS/MS	By MS/MS	By MS/MS	By MS/MS	By MS/MS	By MS/MS
3.383	By matchin	By matchin	By matchin	By matchin	By matchin	By MS/MS	By MS/MS	By MS/MS
5.3749	By MS/MS	By matchin	By matchin	By MS/MS	By MS/MS	By matchin	By matchin	By MS/MS
1.5647	By matchin	By matchin	By matchin	By matchin	By matchin	By MS/MS	By MS/MS	By MS/MS
105.06	By MS/MS	By MS/MS	By MS/MS	By MS/MS	By MS/MS	By MS/MS	By MS/MS	By MS/MS
25.748	By MS/MS	By MS/MS	By MS/MS	By MS/MS	By MS/MS	By MS/MS	By MS/MS	By MS/MS
2.5411	By MS/MS	By MS/MS	By MS/MS	By MS/MS	By matchin	By MS/MS	By matchin	By matchin
11.176			By MS/MS	By MS/MS	By MS/MS			By MS/MS
11.028		By matchin	By matchin	By matchin	By MS/MS	By matchin	By MS/MS	By MS/MS
8.7419	By MS/MS	By matchin	By MS/MS	By matchin	By MS/MS	By matchin	By MS/MS	By MS/MS
6.8624			By matchin	By matching		By MS/MS	By MS/MS	By MS/MS
23.523	By matchin	By matchin	By MS/MS	By MS/MS	By MS/MS	By MS/MS	By MS/MS	By MS/MS
7.4458	By MS/MS	By MS/MS	By matchin	By MS/MS	By MS/MS	By MS/MS	By matchin	By MS/MS
2.1623	By matching			By matchin	By matchin	By MS/MS	By matchin	By MS/MS
1.9963								By MS/MS
10.839	By MS/MS	By MS/MS	By MS/MS	By MS/MS	By MS/MS	By matchin	By matchin	By matchin
1.7068	By MS/MS			By MS/MS	By matchin	By matching		By matchin
26.032	By matchin	By matchin	By MS/MS	By MS/MS	By MS/MS	By MS/MS	By MS/MS	By MS/MS
12.341	By MS/MS	By MS/MS	By matchin	By MS/MS	By MS/MS	By matchin	By matchin	By matchin
63.861	By MS/MS	By MS/MS	By MS/MS	By MS/MS	By MS/MS	By MS/MS	By MS/MS	By MS/MS
14.83	By MS/MS	By MS/MS	By MS/MS	By MS/MS	By MS/MS	By MS/MS	By MS/MS	By MS/MS
27.481	By MS/MS	By MS/MS	By MS/MS	By MS/MS	By MS/MS	By MS/MS	By MS/MS	By MS/MS
24.339	By MS/MS	By MS/MS	By MS/MS	By MS/MS	By MS/MS	By MS/MS	By MS/MS	By MS/MS
71.303	By MS/MS	By MS/MS	By MS/MS	By MS/MS	By MS/MS	By MS/MS	By MS/MS	By MS/MS

3.0962	By matchin	By MS/MS	By matchin	By matchin	By matchin	By matchin	By matching	
2.0502		By MS/MS	By matching					
1.517	By matchin	By matchin	By matchin	By MS/MS	By matchin	By MS/MS		By matchin
61.676	By MS/MS	By MS/MS	By MS/MS	By MS/MS	By MS/MS	By MS/MS	By MS/MS	By MS/MS
1.9374	By matchin	By MS/MS	By matchin	By matching		By matching		By matchin
13.246	By matchin	By MS/MS	By matchin	By MS/MS	By MS/MS	By MS/MS	By MS/MS	By matchin
1.6005	By matchin	By MS/MS	By MS/MS	By MS/MS	By MS/MS	By matching		
6.0154	By matchin	By MS/MS	By matchin	By MS/MS	By matchin	By MS/MS	By matchin	By MS/MS
6.2319	By matchin	By matchin	By matchin	By matchin	By matchin	By MS/MS	By MS/MS	By MS/MS
17.709	By matchin	By MS/MS	By matchin	By MS/MS	By MS/MS	By MS/MS	By MS/MS	By MS/MS
2.9613		By matchin	By MS/MS	By matchin	By MS/MS	By matchin	By matchin	By matchin
16.739	By MS/MS	By MS/MS	By MS/MS	By MS/MS	By MS/MS	By MS/MS	By MS/MS	By MS/MS
7.6502	By MS/MS	By matchin	By matchin	By MS/MS	By matchin	By MS/MS		By matchin
39.576			By matchin	By matchin	By matchin	By MS/MS	By MS/MS	By MS/MS
3.0607	By matchin	By matchin	By MS/MS		By MS/MS			By matchin
3.7712	By matchin	By matchin	By matchin	By MS/MS	By MS/MS	By matchin	By MS/MS	By MS/MS
7.2698	By matchin	By matchin	By matchin	By matchin	By matchin	By matchin	By MS/MS	By MS/MS
2.1835	By matchin	By MS/MS		By MS/MS		By MS/MS	By matchin	By matchin
6.5583	By matchin	By MS/MS	By matchin	By MS/MS	By MS/MS	By MS/MS	By matchin	By matchin
12.364			By matchin	By MS/MS	By MS/MS	By MS/MS	By MS/MS	By MS/MS
1.6962			By matchin	By matchin	By MS/MS	By matchin	By matchin	By matchin
160.28	By MS/MS	By MS/MS	By MS/MS	By MS/MS	By MS/MS	By MS/MS	By MS/MS	By MS/MS
58.086	By MS/MS	By MS/MS	By MS/MS	By MS/MS	By MS/MS	By MS/MS	By MS/MS	By MS/MS
4.9777	By MS/MS	By MS/MS	By MS/MS	By MS/MS	By MS/MS	By MS/MS		By matchin
2.5947	By matchin	By matchin	By MS/MS	By MS/MS	By matchin	By matchin	By matchin	By matchin
2.0532		By matchin	By matching			By matchin	By MS/MS	By matchin
15.384	By matchin	By matchin	By matchin	By MS/MS	By matchin	By MS/MS	By MS/MS	By MS/MS
2.4755				By matchin	By matchin	By MS/MS	By MS/MS	By MS/MS
4.7547	By MS/MS	By MS/MS	By MS/MS	By MS/MS	By MS/MS	By matchin	By MS/MS	By matchin
1.8196								By MS/MS
2.8214	By matchin	By matchin	By matchin	By matching			By matchin	By MS/MS
2.3524	By matchin	By MS/MS	By matchin	By matchin	By matchin	By matchin	By matchin	By MS/MS
21.498	By MS/MS	By MS/MS	By MS/MS	By MS/MS	By MS/MS	By MS/MS	By MS/MS	By MS/MS
6.1081	By matchin	By MS/MS	By MS/MS	By MS/MS	By MS/MS	By matchin	By matchin	By matchin
113.52	By matchin	By MS/MS	By MS/MS	By MS/MS	By MS/MS	By MS/MS	By MS/MS	By MS/MS
15.993	By matchin	By matchin	By MS/MS	By MS/MS	By matchin	By MS/MS	By MS/MS	By MS/MS
58.542	By MS/MS	By matchin	By MS/MS	By MS/MS	By MS/MS	By MS/MS	By MS/MS	By MS/MS
37.059	By matchin	By matchin	By MS/MS	By MS/MS	By MS/MS	By MS/MS	By MS/MS	By MS/MS
15.929	By matchin	By matchin	By matchin	By MS/MS	By MS/MS	By MS/MS	By MS/MS	By MS/MS
1.8983							By MS/MS	By matchin
1.9482	By MS/MS	By MS/MS	By MS/MS	By matchin	By matchin	By MS/MS	By MS/MS	By MS/MS
323.31	By MS/MS	By MS/MS	By MS/MS	By MS/MS	By MS/MS	By MS/MS	By MS/MS	By MS/MS
9.1016	By matching		By matchin	By matchin	By matchin	By matchin	By MS/MS	By MS/MS
2.0279	By matchin	By matchin	By matchin	By MS/MS	By MS/MS	By matching		
9.6998	By matchin	By MS/MS	By MS/MS	By MS/MS	By MS/MS	By matchin	By matchin	By MS/MS
45.831	By MS/MS	By MS/MS	By MS/MS	By MS/MS	By MS/MS	By MS/MS	By MS/MS	By MS/MS
2.8703	By matchin	By matchin	By matchin	By matchin	By MS/MS			
38.985	By MS/MS	By MS/MS	By MS/MS	By MS/MS	By MS/MS	By MS/MS	By MS/MS	By MS/MS
21.14	By MS/MS	By MS/MS	By MS/MS	By MS/MS	By MS/MS	By MS/MS	By MS/MS	By MS/MS
21.014	By matchin	By matchin	By MS/MS	By MS/MS	By MS/MS	By MS/MS	By MS/MS	By MS/MS

50.246	By MS/MS	By MS/MS	By MS/MS	By MS/MS	By MS/MS	By MS/MS	By MS/MS	By MS/MS
14.765	By matchin	By matchin	By MS/MS	By MS/MS	By MS/MS	By MS/MS	By MS/MS	By MS/MS
4.0772	By matchin	By matchin	By matchin	By matchin	By matchin	By MS/MS	By MS/MS	By MS/MS
5.2591						By MS/MS	By MS/MS	By MS/MS
3.5466	By MS/MS	By MS/MS	By MS/MS	By MS/MS	By MS/MS	By MS/MS	By matchin	By matchin
2.3615	By matchin	By matchin	By matchin	By matchin	By matchin	By matchin	By matchin	By MS/MS
139.42	By MS/MS	By MS/MS	By MS/MS	By MS/MS	By MS/MS	By MS/MS	By MS/MS	By MS/MS
20.44	By MS/MS	By MS/MS	By matchin	By MS/MS	By MS/MS	By matchin	By matchin	By MS/MS
4.3478	By MS/MS	By MS/MS	By MS/MS	By MS/MS	By MS/MS	By MS/MS	By MS/MS	By matchin
47.356	By MS/MS	By MS/MS	By MS/MS	By MS/MS	By MS/MS	By MS/MS	By MS/MS	By MS/MS
44.568	By MS/MS	By MS/MS	By MS/MS	By matchin	By matchin	By MS/MS	By MS/MS	By MS/MS
2.8235	By MS/MS	By matchin	By matchin	By matchin	By MS/MS	By matchin	By matchin	By matchin
1.7741	By matchin	By MS/MS	By MS/MS	By matchin	By MS/MS	By MS/MS		
2.4961				By matchin	By matchin	By MS/MS	By MS/MS	By MS/MS
7.2799							By MS/MS	
2.6418						By MS/MS	By MS/MS	
158.41	By MS/MS	By MS/MS	By MS/MS	By MS/MS	By MS/MS	By MS/MS	By MS/MS	By MS/MS
84.796	By MS/MS	By MS/MS	By MS/MS	By MS/MS	By MS/MS	By MS/MS	By MS/MS	By MS/MS
2.4196	By MS/MS	By MS/MS	By MS/MS	By MS/MS	By matchin	By MS/MS	By matchin	By MS/MS
15.601	By MS/MS	By MS/MS	By MS/MS	By MS/MS	By MS/MS	By MS/MS	By MS/MS	By MS/MS
33.545	By MS/MS	By MS/MS	By MS/MS	By matchin	By MS/MS	By MS/MS	By MS/MS	By MS/MS
2.6342		By matchin	By MS/MS		By MS/MS	By matching		By matchin
9.5665	By MS/MS	By MS/MS	By matchin	By matchin	By MS/MS	By MS/MS	By MS/MS	By MS/MS
2.0157	By matching					By matchin	By matchin	By MS/MS
4.6417	By MS/MS	By MS/MS	By MS/MS	By MS/MS	By MS/MS	By MS/MS	By MS/MS	By matchin
32.555	By MS/MS	By matchin	By MS/MS	By MS/MS	By MS/MS	By MS/MS	By matchin	By matchin
11.382	By MS/MS	By MS/MS	By matching			By MS/MS	By MS/MS	By MS/MS
31.806	By MS/MS	By MS/MS	By MS/MS	By MS/MS	By MS/MS	By MS/MS	By MS/MS	By MS/MS
3.9314	By matching		By MS/MS	By MS/MS		By MS/MS	By MS/MS	By matchin
37.208	By MS/MS	By MS/MS	By MS/MS	By MS/MS	By MS/MS	By MS/MS	By MS/MS	By MS/MS
1.9317	By MS/MS	By MS/MS	By MS/MS	By MS/MS	By MS/MS	By MS/MS	By matchin	By MS/MS
50.989	By MS/MS	By MS/MS	By MS/MS	By MS/MS	By MS/MS	By MS/MS	By MS/MS	By MS/MS
11.1	By MS/MS	By MS/MS	By MS/MS	By MS/MS	By MS/MS	By MS/MS	By MS/MS	By MS/MS
2.2548	By matchin	By matchin	By matchin	By MS/MS	By MS/MS			
8.9558	By MS/MS	By MS/MS	By matchin	By matchin	By matchin	By MS/MS	By MS/MS	By MS/MS
9.2491	By MS/MS	By MS/MS	By MS/MS	By MS/MS	By MS/MS	By matchin	By MS/MS	By MS/MS
25.995	By MS/MS	By MS/MS	By MS/MS	By MS/MS	By matchin	By MS/MS	By MS/MS	By MS/MS
1.5049		By MS/MS	By MS/MS	By matchin	By matchin	By MS/MS	By matchin	By matchin
5.4191	By matchin	By matchin	By matchin	By MS/MS	By matchin	By MS/MS	By matchin	By MS/MS
5.4089	By matchin	By matchin	By matchin	By matchin	By MS/MS	By matching		
6.8878	By matchin	By matchin	By MS/MS	By matchin	By MS/MS	By matchin	By matchin	By matchin
2.0026					By MS/MS			
1.6562	By matchin	By matchin	By matchin	By MS/MS	By matchin	By MS/MS	By MS/MS	By matchin
5.5938	By matchin	By matchin	By MS/MS	By MS/MS	By MS/MS	By matching		By matchin
19.286	By matchin	By matchin	By MS/MS	By MS/MS	By MS/MS	By matchin	By MS/MS	By MS/MS
13.406	By MS/MS	By MS/MS	By MS/MS	By MS/MS	By MS/MS	By MS/MS	By MS/MS	By MS/MS
6.1004	By MS/MS	By MS/MS	By MS/MS	By MS/MS	By MS/MS	By matchin	By matchin	By MS/MS
5.856	By MS/MS	By MS/MS	By MS/MS	By MS/MS	By MS/MS	By MS/MS	By matchin	By matchin
14.645	By MS/MS	By MS/MS	By MS/MS	By MS/MS	By MS/MS	By MS/MS	By MS/MS	By matchin
65.797	By MS/MS	By MS/MS	By MS/MS	By MS/MS	By MS/MS	By MS/MS	By MS/MS	By MS/MS

31.058	By MS/MS	By MS/MS	By MS/MS	By MS/MS	By MS/MS	By MS/MS	By MS/MS	By MS/MS
3.1603								By MS/MS
2.2408		By matchin	By matching			By MS/MS	By MS/MS	By matchin
57.528	By MS/MS	By MS/MS	By MS/MS	By MS/MS	By MS/MS	By MS/MS	By MS/MS	By MS/MS
50.296	By MS/MS	By MS/MS	By MS/MS	By MS/MS	By MS/MS	By MS/MS	By MS/MS	By MS/MS
40.899	By MS/MS	By matchin	By MS/MS	By MS/MS	By MS/MS	By MS/MS	By MS/MS	By MS/MS
69.354	By matchin	By matchin	By matchin	By MS/MS	By MS/MS	By MS/MS	By MS/MS	By MS/MS
18.624	By MS/MS	By MS/MS	By MS/MS	By MS/MS	By MS/MS	By MS/MS	By MS/MS	By MS/MS
16.937	By MS/MS	By MS/MS	By matchin	By MS/MS	By MS/MS	By matching		By MS/MS
3.558	By matchin	By matchin	By matchin	By matchin	By matchin	By MS/MS	By MS/MS	By MS/MS
10.342	By MS/MS	By matchin	By matchin	By MS/MS	By matchin	By MS/MS	By MS/MS	By MS/MS
4.0954	By MS/MS	By MS/MS	By MS/MS	By MS/MS	By MS/MS	By MS/MS	By MS/MS	By MS/MS
35.025	By MS/MS	By MS/MS	By MS/MS	By MS/MS	By matchin	By MS/MS	By MS/MS	By MS/MS
23.785	By MS/MS	By MS/MS		By MS/MS	By MS/MS	By MS/MS	By MS/MS	By MS/MS
2.2406	By MS/MS	By matchin	By MS/MS	By matchin	By matchin	By matchin	By matchin	By MS/MS
10.462	By matching		By matchin	By matchin	By matchin	By MS/MS	By MS/MS	By MS/MS
19.854	By matchin	By MS/MS	By matchin	By matchin	By MS/MS	By MS/MS	By MS/MS	By MS/MS
13.16			By matchin	By matchin	By MS/MS	By MS/MS	By MS/MS	By MS/MS
2.1981	By matching		By matchin	By MS/MS	By matchin	By MS/MS	By matchin	By MS/MS
19.007						By MS/MS	By MS/MS	By MS/MS
2.0727		By matchin	By matchin	By matching		By MS/MS	By matchin	By MS/MS
9.185		By matchin	By matching			By MS/MS	By MS/MS	By MS/MS
15.042	By matchin	By matchin	By MS/MS	By matchin	By matchin	By MS/MS	By MS/MS	By MS/MS
21.181	By matchin	By matchin	By MS/MS	By MS/MS	By MS/MS	By MS/MS	By MS/MS	By MS/MS
30.165	By MS/MS	By matchin	By MS/MS	By MS/MS	By matchin	By MS/MS	By MS/MS	By MS/MS
5.0811			By matchin	By MS/MS	By matchin	By MS/MS	By MS/MS	By MS/MS
55.484	By matchin	By MS/MS	By MS/MS	By MS/MS	By MS/MS	By MS/MS	By MS/MS	By MS/MS
36.784	By matchin	By MS/MS	By matchin	By MS/MS	By matchin	By MS/MS	By MS/MS	By MS/MS
5.3674			By matchin	By matching		By MS/MS	By MS/MS	By MS/MS
2.2119			By matchin	By matching		By MS/MS	By MS/MS	By MS/MS
16.83	By matchin	By matchin	By MS/MS	By MS/MS	By MS/MS	By MS/MS	By MS/MS	By MS/MS
2.6717						By MS/MS	By MS/MS	By MS/MS
3.2312	By matchin	By matchin	By matchin	By matchin	By matchin	By MS/MS	By MS/MS	By MS/MS
25.515			By matchin	By matchin	By matchin	By MS/MS	By MS/MS	By MS/MS
10.215	By matchin	By matchin	By matchin	By MS/MS	By MS/MS	By MS/MS	By MS/MS	By MS/MS
4.7582	By MS/MS	By matchin	By matchin	By MS/MS	By MS/MS	By MS/MS	By matchin	By MS/MS
2.3412	By matchin	By matchin	By matchin	By matchin	By matchin	By MS/MS	By MS/MS	By MS/MS
17.541						By matchin	By matchin	By MS/MS
16.494	By matching		By MS/MS	By MS/MS	By MS/MS	By MS/MS	By matchin	By matchin
25.424	By MS/MS	By matchin	By matchin	By MS/MS	By MS/MS	By MS/MS	By MS/MS	By MS/MS
2.7886	By matchin	By matchin	By matchin	By matchin	By matchin	By MS/MS	By matchin	By matchin
1.76					By matchin	By matchin	By matchin	By MS/MS
4.3681	By matching		By matchin	By matchin	By matchin	By MS/MS	By MS/MS	By MS/MS
9.4732	By MS/MS	By MS/MS	By MS/MS	By MS/MS	By MS/MS	By MS/MS	By MS/MS	By MS/MS
4.7493	By matchin	By matchin	By matchin	By MS/MS	By matchin	By matchin	By matchin	By matchin
10.756			By matchin	By MS/MS	By MS/MS	By MS/MS	By MS/MS	By MS/MS
12.353	By MS/MS	By MS/MS	By MS/MS	By matching		By MS/MS	By MS/MS	By MS/MS
3.2811			By matchin	By matchin	By MS/MS	By matchin	By MS/MS	By MS/MS
21.25	By matchin	By matchin	By matchin	By MS/MS	By MS/MS	By MS/MS	By MS/MS	By MS/MS
15.258	By matchin	By MS/MS	By MS/MS	By MS/MS	By matchin	By MS/MS	By MS/MS	By MS/MS

44.801		By matchin	By matchin	By matchin	By MS/MS	By MS/MS	By MS/MS	By MS/MS
67.607	By MS/MS	By MS/MS	By MS/MS	By MS/MS	By MS/MS	By MS/MS	By MS/MS	By MS/MS
139.93	By MS/MS	By MS/MS	By MS/MS	By MS/MS	By MS/MS	By MS/MS	By MS/MS	By MS/MS
99.612	By MS/MS	By MS/MS	By MS/MS	By MS/MS	By MS/MS	By MS/MS	By MS/MS	By MS/MS
94.112	By MS/MS	By MS/MS	By MS/MS	By MS/MS	By MS/MS	By MS/MS	By MS/MS	By MS/MS
2.9027	By matching		By MS/MS	By MS/MS	By MS/MS			
7.264	By matchin	By matchin	By MS/MS	By matchin	By MS/MS	By matchin	By matchin	By matchin
26.033	By MS/MS	By MS/MS	By MS/MS	By MS/MS	By MS/MS	By MS/MS	By MS/MS	By MS/MS
4.0544	By matching		By matchin	By matchin	By MS/MS			
2.6073	By MS/MS	By MS/MS	By MS/MS	By MS/MS	By matchin	By matchin	By matchin	By matchin
19.398	By MS/MS	By MS/MS	By MS/MS	By MS/MS	By MS/MS	By MS/MS	By MS/MS	By MS/MS
1.5557	By MS/MS	By MS/MS		By MS/MS	By matching		By matchin	By matchin
9.5673	By MS/MS	By MS/MS	By MS/MS	By MS/MS	By MS/MS	By MS/MS	By MS/MS	By MS/MS
10.293		By matchin	By MS/MS		By MS/MS	By MS/MS	By MS/MS	By MS/MS
93.405	By MS/MS	By MS/MS	By MS/MS	By MS/MS	By MS/MS	By MS/MS	By MS/MS	By MS/MS
8.9506	By matchin	By MS/MS				By matchin	By matchin	By MS/MS
5.0772	By matchin	By matchin	By matchin	By matchin	By matchin	By matchin	By MS/MS	By MS/MS
2.4668	By MS/MS	By matchin	By matchin	By matchin	By matchin	By matchin	By MS/MS	By matchin
10.226			By MS/MS	By MS/MS				
2.3464	By MS/MS				By matchin	By matchin	By matching	
2.9352	By MS/MS	By matchin	By MS/MS	By MS/MS	By MS/MS	By matchin	By matchin	By MS/MS
2.9553	By matching				By matchin	By MS/MS	By MS/MS	By MS/MS
3.149	By matchin	By MS/MS	By matchin	By MS/MS	By matchin	By matchin	By MS/MS	By MS/MS
2.4188	By matchin	By matchin	By matchin	By MS/MS	By MS/MS			
9.3236	By MS/MS	By MS/MS	By MS/MS	By MS/MS	By MS/MS	By MS/MS	By matchin	By MS/MS
2.5818				By MS/MS	By MS/MS		By matchin	By MS/MS
2.1405	By matchin	By matchin	By matchin	By MS/MS	By MS/MS	By matchin	By matchin	By matchin
18.795	By MS/MS	By MS/MS	By MS/MS	By MS/MS	By MS/MS	By MS/MS	By MS/MS	By MS/MS
9.0219	By matchin	By MS/MS	By MS/MS	By matchin	By MS/MS	By MS/MS	By MS/MS	By MS/MS
1.5938	By matching				By MS/MS			By matchin
2.4213	By MS/MS	By MS/MS	By MS/MS	By MS/MS	By MS/MS	By MS/MS		By MS/MS
41.868	By MS/MS	By MS/MS	By MS/MS	By MS/MS	By MS/MS	By MS/MS	By MS/MS	By MS/MS
11.606		By matchin	By MS/MS	By matchin	By matchin	By MS/MS	By MS/MS	By MS/MS
7.0163		By MS/MS	By MS/MS	By MS/MS		By MS/MS	By matching	
2.2403	By MS/MS	By MS/MS	By MS/MS	By MS/MS	By MS/MS	By MS/MS	By matchin	By matchin
3.5257	By matching		By matchin	By MS/MS	By MS/MS	By MS/MS	By matchin	By MS/MS
6.4254							By MS/MS	By MS/MS
13.919	By matchin	By matchin	By matchin	By matchin	By matchin	By MS/MS	By MS/MS	By MS/MS
38.079	By MS/MS	By MS/MS	By MS/MS	By MS/MS	By MS/MS	By MS/MS	By MS/MS	By MS/MS
1.7012	By matching		By matching			By MS/MS	By matchin	By matchin
11.504	By matchin	By matchin	By matchin	By matchin	By matchin	By MS/MS	By MS/MS	By MS/MS
5.5058	By MS/MS	By MS/MS	By matchin	By MS/MS	By MS/MS	By MS/MS	By matchin	By matchin
8.9356	By MS/MS	By matchin	By MS/MS	By MS/MS	By MS/MS	By matchin	By matchin	By matchin
2.6634	By MS/MS	By MS/MS	By MS/MS	By MS/MS	By matchin	By MS/MS	By MS/MS	By MS/MS
12.223	By MS/MS	By MS/MS	By MS/MS	By MS/MS	By matchin	By MS/MS	By MS/MS	By MS/MS
8.8202	By MS/MS	By MS/MS	By MS/MS	By MS/MS	By MS/MS	By MS/MS	By MS/MS	By MS/MS
2.3068				By matching		By MS/MS	By matchin	By MS/MS
13.198	By MS/MS	By MS/MS	By MS/MS	By MS/MS	By MS/MS	By MS/MS	By MS/MS	By MS/MS
5.5467	By MS/MS	By MS/MS	By MS/MS	By MS/MS	By MS/MS	By MS/MS	By MS/MS	By MS/MS
8.0312	By MS/MS	By matchin	By MS/MS	By MS/MS	By matchin	By MS/MS	By MS/MS	By MS/MS

3.1127	By MS/MS	By matchin	By MS/MS	By MS/MS	By matchin	By matchin	By matchin	By matchin
35.154	By MS/MS	By MS/MS	By MS/MS	By MS/MS	By MS/MS	By MS/MS	By MS/MS	By MS/MS
50.858	By matching		By matchin	By MS/MS	By MS/MS	By MS/MS	By MS/MS	By MS/MS
10.387	By matchin	By MS/MS	By MS/MS	By MS/MS	By MS/MS	By matchin	By matchin	By MS/MS
2.0366	By MS/MS	By matchin	By matchin	By MS/MS	By MS/MS	By MS/MS	By matchin	By matchin
3.4855	By matchin	By MS/MS	By matchin	By matchin	By matchin	By MS/MS	By MS/MS	By MS/MS
9.6619	By matchin	By matchin	By MS/MS	By MS/MS	By MS/MS	By MS/MS	By MS/MS	By MS/MS
2.8041	By matchin	By matchin	By matchin	By matchin	By matchin	By MS/MS	By matchin	By MS/MS
1.5681		By matchin	By matching		By matchin	By matchin	By matchin	By MS/MS
8.0106	By MS/MS	By MS/MS	By MS/MS	By MS/MS	By MS/MS	By MS/MS	By MS/MS	By MS/MS
24.013	By MS/MS	By MS/MS	By MS/MS	By MS/MS	By MS/MS	By MS/MS	By MS/MS	By MS/MS
3.5481	By MS/MS	By MS/MS	By MS/MS	By MS/MS	By matchin	By MS/MS		
1.953	By matchin	By matching					By matchin	By MS/MS
2.8004	By MS/MS	By matchin	By MS/MS	By matchin	By MS/MS	By matchin	By matchin	By matchin
3.2925		By MS/MS			By matchin	By MS/MS	By matchin	By MS/MS
20.777	By MS/MS	By MS/MS	By MS/MS	By MS/MS	By MS/MS	By MS/MS	By MS/MS	By MS/MS
5.2736	By MS/MS	By MS/MS	By MS/MS	By matchin	By matchin	By matchin	By MS/MS	By matchin
88.037			By matchin	By matchin	By MS/MS	By MS/MS	By MS/MS	By MS/MS
16.659	By MS/MS	By MS/MS	By MS/MS	By MS/MS	By MS/MS	By matchin	By matchin	By matchin
6.737	By MS/MS	By MS/MS	By MS/MS	By MS/MS	By MS/MS	By matchin	By MS/MS	By matchin
29.592			By matchin	By MS/MS	By MS/MS	By MS/MS	By MS/MS	By MS/MS
40.213	By matchin	By matchin	By MS/MS	By MS/MS	By MS/MS	By MS/MS	By MS/MS	By MS/MS
12.495	By matchin	By matchin	By matchin	By MS/MS	By MS/MS	By MS/MS	By MS/MS	By MS/MS
5.9237	By MS/MS	By MS/MS	By matching			By matchin	By matchin	By MS/MS
-2						By matchin	By MS/MS	By matchin
1.6754	By MS/MS	By matchin	By matchin	By MS/MS	By matchin	By matching		By matchin
314.52	By MS/MS	By MS/MS	By MS/MS	By MS/MS	By MS/MS	By MS/MS	By MS/MS	By MS/MS
2.2409	By matchin	By matchin	By matchin	By matchin	By matchin	By matchin	By MS/MS	By MS/MS
2.5822	By matchin	By matchin	By matchin	By matchin	By matchin	By matchin	By MS/MS	By MS/MS
3.3457	By matchin	By MS/MS	By matchin	By matchin	By MS/MS	By matchin	By matchin	By MS/MS
3.1745	By matchin	By matchin	By matchin	By matchin	By MS/MS	By MS/MS	By MS/MS	By matchin
3.3836			By MS/MS	By MS/MS	By MS/MS	By matching		
2.2089	By matching				By MS/MS			By MS/MS
4.6519	By matchin	By matchin	By matchin	By matchin	By MS/MS	By MS/MS	By MS/MS	By MS/MS
1.5621	By matchin	By MS/MS	By matchin	By matchin	By MS/MS			
-2	By matchin	By matching			By MS/MS	By matching		
6.8187	By MS/MS	By MS/MS	By matchin	By MS/MS	By matchin	By matchin	By matchin	By MS/MS
16.542	By MS/MS	By MS/MS	By MS/MS	By MS/MS	By MS/MS	By MS/MS	By MS/MS	By matchin
5.5247	By MS/MS	By MS/MS	By MS/MS	By MS/MS	By MS/MS	By MS/MS	By MS/MS	By MS/MS
20.408	By MS/MS	By MS/MS	By MS/MS	By MS/MS	By MS/MS	By MS/MS	By matchin	By matchin
1.8773			By matchin	By MS/MS	By MS/MS		By matchin	By matchin
323.31	By MS/MS	By MS/MS	By MS/MS	By MS/MS	By MS/MS	By MS/MS	By MS/MS	By MS/MS
1.5312	By matchin	By matchin	By matchin	By matchin	By matchin	By matchin	By MS/MS	By matchin
26.856	By matchin	By matchin	By MS/MS	By MS/MS	By MS/MS	By MS/MS	By MS/MS	By MS/MS
5.5721	By MS/MS	By matchin	By MS/MS	By MS/MS	By MS/MS	By matchin	By matchin	By matchin
41.973	By MS/MS	By MS/MS	By MS/MS	By MS/MS	By MS/MS	By MS/MS	By matchin	By MS/MS
2.0292	By matchin	By matchin	By matchin	By matchin	By MS/MS	By matchin	By matchin	By matchin
33.797	By matchin	By MS/MS	By MS/MS	By MS/MS	By MS/MS	By MS/MS	By MS/MS	By MS/MS
5.9182	By matchin	By matchin	By matchin	By MS/MS	By MS/MS	By MS/MS	By MS/MS	By MS/MS
29.828		By matchin	By MS/MS	By MS/MS	By MS/MS	By MS/MS	By MS/MS	By MS/MS

169.93	By MS/MS	By MS/MS	By MS/MS	By MS/MS	By MS/MS	By MS/MS	By MS/MS	By MS/MS
1.8877			By matchin	By matching		By matchin	By matchin	By MS/MS
25.587	By MS/MS	By MS/MS	By MS/MS	By MS/MS	By MS/MS	By MS/MS	By MS/MS	By MS/MS
2.7084	By MS/MS	By MS/MS	By matchin	By matchin	By MS/MS	By MS/MS	By MS/MS	By MS/MS
2.3431	By matchin	By matching			By MS/MS	By matching		By MS/MS
21.469	By MS/MS	By MS/MS	By matchin	By MS/MS	By MS/MS	By MS/MS	By MS/MS	By MS/MS
24.628	By MS/MS	By MS/MS	By MS/MS	By MS/MS	By MS/MS	By MS/MS	By MS/MS	By MS/MS
76.226	By MS/MS	By MS/MS	By MS/MS	By MS/MS	By MS/MS	By MS/MS	By MS/MS	By MS/MS
23.467	By MS/MS	By MS/MS	By MS/MS	By matchin	By MS/MS	By MS/MS	By MS/MS	By MS/MS
53.304	By MS/MS	By MS/MS	By MS/MS	By MS/MS	By MS/MS	By MS/MS	By MS/MS	By MS/MS
6.4061	By MS/MS	By matchin	By MS/MS	By MS/MS	By MS/MS	By matchin	By matchin	By matchin
51.474	By MS/MS	By MS/MS	By MS/MS	By MS/MS	By MS/MS	By MS/MS	By MS/MS	By MS/MS
1.5779	By matchin	By MS/MS	By matchin	By MS/MS	By MS/MS	By matchin	By matchin	By matchin
2.0907	By matchin	By MS/MS	By MS/MS	By matchin	By matchin	By matchin	By matchin	By MS/MS
33.368	By MS/MS	By MS/MS	By MS/MS	By MS/MS	By MS/MS	By MS/MS	By MS/MS	By MS/MS
11.826	By MS/MS	By MS/MS	By matchin	By MS/MS	By MS/MS	By MS/MS	By matchin	By MS/MS
4.7659	By MS/MS	By MS/MS	By matchin	By matching		By matchin	By matchin	By MS/MS
1.5034	By matching		By matchin	By matchin	By MS/MS	By matching		By matchin
25.621	By MS/MS	By MS/MS	By MS/MS	By MS/MS	By MS/MS	By MS/MS	By MS/MS	By MS/MS
14.003	By MS/MS	By MS/MS	By MS/MS	By MS/MS	By MS/MS	By MS/MS	By MS/MS	By MS/MS
104.85	By MS/MS	By MS/MS	By MS/MS	By MS/MS	By MS/MS	By MS/MS	By MS/MS	By MS/MS
1.8454	By matchin	By matchin	By matchin	By MS/MS	By MS/MS	By matchin	By matchin	By matchin
3.5248	By matchin	By matchin	By MS/MS	By matchin	By matchin	By matchin	By matchin	By matchin
5.4945	By MS/MS	By matchin	By MS/MS	By MS/MS	By MS/MS	By matchin	By matching	
9.1516	By MS/MS	By MS/MS	By MS/MS	By MS/MS	By MS/MS	By MS/MS	By MS/MS	By MS/MS
17.102	By MS/MS	By MS/MS	By MS/MS	By MS/MS	By MS/MS	By MS/MS	By MS/MS	By MS/MS
8.3449	By MS/MS	By MS/MS	By MS/MS	By MS/MS	By MS/MS	By MS/MS	By matchin	By matchin
3.3535	By matchin	By MS/MS	By MS/MS	By MS/MS	By matchin	By matchin	By MS/MS	By MS/MS
32.983	By MS/MS	By MS/MS	By MS/MS	By MS/MS	By MS/MS	By MS/MS	By MS/MS	By MS/MS
2.2882	By MS/MS	By MS/MS	By MS/MS	By MS/MS	By MS/MS	By matchin	By MS/MS	By MS/MS
7.5319	By MS/MS	By MS/MS	By MS/MS	By MS/MS	By matchin	By MS/MS	By matchin	By MS/MS
2.9342	By matchin	By MS/MS	By MS/MS	By MS/MS	By MS/MS	By matchin	By matchin	By matchin
10.387	By MS/MS	By MS/MS	By MS/MS	By MS/MS	By MS/MS	By matchin	By matchin	By MS/MS
8.437	By MS/MS	By MS/MS	By MS/MS	By MS/MS	By MS/MS	By MS/MS	By matchin	By matchin
22.622	By matchin	By MS/MS	By MS/MS	By MS/MS	By MS/MS	By MS/MS	By MS/MS	By MS/MS
5.4313	By MS/MS	By MS/MS	By matchin	By matchin	By MS/MS	By MS/MS	By MS/MS	By MS/MS
2.2882	By matchin	By matchin	By matchin	By MS/MS	By MS/MS	By matching		By matchin
3.1343	By matchin	By matchin	By matchin	By matchin	By matchin	By matchin	By matchin	By MS/MS
4.9994	By matchin	By MS/MS	By matchin	By matchin	By matchin	By matchin	By MS/MS	By MS/MS
18.235	By MS/MS	By MS/MS	By MS/MS	By MS/MS	By MS/MS	By MS/MS	By MS/MS	By MS/MS
14.518	By MS/MS	By MS/MS	By matchin	By matchin	By matchin	By MS/MS	By MS/MS	By MS/MS
3.3506	By MS/MS	By MS/MS		By matchin	By matchin	By matchin	By matchin	By matchin
2.166	By matchin	By matchin	By matchin	By matching		By MS/MS	By matchin	By MS/MS
6.4095		By matchin	By matchin	By matchin	By matchin	By MS/MS	By MS/MS	By MS/MS
3.6323				By matchin	By matchin	By matchin	By MS/MS	By MS/MS
4.2162	By matching		By matchin	By matching		By MS/MS	By MS/MS	By MS/MS
26.716	By MS/MS	By MS/MS	By MS/MS	By MS/MS	By MS/MS	By MS/MS	By MS/MS	By MS/MS
21.291	By MS/MS	By MS/MS	By MS/MS	By MS/MS	By MS/MS	By matchin	By matchin	By MS/MS
38.744	By MS/MS	By MS/MS	By MS/MS	By MS/MS	By MS/MS	By MS/MS	By MS/MS	By MS/MS
1.9811	By MS/MS	By MS/MS				By MS/MS	By MS/MS	By MS/MS

7.1326	By matchin	By matchin	By MS/MS	By matchin	By matchin	By MS/MS	By MS/MS	By matchin
6.8456	By MS/MS	By matchin	By matchin	By MS/MS	By MS/MS	By MS/MS	By matchin	By MS/MS
13.495	By MS/MS	By MS/MS	By MS/MS	By MS/MS	By MS/MS	By MS/MS	By MS/MS	By MS/MS
2.2266	By MS/MS	By MS/MS	By MS/MS	By matchin	By matchin	By matchin	By matchin	By matchin
24.973	By matchin	By matchin	By MS/MS	By MS/MS	By MS/MS	By MS/MS	By MS/MS	By MS/MS
4.4499	By matchin	By matchin	By matchin	By matchin	By matchin	By MS/MS	By matchin	By MS/MS
13.99	By matchin	By matchin	By MS/MS	By matchin	By matchin	By MS/MS	By MS/MS	By MS/MS
1.8045								By MS/MS
30.453	By MS/MS	By MS/MS	By MS/MS	By MS/MS	By MS/MS	By MS/MS	By MS/MS	By MS/MS
10.129	By MS/MS	By MS/MS	By MS/MS	By matchin	By MS/MS	By MS/MS	By matchin	By MS/MS
52.228	By MS/MS	By MS/MS	By MS/MS	By MS/MS	By MS/MS	By MS/MS	By MS/MS	By MS/MS
4.0501			By matching			By matchin	By MS/MS	By MS/MS
5.6683	By matchin	By matchin	By matchin	By matchin	By MS/MS	By matchin	By MS/MS	By MS/MS
4.7906	By matchin	By matchin	By matchin	By matchin	By MS/MS	By MS/MS	By MS/MS	By MS/MS
7.9373	By MS/MS	By MS/MS	By MS/MS	By MS/MS	By MS/MS	By MS/MS	By MS/MS	By MS/MS
3.6491						By matchin	By matchin	By MS/MS
52.418	By MS/MS	By MS/MS	By MS/MS	By MS/MS	By MS/MS	By MS/MS	By MS/MS	By MS/MS
9.1999	By MS/MS	By MS/MS	By MS/MS	By MS/MS	By MS/MS	By MS/MS	By MS/MS	By MS/MS
14.636	By MS/MS	By MS/MS	By MS/MS	By matchin	By MS/MS	By matchin	By matchin	By matchin
3.9246	By matchin	By matchin	By MS/MS	By MS/MS	By MS/MS	By MS/MS	By matchin	By MS/MS
17.547		By matchin	By MS/MS	By matchin	By MS/MS	By matching		By MS/MS
6.0924	By MS/MS	By MS/MS	By matchin	By MS/MS	By MS/MS	By matchin	By matchin	By matchin
24.336	By MS/MS	By MS/MS	By MS/MS	By MS/MS	By MS/MS	By MS/MS	By matchin	By MS/MS
9.324	By matchin	By MS/MS	By MS/MS	By matchin	By MS/MS	By matchin	By MS/MS	By MS/MS
6.0201	By MS/MS	By MS/MS	By MS/MS	By MS/MS	By MS/MS	By matchin	By MS/MS	By matchin
7.4001	By matchin	By matchin	By matchin	By matchin	By MS/MS	By MS/MS	By matchin	By MS/MS
10.472	By MS/MS	By MS/MS	By MS/MS	By MS/MS	By MS/MS	By MS/MS	By MS/MS	By MS/MS
1.9127			By matchin	By matchin	By matchin	By matchin	By MS/MS	By MS/MS
10.568	By matchin	By MS/MS	By MS/MS	By matchin	By MS/MS	By MS/MS	By matching	
38.007	By matchin	By matchin	By MS/MS	By MS/MS	By MS/MS	By MS/MS	By MS/MS	By MS/MS
26.451	By MS/MS	By MS/MS	By MS/MS	By MS/MS	By MS/MS	By MS/MS	By matchin	By MS/MS
1.7637	By matchin	By MS/MS						
25.775	By matchin	By MS/MS	By MS/MS	By MS/MS	By MS/MS	By MS/MS	By MS/MS	By MS/MS
43.124	By MS/MS	By MS/MS	By MS/MS	By MS/MS	By MS/MS	By MS/MS	By MS/MS	By MS/MS
16.436	By MS/MS	By MS/MS	By MS/MS	By MS/MS	By matchin	By MS/MS	By MS/MS	By MS/MS
8.888	By MS/MS	By MS/MS	By MS/MS	By matchin	By MS/MS	By matchin	By MS/MS	By MS/MS
-2				By MS/MS				
11.92	By MS/MS	By MS/MS	By MS/MS	By MS/MS	By MS/MS	By MS/MS	By matchin	By matchin
9.1267		By matchin	By MS/MS	By MS/MS	By MS/MS	By matchin	By matchin	By matchin
39.081	By MS/MS	By MS/MS	By MS/MS	By MS/MS	By MS/MS	By MS/MS	By MS/MS	By MS/MS
19.143	By MS/MS	By MS/MS	By MS/MS	By matchin	By MS/MS	By MS/MS	By MS/MS	By MS/MS
38.579	By matchin	By matchin	By MS/MS	By matchin	By MS/MS	By MS/MS	By MS/MS	By MS/MS
22.068	By matchin	By matchin	By MS/MS	By MS/MS	By MS/MS	By MS/MS	By MS/MS	By MS/MS
1.6395	By matchin	By MS/MS	By matchin	By matchin	By matchin	By matchin	By matchin	By matchin
3.9652	By matchin	By matchin	By MS/MS	By MS/MS	By MS/MS	By MS/MS	By matchin	By matchin
11.167	By MS/MS	By MS/MS	By MS/MS	By MS/MS	By matchin	By MS/MS	By MS/MS	By MS/MS
4.3204	By matchin	By MS/MS	By MS/MS	By MS/MS	By matchin	By matchin	By MS/MS	By matchin
2.1757	By matchin	By matchin	By matchin	By MS/MS	By MS/MS	By MS/MS	By MS/MS	By MS/MS
26.206	By MS/MS	By MS/MS	By MS/MS	By MS/MS	By MS/MS	By MS/MS	By MS/MS	By MS/MS
1.9662	By matchin	By matchin	By matchin	By MS/MS		By matchin	By matchin	By matchin

2.356	By MS/MS	By MS/MS	By MS/MS	By MS/MS	By MS/MS	By MS/MS		
2.048	By matchin	By matchin	By MS/MS	By matchin	By MS/MS	By matching		By matchin
5.8806		By MS/MS	By MS/MS	By MS/MS	By MS/MS	By matchin	By matchin	By matchin
4.6173	By matchin	By MS/MS	By matchin	By MS/MS	By MS/MS	By matchin	By MS/MS	By MS/MS
55.573	By MS/MS	By MS/MS	By MS/MS	By MS/MS	By MS/MS	By MS/MS	By MS/MS	By MS/MS
27.215	By MS/MS	By MS/MS	By MS/MS	By MS/MS	By MS/MS	By MS/MS	By MS/MS	By MS/MS
25.258	By MS/MS	By MS/MS	By MS/MS	By MS/MS	By MS/MS	By MS/MS	By MS/MS	By MS/MS
4.3416						By MS/MS	By MS/MS	By MS/MS
88.594	By MS/MS	By MS/MS	By MS/MS	By MS/MS	By MS/MS	By MS/MS	By MS/MS	By MS/MS
20.951	By MS/MS	By MS/MS	By MS/MS	By MS/MS	By MS/MS	By MS/MS	By MS/MS	By MS/MS
4.3024	By matchin	By matchin	By matchin	By matchin	By MS/MS	By matchin	By MS/MS	By MS/MS
2.6002	By matchin	By MS/MS	By matchin	By MS/MS	By MS/MS	By MS/MS	By matchin	By MS/MS
2.4188	By matchin	By MS/MS	By matching			By matching		By matchin
16.565			By MS/MS	By MS/MS	By MS/MS	By MS/MS	By matchin	By matchin
4.072		By matchin	By matchin	By matchin	By matchin	By MS/MS	By MS/MS	By MS/MS
2.798	By matchin	By matchin	By matchin	By MS/MS	By MS/MS	By matchin	By matching	
8.7131	By MS/MS	By MS/MS	By MS/MS	By MS/MS	By MS/MS	By matchin	By matchin	By matchin
21	By MS/MS	By matchin	By matchin	By MS/MS	By MS/MS	By MS/MS	By matchin	By matchin
1.536								By MS/MS
2.236						By matchin	By matchin	By MS/MS
1.4874			By matchin	By matchin	By matchin	By MS/MS		
323.31	By MS/MS	By MS/MS	By MS/MS	By MS/MS	By MS/MS	By MS/MS	By MS/MS	By MS/MS
17.126	By MS/MS	By MS/MS	By MS/MS	By matchin	By matchin	By matchin	By matchin	By MS/MS
63.971	By MS/MS	By MS/MS	By MS/MS	By MS/MS	By MS/MS	By MS/MS	By MS/MS	By MS/MS
30.736	By MS/MS	By MS/MS	By MS/MS	By MS/MS	By MS/MS	By MS/MS	By MS/MS	By matchin
44.352	By MS/MS	By MS/MS	By MS/MS	By MS/MS	By MS/MS	By MS/MS	By MS/MS	By MS/MS
3.6673	By MS/MS	By MS/MS	By MS/MS	By MS/MS	By matchin	By matchin	By matchin	By matchin
1.55					By MS/MS			
22.448	By MS/MS	By MS/MS	By MS/MS	By MS/MS	By MS/MS	By MS/MS	By MS/MS	By MS/MS
16.586	By MS/MS	By MS/MS	By MS/MS	By MS/MS	By MS/MS	By MS/MS	By MS/MS	By MS/MS
1.8347	By MS/MS	By matchin	By MS/MS	By matchin	By matching		By matching	
1.6719	By matchin	By matchin	By matchin	By matchin	By matchin	By MS/MS	By matchin	By matchin
22.101	By MS/MS	By MS/MS	By MS/MS	By MS/MS	By MS/MS	By MS/MS	By MS/MS	By MS/MS
6.0701	By matchin	By MS/MS	By MS/MS	By MS/MS	By matchin	By MS/MS	By MS/MS	By MS/MS
8.8826	By MS/MS	By MS/MS	By MS/MS	By MS/MS	By matchin	By MS/MS	By MS/MS	By MS/MS
9.6978	By MS/MS	By MS/MS	By MS/MS	By MS/MS	By MS/MS	By MS/MS	By MS/MS	By MS/MS
12.537	By MS/MS	By MS/MS	By MS/MS	By MS/MS	By MS/MS	By MS/MS	By MS/MS	By MS/MS
11.906	By MS/MS	By MS/MS	By MS/MS	By MS/MS	By MS/MS	By MS/MS	By MS/MS	By MS/MS
4.5892	By matchin	By matchin	By matchin	By matchin	By matchin	By MS/MS	By MS/MS	By MS/MS
3.0689	By matchin	By MS/MS	By MS/MS	By matchin	By matchin	By matchin	By matchin	By matchin
2.3128	By matching					By matchin	By matchin	By MS/MS
8.1804		By matchin	By matchin	By matchin	By MS/MS	By MS/MS	By MS/MS	By MS/MS
1.6148	By matching		By MS/MS	By matchin	By matchin	By matchin	By matchin	By matchin
3.3322	By matchin	By matchin	By matchin	By MS/MS	By MS/MS	By matchin	By MS/MS	By MS/MS
27.142	By MS/MS	By MS/MS	By MS/MS	By MS/MS	By MS/MS	By MS/MS	By MS/MS	By MS/MS
4.255						By matchin	By MS/MS	By MS/MS
39.711	By MS/MS	By MS/MS	By MS/MS	By MS/MS	By MS/MS	By MS/MS	By MS/MS	By MS/MS
14.998	By MS/MS	By MS/MS	By MS/MS	By matchin	By matchin	By matchin	By matchin	By MS/MS
3.4332	By matchin	By matchin	By matchin	By MS/MS	By matchin	By MS/MS	By MS/MS	By MS/MS
43.644	By MS/MS	By MS/MS	By MS/MS	By MS/MS	By MS/MS	By MS/MS	By MS/MS	By MS/MS

3.2772	By matchin	By matchin	By matchin	By matching		By MS/MS	By MS/MS	By MS/MS
2.6837	By matchin	By matchin	By matchin	By MS/MS	By matchin	By matchin	By MS/MS	By matchin
6.4366	By matchin	By matchin	By MS/MS	By matchin	By matchin	By matchin	By MS/MS	By MS/MS
19.281	By matchin	By matchin	By matchin	By MS/MS	By MS/MS	By MS/MS	By MS/MS	By MS/MS
8.4555	By matchin	By MS/MS	By matchin	By matchin	By matchin	By MS/MS	By MS/MS	By MS/MS
3.8743	By MS/MS	By MS/MS	By matchin	By matchin	By MS/MS	By matchin	By matchin	By MS/MS
12.227	By matchin	By matchin	By matchin	By matching		By MS/MS	By MS/MS	By MS/MS
60.583	By MS/MS	By MS/MS	By MS/MS	By MS/MS	By MS/MS	By MS/MS	By MS/MS	By MS/MS
11.639		By matchin	By matchin	By matchin	By matchin	By MS/MS	By MS/MS	By MS/MS
118.89	By MS/MS	By MS/MS	By MS/MS	By MS/MS	By MS/MS	By MS/MS	By MS/MS	By MS/MS
7.8528	By matchin	By MS/MS	By matchin	By matchin	By matchin	By MS/MS	By matchin	By matchin
4.6053	By MS/MS	By MS/MS	By MS/MS	By matchin	By matchin	By MS/MS		By matchin
1.8273			By matching				By MS/MS	By matchin
3.3838		By matchin	By matchin	By matchin	By matchin	By MS/MS	By MS/MS	By MS/MS
3.1306			By matchin	By matchin	By matchin	By MS/MS	By MS/MS	By MS/MS
16.104	By MS/MS	By MS/MS	By matchin	By MS/MS	By MS/MS	By matchin	By matchin	By matchin
28.836	By MS/MS	By MS/MS	By MS/MS	By MS/MS	By MS/MS	By MS/MS	By MS/MS	By MS/MS
2.1973	By MS/MS	By MS/MS	By matchin	By matching		By MS/MS	By MS/MS	By MS/MS
7.6727	By MS/MS	By MS/MS	By MS/MS	By matchin	By MS/MS	By MS/MS	By matchin	By matchin
87.24	By MS/MS	By MS/MS	By MS/MS	By MS/MS	By MS/MS	By MS/MS	By matchin	By MS/MS
5.7097	By MS/MS	By MS/MS	By MS/MS	By MS/MS	By MS/MS	By MS/MS	By MS/MS	By MS/MS
12.381	By matchin	By MS/MS	By matchin	By matchin	By matchin	By matchin	By MS/MS	By MS/MS
8.9947				By matchin	By matchin	By MS/MS	By MS/MS	By MS/MS
23.775	By matchin	By matchin	By MS/MS	By MS/MS	By MS/MS	By MS/MS	By MS/MS	By MS/MS
18.027	By MS/MS	By MS/MS	By MS/MS	By MS/MS	By MS/MS	By MS/MS	By MS/MS	By MS/MS
4.5131	By matchin	By matchin	By matchin	By matchin	By matchin	By MS/MS	By MS/MS	By MS/MS
6.2822	By matchin	By MS/MS	By matchin	By matchin	By matchin	By MS/MS	By MS/MS	By MS/MS
11.458	By matchin	By matchin	By matchin	By MS/MS	By MS/MS	By MS/MS	By MS/MS	By MS/MS
1.8612		By matchin	By matching			By matchin	By matchin	By MS/MS
2.1516		By matchin	By MS/MS	By MS/MS	By matchin	By matchin	By matching	
13.211	By MS/MS	By MS/MS	By MS/MS	By MS/MS	By MS/MS	By MS/MS	By MS/MS	By MS/MS
3.6933	By matchin	By matchin	By matchin	By matchin	By matchin	By MS/MS	By MS/MS	By matchin
2.977		By matchin	By matchin	By matchin	By matchin	By MS/MS	By MS/MS	By MS/MS
8.0101	By MS/MS	By MS/MS	By MS/MS	By MS/MS	By MS/MS	By MS/MS	By MS/MS	By MS/MS
5.0095	By matchin	By matching			By matchin	By matchin	By MS/MS	By MS/MS
13.958		By MS/MS	By MS/MS	By MS/MS	By matchin	By MS/MS	By MS/MS	By MS/MS
16.351	By MS/MS	By MS/MS	By MS/MS	By MS/MS	By MS/MS	By MS/MS	By MS/MS	By MS/MS
8.9562	By MS/MS	By matchin	By MS/MS	By MS/MS		By MS/MS	By MS/MS	By MS/MS
3.8782	By matchin	By MS/MS	By MS/MS	By matchin	By matchin	By matching		By matchin
5.1732	By matchin	By matchin	By MS/MS	By MS/MS	By MS/MS	By matchin	By MS/MS	By MS/MS
13.094			By MS/MS	By MS/MS	By MS/MS	By MS/MS	By matchin	By MS/MS
1.8689							By MS/MS	
156.83	By MS/MS	By MS/MS	By MS/MS	By MS/MS	By MS/MS	By MS/MS	By MS/MS	By MS/MS
7.16	By MS/MS	By MS/MS	By MS/MS	By MS/MS	By MS/MS	By matchin	By matchin	By matchin
8.4109	By MS/MS	By MS/MS	By MS/MS	By MS/MS	By MS/MS	By MS/MS	By MS/MS	By MS/MS
1.8246	By matchin	By MS/MS	By matchin	By MS/MS		By MS/MS	By matchin	By matchin
63.938	By MS/MS	By MS/MS	By MS/MS	By MS/MS	By MS/MS	By MS/MS	By MS/MS	By MS/MS
10.027	By MS/MS	By MS/MS	By MS/MS	By MS/MS	By MS/MS	By MS/MS	By MS/MS	By MS/MS
2.1854	By matchin	By MS/MS	By MS/MS	By MS/MS	By matchin	By matching		By MS/MS
2.4469		By matchin	By matchin	By matchin	By MS/MS	By matchin	By matchin	By matchin

7.256	By MS/MS	By MS/MS	By MS/MS	By MS/MS	By MS/MS	By matchin	By MS/MS	By MS/MS
2.0823	By matchin	By matchin	By matchin	By matchin	By MS/MS	By matchin	By matchin	By matchin
31.513	By matchin	By matchin	By MS/MS	By MS/MS	By MS/MS	By MS/MS	By MS/MS	By MS/MS
14.368	By MS/MS	By MS/MS	By MS/MS	By MS/MS	By MS/MS	By MS/MS	By MS/MS	By MS/MS
2.2175	By MS/MS	By MS/MS	By matchin	By matching			By matching	
3.7428			By matchin	By matchin	By MS/MS	By MS/MS		By MS/MS
9.3566	By MS/MS	By matchin	By matchin	By MS/MS		By matchin	By matchin	By MS/MS
15.418	By matchin	By MS/MS	By MS/MS	By MS/MS	By MS/MS	By MS/MS	By MS/MS	By MS/MS
5.4869		By matchin	By MS/MS	By matchin	By matchin	By matchin	By MS/MS	By MS/MS
82.751	By matchin	By MS/MS	By MS/MS	By MS/MS	By MS/MS	By MS/MS	By MS/MS	By MS/MS
53.903	By matchin	By matchin	By MS/MS	By MS/MS	By MS/MS	By MS/MS	By MS/MS	By MS/MS
12.155	By MS/MS	By MS/MS	By MS/MS	By MS/MS	By MS/MS	By MS/MS	By MS/MS	By MS/MS
2.8682		By matchin	By MS/MS	By MS/MS				
2.482	By MS/MS	By matchin	By MS/MS	By matchin	By matchin	By matchin	By matchin	By matchin
64.505	By MS/MS	By MS/MS	By matchin	By matching		By MS/MS	By MS/MS	By MS/MS
17.247	By matchin	By matchin	By matchin	By MS/MS	By matchin	By MS/MS	By MS/MS	By MS/MS
6.5064			By matchin	By matching		By MS/MS	By MS/MS	By MS/MS
9.3351			By matchin	By MS/MS	By MS/MS	By MS/MS	By MS/MS	By MS/MS
14.647	By matchin	By MS/MS	By MS/MS	By MS/MS	By MS/MS	By MS/MS	By MS/MS	By MS/MS
15.708	By matchin	By matchin	By matchin	By matchin	By matchin	By MS/MS	By MS/MS	By MS/MS
28.195	By MS/MS	By MS/MS	By MS/MS	By MS/MS	By MS/MS	By MS/MS	By MS/MS	By MS/MS
3.6995	By MS/MS	By MS/MS	By MS/MS	By matching		By MS/MS	By MS/MS	By MS/MS
13.05	By MS/MS	By MS/MS	By matchin	By matchin	By MS/MS	By MS/MS	By MS/MS	By MS/MS
17.944	By MS/MS	By MS/MS	By matchin	By matchin	By MS/MS	By MS/MS	By MS/MS	By MS/MS
61.25	By MS/MS	By matchin	By MS/MS	By MS/MS	By MS/MS	By MS/MS	By MS/MS	By MS/MS
5.1861	By MS/MS	By MS/MS	By MS/MS	By MS/MS	By MS/MS	By MS/MS	By matchin	By matchin
64.757	By MS/MS	By MS/MS	By MS/MS	By MS/MS	By MS/MS	By MS/MS	By MS/MS	By MS/MS
1.4902								By MS/MS
11.377	By MS/MS	By MS/MS	By MS/MS	By MS/MS		By MS/MS	By MS/MS	By matchin
70.108	By MS/MS	By MS/MS	By MS/MS	By MS/MS	By MS/MS	By MS/MS	By MS/MS	By MS/MS
100.13	By MS/MS	By MS/MS	By MS/MS	By MS/MS	By MS/MS	By MS/MS	By MS/MS	By MS/MS
8.0635				By matchin	By matchin	By MS/MS	By MS/MS	By MS/MS
1.6541						By MS/MS	By MS/MS	By MS/MS
3.8311	By MS/MS	By MS/MS	By matching			By MS/MS	By MS/MS	By MS/MS
2.4729	By matchin	By matchin	By MS/MS	By MS/MS	By matchin	By matchin	By MS/MS	
29.623	By MS/MS	By MS/MS	By MS/MS	By MS/MS	By MS/MS	By MS/MS	By MS/MS	By MS/MS
23.493	By MS/MS	By MS/MS	By MS/MS	By MS/MS	By MS/MS	By MS/MS	By matchin	By MS/MS
2.3399	By MS/MS	By MS/MS	By MS/MS	By MS/MS	By MS/MS	By MS/MS	By matchin	By MS/MS
253.05	By MS/MS	By MS/MS	By MS/MS	By MS/MS	By MS/MS	By MS/MS	By MS/MS	By MS/MS
58.759	By matchin	By MS/MS	By MS/MS	By MS/MS	By MS/MS	By MS/MS	By MS/MS	By MS/MS
4.5302	By MS/MS	By MS/MS	By MS/MS	By matchin	By matchin	By matchin	By matchin	By matchin
1.7948	By matchin	By matchin	By matchin	By MS/MS	By MS/MS	By MS/MS	By matching	
8.307	By MS/MS	By MS/MS	By matchin	By matchin	By matchin	By MS/MS	By MS/MS	By MS/MS
11.723	By matching		By matchin	By matchin	By MS/MS	By MS/MS	By MS/MS	By MS/MS
5.3383	By matching		By matchin	By matchin	By MS/MS	By matchin	By MS/MS	By MS/MS
20.609	By MS/MS	By MS/MS	By MS/MS	By matchin	By matchin	By MS/MS	By MS/MS	By MS/MS
32.463	By MS/MS	By MS/MS	By MS/MS	By MS/MS	By MS/MS	By MS/MS	By matchin	By matchin
8.2275	By MS/MS	By MS/MS	By MS/MS	By matchin	By MS/MS	By MS/MS	By MS/MS	By matchin
12.202	By MS/MS	By MS/MS	By MS/MS	By MS/MS	By MS/MS	By MS/MS	By MS/MS	By MS/MS
8.6869	By MS/MS	By MS/MS	By MS/MS	By MS/MS	By MS/MS	By MS/MS	By matchin	By matchin

17.048	By MS/MS	By MS/MS	By MS/MS	By MS/MS	By MS/MS	By MS/MS	By MS/MS	By MS/MS
10.671	By MS/MS	By MS/MS	By MS/MS	By matchin	By MS/MS	By MS/MS	By MS/MS	By MS/MS
60.779	By MS/MS	By MS/MS	By MS/MS	By MS/MS	By MS/MS	By MS/MS	By MS/MS	By MS/MS
5.3027							By matchin	By MS/MS
4.108	By matchin	By matchin	By MS/MS	By MS/MS	By matchin	By MS/MS	By MS/MS	By MS/MS
2.5266	By matchin	By MS/MS	By matchin	By MS/MS	By MS/MS	By matching		
1.8338	By matchin	By matchin	By matchin	By matchin	By MS/MS	By matchin	By MS/MS	By matchin
5.4482	By matchin	By matchin	By MS/MS	By MS/MS	By matchin	By MS/MS	By MS/MS	By MS/MS
20.229	By MS/MS	By MS/MS	By MS/MS	By MS/MS	By MS/MS	By MS/MS	By MS/MS	By MS/MS
4.4091	By matchin	By matchin	By matchin	By matchin	By MS/MS	By MS/MS	By MS/MS	By MS/MS
1.8308							By MS/MS	
15.426	By matchin	By matchin	By MS/MS	By MS/MS	By MS/MS	By MS/MS	By matchin	By matchin
2.1535	By MS/MS	By matchin	By MS/MS	By MS/MS	By MS/MS	By matching		
24.151	By MS/MS	By matchin	By matchin	By MS/MS	By MS/MS	By MS/MS	By matchin	By matchin
40.064	By matchin	By matchin	By MS/MS	By MS/MS	By MS/MS	By MS/MS	By matchin	By matchin
3.0125		By matching		By matching		By matching		By MS/MS
9.3374	By matchin	By matchin	By matchin	By MS/MS	By MS/MS	By matchin	By matching	
22.114	By MS/MS	By MS/MS	By MS/MS	By MS/MS	By MS/MS	By MS/MS	By MS/MS	By MS/MS
2.1102	By matching		By matchin	By matchin	By MS/MS	By MS/MS	By MS/MS	By matchin
8.7437	By matching		By MS/MS	By matchin	By MS/MS		By matchin	By MS/MS
2.9094					By matchin	By matchin	By MS/MS	By MS/MS
29.001	By MS/MS	By MS/MS	By MS/MS	By MS/MS	By MS/MS	By MS/MS	By MS/MS	By MS/MS
2.4238		By matchin	By matchin	By MS/MS	By matchin	By matchin	By matchin	By MS/MS
2.6184	By MS/MS	By MS/MS	By MS/MS	By MS/MS	By matchin	By MS/MS	By matching	
2.2201	By matchin	By MS/MS	By matchin	By MS/MS	By matchin	By MS/MS	By MS/MS	By MS/MS
4.2455							By matchin	By MS/MS
2.9714	By matchin	By MS/MS	By MS/MS	By MS/MS	By MS/MS	By MS/MS		By matchin
11.767	By MS/MS	By MS/MS	By MS/MS	By matchin	By matchin	By MS/MS	By matchin	By matchin
5.6083	By matchin	By MS/MS	By MS/MS	By matchin	By MS/MS	By matchin	By matchin	By matchin
3.1806		By MS/MS	By MS/MS	By MS/MS		By MS/MS		By MS/MS
4.1316	By MS/MS	By MS/MS	By MS/MS	By MS/MS	By MS/MS	By MS/MS	By matchin	By MS/MS
78	By MS/MS	By MS/MS	By MS/MS	By MS/MS	By MS/MS	By MS/MS	By MS/MS	By MS/MS
10.464	By matchin	By MS/MS	By MS/MS	By matchin	By matchin	By MS/MS	By MS/MS	By MS/MS
16.148	By matchin	By MS/MS	By MS/MS	By MS/MS	By matchin	By MS/MS	By MS/MS	By MS/MS
3.3347	By MS/MS	By MS/MS	By matchin	By MS/MS	By matchin	By matchin	By matchin	By matchin
18.988	By MS/MS	By MS/MS	By MS/MS	By MS/MS	By MS/MS	By MS/MS	By MS/MS	By MS/MS
27.676	By matchin	By matchin	By MS/MS	By MS/MS	By MS/MS	By MS/MS	By MS/MS	By MS/MS
17.649	By MS/MS	By MS/MS	By MS/MS	By MS/MS	By MS/MS	By MS/MS	By MS/MS	By MS/MS
4.4122	By matchin	By matchin	By MS/MS	By MS/MS	By MS/MS	By MS/MS	By MS/MS	By MS/MS
4.3502			By matchin	By matchin	By MS/MS	By matchin	By matchin	By MS/MS
2.0938	By matching						By matchin	By MS/MS
40.63	By MS/MS	By matchin	By MS/MS	By MS/MS	By MS/MS	By MS/MS	By MS/MS	By MS/MS
69.054	By MS/MS	By MS/MS	By matchin	By MS/MS	By MS/MS	By MS/MS	By MS/MS	By MS/MS
55.83	By MS/MS	By MS/MS	By MS/MS	By MS/MS	By MS/MS	By MS/MS	By MS/MS	By MS/MS
62.337	By MS/MS	By matchin	By matchin	By MS/MS	By MS/MS	By MS/MS	By MS/MS	By MS/MS
35.12	By MS/MS	By MS/MS	By MS/MS	By MS/MS	By MS/MS	By MS/MS	By MS/MS	By MS/MS
45.62	By MS/MS	By MS/MS	By MS/MS	By MS/MS	By MS/MS	By MS/MS	By MS/MS	By MS/MS
48.063	By MS/MS	By MS/MS	By MS/MS	By MS/MS	By MS/MS	By MS/MS	By MS/MS	By MS/MS
42.205	By MS/MS	By matchin	By MS/MS	By MS/MS	By matchin	By MS/MS	By MS/MS	By MS/MS
5.4234	By matching		By MS/MS	By MS/MS	By MS/MS	By matchin	By matchin	By matchin

3.1055	By matchin	By MS/MS	By matchin	By matchin	By matchin	By matchin	By matchin	By MS/MS
2.0981		By matchin	By matchin	By matchin	By matchin	By MS/MS	By matchin	By MS/MS
4.0829	By matchin	By MS/MS	By MS/MS	By MS/MS	By MS/MS	By MS/MS	By MS/MS	By matchin
8.1302	By MS/MS	By MS/MS	By MS/MS	By MS/MS	By MS/MS	By MS/MS	By MS/MS	By MS/MS
50.828	By MS/MS	By MS/MS	By MS/MS	By MS/MS	By MS/MS	By MS/MS	By MS/MS	By MS/MS
12.823				By MS/MS	By MS/MS			By MS/MS
3.9872	By MS/MS	By MS/MS	By MS/MS	By matching		By matchin	By matchin	By matchin
5.0232	By MS/MS	By MS/MS	By MS/MS	By matchin	By matchin	By MS/MS	By MS/MS	By MS/MS
3.4353	By matching						By MS/MS	By MS/MS
14.238	By matchin	By matchin	By MS/MS	By MS/MS	By MS/MS	By MS/MS	By MS/MS	By MS/MS
2.4297	By MS/MS	By MS/MS	By MS/MS	By MS/MS	By MS/MS	By MS/MS	By matchin	By matchin
5.0892	By MS/MS	By matchin	By MS/MS	By MS/MS	By MS/MS	By MS/MS	By MS/MS	By MS/MS
37.609	By MS/MS	By MS/MS	By MS/MS	By MS/MS	By MS/MS	By MS/MS	By MS/MS	By MS/MS
23.513	By matchin	By matchin	By MS/MS	By matching		By MS/MS	By MS/MS	By MS/MS
18.913	By MS/MS	By MS/MS	By MS/MS	By MS/MS	By MS/MS	By MS/MS	By MS/MS	By MS/MS
13.991	By MS/MS	By matchin	By MS/MS	By MS/MS	By matchin	By MS/MS	By MS/MS	By MS/MS
14.506	By MS/MS	By MS/MS	By MS/MS	By MS/MS	By MS/MS	By MS/MS	By MS/MS	By MS/MS
13.256	By MS/MS	By MS/MS	By MS/MS	By MS/MS	By MS/MS	By MS/MS	By matchin	By MS/MS
5.9871	By MS/MS	By MS/MS	By MS/MS	By MS/MS		By MS/MS	By matchin	By MS/MS
1.8826	By matchin	By matchin	By matchin	By matching		By matching		By MS/MS
43.509			By MS/MS	By MS/MS	By MS/MS	By MS/MS	By MS/MS	By MS/MS
-2							By MS/MS	
41.159	By matchin	By matchin	By MS/MS	By MS/MS	By MS/MS	By MS/MS	By MS/MS	By MS/MS
35.095	By MS/MS	By MS/MS	By MS/MS	By MS/MS	By MS/MS	By MS/MS	By MS/MS	By MS/MS
2.2959							By MS/MS	By MS/MS
2.3658	By matchin	By MS/MS						
4.072			By matchin	By matchin	By matchin	By matchin	By MS/MS	By MS/MS
28.042	By matchin	By MS/MS	By MS/MS	By MS/MS	By MS/MS	By MS/MS	By MS/MS	By MS/MS
50.8	By MS/MS	By MS/MS	By MS/MS	By MS/MS	By MS/MS	By MS/MS	By MS/MS	By MS/MS
24.085	By matchin	By MS/MS	By MS/MS	By MS/MS	By MS/MS	By MS/MS	By MS/MS	By MS/MS
1.8492				By matchin	By matchin	By matchin	By MS/MS	
20.328	By MS/MS	By MS/MS	By MS/MS	By MS/MS	By MS/MS	By MS/MS	By MS/MS	By MS/MS
28.362	By MS/MS	By MS/MS	By MS/MS	By MS/MS	By MS/MS	By MS/MS	By MS/MS	By MS/MS
-2	By matchin	By MS/MS	By matching			By matchin	By matchin	By matchin
11.621	By MS/MS	By MS/MS	By MS/MS	By MS/MS	By MS/MS	By MS/MS	By matchin	By MS/MS
2.6187	By matching		By matchin	By matchin	By MS/MS	By matching		
33.885	By MS/MS	By MS/MS	By MS/MS	By MS/MS	By MS/MS	By MS/MS	By MS/MS	By MS/MS
3.0896			By matchin	By MS/MS	By matchin	By matching		
19.712	By matchin	By matchin	By MS/MS	By MS/MS	By MS/MS	By MS/MS	By MS/MS	By MS/MS
2.588	By matchin	By matchin	By MS/MS	By MS/MS	By MS/MS	By matching		By matchin
8.4867			By matchin	By matchin	By matchin	By MS/MS	By MS/MS	By MS/MS
19.975	By MS/MS	By matchin	By matchin	By MS/MS	By matchin	By MS/MS	By MS/MS	By MS/MS
10.092	By MS/MS	By MS/MS	By MS/MS	By MS/MS	By MS/MS	By MS/MS	By MS/MS	By MS/MS
2.1004						By matchin	By MS/MS	By MS/MS
11.396	By MS/MS	By MS/MS	By MS/MS	By MS/MS	By MS/MS	By MS/MS	By matchin	By MS/MS
1.551						By matchin	By matchin	By MS/MS
28.647	By matchin	By matchin	By matchin	By matchin	By matchin	By MS/MS	By MS/MS	By MS/MS
13.432	By MS/MS	By matchin	By MS/MS	By MS/MS	By MS/MS	By MS/MS		By MS/MS
1.938	By matchin	By matchin	By matchin	By matchin	By matchin	By MS/MS	By matchin	By MS/MS
3.7584	By matchin	By MS/MS	By matchin	By MS/MS	By matchin	By matchin	By MS/MS	By MS/MS

26.472	By MS/MS	By MS/MS	By MS/MS	By MS/MS	By MS/MS	By MS/MS	By matchin	By MS/MS
115.67	By MS/MS	By MS/MS	By MS/MS	By MS/MS	By MS/MS	By MS/MS	By MS/MS	By MS/MS
5.3093	By matchin	By MS/MS	By MS/MS	By MS/MS	By matchin	By MS/MS	By MS/MS	By matchin
9.2726	By matchin	By MS/MS	By MS/MS	By MS/MS	By MS/MS	By MS/MS	By MS/MS	By MS/MS
2.3949	By matchin	By MS/MS	By matchin	By MS/MS	By matchin	By matchin	By matchin	By matchin
2.9329	By MS/MS	By MS/MS	By MS/MS	By MS/MS	By MS/MS	By MS/MS	By MS/MS	By MS/MS
71.618	By MS/MS	By MS/MS	By MS/MS	By MS/MS	By MS/MS	By MS/MS	By MS/MS	By MS/MS
7.2372	By MS/MS	By MS/MS	By MS/MS	By MS/MS	By MS/MS	By matchin	By MS/MS	
62.654	By MS/MS	By MS/MS	By MS/MS	By MS/MS	By MS/MS	By MS/MS	By MS/MS	By MS/MS
65.79	By MS/MS	By MS/MS	By MS/MS	By MS/MS	By MS/MS	By MS/MS	By MS/MS	By MS/MS
12.591	By MS/MS	By MS/MS	By matchin	By matchin	By matchin	By MS/MS	By MS/MS	By MS/MS
7.4158	By MS/MS	By matchin	By matchin	By matchin	By MS/MS	By matchin	By MS/MS	By MS/MS
43.237	By MS/MS	By MS/MS	By matchin	By matchin	By MS/MS	By MS/MS	By MS/MS	By MS/MS
4.6252	By matchin	By MS/MS	By MS/MS	By MS/MS		By matchin	By MS/MS	By matchin
2.3604	By MS/MS	By matchin	By MS/MS	By MS/MS	By MS/MS	By MS/MS	By MS/MS	By matchin
11.005	By MS/MS	By MS/MS	By MS/MS	By MS/MS	By matchin	By matchin	By MS/MS	By MS/MS
2.2679	By matchin	By MS/MS	By MS/MS	By MS/MS	By MS/MS	By MS/MS	By MS/MS	By MS/MS
6.4667	By MS/MS	By MS/MS	By matchin	By matching			By MS/MS	By matchin
8.7327	By MS/MS	By MS/MS	By matchin	By matchin	By MS/MS	By MS/MS	By MS/MS	By matchin
16.429	By MS/MS	By MS/MS	By MS/MS	By MS/MS	By MS/MS	By MS/MS	By MS/MS	By MS/MS
5.0885	By MS/MS	By matchin	By MS/MS	By MS/MS	By matchin	By MS/MS	By matchin	By MS/MS
37.444	By MS/MS	By MS/MS	By MS/MS	By MS/MS	By MS/MS	By MS/MS	By MS/MS	By MS/MS
6.8224	By MS/MS	By MS/MS	By MS/MS	By MS/MS	By matchin	By MS/MS	By MS/MS	By MS/MS
9.1997	By MS/MS	By MS/MS	By MS/MS	By MS/MS	By MS/MS	By matchin	By MS/MS	By matchin
1.9307	By MS/MS	By matchin	By matchin	By matchin	By matchin	By MS/MS	By matchin	By matchin
3.2773		By MS/MS	By MS/MS	By MS/MS	By MS/MS	By matchin	By matchin	By MS/MS
8.1884	By MS/MS	By MS/MS	By MS/MS	By MS/MS	By MS/MS	By MS/MS	By MS/MS	By MS/MS
5.2597	By MS/MS	By MS/MS	By MS/MS	By MS/MS	By MS/MS	By MS/MS	By MS/MS	By MS/MS
16.7	By MS/MS	By MS/MS	By MS/MS	By matchin	By matchin	By MS/MS	By MS/MS	By MS/MS
4.4578	By MS/MS	By MS/MS	By MS/MS	By MS/MS	By matchin	By matchin	By matchin	By MS/MS
8.7799	By MS/MS	By MS/MS	By MS/MS	By MS/MS	By MS/MS	By matchin	By matchin	By MS/MS
4.3701	By MS/MS	By MS/MS	By MS/MS	By MS/MS	By MS/MS	By MS/MS	By matchin	By MS/MS
15.443	By MS/MS	By MS/MS	By MS/MS	By MS/MS	By MS/MS	By MS/MS	By MS/MS	By MS/MS
9.0845	By MS/MS	By MS/MS	By MS/MS	By MS/MS	By matchin	By MS/MS	By matchin	By MS/MS
2.4031			By MS/MS	By MS/MS	By MS/MS			By matchin
-2	By matchin	By matchin	By matchin	By matchin	By matching			By MS/MS
4.5068			By matchin	By MS/MS				
4.735	By MS/MS	By MS/MS	By MS/MS	By MS/MS	By matchin	By matchin	By matchin	By matchin
2.985	By MS/MS	By MS/MS	By matching					
6.2497	By MS/MS	By MS/MS	By MS/MS	By MS/MS		By MS/MS	By MS/MS	By MS/MS
7.9097	By MS/MS	By MS/MS	By MS/MS	By MS/MS	By MS/MS	By matchin	By matchin	By matchin
31.799	By MS/MS	By MS/MS	By MS/MS	By MS/MS	By MS/MS	By MS/MS	By MS/MS	By MS/MS
25.443	By MS/MS	By MS/MS	By MS/MS	By MS/MS	By MS/MS	By matchin	By matchin	By matchin
2.1672	By matchin	By MS/MS	By matchin	By matching		By MS/MS	By MS/MS	
8.3098	By matchin	By MS/MS	By matchin	By matchin	By matchin	By MS/MS	By MS/MS	By MS/MS
40.112	By matchin	By MS/MS	By MS/MS	By MS/MS	By MS/MS	By MS/MS	By MS/MS	By MS/MS
4.7482	By MS/MS	By MS/MS	By MS/MS	By MS/MS	By MS/MS	By MS/MS	By MS/MS	By MS/MS
88.165	By MS/MS	By MS/MS	By MS/MS	By MS/MS	By MS/MS	By MS/MS	By MS/MS	By MS/MS
39.426	By MS/MS	By MS/MS	By MS/MS	By MS/MS	By MS/MS	By MS/MS	By MS/MS	By MS/MS
7.755	By matchin	By matchin	By matchin	By matchin	By matchin	By MS/MS	By MS/MS	By MS/MS

220.03	By MS/MS	By MS/MS	By MS/MS	By MS/MS	By MS/MS	By MS/MS	By MS/MS	By MS/MS
3.9051	By matchin	By MS/MS	By MS/MS	By MS/MS	By matchin	By MS/MS	By matchin	By matchin
4.4883	By MS/MS	By MS/MS	By MS/MS	By matchin	By matchin	By matchin	By matchin	By matchin
59.531	By MS/MS	By MS/MS	By MS/MS	By matchin	By matchin	By MS/MS	By MS/MS	By MS/MS
11.698	By MS/MS	By MS/MS	By matchin	By matchin	By MS/MS	By MS/MS	By MS/MS	By MS/MS
12.243	By MS/MS	By MS/MS	By MS/MS	By matchin	By MS/MS	By MS/MS	By MS/MS	By MS/MS
21.371	By MS/MS	By MS/MS		By MS/MS	By MS/MS	By MS/MS	By MS/MS	By MS/MS
6.9663	By MS/MS	By MS/MS	By matchin	By MS/MS	By MS/MS	By MS/MS	By MS/MS	By MS/MS
58.537	By MS/MS	By MS/MS	By MS/MS	By MS/MS	By MS/MS	By MS/MS	By MS/MS	By MS/MS
7.1405	By MS/MS	By MS/MS	By MS/MS	By MS/MS	By MS/MS	By MS/MS	By MS/MS	By MS/MS
49.903	By MS/MS	By MS/MS	By MS/MS	By MS/MS	By MS/MS	By MS/MS	By MS/MS	By MS/MS
9.4301	By matching		By matchin	By matchin	By matchin	By MS/MS	By MS/MS	By MS/MS
4.8512	By MS/MS	By MS/MS	By MS/MS	By matchin	By MS/MS	By MS/MS	By MS/MS	By MS/MS
153.67	By MS/MS	By MS/MS	By MS/MS	By MS/MS	By MS/MS	By MS/MS	By MS/MS	By MS/MS
5.5759	By MS/MS	By MS/MS	By MS/MS	By matchin	By matchin	By MS/MS	By matchin	By matchin
34.266	By MS/MS	By MS/MS	By MS/MS	By MS/MS	By MS/MS	By MS/MS	By MS/MS	By MS/MS
65.965	By MS/MS	By MS/MS	By MS/MS	By MS/MS	By MS/MS	By MS/MS	By MS/MS	By MS/MS
9.2457	By MS/MS	By MS/MS	By matchin	By MS/MS	By matchin	By matchin	By matchin	By MS/MS
3.8054	By matchin	By MS/MS	By matchin	By matchin	By matchin	By matchin	By MS/MS	By MS/MS
185.92	By MS/MS	By MS/MS	By MS/MS	By MS/MS	By MS/MS	By MS/MS	By MS/MS	By MS/MS
4.3658	By MS/MS	By MS/MS	By MS/MS	By matchin	By MS/MS	By matchin	By matchin	By matchin
323.31	By MS/MS	By MS/MS	By MS/MS	By MS/MS	By MS/MS	By MS/MS	By MS/MS	By MS/MS
3.168	By MS/MS	By MS/MS	By matching			By matchin	By MS/MS	By MS/MS
4.4076			By matchin	By matchin	By matchin	By MS/MS	By MS/MS	By MS/MS
5.6134	By MS/MS	By matchin	By matchin	By matchin	By matchin	By MS/MS	By MS/MS	By matchin
1.4948							By matchin	By MS/MS
2.8367	By MS/MS	By matchin	By MS/MS	By MS/MS	By MS/MS	By MS/MS	By matching	
5.4651	By MS/MS	By MS/MS	By matchin	By matching		By matchin	By MS/MS	By MS/MS
54.994			By matchin	By matching		By MS/MS	By MS/MS	By MS/MS
35.018		By matchin	By MS/MS	By MS/MS	By MS/MS	By MS/MS	By MS/MS	By MS/MS
12.547	By MS/MS	By MS/MS	By MS/MS	By MS/MS	By MS/MS	By matchin	By MS/MS	By MS/MS
2.7564	By matching		By matchin	By matchin	By MS/MS	By matchin	By matchin	By matchin
70.473	By MS/MS	By MS/MS	By MS/MS	By MS/MS	By MS/MS	By MS/MS	By MS/MS	By MS/MS
4.8595	By matching		By matchin	By MS/MS	By matchin	By MS/MS	By MS/MS	By MS/MS
4.5269			By matchin	By matchin	By matchin	By MS/MS	By matchin	By matchin
3.0657	By MS/MS	By matchin	By MS/MS	By MS/MS	By MS/MS	By matchin	By matchin	By matchin
22.123	By matchin	By MS/MS	By MS/MS	By MS/MS	By MS/MS	By MS/MS	By matchin	By MS/MS
10.432	By MS/MS	By MS/MS	By MS/MS	By matchin	By MS/MS	By matchin	By matchin	By MS/MS
16.597	By MS/MS	By MS/MS	By MS/MS	By MS/MS	By MS/MS	By MS/MS	By MS/MS	By MS/MS
3.8492	By matchin	By matchin	By matchin	By matchin	By matchin	By matchin	By MS/MS	By MS/MS
18.342	By MS/MS	By MS/MS	By matchin	By MS/MS	By matchin	By matchin	By MS/MS	By MS/MS
18.291	By matchin	By MS/MS	By matchin	By MS/MS	By matchin	By MS/MS	By MS/MS	By MS/MS
1.7541		By matching					By MS/MS	By matchin
5.5739			By matchin	By matchin	By matchin	By MS/MS	By MS/MS	By MS/MS
14.259		By matchin	By matchin	By matching		By MS/MS	By MS/MS	By MS/MS
1.7019		By matching			By MS/MS	By matchin	By matchin	By MS/MS
4.478	By matchin	By matchin	By matchin	By matchin	By matchin	By MS/MS	By MS/MS	By MS/MS
2.7166	By matchin	By MS/MS	By MS/MS	By MS/MS	By MS/MS	By matchin	By matching	
7.2591		By matchin	By matchin	By matchin	By MS/MS			
7.613		By matchin	By MS/MS	By MS/MS	By MS/MS	By matchin	By matchin	By MS/MS

4.0345	By matchin	By MS/MS	By matchin	By MS/MS	By MS/MS	By matchin	By matching	
3.421	By MS/MS	By MS/MS	By MS/MS	By matchin	By MS/MS	By matchin	By matchin	By MS/MS
5.2698	By matchin	By MS/MS	By MS/MS	By MS/MS	By MS/MS	By MS/MS	By matchin	By matchin
2.6371	By matchin	By matchin	By matchin	By matching		By MS/MS	By MS/MS	By MS/MS
3.6726			By matchin	By matchin	By matchin	By MS/MS		By MS/MS
62.632	By MS/MS	By MS/MS	By matchin	By matchin	By MS/MS	By MS/MS	By MS/MS	By MS/MS
34.584	By MS/MS	By MS/MS	By MS/MS	By matchin	By MS/MS	By MS/MS	By MS/MS	By MS/MS
22.973				By matchin	By matchin	By MS/MS	By matchin	By MS/MS
1.875							By matchin	By MS/MS
35.583	By matchin	By MS/MS	By MS/MS	By MS/MS	By MS/MS	By MS/MS	By MS/MS	By MS/MS
3.5185	By MS/MS		By MS/MS	By MS/MS		By MS/MS		By MS/MS
1.7908	By matchin	By matching				By matching		By MS/MS
1.6313			By matching			By matchin	By matchin	By MS/MS
14.597	By MS/MS	By matchin	By MS/MS	By MS/MS	By MS/MS	By MS/MS	By MS/MS	By MS/MS
27.528		By matchin	By matchin	By matching		By MS/MS	By MS/MS	By MS/MS
2.3122	By matching		By matchin	By matching		By MS/MS	By MS/MS	By matchin
3.6298			By MS/MS	By matchin	By matchin	By MS/MS	By MS/MS	By MS/MS
8.975	By matchin	By matchin	By MS/MS	By MS/MS	By MS/MS	By MS/MS	By MS/MS	By MS/MS
72.259	By MS/MS	By MS/MS	By MS/MS	By MS/MS	By MS/MS	By MS/MS	By MS/MS	By MS/MS
4.708	By matching		By matchin	By matchin	By matchin	By MS/MS	By MS/MS	By matchin
3.1985	By matchin	By matchin	By matching				By matchin	By MS/MS
4.6956			By matchin	By MS/MS		By MS/MS	By MS/MS	By MS/MS
4.1022		By matchin	By matchin	By matching		By MS/MS	By matchin	By MS/MS
11.959	By MS/MS	By matchin	By matchin	By MS/MS	By MS/MS	By MS/MS	By MS/MS	By MS/MS
5.2286	By matchin	By matchin	By MS/MS	By matchin	By matchin	By matchin	By MS/MS	By MS/MS
1.6452	By matchin	By matchin	By matchin	By matchin	By matchin	By MS/MS	By MS/MS	By matchin
3.1593	By matchin	By matchin	By matchin	By MS/MS		By MS/MS	By MS/MS	By MS/MS
175.01	By matchin	By MS/MS	By MS/MS	By MS/MS	By MS/MS	By MS/MS	By MS/MS	By MS/MS
2.2161		By matchin	By matching		By matchin	By matchin	By MS/MS	By MS/MS
2.0482	By matchin	By MS/MS	By matchin	By matching		By MS/MS		By MS/MS
63.16	By MS/MS	By MS/MS	By MS/MS	By MS/MS	By MS/MS	By MS/MS	By MS/MS	By MS/MS
18.555	By MS/MS	By MS/MS	By MS/MS	By MS/MS	By MS/MS	By MS/MS	By MS/MS	By MS/MS
27.815	By MS/MS	By MS/MS	By MS/MS	By MS/MS	By MS/MS	By MS/MS	By matchin	By MS/MS
5.5056	By MS/MS	By MS/MS	By MS/MS	By matchin	By MS/MS	By MS/MS	By MS/MS	By matchin
14.365		By matching		By matchin	By matchin	By MS/MS	By MS/MS	By MS/MS
1.7022		By matchin	By matchin	By matching		By matchin	By MS/MS	By MS/MS
1.5345	By matchin	By matchin	By MS/MS	By matchin	By matchin	By matchin	By matchin	By matchin
19.823	By MS/MS	By MS/MS	By MS/MS	By MS/MS	By MS/MS	By MS/MS	By MS/MS	By MS/MS
35.958	By MS/MS	By MS/MS	By MS/MS	By MS/MS	By MS/MS	By MS/MS	By MS/MS	By MS/MS
38.468	By MS/MS	By MS/MS	By MS/MS	By MS/MS	By MS/MS	By MS/MS	By MS/MS	By MS/MS
4.37	By MS/MS	By MS/MS				By matchin	By MS/MS	By matchin
4.3618	By MS/MS	By MS/MS	By MS/MS	By MS/MS	By MS/MS	By MS/MS	By MS/MS	By matchin
2.028		By MS/MS		By matching			By matching	
10.053		By matching		By matching			By matchin	By MS/MS
11.466	By MS/MS	By MS/MS	By MS/MS	By MS/MS	By MS/MS	By matchin	By matchin	By matchin
3.17	By MS/MS	By MS/MS	By MS/MS	By MS/MS	By matchin	By MS/MS	By matchin	By MS/MS
16.154	By matchin	By matching				By matchin	By matchin	By MS/MS
5.2181	By matchin	By matching		By matching		By MS/MS	By MS/MS	By MS/MS
2.7072	By MS/MS	By matchin	By matchin	By MS/MS	By matchin	By MS/MS	By MS/MS	By matchin
2.0823						By matchin	By matchin	By MS/MS

1.5436	By MS/MS	By MS/MS	By matchin	By matchin	By MS/MS	By MS/MS	By MS/MS	By MS/MS
5.649				By matchin	By MS/MS	By MS/MS	By MS/MS	By matchin
32.504	By matchin	By matchin	By MS/MS	By MS/MS	By matchin	By MS/MS	By MS/MS	By MS/MS
3.2645	By MS/MS	By MS/MS	By MS/MS	By MS/MS	By MS/MS	By MS/MS		
3.1064	By matchin	By matchin	By matchin	By MS/MS	By MS/MS	By MS/MS	By MS/MS	By matchin
4.4119		By matchin	By MS/MS	By MS/MS	By MS/MS	By matchin	By matchin	By matchin
24.156	By MS/MS	By MS/MS	By MS/MS	By MS/MS	By matchin	By MS/MS	By MS/MS	By MS/MS
3.6028	By matchin	By matchin	By matchin	By MS/MS	By MS/MS	By MS/MS	By MS/MS	
2.1569		By matchin	By matching			By matchin	By MS/MS	By MS/MS
10.126	By MS/MS	By matchin	By matchin	By matchin	By matchin	By MS/MS	By MS/MS	By MS/MS
3.9858	By MS/MS	By matchin	By matchin	By MS/MS	By MS/MS	By matching		By matchin
103.92	By MS/MS	By MS/MS	By MS/MS	By MS/MS	By MS/MS	By MS/MS	By MS/MS	By MS/MS
2.3007		By matching			By MS/MS			
49.116	By MS/MS	By MS/MS	By MS/MS	By MS/MS	By MS/MS	By MS/MS	By matchin	By MS/MS
6.992	By MS/MS	By MS/MS	By MS/MS	By MS/MS	By matchin	By MS/MS	By MS/MS	By MS/MS
9.6708	By MS/MS	By MS/MS	By MS/MS	By MS/MS		By MS/MS	By MS/MS	By MS/MS
2.1854	By matchin	By matchin	By MS/MS	By MS/MS	By MS/MS	By MS/MS	By matching	
52.441	By MS/MS	By MS/MS	By MS/MS	By MS/MS	By MS/MS	By MS/MS	By matchin	By MS/MS
4.9984	By matchin	By MS/MS	By MS/MS	By MS/MS	By MS/MS	By matchin	By matchin	By MS/MS
6.4702	By matchin	By MS/MS	By MS/MS	By MS/MS	By MS/MS	By MS/MS	By matchin	By MS/MS
7.9105	By MS/MS	By MS/MS	By MS/MS	By MS/MS	By MS/MS	By MS/MS	By MS/MS	By MS/MS
1.6032	By matchin	By matchin	By MS/MS		By MS/MS		By matching	
2.5381	By matchin	By matchin	By MS/MS	By MS/MS	By matchin	By matching		By matchin
14.314	By MS/MS	By MS/MS	By MS/MS	By MS/MS	By MS/MS	By MS/MS	By matchin	By MS/MS
8.6743	By MS/MS	By MS/MS	By MS/MS	By MS/MS	By MS/MS	By MS/MS	By matchin	By MS/MS
34.819	By matchin	By MS/MS	By matchin	By matchin	By MS/MS	By MS/MS	By MS/MS	By MS/MS
59.552	By matchin	By matchin	By matchin	By MS/MS	By MS/MS	By MS/MS	By MS/MS	By MS/MS
4.2397	By matchin	By MS/MS	By matchin	By MS/MS	By matchin	By matching		
1.651	By MS/MS	By MS/MS	By MS/MS	By MS/MS	By MS/MS	By matchin	By matchin	By matchin
253.01	By MS/MS	By MS/MS	By MS/MS	By MS/MS	By MS/MS	By MS/MS	By MS/MS	By MS/MS
158.99	By MS/MS	By MS/MS	By MS/MS	By MS/MS	By MS/MS	By MS/MS	By MS/MS	By MS/MS
15.038	By MS/MS	By MS/MS	By MS/MS	By MS/MS	By MS/MS	By MS/MS	By MS/MS	By MS/MS
2.2172	By matchin	By matchin	By MS/MS	By matchin	By MS/MS	By matchin	By matchin	By matchin
1.7062	By MS/MS	By matchin	By matchin	By matching		By MS/MS		By MS/MS
10.811	By MS/MS	By MS/MS	By MS/MS	By MS/MS	By MS/MS	By MS/MS	By MS/MS	By MS/MS
2.0557	By matchin	By matchin	By MS/MS	By MS/MS	By MS/MS	By matching		By matchin
19.885	By matchin	By MS/MS	By MS/MS	By MS/MS	By MS/MS	By MS/MS	By MS/MS	By MS/MS
3.7777		By matchin	By MS/MS	By MS/MS		By matchin	By matching	
6.0029	By matchin	By MS/MS	By MS/MS	By MS/MS	By MS/MS	By MS/MS	By MS/MS	By MS/MS
13.758	By matchin	By MS/MS	By MS/MS	By MS/MS	By matchin	By MS/MS	By MS/MS	By MS/MS
5.3514	By MS/MS	By MS/MS	By MS/MS	By MS/MS	By MS/MS	By MS/MS	By MS/MS	By MS/MS
19.191	By MS/MS	By matchin	By MS/MS	By MS/MS	By MS/MS	By MS/MS	By MS/MS	By MS/MS
22.752	By MS/MS	By matchin	By matchin	By matchin	By MS/MS	By MS/MS	By MS/MS	By MS/MS
8.9291	By MS/MS	By MS/MS	By MS/MS	By MS/MS	By MS/MS	By MS/MS	By MS/MS	By MS/MS
4.5202	By matchin	By MS/MS	By MS/MS	By MS/MS	By MS/MS	By matchin	By matchin	By MS/MS
4.8891	By MS/MS	By MS/MS	By MS/MS	By MS/MS	By matchin	By matchin	By matchin	By matchin
9.9299	By matchin	By matchin	By MS/MS	By MS/MS	By MS/MS	By matchin	By matchin	By matchin
7.546		By MS/MS	By MS/MS	By MS/MS	By MS/MS	By MS/MS	By MS/MS	By MS/MS
1.7182							By matchin	By MS/MS
2.334	By MS/MS	By MS/MS	By matchin	By MS/MS	By matchin	By matchin	By matchin	By matchin

2.2171	By matchin	By matchin	By matchin	By matchin	By MS/MS	By MS/MS		
1.9319		By MS/MS	By MS/MS				By matching	
78.79	By MS/MS	By MS/MS	By MS/MS	By MS/MS	By MS/MS	By MS/MS	By MS/MS	By MS/MS
33.434	By MS/MS	By MS/MS	By MS/MS	By MS/MS	By MS/MS	By MS/MS	By MS/MS	By MS/MS
19.911	By matchin	By MS/MS	By MS/MS	By MS/MS	By MS/MS	By MS/MS	By MS/MS	By MS/MS
1.8162							By MS/MS	By MS/MS
2.4589	By matchin	By matchin	By matchin	By matchin	By matchin	By matchin	By MS/MS	By MS/MS
2.3809					By matchin	By matchin	By MS/MS	By matchin
13.554	By matchin	By MS/MS	By MS/MS	By MS/MS	By MS/MS	By MS/MS	By MS/MS	By matchin
2.4146	By matchin	By matchin	By MS/MS	By MS/MS	By MS/MS	By matchin	By matchin	By MS/MS
2.9529						By MS/MS	By matchin	By MS/MS
3.0523	By MS/MS	By matchin	By matchin	By matchin	By matchin	By MS/MS	By MS/MS	By MS/MS
9.337	By MS/MS	By MS/MS	By MS/MS	By MS/MS	By matchin	By MS/MS	By matchin	By MS/MS
3.0915	By MS/MS	By MS/MS	By MS/MS	By MS/MS		By MS/MS	By MS/MS	By matchin
5.3417	By MS/MS	By MS/MS	By MS/MS	By MS/MS	By MS/MS	By MS/MS	By matchin	By matchin
4.8971		By matchin	By MS/MS	By MS/MS	By MS/MS	By matching		
4.5914			By matchin	By matchin	By matchin	By matchin	By MS/MS	By MS/MS

Sequence c	Sequence c	Sequence c	Sequence c	Sequence c	Sequence c	Sequence c	Sequence c	Intensity
68.6	68.6	56.9	56.9	56.9	56.9	61.8	68.6	1.01E+08
10	11.5	10.7	6.4	9.7	11.5	11.3	11.3	9426100
9.7	9.7	9.7	9.7	9.7	9.7	9.7	9.7	1429000
36.9	36.9	34	34	36.9	34	36.9	36.9	35065000
51	39.2	51.8	51.4	54.5	58.4	61.2	54.5	73396000
34.1	30.5	45.1	41.1	39.8	45.1	43.9	43.1	18080000
27.1	23.5	29.1	28.7	31.6	32.4	31.6	27.5	28981000
66.7	63.9	60.6	52.8	67.1	63.9	67.1	71.3	2.43E+08
40.8	40.8	38	38	40.8	38	40.8	45.7	34360000
42	40	44.9	49.8	47.8	49.4	47.3	47.3	1.06E+08
1	0	1	1	1	2	2	2.1	556860
6.3	6.3	6.3	6.3	6.3	6.3	6.3	0	1998800
6.5	6.5	0	0	6.5	0	0	0	610700
23.3	26.4	26.4	26.4	26.4	26.4	19	23.6	16519000
4	2.3	0	0	1.3	2.3	3.7	3.7	1052600
26.8	26.8	20.6	17.6	6.7	23.8	23.8	23.6	9018600
16.9	16.9	7.9	14.4	5.4	20.2	20.2	20.2	6197800
15.4	15.4	15.4	11.6	11.3	15.4	18.4	14.4	5644200
18.9	18.9	5.4	5.4	16.5	18.9	8	16.5	2762500
24.9	19.2	19.4	23.8	13.4	28.9	31.6	31.6	11123000
19	14.4	9.5	19	10.3	19	23.6	19	5347500
4.2	5.9	0.9	3.1	2.8	5.4	5.4	10.7	3130900
11.9	11.9	0	11.9	0	17.3	17.3	17.3	1035100
17.5	12.6	9	9	6.2	19.4	28.9	25.8	7285000
4.1	9.2	2.1	2.1	4.8	14	14	8.5	3713700
8	8	10.9	8	10.9	17.2	19.8	22.3	4552300
4.2	4.2	4.2	4.2	0	4.2	4.2	4.2	835590
10.5	15.5	9.1	9.1	5.7	9.4	12.3	12.3	10713000
14.9	12.4	14.9	12.4	6.5	14.9	14.9	14.9	4560300
0	0	0	2.6	0	2.6	0	4.7	444720
0	0	0	0	3.1	3.1	10	9.7	659460
4	17.5	13.6	17.5	17.5	23.2	23.2	23.2	1828000
10.5	10.5	10.5	10.5	10.5	10.5	10.5	17.6	1102400
0	0	6.1	6.1	13.3	13.3	22.1	17.7	3580900
15	15	15	15	15	15	15	15	1139400
18.2	18.2	18.2	18.2	18.2	18.2	18.2	18.2	2236500
22.5	29.5	29.5	29.5	22.5	29.5	22.5	29.5	4155300
15.1	23.6	23.6	23.6	23.6	23.6	23.6	15.9	6692600
9.8	9.8	9.8	9.8	3.7	9.8	9.8	9.8	2403700
16.1	16.1	16.1	0	16.1	16.1	16.1	0	705820
11.9	8.5	11.9	7.2	8.5	8.5	8.5	3.8	2206000
6.6	6.6	13.2	6.6	6.6	6.6	6.6	6.6	838480
24.3	24.3	11.6	7.5	19.1	24.3	19.1	7.5	2888100
12	12	12	12	8	4	0	4	1530100
10.2	0	10.2	10.2	10.2	4.8	4.8	0	759050
29.1	29.1	29.1	29.1	29.1	29.1	17.5	0	1216900
3.3	3.3	3.3	3.3	3.3	3.3	3.3	3.3	915050
10.4	10.4	10.4	10.4	0	10.4	10.4	10.4	991220

19.8	19.8	19.8	19.8	13.4	19.8	13.4	7	3646100
4	6.8	4	4	0	6.8	2.8	6.8	972770
16.3	19.6	21.8	20.8	15.7	16.6	15.5	10.7	22405000
5.2	5.2	5.2	5.2	5.2	5.2	5.2	5.2	970750
12.7	12.7	23.5	23.5	7.2	18.1	7.2	23.5	2356600
15.4	7.7	10.2	7.7	13.6	10.4	10.4	10.4	3896300
53.2	53.2	53.2	21.3	53.2	53.2	0	0	915350
15	12.8	12.8	15	15	12.8	0	7.2	1479800
11.1	0	11.1	11.1	11.1	11.1	11.1	11.1	2292100
11.8	11.8	11.8	11.8	5.7	11.8	5.7	5.7	1069000
11.1	17.1	11.1	11.1	11.1	0	0	0	1099600
6.1	6.1	6.1	6.1	6.1	6.1	6.1	6.1	928410
8.4	2.6	8.4	8.4	8.4	8.4	5.8	5.8	1561400
0	5.1	0	5.1	0	0	0	0	443630
15	15	15	15	15	15	15	0	813950
14.9	14.9	14.9	14.9	14.9	14.9	7.4	7.4	2305400
5.7	5.7	5.7	5.7	5.7	5.7	0	0	595410
13.1	13.1	13.1	13.1	13.1	13.1	13.1	13.1	913760
14.8	23	14.8	14.8	0	14.8	8.1	0	1084400
11.6	11.6	11.6	11.6	20.5	11.6	11.6	11.6	1093300
3.7	3.7	3.7	3.7	0	3.7	3.7	3.7	705420
16.5	16.5	9.6	4.3	9.6	9.6	11.3	16.5	19303000
35.2	41.8	41.8	48.7	42.9	48.7	54	28.4	32519000
8.6	8.6	8.6	8.6	8.6	4.8	3.9	8.6	2150500
5.4	5.4	5.4	5.4	5.4	5.4	0	5.4	1646600
3.1	0	3.1	3.1	3.1	3.1	3.1	3.1	2674500
9.7	9.7	5.5	18.8	0	18.8	18.8	27.9	5850500
48.1	48.1	48.1	41.1	41.1	41.8	53.2	43	36741000
53.8	53.8	53.8	46.2	53.8	46.2	53.8	53.8	28972000
43	37.1	37.1	22.5	19.2	29.1	37.1	37.1	18051000
37.7	38.4	31.8	30.5	31.8	31.1	37.7	38.4	46066000
40	33	33	33	24	40	47	47	16204000
33.6	28.1	25.3	25.3	19.9	39	39	33.6	20350000
51.9	45.2	48.9	42.2	28.9	48.9	48.9	48.1	23555000
32.9	32.9	32.9	32.9	27.6	32.9	32.9	32.9	36391000
43.4	29.7	42.8	42.8	22.8	42.8	42.8	36.6	22246000
27	29.4	29.4	24.2	26.6	26.6	31.7	31.7	42332000
22.7	22.7	22.7	22.7	22.7	19.3	22.7	22.7	8200700
34.6	23.5	40.7	23.5	12.3	42	42	46.9	8296900
28.7	28.7	23.1	28.7	23.1	28.7	28.7	28.7	12464000
17.6	17.6	17.6	17.6	17.6	17.6	26.7	26.7	9131500
15.2	15.2	15.2	15.2	8	22.4	15.2	22.4	10712000
31.3	31.3	31.3	20.9	20.9	31.3	31.3	20.9	13999000
39.3	39.3	38.1	38.1	38.1	39.3	39.3	39.3	22209000
17.4	33.3	33.3	33.3	17.4	33.3	33.3	33.3	24747000
12.5	12.5	12.5	12.5	12.5	12.5	32.1	0	2060700
51.4	51	47.7	37.4	31.7	42.8	47.7	45.7	75349000
10.2	10.2	10.2	10.2	10.2	10.2	10.2	10.2	5821100
40.9	47.3	39.4	35.6	33	40.9	43.6	47.3	50478000
35.7	41.4	27.8	27	27.8	30.8	40.3	40.3	50438000

35	35	35	13.5	18.5	28	28.5	28.5	20623000
22.9	26.1	18.9	22.9	18.1	22.9	18.9	26.1	49899000
12.9	24.2	12.9	8.8	8.8	8.8	24.2	12.9	17974000
50	50	50	43.8	39.9	43.8	49.5	50	82760000
37.6	37.6	34	34	26.3	37.6	37.6	38.7	52763000
40.7	40.7	34.2	30.4	28.5	40.7	40.7	37.6	56848000
48.2	46.5	46.3	44.4	38.4	45.4	48.4	47.6	3.08E+08
2.7	2.7	2.7	2.7	2.7	2.7	2.7	1.4	1363900
4.4	2.5	4.4	4.4	2.5	2.5	2.5	1.9	718560
51.7	55.7	55.7	51.7	55.7	51.7	55.7	55.7	5.33E+08
27.5	38.9	38.9	41.7	26.3	41.7	41.7	38.9	59692000
28.9	28.9	28.9	28.9	14.9	28.9	28.9	28.9	20113000
79.1	79.1	78.3	78.3	71.3	47	79.1	47	67609000
36.5	40.5	35.5	31	31	34.5	40	35.5	48632000
33.6	33.6	27.2	31.3	27.2	30.9	33.6	36.9	28764000
23	23	19.1	23	18.5	18.5	23	16.3	34762000
22.6	22.6	18.6	22.6	18.1	18.1	22.6	16.4	494060
45.5	54.5	45.5	45.5	45.5	45.5	54.5	54.5	60744000
25.1	29.9	25.1	25.1	20.4	29.4	25.1	29.9	42617000
27.6	33.5	27.6	27.6	27.6	27.6	33.5	30	25268000
36.3	36.3	36.3	36.3	36.3	26.6	36.3	36.3	27297000
25	25	25	25	25	25	25	25	38857000
34.3	34.3	34.3	18.9	18.9	8.3	39.6	44.4	17867000
27.4	27.4	27.4	27.4	27.4	27.4	27.4	27.4	42542000
37.6	31.9	37.6	37.6	37.6	37.6	37.6	37.6	33305000
29.9	17.2	29.9	17.2	29.9	17.2	29.9	29.9	8741300
51.1	51.1	51.1	51.1	51.1	51.1	51.1	51.1	28258000
37.9	37.9	37.9	37.9	27.1	37.9	37.9	37.9	34178000
21.5	26.6	20.3	26.6	14.6	20.3	26.6	26.6	20866000
28.9	28.9	28.9	28.9	28.9	21.5	29.8	28.9	24355000
23.4	29	17.9	17.9	11.7	17.9	29	29	30945000
30.1	36	30.1	14.7	6.6	36	36	36	20292000
30.6	30.6	30.6	30.6	21.3	30.6	30.6	38	25411000
35.7	35.7	35.7	35.7	35.7	35.7	35.7	42	25276000
0	14.5	14.5	14.5	14.5	14.5	14.5	14.5	7241700
34.2	39.7	27.8	25.1	27.8	27.8	38	36.2	72703000
45.2	59.1	59.1	48.7	59.1	48.7	59.1	45.2	33280000
44.4	44.4	37	37	37	15.7	23.1	44.4	17216000
33.1	33.1	33.1	33.1	33.1	33.1	33.1	38.3	20762000
35.9	36.8	36.8	36.8	35.9	29.1	36.8	29.1	28445000
24	24	24	10.4	10.4	24	24	24	10288000
35.1	35.1	35.1	35.1	44.7	44.7	44.7	35.1	19591000
28.6	28.6	28.6	28.6	28.6	28.6	28.6	28.6	21966000
15.2	15.2	15.2	15.2	15.2	15.2	15.2	15.2	15492000
29.4	55.9	55.9	11.8	11.8	29.4	55.9	51.5	10511000
15.6	15.6	15.6	15.6	15.6	15.6	15.6	15.6	2270100
34.2	34.2	31.9	30.2	28.3	30.2	32.3	32.8	91580000
27.9	38	32	24.6	17.5	38	38	38	40237000
26	26	29.5	26	23.3	29.5	29.5	26.7	47588000
33.2	33.2	33.2	33.2	22.6	33.2	27.4	33.2	42718000

40.2	44	44	44	24.8	44	43.6	37.2	67566000
5.1	5.1	5.1	5.1	5.1	5.1	5.1	5.1	1148200
22.6	22.2	22.2	22.2	22.2	24.9	25.3	22.6	37192000
35.4	28.1	28.1	20.8	15.1	28.1	35.4	35.4	15463000
0	9.1	0	0	0	9.1	9.1	17.3	1634200
11.5	15.1	17.4	13.8	10.2	24	28.5	30.9	21744000
0	0	0	8.9	8.9	8.9	8.4	8.4	678190
0	7.7	7.7	16	7.7	7.7	7.7	16	613610
45.1	46.3	44.6	43.9	43.6	46.8	46.5	46.5	3.92E+08
12.8	12.8	12.8	14.3	7.8	12.4	10.9	9.1	22183000
3.5	0	0	0	0	3.5	3.5	3.5	1530500
0.8	0.8	1.9	1.3	1.3	1.3	1.9	1.3	998090
0	4	4	0	4	0	4	0	774980
5	6.8	6.8	0	6.8	11.8	11.8	5	3042800
21.1	21.1	21.1	21.1	21.1	21.1	21.1	21.1	13141000
1	1	1	0	1	1	1	1	1214500
9	0	15.3	9	0	27.7	27.7	27.7	8504000
8.2	0	8.2	8.2	0	13.8	13.8	13.8	2937600
11.4	13.9	9.6	9.6	10.7	11.4	13	15	12072000
69.6	69.6	69.6	61.6	61.6	69.6	69.6	69.6	20226000
69.6	69.6	69.6	61.6	61.6	69.6	69.6	69.6	1.82E+09
26.6	26.6	26.6	26.6	26.6	26.6	26.6	26.6	2.23E+08
12.8	12.1	11	12.8	12.8	12.8	19.8	19.8	6739900
0	6.6	3	6.3	6.3	9.9	14.7	12.7	3589800
10.2	5.6	5.6	5.6	5.6	8.1	16.4	11.8	2972100
3.3	8	8	3	6.3	14	14	9.3	2754200
0	0	0	0	0	0	13.1	0	357350
26.5	26.5	19.5	19.5	30.1	26.5	37.2	37.2	4487300
25.9	25.9	25.9	25.9	25.9	25.9	25.9	25.9	2058600
0	7.8	7.8	7.8	0	7.8	7.8	7.8	770810
18.2	13.2	17.9	18.2	13.4	23.2	23.2	23.2	10460000
26	26	26	26	26	26	26	26	7974000
3.1	7.3	7.6	10.8	7.6	17.7	33	25.3	6137500
0	0	0	0	9.6	0	0	0	234580
14.7	14.7	9	5.8	9	14.7	14.7	9	1479600
1.6	1.6	2.8	2.8	2.8	2.8	2.8	2.8	19306000
11.3	11.3	11.3	11.3	7.2	11.3	11.3	11.3	9311800
10.6	10.6	10.6	10.6	10.6	10.6	10.6	10.6	10525000
20.6	28.9	28.9	28.9	37.1	28.9	45.4	28.9	14463000
10.3	10.3	10.3	10.3	10.3	10.3	0	0	1070800
18.7	18.7	18.7	18.7	18.7	18.7	11	7.7	1592700
19	19	19	19	19	19	19	19	11606000
6.6	14.5	19.3	19.3	4.8	11.4	11.4	11.4	3384100
15.9	15.9	15.9	11	15.9	15.9	15.9	15.9	1634400
13.8	13.8	13.8	0	0	13.8	0	13.8	930880
38.1	58.7	17.5	0	17.5	58.7	58.7	58.7	2408100
4	4	4	0	4	7.7	4	4	1759100
9.8	0	0	0	0	9.8	9.8	9.8	752800
26.6	28.9	30.3	22.2	16.2	32.2	39.1	39.6	28207000
24.6	16.4	15.1	14.2	18.5	15.1	22	19.8	5006100

4	4	9	9	9	9	9	0	2201100
10	16.2	20.5	10	20.5	32.4	32.4	32.4	3691200
0	0	0	0	3.1	5.8	5.8	8.9	683560
0	0	7	9.2	0	15.4	15.4	12.3	4049100
16.9	18.6	22.2	22.2	16.7	24.3	24.3	26.4	23862000
20.1	29.4	20.1	20.1	13.8	29.4	17	20.1	15238000
0	0	0	18.4	0	18.4	18.4	18.4	823160
42.6	42.6	42.3	43	32.9	42.6	40.3	39.6	1.61E+08
42.3	42.3	42.3	42.3	32.6	42.3	39.9	42.3	17010000
7.7	10.9	10.9	10.9	6.2	7.9	10.9	7.9	2976900
29.8	30.4	38.1	30.4	13.8	38.1	43.6	43.6	24659000
33.1	33.1	33.1	33.1	16.5	33.1	33.1	33.1	3977200
31.3	31.3	21.3	31.3	14	38	38	38	1132400
24.6	24.6	24.6	24.6	12.6	24.6	12.6	24.6	3748900
10.2	10.2	10.2	10.2	10.2	10.2	10.2	10.2	3474100
0	0	13.3	8.3	13.3	13.3	13.3	20.6	1078500
1.3	2.3	2.3	2.3	2.3	2.3	1.3	2.3	1505000
3.6	3.6	3.6	3.6	3.1	2.8	3.6	3.6	4457300
0	4.2	4.2	4.2	4.2	4.2	4.2	4.2	1004700
0	0	6.8	0	0	6.8	0	6.8	0
6.4	6.4	6.4	6.4	0	6.4	6.4	6.4	1112200
9.4	9.4	8	4.2	5.7	9	7.7	7.7	4942100
0	0	4.6	10.8	8.3	9.2	9.2	9.2	1844000
4.5	7	7	7	4.5	7	9.4	14.2	2653100
28.1	27.5	26	21.3	26.2	29.1	35.2	33.6	28633000
5.8	5.8	5.8	8.1	8.1	7.4	3.1	5.8	1543400
26.9	35.9	26	18.9	18.9	39.9	36.5	33.7	48850000
26.6	33.3	29	21.2	17.8	44.1	34	31	5442900
14.6	23.5	11.8	5	5	13.6	26.3	19.5	1561800
6	6	6	9.5	9.5	9.5	18.7	18.7	4094900
24	24	24	21.6	14	21.6	19.8	16	20032000
3.7	3.7	3.7	3.7	0	0	0	0	728740
0	0	0	1.5	1.5	1.5	1.5	1.5	1155800
7.3	4.5	4.5	4.5	2.8	7.3	4.5	0	1153000
22	26.3	29.1	29.4	20.3	41.6	46.3	43	54444000
16.4	22.5	28	26	16.8	37.8	43.6	42.7	23709000
14.2	6.5	14.2	11.9	3.5	19.8	12.1	19.8	3844000
5.4	5.4	0	0	5.4	5.4	5.4	5.4	1758100
0	0	14.3	0	0	0	0	14.3	454260
35.5	40.8	42.9	36.4	42.9	49.8	60.1	48.2	3.07E+08
2.1	1.1	2.1	2.1	2.1	2.1	0	1.1	873650
0	0	0	0	3	0	0	3	2839100
14.8	18.8	22.7	17.6	17.6	24.6	14.5	15.6	4046500
3.5	3.5	0	3.5	3.5	3.5	3.5	3.5	393600
9.9	9.9	9.9	9.9	7.8	9.9	9.9	9.9	5422400
18.6	18.6	10.3	10.3	6.4	18.6	18.6	18.6	7256700
11.9	17.2	11.9	11.9	11.9	13.1	17.2	17.2	6755800
6.9	4.3	8.7	8.7	6.9	8.8	10.6	8.8	3024600
5.7	5.7	5.7	5.7	5.7	2.7	5.7	5.7	2812200
11.2	13.4	13.4	14.7	14.7	12.3	14.7	10.1	12880000

9.1	10.4	11.7	10.4	8.9	10.4	11.7	10.4	7819800
3.2	0	3.2	3.2	3.2	0	0	3.2	753680
1.1	1.1	1.1	0	0	0	1.1	1.1	280270
63	62.1	62.1	62.1	55.2	62.1	64.5	56.9	3.05E+08
4.9	4.9	4.9	0	0	4.9	4.9	4.9	619350
12.3	11.4	10.8	9.1	7.8	10.8	15.9	17.6	5975100
58.4	58.4	57.8	57.8	57.5	57.5	58.4	56.3	9.33E+08
29.7	19.8	35	30	27.2	35	34.4	38.4	16416000
21.9	40.4	35.4	35.4	34.2	40.4	40.4	36.7	13233000
28.4	23.1	23.4	23.4	15	30.9	30.6	25.9	26690000
22.1	24.7	16.7	21.9	9	16.5	16.7	22.5	9245500
31.2	31.2	27.8	30.6	16.5	33.6	23.2	26.9	27543000
3.2	4.8	6.8	6.6	7.4	4.9	4.7	4.4	8872900
1.7	1.7	1.7	3.1	1.7	3.1	3.1	1.7	1456400
2	2	4.7	4.7	3.8	7.4	7.4	7.6	4491300
8.4	0	8.4	8.4	8.4	13.1	13.1	13.1	888520
0	3.6	2.3	3.2	1.2	1.2	1.2	2.4	1055100
2.7	5.6	5.6	5.6	1.5	6.5	13.3	12.1	2986100
7.1	7.1	9	5.3	5.3	9	5.5	3.7	1435200
0	1.5	0	0	0	1.5	0	1.5	250630
0	5.5	0	0	0	5.5	5.5	5.5	782290
7.8	13.5	7.8	7.8	0	7.8	7.8	7.8	527540
1.5	1.5	1.1	1.7	1.1	1.1	0.9	1.1	12427000
13.7	13.7	13.7	13.7	0	13.7	13.7	13.7	403590
4.9	4.9	4.9	4.9	4.9	4.9	4.9	2.4	2345900
22.8	0	22.8	22.8	22.8	0	9.6	0	1247600
0	0	3.2	0	0	10.3	10.3	10.3	2394100
7.9	7.9	7.9	7.9	7.9	7.9	15.9	22.6	1947200
18.6	18.6	18.6	16.7	16.7	14.4	10.5	11	17456000
7.3	8.7	9.4	8.1	9.9	9.9	7.6	8.7	7042600
0	0	0	2.9	2.9	2.9	2.9	2.9	270760
12.1	10.8	8.9	7.3	3.9	10.8	15.6	14.7	4160000
11.3	13.3	7.4	11.3	2.6	13.3	7.4	13.3	6525600
8.2	10.9	10.9	10.9	12.4	13.1	9.7	14.6	8908100
0	0	5.8	5.8	5.8	14.3	10.9	11.4	2554300
28.8	31.4	30.9	29.1	25.3	30.9	31.4	29.1	84658000
31.7	26.5	23.8	30.1	17.2	33.5	41.1	38.7	20341000
2.8	2.8	2.8	0	1.4	0	1.4	1.4	823730
8.8	8.8	8.8	8.8	6.5	8.8	7.5	8.8	9575400
9.3	9.3	9.3	9.3	9.3	9.3	9.3	9.3	1213000
0.9	0	2.4	3.3	3.3	3.3	2.4	0	654910
13.1	9.4	9.4	13.1	13.1	9.4	9.4	9.4	4219100
9	9	0	5	5	5	9	9	2945100
30.3	32.3	30.3	26.2	24.6	27.7	22.1	25.6	20219000
4.4	4.4	4.4	4.4	4.4	4.4	4.4	4.4	5177200
43	41.6	32.9	35.3	38	30.9	34.7	32.2	2.75E+08
51.8	51.8	42.7	42.7	43.3	47.1	49.9	47.3	3.14E+08
60.2	41.6	54.7	41.6	50.3	35.4	31.1	37.3	23697000
13.7	13.7	13.7	13.7	13.7	13.7	13.7	13.7	16726000
30.4	30.4	30.4	30.4	30.4	30.4	30.4	30.4	6215200

23.6	47.3	47.3	23.6	0	23.6	47.3	0	5558100
31.6	51.3	31.6	51.3	17.1	31.6	31.6	31.6	9552700
33.3	33.3	26.6	29.6	17.5	26.6	23.9	30.3	28448000
27.2	46.5	37.6	20.7	10.3	34.3	40.8	40.8	26723000
45.4	45.4	45.4	45.4	32.4	32.4	45.4	45.4	17342000
11.1	9.6	11.1	11.1	9.7	7.7	5.8	9.2	7684000
5.3	5.3	5.3	5.3	5.3	0	0	5.3	449570
4.4	4.4	4.4	4.4	4.4	2.3	2.3	2.3	1472100
3.7	2	2	3.2	2	9.5	9.5	6.3	1867200
4.7	4.7	0	2.5	0	4.7	4.7	4.7	1269300
2.2	2.2	2.2	2.2	0	2.2	2.2	2.2	1177900
1.6	1.6	0	0	1.6	1.6	1.6	1.6	481630
7.7	9.1	13.6	13.6	11.9	18.7	20.8	25.1	40591000
7.8	7.8	6.7	6.7	6.7	6.7	7.8	7.8	2982800
6.5	5.5	0	0	3.1	4.3	5.5	5.5	1219300
22.2	19.4	19.6	16.8	12.6	18.4	22.2	20.7	18282000
9.8	8.2	8.2	6.2	9.8	6.2	6.2	6.2	2685100
22.1	22.1	22.1	22.1	14.2	7.9	7.9	0	2874800
1.7	3.4	1.7	3.4	0	5.1	3.4	3.4	1476400
19.5	18.9	17.3	14.8	12.4	18.9	20.6	23.6	48721000
4.2	11.2	8	6.4	6.7	16.1	18.8	13.4	5712100
11	13	12.8	14.3	9.4	14.5	11.5	14.3	12171000
3.4	3.4	0	3.4	3.4	3.4	7.2	7.2	699630
4.5	6.6	12.9	8.9	13	6.6	6.6	4.5	12333000
13.3	16.2	5.9	5.6	0	16.2	21.8	19	1442100
18	22.5	12.2	15.6	12	18.3	22.2	17	16640000
0	0	0	0	0	0	3	3	294950
8.9	6.1	8.9	8.9	6.2	6.1	4.7	8.6	2914500
4.1	4.1	4.1	4.1	4.1	2	2	2	1127400
0	0	20.3	20.3	20.3	0	20.3	0	279150
5.2	0	5.2	5.2	0	5.2	5.2	5.2	1199800
5	4.7	4.7	2.3	4.7	4.7	7.5	7.5	2653400
1.8	1.8	0	0	0	1.2	3.1	1.2	541080
27	27	27	27	27	27	27	27	21815000
0	0.3	0	0	0	0	0	0	313070
2.5	2.8	2.5	2.5	0	11.3	11.3	11.3	2086700
0	0	3.3	3.3	6.9	13.9	6.9	10.2	1373900
46.3	46.3	47.8	47.8	47.8	47.8	36.1	42.9	20941000
9.3	9.3	13.4	13.4	19.5	19.5	15.4	13	11423000
22.2	22.2	0	22.2	22.2	0	22.2	22.2	682650
13.3	13.3	13.3	13.3	13.3	13.3	13.3	8.6	7165800
11.1	8	11.1	11.1	11.1	8	11.1	8.7	3920900
35.3	35.3	16.8	16.8	16.8	16.8	35.3	35.3	12454000
2.4	2.4	2.4	2.4	2.4	2.4	2.4	2.4	919460
0	4	0	0	0	0	4	4	437420
10.1	7.6	10.1	3.8	3.8	3.8	10.1	10.1	1331300
0	0	12.3	0	5.6	26.7	26.7	12.3	1739000
4.8	6.7	4.8	2.1	2.7	6.7	6.7	4.1	1415100
17.9	15.7	16.1	9.6	14.6	15.1	19	19.1	26267000
14.1	9.3	4.8	0	4.8	4.8	4.8	0	945780

14.9	17.9	15.1	15.7	12	31.6	31.6	31.6	12641000
0.3	0.3	0.3	0.3	0.3	0.3	0	0.3	949020
10.5	0	10.5	16.4	16.4	16.4	16.4	10.5	2380600
16.3	16.3	16.3	16.3	16.3	16.3	16.3	16.3	1912100
9.2	8.8	12.9	12.9	9.2	12.9	12.9	9.2	3695500
0	0	0	0	0	0	4.6	4.6	178230
5.5	13.4	7.9	0	0	13.4	5.5	13.4	1536700
6.8	14.3	14.3	14.3	6.8	14.3	14.3	6.8	2877300
0	1.3	0	0	0	0	0.5	1.8	2397500
3.7	0	0	4.6	14.8	17.8	17.8	17.8	1560300
4.9	4.9	0	4.9	0	4.9	4.9	0	216470
2.6	2.4	2.6	6.7	6.7	7.7	9.3	9.3	3314300
18.8	17.6	15.8	13.7	11.1	18.8	17.9	15	18255000
10.5	10.5	10.5	10.5	10.5	10.5	10.5	8.2	11459000
6.8	6.8	6.8	6.8	6.8	6.8	6.8	6.8	578800
10	12.3	7.5	6.9	5.9	8	10.9	10.6	18134000
1.8	0	1.8	3.1	3.1	3.1	1.8	1.8	2579300
8	8	8	8	8	6.6	6.6	8	6854000
16.4	16.4	16.4	24.6	10.3	16.4	16.4	6.2	3372900
2.2	0	0	2.2	2.2	2.2	2.2	0	2648600
23.4	25.9	24.7	23	17.3	25.9	26.1	20	49784000
14	15.7	14	14	9.4	14	10.5	12.2	8313500
8.9	9.2	10.8	8.9	9	10.8	9.2	9.2	20609000
45.4	45.4	40.5	29.7	28.6	45.4	45.4	45.4	9259700
31.2	30.9	25.5	27.5	21.6	28.7	28.2	27.7	1.22E+08
23.3	23.3	38	23.3	14.1	38	38	38	7088200
2.5	7.1	5.9	7.7	4.2	7.3	10.4	11.2	6194700
10	11.4	7.4	5.9	1.4	11.4	11.4	11.4	4518400
0	0	0	7.3	13.7	12.8	11.8	17.3	1994400
9.1	4.7	12.8	12.8	9.1	16.4	31.2	28.2	6129000
47	47	45.1	45.1	41.7	45.1	47	47	1.53E+08
7.1	0	12.9	12.9	5.8	12.9	7.1	12.9	2436100
10	4.5	7.1	10	7.1	5.5	7.3	4.5	4548700
10	10	10	10	10	10	10	10	750130
3.5	3.5	3.5	5.6	3.5	5.6	7.3	7.3	4587600
11.2	15.8	15.8	19.2	11.2	19.2	19.2	15.8	9911200
10.1	10.5	30	30	31	53.8	54.5	58.8	10277000
7.7	6.7	8.8	5.5	8.6	7.4	5.5	5.3	5297000
12.4	14	14	14	11.4	12.7	11.6	9.9	13100000
7.9	6.1	7.9	6.1	4.9	6.1	1.4	6.4	2433700
8.9	13.6	8.9	3.8	3.8	3.8	8.5	13.6	2637800
4.7	4.7	4.7	0	0	0	0	9.9	647940
11	11	4.8	4.8	0	11	11	6.1	2061100
6.2	2.4	2.4	2.4	2.4	2.4	6.2	2.4	850040
4.8	4.8	0	3.6	4.8	3.6	3.6	0	476000
11.8	14	12.7	16.3	16.7	14	10.4	13.7	6754900
21.7	21.7	21.7	6.8	0	6.8	41.6	38.9	5650300
21.3	19.2	17.8	24.2	16.2	21	26.4	27	24447000
6.8	8.1	9.7	10.1	8.5	11.4	4.9	11.4	4194900
16.2	20.1	17.2	15.9	18.9	17.4	21.5	17.6	18985000

9.1	9.6	17	11.6	9.9	17	10.1	17	4153400
11.2	11.2	11.2	7.9	7.9	7.9	8.3	11.2	6819900
2.5	1.8	3.6	3.6	2.9	2.4	2.5	2.5	6935000
51.1	51.1	51.1	51.1	41	46.6	46.6	51.1	55693000
0.9	0.9	2.3	2.3	1.4	2.3	2.3	0.9	1101900
3.1	3.1	3.1	3.1	1.2	1.2	0	0.6	1076500
15.7	13.1	15.7	13.1	13.1	13.1	13.1	13.1	6389700
10.3	10.3	10.3	10.3	6.7	10.3	10.3	10.3	4714700
26.7	31.6	21.8	22.8	27.7	26.7	31.6	31.6	30236000
29.6	29.6	22.2	29.6	29.6	29.6	29.6	29.6	8933100
17.4	13.2	17.4	17.4	4.1	17.4	13.2	17.4	2316600
6.3	3.6	6.3	6.3	2.3	3.6	2.3	5	2606800
42.6	42.6	42.6	42.6	0	42.6	42.6	42.6	2145300
4.6	4.6	4.6	4.6	8.9	4.6	4.6	4.6	1189600
3.7	3.7	4.3	1.1	0	2.2	2.6	6.9	1969000
11.5	22.5	20.9	20.9	9.9	15.2	26.7	22.5	7659800
5	1.2	0	3.5	3.5	6.2	6.2	7.4	1251700
3.4	3.7	7.1	7.1	3.4	7.1	7.1	7.1	1812300
8.2	12.9	20.6	12.9	12.9	29.4	29.4	36.5	8029300
0	0	0	0	0	0	2.6	0	0
16.1	28	16.1	28	28	16.1	16.1	16.1	1335400
41.6	41.6	41.6	41.6	14.3	27.3	41.6	27.3	2318100
6.3	6.3	6.3	6.3	0	6.3	6.3	6.3	572520
25.9	25.9	25.9	25.9	22.8	25.9	25.9	25.9	7313500
25.3	43.6	39.8	39.8	20.3	48.5	52.3	52.3	27309000
4.3	4.3	6	6	4	4.3	4.3	4.3	2756200
0	0	0	1.7	1.7	0	1.7	1.7	494400
3.9	3.9	3.9	3.9	0	3.9	3.9	0	818040
15.3	15.3	15.3	15.3	0	15.3	0	15.3	1454100
17.1	17.1	17.1	17.1	17.1	17.1	17.1	17.1	1595600
23	23	14.2	23	36.1	23.5	30.1	36.1	14522000
1.7	1.7	0.6	0.6	1.7	1.7	1.7	1.7	1669800
18.1	20.5	20.5	20.5	14.1	18.1	12.8	15	38479000
22.9	25.4	23	19.5	12.7	29.2	32.6	32.2	82461000
13.8	9.6	13.8	13.8	13.8	13.3	13.3	13.3	5553400
3.3	3.8	7.1	7.1	7.1	7.1	7.1	3.3	1272100
25.6	25.6	25.6	13.2	13.2	13.2	13.2	13.2	2653400
19	19	14.7	19	11.4	19	19	19	12320000
6.1	6.1	6.1	6.1	6.1	6.1	6.1	6.1	3252000
12.9	12.9	12.9	12.9	12.9	12.9	12.9	12.9	6832300
7.9	14.5	14.5	7.9	0	14.5	14.5	14.5	2975500
11.9	9	6.1	6.1	6.1	11.9	9	5.6	2417400
0	5.4	0	0	0	0	5.4	5.4	287520
0	4.9	4.9	4.9	4.9	4.9	4.9	4.9	527130
8.5	2.2	8.4	10	9.9	8.5	7	7.2	3285500
6.5	10.8	10.8	10.8	10.8	10.8	7.9	3.5	1867900
0	1.1	0	1.1	0	0	1.1	3.1	481180
25.4	25.4	31	38.7	31	38.7	38.7	43.7	18114000
11.6	11.6	11.6	11.6	11.6	0	0	0	362110
6.9	5.2	6.9	3.6	3.3	8.2	9.3	8.7	7792900

3	3	3	3	3	4.6	3	2.4	2216100
14.8	10.4	9.5	9.5	11.7	9.5	13.8	14.5	11742000
8.8	10.8	8.8	8.8	8.8	9	11	10.2	3335200
13	13	2.7	2.7	2.7	13	13	13	2046600
1.9	1.9	1.9	0	3	3.2	3.2	5.7	2540400
63.9	59.6	54.8	54.8	46.4	59.6	63.9	59.6	1.1E+08
0	0	2.2	0	2.2	0	0	0	170090
13.5	13.5	7.9	7.9	7.9	7.9	12.1	13.5	6779700
7.3	7.3	7.3	7.3	7.3	7.3	7.3	7.3	1061600
8.5	8.5	8.5	8.5	0	8.5	8.5	0	516860
0	26.1	0	26.1	26.1	0	26.1	26.1	375100
31.1	24.5	31.1	31.1	31.1	31.1	24.5	31.1	4945500
3.8	3.8	3.8	3.8	1.6	5.5	7.8	6.8	2537100
5.8	8.6	8.6	7.3	10	5.8	8.2	4.1	17056000
33.3	28	28	23	28.7	33.3	33.3	28	64927000
0	0	0	0	0	0	0	1	187230
1.8	4	1.6	3.7	2.3	4.8	5.8	7.1	1615800
1.4	1.2	1.4	1.4	1.4	2.6	2.6	2.3	775930
7.2	0	0	7.2	0	7.2	0	0	3995500
7.6	10.3	8.7	8.7	9.3	10.3	10.3	11.6	6412700
3.2	0	3.2	3.2	3.2	0	5.7	3.2	616150
0	0	0	3.8	0	3.8	3.8	6.7	514910
12.1	12.1	12.1	12.1	12.1	12.1	20.8	12.1	1073300
24.4	24.4	19.7	18.8	11.7	16.4	22	22	24916000
4.2	4.2	4.2	4.2	0	1.6	1.6	1.6	5818900
26.8	27.4	26.8	26.8	26.8	29.3	29	27.4	36828000
17.8	17.8	17.8	17.8	17.8	17.8	17.8	0	1417600
7.6	0	7.6	7.6	7.6	7.6	7.6	7.6	1502000
6.8	8.2	11	9.3	9.3	11	15.2	13.5	7717800
2.3	2.3	2.3	2.3	2.3	2.3	0	2.3	329170
0	0	0	0	0	0	23.9	23.9	699390
11.3	11.3	11.3	11.3	11.3	11.3	11.3	9.9	2117200
0	9.8	5	0	6	14.9	14.9	8.9	1674200
0	0	5.9	9.9	9.9	5.9	5.9	9.9	1146900
0	0	10.2	0	0	10.2	10.2	10.2	4056700
7.6	7.6	3.9	0	7.6	7.6	7.6	7.6	1665400
10.3	12.4	8.6	7.6	8.6	12.4	12.4	12.4	7118700
2.6	2.6	1.2	3.5	3.7	1.2	1.2	3.5	1141700
0	2.5	0	0	0	2.5	2.5	2.5	588420
0	0	0	0	1.3	1.3	1.3	1.3	315890
0	0	2.6	3.3	2.4	7.6	6.1	6.6	3838000
8.1	11.4	10.8	10.8	11.4	14.1	11.4	14.1	2771000
14.1	9.5	14.1	9.5	14.1	17.2	22.4	22.4	4672300
0	0	0	0	18.4	18.4	18.4	18.4	736250
36.7	12.2	36.7	36.7	36.7	36.7	49	49	5529400
11.6	11.6	11.6	0	11.6	0	11.6	0	427690
16.1	24.9	24.9	32.6	21.2	41.5	41.5	46.6	6632200
9	4.5	9	9	9	9	9	9	2724600
10.3	10.3	10.3	10.3	10.3	10.3	10.3	0	495570
4.8	3.7	3.7	4.8	4.8	4.8	3.7	3.7	2608400

0	0	16.7	0	16.7	16.7	16.7	16.7	2711900
4.9	4.9	4.9	4.9	4.9	4.9	4.9	4.9	647790
21.4	21.4	21.4	21.4	21.4	0	0	0	1107300
37.9	37.9	37.9	13.6	13.6	13.6	37.9	37.9	6014400
40.4	41.9	30	25.4	27.3	31	35.6	43.5	45891000
32	30.2	29.6	29.8	22.3	31.3	36.6	34.2	28037000
36.3	7.7	16.5	16.5	16.5	16.5	36.3	27.5	5221400
25.2	25.2	25.2	25.2	13.5	25.2	25.2	13.5	5173300
27	27	27	27	27	27	27	27	3841600
13.1	20.4	17.5	20.4	10.9	8	14.2	12.8	8390700
24.8	24.8	13.9	13.9	13.9	13.9	24.8	24.8	964520
26.4	26.4	26.4	17.9	26.4	26.4	17.9	26.4	6328500
4.4	4.4	4.4	4.4	4.4	0	0	0	654900
16.3	16.3	16.3	16.3	16.3	16.3	16.3	16.3	986870
14.5	14.5	14.5	14.5	14.5	11.5	11.5	14.5	18919000
26	20.1	26	26	13.6	26	26	26	28766000
15.7	15.7	15.7	15.7	9.8	15.7	15.7	15.7	11459000
25.6	25.6	24.8	25.6	25.6	24.8	24.8	18.6	17041000
55.8	47.7	43	29.1	20.9	43	41.9	41.9	5748800
28	28	10.7	10.7	10.7	26.7	28	17.3	3105900
25.3	25.3	25.3	25.3	11	25.3	25.3	25.3	9296700
36.8	28.9	28.9	36.8	7.9	28.9	28.9	28.9	2633000
32.1	32.1	32.1	32.1	32.1	32.1	32.1	32.1	13858000
27.2	27.2	27.2	27.2	27.2	27.2	27.2	27.2	5924900
12.3	12.3	4.9	4.9	0	4.9	4.9	12.3	1861100
26.5	26.5	26.5	22.4	22.4	13.4	13.4	13.4	3412600
7.9	7.5	6.2	5.6	4	8.1	10.7	10.6	29346000
0	0	0	0	0	5.2	5.9	5.2	624430
19.8	16.8	16.8	9.6	26.9	26.9	26.9	26.9	1739300
1.8	1.4	0.8	1.8	1	3.2	3.2	4.1	1353000
37.9	38	38	38	38.4	36.2	30.6	32.7	71594000
2.5	2.5	2.5	2.5	2.5	4.9	2.5	4.9	615140
0	6.5	0	0	6.5	0	6.1	12.6	695130
8	4.4	12.4	8	12.4	12.4	14.9	12.8	3715500
0	0	0	0	0	11.6	11.6	31.5	410360
22.8	22.8	15.6	18.4	4.4	20	21.9	20.5	15230000
9.5	9.5	9.5	4.6	4.6	9.5	14.4	14.4	2628000
51.4	51.4	51.4	51.4	51.4	51.4	32.9	51.4	1341000
0	0	0	7.6	0	7.6	15.3	15.3	1531200
2.3	2.3	4	4	4	2.3	2.3	2.3	1318000
11	35.6	26.3	26.3	0	35.6	35.6	35.6	6270700
4.4	4.4	10.7	10.7	10.7	10.7	10.7	10.7	1775300
14.2	14.2	6.6	6.6	6.6	6.6	7.6	10.4	3716800
10.5	10.5	10.5	10.5	5.9	10.5	8.7	8.7	14642000
17.4	14.6	6.4	9.1	6.1	17.4	9.1	17.4	5807800
6.1	3.1	3.1	3.1	6.1	6.1	3.1	3.1	911450
17.4	17.4	19.4	17	12.7	19	14.1	16.4	22017000
0	0	0	0	0	8.6	0	8.6	533610
2.5	6.8	6.8	6.8	4.3	6.8	4.7	6.8	1378600
0	0	0	0	0	0	0	5.2	0

12.1	0	22	22	12.1	22	36.2	43.3	5738400
7.3	7.3	7.3	7.3	0	7.3	7.3	7.3	1729500
19	0	19	0	0	19	19	19	1041000
0	0	0	1.2	0	1.2	1.2	0	307900
3.7	7.2	5.6	5.4	5.4	5.2	3.5	3.8	2324500
15.6	18.7	18.7	17.5	19.5	15	15.7	19.5	25715000
17	15.5	16.3	16.3	15	12.5	6.2	10.9	13349000
16.2	17.1	16.1	16.1	14.8	13.3	15.2	13.8	44207000
18.2	21.8	21.8	21.8	13.9	32.1	42.4	40	10765000
3.3	3	6.3	6.3	6.3	6.3	6.3	6.3	1327700
12.2	6.7	6.9	12.2	10.2	6.1	8.8	8.1	12140000
7.9	7.9	7.9	7.9	8.3	7.9	5.7	7.9	5972000
17.7	23.8	23.8	23.8	21.9	23.8	23.8	10.2	20760000
0	0	0	0	0	3.3	3.3	3.3	615660
1.1	1.1	3.1	3.1	3.1	3.1	3.1	1.1	1572500
8.3	8.3	8.3	8.3	11.9	8.3	8.3	6.4	4538500
13.9	13.9	13.9	13.9	13.9	13.9	13.9	13.9	643800
8	7.2	5.5	5.7	4.1	8.7	10.4	11.4	5157600
8.3	0	0	0	0	0	8.3	8.3	323770
9.8	9.8	3.3	3.3	7.6	3.3	13.9	13.9	4760300
5.1	8.7	4.2	0	2.3	8.1	8.7	10.9	3923100
3.5	3.5	2.4	0	0	3.5	3.5	3.5	1050200
3.2	3.2	2.2	0.9	1	2.3	2	2	1467700
0	0	9.9	0	0	9.9	0	0	123440
7.5	4.4	1.6	1.6	3.1	12.8	18.4	16.5	6815900
10.1	8.5	4.5	6.1	2.6	11.1	8.8	11.1	6293600
12.9	17.4	8.3	5.3	5.3	16.1	18.9	16.9	11877000
7.8	7.8	7.8	5.8	4.4	8.8	10.4	9.3	5022900
20.7	25.5	15.4	7.8	8.5	23.1	24.1	23.6	16031000
13.3	19	18.8	18.8	15.2	17.4	17.9	14.1	23540000
8.6	8.9	10.2	10.5	10.4	10.1	11.2	9.5	15351000
0	12.4	4.5	12.4	12.4	21.5	29.3	29.3	5440400
11.4	11.9	9.7	9.8	8.6	14.1	14.7	15.1	57332000
1.6	1.6	2.3	2.3	2.3	1.6	1.6	0	1017800
0	7.5	7.5	7.5	7.5	4.7	4.7	4.7	1829500
0	0	0	0	0	0	0	3.2	0
6.9	6.9	6.9	6.9	6.9	6.9	6.9	6.9	1013700
3.7	3.7	3.7	2.2	3.7	2.2	3.7	2.2	1257300
0	0	4.4	4.4	8.2	4.4	3.8	4.4	719370
0	0	9.2	9.2	0	0	9.2	0	336530
3.9	11.5	11.5	3.9	3.9	0	10.9	10.9	2462100
17	14.6	14.6	14.6	17	7	4.4	3.2	3446100
6.2	8.8	6.2	8.8	8.8	6.2	5.3	2.6	2972600
2.9	10.6	7.6	7.6	7.6	13.5	10.6	7.6	2642900
18.4	14	18.4	18.4	18.4	18.4	15.4	18.4	10000000
6.4	0	6.4	6.4	6.4	0	0	0	331160
4.5	4.1	4.1	4.1	6.1	4.1	0	2.6	1109200
0	0	0	0	0	17.7	0	17.7	155100
2.2	11.5	11.5	11.5	11.5	7.3	0	2.2	1253000
17.2	17.2	17.2	17.2	17.2	17.2	17.2	17.2	1788600

5.5	4.1	7.5	5.5	5.5	5.5	7.5	7.5	6747300
0	0	0	0	0	0	0	4.2	284720
13.2	13.2	13.2	13.2	13.2	9.4	9.4	13.2	4235600
23.9	13	23.9	23.9	23.9	23.9	23.9	23.9	4789100
17.8	17.8	11.8	11.8	9.3	15.3	15.3	15.3	17929000
36.2	39.2	34.9	34.3	32.1	28.7	40.4	33.6	80028000
27.4	27.4	27.4	21.7	17.9	23.8	26.1	27.9	42254000
47.1	37.6	47.1	47.1	47.1	47.1	37.6	37.6	15837000
5.8	7.1	5.8	5.8	5.8	5.8	3.3	5.8	10674000
2.7	2.7	1.6	2.7	1.6	1.1	0	0	1506500
4.1	0	0	4.1	0	4.1	0	4.1	518550
2.8	6.1	6.1	6.1	2.8	6.1	6.1	6.1	1470000
1.9	2.1	1.3	1.3	0.8	2.1	1.3	1.5	1129200
4.7	4.7	4.7	4.7	4.7	4.7	0	0	889890
11.1	11.1	11.1	11.1	11.1	7.5	6.7	3.1	2501100
0	0	0	0	0	3.3	3.3	3.3	377100
0	0	0	0	0	0	0	8.8	175710
4.4	12.3	4.4	4.4	8.8	8.8	8.8	4.4	735180
2.9	1	2.9	2.9	0	2.9	2.9	1	1190400
0	0	1.7	1.7	1.7	3.1	5.6	5.6	1191300
4.4	4.4	2.6	0	1.8	3.3	3.3	3.3	1367500
0.3	0.3	0.3	0.3	0	0.3	0	0	697580
37.1	37.1	24.7	24.7	24.7	37.1	37.1	49.4	6413900
37.1	37.1	24.7	24.7	24.7	37.1	37.1	49.4	3209800
0	0	0	0	12.5	16.7	38.5	29.2	1562000
0	0	0	0	0	0	0	1.5	138380
4.5	4.5	4.5	4.5	4.5	4.5	3.2	0	3571300
10.5	11.1	10.9	10.4	9.8	9.1	8.5	8.7	20108000
4.6	4.6	4.6	4.6	4.6	6.5	14.1	14.1	3327500
6.5	6.5	6.5	6.5	6.5	6.5	6.5	6.5	1342300
0.3	0.5	0.8	0.6	1.2	1.5	1.4	0.8	3184300
6.3	12.8	12.8	12.8	8.5	8.5	8.5	10.6	7846200
8.1	8.1	8.1	8.1	8.1	8.1	0	8.1	499180
10.2	10.2	5.1	5.1	5.1	10.2	10.2	10.2	1008100
4.3	4.3	1.5	1.5	1.5	1.5	3.5	2.9	1421800
12.5	26.7	29.6	29.6	15	33.8	33.8	33.8	6038700
8.7	4.9	8.7	8.7	8.7	8.7	8.7	8.7	2581300
0	4.9	8.5	8.5	4.9	8.5	3.6	8.5	1368900
11.8	6.8	9.8	5	6.8	11.8	11.8	13.3	5258200
21.5	9.2	17.5	14.7	9.2	21.5	21.8	25.8	10216000
22.8	22.8	22.8	28.4	22.8	18.7	14.9	19.4	16633000
32.5	37.6	41.6	37.6	41.6	36.5	36.1	41.6	23151000
15.6	15.6	15.6	14	11.4	20.7	20.7	20.7	616200
38.7	30.7	38.7	24.4	24.4	38.7	38.7	38.7	35034000
32.2	32.2	22.2	37.2	27.6	31.8	37.2	46.4	37559000
23.3	23.3	13.2	36.6	26.8	31.1	36.6	45.9	605880
20.1	25.9	23.8	23.6	18.5	27.7	32.5	29.5	81190000
37.3	35.3	27.6	26.9	25.2	39.4	38.7	40.8	1.87E+08
10.7	8.6	7.4	5.9	4.4	5.4	7.1	5.4	4225000
17.5	9.2	9.2	17.5	12.3	12.3	17.5	12.3	6269100

38.9	42.5	35.4	37.6	29.9	36.9	46	38.5	88720000
5	5	5	5	5	5	5	5	2883600
5	5	5	5	5	5	5	5	1746300
2.2	2.2	2.2	2.2	2.2	2.2	2.2	2.2	902720
22.8	22.8	14.6	17.7	13.8	22.8	17.7	19.7	8540000
9.8	9.8	9.8	9.8	9.8	7.2	7.2	7.2	3728600
0	0	0	0	10.2	10.2	0	10.2	381190
7.5	6.5	9.5	9.5	5.7	9.5	6.2	6.2	5952700
5.1	5.1	5.1	5.1	3.9	3.4	3.4	3.4	2671700
27.6	26.8	27.6	27.6	27.6	27.6	31.4	26.8	28994000
13.8	13.8	13.8	16.7	13.8	16.7	6.7	10.1	23185000
15.3	15.3	15.3	6.7	15.3	4.3	12.9	12.9	2554800
36.7	34.6	33.9	27.9	28.6	32.5	34.9	34.5	1.18E+08
0	0	0	0	0	12.9	12.9	12.9	461620
10.6	37.5	37.5	37.5	26.9	37.5	37.5	21.2	19497000
15.1	18.6	18.6	18.6	12.3	15.1	14	16.1	18472000
16.2	16.2	16.2	12.1	7.4	7.4	9.9	11.5	3879900
28.6	34.5	28.6	28.6	23.8	28.6	34.5	21.4	18155000
5.2	5.2	5.2	5.2	5.2	0	0	0	592250
29.3	27.9	29.6	27.4	22.2	26.4	32.1	26.5	89194000
21.3	40.2	40.2	21.3	21.3	32	32	40.2	5468100
24	22.5	22.5	22.5	20.2	26.3	22.5	22.5	37699000
5.2	3	0	3	3	3	3	3	1069200
12.5	12.5	12.5	8.6	10.3	12.5	10.3	15.6	6704800
2.2	0	2.2	2.2	2.2	2.2	2.2	2.2	1439400
8.6	8.6	5	8.6	0	5	5	8.6	7661300
3.1	0	1.8	0	0.7	1.8	0	1.3	878430
8.4	8.4	11.8	8.4	7.4	11.8	11.8	11.8	3767900
0	0	17.9	0	17.9	0	17.9	17.9	280650
8.1	3.8	3.8	3.8	0	8.1	4.3	3.8	1032500
15.9	15.9	12.4	15.9	15.9	15.9	12.4	10.4	2729100
17.2	21.6	18	21.6	17.2	21.6	13.6	7.1	9444000
17.7	29.1	28.2	28.2	17.5	28.2	29.1	29.1	29609000
25.5	25.5	25.5	0	25.5	18.2	18.2	18.2	2629000
0	1.5	1.5	1.5	0	0	0	1.5	287790
5.7	5.7	5.7	5.7	5.7	5.7	0	0	353760
29.8	53.4	32.8	28.8	19.7	46.3	51.5	53.7	58839000
11.2	11.2	9.5	13.4	10.5	11.7	16.5	16.8	5977500
7.4	11.5	8.8	4.9	3	10.1	13.2	12.9	4320400
0	0	3.1	5.2	0	10.6	13.7	14.4	3390200
14	14	7.3	7.3	7.3	14	14	0	914250
0	0	0	0	0	33.1	19.8	33.1	1220900
0	0	6.9	13.9	6.9	21.5	21.5	14.6	2088300
7	6.3	14.9	11.7	9.2	19.7	19.7	9.5	5017500
9.9	19.5	15.9	15.9	15.9	23.1	26.7	26.7	9719100
9.1	10.8	10.6	10.6	9.1	16.9	16.9	17.8	7975900
0	0	4.2	0	4.2	11.4	11.4	11.4	667480
2	2.8	2	3.3	3.8	8.5	8.2	8.2	6678400
6.3	5.7	3.7	2.6	1.5	10.1	11.2	11.2	6074900
6.1	2.2	4.3	4.3	3.2	7.5	7.5	8.2	5000900

16.2	15	13.4	8.4	11	11.2	22.8	20.2	7257300
17.5	17.5	17.5	13	9.2	19.6	19.6	17.5	7806300
5.3	5.3	5.3	5.3	5.3	8.4	8.4	8.4	2561700
8.4	0	0	3.4	3.4	5	14.5	11.5	1632200
7.7	10.3	7.7	5.4	0	7.7	13.2	4.9	2143800
20.3	20.6	18.2	15.4	18.8	22.5	32.9	33.8	7044000
0	11.3	18.9	18.9	0	18.9	18.9	18.9	1087200
0	10.2	10.2	10.2	10.2	10.2	10.2	10.2	1180400
4.9	3.3	9.9	9.9	7.6	13.7	15	13.3	4448100
0	0	13.5	0	0	13.5	13.5	0	419870
9.7	8.9	6.2	8.6	10.8	14.1	14.1	12.9	16044000
3.4	0	3.4	0	0	5.4	2	5.4	557030
0	0	4.9	7	4.7	9.6	7	6.8	4235800
6.1	19.3	19.3	22.8	19.3	28.5	34.6	34.6	9787800
2.3	0	0	2.3	0	6.5	6.5	6.5	1294800
29.5	29.5	24	24	29.5	29.5	32.2	32.2	28823000
3.8	5.1	2.9	1.7	1.7	4.6	7.1	5.6	3590800
22.9	29	11.4	11.4	11.4	25.7	33.1	33.1	8737200
11.5	11.5	11.5	11.5	11.5	11.5	11.5	11.5	598050
20.2	20.2	5.9	20.2	9.2	20.2	20.2	23.5	1841500
0	0	0	5.7	0	5.7	5.7	5.7	310470
7.6	12.4	7.6	7.6	7.6	7.6	7.6	12.4	1661900
7.9	5.2	0	0	2.7	7.9	7.9	7.9	931280
1.8	0.8	3.2	0.8	5.3	6.9	6.4	5.6	3778500
7.1	5.4	7.8	9	8	13	11.7	13	30012000
2.2	2.2	1.2	1.2	1.2	2.2	1.2	2.2	764030
10.5	13.1	11.7	11.2	7.5	11.5	10.4	10.7	12636000
10.6	10.4	15.4	12.3	14.7	16.9	20.1	22.4	10743000
6.8	7.8	6.7	6.7	6.1	9.7	9.9	12.8	12129000
17.9	14.5	16.4	14.2	12	15.7	15.7	15.7	14643000
33.9	39.5	39.5	32.5	29	36	30.4	33.9	13924000
15	15	15	15	16.4	11.5	24.5	32.9	3826900
22.1	23.9	28.3	28.3	18.4	33.1	33.1	30.1	13298000
0	1.2	7.4	8.9	4	17.9	22.4	19.9	7495500
12.4	11.5	9.8	12.8	7.2	25.9	25.5	27.1	13470000
17.5	20.1	16.6	9.3	4.2	18.7	23.3	24.1	7519500
5.1	5.1	5.1	7.9	5.1	7.9	11.3	11.3	3223400
22.2	20.2	22.2	14.2	20.2	18.2	12.1	14.2	8044200
32.3	32	28.4	31.2	29.6	35.1	44.4	41	51955000
20.4	18.5	16.5	14.3	12.4	21.6	22.3	22.2	65658000
0	0	22.2	22.2	37	32.6	47.4	47.4	4983000
11.3	11.3	11.3	11.3	7.5	7.5	7.5	7.5	1618000
12	15	18.6	11.5	14.8	11.5	12	18.6	15913000
8.3	5.2	6.7	8.3	5.2	5.2	8.8	8.3	1928800
35.5	31.7	35.5	35.5	35.5	35.5	27.3	27.3	17299000
0	0	0	0	0.5	0	0	0	30630
40.1	39.4	37.5	39	37.2	41.6	44.6	44	3E+08
19	19.1	20.4	21.7	21.8	23.8	26.4	23.9	56583000
5.8	0	5.8	0	5.8	5.8	12.3	12.3	680540
18.4	21.6	20.8	10	12.4	26.8	26.8	22.6	11290000

0	9.2	16	15	4.9	29.1	29.1	32.5	2927500
15.3	15.3	15.3	15.3	12.4	15.3	15.3	15.3	5651100
13.6	15.4	15.4	15	10.5	12.4	12.1	15.4	4838100
6.2	0	6.2	6.2	6.2	2.3	6.2	6.2	1279700
8.6	8.6	8.6	5.6	9.5	8.6	9.5	8.6	5888400
22.2	22.2	22.2	22.2	22.2	22.2	11.1	22.2	2034500
2.7	2.7	2.7	2.7	2.7	2.7	0	0	386780
48.9	44.5	52.7	49.2	48.1	57.1	63.2	62.9	1.89E+08
16.5	14	18.7	11	16.8	18.7	19	18.7	12757000
25.9	27.6	22.3	22.3	16.1	22.9	24.4	24.6	24252000
16.4	16.4	16.4	16.4	16.4	16.4	16.4	16.4	5553300
15.2	15.2	15.2	15.2	18.6	15.5	15.5	15.5	3159600
58.5	71.9	71.9	64.4	54.1	71.9	58.5	71.9	81018000
0	4.4	0	0	0	9.6	9.6	9.6	699140
8.5	6.2	8.5	4.4	8.5	8.5	6.2	8.5	3772700
18.8	18.8	18.8	18.8	18.8	18.8	18.8	12.8	1756600
0	10.6	21.3	33	21.3	33	33	22.3	2501500
13.4	13.7	15.3	13.8	12	13.5	12	12.5	13719000
7.5	7.5	7.5	7.5	7.5	4.9	4.9	4.9	1594500
20.1	21.3	17	10.9	10	9.7	9.7	8.1	15006000
1.2	1.2	0	0	0	0	1.2	1.2	503820
10.9	10.8	5.9	7.6	7.6	8.8	8	10.1	6706600
0	0	1.9	2.9	1.9	2.9	2.9	1.9	538860
0	0	0	0	0	0	15.3	15.3	233400
0	0	2.7	2.7	2.7	2.7	2.7	4.2	424310
0	0	0	0	0	9.7	9.7	9.7	180450
0	5.1	5.1	8.4	8.4	20.7	10.5	24.9	2336500
19.4	20.6	13.6	12.8	8.9	26.6	30.9	32.8	17051000
8.6	8.6	10.1	10.1	5.4	18.3	20.2	14.1	22418000
37.9	37.6	37.6	36.4	31.2	35.3	28.2	33.5	78085000
37.3	37.3	31.9	34.1	34.4	36	30.3	30.3	66980000
0	0	0	0	1.8	1.8	1.8	3.3	361540
0	0	0	0	0	5.2	5.2	5.2	378860
0	0	0	0	0	3.6	8.7	2	600190
20.9	22.6	18.1	18.1	12.7	20.1	20.1	22.6	10111000
9	9	7.3	9	7	9	6.9	9	3293300
9.3	8.8	7.5	5.6	5.4	10.7	8.8	7.3	3372500
0	0	4.2	4.2	4.2	7.8	7.8	7.8	1518400
0	8.9	8.9	8.9	8.9	8.9	8.9	8.9	1810600
4.9	6.4	11.3	10.2	4.9	10.2	14.1	14.1	2625200
8.4	8.4	8.4	8.4	20	12.6	8.4	8.4	3907600
0	5.8	12.9	9.5	9.5	20.7	19.9	20.3	2804100
21.4	26.7	36.2	36.2	21.9	49	52.9	50.5	68356000
52.9	51.5	52.9	46.8	44.4	44	53.2	56	4.34E+08
2.8	2.8	2.8	2.8	0	2.8	2.8	0	460300
12.5	14.4	13.1	13.1	13.1	11.7	11.7	11.4	12011000
30	10	30	30	30	30	30	10	4253000
5	2.8	3.7	7.6	3.7	5.4	3.9	7	3550300
5.7	5.7	5.7	5.7	5.7	5.7	5.7	0	897140
0	0	0	7.3	0	7.3	7.3	7.3	301560

0	0	6.6	4.6	4.6	6.6	6.6	6.6	1707900
0	6.7	6.7	6.7	6.7	6.7	6.7	6.7	1257000
12.5	16.5	18.3	15.1	10.8	10.6	10.8	13.1	5597400
7.6	7.6	5.6	7.7	5.9	11.1	12.5	14.1	13680000
6.7	6.7	5.2	4.7	6	5.2	6.7	2	4995500
4.9	3.3	1.9	1.9	3.3	1.9	1.9	1.9	1132200
5	5	5	5	5	10.4	5.4	5	1295400
9.6	7.3	12.8	14.8	2.1	12.8	12.8	7.3	3503700
2.5	2.5	2.5	0	2.5	2.5	2.5	2.5	506210
16	16	16	16	16	16	16	0	1159500
5.3	6.9	8.7	6.9	6.9	1.9	1.9	5.3	1755300
11.2	11.2	11.2	11.2	11.2	11.2	11.2	11.2	1512200
0	0	0	7.3	0	0	0	0	32450
5.6	3.3	0	3.3	5.6	0	3.3	0	1483300
7.2	7.2	7.2	7.2	4.8	0	0	0	1148000
3.2	3.2	0	0	0	0	0	3.2	269260
24.9	28.6	17.1	17.1	17.1	17.1	28.6	12.9	4717800
9.6	9.6	9.6	9.6	9.6	0	9.6	9.6	1002400
12.2	6.4	12.2	12.2	12.2	12.2	12.2	12.2	1906200
22.7	18.5	27.5	25.8	25.8	27.5	23.2	15.9	43544000
20.3	20.3	20.3	0	20.3	20.3	20.3	0	832880
10.4	10.4	10.4	10.4	0	10.4	10.4	0	1265200
2.3	4.3	2.3	2.3	4.3	4.3	4.3	4.3	1044300
19.5	22.3	19.5	12.1	11	27.1	22.3	21.2	2336300
23.4	23.4	23.4	19.7	19.4	28.2	28.2	17.5	6869500
69.4	69.4	59.7	69.4	59.7	69.4	59.7	25	6585900
23.2	19.1	19.1	23.2	23.2	18.8	22.1	23.2	6773300
27.1	26.8	27.1	27.1	27.1	25	30.3	23.2	33720000
21.8	21.8	21.8	15.8	19.5	24.3	22.3	20.6	13363000
7.2	7.2	7.2	7.2	7.2	7.2	7.2	7.2	1502400
11.3	11.3	11.3	11.3	11.3	9	11.3	9	3797900
7	7	7	7	7	7	3.1	7	636200
4.3	0	0	4.3	0	0	0	0	98521
46.4	57.7	50.5	33.8	28.4	50.5	68.1	64.4	62096000
1.9	6.3	6.3	6.3	6.3	1.9	6.3	6.3	2040400
0	0	0	1.2	1.2	0	0	0	133450
14.6	14.6	14.6	14.6	10.1	14.6	14.6	14.6	4636600
24.8	24.8	24.8	23.5	31.4	24.8	24.8	23.5	7973700
8	13.4	10.9	10.5	8.9	13.4	5.8	10.5	5631000
5.3	5.9	5.9	4.3	3.1	4.3	2.1	4.1	5856200
2.2	2.2	2.2	2.2	2.2	2.2	0	2.2	5093200
34.4	41	36	31.2	27.4	38.9	52.5	47.2	2.97E+08
10.7	16.5	19.9	23.2	12.7	30.7	25.6	26.4	23160000
15.1	15.1	15.1	15.1	13.7	15.1	15.4	15.4	1.01E+08
44.1	40.6	37	42.1	34.8	46.7	48.3	43.7	2.51E+08
32.9	32.9	32.9	32.9	32.9	32.9	32.9	32.9	2207000
15.1	22.5	22.5	20.4	19	27.1	29.5	34	43785000
21.8	20.3	19	17.7	14.4	21	20.1	16.3	28664000
62.9	72.2	72.2	72.2	59	72.2	72.2	72.2	2.25E+08
31.1	33.7	35.9	33.7	27.9	35.9	45.2	43.7	1.08E+08

44.8	40.1	49.3	45.6	37.3	49.2	50.6	52.2	5.02E+08
13.8	14.2	14.2	15.8	14.4	15.8	15	15	386650
0	0	37.5	37.5	31.7	54.8	54.8	54.8	3696400
0	0	0	0	14.8	27	27	14.8	644860
15.5	15.5	5.1	8.9	5.1	8.9	5.1	11.7	2264000
0	0	12.9	12.9	12.9	12.9	12.9	12.9	474800
48.6	48.6	21.8	21.8	21.8	21.8	32.4	38	25171000
6.1	6.1	6.1	27.9	0	6.1	6.1	6.1	560130
7.7	7.7	4.4	4.4	4.4	7.7	7.7	4.4	2299900
4.2	4.2	4.2	4.2	4.2	4.2	4.2	4.2	1173300
0	0	11.2	19.6	14.6	30	30	34.2	5864600
14.6	8.7	18.2	13.8	22.1	18.2	18.2	18.2	4968000
2.5	7.1	13.9	12.1	9.6	16.4	13.9	16.4	11568000
23.6	16.7	16.7	11.5	6.6	16.7	23.6	23.6	15372000
6.8	12.7	14	16.9	13.7	21.5	40.7	30.3	36423000
20.2	11.2	9.3	15.4	13.5	21.9	28.1	17.7	21056000
15.7	6.7	11.4	20.5	11.4	13.8	30	38.6	20981000
4.1	8.5	4.4	4.4	4.4	8.5	10.7	8.5	2381600
33	33	28.4	28	28.2	30.1	37.3	30.6	40228000
33.6	33.6	29.1	29.1	29.1	29.1	31.9	33.6	47193000
23.6	23.6	23.6	23.6	23.6	23.6	19.6	27.8	7279800
29	29	17.5	18.7	12.4	22.7	29	13.6	13528000
31.1	36.4	23.9	20.7	28.2	35.5	43.2	41.1	75005000
27.2	26.1	13.4	11.2	23.4	22.8	35.7	33.4	34482000
36.5	36.2	33	29.4	17.2	36	40.1	40.5	51675000
19.9	23	18	17.3	19.7	33.6	33.2	22.2	29890000
24.2	25.6	17.4	21.2	17.2	21.5	25.6	25.6	18494000
25.3	28.7	21.8	18.3	19.3	24.6	29.6	28.6	1.25E+08
4.1	4.1	4.1	5.6	5.6	5.6	4.1	5.6	1705600
21.2	19.7	18.3	17.8	15.4	18.5	17.4	19.1	13837000
27.5	26.1	32	38.2	31.2	48.2	56.4	36.3	1.42E+08
42	44.7	46.6	37	31.3	41.2	42	40.8	46662000
10.5	10.9	10	11.6	5.5	7.1	5.8	9.2	4003400
16.9	19.4	19.4	15.7	10.1	18.2	14	14.8	19925000
2.9	2.9	2.9	2.9	2.9	1.6	1.6	2.9	2317400
29.7	29.7	29.7	11.4	0	29.7	37.3	37.3	12959000
0	10.4	0	10.4	0	0	19.4	9	400430
0	0	0	0	0	8.5	8.5	8.5	584220
15	22.4	23.4	23.4	23.4	23.4	23.4	15.9	9871300
0	0	23.2	0	23.2	0	0	23.2	246320
21.4	12.7	5.6	12.7	12.7	19.8	35.7	35.7	3720900
5.5	5.5	5.5	5.5	10.5	5.5	0	0	574300
0	0	3.9	0	0	3.9	3.9	3.9	442100
5.2	9.4	9.4	9.4	5.2	9.4	11.4	6.8	5028100
9.8	17	17	17	11.9	16.5	12.4	16.5	15140000
20.2	31.9	31.9	31.9	31.9	27.7	27.7	31.9	2.78E+08
26.9	26.9	26.9	26.9	26.9	31.1	26.9	31.1	10799000
25.7	26.1	26.1	26.1	25.7	26.1	21.7	26.1	89649000
5.6	5.6	5.6	5.6	5.6	5.6	10.3	10.3	1600500
27.2	27.2	27.2	27.2	27.2	27.2	27.2	27.2	7.86E+08

31.2	31.2	31.2	31.2	31.2	31.2	31.2	31.2	26236000
3.7	3.7	3.7	3.7	14.5	14.5	0	14.5	380270
21.7	21.7	21.7	21.7	21.7	21.7	16.8	21.7	10265000
53.2	47.6	53.2	46.8	35.7	53.2	46.8	53.2	13227000
46.8	52.4	52.4	46	40.5	52.4	40.5	52.4	9275500
52.4	41.3	52.4	40.5	40.5	52.4	40.5	46.8	2589000
36.1	40.4	40.4	35.5	31.3	40.4	31.3	40.4	9.76E+08
26.5	19.9	26.5	26.5	19.9	19.9	18.4	18.4	1.86E+08
52.4	52.4	52.4	52.4	52.4	51.5	44.7	52.4	1.07E+09
12.4	8.5	14.1	15.4	12.2	17.3	17.3	20.2	12731000
12.2	12.2	6.6	4.9	5.6	10.6	17.9	14.1	2477000
43	33.7	35.9	35.9	35.1	39.2	39.2	35.9	8157100
43	33.7	35.9	35.9	39.7	39.2	39.2	40.5	64100000
6.8	6.8	6.8	6.8	3.8	6.8	6.8	6.8	1862800
25.7	25.7	22.4	22.4	22.4	25.7	25.7	23.2	9447800
30.3	26.5	23.2	23.2	27	26.5	30.3	30.3	18412000
23.1	22.6	19.4	19.4	23.1	22.6	26.3	26.3	2130800
15.7	15.7	19	19	13.3	20.9	20.9	20.9	11314000
12.7	12.7	12.7	16.4	12.7	26.7	18.8	14.3	8531000
8	8	8	8	8	8	8	8	1371600
14.8	14.8	14.8	12.3	11.9	10.7	10.7	7.9	8988700
4.3	8	8	8	0	8	3.7	4.3	1243600
9	4.8	9	16.2	9.2	13.5	15.8	18.5	9630700
20.6	20.6	20.6	20.6	20.6	20.6	14.5	12.5	11936000
5.5	0	10.1	28	16.1	28	28	28	4520200
12.5	12.5	13.6	10	8.8	11.3	11.3	11.3	9757000
29	29	29	29	0	29	29	29	3319900
18.1	20.2	16.6	16.3	15.5	21.7	22.5	20.8	13159000
19.4	13.1	15.7	13.1	13.1	13.1	22.1	16.5	6856300
1.9	1.9	4.6	1.9	1.9	1.9	4.6	4.6	994670
2.6	4.6	2.6	2.6	0	4.6	2.6	2.6	1416100
16.1	19.2	15.5	15.5	10.4	18.4	20	19.1	29775000
2.5	0	4.7	0.9	2.5	10.4	10.9	13.3	5132500
1.3	1.3	1.3	1.3	1.3	2.8	4.4	6.5	2451300
0.9	0	0	0	0.9	1.9	1.1	0	880950
1	2.2	2.2	2.2	2.2	2.2	2.2	1.3	591470
6.9	6.9	6.9	6.9	3.4	3.4	6.9	6.9	1086700
6.6	6.6	6.6	6.6	3.4	6.6	3.2	6.6	1876300
1.2	1.2	3.1	3.1	3.1	0	1.2	1.9	605310
0	1.3	4.5	4.5	3.2	4.5	1.3	0	640680
27.7	27.7	20.4	28	22.5	40.8	47.4	45	17097000
9.6	0	0	9.6	9.6	9.6	18.6	18.6	1093500
25.3	31.9	23.6	17.9	23.6	26	31.9	28.9	20208000
3.8	3.8	3.3	3.3	3.8	3.1	1.5	1.8	5882900
10	10	7.3	2.8	10	10	10	10	2687500
14.7	14.7	14.7	14.7	14.7	7.9	7.9	18.8	14775000
2.6	2.6	2.6	2.6	2.6	2.6	2.6	0	3359600
15.1	16.6	15.4	13	11.7	14	17.1	12.6	30330000
5	4.1	4.1	4.1	4.1	3.1	4.1	3.1	5402600
28.4	28.2	27.5	21.1	21.9	24	24.1	23.1	31883000

8.2	8.2	6.3	8.2	6.3	4.2	2.9	4.2	5862200
3.5	3.5	3.5	3.5	3.5	3.5	3.5	3.5	5117100
19.5	20.8	19.5	20.8	19.3	18.2	18.2	16.5	62654000
23.8	25.9	24.9	26.5	24.3	24.1	25.8	24.4	71654000
5.3	5.3	5.3	5.3	2.6	5.3	5.3	5.3	1300700
6.5	6.5	6.5	6.5	8.8	6.5	4.9	6.5	1989600
0	0	4.3	4.3	0	9.8	9.8	10.5	749410
0	0	21.6	21.6	0	21.6	21.6	21.6	1991200
8.9	10.3	10.3	5.1	6	10.3	8.7	10.3	3624600
13.1	23	23	19.9	13.4	26.4	23.6	32.7	14736000
9.7	15.4	17.2	16.6	17	22.1	29.2	27.6	26268000
0	0.9	0.9	0	0	0	0	0.9	496590
0	0	0	0	0	5.9	5.9	5.9	197260
21.8	21.8	19.3	21.8	22.4	12.4	19.9	19.3	12164000
8.7	8.7	16.5	10.7	8.7	16.5	8.7	16.5	5836800
18.4	18.4	16.9	22	13.8	22.7	34.8	29.2	11540000
8.8	9.3	6.2	5.2	4.6	7.4	9.8	9.8	8511600
7.5	8.7	8.7	8.7	7.2	8.7	7.6	7.6	7492100
25.8	25.8	25.8	25.8	25.8	25.8	25.8	25.8	1290300
28.9	32.6	31.9	32.3	27.8	30.9	35.4	35.3	55681000
33.4	24.4	27.4	35.8	35.8	27.4	30.1	27.4	18586000
9.4	9.4	9.4	9.4	9.4	9.4	9.4	9.4	2064400
6.4	8.9	7.4	7.3	7.8	12	8.8	6.9	1512000
60.3	60.3	60.3	49.3	49.1	47.8	46.1	49.8	3.06E+08
31.6	30.3	27.4	30.3	27.2	28.3	30.3	25	9767000
55.1	53.5	53	55.1	56.3	55.1	55.1	55.1	2.93E+08
73.8	78.8	77	75.8	76.2	75.5	78.8	54.8	7.39E+08
1.1	1.1	9.5	9.5	9.1	1.1	11.4	8.3	1457800
6.4	8.9	8.2	10.4	6.4	8.2	9	8.2	2944300
7.4	7.4	7.4	7.4	5.6	9	9.6	8.2	179150
42.5	45.1	42.5	40.7	38.1	40.5	44.2	43.9	1.82E+08
29.4	41.1	38.1	38.1	34.2	33.3	34.4	29.1	20034000
23	33	31.7	31.7	27.8	27	26.2	22.7	739340
61.8	59.7	61.8	61.4	61.6	63.1	61.4	64.4	1.59E+09
61.1	61.5	61.1	59.6	52	59.6	54	54.2	5.26E+08
5.7	5.7	2.4	2.4	0	9.1	9.1	9.1	4481200
0	0	0.8	0.8	0.9	3.5	1.8	4.2	1652400
7.2	8.3	4.2	3.9	7.2	7.2	8.3	8.3	3933100
1.1	1.1	1.1	1.1	1.1	1.1	0	0	401300
4.7	7.7	7.7	7.7	6.3	4.8	6.1	6.1	4242200
3.7	3.7	0	3.7	3.7	0	0	0	1017800
32.7	32.7	32.7	18.8	32.7	32.7	18.8	12.9	28881000
0	0	0	0	0	2.4	2.4	0	223630
5.9	5.9	16	0	5.9	16	16	16	2495700
12.2	16.9	16.9	16.9	16.9	16.9	18.1	12.6	7665800
19.9	19.9	19.9	15.4	15.4	20.7	16.7	16.7	3504000
32.8	32.8	32.8	32.8	32.8	35.7	32.8	33	28398000
3.1	7.5	7.5	4.4	7.5	6.2	7.5	3.1	5647000
44.9	50.2	49	47.8	52.4	44.2	47.8	46.1	1.03E+08
46	43.2	43.1	43.2	40.3	36.9	37.1	26.6	62583000

1.9	1.9	1.9	1.9	0	0	0	1.9	791950
1.1	1.4	0.5	0.5	0.5	0	0	0	509610
6.7	6.7	6.7	6.7	4.5	6	6	3.2	5097100
5.2	5.2	5.2	4.4	5.2	4.1	3.6	2.5	2585900
9.8	7.3	9.8	9.8	7.7	6.1	7.7	6.6	4050200
0	0	14.6	14.6	0	14.6	14.6	14.6	273480
7.1	14.1	9.4	7.1	7.1	11.8	14.1	11.2	7047800
5.4	5.4	5.4	5.4	5.4	3.6	0	3.6	1829900
9.9	9.9	9.9	9.5	9.9	9.5	9.5	9.9	55184000
0	0	0	0	0	2.5	1	3.7	631510
14.5	14.5	14.5	6	6	6	6	14.5	1731000
11.9	11.9	11.9	6.5	6.5	6.5	6.5	6.5	914190
11.1	11.1	11.1	7.4	9.2	9.2	6.9	9.2	4563200
23.3	23.7	24.2	22	17.6	23.2	23.2	23.5	67890000
4	2.1	2.1	4	3.3	8.4	6.8	5.2	1666000
1.1	1.1	1.1	1.1	1.1	1.1	1.1	1.1	1890600
3.1	3.1	3.1	3.1	6.7	5.1	0	8.7	1523600
28	28	28	28	15.6	28	29	32.2	31091000
5.3	3.8	2.6	3.8	3.8	5.3	5.3	6.1	2562400
1.2	3.1	4.4	3.1	3.1	1.9	0	0	1054600
2.9	0	7.9	7.9	5.5	13.2	13.2	13.2	3502700
9.6	9.6	9.6	9.6	9.6	9.6	6.8	9.6	19407000
2.1	2.8	7.9	10.6	6.5	12.6	12.6	10.6	4505600
15.7	16.9	19.5	21.8	14.6	24.5	24.5	38.3	12102000
10	6.7	15.9	19.9	17.9	21.4	25.6	18.9	13222000
4.2	4.2	4.2	4.2	6.7	2.3	0	1.8	1047900
21.1	28	22.3	19.6	19	42.2	42.2	42.2	1.38E+08
15.3	7.2	16.8	19.8	14.7	28.4	28.4	32.9	11547000
11	9.4	11	11	11	7.7	4.7	6.3	6018800
9.5	9.5	9.5	5.8	5.8	8.2	7	4.4	2092500
22.1	22.1	19.9	19.9	19.9	19.9	20.7	22.2	30014000
10.1	8.2	8.2	8.2	8.2	6	8.2	8.2	1818100
7.6	13.3	0	7.6	0	20.3	20.3	20.3	1158200
5.6	4.1	5.6	6.8	6.8	5.6	2.3	4.1	5955200
2.9	0	2.9	2.9	2.9	0	0	0	506270
3.7	6.5	3.7	3.7	3.7	6.5	8.8	5.1	1295900
0	0	2.7	2.7	0	5.4	10.8	7.6	3570300
0	20	20	20	20	20	20	20	385110
12.1	12.1	12.1	12.1	12.1	12.6	14.9	14.9	9720700
8.8	8.8	8.8	8.8	3.5	8.8	8.8	8.8	3085600
4.3	9.4	9.4	9.4	4.3	9.4	0	4.3	1391000
1.5	5.3	1.5	1.5	2.2	0	0	0	552570
2.5	2.5	2.5	2.5	2.5	2.5	2.5	0	1003000
10.3	13.3	10.3	10.3	13.3	10.3	13.3	10.3	7597800
9.4	9.4	9.4	9.4	9.4	9.4	0	0	612090
8.6	6.7	8.6	8.6	8.6	8.6	8.6	8.6	32971000
4.1	4.1	4.1	4.1	0	4.1	4.1	4.1	5772500
17.9	17.9	17.9	8.2	8.2	17.9	17.9	9.7	931300
17.4	7.8	17.4	17.4	17.4	17.4	17.4	17.4	24398000
3.9	3.9	7.5	14.1	11.1	14.1	14.1	14.1	3323800

9	9	9	9	6.3	5.2	6.3	6.3	2545000
8	6	4	4	9.8	4	0	2	1298400
0	2.5	2.5	2.5	0	2.5	0	2.5	188510
5.2	5.2	5.2	5.2	5.2	5.2	5.2	5.2	3292400
8	4.8	8	8	8	8	8	8	3093300
12.2	10.9	15	16	13.5	15	12.8	16	10358000
8.1	13.8	18.1	25.7	25.7	25.7	35.2	39	10738000
65.7	65.7	59.2	59.2	53.6	59.2	55.9	62.7	2.96E+08
19.2	19.2	14.7	19.2	14.7	15.1	15.1	4.5	3686600
0	0	0	0	0	11.2	11.2	11.2	625810
0	0	0	0	0	0	0	3.3	66671
19.2	19.2	19.2	19.2	38.5	19.2	19.2	19.2	1123800
6.7	6.7	6.6	3	6.6	6.2	1.6	5.7	3386600
0	0	0	0	0	6.9	6.9	0	233430
0	0	0	6.7	0	6.7	6.7	14.4	1264200
20.4	22	15.2	11.2	9.3	16.4	21.4	22	28523000
8.4	5.5	6.7	5	5	3.3	3.8	8.4	5580200
4.8	7.5	8.6	9.2	11	6.5	6.5	3.9	4538600
0	0	0	0	6.3	0	0	0	50231
3.4	6.5	6.5	3.4	3.4	6.5	3.4	3.4	1765900
0	1.4	3.1	1.4	3.5	0	0	0	308650
18.3	18.3	18.3	18.3	18.3	18.3	18.3	18.3	3028300
26.2	26.2	26.2	26.2	26.2	26.2	26.2	26.2	5918700
16.6	16.6	16.6	16.6	16.6	16.6	16.6	16.6	4531000
18.4	18.4	18.4	0	18.4	0	0	0	423540
22.2	26.5	26.5	26.5	16.8	26.5	26.5	17.3	4337000
5	2.6	2.6	2.6	5	5	5	5	1364400
19.7	19.7	19.7	19.7	19.7	19.7	32.8	19.7	4681900
5.5	4	7.8	7.8	3.9	6.3	3.9	4	4332400
15.5	12.3	12.3	15.5	15.5	12.3	12.3	7.9	2383100
13.1	13.1	13.1	13.1	13.1	13.1	13.1	8.3	2861800
0	0	6.6	6.6	0	6.6	6.6	6.6	388910
11.1	11.1	11.1	11.1	11.1	11.1	11.1	8.3	2770000
0	0	4.3	7	7	11.3	11.3	11.3	984070
5.3	6.8	5.3	5.3	4.1	6.9	8.6	9.7	5227700
0	5.8	5.8	0	5.8	5.8	5.8	5.8	376340
9.7	6.3	9.7	9.7	6.3	9.7	6.3	2.5	2747300
3	3	3	3	3	3	3	3	850890
0	5.4	5.4	5.4	5.4	0	5.4	5.4	702880
18.6	18.6	18.6	18.6	18.6	18.6	0	0	942180
7.8	11.3	7.8	11.3	0	11.3	11.3	11.3	1155100
11.3	24.2	24.2	24.2	24.2	24.2	0	11.3	1216900
25	21.6	18.5	18.1	14.7	16.8	21.6	11.2	7449000
17.1	17.1	17.1	17.1	17.1	17.1	0	17.1	579220
24.8	27.3	22.8	24.9	24.8	24.9	16.3	16.5	36673000
18.2	18.2	18.2	18.2	18.2	18.2	18.2	18.2	2371700
13.3	18.1	13.3	18.1	13.3	18.1	13.3	13.3	1735700
0	0	0	0	0	0.6	0.6	0	242270
0	6.3	10.1	10.4	23.1	27.2	37.3	45.1	6412600
0	3.2	3.2	3.2	3.2	5.6	9.3	9.3	1325600

23.7	23.7	23.7	23.7	23.7	7.6	23.7	0	947520
3	0	0	3	0	3	3	3	380710
10.7	10.7	10.7	10.7	10.7	13.4	13.4	13.4	23884000
2	2	2	2	2	2	2	2	631180
21.3	21.3	21.3	21.3	5.4	21.3	21.3	21.3	6206800
9.3	9.3	9.3	9.3	3.3	12.5	12.5	3.5	1873800
10.2	10.2	10.2	10.2	8.1	10.2	6.1	7.4	3483600
7.3	19.5	15.2	9.9	13.9	15.2	19.5	22.1	4097700
11.8	11.8	12.2	8.4	5.6	8.4	11.8	8.4	242070
8.6	16.4	8.6	8.6	8.6	8.6	16.4	16.4	2817900
4.8	7.3	7.3	7.3	7.3	4.8	4.8	4.8	1621900
26.3	26.3	0	0	0	14.7	14.7	14.7	1944700
13.7	11.1	16.2	16.2	13.7	13.1	10.2	10.8	5193200
7	7	7	7	7	7	7	7	1342200
22.7	22.7	22.7	22.7	11.3	22.7	22.7	22.7	1998100
25	25	25	25	0	25	25	25	1163900
65.5	65.5	65.5	65.5	65.5	65.5	65.5	65.5	22516000
14.2	10.7	5.8	14.2	10.7	17.2	9.4	14.2	2556100
39.6	32.1	27.1	39.6	26	39.3	27.1	29.9	30797000
16.6	17.9	16.6	16.6	12.5	17.9	12.2	13.5	11113000
0	10.7	0	0	0	0	10.7	10.7	260270
1.3	1.3	1.3	1.3	1.3	0	0	1.3	439190
16	16	16	16	7.7	16	16	16	4410800
5.1	5.1	9.5	9.5	9.9	3.6	3.6	3.6	3743100
9.4	7	9.4	3.3	5.3	4.7	2.2	10.8	2667400
0	0	0	0	0	2.5	8.5	8.5	632460
20.9	18	20.9	20.9	18	20.9	20.9	20.9	5538000
2.4	2.4	2.4	2.4	2.4	2.2	4.6	2.4	693760
14.3	14.3	14.3	14.3	14.3	14.3	14.3	14.3	5559400
15.9	9	20.8	21.8	19.4	30.3	34.7	34	28228000
4.2	4.2	4.2	4.2	4.2	4.2	4.2	4.2	6675500
4.6	4.6	4.6	2.7	2.7	2.7	1.6	2.7	2341700
6.1	6.1	6.1	6.1	6.1	6.1	6.1	11.3	879870
25.3	25.3	25.3	33.6	33.6	21.7	25.3	25.3	6062900
10.9	10.9	16.3	16.3	10.9	16.3	10.9	11.7	3538900
0	3.2	3.2	3.2	3.2	3.2	0	0	345470
17.3	17.3	17.3	17.3	17.3	17.3	17.3	0	1251100
16	7.7	7	10.2	7	15.3	22.3	15.3	7591600
8.6	8.6	0	2.6	2.6	8.6	8.6	2.6	741480
11.2	15	14.4	13.5	12	15.7	13.6	15.2	23702000
7.8	7.8	7.8	4.9	7.8	7.8	4.9	4.9	1547300
24.8	23.4	33.8	14.5	14.5	24.8	14.5	24.8	3563100
6	6	6	6	6	0	0	6	1858200
22.6	22.6	22.6	22.6	15.8	22.6	22.6	22.6	1148800
26.8	27.4	26.1	25.6	20	26.5	27.1	26.8	97891000
8	8	0	8	8	8	0	8	583370
51.7	43.4	38.6	43.4	35.9	43.4	54.5	46.9	72637000
1.8	3	3	1.8	3	1.8	0	1.2	788890
50.3	60.5	44.1	44.1	28.2	50.3	60.5	60.5	43159000
6.7	6.7	5.3	4.4	3.3	5.8	6.7	6.4	6908800

5.6	6	5.2	5.2	5.2	5.6	5.6	5.2	6188400
4.7	4.7	3.1	3.1	3.7	4.7	4	4	10237000
49.7	46.6	38.7	35.8	36.4	44.1	44.6	44.7	5.43E+08
0	0	32.7	51.9	51.9	51.9	51.9	51.9	2239700
30.4	30.4	30.7	34	25.6	35.2	35.2	35.2	36064000
0	0	0	0	0	7.8	7.8	7.8	664100
4.6	4.6	4.6	4.6	4.6	0	2.1	2.4	1112200
14.1	11.9	11.4	13.8	6.1	11.6	12.5	8.5	11177000
5.4	4.4	3.3	2.1	0	3.3	4.4	3.2	1333300
2.8	2.8	1.2	1.9	3.7	2.1	1.2	1.2	2084700
16.8	7.9	12.9	19.3	15.3	23.3	23.3	23.3	16093000
8.8	8.8	8.8	6	6.7	8.8	6.9	6	3401700
6	6	6	6	6	6	6	6	931080
12.9	12.9	12.9	12.9	12.9	12.9	0	0	579440
14.7	14.7	0	0	0	0	14.7	0	422290
6.8	6.8	6.8	6.8	6.8	6.8	6.8	13.7	1306300
13.1	13.1	13.1	13.1	13.1	5.2	0	7.9	1082800
0	1.8	4.4	4.4	0	0	0	4.4	645280
4.9	4.9	4.9	4.9	4.9	4.9	0	0	946160
4.9	4.9	4.9	4.9	0	4.9	4.9	4.9	658460
23.4	23.4	23.4	23.4	16.5	21.9	21.9	20.9	11169000
0	0	0	0	0	2.2	2.2	0	137130
5	2.1	7.1	7.1	7.1	4.8	2.1	2.7	1351500
8.3	6.4	6.9	6.9	6.9	3.4	4.7	6.4	4357500
7.7	0	7.7	7.7	0	7.7	16.7	16.7	680980
20.6	20.6	20.6	20.6	20.6	20.6	20.6	20.6	5865000
0	0	0	1.2	1.2	0	0	1.2	497000
8.1	0	0	0	0	8.1	0	0	213880
22.6	22.6	13	22.6	22.6	22.6	22.6	13	7858600
0	0	0	0	11.5	15.8	15.8	10.4	1883300
15.1	6.4	15.1	15.1	15.1	0	6.4	0	1340900
37.8	39.8	42.5	42.3	39.8	40.6	40.2	42.6	3.65E+08
2.7	0	2.7	0	0	0	2.7	2.7	403300
11.3	14.9	14.9	11.3	14.9	11.3	11.3	11.3	2754100
29	29	21.9	17.7	13.7	24.9	28.1	27.8	82941000
28.2	28.2	21.2	17.1	13.1	24.1	27.2	26.9	2526700
7.1	7.1	7.1	7.1	7.1	7.1	5.1	5.1	1653900
20	20	20	20	9.1	18.1	13.9	16.5	25410000
11.8	11.8	11.8	9.1	11.8	11.8	11.8	8.2	5320100
32	32	32	32	26.5	32	32	32	30288000
2.4	2.4	2.4	2.4	2.4	2.4	2.4	0	1126800
18.2	25.8	25.8	25.8	18.2	25.8	25.8	25.8	8934900
1.4	0	0	3.7	0	4.6	4.6	4.6	1868400
5.7	5.7	5.7	3.2	7.1	4.1	0	1.6	1636300
7.7	7.9	9.8	9.8	5.1	18.7	16.9	20.2	9591600
0	0	0	2.3	0	2.3	2.3	2.3	279660
1.2	1.2	1.2	1.2	1.2	0	0	0	1495100
5.5	5.5	5.5	5.5	5.5	4.7	5.5	4.7	8020200
0	0	0	0	28.9	0	13.3	23.3	2292800
22.9	27.8	23.1	16.6	17.4	30.6	30.6	32.1	24296000

29.3	21.4	29.3	29.3	16.4	29.3	29.3	29.3	6626200
2	1.6	1.6	1.6	1.6	3.6	1.6	6	663480
0	0	11.4	14.6	16.5	19.7	24.3	24.3	4753200
10.3	10.3	10.3	10.3	10.3	10.3	10.3	0	690480
0	0	0	4.6	4.6	21.1	21.1	21.1	1186600
4.1	4.1	1.4	4.1	4.1	4.1	5.4	7	2208300
0	0	20.4	20.4	20.4	20.4	20.4	20.4	520980
0	11.6	11.6	0	11.6	11.6	11.6	11.6	800320
4.6	6.5	6.5	3	4.6	4.6	6.5	6.5	1966400
7.3	10.9	10.9	10.9	10.9	16.9	13.3	9.7	4202100
22.2	20.8	25.5	23.3	24.5	20.3	19	23.8	1.09E+08
9.2	8.2	9.9	8.5	7.7	6.7	3.1	4.9	4331900
7	5.8	5.2	5.2	5.2	6.6	5.8	4.6	4520800
3.4	4.3	4.3	4.2	3.6	4.2	1.9	2.7	3044900
11.7	10.2	12.7	11.8	10.9	11.7	11.2	11.2	13829000
4.4	4.6	4.3	4.3	3.6	5.3	3.4	3	3268600
4.6	3.5	4.5	3.5	2.9	5.4	3.2	4.9	6881800
2.4	4.8	4.8	4.8	5.7	4.8	4.8	3.9	2722800
3.4	3.4	3.4	3.4	3.4	3.4	3.4	3.4	924110
4.4	4.4	4.4	4.4	4.4	2	0	4.4	930090
12.4	12.4	12.4	12.4	12.4	12.4	9.6	9.6	6258000
13.4	13.4	8.9	9.8	7.5	13.4	13.4	13.4	16831000
3.4	2.9	3.4	2.5	3.4	2.3	2.2	1.2	4354000
6.5	6.5	6.5	9.2	4.6	6.5	6.5	6.5	3371500
2.5	3.3	2.8	2	2	2.8	0	0	2127700
0	0	0	0	0	0	1.6	1.6	162200
15.3	11.1	11.1	13.2	13.2	15.3	7.3	7.2	6534700
11	22.8	22.8	11.8	16.5	22.8	56.7	38.6	5423200
0	0	8.2	0	11.5	11.9	11.9	11.9	1513800
2.3	2.3	2.3	2.3	2.3	2.3	0	2.3	540990
29.3	36.4	42.3	39.8	42.6	42.3	42.3	39.8	63787000
6.1	6.1	0	3.9	6.1	0	3.9	3.9	1083100
2.7	1.3	6.2	2.7	4.4	2.7	2.7	4.2	4548600
1.5	2.7	4	5.6	4	4	1.5	1.5	2036000
0	0	0	0	1.9	0	0	0	389110
9.5	12.8	13.2	11.5	9.6	13.2	6.8	15	8019600
0	0	0.7	0.7	0	0	0	0	51649
3.9	3.9	3.9	3.9	3.9	3.9	3.9	3.9	1574300
0	1.8	1.8	1.8	0	1.8	1.8	1.8	340350
0	14.7	14.7	14.7	0	14.7	0	0	566490
15.2	16.5	19.4	19.4	14.8	16.8	19.4	15.5	21823000
22.1	23.8	23.8	20.8	16.4	20.8	23.8	23.8	23070000
2.4	2.4	1.4	2.4	2.4	1.4	1.4	1.4	874330
24.9	23.8	24.6	25.7	21.5	23.6	25.2	29.2	49501000
3.8	3.8	5.2	5.2	5.2	5.2	1.6	3.8	3079100
21.1	24.1	19.7	22.1	23.7	23.4	30.7	24.8	85732000
0	0	0	0	0	0	11.4	0	167920
33.7	36.4	38.8	38.8	39.5	38.8	38.8	38.8	2.19E+08
5.3	5.3	5.3	5.3	5.3	5.3	0	0	696850
10.5	10.5	7.9	10.5	2.6	10.5	10.5	3.7	1678100

20.1	20.1	24.3	20.1	24.3	20.1	24.3	14.2	5376900
9.6	9.6	14.1	14.1	14.1	14.1	9.6	5.5	1803000
21.4	21.4	10	16.1	19.2	16.1	16.1	21.4	3839500
6.3	5.5	6.1	5.7	7.4	6.9	5.9	7.5	10354000
0	20	0	20	0	20	20	20	537250
5.9	25	17.1	17.1	17.1	34.2	40.8	31.6	486650
12.7	32.5	20.9	24.3	24.3	36	45.9	36.3	29044000
23.2	23.2	23.2	14.1	14.1	23.2	23.2	23.2	13501000
20.1	6.5	19.8	23.7	19.8	23.7	23.7	19.8	3849800
0	0	0	0	2.2	4.4	4.4	2.2	607260
8.3	8.3	8.3	8.3	8.3	8.3	8.3	8.3	411800
9.5	0	9.5	9.5	0	9.5	9.5	9.5	750540
17.5	17.5	17.5	17.5	17.5	12.3	11.7	11.7	5826100
14.5	14.5	14.5	14.5	14.5	14.5	14.5	14.5	2994400
33.7	33.3	33.3	33.3	37.3	33.3	30.8	37.3	7653300
5.3	5.3	5.3	5.3	5.3	5.3	5.3	5.3	1074700
13.9	13.9	0	0	0	0	0	13.9	418610
11.6	11.6	11.6	11.6	0	0	0	0	212750
26.7	26.4	24.4	24.4	18.7	23.5	23	26.7	33205000
0	0	11.7	0	11.7	11.7	0	11.7	810790
13	13	11	6.8	8.6	11	13	10.6	2511500
0	0	2.3	2.3	2.3	0	0	0	306620
7.4	7.4	7.4	3	3	5.6	4.4	7.4	1940000
17.2	17.2	17.2	17.2	17.2	17.2	17.2	17.2	860420
5.8	5.8	5.8	5.8	0	5.8	5.8	5.8	727740
2.8	0	0	2.8	2.8	10.7	10.7	10.7	1849600
8.2	8.2	23.1	23.1	19.8	20.7	38.6	38	15999000
4.2	4.2	4.2	4.2	4.2	7.5	4.2	4.2	575470
4.6	1.7	4.6	4.6	4.6	4.6	2.9	2.9	1331900
78.1	78.1	78.1	52.4	43.8	78.1	78.1	78.1	1574700
60	60	58.2	41.8	41.8	58.2	60	70.9	4.1E+08
41.2	44.9	38	41.2	19.9	36.1	39.8	38.9	73943000
6.1	6.1	6.1	6.1	6.1	6.1	6.1	6.1	1150000
0	0	4.3	4.3	21.7	0	0	3.4	1862000
0	13	13	13	25	25	40.7	40.7	7712000
9.9	9.9	18.3	18.3	18.3	18.3	18.3	18.3	5847700
0	0	4.9	4.9	0	14.7	14.7	14.7	1858800
11.5	18.7	13.7	23.3	23.3	24.4	30.1	26.6	11753000
6.2	6.2	6.2	6.2	6.2	6.2	6.2	6.2	1390200
10.2	0	0	8.4	10.2	8.4	10.2	10.2	724870
0	0	0	0	0	0	0	2.8	70840
23.3	23.3	23.3	23.3	10.6	23.3	23.3	23.3	3949600
2.2	0	0	2.2	2.2	2.2	0	2.2	226320
7.8	11.1	26.5	24.9	19.7	23.2	26.5	21.6	8318800
6.5	4.5	3.8	2.1	1.7	1.7	4.5	1.7	1883600
55.3	55.3	46.2	49.7	49.7	60.8	72.9	66.8	1.42E+08
24.7	24.7	28.8	24.7	28.8	32.3	53.5	49.5	10840000
50.9	46.1	40.6	21.8	32.1	42.1	46.1	43.9	17370000
48.1	48.1	48.1	48.1	38.3	48.1	48.1	48.1	30821000
29.5	33	36.2	36.2	36.2	40.2	40.2	36.2	27706000

28.2	28.2	28.2	28.2	28.2	28.2	28.2	0	983890
0	2.5	2.5	0	0	0	0	0	135400
2.7	6.3	2.7	6.3	6.3	2.7	0	2.7	648700
30.7	28.7	32.2	30.7	26.6	28.7	24.3	27.3	30059000
4.6	4.6	4.6	4.6	0	4.6	0	4.6	286520
5.3	9.6	8.9	5.4	13.5	7	7.7	6	2875900
38.2	38.2	38.2	38.2	38.2	38.2	0	0	5338900
26.4	26.4	26.4	11.8	11.8	26.4	26.4	26.4	2057700
2	3.8	3.8	2	3.8	2	4.4	6.2	1378800
5.3	5.3	3.4	3.4	6.6	13.6	15.1	14.9	4117800
0	0.7	1.4	1.4	1.4	0.7	0.7	1.4	673900
20.2	20.5	15.8	15.8	15.8	22.4	22.4	19.9	46354000
4.3	4.3	4.3	4.3	4.3	4.3	0	4.3	563900
0	0	17.6	8	22.5	22.5	54.5	54.5	8539500
14.3	14.3	14.3	0	14.3	0	0	14.3	1103800
7.7	7.7	7.7	7.7	13.3	13.3	16.6	16.2	2426700
4.5	4.2	8.3	10.3	8	10.3	12.5	12.5	2711700
4	4	0	4	0	4	4	4	981490
8.3	8.3	8.3	8.3	7.8	8.3	8.3	6.1	2802500
0	0	5.5	4.6	5.5	11	15.5	13.5	2834500
0	0	8.2	8.2	8.2	8.2	8.2	8.2	1001000
38.3	47.8	43.4	38.8	38.6	47.6	49.9	47.6	49671000
26.8	22.8	31.1	31.1	31.1	37.4	40.6	49.2	46206000
4.5	2.4	2.4	2.4	2.4	4.5	0	4.5	801620
4.2	4.2	7.4	7.4	3.2	4.2	4.2	4.2	1301300
0	8	8	0	0	8	8	8	393430
5.7	5.7	8.4	14.9	6.2	18.1	26.5	23.8	6609300
0	0	0	7	7	7	7	7	395890
6.1	6.1	6.1	6.1	6.1	6.1	6.1	3.8	4104500
0	0	0	0	0	0	0	3.1	435090
3.3	5.2	5.2	3.3	0	0	5.2	5.2	817320
2.4	2.4	2.4	2.4	3.7	2.4	3.7	1.3	709190
6.5	14.9	14.9	14.9	12.3	10.2	12.2	10.7	7686600
3.8	3.8	3.8	3.8	3.8	2.8	2.4	0.9	1898800
8.9	10.2	20.9	23.7	23.7	26	30.8	37.4	25209000
8.4	8.4	6	13	2.4	18.8	18.8	16.4	9447200
13.6	12.9	19.5	23.3	15.3	31.7	33.3	36.7	31498000
10.2	9.6	9.6	13.3	13.3	26.4	27.1	24.8	9969300
8.8	9.8	14.9	16.6	12.7	16.6	16.6	16.6	4092800
0	0	0	0	0	0	14.4	14.4	278780
3.5	3.5	3.5	3.5	3.5	3.5	3.5	3.5	889960
23.7	24.2	24.3	21.6	19	20.5	20.8	20.9	1.39E+08
2.2	0	7.5	7.5	7.5	2.2	7.5	7.5	1452600
0.8	0.8	0.8	0.8	0.8	0.8	0	0	379040
4.6	4.1	4.6	4.1	3.4	4.1	3.8	3.9	3176600
17.6	18.2	15.3	14.3	15.5	16.6	19.1	17.9	39277000
2.1	2.1	2.1	2.1	2.1	0	0	0	467170
25.3	36.2	18.8	21.9	18.3	34.8	34.8	38.8	39022000
19.4	23.8	20.8	23.8	15.5	23.8	30.7	27.4	14542000
6.3	6.1	20.8	20.8	17	20.2	20.2	17	5731700

29.6	31.1	23.6	24.2	18.9	25.8	32.5	32.2	40955000
3.7	3.7	3.7	9.1	9.1	9.1	9.1	9.1	1742600
7.8	10.2	6.3	8.8	6.3	11.7	13.7	11.7	1804000
0	0	0	0	0	6.6	3.1	6.6	409640
3.4	3.4	3.4	3.4	3.4	3.4	3.4	3.4	1098800
6.2	3.2	3.2	3	3	6.2	6.2	6.2	580050
21	24.4	26.2	22.8	17.9	22.8	22.3	20.3	38498000
18.2	17	17	17	14	13.8	13.5	11.7	8365200
12.4	9.7	12.4	12.4	8.9	12.4	3.5	9.7	2122800
18.2	21.3	21.3	17.5	8.2	25	28.8	28.8	34937000
19.5	19.5	13.3	9	6.8	14.4	21.2	16	5686400
4.5	3.3	3.3	1.3	2.4	1.3	1.3	3.3	1511000
6.9	6.9	6.9	6.9	6.9	6.9	0	0	932480
0	0	0	9.4	9.4	9.4	9.4	9.4	533400
0	0	0	0	0	0	9.1	0	0
0	0	0	0	0	8.8	8.8	0	282780
52.7	57.1	54.5	52.1	51.8	56	57.1	58.6	2.5E+08
15.5	21.8	21.8	21.8	24.8	21.8	26.6	29.6	24315000
2	2	1	1	2	2	2	2	1102100
4.4	8	4.4	5.8	5.8	4.6	5.5	7	3970500
6.3	6.6	6.3	5.1	3.8	6.9	6.8	6.8	11193000
0	3.8	3.8	0	3.8	3.8	0	3.8	305710
7.2	7.2	7.2	7.2	4.7	7.2	8.7	6	4606800
4.9	0	0	0	0	4.9	4.9	4.9	246830
3.7	2.6	3.7	3.7	6.3	2.6	3.7	2.6	2389300
8.3	11.1	13	14.6	14.6	11.1	9.9	7.3	10945000
7.7	7.7	1.6	0	0	7.7	7.7	7.7	2342700
10	8.7	8.6	8.3	6.9	10	7.3	6.6	7861400
3.8	0	3.8	3.8	0	3.8	3.8	3.8	504570
15.1	13.3	19.7	20.8	17.2	15.9	16.3	14.3	19692000
7	7	7	7	7	7	7	7	1.96E+08
28.5	30	25.5	23	21.8	29.8	31.4	33.1	36487000
4.4	6.3	10.5	8.8	10.5	9.2	10.5	7.5	6886600
3.2	5.5	3.2	5.5	2.3	0	0	0	708550
8.2	7.2	4.1	1.8	1.9	6.6	7.2	6.3	3009800
7.6	7.6	7.6	7.6	7.6	7.6	7.6	7.6	1942000
26.2	25.1	16.9	20.7	15.3	25.2	28	26.2	17250000
0	7.6	7.6	7.6	7.6	7.6	7.6	7.6	1010600
7.2	5	7.2	7.2	5	7.2	7.2	7.2	2317200
2.3	2.3	4.6	2.3	4.6	2.3	0	0	1104200
2.4	3.3	2.4	0.9	3.3	2.4	0.9	2.4	758930
0	0	0	0	2.8	0	0	0	630090
18.2	18.2	18.2	18.2	18.2	18.2	18.2	18.2	1220300
3	1.4	3	3	3	3	0	1.7	944460
8.6	14.7	14.7	16.9	14.1	11.4	14.1	15	6092500
14	11.6	11.6	14	12.5	12.7	10	8.4	10737000
6.1	4.5	6.1	7.6	6.1	6.1	2.1	4.5	4590800
3.7	3.7	3.7	3.7	3.7	3.7	3.7	3.7	1435000
13	10.8	10.7	10.8	6.5	9.5	11.7	9.5	10548000
59.3	59.3	59.3	69.3	63.6	70	60	60	1.63E+08

6.5	8.2	8.1	8.1	6.5	9.2	8.2	8.8	6695400
0	0	0	0	0	0	0	4.6	295450
0	19	8.3	0	0	19	19	19	1054000
48.2	44.5	44.5	46	38.6	47.8	47.8	44.5	80336000
41.8	47.5	45.2	43.1	29.1	32.8	51.2	46.2	64651000
23	13	38.3	30.3	33.3	31	33.3	38.3	21763000
19	23.4	18.3	18.8	10.7	35	45.2	41.1	24992000
14.3	11.8	12	12.4	9.3	7.6	7.6	7.6	7880500
4.9	4.5	2.3	2.3	2.3	2.3	0	4.9	1204500
4.1	2.4	4.1	2.4	2.4	4.1	5.9	4.1	1393900
10.4	10.4	7.4	5.9	5.7	5.7	14.2	14.2	5297400
11.8	11.8	11.8	11.8	6.6	11.8	5.3	11.8	21095000
19.2	19.2	19.2	19.2	5.3	19.2	19.2	19.2	4980400
33.5	24.4	0	7.9	7.9	18.9	29.3	15.2	12789000
1.6	2.7	2.7	1.6	1.6	2.7	2.7	1.6	1047000
6.3	0	17.9	3.7	10	21.3	28.9	21.9	2977900
8.8	8.8	8.8	3.9	3.9	3.9	3.9	8.8	1661800
0	0	20.2	15.5	10.7	20.2	23.2	18.5	3596300
3.9	0	3.9	7.9	3.9	7.9	3.9	3.9	1557300
0	0	0	0	0	22.9	14.5	22.9	2001200
0	11.2	11.2	11.2	0	11.2	11.2	11.2	495850
0	12.8	17.1	0	0	17.1	17.1	12.8	2519400
26.4	22.7	22.7	22.7	19.1	26.4	35	35	6916700
11.8	26.1	36.9	31	31	36.9	41.9	36.9	11676000
17.9	23.6	23.6	19	16	23.6	39.9	39.9	10341000
0	0	4.8	4.8	8.5	12.5	8.9	12.5	3052000
25.3	30.3	19.9	15.8	29.5	28.6	43.6	42.7	13224000
31	25	31	25.4	16.9	31	51.6	48.4	14539000
0	0	3.6	3.6	0	7.6	12.7	3.6	855830
0	0	7.6	7.6	0	7.6	7.6	7.6	572520
27	27	30.7	27	21.2	30.7	30.7	24.9	10628000
0	0	0	0	0	9	9	9	1257700
8.4	21.5	21.5	21.5	8.4	21.5	8.4	21.5	2565100
0	0	7.6	7.6	7.6	7.6	7.6	8.3	1694500
13.7	8	8	12.9	13.7	28.9	28.9	28.5	4315100
13	8.8	13	13	8.8	13	13	13	5643300
3.2	3.2	3.2	3.2	3.2	6.9	6.9	3.2	1531000
0	0	0	0	0	0.8	0.8	1.4	955020
3	0	8.9	3	11.4	5.5	8.9	3	1256400
21	23.2	24.1	21	24.1	31.7	18.7	27.5	8600700
10.4	10.4	10.4	10.4	7.2	10.4	10.4	7.2	1499800
0	0	0	0	1.2	1.2	1.2	1.2	264330
7.6	0	7.6	7.6	7.6	7.6	13.9	7.6	829540
18.3	12.5	12.5	17.5	14.1	12.5	18.3	23.2	6889100
19.5	10.2	19.5	19.5	10.2	19.5	10.2	19.5	1427600
0	0	25.8	48.8	48.8	48.8	48.8	48.8	5168700
14.6	14.6	14.6	14.6	0	14.6	14.6	14.6	3449300
0	0	10.4	10.4	10.4	10.4	10.4	10.4	912490
29.6	29.6	29.6	34.4	29.6	34.4	38.1	38.1	14085000
12.8	12	15.5	15.5	9.3	15.5	15.5	15.5	10303000

0	1.8	1.3	1.1	4	5.1	5.6	5.9	4302300
36.8	36.8	32.1	35.2	22	35.2	30.5	28.7	1.08E+08
42	42	37.2	37	33.1	41.6	46.5	44.8	1.91E+08
23.6	20.2	23.4	24.5	21.1	17.4	20.8	16.3	31065000
29.3	22	28.4	25.6	25.2	24.5	24.5	21.7	84720000
12.5	0	12.5	12.5	12.5	0	0	0	515750
1.7	1.7	2.9	2.9	2.3	0.6	1.1	1.1	2390900
17.8	15.7	16.3	17.8	13.7	14.7	16.3	10.4	21658000
12.3	0	12.3	12.3	12.3	0	0	0	502000
1.8	1.8	1.8	2.8	1.8	1.8	1.8	1.8	2697500
21.5	21.5	19.4	19.4	19.4	19.4	19.4	21.5	6954000
11	11	0	11	11	0	11	11	7510000
12.8	24.7	21.1	12.8	20.7	24.7	21.1	19.8	7782200
0	4	13	0	4	13	13	13	2107700
25.1	26.4	23.9	24.3	22.9	25.4	19.8	17.3	32733000
4.2	4.2	0	0	0	4.2	4.2	4.2	568520
7.6	7.6	3.6	7.6	7.6	7.6	11.8	11.8	1418700
2.1	1.2	2.1	1	1.2	1	1	1	833300
0	0	1.9	1.9	0	0	0	0	423070
6.6	0	0	0	6.6	6.6	6.6	0	376210
2.8	2.8	2.8	2.8	2.8	2.8	2.8	2.8	1529900
3.5	0	0	0	3.5	6	6	6	606390
3.4	6.5	6.5	6.5	3.4	6.5	6.5	6.5	2148800
2.3	2.3	2.3	2.3	2.3	0	0	0	2166500
14.2	14.2	14.2	14.2	14.2	13.2	13.2	13.2	4749200
0	0	0	7.5	13.3	0	7.5	13.3	630870
2.6	10.8	10.8	10.8	10.8	10.8	10.8	10.8	1017700
6.7	9.8	11.7	11.7	9.6	11.7	8.6	9.8	9725600
30.4	30.4	19.6	19.6	30.4	30.4	30.4	10.8	5459900
8	0	0	0	8	0	0	8	270620
6.2	6.2	6.2	6.2	6.2	6.2	0	6.2	2756600
20.1	17.8	22.3	17.8	14	30.5	39.6	39.6	10972000
0	1.1	1.5	2.7	1.5	5.2	5.2	5.2	2771100
0	7.7	2.8	4.9	0	4.9	7.7	0	945620
4	4	4	4	4	4	4	4	1681200
17	0	17	17	17	17	17	17	1914800
0	0	0	0	0	0	6.9	6.9	1088700
2	2	2	4.9	4	12.1	14.3	7.1	3312300
19.5	20.5	18.2	20.5	24.5	18.2	18.9	18.6	13174000
0.6	0	0.6	0	0	1.3	1.3	0.6	400360
12.8	10.3	11.1	11.1	12.8	15.7	15.7	17.8	5256800
11.4	11.4	11.4	11.4	11.4	11.4	11.4	11.4	3210400
2.8	2.8	2.2	4	4.8	2.2	2.2	2.8	3682100
9.4	9.4	9.4	17.6	17.6	17.6	17.6	17.6	25104000
17.5	35.1	17.5	17.5	17.5	17.5	35.1	35.1	27511000
34.3	34.3	34.3	34.3	23.8	34.3	34.3	34.3	65928000
0	0	0	11.2	0	11.2	11.2	11.2	810730
54.8	65.4	65.4	44.2	14.4	54.8	65.4	54.8	8085700
11.7	22.3	22.3	22.3	10.7	22.3	22.3	22.3	4148900
27.6	27.6	27.6	17.3	17.3	26.5	27.6	27.6	28664000

10.5	10.5	10.5	10.5	6.8	10.5	10.5	10.5	1311200
23.3	19.9	15.2	23.3	23.3	23.3	19.9	23.3	7780400
5.1	0	11.6	19.9	19.9	29.2	30.7	29.2	32131000
3	2.4	3.1	3.7	3	1	1	2.7	2440100
7.9	7.9	7.9	7.9	7.9	7.9	7.9	7.9	563830
7.7	10.6	10.6	7.7	7.7	7.7	7.7	7.7	2556300
22.5	13.9	22.5	16.8	22.5	16.8	25.4	25.4	6670500
2.7	1.4	2.7	2.7	2.7	2.7	2.7	1.2	1023300
0	1.5	1.5	0	1.5	1.5	1.5	1.5	324220
8.5	8.5	8.5	8.5	8.5	6.2	8.5	8.5	11893000
37.5	37.5	37.5	37.5	37.5	26	26	26	18401000
17.6	17.6	17.6	17.6	17.6	17.6	0	0	4299900
2.7	2.7	0	0	0	0	2.7	2.7	551070
19	19	19	19	19	19	19	19	1601500
0	4.7	0	0	4.7	4.7	4.7	4.7	704950
16.5	16.5	16.5	20.4	23.5	23.5	23.5	23.5	4239300
3	3	2.1	2.1	2.1	2.1	2.1	1.4	1388100
0	0	25.2	27.1	26.2	36.4	36.4	22.4	29695000
11.4	7.4	7.2	11.4	9.7	9.7	4.3	9.1	5457100
3.6	3.6	3.6	2.3	1.1	2.4	1.2	1.1	1031200
0	0	6.2	12.1	6.2	15.6	34.3	28.4	3598600
14.5	7.4	14.5	7.4	14.5	25.6	25.6	25.6	3949000
2.6	2.6	7.1	7.1	2.6	9.5	9.5	10.5	4958100
4.3	4.3	4.3	0	0	6.8	6.8	6.8	926820
0	0	0	0	0	6.4	6.4	6.4	547390
2.1	2.1	2.1	2.1	2.1	2.1	0	2.1	1012500
42.9	42.9	39.6	35.3	30.1	42.9	40.9	36.8	7.91E+08
14.2	18.9	18.9	18.9	15.2	18.9	22.8	22.8	1769900
6.1	6.5	6.5	6.5	4.4	6.5	9.9	9.9	498250
2.3	2.3	1.3	1.3	3.9	2.3	2.3	3.9	1404900
7.2	7.2	7.2	3.5	7.2	7.2	7.2	3.5	1357300
0	0	2.7	2.7	2.7	2.7	0	0	465650
2.5	0	0	0	2.5	0	0	1.3	115480
3.3	5.9	5.9	5.9	9.2	9.2	5.9	5.9	1603600
4.2	4.2	4.2	4.2	4.2	0	0	0	271190
8.3	8.3	0	0	8.3	8.3	0	0	126010
12.2	12.2	6.9	6.9	6.9	12.2	12.2	12.2	1812400
7.6	5.6	8.1	6.9	6.6	5.6	5.6	5.1	6997800
8.9	8.9	8.9	8.9	6.4	8.9	8.9	8.9	3846300
17	8.8	12.3	17	17	12.3	5.6	13.5	6861000
0	0	3.9	3.9	3.9	0	3.9	3.9	806170
54.2	59.3	52.5	52.4	54.8	59.3	64.2	71.2	6.43E+08
47.8	52.9	46.1	46	48.4	57.1	58.9	65.9	290240
18.4	13.9	26.5	22.9	15.1	33.5	31.2	30.8	18575000
7.9	7.9	7.9	7.9	7.9	5.4	5.4	7.9	3804300
42.2	42.2	42.2	41.6	23	42.2	42.2	42.2	9986300
25.3	25.3	25.3	25.3	25.3	25.3	25.3	25.3	1406200
13.1	13.1	15.5	16.7	12.9	18.1	17.5	17.2	24225000
22.5	22.5	22.5	27	22.5	27	27	27	13485000
0	11.2	9.9	12.2	8.8	15.2	26	23.2	12609000

17.1	17.1	19.8	21.5	16.1	28.1	27.7	28.5	49582000
0	0	7.6	7.6	0	7.6	7.6	11.2	607060
23.4	38	29.2	40.1	33.3	30.2	47.4	49.5	22027000
23.4	38	17.2	40.1	33.3	30.2	40.1	42.2	4104800
3.5	3.5	0	0	3.5	3.5	0	3.5	636850
27	27	30.5	30.5	30.5	30.5	30.5	30.5	17119000
44	45	48.2	44	30.7	47.2	50.9	47.2	38319000
72.6	72.6	72.6	22.8	53	61.9	68.8	68.8	40919000
39.8	37.4	46.6	30.6	29.1	39.8	30.6	46.6	11819000
59.5	53.7	59.5	59.5	56.1	55.6	59.5	59.5	75766000
50.7	50.7	50.7	50.7	54.7	46.8	50.7	43.3	8000900
23.6	23.6	23.6	16	23.6	16	16.9	23.6	7150300
8.9	8.9	8.9	8.9	8.9	5.2	8.9	5.2	556210
15.5	15.5	15.5	15.5	15.5	15.5	15.5	15.5	828410
37.7	37.7	32.1	25.9	27.4	32.1	32.5	29.2	20645000
24.9	29.3	29.3	29.3	29.3	29.3	24.9	15.6	6617400
15.7	15.7	8.1	8.1	0	15.7	7.6	15.7	4087500
5.2	0	5.2	5.2	5.2	5.2	0	5.2	352100
38.9	38.9	38.9	34.3	27.8	32.4	38.9	38.9	34666000
17.3	27.4	21.6	21.6	21.6	21.6	21.6	21.6	43434000
64.7	76.3	71.5	65.2	63.8	73.4	74.9	76.3	1.07E+08
10.8	10.3	10.3	10.3	10.8	10.3	10.3	10.3	625990
8.7	8.7	14.5	8.7	14.5	8.7	14.5	14.5	350430
12.9	18.4	18.4	18.4	18.4	11.4	5.5	0	2143400
24.4	24.4	29.3	24.4	24.4	24.4	24.4	24.4	9136100
30.3	30.3	30.3	30.3	30.3	30.3	37.6	23.6	10369000
20.2	26.8	20.2	20.2	20.2	20.2	20.2	8.7	3609700
17.9	17.9	17.9	17.9	12.4	17.9	17.9	17.9	1014600
23.5	23.5	23.5	23.5	19.1	23.5	23.5	23.5	5906800
28.8	28.8	28.8	28.8	28.8	28.8	28.8	28.8	2833000
15.9	15.9	15.9	15.9	11.1	11.1	11.1	11.1	7464800
3.3	1.7	3.3	3.3	3.3	3.3	3.3	1.7	1451800
3.4	2.3	2.3	2.3	3.4	2.3	2.3	3	4142400
21.1	21.1	21.1	21.1	9.2	15.6	15.6	15.6	7301000
16.4	28	24	20.9	16.4	31.1	31.1	26.6	13809000
12	7.4	7.4	7.4	12	12	7.4	12	2887100
6.8	6.8	6.8	6.8	6.8	6.8	0	6.8	484420
1.5	1.5	1.5	1.5	1.5	1.5	1.5	1.5	559340
5.8	5.8	5.8	5.8	2.8	5.8	5.8	5.8	1632500
8.5	8.5	8.5	8.5	8.5	8.5	8.5	13.8	2485300
24.6	24.6	22	15.1	9.2	17.7	11.5	24.6	2983700
9.2	9.2	0	6	3.3	6	9.2	9.2	1451200
4.7	4.7	4.7	4.7	0	4.7	4.7	11.3	800370
0	40.2	19.5	19.5	12.2	31.7	52.4	40.2	1562300
0	0	0	4.4	4.4	9.6	9.6	9.6	843560
1.6	0	1.6	1.6	0	1.6	1.6	3.6	957060
29	31.1	14.2	14.2	6.3	14.2	29	29	13883000
12.9	12.9	21.9	21.9	12.9	21.9	22.6	22.6	3327100
16.9	27.1	23.3	27.1	20.6	20.6	30.8	30.8	46293000
1.4	1.4	0	0	0	1.4	1.4	1.4	333470

8.7	8.7	8.7	8.7	3.6	8.7	8.7	8.7	4195400
12.3	7	7.9	12.3	7	7.9	5.3	7.9	1727900
23.6	23.6	23.6	23.6	14.5	23.6	17.3	23.6	9789200
3.6	7.4	7.4	3.6	7.4	7.4	3.9	7.4	582270
24	24.8	24	24	24	24	24.8	24.8	8819900
14.8	14.8	34.1	34.1	14.8	34.1	34.1	34.1	1644100
1.8	4.6	4.6	9.1	1.8	11.6	13.2	13.2	4385400
0	0	0	0	0	0	0	8.1	1560200
24.5	24.5	24.5	17	17	24.5	24.5	24.5	14147000
26.7	32.5	32.5	32.5	41.4	35.6	32.5	32.5	3910900
17.8	15.2	17.8	16.7	16.7	28.9	24.3	30.4	9602300
0	0	1.6	0	0	2.8	2.8	4.7	781030
5.2	9	9	3.8	8.3	13.8	18.3	18.3	2093100
4.1	8.8	4.7	4.7	4.7	8.8	8.8	11.3	1295300
10.1	10.1	4.7	4.7	4.7	10.1	14.2	16.7	2410900
0	0	0	0	0	4.8	4.8	4.8	350670
19	19.6	23.3	23.3	28	21.8	17.8	21	36323000
13	13	17.6	14	14	14	17.6	17.6	23159000
14.1	14.1	14.1	14.1	16.7	12.1	2.8	12.1	5024400
8.6	8.6	11.9	11.9	11.9	11.9	11.9	11.9	2221900
0	8.6	8.6	8.6	8.6	8.6	0	8.6	1223600
4.4	2.7	5.5	5.5	5.5	1.9	3.6	1.9	2297200
9.5	7.6	9.5	6.4	10.6	6.2	10.6	5.8	8512000
3.1	12.5	4	3.1	3.1	5.4	14.2	9.1	3734900
17.5	17.5	17.5	12.2	17.5	17.5	17.5	12.2	2891600
15.8	11.8	11.8	15.8	13.5	11.8	9.5	9.5	5357600
7.4	7.4	7.4	7.4	7.4	7.4	7.4	3.3	3041200
0	0	11.6	20.9	9.3	20.9	20.9	9.3	684590
11.7	19.6	11.7	11.7	11.7	11.7	19.6	0	2132100
4	5.8	11.6	13.2	13	13.3	10.3	13	11389000
22.9	30	30	30	22.4	22.9	17.1	13.3	8004800
4.2	4.2	0	0	0	0	0	0	290010
14.3	10.7	25.1	25.1	26.6	28.6	30.7	28.6	28285000
17.3	17	17	17	17	15.5	17	14.3	17622000
19.2	19.2	19.2	19.2	19.2	19.2	19.2	19.2	3351900
6.4	6.4	3.8	6.4	6.4	6.4	6.4	6.4	3781300
0	0	0	0.9	0	0	0	0	0
6.9	8.4	8.4	8.4	6.7	8.4	7.2	7.2	4229400
0	6.2	10.4	10.4	10.4	10.4	10.4	6.2	1103400
24.9	29	24.9	18	24.9	29	34.7	30.6	9218000
10.9	15.5	20.7	20.7	20.7	20.7	20.7	15.5	6831000
3.9	3.1	8.4	7	8.6	11.7	14.1	11.7	8088600
1.7	4.4	13.9	13.9	13.9	13.9	13.9	13.9	6485400
13.6	13.6	13.6	13.6	13.6	13.6	13.6	13.6	375650
3.2	9.5	9.5	9.5	9.5	9.5	6.3	9.5	1016200
17.7	21.1	21.1	16	5.1	24.9	24.9	21.1	5085500
12.8	12.8	7.1	7.1	7.1	7.1	5.7	7.1	2593100
0.6	1.4	1.4	1.4	1.4	1.4	0.6	1.4	1532100
27.9	27.9	27.9	27.9	27.9	22.3	22.7	22.7	22484000
3.1	3.1	3.1	3.1	0	3.1	3.1	3.1	328070

3.3	3.3	3.3	3.3	3.3	3.3	0	0	3026500
18	18	18	18	18	18	0	18	577720
0	1.8	1.8	1.8	2.8	1.8	2	1.2	1926200
3.9	7.8	7.8	7.8	8.5	3.9	3.9	3.9	1594800
26.3	27.4	27.4	26.5	17.1	36.2	32.5	29.4	22745000
23	28.5	23.7	18.4	26.1	19.4	29.7	33.5	11797000
6.6	15.7	6.6	11.9	6.6	21.5	21.5	21.5	5983000
0	0	0	0	0	9.4	4.7	9.4	956140
23.9	23.2	23	23	22.3	20.6	21.4	19.7	72875000
9.6	14.6	15	14.9	15	16.3	8.5	13.1	12681000
6.3	8.2	11.6	11.5	13.2	11.5	4	9.4	849950
2.5	2.5	2.5	2.5	2.5	2.5	2.5	2.5	2747400
1.6	1.6	1.6	0	0	1.6	0	1.6	356750
0	0	9.3	9.3	4.8	9.3	6.7	4.2	2867700
0	3.1	3.9	9.7	9.7	9.7	9.7	9.7	1896400
13.4	13.4	13.4	13.4	13.4	13.4	13.4	0	1403500
6.1	6.1	6.1	6.1	6.1	6.1	6.1	6.1	2803800
19	14.4	14.4	19	19	19	9.8	10.4	3957300
0	0	0	0	0	0	0	5.6	140050
0	0	0	0	0	5.7	5.7	5.7	216180
0	0	8.7	8.7	8.7	8.7	0	0	217360
53	53.2	53.2	49.8	50.9	48.6	53.2	53	3.57E+08
4	9.9	4	4	4	4	4	9.9	1355000
37.5	33.1	30.8	33.1	28.6	30.4	37.5	32.9	40162000
5.5	2.6	9.3	6.6	6.6	8.6	5.7	4.9	2753800
21.6	18.6	21.6	15	21.6	17.1	14.2	21.6	17486000
2	4.1	4.1	4.1	2	4.1	4.1	2.1	1328300
0	0	0	0	1.5	0	0	0	0
4.8	4.3	5.7	5	4.8	5.7	3.7	4.2	9462700
26.9	30.4	30.4	30.4	16.6	30.4	26.9	26.9	18141000
13.4	13.4	7	7	7	0	7	0	919030
4.4	4.4	4.4	4.4	4.4	4.4	4.4	4.4	993400
42.1	36.1	42.1	42.1	36.1	42.1	42.1	42.1	12764000
32.6	32.6	32.6	32.6	32.6	17.9	40	40	10707000
13.4	13.4	13.4	13.4	7.8	13.4	13.4	10	3079600
12.5	12.5	12.5	15.2	15.2	12.5	15.2	9.9	10195000
30.7	42.3	37.2	32.1	30.7	15.3	35.8	42.3	16978000
23.1	28.5	21.3	33	21.3	22.2	33	21.3	8865600
4.3	8.4	8.4	4.1	4.3	8.4	8.4	4.1	1275500
3	4.6	4.6	4.6	4.6	3	3	4.6	972280
3	0	0	0	0	3	3	3	483590
0	1.7	7.6	13.9	8.4	15.8	13.4	12	3073600
41.5	0	41.5	41.5	41.5	41.5	41.5	41.5	1131700
5.1	5.1	5.1	5.1	5.1	5.1	5.1	5.1	1785000
10.7	12.2	14.4	16.5	11	18.3	22.9	19.4	12268000
0	0	0	0	0	8.7	17.1	17.1	1012500
23.9	29.8	18.4	24.3	19.7	33.3	33.3	33.3	11162000
16.5	21.2	16.5	16.5	8.6	16.5	16.5	21.2	4514100
17.5	21	18.5	9.1	3.1	21.7	31.5	31.5	1805300
25.7	28.7	18.7	11.3	5.8	22.9	31.5	31.5	16024000

4	4	4	4	0	6.9	2.8	6.9	1713400
11.3	6.9	6.9	4.4	6.9	11.3	26.1	6.9	1121100
19.7	23	21.4	13.5	6.2	21.4	30.6	30.6	1216800
14.9	18.3	9.7	14	14.9	18.6	22.9	22.9	4562200
4.3	5.4	4.3	2.6	4.3	9.5	7.1	9.5	1907700
6.9	6.9	6.9	6.9	3.5	6.9	3.5	3.5	1564100
11.5	7.3	11.5	11.5	0	15.8	27.9	27.9	2453600
16.3	26.9	25.1	28	21.9	35.7	46.1	48	29638000
0	3.9	11.1	9.5	3.9	20.8	20.8	17.6	4974800
35.2	35.2	35.2	32.1	23.2	35.2	32.3	28.9	66101000
2.4	1.2	2.4	1.2	1.2	3.7	2.5	3.7	1984800
4.7	4.7	4.7	4.7	4.7	4.7	0	4.7	2028200
0	0	15	0	0	0	15	15	529600
0	11.4	20.5	20.5	20.5	20.5	20.5	20.5	5960500
0	0	6.7	6.7	2.8	6.7	6.7	6.7	1309400
11.5	11.5	19	19	23.4	6.9	8.7	4	5995800
23.6	20.5	20.5	17.7	17.7	17.7	17.7	20.5	11098000
4.9	11.7	11.7	4.9	0	4.9	4.9	11.7	642360
31.4	31.4	31.4	14.7	26.5	26.5	14.7	31.4	5698900
31.8	17.8	31.8	31.8	31.8	17.8	31.8	31.8	6030300
17.8	17.8	11.7	11.7	5	11.7	17.8	5	7585200
8.7	20.9	8.7	20.9	15.7	20.9	20.9	39.1	3366100
0	0	0	11.7	11.7	24.2	24.2	24.2	1281900
9.3	9.3	32.6	22.1	32.6	36	34.9	45.3	7196300
24.4	24.4	24.4	24.4	24.4	28	19.6	12.5	7550300
6.1	3.9	1.7	2.2	1.7	6.1	6.1	6.1	2001500
4	1.9	1.9	1.9	1.9	4.3	6.4	6.4	2428500
5.6	5	7.3	7.3	3.4	7.3	9.8	8.1	3399700
0	3	3	0	0	3	3	3	525810
0	4.1	4.1	4.1	4.1	4.1	4.1	0	642280
36.5	36.5	36.5	36.5	36.5	27.7	27.7	36.5	13327000
18.5	18.5	18.5	18.5	18.5	18.5	18.5	18.5	1248900
0	8	8	8	16	16	16	16	1074800
15.1	15.1	15.1	15.1	15.1	24.4	24.4	24.4	4891100
10.9	10.9	0	0	10.9	20.2	20.2	20.2	1114000
0	9.3	9.3	9.3	26.9	36.1	26.9	18.5	6075200
50	50	32.5	32.5	32.5	50	50	50	11469000
21.9	13	13	17.2	0	21.9	30.2	26	3136700
0.4	0.8	0.8	0.4	0.4	0.4	0	0.4	1269000
19.2	19.2	25.3	19.2	25.3	19.2	19.2	19.2	2660100
0	0	31	31	31	31	31	31	8713900
0	0	0	0	0	0	13.5	0	224980
27.6	31.6	30.8	28.2	28.8	26.7	28.7	28.3	97953000
15.3	15.3	15.3	15.3	13.6	15.3	15.3	11.6	7378700
12.2	9.3	8.2	8.2	5.4	5.4	12.2	12.2	7937000
1.8	2.2	1.8	4	0	4	4	4	1196900
7.3	7.3	9.5	7.3	4.2	7.3	7.3	7.3	26247000
8.7	8.7	8.7	8.7	9.3	8.7	8.7	6.6	5033800
4.7	4.7	4.7	4.7	4.7	4.7	0	4.7	1279800
0	3.4	3.4	3.4	3.4	3.4	1	1	1054600

10.4	10.4	10.4	10.4	10.4	10.4	10.4	10.4	18494000
4.6	4.6	4.6	4.6	4.6	4.6	4.6	4.6	1268600
12.3	11.2	12.3	20.7	12.9	19.3	34.2	26.1	5043200
14.8	42.6	14.8	22.6	14.8	22.6	22.6	22.6	5809000
3.4	2.3	2.3	3.4	0	0	3.4	0	711940
0	0	8.1	8.1	7.6	8.1	0	15.7	2779300
36.2	10.8	10.8	10.8	0	36.2	36.2	36.2	1576700
16.2	24.9	22.3	19.8	19.8	22.3	20.4	20.7	8787100
0	7.9	12.1	12.1	12.1	12.1	12.1	12.1	1105800
3.7	4.4	10.8	11.6	11.2	13	14.2	13.8	21800000
1.6	2.4	7.5	5.5	6.1	8.3	8.7	8.2	10197000
13.5	20.9	20.9	15.3	15.3	20.9	20.9	20.9	4907100
0	2	1.6	3.6	0	0	0	0	409550
19.3	19.3	19.3	19.3	11.1	19.3	11.1	11.1	1369600
11.2	14	3.7	5.6	0	16.8	26.9	24.1	15727000
4.7	1.8	4.7	4.7	4.7	7.3	7.3	7.1	2718900
0	0	3.9	1	0	5.5	3.9	3.9	1090400
0	0	12.4	6.6	10.6	12.4	8.4	8.2	2468300
3.6	7.9	7.8	9.7	9	8.9	9.7	7.8	9192600
1	4	7.8	7.8	2.7	10.6	9.3	10.6	5121700
6.5	5.2	5.2	6.1	5.9	6.4	7.3	7.1	6852900
9.6	20.8	20.8	20.8	0	20.8	20.8	20.8	2087100
20	20	12.5	12.5	7.1	12.5	20	16.7	6694900
23.1	22.5	22.5	17.3	16.3	22.5	22.5	17.9	15723000
14	7.9	16.8	13.4	14.3	22.5	23.2	23.8	36402000
15	15	15	15	10.1	10.1	15	4.8	2119600
17.8	28	18.8	14.1	9.8	27.3	28	28	20582000
0	0	0	0	0	0	0	3.1	105210
26.1	41.3	26.1	41.3	0	26.1	15.2	15.2	4613000
26.4	24.9	28.9	29.8	31.6	30.7	34.7	33.8	17176000
17.5	14.9	12.6	12.6	15.5	22.5	20.9	19.5	19742000
0	0	0	8.1	18.1	31.5	31.5	26.2	2967100
0	0	0	0	0	6	6	6	2240600
0.8	1.4	0.6	0	0	1.4	1.4	1.4	643940
2.3	2.3	2.3	2.3	2.3	2.3	2.3	0	45841000
20.4	20.4	15.5	22.3	15.5	15.5	22.3	22.3	7701000
29	29	25.8	32.6	24.2	32.6	17.4	29	14790000
1	1	1	1	1	1	1	1	8113400
49.2	54.8	54.8	52.4	44.9	52.1	46.5	45.2	2.48E+08
21.9	20.1	24.9	23.4	21.9	30.8	32.8	29.7	30948000
9	14.9	14.9	9	9	9	9	9	1084400
2.5	2.5	2.5	2.5	2.5	2.5	2.5	0	464440
2.8	2.8	2.8	2.8	1.9	3.7	3.7	5.4	3006900
1	0	2.1	2.1	2.6	4.8	3.2	2	1347700
0.8	0	1.7	1.7	1.7	1.7	3.8	3	1200200
6	4.4	3.4	2.6	4.2	3.4	5.1	5.1	7979800
21.4	21.4	16.7	16.7	19.6	21.4	19.6	19.6	11250000
11.6	9	9	11.6	14.2	9	4.6	4.6	7105200
9.3	9.5	11.6	11.6	9.3	9.3	9.3	4.8	6786600
13	13	13	13	13	13	4.9	2.3	3885800

10.2	15.4	15.4	13.3	15.4	15.4	10.2	10.2	8080300
12.5	8.5	8.5	12.5	12.5	8.9	8.5	8.9	3318700
33.8	33.8	33.8	22.9	16.2	27.1	28	27.6	19950000
0	0	0	0	0	0	10.7	10.7	513210
40	14.3	14.3	14.3	40	40	41.4	41.4	3383600
2.8	2.8	2.8	2.8	2.8	2.8	0	0	651920
1.3	1.3	1.3	1.3	1.3	1.3	2	1.3	2045300
9.1	9.1	9.1	16.9	9.1	21.4	21.4	21.4	5426600
24.4	32.4	24.4	24.4	20.5	24.4	32.4	32.4	21568000
8.7	4.5	13.2	4.2	8.7	16.2	16.2	16.2	2125800
0	0	0	0	0	0	6.3	0	0
8.8	2.9	14	14	15.7	11.7	8.8	8.8	4002800
5.6	5.6	5.6	5.6	5.6	5.6	0	0	946360
15.1	5.4	5.4	15.1	5.4	5.4	5.4	5.4	5382800
6.6	9.7	6.6	6.6	12.7	6.6	3	6.6	2909300
0	8.8	0	8.8	0	8.8	0	13	482490
2.6	1.2	4.3	5.5	4.3	2.9	3.8	0	1465000
6.1	7.6	9	7.7	6.7	9	8.9	8.9	8247300
4.4	0	4.4	4.4	7.2	4.4	4.4	4.4	625410
8.5	0	8.5	8.5	8.5	0	8.5	15.8	1112100
0	0	0	0	1.9	3.6	1.7	1.9	557620
17.6	13.7	29	31.6	24.7	37.9	37.9	29	10919000
0	4.9	5.5	10.4	5.5	10.4	10.4	4.9	725840
10.4	10.4	10.4	10.4	10.4	10.4	10.4	0	2328200
1.5	1.5	1.5	1.5	1.5	1.5	1.5	1.5	8168200
0	0	0	0	0	0	7.8	7.8	210490
19.7	19.7	19.7	19.7	19.7	7	0	19.7	2325300
5.5	11.6	11.6	11.6	5.5	11.6	5.5	6.1	1138400
16.3	16.3	8.5	16.3	16.3	16.3	16.3	16.3	2450600
0	5.1	5.1	4.3	0	5.1	0	5.1	366260
11.7	11.7	11.7	11.7	11.7	11.7	11.7	11.7	2264900
51	58.7	55.4	48.7	48.3	55	51	44	41350000
50.8	58.6	55.2	48.5	42.4	54.9	50.8	43.8	1887200
1.8	1.8	2.8	1.8	1.9	4.1	4.2	4	5061300
5.1	5.1	5.1	5.1	2.2	5.1	2.9	2.9	2112700
14.3	17.1	12.3	7.2	14.3	17.1	17.1	15.2	7920000
1.5	3.9	18.8	13.1	18.8	21.4	18.8	15.6	20429000
11.2	11.2	11.2	11.2	9.8	11.2	11.2	8.7	7644500
13.7	13.7	13.7	13.7	13.7	13.7	13.7	13.7	2725700
0	0	5.2	2.2	2.2	2.2	2.2	3	541790
3.6	0	0	0	0	0	3.6	3.6	179360
27.9	24.3	31.1	27.5	33.1	33.3	40.1	40.1	22465000
22.6	17.9	14.2	18.3	9.2	22.6	36.1	32.3	19911000
24.2	27.7	18.9	19.1	18.1	29.3	34.2	35.6	25884000
20.8	23.3	16.7	10.1	10.5	30.7	30.7	33.5	13292000
14.8	12.6	14.2	16.6	16	15.6	19.4	20.2	21430000
25.9	23.1	15.8	21.8	21.7	31.6	36.1	36.1	21240000
26.1	17.9	25.7	27.6	27.6	33.8	36.5	34.5	29540000
5.3	10.7	12.4	8.5	7.5	17.3	18.6	20.5	16836000
0.5	0	1.8	1.8	1.8	0.5	1.2	1.8	1715200

0.8	2.1	2.1	0.8	0.8	0.8	2.1	1.2	668590
0	2.2	2.2	2.2	2.9	5.1	5.1	5.1	652980
9.6	14	5.1	9.6	14	9.6	14	4.5	12337000
27.6	27.6	12.4	12.4	12.4	12.4	12.4	12.4	13714000
35.8	35.8	35.8	35.8	26.1	35.8	21.8	35.8	13403000
0	0	0	6.3	6.3	0	0	15.7	1736700
5.4	5.4	5.4	5.4	0	5.4	5.4	5.4	1614700
10.3	10.3	10.3	10.3	10.3	10.3	10.3	10.3	2205900
1.6	0	0	0	0	0	1.6	1.5	375510
17.1	21	29.5	21	27.6	31.4	51.4	51.4	1.03E+08
5.2	14	14	14	14	14	8.7	8.7	1349200
11.4	11.4	18.7	18.7	18.7	18.7	26.8	26.8	4772900
25.7	21.1	23.6	18.5	23.6	21.1	21.1	18.5	23924000
3.1	3.1	5	1.9	0	11.4	19.3	19.3	4035000
39.1	39.1	34	34	30.7	29.8	34.9	29.8	21287000
19.4	30.4	32.9	32.9	30.4	30.4	30.4	29.8	6999400
7.9	13.9	13.9	11.1	10.7	13.9	7.5	10.4	8634400
25.2	34.1	29.8	25.2	16.7	24.4	24.4	26.4	7633500
8.1	8.1	8.1	4	0	8.1	4	4	1960700
0.9	0.9	0.9	0.9	0	0.9	0	0.9	526630
0	0	12.1	12.1	16.3	30.4	29.2	30.4	6781900
0	0	0	0	0	0	5.4	0	113830
12.2	12.2	13.4	13.6	11.1	24.3	24.3	26.8	19495000
10.5	11.7	11.8	12.7	10.2	11.8	10.3	12.7	15696000
0	0	0	0	0	0	10.3	10.3	207590
6.7	6.7	0	0	0	0	0	0	226370
0	0	31.8	31.8	31.8	31.8	31.8	25	2677200
4.5	6.8	10.3	8.1	12.4	5.9	7.2	8.2	9129900
35.1	37.8	20.8	17.4	20.8	37.8	46	40.5	16145000
6	7.3	8.4	7.5	7	9	9	9.5	6271700
0	0	0	6.2	6.2	6.2	6.2	0	271200
20.3	18.1	18.1	7.7	12.4	14.9	15.3	18.1	11056000
11.2	11.2	11.2	11.2	11.2	10.2	10.2	11.2	11930000
0	0	0	0	0	0	0	0	866690
7.5	5.3	7.5	10.2	10.2	7.9	2.4	7.7	2956600
17.5	0	17.5	17.5	17.5	17.5	0	0	495240
11.5	11.5	11.5	11.5	14	11.5	14	10.9	8752000
0	0	3.2	3.2	3.2	3.2	0	0	411200
6.8	3.3	10.7	13.1	7.1	19.9	23.4	20.8	9925100
0.7	0.7	0.7	0.7	0.7	0.7	0	0.7	814540
0	0	3.3	11	3.3	20.9	20.9	16.9	1983600
38.2	38.2	38.2	38.2	38.2	48.3	38.2	48.3	4268700
17.7	17.7	17.7	17.7	11.5	28.3	28.3	22.1	3913500
0	0	0	0	0	1.2	2.2	1.2	222780
14.6	14.6	14.6	14.6	8.5	14.6	14.6	10.6	6274600
0	0	0	0	0	17.3	17.3	17.3	1230200
11	4.4	8.9	8.6	8.4	15.9	22.4	20.4	9190400
3.3	3.3	4	7.3	7.3	7.3	0	3.3	1470500
4.2	4.2	4.2	4.2	4.2	4.2	4.2	4.2	709060
5.4	5.4	3.2	8.3	2.2	5.4	5.4	5.4	1056500

12.6	15.5	12.1	12.1	8.4	12.6	12.6	10.6	6015700
31.1	32.4	30.4	30.4	22.6	31.7	34.2	34.2	1.03E+08
12.4	12.4	12.4	12.4	12.4	12.4	12.4	12.4	2042800
10.8	16.3	16.3	16.3	16.3	12.8	12.5	9	6859900
4.2	4.2	4.2	2	2	2	2	2	1148500
23.8	23.8	23.8	16.6	16.6	23.8	23.8	23.8	4115900
42.7	46.7	46.7	50.8	39.7	50.8	61.8	59.3	60181000
11.7	11.7	11.7	11.7	11.7	11.7	11.7	0	1674800
25.6	24.7	28.7	27.5	25.2	28.5	31.4	30.9	44124000
19.9	27	19.3	26.6	20.5	34.2	35.5	36.8	72236000
2.4	3.4	1.9	2	1.8	2.4	3.3	3.6	32002000
12.8	12.8	12.8	12.8	12.8	6.7	12.8	12.8	3033100
11.2	23.4	24.5	18.1	14.4	29.8	33.5	33.5	18887000
8.7	11.1	8.7	3.8	0	7.3	11.1	6.2	1040500
10.6	21.2	10.6	21.2	10.6	21.2	21.2	10.6	2942000
7	7	7	7	3.6	7	7	7	3661700
7.5	7.5	15	7.5	7.5	7.5	7.5	15	2700200
7.2	7.2	4.2	4.2	0	0	5.7	4.2	2659100
24.3	24.3	24.3	13.3	24.3	24.3	24.3	24.3	7996400
8	8	12.6	8	12.6	8	8	8	8595300
5.8	3.9	5.8	5.8	3.7	5.8	5.8	3.9	3462700
15.2	15.2	12.4	8.6	12.4	11.5	13.3	13.3	16210000
6.6	6.6	6.6	6.6	3.7	6.6	3.7	6.6	5741600
11.4	11.4	8.3	8.3	8.3	11.4	11.4	4.5	4613500
7.1	7.1	7.1	7.1	3.8	7.1	3.3	3.3	2210400
0	5.7	5.7	5.7	5.7	5.7	5.7	5.7	1438500
15	15	15	15	15	15	7.2	15	4023700
7.8	7.8	7.8	7.8	7.8	7.8	7.8	7.8	4926500
21.3	17.1	13.3	9	17.5	17.1	8.1	8.1	4808800
9.6	9.6	9.6	9.6	4.4	9.6	5.2	5.2	2255600
21.3	21.3	14	14	21.3	21.3	21.3	11.9	7213800
5.9	5.9	5.9	5.9	5.9	5.9	5.9	4.3	2667400
9.1	9.1	9.1	9.1	9.1	9.1	9.1	9.1	13975000
13.3	21.3	13.3	15.7	7.7	15.7	15.7	13.6	4039100
0	0	9.7	4.1	9.7	0	0	5.6	602110
11.2	11.2	11.2	11.2	11.2	0	0	11.2	1406000
0	0	10.8	10.8	0	0	0	0	663550
5.6	5.6	7.8	7.8	6.2	4.2	1.4	2.2	1740300
68.3	68.3	68.3	0	0	0	0	0	688160
9.9	9.9	9.9	9.9	0	9.9	9.9	9.9	3334400
14	14	14	14	11.5	14	7	12	6518900
13.6	13.6	17.6	14.9	17.6	17.6	8.7	10.5	8197700
6.1	8.5	6.7	8.5	4.3	8.5	6.7	6.7	4386600
10.2	10.2	10.2	10.2	0	14	3.8	0	1280400
6	6	6	6	2.1	3.8	4.8	4.8	4319200
8.3	10	8.5	9.8	11.2	10.5	11.4	8.3	18188000
10	10	10	10	2.6	10	10	10	7038700
27.8	27.8	32.4	26.2	22	26.7	28.4	26.2	61511000
31.6	29.2	31.6	29.4	29.4	28.5	25.7	29.6	33107000
3.6	2.5	3.6	3.6	1.6	5.6	5.6	5.6	2437300

33.3	30.9	36.9	30.1	41.4	71.1	78.7	78.7	1.15E+08
2.6	2.6	2.6	2.6	2.6	4.7	4.7	2.6	1046400
12.5	12.5	12.5	12.5	12.5	8.8	8.8	3.8	1042500
28.4	25.7	21.6	20.2	17.3	22.8	25.8	23.6	13908000
8	7.8	4.8	4.8	8	11	7.8	7.8	1482700
8.5	14.5	8.7	10.9	6.5	16.2	10.5	13.1	4684000
12.2	12.2	0	5.4	12.2	9.4	7.1	15.3	3973500
26.4	29.6	20.8	21.8	23.6	23.6	29.9	29.9	3485600
46.4	43.1	44.8	49.6	46.4	41.5	48	49.2	61627000
46.8	40.3	41.9	44	44	39.9	47.2	40.7	7580600
40.1	37.7	39.1	37.7	37.7	37.3	43.7	41.9	28285000
4	0	6.5	4	4	15.8	18.1	17.4	2985800
47.8	50	52.9	41.1	43.8	50	59.9	55	10335000
44.1	46.1	48.8	40.8	40.4	46.1	55.2	50.8	5.55E+08
38.3	40.1	42.4	28.3	30.4	40.1	43.4	44.1	2666100
46	46	50.8	46	42	46	57.5	50.8	22343000
48.4	42	41.8	38.3	29.6	41.8	45.5	48.4	1.04E+08
42.7	46.5	42.5	31.9	22.2	31.9	40.4	43.1	2247300
15.7	15.7	14.1	13.7	12.2	15.6	15.7	15.7	1174500
44	47.9	43.8	43.8	32.1	43.8	47.9	47.9	4.25E+08
18.4	18.4	14.3	14.3	15.7	14.3	14.6	14.6	1569700
21.4	21.4	21.2	20.9	20.9	21.2	21.4	21.4	1.6E+08
21.5	21.5	21.5	0	0	21.5	21.5	21.5	1163000
0	0	9.5	9.5	20.2	41.7	41.7	20.2	1630900
7.3	7.3	1.9	1.9	1.9	7.3	8.9	5.4	1846900
0	0	0	0	0	0	1.4	1.4	140750
3.6	3.6	3.6	3.6	3.6	3.6	3.6	0	702320
6.2	6.2	3.2	3.2	0	3	6.2	6.2	917640
0	0	8.2	25.5	0	31	33.2	33.2	2648800
0	4.5	23.4	23.4	8.6	30.6	30.6	30.6	4837200
18.8	18.8	18.8	18.8	14	22.8	22.8	22.8	3260400
1.1	0	1.1	1.1	1.1	1.1	1.1	1.1	558040
27.2	27.2	27.2	27.2	27.2	27.2	27.2	27.2	85846000
3.7	0	3.7	7.4	7.4	7.4	7.4	7.4	1195000
0	0	7.5	7.5	7.5	7.5	7.5	7.5	1004700
14.5	14.5	14.5	14.5	14.5	14.5	14.5	14.5	4078400
8	6	9.3	7.6	10.6	9.2	3.4	5.4	6911200
13.4	10.6	13.4	13.4	10.4	10.8	7.7	10.8	3185400
14.3	12	12	16.3	14	21.1	18.2	21.1	7302800
6.1	6.1	4	4.2	4.2	6.1	6.1	6.1	1927500
12.7	12.7	12.7	6.5	3.4	12.7	12.7	12.7	2688100
8.5	6.2	10.3	12.6	10.3	14.6	13.5	17.6	4762400
0	5.6	0	0	0	0	14.8	14.8	244760
0	0	6.7	16.7	10	16.7	16.7	16.7	4610700
0	9.8	16.5	9.8	0	16.5	9.8	21.2	2379200
0	3.6	0	0	4.9	4.9	8.4	8.4	1456500
3.1	3.1	1.9	3.1	1.9	1.9	3.9	4.1	1871900
15.2	15.2	8.2	15.2	15.2	8.2	15.2	0	2190600
0	4.7	4.7	4.7	4.7	0	0	0	857370
0	3.5	5.8	8.5	10	3.6	5.8	8.5	1899200

5.2	5.2	2	2	2	5.2	5.2	0	1961000
4.4	4.4	4.4	4.4	2.5	4.4	4.4	4.4	1271900
5.7	8	8	5.9	5.9	5.9	5.9	2.3	2129600
3.1	3.1	3.1	3.1	0	3.1	3.1	3.1	544140
0	0	3.1	5.5	2.5	5.5	0	5.5	808780
6.6	7	5.4	6	3.9	7.5	8.6	8.1	13276000
11.8	3.4	11.8	11.8	3.4	11.8	14.6	14.6	2737500
0	0	0	20	20	20	20	20	734910
0	0	0	0	0	0	23.9	23.9	209730
8.6	8.6	2.9	5.9	5.9	14.1	14.1	11.2	6611400
20.6	0	20.6	20.6	0	20.6	0	20.6	832740
10.9	10.9	0	0	0	10.9	0	10.9	405270
0	0	1.6	0	0	3	1.6	3	492230
12.6	12.6	22.6	31.3	31.3	35.7	35.7	35.7	5448500
0	2.9	4.2	2.5	0	18.1	25	21.4	4590500
11	0	5	5	0	16.1	16.1	16.1	944440
0	0	8.1	3.9	3.9	8.1	8.1	3.9	540820
14.9	14.9	22.4	14.9	18.7	22.4	22.4	22.4	3700100
50.6	50.6	50.6	50.6	50.6	50.6	50.6	50.6	2.98E+08
1.4	0	1.4	3.7	5.1	3.7	6.5	3.7	1572100
15.7	15.7	15.7	0	0	0	27.9	27.9	1831900
0	0	7.5	7.5	0	12.5	21.5	21.5	1213500
0	11.7	11.7	11.7	0	11.7	11.7	11.7	1519100
13.8	13.8	6.6	19.7	19.7	19.7	26.3	32.9	5564200
2.7	2.7	7.8	7.8	7.8	10.5	7.8	7.8	4076000
6.9	6.9	13.8	13.8	13.8	20.7	20.7	20.7	2808400
18.5	18.5	18.5	18.5	0	18.5	18.5	18.5	1278400
12.6	16.4	18.4	17.6	14.3	24.3	28.7	25.1	33297000
0	12.3	24.7	0	12.3	24.7	24.7	12.3	773020
5	5	5	5	0	5	0	5	1008900
13.6	13.6	13.6	13.6	13.6	13.6	13.6	10.8	8671200
22.5	24.9	14.2	16.8	16.8	21.9	21.9	29.4	11713000
8.5	8.5	9.3	8.5	7.9	7.5	6.3	5.1	12382000
7	9	6.8	4.7	4.7	4.5	6.8	4.3	1737200
0	4.1	0	4.1	4.1	10.2	27	26	2436900
0	3.3	3.3	3.3	0	3.3	3.3	3.3	957720
3.8	3.8	3.8	3.8	3.8	3.8	3.8	3.8	544210
17.2	17.2	17.2	17.2	10.9	17.2	17.2	17.2	3593300
17.6	16.4	14.4	15.4	9.2	13.7	16.4	16.4	16734000
13.9	13.9	13	11.8	10.3	13.1	14	14	15580000
2.1	2.1	0	0	0	3.4	3.4	3.4	1055400
2.4	2.4	2.4	2.4	2.4	1.2	1.2	1.2	1657200
0	7	0	7	0	0	7	0	108890
0	12.4	0	12.4	0	0	12.4	12.4	828440
6.9	6.9	6.9	6.9	6.9	6.9	6.9	6.9	1344600
43.1	43.1	43.1	43.1	25.9	43.1	25.9	43.1	11364000
11.9	2.6	0	0	0	2.6	11.9	14.2	1905700
7.6	15.3	0	7.6	0	16.7	24.3	16.7	906390
6.4	6.4	6.4	6.4	6.4	6.4	6.4	6.4	12812000
0	0	0	0	0	8.3	8.3	8.3	359490

5.8	5.8	5.8	5.8	5.8	5.8	5.8	5.8	12997000
0	0	0	2.4	2.4	4.7	8.3	5.9	1166800
13.2	11.2	13.7	17.6	13.7	19.8	23	23	14305000
11.9	11.9	11.9	11.9	11.9	11.9	0	0	1234100
5.2	14.4	14.4	14.4	14.4	14.4	8.9	8.9	2680900
0	71.4	71.4	71.4	71.4	71.4	71.4	71.4	1258600
10.1	7.2	10.1	11.8	4	11.8	8.9	8.9	6378100
5.6	5.6	5.6	5.6	2.9	5.6	2.7	0	916380
0	3.6	3.6	0	0	3.6	6.8	3.6	328290
5.2	3.7	3.6	3.6	2.6	6	6	6	3582300
3.1	3.1	3.1	3.1	3.1	3.1	0	3.1	402090
35.9	39.5	34.4	31.5	22.3	32.8	36.5	29	48113000
0	3.9	0	0	3.9	0	0	0	558160
19.3	19.3	15.2	13.5	10.8	15.2	15.2	11.3	12700000
4.3	6.9	6.9	4.3	2.5	6.9	12.3	6.9	8218500
7.1	7.1	7.1	7.1	0	7.1	7.1	10.4	3189900
9.3	9.3	9.3	9.3	9.3	9.3	9.3	0	2150200
52	52	52	52	52	45	36	40	12280000
23	33	33	33	33	33	10	10	6049100
12.4	12.4	12.4	12.4	12.4	12.4	7.6	12.4	4024900
25.5	22.6	22.6	22.6	25.5	22.6	17.7	25.5	7874000
1.6	1.6	1.6	0	1.6	0	1.6	0	400220
32.4	32.4	32.4	32.4	32.4	32.4	0	32.4	515920
20	20	20	20	20	20	20	20	5746300
15.7	7	9.6	9.6	6.5	9.6	3.1	3.1	5183300
5.3	7.4	4.5	3.3	5.6	6.8	8.2	8.2	6833100
5.3	4.5	6.6	10.5	11	17.7	18.2	20	24304000
9.3	9.3	4.6	4.6	4.6	4.6	0	0	3035200
5.7	5.7	5.7	5.7	5.7	5.7	5.7	5.7	1269400
68.6	68.6	68.6	55.1	51.6	71	71	71	1.55E+08
70.4	70.4	66	70.4	60.9	65.6	55.8	63.3	1.65E+08
28.3	30	23	25.8	17.3	31.1	30	23.3	30677000
3.3	3.3	3.3	3.3	2.2	3.3	2.2	2.2	1193900
11.6	11.6	11.6	11.6	0	11.6	0	11.6	4476800
7.7	11.8	13.9	13.9	11.3	11.8	11.8	13	5944700
16.2	16.2	16.2	16.2	16.2	16.2	0	16.2	926680
8.8	8.8	10.8	12.5	11.9	18.2	15.9	18.2	9391900
0	36.4	36.4	36.4	0	36.4	36.4	0	564550
2.8	6.1	6.1	6.1	6.1	11.7	11.7	2.8	3611900
4	14.6	5.8	14.6	5.8	14.6	15.9	20.8	5277000
9.3	22	33.9	33.9	33.9	22	22	22	4608200
14.7	14.7	17.2	17.2	14.7	17.2	14.7	17.2	6014600
12.7	15	5	16.2	13.5	22.4	20.8	20.8	6904700
12.4	8.9	8.6	8.6	8.6	5.1	14.5	14.5	2932200
1	2.6	3.9	3.9	2.9	1	1	2.3	2065900
6.6	6.6	6.6	6.6	6.6	6.6	4	2.4	4438300
8.2	8.2	8.2	8	11.6	4.6	5.2	8.2	2151500
0	4.5	4.5	4.5	4.5	4.5	4.5	4.5	815080
0	0	0	0	0	0	33.3	33.3	199250
7.6	7.6	4.3	4.3	4.3	4.3	4.3	4.3	635030

1.1	1.1	1.1	1.1	1.1	2.2	0	0	412680
0	3.6	3.6	0	0	0	3.6	0	272920
23.4	24.5	16.8	11.2	11.1	25.4	29.8	28	23172000
11.8	13.5	15.4	19	16.7	21.2	25.8	25.1	23887000
37.6	20.5	43.9	41.3	43.9	43.9	43.9	41.3	8266800
0	0	0	0	0	0	4.3	4.3	354660
2.3	2.3	6	6	2.3	6	6	6	720470
0	0	0	0	13	13	13	13	519510
5.4	6	5.4	5.4	5.4	7.2	5.4	4.2	2971000
8.7	2.9	8.7	8.7	8.7	8.7	2.9	2.9	1246100
0	0	0	0	0	1.5	3.1	3.1	452250
4.3	2.4	4.3	1.9	1	4.3	3.3	2.9	915800
28.7	28.7	28.7	28.7	28.7	28.7	28.7	17.2	1977900
5.8	5.8	5.8	5.8	0	5.8	5.8	5.8	2286700
12.9	12.9	12.9	12.9	12.9	12.9	12.9	12.9	2337800
0	4	4	8	8	4	0	0	498690
0	0	3.5	9.8	9.8	9.8	9.8	9.8	1312000

Intensity 1_2	Intensity 2	Intensity 3	Intensity 4	Intensity 5	Intensity 6	Intensity D1	Intensity D2
11453000	15987000	16064000	14708000	18461000	9468700	6618700	8236300
1037900	2007600	972010	516540	816860	996550	1119100	1959600
71960	61352	115180	119180	105780	250870	275430	429240
1964000	3060000	3334600	3551500	3692400	6411200	6689600	6361700
2121400	2504200	5742300	6516700	4444400	17366000	15051000	19650000
1011000	1474800	1626300	1695300	1944800	3429100	3212100	3686400
1198600	819200	2621500	3374900	3967100	5592800	5719500	5687900
9021700	13029000	14640000	14338000	19521000	42662000	54546000	75320000
1582000	1778400	2881100	3302200	3144100	6731700	6609900	8330100
3488900	5301300	7167100	7997900	9486200	24082000	20737000	27581000
35520	0	41730	39974	36716	104350	108500	190070
240790	295520	399380	379220	379900	205070	98885	0
184270	137510	0	0	288920	0	0	0
1264600	3214100	2988600	2580500	2151100	1810800	1025400	1483600
231740	108640	0	0	62457	150910	197990	300870
1115100	1037600	516330	386050	241510	1162200	1271000	3288900
558380	718000	206110	552630	211020	1072400	1228800	1650400
829270	643080	512420	440690	435540	761490	997540	1024100
554330	511460	87554	92823	315430	435210	168600	597130
1068900	982250	536380	948680	658460	1828700	1906200	3193900
536820	788350	229930	588940	204710	548440	1187700	1262700
212160	335250	71445	137840	194340	464130	468690	1247000
92075	81590	0	73839	0	263360	249000	275210
541750	426780	302250	294400	352660	1300000	2017700	2049500
140190	307810	97277	123300	82380	640750	1143300	1178800
214490	157250	234470	228460	307920	787300	1216900	1405500
88417	97999	73410	49820	0	150710	163240	211990
905180	1821700	684260	672290	477950	1364400	2017600	2769500
498980	529920	454110	399970	219870	834550	687340	935610
0	0	0	52321	0	148940	0	243460
0	0	0	0	26280	22255	263810	347110
29951	202190	84092	114100	153140	518300	345520	380670
67134	48662	54226	63477	118110	154460	97227	499140
0	0	63193	39069	269710	808050	1176200	1224700
251420	233500	141980	61416	135700	97706	94870	122790
405870	325000	345360	304170	249770	251100	163000	192250
635220	555320	754630	725070	448300	366070	204720	465980
510770	1441100	1048900	935010	1165500	683950	459690	447730
394350	514550	387770	274540	156580	239660	204030	232230
71777	121510	31911	0	40196	407750	32679	0
440570	432540	370610	301990	220500	191950	156330	91535
112190	0	236070	151970	36003	129940	71060	101260
533900	807100	248310	93160	477370	377440	175520	175280
339510	303710	279940	292570	133370	119800	0	61192
125910	0	139770	231860	212780	32263	16462	0
159660	127070	202860	223720	277420	176420	49725	0
135040	151490	135110	163920	118890	81133	60809	68655
114080	156080	129120	196740	0	141210	179150	74845

599000	683740	644530	641580	265370	528000	189890	94034
44035	277170	135200	120170	0	166600	83053	146550
2691200	4884200	3498300	2901600	3008400	2224500	1580700	1616500
97132	148040	143600	161280	231870	83551	58615	46647
217760	309730	402500	510220	248360	249920	83995	334130
476600	485530	536820	468540	565320	330690	447570	585190
255460	216030	110670	60645	181500	91040	0	0
128280	157360	315850	324490	258070	198730	0	97007
258080	0	668330	203020	554850	181360	80143	346280
223680	190130	181280	174510	75577	120880	57769	45131
209310	186620	167000	119100	417580	0	0	0
133660	173970	88412	103200	157660	85592	83774	102140
359150	82589	339500	273490	119700	146250	98205	142480
0	197730	0	245900	0	0	0	0
113820	97851	117320	119950	233660	73723	57624	0
292820	275510	464410	441770	439040	255770	76422	59692
107150	94951	90956	83661	144200	74491	0	0
154920	189800	110420	95729	119040	81271	73469	89115
175330	262340	304290	79256	0	189990	73188	0
94318	111910	136470	125350	419170	95551	52469	58018
81529	127270	116130	85387	0	112120	70671	112300
2389300	6688900	2935700	1335000	796670	1587000	1547700	2022600
2999300	3958700	4893200	6503200	7352600	2984500	1882100	1945800
268240	317720	372510	357810	476520	79756	103840	174050
291300	350010	283800	255840	107860	200950	0	156880
142760	0	235890	284670	206860	498600	506140	799540
1325000	75778	46652	402350	0	855540	249840	2895400
4907400	6031400	3695500	3167500	2765300	3356900	4304300	8512800
3068700	4088600	2266600	1702000	1246900	3891100	5941000	6767300
1245000	2888100	1572200	1455100	522000	2272100	4097700	3999200
5613400	7126700	6061000	5270400	4440100	5873000	5118100	6563400
3256800	3185800	1840100	1060900	420920	1770600	2317900	2350500
2548100	4029700	2035000	1455500	836420	2159600	3284200	4001800
4524000	3994400	2170000	1502200	1601800	3369800	4550900	1842000
4915600	6834400	3139600	2768800	1472700	4645600	6322100	6292300
2744600	1686100	1462900	2582500	458200	4338500	5278400	3694700
7285200	7566800	4923300	3672400	2143600	5690300	4619900	6430600
864670	1296200	822330	508320	790680	1108500	1207500	1602500
390390	605520	812690	479100	295300	1933000	1875500	1905300
1601900	2479900	890570	630050	283290	1128400	1693100	3757000
1369100	1523900	1029900	592830	879780	1129300	1687500	919190
1046700	1658200	910880	583030	457370	1979400	1737400	2339200
2485000	3039000	1463400	1306300	346020	1958000	2262000	1139000
3186200	4004700	2646700	1954100	2147100	2258200	2534200	3477900
394700	2942000	3018900	2518600	1198900	4408600	5050700	5214700
135630	196430	184180	145170	101260	405070	892960	0
12501000	15017000	9003900	6161400	4446600	9027300	8428900	10763000
146810	354120	472070	542970	242560	1268600	1393900	1400000
5137100	11865000	5382300	2874400	2179200	4172500	8197000	10670000
7238100	11813000	4029600	3945700	2151600	5109000	6635500	9515400

2964000	4560300	1946000	1543800	958440	2234500	2336200	4079100
4436000	10064000	5010900	6909700	4501600	5140700	4641300	9195000
915760	4036900	1226000	1101700	488020	1447400	6795900	1962400
11761000	14846000	11042000	9450100	7748300	8169300	9510400	10233000
5930100	11339000	6437400	4620300	1877500	6601000	7339500	8619200
4573700	5762600	4223800	3181100	3358600	7760000	8370200	19618000
33902000	48372000	46388000	38776000	30505000	28740000	27505000	53493000
165920	261010	133310	113100	121890	242570	326070	0
162640	50036	188690	46191	60089	57639	47822	105450
66668000	90429000	85830000	75586000	83420000	49494000	31164000	50262000
3190900	8136900	7152100	6253600	2534400	10172000	9953500	12299000
50330	5348900	2054500	1137900	0	3796900	4908000	2816000
4039400	7382800	7325300	7507000	3004000	7009600	6818400	24522000
9383500	9733100	6754500	3029000	5214100	4335200	5446700	4735900
2661000	4631000	3935800	2729700	2102100	3331200	4110500	5262800
4772200	6451700	4978700	5419400	2500300	3194400	3727100	3718100
92545	91331	68958	67444	71849	37349	64580	0
4437900	5350600	7312700	6617800	8865300	8359000	8035500	11765000
3533200	10331000	4978100	3509300	3933400	4554700	5004800	6773300
2817200	4934500	3065500	2529100	2099700	2892000	3587800	3342600
3895300	4625200	3624700	2651800	3748500	2480700	2870700	3400300
5590800	7318200	4763000	3251700	2781800	3957500	4512800	6681500
2687500	4324500	844350	603410	566820	564420	3068500	5207900
5237900	6795700	6477100	6233800	4851700	4073000	3981100	4892100
5006700	4941400	4711600	3774600	3914200	3483700	3374600	4098600
1613000	1551300	716870	130420	217990	409440	2197900	1904400
2471300	3218400	4047300	3748800	3313700	4151800	3389500	3917300
5246200	4158900	3571000	3880800	2149400	3289000	2762100	9120900
1293400	2301100	2193600	2107500	2242600	2928600	3324300	4475300
3695000	4831800	3577200	2794900	1890200	1671300	3724800	2170100
1549500	6339200	4987600	2117800	1965100	3407500	4658400	5919500
2875900	3453900	2078900	2107600	1209700	1922600	3321700	3322000
3294100	3484200	2562800	2624200	302850	3153300	4027100	5962600
3647300	5362300	2623700	2065800	2316900	2424500	3163400	3672000
0	897910	740180	629860	584910	1046600	1261200	2081100
11120000	13340000	9021600	6417700	7128600	6150700	9157600	10366000
3320800	6412200	3486400	3026400	3199400	3102000	5148300	5584300
2126300	2458600	2253200	1571200	2581200	1582300	1960000	2683600
2362800	3500200	2616000	2108900	1402700	2337800	3035600	3398000
3754600	5262500	4048500	3847800	1021300	2869400	2942000	4698800
843150	1131100	1018000	902970	102540	1235700	1548900	3506100
2563800	3747400	1846000	1672200	2078700	2672800	2301100	2708900
2918600	3126800	2901300	2427300	1040600	3208600	2846500	3496400
1544700	2094300	1511500	1394900	1689600	1861100	2352400	3043800
543940	1417500	1419200	824290	774630	781590	1596200	3153600
199590	184160	280160	252370	367200	157150	248140	581290
10642000	14587000	14231000	10265000	6949800	9962400	9680900	15263000
3515800	5955500	4561100	3230700	1773800	5228000	7526400	8446000
6032200	7728500	6377900	3759700	3043100	6238000	6902700	7505700
5237300	7654000	5167800	3989200	3379800	4407500	5950100	6932400

8008300	12235000	8384200	6843700	5376800	7213500	8714500	10790000
164740	209590	183710	125800	151420	119780	96171	96959
2408900	5424900	5567500	3585000	5499000	4364200	3492000	6851000
2807400	2261900	1410200	1894800	1500600	1448100	1985200	2154500
0	29287	0	0	0	66931	96972	1441000
265560	553550	908020	646280	653490	4450900	5859600	8406600
0	0	0	91771	115420	247170	108440	115390
0	37376	44499	66643	33810	86726	62244	282310
43904000	56724000	59068000	55383000	64737000	43860000	31516000	36756000
2496900	4039800	3670300	4164100	2387900	2489200	1646800	1288300
570900	0	0	0	0	398210	225330	336040
98457	61530	154010	116040	162420	112620	140000	153020
0	224610	249670	0	248060	0	52638	0
44718	166850	125620	0	44810	519620	1791500	349760
1751500	1816800	2131900	2140900	2266700	1272600	723310	1037800
203680	191300	124850	0	163610	207460	158220	165360
64953	0	144270	85135	0	1727800	2086300	4395500
0	0	0	0	0	240120	1089400	1608200
1495400	1790800	1915400	1375800	2254800	1143200	666280	1430200
1262500	1037200	1686500	1993200	6187500	3263900	3290400	1504900
97604000	171190000	167840000	194380000	173230000	267030000	315300000	432430000
23912000	32973000	19961000	10805000	2870900	31860000	49814000	51247000
425600	386730	806690	693270	1198300	674060	930910	1624300
0	180430	196580	212650	375880	687420	969200	967680
220710	195510	226550	284750	273010	265360	803050	703110
43083	129700	140700	83448	121400	662980	830550	742360
0	0	0	0	0	0	357350	0
159130	222870	315980	347130	592150	602420	791320	1456300
190310	112280	232240	150170	207220	567720	213210	385500
0	26181	20802	29208	0	77622	81225	535780
499020	311880	952320	855850	681930	2069500	2477100	2612100
508810	763010	673980	735760	791610	2241700	992040	1267100
39202	120160	97473	393810	343770	1276400	1805800	2060900
0	0	0	0	234580	0	0	0
168670	353090	201020	105660	123050	312780	159540	55766
1750800	5602500	3379100	1503300	213870	2351400	2945500	1559900
1627600	1690300	1601500	1249300	711720	917200	569130	945020
1655600	1694600	1394100	1674600	1485100	778270	883880	958500
240550	312450	642540	777880	447940	3668200	4308100	4065300
159100	185440	171410	262100	292760	0	0	0
188490	167860	284620	326830	396400	129140	49341	49992
1880500	1470800	1860700	1398400	3149300	707240	466530	672890
120620	488370	657320	783520	113050	442360	320430	458470
123560	155170	153120	126180	142100	330410	310350	293470
243190	276510	127220	0	0	169860	0	114110
206570	475770	123310	0	164730	490770	537800	409190
209780	295470	208970	0	202220	455970	164590	222060
78360	0	0	0	0	93960	245950	334530
975920	1588900	1672900	2010100	781770	5843100	7787900	7546100
995890	571910	293390	343850	512740	535590	691330	1061400

140200	183450	301910	471670	552000	359540	192370	0
77529	126620	243420	202700	247520	790180	928990	1074200
0	0	0	0	27964	79536	173120	402930
0	0	175170	349690	0	1264300	1247700	1012300
515680	655400	2176000	2263200	1558300	5145600	4195000	7352500
1876000	4396800	1802400	1540800	1405100	1804600	1047200	1364900
0	0	0	42582	0	261750	173220	345610
18360000	29500000	22344000	20003000	12050000	21800000	18521000	18884000
2515400	3018200	2723000	2533700	1140100	1928000	1349900	1801600
242660	611560	548780	485420	336220	295930	252280	204020
1927700	2099100	1982100	1750600	683600	4915800	6466700	4833000
461190	519850	433090	437820	243130	610970	513460	757700
82182	73878	0	102780	214180	444800	99821	114770
400700	340330	468340	454030	133450	439910	431340	1080800
272280	465230	691750	572060	586410	357020	226090	303230
0	0	74536	60257	132290	227200	261360	322840
82506	319820	202300	153420	290420	184220	41586	230710
654530	624180	782590	702960	714480	359760	276140	342670
0	26403	39076	70326	270320	133900	107480	357160
0	0	0	0	0	0	0	0
150600	223800	239630	244930	0	117310	48129	87839
795140	924130	287340	322240	387240	770600	673810	781600
0	0	66556	152980	203420	391030	648470	381550
73868	89732	184350	263640	177870	451500	454920	957270
2220100	1846900	2321600	2351600	1964400	4815000	6034400	7079400
170800	318120	195670	327380	213980	195530	0	121880
2982100	2919800	1590200	1581800	1347500	11077000	12808000	14544000
0	371350	182470	140520	0	684770	1183300	2880500
104270	115090	19568	0	0	0	1062500	260340
84992	130120	201770	244100	166650	666960	1134900	1465400
2702400	2984400	3441700	3412200	2306600	2488700	1361700	1333900
214900	185280	178620	149940	0	0	0	0
0	0	0	113390	191510	260510	275540	314830
337160	116470	110210	99195	153270	254930	81787	0
1292000	1851000	3310100	3586300	2410400	10910000	14489000	16595000
388220	693910	1830100	2050400	1611200	4495700	5969600	6669900
363090	295120	550270	478000	123460	657160	382590	994330
154070	204840	0	0	181570	402930	397050	417680
0	0	123020	0	0	0	0	331240
6161600	6305400	19168000	22972000	25818000	71147000	68051000	87021000
169150	107890	162860	177530	80098	116070	0	60041
0	0	0	0	2523100	0	0	316010
260790	511320	652140	638230	586330	691700	465950	240000
48522	27454	0	32351	21389	48455	110660	104780
651720	866840	939790	810040	776080	532300	391700	453980
1021500	773780	279200	234060	283840	1246200	1254400	2163700
946320	680960	441800	579560	640300	971930	1146300	1348600
153020	42913	209930	284600	161090	688930	744730	739400
241960	315450	369900	246400	574180	193740	313900	556670
1235200	1508100	1917600	1608200	2019600	1663000	1549300	1379000

880780	858060	1306400	955270	1246000	975920	709630	887720
136000	0	171100	153600	231900	0	0	61077
33558	31762	26017	0	0	0	32265	156670
20730000	21308000	44932000	34002000	34107000	47783000	50851000	51379000
32642	52776	25876	0	0	75393	116090	316570
374150	624240	640580	585680	890270	680790	1014100	1165300
103720000	145980000	141540000	105010000	94805000	89728000	114260000	138060000
900220	615240	2262000	1836600	1714900	3173500	2578500	3335400
751020	1346800	2026000	1593000	1249800	2018200	1810200	2437800
1522900	1868500	3765900	3221100	2266200	4323400	4521400	5200700
733440	1546000	1099300	1444700	658700	1068900	1074000	1620500
3721600	4224800	3994700	2959000	2165300	2488300	2713900	5275000
263000	752480	1923900	1338400	2179200	534330	517740	1363800
148600	122910	192000	301930	265690	209770	137790	77690
118840	150700	405850	478980	360880	911550	933890	1130700
50232	0	78628	76886	129900	157880	190530	204460
0	112760	231740	229880	108590	75783	71475	224830
0	75226	44222	29702	0	0	1374600	1462400
101240	86170	319140	109470	223830	177280	189700	228420
0	50275	0	0	0	40549	0	159810
0	88053	0	0	0	79186	91342	523710
94565	57967	43777	54957	0	80086	87637	108550
867020	1032000	1424500	3488400	2687500	1172500	569120	1186200
49661	80183	61722	63369	0	55831	37819	55001
274690	474920	388800	243420	300380	240890	165230	257570
352140	0	196940	195620	433650	0	69240	0
0	0	26734	0	0	705740	758760	902820
74338	86498	239200	268140	417590	214840	268810	377820
2111400	2014000	3793200	2520000	2864000	2032500	778450	1341900
463430	679110	999920	939700	1280600	906710	657960	1115100
0	0	0	37393	47602	59884	68414	57466
560580	513300	183360	184930	68809	497520	957690	1193800
674990	1309500	667950	617010	333020	895290	700690	1327200
506830	570190	618510	588060	773030	1885200	1339200	2627100
0	0	58430	38278	55047	1005900	807820	588870
8756200	12726000	14710000	13337000	11516000	10047000	5834400	7732200
2450600	2053900	1008500	1739100	1120100	3479800	4125600	4363700
150540	173830	172460	0	26523	0	40747	259620
1538300	1932100	1746800	1487400	429360	972890	847370	621200
38819	53779	89915	74502	75156	294780	285630	300440
27143	0	71250	139920	134040	185070	97480	0
552840	475330	481450	738450	1079100	295470	236630	359800
796110	530800	0	148580	222650	248690	420730	577570
2678300	3332800	4312600	2647500	1623500	2798900	1388800	1436700
596570	1326000	891560	512890	113150	602520	514960	619540
41647000	46912000	36930000	45938000	49939000	23324000	13791000	16458000
39374000	42407000	48429000	47135000	56956000	26704000	18380000	34468000
3843100	3756600	4786100	3422400	2642400	2593100	1234800	1418100
1834700	2094000	2744200	2719000	3650400	1674500	817240	1192000
886220	821610	1031900	953230	1329400	553270	337040	302470

628710	1853900	1007900	284250	0	649590	1133700	0
1349200	1957900	1780800	1572400	182120	901080	611090	1198300
3810200	5853400	4924500	4161700	1855000	3073200	1748600	3021000
3505000	4894000	4416200	5813300	1435200	2710600	1951600	1996900
2929800	3020500	3116800	2974000	1211900	1746300	1138100	1204600
1146200	1306900	1431600	1021300	1055500	659850	415970	646760
58058	87522	95825	84706	44903	0	0	78561
242980	316850	247290	228700	210240	68620	79398	77990
108490	69757	69418	38239	141510	479380	533640	426720
219230	205810	0	64195	0	248020	273910	258170
502840	168840	83483	81946	0	73570	85616	181630
133290	100460	0	0	42288	69374	61955	74261
4045000	3936300	5907700	6917500	3071100	3856600	4168200	8689100
553160	581570	228840	247670	196370	362680	407220	405320
245180	108780	0	0	90399	228720	246070	300100
2572500	2333700	1548500	1462500	1040500	2777100	2979200	3567700
391220	500390	468730	230190	572280	192450	107340	222550
460070	332660	605400	602930	669960	142280	61459	0
72803	102890	54767	86291	0	492580	202110	464970
3691700	5575800	5552000	5809700	7018500	6831000	5609900	8632400
250230	376620	203780	251690	271770	1219400	1386800	1751900
989440	891320	2188100	2088100	1123300	1795000	1474400	1621500
43468	50710	0	34261	56003	106180	199630	209390
1353500	2464100	2683700	3272800	1607600	191430	148040	611610
156070	230380	98172	0	0	278150	276930	402450
2096900	3644100	1076000	1292800	1004500	2285100	2589000	2652000
0	0	0	0	0	0	103620	191340
273490	261130	431000	455800	712110	192920	202060	386040
163960	152340	187010	205240	284460	42239	39895	52266
0	0	63185	58250	117010	0	40697	0
41793	0	116840	240280	0	171750	65550	563580
111850	163320	239370	113250	466310	313580	412280	833400
55020	100020	0	0	0	49101	285490	51450
4034400	3062400	2597900	1204100	358250	2961000	1536400	6060200
0	313070	0	0	0	0	0	0
50699	25418	76510	71008	0	698540	479930	684620
0	0	19423	18692	114190	360020	283630	577980
2687900	3038400	4065600	2990000	3121000	2420100	1256200	1362000
1078900	1432600	2028500	1845600	2579500	1321800	519780	616540
217600	98344	0	94765	154800	0	48087	69049
395490	766540	1361500	987360	1441400	969490	571920	672110
355190	254960	842460	630380	710790	413990	310480	402640
1410800	1713600	1070000	681300	613820	425550	4226100	2312900
149820	151950	99379	102790	119060	96175	85959	114340
0	183550	0	0	0	0	94806	159060
137000	133440	222240	150070	152760	90524	154250	291040
0	0	90717	0	73552	597080	576920	400780
146370	270090	102560	63869	34765	270430	317350	209700
3420800	2480900	1738900	1393300	2596700	3854800	4614200	6167500
365610	162600	135800	0	132300	83840	65631	0

548290	590880	669030	719760	881890	2434400	2903100	3893900
140960	46571	98762	469500	75538	26263	0	91422
120230	0	159260	126190	220170	619390	680470	454900
198720	229220	314400	243870	251280	240420	192000	242180
206530	293600	421510	441430	277840	517460	669930	867180
0	0	0	0	0	0	33870	144360
29724	100950	48598	0	0	140420	143970	1073000
181470	544040	590330	568210	186290	417000	193010	196980
0	69320	0	0	0	0	802760	1525400
54487	0	0	51609	203300	407450	407880	435550
61162	54635	0	30785	0	32800	37090	0
40737	132530	81513	270460	133720	695450	950910	1009000
2168400	2148500	1375100	1605200	1061000	3062200	2975200	3859200
1641900	1766800	1941400	1469900	1849500	1084200	830150	874950
58838	82048	134160	48151	103810	37996	37008	76786
2091500	3165000	1632400	1328500	1188200	2219300	2346400	4162400
60282	0	505510	360120	493010	363560	162520	634340
918510	1057100	1174500	1171400	1305700	338580	273820	614400
399980	456810	562610	626120	254880	343940	203220	525360
728410	0	0	649340	463180	416830	390880	0
5475600	8154200	9628900	6588900	6362900	5083500	3063700	5426300
902350	2139000	1403800	1176200	282270	1039100	385150	985600
2334300	2815100	3324000	2964400	4786700	2025300	931460	1427900
261970	341650	471380	467340	558160	2103900	2408000	2647200
19673000	24267000	18285000	14890000	9180200	15508000	9857300	10713000
304370	372080	619590	565440	291510	1160400	2311100	1463800
148460	384500	567950	581580	408860	920920	1160200	2022300
331750	462360	258860	157020	52364	699280	1171200	1385500
0	0	0	126670	158040	516490	331190	861990
90308	64284	229120	324220	261760	856170	2472400	1830700
21309000	24695000	23838000	16875000	20725000	18690000	11119000	15664000
84756	0	672220	507720	571660	211690	107050	281010
599490	263590	627750	765720	943900	104590	199010	1044700
114130	96268	45011	24408	56439	99490	118340	196040
134680	213480	304450	382360	345120	774460	1212000	1221000
264940	319550	592350	902090	593100	2381600	2113800	2743800
102920	92532	703840	820440	860480	2089100	2073100	3534700
842570	519270	1059200	658450	1018800	384320	248890	565560
1569800	2142300	2229100	2054000	1965500	1362000	789230	988450
291910	287820	455980	229110	441170	264450	60259	403040
233020	371140	368760	246770	142100	182260	243020	850780
187410	100710	49378	0	0	0	0	310430
287210	412150	198500	203180	0	367770	444430	147900
253380	80501	53826	44860	57090	59315	217750	83321
99083	75933	0	32393	69044	101640	97909	0
598680	786450	1137100	1107400	1253300	736120	402680	733200
536970	847670	214180	39502	0	225760	1751500	2034700
984530	1229500	3603600	3844500	2642500	2831200	3337600	5973300
188190	342540	569720	806750	583300	747590	324430	632380
1876200	2421800	2048800	2245600	2254100	2095200	2720600	3322400

331320	345320	676890	524730	229170	587740	687380	770850
995690	1182900	1247800	740660	864500	648970	344060	795310
383370	360490	982700	990550	1089000	1147200	978330	1003400
6390600	10239000	8427200	7034000	5140900	6086800	6798100	5575800
44649	73186	305430	188530	241100	137190	94465	17314
125700	155590	313510	305900	108830	40277	0	26695
813110	609090	1111600	1057300	833550	582830	502770	879370
541650	728360	860020	685880	865080	404260	315690	313780
2753500	3334500	4621700	3931500	5751600	2949400	2765200	4129100
1230500	1755500	964100	1038500	885210	855170	986030	1218100
207030	133860	332830	339940	173380	478430	167330	483820
400580	341120	499910	346180	226280	229840	103240	459670
142810	273550	225270	281830	0	377820	365110	478900
0	116780	168060	268560	466400	79352	35586	54856
242300	302470	101460	29237	0	179500	216700	897320
134680	899990	1037900	830480	248900	1302100	1724900	1480800
76851	25005	0	77580	168060	186410	254380	463400
50655	53120	240670	367280	232300	245370	195960	426920
54979	96383	258230	417720	377780	1729000	2122300	2972900
0	0	0	0	0	0	0	0
83469	161940	173900	246920	462870	74332	44710	87214
355670	770740	523500	222330	0	44187	262430	139280
43916	134510	97551	74635	0	63774	52005	106120
531650	1040400	884760	686230	741220	880710	803810	1744800
711550	996980	1622300	2393800	887880	6184400	5412100	9100200
159840	330400	584310	505080	326510	347520	243040	259480
0	0	0	0	330660	0	71658	92079
80963	255880	166080	160140	0	81455	73522	0
310620	443760	149100	94173	0	221950	0	234480
23137	56165	266550	192400	362900	259030	215290	220090
684670	861420	2000900	1752200	3137300	1141500	2427800	2516600
203110	142660	110110	118580	355760	233430	262520	243620
4354700	6545600	7233500	6787600	6183000	4193800	1527800	1653000
7506900	6219300	6059200	5933000	5399800	10869000	17607000	22867000
416160	239410	609200	733430	1182900	873900	725470	772950
17877	32754	179310	159870	147420	287500	241700	205640
496490	584660	640070	316530	38236	228110	216230	133060
1303400	1994300	1359700	2145500	424360	2351600	1016000	1725100
254610	155620	519830	1038600	488000	258450	68800	468080
785300	771820	1299800	1303300	750560	736340	483520	701670
77274	400240	233240	128140	0	583140	661180	892340
378120	302620	136210	106920	154980	640580	425970	271980
0	87124	0	0	0	0	87876	112520
0	28406	32955	30399	32125	115580	131650	156010
504640	88057	493240	596860	610690	414870	314820	262300
210550	307840	319000	338530	286870	217250	120120	67761
0	61226	0	35473	0	0	83899	300580
712100	517750	1339500	1253000	568370	3787600	4421500	5514600
112040	74155	57117	61297	57503	0	0	0
781540	453510	725940	424970	403500	1534500	1147800	2321100

212270	230550	212790	241300	297550	486340	345920	189360
1762600	1189500	872110	782490	1610700	1366900	1842900	2314500
252650	310980	236930	208260	234980	500610	529630	1061200
221620	228360	49241	42605	36926	260000	432470	775380
173350	203750	163270	0	225500	381070	399110	994350
3200400	5097900	5377900	6580800	7317800	18932000	28878000	34679000
0	0	32105	0	137980	0	0	0
843760	1300100	639950	909020	1291900	494930	433060	866920
143900	153570	160650	135130	209740	114130	84914	59532
64746	109190	58477	37436	0	127620	119400	0
0	42340	0	65186	105950	0	59528	102100
790700	476730	648130	633600	624830	630920	416050	724560
194230	224780	115440	126150	65151	359150	626550	825670
1227900	2942600	3388100	2690900	3291000	1182800	1284300	1048700
10960000	8075500	10325000	8313700	8931000	7188800	4902600	6230800
0	0	0	0	0	0	0	187230
71631	119450	39817	111310	69994	320220	237030	646320
99452	28798	45376	25018	50910	195270	229570	101540
1593800	0	0	1214000	0	1187700	0	0
706420	1128600	540020	475380	572910	853470	815980	1319900
80397	0	68253	86177	61987	0	166800	152530
0	0	0	41479	0	84141	57566	331720
53645	60525	88287	117290	185790	150580	255650	161540
3008200	4586000	2927700	2399000	1779100	2729500	3631800	3854600
753860	927770	1412100	2725200	0	0	0	0
2589500	3241200	6686100	5973400	7309900	3918600	2173200	4935700
74325	174140	270160	296180	420970	100930	80887	0
98976	0	114390	169900	145020	403020	291400	279310
154920	227580	784250	724180	787600	1361500	1966400	1711400
19595	31772	55399	38051	66103	38684	0	79567
0	0	0	0	0	0	532890	166500
220840	244430	307640	338120	455260	224680	107860	218420
0	345020	99141	0	87352	355480	509470	277780
0	0	37167	114150	155130	119850	107310	613270
0	0	261780	0	0	1261000	916000	1617900
296030	330760	51564	0	144600	213440	278260	350720
1001900	1192300	517780	382230	492340	1150700	1005800	1375600
175870	238570	70849	249800	171870	48449	41086	145250
0	149050	0	0	0	108880	165310	165180
0	0	0	0	49501	92595	92224	81570
0	0	119990	217360	367230	1039800	981540	1112100
185030	373050	324940	284240	350680	263660	405770	583630
382930	401970	498220	315720	560560	657160	863770	991950
0	0	0	0	172920	206680	144260	212390
151850	75884	374020	446360	571610	1305000	1269400	1335400
76212	149080	62806	0	66507	0	73083	0
85901	175980	319320	566780	441060	1384600	1610200	2048400
174190	49058	183890	213510	342370	659010	653160	449430
73505	71895	82380	60227	63590	73641	70330	0
376610	304450	399200	481510	425060	261610	188160	171820

0	0	141810	0	135870	275070	270360	1888800
93706	81765	100530	75929	121450	60829	42122	71462
116630	139370	297650	267280	286390	0	0	0
1497200	1849600	501870	92721	151890	394530	887330	639240
6725600	7345300	6505500	4861800	6377600	4496500	3217400	6361700
3690700	4542600	4535300	3924000	2725800	3033500	2567000	3018100
538930	129370	1450100	885730	933550	441530	524760	317440
738080	936410	1050800	808000	372840	659920	444520	162730
526160	632280	776410	476410	60494	681800	304530	383550
353830	1339000	1675400	1389400	554310	388340	544170	2146200
129960	164830	53429	49965	32399	109690	204070	220170
769640	1137500	1015900	539350	1145800	717560	351590	651200
211840	144990	148380	62250	87435	0	0	0
152570	123650	99918	89208	110310	113700	121480	176030
2198000	2777000	3127000	3501900	2665300	1685700	842340	2121800
3742100	4170500	5233600	4331100	3113200	3524400	2211800	2438800
1308800	1729800	1907800	2261200	1067900	1405000	743920	1034600
1770700	2518500	2879900	3111900	3806900	1413800	935900	602960
978940	1040200	856220	382980	358360	798940	907830	425350
511190	623710	428010	460650	226170	291930	365920	198350
1296000	1330700	1737000	1437900	79138	1181600	739670	1494700
514700	519870	294890	376590	184930	219620	264940	257440
159680	4017600	1686900	1923300	1656100	1149300	872190	2393100
913840	1183800	569330	300880	370770	683380	929770	973150
358240	507360	91970	21177	0	93870	313710	474770
491260	502860	460380	460150	423890	322390	363170	388510
2526500	2533100	1782100	1848300	1737900	4098500	6363400	8456700
0	0	0	0	0	146380	37296	440760
62784	73035	88345	39158	191990	337300	282500	664220
67837	49402	50610	127570	93624	201690	274360	487910
9830600	12414000	11331000	10879000	12190000	7229900	3378100	4341400
0	58597	99622	107200	120810	128150	100760	0
0	95947	0	0	30082	0	136300	432800
122780	72029	347670	310510	500960	894120	828530	638890
0	0	0	0	0	32368	61847	316140
2385700	2127700	1085800	1713600	752730	2362600	2582300	2219500
97668	146880	88613	76749	89283	354870	758290	1015700
112210	133490	188630	197270	213080	194030	89569	212730
0	0	0	44490	0	105300	650810	730620
80936	80089	318110	305070	346680	66508	45629	74956
38150	396030	258910	215730	0	1271300	1649800	2440700
118280	152060	223140	360730	349000	290680	141720	139650
472500	544180	643020	590910	618470	440010	77231	330460
1431500	3290900	2599400	2340300	531690	2123700	844520	1479800
1099300	851700	598260	733030	842550	688510	422320	572130
194710	77031	86870	113190	219800	138010	39976	41851
2787100	3162000	4907100	3786700	1776000	2709500	1051100	1837000
0	0	0	0	0	222390	0	311220
26435	227380	272050	278670	169090	151090	106370	147480
0	0	0	0	0	0	0	0

98496	0	337750	567080	342780	1159000	1334800	1898500
227190	459020	192370	100880	0	182520	322390	245180
500710	0	99788	0	0	41995	212290	186240
0	0	0	138170	0	112380	57346	0
236420	513670	306450	405310	417690	205010	91610	148360
1690300	2472100	3964300	4089300	5589300	2093100	1790800	4025600
1942600	1833800	2512800	2373700	2223200	930240	558490	974680
6877100	8637600	6261000	6150000	5172800	2504200	3745100	4859000
473910	656420	646060	667370	1016400	2066800	2387000	2850900
33545	31596	68106	81890	117690	265650	264780	464480
2156500	1592600	1735600	2396300	2239900	827100	481770	710280
802020	714030	779240	794570	1580600	579220	180560	541790
2227700	3345200	3605900	3268200	3611500	2271800	1316500	1113700
0	0	0	0	0	87818	83565	444270
59254	73597	390500	359040	146800	302630	181620	59045
522340	620630	782470	745350	868280	392660	287940	318790
54318	73423	108180	107160	140680	64794	41270	53981
231750	272600	337540	308540	308250	783440	1340400	1575000
161440	0	0	0	0	0	61417	100910
1083100	1007600	112130	117420	314760	129800	944400	1051100
274120	337670	112540	0	80142	714700	837310	1566600
143750	193660	60688	0	0	205960	203460	242690
365940	215870	93314	15953	123050	125500	186630	341400
0	0	34051	0	0	89385	0	0
242060	130110	83103	93898	161700	987130	1595000	3522900
691410	729550	323730	366000	133660	1169900	1001700	1877600
1252400	1451700	485170	336930	508810	1644800	2649800	3547800
271270	373660	244500	256100	225050	1114800	1342200	1195300
2747500	3136500	911080	465020	425300	1784000	2430300	4130800
1560100	2430300	3428800	4285200	3957100	3346500	2186900	2345600
1134500	1602400	2510400	1813500	2461500	1758400	1521000	2549500
0	200460	49077	186400	123270	1139600	1587600	2154000
5132400	9658800	2617000	2770000	3617200	9335100	9683600	14518000
62223	59492	206370	232930	317610	80152	59035	0
0	97265	380060	381390	452690	215900	127380	174800
0	0	0	0	0	0	0	0
204280	172880	183160	160700	69178	102070	44266	77147
152540	174070	140890	175750	228870	119910	92825	172470
0	0	90703	105560	254430	104300	50288	114090
0	0	122660	48282	0	0	165580	0
298320	510850	319240	264760	117400	0	402380	549190
688110	742380	504090	392020	543630	64296	70557	441050
375860	507650	288840	567260	907910	165370	87453	72241
88508	225280	250780	210140	234630	580510	663810	389280
1565400	968570	1722300	1526300	2000500	1119800	431230	665980
69631	0	98301	70612	92620	0	0	0
39360	124150	191090	200400	371240	100750	0	82213
0	0	0	0	0	28238	0	126870
78105	212920	267810	252980	292670	113780	0	34725
274380	151000	359770	317740	297440	182000	127420	78804

207270	3796500	432810	174360	254920	313270	251040	1317200
0	0	0	0	0	0	0	284720
625090	731500	767390	675410	497810	315700	183820	438910
341720	217190	449780	487690	725510	650250	790310	1126700
2892000	3172200	2668400	2707600	1713500	2240400	1349400	1185400
10660000	14337000	10635000	10052000	12537000	7169700	6483700	8154600
5140900	5413300	5611400	7474900	6677700	4122200	2674200	5139200
2514500	2335900	2620400	2734100	1494400	1992600	1009900	1135700
1746300	2128700	1605900	1534700	947380	1318700	403650	988730
190700	514080	331800	335250	106150	28516	0	0
135820	0	0	177340	0	119520	0	85876
83826	171170	198950	178040	140200	171200	157360	369270
211690	168380	109910	61135	58256	189940	152550	177330
97992	155340	222430	255040	95445	63642	0	0
261670	241090	272560	343220	506590	394980	261680	219300
0	0	0	0	0	87729	128640	160730
0	0	0	0	0	0	0	175710
41246	174020	38467	41027	78734	85818	145260	130610
174940	65287	150390	134270	0	263120	265390	137050
0	0	58425	55375	96277	163050	302890	515330
320840	314570	100150	0	67291	139540	166600	258510
212300	259910	119170	61505	0	44690	0	0
726090	121410	968780	702710	1427700	913360	878290	675500
210960	176720	417610	232640	489940	441900	543770	696210
0	0	0	0	202210	19147	512370	828260
0	0	0	0	0	0	0	138380
719370	540010	716320	491610	600330	292590	211040	0
2349400	2676600	3355300	2897200	4505400	1509300	1113100	1701500
156270	188270	141450	183060	222950	347070	1035700	1052700
120460	113070	144320	258320	224700	111590	98567	271280
31130	342760	419860	104290	216570	934230	367680	767760
940920	1003600	1360200	1226300	1421100	698370	480720	715020
44869	88283	76139	115570	75894	31439	0	66982
168940	201740	95838	110120	108260	84716	96284	142240
151640	97232	65261	65644	61839	84222	784110	111850
157040	247460	457960	571960	318640	1448700	1481400	1355500
360020	264360	270000	290320	320440	361940	309380	404840
0	33751	170250	237260	69344	217650	86078	554530
764130	447730	438210	315710	368380	966140	719630	1238300
803160	299430	733950	657350	531400	2071700	1516200	3602700
1319600	2990000	3210200	2574300	2505700	1789400	615770	1627600
2578300	3783200	3797600	3451300	3550900	1734800	1446600	2808800
0	0	0	0	0	55388	189440	371370
1147400	1075700	2376800	1978000	1086400	6648500	6824000	13898000
950090	1325500	2238900	2762200	2900300	8256800	8106900	11018000
0	0	0	57364	82823	123350	147600	194740
2076600	5359300	4289900	4796900	3470900	12813000	29066000	19317000
9499000	11366000	10234000	11161000	11225000	37552000	43859000	52111000
710390	753220	794810	832620	253250	309120	288440	283150
979560	805860	520560	1305500	1043300	535640	499570	579110

10157000	12867000	13744000	13648000	11611000	9994300	6958900	9740000
444660	326900	437570	497610	622050	234920	150810	169030
295320	259020	232630	199900	361990	207370	141840	48260
167680	142990	115750	79317	96943	89143	103940	106950
649970	1370500	988400	1126900	955530	1252200	830020	1366500
583790	392200	537830	652090	1044600	237470	122890	157780
0	0	0	0	93193	151980	0	136020
620220	967540	973920	836140	769130	861000	437830	486880
354670	460110	495010	316030	503800	228590	121080	192370
3562100	3051600	5499700	4634800	4814200	3000200	2440400	1991400
2853300	3382200	3740900	3551500	4449200	2497700	630140	2080400
502040	438440	436840	173430	435940	86486	158280	323290
14569000	22439000	16326000	16187000	13278000	13897000	9872100	11830000
0	0	0	0	0	116400	140020	205200
473920	1450100	3231800	3579200	3814300	2596600	2541200	1810200
1490600	2879500	4106800	2884700	3094400	2108600	1143100	764120
465310	594820	745730	580630	652090	157530	190500	493340
2443600	3196800	3134700	3267300	1767000	1978400	1411800	955110
73247	74210	124030	135120	185640	0	0	0
9151000	12416000	14809000	12926000	8397300	9827800	8776600	12890000
447710	1068600	676230	557080	640190	334280	723720	1020300
5527500	5023400	6073200	4328000	6238500	3784400	2789000	3935100
211660	175630	0	108200	172680	117710	129540	153780
995780	1220300	1110700	881980	436150	788010	500840	770950
165870	0	292250	283600	229150	179770	107310	181470
1572000	2122700	85449	2091300	0	355180	154990	1279700
525200	0	109270	0	54190	118780	0	70988
460030	519160	782760	484140	494480	436230	232910	358220
0	0	0	0	157270	0	52424	70954
197220	268760	130980	101280	0	172190	54784	107330
296080	416860	399510	445170	458570	273330	154690	284900
1174900	1451400	1216700	1643600	2008400	1178000	470150	300930
2361900	4033200	4709000	4464400	4580600	3976200	2499000	2985300
411390	604300	763250	0	282710	241020	141410	184910
0	49463	89714	83604	0	0	0	65014
47311	52590	64333	76828	76342	36356	0	0
1827800	3179600	2141400	2172800	1639200	8297100	15068000	24513000
341940	453750	435420	535270	699610	788070	988540	1734900
216350	317920	227490	176010	206390	744680	923990	1507500
0	0	32961	159260	0	677940	770080	1750000
150800	129320	51666	56629	138200	135670	251970	0
0	0	0	0	0	206030	303730	711130
0	0	50294	127850	32353	661970	661650	554140
127570	177940	443660	311460	347370	1331300	1272300	1005900
369150	528770	554850	782680	403590	2316800	2084200	2679100
310500	379950	527910	700330	849320	1567000	1741800	1899100
0	0	30700	0	54178	144580	232840	205180
234650	195110	243040	425180	427070	1635100	1281000	2237200
155880	219420	148840	105210	27976	1120300	2251200	2046100
290830	133840	195700	226430	165470	884840	962850	2140900

835870	476500	326900	250980	411110	822130	2177900	1955800
695650	657240	585510	419260	326330	1495400	1668000	1958900
127840	127080	144110	227070	125850	425090	563940	820680
95875	0	0	134000	115100	212930	626530	447750
115800	297560	153700	119360	0	418930	674060	364390
368010	408550	441810	371710	539030	1100900	1887700	1926300
0	29821	96578	144410	0	275100	259060	282180
0	75383	86796	87021	63076	143200	140280	584640
132970	38057	247060	317170	372370	995990	1357400	987100
0	0	33788	0	0	157330	228750	0
1121700	1553800	662170	1006200	1173300	3284700	3515400	3726400
97665	0	33905	0	0	184660	89595	151200
0	0	71605	79733	233600	1807100	943360	1100400
103810	208980	374130	557530	319120	2245200	2960700	3018300
65905	0	0	91913	0	420190	390420	326350
1183600	1143600	1864000	1245600	2317400	7922800	6140400	7006200
114090	387020	114150	41965	64717	483040	950450	1435400
716190	611580	571680	706120	1150400	1601800	1472700	1906700
50631	38648	38822	48184	101570	95087	97427	127690
188570	150400	59919	135380	185820	277760	237830	605830
0	0	0	33332	0	48091	64922	164130
189580	151710	114260	100090	274470	236980	158480	436310
142970	112790	0	0	26986	235930	220410	192190
142290	40895	487760	45790	373580	1028800	617000	1042400
294980	256720	523220	788450	668170	3292700	20834000	3353900
118710	112890	36287	33445	59322	161510	68965	172900
1740500	2039000	2132800	1790800	1080500	1320300	769120	1762600
229380	335370	822880	591640	1001500	1926800	2416300	3419000
567740	916220	1854000	1345600	937770	2350400	1727800	2429900
1105500	1382700	2507000	1820400	1388000	1949800	2085900	2404100
856670	986520	1150800	1212600	808300	2279500	2502900	4126800
111220	113470	187180	169150	218450	436160	910220	1681100
597420	600490	1007700	1082700	813520	2768700	2636600	3791100
0	0	144380	305320	202630	1604900	2604600	2633700
332260	334550	205410	521380	416730	2507900	3801200	5350300
872950	1274500	689330	447170	92454	1032900	1449700	1660500
89865	120350	176860	302200	155000	773040	666230	939800
1242600	1338700	1402700	873830	1092000	710500	353240	1030600
1769700	2390900	3955900	4115000	3387300	7206200	14005000	15125000
5332000	4873400	4740300	5246000	5803400	12032000	12505000	15126000
0	0	136310	230900	274380	1362100	1617100	1362100
157060	306100	316710	290000	179490	108810	98782	161000
1979900	2712700	2166900	2156400	3091300	1389900	813580	1602500
218780	206740	148580	228770	130840	245300	257620	492220
1881600	3285700	2611000	2529700	3168700	1661000	1023300	1137700
0	0	0	0	30630	0	0	0
19128000	21494000	25410000	28483000	35666000	51418000	51102000	67147000
2662900	3085400	5551300	6326400	8893900	9023200	9472100	11568000
35347	0	45401	0	76994	103830	203700	215270
1254100	931300	1358800	556540	943720	1576200	1994400	2674600

0	47226	155160	142080	45290	689440	673060	1175300
511970	614100	936740	995820	496470	596350	327930	1171700
527460	677090	826700	585970	865090	359980	241220	754550
83151	0	123700	152530	173430	106930	286440	353520
1051500	1269700	428540	296060	487660	647000	914390	793540
206980	314370	278050	361660	394580	228510	84061	166260
39525	59910	84859	75072	87935	39481	0	0
2653100	4492500	10228000	10582000	9280200	36728000	47756000	67261000
410540	184960	952530	1432900	2213200	1836300	2181900	3544500
1998100	4221100	4072100	4317300	2777100	2472700	1844100	2549500
942840	748690	778560	792240	957630	516360	401870	415100
163320	425830	539430	416410	555260	332480	323490	403370
1990800	2625700	5800900	6357800	5714400	18572000	17287000	22670000
0	20534	0	0	0	147920	281810	248870
526330	369120	720600	405420	484760	349250	214680	702550
162450	214470	300450	332930	156890	312920	186920	89527
0	22555	110560	218400	181050	560990	802590	605380
1158000	2324600	2350500	1579000	1702200	1678500	1295100	1630700
193460	291620	358200	332660	173010	108360	63662	73512
3561100	7908100	877220	659260	629830	534140	352040	484380
207390	107990	0	0	0	0	77215	111230
1040100	1371300	461760	499430	625040	857310	593820	1257700
0	0	34981	76371	64191	141490	112910	108910
0	0	0	0	0	0	54725	178680
0	0	49903	42288	81253	46037	46924	157900
0	0	0	0	0	53873	56114	70462
0	24051	47329	98025	82105	628660	369180	1087200
941580	713580	945560	1307500	1309800	2901900	4361200	4570300
532590	649070	1128300	1302300	392940	5431800	6386900	6594000
9133300	12000000	13237000	11741000	11232000	8412000	5910500	6419900
9301500	9727100	9799700	9240800	13788000	6066700	3606600	5449600
0	0	0	0	20548	41179	63703	236110
0	0	0	0	0	115250	111450	152160
0	0	0	0	0	251950	303820	44430
1829100	1688800	1554300	780900	833040	881370	1227800	1315900
512370	369070	262990	340840	273840	491990	438400	603800
425600	368570	213030	279610	280520	613080	571520	620510
0	0	43990	61800	62418	499580	382510	468130
0	270990	386040	327870	261710	229450	134590	200010
54290	89908	205330	307500	173580	551990	687390	555240
407770	456660	542130	560150	1047300	439640	197720	256180
0	27825	143830	157360	159590	658460	802400	854660
1389300	1379400	2905900	3226900	2009700	13500000	14781000	29165000
36872000	56452000	28694000	31834000	13663000	61407000	97157000	107830000
83286	94259	111640	82014	0	57150	31948	0
1788500	1939400	2116200	2122500	1131900	1191200	846290	875100
592340	284190	853000	651520	1009000	436690	229270	197020
318160	265840	287070	493600	319540	801240	361540	703320
144540	123270	115870	139460	98307	106840	168860	0
0	0	0	23095	0	93045	73862	111560

0	0	104940	109750	187840	310230	276720	718460
0	63971	100360	108350	34106	259840	72161	618240
585470	1070200	1134900	1046400	558390	274250	259150	668650
1987400	2213900	1644400	1541300	2204100	1502900	919020	1667400
981080	818260	745870	421690	773930	425930	497680	331050
205480	302450	76100	94507	293600	67692	36208	56188
121060	74926	97904	123550	205210	174540	51952	446300
174450	394630	401670	863060	201760	524630	359380	584090
34311	48052	53804	0	75480	113890	91575	89089
86767	136480	201790	191640	181950	202430	158460	0
170930	295860	341340	334530	303530	87736	64113	157230
75893	156850	287570	137840	275500	179920	59250	339390
0	0	0	32450	0	0	0	0
323390	295550	0	311930	452100	0	100380	0
227850	235890	96766	184690	402830	0	0	0
122450	146810	0	0	0	0	0	0
597900	790160	763830	751020	520040	543330	427420	324060
81114	86434	188840	206030	173920	0	28950	237120
198420	136550	257130	289180	390190	204530	184970	245250
2652000	2716800	3568000	4409300	3304400	11077000	8914300	6902300
141400	172130	115780	0	129080	134000	140500	0
260720	267420	133430	108580	0	244580	250500	0
72904	118430	57972	43768	93534	199470	201570	256680
198290	341850	371680	261630	314410	328330	199240	320890
766400	1047700	1175000	924850	906970	723750	597110	727640
1061800	1019400	769550	775760	1294000	940190	591500	133770
507040	460010	1032600	1633100	1707800	297680	179730	955160
3118800	3140800	5763300	4781300	5774200	2606100	2701700	5833800
1470600	1801700	1976400	1643400	1663200	1805400	1491800	1510600
134920	164280	227300	269900	285000	146330	124280	150360
369280	465690	688890	665390	576810	325040	368650	338190
86384	67679	132160	98393	108910	75278	0	67398
27603	0	0	70918	0	0	0	0
6739700	8186900	4972900	2865500	1714400	8397200	14538000	14682000
130690	345980	345800	226150	419900	101140	182710	287990
0	0	0	56893	76554	0	0	0
321370	650000	912980	763520	93694	791230	507670	596120
555960	805150	1204800	1018900	2337600	947340	572740	531200
331890	866130	814130	779420	878080	913540	318770	728980
775830	899640	1060600	820050	780040	726190	246670	547190
736480	552830	756670	853720	1279600	475400	0	438430
10210000	12004000	31477000	34551000	35681000	52150000	54673000	66297000
498760	852310	1204500	1775300	1240900	6373700	4308300	6905700
5489200	8212400	12818000	11635000	14794000	17162000	14423000	16328000
7945500	12393000	23972000	25125000	26151000	51896000	50220000	52933000
182640	107070	341340	194260	252180	221080	257130	651330
1351400	1972300	2696600	3278200	3255500	7828700	9494500	13908000
3539900	3913700	4859500	4799200	4606700	3308000	1875500	1761800
17291000	28606000	27425000	28081000	24339000	27987000	23968000	47173000
1898300	3987300	5699900	6552200	5208100	24146000	29290000	31353000

26375000	32897000	43335000	43427000	31166000	102150000	98804000	123900000
0	21433	41363	61087	81577	58766	69280	53149
0	0	126580	117240	122570	951650	982350	1396000
0	0	0	0	82048	200830	173980	188000
343780	474070	162630	526810	204850	366210	57930	127730
0	0	51018	50275	60791	124990	81281	106450
5835400	7174800	3847100	2958100	2978200	1078100	388210	911070
0	0	0	560130	0	0	0	0
295870	342810	395360	369170	265700	241560	191380	198060
56607	59041	273550	204180	210130	161920	87443	120420
0	0	100010	232910	130310	1676100	1502000	2223300
270130	353980	987510	540860	1152500	666750	460310	535890
39262	84548	506450	871270	870040	2789200	2662600	3745000
1256300	1611900	1113900	1007600	341400	2355100	2729900	4956400
52767	434950	1889200	1959900	1452400	5578900	12978000	12077000
2063100	854140	513980	336970	785600	5183800	6153200	5165200
190200	180330	1077900	1679900	1218400	4018300	5947400	6669000
29098	79831	64476	80279	41151	408140	890480	788180
4071600	5943800	2022500	1603600	2754000	6368500	10423000	7041000
6513700	7486400	4035100	2880800	4765700	6621700	7086900	7802300
1189700	1136100	871630	604260	600140	958320	648180	1271400
1863900	2396200	581680	741380	525800	3404100	1969900	2045200
5820000	5764300	2989700	3909500	4687600	13073000	16563000	22198000
4760900	2643100	1106300	1273300	2091400	4936800	8311800	9358400
5984600	7986800	3714700	2933400	2861700	6796100	9107900	12290000
1787700	2021400	2384100	2191000	1846700	6477400	6820000	6361200
2161100	1804000	1353900	1652400	2033700	3320300	2647900	3520600
17152000	26234000	8520500	7625100	7330600	15382000	19244000	23954000
133980	163180	107010	176540	272060	290320	158580	403950
1883500	1960200	1063200	1018300	1144100	2293200	2050300	2424000
837360	1278200	7048100	9670800	7850100	33249000	41347000	40471000
1903300	4771700	5846100	1941700	4044800	7600600	8260300	12294000
493020	443550	738160	863190	321270	358100	171790	614270
1875000	3407000	3784400	2983800	2129400	2444400	1404200	1896800
280240	375470	388550	331390	295400	175320	168270	302790
305310	263290	286720	183640	0	2132700	5561600	4225700
0	44452	0	27915	0	0	328060	0
0	0	0	0	0	195550	156360	232310
126340	2243600	842630	797260	819870	1318700	846170	2876700
0	0	60500	0	144950	0	0	40868
132430	93861	65986	165900	104960	882130	916110	1359500
50374	46036	87683	113060	169230	107910	0	0
0	0	26621	0	0	139430	144100	131950
445640	609060	659770	540270	477870	830170	759080	706220
901840	2162000	2481800	3078800	2510000	1738100	680320	1586800
23884000	58264000	67822000	39201000	41295000	16304000	10129000	21512000
462990	3642700	958410	832330	997010	1031000	723520	2151500
5710100	9793300	17242000	16392000	18634000	10484000	5388400	6005600
89103	155000	247770	288270	143030	121190	266220	289890
54760000	122440000	171190000	110090000	55446000	130360000	62037000	80075000

2249100	2969900	5054500	5535500	2970800	3279000	1820300	2357500
0	0	0	0	219090	79106	0	82074
518280	1295900	1411900	1218000	0	2434200	2267200	1119500
1375700	0	4034300	3974200	0	1676200	633180	1533200
975050	1830300	1101800	598830	524890	1128100	1533100	1583500
869140	0	582190	0	819200	318500	0	0
55044000	197730000	200400000	135400000	108940000	107910000	61130000	109450000
14622000	24870000	30403000	23578000	28048000	24096000	15739000	24164000
83949000	173610000	192100000	171570000	27126000	167150000	105610000	153620000
480120	454870	1981200	1126600	865010	2417700	2399100	3006500
90379	54600	0	0	156350	775710	963900	436120
1462600	1982100	1055600	800660	187400	676540	1553000	439130
8725400	7580800	11347000	9519500	8713800	6802300	5493200	5917900
222130	257720	309800	288620	0	394270	215880	174380
1009000	1314600	1492000	1597400	2029600	793900	642110	569130
2942700	2458000	2771300	2017000	2790900	1912700	1564400	1955100
199200	275960	267290	286260	303830	174500	243150	380620
379180	352410	931820	1129700	847870	2577300	2497100	2598300
276470	402180	641060	801380	947460	2380100	1541400	1540900
210730	97734	265420	255010	294920	94374	56526	96874
1083400	1442500	1727000	1211000	1180900	1076700	658230	609080
72280	339930	261040	230690	0	204940	78545	56167
195420	161030	365020	805260	363240	1442200	2384700	3913800
1892900	1947400	1970300	1359100	2394100	1300200	394140	677510
31337	0	127580	445650	457830	950420	1297300	1210100
1551200	1305300	1453800	1565900	1172000	932190	629410	1147300
446260	529630	528230	596340	0	550300	292330	376770
1040600	1531400	1525000	1010500	1694100	1764600	1968400	2624200
606310	508310	1017300	739860	646530	933570	1172300	1232000
54667	124710	115300	81807	118180	145470	134170	220360
162310	374560	124910	87981	0	297640	188950	179740
1322500	2260500	4356400	3524600	1610100	4834100	4298700	7568200
64638	0	171540	43835	93055	1033600	1472900	2253000
53391	56570	91565	149360	99265	342310	710430	948440
228690	0	0	0	343550	231070	77637	0
36058	71689	91300	102030	103520	81717	74193	30966
127790	208340	179510	148370	219170	0	76574	126960
223900	281550	319220	369770	223720	217700	84106	156280
26999	52694	143630	125600	190500	0	30798	35081
0	57596	186720	170790	85646	119180	20751	0
484050	519900	590590	1169500	1587300	3555900	4654000	4535700
52226	0	0	79074	134850	158290	149270	519800
2889100	4721300	1339500	1182700	1141000	2217500	3008300	3708900
986480	898370	854060	820620	1255900	466430	198120	402840
561120	482660	180570	41423	293980	423560	347530	356610
2341000	2024200	2231700	2590400	2541400	883170	376540	1786600
374970	550830	650880	703680	430460	446250	202550	0
3803500	4433400	4875300	4602100	3744300	2706400	2878500	3286700
667680	834900	907820	871480	820210	352990	544600	402950
5095400	4707500	5348800	3943400	3534900	2934800	2487200	3830800

1164300	1570200	926930	831460	675890	266600	99653	327180
623020	796930	928860	954810	112170	577500	484270	639560
6601500	9251400	10149000	9679200	7160500	5633700	5124200	9054200
7732900	11215000	12750000	9034500	10508000	5907000	6218300	8288700
166450	229390	241530	232460	118250	112190	110580	89794
240840	229140	338130	300510	436310	206710	86440	151490
0	0	33190	48810	0	153980	178600	334830
0	0	535650	553330	0	463360	204560	234350
604250	804310	348160	248730	162410	383700	414500	658560
260850	690030	1372800	2065500	1291800	2962500	2140000	3952200
321350	613030	1974600	1976700	1963800	4921100	5374400	9122800
0	100200	97969	0	0	0	0	298420
0	0	0	0	0	35746	74152	87361
2657400	1821400	1641400	1653700	1798900	657960	960080	973340
728540	534440	1645400	192010	1226700	445120	252270	812290
406030	504110	556440	782820	463920	2251700	3393300	3181700
726340	1065100	409230	657620	559770	1109200	1791700	2192600
908820	1378800	1389100	1086600	748400	816940	562120	601280
162520	124370	62798	72364	54609	165450	209990	438230
4833500	5829500	8990100	7986200	6197900	5242000	6404300	10197000
2253700	1876600	2965400	3689600	2959500	1775000	1332400	1733800
136920	101750	259160	289970	134960	378880	365980	396800
137000	248550	132480	127960	164600	305330	246810	149290
34184000	53112000	52163000	44351000	46881000	31909000	21083000	22141000
1337900	1255500	1337100	1600500	2024600	975050	726300	510030
29851000	38040000	48502000	48350000	38496000	30654000	21029000	37867000
79453000	135530000	118360000	110970000	80752000	84231000	62536000	67597000
0	0	191630	182890	741250	0	250240	91758
71989	928610	306030	545830	215040	231840	431560	213410
0	0	0	0	0	68314	55146	55685
23186000	30082000	32857000	23026000	23728000	21292000	12900000	14544000
1902400	2690400	3963500	3596100	3371700	2253200	1124800	1132000
95905	0	137710	96499	158250	113650	0	137340
150110000	261420000	294320000	243330000	192550000	170100000	105790000	175590000
68615000	84862000	90311000	73701000	70258000	51891000	32382000	53666000
286560	498840	123230	154060	0	406250	1463000	1549200
0	0	113630	94595	126030	302430	127890	887860
485320	551200	159610	199430	432830	592210	667230	845280
73340	83316	79244	53776	68997	42625	0	0
471120	765390	677570	807240	762730	220690	269450	268030
145630	116260	0	279930	475970	0	0	0
4124100	1270500	6273500	6233800	1303800	8582200	551270	541510
0	0	0	0	0	93521	130110	0
40942	51186	75945	0	35550	451400	748050	1092600
279760	537230	885670	1428500	1327700	1239300	1057300	910400
769890	870460	521190	361370	223040	418960	157690	181350
2918500	2745100	3592700	4239200	6286600	3378600	2079700	3157600
436310	982470	894670	729980	1237200	568270	531210	266950
6024300	13721000	16273000	17106000	24553000	10592000	5894800	9200200
5121700	8647200	11255000	9449300	12528000	7162500	4207000	4212300

60431	256080	159700	197750	0	0	0	117980
189610	145040	63004	61987	49969	0	0	0
850010	1078800	739290	629920	545880	374460	457540	421220
475840	352380	428050	395320	491670	218140	128030	96509
612930	520110	677470	606510	779100	287980	297050	269100
0	0	27164	29860	0	71287	51944	93223
883650	1593700	854960	679920	857920	581360	918340	677900
246510	316420	325500	337980	197370	139190	0	266890
4836200	7584800	7929800	8050700	7349800	6412000	5323300	7697400
0	0	0	0	0	102280	71371	457860
337650	349710	195190	147390	251890	99549	76031	273620
141180	198600	200430	109490	48559	96608	66021	53303
783440	701020	750320	604020	827870	282960	258650	354870
9420300	13490000	11425000	9537500	3822600	7085000	5129100	7980400
89173	37487	51516	100380	61206	587370	367910	370980
312690	341850	277510	357290	410050	100040	39827	51299
31923	32717	75112	95179	203310	291990	0	793360
2428700	3344300	3674400	3229300	1011700	4035400	3299800	10068000
449150	210590	104510	182870	227470	437920	442780	507060
79048	119940	260130	249480	239250	106800	0	0
32579	0	205520	193360	125930	756700	935620	1253000
2036400	2859900	3113800	3155500	3989900	1792900	909920	1548900
32157	42405	195290	386850	296230	1288100	1218300	1046300
335260	417140	773080	830020	841260	2508900	2373700	4022200
352810	308090	1250300	2435700	1797200	1920100	2638800	2519400
113890	176350	190330	142520	324600	40800	0	59382
1540500	3091800	6524500	8864200	5006800	29711000	33445000	49468000
266670	184190	565450	1051000	1151600	2486000	2693600	3148900
948460	843090	1108900	967470	1043700	445110	126080	536010
364360	337280	384670	252770	306090	220560	137140	89610
3992600	4304300	4551200	4411300	5346100	3231800	1986000	2190500
246060	253700	290760	239440	346730	136650	126550	178200
57889	173950	0	40441	0	330500	158890	396560
662950	472460	799470	1321500	1571000	626710	195890	305230
70690	0	130760	111940	192880	0	0	0
58148	141110	83352	78752	63349	200950	274570	395680
0	0	65496	66672	0	883000	1246400	1308700
0	59790	66147	71360	80757	39765	33942	33349
531960	581920	779880	645730	948880	1580700	2201000	2450600
509620	576420	435430	402910	171970	396900	265230	327130
101410	265840	351060	208030	100510	208170	0	156020
46885	213370	79776	74705	137830	0	0	0
145190	174440	229770	173930	73810	132490	73374	0
966170	933410	988500	1150700	1445200	884290	570490	659040
94798	104170	104720	111950	125120	71333	0	0
3165000	3421900	5810800	5337900	6365900	3723600	2276200	2870000
644580	948340	1088100	986330	0	775450	623770	705930
209030	196650	129360	77048	83806	67611	98135	69666
528710	640260	769490	1061700	320880	4467200	7844100	8765300
51514	46459	174100	230560	392300	723970	675430	1029500

400860	538460	408300	421700	202760	258720	152780	161440
223350	105300	218100	230890	324710	166800	0	29231
0	31910	46884	42528	0	37574	0	29618
430700	640660	647590	740230	64165	429940	160760	178320
252980	156770	468760	396590	536380	348370	378150	555250
758470	643740	1473200	1566700	1654400	1501300	981080	1779000
89682	230590	586950	887120	770360	2160200	2479400	3533200
28142000	49865000	50116000	52013000	47040000	29824000	18638000	19879000
775440	687020	511310	599780	231670	515120	277540	88741
0	0	0	0	0	209020	237030	179750
0	0	0	0	0	0	0	66671
129350	209220	280930	151910	55496	91219	52203	153450
406820	701490	647580	297770	401450	350030	168350	413070
0	0	0	0	0	118740	114690	0
0	0	0	0	0	288370	288870	686990
4696600	5678400	2706200	1851800	1839300	3984500	3380400	4385800
637790	328570	783300	1214700	1081700	307100	423090	803880
195030	427580	842650	883900	1562700	248320	252720	125650
0	0	0	0	50231	0	0	0
171840	365860	414130	173690	172650	289790	62255	115680
0	41153	67355	36002	164140	0	0	0
495500	568310	508770	376550	382960	274130	174760	247360
933650	1124600	817880	613900	634650	632760	612120	549220
629600	614240	768280	782710	455260	586600	261310	433030
183610	111630	89769	0	38526	0	0	0
462120	646210	760160	659680	407620	600650	407540	393010
194090	135440	144820	150720	238670	201040	106270	193400
105300	135070	149230	287030	274950	497650	1995300	1237400
463980	364030	617470	693170	549280	530610	516210	597610
372910	307110	397020	450210	345430	225740	176080	108570
310320	392730	372330	435490	814930	243560	163870	128560
0	0	38288	43603	0	102610	83176	121230
240730	370010	344750	283870	218790	456040	504670	351140
0	0	25277	50160	86577	238350	246190	337520
747310	561220	353280	294810	254840	819710	714940	1481600
0	61692	32986	0	54607	61963	60718	104370
399240	284420	653300	459870	488830	220340	141610	99689
181390	106240	0	167140	174220	107960	49837	64107
0	94555	99856	93436	65260	0	166020	183760
137740	51837	168510	142410	201140	240540	0	0
91166	149080	101820	110850	0	187510	198910	315730
129830	235950	212940	242530	150750	165120	0	79795
1043800	1208300	1258400	1328800	1286200	500010	514080	309410
115800	79783	108400	80738	82868	58384	0	53238
4660200	6028500	6123400	5813400	5836700	3879000	1867600	2464100
280430	292580	354960	404880	536500	235780	107780	158810
420030	311220	148490	54678	133230	114530	281570	271970
0	0	0	0	0	108160	134110	0
0	35104	192360	67082	770480	1412500	1641600	2293500
0	36909	54013	35169	72251	250720	364510	512060

130800	164890	152340	157520	160980	70770	110230	0
40665	0	0	23307	0	56522	139380	120840
3874400	2837500	4297200	3326700	4569200	2018900	1112600	1847800
45833	73027	109160	89924	108410	65743	43777	95310
616100	1254800	839370	961220	286940	888610	644360	715360
159980	219450	274550	346070	137030	373180	288110	75416
418270	569040	676160	638860	493250	405640	132170	150220
216260	852000	771680	441800	481980	473390	325210	535370
93536	121620	0	0	0	0	26907	0
201780	345540	353090	324880	70331	368750	577770	575780
163690	275840	353800	382020	173350	114640	82498	76098
277570	436440	0	0	0	102200	104120	1024400
500420	560830	669840	898580	879360	518700	427140	738380
166210	150570	212720	281070	216280	117780	72307	125210
340070	374610	174880	153220	173950	222710	295590	263070
105740	230570	172640	192220	0	200830	115780	146130
2705400	2499700	3836700	4785900	3758500	2148800	1248300	1532400
519750	321980	60977	297350	145700	607820	273330	329200
5008400	4918900	4940500	4577700	3645800	3476400	1698300	2530700
1094700	1353000	1935200	1782800	1012700	1524500	1054000	1356000
0	108880	0	0	0	0	117350	34045
52537	60521	86105	86463	131970	0	0	21592
194040	698700	718080	911640	533360	539910	369880	445180
376600	484180	794430	719130	854020	203750	142370	168590
465660	264240	602260	116010	314960	73873	53783	776640
0	0	0	0	0	125260	212680	294520
379490	367300	421090	500790	469310	1023000	1031300	1345800
49525	60543	91687	79051	83041	75334	159290	95286
501780	606880	844520	891440	1280100	480470	388560	565670
527510	621300	1455300	1602400	1481500	6285300	8530200	7724000
881040	816540	1011700	1046900	1234600	521120	508270	655330
341080	357400	407970	390220	460510	142190	50749	191540
50893	54720	44357	77280	142180	51689	62468	396280
590110	774530	945540	835230	1206700	535730	496180	678950
91624	131170	708010	551380	315820	747220	341880	651800
0	52432	60291	90767	108200	33786	0	0
135080	153420	165510	148250	227580	221750	199550	0
368760	329860	325600	632760	560290	1731500	1968800	1674000
74384	119020	0	25354	30844	152950	240060	98868
1415300	3048700	4521400	4260000	2621400	2714500	1833400	3287100
247050	400630	317350	106960	191350	96824	105160	81934
548720	627360	796060	347940	291080	392540	178140	381240
250190	244800	0	475890	678250	0	0	209060
122510	150510	155370	160650	94376	130570	94047	240790
11835000	13559000	15531000	14482000	12581000	10415000	8477300	11009000
148150	163510	0	88308	77827	0	0	105580
14703000	18927000	6001600	5686700	4141700	6848000	7881500	8448000
69112	235340	183710	63567	163530	38343	0	35293
7658800	11926000	2140600	2071400	1998800	5112100	6145700	6106200
539570	2372300	1141500	1189800	159860	631610	322660	551430

0	6188400	0	0	0	0	0	0
2628100	2975700	0	0	925580	1243900	1006800	1456600
127760000	125750000	36662000	31534000	36785000	53167000	51707000	79287000
0	0	85232	169220	267790	508790	649630	559060
1121600	2081000	3948700	4437500	3275800	5748200	6014200	9437000
0	0	0	0	0	214950	219570	229570
169780	134900	224160	259610	220630	0	47640	55510
1770100	2010100	1286400	1496200	1150800	1203500	1135500	1124400
270190	323040	131360	64558	0	132350	203900	207870
364970	356240	230330	261830	568810	157440	75622	69460
785430	199600	1126600	475160	1133700	3356700	4349600	4666500
507120	688510	649680	377810	275550	408450	274690	219920
106010	102970	185260	133180	188980	86718	61382	66576
74279	161750	76525	100160	76192	90529	0	0
144780	167910	0	0	0	0	109600	0
182970	248280	233360	152790	130420	92993	108670	156850
172840	168840	210110	185720	254110	32762	0	58458
0	128490	136740	120290	0	0	0	259760
132590	136030	162140	155700	304240	55462	0	0
72325	110320	129390	147170	0	81281	54157	63820
1601500	1710700	1818900	1711100	1055600	1146100	832880	1292600
0	0	0	0	0	69987	67147	0
116350	94690	310210	276240	334170	133640	42116	44119
761090	589800	700900	741740	689250	352950	208390	313380
32717	0	40477	51116	0	94687	252070	209910
114240	121590	286000	376890	471420	1227000	1577300	1690500
0	0	0	92735	20277	0	0	383990
105870	0	0	0	0	108010	0	0
950940	852930	1013300	1161000	1855000	867830	573560	584060
0	0	0	0	139820	668970	639380	435130
181330	75458	259610	293120	467800	0	63557	0
19898000	30933000	67453000	64446000	42065000	30887000	31178000	77847000
65515	0	108090	0	0	0	75540	154150
319500	609040	530810	177800	268520	268960	269240	310240
10939000	16312000	11474000	10264000	5165000	10331000	8777700	9678200
283920	397640	295000	564040	321060	279210	212350	173480
218670	156200	275430	272450	277510	234900	92782	126010
2554300	2454100	3868500	4002400	4509200	2678400	1918800	3424400
741170	740680	667770	657870	1488600	437780	275680	310510
4146100	3975100	5068000	4150200	5206200	2726700	2570700	2444600
182430	162750	132640	226610	193990	112720	115680	0
628990	1027000	1722700	1952400	412130	1274500	931090	986090
62784	0	0	188080	0	515270	459290	642990
350240	283780	334400	189130	252490	150610	0	75608
208560	187590	445620	483810	115640	1693200	1965500	4491600
0	0	0	20614	0	82285	85295	91470
180910	231590	310560	325640	446350	0	0	0
1033500	1392300	1239300	1278400	1336200	650690	479900	609840
0	0	0	0	982820	0	0	1309900
1411300	2624200	2136300	1114200	1292100	4756600	5108200	5853100

803240	839720	1321400	1133800	283440	853210	592420	799040
134830	59855	54005	44559	47725	163140	25094	134260
0	0	264060	305250	359010	907610	1111900	1805400
92658	116370	86505	81488	84843	102170	126450	0
0	0	0	26982	47109	400780	334710	377030
263540	159160	76666	128240	222580	346380	417250	594480
0	0	73937	54133	77828	159240	155840	0
0	87174	40487	0	33666	64288	185310	389400
168040	316140	215020	48047	133220	208120	547220	330570
117060	182050	294640	364080	413580	1031200	959630	839860
10184000	14539000	19502000	19031000	22186000	8675200	5093700	9503800
618810	542650	689340	716260	967320	337330	107680	352520
669200	728360	466080	523480	439470	529410	377470	787340
274720	396020	456900	559610	771590	271620	135590	178890
2143400	1772000	2118000	1653600	2503200	1230900	1075800	1332300
409780	486380	557830	509450	508620	485530	189880	121130
1013400	924310	1177100	762080	849830	881040	341870	932200
132770	304340	402230	366900	593760	326970	291700	304090
132710	88501	161840	150370	133740	107670	68965	80316
85493	118120	179740	102770	242660	75186	0	126120
860680	745880	951700	840210	1248200	614400	401390	595620
3232400	2463500	1780600	1741800	2500800	2045700	1416300	1649700
475540	347780	471280	464530	633270	1705800	119490	136300
353490	471280	549270	511400	51340	384670	400890	649130
364990	537060	291100	204160	484540	245850	0	0
0	0	0	0	0	0	80915	81287
1034100	710380	1073500	1210400	1310300	703480	184370	308210
87367	117070	202430	114830	133700	787190	1712500	2268100
0	0	36668	0	67434	568670	363340	477660
63547	71661	102910	85370	110040	56703	0	50749
1716900	2817200	7904000	5456300	7067900	11094000	12717000	15014000
347490	124440	0	81565	328710	0	97459	103440
96498	239410	1037000	317810	469280	607360	528690	1252600
102950	194390	646580	368770	287260	229390	75657	131020
0	0	0	0	389110	0	0	0
703930	1145000	1681600	1262300	810370	777390	335870	1303200
0	0	21644	30005	0	0	0	0
140710	108700	332270	194380	87945	142750	114590	452970
0	54924	90248	67142	0	52738	33231	42070
0	122100	163150	174330	0	106920	0	0
1822100	2522500	3734900	3508800	4437500	2265600	1955700	1575400
1662400	2398900	3603300	3703600	3425800	2625100	1776000	3874800
103920	87851	103280	119850	272300	72285	35580	79264
5134600	5984600	7375300	8658900	5546000	5630700	4245700	6924800
278550	339650	614990	465920	717210	362340	84585	215880
1501400	2808300	5781600	5513700	7459500	17260000	23132000	22276000
0	0	0	0	0	0	167920	0
5644500	20846000	47056000	21909000	14895000	36663000	31929000	39878000
90803	110150	124650	110450	153210	107600	0	0
231530	318040	268580	439640	16933	168260	148280	86846

567300	614970	817110	722210	1582900	404540	286660	381240
123000	182800	288370	289430	540100	207350	116090	55903
751690	614190	173100	324320	874790	254190	238830	608350
1035600	1188100	1538100	1623500	1914100	1068500	733070	1252800
0	77734	0	58782	0	164810	116300	119620
0	0	0	0	0	268060	218580	0
455450	985580	1003700	1122400	1515400	4659700	9599600	9701800
648000	763400	1096400	753840	354690	2444900	2154300	5286000
264880	48689	281000	395220	364360	923220	813190	759270
0	0	0	0	36634	196810	208480	165340
18368	37673	26342	26877	38580	70863	90822	102270
31113	0	65928	111180	0	169160	196520	176640
911700	820200	991950	753090	1002500	570530	331900	444280
386800	473820	475890	214130	386550	346170	231650	479420
555880	646580	1007100	959600	1005400	1182700	1088300	1207700
218830	263060	129990	48287	85927	78431	124800	125350
157840	179290	0	0	0	0	0	81473
43414	53561	53504	62267	0	0	0	0
4856300	4955800	6026800	5228200	3846300	3425000	2174500	2692400
0	0	130750	0	154810	184540	0	340690
431130	491780	231620	119680	154470	274370	353750	454710
0	0	82824	98837	124950	0	0	0
330700	442090	168060	137930	127850	241270	127360	364720
72611	68604	109930	132190	196270	106520	83983	90319
89942	114960	190050	146470	0	67716	51719	66888
34419	0	0	59220	40676	342860	472870	899550
166520	224950	932840	1258400	1167800	2445500	3434000	6368600
30354	39328	55244	48972	69693	138910	86636	106340
417430	82438	214480	116940	241260	134840	56690	67803
19597	31596	59011	52098	0	613310	337940	461140
13141000	16758000	26409000	26640000	20741000	83618000	102000000	120700000
10165000	10468000	12664000	10658000	6806200	8938300	6794000	7449000
185880	189200	210720	160720	71939	168540	89808	73145
0	0	290400	314850	1124600	0	0	132100
0	91410	158810	174450	731720	1923500	2291200	2340800
378070	422310	1065200	967400	1339200	725220	481550	468760
0	0	39645	49909	0	384370	752420	632470
452340	537220	722910	1181900	1206200	2292100	2612400	2748300
125850	116300	211240	210930	373210	136850	76689	139110
29857	0	0	49893	117240	171740	185140	171000
0	0	0	0	0	0	0	70840
552840	519550	685810	788080	339530	549750	237240	276820
0	0	0	42360	50146	53212	0	80606
165600	245150	1317500	837350	571230	1261900	1278000	2642100
374150	463390	422500	58224	183480	165080	162540	54206
7056900	9382300	10576000	13811000	17297000	27351000	26966000	29795000
373700	296240	438120	79057	689140	2552200	3750000	2661500
2568800	3206200	1921400	2140700	2347500	1410600	1585800	2189200
2672200	3032300	4140800	3503900	3075500	5232600	4786600	4377400
711040	958270	1900400	1823700	2910900	6240300	5790700	7370700

166270	135790	137480	79463	269260	127320	68320	0
0	64695	70702	0	0	0	0	0
32813	191080	69921	160910	121050	52925	0	20009
4000500	4628600	5258800	3860000	3414200	3136300	1818500	3941600
33272	59741	50324	56506	0	40746	0	45930
89000	249150	300790	258560	1261100	240170	294220	182850
809180	1119900	1204500	1011700	1090600	103070	0	0
145150	130130	170080	138920	130130	366220	377980	599110
52107	137280	91224	41408	93264	146040	254210	563290
152290	130350	113800	141580	325820	850170	823090	1580700
0	44905	144970	168730	188370	29862	25500	71558
4696900	7441000	8100500	7134300	5583200	5805400	3889400	3703300
77227	55306	90889	106210	93626	82372	0	58268
0	0	222030	76942	341140	1027100	2952300	3920000
142270	130060	273990	0	321790	0	0	235720
91227	83523	146630	192890	472800	354750	411640	673260
71615	138150	218410	320160	249210	336140	678140	699850
217330	250310	0	195170	0	163180	88296	67199
374830	349950	465750	545090	410450	297920	181990	176550
0	0	122240	116510	183500	689630	1043100	679620
0	0	95696	96789	151390	237370	210140	209570
1150500	1651200	2524600	3446200	2530600	9250100	13082000	16036000
794820	872630	2577100	1915600	2539800	10674000	11405000	15426000
165560	0	117530	106090	196450	102640	0	113350
31143	37225	385930	362880	367000	46522	32476	38145
0	43813	35075	0	0	88387	102440	123710
67815	85239	193500	377660	126390	805790	1806900	3146100
0	0	0	40814	74346	89644	62203	128880
426010	917530	478830	409500	680360	405470	426860	359900
0	0	0	0	0	0	0	435090
122950	145290	84576	46513	0	0	145070	272920
92286	94538	59437	47018	131570	52773	127320	104240
618460	1240800	1192400	1168800	966060	724500	746490	1029100
229260	255700	347840	460610	333410	168200	61347	42401
147280	497090	1822200	1617100	2469500	4784500	5472500	8399200
181050	213860	366510	642830	329890	2523000	2349600	2840400
476710	606620	1262600	2118000	2335000	6874300	7359900	10465000
60995	153590	217290	446960	413510	1796400	2698000	4182400
143990	125380	355850	390680	338740	958710	865930	913550
0	0	0	0	0	0	196380	82396
153880	185060	74996	63577	49907	0	132000	230540
17223000	21534000	25483000	20592000	18236000	11278000	9897100	15193000
18032	0	180380	122340	204390	81867	389190	456360
64730	54955	72262	59335	68295	59466	0	0
470530	389310	601250	510510	341650	279270	249180	334870
5282800	5485100	4495500	4808000	4982900	5232800	4445400	4544700
51112	58157	60006	71211	226680	0	0	0
2722500	3902600	1012900	2978500	3299100	7336100	7700000	10070000
1059500	1414300	806290	863130	682850	2209400	3447800	4058300
115280	58759	703980	712600	634960	1060800	1130000	1315400

2325000	3601300	3055800	3244500	2379700	6326100	9208900	10813000
63203	53799	116630	167340	235480	266550	297800	541810
101500	138300	131260	163520	156810	243590	573680	295400
0	0	0	0	0	129300	147790	132550
117300	139780	167780	211970	235200	90444	73362	62948
80344	39333	29813	39402	29551	72242	137090	152280
2706600	4031500	6174100	6345900	3724600	4329800	3133200	8052600
1331300	1340100	1313900	858770	963280	856270	554820	1146700
406320	288120	254900	309720	332970	141290	137080	252390
765560	2238000	1208600	1566700	257550	6910400	10258000	11732000
1172700	1198300	420070	161780	71962	623910	1188200	849370
316800	297460	197190	143250	301510	83394	50248	121140
124490	136500	167890	154100	250840	98662	0	0
0	0	0	35056	71060	137460	103040	186790
0	0	0	0	0	0	0	0
0	0	0	0	0	153190	129590	0
16508000	29753000	34067000	34109000	46175000	31645000	24404000	33525000
1644900	2644400	1748000	1628100	2282100	3905500	3715800	6746400
157280	157070	117340	90978	95761	172940	139220	171460
451030	1062000	283060	322550	352510	390610	395390	713440
1847000	2033800	1056000	744430	704030	1589100	1538100	1681000
0	41188	64956	0	94182	51111	0	54270
446140	489760	412870	597540	500170	835110	523960	801270
33871	0	0	0	0	68069	52785	92104
404230	198870	275020	392360	462560	0	150910	505310
663310	873240	2010700	2172000	3687000	858120	343290	337750
453680	508030	50545	0	0	344140	416440	569820
1169600	1119400	1314700	1309700	1227200	963050	356510	401340
24016	0	45997	59870	0	107360	127420	139900
837590	1446800	3708700	3786700	4654100	1884800	1347600	2025500
21750000	28201000	34250000	30507000	41336000	19063000	10372000	10342000
4137700	3903700	2207700	2241900	2159500	4824500	7359800	9651900
221910	531220	1389900	973990	1783200	734390	567590	684330
92035	180270	98264	150210	187770	0	0	0
615190	542700	194070	49291	54445	455210	454850	644080
303450	245210	249040	222270	214910	202210	210090	294790
1987900	1671800	631240	896240	349410	1921900	4198400	5593500
0	96095	208110	103720	351130	83986	88478	79062
109670	122500	136690	229510	98656	457670	645090	517410
78189	96052	299000	126340	435510	69114	0	0
25276	66298	113380	0	254550	67333	0	232100
0	0	0	0	630090	0	0	0
109330	113580	157270	196770	267800	143260	117140	115140
91367	57669	208930	177250	247470	108120	0	53652
172890	433130	1067200	1182300	1356400	543700	422290	914600
1424900	1664900	1600400	1633100	1939200	1029300	568900	876270
707200	402170	1188800	645190	367130	478080	116010	686230
185490	200780	190360	218760	312690	143640	89043	94239
1823500	1580300	1705300	1355300	2020500	772750	636780	653220
5466500	5936600	13221000	14616000	15428000	33343000	27064000	47965000

297620	457720	894840	1217000	697860	889120	826710	1414500
0	0	0	0	0	0	0	295450
0	244540	15850	0	0	163160	426680	203730
12382000	12390000	12396000	11841000	11513000	7886500	4977700	6949600
6183200	15348000	9187400	10476000	7476200	3976800	5833900	6169700
405940	229420	2029600	1239100	1937200	4105800	3761600	8054300
463820	593040	763430	1025000	573170	4546400	7762900	9264800
1347800	1677600	1366700	1354100	856920	528310	416870	332230
357190	525510	67749	59856	78920	57671	0	57602
73914	50649	138300	75442	82275	230910	255170	487210
383240	373240	675490	302710	264520	641600	1121300	1535200
813780	4607900	3570200	2746000	1896300	2659700	1024200	3776700
704780	874540	678570	445490	118730	709470	501630	947180
1155000	1552000	0	1022800	643640	2717600	3521500	2176900
104570	183490	205100	116200	121210	137800	84370	94252
25712	0	123780	41587	138100	805730	1149100	693930
228420	248690	106450	120860	95022	186070	242840	433440
0	0	267820	155590	261360	948760	1034700	928060
52570	0	115730	176810	34503	668430	254040	255200
0	0	0	0	0	582950	465700	952550
0	29228	32054	53311	0	145480	100540	135240
0	65260	78073	0	0	704790	1043600	627610
232760	194340	403440	249830	134810	1718900	1563500	2419200
90653	230860	825010	912080	735020	2228300	2904400	3749600
148470	266610	520190	573740	439370	1587200	2925900	3879400
0	0	177000	213790	409790	898970	612470	739960
555200	648620	660760	498630	689330	2273600	3762600	4134900
303570	405240	850500	732640	224620	2326500	2911400	6784100
0	0	51191	34394	0	207120	365590	197530
0	0	34798	53333	0	152540	136220	195640
343020	377350	801080	869210	321410	2623400	3037400	2255600
0	0	0	0	0	368770	393280	495640
64532	178240	156220	187530	115850	609840	169560	1083300
0	0	115910	165390	185820	384300	375780	467280
115420	75480	104350	187290	142670	1043200	1304400	1342300
182180	209180	431340	557560	548870	1194700	1065200	1454300
19728	38668	70322	94803	81348	586180	241850	398100
0	0	0	0	0	92416	152040	710570
31146	0	259730	77856	672500	60968	102730	51499
537730	411060	421720	556990	511030	1910300	2004400	2247500
168260	152740	165060	192530	63705	328150	231270	198110
0	0	0	0	48415	52898	71547	91465
29677	0	36909	34947	67999	116080	267950	275990
659060	739570	912330	916270	1602000	663120	437580	959220
138730	109880	171890	411090	278760	131360	46266	139640
0	0	181510	447310	430020	1198400	1432500	1478900
754890	967290	435030	138750	0	311230	475380	366760
0	0	123640	100670	95527	174940	135610	282100
477580	530640	1113200	1447700	1603800	3638200	2887000	2386900
419130	360690	1525900	1559600	1050400	1942700	1492100	1952800

0	139130	92718	121410	913390	947730	518370	1569600
12812000	17916000	15907000	15487000	13781000	12865000	7908400	11148000
26665000	32565000	26272000	25987000	18992000	22562000	17309000	20683000
4188200	4569300	5823700	6042000	4176900	2909900	1560800	1794400
10455000	9803800	17116000	13380000	7836800	8959900	5468300	11701000
115980	0	116600	80093	203090	0	0	0
126670	241220	518670	471640	755980	43997	152400	80312
3541300	2933400	3408800	3388000	3109200	2254900	1583300	1439200
76925	0	153640	91435	180000	0	0	0
416550	444250	347790	872040	285710	149430	69467	112240
1045700	998350	849050	743530	748460	818010	321940	1429000
0	4735700	0	0	263740	0	864430	1646100
824800	1507800	903290	701650	876650	1005200	488090	1474600
0	46185	218470	0	69745	511130	553670	708480
4608200	5797800	5180600	4221900	4097600	2799000	2064100	3963700
127930	89686	0	0	0	80314	124470	146120
73554	81797	33775	80811	117780	260420	352920	417680
131990	60745	95291	69594	54441	87712	98660	234860
0	0	423070	0	0	0	0	0
65039	0	0	0	115270	78668	117230	0
216860	223300	234200	191410	284820	122080	100810	156380
24513	0	0	0	34285	123000	268080	156520
79117	336870	316790	376950	144300	274990	237170	382570
364300	506660	399070	343400	553080	0	0	0
625690	568000	856810	830240	968300	411480	209760	278870
0	0	0	96797	248340	0	83085	202650
81000	145660	141330	173530	177160	103620	76416	118950
708940	1151900	1645700	1943000	906400	1572800	825250	971590
559830	367730	371830	654410	1029300	1365100	816610	295120
58924	0	0	0	150660	0	0	61036
273100	273350	461330	491590	826090	195610	0	235560
644970	567240	610570	679600	788330	1735900	2477000	3467900
0	53039	214750	257340	541570	557920	501650	644810
0	457230	44521	239160	0	0	204710	0
299860	251130	318380	170330	211090	226930	121480	82041
37036	0	131920	132720	109700	392530	441620	669300
0	0	0	0	0	0	130100	958580
31898	49632	28999	64116	115570	894110	689960	1438100
1522100	1662800	1519300	1666600	3135100	1624600	917440	1126000
34188	0	27938	0	0	128800	128480	80961
259870	226250	212390	293670	233690	1131400	1155400	1744200
438450	361530	492670	458820	686390	317890	216340	238310
418720	434210	483440	673950	1023600	252530	163870	231740
530380	1115900	1307300	2412800	1694500	6232800	5033500	6776500
3655700	4875400	4556000	2526900	511090	2513500	4235100	4637000
3858500	6882800	10080000	8248400	2328000	9384600	10395000	14751000
0	0	0	47298	0	214040	248280	301110
836360	1008800	1079500	1341100	271730	792180	1021700	1734200
77021	184180	658700	854240	0	769460	590690	1014600
1596500	3201200	1163200	2017700	103550	5112800	7040800	8428600

175660	191180	240230	206430	100620	141820	94351	160880
1088700	658110	752560	988750	1493400	765770	743230	1289900
34241	0	199810	1700300	1357100	9365300	10526000	8948200
348680	215910	366430	627570	577870	53546	37198	212850
61151	66498	88814	98552	116770	55246	29198	47609
387870	530530	504300	391100	42717	283560	197050	219150
282500	162500	776040	522970	977010	1215800	1783400	950270
98715	52700	95439	146260	107320	203550	218810	100480
0	22120	34573	0	37258	89462	70275	70531
1870600	1898600	2111400	1716600	1444200	1117700	611620	1122200
2343700	3027400	2956900	2899400	3306800	2050100	772450	1044700
646370	632590	821730	926700	690480	582050	0	0
143420	123560	0	0	0	0	77752	206350
219390	185680	258040	262440	354000	151590	85194	85126
0	133930	0	0	165600	147540	118590	139290
203500	208160	275160	453950	501700	755550	640100	1201100
313520	354120	162090	109880	92523	94711	134300	126920
0	0	248780	931580	880730	12032000	4775400	10826000
993130	484990	584310	1007400	1347100	499980	139920	400310
178640	237000	263400	91842	91233	81485	51975	35612
0	0	82036	146570	163350	725540	1095700	1385400
68410	60044	193180	91970	316160	594820	562530	2061900
83922	60189	262350	429730	221780	992830	970510	1936800
174870	153430	47461	0	0	124660	164850	261550
0	0	0	0	0	140120	217960	189310
183390	189520	263270	100210	52366	94789	0	128990
82063000	122310000	72177000	64668000	40428000	122550000	129460000	157700000
0	0	0	0	0	0	416260	1353700
0	0	0	0	0	0	281590	216660
127550	209790	90199	85109	215850	174090	152930	349340
163630	248370	178260	108440	239930	146660	201820	70185
0	0	103540	142790	149860	69465	0	0
36305	0	0	0	79175	0	0	0
32447	116830	133520	162040	239110	366200	250130	303290
41837	58324	64440	51145	55448	0	0	0
26673	30401	0	0	51329	17606	0	0
285120	292090	152710	192140	30517	213010	140020	506740
986400	876940	1269200	1112500	1414600	657310	344550	336250
393990	602190	511740	574880	532070	445370	307110	478950
1008500	465720	705870	1067800	1819500	649660	141060	1002800
0	0	240730	166020	306970	0	46483	45968
22948000	32409000	41192000	50005000	73684000	110980000	110370000	201340000
0	0	0	0	0	81894	111990	96350
370610	379170	1202300	1482000	988910	4281000	4429600	5441600
520890	749280	692510	643230	508280	202070	165610	322400
1284800	1117600	1472200	1307400	1565700	1008900	782490	1447200
111290	145460	202290	184060	252290	128370	177800	204620
2360100	3401800	3565200	4482200	3748400	2589800	1790500	2287100
705510	523690	831630	1165600	1226700	3099400	2348000	3584600
0	341280	740360	1290600	694800	2103900	3426700	4011600

2362400	2528300	4082600	5720700	5298000	9103700	8562700	11924000
0	0	62845	80929	0	64772	46382	352130
1549300	2844600	2306800	3852900	2343900	1877600	3457300	3794200
682740	830170	0	530760	353110	547190	433590	727200
114490	76225	0	0	114490	54251	0	277400
1185600	2601800	1662600	1934900	1687200	1450700	3090200	3506100
3641000	4118900	6910000	5233900	6580800	3515300	3851000	4468200
5735500	8014200	6016800	2927000	4783300	3556600	4543400	5342200
1607600	1563000	1857300	1217400	1741600	1307300	698920	1825500
7682500	13233000	13498000	10029000	8271800	8280900	7004700	7766600
678810	1292300	1116900	993190	1558900	1102600	1101500	156680
927870	1208200	1067600	741610	1354800	627190	578960	644040
50611	95139	95143	93764	71046	62453	40181	47875
99597	112920	161560	138710	104630	81653	62005	67334
3228900	3041800	2779300	2368400	2683000	2478300	1975500	2089700
798380	1185500	836360	850180	832420	695010	609090	810440
638580	830490	579830	502010	0	654140	319320	563110
74509	0	55599	63428	66288	45855	0	46424
4535400	5659200	6024100	5299400	2274900	3748100	3166500	3958300
4902700	6449700	6957100	5613900	5005700	5608300	4071900	4824900
10878000	13230000	17383000	14086000	15141000	12301000	9652700	14800000
67244	84491	88716	74373	108090	83035	53264	66773
0	0	110700	0	78712	0	47879	113130
250900	384960	434410	413200	488760	132210	38950	0
957210	1140400	1628900	1309000	1301100	871160	927930	1000300
1210000	1375700	1433800	1388200	1577300	1326400	1137600	920260
475070	583390	651380	604360	491890	430990	303710	68923
132270	157930	173730	91355	0	64885	68659	325730
843620	952920	1028700	829900	671430	539810	371120	669350
562950	364030	447700	444860	274500	240460	196590	301910
858710	1417600	1273900	1151600	194450	900460	711250	956940
192010	109810	244210	230940	343260	144970	110850	75712
370300	233490	394460	337600	635600	213150	162560	1795200
733330	1404900	1407600	1221500	593330	994400	609290	336630
654460	721690	2053700	1746300	2167500	2352100	1800600	2312600
641580	337790	245310	191460	263530	436170	273280	497970
68001	55219	69427	94170	119140	39691	0	38773
70799	40853	39160	37663	39726	102370	103760	125010
133210	175500	187930	175510	133030	264310	259230	303770
352680	372540	190740	217520	237310	209920	228720	675890
435380	689690	307160	261270	73202	264570	208520	743890
369730	342750	0	129700	73848	145750	202320	187140
71812	128350	59914	46297	0	100850	106960	286190
0	99547	39158	24484	32876	204180	687290	474730
0	0	0	57000	77369	248810	247820	212570
61539	0	91638	113630	0	185880	160260	344110
1469400	3172900	2015200	1853100	1168800	1205500	1403200	1595100
335400	463480	561770	375620	655940	259930	177780	497150
4009900	6057100	7030400	7172700	8342500	5350700	4224600	4105300
40399	71597	0	0	0	71054	68099	82318

396170	564650	854360	716820	196100	617020	420880	429440
335990	226570	238180	420500	188980	63753	91766	162110
1348000	1953100	1502600	1431400	1079200	1022200	439280	1013400
49409	115920	135630	51328	50009	80362	26438	73178
200180	307560	509450	660780	959670	1620100	2244600	2317600
24873	32386	90296	136100	105290	376100	331260	547790
38042	181510	249620	332320	46207	789900	886460	1861300
0	0	0	0	0	0	0	1560200
1635300	1912900	1217000	1170500	1089300	1793300	2217500	3111600
449240	542500	579560	567390	489370	538430	350610	393800
484470	468920	473680	672150	885360	1840800	1987800	2789100
0	0	134970	0	0	142390	177650	326020
27257	101420	103000	66427	168120	255780	608740	762390
14691	155210	71832	73331	73994	269630	269690	366950
212530	344450	103190	103290	80905	249960	567460	749090
0	0	0	0	0	73858	59114	217700
2622500	3338200	5830000	6954900	7982700	3332200	1989600	4273400
1617600	1833600	2837300	2391000	3543100	3059000	3323400	4554200
494620	870880	782600	771250	1503200	295710	87991	218160
47327	92195	280980	315940	554890	335880	263160	331530
0	87190	237010	166460	357230	89510	0	286200
224370	181930	531490	353170	654460	121360	142890	87518
1210500	841500	1847200	991460	1996400	461420	791770	371640
123940	1050600	332640	43697	157070	250220	766320	1010500
309980	379260	498230	368470	525360	297500	278490	234300
642770	584490	1013100	639230	503210	589360	378210	1007300
163250	260120	552750	532870	815010	355660	244710	116840
0	0	67461	104210	28095	186750	225620	72451
177910	484180	360590	158030	315000	78318	558080	0
212430	251530	1591900	1701600	1964000	1825200	1849900	1992400
636500	1496200	1617900	1668300	1084600	767280	272040	461840
176470	113550	0	0	0	0	0	0
192570	610580	3875300	4160000	4146300	4777400	5214800	5308300
2100700	2927700	2518800	2240300	2125800	2029100	1861600	1817800
427500	437860	442920	398420	341940	312780	405100	585420
317940	446350	330350	391400	542030	624170	491390	637650
0	0	0	0	0	0	0	0
294950	533760	797900	773860	816420	445540	262040	304880
0	32881	242490	224210	339340	139160	80628	44658
1015200	1396000	1185100	869730	1230000	1105600	1219400	1196900
348370	280560	846910	754860	1144200	1354800	1184800	916470
44507	41063	259620	279880	324320	1912000	2658600	2568600
0	0	333140	381410	432440	1561800	1730900	2045700
50180	72033	55884	37316	28040	42749	35197	54252
37732	102370	174430	154540	151380	145440	86850	163430
317230	634850	569200	445010	159410	787320	1402000	770520
570580	423130	280840	242650	391900	261860	162950	259170
98863	221220	144910	150550	213310	206980	233570	262660
2356100	2910900	4063400	3453800	6729300	1190100	938720	841310
27213	64880	47043	77218	0	46081	16388	49244

657860	0	0	0	2368600	0	0	0
67814	58500	93644	88596	138000	58979	0	72184
0	244200	202130	296720	594790	283010	192830	112490
63496	259310	409110	383840	141970	170110	0	166920
1667000	2812400	2037400	2655500	2622800	3356300	3087300	4506300
994620	1410800	1105500	1124300	977300	1271300	2261900	2651000
264970	442250	262910	390990	327780	1354400	1290600	1649100
0	0	0	0	0	284170	221080	450890
9080900	10813000	10508000	11941000	13416000	6415600	4639000	6061100
584150	1066700	1522300	1922100	2559800	2205900	1029800	1790100
33642	0	135240	123620	263530	139330	52500	102090
332730	369950	374770	500980	490620	312940	204080	161320
96208	56598	38686	0	0	54242	0	111020
0	0	621250	641580	357620	614890	355970	276350
0	67265	44934	252820	246920	452940	354120	477430
162820	263300	291990	287680	128270	188560	80879	0
308020	409390	496520	331540	556250	370810	202740	128540
613220	428470	427300	765100	932670	429440	122230	238910
0	0	0	0	0	0	0	140050
0	0	0	0	0	42657	51242	122280
0	0	52637	43096	30027	91596	0	0
51549000	53630000	54396000	49489000	79483000	23843000	20752000	23738000
129270	257210	128570	97004	61063	123340	70122	488440
5792300	7430600	5482900	4789100	4993600	3832600	3687500	4153500
140910	0	407150	468980	726480	534990	209820	265430
2778700	2975000	2820100	1512800	3397200	1465900	906840	1629500
120630	184320	288680	424480	25881	156260	75475	52558
0	0	0	0	0	0	0	0
1010700	1405500	1455500	1593100	1436900	942180	641270	977560
1944300	2931900	2499900	1755700	1215600	2738900	2161600	2893200
250240	351240	145930	30568	55700	0	85360	0
73872	89846	116630	53458	121020	134170	95076	309320
1305500	1393500	1245800	973920	900830	2063400	1695200	3185800
994010	1652000	981990	747750	776710	1425400	2042400	2086900
331580	399580	437760	394480	95867	537870	425020	457420
1002100	1778000	1543000	1517800	1214100	1209700	1235400	694900
1519800	3413800	2061400	1327200	1450000	912310	3206700	3086700
600330	1066200	1222900	1748300	1414900	893540	990070	929430
80606	149040	150030	51674	101620	290520	303750	148280
113890	166290	122450	126980	153010	72959	69133	147560
33920	0	0	0	0	121170	69102	259400
0	16101	164970	271070	308900	851710	603180	857670
118950	0	155420	83905	130950	213160	170870	258480
199040	202200	205500	247870	165220	261440	210810	292950
525900	857730	989870	1040600	483530	2091900	3113700	3164300
0	0	0	0	0	148080	376520	487890
979370	869950	573810	886660	592150	1884800	2704100	2671700
644650	1054500	662660	510070	236220	459440	374060	572470
0	0	104420	106970	0	405850	341440	846580
1201800	1710800	755160	504030	365010	1819900	4199400	5467500

54580	88845	55994	77135	0	254060	131880	1050900
109720	0	0	81740	0	289800	639850	0
41748	58356	227620	171750	136890	152870	195420	232120
194650	317030	121250	330700	270570	787770	1139800	1400500
82642	99118	78292	66766	91631	394300	313590	781400
183130	267920	289860	244310	300030	153100	42310	83429
53886	45748	59595	51706	0	464940	865310	912400
282720	624620	1201800	1676500	1006200	6156900	7148000	11541000
0	28637	162180	227850	59204	913580	1305400	2277900
8405600	9694600	11241000	10080000	6223400	7606600	5035300	7814600
183380	103570	79171	62735	89850	453200	451760	561160
391090	354780	350570	298200	277430	206310	0	149800
0	0	64892	0	0	0	311770	152930
0	167690	523830	623890	460880	1364900	739630	2079700
0	0	75214	93279	37103	346430	397250	360100
837550	659420	1225300	1224600	1429200	385460	131880	102460
1787600	1683800	1832100	1660800	1101700	1027400	526040	1478200
115470	189990	78695	27635	0	35173	71437	123960
1163000	1174600	861190	397910	548780	594170	280880	678420
1406100	377050	1161300	1112200	658530	542280	409580	363200
1015700	1365300	1220200	1228100	959010	922970	576430	297630
21153	228980	67173	144870	163300	596750	1141500	1002400
0	0	0	95254	46407	316590	317040	506650
31319	30388	618120	297800	877720	1550100	1193600	2597100
1170900	914190	1356900	1406700	1103900	745590	437970	414230
349180	279030	34195	78657	49173	382640	384770	443890
248730	152750	163520	204950	326190	341050	383310	607970
233980	271740	384280	280110	181730	661350	785560	600920
0	57988	101210	0	0	78415	75098	213100
0	52876	131540	142420	154500	89143	71802	0
1697900	2079200	2051400	1798600	2819200	1227200	766650	886730
92225	50164	91904	121610	180960	255980	158980	297100
0	43221	82167	77585	127520	219040	214120	311110
254560	198490	339270	345730	427380	934220	1037900	1353500
49674	108290	0	0	34321	113500	466500	341700
0	0	280920	104390	602050	2245100	1223500	1619300
962760	1393400	663900	594690	603230	1667600	2788100	2795500
207620	263220	168870	207450	0	526510	1054900	708150
69751	184520	446820	69618	61630	64577	0	372030
94368	157330	476680	336610	530900	308120	327630	428450
0	0	1361800	1380300	2257100	1192000	1147600	1375200
0	0	0	0	0	0	224980	0
9705100	13789000	16448000	15353000	13120000	9199700	7497900	12841000
790080	928060	1373900	1435300	915110	823400	554150	558750
1169000	1139400	1460100	1236600	531310	501490	907740	991350
85878	124680	121520	130670	0	274360	202860	256940
3636100	3318700	4891000	3370500	3286000	3034500	1917000	2793100
544640	895640	998090	808200	402640	419340	674770	290520
177450	158610	212100	177250	212720	188600	0	153030
0	160990	229230	246530	247120	91736	28320	50689

2292300	2330300	2703700	2972800	4151800	1428400	1139500	1474900
96033	90058	265410	167210	312730	133950	72867	130340
121230	115620	203450	395080	470830	996190	1717400	1023300
325650	829650	496330	585240	198120	1102200	1416100	855740
221040	111770	104300	206380	0	0	68458	0
0	0	50447	56605	2188800	134630	0	348860
161550	108210	180410	158710	0	340090	277010	350700
625010	1243100	1223700	1024100	1067100	1054100	1262700	1287300
0	48863	87352	118660	98400	157490	282730	312300
269280	506310	2579600	2755900	2733200	3260900	4141200	5553800
132450	282900	1333300	1117000	1207500	1849400	2037000	2237600
245010	467370	532870	473060	408440	999930	947030	833420
0	112460	117950	179140	0	0	0	0
222060	315720	261010	255830	44246	180420	37643	52684
835620	967660	203310	204030	0	1903200	4003500	7609200
63089	61853	172520	176200	208150	598370	579020	859650
0	0	137040	34366	0	382480	250010	286520
0	0	240710	159640	383590	682350	428380	573670
214370	559220	904290	960560	1271700	1512300	1842900	1927300
172360	285490	466990	448430	291700	951040	993600	1512100
537230	499370	558370	691730	802250	1316800	1065700	1381400
129820	266180	146000	123280	0	352700	557800	511340
872710	1479200	349400	342720	178260	735650	1244200	1492700
2364200	1555900	1178400	1214500	2414900	941840	1312500	4740500
636970	465400	1636500	1560800	1579900	7024700	8635100	14862000
244440	418480	394480	348310	250190	255400	150840	57433
1094400	2945500	3040100	2864200	2322900	2884900	2526200	2903900
0	0	0	0	0	0	0	105210
630450	1241800	1089400	1118900	0	187130	191780	153460
597280	487860	1415500	1625300	1969600	3129700	3333100	4617400
1659800	2061200	1870800	2056300	2113100	3152800	3121200	3707100
0	0	0	97553	92567	568180	1114000	1094900
0	0	0	0	0	640290	697340	902960
0	142160	49371	0	0	99032	167210	186170
5029100	7994400	8264800	7478500	7840400	7088400	2145700	0
849740	1511200	872640	1237400	281470	916840	868390	1163300
2005800	2061200	2051700	2999000	2870700	1342300	516110	943720
632230	951280	1479300	1734600	76525	678030	347610	2213800
28741000	42988000	42054000	39885000	40936000	22526000	11801000	18962000
755560	1392200	1653500	1765100	2337100	6811000	6231900	10002000
228490	290020	87889	40706	111070	55401	135010	135840
61733	68230	64415	82851	69750	79758	37700	0
358800	308680	230200	265250	132510	521700	496690	693080
27198	0	114610	110400	219970	399120	241390	234990
28048	0	100400	137450	179060	200950	256000	298310
1255900	1148000	1095300	785760	877820	746820	848540	1221700
1578900	1825600	1782500	1600000	1625300	1261200	766930	809810
899390	763470	963570	1255500	2128600	585630	215390	293720
824380	1008000	1407000	1269600	952940	687100	398490	239120
346950	628080	836640	793130	583780	472560	149050	75609

663730	1157200	1682000	1213900	1638200	971030	317810	436450
492370	338030	418780	528440	757200	372000	198120	213800
3356100	4706000	4215600	2380800	1272200	1457400	1257400	1304900
0	0	0	0	0	0	219720	293490
180190	67989	130410	107190	103730	839340	1113300	841470
89005	111620	107720	105820	169990	67772	0	0
35208	48299	77833	75566	146680	137610	1360400	163740
78049	100680	220860	335900	117210	1343100	1188500	2042400
2356600	4025400	2434400	4023300	1934000	2129600	2447700	2216600
60951	60212	99406	33342	148970	465910	522640	734360
0	0	0	0	0	0	0	0
228160	129830	968010	803660	1415900	294310	51486	111500
115880	73642	142990	229670	322990	61182	0	0
687540	831110	881320	807150	743600	594520	303900	533700
83902	265050	409150	379080	1256400	212260	82265	221150
0	32967	0	52164	0	73665	0	323700
81529	60177	144240	394600	418060	183160	183240	0
476880	928870	1422100	1229900	1907500	854650	689840	737570
37893	0	98998	133500	109440	89000	97879	58699
19682	0	148110	120090	337500	0	52194	434570
0	0	0	0	57072	222810	152780	124950
235380	240310	1202300	941460	1455200	2270200	2296800	2277600
0	20938	88733	114900	81867	195010	160080	64315
463630	316410	411520	425490	232350	272220	206600	0
1359700	1745200	1372200	1207400	848930	740600	349210	545010
0	0	0	0	0	0	90513	119980
253350	277210	475450	446840	378220	136560	0	357660
159300	182520	215730	234960	99897	159070	40751	46217
336880	460290	131440	486370	369280	248460	193080	224850
0	0	0	0	0	242540	0	123710
206700	249970	334160	329130	477610	236740	213650	216980
3973300	4364100	6384000	6673200	9049200	4110800	2988600	3806800
138830	199670	391580	397240	0	271380	169230	319270
267780	413230	499360	465960	329740	963900	867830	1253500
317130	360890	383600	409470	76188	342950	101730	120700
1062800	1800000	819420	270080	656760	951790	1282700	1076500
24238	107360	1708400	1250700	1544100	4818600	4667700	6308100
1002300	1006600	1141400	1261100	1775900	659130	385680	412410
157810	141280	247020	296820	468290	400720	459800	553950
0	0	72452	47817	74741	43365	42306	261110
32739	0	0	0	0	0	67260	79359
1400200	1223300	1600800	1934800	2586500	3685000	4010100	6024800
830410	1213300	996310	1271300	852770	3116700	5662300	5968100
1456000	2379300	1628300	1908800	1732100	4255100	4030900	8493200
617910	643200	427910	391370	591390	2420300	3107100	5092400
1954100	1992800	2732900	3046700	3122500	2719000	3258800	2602800
1222000	1043200	1352600	1698600	1639400	4588100	4411600	5284400
1440300	1237100	1811400	2583800	2156800	5906400	5218600	9185100
445810	846210	892140	1177400	834830	2209100	2913800	7517200
63431	0	298730	335770	632050	61754	76778	246690

66273	185210	109200	44594	62188	37554	113340	50239
0	22758	28546	41514	38037	165670	160540	195920
1110900	3659100	614090	1572700	1576100	1150400	1448600	1205100
1107600	1922700	2568500	2204900	473990	2426000	1282200	1728400
974310	1919300	1991600	1913500	1255300	1019500	388200	3941300
0	0	0	519560	234840	0	0	982280
519200	275990	185380	109760	0	184960	202440	136950
314320	396590	317790	217230	124670	304620	308330	222350
63566	0	0	0	0	0	220470	91474
961350	1333600	4166200	3078000	6258600	16226000	30088000	40470000
113680	276750	220270	191700	320360	134020	46698	45735
113890	103950	262230	359440	449530	1081300	1158400	1244200
2939300	3374800	3915800	3186000	4425900	2426700	1828900	1826700
102520	131310	229590	40333	0	637870	1196600	1696700
3405600	4243100	3150100	2379300	1868900	2044100	1856100	2339600
286480	402730	510520	685260	677900	1382700	1396200	1657600
787000	1662200	1134100	977430	1692000	1146400	505660	729650
1090100	1517100	1338800	1119700	515250	813920	464150	774500
365710	424340	364960	251870	0	368230	90953	94656
89890	48275	49965	70735	0	107840	0	159930
0	0	457220	579950	813220	1196400	1365300	2369800
0	0	0	0	0	0	113830	0
558370	610930	1078700	1147500	1089100	4700400	4879100	5430600
1876600	1992400	2044100	2136700	1858900	1346300	1454800	2986200
0	0	0	0	0	0	101800	105790
97176	129200	0	0	0	0	0	0
0	0	48047	69934	67949	524290	1509000	457960
234650	581460	2263500	1265500	2393300	823850	781610	786040
1536100	1449200	871230	1087400	1065900	2316700	3486600	4331900
393650	617520	502490	432630	581250	991150	1296100	1456900
0	0	0	43419	53871	76632	97278	0
1857800	3151200	1506600	1249000	733200	858570	779100	920350
1051600	1318900	1779800	2126300	1969200	1120500	778480	1784700
146730	162160	212670	0	0	143380	69110	132640
333030	324090	480310	622880	613360	271570	60489	250850
73176	0	87194	77778	170430	86661	0	0
845210	1098000	1301800	1340700	1964900	965780	624540	611090
0	0	93668	114300	143960	59270	0	0
67097	18898	394430	478960	325130	2457200	3616300	2567100
52115	42248	112690	91613	336240	54512	0	125120
0	0	41379	110050	42370	534970	638620	616230
284910	249640	457840	352860	285780	907230	743780	986670
118800	112130	242700	305000	123070	997650	870480	1143700
0	0	0	0	0	68943	59008	94832
990790	1156200	1121500	876680	412550	739500	492380	484960
0	0	0	0	0	86014	436900	707300
295360	119850	184250	326020	549410	1492300	2320200	3903000
92021	96209	151090	229610	454000	301880	0	145670
44554	72202	71346	74876	99782	150030	87357	108920
135990	192080	71184	147120	98257	136880	111290	163660

668500	1218400	825040	770780	893230	553830	495360	590550
10878000	15543000	15589000	13694000	13237000	10578000	10853000	12693000
222280	316250	349070	358870	126970	278980	199460	190960
388230	1142900	1402500	906600	1128200	451390	658040	782120
253430	228760	237830	109020	110620	59232	84552	65094
440680	426290	511510	537420	463820	570920	485160	680150
1218300	1494100	3343400	3874600	2581700	13175000	14942000	19552000
259730	275870	228000	281800	219730	198950	210710	0
1972600	1843400	5291600	5846500	4364900	7722200	6523700	10559000
1209900	1581400	3074500	5033800	3996100	16874000	17874000	22593000
545270	861540	329080	298080	193570	463950	568660	28742000
486010	563870	413980	289510	378190	201080	352520	347970
474490	846190	600650	893630	621110	2662600	5229700	7559100
127620	241770	132160	110140	0	77942	194580	156340
204760	575720	363860	582430	330080	415940	272570	196600
534590	804400	539050	452900	264740	403250	320850	341890
84771	283900	631640	444540	208420	379190	195170	472520
584180	737660	522690	538460	0	0	0	276080
1399500	1580200	1038700	633850	1252700	706310	902680	482440
1023900	795530	2829100	799680	1716200	705890	309110	416000
400650	340420	574580	668740	292760	395010	280760	509800
2250200	2646500	3101800	2445900	1987100	1653200	1053500	1071700
733840	904190	998920	914870	513050	690770	353070	632850
767710	721200	722470	692520	609720	426300	391440	282160
265430	445060	438340	509090	187440	250140	64425	50515
0	215440	274760	281980	315910	147130	70214	133050
538540	608790	508380	358260	663810	319610	247250	779040
785360	886710	712970	368570	408400	611840	486050	666570
1362800	609150	328290	464600	674110	587610	331300	450920
268780	309150	349970	451460	300930	360810	125460	89002
1406800	1280100	1044100	1010000	645290	975520	489310	362720
271470	313090	440110	448930	452950	355080	203250	182540
1603900	2365200	1937600	2178100	2668800	1144200	596660	1480100
760100	675470	628840	625190	421030	331970	252240	344270
0	0	227050	114330	207080	0	0	53649
202410	239910	291840	224550	335420	0	0	111860
0	0	362290	301270	0	0	0	0
177600	270290	366230	387190	305430	151670	29522	52335
161790	419690	106680	0	0	0	0	0
148720	571560	348980	648760	0	470300	348420	797650
1033300	1011900	1053900	1095000	708910	837270	289780	488820
763060	1030600	1434800	1441700	1279600	1006400	521190	720320
540680	647730	877600	510680	649930	464830	301600	393530
108730	76511	59051	30344	0	386150	619620	0
578020	715160	486250	398160	327740	405380	720090	688440
875150	2703900	2849600	2713500	3098400	2459400	2048200	1439700
698160	1167600	1064200	1291800	504170	964260	588790	759720
7070000	8875400	10822000	8679000	8357200	6585800	4599900	6522400
5411700	4918500	5965900	4342900	3537500	3686300	2170000	3073800
211480	161070	152470	219530	83363	458730	534680	615980

1306100	713570	2882500	3691800	2130200	26952000	31364000	45786000
65576	78471	107790	110230	104340	223820	201280	154850
246520	169930	151900	173850	150920	54609	38691	56088
2156800	2068100	1124300	605830	885590	1803000	2299000	2965600
185190	332270	101520	38380	216240	192360	192600	224160
313650	507530	364230	425400	465560	1055700	669290	882680
563530	562090	0	0	450210	488460	354500	1554700
309910	432830	227340	396510	363430	564610	484210	706790
3542600	4287300	5493100	8783600	7375300	11905000	10336000	9904900
489670	674090	389670	1021900	699190	1497000	1810800	998400
2036800	2377200	2514900	2881000	4870100	4825200	4710200	4069400
34507	0	135470	82116	66873	1022100	629320	1015500
1787700	2479400	616250	0	231000	940160	3062900	1217900
89656000	98121000	52634000	34216000	32922000	52577000	101070000	93746000
491920	848120	336280	99054	0	312930	280000	297800
3390400	4123500	1999100	1759300	2561300	2762200	3750700	1996500
21201000	18322000	11097000	5524200	9888100	11810000	7553000	18439000
483360	498290	360190	202040	54233	70392	84658	494120
122830	235630	34041	42533	18518	84581	225840	410490
62994000	81995000	50727000	32560000	26025000	46490000	59163000	64858000
350910	492510	139970	85022	195550	98853	109310	97607
33285000	27815000	13813000	3268800	1026800	9173100	14612000	57155000
265810	247010	72097	0	0	127570	221480	229060
0	0	41869	44471	111630	439800	598780	394370
305510	256230	68143	97435	104180	273060	487100	255240
0	0	0	0	0	0	42725	98024
82213	92674	125050	127790	157780	86515	30292	0
171810	243710	45723	48643	0	53383	140080	214290
0	0	55644	213190	0	738210	576290	1065400
0	20456	324370	380150	332130	1019300	823550	1937300
389330	438870	490430	356600	356370	356810	348210	523770
29983	0	50184	76048	139360	103910	70419	88124
10903000	13121000	12931000	11392000	12512000	8654100	7927400	8405000
16849	0	43040	132890	122170	293650	290230	296190
0	0	62376	77902	38583	244920	241360	339550
481570	404950	625510	600230	1047000	451520	252960	214630
624500	537330	1086700	915170	2050900	808190	166980	721390
373500	329510	521180	555800	639690	253010	154460	358250
682610	743480	689350	936320	998550	1181000	854030	1217500
166940	202370	125830	126560	130990	382720	325090	466990
365060	557590	303210	221390	63805	198850	295590	682600
267560	178470	225820	338000	180970	1045500	1072900	1453100
0	0	0	0	0	0	134600	110160
0	0	211460	274240	53729	1038200	1336800	1696300
0	62500	208380	157760	0	418900	337820	1193900
0	30669	0	0	190160	249650	402230	583790
110230	131820	38565	91388	55948	363610	515280	565080
166050	274000	299540	372030	787630	150440	140880	0
0	71131	159050	102820	524370	0	0	0
0	55396	258920	372490	569970	118430	133920	390030

187740	827180	118800	112350	148400	373720	192830	0
125960	178420	231240	208490	212710	143030	88780	83289
109340	247350	527590	268310	518050	265430	121250	72330
32741	60232	45536	55988	0	114240	121410	114000
0	0	84084	106770	58839	228620	0	330460
1050000	1068100	615350	896510	694900	2006600	2014500	4929700
307420	61223	243930	226560	114120	415880	547450	820890
0	0	0	93903	72923	92166	73256	402660
0	0	0	0	0	0	97464	112270
263760	295980	229560	602840	229440	1392800	1850300	1746700
122910	0	292930	188320	0	159200	0	69381
57422	63492	0	0	0	68759	0	215590
0	0	33288	0	0	146910	87371	224670
111310	124620	277900	384560	432020	1442400	1103100	1572700
0	49199	123490	82218	0	1104700	1502400	1728600
152650	0	61776	48904	0	130780	188200	362120
0	0	88703	27702	47499	152270	138380	86268
166430	170660	279690	258860	245930	908350	784390	885820
26762000	30722000	39835000	49523000	46160000	42807000	29417000	32990000
27894	0	42748	108500	224010	285800	653720	229430
200090	129240	143500	0	0	0	232930	1126200
0	0	58033	75072	0	287490	397060	395860
0	38113	136410	60772	0	273210	385160	625480
87959	155350	104760	410610	612280	1239700	1327300	1626200
45320	60540	243670	324720	434840	895040	1032300	1039500
429320	548870	675750	566650	92715	243020	143250	108810
25796	30928	112800	128010	0	297720	277250	405920
708770	824340	1679300	2016300	2189700	7091800	6894300	11893000
0	81046	75520	0	38680	146200	231940	199640
121210	152850	147050	169990	0	196290	0	221510
1119000	954840	1538300	1442700	1209100	892230	771550	743530
1571100	1451500	537220	680030	721260	1687100	1842800	3222300
1369100	1504800	2136800	2057200	2437700	1104700	498680	1273100
242410	398860	256870	238940	254910	135080	139080	71016
0	24431	0	13367	18119	369430	841970	1169600
0	17566	35729	61649	0	315200	243120	284460
64499	102200	90529	64207	68461	54409	50363	49541
253470	348230	581100	471750	329500	555090	572560	481560
2445900	3262000	1925800	2083300	1574000	1397200	1736100	2309500
2151500	2951300	2060400	1973700	1459400	1417600	1452400	2113300
168270	274610	0	0	0	168440	244270	199770
268000	292340	273850	244390	377110	80794	69039	51645
0	52303	0	22593	0	0	33991	0
0	64111	0	130580	0	0	164080	469670
154720	136800	199600	186480	307270	106650	130020	123030
2048100	2006100	1970600	1873000	412980	1402700	586450	1064500
273860	51611	0	0	0	67021	369470	1143800
39957	80869	0	31960	0	257730	296880	199000
1572700	1837300	1986200	2243300	3527700	829070	263960	551870
0	0	0	0	0	112050	119030	128410

1165400	1577700	2196400	2204200	2587300	1370000	766890	1129000
0	0	0	67280	115160	353140	459050	172210
193130	416360	779750	1183100	468870	2966100	3914000	4384100
201020	286490	249820	0	331460	165340	0	0
60263	223990	364860	480450	363160	504460	294690	389050
0	186470	239180	184250	175430	178640	149370	145220
591610	367090	634270	736850	528950	1111000	1130900	1277500
130620	119840	113660	128160	137430	180640	106030	0
0	23410	27217	0	0	55354	130310	92000
441590	429510	134960	160560	164040	610870	725930	914850
55849	55315	62246	65531	94775	31486	0	36892
6432200	8845500	8058100	6849100	4058300	5690100	3553000	4627100
0	219150	0	0	339010	0	0	0
1931700	2684200	1558900	1731000	913330	1629100	1241100	1010300
980850	1535800	1438200	883980	267300	999520	715520	1397300
282290	740810	354360	351690	0	387660	374730	698400
168870	366910	438990	361380	430870	213070	170070	0
1290300	1249500	1807300	1871200	3867600	558100	432510	1202900
421980	919990	1108700	1084300	1349100	825920	177210	161870
423110	297520	683320	515710	1000600	402990	219640	481970
966960	725320	1055600	1122800	1440100	1140100	433940	989100
53163	50982	82684	0	146460	0	66931	0
84490	59257	95160	83764	105830	36799	0	50618
655190	676470	788470	851700	997400	666660	447100	663270
1189200	578990	796350	701440	804340	660700	203450	248820
462250	594820	299290	215030	311980	828750	1326300	2794700
1402700	2478400	1550200	1796300	4535800	3341200	3316600	5882900
1007600	689290	403710	279370	435180	220090	0	0
173530	197380	183550	194430	221610	122820	66993	109120
21316000	28129000	20106000	19939000	22854000	14082000	10293000	18589000
21137000	32246000	23480000	23603000	24737000	16262000	11335000	12692000
3978200	5902100	7120700	5548600	1930900	2565200	1654400	1977300
118540	202990	264140	147230	207480	127020	61186	65323
585640	1024900	1013200	791940	0	593260	0	467860
379030	580210	922220	897880	791650	785330	820160	768240
87805	82969	173240	167430	220440	118300	0	76492
355610	747870	846350	1049000	1125500	1423000	1695100	2149500
0	86376	123970	173150	0	144050	37012	0
167820	444700	582650	564580	595880	669410	431870	154960
77234	554320	637010	949490	541430	798890	516130	1202500
169710	276990	845770	910270	867640	521790	418320	597660
602870	534570	706740	791350	1084600	794620	698340	801500
290660	400030	186780	486620	490180	1034500	1512000	2504000
538550	425150	269870	261150	383660	189800	382200	481840
75422	243780	315720	526330	402610	51977	49520	400500
640390	804740	711230	768410	615670	564200	249670	83954
140680	179130	385690	299260	827780	90833	63881	164240
0	80697	104650	86519	104760	144470	115200	178790
0	0	0	0	0	0	58721	140520
146060	62803	78836	68917	112800	61318	48339	55961

55231	48902	56684	56174	95211	100470	0	0
0	101290	133390	0	0	0	38243	0
1996000	2288200	1119900	1062300	1238200	4352100	5166000	5949300
945300	1322900	1309800	2020700	1773600	4509200	4361000	7644700
212450	134510	844830	794760	816550	1343000	1635700	2485100
0	0	0	0	0	0	180760	173900
19999	31315	83711	115820	43263	120130	143940	162300
0	0	0	0	37228	125250	191570	165460
263660	496350	380380	355060	388690	534220	252990	299620
121080	65342	249250	232380	256510	153410	72327	95782
0	0	0	0	0	86834	141360	224060
143440	108350	130560	49054	41951	172080	128160	142200
252220	261690	302550	332040	416300	212590	99070	101490
167960	362330	311830	344770	0	341760	303710	454400
325160	262920	419600	312470	547750	209900	101730	158290
0	54230	71352	68480	261210	43419	0	0
0	0	64644	165530	169070	301080	273260	338460

LFQ intensi	LFQ intensi	LFQ intensi	LFQ intensi	LFQ intensi	LFQ intensi	LFQ intensi	LFQ intensi	MS/MS cou
11281000	13085000	13435000	12840000	15353000	7149100	4838900	4604400	7
1120100	965520	485000	635340	513220	718010	932150	1932500	1
57716	42715	68677	73925	71525	207590	242940	290690	0
1772500	2505600	2560400	2604100	3041900	5024600	5227700	4167500	1
2166300	2478000	5422100	5614400	3587400	12977000	11754000	9969800	2
662940	707410	1558900	1102400	2533400	2093900	2589400	2538200	0
1143600	777400	1949200	2575700	3274000	3990200	4338400	4127900	2
8095800	8965200	13493000	14946000	16414000	30296000	35782000	39972000	8
1471900	1221700	2188400	2626000	3025600	4578000	5240700	5438700	3
3372800	3421600	6377800	6997400	9809300	16025000	15135000	17285000	2
50291	0	48382	48894	48655	55343	54598	101170	0
184120	170290	282650	393620	426810	143810	73615	0	0
207560	98985	0	0	247320	0	0	0	0
2140700	2203700	2128800	2114500	1797400	1250300	865950	740310	5
166150	107260	0	0	141040	166660	97833	135330	2
1048300	779210	347560	274270	689110	768440	861300	1885200	1
529810	504930	338440	419500	705110	583400	751050	812270	2
756940	456990	371800	378480	487940	658450	770380	550910	2
214020	482350	141860	146580	253470	365200	265220	319440	0
1085500	1052900	491280	841780	1046800	1197800	1206700	1424100	4
507190	514500	381610	403630	196750	582390	680700	813600	2
237400	210200	87024	96616	251630	332810	399990	645530	0
131180	103010	0	100800	0	185800	124660	119030	0
540440	451900	412570	400880	620310	695360	1189600	1086100	0
189110	214630	148240	200330	67346	269850	952600	640760	0
325810	232650	220830	282420	273370	759820	502510	739730	0
96048	68562	55342	43416	0	89147	136950	136560	1
858200	1148000	646810	775740	838590	1146900	1192800	1454300	4
438270	410880	337880	327840	413190	574000	471630	524500	1
0	0	0	54462	0	133750	0	108020	0
0	0	0	0	69758	57423	132450	189870	0
103410	161520	152300	113440	148080	188460	202390	288000	0
72664	46291	50613	60428	151500	158530	106360	144940	0
0	0	118720	118780	233710	386060	788470	901190	0
285140	207930	82086	45938	139350	58250	59770	59490	1
329980	278590	252750	242360	246920	193280	151200	141530	0
693010	402080	587560	578880	442870	250690	172490	252780	1
886990	867790	792580	739870	979090	435860	322710	400520	1
394950	335920	283490	224420	287010	149100	146180	124080	2
108600	75329	49493	0	59553	169670	49590	0	1
324210	362250	316810	275140	228730	144190	99288	88952	3
91689	0	195390	134100	34114	98917	57660	55606	0
603210	289970	237630	168430	613780	165780	113820	194130	2
251260	213150	158880	169810	140910	255990	0	98112	1
90968	0	85539	233830	137410	64336	47445	0	1
116470	83898	119450	199700	298680	138690	67721	0	0
105420	134570	120420	155750	88939	56498	47740	42890	0
113030	129400	104710	144700	0	108040	128540	60236	0

488470	551060	416480	491150	253910	315900	242640	248170	1
73288	191390	113980	110950	0	107170	84270	59805	0
2774700	3125400	2540000	2497900	3150300	1631300	1226400	1288500	6
112380	89452	130890	100330	242930	54839	44098	30477	1
195780	211660	239110	343120	459930	162580	131850	131540	0
447670	589710	553540	529890	366600	217580	215550	197910	1
213850	155200	69218	102780	188170	64295	0	0	2
164030	165920	221020	233800	197100	113320	0	113210	0
258080	0	526500	170520	502430	131410	61895	205340	1
243500	159280	103960	105640	88493	75028	66534	47323	2
154060	128900	122140	106500	453970	0	0	0	0
135470	125290	71475	87854	144700	64885	65575	61389	1
442630	178840	157960	164980	89479	84367	86401	91194	1
0	155260	0	206530	0	0	0	0	0
115360	77876	93681	102120	214460	48325	41505	0	1
215900	436840	305610	311060	271980	150140	130650	101180	1
118200	65730	64014	63185	144050	52995	0	0	1
163300	157100	75931	71298	113630	55575	59811	50964	1
241710	139940	183750	123180	0	82386	110810	0	1
119250	111100	135930	133120	246960	87537	45374	39435	1
85517	104820	95964	67580	0	74204	55066	63928	1
1863100	3811500	2509900	3160600	1177900	1374800	720250	723200	3
3310000	3451500	4051200	4684000	6096700	2225500	1265700	1767800	6
252910	254770	274450	324470	365590	117750	113140	81319	1
291300	274830	223570	214880	97670	145600	0	93028	1
154950	0	188400	242400	189910	366260	358700	480680	0
835600	627420	493100	240780	0	382490	767900	941670	1
5106400	4756600	2914400	3041300	2482500	2729600	3684400	3808300	4
3453100	3149500	1703200	1448800	1372600	2677900	4103400	4135500	4
1312200	2244900	1258200	1731100	378440	1946100	2493200	2264600	5
5810700	5170400	4634400	4366900	4300500	4381500	4104800	3762300	4
2290400	2794200	1560100	1135000	411430	1464400	1851100	1440300	1
2562100	3620600	1684800	1301000	825370	1401600	2155100	2219000	3
4025200	3240600	1576000	1420400	2456300	2362800	2549700	1499900	5
5321400	5494900	2957700	2723400	910660	3423800	4014500	3548200	4
1650400	2578400	1192800	1326300	435310	2573600	3488100	3970800	3
6516500	7069500	3724700	3717900	1127200	4015100	4185800	3278200	7
907430	1023200	593080	469560	753890	911150	806240	894660	2
441630	432230	666820	406020	513210	1326100	1253900	1114900	0
1208600	2029000	1097200	539600	400370	743630	1168600	2202500	1
1405600	1234500	886950	599680	814490	820760	1126700	449460	1
997420	1301800	712930	783630	662780	1212700	1303200	1158800	2
2161400	2253500	1267700	1052600	316680	1355900	1795000	1072900	1
3251700	2954400	2070300	1671100	1823700	1651500	1954200	2280200	3
754920	1194900	1860000	1675000	2076500	2104300	2891300	5914500	1
185230	183950	175180	166510	121350	400830	398680	0	1
11772000	10900000	6204200	5461500	4816300	6769900	7638000	6342200	8
146810	278050	371890	456040	219640	919200	1076500	830220	1
6462300	7688900	3686300	3157900	3845500	3529900	5861200	4530500	5
8033000	7081400	3198500	5073800	1239000	4068400	4996000	5729700	7

2611900	3702100	1433800	1487700	753990	1747300	1822000	2526000	4
5829200	6452600	4241000	5855200	5752000	3482600	3698100	3365200	5
1177700	1742700	1502500	1478100	446760	2153200	3338500	2040100	0
12251000	12299000	8073400	6657400	7295100	5602000	7434300	6790000	7
6292000	9305400	5083400	3953800	1380900	4731500	5296400	5004100	5
4189100	4410800	3705000	3554900	3859200	5545000	6788200	9807300	4
29413000	36134000	37791000	37206000	26249000	26200000	22909000	26502000	19
174150	168010	137640	87095	81168	144000	316770	0	1
102320	59370	105310	58626	80268	64386	55811	58917	1
66335000	70540000	71911000	65089000	64825000	43908000	24195000	26086000	24
3927000	5481600	6551400	5415900	2607300	6262900	7934900	6931100	4
155580	2970900	1573300	1306700	0	2240800	5182300	1606400	0
4626900	7483100	6609100	6856400	1093300	5683900	7634200	9532100	3
7913500	8359600	4931000	2569000	4810000	3325100	4034800	2729900	6
2646300	4494000	3034800	1466200	1992200	2397400	3462200	2809900	3
4267600	5344800	3636700	3259800	2869100	2191200	3239900	3164900	3
75738	78787	53098	54600	71479	33367	50158	0	0
3606600	4057600	5945200	6336600	8978200	6698100	5930200	5672900	3
4672600	5483600	3893100	3829600	3385300	3639400	4126600	4227600	5
2841400	2960600	2522700	2601200	1971200	2603600	2526300	1953800	4
5246400	4130300	1918600	1209300	861530	2319500	3726600	2622800	3
5667800	5607200	3887500	2527300	1865600	3103800	3904800	4090300	5
1572900	2636500	466310	459150	463720	1085300	3092200	3859300	1
5218600	5242900	5185800	4684200	4492900	3042000	3461900	2904000	3
4462000	5009900	3600900	2830400	3294700	2526400	2533400	2616200	4
1510700	1720800	491930	135620	147520	407580	1574700	837350	2
2457300	2631200	3251000	3109500	2959000	2958200	2538000	2380700	2
4625100	3895200	2487000	2751200	2723800	2758300	2602600	4612500	4
1473400	1729200	1494200	1724500	1291800	2455000	3155200	2530300	2
3875000	3656400	2279600	1968800	839310	2309400	2711700	2100300	3
2772600	3436100	2728800	1893500	2958100	2614400	3828700	3359200	1
2523000	2609100	1175700	1613400	1246100	1094900	3694000	2063500	5
3200500	3522200	2108100	2437300	286010	2461500	2822100	2620100	3
3990500	3896200	2069100	1766700	2174900	1729200	2394700	2113700	3
0	672460	543380	468890	477810	727750	1042200	1380700	0
8483500	10195000	8144800	6836000	6162700	5354200	6394500	6653400	10
5412500	4921600	1939900	1386100	1755900	2185900	3443700	5000600	4
1573000	1793000	1950700	1511800	2242500	1580200	1633100	1456200	1
2218000	2945100	1883900	1792700	980930	1789900	2517600	2138600	2
2770900	4985900	2587100	2391400	1249400	2631100	2771400	2982900	3
735410	982770	609740	850400	104120	619760	1197900	2455000	1
2513600	3129100	1546600	1389800	1963000	1621000	1694900	1709400	2
2875200	2565000	2229600	1918900	1113800	2277500	2290900	1966200	2
1494000	1504400	1160200	1186600	1354600	1235600	1474100	2642100	1
712540	947340	1062700	978450	991340	701900	1260800	1182900	0
193870	156390	207310	201800	272440	133360	212390	382030	0
9441200	12004000	10728000	7856400	8393100	7660200	7230000	8652800	9
3424400	4648600	3718600	3511900	1360300	3562500	5185500	5302200	5
7027300	5787300	5062300	3689200	2057800	4287200	5032300	4396800	7
5725500	6452600	3423900	1630000	2275600	3568000	6748900	3804400	4

7511800	10317000	5892600	5501500	7181500	4854600	6494500	5439000	9
166440	166270	146220	106750	138530	79950	75040	56173	1
3507100	4121000	4261500	2826100	5216200	2903000	2518700	3613000	2
2263100	1642300	1096200	1536700	1737000	1067800	1819900	1341800	2
0	105840	0	0	0	174050	349930	371060	0
290350	555380	957770	910570	706720	3326400	4465300	4073100	0
0	0	0	78745	91696	190250	0	0	0
0	43427	47463	46599	44355	63546	55580	128340	0
43110000	43504000	46193000	48615000	56383000	30055000	23197000	24057000	24
2611900	2947100	2557100	2930300	2838100	1604700	1214000	1356200	4
503550	0	0	0	0	296400	228070	204700	0
165530	73353	90002	82835	112490	83138	79334	106410	1
0	143890	151950	0	273410	0	69081	0	0
144430	216200	187900	0	120320	448850	763350	401680	0
1787000	1440600	1614000	1896700	2009600	945620	514130	596680	4
205330	151430	97558	0	149350	151540	116900	98854	1
51593	0	70498	55449	0	1342500	1917700	2282100	0
0	0	0	0	0	262420	577070	1129400	0
1589100	1251100	1504700	1449400	1841800	904360	566580	691770	2
1582800	828780	1637600	1910400	4274400	2405200	2586000	1255900	0
92559000	1.36E+08	1.31E+08	1.64E+08	1.43E+08	2.11E+08	2.34E+08	2.24E+08	31
25873000	23597000	15917000	9185900	2361300	21802000	36440000	33973000	2
400180	362540	697460	798070	1170400	597820	619940	556290	1
0	180730	262520	241810	376050	423330	641700	509810	0
152080	165930	206710	271370	336920	280050	431950	423490	0
114260	104000	114110	173830	138810	455400	525900	371520	0
0	0	0	0	0	0	275980	0	0
148110	162160	244570	356610	552180	428380	686640	743370	1
156330	93597	152280	118830	154870	489780	141950	272180	0
0	14808	13222	16177	0	24494	25868	403590	0
503440	401350	706810	814090	1205000	1143100	1442100	1334100	3
429790	444130	403060	583690	773970	1365600	1035900	1079400	1
78194	161190	189080	381440	316800	1096600	1144700	1026000	0
0	0	0	0	212420	0	0	0	0
94279	337200	247180	116620	134410	102890	81574	73221	0
1792400	2841200	1834300	1508100	941690	1903600	2806500	1544100	0
1291900	1193200	1136100	1210900	1065100	759920	373210	544410	3
1695700	1358300	1100900	1423100	1357400	568430	560780	586110	2
189220	146120	449140	227320	260530	2964200	3394400	2815900	0
159100	145600	135040	220140	265100	0	0	0	1
183740	127890	170240	206470	375060	118380	63096	94427	1
1665900	1368800	1561700	1452900	1958200	763450	452640	575760	4
206390	282810	248710	390160	375690	407020	327530	383920	1
96886	102470	98242	114550	93654	272180	242400	233430	0
170520	260020	109470	0	0	121310	0	89947	0
188030	311430	235020	0	312800	292130	291070	209560	0
245420	271410	192590	0	214220	152300	148710	154050	1
78360	0	0	0	0	68081	189950	198370	0
1346800	1228600	1555300	2329900	846110	4156500	5201500	3996200	4
723960	422510	294380	376160	561720	514380	503060	584400	4

192350	196560	347110	305260	492250	187350	106300	0	0
123050	133750	211600	292820	196640	416020	708870	607360	0
0	0	0	0	88883	64658	98486	203570	0
0	0	224860	365060	0	894080	826650	600960	0
602350	708120	1557700	2011700	2355600	3668400	3038700	3442100	0
2106100	2276100	1649600	1312400	1586900	1452500	1036700	820100	5
0	0	0	40428	0	204890	97419	221410	0
20043000	23327000	16136000	11764000	11005000	18895000	13815000	12473000	10
2569900	2398100	2189700	2117700	781720	1460900	1099300	1081500	4
270840	304960	384490	360880	539280	225150	160910	151060	1
2132500	2180200	1289000	1244200	1431300	2848800	4759300	2763500	3
427420	415590	347370	353910	230800	450720	403730	457460	0
135840	107690	0	140360	114930	143000	130170	115900	0
406850	242330	282820	292500	169910	256030	474450	706940	1
317260	330680	553580	474300	529090	243540	175690	182990	1
0	0	57404	72663	138410	124390	210690	183480	0
108350	229980	97400	169050	377890	82756	57766	64042	0
730780	480170	594980	590200	740570	173330	165800	199860	2
0	17163	23547	39660	256400	101620	86944	221840	0
0	0	0	0	0	0	0	0	0
191970	181550	192810	208640	0	55555	27541	37013	1
629690	562650	237120	398750	447300	525520	609010	500620	5
0	0	152230	118540	236930	344610	331450	191770	0
85554	77529	135810	283020	159610	408270	377170	391210	0
1894900	1895700	1699900	2235500	1694200	3727300	4511500	3941400	6
208650	305130	158220	193330	129370	173060	0	89649	1
2755200	2477000	1827000	1855400	1214400	5296300	11631000	7533800	2
0	222000	200790	160980	0	382850	870080	1834800	0
42333	39408	16277	0	0	0	932620	0	0
74541	78478	121090	164410	135840	616410	1104400	635570	0
2148300	2151100	2428500	2985800	3024400	1763700	1045400	810270	3
231240	135830	133590	126380	0	0	0	0	1
0	0	0	106530	158850	199620	225050	166860	0
202940	149940	137960	135140	134780	106990	117670	0	1
1714900	1699500	3841900	4141600	4296600	7598300	8418600	7465500	2
670920	688810	1562300	2090000	2388100	3084700	3850400	3043700	1
405640	273860	451080	426460	351890	364150	225150	404630	1
160220	162110	0	0	165710	294260	309060	234240	0
0	0	96914	0	0	0	0	196420	0
8104200	6716200	15524000	22657000	25680000	49833000	49062000	46624000	7
162840	117460	133190	137250	62592	63654	0	46529	1
0	0	0	0	2284700	0	0	187390	0
407590	476450	397940	430640	326790	488700	506680	211580	2
36445	19832	0	23592	18302	38654	94090	68405	0
661100	705940	547570	632210	868590	397130	328430	272270	3
926570	584580	305090	262290	443010	865390	914330	1156200	1
1044200	590710	383850	541920	615310	693410	733910	681610	3
152480	155130	139150	158930	279780	498170	482330	383850	0
234630	279550	283820	230750	462700	211640	213050	304710	1
1442900	1083500	1631900	1393800	1317800	1154000	1111200	1193600	2

922340	569680	901160	878280	1469200	646800	395350	513080	2
142990	0	106760	135640	220780	0	0	39844	1
24073	20753	18813	0	0	0	20744	112430	0
19226000	16399000	35275000	30089000	23807000	37505000	40215000	34149000	11
24862	29150	18165	0	0	35046	103190	216060	0
352650	541030	518840	736360	978550	579120	496810	431120	1
1.05E+08	1.16E+08	1.12E+08	90860000	88515000	62935000	86702000	76485000	27
991940	769290	1649300	1822900	1484200	1998500	1852800	1932700	1
1134400	967570	1624000	1348300	1150900	1420600	1188900	1345700	1
1512600	2032100	2840400	2328000	1573300	3377800	3335100	3423400	5
673640	977730	903980	895540	948370	944170	1073400	771340	1
3214200	3234700	2589900	2077000	2332700	1846700	3283800	3079900	6
222050	451090	1418500	1160600	1836900	370080	346300	1257100	0
194990	126640	198470	158670	315690	86669	53437	60452	1
128540	126130	367490	331880	333090	696620	603800	750640	0
75071	0	83364	85118	114880	112120	110410	96211	0
0	56761	110180	158470	101270	105410	105690	168160	0
0	92252	64880	51930	0	0	577870	1260700	0
101240	78171	166600	99421	285090	103010	109470	182340	0
0	33203	0	0	0	28644	0	101770	0
0	52127	0	0	0	47487	52655	355350	0
73009	50652	44090	51008	0	57192	74621	60235	0
1096100	1038200	1407000	1929900	2292300	1093500	666560	631910	0
50195	63636	47100	50631	0	40889	31328	32965	1
290040	357240	298620	200300	287200	145170	111420	195250	1
223200	0	151880	155400	473120	0	114140	0	0
0	0	136140	0	0	385000	736320	396320	0
94476	80525	223420	244300	448330	184570	120250	125490	0
1784000	1648700	2461000	2112300	2601600	1478900	965140	1209000	4
497060	544280	808980	800610	1053800	592670	642380	619890	0
0	0	0	35506	44536	41734	46054	36985	0
686670	475110	126760	156970	102880	386920	760710	437720	1
558930	831700	631610	563120	590680	613000	655420	581570	1
661750	444930	472170	535900	558010	1183600	1432300	1305200	1
0	0	60394	46141	64111	581760	693130	384400	0
9555300	10822000	10684000	10413000	11344000	7003300	4560300	3954900	6
2377400	1645300	1023700	1456600	1552000	2605900	2622400	2344500	4
137470	132200	111970	0	56866	0	49933	143900	2
1530600	1273200	1052900	1165900	516840	856860	646210	754860	3
29587	31514	46451	42326	45077	244130	252130	203630	0
70333	0	72400	84019	92268	117220	95318	0	0
446020	472440	489680	544010	787210	272420	234420	266740	0
837000	466040	0	165300	227600	211180	217390	262420	2
2040000	2627300	2106000	3160000	1206800	2049400	1718300	1431100	3
608670	1062300	716620	439520	104680	362590	405780	374840	1
31723000	30575000	36253000	36115000	52527000	19482000	11112000	10175000	18
37390000	40030000	41338000	41926000	35576000	23756000	15643000	15710000	28
2936300	2743900	3276800	2831100	3493600	2136000	1042000	975990	4
1850500	1666200	2152300	2255400	3298800	1206800	636190	715010	2
897440	676450	782720	877310	1140400	373820	254790	186360	1

645290	1378900	665470	297390	0	573780	902590	0	0
1221100	1532400	1413900	1299700	262740	693220	427230	759950	1
3690500	4086600	4290200	2578000	2385600	2274000	1796500	1728200	8
3263900	3705000	2111100	3908400	2281600	2723500	2144000	1526900	3
2755800	2263700	2440500	2438800	1183400	1525200	958660	644740	2
1047700	865390	1064700	692110	1005200	784930	491560	344980	2
55112	73132	65654	75710	43463	0	0	47589	0
156170	265210	149160	136680	144250	108350	163270	103240	1
126550	109700	109590	124980	193320	317070	223510	186020	0
175660	107370	0	121720	0	207820	187430	179090	0
370260	146100	95402	98338	0	58747	95745	132550	0
165540	56473	0	0	39347	45081	43983	42194	1
1753500	1008000	2808000	5214800	3853300	6034400	4803700	6049800	0
543320	455490	183080	194490	141110	268450	345570	262030	2
186220	165320	0	0	90618	160510	179870	163650	2
2159500	2120300	1254800	1435100	1031200	2208500	2101600	1913100	7
298620	370010	389850	340340	434590	144330	91180	150330	2
390450	159900	491280	291820	796870	207670	123820	0	1
114880	95225	81051	88522	0	288830	233920	155510	0
4424600	4365000	4739600	5032600	6820300	4543800	4101000	4052800	9
197970	332510	284570	369470	501920	913740	810700	746510	0
1162100	1015300	1639600	1595200	991010	1292900	1088100	800770	0
56773	52005	0	37583	66233	100480	111890	93081	1
678760	665740	1825900	2137200	3715600	540490	456940	202060	0
159970	185420	82217	0	0	176900	219230	244620	1
2146800	2426900	889100	1208400	1264200	1517300	1839600	1736700	6
0	0	0	0	0	0	80024	113460	0
193470	331340	311020	349790	728150	131750	180410	144530	0
118190	95813	152810	121450	229450	64301	85504	85757	0
0	0	41544	41186	120350	0	33012	0	0
52107	0	77331	114500	0	89916	57349	453720	0
122860	142280	205200	195330	534270	243130	239710	303090	1
48061	57420	0	0	0	85866	148700	80077	0
3115100	2083200	2043000	1107300	373530	2212800	1217000	4022900	0
0	245820	0	0	0	0	0	0	0
84687	65768	96419	95657	0	282420	346260	502130	0
0	0	39160	39920	78695	192850	380680	225740	0
2653700	2438800	2836000	2427900	2476700	1487000	1528100	1297000	5
1278800	1189800	1565600	1519700	2060200	788350	462140	547890	2
287350	63819	0	64792	85985	0	44258	46472	1
351850	569670	1063800	867910	1293400	673660	444370	482590	1
276950	260340	549180	528060	664180	447720	188280	255980	0
1092300	1372300	1262100	1548300	1507600	639000	922020	1327500	3
91205	107640	80675	88966	111090	71914	69344	70450	0
0	133670	0	0	0	0	69866	108130	0
93526	95632	112640	201910	143570	98861	87165	205240	0
0	0	96959	0	182840	357110	313170	303830	0
137580	152110	100790	118590	73309	128620	234320	144420	0
3289400	2085600	1327000	2010500	2691800	2964400	2945300	2960200	6
194440	101320	142390	0	209170	97639	86537	0	3

815090	434440	543360	609710	599300	1901100	2330300	2024200	1
61064	31102	45368	550930	42538	22437	0	37870	0
161260	0	164740	128340	239670	364410	528570	208130	0
198720	179980	247680	204820	227540	174200	148280	143610	0
220980	262970	287790	306180	361620	354880	463030	540580	1
0	0	0	0	0	0	26158	85602	0
36034	43779	41658	0	0	32688	228100	614260	0
262980	386240	363330	422600	248060	269430	138370	196640	0
0	644470	0	0	0	0	435950	498560	0
117250	0	0	99589	196610	336240	182920	217830	0
45456	47876	0	29555	0	28335	31108	0	0
92705	174710	163050	161130	167180	476850	638470	519800	0
2262500	1913400	1058900	1470200	1141900	2108500	2247200	1850200	6
1597100	1384400	1499700	1252900	1603800	796850	599290	679400	3
53114	55578	71187	44035	121050	36332	37019	46725	0
2311100	2137000	1439200	1445800	1232600	2101500	1656500	1619300	8
81563	0	500850	204260	316870	238040	157850	473080	0
782050	679150	943310	1140300	1288200	336220	226960	264950	3
308580	289360	337890	390900	666470	188060	123810	371170	1
0	0	0	0	0	0	301880	0	0
5900400	5848900	5811300	5801900	7455700	3505100	2492600	2405000	13
909500	1442100	1111900	1053600	379620	727240	391360	550830	3
2403100	1972300	2196300	2764300	3571100	1664500	1145800	1303700	2
247230	244020	442900	602150	609090	1389900	1517800	1700300	0
18217000	18348000	14433000	12649000	9794900	11269000	7589800	6852100	15
364500	396050	488740	657560	471070	1013100	972150	954060	1
336410	285340	478510	463410	585010	788610	723270	858420	1
295980	276560	237030	211590	102120	357940	813340	1016300	1
0	0	0	183060	131230	440940	305660	329770	0
166980	71921	275470	308000	434950	851230	1430600	906920	0
19656000	19386000	19853000	15072000	15861000	12165000	9862400	9517500	14
280270	0	250970	239400	443990	258640	276810	211020	0
402860	188370	458710	617410	796860	377420	147000	659200	2
92105	89576	42251	29322	53910	67805	108310	93244	0
162210	195260	256770	370010	288570	654330	823760	646160	0
264030	206550	354680	488790	660170	1820700	1696600	1770900	0
117870	189330	431010	780370	548180	1408200	1798800	2135500	0
664980	347930	689090	710910	809120	240810	223760	542010	1
1628900	1450800	1534000	1654000	2089700	956810	653630	727610	3
195500	232350	266350	223330	346880	211700	205870	264210	1
232470	246310	297850	299340	178630	190730	164560	365250	1
245030	79882	49778	0	0	0	0	114790	1
234830	249170	194710	205290	0	196840	306830	247610	1
114860	102030	71027	78771	92269	84130	75636	90204	0
0	0	0	35224	0	57953	83292	0	1
769780	839140	891940	863240	879970	505410	341120	365540	2
475150	769830	116760	54759	0	329280	1110400	1271100	1
942040	1033500	3286800	3249400	3110800	2128600	2502200	2328600	3
223090	220380	537790	530440	654650	420950	373120	318570	0
1821600	1795100	1970000	2083900	2167100	1505900	1833300	1731500	5

435850	285220	475760	502990	337380	385410	390360	384810	0
941320	796690	918270	618820	1091600	488330	327210	337720	3
449370	511050	1059000	901330	915240	595380	488740	520310	1
5915400	7585200	6424200	6176000	6045100	4364400	4351300	3737900	1
93311	216250	155330	102200	196250	52721	41221	44745	0
105930	78210	214080	238960	156640	41013	0	60498	1
748820	460420	886930	1019700	614900	475380	406430	529380	2
275920	393280	536160	557450	1563800	290930	199040	56703	0
2217300	2218300	3759900	3481900	4938900	2026600	2202300	2099800	0
1215700	1269800	1085100	627200	701910	616550	823840	805670	2
160190	177770	213850	192090	266620	325740	192510	250870	0
270160	327710	324440	230050	323680	206100	147420	247210	2
181760	295590	247320	333100	0	202770	107080	243880	1
0	98850	118780	155040	425560	78276	54118	58876	1
167260	301220	66317	69737	0	211370	122530	475380	1
328800	579620	637610	716780	509640	697520	1278800	986910	0
55773	95181	0	70031	110200	157560	182770	248640	0
127390	80040	160750	188890	259590	130480	116630	319310	0
118900	83500	296320	457620	603750	1325600	1589400	1206700	0
0	0	0	0	0	0	0	0	0
117890	118180	193490	167980	356690	60225	41580	58222	1
268210	475320	321420	151360	0	157780	249790	253410	1
46368	127850	61339	55398	0	47564	44343	55506	0
553420	817810	649590	618780	749990	712660	713190	771250	1
998990	929290	1166000	1629000	1884000	4807400	3871100	4254500	2
313130	241830	380940	324460	396810	224110	166090	145460	2
0	0	0	0	320610	0	44525	44227	0
80963	200920	130840	134500	0	59020	56782	0	0
356260	399640	96976	74504	0	119570	0	108520	1
23137	44100	209990	161600	328620	187690	166270	130510	0
668010	684350	1569200	1461600	2486300	1491500	1847700	1235600	1
176000	117940	115330	123580	368570	156760	175920	105880	0
4041200	4736900	5493000	5211300	5767700	2798900	1947500	1695000	6
8076300	4741000	5287500	6148900	6942800	9148500	10866000	10859000	14
475970	206160	436620	572470	1073400	627250	544230	487040	1
37457	73264	98607	94186	94539	194900	151480	225120	0
295950	487570	552290	264130	67865	192400	193730	117530	1
1193800	1469000	1530800	1799500	617470	1441600	711750	874300	3
182190	101270	447410	953060	482780	204590	52016	195220	0
780820	604110	1094000	1060400	658350	531630	372120	411230	0
107480	305380	168540	116020	0	392820	498490	556500	0
238420	242860	212220	179140	227010	334410	292120	181420	0
0	68409	0	0	0	0	67868	66725	0
0	16829	18859	18624	20539	92196	111930	101840	0
433230	359230	411470	271310	515080	250930	297270	177410	1
261600	199690	179930	204160	277450	133850	126520	154820	0
0	67944	0	53488	0	0	78880	120600	0
796560	452040	1080100	958300	468110	2769700	3495700	3149700	1
86370	60280	54736	58548	58881	0	0	0	0
973550	538700	501440	343280	735580	759000	815700	1139300	2

232630	144920	141600	162870	465090	201730	303730	0	1
1392100	960000	881720	1120000	1161900	1337100	1402600	1030200	7
373540	192350	168810	157850	245120	440960	384130	509470	1
188510	134210	155380	149230	144260	238370	233620	247550	1
268880	253180	214960	0	217670	242150	313780	329530	0
3077700	3979200	4483000	6491200	8671700	17098000	17576000	18801000	3
0	0	25292	0	124940	0	0	0	0
789020	820040	661830	880910	1180700	538970	379510	258210	3
126910	112800	143940	129080	152700	87722	75156	49734	0
63773	98343	53793	44441	0	76212	76109	0	0
0	43651	0	56017	74152	0	51332	65297	0
662660	598430	449980	440710	552630	393450	515150	368690	3
214990	188730	121770	131920	167900	248590	339620	446840	0
1232600	2096600	2388000	2078600	2582300	1163600	1054700	1322100	3
11392000	6200600	8627400	7832500	7711400	5195500	3137400	3097600	8
0	0	0	0	0	0	0	111030	0
111310	117520	115020	89764	137630	187030	177070	216670	0
122820	46616	35956	25230	42599	116320	77811	136560	1
1672800	0	0	938770	0	862470	0	0	1
865040	877720	406390	417580	468590	593330	678930	659770	3
80487	0	65823	76369	67253	0	85282	106730	0
0	0	0	0	0	0	0	196710	0
81036	76273	92272	109820	143510	115570	97136	124200	0
2122000	3550200	2460500	1979500	2222800	2649400	2500800	2124700	4
982980	966290	1221600	1712800	0	0	0	0	0
2429200	2748800	4952800	5132700	7033300	2908400	1894900	2382400	3
65347	98111	131770	304600	466780	64647	58199	0	0
126890	0	121070	116330	146160	276160	195050	164140	0
205250	172900	556680	619520	1045900	760690	1159300	1272700	0
23828	26886	35561	30431	73034	28499	0	36975	0
0	0	0	0	0	0	411550	98730	0
183250	169420	262330	293620	441850	174710	88076	113710	0
0	281430	139350	0	98177	142770	285770	296380	0
0	0	58088	79697	111620	165490	157940	226170	0
0	0	206230	0	0	913710	707440	959400	0
353290	197700	115180	0	134270	151470	176360	176550	2
1020900	835020	498010	493410	678230	736370	695100	582130	2
114820	122310	169060	115040	160990	96480	91727	66980	1
0	117030	0	0	0	78891	127670	97951	0
0	0	0	0	47786	58463	75622	49641	0
0	0	107810	187730	592660	629520	643650	619170	0
252680	237920	285510	284810	297470	257280	246420	278650	0
411460	437540	425610	353100	541420	432890	504620	488660	0
0	0	0	0	156590	149760	111410	125950	0
122020	108830	316100	262540	515560	1045300	936640	809420	0
63329	134270	51027	0	56295	0	54500	0	0
111740	147180	248330	350830	553550	866130	1164400	1370400	0
133030	67598	154090	189600	314880	483170	484900	268110	0
67496	57474	66074	52611	57358	54517	55168	0	0
390110	230050	358120	372700	344740	163810	159760	136930	0

0	0	97580	0	104910	203520	213210	1143700	0
77139	63851	91407	63637	83568	52904	45451	51875	0
118950	122780	239160	228970	234510	0	0	0	1
1506000	1861400	202870	208580	229430	347110	337900	217220	1
5832500	5513600	5794700	4853600	5665200	3426200	2713900	3192200	9
3935800	3774900	3577200	3571600	2722000	1998100	1397300	1587800	8
359460	563570	945390	619510	702550	242960	245900	606290	1
584450	619350	678720	627690	821540	383800	284980	234820	3
551320	520200	640900	419270	51881	517640	174720	169920	1
832750	684260	1072700	983510	990320	553250	431850	819650	3
135680	98417	58072	57957	45652	88716	122250	133690	1
691600	645850	659050	733750	1252900	474370	351420	322330	0
230850	124070	93002	54393	71728	0	0	0	1
155550	98985	75145	72118	101840	78054	95655	106430	1
2270400	2045600	2497700	2813600	2456300	1336200	697950	1208800	2
2855000	4168100	3825600	2958100	3788300	2581200	1573400	1554900	3
1163900	1148700	1529500	2016600	1294300	987700	548520	553020	2
2159200	1689700	2161500	2170700	2662600	1491800	1002400	844910	4
734840	541940	481190	497780	544750	929700	686140	232280	4
430960	415100	406190	466090	246710	187880	176910	211710	1
1132300	1314300	1176000	1186800	266830	889020	624720	712330	1
455400	517460	198700	203900	292770	154400	182570	150180	1
1187500	2260100	1298000	1608400	1247700	1150000	681730	1250200	1
973320	873270	421970	260380	259410	530100	747070	605130	2
330100	372920	57874	18815	0	55020	316490	287470	2
395430	363810	380350	393800	405750	308070	249700	266670	2
2707100	2189800	1724100	2538600	2634600	3419700	3503200	3227300	4
0	0	0	0	0	268310	61599	66323	0
86517	76436	85207	117050	100840	248670	199280	338920	0
76160	59361	58881	126420	124040	147950	147160	245790	0
9420000	9460800	8159800	8807600	9776200	5300900	4027800	4148800	15
0	46010	78482	90037	109400	92853	77821	0	1
0	81696	0	0	49124	0	119010	214660	0
187870	124580	220640	295440	420970	686160	549580	348990	0
0	0	0	0	0	59259	90614	108810	0
1717800	1760800	1013300	1148800	1219300	2105700	1699600	1389500	1
56416	68780	37646	120290	134720	293140	616650	545530	0
115140	109790	141450	159330	173470	136850	99882	124260	0
0	0	0	116050	0	236960	338780	357760	0
147370	142380	189850	201850	146660	97331	75771	91236	0
61134	80753	122940	229860	0	1027500	1085600	1769100	0
152250	208520	157750	279210	292480	170210	97398	77541	0
327110	261440	448850	497440	493000	269440	239250	500590	1
1424400	2214600	1938400	1853800	799670	1472500	1016400	859040	1
904660	847470	777730	571140	641510	427120	338640	274060	2
106920	121400	137360	129640	99544	64357	61252	52954	0
2625300	2965100	3010900	3064500	2074800	1888300	1017300	1142600	5
0	0	0	0	0	161140	0	184550	0
53826	148410	195190	229290	255940	69665	74564	58656	0
0	0	0	0	0	0	0	0	0

271540	0	264820	452240	482030	890890	1030900	755290	0
227190	360420	151550	84729	0	132250	248990	145390	1
634800	0	58647	0	0	36487	84696	69513	1
0	0	0	124740	0	67353	49672	0	0
315290	242990	210710	397180	340430	151880	122130	126490	1
1994700	1892600	3060100	3577100	4941500	1821500	1697000	1552600	7
1552100	1337000	1919800	1773000	1807800	863650	970440	828410	5
6933800	6636400	5323900	4354400	4824600	1817900	3058800	3079300	19
603310	618360	622640	672340	1129000	1403700	1609100	1352400	1
81490	65349	57608	67918	145720	135170	238770	167750	0
1400100	1450000	1691100	1750400	1871000	1083000	447140	515040	2
887730	559950	630160	836570	1196800	387230	166540	290560	3
2400700	2651300	2675500	2619100	3130100	1645100	1044900	866800	4
0	0	0	0	0	54174	54558	282890	0
177590	175380	237700	234590	76118	128700	87130	136510	0
528930	487640	620320	634100	627500	317070	258550	260170	1
57599	59340	104240	74141	88206	53549	44121	44216	0
185850	206020	351520	425620	496150	529120	809030	783520	0
173410	0	0	0	0	0	44887	50418	1
844430	567800	231080	310480	243830	300800	682980	611540	2
283400	302740	138530	0	147510	469590	646820	805390	0
115080	168290	71276	0	0	112090	165840	161320	0
269440	197290	66193	45614	141010	77986	98472	275310	1
0	0	26826	0	0	64766	0	0	0
175170	115040	105960	60241	178080	901690	1245900	1889000	0
491010	472250	349520	284380	155300	864510	876990	1154200	1
1069800	739870	600300	448700	742290	1250500	2049200	1959600	1
268310	279670	192180	228410	242020	849180	926960	742570	1
2346100	2064800	794150	729100	624190	1388300	2182100	2194100	8
1632000	1541800	2988500	3642200	3341900	2465400	1634200	1610600	4
1306200	1476000	2039200	1775400	2059000	1118000	1001000	1308300	4
0	87008	68512	100820	87008	1553200	932540	964260	0
6987700	5089100	3115100	3038800	4826900	6258000	6871400	7045000	15
87346	75681	104060	245750	186440	84385	74769	0	0
0	51164	285130	294150	420630	190480	96730	126210	0
0	0	0	0	0	0	0	0	0
140510	160050	170130	159140	57902	65579	36766	45743	0
125950	137580	101580	137340	214380	105370	79387	114320	0
0	0	76568	101790	151680	93975	59647	88916	0
0	0	75735	49061	0	0	140270	0	0
371540	347010	97535	221230	152960	0	354210	371530	1
594130	621510	314500	262140	311100	141570	227790	379570	1
353950	337760	341450	420210	728650	144680	70673	133400	1
0	127420	226250	288440	227170	362600	404770	290900	0
1596600	626760	1498300	1286500	1676400	880890	362140	388050	4
68189	0	71911	62931	87216	0	0	0	0
108850	110130	155200	147270	191330	85851	0	114980	1
0	0	0	0	0	0	0	75230	0
137630	114460	131510	257370	221540	103620	0	70668	0
321220	98889	331800	312420	182980	107100	85991	49190	1

552540	658460	431920	286500	459840	480590	138960	704690	1
0	0	0	0	0	0	0	168840	0
611280	545080	574100	529990	415900	340430	187080	249180	3
300210	470260	347780	367790	608920	456600	539400	591840	1
2481900	2188300	1854700	2201500	2304100	1589600	1071000	988100	6
9587700	11027000	9138000	9517500	10731000	5430400	4063100	5633100	11
4914700	5049900	4649300	5206300	6865600	3051000	2000500	2499300	8
2180400	1837800	2083200	2458900	905710	1430500	781120	665510	3
1330000	1524300	1439100	1405000	620620	1084200	654690	625470	3
195620	491970	228820	132920	138760	66015	0	0	2
135820	0	0	148950	0	86602	0	50924	1
149140	113030	135150	150390	225890	106350	107570	128480	1
139910	112780	100080	59347	118010	114830	136170	114060	1
116770	130270	156140	172640	86026	80100	0	0	0
228340	174870	189440	231930	402180	300960	267530	235780	0
0	0	0	0	0	71090	88875	98267	0
0	0	0	0	0	0	0	104200	0
77645	106030	56938	67243	65129	48281	74476	70015	0
108050	114760	92284	90214	0	187680	196850	144490	0
0	0	107800	108360	148360	120760	130250	221830	0
286510	200770	95393	0	107630	100780	116910	182740	2
126180	272690	83910	62243	0	49280	0	0	0
798750	525780	796380	702720	999170	425490	694860	265180	0
147220	140910	466300	276940	628800	269980	294480	246130	0
0	0	0	0	181480	95761	185140	621460	0
0	0	0	0	0	0	0	82059	0
869480	466300	559780	285710	404880	248780	204260	0	1
2318900	1665100	2523400	2488400	3993400	1428400	1006800	1145400	6
148030	139400	122860	143900	177170	375650	737130	602680	0
122270	97255	116590	222490	181150	82914	86658	151890	0
241800	212130	205080	206740	301230	494120	299760	370030	0
1149900	733370	955690	850830	1397400	532090	386190	413090	1
0	0	0	0	0	0	0	39720	0
134780	103910	113210	125300	129000	63180	69442	74644	2
170970	138940	125210	131950	133490	151860	141520	129510	2
217700	207620	370670	517020	461300	932800	977970	793560	1
225220	250990	170340	189820	690510	269840	137210	121630	0
0	86313	118990	141640	86857	184540	138090	219280	0
692200	663460	300890	418350	401210	594830	456540	522280	2
756640	470890	668000	791660	726530	1107100	1175100	1722900	4
1522800	2044500	2249300	2035500	2513700	1433400	778170	787280	3
2595300	2842700	2843300	2935100	3581700	1536800	1155500	1204000	6
0	0	0	0	0	53196	101570	251890	0
1185700	1148900	1786500	2168600	1197900	5425000	5561800	6363700	2
1038100	1082800	2143100	3090600	3407100	5638600	5549000	5499400	3
0	0	0	50759	68176	76634	122430	124030	0
4077000	4197700	4421000	5537200	4812700	12727000	12441000	10388000	2
9218500	8713800	9704500	11596000	11803000	26346000	31741000	28860000	16
574660	599400	472910	789760	239820	282830	229450	282410	4
742230	1124800	725780	746680	688210	598700	296950	257600	2

10657000	11484000	10319000	10468000	11073000	7411300	4852100	5191900	13
441220	273180	342050	414890	558930	168900	115570	99458	1
256240	229020	179160	189060	277100	169200	121790	44505	0
208980	88787	80016	68392	78509	67343	75075	66732	1
569300	1022500	800040	815760	1194800	792080	639280	841300	2
576810	342410	381460	500500	828480	260670	119690	159530	3
0	0	0	0	84388	110120	0	80658	0
608880	782360	560780	653500	1053800	498830	362340	276170	4
315200	294880	350310	265770	503430	220790	103860	146530	1
3274700	3245900	3865500	3826800	3726400	2436500	1701800	1704800	6
2706200	2495800	2787300	2716200	3845300	1694300	1317200	1435600	2
456710	370470	242920	232960	259610	205480	153970	185360	2
14248000	14471000	13988000	14158000	14274000	10446000	7225600	6568100	20
0	0	0	0	0	78753	107710	127700	0
971530	1026400	2119100	2608500	3903700	1729400	1406800	1770700	0
2132200	2253800	3010200	1733600	2948700	1402400	692600	902130	1
345540	370610	475880	485490	868170	146100	119260	340760	1
2502300	2333200	2497900	2703300	1589800	1477300	854300	899650	6
66323	55867	100110	116280	172230	0	0	0	0
8354200	9731200	11588000	10658000	11140000	6411800	6130900	6556900	19
574790	806260	327070	669930	711950	232290	465590	485410	0
5467200	3944500	4511800	3256200	6290200	2461200	2298300	2540400	3
164290	152760	0	93080	173210	88468	100520	101020	2
846840	777470	964430	887060	532780	451990	497720	421420	4
165870	0	230240	238190	207500	130260	82873	107610	1
616800	879030	485830	740170	0	2180900	832830	462870	1
454300	0	110440	0	0	110430	0	64268	2
428780	435560	509080	447300	632860	257930	145830	189750	0
0	0	0	0	152880	0	35700	36393	0
152650	173890	121600	102340	0	110230	71004	95498	0
284010	356770	291560	343080	401400	229140	152990	91767	1
1167800	997040	1081200	1276800	1691000	797390	458620	397390	2
3480400	2657400	3319000	3218400	5084400	2727900	1551100	1678600	2
394820	381870	450830	0	256530	281530	205890	165190	1
0	38838	70676	70219	0	0	0	38553	0
47002	43212	49209	67526	60523	31812	0	0	0
2631000	2964100	2196100	1933400	1789500	7715500	11340000	10937000	3
413770	475310	420910	549110	380200	742320	776640	729330	0
257740	240470	214800	237710	380050	465930	571750	758600	0
0	0	78643	96081	0	339310	624160	1145200	0
81345	69219	66440	71821	200990	59428	209410	0	0
0	0	0	0	0	127120	350830	327590	0
0	0	192050	135850	0	460830	444160	233370	0
167020	228920	296740	328730	580840	824260	580320	729830	0
401450	320770	508670	928760	473130	1852500	1408500	1227400	0
424500	371830	432490	679790	1159800	1000200	950260	969810	2
0	0	59338	0	84512	128020	119230	88394	0
352290	198260	284530	458080	283620	812240	903110	1531800	1
86381	78747	89671	63349	29647	643280	2411900	919850	1
245160	195430	302930	368150	218490	750170	684340	779750	1

828480	487770	271360	399450	305750	928530	1235900	1031000	1
719250	467910	397100	479330	439330	1150400	1123900	1076700	2
149930	126090	136670	190470	135960	376860	366710	393330	0
123310	0	0	156010	150130	215400	392010	179450	0
105980	127120	99968	105730	0	353380	373880	444940	0
478550	419240	478240	466240	503840	942200	1148800	797850	1
0	38986	75688	124510	0	205440	173350	169550	0
0	50664	55779	58313	49473	121290	75810	405240	0
186620	83986	134910	274120	446450	682300	849990	658040	0
0	0	26618	0	0	114000	176660	0	0
1478200	1139600	819500	935600	1219500	2125500	2186600	2171000	5
96316	0	50370	0	0	106790	101020	62541	0
0	0	239720	261190	186980	776410	1123200	437920	0
180880	99454	290760	415600	438460	1478300	2277500	1842300	1
100850	0	0	112060	0	259450	327880	142360	0
1495100	1288500	2124000	1259700	1398400	4450200	5319100	3997100	2
161770	219250	169750	98873	127490	490700	698020	571110	0
550380	474090	592300	864040	1413400	1091400	865540	859110	1
43301	28264	28449	41796	94984	71156	77710	78202	0
146190	107450	108560	94039	310710	216060	159370	237660	1
0	0	0	28656	0	31970	38350	111330	0
222430	91131	105610	92896	291610	201460	127040	151960	1
126980	139830	0	0	72590	136410	155160	80141	0
167430	85767	188540	98193	188390	922540	459100	489360	0
578650	558140	1097800	2006000	2180000	4539300	7345700	4144100	0
77545	66362	54570	54094	74804	90693	74487	98010	0
1872700	1408300	1499800	1676400	1207700	895570	690610	848850	5
223340	321820	535760	742610	1304700	1538800	1577000	1590400	0
583430	749290	1529800	1272500	1316500	1427800	1231900	1094100	1
929110	1056200	1863600	1458600	1807500	1549100	1330000	1407200	2
770700	588940	823820	924090	819120	1778000	2296800	2318800	1
129330	113810	162090	146150	99077	687240	506380	859440	0
755370	447100	851190	891740	894520	2021200	2002800	1935400	2
0	0	63953	246470	219600	1071100	2303900	1384800	0
393880	182900	101340	494520	239160	1759600	3974600	2290900	0
1091000	902670	506290	461920	295140	651630	900080	919930	2
144640	143370	198140	229520	199350	562990	405520	466300	1
917210	970040	919080	950040	1152400	569460	320680	721400	2
1959800	2027800	3106200	4520600	3000800	6331000	8853900	8493500	3
4407300	3465300	4240600	5920700	8527800	8161400	7738100	7438400	13
0	0	187440	297720	390930	1120600	670820	925870	0
121840	202100	194100	200400	153380	130010	138890	162890	1
1705200	2259700	1706100	1748200	1647100	1230200	1682800	1033900	2
223610	159060	108860	154850	150120	223970	233110	223770	1
1937600	2420100	2153400	2062400	2659800	1459200	736430	752120	3
0	0	0	0	27736	0	0	0	0
18891000	17502000	20841000	26493000	33028000	37302000	38523000	36157000	32
3156400	2779900	4572400	6024100	8495700	6055400	6121000	6334000	5
52731	0	53147	0	82934	87247	125330	99653	0
1125700	915780	866210	629910	978160	1052900	1298400	1779200	1

0	46938	109000	103720	77782	389230	466620	842650	0
467450	491270	667820	980330	500320	404190	240040	646840	2
416230	417160	572850	634310	952170	299560	247140	278550	0
87380	0	102130	119820	155870	113640	229740	165510	0
815510	792210	359570	428770	507380	573210	694930	550290	3
198500	260530	260670	255790	281140	171870	115880	118610	0
42082	45909	54729	53151	93032	35801	0	0	0
3746500	3544500	9161100	12443000	10829000	28478000	32099000	34609000	3
308200	183150	841320	1265100	2176200	1086100	1610500	2160500	0
2000500	2547200	2812300	3213900	2809000	2304700	1813300	1887900	5
942840	587870	613350	665400	867160	374140	310370	246150	1
211380	302810	429010	352720	424490	284000	261760	238970	1
3103100	2459600	4120900	4865700	4964700	12067000	12323000	15483000	3
0	35020	0	0	0	74750	224070	154680	0
619150	428330	443510	370460	426700	176630	225150	308860	2
138830	163420	230720	277590	75758	270690	149250	107170	1
0	98705	105620	100690	149820	463630	523260	395760	0
1080000	1436100	1563500	1205800	1846500	1386700	1337600	1029700	4
163250	174790	282810	213240	148790	138480	99125	91483	0
4121400	4501900	1099500	1150900	573200	397270	295740	391800	12
236700	66555	0	0	0	0	55815	58701	1
913790	855910	441790	481640	628700	650340	587390	732200	0
0	0	45641	51979	66285	76837	67905	95488	0
0	0	0	0	0	0	42264	105960	0
0	0	40065	40271	57962	39027	40678	0	0
0	0	0	0	0	39035	43337	41784	0
0	52653	82866	109470	105230	416780	342180	488990	0
915080	544830	1052400	1315600	1503100	1913300	3024700	2443000	1
367270	425010	1111100	1537400	1159100	3834100	3325800	4399500	1
8313100	8968800	10099000	9538400	11202000	6456200	4779900	4124800	7
9236600	7717100	9445500	7797000	9987400	4657400	3219500	3258700	9
0	0	0	0	37290	47221	60636	92508	0
0	0	0	0	0	83510	86070	90232	0
0	0	0	0	0	138780	153710	151050	0
1229800	1670600	997920	905140	1210300	550450	998200	594590	6
465440	292080	227500	212490	261280	406860	462610	268450	1
307580	270650	157810	300830	473600	281970	407430	425400	0
0	0	108590	132900	138680	223530	182680	291690	0
0	214780	306970	277960	221310	167810	104920	124300	0
150950	119290	144650	171960	256840	369900	437320	311260	0
530030	478210	550020	594280	510780	244050	164460	163610	0
0	55077	100900	131490	142260	471370	627010	487340	0
2397700	1640900	2873300	3746000	3800300	11956000	10663000	10438000	3
37950000	44402000	22896000	23419000	13403000	47865000	70648000	62304000	10
85103	75627	89870	70386	0	35661	23567	0	1
1671200	1742900	1540200	1889800	812370	952110	672620	540410	6
556150	310190	660060	549560	875190	264720	151840	190920	1
302560	272650	283930	298460	405570	471300	332530	366820	0
136730	95686	96865	116790	95059	76528	128930	0	0
0	0	0	19397	0	67418	57044	66153	0

0	0	132870	122400	159590	199170	188960	406510	0
0	46458	58286	62532	36429	89944	48935	519210	0
573530	668000	734210	665380	1074200	205910	249870	328640	1
1303000	570900	1278200	711200	988080	3029500	1988300	1229900	0
877970	385240	453510	622420	807390	317740	334530	356670	3
189110	173990	81589	100700	244330	70186	46050	52518	2
94057	58139	69642	84874	226830	81121	0	323060	0
216270	436520	314570	436960	229270	375380	305140	398250	0
41887	43925	46556	0	59119	64962	80429	51976	0
86767	107170	158970	160960	164760	146670	122380	0	1
190620	220060	203210	235540	232190	137130	104950	110590	1
83697	80197	144600	103370	343350	88026	64990	123160	0
0	0	0	27255	0	0	0	0	0
221490	267370	0	382330	304440	0	128730	0	0
176880	146250	148450	211760	325870	0	0	0	0
122450	115280	0	0	0	0	0	0	1
461390	532940	647580	682210	568680	440020	221810	283070	1
60200	52198	157760	183500	167010	0	21472	149110	0
226390	130310	179780	156880	352290	161830	144430	189000	1
2250200	2134300	2349000	2664700	2355300	6312200	6703200	8526600	3
141400	135160	91208	0	116880	97090	108510	0	1
260720	209980	105120	91200	0	177220	193470	0	1
88163	74817	69779	62603	77992	119450	181240	111390	0
159360	206780	285590	353310	384740	149530	156550	150170	0
787800	744100	871910	680490	980580	495530	469710	499740	1
548710	571550	1319500	745840	919880	488650	427080	487930	2
404140	444040	982010	1175300	1202500	486560	314730	511530	1
2916200	2971500	5177100	3892100	4336100	2355000	2100300	3055800	5
1557000	1512900	1510000	1677000	1618000	971050	1038700	800120	4
106990	106940	157500	272380	293660	89770	83809	107850	0
264960	299290	459310	563630	611870	319920	283270	277350	0
71785	51924	118230	69696	111990	52834	0	42942	0
27603	0	0	59564	0	0	0	0	0
7138200	6170100	4706000	3824600	2221800	6162700	8799800	8039800	5
206570	248960	208830	189870	348410	133860	121680	171940	0
0	0	0	47784	69321	0	0	0	0
304780	514950	691480	643980	110120	577070	392250	361370	2
533400	594500	831950	1191300	1879900	664820	461750	395930	1
428390	571250	713410	743600	713370	524360	355340	393790	1
624610	521680	595240	713980	1073300	579550	304190	341410	1
674290	474010	585620	662370	1179600	406280	0	264680	0
10162000	12120000	24511000	26122000	28070000	37399000	41119000	45262000	7
531360	482660	1119700	1549300	2457600	3575800	3710800	3345000	1
5761800	6506900	10190000	9861000	13518000	11614000	11240000	9770500	0
9071300	9577300	19532000	23397000	23590000	32857000	37033000	34063000	12
163850	97684	212060	151980	268700	150140	173250	454470	0
1877400	1628700	2377100	2988200	3378800	6165400	7707800	5854900	4
3250600	2759000	3777100	4098800	5021900	2334800	1202900	1088600	6
17326000	22958000	23027000	21700000	23939000	21293000	19544000	23639000	10
3069900	3323500	5302100	6118000	5571500	17009000	20384000	17669000	4

24836000	22612000	30240000	32376000	33516000	70014000	79846000	69104000	24
0	23607	32850	41220	92566	37551	42094	32307	0
0	0	88644	79178	143160	743740	692740	837730	0
0	0	0	0	86635	156390	116510	106120	0
211020	242230	271180	377050	392620	166680	94698	102440	2
0	0	40507	41863	49957	98454	54529	68618	0
5219300	4656200	3332300	2593600	2197100	847690	420060	422380	5
0	0	0	470450	0	0	0	0	0
215800	221650	387470	369240	297350	146750	101030	128170	0
51205	43148	217730	173260	192240	118530	68231	72146	0
0	0	100160	170040	176550	1615300	783550	1239600	0
352650	406890	776860	660730	729680	468740	315710	269100	2
204270	122820	285660	570590	595830	1621200	2243300	2678600	0
1240800	1344600	828860	919660	565670	1773200	1804000	2484200	2
69627	378230	1635200	1616300	1407200	4359600	8532600	8071500	0
1749800	1118300	885300	624430	518550	3061700	4241800	3504400	5
270740	297150	638440	839140	768610	1900400	3233600	3915000	0
46845	41695	77616	93748	63133	513030	703880	158150	0
5263100	4114500	2282100	2029800	2386700	4103700	6516700	4315100	7
6502200	5477800	3217800	2657200	3529400	4826200	5582100	4946200	6
1109800	987380	633580	516970	626460	646020	618720	629430	2
1505500	1167800	798500	696630	749960	1951400	1704700	1928700	4
5505200	4098100	3328700	4221100	4659700	9591300	12026000	12227000	8
4263600	2531100	1648800	1113300	1279800	3817200	6264000	5299100	4
7001800	6398600	3355400	2809800	4348200	5107000	5052700	5410300	13
1941700	1865100	1868800	2332000	1622400	4139000	3837200	4891900	1
2259100	1438000	1135100	1299000	1976800	2167300	2103200	2033400	5
18063000	18847000	7420800	7886600	5929800	11851000	13663000	14055000	10
208880	152130	146810	145500	151620	188590	150170	169720	0
1565100	1384900	807610	833630	1460500	1569300	1635100	1577800	6
909820	1246100	6625400	10313000	9814100	23979000	25690000	23433000	1
1371100	2672100	3464700	3304400	3809500	6401800	6979400	6722700	2
400530	461890	480100	453570	669260	306770	151690	271330	1
1908900	2327100	2411100	2623600	2695200	1515400	1023200	1039500	6
260310	267540	277640	247030	233800	203260	207930	166000	1
162440	112990	96261	112210	0	1411900	4826300	2516300	0
0	23344	0	19133	0	0	269240	0	0
0	0	0	0	0	141730	118720	139750	0
211820	550620	1054000	1019500	1143100	1490000	1046600	763090	0
0	0	35038	0	143130	0	0	24985	0
164450	122610	174190	280480	175880	563230	706500	458040	0
58427	46830	72115	119930	85942	98753	0	0	0
0	0	24059	0	0	109250	120350	57875	0
607800	385750	471750	413900	556870	548270	512140	440200	1
867320	1557800	1613000	1964200	3442400	1009500	859350	825520	2
23931000	28986000	41538000	36686000	43794000	20006000	15016000	15817000	6
587980	2968700	958850	887790	1146500	422900	290860	475570	1
7671400	8002300	12959000	14241000	12047000	7236500	4455500	3979100	6
92261	163550	262300	325360	122130	91257	105050	81030	0
55265000	82363000	1.29E+08	79193000	15094000	1.03E+08	98089000	56463000	6

2203800	3914900	4857600	2754800	2058800	2100100	1590600	1601500	0
0	0	0	0	213240	47433	0	43708	0
514890	1010900	1105000	1016300	0	1298800	1115200	736790	1
1711600	0	2601500	2666100	0	1608200	1020400	896780	0
1073200	1131400	879300	669350	650680	853280	1027000	914560	1
642100	0	466440	0	860950	330870	0	0	0
58446000	1.56E+08	1.39E+08	1.05E+08	1.14E+08	75988000	56308000	59166000	7
17167000	22235000	18695000	22149000	13199000	25964000	12770000	15067000	5
80597000	1.5E+08	1.51E+08	1.35E+08	16749000	1.21E+08	96632000	73758000	16
514530	419990	895680	1079800	1322900	1725800	2002400	1554000	2
139260	84702	0	0	110750	439680	499100	566430	0
1653700	1686200	435040	226300	521810	297100	1345600	477010	1
9169900	6946300	8812600	7440800	6313800	4775200	4681200	4043400	8
222130	202360	244060	242410	0	285680	166720	103400	1
946340	1107900	1188400	1347800	1674800	616630	524880	398020	1
2688500	1881400	2505100	1810600	2409700	1405100	1155600	1174800	3
242380	162710	256210	292550	334760	115310	144050	133960	1
404840	333730	800030	784270	804070	1855800	1879000	1581600	1
291730	348330	503380	578400	1095400	1272700	1261400	1105700	0
231560	84325	147590	235350	293450	60358	42152	52504	1
987220	939780	1314400	1252600	1244500	718670	441020	414410	3
159740	205670	156020	158300	0	122240	70659	108430	0
325030	211860	365040	698350	433360	1140400	1707200	1941000	1
1532900	1504300	1626000	1286500	2017200	848350	499450	617160	2
118930	0	179140	238060	413330	691880	1079900	607640	0
1229400	888030	1159400	1425200	1037900	869360	497090	833330	3
446260	415860	416140	500860	0	398730	225770	223420	1
927900	1153600	1040600	950780	2161300	1023100	1264800	1660000	2
469040	423900	692000	739250	545280	872140	676770	907800	1
57280	93104	92321	69834	108770	107130	97595	132810	0
201870	192050	133030	94772	0	151300	187160	136700	0
1512300	1960200	3114400	3406500	1544000	3075300	3783900	3861500	4
281180	0	176150	207440	321030	690030	1122700	744780	0
93115	82366	113800	164570	131780	380440	305910	472420	0
227690	0	0	0	265560	102340	171580	0	0
64836	48564	53846	61114	123630	46343	45185	35055	0
112670	135600	153160	134960	214940	0	63216	75764	0
197750	142020	228310	214000	417510	93758	155710	75916	1
55478	68678	67289	65678	159550	0	52072	35368	0
0	81973	119750	101960	97339	65876	48798	0	0
774250	596290	718660	900240	1473500	2657000	2932700	2585000	1
67306	0	0	75900	102920	99743	85833	347260	0
3434300	3204200	1171700	1315200	1253500	1726800	1821200	1880500	4
907650	617070	644120	739230	1136800	323120	212670	340430	1
608920	416280	159020	117630	224830	289800	197540	156100	3
2032500	1331200	1554200	2229500	1826400	1690700	683860	807350	2
382390	381930	522910	602710	397500	329740	163650	0	1
3694900	3566500	3975100	4068100	3119400	2087300	2020200	1982900	10
630130	597150	651890	700240	653560	416420	413320	365700	3
4420700	3420800	4047000	4072700	4188500	2184700	1670700	1832300	12

947820	797890	640100	664960	1036600	293710	239770	281060	3
695240	698650	515240	894630	72709	338270	417610	423400	1
6567800	6990700	8106100	8259900	7545500	4119400	4076200	4217800	10
8419600	8125100	8853500	8702900	9020300	4710500	4663500	5188400	17
171750	182550	159450	131030	218930	72945	78033	44415	1
207240	224060	219870	207680	366060	146840	161540	107700	0
0	0	63469	79474	0	140780	118490	112990	0
0	0	421980	464740	0	335740	157980	138970	0
579940	544540	231610	302230	184630	357410	327770	326580	2
226950	405460	863310	1358800	1895900	1976800	2125000	2079300	0
496960	589650	1976500	2409600	2336300	3658800	3424800	4030400	0
0	78677	77179	0	0	0	0	176960	0
0	0	0	0	0	30556	61203	43214	0
1825000	1367500	1542900	1434400	1805000	972830	571740	674530	5
861540	594240	874540	298170	1162100	291180	197410	436400	2
400440	365600	552520	688210	744560	1773200	2487700	1444500	0
642180	750870	365890	594530	680620	918510	1456200	1023100	1
906830	998150	995910	948910	832970	525710	441790	408440	3
109580	78033	49588	56882	49573	89464	203160	325530	0
4775500	4736500	6731500	7333400	6965600	4109400	3982200	4970000	7
1969500	1993200	2743200	2497000	2189300	1607700	1132700	1052800	6
142410	80111	204720	244210	122540	275280	283420	226510	0
184950	173190	117210	122280	148500	113330	197810	136150	0
34884000	37934000	40774000	38937000	41325000	23856000	16669000	13257000	16
1018800	1125200	1366200	1366300	1345400	808570	551040	542950	3
29696000	37584000	41772000	40242000	26191000	24131000	16296000	18247000	15
82826000	1.01E+08	92417000	80122000	72450000	60072000	46954000	43128000	34
0	0	93749	92725	830550	0	146060	60383	0
117050	443900	315930	326000	436730	221250	304980	157380	0
0	0	0	0	0	49499	42590	33021	0
21926000	23110000	24826000	23514000	21470000	15354000	8986900	7621700	15
2267200	2062800	2899100	3033000	2871900	1574400	962380	712740	3
93141	0	109110	81408	144120	82821	0	81914	0
1.87E+08	2.11E+08	2.16E+08	1.91E+08	1.47E+08	1.26E+08	93810000	96877000	32
53774000	68148000	69492000	68475000	68456000	40535000	28055000	28330000	25
271030	523550	213710	271530	0	479250	906210	582370	1
0	0	127470	120090	0	195390	110110	460310	0
531200	330600	150070	197300	345820	441730	492150	560090	2
63477	68376	65249	44129	65301	33184	0	0	0
402730	435310	530190	696890	922130	176590	191190	146470	0
119000	87159	0	245970	450910	0	0	0	0
3576600	3491200	3779300	3818500	3270500	2540600	1229400	1739400	4
0	0	0	0	0	67763	100490	0	0
47629	46972	35538	0	39770	388900	597600	569430	0
260230	390000	656380	1239400	1202400	816520	777180	713580	0
670580	586000	293200	340330	375010	304050	174360	158820	3
2598900	2405100	3365200	3909900	4784000	2530600	1901900	1588600	7
639620	652830	672680	738580	1018000	411460	247510	245580	2
6749700	9378100	13797000	14810000	18787000	9214800	5331200	5136200	10
4344700	5619500	8006000	8285900	10021000	5480000	3175600	2802900	10

55186	206630	129280	170680	0	0	0	61595	0
123130	73244	85287	87348	81431	0	0	0	2
678310	642000	537670	546930	695460	282120	365950	428870	5
440450	265180	301310	429160	333900	151060	127260	132840	1
563770	462450	452810	405830	807460	235270	235610	204380	3
0	0	20046	22580	0	53006	41168	56729	0
1018300	1055200	648240	686020	776280	379300	476950	648740	2
267570	202820	268820	222520	150200	165130	0	196050	2
4140700	6098800	6779700	6409600	5948500	4290100	4097100	6013100	3
0	0	0	0	0	103260	128200	169270	0
219830	177760	135540	241810	283770	131720	114840	105740	1
99665	160930	105620	96881	66991	84524	72138	56798	0
607970	401360	490360	624380	1017600	226630	242230	186590	3
8846600	10249000	9283400	8319600	4109800	5152100	3605800	4746200	18
85113	48973	62313	77276	71039	288190	319570	276160	0
315020	270430	220260	302330	374080	73030	24944	24709	1
61220	52966	92379	112890	128810	217990	0	396580	0
2698900	3424600	3045700	2450400	1243200	3336800	2704500	4091600	4
327960	215800	203510	196210	283990	308010	288410	192480	2
129380	89531	185440	186140	156580	134650	0	0	0
117150	0	191200	213120	181350	612740	658010	511220	0
2012500	2226200	2368700	2580800	3593200	1287500	944750	904980	2
34142	44001	109520	235680	338920	933850	929670	681360	0
414370	344120	576060	739330	1094400	1879700	1911400	1807600	2
476790	516050	1257000	1812300	1556100	1446900	1531400	1579600	0
135210	138660	162920	139550	149090	82264	0	73020	0
2315100	3026300	6618400	9325000	7604200	17615000	25607000	25668000	4
260160	224130	549140	841440	1085100	1636700	2141300	1793100	0
742890	719060	801020	717440	894220	441380	186280	477150	3
344930	258290	214630	263130	334750	136130	103010	85705	2
3924000	3450000	3712200	3848300	4143800	2379700	1851100	1369100	6
238490	170350	186510	171740	351320	156420	105180	111820	2
92294	151290	0	64685	0	193250	135920	188350	0
661210	565230	637010	883220	1154700	480280	330820	270110	3
70690	0	103010	94020	174660	0	0	0	1
98092	106110	112100	108040	97099	125920	150470	152570	0
0	0	95815	103990	0	684090	878180	723960	0
0	42748	45037	72608	53352	33489	31943	27745	0
500030	442670	559400	805820	1073900	1212200	1451800	1257400	0
516620	425830	308420	327210	296120	265730	170390	175480	1
182910	137580	178780	130210	183690	111880	0	170750	0
75058	81991	86901	96067	0	0	0	0	0
151850	143250	189310	152780	59636	79672	52260	0	1
1008300	676390	735000	962410	1414900	646610	418130	363350	2
87385	79196	86920	86908	119370	58320	0	0	0
2984100	3248200	4369400	4719000	5881500	2526200	1699800	1406900	3
658050	752790	865070	810280	0	587790	461180	401900	1
233540	110970	66341	103110	111660	40723	53424	52266	1
261790	776500	130240	557090	434870	2218500	8137100	4796400	0
116330	92424	150170	133460	215350	472800	543080	707120	0

406830	249010	341920	373350	234160	245940	119930	113160	2
135850	112620	123790	169920	380860	132400	0	48567	0
0	24869	32211	37337	0	28458	0	19624	0
395840	525010	455820	648870	74565	325130	129580	110360	0
286720	194030	354480	325810	481580	229870	252610	312810	1
926850	635070	986630	1102500	1870400	1009900	838530	769060	2
214950	224570	532200	745810	451260	1719600	1798900	2063800	1
32805000	34889000	39663000	44253000	38358000	20864000	14347000	14885000	13
529800	480110	464490	578260	283810	405070	215330	114580	3
0	0	0	0	0	151450	183060	106590	0
0	0	0	0	0	0	0	39535	0
113620	137560	236620	136410	53329	70664	44711	97289	0
421590	435250	374580	518380	264390	220150	329740	145920	2
0	0	0	0	0	86037	88575	0	0
0	0	0	0	0	254770	272020	312640	0
3665300	3981400	1927000	2005500	2485600	2934400	2809000	2798400	7
451290	394780	678300	1264400	624230	316710	494620	314260	2
295240	304610	437160	705230	1388800	225130	246750	198770	0
0	0	0	0	45485	0	0	0	0
247310	184870	228290	225500	225000	135350	69195	98719	1
0	63816	49009	61733	89686	0	0	0	0
495500	446240	400800	316260	346770	198620	134970	146680	1
1081400	744820	619380	688270	339060	468810	535980	330460	1
649110	474150	579940	655790	439460	433790	191170	247020	4
133670	101980	82956	0	58265	0	0	0	0
473890	447690	518360	513130	631950	402830	256180	230510	2
179480	162800	168620	177620	151160	90411	79465	90107	1
76230	76668	83254	147880	265020	383830	1640400	781060	0
427250	361750	320090	423880	623260	331260	482050	483810	2
315020	302650	343370	279030	302340	207570	137130	94573	2
354900	438830	292650	339070	443400	208600	165550	151980	1
0	0	0	0	0	0	0	71888	0
257540	276310	261080	242890	183510	302300	337370	306810	1
0	0	56465	55686	103630	143340	151800	192510	0
910980	411510	289050	274050	312200	639900	412460	719200	1
0	44408	32526	0	44867	42753	43693	69310	0
388390	282650	390830	322020	574960	113000	115740	106680	2
190690	87695	0	147580	165850	59139	33534	33203	1
0	0	0	0	0	0	0	108970	0
154830	72649	131200	124540	153680	150330	0	0	1
107880	69241	101200	64177	0	123460	85512	306780	0
0	186400	145600	236410	114070	130400	0	0	0
915330	771960	1064600	1187200	1126600	536700	324170	280900	4
94346	68409	93256	66036	70648	48213	0	39666	0
4693500	4073000	5145100	4718800	4345300	3126000	2205000	1793200	6
288830	236610	242930	350250	500370	161120	85735	98080	2
453930	264090	86656	41016	88822	65845	235010	174290	1
0	0	0	0	0	0	103570	0	0
0	65590	219280	223320	509790	931070	1166900	1468500	0
0	72568	87932	73264	109030	140980	152480	297070	0

134540	111240	99141	145740	127370	89988	86724	0	2
36509	0	0	25331	0	36639	133550	48464	0
3673300	2210300	3218500	2759900	3937500	1405700	817960	1047800	1
50081	58147	90761	69869	83213	50277	40886	57590	0
475370	1035500	589470	781710	722200	468900	441280	381050	1
143500	147080	254610	345370	211220	172980	124660	101520	0
420420	351550	518510	471050	522640	313490	125600	142800	2
471720	689780	594040	321920	376980	331590	253060	173220	1
66336	112210	0	0	0	0	31267	0	0
235490	167080	358610	273850	132300	270990	522820	181510	0
232930	188210	240170	269680	120990	118200	94353	64211	2
123130	149230	0	0	0	107860	117130	884840	1
367980	632130	569020	593490	480800	522250	398880	598490	1
159590	127170	173970	201640	178030	88594	77126	93256	0
309590	256100	129230	144620	290080	144700	205160	124370	2
105740	181040	136010	161440	0	145520	89417	86657	1
2469300	2394600	2670800	3401900	3772500	1815500	1141700	877160	3
434050	239310	195860	326630	147050	335280	218730	152110	2
4237000	4065600	4121300	3588700	3980600	2339800	1410300	1496900	6
1088800	1014400	1449900	1437300	1056900	1132800	908630	729980	2
0	100410	0	0	0	0	65152	30750	0
44197	51991	74213	56343	130740	0	0	15330	0
809760	466660	423310	377280	559000	378510	223320	260030	2
498050	470360	449830	413230	661670	257270	151430	215700	1
316970	346610	302420	371670	242040	211140	113930	181060	2
0	0	0	0	0	166050	137400	126210	0
361240	326400	279600	414870	432730	730740	864200	771160	1
63551	62264	76749	73584	78308	53799	68282	68460	0
479710	396170	820230	791850	892610	450420	392410	311750	1
949340	979830	1647400	1670500	2002000	4156400	4730100	4398900	0
651180	521820	671050	779160	1426100	579480	434980	411460	0
307320	231680	278270	387450	485660	91982	66783	94521	2
60589	55671	50205	68424	228220	51976	58991	69079	0
503910	627510	853970	740380	972870	481230	357510	373920	2
150690	164970	499820	325800	309720	347920	411370	483150	0
0	0	0	0	0	24481	0	0	0
139500	129250	131350	132130	184880	161860	152320	0	0
307620	298160	444430	781040	640880	1125200	1125700	967890	0
53692	63206	0	52459	60077	69584	160940	111960	0
2065500	1972600	3131200	3346400	2529000	1878400	1623600	2108200	5
151970	200190	217770	154700	152520	136700	147090	113760	1
522720	503900	443870	399380	388940	279120	165660	168760	3
250190	192220	0	399700	614170	0	0	123970	0
90729	94616	137470	103270	127540	81663	67877	190330	0
10842000	10259000	11997000	12932000	14458000	7327100	5471500	5608900	21
110920	145760	0	78486	76505	0	0	72108	0
16039000	14522000	3413600	2609000	5189600	5043600	6332000	5728800	4
79803	182420	147990	70142	81036	50598	0	36809	0
7950000	8988900	2014400	2292100	2563700	3528400	3766200	3226600	6
545090	1881800	908440	1009500	131860	462330	210050	330340	1

0	4859200	0	0	0	0	0	0	0
2741800	2155700	0	0	855580	916770	793720	881770	0
1.16E+08	96894000	29094000	30150000	34430000	44367000	41492000	42933000	91
0	0	105010	127440	182040	241740	622170	375260	0
1096400	1641000	2782400	3735500	2770900	4528100	4591600	5819700	2
0	0	0	0	0	153540	174600	133320	0
129380	112840	156830	198390	202990	0	93080	46318	1
1423500	1461600	951030	1087300	1562000	848560	756350	985980	6
268130	217430	98226	99576	0	95416	110450	168970	3
223630	235860	213580	246700	508270	185240	91268	70239	0
691940	277890	1001500	313150	1192100	2224300	2895600	3217500	1
415910	473620	456040	379660	307870	288080	255890	187830	1
96641	93048	167960	100160	132980	68189	56512	50022	0
68497	146800	61708	72895	66015	64368	0	0	0
144780	131840	0	0	0	0	84645	0	1
188400	200730	160670	132130	121600	69612	83591	95771	1
191410	142070	123310	111930	215690	80739	0	50273	1
0	192980	71487	69003	0	0	0	130210	0
142350	89581	102440	140400	295780	43034	0	0	1
79323	95000	89428	101690	0	62035	49380	46195	1
1558600	1312800	1274300	1212600	1588900	934260	689120	440190	1
0	0	0	0	0	50711	51858	0	0
129360	130190	180820	185800	219030	106950	75026	98026	0
578130	485630	534620	587110	643580	393650	213790	189480	3
55159	0	54455	63186	0	79876	169010	73614	0
80546	69576	138120	349900	208110	953310	1429700	1058900	0
0	0	0	45343	22015	0	0	256590	0
105870	0	0	0	0	78264	0	0	1
853510	666920	965830	918860	1556800	643880	467050	419060	1
0	0	0	0	134240	376010	432580	420320	0
140310	103470	192750	173390	462790	0	91271	0	0
23345000	27583000	51171000	51392000	41397000	23940000	28497000	33048000	38
68733	0	81861	0	0	0	58369	91456	0
441430	358290	299580	191470	143550	261730	232370	266740	2
10066000	11447000	10137000	9051500	4021200	8420800	6955000	5946800	16
283920	312230	232400	473730	290730	202310	164000	102870	1
135140	121820	181980	204860	300890	120140	143570	146610	0
2279000	2529500	2145300	2232500	5916600	1993300	1645900	1685000	4
807600	681690	382600	567650	1350100	242500	201750	229650	2
3974500	2871700	3618300	3182400	5648200	1892800	2043300	1639500	4
183930	118630	97000	189880	178790	75817	107680	0	0
745570	777370	1351100	1583200	432990	897320	675880	569320	1
192790	0	0	236590	0	293880	264730	342120	0
237070	211090	191930	215040	304820	115640	0	102360	3
98578	212150	295330	349940	122040	1586500	2135900	1825900	0
0	0	0	21469	0	64541	52324	58716	0
180910	181850	244660	273510	404180	0	0	0	1
1020900	988500	875520	1000500	1391000	540720	361500	411930	5
0	0	0	0	853030	0	0	813720	0
2075400	1802000	1606200	1298800	1411200	3454900	3419200	2776500	4

595130	1044600	1001100	819680	205490	531750	568340	495920	1
79788	67162	63901	59932	79063	49633	43128	0	1
0	0	312200	362110	397240	740690	759060	805120	0
90319	91508	68250	70168	76943	74138	97805	0	0
0	0	0	79127	108560	199110	296840	154160	0
303110	129740	105540	105520	189660	280290	276780	293280	1
0	0	60924	51650	75274	123240	98836	0	0
0	41017	24653	0	23921	31735	164570	265540	0
160130	262360	178770	104680	147810	172750	243490	246100	0
130560	132400	199570	248600	442530	739890	719370	545870	0
9570600	9251100	14981000	14782000	19036000	7731400	6151100	7389500	9
437540	442860	545670	609470	954920	241230	161630	208770	2
407830	587070	529210	491530	300770	228550	200880	842060	1
258190	250480	395460	410510	745200	150100	140410	171600	0
2147800	1497200	1462500	1372400	2485800	859440	707080	839370	8
308350	447300	451870	441670	453630	296680	138870	151480	2
734140	819130	809600	782340	1223800	518830	276700	366740	2
189600	207090	268390	274510	574240	215850	204830	242450	0
137140	71814	131760	130520	103960	80620	52428	47763	2
140240	147530	93407	86335	133160	96385	0	58096	2
814270	594350	672500	709730	1149200	431550	353730	415020	4
2093700	1543300	1691500	1791100	3582400	1317100	958380	873850	4
603360	456010	399590	627020	663330	296260	195840	251180	2
387510	404110	412340	401960	58291	270100	291470	379750	2
340010	238160	173170	322920	588240	141880	0	0	1
0	0	0	0	0	0	62491	48203	0
966150	513020	659620	1035800	1326100	458760	184050	331980	3
107660	80218	127470	122820	88160	853220	1474600	940000	0
0	0	62704	0	66760	377270	271140	287970	0
61557	60893	87740	66717	83084	46026	0	37400	0
2731000	2147600	5455000	3907700	4502700	8474400	11286000	9396500	2
344390	125840	0	105370	162220	0	110450	99704	2
77208	282750	650110	357200	449140	504000	267330	796710	0
190500	259550	280130	302220	180520	166490	113970	143780	1
0	0	0	0	352350	0	0	0	0
739980	771880	1126400	1047000	1213700	544820	352100	521200	1
0	0	17051	25201	0	0	0	0	0
129050	104310	219430	136500	88590	117880	105860	289630	0
0	34967	79857	63341	0	32258	24740	24276	0
0	99546	136240	123020	0	89485	0	0	0
1874700	1889400	2177500	2701700	4791700	1712200	1306200	1343300	3
1621500	1980200	2823000	3166400	3619400	2059000	1265200	1634300	4
79104	58863	93350	88087	230160	67085	41355	70351	0
4686600	4339400	6017300	6473900	6420900	4569800	3502700	3393200	7
245940	300970	435340	398010	642820	252460	126430	124420	0
2098200	2254200	5588900	5924600	6077400	12913000	14500000	13871000	2
0	0	0	0	0	0	129680	0	0
6453300	13795000	31901000	20282000	15008000	26124000	24928000	25378000	8
90803	86489	98195	92763	138730	77967	0	0	0
143390	152650	198970	519820	66837	119060	113140	0	0

610550	505730	672150	584830	1250300	326080	237910	286790	2
130990	182780	167080	177280	515740	130190	108290	86581	0
821460	427650	192650	274430	824970	153820	151920	317320	2
978580	895520	1079000	1217500	2022300	875510	627940	664100	2
0	61037	0	49371	0	119420	89822	70934	0
0	0	0	0	0	194230	168810	0	0
571130	673760	926420	1111100	1597700	3668800	7094300	5235000	1
640910	572840	816750	953950	494000	1669800	1623300	2863700	3
206560	229980	242680	256770	400620	532180	504290	560480	0
0	0	0	0	73779	118640	115580	126840	0
24369	30926	25902	27016	33608	40744	47621	78159	0
50268	0	64947	87085	0	149960	111020	92232	0
850020	699910	615580	686180	850150	435240	343340	330200	2
386800	372040	374910	179850	350030	250820	178900	284290	1
493640	501220	826000	861220	1026000	888300	748960	641610	3
132210	283130	90441	56916	78835	67374	87741	77055	0
157840	140780	0	0	0	0	0	48313	1
43414	42056	42151	52298	0	0	0	0	1
4056300	4743700	3955300	4080500	4609800	2389900	1548100	1743400	4
0	0	105440	0	129500	136990	0	206990	0
464590	394890	146920	145360	136900	212560	235340	245240	3
0	0	65249	83013	113150	0	0	0	0
307010	259170	100290	159860	159790	161450	197420	186290	1
66926	52184	88535	113510	181700	78906	60918	54755	0
79481	79624	102550	155520	0	58704	52967	52781	0
71964	0	0	91985	75290	162350	395040	471410	0
334670	311750	818130	1179100	1148700	2567800	2745200	2287700	1
36141	36558	45954	44257	58874	50736	86021	81071	0
361490	108210	122350	107360	209960	80330	82606	77190	1
11247	13423	21498	20544	0	475230	279110	292430	0
17523000	14691000	19771000	21228000	16905000	54664000	83591000	67468000	8
8617400	9114500	7580800	9010800	7333800	6392000	6056700	5511000	8
192630	153950	172030	139890	54362	126560	57279	38735	1
0	0	480350	555230	476010	0	0	0	0
0	335070	442390	478740	527620	971470	1123700	1678400	0
753040	608830	756780	713970	847840	504140	325200	239540	1
0	0	111990	129750	0	288560	561750	215750	0
412100	342670	786240	1070200	1077200	1679700	1611200	1857400	0
127430	92460	151640	179380	342180	100400	62552	83522	1
51801	0	0	0	97678	0	113360	117570	0
0	0	0	0	0	0	0	42008	0
524420	421360	471810	639770	645960	246880	148810	117120	1
0	0	0	34237	45159	41612	0	46333	1
205050	202080	1000900	538100	359940	990460	894590	1893600	0
315180	291090	243980	170680	236430	145580	99617	60625	2
7614200	9002800	9148200	11174000	14376000	19780000	18626000	18610000	8
331120	166460	359390	75989	535040	1706100	3133800	1657600	1
2135500	2071000	1632900	2505300	2644600	1122500	959700	997180	3
3036900	2518200	3422200	2663100	2046200	3876400	4053600	2510400	4
824690	708920	1410600	1671100	2413400	4737500	4564200	4162200	3

145170	116740	117170	91976	175800	108140	81780	0	0
0	50798	55699	0	0	0	0	0	0
70882	93160	91839	76860	76881	80658	0	42624	0
4299400	4346300	3538700	3457300	2140700	2677000	1803400	1863000	13
33646	51354	36727	40183	0	31694	0	30441	0
101490	248260	256480	268250	936540	251960	199840	127610	0
568790	934640	1008500	903170	1049600	84543	0	0	0
123350	107990	124360	194960	175050	247940	220390	334330	0
76942	98178	73946	58763	79682	226510	113860	259280	0
186600	169870	186600	202520	331260	513660	616990	739800	0
0	56371	127670	74613	172000	40420	42129	32320	0
5036100	7108500	6901400	6660100	2136000	4747300	2341400	2444200	7
82144	44446	64670	94883	73407	63484	0	37443	1
0	0	309610	108490	296870	1074200	2369400	1738700	0
123170	98748	233090	0	314670	0	0	121730	0
128390	103030	150270	258670	527300	185950	225120	257930	0
113290	147390	201650	223410	287000	217880	521830	316570	0
162000	217100	0	181070	0	130600	67916	45392	0
293090	305460	305680	516540	353170	234170	136280	162750	0
0	0	134330	176820	280710	513790	524250	438680	0
0	0	95878	99561	148300	144820	140670	123100	0
1443600	1226900	2296000	3225700	3016200	7398700	8652300	8677500	1
1318300	1361700	2452500	1499000	3271900	6719600	8842500	7643600	3
0	0	92593	89101	177890	0	0	0	0
131160	127070	126050	177570	155100	136460	117710	111780	0
0	39442	35349	0	0	53815	92352	57596	0
56219	58726	142950	400610	67673	647780	867120	2322700	0
0	0	0	36671	60837	66358	49078	78078	0
351420	435700	429200	511020	831260	279140	202500	280320	1
0	0	0	0	0	0	0	258010	0
135920	89962	70306	76614	0	0	86950	156860	0
84410	103840	60126	55217	68905	54334	60749	82779	0
1066700	786310	769170	721560	1064200	546370	513780	632210	1
173860	138360	294740	269910	289680	131730	77784	83479	0
272200	621430	1201200	763890	1338300	2945900	5732600	5365800	0
133960	133170	362090	495610	348210	1417000	1362300	2551100	0
735130	557270	1412700	2002900	2839200	4818400	5029100	5317100	1
89653	162610	275840	635970	743940	1261000	2267300	1531800	0
171700	119530	174780	382760	427190	693880	600370	492580	0
0	0	0	0	0	0	151670	48861	0
155990	147300	59891	48343	42067	0	103340	138580	1
15797000	15607000	18462000	17401000	19650000	9439400	7649300	8834300	38
103520	0	102830	83085	122970	187760	274090	204230	0
59611	45490	54720	51008	63298	45446	0	0	0
302710	276170	420390	461440	582330	172560	178580	187230	0
4785700	4766300	3334100	3345600	4843000	3864000	3569500	3093200	7
40410	38197	38863	43714	247940	0	0	0	0
2831100	2892900	1918800	2605300	3678200	4838500	5255300	5287800	4
1111100	1100300	626960	847080	818660	1956300	2379000	1979200	2
154390	135310	393150	440860	412540	840670	1105900	827930	0

2635000	2847300	2608200	2758900	2789300	4790000	6390200	5729100	5
74001	52391	123140	136400	207420	187800	222900	291470	0
118780	122990	151170	182650	186750	212460	199370	213380	0
0	0	0	0	0	93684	114140	78601	0
118630	111000	133670	180050	215390	66275	49874	34877	1
57368	55026	47986	47630	42829	46320	65876	80054	0
3434300	3476700	4803400	5068200	3308300	3642800	2114800	3922400	4
1195800	931280	719140	764950	1243700	506430	369030	1010800	4
272340	233900	199850	157390	473030	109950	188140	118300	1
587950	1146400	1322300	1653500	1084600	5649700	7296800	6169300	1
878370	916290	380840	243440	253350	425610	713240	707880	1
266930	174240	133830	169730	212680	120280	96394	96031	3
125320	107040	132100	129280	226850	71397	0	0	0
0	0	0	29865	53679	103120	82391	114680	0
0	0	0	0	0	0	0	0	0
0	0	0	0	0	111000	100080	0	0
17219000	21376000	28318000	27402000	42025000	22490000	20228000	19690000	17
1847800	2024300	1649500	1585100	2195200	3020700	2855400	3054500	3
154390	114830	129660	107180	77626	108950	85139	92918	1
583690	590230	323350	256140	317670	307390	363470	367510	3
1726200	1216500	669550	702140	974630	1117600	1367100	1101100	6
0	31902	40129	0	100020	34139	0	31824	0
427730	352820	220810	272880	799550	570010	412110	539750	1
36571	0	0	0	0	44130	40120	57755	0
390840	280100	209480	318620	280460	0	112690	349450	1
1372600	627620	1399100	1550800	2703600	813620	364410	351300	2
444640	364970	208340	0	0	260540	272130	250660	2
907980	802430	965010	1069900	1615300	529130	322990	293860	4
26841	0	37308	51773	0	80092	101320	72361	0
811760	1262400	2499200	2770200	4595500	1493000	1209800	1255800	2
21750000	22143000	26982000	25623000	37431000	13813000	8010400	6132500	1
3866600	3153800	1863600	2274900	2403800	3894900	5150800	4964600	9
360640	644400	953230	959810	1242200	396420	336010	650350	1
103930	71588	95321	85556	250790	0	0	0	0
426180	499540	154610	158160	100580	230300	275460	443380	3
281770	182690	202510	192860	208900	141420	154870	192030	2
2037600	1625500	882470	766420	383550	1679700	3039700	2404100	5
0	80769	175500	97823	258310	65141	81533	61466	0
99453	125190	110130	230140	118440	386030	346410	316500	0
115490	113430	134800	134540	349030	92426	0	0	0
33814	39904	132570	0	220200	52421	0	104660	0
0	0	0	0	570560	0	0	0	0
113660	97553	124830	170200	206570	106900	93169	79842	0
71956	81946	168770	137410	213520	64030	0	46728	0
165360	159440	829250	975020	1267900	210140	180660	1049700	0
1355100	1206600	1232600	1360600	1940400	764150	451020	514960	1
576730	250030	490850	440210	320260	260140	186630	526980	4
187170	155380	151090	197680	284920	104880	58506	49076	2
1255100	1118700	1385500	1242900	2217200	725540	458990	410910	5
3870800	4139000	9051400	7244300	8777900	22150000	33294000	31767000	2

407690	317170	732520	850430	884500	644900	585650	714680	2
0	0	0	0	0	0	0	175200	0
0	133480	40124	0	0	98844	401170	99439	0
9896500	9356200	10348000	10120000	12989000	5814600	3612000	3789400	9
6430900	9418500	7680200	7175800	9885400	4346200	3567800	3581800	7
692950	226090	1208300	1255500	1738800	3477000	3267600	3769900	1
591650	492290	652060	1557500	1064700	3421800	4657300	5256900	0
1048100	994470	903140	760940	851340	505850	520130	520230	2
230650	139550	125050	137130	194930	104090	0	89480	2
70478	65976	87485	83278	90302	164360	116600	335320	0
408470	224850	337880	329580	195010	844990	791430	811310	1
1885100	2628000	2258000	1961200	3977700	1411700	970470	1133600	2
602300	702720	527160	329750	324660	505320	402170	476800	1
785830	863480	0	1101700	747460	1819100	1997500	2480100	3
136790	104200	142320	118570	128230	78576	56660	73113	1
0	0	133540	185550	262400	460310	672150	426290	0
176430	188030	73693	116520	102090	178980	248980	189780	0
0	0	157550	175870	391990	683170	537840	668790	0
93322	0	134710	92502	60686	364380	224540	185210	0
0	0	0	0	0	373040	432570	541300	0
0	23574	25125	36808	0	123580	53130	94017	0
0	46477	31674	0	0	511760	735690	476000	0
173200	130900	301850	214260	155140	1199800	1472200	1275300	0
116190	207900	426930	739840	793750	1682300	2061100	2406600	0
271560	246160	476710	685920	511380	1397200	2213600	1555000	2
0	0	197660	254510	326240	519990	476950	477910	0
711180	601400	628810	560250	632270	2151400	2224800	2123100	0
306400	519130	477670	648460	240640	2217000	2375200	3283200	0
0	0	69852	55921	0	143660	133350	215990	0
0	0	25301	36567	0	113970	108480	119630	0
299450	218700	593110	704430	368670	1774800	2094700	1821800	0
0	0	0	0	0	274930	309810	280100	0
102610	107410	94393	120970	147730	428200	273140	397210	0
0	0	90512	119720	136040	304020	316860	0	0
127770	101810	126630	352930	125930	890170	659700	717690	0
206710	216360	344140	491890	651120	792270	621650	878090	1
28032	37366	55795	71060	67466	123560	319400	403680	0
0	0	0	0	0	188800	250010	166940	0
105170	0	205660	252400	186280	132760	78095	103800	0
431290	358610	326760	540620	451470	1375400	1626100	1278000	1
150060	131620	126460	146500	88531	246380	155590	126340	0
0	0	0	0	44252	41377	49680	56355	0
25490	0	25102	25281	44066	103970	178170	202310	0
623630	655730	815510	919750	1359600	518980	264040	408700	1
123520	94242	124110	414590	161200	103150	60649	90377	0
0	0	201120	344350	399850	785610	1162600	866190	0
754890	759510	342720	116530	0	225510	367140	217480	1
0	0	97038	87269	87993	118230	106540	170170	0
464380	415330	757070	1357900	1644100	2559800	2057900	1464100	0
453210	411950	1135100	1292900	908650	1501200	1131000	1049600	0

0	251450	151400	334280	486750	452540	432190	1020600	0
12338000	13274000	12917000	12284000	14409000	8497200	5728900	7022100	14
23857000	23821000	24468000	22588000	14928000	18782000	13050000	11813000	16
3494900	3095500	3688900	4002800	4776900	2373400	1428300	1341000	7
8890200	11019000	11899000	10343000	7111200	7646800	4552300	6164100	6
115980	0	91854	67270	183900	0	0	0	0
114130	150730	425250	195860	806080	111060	128930	70533	0
2639900	2658200	2417100	2555100	3338300	1639900	1218900	1356900	3
79059	0	99168	78992	180530	0	0	0	0
591400	495240	388990	306510	217850	113350	66944	78691	1
944000	735080	822280	690500	840460	741290	299230	416560	1
0	4379200	0	0	260320	0	435240	526290	1
1188600	937800	849710	764010	927980	633990	366590	414530	2
0	89369	162000	0	132830	348330	334070	423000	0
4330200	4214500	4238900	4089200	3781800	2005600	1616000	2194700	6
117080	70038	0	0	0	69251	96770	86178	0
81830	73371	63014	68652	98399	135070	270430	257090	0
111820	68066	54974	0	69199	0	0	0	1
0	0	333290	0	0	0	0	0	0
63371	0	0	0	94963	70177	88444	0	1
219960	111880	174020	172080	335690	72263	69298	99221	2
48503	0	0	0	54585	136560	110510	94381	0
146380	256970	188560	310000	213260	165510	188020	181040	0
359900	381660	326230	291610	506370	0	0	0	0
630210	457660	648080	620070	835780	359640	195410	199470	1
0	0	0	132730	160850	0	101770	95156	0
122980	85416	79081	150420	206430	55592	54814	62769	0
978510	1015800	1296300	1547500	708170	1058100	638020	473340	3
392510	222520	326250	622410	608020	939800	608710	697740	0
47096	0	0	0	147540	0	0	36911	0
273100	214630	363440	412890	748040	141740	0	139690	1
984260	434430	782650	672330	1116200	1528400	1320500	1244600	1
0	201410	210580	246830	625580	226900	393550	186560	0
0	265120	176300	182530	0	0	129100	0	0
303680	199700	254010	144880	193580	166520	80649	45934	1
55920	0	92334	104220	141370	296150	246580	437570	0
0	0	0	0	0	0	100480	568430	0
73281	0	0	175200	107470	283310	332330	1252300	0
1479800	1431000	1435400	1655500	1921100	1257300	866080	770450	3
53859	0	43214	0	0	74075	61783	63824	0
181680	156330	144810	169820	147180	874410	1150800	984460	0
455120	294670	322910	400010	645170	239090	159970	139130	1
547960	218190	440460	660020	780860	165390	127470	140090	1
735030	1214300	1427300	1907800	1727500	4143800	3268600	3994900	1
3425100	3161100	3453300	2452300	505330	2469600	3435100	2598200	1
3878800	4996300	8574600	6375700	1194000	7617800	9000700	8176900	3
0	0	0	51141	0	171010	146060	196900	0
810600	727090	828680	1015800	697670	595020	680160	887820	2
240450	451470	452930	541370	0	483120	449330	454740	1
1369700	2808800	1652400	1284200	344860	2850900	4800100	5844300	2

167040	119250	204850	163950	151620	89026	69591	85220	1
971750	635720	754020	874200	1111600	590370	525400	811680	4
50978	0	192540	1288400	650390	7293800	9407400	4186800	0
190920	172410	391650	460800	520600	110760	88411	115450	0
65654	44780	56595	88869	113520	42977	22876	27381	1
391400	374520	326850	342670	61898	235610	174510	149030	0
224030	186980	649920	854650	1116900	806320	748550	579780	0
67352	69790	62724	88138	89271	147380	93752	192940	0
0	26218	32832	0	36541	50651	46347	46674	0
1698000	1506900	1567700	1369700	1504700	1006700	433460	634760	2
2145600	2324400	2145300	2164900	2886000	1943800	772580	798650	3
646370	496710	647350	778340	625240	421740	0	0	1
117670	96778	0	0	0	0	76139	132260	0
255150	112880	236420	153910	372810	91281	62153	50951	1
0	104480	0	0	143980	106210	99476	82060	0
256070	215490	301820	406200	462110	516080	462170	553410	1
164720	214380	170900	101710	94814	85851	167380	143210	1
0	0	279610	498320	560730	6224600	5401200	7637500	0
749810	529020	715350	749880	1218700	250220	193400	201460	3
99033	101550	157910	115510	183030	62558	90185	42504	1
0	0	109140	144540	249790	454700	628430	942540	0
65409	66959	188560	130930	369380	462810	462600	972750	0
136630	54575	176930	336290	542060	505910	748290	1016300	0
222790	153480	43194	0	0	61896	85682	138420	1
0	0	0	0	0	100670	175600	105850	0
206660	130240	162500	94843	60761	77782	0	83572	1
90691000	97679000	50397000	44348000	21459000	84169000	97759000	1.05E+08	15
0	0	0	0	0	0	356620	767590	0
0	0	0	0	0	0	217480	128480	0
133250	146750	148420	149090	118680	131310	137740	116440	0
160130	189170	126740	159050	152730	89306	126530	107520	0
0	0	81566	119930	135700	50332	0	0	0
36305	0	0	0	71694	0	0	0	0
96456	94796	105300	119770	152720	237880	179690	233740	0
42623	46304	48490	43381	50766	0	0	0	0
0	0	0	0	0	12757	0	0	0
299370	202720	157030	297580	48406	111400	70578	199680	1
872340	719580	903130	900610	1320000	565730	276550	274040	5
400010	474350	406720	434010	484440	330430	240750	307800	2
775170	639380	786540	861540	1231200	597810	377240	380130	2
0	0	108530	165730	330380	0	35782	29781	0
24846000	20980000	36452000	47221000	57238000	95319000	94896000	97254000	18
0	0	0	0	0	57125	89787	56054	0
348080	361620	706490	1446800	1531800	2932400	3384500	2793700	0
628920	385560	556490	579730	555840	135870	122840	155540	2
890420	571490	1080100	1348400	2647600	554320	386550	552600	3
113310	114790	135590	133550	274560	103590	125860	118310	0
1707800	1798100	2464100	2725400	2511300	2696000	2026200	3685300	0
616020	526000	672820	971710	1638900	2023800	1773600	1823500	0
0	471420	688930	1163100	728610	1617100	2277400	2167500	0

2901600	2517400	3551500	5716200	5362600	5931100	5588000	5877900	4
0	0	52608	61642	0	51221	44748	198830	0
2019800	1977400	2212800	2866900	1724000	2322700	2065600	2050000	1
718910	719640	0	421480	295920	384100	347090	375540	1
86558	62583	0	0	117460	50718	0	0	0
1835300	2966400	1589500	1694400	1862000	1173000	850490	924930	2
3240000	3177600	5009400	4854200	5694400	3345300	2871500	2652200	3
5593500	7275400	3584300	3509100	3234100	2009300	3810500	3795400	9
1651000	1143900	1295200	1021900	2141400	868470	527790	817560	5
6997000	9859500	9793400	7756100	8243100	7161600	5410700	4899900	6
661100	1243100	823720	648070	1144000	742640	1064400	234770	1
822240	814760	797250	807940	1110600	646110	489260	362610	1
44663	83158	59960	87666	71616	41067	30948	28949	0
95845	89262	128140	107810	92317	64852	55341	40199	0
2929000	2213000	2082700	2151200	2316000	1895700	1864600	1333900	6
867000	754380	622540	615960	980140	478840	461410	530260	2
390430	642190	563150	541040	0	288690	404620	393490	1
57806	0	44321	48879	67619	38601	0	35137	0
4404400	4370500	4593600	5277200	1599700	2930900	2212500	2355600	5
4841500	4544500	5203600	4929000	3187200	3694000	2448900	2380300	4
11363000	10509000	13715000	13619000	11782000	9858900	7031800	7766600	7
0	60253	61843	66126	0	57380	47446	46549	0
0	0	95556	0	62070	0	44707	60218	0
224060	226470	321350	292470	397980	172050	176520	0	1
887100	819640	1361900	1381500	1033600	700570	626600	543640	3
1182900	1016900	1106600	1193600	1388800	885730	871310	753580	4
495780	463680	513370	489380	435450	261440	215930	112010	1
119130	126090	139170	78023	0	50312	53920	196410	0
843420	732640	785470	683760	670760	396100	275210	394580	2
562950	285840	352690	373630	248570	174230	151830	179030	1
791570	862140	899640	832070	496140	676260	499720	830290	1
141220	125210	201410	218200	317140	84451	66288	57035	0
516350	293810	512450	459930	755580	205920	173350	150630	2
874620	915490	830670	731090	739240	922640	560440	325080	2
773250	593060	1475400	1515700	2382900	1558300	1134500	1301600	0
535560	351360	221660	194330	203290	318760	254470	242460	2
49713	36828	42995	94378	128730	28010	0	24128	0
58558	39416	38654	39142	41741	59937	62298	90022	0
128530	129270	152950	140410	168880	171120	193360	174270	0
401680	340520	167470	200660	248370	165250	204900	193720	3
444870	512100	102530	245360	149460	206620	294660	342890	2
284270	274430	0	140480	136690	138320	118180	95119	1
84438	100030	68456	62134	0	93378	90561	85065	0
0	55686	70948	57926	128190	179480	424350	203020	0
0	0	0	113400	137180	128590	148170	88309	0
70810	0	79817	98404	0	165500	152080	125070	0
1249000	1678800	1579400	1571200	2623500	857650	688820	817880	3
379910	327800	473360	338990	709210	169500	110010	162990	1
4914800	5060400	6242500	5526700	6690200	4170100	2939300	1913100	3
40399	56218	0	0	0	51484	52594	48814	1

382180	358430	986340	429190	0	377400	333820	274060	0
231680	202080	156260	252230	235050	139430	127460	94831	1
1290000	1286300	1143500	1206900	1221500	691460	491300	594600	2
82285	95032	105240	54366	27730	34988	28921	29147	1
206280	152190	511750	658730	1119700	1545600	1262700	1091800	0
71900	72700	58168	79440	140770	268780	141130	351380	0
229760	257590	289030	345340	240960	681420	540780	474000	0
0	0	0	0	0	0	0	925210	0
1726900	1605400	1042200	1184400	1198800	1258500	1487100	1419200	6
529320	396490	435950	401590	577240	326750	265040	213520	1
617450	472640	455230	622920	918270	1385300	1379800	1263400	1
0	0	157990	0	0	105320	140890	135830	0
44735	79440	75254	85214	140990	106410	486040	485540	0
27934	84617	75223	80497	95689	186570	189450	203020	0
230970	209770	135870	145000	122450	233670	343880	366270	1
0	0	0	0	0	46879	43299	138090	0
2709300	2911400	4870500	5819400	5443600	2980800	1984300	2672200	2
1984100	2588600	1944700	1789800	3089500	2113900	2273500	2208900	1
594140	510800	645960	439010	1310900	264120	274210	176390	1
81898	99755	174980	234120	451470	287130	165570	257170	0
0	61317	199950	104750	346400	58879	0	181740	0
175800	179460	245820	354790	543530	146840	131510	147600	1
1004000	746810	1009700	1158700	1837100	570400	432340	374250	3
298070	284700	324120	132420	451360	127490	249900	894080	0
301660	336240	357630	387260	465400	167920	203300	135640	1
532890	443550	668940	471590	671880	434710	390190	595050	0
153310	193020	472560	448660	608880	281300	152400	194380	1
0	0	63905	56381	55205	87683	153910	101560	0
212810	323570	268540	106910	352870	139670	258110	0	0
317360	274020	1144900	1255100	1792700	1173600	1479700	1366900	0
863910	973450	1073700	1111200	862280	722520	377380	524810	2
176470	89157	0	0	0	0	0	0	0
235050	694270	2981400	3665700	3974100	3336800	3610300	3112600	0
2241500	2176300	2003000	1923800	1790600	1517300	1331700	1191900	8
468840	350700	330520	327940	294160	242660	297460	338870	1
280110	315270	344070	343620	502920	425520	350360	396240	1
0	0	0	0	0	0	0	0	0
304320	410420	575330	610620	899380	300820	168440	168560	2
0	44982	215630	233270	252420	65207	44780	45750	0
997960	1068200	966600	979520	1170000	730620	713220	715780	3
520370	278610	604000	497150	981360	947820	827830	688940	1
53488	35350	248320	194900	319400	1290900	1980100	1649400	0
0	0	340890	433220	345910	1074000	1349600	1112200	0
40039	67174	37503	31643	28481	31457	29469	32059	0
99360	63878	97657	119170	138130	95145	88685	89743	0
348450	484150	426230	450190	301470	561440	674740	645730	1
342930	253740	228190	210200	361800	226220	335780	193150	0
129960	149090	77968	114140	153390	148460	255680	163740	0
1928100	2176500	2691000	2545500	5502000	1810900	1266300	853380	3
27132	37122	31662	82741	0	30053	18503	28106	0

657860	0	0	0	2144800	0	0	0	1
57003	43965	84257	60643	142730	41899	0	41945	0
0	136790	296520	250950	489620	152320	109010	124290	0
100640	179100	194930	256310	198450	194330	0	138820	0
1712900	2273300	1637000	1767200	3019100	2175200	2094400	2895100	5
1303400	1116100	925700	1084500	658220	1430500	1305500	1218700	3
426940	340680	313950	378230	453160	887840	822280	777510	1
0	0	0	0	0	165430	258240	220350	0
8790300	9113500	8219200	9008900	12437000	5450200	3585000	3249400	12
719110	705720	1407500	1374800	2156000	1421200	1298500	925500	1
71640	0	73997	73588	177360	72488	85991	129620	0
316920	292790	286870	424120	447800	228550	170030	96422	0
76227	45354	42903	0	0	48721	0	63056	0
0	0	471730	464450	442050	254780	213820	248960	0
0	183890	140770	157620	185090	266620	220830	254110	0
159470	187000	200780	258750	124390	141870	84196	0	0
372610	266230	422500	312130	531510	185750	121060	92470	1
462660	422400	392090	582850	759260	257080	209690	234630	1
0	0	0	0	0	0	0	83049	0
0	0	0	0	0	30938	35008	77048	0
0	0	35569	33232	28803	73618	0	0	0
47497000	35987000	41315000	46915000	62178000	22245000	16521000	15506000	11
216580	151590	169690	105530	78909	113110	77688	89352	1
5575200	6287400	4560900	4671300	3241700	3102800	2934000	2204900	9
366850	0	372160	393530	510780	278230	120900	178050	2
2228100	2233100	1862500	1764000	3019600	1434400	847830	1022300	7
217390	145610	199910	233360	36382	86518	52209	104040	1
0	0	0	0	0	0	0	0	0
1045300	990720	1069100	1265900	1508600	569200	598690	610250	4
2029300	1850700	1828500	1388600	1805400	1809300	1707700	1741100	3
126870	138880	247470	67382	94444	0	107970	0	1
83181	81287	92767	64849	101310	107460	82930	131070	0
1440600	1311100	962340	852600	924090	1509100	1312900	1395400	3
1033200	1577200	914620	631030	515660	1210000	1288600	1073600	0
258550	259770	279150	214940	127300	200800	253960	224240	3
1062500	1425200	1218200	1114300	838490	973530	837290	761110	2
1795600	2290700	1628700	1233000	931400	1408500	2239300	1279900	2
761410	841090	1050200	1250100	1292300	732700	622610	563270	2
89299	71070	73675	81509	97540	157490	198240	215440	0
109710	125550	71696	100610	127200	74748	75120	95255	0
38596	0	0	0	0	62095	48413	179800	0
0	97568	187280	174140	372730	621530	437030	351270	0
122170	0	125400	94032	121970	139210	128670	138680	0
195380	152950	163740	201780	162740	169920	191360	165590	0
1115300	764410	904430	928580	1009700	1504900	1564000	1296600	2
0	0	0	0	0	74342	236740	376310	0
965480	619900	717200	740500	796100	1256100	1984600	1353800	3
992800	659770	640950	351070	134490	252450	239060	327670	3
0	0	175120	139860	0	283590	218740	414580	0
891110	901740	935480	1236800	1190400	1720300	2869300	1952900	4

0	0	0	0	0	341390	366560	201150	0
0	0	0	0	0	0	494160	0	0
68879	73290	192860	100650	142290	80776	165020	110660	0
234910	197540	138310	304440	371250	513730	835660	747580	0
105340	118500	86590	115930	100540	237000	261470	327070	0
143030	193990	191800	166350	349260	92176	91602	63599	1
59589	131040	54359	51605	0	494650	706740	228430	0
539580	535450	1133700	1908300	1597800	4365200	5526000	5216100	1
0	19865	130620	168880	41044	662840	1057200	1335700	0
7992000	8559700	6837800	7153600	7021400	5878700	5429600	4136600	5
224730	0	162070	0	0	318660	268890	281440	0
429830	306170	303540	205770	206240	139730	0	94569	1
0	0	51917	0	0	0	261530	69149	0
0	262060	363480	434900	365040	1080700	414330	1358600	0
0	0	50344	61768	60988	305780	231770	231890	0
753210	770110	686480	1123800	782080	442520	290880	236120	1
1533700	1116200	1420700	1688600	1162200	902000	436400	712950	4
152670	126140	39634	28647	0	33695	72942	50299	1
1159100	927730	646590	367080	569570	454420	259780	260290	1
1055300	544700	664100	1018600	787910	527560	189120	284850	3
808820	805350	832410	879810	1719200	547130	297120	349410	2
71107	200880	131040	167900	196760	402280	754950	506890	0
0	0	0	149580	97373	218320	221180	210270	0
279030	224470	581550	430050	982670	732480	994090	947840	0
1092800	922000	999240	1118700	390140	686890	484750	568360	3
191380	186630	108650	140780	139680	219180	295330	261810	0
272610	159420	212700	264350	396320	167620	211370	184290	0
232930	272410	259940	192660	292420	431450	476050	434280	0
0	47960	63467	0	0	53575	54129	147320	0
0	46832	117410	135530	105260	62878	56798	0	0
1441500	1668800	1816300	1556500	2403700	935800	604470	590180	2
91057	51641	77490	97471	133580	218430	144590	140190	0
0	41747	67757	68096	87070	165160	169130	188910	0
303740	185960	318910	346480	461770	594080	713690	711580	1
72576	94952	0	0	57406	61574	261210	263210	0
0	0	337870	209830	306970	1182400	1124000	1224900	0
950630	931090	730040	742580	895080	1205600	1777900	1412000	3
183850	192630	161870	209960	0	378720	622060	588600	1
132650	136700	103260	121450	118650	108650	0	226960	0
133450	161160	321200	312120	307620	242040	300800	308850	0
0	0	1066200	1059100	1984800	859740	877220	994390	0
0	0	0	0	0	0	173750	0	0
10066000	10258000	12053000	12973000	12441000	7384100	6240700	6920300	14
900450	775520	1104200	1235700	860540	432320	352320	330240	2
984600	926930	1229200	1042600	687540	519270	559760	436000	1
137760	121420	145450	133040	0	126350	115050	118030	0
2924700	2421000	3342200	3325300	3683100	2391300	1499900	1649600	3
592350	652360	792790	682060	403360	431530	311290	209090	2
180920	121220	162630	144900	193190	146800	0	88320	0
0	104050	124460	132590	283850	75612	62455	73216	0

2608300	1951300	2120300	2047100	3501400	1133300	1054800	881470	2
89004	76375	131330	107630	388280	89476	68133	79849	0
175440	106810	227130	353350	671700	676630	941620	632800	0
367840	403820	446750	452450	418170	631520	872850	845390	1
250300	0	0	126880	0	0	70073	0	1
0	0	111400	125540	649420	305610	0	190710	0
125410	129410	190340	168620	0	212230	188210	176030	1
731500	761510	870420	914610	952620	801350	995510	866330	0
0	71619	61032	89676	82857	99952	248710	159760	0
446210	490460	2012700	2301000	2391700	2275700	2965900	3459600	0
193550	246020	733190	877690	1149400	1268400	1660400	1547900	0
361830	300920	404460	456000	451270	629480	660360	484760	1
0	133350	106080	92255	0	0	0	0	0
305230	154540	165700	144510	91024	102660	77537	80377	1
1164200	534090	226760	151860	0	1897400	1982700	4953000	2
75460	99095	153830	177150	215910	342090	280680	630340	0
0	0	107320	109540	0	150020	191280	218770	0
0	0	164400	179260	383030	363480	304690	441650	0
430610	492140	640160	632330	1264500	1336100	1031900	1158400	0
223730	134400	251170	248120	334640	765430	850480	950370	0
592070	440560	493950	609600	752490	837800	810250	736320	2
199100	206930	101780	101670	0	235350	414580	287550	1
735060	987110	284170	298490	342080	759010	796130	935770	1
1908800	2123800	702030	1036200	2192300	1261200	1006600	1997100	2
679310	648950	1839900	2071500	1603800	5812900	5598800	7350000	1
190720	304980	273150	220710	358370	233360	77127	80103	1
1231900	1653400	2138300	3365500	2510500	1677000	2305200	1192700	2
0	0	0	0	0	0	0	62387	0
520110	785770	609210	717470	0	404450	441810	299420	2
777660	585240	1300400	1512400	1497500	2359600	2487200	2304000	2
1645200	1466200	1537000	2099100	1639900	2372000	2167900	2234600	4
0	0	0	202700	163980	293370	762640	664340	0
0	0	0	0	0	463940	538560	535450	0
0	92258	75752	0	0	104470	99036	90282	1
5457400	6444600	6072800	5858600	7068000	5544300	1545700	0	0
715810	1332200	800630	876140	185480	797700	619600	715290	3
1852700	1318200	1855000	2160600	2040100	1183300	538020	611440	4
632230	746950	1165400	1456900	69295	491280	268460	1312800	1
29677000	28095000	33401000	32209000	38497000	16380000	11364000	13250000	20
948810	912460	1694700	1977900	2529800	4895400	4457700	5012600	0
288890	287920	51201	31987	65672	35600	67270	56636	1
55358	49775	47791	73508	66720	61048	31506	0	0
393140	293730	187460	206220	206600	340690	327610	342470	1
63264	0	132610	130370	158090	181370	180660	178010	0
80478	0	93847	126110	178370	148770	143270	134110	0
1295300	968410	681780	842190	734860	820540	672000	380760	4
1493000	1378800	1510100	1348000	1535200	849450	592640	511350	5
740390	763700	970980	813410	1477700	528390	338920	371250	1
801680	840140	934090	914360	1037400	480330	292240	300680	3
492430	450300	543470	446900	570230	345320	189870	157820	2

960570	822700	1157800	1000000	1347700	614860	334180	370340	3
400050	338830	423590	348830	571960	323840	206770	152660	2
2609500	3056500	2383900	2620800	2444600	1296400	1085400	827760	9
0	0	0	0	0	0	169690	174040	0
129890	112570	241970	212050	83504	599300	516880	591060	0
79926	91556	77120	92848	160810	51165	0	0	0
96139	101020	139160	142400	399520	192440	318410	189070	0
73436	74220	227520	343150	94873	1005500	908470	1094300	0
2470700	3159500	2226200	2184800	2644200	1858700	1649900	1119500	2
75995	79507	59778	112700	213700	306820	298020	379620	0
0	0	0	0	0	0	0	0	0
180800	320820	332410	746210	1445300	207830	56410	79176	0
118070	49021	114770	196550	298010	39634	0	0	1
728970	573040	602160	718780	713920	456740	252870	321180	2
90636	109150	435970	430650	915780	208030	99128	129560	0
0	43584	0	56702	0	62585	0	152150	0
99583	108020	94495	248930	377630	101990	97430	0	0
592360	752580	970290	1050100	1888600	590250	458410	373600	2
48473	0	78430	99906	61084	77479	90821	45805	0
25793	0	174760	76668	457730	0	41597	64294	0
0	0	0	0	84176	90768	114050	116220	0
391340	393350	1072600	935550	1271100	1531700	1435900	1217400	1
0	52865	77908	69903	80230	89768	86765	102610	0
469080	251370	328000	361560	195780	199560	155480	0	1
1389600	1295400	1147800	958710	863970	507300	254960	305530	0
0	0	0	0	0	0	0	71145	0
218700	246680	317220	358560	318630	210120	0	204490	0
207110	135150	154630	119970	88106	110020	39913	79611	1
248050	394060	335870	286660	329410	154920	134530	123730	0
0	0	0	0	0	175740	0	73362	0
208740	193970	263210	290800	430160	167510	158140	127830	1
4975100	2741200	4778400	5967600	4860300	3828100	3079600	2921300	9
152350	157380	309670	316360	0	197390	131190	190050	0
326090	408370	381310	374910	245590	602800	828860	619610	0
343960	292330	213560	287760	159720	173990	125240	117690	1
1120000	1141900	801840	501360	419890	622870	843100	810860	1
72998	112640	922560	983290	885450	4573600	4135100	3054500	0
887960	707870	792230	995680	1842900	497000	278710	376790	4
142660	105460	196780	252090	428800	293610	359080	332160	0
0	0	24856	44164	100460	39064	39834	135470	0
36915	0	0	0	0	0	46499	48330	0
1375000	1162800	1439500	1685800	2761400	2878600	2825500	2800200	2
1211100	1355900	895950	1169900	1386700	2980900	2914600	2663300	1
1724300	1711000	1428400	2069000	2866000	3071800	3186800	2954000	2
671300	555710	658820	850970	846100	1798000	2011900	1928800	1
1595600	976910	1412500	1690600	2434600	3416200	2990400	2571700	1
1366600	1088400	1280600	1727400	1473700	3323200	3031400	2591800	1
1597900	1395700	1596700	2386100	2063000	4368800	4016000	4294500	5
1059400	827810	845250	1385900	1068700	1670300	2356400	2652900	1
88251	0	242570	290310	587210	67929	61331	65837	0

62838	138030	65073	47239	57923	40264	64921	59735	0
0	41074	46078	57375	62375	75751	82070	105150	0
1278700	1426200	1526400	1393800	1224000	1007800	776270	1249700	0
933500	1433800	2092100	1942800	419970	1765900	1032900	1073800	3
1003900	1032600	1384400	1647500	2873400	614050	536340	1077700	2
0	0	0	436380	212650	0	0	0	0
566770	236570	159430	81143	0	107430	120590	73783	1
326440	323410	226840	176120	119960	205100	247300	136930	1
63566	0	0	0	0	0	170270	0	0
1652500	1129000	2867300	4149900	5156700	13058000	23507000	21015000	0
161540	191300	131770	124870	301640	76308	68811	59671	1
148570	120490	227810	335750	450840	743660	783200	716630	1
2588700	2805700	2741300	2841500	3804000	2006100	1408300	1416200	8
196890	197730	266020	254990	0	631650	557970	707600	0
3150700	2885900	2580600	1933500	2427500	1545700	1249000	1438800	5
384100	335250	361260	565770	717240	998430	1018000	877370	1
994900	1014800	1018200	976710	1381600	695470	493050	417800	1
1195100	890320	899540	930320	612170	755250	435130	432740	1
292640	294960	231280	263790	0	234550	159410	114510	1
71125	46187	47066	57823	0	66312	0	111030	0
0	0	526940	671390	788030	607930	816310	1499700	0
0	0	0	0	0	0	87909	0	0
772470	597780	1228300	1391000	1975900	2670000	3066500	2530200	0
2180600	1564800	1517700	1592600	1988100	908010	1145300	1501700	5
0	0	0	0	0	0	78618	62734	0
97176	101450	0	0	0	0	0	0	0
0	0	63281	78829	80681	177200	1330800	244190	0
433230	565310	1283500	1271900	1383300	1140800	681500	611610	0
1738900	1197000	1042700	1396100	1222800	1745900	2113500	1722100	3
445700	510960	334010	346900	601120	669550	986700	852260	0
0	0	0	39568	45763	48824	81748	0	0
1599900	2605700	595350	1151800	682180	769650	890070	706790	3
1117100	1031000	1485100	1827100	1177000	1044100	760760	1087700	3
0	0	0	0	0	0	0	78655	0
331930	362960	364180	386790	423290	236200	169220	162130	3
63729	0	61745	60214	179590	59035	0	0	0
954300	913860	1038200	1058500	1561500	765520	449600	441170	2
0	0	76723	105870	101980	58531	0	0	0
68652	15934	231730	178830	238740	1650400	2956100	1844600	0
40496	28859	97072	84138	332930	32895	0	52782	0
0	0	48575	74322	54889	461520	400050	370290	0
383210	173870	343340	323290	224050	638190	609580	518120	1
177980	118950	294120	402500	206730	619400	527350	491990	1
0	0	0	0	0	48348	46178	57236	0
921160	830520	832800	643680	612570	495460	317820	441740	2
0	0	0	0	0	95330	221810	502020	0
281200	117230	120160	175110	387370	1069700	2480800	1862100	0
142690	146900	137290	153720	322350	158770	0	133940	1
47987	56349	56026	60382	76883	119530	63279	71017	0
110040	131670	111520	99099	113280	85281	92551	94163	0

751040	760050	683270	608490	816900	441060	310060	494550	3
10903000	12079000	12552000	11815000	12276000	8217400	7048300	7534000	13
192670	256050	283550	310800	111230	208430	158840	109830	0
569560	694390	871330	656580	979150	422600	524610	754360	0
154220	109990	127220	159180	132470	86706	106950	82229	0
440680	334720	402960	451370	420000	413680	374690	403330	1
1467600	1232700	3199200	3863100	2339500	9440300	11017000	10738000	3
247120	235300	186610	191020	220060	131900	186480	0	1
2211300	1825400	4556000	4601700	4645700	5658600	5023500	4824300	4
1312800	1370900	3028100	4305700	5054000	11845000	12706000	12525000	2
2398800	4190500	1119900	1267100	1586200	2813200	2833900	3516200	1
480680	377480	306490	266420	401020	239120	232860	160740	2
920030	722790	459010	391840	860410	2037800	4086500	3897300	2
105670	143070	140290	171280	0	65156	111440	76627	0
273650	342960	383090	382810	399460	238790	176510	162750	1
577770	663560	446290	387090	193780	234820	253910	196480	3
147200	473850	326460	364740	184370	268410	147250	160780	0
454450	555480	440990	462160	0	0	0	278060	1
1555000	1363000	415630	477570	918210	486180	732700	274880	3
1340400	804370	1069500	969680	1593500	659140	307080	356140	2
402650	290950	369270	454090	452880	254560	190200	338130	2
1994800	2031800	2226000	2138400	1999700	1372000	812720	697030	4
915830	635420	832850	702480	128990	545340	544230	307050	2
687760	474570	658090	576820	582760	195180	320850	319400	2
191070	291470	242650	270730	0	240240	232330	180270	0
0	177550	227190	248590	300250	82195	47862	64605	0
480590	430490	360650	274100	546690	208950	263820	638260	2
785360	696240	561670	309560	369820	443330	375380	395270	1
909520	499710	437020	528730	571190	365050	302540	435650	2
222710	199240	262840	328270	255340	231490	226750	123380	1
1437400	1044500	821490	869600	561120	527840	294760	410170	1
342730	253150	298290	371370	380600	234170	122960	170460	1
1673200	2079500	1479400	1627900	2281700	858860	503890	896700	2
674820	397080	432190	460000	368530	210330	169270	619490	2
0	0	130350	127310	204750	0	0	0	0
0	0	0	0	0	0	0	66330	0
0	0	0	253030	0	0	0	0	0
168730	238000	202010	229660	229300	142860	110270	123020	1
161790	329540	84043	0	0	0	0	0	1
163990	413420	278470	617210	0	367070	207480	452540	1
1102400	816910	592390	818430	1143700	387610	271760	206800	2
847880	939170	1022700	1021700	1122800	606790	502670	567410	1
572400	461390	673020	419460	785550	234040	208920	206390	1
217680	215250	142380	105390	0	97019	221420	0	0
283080	469620	201730	226350	386050	546290	590500	708300	0
811630	1514000	2319200	2325400	2791800	1917000	1414300	1452300	0
669700	766440	780210	1006200	976460	618910	413860	367040	2
7043300	7297200	7607700	7221000	9126600	4828700	2851700	3328500	13
4635600	4802500	4398600	3783000	2574100	3133100	1922600	1744500	12
162950	163030	121540	143660	138180	384320	392740	322100	0

1516500	1451500	3503300	5881000	3645400	18675000	20834000	24561000	2
83094	76349	105220	114720	111290	122750	86420	108780	0
260970	108500	111740	103270	118020	58533	50866	73104	2
2228000	1517900	803340	519380	934340	1422600	1681500	1710400	6
209680	311320	88495	44764	143310	88677	177930	110970	1
417950	443360	329170	349660	490890	545830	552450	453890	1
481670	609130	0	0	472590	388660	436740	573410	1
306610	262610	267790	355630	353920	436070	330120	380420	2
3543100	4024200	4202300	5860800	5817900	9241600	7529600	7313100	5
454810	454920	619780	755420	551120	966820	1187700	901990	2
1977500	1490100	1684400	2403200	3662100	3684400	3570200	3789400	2
118750	0	171080	167880	157310	353580	871170	259740	0
1787700	1946900	485480	0	209180	681210	2365500	722200	1
74636000	1.02E+08	40802000	24069000	14542000	52904000	71606000	53636000	14
399010	564920	290980	162570	0	267280	332790	108010	2
3619800	3308700	1708900	1657900	2121700	2009000	2145900	1509900	3
18851000	13931000	8738900	6856300	8812300	7992400	7917700	9574000	4
325390	306870	166520	193540	178770	182190	99703	333590	1
68009	198060	26338	25298	47138	38669	210830	251930	0
66644000	64194000	36527000	25354000	19178000	35192000	41573000	35504000	33
191020	209510	217950	133970	157280	134270	151880	114440	1
28041000	25318000	10464000	2966200	1004600	4141900	11788000	37782000	2
271440	198060	53702	0	0	79283	174670	138710	1
0	0	72009	78233	96196	330280	392420	217250	0
257740	147330	124620	153860	165200	147390	344840	120970	1
0	0	0	0	0	0	32997	58128	0
84058	61281	100730	109740	146080	64093	23803	0	2
130970	164770	64119	70916	0	71814	97404	114000	1
0	0	126190	135070	0	547910	466580	558910	0
0	59734	196150	299110	490950	598620	774060	996400	0
400680	363450	442540	345310	443610	168870	197570	218520	1
35686	0	40977	52085	161050	56550	48061	47111	0
10446000	9919800	10705000	9821900	9146100	6695400	6281900	5651700	6
58299	0	82702	101150	68678	197820	234230	142680	0
0	0	68014	78482	57350	240740	132470	137680	0
601020	234160	557720	568780	902740	240850	158350	130690	1
442780	590480	891390	994380	1551500	522080	314450	363540	0
362350	203990	298320	327100	779360	208760	139540	284510	1
756920	729150	707200	765960	1012700	640210	584470	540750	4
151780	149430	128400	144500	157050	210850	234040	279130	0
333650	465160	99638	316340	252880	86464	258000	250470	1
213600	238760	232120	413530	154670	717790	852170	658590	0
0	0	0	0	0	0	103960	65321	0
0	0	217810	148330	139900	734790	1240200	755060	0
0	51911	92912	130180	0	407180	415450	520490	0
0	95623	0	0	198680	213620	222800	303280	0
158440	155650	114940	122030	148420	179580	219110	269880	0
126830	196900	315600	348960	670500	113200	88654	0	0
0	51628	77327	64196	549180	0	0	0	0
0	90189	326230	317390	328230	151490	136650	146790	0

122690	414720	196070	251240	357800	145380	91397	0	0
101150	121400	160130	133700	327120	86577	61677	45773	1
102000	137870	287710	217450	582500	237680	88269	89034	0
32465	41485	34504	41327	0	95954	108700	52640	0
0	0	85699	63441	0	157020	0	211370	0
1179700	1022100	593270	662850	1140500	1492500	1157800	2440000	2
218000	177020	173880	168980	380500	321300	343660	268860	1
0	0	0	67126	59630	71356	53791	255130	0
0	0	0	0	0	0	75272	66574	0
219630	195280	463670	665640	313700	824950	1037300	1145000	0
122910	0	230770	158170	0	115350	0	41143	1
49277	45915	0	0	0	45900	0	143850	0
0	0	64507	0	0	70743	103480	94646	0
225480	217100	212740	351920	618310	926620	620750	798940	1
0	71802	111980	73221	0	602440	1576400	754820	0
143540	0	106890	98200	0	107020	99542	142040	0
0	0	39166	39832	65119	79722	95037	85642	0
160800	137470	204140	316620	243810	629170	564580	493530	0
30040000	31213000	34307000	28267000	31326000	26744000	25198000	26910000	9
65906	0	72416	82087	118870	238320	506910	119040	0
146820	104560	110360	0	0	0	162050	738540	0
0	0	76453	105440	0	273980	235760	166840	0
0	35912	84213	51265	0	249630	166020	467720	0
109690	177880	225470	338840	587910	952970	881640	805030	1
424350	434600	259210	226910	233970	283050	658530	492910	0
369810	402280	413320	386460	177550	234190	164500	155660	0
20521	19553	55196	113550	0	227830	226140	254220	0
1069100	776270	1541600	1904400	3749100	4838100	4708600	5283500	0
0	70708	44205	0	64858	64522	149270	168040	0
121940	120680	117870	137870	0	143000	0	132070	0
1033200	709520	1143800	1285100	1172500	616000	549900	560430	1
1340500	992370	641940	592760	813930	1394900	1623400	1514800	4
1336600	1230600	1607500	1662500	2179100	893190	479550	720690	3
224740	217100	217320	184790	209800	130150	139920	113040	1
0	63020	0	48211	58283	199470	1069300	220020	0
0	17597	28311	42504	0	114320	250650	225170	0
57261	63871	82177	52358	56137	44767	44466	38644	0
227750	267630	407310	354710	366410	392350	442380	350680	1
2110300	2128600	1415500	1868000	2311100	1112900	1273600	1202200	5
2063500	2217000	1742100	1908200	1338800	1080100	1103300	1020400	7
206460	268200	0	0	0	78483	177470	82431	1
190370	190810	194900	219540	296300	127600	116220	66755	1
0	43471	0	19679	0	0	23146	0	0
0	58835	0	86844	0	0	93347	326220	0
159570	110780	162170	161530	286960	70017	87101	66743	1
1917200	1773100	1487800	1075400	624730	1083300	709830	551930	1
226720	190390	0	0	0	208410	235240	465760	0
79468	70506	0	82759	0	139590	169100	122900	0
1525600	1562200	1667100	1827700	2950700	582720	197750	476790	1
0	0	0	0	0	84373	89779	75112	0

1142700	1214600	1805200	1912900	2297200	973300	580730	656410	1
0	0	0	117610	182870	243000	216020	113810	0
256470	339170	834210	1279900	476330	2004200	2804500	2329600	0
201020	224950	196800	0	300150	119800	0	0	1
190480	126770	177620	435690	395680	353560	203190	196780	0
0	130920	199540	163880	168230	119360	109480	87930	0
815050	303360	438950	462160	538930	598430	936930	819470	2
80406	56158	66052	102140	185980	96602	171780	0	0
0	22275	24683	0	0	37474	76966	73725	0
355950	335230	171440	199080	268950	432310	481790	469560	1
61450	35477	38858	60559	94426	21889	0	21211	1
5499500	6249300	5826800	5967600	5661200	4098300	2617600	2843600	8
0	172070	0	0	306980	0	0	0	0
1693100	1755000	1566200	1657100	1085700	1155100	757080	617130	4
1048900	968470	962640	794010	913300	626230	385960	556760	1
291750	617920	209260	295590	0	295280	338030	375140	1
162240	248740	358770	314870	404750	160160	132700	0	0
1526400	1252900	1354100	1564900	2433600	1047900	480580	560410	3
436270	652200	723650	939280	1161900	510110	337670	220370	0
384310	236470	544890	438440	917150	295560	175560	289300	0
717450	628440	1000800	1076400	783500	1030400	593710	532120	1
46276	38302	70043	0	142610	0	45418	0	0
70322	47246	86114	80815	76481	32596	0	35274	0
655490	544920	613230	695490	1069100	449220	275760	344470	2
740380	794580	595220	560050	663590	409740	347900	260620	3
375980	308210	276370	266340	518290	847190	986870	1330900	0
380030	766080	723140	1285900	2246100	4629000	5691000	2935600	0
705690	493120	422420	279920	487300	266610	0	0	0
179900	160670	149900	169290	208030	72718	46277	55759	1
18770000	20356000	14328000	14953000	27816000	10384000	7905200	8834900	13
20456000	20916000	18528000	19614000	23876000	13247000	9889900	8713600	11
3821500	4453600	4505100	4072800	2011000	2511500	1819500	1745000	1
93012	112130	150780	94968	318540	76464	68062	61623	0
531170	834890	829740	723710	0	389880	0	251630	1
435110	399400	630400	886580	656790	659320	530920	491610	1
69704	55724	150130	154680	219580	68456	0	42474	0
409150	653050	941400	1312100	1147300	966590	840070	854970	0
0	59682	108910	162180	0	79554	33549	0	0
310570	355200	478400	473840	576060	297080	221600	187510	0
301260	359360	784350	620150	491080	567790	456760	411850	0
331760	244430	606380	647910	665300	461260	340530	361690	1
626330	369660	518060	659700	1045400	550790	562060	484630	1
526740	264980	194920	272350	548680	783520	1210100	1205400	1
383190	334570	253570	280260	395680	280940	230780	211180	2
164000	174040	210610	253690	307570	103220	104290	318200	0
617570	555020	608520	623860	408520	324360	257010	292030	2
97239	100770	334530	326970	707370	84060	73258	74450	0
0	63363	82442	72667	94859	104680	88971	106020	0
0	0	0	0	0	0	45351	83330	0
54969	63948	71765	80940	92032	60699	55640	52458	1

51680	43091	46469	47765	120840	34638	0	0	0
0	79534	105080	0	0	0	29535	0	0
1871700	1678700	885620	1372000	1839200	3355600	3423400	2933100	6
1274500	1051000	1038600	1446900	1526400	4086800	3041700	4021800	3
209430	147980	677200	673230	649600	1031900	1280300	1430800	0
0	0	0	0	0	0	150990	91732	0
50391	55874	59778	76011	70527	72058	72740	84057	0
0	0	0	0	45500	74655	172750	77624	0
260040	280580	319680	320510	459570	268390	193750	260850	0
67115	81758	208850	182270	190610	93705	87511	108180	0
0	0	0	0	0	89099	98105	117750	0
109490	70023	59678	79329	77273	101530	102040	119180	1
228180	237010	244780	286790	315000	141420	85883	103550	1
167960	284500	245660	289570	0	247630	234550	269450	1
331440	257470	345640	252330	437720	189680	79846	51005	1
0	70684	96072	98304	101470	57768	0	0	0
0	0	125020	118160	136050	168690	206100	218930	0

MS/MS cou	MS/MS cou	MS/MS cou	MS/MS cou	MS/MS cou	MS/MS cou	MS/MS cou	MS/MS cou	Only identi
6	3	5	4	5	4	4	38	
2	1	0	0	2	4	4	14	
0	0	0	0	1	1	1	3	
1	4	1	2	4	4	5	22	
4	6	7	5	12	10	10	56	
0	4	1	4	2	4	7	22	
1	1	3	4	5	5	2	23	
10	12	11	12	14	15	18	100	
2	2	3	5	6	8	6	35	
4	6	7	6	8	9	8	50	
0	0	0	0	0	1	2	3	
0	1	1	1	1	0	0	4	
1	0	0	0	0	0	0	1	
5	4	4	4	4	2	3	31	
0	0	0	0	1	0	1	4	
3	0	0	0	2	3	3	12	
2	0	1	0	1	3	3	12	
1	1	0	1	1	3	3	12	
1	0	0	0	1	0	4	6	
4	1	2	3	5	6	6	31	
2	1	2	0	3	2	3	15	
0	0	0	0	2	1	5	8	
0	0	0	0	0	1	1	2	
1	1	1	1	1	3	6	14	
1	1	1	0	2	5	3	13	
0	0	0	0	2	2	4	8	
0	0	0	0	0	1	1	3	
7	3	3	2	3	3	6	31	
5	1	0	1	3	2	4	17	
0	0	0	0	1	0	2	3	
0	0	0	0	0	3	1	4	
0	0	0	0	0	2	1	3	
0	0	0	1	1	1	2	5	
0	0	0	0	0	4	4	8	
1	0	0	1	0	0	0	3	
1	0	0	1	0	0	0	2	
2	2	1	1	1	1	1	10	
1	1	2	2	1	2	1	11	
0	1	1	1	2	0	1	8	
1	0	0	0	0	0	0	2	
2	2	1	1	0	1	0	10	
1	2	1	0	1	1	0	6	
1	0	0	1	0	0	0	4	
2	1	1	0	1	0	0	6	
0	1	2	0	0	0	0	4	
0	0	2	1	1	0	0	4	
2	1	1	0	0	0	0	4	
1	0	0	0	1	0	0	2	

2	1	2	1	1	0	0	8
1	0	0	0	1	0	0	2
12	5	8	6	3	3	3	46
0	1	0	1	0	0	0	3
0	2	1	1	0	0	0	4
2	3	1	1	1	1	1	11
1	0	0	1	1	0	0	5
1	1	2	1	0	0	0	5
0	1	1	1	1	0	1	6
2	0	0	0	0	0	0	4
1	0	0	1	0	0	0	2
0	0	1	1	0	1	1	5
0	0	1	0	0	0	0	2
1	0	0	0	0	0	0	1
1	1	1	1	0	0	0	5
2	0	0	1	1	0	0	5
0	0	0	1	0	0	0	2
1	0	0	1	0	1	0	4
1	2	0	0	0	0	0	4
1	1	1	2	1	0	0	7
1	1	0	0	0	0	0	3
1	0	0	1	1	2	1	9
7	6	8	7	9	4	4	51
1	1	1	1	0	0	0	5
0	1	1	1	1	0	1	6
0	1	1	1	1	0	1	5
1	0	2	0	1	3	4	12
7	5	3	5	4	7	8	43
5	3	1	2	2	7	6	30
4	2	2	1	2	3	5	24
4	3	3	4	2	5	4	29
4	3	1	1	3	3	2	18
2	1	1	1	3	5	3	19
4	5	3	3	4	6	4	34
4	2	3	0	4	3	5	25
2	2	1	1	4	6	7	26
5	4	4	2	6	9	6	43
3	2	1	2	2	2	3	17
0	1	0	0	2	2	4	9
1	1	1	0	1	4	1	10
1	2	1	1	2	2	2	12
2	2	2	1	3	1	4	17
2	1	0	0	1	2	1	8
4	3	2	2	3	3	4	24
2	2	2	1	2	2	2	14
0	0	1	0	1	2	0	5
9	5	7	5	8	10	10	62
1	1	1	1	1	1	1	8
11	3	5	6	6	10	10	56
7	4	2	1	4	8	6	39

3	3	2	1	2	3	5	23
8	2	1	4	2	4	6	32
2	1	0	0	1	3	2	9
7	6	5	4	7	10	6	52
6	5	4	2	5	9	4	40
8	2	1	4	8	8	7	42
19	17	18	13	17	15	18	136
0	1	0	0	0	1	1	4
1	1	2	0	0	1	1	7
29	26	28	23	24	22	23	199
4	2	4	0	8	4	8	34
0	0	1	0	0	1	0	2
6	4	5	2	2	6	5	33
8	5	4	4	3	3	3	36
5	1	1	2	4	5	7	28
2	2	1	2	1	2	1	14
1	0	0	1	0	0	0	2
3	4	4	4	6	4	6	34
5	3	3	1	5	3	6	31
4	5	4	1	7	5	5	35
3	2	2	1	3	4	4	22
3	2	3	2	4	4	4	27
4	1	0	0	0	4	4	14
3	4	1	2	4	4	3	24
4	4	3	2	3	2	4	26
1	2	0	1	1	2	3	12
0	0	2	2	2	1	1	10
5	3	3	2	4	3	2	26
4	0	2	0	3	4	6	21
5	1	2	1	1	4	4	21
1	0	3	2	2	3	6	18
2	0	1	0	1	5	5	19
3	3	2	1	2	2	3	19
4	4	2	2	3	3	3	24
0	1	1	1	1	1	2	7
11	6	6	3	9	14	8	67
6	2	2	2	4	4	3	27
3	2	0	3	1	0	1	11
4	3	4	3	3	4	5	28
3	3	3	3	2	5	3	25
2	1	1	1	0	2	3	11
2	1	1	4	4	3	2	19
3	2	2	4	2	3	1	19
1	1	1	0	1	1	1	7
2	1	1	1	1	3	2	11
0	0	0	0	0	1	1	2
12	10	5	7	6	6	12	67
5	6	5	2	6	10	7	46
7	5	4	1	4	7	7	42
6	3	2	3	5	4	5	32

10	4	7	3	6	8	8	55
1	1	1	1	0	1	0	6
3	3	3	5	4	2	5	27
2	2	3	1	3	5	5	23
0	0	0	0	0	1	2	3
1	1	1	1	5	5	8	22
0	0	0	0	1	1	1	3
0	0	0	0	0	0	1	1
27	23	20	22	18	24	24	182
4	2	6	2	3	4	1	26
0	0	0	0	1	0	1	2
0	1	0	0	0	0	1	3
0	0	0	2	0	0	0	2
0	0	0	0	2	1	0	3
5	4	3	3	3	1	3	26
1	0	0	1	1	0	1	5
0	0	0	0	2	3	3	8
0	0	0	0	0	0	1	1
5	3	3	4	2	2	4	25
1	0	0	0	0	1	0	2
32	26	29	26	36	42	33	255
1	1	1	1	2	3	2	13
1	3	3	4	1	3	1	17
1	1	1	1	2	4	4	14
0	1	1	1	1	2	1	7
0	0	0	0	3	1	1	5
0	0	0	0	0	1	0	1
0	0	1	1	1	2	2	8
0	0	0	0	2	0	1	3
0	0	0	0	0	0	1	1
2	3	5	1	4	7	7	32
1	2	1	2	2	3	3	15
0	2	1	0	4	5	4	16
0	0	0	1	0	0	0	1
1	1	0	0	0	0	0	2
0	0	0	0	0	2	0	2
5	3	4	1	3	1	1	21
2	1	1	1	2	1	1	11
0	1	0	0	2	3	2	8
1	1	1	1	1	0	0	6
0	1	1	2	0	0	0	5
4	4	4	4	0	1	1	22
0	0	1	0	0	1	1	4
0	0	0	0	1	2	2	5
1	0	0	0	0	0	0	1
1	0	0	0	3	2	2	8
1	1	0	1	2	1	0	7
0	0	0	0	1	1	1	3
1	2	2	0	6	11	9	35
1	0	0	2	4	3	4	18

0	2	3	1	0	1	0	7
0	0	2	1	1	3	4	11
0	0	0	0	0	0	2	2
0	0	0	0	3	2	1	6
1	3	1	3	8	4	10	30
5	4	2	1	4	4	4	29
0	0	0	0	2	0	1	3
13	8	6	4	9	13	14	77
2	3	3	3	5	2	4	26
2	2	2	2	2	0	1	12
5	2	3	1	5	6	4	29
1	1	0	0	1	1	1	5
0	0	0	1	2	0	1	4
0	0	0	0	0	1	3	5
1	1	1	1	0	1	1	7
0	0	0	1	0	2	2	5
1	0	1	1	0	0	0	3
4	1	3	2	0	0	0	12
0	0	0	1	1	1	1	4
0	1	0	0	1	0	1	3 +
1	2	1	0	0	0	0	5
4	1	2	2	4	3	2	23
0	0	0	1	2	3	1	7
0	0	1	0	2	2	4	9
4	3	4	2	10	15	18	62
1	0	1	0	1	0	0	4
2	2	4	0	6	7	9	32
0	1	0	0	1	2	3	7
0	0	0	0	0	1	1	2
0	0	0	0	2	5	4	11
10	8	7	3	5	3	4	43
0	0	0	0	0	0	0	1
0	0	0	0	1	1	0	2
1	0	0	0	1	0	0	3
2	7	9	9	24	27	28	108
1	2	5	4	9	14	14	50
1	1	1	1	2	1	3	11
1	0	0	1	1	1	0	4
0	0	0	0	0	0	1	1
5	10	6	11	16	22	17	94
1	1	2	1	0	0	0	6
0	0	0	1	0	0	0	1
3	2	2	3	5	3	0	20
0	0	0	0	1	1	1	3
2	1	1	3	1	0	2	13
2	1	1	1	3	4	3	16
2	1	1	1	1	2	2	13
1	0	0	1	3	3	2	10
1	2	1	2	1	0	1	9
2	4	4	2	2	6	3	25

2	4	3	2	4	3	3	23
0	0	1	1	0	0	0	3
0	0	0	0	0	0	1	1
12	12	15	7	19	21	15	112
0	0	0	0	0	1	1	2
0	0	2	3	4	3	4	17
25	23	21	24	21	25	27	193
2	7	5	2	6	7	10	40
3	4	4	2	6	8	10	38
4	6	3	3	7	8	5	41
4	2	2	2	3	4	5	23
7	4	4	1	4	5	7	38
3	6	9	12	1	1	2	34
1	1	3	1	1	0	1	9
0	1	0	0	5	2	5	13
0	0	0	0	1	1	0	2
0	0	2	0	0	0	2	4
0	0	0	0	0	2	1	3
0	1	0	2	1	0	0	4
0	0	0	0	0	0	1	1
0	0	0	0	0	0	1	1
1	0	0	0	0	1	0	2
2	4	5	1	4	2	3	21
2	0	0	0	1	0	1	5
0	1	1	1	1	1	1	7
0	0	0	3	0	0	0	3
0	0	0	0	2	3	2	7
1	1	0	1	1	2	3	9
6	8	2	3	6	4	1	34
3	6	4	3	3	2	5	26
0	0	0	1	0	0	0	1
1	0	0	0	1	4	3	10
2	1	2	1	2	2	2	13
0	1	1	1	2	3	3	12
0	0	0	0	3	1	3	7
9	6	8	9	10	10	7	65
4	2	3	3	10	12	14	52
1	1	0	0	0	0	0	4
3	2	3	2	4	3	3	23
0	0	0	0	1	1	1	3
0	0	0	0	2	1	0	3
1	2	1	2	1	0	1	8
1	0	0	0	0	0	0	3
4	2	4	3	4	2	2	24
1	1	1	0	0	1	1	6
16	15	22	19	11	16	14	131
21	17	17	16	16	20	19	154
4	5	4	5	3	2	2	29
2	2	2	3	2	1	2	16
2	2	2	1	0	0	1	9

2	1	0	0	1	1	0	5
3	1	1	1	1	0	1	9
5	7	6	4	5	4	5	44
5	2	3	1	4	5	3	26
4	3	4	1	2	2	0	18
3	3	1	2	2	0	0	13
1	0	1	0	0	0	0	2
2	1	1	1	0	1	0	7
0	0	0	0	3	1	2	6
1	0	0	0	1	1	1	4
1	0	0	0	1	0	0	2
0	0	0	0	0	0	0	1
0	2	7	1	10	10	16	46
2	2	1	0	2	1	1	11
1	0	0	1	0	2	2	8
6	3	5	1	5	6	9	42
3	2	1	3	0	0	0	11
0	1	0	2	0	0	0	4
0	0	0	0	2	1	0	3
11	8	8	8	10	17	17	88
1	1	0	1	6	4	6	19
4	3	4	1	4	4	6	26
1	0	1	1	1	2	0	7
0	2	2	5	1	1	0	11
1	0	0	0	0	2	1	5
6	3	2	2	5	8	7	39
0	0	0	0	0	0	1	1
1	1	2	2	0	2	2	10
0	1	0	1	0	0	0	2
0	0	0	1	0	0	0	1
0	0	0	0	0	0	1	1
0	1	0	1	0	1	1	5
0	0	0	0	1	1	0	2
1	2	2	0	2	1	2	10
1	0	0	0	0	0	0	1
0	0	0	0	2	2	5	9
0	0	0	0	1	2	2	5
5	6	4	2	4	2	3	31
2	4	3	4	1	1	2	19
0	0	0	0	0	0	0	1
1	2	2	3	2	0	2	13
0	1	1	2	1	0	1	6
4	4	2	2	0	2	5	22
0	1	1	1	0	0	0	3
0	0	0	0	0	1	0	1
1	0	1	0	0	0	1	3
0	1	0	1	2	3	1	8
0	0	0	0	0	3	0	3
6	4	1	2	10	9	11	49
0	0	0	1	0	0	0	4

0	0	0	0	5	8	6	20
0	0	1	0	0	0	0	1
0	0	1	1	2	1	0	5
1	1	1	1	1	1	1	7
2	2	2	2	2	2	3	16
0	0	0	0	0	0	1	1
0	0	0	0	0	1	2	3
2	1	2	0	1	0	1	7
0	0	0	0	0	1	2	3
0	0	0	0	4	1	2	7
1	0	0	0	0	0	0	1
0	1	0	0	1	3	3	8
6	2	4	2	8	11	9	48
1	1	3	3	3	3	0	17
0	0	0	1	0	0	0	1
10	3	4	5	7	8	10	55
0	1	0	1	1	1	1	5
2	2	4	4	1	0	1	17
0	1	2	1	1	1	1	8
0	0	0	0	0	0	0	0
10	13	9	5	11	11	6	78
3	2	1	1	1	1	2	14
5	5	7	4	4	2	2	31
0	1	1	0	4	5	4	15
15	11	11	6	13	11	16	98
2	2	2	1	4	4	5	21
0	2	2	2	5	4	4	20
0	0	0	0	2	3	6	12
0	0	0	0	3	1	2	6
0	0	0	1	3	7	5	16
17	10	10	10	9	7	12	89
0	0	1	1	1	0	1	4
0	1	4	1	1	1	1	11
1	0	0	0	0	2	0	3
0	1	1	0	2	3	1	8
0	0	0	1	3	2	2	8
0	0	3	1	6	7	8	25
1	3	2	4	1	0	2	14
4	6	6	4	6	2	3	34
1	2	3	1	2	0	2	12
2	1	1	0	1	0	2	8
0	0	0	0	0	0	2	3
1	0	1	0	0	2	1	6
0	1	0	0	0	1	0	2
0	0	0	0	0	1	0	2
4	4	3	6	1	2	2	24
2	0	0	0	1	3	5	12
1	6	8	4	9	12	15	58
1	4	2	3	1	2	2	15
9	5	8	2	4	6	9	48

1	1	2	2	3	1	2	12
3	3	1	2	1	2	3	18
0	2	3	4	1	1	1	13
5	4	5	3	4	6	9	37
1	1	1	1	0	0	0	4
1	2	4	1	0	0	0	9
2	3	2	2	2	2	2	17
2	2	1	1	1	1	0	8
1	2	2	2	1	2	4	14
4	1	2	0	1	3	3	16
0	0	0	1	3	0	1	5
2	4	0	0	1	0	1	10
1	1	1	0	1	0	1	6
0	0	0	2	0	0	0	3
1	0	0	0	1	0	3	6
1	2	3	0	2	4	2	14
0	0	0	0	1	1	2	4
0	1	0	0	0	0	1	2
0	1	1	1	4	4	4	15
0	0	0	0	0	1	0	1 +
1	1	2	2	0	0	0	7
1	1	1	1	0	1	0	6
1	0	0	0	0	0	0	1
1	2	2	2	2	2	4	16
2	2	3	1	5	8	9	32
1	2	3	1	1	1	1	12
0	0	1	1	0	0	0	2
1	1	1	0	1	1	0	5
1	0	0	0	0	0	0	2
1	1	1	1	1	1	1	7
1	1	2	3	2	3	4	17
0	0	0	1	0	0	1	2
5	9	5	3	5	3	4	40
12	6	10	6	17	35	33	133
0	1	1	3	2	2	2	12
0	0	0	0	1	1	1	3
2	1	0	0	0	0	0	4
2	2	3	0	2	3	3	18
0	1	1	1	1	0	0	4
1	2	2	1	1	1	1	9
2	1	1	0	1	1	2	8
0	1	0	0	3	2	2	8
1	0	0	0	0	0	1	2
0	0	0	0	1	1	1	3
1	2	1	2	0	1	1	9
1	1	0	1	1	0	0	4
0	0	0	0	0	0	3	3
0	1	1	1	2	2	4	12
1	0	0	0	0	0	0	1
1	0	1	0	5	4	7	20

0	0	0	1	1	1	2	6
3	2	3	3	7	3	3	31
0	1	0	1	2	5	3	13
0	0	0	0	1	1	1	4
0	0	0	0	2	1	3	6
3	4	5	3	12	12	12	54
0	0	0	1	0	0	0	1
3	2	3	2	2	2	1	18
0	1	1	0	0	0	0	2
1	0	0	0	0	0	0	1
0	0	0	0	0	0	1	1
0	1	1	1	1	0	1	8
0	0	0	0	2	2	3	7
7	4	3	6	2	1	3	29
3	3	2	5	4	2	4	31
0	0	0	0	0	0	1	1
0	0	0	0	2	1	3	6
0	0	0	0	1	0	2	4
0	0	0	0	0	0	0	1
3	1	0	2	2	3	2	16
0	0	0	0	0	2	1	3
0	0	0	0	0	0	1	1
0	0	0	0	0	1	1	2
5	3	2	3	5	5	6	33
0	1	0	0	0	0	0	1
2	6	7	9	5	5	6	43
0	0	1	1	0	0	0	2
0	0	0	0	2	1	0	3
0	4	1	3	1	4	4	17
0	0	0	1	0	0	0	1
0	0	0	0	0	1	0	1
0	2	1	3	2	0	1	9
1	1	0	0	1	2	1	6
0	0	0	0	1	1	2	4
0	1	0	0	1	1	1	4
0	0	0	0	0	0	0	2
4	0	0	1	3	2	5	17
1	1	0	3	0	0	0	6
1	0	0	0	1	1	1	4
0	0	0	0	0	1	0	1
0	0	0	1	4	4	4	13
2	0	1	1	3	2	3	12
2	2	1	2	4	4	2	17
0	0	0	0	1	1	1	3
0	1	0	1	2	2	3	9
1	0	0	0	0	0	0	1
0	0	1	2	2	2	5	12
0	1	1	2	2	2	2	10
1	1	0	0	0	0	0	2
2	1	2	1	1	1	1	9

0	0	0	0	1	1	1	3
0	1	0	0	0	0	0	1
0	1	1	0	0	0	0	3
3	0	0	0	0	0	0	4
8	7	4	7	6	8	11	60
3	7	5	2	6	3	5	39
1	1	2	1	0	0	1	7
3	2	3	1	3	1	1	17
1	1	1	0	1	0	0	5
2	2	2	3	0	0	1	13
1	0	0	0	0	1	2	5
0	2	0	1	0	0	2	5
1	0	0	0	0	0	0	2
1	0	0	1	0	1	1	5
1	2	3	2	2	0	2	14
2	3	3	1	3	4	2	21
0	1	1	1	1	0	1	7
2	2	3	5	1	1	1	19
3	0	2	1	3	3	1	17
1	1	1	1	1	1	1	8
1	1	1	1	2	2	2	11
1	0	1	0	0	0	0	3
2	0	1	1	2	1	2	10
2	1	2	1	2	1	1	12
1	0	0	0	0	1	1	5
3	2	3	2	2	2	2	18
3	1	5	2	17	20	25	77
0	0	0	0	2	1	2	5
0	0	0	0	1	1	3	5
0	0	1	0	2	1	4	8
17	14	12	8	12	7	14	99
1	1	1	1	2	1	2	10
0	0	0	0	0	0	2	2
0	0	1	1	3	4	1	10
0	0	0	0	0	1	1	2
5	2	3	1	4	7	5	28
0	0	0	0	1	2	2	5
0	0	1	0	0	1	1	3
0	0	0	0	1	1	2	4
1	2	1	2	0	0	0	6
0	0	1	0	4	2	2	9
1	0	1	2	1	0	1	6
1	1	2	1	1	0	1	8
3	3	4	1	4	3	3	22
2	1	2	0	1	1	0	9
1	1	0	1	0	0	0	3
12	9	9	4	8	4	4	55
0	0	0	0	1	0	0	1
1	2	1	1	0	0	0	5
0	0	0	0	0	0	1	1

0	1	1	0	0	4	3	9
1	1	0	0	1	1	1	6
0	0	0	0	0	0	0	1
0	0	1	0	0	0	0	1
1	0	2	1	0	0	0	5
7	6	7	10	5	4	5	51
3	3	5	5	2	2	2	27
27	13	12	12	1	7	12	103
2	1	2	2	4	5	4	21
0	0	0	1	0	1	0	2
1	2	4	1	2	2	2	16
1	2	2	4	0	0	1	13
5	4	5	4	4	2	2	30
0	0	0	0	0	0	1	1
0	1	1	0	2	0	0	4
3	3	3	4	2	1	2	19
0	1	0	0	0	0	0	1
0	1	2	1	1	6	6	17
0	0	0	0	0	0	0	1
1	1	0	1	0	4	4	13
1	0	0	0	2	3	4	10
1	0	0	0	0	2	1	4
2	0	0	0	0	0	1	4
0	0	0	0	1	0	0	1
0	1	1	1	5	10	11	29
1	0	0	0	1	5	5	13
2	1	1	1	7	5	7	25
2	0	0	0	4	2	4	13
11	3	2	1	8	10	7	50
3	9	8	5	10	8	5	52
7	7	9	6	5	3	2	43
0	0	0	0	4	4	4	12
13	4	1	5	21	25	38	122
0	0	1	1	0	0	0	2
0	1	3	1	1	0	1	7
0	0	0	0	0	0	1	1
2	1	1	0	0	0	0	4
1	0	0	1	0	0	0	2
0	1	0	1	0	0	0	2
0	0	0	0	0	1	0	1
1	0	0	0	0	2	1	5
3	1	0	1	0	0	1	7
3	2	2	2	1	0	1	12
1	1	1	0	2	2	2	9
0	2	3	3	2	0	0	14
0	0	0	1	0	0	0	1
1	1	0	2	0	0	0	5
0	0	0	0	0	0	0	0
0	0	1	2	0	0	0	3
0	1	1	0	0	0	0	3

1	0	1	0	1	0	1	5	
0	0	0	0	0	0	1	1	
2	3	2	1	2	0	2	15	
0	0	1	2	0	0	0	4	
5	1	2	3	3	2	5	27	
18	11	13	12	6	9	12	92	
6	5	5	5	5	6	7	47	
3	3	3	3	4	1	1	21	
4	4	3	1	2	1	2	20	
1	0	0	0	0	0	0	3	
0	0	1	0	1	0	0	3	
1	1	1	1	2	1	1	9	
1	1	1	0	0	1	1	6	
0	0	0	1	0	0	0	1	+
0	2	2	1	0	0	1	6	
0	0	0	0	0	0	1	1	
0	0	0	0	0	0	1	1	
3	1	0	0	1	2	0	7	
0	0	0	0	1	1	0	2	
0	0	0	0	0	1	2	3	
1	0	0	0	0	0	2	5	
1	0	0	0	0	0	0	1	+
0	0	0	0	1	0	1	2	
1	1	1	1	1	3	3	11	
0	0	0	1	0	2	2	5	
0	0	0	0	0	0	1	1	
2	1	0	0	1	0	0	5	
5	9	10	17	2	1	8	58	
0	0	0	0	2	4	4	10	
0	1	1	0	1	0	0	3	
0	0	0	1	2	1	2	6	
1	2	0	2	1	1	0	8	
0	0	0	0	0	0	0	0	
0	0	0	0	0	0	0	2	
1	0	0	0	1	2	0	6	
0	2	1	2	2	6	3	17	
0	0	0	1	1	0	0	2	
0	0	0	1	1	0	0	2	
3	1	1	1	4	3	4	19	
2	2	1	1	6	2	6	24	
2	3	6	3	3	4	2	26	
3	6	5	5	5	4	4	38	
0	0	0	0	0	0	1	1	
2	1	2	2	5	5	7	26	
4	3	5	4	6	6	8	39	
0	0	0	0	0	1	1	2	
3	2	2	3	10	11	13	46	
16	14	12	11	26	27	32	154	
2	1	2	1	0	0	1	11	
1	1	2	1	2	1	1	11	

14	13	12	9	9	15	10	95
0	2	0	1	1	1	1	7
1	0	1	0	1	0	0	3
0	0	0	0	0	0	0	1
3	1	1	2	2	2	2	15
2	2	3	2	1	0	1	14
0	0	0	0	1	0	1	2
5	3	3	3	1	1	1	21
2	4	4	2	2	0	0	15
6	6	7	6	5	7	6	49
3	4	4	3	4	2	5	27
2	0	1	0	0	0	0	5
18	13	20	12	18	14	12	127
0	0	0	0	0	1	1	2
2	1	2	2	1	2	2	12
4	2	1	1	1	2	2	14
3	2	3	1	0	1	1	12
8	8	5	2	4	4	3	40
0	1	1	1	0	0	0	3
20	24	19	16	20	19	15	152
1	0	1	0	0	2	3	7
3	2	3	2	1	2	2	18
1	0	0	1	0	0	1	5
3	4	2	0	1	2	2	18
0	1	1	2	1	1	0	7
1	0	1	0	2	1	0	6
0	0	0	1	0	0	0	3
1	1	3	1	3	0	1	10
0	1	0	1	0	0	0	2
0	0	1	0	0	1	0	2
0	2	0	0	0	0	1	4
1	3	2	3	2	1	1	15
7	7	8	3	8	2	6	43
0	0	0	1	0	0	1	3
1	1	1	0	0	0	0	3
1	0	1	0	0	0	0	2
5	4	4	1	12	13	17	59
1	1	1	1	1	2	3	10
0	1	1	2	2	4	4	14
0	0	0	0	3	2	3	8
0	0	0	1	0	1	0	2
0	0	0	0	0	1	2	3
0	0	0	0	1	2	0	3
1	1	1	1	4	2	3	13
1	1	1	0	4	6	7	20
2	2	2	2	4	4	5	23
0	0	0	0	1	0	1	2
1	1	2	1	5	7	7	25
0	0	0	0	4	5	5	15
0	1	1	0	4	3	5	15

2	0	0	0	2	3	2	10	
3	1	0	0	4	3	6	19	
0	0	0	0	1	3	2	6	
0	0	0	0	0	3	1	4	
1	0	0	0	2	2	1	6	
1	1	1	2	6	5	3	20	
0	1	1	0	2	1	2	7	
0	0	0	0	1	0	1	2	
0	0	1	2	5	4	3	15	
0	0	0	0	1	1	0	2	
6	3	2	4	5	12	9	46	
0	0	0	0	1	0	1	2	
0	1	2	1	3	3	3	13	
0	0	1	1	4	5	5	17	
0	0	0	0	1	1	1	3	
2	2	1	2	5	6	8	28	
0	0	0	0	1	4	2	7	
1	0	1	1	1	1	4	10	
0	0	1	1	1	1	1	5	
0	1	0	1	1	1	5	10	
0	0	0	0	0	0	1	1	
0	1	0	1	1	0	2	6	
0	0	0	0	0	1	1	2	
0	0	0	1	6	4	2	13	
0	1	1	1	8	7	6	24	
0	0	0	0	1	0	1	2	
6	2	4	2	3	2	4	28	
1	0	1	2	5	6	5	20	
3	3	3	3	5	7	6	31	
1	2	4	2	5	6	6	28	
5	2	2	1	6	3	5	25	
0	0	0	0	1	3	4	8	
0	3	3	2	6	8	9	33	
0	0	1	1	3	7	8	20	
0	0	1	1	6	7	12	27	
3	1	1	0	2	4	5	18	
0	0	1	0	2	3	2	9	
3	4	3	1	2	0	2	17	
4	6	8	7	12	19	14	73	
10	10	11	15	28	29	35	151	
0	0	0	0	2	1	3	6	
2	0	1	0	0	0	0	4	
4	2	1	3	2	2	2	18	
0	0	0	0	1	3	0	5	
3	6	4	5	5	3	2	31	
0	0	0	0	0	0	0	0	+
37	35	41	38	59	70	67	379	
4	14	11	17	29	22	22	124	
0	0	0	0	0	1	2	3	
2	1	1	1	3	2	3	14	

0	0	0	0	4	3	5	12
2	4	4	1	3	3	3	22
0	0	2	4	0	1	0	7
0	0	0	1	0	2	1	4
2	1	1	0	1	3	2	13
2	1	0	0	1	0	0	4
0	0	0	1	0	0	0	1
4	10	10	7	17	19	24	94
0	2	2	3	1	1	2	11
7	6	6	2	4	6	6	42
1	1	1	1	0	1	1	7
0	1	2	4	1	1	1	11
4	4	6	4	9	7	8	45
0	0	0	0	0	2	2	4
1	3	1	2	0	1	3	13
1	1	2	0	1	1	0	7
0	0	0	0	1	3	1	5
4	3	4	4	5	3	2	29
0	3	0	0	1	0	0	4
14	3	1	2	0	0	0	32
0	0	0	0	0	0	0	1
2	2	1	0	2	1	5	13
0	0	0	0	1	1	1	3
0	0	0	0	0	0	1	1
0	1	0	0	0	0	1	2
0	0	0	0	1	1	1	3
0	0	0	0	3	1	3	7
1	4	3	0	3	9	9	30
1	3	2	1	6	5	5	24
10	9	7	6	9	8	10	66
12	8	7	8	11	8	10	73
0	0	0	0	0	0	2	2
0	0	0	0	0	1	1	2
0	0	0	0	0	2	1	3
6	3	2	2	3	4	6	32
2	1	0	0	1	2	3	10
0	0	0	1	1	2	2	6
0	0	0	0	1	0	1	2
1	1	1	0	1	1	0	5
0	0	1	0	3	2	2	8
0	0	0	2	2	1	1	6
0	0	0	0	2	3	3	8
3	3	5	3	8	8	9	42
13	12	7	6	14	13	14	89
1	1	1	0	0	0	0	4
6	4	4	4	4	2	1	31
0	2	1	2	1	0	0	7
0	0	1	1	3	0	2	7
1	0	0	0	1	1	0	3
0	0	0	0	1	1	1	3

0	0	0	0	0	0	3	3
0	0	0	0	0	0	1	1
1	2	1	2	0	0	0	7
0	0	0	0	4	4	5	13
2	1	2	4	2	2	0	16
2	0	0	2	0	0	0	6
0	0	0	1	0	0	1	2
2	2	2	1	2	3	1	13
0	0	0	0	0	1	0	1
1	1	1	1	1	1	0	7
1	2	1	2	0	1	1	9
0	0	0	1	0	0	0	1
0	0	0	0	0	0	0	0
0	0	1	2	0	0	0	3
1	1	1	2	0	0	0	5
1	0	0	0	0	0	1	3
4	2	2	2	1	0	0	12
0	1	1	1	0	0	1	4
0	0	1	2	1	0	1	6
2	5	2	3	5	5	4	29
1	0	0	1	1	1	0	5
1	1	0	0	1	2	0	6
0	0	0	0	1	1	0	2
2	1	1	0	0	1	0	5
1	1	2	3	3	1	0	12
2	3	2	2	3	2	0	16
2	2	3	1	1	1	2	13
4	5	6	8	6	4	7	45
4	4	3	1	5	4	3	28
0	1	1	1	0	0	1	4
2	1	3	1	1	1	2	11
0	1	0	1	0	0	0	2
0	0	1	0	0	0	0	1
11	6	5	4	9	12	12	64
2	0	1	2	0	0	1	6
0	0	0	1	0	0	0	1
2	1	2	0	2	2	2	13
1	5	2	5	1	0	2	17
2	4	1	0	3	1	2	14
3	4	3	5	1	0	2	19
0	0	0	1	0	0	1	2
10	9	12	12	15	23	21	109
1	1	3	4	11	9	9	39
1	1	1	1	0	1	1	6
13	13	15	8	19	18	22	120
0	0	0	1	0	0	1	2
4	3	4	5	14	12	15	61
7	7	5	7	8	5	6	51
12	10	8	11	11	11	13	86
6	10	8	2	15	18	18	81

19	21	20	15	33	40	42	214
0	0	0	1	0	0	0	1
0	0	0	0	2	3	3	8
0	0	0	0	2	1	0	3
2	1	1	1	1	1	2	11
0	0	0	0	1	0	1	2
5	1	3	5	1	2	1	23
0	0	2	0	0	0	0	2
1	1	2	2	0	0	1	7
0	1	1	1	1	1	1	6
0	0	0	0	3	1	5	9
2	2	3	2	2	2	2	17
0	1	2	1	2	3	3	12
1	1	1	0	2	3	6	16
0	2	2	1	4	12	8	29
2	1	1	1	5	7	5	27
1	0	0	1	2	5	6	15
0	0	0	0	1	3	0	4
11	3	3	4	12	13	10	63
6	5	4	3	6	9	11	50
1	0	1	1	1	0	2	8
5	2	1	0	4	5	3	24
8	8	4	6	13	14	15	76
4	2	1	2	5	9	7	34
15	6	5	5	15	18	15	92
2	1	2	3	7	9	8	33
6	3	2	3	4	5	6	34
11	9	5	5	13	13	16	82
1	0	1	1	3	1	2	9
3	2	2	5	7	8	4	37
2	5	5	5	13	14	15	60
8	6	6	5	12	10	9	58
3	2	4	3	1	0	1	15
8	6	7	4	6	5	6	48
2	2	2	1	1	1	0	10
0	0	0	0	3	6	3	12
0	0	0	0	0	1	0	1
0	0	0	0	0	1	0	1
1	1	2	1	3	1	1	10
0	0	0	1	0	0	0	1
0	0	1	0	2	3	1	7
0	0	1	1	1	0	0	3
0	0	0	0	1	1	0	2
3	1	1	1	1	2	2	12
2	1	4	2	2	1	2	16
7	9	8	10	5	4	7	56
2	1	1	1	2	2	1	11
5	10	7	6	6	3	7	50
1	1	1	0	0	0	1	4
7	4	4	4	5	5	4	39

2	1	0	0	0	0	0	3	
0	0	0	1	0	0	0	1	
2	1	1	1	0	0	0	6	
0	0	0	0	0	0	1	1	+
0	0	0	0	0	0	0	1	+
0	0	0	1	0	0	0	1	+
9	9	10	7	9	8	10	69	
2	3	3	2	4	2	4	25	
17	15	13	9	14	12	15	111	
2	4	2	1	6	4	5	26	
0	0	0	0	1	2	1	4	
1	1	0	0	2	1	1	7	
9	9	8	7	9	10	6	66	
1	1	1	0	1	1	1	7	
2	1	2	2	2	2	1	13	
3	4	3	3	2	4	3	25	
0	1	1	1	1	1	0	6	
1	1	1	1	4	7	4	20	
0	0	0	1	6	5	3	15	
1	0	1	2	0	0	0	5	
3	1	1	1	2	2	0	13	
1	1	1	0	2	0	0	5	
0	0	4	0	5	7	8	25	
4	4	3	4	3	1	1	22	
0	0	0	1	1	3	3	8	
3	5	5	1	2	1	3	23	
1	1	1	0	1	1	1	7	
3	3	3	2	5	6	3	27	
0	2	1	0	3	1	4	12	
0	1	1	1	2	0	1	6	
2	0	1	0	1	1	1	6	
4	6	6	2	7	9	13	51	
0	1	0	0	4	4	8	17	
0	0	0	0	1	2	3	6	
0	0	0	0	2	1	0	3	
1	0	0	1	0	0	0	2	
0	1	1	1	0	0	0	3	
0	1	0	1	0	0	1	4	
0	0	0	2	0	0	0	2	
0	1	0	0	1	0	0	2	
0	1	0	1	6	4	4	17	
0	0	0	0	0	0	2	2	
8	2	2	4	4	8	9	41	
2	1	3	4	0	0	1	12	
5	0	0	0	2	0	0	10	
3	4	2	3	2	1	1	18	
0	1	1	1	1	0	0	5	
10	10	10	7	6	8	8	69	
2	2	3	2	2	4	1	19	
13	10	7	10	9	6	7	74	

2	2	2	2	0	0	0	11
1	1	1	0	0	1	1	6
10	10	12	9	8	8	7	74
24	23	24	25	16	19	17	165
1	1	1	1	0	0	0	5
1	0	0	2	0	0	0	3
0	0	0	0	1	1	1	3
0	1	1	0	1	1	1	5
3	0	0	0	1	1	2	9
0	1	6	2	7	4	4	24
0	2	4	3	8	11	15	43
1	0	0	0	0	0	1	2
0	0	0	0	0	1	0	1
5	4	4	4	1	2	4	29
2	3	0	1	0	1	1	10
0	1	2	3	5	9	6	26
2	1	2	0	3	6	6	21
2	3	4	2	4	2	3	23
0	0	0	0	0	1	1	2
12	12	16	10	12	17	17	103
2	3	7	4	3	2	2	29
1	1	1	1	1	1	0	6
2	1	2	1	2	1	0	9
21	18	15	14	15	10	14	123
5	4	3	4	5	1	1	26
23	18	24	17	16	14	14	141
35	34	33	26	27	30	22	241
0	0	0	3	0	2	1	6
2	0	2	0	0	2	1	7
0	0	0	0	1	0	1	2
20	16	17	16	13	14	13	124
7	7	6	5	8	4	4	44
0	1	0	1	1	0	1	4
32	27	28	31	25	28	29	232
27	24	27	23	23	19	19	187
1	0	0	0	1	3	1	7
0	0	0	1	1	0	3	5
2	0	0	0	4	4	3	15
1	1	0	1	0	0	0	3
2	2	3	2	0	0	0	9
0	0	1	1	0	0	0	2
4	4	3	2	2	1	1	21
0	0	0	0	1	1	0	2
0	0	0	0	2	2	2	6
2	4	4	3	4	3	4	24
4	1	1	0	0	0	0	9
7	8	5	9	10	6	5	57
2	3	1	2	3	1	2	16
18	15	18	23	12	12	13	121
12	13	16	16	7	7	4	85

1	1	1	0	0	0	0	3
1	0	0	0	0	0	0	3
3	3	2	2	0	3	2	20
1	1	3	2	0	0	0	8
4	4	1	3	0	1	0	16
0	0	0	0	1	1	1	3
4	3	2	2	1	1	2	17
0	1	0	0	0	0	0	3
3	5	5	5	5	5	5	36
0	0	0	0	0	0	2	2
1	1	1	0	0	0	1	5
2	0	0	0	0	0	0	2
3	1	2	3	1	0	1	14
17	13	14	5	10	7	17	101
0	0	0	0	3	4	2	9
1	1	1	1	1	0	0	6
0	0	0	0	2	0	2	4
6	4	5	1	4	4	8	36
0	0	1	0	4	1	2	10
0	2	1	0	0	0	0	3
0	0	0	0	2	2	1	5
1	3	3	2	2	2	1	16
0	0	1	1	5	5	4	16
2	2	2	1	4	6	8	27
1	2	5	4	6	9	3	30
1	0	0	1	0	0	1	3
4	5	6	3	9	10	15	56
0	1	2	1	5	5	5	19
4	4	2	3	2	0	1	19
3	1	1	1	1	0	0	9
7	6	7	2	5	4	5	42
0	0	0	1	0	0	0	3
2	0	0	0	2	3	3	10
2	3	3	4	1	2	1	19
0	0	1	1	0	0	0	3
1	1	0	0	0	2	2	6
0	1	1	0	2	4	2	10
0	0	1	0	0	0	0	1
0	0	1	2	1	5	7	16
3	1	1	1	1	1	1	10
2	2	1	1	1	0	0	7
2	0	1	1	0	0	0	4
1	1	1	0	0	0	0	4
2	1	2	2	1	2	2	14
0	1	0	3	0	0	0	4
3	2	3	2	2	2	1	18
2	1	1	0	2	2	1	10
1	0	0	0	0	0	0	2
0	0	1	1	1	1	1	5
0	0	0	0	2	2	3	7

0	1	1	1	2	0	1	8
0	0	1	3	1	0	0	5
0	0	1	0	1	0	0	2
1	0	1	0	1	1	1	5
0	1	2	1	1	0	1	7
2	5	8	5	6	3	5	36
1	0	1	1	1	3	5	13
14	15	15	13	14	13	18	115
4	2	3	1	3	2	0	18
0	0	0	0	1	1	0	2
0	0	0	0	0	0	1	1
0	1	1	1	1	0	1	5
2	2	2	2	0	0	2	12
0	0	0	0	1	1	0	2
0	0	1	0	1	1	1	4
10	2	5	1	4	7	11	47
0	1	1	0	0	0	1	5
0	0	2	8	0	0	0	10
0	0	0	0	0	0	0	0 +
1	1	0	2	1	1	1	8
0	1	0	1	0	0	0	2
1	1	1	1	1	0	1	7
2	1	2	0	1	1	0	8
3	2	3	2	3	1	0	18
1	0	0	0	0	0	0	1
3	1	1	1	4	1	1	14
0	0	0	1	1	0	0	3
0	0	0	1	1	2	1	5
2	1	2	2	1	1	1	12
2	2	1	2	2	0	0	11
2	0	0	0	0	0	0	3
0	0	0	0	0	0	0	0
1	1	1	0	0	0	1	5
0	0	0	1	1	1	2	5
1	0	1	0	2	2	4	11
0	0	0	0	0	0	1	1
1	1	1	1	0	0	0	6
2	1	1	1	0	0	0	6
0	0	0	0	0	0	0	0
0	0	0	0	0	0	0	1
0	0	0	0	1	0	1	2
1	0	1	0	0	0	0	2
1	3	3	1	1	1	2	16
1	1	0	0	0	0	0	2
9	8	7	10	6	7	4	57
2	0	1	2	0	1	0	8
2	0	1	0	1	1	1	7
0	0	0	0	0	0	0	0 +
0	0	0	0	2	4	7	13
0	0	0	0	0	1	3	4

0	0	1	0	0	0	0	3
0	0	0	0	0	1	0	1
1	2	2	2	1	2	3	14
0	1	0	0	1	0	0	2
5	3	5	1	2	3	3	23
0	2	1	0	1	0	0	4
0	3	2	1	2	0	1	11
4	1	0	2	2	0	1	11
1	0	0	0	0	0	0	1
0	1	0	0	0	1	0	2
3	3	3	0	1	0	1	13
2	0	0	0	1	1	1	6
3	3	2	1	1	1	1	13
0	1	0	0	1	0	0	2
2	1	1	1	1	0	1	9
1	1	1	0	1	2	1	8
4	3	5	5	2	3	3	28
0	0	1	0	2	0	1	6
4	5	7	5	6	2	4	39
4	5	6	1	2	3	3	26
1	0	0	0	0	0	0	1
1	1	0	1	0	0	0	3
2	2	1	0	3	2	2	14
2	3	1	4	1	1	2	15
2	2	0	1	1	1	2	11
0	0	0	0	0	1	2	3
2	1	3	0	3	1	3	14
0	0	0	0	0	1	1	2
3	2	4	2	3	3	3	21
0	4	2	2	12	15	15	50
1	0	0	1	1	0	1	4
1	2	2	2	0	0	0	9
0	0	0	1	0	0	1	2
4	3	2	4	2	1	1	19
0	2	0	0	0	2	2	6
0	0	0	0	0	0	0	0
0	1	0	0	1	0	0	2
0	0	1	0	3	5	3	12
0	0	0	0	0	1	1	2
6	8	9	4	9	7	11	59
0	2	0	1	1	0	0	5
1	4	2	1	2	0	0	13
1	1	1	1	0	0	1	5
0	1	0	0	0	0	1	2
26	26	25	20	21	19	16	174
1	0	0	0	1	0	0	2
6	5	5	5	7	8	7	47
2	1	0	0	0	0	0	3
7	3	2	2	6	7	4	37
1	1	1	0	1	0	1	6

1	0	0	0	0	0	0	1
1	0	0	1	1	1	1	5
86	37	36	36	50	62	69	467
0	0	0	0	0	2	1	3
3	3	7	3	4	5	5	32
0	0	0	0	1	1	1	3
1	1	1	1	0	0	0	5
6	3	5	1	3	2	5	31
1	0	0	0	0	0	1	5
2	0	1	3	2	0	0	8
0	0	1	1	2	3	4	12
1	2	1	0	1	1	0	7
1	1	0	0	0	0	0	2
1	0	0	0	0	0	0	1
1	0	0	0	0	0	0	2
1	0	1	1	0	0	2	6
2	0	0	2	0	0	0	5
1	0	0	0	0	0	1	2
0	0	1	1	0	0	0	3
1	0	0	0	0	0	0	2
2	3	3	2	2	2	2	17
0	0	0	0	1	0	0	1
0	1	2	1	0	0	0	4
2	2	1	2	1	1	1	13
0	0	0	0	0	2	0	2
0	0	1	0	2	1	2	6
0	0	0	0	0	0	1	1
0	0	0	0	1	0	0	2
2	1	0	2	2	2	1	11
0	0	0	0	2	1	2	5
0	2	0	2	0	0	0	4
57	83	82	57	66	57	84	524
0	0	0	0	0	1	1	2
2	1	0	0	0	0	1	6
16	8	10	6	9	17	14	96
1	1	1	1	1	1	0	7
1	0	1	1	0	0	0	3
4	5	4	3	4	4	4	32
1	0	3	3	0	1	2	12
4	4	2	3	4	5	2	28
1	1	0	0	1	0	0	3
3	3	1	1	3	1	1	14
0	0	0	0	1	1	4	6
3	2	2	3	1	0	0	14
0	0	0	0	3	5	5	13
0	0	0	0	1	0	1	2
1	1	1	1	0	0	0	5
3	1	4	3	1	0	2	19
0	0	0	2	0	1	1	4
4	6	2	3	9	9	9	46

1	2	1	0	1	1	3	10
0	0	0	1	0	0	1	3
0	1	1	1	4	4	4	15
1	1	0	1	1	1	0	5
0	0	0	0	1	1	1	3
1	0	0	1	1	3	4	11
0	0	0	1	1	0	1	3
0	0	0	0	0	1	1	2
2	1	0	0	0	0	1	4
0	0	0	1	3	3	3	10
7	21	17	27	10	6	13	110
1	3	2	4	0	0	1	13
2	4	2	1	0	0	2	12
0	3	2	4	0	0	1	10
4	4	3	3	2	1	5	30
4	2	2	0	3	0	0	13
4	2	3	4	3	2	1	21
0	0	2	2	0	0	1	5
1	1	1	0	1	0	0	6
1	2	1	2	0	0	1	9
4	4	3	4	2	1	2	24
3	1	4	3	5	1	3	24
2	1	2	3	1	0	0	11
2	1	2	0	1	1	2	11
1	0	1	3	0	0	0	6
0	0	0	0	0	0	1	1
1	2	3	2	4	0	1	16
0	0	0	0	3	4	3	10
0	0	0	0	2	2	3	7
1	1	0	0	0	0	0	2
6	7	4	4	11	7	6	47
1	0	0	1	0	0	0	4
0	2	1	3	1	0	1	8
1	2	4	1	2	0	1	12
0	0	0	1	0	0	0	1
4	2	2	3	4	1	5	22
0	1	0	0	0	0	0	1
0	0	1	0	0	0	1	2
0	1	1	0	0	0	0	2
0	1	0	0	0	0	0	1
4	5	5	3	3	4	3	30
6	5	6	4	6	3	5	39
0	0	1	2	0	0	1	4
9	8	9	7	9	9	9	67
2	3	2	2	2	0	0	11
2	6	4	8	11	18	16	67
0	0	0	0	0	1	0	1
8	9	7	10	13	10	8	73
1	1	1	1	1	0	0	5
0	0	2	0	0	0	0	2

2	4	3	4	1	1	1	18
1	0	0	3	0	0	0	4
2	0	1	2	0	0	2	9
4	4	3	5	3	2	6	29
1	0	0	0	1	1	1	4
0	0	0	0	1	1	0	2
1	1	2	2	5	7	5	24
3	2	1	1	3	3	4	20
0	1	0	1	2	1	2	7
0	0	0	0	2	1	0	3
0	0	0	0	0	0	1	1
0	0	0	0	1	0	0	1
3	0	2	1	1	1	0	10
1	1	1	1	1	1	1	8
2	2	3	3	5	4	4	26
1	0	0	0	0	0	0	1
1	0	0	0	0	0	1	3
1	1	1	0	0	0	0	4
8	3	6	3	3	3	2	32
0	0	0	0	1	0	1	2
1	0	0	0	1	1	2	8
0	1	1	1	0	0	0	3
1	0	0	0	0	1	1	4
0	1	1	1	1	0	1	5
0	0	1	0	0	0	0	1
0	0	0	0	0	3	3	6
0	2	2	1	4	9	8	27
0	0	0	0	1	1	1	3
0	0	1	1	0	0	1	4
0	0	0	0	1	1	1	3
4	8	5	7	11	13	15	71
6	7	6	4	6	7	8	52
1	1	1	0	1	0	0	5
0	2	2	4	0	0	1	9
0	0	0	1	0	1	2	4
0	1	0	1	0	1	2	6
0	0	0	0	2	2	2	6
0	1	1	3	8	8	7	28
1	0	1	1	1	0	1	6
0	0	0	0	1	0	1	2
0	0	0	0	0	0	1	1
2	1	2	1	0	0	0	7
0	0	1	0	0	0	0	2
0	2	3	2	4	5	5	21
2	0	1	1	0	0	0	6
6	6	5	7	8	12	11	63
1	1	1	3	4	5	6	22
6	3	2	3	3	5	9	34
4	4	5	4	5	6	5	37
2	6	6	4	10	8	5	44

1	0	0	0	0	0	0	1
1	0	0	0	0	0	0	1
0	0	1	0	1	0	0	2
8	7	7	4	7	7	5	58
1	0	0	0	0	0	0	1
1	0	1	4	3	1	0	10
1	1	1	1	0	0	0	4
1	0	1	0	1	0	2	5
0	0	0	0	1	1	4	6
1	0	1	1	4	6	6	19
0	1	0	1	0	0	0	2
6	5	6	1	7	5	3	40
0	0	1	0	1	0	0	3
0	0	0	0	1	5	7	13
0	1	0	1	0	0	0	2
0	0	1	1	0	1	3	6
0	0	0	0	0	2	2	4
1	0	1	0	1	0	0	3
1	0	3	1	1	0	0	6
0	0	1	1	3	3	4	12
0	0	0	1	0	0	0	1
4	6	6	6	13	16	13	65
2	1	2	2	5	7	13	35
1	1	1	1	1	0	0	5
0	1	1	0	0	0	0	2
0	0	0	0	0	1	0	1
0	0	2	0	2	5	6	15
0	0	0	0	1	1	1	3
1	1	2	1	0	0	0	6
0	0	0	0	0	0	1	1
0	0	0	0	0	0	2	2
1	0	0	0	0	0	1	2
5	6	3	2	2	3	4	26
1	3	3	2	0	0	0	9
1	2	1	2	6	8	9	29
0	1	3	0	5	4	4	17
0	1	4	4	12	14	12	48
0	1	3	2	7	6	7	26
0	0	2	1	3	1	2	9
0	0	0	0	0	1	0	1
1	1	0	0	1	1	1	6
58	61	52	49	37	29	36	360
0	0	0	0	0	1	2	3
0	0	1	1	0	0	0	2
1	1	2	1	0	0	1	6
14	7	6	9	10	9	9	71
0	0	0	1	0	0	0	1
6	3	3	4	8	5	6	39
2	1	2	1	3	4	2	17
0	1	2	2	4	4	4	17

4	3	2	3	7	13	13	50
0	1	1	2	2	3	2	11
0	0	0	0	1	1	2	4
0	0	0	0	2	1	2	5
1	1	1	1	1	0	0	6
0	0	0	0	0	0	3	3
9	4	7	1	5	4	6	40
4	0	2	1	0	0	3	14
1	1	1	1	2	1	0	8
2	2	1	2	5	10	10	33
3	1	0	0	2	3	4	14
0	0	0	1	0	0	0	4
1	1	0	1	1	0	0	4
0	0	0	0	1	1	1	3
0	0	0	0	0	1	0	1
0	0	0	0	1	1	0	2
19	19	22	24	23	29	23	176
5	8	5	3	6	5	8	43
2	1	1	0	1	0	0	6
6	1	1	1	2	4	5	23
5	1	0	2	4	6	7	31
0	0	0	1	0	0	0	1
1	0	0	1	4	3	3	13
0	0	0	0	0	0	1	1
1	1	1	1	1	1	0	7
0	4	4	6	2	0	0	18
2	0	0	0	2	1	2	9
5	4	3	3	3	2	2	26
0	1	1	0	1	1	0	4
4	7	12	10	5	5	6	51
1	1	1	1	1	0	1	7
11	4	5	5	9	10	15	68
1	4	4	4	1	1	1	17
0	0	1	1	0	0	0	2
4	0	0	0	2	1	2	12
1	2	2	1	0	0	1	9
4	1	2	0	4	11	6	33
1	1	0	0	1	0	0	3
0	0	1	0	2	0	1	4
0	0	0	2	0	0	0	2
0	1	0	2	0	0	0	3
0	0	0	1	0	0	0	1
0	0	1	0	1	1	0	3
0	2	1	2	0	0	0	5
0	3	5	4	0	1	2	15
5	3	5	5	3	1	2	25
1	1	2	1	0	0	1	10
2	2	1	2	1	0	0	10
2	4	3	4	2	2	0	22
6	5	4	4	9	8	7	45

1	3	3	2	6	2	3	22
0	0	0	0	0	0	1	1
0	0	0	0	1	1	0	2
11	9	10	11	11	8	7	76
8	7	8	8	2	9	7	56
0	3	2	2	7	7	5	27
0	0	2	1	7	10	12	32
4	3	4	5	2	1	2	23
1	0	1	1	0	0	1	6
0	0	0	0	1	1	2	4
0	0	1	0	2	4	3	11
1	1	3	1	3	1	2	14
3	1	1	0	4	3	3	16
3	0	1	1	1	3	2	14
0	1	0	0	0	0	1	3
0	0	0	0	3	2	2	7
1	0	0	0	1	1	1	4
0	0	0	1	3	1	3	8
0	0	1	0	1	0	1	3
0	0	0	0	2	1	1	4
0	0	0	0	1	0	1	2
0	0	0	0	2	1	2	5
0	1	0	0	4	5	5	15
0	1	2	3	4	4	6	20
0	0	1	0	4	10	10	27
0	0	1	0	3	1	2	7
1	1	2	3	4	5	5	21
1	0	0	0	5	8	6	20
0	0	0	0	2	1	1	4
0	0	0	0	1	1	1	3
0	2	2	1	4	4	3	16
0	0	0	0	1	1	2	4
0	0	0	0	2	1	1	4
0	0	0	0	1	1	1	3
0	0	1	1	4	3	2	11
0	0	1	1	1	0	3	7
0	0	0	0	1	2	1	4
0	0	0	0	0	0	2	2
0	1	1	3	1	0	0	6
0	0	2	1	5	3	6	18
0	0	0	0	2	0	0	2
0	0	0	0	0	0	1	1
0	0	0	0	1	2	1	4
1	1	3	1	1	1	2	11
0	0	2	0	0	0	0	2
0	0	1	1	3	1	3	9
1	1	0	0	1	1	1	6
0	0	0	1	0	1	1	3
0	0	4	3	4	3	4	18
1	2	3	0	5	4	5	20

0	0	0	2	2	3	4	11
13	10	13	7	11	10	16	94
20	16	18	12	17	15	17	131
10	10	11	9	5	3	2	57
7	9	9	5	7	6	6	55
0	1	1	1	0	0	0	3
0	1	0	3	0	0	0	4
7	3	3	4	3	2	4	29
0	0	0	1	0	0	0	1
1	1	1	0	0	0	0	4
1	2	1	1	2	1	3	12
1	0	1	0	0	0	0	3
2	2	1	2	2	1	1	13
0	2	0	1	2	2	2	9
13	13	10	4	6	6	7	65
1	0	0	0	0	0	1	2
0	0	0	0	0	2	2	4
0	0	0	0	0	0	0	1
0	2	1	0	0	0	0	3
0	0	0	0	0	0	0	1
0	1	1	1	0	0	1	6
0	0	0	0	2	1	1	4
2	0	1	0	0	1	2	6
0	0	1	1	0	0	0	2
1	2	2	2	1	0	1	10
0	0	1	2	0	0	1	4
0	0	2	1	0	0	0	3
4	5	5	2	3	2	1	25
1	1	0	1	2	2	1	8
0	0	0	1	0	0	0	1
1	1	1	1	1	0	1	7
0	1	1	1	4	6	6	20
0	1	0	0	2	3	2	8
2	1	1	0	1	0	0	5
1	1	1	1	1	0	0	6
0	0	1	1	2	0	1	5
0	0	0	0	0	1	1	2
0	0	0	0	1	3	2	6
5	5	5	7	5	4	4	38
0	0	0	0	2	0	0	2
0	0	0	0	3	2	8	13
1	0	1	1	1	0	0	5
0	2	1	3	0	0	0	7
1	1	1	0	1	1	2	8
1	1	2	0	2	2	2	11
3	1	2	1	4	3	1	18
0	0	0	0	1	0	1	2
4	3	2	1	3	3	3	21
2	1	2	1	1	2	1	11
0	1	1	0	2	1	5	12

0	1	1	0	0	0	0	3	
1	3	3	3	2	1	3	20	
0	0	3	1	6	7	5	22	
1	2	5	2	0	0	1	11	
0	0	1	1	1	0	0	4	
2	0	0	0	2	1	1	6	
0	2	3	2	2	2	4	15	
0	0	0	0	2	0	1	3	
0	0	0	0	0	0	1	1	
4	2	2	3	3	2	3	21	
2	2	2	3	1	2	1	16	
1	1	1	0	1	0	0	5	
0	0	0	0	0	0	1	1	
0	1	0	2	0	0	0	4	
1	0	0	0	1	0	1	3	
1	1	3	1	4	4	5	20	
3	1	0	0	0	1	0	6	
0	0	0	1	5	6	3	15	
2	2	3	4	0	0	0	14	
2	2	1	1	0	1	0	8	
0	0	1	1	2	4	5	13	
0	1	1	1	1	3	3	10	
0	0	1	2	2	5	4	14	
1	0	0	0	0	0	2	4	
0	0	0	0	0	1	0	1	+
0	0	1	0	0	0	0	2	
15	12	15	8	13	16	20	114	
0	0	0	0	0	1	1	2	
0	0	0	0	0	1	1	2	
1	0	0	1	0	0	3	5	
0	0	0	0	1	2	0	3	
0	1	1	1	0	0	0	3	
0	0	0	1	0	0	1	2	
0	0	0	1	1	1	1	4	
1	0	0	1	0	0	0	2	
0	0	0	0	0	0	0	0	+
1	0	1	0	0	0	2	5	
3	3	5	2	3	1	0	22	
2	2	2	1	4	2	1	16	
1	3	4	3	1	0	0	14	
0	0	1	1	0	0	0	2	
21	19	21	25	30	29	38	201	
0	0	0	0	0	1	0	1	
0	0	3	1	10	5	9	28	
0	2	2	1	0	0	0	7	
2	2	1	1	2	0	1	12	
0	0	0	1	0	0	0	1	
1	0	3	1	4	3	4	16	
0	0	1	1	2	3	1	8	
0	1	2	1	4	8	6	22	

4	9	9	5	23	23	22	99
0	0	0	0	0	0	2	2
4	2	5	3	5	5	6	31
1	0	0	1	1	1	1	6
0	0	0	1	0	0	1	2
3	0	5	2	2	2	3	19
5	5	5	4	4	6	6	38
8	4	2	3	4	7	7	44
3	3	0	2	1	3	4	21
10	6	8	7	8	7	9	61
0	1	1	3	0	0	0	6
3	4	2	4	2	2	2	20
1	0	1	1	0	0	0	3
1	1	0	0	0	0	2	4
5	4	4	3	2	5	3	32
3	0	2	2	1	0	2	12
1	0	0	0	0	0	1	3
0	0	0	1	0	0	0	1
7	5	5	4	5	7	5	43
6	2	2	2	3	2	3	24
11	10	12	8	13	13	14	88
0	0	1	1	0	0	0	2
0	1	0	0	0	0	0	1
0	1	1	1	0	0	0	4
1	3	1	2	2	2	3	17
3	3	2	4	4	2	2	24
2	2	2	2	2	0	0	11
1	1	1	0	0	1	1	5
2	3	3	3	1	1	3	18
1	1	1	2	0	1	1	8
2	2	1	0	0	0	1	7
1	1	1	2	0	0	0	5
1	2	1	3	0	0	1	10
2	3	1	1	1	0	0	10
1	2	4	4	3	3	7	24
1	0	0	1	2	1	2	9
0	0	1	1	0	0	0	2
0	0	0	0	0	0	1	1
1	0	0	0	0	2	3	6
3	1	2	1	1	2	2	15
2	0	0	0	1	1	3	9
1	0	0	0	0	0	0	2
0	0	0	0	1	0	1	2
0	0	0	0	1	2	1	4
0	0	0	0	0	1	1	2
0	0	0	0	1	1	2	4
6	4	3	2	4	3	4	29
1	2	1	1	0	0	1	7
2	4	6	2	4	5	3	29
1	0	0	0	1	1	1	5

0	1	0	0	0	0	0	1
0	0	1	2	1	0	2	7
4	3	2	1	2	1	4	19
1	1	0	0	0	0	0	3
0	2	2	1	2	2	2	11
0	0	0	0	1	0	2	3
0	1	0	0	3	3	2	9
0	0	0	0	0	0	1	1
6	1	2	2	2	5	4	28
2	2	0	2	3	0	1	11
1	4	4	3	4	8	10	35
0	0	0	0	0	2	2	4
0	0	0	1	0	4	2	7
0	0	0	1	2	3	2	8
1	1	1	1	1	3	4	13
0	0	0	0	0	0	1	1
4	3	8	9	4	3	8	41
3	2	1	1	2	2	2	14
1	1	0	4	0	0	0	7
0	0	1	2	1	0	1	5
0	1	0	1	0	0	1	3
0	0	2	2	0	0	0	5
2	4	4	5	1	0	1	20
2	1	0	1	0	2	2	8
2	2	1	2	0	1	0	9
0	0	0	2	2	0	2	6
1	2	2	1	2	1	1	11
0	0	0	0	0	2	1	3
3	3	0	1	1	0	0	8
0	3	3	5	7	3	7	28
3	3	2	3	2	0	2	17
1	0	0	0	0	0	0	1
1	4	7	6	9	9	8	44
9	5	7	3	7	3	6	48
1	2	1	0	1	1	2	9
2	1	0	2	0	3	2	11
0	0	1	0	0	0	0	1 +
2	4	3	2	3	0	0	16
0	1	1	1	0	0	0	3
4	2	2	2	4	5	2	24
1	1	0	1	3	3	2	12
0	2	0	1	4	6	6	19
0	1	1	1	4	3	3	13
1	0	0	0	0	0	0	1
0	1	1	1	2	0	0	5
1	1	3	0	3	4	4	17
2	1	1	0	0	1	0	5
0	0	2	1	1	1	1	6
4	4	6	6	3	1	2	29
0	0	1	0	0	0	0	1

1	1	1	1	1	0	0	6
0	1	0	1	0	0	0	2
2	2	2	3	0	0	0	9
1	0	1	1	0	1	1	5
5	4	4	4	7	8	9	46
5	2	2	1	3	7	7	30
1	1	1	1	4	4	5	18
0	0	0	0	2	1	2	5
15	11	9	12	10	6	7	82
2	4	2	5	6	3	4	27
0	0	0	2	0	0	1	3
1	0	1	1	1	0	1	5
1	0	0	0	0	0	0	1
0	2	2	2	3	0	0	9
0	0	0	0	1	1	3	5
0	0	1	1	0	0	0	2
1	2	1	2	0	0	0	7
0	0	2	3	2	0	0	8
0	0	0	0	0	0	1	1
0	0	0	0	0	0	1	1
0	0	0	0	1	0	0	1
17	9	12	14	9	9	11	92
1	1	0	0	0	0	1	4
10	7	7	5	9	8	8	63
1	3	2	2	2	1	0	13
6	1	1	4	4	4	6	33
2	1	1	0	0	0	0	5
0	0	0	1	0	0	0	1
4	4	3	5	4	2	2	28
5	5	2	4	7	5	8	39
0	1	0	0	0	0	0	2
0	0	0	0	1	0	0	1
4	2	1	1	5	4	3	23
2	1	1	0	2	3	1	10
4	3	2	0	1	1	2	16
3	3	1	3	3	4	1	20
2	1	1	1	1	1	4	13
3	4	4	4	2	3	4	26
0	0	0	0	1	2	1	4
2	1	0	0	0	0	0	3
0	0	0	0	0	0	1	1
0	0	0	1	3	2	2	8
0	1	0	0	0	0	0	1
0	0	1	1	0	1	1	4
2	1	2	1	4	6	5	23
0	0	0	0	0	3	1	4
2	2	2	2	5	6	4	26
4	1	0	0	0	0	2	10
0	0	1	0	2	1	2	6
3	1	2	1	3	7	6	27

0	0	0	0	0	1	1	2
0	0	0	0	0	2	0	2
0	1	0	0	0	1	1	3
0	0	1	1	2	3	3	10
1	0	0	0	2	2	2	7
2	0	0	1	0	0	1	5
0	0	0	0	2	2	2	6
1	3	3	1	8	9	11	37
0	0	0	0	5	5	5	15
8	6	8	3	9	6	6	51
0	0	0	0	2	0	0	2
1	1	0	0	0	0	0	3
0	0	0	0	0	1	0	1
0	0	0	0	2	1	2	5
0	0	0	0	1	1	2	4
2	0	4	4	0	0	0	11
2	3	2	2	3	1	3	20
1	0	0	0	1	1	0	4
3	1	0	1	1	0	0	7
1	2	2	3	2	0	2	15
2	2	2	1	2	2	1	14
1	0	0	0	0	1	4	6
0	0	0	0	2	2	1	5
0	2	1	2	2	2	2	11
4	2	3	2	5	2	1	22
0	0	0	0	1	1	1	3
1	0	0	0	2	2	2	7
0	0	1	0	2	3	2	8
0	0	0	0	0	0	1	1
0	1	1	0	0	0	0	2
4	2	2	3	2	2	2	19
0	0	0	0	1	1	0	2
0	0	0	0	1	2	1	4
1	1	1	1	1	2	2	10
0	0	0	0	0	1	1	2
1	1	1	0	3	1	2	9
2	1	1	1	2	5	4	19
0	1	2	0	2	7	3	16
2	1	0	0	0	0	0	3
0	1	1	1	0	2	2	7
0	1	2	1	1	0	2	7
0	0	0	0	0	1	0	1
18	17	15	14	16	16	15	125
2	3	1	1	0	0	0	9
1	1	1	1	1	3	2	11
1	0	1	0	1	0	0	3
2	3	3	1	2	2	2	18
2	3	2	3	3	1	1	17
1	1	1	0	0	0	1	4
0	0	0	2	0	0	0	2

2	1	1	2	0	1	1	10
0	0	0	1	0	0	0	1
0	1	2	2	2	7	4	18
4	2	2	1	3	3	3	19
0	0	0	0	0	0	0	1
0	0	0	1	1	0	1	3
0	0	1	0	0	0	3	5
4	3	3	3	4	3	6	26
0	1	0	0	0	1	1	3
1	6	10	10	14	14	17	72
0	1	2	4	5	8	10	30
1	2	2	1	2	2	2	13
0	1	1	0	0	0	0	2
0	1	0	0	0	0	0	2
1	0	0	0	4	7	7	21
0	0	1	0	2	1	2	6
0	0	0	0	2	1	2	5
0	0	1	2	4	1	1	9
1	1	1	2	3	2	4	14
0	0	0	0	3	5	4	12
2	1	3	4	5	6	5	28
1	1	0	0	1	1	2	7
3	0	0	1	2	3	3	13
1	0	0	1	3	2	3	12
0	2	4	2	13	13	10	45
1	1	1	1	1	0	0	6
3	3	4	1	6	6	3	28
0	0	0	0	0	0	1	1
1	1	2	0	2	1	0	9
1	3	6	7	6	6	9	40
3	2	2	1	8	8	8	36
0	0	0	0	1	5	2	8
0	0	0	0	1	1	1	3
3	0	0	0	3	1	2	10
0	1	1	0	0	1	0	3
4	2	4	1	2	3	3	22
3	3	6	6	5	0	1	28
1	1	1	1	1	0	1	7
28	21	29	17	15	14	16	160
1	3	3	2	12	9	12	42
2	1	0	0	0	0	0	4
0	0	1	1	1	0	0	3
1	0	0	0	1	2	3	8
0	0	0	2	2	3	1	8
0	0	0	1	0	2	1	4
1	1	0	0	1	3	2	12
4	4	4	2	2	0	0	21
2	2	0	4	1	1	0	11
5	5	3	3	3	1	1	24
2	2	1	2	3	0	0	12

4	4	3	3	4	1	2	24
2	1	0	2	2	1	1	11
10	8	6	3	6	4	4	50
0	0	0	0	0	0	1	1
0	1	1	0	2	1	1	6
1	0	1	1	0	0	0	3
0	0	0	1	0	2	0	3
0	1	1	0	2	3	1	8
3	2	2	3	3	5	4	24
0	0	0	1	2	2	2	7
0	0	0	0	0	1	0	1
0	1	3	3	1	0	0	8
0	1	1	1	0	0	0	4
0	0	2	1	1	0	0	6
0	1	2	3	1	0	0	7
0	0	0	0	0	0	2	2
0	0	2	2	0	0	0	4
3	5	3	5	5	3	3	29
0	0	0	1	1	1	0	3
0	1	0	1	0	0	1	3
0	0	0	0	0	1	1	2
1	3	2	4	4	5	3	23
0	0	1	0	0	0	1	2
1	2	3	0	1	0	0	8
1	0	1	0	1	1	1	5
0	0	0	0	0	0	0	0
1	1	2	2	1	0	0	7
1	1	0	0	1	0	0	4
1	1	0	1	0	0	0	3
1	1	1	0	1	0	1	5
2	2	1	2	1	0	1	10
5	4	6	6	6	7	5	48
1	1	0	0	1	1	1	5
1	2	1	0	6	6	1	17
1	0	1	0	0	0	0	3
3	1	1	1	2	4	1	14
0	2	1	1	4	5	5	18
3	4	4	4	3	1	1	24
0	2	2	1	1	1	1	8
0	0	0	1	0	0	2	3
0	0	0	0	0	0	1	1
0	1	3	6	12	9	10	43
2	0	1	2	7	13	10	36
6	2	4	5	8	11	13	51
0	0	1	2	7	10	9	30
2	2	2	1	4	7	9	28
2	3	3	4	8	10	11	42
1	2	5	2	8	8	11	42
0	1	1	0	1	4	8	16
0	1	1	3	0	0	0	5

1	0	0	0	0	0	1	2	
0	0	0	0	1	0	1	2	
1	1	1	1	2	2	0	8	
3	2	3	1	2	2	2	18	
1	2	3	3	2	1	2	16	
0	0	1	1	0	0	1	3	
1	1	0	0	0	0	0	3	
1	0	0	0	0	1	1	4	
0	0	0	0	0	1	1	2	
0	1	2	2	2	5	5	17	
1	1	1	2	1	0	0	7	
0	1	1	1	1	2	3	10	
3	5	5	3	4	4	3	35	
0	1	0	0	2	3	3	9	
5	3	4	4	3	4	3	31	
0	1	1	0	4	2	1	10	
2	3	2	2	3	1	2	16	
2	3	3	2	3	0	1	15	
1	1	1	0	2	0	1	7	
0	0	0	0	0	0	1	1	
0	1	1	1	1	2	8	14	
0	0	0	0	0	0	0	0	+
0	2	4	3	8	13	12	42	
5	3	6	4	1	2	7	33	
0	0	0	0	0	1	1	2	
1	0	0	0	0	0	0	1	
0	0	0	0	0	2	1	3	
1	5	5	8	2	1	2	24	
5	2	3	3	6	10	8	40	
1	2	1	3	5	6	7	25	
0	0	0	0	0	1	0	1	
4	1	1	1	2	4	2	18	
3	3	3	3	2	2	2	21	
0	0	0	0	0	0	0	0	+
2	3	1	3	2	0	2	16	
0	0	0	1	0	0	0	1	
4	3	4	5	2	2	2	24	
0	0	1	0	0	0	0	1	
0	1	0	1	4	7	6	19	
0	1	1	1	0	0	0	3	
0	0	0	0	5	2	2	9	
0	0	1	0	1	3	4	10	
1	1	1	1	2	2	2	11	
0	0	0	0	0	1	1	2	
1	1	1	1	2	0	1	9	
0	0	0	0	0	0	1	1	
0	0	0	0	4	5	9	18	
0	2	2	2	1	0	1	9	
0	0	0	0	1	0	1	2	
1	0	2	0	0	1	1	5	

7	3	3	3	1	0	1	21
14	14	13	7	17	14	19	111
1	1	1	0	1	1	0	5
2	3	2	4	1	1	1	14
1	0	1	0	0	0	0	2
1	1	1	1	1	1	1	8
3	4	4	2	6	9	10	41
1	2	1	1	0	1	0	7
2	8	13	10	14	11	13	75
4	4	5	3	10	11	15	54
7	0	0	0	1	1	2	12
0	0	0	1	0	1	1	5
1	0	0	2	2	5	4	16
1	1	1	0	0	1	0	4
0	1	2	1	1	1	0	7
3	2	2	0	0	1	1	12
1	1	1	1	1	1	1	7
1	0	0	0	0	1	0	3
3	0	0	2	1	1	0	10
1	1	1	3	1	1	1	11
0	1	2	0	2	0	1	8
5	5	4	3	3	3	2	29
1	2	1	0	2	1	1	10
3	2	3	1	0	1	0	12
0	0	0	0	1	0	0	1
1	1	1	1	0	0	0	4
2	2	2	1	1	1	2	13
1	1	1	1	1	1	1	8
2	2	0	0	3	2	1	12
1	1	2	0	0	0	1	6
2	2	1	1	0	0	1	8
1	2	1	1	2	0	1	9
2	1	2	1	2	1	2	13
1	1	1	0	1	0	1	7
0	0	1	1	0	0	0	2
0	0	0	0	0	0	0	0 +
0	0	0	0	0	0	0	0
2	2	1	0	0	0	0	6
1	0	0	0	0	0	0	2
1	1	1	0	2	1	1	8
1	1	3	2	0	0	0	9
1	3	3	3	2	2	3	18
3	2	3	1	0	0	0	10
1	0	0	0	1	1	0	3
2	0	0	0	2	2	2	8
3	2	7	7	5	2	7	33
1	1	2	1	2	1	1	11
15	13	16	9	11	7	9	93
6	7	10	7	11	7	7	67
0	0	0	0	1	4	2	7

1	2	3	2	12	16	20	58
1	1	1	0	1	0	0	4
1	1	0	0	0	0	0	4
4	3	0	0	5	6	6	30
2	0	0	1	1	1	2	8
1	1	0	3	6	3	2	17
3	0	1	2	1	1	1	10
1	0	2	1	1	2	1	10
9	7	5	6	7	10	9	58
1	1	3	1	2	3	1	14
3	2	3	4	5	6	6	31
0	0	0	0	2	5	2	9
1	1	0	1	1	1	1	7
14	9	10	7	13	22	21	110
1	1	0	0	1	0	0	5
3	2	1	3	3	4	3	22
6	3	2	3	3	3	5	29
2	0	1	0	0	0	1	5
2	0	0	0	0	1	2	5
32	13	17	12	21	24	30	182
1	1	0	2	0	0	0	5
1	1	1	1	2	1	3	12
1	0	0	0	0	1	1	4
0	0	0	0	1	3	1	5
0	0	0	0	1	2	0	4
0	0	0	0	0	0	1	1
0	1	2	1	1	0	0	7
1	0	0	0	0	2	2	6
0	0	0	0	2	2	2	6
0	0	2	1	3	3	4	13
2	1	1	1	0	1	1	8
0	0	0	1	0	0	0	1
8	7	5	6	5	7	8	52
0	0	1	0	1	2	1	5
0	0	0	0	1	0	0	1
0	1	2	1	0	0	0	5
3	3	4	9	3	0	2	24
0	0	0	3	0	0	1	5
4	3	3	4	5	3	4	30
0	0	0	0	0	2	3	5
1	0	1	0	0	1	2	6
1	0	1	0	4	3	5	14
0	0	0	0	0	1	0	1
0	0	0	0	1	2	1	4
0	0	0	0	3	1	3	7
0	0	0	1	0	0	1	2
0	0	0	0	1	2	2	5
1	1	1	2	0	0	0	5
0	0	0	1	0	0	0	1
0	1	2	1	0	0	2	6

1	0	1	1	0	0	0	3
2	2	0	1	0	0	1	7
1	1	1	2	1	0	0	6
0	0	0	0	1	1	0	2
0	0	0	0	1	0	1	2
4	0	0	4	7	7	10	34
1	2	0	1	2	3	2	12
0	0	0	0	1	0	1	2
0	0	0	0	0	0	1	1
1	1	1	1	3	3	4	14
0	1	2	0	1	0	1	6
0	0	0	0	0	0	1	1
0	0	0	0	0	0	2	2
0	1	1	1	3	1	5	13
0	0	0	0	3	7	5	15
0	0	0	0	1	1	0	2
0	1	0	0	2	1	1	5
0	1	1	1	2	2	2	9
11	13	12	17	5	7	11	85
0	0	0	0	1	3	0	4
0	0	0	0	0	0	2	2
0	0	1	0	1	2	2	6
0	0	0	0	1	0	1	2
0	0	1	1	2	1	2	8
0	1	0	0	0	2	2	5
0	0	0	0	1	0	0	1
0	0	1	0	1	1	1	4
1	2	2	6	15	18	17	61
0	0	0	0	0	1	1	2
1	0	0	0	1	0	1	3
2	3	3	4	2	2	1	18
4	2	2	1	4	4	8	29
4	8	7	6	4	0	4	36
1	1	0	1	1	1	0	6
0	0	0	0	1	4	3	8
0	0	0	0	0	1	1	2
0	1	0	0	0	0	0	1
2	1	1	1	2	2	2	12
13	3	6	3	4	8	7	49
8	9	6	4	6	5	6	51
2	0	0	0	0	2	0	5
1	1	1	2	1	1	0	8
1	0	0	0	0	0	0	1
0	0	0	0	0	0	1	1
1	1	1	1	0	0	0	5
2	1	1	0	2	0	0	7
0	0	0	0	0	0	3	3
0	0	0	0	2	1	1	4
0	0	2	0	1	1	0	5
0	0	0	0	0	0	1	1

1	0	0	1	1	1	1	6
0	0	0	1	1	2	0	4
0	1	2	0	3	7	7	20
1	1	1	1	1	0	0	6
0	0	1	1	1	0	0	3
0	1	1	1	0	0	0	3
1	1	1	0	1	2	4	12
0	0	1	1	1	1	0	4
0	0	0	0	0	2	1	3
0	0	0	0	3	2	5	11
0	0	1	1	0	0	0	3
15	10	9	6	9	9	10	76
0	0	0	1	0	0	0	1
4	3	4	2	2	0	3	22
1	1	1	0	1	2	2	9
2	1	1	0	1	1	2	9
0	1	1	1	1	0	0	4
2	4	4	3	1	0	2	19
4	1	2	2	0	0	1	10
1	1	1	1	1	0	1	6
2	2	1	1	2	1	1	11
0	1	0	1	0	0	0	2
0	1	2	0	0	0	0	3
1	3	1	1	3	0	1	12
1	2	1	1	1	0	1	10
1	0	0	1	3	5	3	13
0	0	1	3	8	8	13	33
2	0	1	0	0	0	0	3
1	1	1	1	0	0	0	5
15	10	8	11	11	10	14	92
15	13	12	11	11	11	12	96
2	1	3	2	3	1	2	15
0	1	0	1	0	0	0	2
0	0	0	0	1	0	1	3
1	3	3	2	1	3	2	16
0	1	1	1	0	0	0	3
3	3	3	4	4	3	5	25
0	1	1	0	0	0	0	2
1	2	2	2	3	1	1	12
1	1	1	0	2	2	2	9
1	2	1	2	1	1	1	10
0	3	2	2	1	1	4	14
0	0	0	1	5	2	2	11
2	1	2	2	1	1	2	13
1	1	1	2	0	0	1	6
1	2	1	0	0	0	0	6
0	2	1	4	0	0	0	7
1	1	1	1	1	1	1	7
0	0	0	0	0	0	1	1
1	0	1	0	0	0	0	3

0	0	0	1	1	0	0	2
1	1	0	0	0	0	0	2
3	0	1	3	6	8	6	33
5	1	3	2	10	7	8	39
1	4	2	3	3	2	3	18
0	0	0	0	0	1	1	2
0	0	0	0	0	1	1	2
0	0	0	0	0	1	0	1
2	2	2	1	4	1	0	12
0	1	2	1	0	0	1	5
0	0	0	0	1	0	2	3
0	0	0	0	1	1	1	4
1	2	2	0	1	0	1	8
1	1	1	0	1	1	0	6
1	1	1	2	1	0	0	7
0	1	2	1	0	0	0	4
0	0	0	0	0	3	1	4

Reverse	Potential c	id	Peptide IDs	Peptide is r	Mod. pepti	Evidence ID	MS/MS IDs	Best MS/M
		1408	1545;3188;	True;True;T	1558;3212;	9499;9500;	5117;5118;	5117;1041
		1637	1912;2369;	True;True;F	1927;2386;	11805;118	6255;6256;	6257;7820;
		2030	7931	TRUE	7994	49001;490	25959;259	25959
		1188	1120;1927;	True;False;	1127;1942;	6855;6856;	3682;3683;	3687;6312;
		1422	101;1806;1	True;True;T	101;1820;1	596;597;11	313;314;31	316;5924;5
		1523	1117;1118;	True;True;F	1124;1125;	6840;6841;	3670;3671;	3673;3675;
		1414	1035;1165;	True;True;F	1042;1174;	6333;6334;	3395;3396;	3396;3828;
		1189	1805;1927;	True;True;T	1819;1942;	11126;111	5923;6311;	5923;6312;
		1135	1119;1927;	True;False;	1126;1942;	6847;6848;	3676;3677;	3681;6312;
		1461	1619;1927;	True;False;	1632;1942;	9921;9922;	5349;5350;	5350;6312;
		1972	1711;1877;	True;True;T	1724;1892;	10517;105	5624;6177;	5624;6177;
		2036	2778;1071	True;True	2798;1079	17297;172	9131;9132;	9132;3540
		982	10374	TRUE	10457	64342;643	34264	34264
		1679	462;971;27	True;True;T	463;978;27	2793;2794;	1543;3155;	1543;3156;
		2165	938;1919;2	True;True;T	945;1934;2	5706;5707;	3055;6297;	3055;6297;
		64	180;662;10	True;True;T	180;666;10	1085;1086;	607;608;60	607;2160;3
		1419	495;784;10	True;True;T	496;791;10	2995;2996;	1656;2562;	1656;2562;
		452	703;4144;5	True;True;T	707;4171;5	4261;4262;	2286;2287;	2287;1359
		1259	824;4143;5	True;True;T	831;4170;5	5015;5016;	2690;1359	2690;1359
		1213	579;2978;3	True;True;T	581;3002;3	3485;3486;	1914;1915;	1916;9762;
		676	1040;4142;	True;True;T	1047;4169;	6368;6369;	3414;1359	3414;1359
		32	1804;2115;	True;True;T	1818;2131;	11122;111	5921;5922;	5922;6997;
		210	959;1613	True;True	966;1626	5830;5831;	3116;3117;	3117;5336
		731	291;868;16	True;True;T	292;875;16	1764;1765;	980;2839;5	980;2839;5
		732	1842;3052;	True;True;T	1856;3076;	11374;113	6060;6061;	6061;1001
		653	1750;2002;	True;True;T	1763;2018;	10736;107	5730;6600;	5730;6601;
		739	1047	TRUE	1054	6405;6406;	3438;3439;	3438
		1573	804;1672;1	True;True;T	811;1685;1	4893;4894;	2614;2615;	2615;5503;
		788	529;2674;3	True;True;T	531;2694;3	3193;3194;	1738;1739;	1740;8777;
		1355	5137;7102	True;True	5174;7160	31780;438	17025;232	17025;232
		1634	4716;8300;	True;True;T	4746;8363;	29022;511	15475;270	15475;270
		607	1720;7218;	True;True;T	1733;7277;	10559;105	5638;2363	5638;2363
		473	3721;4349	True;True	3747;4376	23089;230	12234;122	12237;142
		1586	1448;4123;	True;True;T	1459;4150;	8873;2556	4811;1352	4811;1352
		1487	927	TRUE	934	5629;5630;	3011;3012;	3012
		178	3355	TRUE	3380	20814;208	10980;109	10981
		427	6762;1055	True;True	6810;1063	41839;418	22217;348	22217;348
		2159	787;6860;1	True;True;T	794;6908;1	4803;4804;	2572;2573;	2572;2256
		2129	2753;3210;	True;True;T	2773;3235;	17135;171	9052;9053;	9053;1046
		1488	10084	TRUE	10162	62393;623	33101;331	33101
		551	1859;5428;	True;True;T	1873;5467;	11473;114	6114;1798	6114;1799
		673	331;5816	True;True	332;5859	2000;3606	1106;1922	1106;1922
		1484	2174;7243;	True;True;T	2190;7302;	13514;135	7188;2369	7188;2369
		377	676;7265	True;True	680;7324	4093;4094;	2191;2192;	2193;2379
		2118	399;6120	True;True	400;6163	2410;2411;	1344;1345;	1345;2016
		1486	6806;9001	True;True	6854;9069	42098;420	22378;223	22378;293
		1485	2235	TRUE	2251	13886;138	7377;7378;	7377
		1489	2170	TRUE	2186	13486;134	7180;7181	7181

674	3404;8883;	True;True;T	3429;8950;	21098;2109	11139;1114	11141;2894
1490	6240;6520	True;True	6283;6566	38709;3871	20544;2141	20544;2141
422	1794;1822;	True;True;T	1808;1836;	11042;1104	5870;5871;	5871;5986;
574	10233	TRUE	10313	63402;6340	33680;3368	33682
2133	821;1764;4	True;True;T	828;1777;4	5002;5003;	2686;2687;	2687;5764;
230	3448;4985;	True;True;T	3473;5022;	21406;2140	11318;1131	11321;1649
390	7436;8301	True;True	7496;8364	45922;4592	24330;2433	24332;2705
1980	6481;6482;	True;True;T	6527;6528;	40132;4013	21288;2128	21288;2128
266	2315	TRUE	2331	14347;1434	7633;7634;	7636
407	2874;6736	True;True	2897;6784	17932;1793	9454;9455;	9454;22125
1873	178;9753	True;True	178;9829	1072;1073;	601;31957	601;31957
182	10657	TRUE	10742	66159;6616	35228;3522	35230
1882	7882;9400	True;True	7944;9471	48712;4871	25813;3061	25813;3061
294	4669	TRUE	4699	28721;2872	15321	15321
692	995	TRUE	1002	6098;6099;	3268;3269;	3268
212	5521;9267	True;True	5561;9337	34244;3424	18274;1827	18277;3016
2005	6016	TRUE	6059	37268;3726	19836;1983	19837
441	3356	TRUE	3381	20822;2082	10982;1098	10983
1797	1172;2865	True;True	1181;2888	7190;7191;	3857;9434;	3857;9435
1885	9225;9539	True;True	9295;9612	56869;5687	30063;3006	30064;3115
1964	11179	TRUE	11264	69424;6942	36880;3688	36881
1644	122;4335;1	True;True;T	122;4362;1	717;718;71	397;398;39	401;14187;
1922	1689;2011;	True;True;T	1702;2027;	10373;1037	5563;5564;	5564;6629;
1185	1719;4045	True;True	1732;4071	10552;1055	5635;5636;	5635;13234
706	3014	TRUE	3038	18775;1877	9892;9893;	9892
1324	8859	TRUE	8926	54549;5455	28846;2884	28849
1271	237;2041;4	True;True;T	237;2057;4	1438;12660	795;6735;1	795;6735;1
1428	1312;2008;	True;True;T	1322;2024;	8046;8047;	4351;4352;	4351;6612;
1122	1989;5489;	True;True;T	2005;5529;	12343;1234	6544;6545;	6547;18163
1427	1909;3776;	True;True;T	1924;3802;	11791;2341	6250;12404	6250;12407
1423	215;1496;2	True;True;T	215;1508;2	1306;1307;	716;717;71	719;4969;8
648	1254;3200;	True;True;T	1263;3225;	7709;19884	4152;10453	4152;10453
1421	683;1639;3	True;True;T	687;1652;3	4133;4134;	2219;5401;	2219;5401;
972	1820;1821;	True;True;T	1834;1835;	11229;1123	5973;5974;	5980;5985;
1425	386;4954;8	True;True;T	387;4991;8	2338;2339;	1296;1297;	1296;16343
1231	1998;2483;	True;True;T	2014;2501;	12404;1240	6586;6587;	6586;8152;
1052	254;998;12	True;True;T	254;1005;1	1531;1532;	860;861;86	862;3281;4
1383	1941;6026;	True;True;T	1957;6069;	12023;1202	6380;6381;	6383;19876
1464	1599;1600;	True;True;T	1612;1613;	9818;9819;	5295;5296;	5295;5297;
1424	480;734;36	True;True;T	481;741;37	2889;2890;	1602;1603;	1602;2396;
25	5439;7044;	True;True;T	5478;7099;	33674;3367	18024;1802	18025;2309
1443	74;6071;63	True;True;T	74;6114;63	431;432;43	229;230;23	230;20000;
1444	1652;6012;	True;True;T	1665;6055;	10131;1013	5443;5444;	5444;19832
1252	1732;1733;	True;True;T	1745;1746;	10627;1062	5673;5674;	5674;5682;
1445	2320;9612	True;True	2336;9685	14379;1438	7649;7650;	7656;31417
1426	3696;11384	True;True	3722;11469	22923;7060	12151;3750	12151;3750
1111	156;288;14	True;True;T	156;289;14	931;932;93	526;527;96	526;975;49
468	3373	TRUE	3398	20920;2092	11039;1104	11045
1403	153;748;44	True;True;T	153;755;45	916;917;91	514;515;51	520;2440;1
1437	1418;3003;	True;True;T	1429;3027;	8703;8704;	4718;4719;	4718;9852;

718	820;4051;5	True;True;T	827;4077;5	4994;4995;	2684;2685;	2685;13260
1439	1645;6034;	True;True;T	1658;6077;	10079;1008	5417;5418;	5421;1989
1416	2555;4428;	True;True;T	2574;4457;	15892;1589	8390;14518	8390;14518
1420	179;2504;4	True;True;T	179;2523;4	1077;1078;	602;603;60	606;8228;1
4	4298;4572;	True;True;T	4325;4602;	26558;2655	14088;1408	14088;1500
109	182;539;29	True;True;T	182;541;29	1093;1094;	611;612;61	612;1761;9
963	203;204;14	True;True;T	203;204;14	1209;1210;	670;671;67	670;673;48
1976	4127;7563	True;True	4154;7624	25592;2559	13544;1354	13544;2474
1097	5798;9688	True;True	5840;9763	35940;3594	19157;1915	19157;3170
999	112;764;12	True;True;T	112;771;12	657;658;65	358;359;36	364;2501;4
502	369;1128;1	True;True;T	370;1135;1	2210;2211;	1227;1228;	1230;3712;
933	59;5309	True;False	59;5347	346;347;34	177;178;17	178;17664
934	4822;4823;	True;True;T	4855;4856;	29756;2975	15888;1588	15893;1590
1137	554;2406;3	True;True;T	556;2423;3	3338;3339;	1822;7917;	1822;7918;
1449	1055;1056;	True;True;T	1062;1063;	6464;6465;	3467;3468;	3468;3469;
1450	261;826;77	True;True;T	261;833;77	1579;1580;	885;2692;2	885;2693;2
1451	212;261;77	True;False;	212;261;77	1286;1287;	708;709;88	708;885;25
1164	1349;2432;	True;True;T	1359;2449;	8262;8263;	4473;4474;	4477;7991;
1130	2064;3619;	True;True;T	2080;3645;	12806;2240	6815;11890	6815;11896
1237	323;1222;5	True;True;T	324;1231;5	1949;1950;	1079;1080;	1084;4050;
398	684;1297;6	True;True;T	688;1306;6	4136;4137;	2220;2221;	2227;4285;
1405	7603;7960;	True;True;T	7665;8023;	46969;4697	24894;2489	24894;2603
36	2698;3783;	True;True;T	2718;3809;	16792;1679	8860;12430	8860;12436
537	3473;4882;	True;True;T	3498;4915;	21577;2157	11414;1141	11419;1610
721	484;1772;4	True;True;T	485;1785;4	2913;2914;	1611;1612;	1612;5792;
530	4288;1115	True;True	4315;11230	26520;2652	14054;1405	14057;3680
689	433;5174	True;True	434;5211	2630;2631;	1471;1472;	1471;17149
1442	2167;4013;	True;True;T	2183;4039;	13448;1344	7159;7160;	7160;13140
197	3252;5577;	True;True;T	3277;5617;	20166;2016	10631;1063	10638;1843
300	1226;4001;	True;True;T	1235;4027;	7527;7528;	4067;4068;	4068;13081
1404	1451;3231;	True;True;T	1462;3256;	8889;8890;	4816;10537	4816;10539
1406	7312;11088	True;True;T	7371;11173	45157;4515	23932;2393	23935;3660
449	6833;7532;	True;True;T	6881;7593;	42281;4228	22497;2249	22501;2464
591	4173;7207;	True;True;T	4200;7266;	25876;4451	13710;2359	13710;2359
1279	808;5635	True;True	815;5676	4922;4923;	2637;2638;	2637;18645
1232	348;431;14	True;True;T	349;432;14	2091;2092;	1164;1165;	1164;1462;
1448	5450;6755;	True;True;T	5489;6803;	33758;3375	18063;1806	18064;2219
251	2613;2926;	True;True;T	2633;2950;	16261;1626	8612;8613;	8612;9604;
361	95;2515;32	True;True;T	95;2534;32	559;560;56	290;291;29	291;8256;1
1290	347;1150;5	True;True;T	348;1157;5	2083;2084;	1156;1157;	1157;3773;
472	8020;10733	True;True	8083;10818	49482;4948	26193;2619	26195;3548
314	477;507;24	True;True;T	478;508;24	2871;2872;	1590;1591;	1592;1685;
2140	2261;2845;	True;True;T	2277;2866;	14033;1403	7443;7444;	7450;9341;
565	4258;5733	True;True	4285;5775	26379;2638	13981;1895	13981;1895
280	1078;5358;	True;True;T	1085;5396;	6616;6617;	3563;3564;	3563;17803
667	11501	TRUE	11587	71429;7143	37919;3792	37920
1218	1;134;2970	True;True;T	1;134;2994	5;6;7;8;9;1	5;6;7;8;9;1	10;443;973
1270	1633;3458;	True;True;T	1646;3483;	9994;9995;	5375;5376;	5377;11362
1514	526;1061;2	True;True;T	528;1068;2	3180;3181;	1734;3491;	1734;3491;
196	2132;4475;	True;True;T	2148;4505;	13240;1324	7059;7060;	7061;14676

1435	467;468;44	True;True;	468;469;44	2822;2823;	1561;1562;	1561;1570;
1725	4538	TRUE	4568	27920;279	14880;148	14881
1452	852;919;11	True;True;	859;926;11	5188;5189;	2776;2777;	2781;2977;
1194	1521;3157;	True;True;	1533;3181;	9348;9349;	5041;1032	5041;1033
675	5509;6800	True;True	5549;6848	34180;420	18241;223	18241;223
1327	442;2032;3	True;True;	443;2048;3	2688;1260	1490;6700;	1490;6700;
719	2559;6775	True;True	2578;6823	15909;159	8397;2226	8397;2226
1219	3265;1086	True;True	3290;1095	20251;202	10684;359	10684;359
1000	546;1404;1	True;True;	548;1414;1	3272;3273;	1774;1775;	1774;4665;
2075	3187;6518;	True;True;	3211;6564;	19797;197	10410;104	10411;214
1752	1660	TRUE	1673	10183;101	5464;5465	5464
135	5110;9750;	True;True;	5147;9826;	31619;316	16944;319	16944;319
173	2731	TRUE	2751	17002;170	8975;8976	8976
1959	1027;1077	True;True	1034;1086	6293;6294;	3377;3562	3377;3562
1119	2164;3085;	True;True;	2180;3109;	13423;134	7145;7146;	7147;1011
748	5168	TRUE	5205	31965;319	17125;171	17126
599	1315;1765;	True;True;	1325;1778;	8065;8066;	4359;5765;	4359;5765;
1853	4638;8827	True;False	4668;8894	28533;285	15216;287	15216;287
174	1718;3229;	True;True;	1731;3254;	10549;105	5634;1053	5634;1053
1381	391;1073;1	False;False	392;1080;1	2359;2360;	1308;1309;	1315;3539;
1466	391;1073;1	True;True;	392;1080;1	2359;2360;	1308;1309;	1315;3539;
1438	391;1073;1	False;False	392;1080;1	2359;2360;	1308;1309;	1315;3539;
890	471;6018;7	True;True;	472;6061;8	2844;2845;	1578;1983	1578;1984
1397	1255;1741;	True;True;	1264;1754;	7710;1068	4153;5700;	4153;5701;
770	1156;2961;	True;True;	1164;2985;	7060;7061;	3792;9705;	3792;9705;
771	1450;4741;	True;True;	1461;4771;	8882;8883;	4814;4815;	4814;1555
310	6038	TRUE	6081	37406	19907	19907
100	293;2545;9	True;True;	294;2564;9	1776;1777;	982;983;83	982;8360;3
213	5469	TRUE	5509	33886;338	18111;181	18112
1933	573	TRUE	575	3458;3459;	1899	1899
1396	302;2029;3	True;True;	303;2045;3	1836;1837;	1016;1017;	1016;6695;
1345	1463;2697;	True;True;	1474;2717;	8969;8970;	4848;4849;	4850;8853;
874	1429;1490;	True;True;	1440;1502;	8757;8758;	4745;4746;	4745;4957;
1774	9242	TRUE	9312	56962	30095	30095
752	6252;6839	True;True	6295;6887	38777;387	20578;225	20578;225
295	9845;1047	True;True	9922;1055	60868;608	32312;346	32312;346
2000	3054;3726;	True;True;	3078;3752;	19010;190	10012;100	10012;122
749	4057;7710	True;True	4083;7772	25141;251	13296;132	13298;252
96	7849;9693;	True;True;	7911;9768;	48483;484	25707;257	25709;317
2021	9700	TRUE	9775	59936;599	31781;317	31782
637	4451;7871	True;True	4480;7933	27385;273	14586;257	14586;257
2125	1900;4464;	True;True;	1915;4493;	11731;117	6231;6232;	6233;1463
57	626;908;97	True;True;	630;915;98	3789;3790;	2060;2950;	2060;2950;
871	3059;1013	True;True	3083;1021	19042;190	10028;100	10028;333
1735	8413	TRUE	8477	51823;518	27372	27372
613	216;1654;8	True;True;	216;1667;8	1314;1315;	724;725;54	724;5446;2
1814	3379;9327	True;True	3404;9397	20946;575	11059;303	11059;303
1359	36	TRUE	36	189;190;19	88;89;90	89
1112	413;582;98	True;True;	414;584;99	2511;2512;	1397;1919;	1397;1921;
511	718;792;10	True;True;	725;799;11	4374;4375;	2357;2358;	2359;2584;

1134	1083;6264	True;True	1090;6308	6638;6639;	3575;3576;	3575;2061
903	996;2885;4	True;True;	1003;2908;	6105;6106;	3273;3274;	3275;9480;
66	941;10720	True;True	948;10805	5722;5723;	3065;3543	3065;3543
1175	2570;5676;	True;True;	2589;5718;	15977;159	8444;8445;	8444;1876
1505	639;2618;2	True;True;	643;2638;2	3867;3868;	2089;8621;	2089;8623;
2018	3797;5238;	True;True;	3823;5275;	23552;235	12477;124	12479;173
1669	9387	TRUE	9458	57896;578	30578;305	30580
929	125;1514;2	True;True;	125;1526;2	746;747;74	410;411;41	411;5023;8
1017	126;1513;2	True;True;	126;1525;2	754;755;75	418;419;42	421;5014;8
1940	783;8914;1	True;True;	790;8981;1	4774;4775;	2558;2559;	2559;2902
1399	1436;4863;	True;True;	1447;4896;	8797;8798;	4766;1604	4766;1604
301	4863;4994;	False;True;	4896;5031;	29989;299	16042;160	16045;165
276	1436;4863;	False;False	1447;4896;	8797;8798;	4766;1604	4766;1604
1434	3239;4862;	True;True;	3264;4895;	20110;201	10565;105	10567;160
2043	1748;2412	True;True	1761;2429	10725;107	5727;5728;	5728;7927
177	2725;8243;	True;True;	2745;8306;	16962;169	8949;8950;	8952;2692
2145	4259;1050	True;True	4286;1058	26387;263	13982;347	13982;347
60	2060;3004;	True;True;	2076;3028;	12779;127	6802;6803;	6803;9859;
730	10653	TRUE	10738	66128;661	35211;352	35211
590	6265	TRUE	6309;6310	38858;388	20619;206	20619
508	4809;6217	True;True	4842;6260	29670;296	15832;158	15833;204
1295	905;1798;5	True;True;	912;1812;5	5494;5495;	2942;2943;	2946;5880;
1036	100;3794;7	True;True;	100;3820;7	591;592;59	311;312;12	311;12462;
1013	336;382;45	True;True;	337;383;45	2022;2023;	1117;1277;	1117;1279;
1277	740;1063;2	True;True;	747;1070;2	4505;4506;	2403;2404;	2408;3495;
1179	2252;1049	True;True;	2268;1057	13993;139	7425;3468	7425;3468
1521	496;2269;4	True;True;	497;2285;4	2999;3000;	1657;1658;	1658;7478;
229	496;2269;4	False;False	497;2285;4	2999;3000;	1657;1658;	1658;7478;
88	4172;4301;	False;False	4199;4328;	25873;258	13709;140	13709;140
1045	4297;9033;	True;True;	4324;9101;	26555;265	14085;140	14085;294
726	940;2766;2	True;True;	947;2786;2	5714;5715;	3061;3062;	3061;9094;
1994	3311	TRUE	3336	20538;205	10836	10836
445	10742	TRUE	10827	66712;667	35515;355	35515
1176	4223;1093	True;True	4250;1101	26173;261	13860;138	13861;361
1021	145;456;45	True;True;	145;457;45	886;887;88	498;499;50	498;1532;1
796	146;147;45	True;True;	146;147;45	894;895;89	503;504;50	505;507;15
149	3571;4018;	True;True;	3596;4044;	22212;222	11752;117	11754;131
1398	157	TRUE	157	939;940;94	528;529;53	530
168	7441	TRUE	7501	45946;459	24348	24348
942	119;120;10	True;True;	119;120;10	702;703;70	383;384;38	387;391;35
1680	6104;1068	True;True	6147;1076	37840;378	20107;201	20110;353
116	10067	TRUE	10145	62285;622	33041	33041
720	2083;4579;	True;True;	2099;4609;	12905;129	6871;6872;	6873;1502
1235	10867	TRUE	10952	67473;674	35899;359	35900
1096	3359;4636;	True;True;	3384;4666;	20845;208	10996;109	10997;152
1558	3426;4665;	True;True;	3451;4695;	21244;212	11227;112	11227;153
1569	3297;8432;	True;True;	3322;8496;	20448;204	10789;107	10789;274
186	266;339;49	True;True;	266;340;50	1606;1607;	901;1123;1	901;1123;1
1767	5858;9590	True;True	5901;9663	36289;362	19363;193	19368;313
433	1184;1292;	True;True;	1193;1301;	7261;7262;	3896;3897;	3897;4266;

1529	208;1050;2	True;True;T	208;1057;2	1259;1260;	694;695;69	695;3452;9
2161	8041	TRUE	8104	49618;4961	26259;2626	26261
163	5726	TRUE	5768	35506;3550	18937	18937
916	128;129;69	True;True;T	128;129;69	768;769;77	423;424;42	423;425;22
2093	4133	TRUE	4160	25629;2563	13565;1356	13566
1316	1423;1497;	True;True;T	1434;1509;	8724;8725;	4734;4973;	4734;4974;
952	252;1170;1	True;True;T	252;1179;1	1508;1509;	840;841;84	841;3839;3
1019	636;931;14	True;True;T	640;938;15	3846;3847;	2084;3024;	2084;3025;
981	102;277;14	True;True;T	102;278;14	598;599;60	318;319;32	318;941;49
974	3144;3436;	True;True;T	3168;3461;	19549;1955	10292;1126	10292;1126
1087	712;1488;2	True;True;T	719;1500;2	4352;4353;	2345;4952;	2345;4953;
1032	1352;1474;	True;True;T	1362;1485;	8279;8280;	4484;4485;	4484;4902;
1264	242;349;81	True;True;T	242;350;82	1463;2099;	816;1168;2	816;1168;2
148	8187;8612	True;True	8250;8678	50571;5057	26743;2674	26748;2806
1549	1440;1446;	True;True;T	1451;1457;	8827;8828;	4787;4788;	4788;4808;
631	505;2554	True;True	506;2573	3055;3056;	1683;8389	1683;8389
881	150;1517;6	True;True;T	150;1529;6	906;907;93	511;5028;2	511;5028;2
19	1440;5136;	False;False;	1451;5173;	8827;8828;	4787;4788;	4788;17024
39	4974;8782;	True;True;T	5011;8849;	30719;3072	16421;2857	16421;2857
753	1726	TRUE	1739	10591;1059	5651	5651
2120	3864	TRUE	3890	23978;2397	12689	12689
1847	3403;8511	True;True	3428;8575	21091;2109	11138;2768	11138;2768
917	2633;3589;	True;False;	2653;3615;	16402;1640	8678;8679;	8679;11803
205	1416	TRUE	1427	8689;8690;	4707;4708;	4708
661	562;5035	True;True	564;5072	3393;3394;	1861;16657	1861;16662
531	9081;10273	True;True	9149;10355	55950;5595	29572;3384	29572;3385
1970	2566;2858;	True;True;T	2585;2881;	15953;1595	8436;8437;	8438;9406;
1534	4667;6982;	True;True;T	4697;7035;	28705;2870	15311;1531	15314;2292
884	76;1280;15	True;True;T	76;1289;15	442;443;44	240;241;42	240;4235;5
2189	286;3155;4	True;True;T	287;3179;4	1736;1737;	962;963;96	963;10325;
55	9451	TRUE	9522	58310;5831	30860	30860
1346	1162;1681;	True;True;T	1171;1694;	7109;7110;	3816;3817;	3816;5535;
966	715;2093;4	True;True;T	722;2109;4	4362;4363;	2349;2350;	2352;6909;
798	2340;2665;	True;True;T	2356;2685;	14511;1451	7701;7702;	7702;8762;
1067	5175;7607;	True;True;T	5212;7669;	32010;3201	17152;2491	17152;2491
900	886;1400;2	True;True;T	893;1410;2	5382;5383;	2889;2890;	2889;4639;
1042	169;1032;2	True;True;T	169;1039;2	1011;1012;	564;3392;8	564;3392;8
1736	4737;8629	True;True	4767;8695	29148;2914	15533;1553	15533;2810
1555	819;2341;5	True;True;T	826;2357;5	4987;4988;	2679;2680;	2680;7703;
211	5048	TRUE	5085	31204;3120	16705;1670	16707
1837	5071;9767	True;True	5108;9844	31363;3136	16800;3203	16800;3203
247	7438;8928;	True;True;T	7498;8995;	45933;4593	24337;2433	24338;2905
494	1671;2760	True;True	1684;2780	10251;1025	5501;5502;	5501;9072
1117	4430;4431;	True;True;T	4459;4460;	27290;2729	14524;1452	14525;1453
904	6072	TRUE	6115	37611;3761	20006;2000	20007
1124	765;1060;2	True;True;T	772;1067;2	4664;4665;	2505;2506;	2506;3486;
940	194;476;49	True;True;T	194;477;49	1160;1161;	640;641;64	640;1588;1
853	425;5191;5	True;True;T	426;5228;5	2591;32100	1448;17205	1448;17206
1163	811;4568	True;True	818;4598	4943;4944;	2647;2648;	2649;14973
1369	2473;10845	True;True	2491;10930	15363;1536	8122;8123;	8124;35830

1366	963;1526	True;True	970;1538	5852;5853;	3121;5052;	3121;5052
456	5202;9819;	True;True;	5239;9896;	32177;321	17246;172	17248;322
1215	191;2879;3	True;True;	191;2902;3	1144;1145;	630;9463;9	630;9466;1
1281	3573;6570;	True;True;	3598;6616;	22228;222	11761;117	11765;215
1080	3015;8082;	True;True;	3039;8145;	18782;187	9898;2639	9898;2639
2042	1414;4919;	True;True;	1425;4956;	8677;8678;	4702;4703;	4702;1623
31	365	TRUE	366	2189;2190;	1212;1213	1212
1147	4478;9210	True;True	4508;9280	27569;275	14690;146	14692;300
1400	4023;7615;	True;True;	4049;7677;	24913;249	13159;249	13159;249
586	4674;6445	True;True	4704;6491	28759;287	15344;211	15344;211
2081	6233	TRUE	6276	38667;386	20532;205	20532
2038	4603	TRUE	4633	28323;283	15102	15102
1338	334;531;11	True;True;	335;533;11	2015;2016;	1113;1114;	1113;1742;
1071	511;9553;9	False;False	512;9626;9	3099;3100;	1698;1699;	1701;3120
964	4624;4695;	True;True;	4654;4725;	28448;284	15167;151	15171;154
1508	511;1758;2	True;True;	512;1771;2	3099;3100;	1698;1699;	1701;5754;
858	1241;1986;	True;True;	1250;2002;	7640;7641;	4125;4126;	4126;6536;
724	3980;8142;	True;True;	4006;8205;	24671;246	13026;265	13026;265
1266	84;8927;10	True;True;	84;8994;10	492;493;54	271;29051;	271;29051;
1537	37;82;296;	True;True;	37;82;297;	193;194;19	91;92;93;9	96;264;988
62	1816;3069;	True;True;	1830;3093;	11207;112	5965;5966;	5966;1006
2044	2480;2670;	True;True;	2498;2690;	15405;154	8143;8144;	8144;8769;
496	2339;8968	True;True	2355;9036	14509;145	7700;2919	7700;2920
546	816;7145;7	True;True;	823;7204;7	4972;4973;	2671;2339	2671;2339
727	540;2476;3	False;False	542;2494;3	3243;3244;	1762;1763;	1763;8136;
123	1673;1729;	True;True;	1686;1742;	10265;106	5504;5663;	5504;5663;
86	7288	TRUE	7347	45016;450	23873	23873
1821	424;5962;7	True;True;	425;6005;7	2584;2585;	1447;1970	1447;1970
1786	2508;8952	True;True	2527;9020	15587;155	8235;8236;	8236;2914
663	10750	TRUE	10835	66747;667	35525	35525
882	6157	TRUE	6200	38189;381	20279;202	20280
883	2538;4549;	True;True;	2557;4579;	15787;157	8340;8341;	8342;1492
122	2047;5033	True;True	2063;5070	12698;126	6758;1665	6758;1665
842	1532	TRUE	1544;1545	9417;9418;	5077;5078;	5084
1494	10901	TRUE	10986	67677	35986	35986
1751	322;1641;2	True;True;	323;1654;2	1946;1947;	1077;1078;	1078;5404;
413	1420;1948;	True;True;	1431;1964;	8713;8714;	4728;6406;	4728;6406;
1209	3058;4020;	True;True;	3082;4046;	19034;190	10021;100	10027;131
1322	298;5706;6	True;True;	299;5748;6	1808;1809;	998;999;10	999;18866;
1676	4377	TRUE	4406	26968;269	14337	14337
164	6886;9064;	True;True;	6934;9132;	42583;425	22616;226	22616;295
12	2272;2573;	True;True;	2288;2592;	14092;140	7483;7484;	7484;8449;
1409	8961;1036	True;True;	9029;1044	55220;552	29179;291	29180;342
1584	9044	TRUE	9112	55707;557	29451;294	29451
1247	10745	TRUE	10830	66723;667	35520	35520
1697	1611;4853;	True;True;	1624;4886;	9877;9878;	5333;1602	5333;1602
1365	2491;4313;	True;True;	2509;4340;	15477;154	8185;1412	8185;1412
1566	4400;6199;	True;True;	4429;6242;	27084;270	14400;204	14400;204
956	528;1173;1	True;True;	530;1182;1	3189;3190;	1737;3858;	1737;3858;
1033	2308;6311	True;True	2324;6357	14300;143	7603;7604;	7604;2076

1186	356;613;20	True;True;T	357;616;20	2147;2148;	1198;1199;	1198;2013;
1808	5498	TRUE	5538	34106;3410	18194	18194
48	1698;9774	True;True	1711;9851	10422;1042	5582;5583;	5583;32068
477	11530	TRUE	11616	71590;7159	38007;3800	38011
2088	6140;7649;	True;True;T	6183;7711;	38085;3808	20214;2021	20214;2504
1349	11493	TRUE	11579	71392;7139	37901	37901
2066	800;3183	True;True	807;3207	4865;4866;	2598;10403	2598;10403
1842	9162;10080	True;True	9231;10164	56462;5646	29833;2983	29833;3310
2028	5943;6599	True;True	5986;6645	36849;3685	19668;2165	19668;2166
1879	2090;3226;	True;True;T	2106;3251;	12956;1295	6897;6898;	6898;10528
617	294	TRUE	295	1781;1782;	984	984
680	2805;6043;	True;True;T	2826;6086;	17489;1749	9213;9214;	9216;19920
486	88;1468;34	True;True;T	88;1479;34	517;518;51	276;4869;1	276;4869;1
849	1649;7241;	True;True;T	1662;7300;	10108;1010	5436;5437;	5438;23695
440	7537	TRUE	7598	46554;4655	24664	24664
499	50;80;629;1	True;True;T	50;80;633;1	288;289;29	141;253;25	141;256;20
35	7228;7717	True;True	7287;7779	44659;4466	23665;2525	23665;2525
1101	1824;3338;	True;True;T	1838;3363;	11258;1125	5996;10934	5996;10934
1921	4763;4957;	True;True;T	4794;4994;	29322;3061	15628;1635	15628;1635
1778	5434	TRUE	5473	33637;3363	18004	18004
2094	445;913;17	True;True;T	446;920;17	2705;2706;	1503;1504;	1505;2958;
1729	4396;4482;	True;True;T	4425;4512;	27070;2707	14393;1439	14395;1470
242	34;1773;40	True;True;T	34;1786;40	181;182;18	83;84;85;86	84;5799;13
2010	287;2464;5	True;True;T	288;2482;5	1742;1743;	967;968;81	968;8105;1
1141	258;547;80	True;True;T	258;549;81	1559;1560;	872;873;87	873;1776;2
9	4843;8944;	True;True;T	4876;9012;	29886;2988	15988;1598	15988;2912
953	3620;3688;	True;True;T	3646;3714;	22474;2247	11899;1190	11902;1213
1069	4912;5770;	True;True;T	4949;5812;	30317;3031	16212;1621	16212;1908
113	1962;5408;	True;True;T	1978;5446;	12173;3348	6448;17940	6448;17948
223	443;1187;1	True;True;T	444;1196;1	2689;2690;	1491;1492;	1494;3906;
1140	2688;3371;	True;True;T	2708;3396;	16733;1673	8827;8828;	8832;11029
802	1670;3689;	True;True;T	1683;3715;	10250;2289	5500;12137	5500;12137
998	6706;8572;	True;True;T	6754;8636;	41508;4150	22025;2202	22030;2789
632	168	TRUE	168	1003;1004;	561;562;56	563
526	6313;7924;	True;True;T	6359;7987;	39131;3913	20764;2593	20764;2593
905	2667;8366;	True;True;T	2687;8429;	16610;1661	8765;8766;	8766;27220
1057	1970;2392;	True;True;T	1986;2409;	12221;1222	6476;7883;	6476;7883;
855	1759;2058;	True;True;T	1772;2074;	10786;1078	5755;6800;	5755;6800;
1311	2611;3499;	True;True;T	2631;3524;	16247;1624	8601;11508	8601;11509
1114	592;2072;6	True;True;T	594;2088;6	3554;3555;	1949;1950;	1949;6839;
372	3018;9959;	True;True;T	3042;10030	18802;1880	9903;32675	9903;32675
414	3364;3669	True;True	3389;3695	20870;2278	11004;1207	11004;1207
130	3227;11557	True;True	3252;11643	20035;2003	10529;1053	10529;3810
475	2539;3129	True;True	2558;3153	15795;1579	8343;10239	8343;10239
1962	7036;7778	True;True	7091;7840	43467;4346	23068;2544	23068;2544
913	218;227;22	True;True;T	218;227;22	1328;1329;	729;730;73	731;752;75
1099	4049;4103;	True;True;T	4075;4130;	25080;2508	13246;1324	13248;1343
1201	11;488;521	True;True;T	11;489;523	69;70;71;7	26;27;28;29	28;1628;17
226	381;4366;6	True;True;F	382;4393;6	2303;2304;	1271;1272;	1272;14273
313	663;3115;4	True;True;T	667;3139;4	4018;4019;	2161;2162;	2164;10205

151	1826;2312;	True;True;T	1840;2328;	11274;1127	6005;6006;	6009;7626;
1091	1129;4407;	True;True;T	1136;4436;	6910;6911;	3714;3715;	3714;14432
1012	1199;1538;	True;True;T	1208;1551;	7350;7351;	3954;5101;	3954;5103;
1515	696;909;24	True;True;T	700;916;24	4216;4217;	2263;2264;	2263;2951;
1518	638;4070	True;True	642;4096	3860;3861;	2088;13337	2088;13337
1743	4979;5116;	True;True;T	5016;5153;	30751;3075	16435;1695	16435;1695
476	5458;7977;	True;True;T	5497;8040;	33810;3381	18082;1808	18083;2606
115	9046;10159	True;True	9114;10239	55723;5572	29459;2946	29461;3338
558	665;2781;6	True;True;T	669;2801;2	4032;4033;	2167;9139;	2167;9140;
461	422;3024;6	True;True;T	423;3048;6	2570;2571;	1440;1441;	1440;9931;
1139	3531;7900;	True;True;T	3556;7962;	21973;2197	11623;1162	11625;2586
1288	6728;11164	True;True;T	6776;11249	41633;4163	22101;2210	22103;3684
642	6398;10881	True;True	6444;10960	39612;3961	21020;2102	21023;3593
221	6985;7443	True;True	7038;7503	43198;4319	22937;2293	22938;2435
1789	5302;9698;	True;True;T	5340;9773;	32834;3283	17644;3177	17644;3177
1386	1371;1467;	False;True;	1381;1478;	8387;8388;	4532;4533;	4532;4867;
1913	4859;5409;	True;True;T	4892;5447;	29972;3349	16035;1794	16035;1794
2050	2541;10425	True;True	2560;10509	15808;1580	8345;34459	8345;34459
1436	1246;1378;	True;True;T	1255;1388;	7670;7671;	4133;4134;	4133;4548;
1705	4594	TRUE	4624	28277	15077	15077
1765	8949;8973	True;True	9017;9041	55145;5514	29139;2914	29140;2923
139	4285;7962	True;True	4312;8025	26501;2650	14045;1404	14045;2603
722	8970	TRUE	9038	55287;5528	29215	29215
1990	73;2119;36	True;True;T	73;2135;36	424;425;42	224;225;22	228;7025;1
734	2241;3624;	True;True;T	2257;3650;	13925;1392	7392;7393;	7393;11916
1836	6796;7405;	True;True;T	6844;7464;	42044;4204	22349;2235	22354;2422
1782	4630	TRUE	4660	28478;2847	15190;1519	15191
119	5862	TRUE	5905	36321;3632	19385;1938	19385
273	4535	TRUE	4565	27902;2790	14875;1487	14875
54	945	TRUE	952	5740;5741;	3072;3073;	3075
1572	4749;5472;	True;True;T	4779;5512;	29230;2923	15578;1557	15580;1811
114	127;2631	True;True	127;2651	762;763;76	422;8672	422;8672
228	617;1638;2	True;True;T	620;1651;2	3721;3722;	2026;5400;	2026;5400;
29	479;487;59	True;True;T	480;488;60	2882;2883;	1596;1597;	1601;1626;
979	40;2503;57	True;True;T	40;2522;57	213;214;21	106;107;10	107;8227;1
980	1704;10989	True;True	1717;11074	10482;1048	5608;36288	5608;36289
489	3869;7134	True;True	3895;7193	24009;2401	12702;1270	12703;2335
885	1272;5209;	True;True;T	1281;5246;	7798;7799;	4214;4215;	4217;17285
1372	6509	TRUE	6555	40331;4033	21381;2138	21382
750	1350;11140	True;True	1360;11225	8270;8271;	4481;4482;	4482;36764
799	6362;6363;	True;True;T	6408;6409;	39415;3941	20891;2089	20891;2089
524	3417;3581;	True;True;T	3442;3607;	21181;2118	11184;1118	11185;1178
481	8095	TRUE	8158	49949;4995	26426;2642	26427
94	5655	TRUE	5696	35086;3508	18705;1870	18707
888	22;2234;39	True;True;T	22;2250;39	127;128;13	56;57;7374	57;7374;12
552	3826;9237;	True;True;T	3852;9307;	23726;2372	12585;1258	12585;3008
646	1081;9443;	True;True;T	1088;9514;	6632;6633;	3573;30833	3573;30833
1602	130;395;25	True;True;T	130;396;25	782;2389;2	429;1329;8	429;1329;8
191	4108	TRUE	4135	25468;2540	13458	13458
1342	1136;1314;	True;True;T	1143;1324;	6946;6947;	3730;4356;	3730;4357;

1341	2197;6080;	True;True;T	2213;6123;	13640;1364	7248;20037	7248;20037
1208	46;1784;18	True;True;T	46;1798;18	260;261;26	137;5848;5	137;5848;5
1284	2659;4595;	True;True;T	2679;4625;	16554;1655	8747;8748;	8749;15078
711	1911;3108;	True;True;T	1926;3132;	11797;1179	6254;10191	6254;10191
2160	371;659;92	True;True;T	372;663;93	2231;2232;	1236;2152;	1236;2154;
1113	924;1090;1	True;True;T	931;1097;1	5607;5608;	2995;2996;	2998;3594;
1724	9470	TRUE	9541	58431;5843	30939	30939
1872	2542;2543;	True;True;T	2561;2562;	15814;1581	8346;8347;	8346;8350;
1886	3860	TRUE	3886	23952;2395	12677;1267	12677
302	7195	TRUE	7254	44438;4443	23553	23553
584	685	TRUE	689	4144;4145;	2228	2228
2164	5640;7720;	True;True;T	5681;7782;	34988;3498	18657;2526	18657;2526
844	1015;1330;	True;True;T	1022;1340;	6234;6235;	3340;4415;	3340;4415;
2102	485;1588;5	True;True;T	486;1601;5	2921;2922;	1615;1616;	1619;5256;
1531	358;359;68	True;True;T	359;360;69	2158;2159;	1203;1204;	1203;1204;
1635	1694	TRUE	1707	10402	5576	5576
436	776;5764;9	True;True;T	783;5806;9	4736;4737;	2545;2546;	2546;19065
1932	6218;9893;	True;True;T	6261;9970;	38569;3857	20487;2048	20488;3248
1898	6614	TRUE	6660	40943;4094	21705	21705
2058	275;2466;7	True;True;T	276;2484;7	1677;1678;	935;936;93	935;8110;2
67	2379;6438	True;True	2396;6484	14812;1481	7857;21142	7857;21143
370	1024;7147	True;True	1031;7206	6289;44140	3373;23399	3373;23399
435	3139;8396	True;True	3163;8459	19507;1950	10278;2733	10278;2733
825	1669;3109;	True;True;T	1682;3133;	10243;1024	5496;5497;	5498;10195
396	1669;9520	False;True	1682;9592	10243;1024	5496;5497;	5498;31063
830	98;463;533	True;True;T	98;464;535	577;578;57	301;302;30	304;1545;1
103	8354	TRUE	8417	51462;5146	27184;2718	27184
1938	7648;7716	True;True	7710;7778	47244;4724	25038;2525	25038;2525
2101	4417;7151;	True;True;T	4446;7210;	27212;2721	14481;1448	14482;2340
1858	8866	TRUE	8933	54609;5461	28889	28889
1710	7053	TRUE	7108	43575;4357	23112	23112
1477	1379;2378;	True;True;T	1389;2395;	8431;8432;	4549;4550;	4549;7854;
1020	4079;5998;	True;True;T	4106;6041;	25285;2528	13368;1336	13369;1979
1268	1925;9378	True;True	1940;9449	11906;1190	6305;6306;	6307;30544
862	6920	TRUE	6970	42805;4280	22734;2273	22737
1371	1693;3413	True;True	1706;3438	10396;1039	5575;11175	5575;11175
1070	2634;3360;	True;True;T	2654;3385;	16409;1641	8681;8682;	8684;10999
1995	6564;7978;	True;True;T	6610;8041;	40680;4068	21557;2607	21557;2607
553	10752	TRUE	10837	66758;6675	35528;3552	35531
685	2830	TRUE	2851	17635;1763	9293	9293
1773	672;2366;5	True;True;T	676;2383;6	4071;4072;	2181;7813;	2181;7813;
1	5032;5516;	True;False;	5069;5556;	31111;3111	16654;1826	16654;1826
1498	3384;4825;	True;True;T	3409;4858;	20980;2098	11081;1590	11081;1590
907	8889	TRUE	8956	54753;5475	28960;2896	28962
915	7037;9101;	True;True;T	7092;9169;	43470;4347	23069;2307	23070;2963
906	6745	TRUE	6793	41742;4174	22156	22156
1094	1339;3595;	True;True;T	1349;3621;	8206;22339	4445;11820	4445;11823
2085	9934;9985	True;True	10011;1006	61430;6143	32598;3259	32600;3274
1981	10022	TRUE	10100	61978;6197	32882;3288	32883
2061	6617;9865;	True;True;T	6663;9942;	40962;4096	21718;2171	21718;3237

	583	4147	TRUE	4174	25706;257	13614;136	13616
	1740	2399	TRUE	2416	14919;149	7900	7900
	891	2680	TRUE	2700	16678;166	8798;8799;	8800
	620	3184;8248	True;True	3208;8311	19785;197	10405;104	10405;269
	1184	207;1068;1	True;True;	207;1075;1	1243;1244;	684;685;68	689;3513;3
	1106	970;1037;2	True;True;	977;1044;2	5904;5905;	3153;3154;	3154;3400;
	958	2681;5776;	True;True;	2701;5818;	16683;166	8801;8802;	8801;1909
	1044	1470;1131	True;True	1481;1139	9015;9016;	4871;4872;	4871;3729
	2079	333	TRUE	334	2007;2008;	1108;1109;	1109
	1280	2421;2772;	True;True;	2438;2792;	15044;172	7956;9114;	7956;9116;
+	293	9595;9599	True;True	9668;9672	59196;591	31372;313	31372;313
	2119	3156;7817;	True;True;	3180;7879;	19619;196	10326;103	10326;255
	1750	2615	TRUE	2635	16274;162	8617;8618	8617
	284	7742	TRUE	7804	47819;478	25340;253	25344
	896	4892;9435;	True;True;	4925;9506;	30162;301	16118;161	16119;308
	1024	967;1607;8	True;True;	974;1620;8	5876;5877;	3133;3134;	3135;5312;
	609	3735;6275	True;True	3761;6320	23186;231	12289;122	12291;206
	997	2209;2210;	True;True;	2225;2226;	13719;137	7278;7279;	7279;7286;
	1040	255;3635;7	True;True;	255;3661;7	1539;1540;	864;11955;	864;11956;
	985	411;7684;7	True;True;	412;7746;7	2496;2497;	1391;1392;	1394;2515
	376	3679;5861	True;True	3705;5904	22832;228	12105;121	12107;193
	751	3832;5540	True;True	3858;5580	23760;237	12596;183	12596;183
	366	7404;8735	True;True	7463;8802	45715;457	24221;242	24223;284
	738	3406;5562	True;True	3431;5602	21112;211	11152;111	11152;183
	968	2469;5834	True;True	2487;5877	15340;153	8113;1928	8113;1929
	1343	1146;3480;	True;True;	1153;3505;	7001;7002;	3754;3755;	3758;1144
	1611	108;196;73	True;True;	108;196;73	639;640;64	345;346;34	346;647;23
	1582	158;2158;2	True;True;	158;2174;2	945;946;13	532;533;53	533;7128;7
	664	3666;9611;	True;True;	3692;9684;	22765;227	12063;314	12063;314
	1753	5987;9788;	True;True;	6030;9865;	37099;605	19766;321	19766;321
	1532	965;1530;1	True;True;	972;1542;1	5861;5862;	3123;3124;	3125;5071;
	1149	3749;4138;	True;True;	3775;4165;	23284;256	12341;135	12341;135
	729	8693;9802	True;True	8760;9879	53586;535	28305;321	28305;321
	1900	1985;2337;	True;True;	2001;2353;	12328;123	6534;7693;	6534;7694;
	1305	4315;4709	True;True	4342;4739	26635;266	14126;154	14126;154
	169	453;660;17	True;True;	454;664;17	2747;2748;	1524;1525;	1524;2157;
	690	4653;5874;	True;True;	4683;5917;	28618;286	15259;152	15259;194
	1701	6911;8923	True;True	6961;8990	42755;427	22714;290	22714;290
	1997	3511;9446	True;True	3536;9517	21836;218	11547;308	11547;308
	11	5618;8796	True;True	5658;8863	34837;348	18590;185	18592;286
	1161	3051;5638;	True;True;	3075;5679;	18994;189	10005;186	10005;186
	81	99;10213	True;True	99;10293	585;586;58	309;310;33	309;33592
	2128	1097;2457;	True;True;	1104;2475;	6731;6732;	3620;8079;	3620;8080;
	1652	2352;3786;	True;True;	2368;3812;	14619;146	7748;7749;	7755;1244
	1563	6653;9296;	True;True;	6700;9366;	41175;411	21847;302	21847;302
	1156	9209;1019	True;True	9279;1027	56763;567	30019;335	30019;335
	1353	2162;2163;	True;True;	2178;2179;	13409;134	7140;7141;	7141;7143;
	793	11219	TRUE	11304	69689;696	37014	37014
	655	7983;8001;	True;True;	8046;8064;	49278;492	26088;261	26088;261
	464	4664	TRUE	4694	28692	15301	15301

117	3907;4030;	True;True;	3933;4056;	24226;242	12814;131	12814;131
241	7279	TRUE	7338	44967;449	23845;238	23846
645	508	TRUE	509	3078;3079;	1686	1686
1509	4678	TRUE	4708	28782;287	15349	15349
1617	1696;4962;	True;True;	1709;4999;	10408;104	5579;5580;	5580;1637
1604	2740;3987;	True;True;	2760;4013;	17054;170	9004;9005;	9004;1303
1193	1435;1908;	True;True;	1446;1923;	8790;8791;	4761;4762;	4762;6247;
1053	344;534;73	True;True;	345;536;74	2063;2064;	1140;1141;	1140;1747;
1387	926;1082;1	True;True;	933;1089;1	5621;5622;	3005;3006;	3010;3574;
2116	4676;4743	True;True	4706;4773	28770;287	15347;155	15347;155
983	177;2131;7	True;True;	177;2147;7	1066;1067;	595;596;5	597;7058;2
994	2003;4126;	True;True;	2019;4153;	12431;124	6602;6603;	6602;1353
1222	550;887;38	True;True;	552;894;38	3299;3300;	1792;2894;	1792;2894;
528	10683	TRUE	10768	66332;663	35312	35312
754	498;3253	True;True	499;3278	3014;3015;	1665;1666;	1665;1064
172	2171;3663;	True;True;	2187;3689;	13493;134	7182;7183;	7185;1205
1526	2057	TRUE	2073	12762;127	6798;6799	6798
1672	2146;3205;	True;True;	2162;3230;	13325;133	7099;1045	7099;1045
1739	10334	TRUE	10416	64098;640	34133	34133
201	1940;2984;	True;True;	1956;3008;	12015;120	6378;6379;	6379;9781;
207	1344;4961;	True;True;	1354;4998;	8234;8235;	4456;4457;	4456;1637
437	6697;7809;	True;True;	6744;7871;	41439;414	21995;255	21995;255
1332	3244;5574;	True;True;	3269;5614;	20140;201	10589;184	10589;184
381	9922	TRUE	9999	61366;613	32565	32565
573	1355;1966;	True;True;	1365;1982;	8300;8301;	4495;4496;	4497;6461;
1121	459;1343;4	True;True;	460;1353;4	2778;2779;	1540;4453;	1540;4453;
1196	278;560;17	True;True;	279;562;17	1696;1697;	944;945;94	946;1857;5
1615	1536;3500;	True;True;	1549;3525;	9455;9456;	5099;1151	5099;1151
1197	1338;3191;	True;True;	1348;3215;	8203;8204;	4442;4443;	4444;1043
1008	263;432;12	True;True;	263;433;12	1590;1591;	888;889;8	888;1464;3
1009	1676;3159;	True;True;	1689;3183;	10282;102	5510;5511;	5510;1033
542	2393;3785;	True;True;	2410;3811;	14889;148	7884;7885;	7885;1243
1482	75;766;101	True;True;	75;773;102	438;439;44	237;238;23	239;2511;3
877	5462;8888	True;True	5501;8955	33832;338	18091;289	18091;289
783	2964;3698	True;True	2988;3724	18476;184	9708;1215	9708;1215
1120	10069	TRUE	10147	62295	33046	33046
699	6556	TRUE	6602	40634;406	21527;215	21527
277	5185;1010	True;True	5222;1018	32075;320	17188;331	17188;331
772	1237;4858	True;True	1246;4891	7614;7615;	4110;1603	4110;1603
30	4208	TRUE	4235	26095;260	13807	13807
1183	1368;4723;	True;True;	1378;4753;	8372;8373;	4528;1549	4528;1550
822	6838;7642;	True;True;	6886;7704;	42309;423	22510;250	22510;250
1887	3673;4039;	True;True;	3699;4065;	22800;228	12080;120	12083;132
1123	1582;2056;	True;True;	1595;2072;	9720;9721;	5235;6797;	5235;6797;
2077	1566;3261;	True;True;	1579;3286;	9612;9613;	5182;5183;	5182;1067
658	7626	TRUE	7688	47112;471	24978	24978
2041	420;2746;6	True;True;	421;2766;6	2561;1709	1423;9022;	1423;9022;
575	6144	TRUE	6187	38116;381	20230	20230
1574	2979;6032;	True;True;	3003;6075;	18561;185	9767;1989	9767;1989
1989	2150	TRUE	2166	13348;133	7106;7107;	7107

1918	2121;2867;	True;True;T	2137;2890;	13175;1317	7028;7029;	7030;9438;
1841	5072	TRUE	5109	31367	16801	16801
562	2360;8061;	True;True;T	2377;8124;	14690;1469	7795;7796;	7796;26317
2071	2577;10222	True;True	2596;10302	16023;1602	8459;8460;	8461;33625
118	2527;3588;	True;True;T	2546;3614;	15714;1571	8292;8293;	8294;11798
923	677;751;10	True;True;T	681;758;10	4098;4099;	2194;2195;	2195;2445;
924	2275;3027;	True;True;T	2291;3051;	14106;1410	7488;7489;	7488;9944;
478	173;3161;5	True;True;T	173;3185;5	1038;1039;	577;578;57	581;10349;
1273	2630;3096;	True;True;T	2650;3120;	16380;1638	8666;8667;	8666;10155
1817	449;3067	True;True	450;3091	2729;2730;	1514;10058	1514;10059
2158	2811	TRUE	2832	17525;1752	9231;9232;	9231
52	2553;6219	True;True	2572;6262	15883;1588	8388;20489	8388;20494
580	1579;5956;	True;True;T	1592;5999;	9692;9693;	5224;19694	5224;19694
195	6976	TRUE	7029	43154;4315	22916	22916
222	259;3262;1	True;True;T	259;3287;1	1567;1568;	881;10677;	881;10679;
1510	5381	TRUE	5419	33330;3333	17875	17875
1214	8295	TRUE	8358	51176	27044	27044
1269	3312;4227;	True;True;T	3337;4254;	20542;2054	10837;1083	10837;1386
417	2595;2832	True;True	2614;2853	16137;1613	8534;9301	8534;9301
555	6868;8406;	True;True;T	6916;8470;	42507;4250	22585;2730	22585;2736
380	1300;4554;	True;True;T	1309;4584;	7958;7959;	4292;4293;	4293;14936
8	5555	TRUE	5595	34450;3445	18368	18368
1463	7150;7270;	True;False;	7209;7329;	44164;4416	23407;2340	23408;2380
1889	7149;7270;	True;True;T	7208;7329;	44156;4415	23401;2340	23403;2380
2020	320;1627;4	True;True;T	321;1640;4	1938;9965;	1074;5364;	1074;5366;
408	9297	TRUE	9367	57295	30260	30260
1603	4153;6661	True;True	4180;6708	25744;2574	13634;1363	13636;2187
1303	576;767;13	True;True;T	578;774;13	3474;4678;	1906;2512;	1906;2512;
1613	632;892;26	True;True;T	636;899;26	3822;3823;	2074;2075;	2074;2906;
616	6293	TRUE	6339	39026;3902	20705;2070	20705
1763	3840;4035;	True;True;T	3866;4061;	23824;2382	12619;1320	12619;1320
519	2310;3057;	True;True;T	2326;3081;	14312;1431	7612;7613;	7613;10020
1565	2	TRUE	2	13;14;15;1	11;12	11
1971	5826;7681	True;True	5869;7743	36121;3612	19263;1926	19263;2515
15	2179;4608;	True;True;T	2195;4638;	13549;1355	7200;15110	7200;15110
1876	12;206;155	True;True;T	12;206;156	77;78;79;8	31;32;33;6	33;683;515
803	3891;4234	True;True	3917;4261	24131;2413	12771;1388	12771;1388
5	213;6127	True;True	213;6170	1293;1294;	710;20185	710;20185
1983	393;3083;5	True;True;T	394;3107;5	2378;2379;	1319;1320;	1322;10114
1663	1419;1979;	True;True;T	1430;1995;	8705;8706;	4720;4721;	4727;6515;
592	3818;4097;	True;True;T	3844;4124;	23675;2539	12566;1341	12566;1342
1226	2415;2868;	True;True;T	2432;2891;	15005;1500	7940;7941;	7943;9440;
1525	2062;2395;	False;False	2078;2412;	12789;1279	6806;6807;	6806;7889;
1116	6781;8770;	True;True;T	6829;8837;	41943;4194	22285;2228	22285;2854
444	976;1014;3	True;True;T	983;1021;3	5957;5958;	3184;3185;	3186;3339;
1153	976;1014;3	False;False	983;1021;3	5957;5958;	3184;3185;	3186;3339;
1132	20;56;623;	True;True;T	20;56;627;	112;113;11	49;50;51;5	50;162;204
1027	412;882;11	True;True;T	413;889;11	2503;2504;	1396;2872;	1396;2874;
267	3754;5750;	True;True;T	3780;5792;	23296;2329	12350;1235	12350;1901
1260	3610;5969;	True;True;T	3636;6012;	22417;2241	11863;1186	11865;1971

1293	238;1709;2	True;True;	238;1722;2	1439;1440;	796;797;79	797;5617;7
1958	1210	TRUE	1219	7416;7417;	3990;3991;	3992
97	6936	TRUE	6987	42914;4291	22790;227	22790
488	10525	TRUE	10609	65290;652	34786	34786
1310	761;4209;4	True;True;	768;4236;4	4636;4637;	2485;2486;	2487;1380
864	4836;6200;	True;True;	4869;6243;	29838;298	15937;159	15940;204
714	8088	TRUE	8151	49913;499	26408;264	26408
2057	1788;4807;	True;True;	1802;4840;	11011;110	5856;5857;	5857;1581
406	378;829;34	True;True;	379;836;34	2283;2284;	1260;1261;	1262;2706;
1157	1543;2793;	True;True;	1556;2814;	9489;9490;	5114;5115;	5114;9183;
1943	1686;2221;	True;True;	1699;2237;	10352;103	5548;5549;	5550;7325;
567	7246;9807;	True;True;	7305;9884;	44763;447	23711;237	23712;321
1039	818;1658;2	True;True;	825;1671;2	4983;4984;	2675;2676;	2676;5456;
821	525;7832	True;True	527;7894	3177;3178;	1733;2564	1733;2564
1492	222;8731;1	True;True;	222;8798;1	1348;1349;	740;28418;	740;28424;
1248	1388;1946;	True;True;	1398;1962;	8497;8498;	4587;4588;	4587;6398;
112	5999;7751;	True;True;	6042;7813;	37166;371	19795;253	19795;253
1165	327;831;23	True;True;	328;838;23	1972;1973;	1091;1092;	1094;2715;
1495	3678	TRUE	3704	22827;228	12102;121	12104
901	136;137;10	True;True;	136;137;10	814;815;81	445;446;44	450;460;33
394	1126;1445;	True;True;	1133;1456;	6895;6896;	3707;3708;	3708;4805;
245	551;3567;5	True;True;	553;3592;5	3306;3307;	1793;1794;	1793;1173
578	4467;6938	True;True	4496;6989	27495;274	14641;146	14641;227
947	1980;2639;	True;True;	1996;2659;	12301;123	6516;6517;	6517;8695;
1926	5763	TRUE	5805	35748;357	19057;190	19060
723	1036;8425	True;True	1043;8489	6336;6337;	3397;3398;	3398;2742
1798	3235;7988;	True;True;	3260;8051;	20091;200	10554;260	10554;260
1633	4873;1128	True;True;	4906;1137	30039;300	16065;160	16068;372
2063	6689	TRUE	6736	41395;413	21971;219	21972
791	5039;6705	True;True	5076;6753	31157;311	16680;220	16680;220
838	160;2120;5	True;True;	160;2136;5	950;951;95	536;537;70	536;7026;1
387	1647;5049;	True;True;	1660;5086;	10093;100	5426;5427;	5429;1671
1892	1232;1438;	True;True;	1241;1449;	7571;7572;	4090;4091;	4090;4780;
65	5129;7428	True;True	5166;7488	31733;317	16999;170	17000;243
1993	1592	TRUE	1605	9777;9778;	5273;5274;	5274
1844	5542	TRUE	5582	34375;343	18335;183	18336
1382	1017;1531;	True;True;	1024;1543;	6240;6241;	3343;3344;	3343;5076;
1230	1486;1988;	True;True;	1498;2004;	9136;9137;	4948;6539;	4948;6543;
1047	2644;4352;	True;True;	2664;4379;	16472;164	8707;8708;	8708;1423
255	3601;6609;	True;True;	3627;6655;	22374;223	11836;216	11836;216
275	3;541	True;True	3;543	20;21;22;2	13;1764	13;1764
696	2932;3491	True;True	2956;3516	18278;182	9625;9626;	9625;1148
1276	1148;2195;	True;True;	1155;2211;	7012;7013;	3766;3767;	3767;7246;
930	2627;4926;	True;True;	2647;4963;	16361;163	8657;1625	8657;1625
1086	1427;2053;	True;True;	1438;2069;	8746;8747;	4740;4741;	4740;6787;
1242	397;4306;4	True;True;	398;4333;4	2400;2401;	1334;1335;	1341;1410
269	790;6863	True;True	797;6911	4820;4821;	2580;2257	2580;2257
1608	728;2183;2	True;True;	735;2199;2	4438;4439;	2378;7209;	2378;7214;
1364	815;3296;3	True;True;	822;3321;3	4968;4969;	2669;2670;	2670;1078
1917	1504;2544;	True;True;	1516;2563;	9251;9252;	4994;8354;	4994;8355;

780	3461;4642;	True;True;	3486;4672;	21504;215	11377;152	11377;152
1377	5931;6086;	True;True;	5974;6129;	36761;367	19615;196	19618;200
735	3180;1056	True;True;	3204;1065	19769;197	10396;103	10397;349
693	2203;2463;	True;True;	2219;2481;	13678;153	7259;8100;	7259;8100;
47	2302;2381;	True;True;	2318;2398;	14265;142	7586;7587;	7586;7860;
1581	1849;2318;	True;True;	1863;2334;	11406;114	6079;7645;	6079;7647;
585	1453;5156	True;True	1464;5193	8898;8899;	4818;4819;	4818;1709
707	2629	TRUE	2649	16373;163	8664;8665	8665
200	3505;5545;	True;True;	3530;5585;	21785;217	11519;183	11519;183
469	6250	TRUE	6293	38766;387	20572;205	20572
1519	77;530;166	True;True;	77;532;167	449;450;45	242;1741;5	242;1741;5
360	1723;8882	True;True	1736;8949	10580;105	5648;2894	5648;2894
416	2186;9095;	True;True;	2202;9163;	13585;135	7219;7220;	7221;2961
1640	1168;2125;	True;True;	1177;2141;	7154;7155;	3836;7036;	3836;7036;
1354	2248;6422	True;True	2264;6468	13975;139	7417;2110	7417;2110
647	2204;2205;	True;True;	2220;2221;	13679;136	7260;7261;	7262;7267;
26	4406;4870;	True;True;	4435;4903;	27133;271	14426;160	14426;160
1370	906;4283;6	True;True;	913;4310;6	5502;5503;	2947;2948;	2948;1404
216	5647	TRUE	5688	35030;350	18676;186	18679
1708	2477;3650;	True;True;	2495;3676;	15389;153	8137;1200	8137;1201
1600	5968	TRUE	6011	36983;369	19716	19716
1636	6607;1064	True;True	6653;1073	40907;409	21680;351	21680;351
471	5514;9102	True;True	5554;9170	34204;342	18254;296	18254;296
766	1503;1938;	True;True;	1515;1954;	9243;9244;	4991;4992;	4991;6376;
1356	30;31;673;	True;True;	30;31;677;	163;164;16	75;76;2182	75;76;2184
2007	1124;8431	True;True	1131;8495	6883;6884;	3699;2743	3699;2743
1945	671;1133;1	True;True;	675;1140;1	4065;4066;	2180;3722;	2180;3722;
404	656;752;82	True;False;	660;759;83	3976;4578;	2146;2446;	2146;2450;
2148	103;762;11	True;True;	103;769;11	606;607;60	325;326;32	325;2493;3
1541	226;317;95	True;True;	226;318;95	1371;1372;	748;749;75	750;1063;3
1335	2005;2143;	True;True;	2021;2159;	12440;124	6605;6606;	6605;7093;
1274	200;2149;2	True;True;	200;2165;2	1194;1195;	658;7103;7	658;7104;7
1275	2042;5386;	True;True;	2058;5424;	12663;126	6736;6737;	6737;1788
1874	4485;4677;	True;True;	4515;4707;	27621;276	14727;147	14727;153
13	524;3647;3	True;True;	526;3673;4	3171;3172;	1732;1199	1732;1200
1894	4556;4995;	True;True;	4586;5032;	28031;280	14938;149	14939;165
1035	1016;7869;	True;True;	1023;7931;	6236;6237;	3341;3342;	3342;2577
1878	482;3267;5	True;True;	483;3292;5	2902;2903;	1605;1606;	1607;1069
1677	2883;3553;	True;True;	2906;3578;	17985;179	9474;1168	9474;1168
140	143;165;42	True;True;	143;165;42	874;875;87	491;492;54	491;550;14
1504	1033;6814;	True;True;	1040;6862;	6323;6324;	3393;2242	3393;2242
462	565;4728;7	True;True;	567;4758;7	3414;3415;	1872;1873;	1873;1550
677	2456;2989;	True;True;	2474;3013;	15263;152	8078;9796;	8078;9796;
534	3471;4523;	True;True;	3496;4553;	21563;215	11405;148	11405;148
911	2520;4646;	True;True;	2539;4676;	15668;156	8265;8266;	8266;1523
1693	6885	TRUE	6933	42582	22615	22615
1095	241;267;27	True;True;	241;267;27	1456;1457;	810;811;81	815;903;91
831	188;279;28	True;True;	188;280;28	1126;1127;	625;626;94	625;947;95
1830	619;1578	True;True	622;1591	3735;3736;	2033;5222;	2033;5222
1233	2110;6015;	True;True;	2126;6058;	13091;130	6977;6978;	6982;1983

1159	1290;6385;	True;True;T	1299;6431;	7894;7895;	4263;4264;	4263;20982
854	866;5076;5	True;True;T	873;5113;5	5274;5275;	2828;2829;	2830;16813
393	2840;6103;	True;True;T	2861;6146;	17688;1768	9320;20100	9320;20106
85	1180;2172	True;True	1189;2188	7238;7239;	3877;3878;	3877;7186
239	726;2202;2	True;True;T	733;2218;2	4425;4426;	2376;7258;	2376;7258;
287	5917;8002	True;True	5960;8065	36674;3667	19575;1957	19575;2613
809	6500	TRUE	6546	40277;4027	21359	21359
914	92;93;569;(	True;True;T	92;93;571;(	543;544;54	282;283;28	283;284;18
989	1328;1537;	False;False	1338;1550;	8146;8147;	4409;4410;	4411;5100;
960	8;32;502;4(	True;True;T	8;32;503;4(	52;53;54;5	22;77;78;7	22;77;1678
1626	8682	TRUE	8749	53526;5352	28275;2827	28275
457	373;4559;6	True;True;T	374;4589;6	2241;2242;	1238;14952	1238;14952
976	140;1555;1	True;True;T	140;1568;1	850;851;85	476;477;47	486;5159;6
1072	5024;8155	True;True	5061;8218	31066;310(	16635;1663	16636;2664
1539	1062;2585;	True;True;T	1069;2604;	6512;6513;	3492;8503;	3492;8503;
1379	10088;108(	True;True	10166;109	62414;6241	33109;3588	33109;3588
826	503;7509;1	True;True;T	504;7570;1	3042;3043;	1680;2457	1680;2457
1678	2847;3540;	True;True;T	2868;3565;	17750;1775	9354;9355;	9358;11656
1991	1587;10593	True;True	1600;10678	9750;9751;	5253;5254;	5253;35015
352	370;859;18	True;True;T	371;866;18	2226;2227;	1235;2804;	1235;2805;
1553	6504	TRUE	6550	40303;4030	21366	21366
1478	729;793;29	True;True;T	736;800;29	4442;4443;	2379;2586;	2379;2589;
818	2682;9167	True;True	2702;9236	16689;1669	8806;8807;	8806;29850
544	6817	TRUE	6865	42179;4218	22432	22432
2060	8453;9947	True;True	8517;10024	52095;6150	27498;3263	27498;3263
545	10342	TRUE	10425	64156;6415	34168;3416	34170
365	3039;4774;	True;True;T	3063;4806;	18929;1893	9976;15682	9976;15682
1010	1563;1800;	True;True;T	1576;1814;	9594;9595;	5173;5896;	5173;5896;
99	1994;4265;	True;True;T	2010;4292;	12380;1238	6570;13991	6570;13992
694	863;1231;1	True;True;T	870;1240;1	5251;5252;	2814;2815;	2814;4089;
893	552;572;11	True;True;T	554;574;12	3314;3315;	1800;1801;	1803;1897;
379	11077;1119	True;True	11162;1127	68763;6951	36570;3692	36570;3692
1286	5851	TRUE	5894	36249;3625	19341;1934	19342
1935	871;4006;1	True;True;T	878;4032;1	5300;24832	2847;13101	2847;13101
865	1369;1570;	True;True;T	1379;1583;	8374;8375;	4529;4530;	4529;5198;
1527	1322;2422;	True;True;T	1332;2439;	8104;8105;	4383;4384;	4383;7959;
1278	1001;2074;	True;True;T	1008;2090;	6137;6138;	3284;6841;	3284;6841;
856	2496;5722	True;True	2514;5764	15512;1551	8205;18927	8205;18927
1768	2027	TRUE	2043	12564;125(	6676;6677;	6676
895	637;3685;5	True;True;T	641;3711;5	3854;3855;	2085;2086;	2085;12132
431	430;2828;7	True;True;T	431;2849;7	2613;17627	1460;9290;	1460;9290;
1480	4014;4255;	True;True;T	4040;4282;	24873;2487	13142;1314	13143;1396
975	648;895;18	True;True;T	652;902;18	3918;3919;	2117;2118;	2122;2915;
921	3447;4780;	True;True;T	3472;4812;	21398;2139	11307;1130	11316;1570
1696	2244	TRUE	2260	13946;1394	7399;7400;	7399
1256	1366;1452;	True;True;T	1376;1463;	8357;8358;	4519;4520;	4522;4817;
167	7104;8450	True;True	7162;8514	43892;4389	23267;2326	23268;2749
1244	3539;9022;	True;True;T	3564;9090;	22024;2202	11650;1165	11651;2939
1304	11032	TRUE	11117	68510;6851	36426;3642	36426
303	6816	TRUE	6864	42175;4217	22429;2243	22430

497	2560;4883;	True;True;T	2579;4916;	15912;1591	8398;1610	8398;1610
2076	4447	TRUE	4476	27361;2736	14573	14573
1200	3475;3621;	True;True;T	3500;3647;	21593;2159	11430;1143	11430;1190
1306	154;2526;4	True;True;T	154;2545;4	924;925;15	522;523;82	522;8291;1
1861	189;642;29	True;True;T	189;646;29	1134;1135;	627;2097;2	627;2097;9
515	5628;5728;	True;True;T	5669;5770;	34914;3491	18627;1862	18628;1893
1992	2394;8458	True;True	2411;8522	14896;1489	7886;2750	7886;2750
2002	795;2824;5	True;True;T	802;2845;5	4843;4844;	2591;9280;	2591;9280;
1988	5012	TRUE	5049	30981;309	16591	16591
233	57	TRUE	57	333;334;33	167;168;16	171
1538	2505;5721;	True;True;T	2524;5763;	15568;1556	8230;1892	8230;1892
1762	5232	TRUE	5269	32379;323	17374;1737	17375
363	7402	TRUE	7461	45707	24218	24218
2157	6370;8329	True;True	6416;8392	39455;3945	20914;2713	20914;2713
492	5153;9569	True;True	5190;9642	31886;318	17086;170	17086;312
1986	325;8769	True;True	326;8836	1964;5401	1089;2853	1089;2853
2008	1730;2666;	True;True;T	1743;2686;	10615;1061	5664;5665;	5666;8763;
2091	10058	TRUE	10136	62223;622	33004;330	33005
908	6822;9328	True;True	6870;9398	42207;422	22450;224	22450;303
248	94;3238;43	True;True;T	94;3263;43	555;556;55	286;287;28	287;10562;
285	564	TRUE	566	3408;3409;	1867;1868;	1868
566	2423	TRUE	2440	15052;150	7960;7961;	7960
1333	3341;3737	True;True	3366;3763	20737;207	10938;122	10938;122
1460	1907;2455;	True;False;	1922;2473;	11779;117	6246;8077;	6246;8077;
925	706;2455;2	True;False;	712;2473;2	4296;4297;	2321;8077;	2321;8077;
2074	885;5730;8	True;True;T	892;5772;8	5375;5376;	2883;2884;	2883;1894
1446	131;5257;5	True;True;T	131;5294;5	783;784;78	430;17469;	430;17471;
1447	138;2563;5	True;True;T	138;2582;5	830;831;83	461;462;46	463;8408;1
973	2455;2920;	True;True;T	2473;2944;	15256;152	8077;9578;	8077;9578;
1307	6185;1016	True;True	6228;1024	38363;383	20374;203	20374;334
1459	688;6184;7	True;False;	692;6227;7	4165;4166;	2238;2036	2238;2036
1155	6185;9784	False;True	6228;9861	38363;383	20374;203	20374;321
1614	777	TRUE	784	4739;4740	2547	2547
1465	305;1498;1	True;True;T	306;1510;1	1850;1851;	1028;1029;	1028;4980;
1956	8667;8994	True;True	8734;9062	53448;534	28227;282	28227;293
557	2880	TRUE	2903	17972;179	9468	9468
2051	1353;1027	True;True	1363;1035	8285;8286;	4486;4487;	4490;3384
2048	211;4791;9	True;True;T	211;4824;9	1278;1279;	706;707;15	706;15755;
820	786;1895;4	True;True;T	793;1910;4	4795;4796;	2570;2571;	2570;6221;
1715	243;1499;2	True;True;T	243;1511;2	1464;1465;	817;4983;7	817;4983;7
1547	2952	TRUE	2976	18407;184	9682;9683	9682
127	14;249;363	True;True;T	14;249;364	89;90;91;1	37;38;830;	38;830;121
1199	314;353;40	True;True;T	315;354;40	1906;1907;	1052;1053;	1053;1184;
1065	880;975;18	False;False	887;982;18	5353;5354;	2866;2867;	2866;3171;
1003	881;1028;1	True;True;T	888;1035;1	5356;5357;	2869;2870;	2869;3379;
841	7308	TRUE	7367	45130;451	23915;239	23916
1850	403;1659;1	True;True;T	404;1672;1	2437;2438;	1355;1356;	1358;5463;
1561	346;850;23	True;True;T	347;857;23	2075;2076;	1147;1148;	1149;2766;
922	848;849;15	True;True;T	855;856;16	5164;5165;	2760;2761;	2762;2764;
957	202;760;12	False;True;	202;767;12	1202;1203;	663;664;66	669;2483;4

	965	181;202;63	True;True;	181;202;63	1089;1090;	610;663;6	610;669;20
	1348	881;1405;3	False;True;	888;1416;3	5356;5357;	2869;2870;	2869;4670;
	666	2201;6891;	True;True;	2217;6939;	13673;136	7257;2263	7257;22635
	179	6890;9935	True;True	6938;1001	42618;426	22634;326	22634;326
	1174	1313;3445;	True;True;	1323;3470;	8054;8055;	4355;1130	4355;1130
	1719	755	TRUE	762	4594;4595;	2460;2461	2460
+	323	1066;6949;	True;True;	1073;7000;	6530;6531;	3501;3502;	3502;2282
	1476	3043;6011;	True;False;	3067;6054;	18949;372	9985;1982	9985;1983
+	316	1320;8452;	True;True;	1330;8516;	8092;8093;	4377;2749	4377;2749
	643	10248	TRUE	10328	63496;634	33739;337	33744
	1325	1244;3951;	True;True;	1253;3977;	7662;7663;	4131;1292	4131;1292
	204	3453;5741;	True;True;	3478;5783;	21444;214	11338;113	11338;1897
	364	2900;4654;	True;True;	2924;4684;	18072;180	9519;9520;	9522;1526
	1568	2199;3690;	True;True;	2215;3716;	13657;136	7251;7252;	7253;1213
	513	2216;2219;	True;False;	2232;2235;	13762;137	7308;7309;	7309;7320;
	1326	2217;2220;	True;True;	2233;2236;	13768;137	7310;7311;	7311;7324;
	576	2737;2910;	True;True;	2757;2934;	17040;170	8994;9549;	8994;9550;
	22	3583;1044	True;True;	3609;1053	22282;222	11791;345	11791;345
	1331	994;1766;4	True;True;	1001;1779;	6085;6086;	3256;3257;	3258;5770;
	423	994;1744;3	False;True;	1001;1757;	6085;6086;	3256;3257;	3258;5719;
	1362	1744;2377;	False;True;	1757;2394;	10698;106	5712;5713;	5719;7853;
	1187	993;1000;1	True;True;	1000;1007;	6077;6078;	3252;3253;	3254;3283;
	1413	2656;3636;	True;True;	2676;3662;	16542;165	8740;8741;	8742;1195
	1041	535;5104;5	True;True;	537;5141;5	3218;3219;	1750;1692	1750;1692
	1328	58;152;192	True;True;	58;152;192	340;341;34	174;175;17	175;513;63
	813	439;1705;1	True;True;	440;1718;1	2669;2670;	1484;5609;	1484;5609;
	789	439;1778;2	False;True;	440;1792;2	2669;2670;	1484;5834;	1484;5835;
	1502	782;1131;1	True;True;	789;1138;1	4772;4773;	2557;3719;	2557;3719;
	74	7192;7689;	True;True;	7251;7751;	44425;444	23550;251	23550;2517
	1685	42;732;121	True;True;	42;739;122	230;231;23	117;118;11	119;2383;4
	1105	2031;2219;	True;True;	2047;2235;	12595;125	6698;6699;	6699;7320;
	218	111;3577;5	True;True;	111;3603;5	652;653;65	354;355;35	354;11778;
	419	518;911;12	True;True;	519;918;12	3132;3133;	1715;2954;	1715;2954;
	1083	519;925;98	True;True;	520;932;99	3136;3137;	1716;3000;	1716;3001;
	1178	3153;9828	True;True	3177;9905	19598;195	10313;103	10317;3225
	977	4354;4796;	True;True;	4381;4829;	26808;268	14234;142	14235;1577
	362	5353;7091	True;False	5391;7148	33146;331	17792;232	17792;2322
	405	9226	TRUE	9296	56877;568	30069	30069
	1066	2675;5440;	True;True;	2695;5479;	16646;166	8779;1802	8779;1803
	485	3541	TRUE	3566	22038;220	11657	11657
	1301	1176;1269;	True;True;	1185;1278;	7211;7212;	3866;3867;	3868;4204;
	220	1864;1129	True;True	1878;1138	11502;115	6126;6127;	6126;3724
	764	6247	TRUE	6290	38752;387	20566;205	20566
	1855	6005;8780;	True;True;	6048;8847;	37202;372	19807;198	19809;2856
	951	4128;7099;	True;True;	4155;7157;	25600;256	13547;135	13550;2325
	1060	555;921;40	True;True;	557;928;41	3342;3343;	1823;1824;	1823;2990;
	993	555;921;40	False;False	557;928;41	3342;3343;	1823;1824;	1823;2990;
	1059	556;1002;4	True;True;	558;1009;4	3350;3351;	1830;1831;	1832;3295;
	1846	687;11588	True;True	691;11674	4157;4158;	2235;2236;	2237;3821
	144	421;4331;7	True;True;	422;4358;7	2562;2563;	1424;1425;	1439;1416

990	421;1005;3	False;True;	422;1012;3	2562;2563;	1424;1425;	1439;3311;
1709	463;1897	False;True	464;1912	2801;2802;	1544;1545;	1545;6227
1055	421;4331;7	False;False	422;4358;7	2562;2563;	1424;1425;	1439;1416
1929	705;2441;2	False;False	709;710;71	4273;4274;	2294;2295;	2317;8013;
946	705;2441;2	False;False	709;710;71	4273;4274;	2294;2295;	2317;8013;
1791	705;2794;2	False;False	709;710;71	4273;4274;	2294;2295;	2317;9184;
819	705;2441;2	True;True;	709;710;71	4273;4274;	2294;2295;	2317;8013;
1718	2411;7750;	True;True;	2428;7812;	14979;149	7925;2536	7925;2537
1440	1437;1802;	True;True;	1448;1816;	8799;8800;	4767;4768;	4772;5900;
1544	193;2066;3	True;True;	193;2082;3	1152;1153;	632;633;63	636;6818;1
1675	4353;6334;	True;False;	4380;6380;	26805;268	14232;142	14233;208
1058	806;1160;1	True;False;	813;1169;1	4908;4909;	2624;2625;	2625;3804;
1171	805;1160;1	True;True;	812;1169;1	4900;4901;	2616;2617;	2616;3804;
131	2039;1116	True;False	2055;1124	12648;126	6724;6725;	6724;3683
1172	805;1160;1	False;False	812;1169;1	4900;4901;	2616;2617;	2616;3804;
1173	807;1160;1	True;False;	814;1169;1	4914;4915;	2630;2631;	2636;3804;
1867	807;1159;1	False;True;	814;1168;1	4914;4915;	2630;2631;	2636;3802;
1313	497;501;29	True;True;	498;502;29	3006;3007;	1660;1661;	1663;1676;
1673	2010;2324;	True;True;	2026;2340;	12471;124	6625;6626;	6626;7663;
615	8960	TRUE	9028	55212;552	29174;291	29178
125	374;1954;3	True;True;	375;1970;3	2249;2250;	1239;1240;	1239;6425;
1212	4216;7343	True;True	4243;7402	26137;261	13836;240	13836;240
1506	951;6546;7	True;True;	958;6592;7	5786;5787;	3096;3097;	3097;2149
1744	1540;5541;	True;True;	1553;5581;	9472;9473;	5106;5107;	5108;1833
899	8768;9278;	True;True;	8835;9348;	54007;540	28536;302	28536;302
50	240;2103;3	True;True;	240;2119;3	1448;1449;	805;806;80	805;6962;9
158	7329	TRUE	7388	45273;452	23990;239	23991
1329	2156;3734;	True;True;	2172;3760;	13385;133	7123;7124;	7124;1228
746	1079;2075;	True;True;	1086;2091;	6623;6624;	3566;3567;	3566;6843;
740	297;2075	True;False	298;2091	1800;1801;	992;993;99	997;6843
1330	2077;9008	True;True	2093;9076	12861;128	6845;6846;	6845;2934
1630	2748;2785;	True;True;	2768;2806;	17104;171	9029;9030;	9031;9153;
572	3168;5643;	True;True;	3192;5684;	19694;350	10365;186	10365;186
872	2004;7513;	True;True;	2020;7574;	12439;463	6604;2458	6604;2458
1905	4329;9938	True;True	4356;1001	26688;266	14160;326	14160;326
1580	1494;1108	True;True	1506;1116	9184;9185;	4966;3657	4966;3657
1746	5813;7326	False;True	5856;7385	36044;360	19220;239	19220;239
1747	5813;9717	True;True	5856;9792	36044;360	19220;318	19220;318
2024	1915;1062	True;True	1930;1070	11833;118	6280;3512	6280;3512
2121	1541;9769	True;True	1554;9846	9479;9480;	5112;3204	5112;3204
1646	86;1839;37	True;True;	86;1853;37	501;502;50	273;274;60	273;6052;1
1963	4543;6093	True;True	4573;6136	27958;279	14902;200	14902;200
564	710;1683;2	True;True;	716;1696;2	4335;4336;	2335;2336;	2335;5542;
1616	714;4673;5	True;True;	721;4703;5	4357;4358;	2347;2348;	2348;1534
737	1878;5145;	True;True;	1893;5182;	11609;116	6178;1704	6178;1704
2115	1827;6627;	True;True;	1841;6673;	11282;112	6010;6011;	6011;2174
41	6807	TRUE	6855	42104;421	22380;223	22382
1068	375;494;13	True;True;	376;495;13	2256;2257;	1241;1242;	1244;1655;
1127	53;8599;10	True;True;	53;8665;10	305;306;30	149;150;27	149;28000;
1107	244;343;13	True;True;	244;344;13	1467;1468;	818;819;11	818;1139;4

	945	458;912;30	True;True;	459;919;31	2771;2772;	1537;1538;	1538;2955;
	37	1359;5912	True;True	1369;5955	8324;8325;	4504;1956	4504;1956
	936	1198;1678;	True;True;	1207;1691;	7344;7345;	3953;5520;	3953;5527;
	1056	598;1308;2	True;True;	601;1317;2	3596;3597;	1976;4326;	1976;4327;
	932	966;10368	True;True	973;10451	5868;5869;	3129;3130;	3129;3424
	1048	2782;8405;	True;True;	2803;8469;	17334;173	9141;2735	9141;2735
	209	2912;3768;	True;True;	2936;3794;	18144;181	9555;1238	9555;1238
	466	7113	TRUE	7172	43940;439	23295;232	23297
	1084	2420;4086;	True;True;	2437;4113;	15038;150	7954;7955;	7955;1338
	240	4857;4871;	True;True;	4890;4904;	29964;299	16033;160	16033;160
	1559	304;1114;1	True;True;	305;1121;1	1848;1849;	1026;1027;	1026;3667;
	1686	9295	TRUE	9365	57286;572	30256;302	30257
	375	11349	TRUE	11434	70486;704	37404	37404
	595	770;1333;2	True;True;	777;1343;2	4702;8170;	2531;4418;	2531;4421;
	1320	1085;2654;	True;True;	1092;2674;	6647;6648;	3579;8733;	3579;8736;
	850	985;1190;1	True;True;	992;1199;1	6027;6028;	3225;3226;	3226;3916;
	1245	774;3110;5	True;True;	781;3134;5	4724;4725;	2540;1019	2540;1019
	2034	1088;1904;	True;True;	1095;1919;	6666;6667;	3590;6242;	3590;6242;
	291	5140	TRUE	5177	31797;317	17037;170	17038
	1043	83;376;641	True;True;	83;377;645	484;485;48	266;267;26	267;1251;2
	2155	1100;1558;	True;True;	1107;1571;	6748;6749;	3627;3628;	3629;5163;
	686	3816	TRUE	3842	23660;236	12555;125	12556
+	334	1389;4043;	True;True;	1399;4069;	8504;8505;	4589;1323	4589;1323
+	335	418;594;13	True;True;	419;597;14	2548;2549;	1414;1415;	1415;1960;
+	339	417;594;12	True;False;	418;597;12	2542;2543;	1410;1411;	1411;1960;
	937	838;874;93	True;True;	845;881;94	5107;5108;	2736;2737;	2738;2853;
+	330	68;590;789	True;True;	68;592;796	389;390;39	197;198;19	200;1944;2
+	1205	1629;3315;	True;True;	1642;3340;	9976;9977;	5370;1084	5370;1084
+	327	3165;4031;	True;True;	3189;4057;	19677;196	10359;131	10359;131
+	341	877;2953;3	False;False	884;2977;3	5336;5337;	2859;2860;	2860;9685;
+	336	876;1971;2	True;True;	883;1987;2	5328;5329;	2856;2857;	2858;6479;
+	324	220;236;50	True;True;	220;236;51	1334;1335;	733;734;73	736;794;16
	920	220;236;87	False;False	220;236;88	1334;1335;	733;734;73	736;794;28
+	350	234;548;54	True;True;	234;550;55	1414;1415;	776;777;77	777;1788;1
+	328	235;875;93	True;True;	235;882;94	1423;1424;	788;789;79	792;2854;3
	1533	4847;5282;	True;True;	4880;5319;	29911;299	16003;160	16004;175
	1771	7156;8085;	True;True;	7215;8148;	44200;442	23417;264	23417;264
	1195	3643;6065;	True;True;	3669;6108;	22608;226	11983;119	11987;199
	1641	474	TRUE	475	2857;2858;	1584;1585;	1585
	625	257;2104;2	True;True;	257;2120;2	1552;1553;	870;871;69	870;6964;9
	1592	4872	TRUE	4905	30035;300	16063;160	16063
	697	7142;7548;	True;True;	7201;7609;	44115;441	23388;233	23389;246
	1681	6459	TRUE	6505	39973;399	21199;212	21199
	1520	1516;3603	True;True	1528;3629	9320;9321;	5025;5026;	5026;1184
	426	3830;3977;	True;True;	3856;4003;	23750;237	12591;125	12593;130
	1249	1144;3264;	True;True;	1151;3289;	6990;6991;	3751;3752;	3752;1068
	1250	2612;3412;	True;True;	2632;3437;	16253;162	8602;8603;	8606;1117
	1624	2934;7222;	True;True;	2958;7281;	18289;182	9632;2364	9632;2364
	1092	408;694;13	True;True;	409;698;13	2468;2469;	1376;1377;	1379;2254;
	1517	285;406;60	True;True;	286;407;60	1729;1730;	960;961;13	961;1373;1

84	10855	TRUE	10940	67396;673	35865;358	35867
775	3401;4253;	True;True;	3426;4280;	21083;210	11136;139	11136;139
1595	43;2333;34	True;True;	43;2349;34	234;235;23	120;121;12	126;7683;1
1001	1583;2141;	True;True;	1596;2157;	9726;9727;	5236;5237;	5236;7088;
1596	622;834;11	True;True;	626;841;12	3761;3762;	2042;2043;	2042;2728;
439	8810	TRUE	8877	54245;542	28668;286	28668
107	1260;2909;	True;True;	1269;2933;	7740;7741;	4180;9547;	4180;9547;
1285	2727;2812;	True;True;	2747;2833;	16975;169	8957;9234;	8957;9234;
1507	557;558;55	True;True;	559;560;56	3358;3359;	1838;1839;	1838;1848;
7	2759;6167;	True;True;	2779;6210;	17173;171	9071;2032	9071;2032
1621	1178;6153	True;True	1187;6196	7225;7226;	3874;3875;	3874;2026
1805	1064;3530	True;True	1071;3555	6522;6523;	3496;1162	3496;1162
867	164;6147;9	True;True;	164;6190;9	982;983;98	547;548;20	548;20243;
1253	328;377;47	True;True;	329;378;47	1980;1981;	1098;1099;	1099;1257;
1688	398;720;44	True;True;	399;727;44	2408;2409;	1342;1343;	1342;2362;
837	9030	TRUE	9098	55622;556	29415;294	29419
1790	2547;7169;	True;True;	2566;7229;	15850;158	8365;2347	8365;2347
1875	1008;1106;	True;True;	1015;1113;	6190;6191;	3321;3322;	3322;3646;
2068	2980;4081;	True;True;	3004;4108;	18566;185	9768;9769;	9769;1337
2092	3805;8378;	True;True;	3831;8441;	23598;235	12509;272	12509;272
1177	2962;5357;	True;True;	2986;5395;	18465;184	9706;1780	9706;1780
1540	2331;4618;	True;True;	2347;4648;	14454;144	7679;7680;	7681;1513
988	517;1552;2	True;True;	518;1565;2	3130;3131;	1713;1714;	1714;5156;
1627	3623;5226;	True;True;	3649;5263;	22489;224	11907;119	11909;173
500	2590;3911;	True;True;	2609;3937;	16117;161	8527;1282	8527;1282
1775	1939;7947;	True;True;	1955;8010;	12009;120	6377;2599	6377;2599
892	1684;1868;	True;True;	1697;1882;	10341;103	5543;5544;	5544;6145;
948	3132;3557;	True;True;	3156;3582;	19456;194	10250;102	10252;116
688	3319;4811;	True;True;	3344;4844;	20584;205	10859;108	10860;158
1733	428;3924;8	True;True;	429;3950;8	2604;2605;	1453;1454;	1455;1285
876	2878;3556;	True;True;	2901;3581;	17957;179	9462;1169	9462;1169
811	4714;6412;	True;False;	4744;6458;	29007;290	15472;210	15472;210
1118	4602;6810;	True;True;	4632;6858;	28320;283	15099;151	15099;224
650	4713;7245;	True;True;	4743;7304;	29000;290	15470;154	15471;237
1761	9100	TRUE	9168	56071;560	29626;296	29628
657	5571;9239;	True;True;	5611;9309;	34542;345	18419;184	18420;300
935	2897;3291;	True;True;	2921;3316;	18060;204	9512;1077	9512;1077
305	7641	TRUE	7703	47200;472	25025	25025
1638	2413;4481;	True;True;	2430;4511;	14993;149	7931;7932;	7933;1470
1738	9994;1057	True;True;	10071;106	61802;618	32783;349	32783;349
132	6340;9710	True;True	6386;9785	39289;392	20831;208	20832;318
1754	1111;2887;	True;True;	1118;2910;	6808;1800	3656;9482;	3656;9482;
1732	6056	TRUE	6099	37509;375	19949;199	19950
1904	104;105;40	True;True;	104;105;40	613;614;61	328;329;33	328;331;13
159	10354	TRUE	10437	64219;642	34199;342	34199
1007	653;1299;8	True;True;	657;1308;8	3956;3957;	2132;2133;	2135;4291;
1025	1307;4966	True;True	1316;5003	8004;8005;	4323;4324;	4324;1639
577	3216;5967	True;True	3241;6010	19967;199	10487;197	10487;197
1034	6110;7764	True;True	6153;7826	37878;378	20120;254	20120;254
1234	723;2363;7	True;True;	730;2380;7	4401;4402;	2366;7803;	2366;7803;

18	3617;7813;	True;True;	3643;7875;	22450;2245	11885;118	11889;2558
949	4289;4773;	True;True;	4316;4805;	26525;294	14059;156	14059;156
367	788	TRUE	795	4810;4811;	2576;2577	2576
1866	2711	TRUE	2731	16875;168	8893;8894;	8893
175	2779;1106	True;True	2799;1114	17304;173	9134;3652	9134;36527
1625	813;845;98	True;True;	820;852;99	4958;4959;	2662;2663;	2663;2754;
264	2593;2637;	True;True;	2612;2657;	16127;161	8532;8691;	8532;8691;
1238	368;741;23	True;True;	369;748;24	2202;2203;	1216;1217;	1219;2411;
1609	2940;5303;	True;True;	2964;5341;	18328;183	9641;1764	9641;17647
2099	3370	TRUE	3395	20901;209	11027;110	11027
2152	11399	TRUE	11484	70776	37561	37561
641	35;6244	True;True	35;6287	188;38732;	87;20554;2	87;20556
1594	2875;3335;	True;True;	2898;3360;	17940;179	9456;9457;	9456;1091
1807	8462	TRUE	8526	52139;521	27523;275	27523
1289	24;3517	True;True	24;3542	133;134;13	60;61;62;1	62;11567
138	1697;1903;	True;True;	1710;1918;	10416;104	5581;6239;	5581;6241;
1308	3829;7511;	True;True;	3855;7572;	23742;237	12590;245	12590;245
1618	47;475;752	True;True;	47;476;758	266;267;26	138;1587;2	138;1587;2
395	387	TRUE	388	2346	1298	1298
257	2253;5248	True;True	2269;5285	13995;139	7426;7427;	7427;17427
970	219;1291;8	True;True;	219;1300;8	1331;1332;	732;4265;2	732;4265;2
1796	10661	TRUE	10746	66184;661	35243;352	35248
733	1528;2326;	True;True;	1540;2342;	9385;9386;	5062;7666;	5062;7666;
773	1524;3777;	True;True;	1536;3803;	9356;9357;	5045;5046;	5046;12412
183	3523	TRUE	3548	21919;219	11592	11592
193	1884;1920;	True;True;	1899;1935;	11647;116	6191;6298;	6191;6298;
263	8214;1048	True;True	8277;1056	50723;507	26827;346	26827;3465
912	1186;8427	True;True	1195;8491	7276;5193	3904;2742	3904;2742
860	1656;2285;	True;True;	1669;2301;	10159;101	5449;5450;	5450;7530;
1590	2175;5095;	True;True;	2191;5132;	13520;135	7189;7190;	7189;16887
288	722;9708	True;True	729;9783	4394;4395;	2365;3180	2365;3180
630	1089	TRUE	1096	6673;6674;	3591;3592	3592
1339	5934;6226;	True;True;	5977;6269;	36795;367	19641;205	19641;205
536	584;3956	True;True	586;3982	3511;3512;	1927;1294	1927;1295
1368	3353;3792;	True;True;	3378;3818;	20799;208	10975;124	10975;124
2072	11470	TRUE	11556	71247;712	37814	37814
2014	107;5114;1	True;True;	107;5151;1	632;633;63	344;16955;	344;16955;
432	8581	TRUE	8645	52879;528	27921;279	27924
1936	8469	TRUE	8533	52180;521	27547	27547
453	9479	TRUE	9550	58480;584	30956	30956
156	6651;8990	True;True	6698;9058	41161;411	21843;292	21843;292
17	1268;8583	True;True	1277;8647	7776;7777;	4202;4203;	4202;2793
306	18;1331;65	True;True;	18;1341;65	104;8164;8	47;4416;21	47;4416;21
683	7003	TRUE	7057	43299;433	22995;229	22995
1684	81;1054;24	True;True;	81;1061;25	474;475;47	259;260;26	259;3465;8
1923	11020	TRUE	11105	68447;684	36391;363	36397
761	8686;1040	True;True	8753;1049	53552;535	28292;282	28293;343
1002	716	TRUE	723	4369;4370	2354	2354
1662	1103;3038;	True;True;	1110;3062;	6765;6766;	3640;9974;	3640;9975;
249	5615;1001	True;True;	5655;1009	34810;348	18569;185	18569;328

450	5332;1151	True;True	5370;1159	33010;330	17727;379	17727;379
1947	11563	TRUE	11649	71799;718	38118	38118
1819	3050;3574;	True;True;	3074;3599;	18991;189	10004;117	10004;117
1793	7364	TRUE	7423	45498;454	24115;241	24115
644	106;1132;2	True;True;	106;1139;2	624;625;62	332;333;33	341;3721;7
1760	6393;1085	True;True;	6439;1094	39583;395	21006;358	21006;358
571	1298;2826;	True;True;	1307;2847;	7944;7945;	4288;4289;	4288;9285;
2163	3802;5650;	True;True;	3828;5691;	23575;235	12499;186	12499;186
2078	3448;5866;	False;False	3473;5909;	21406;214	11318;113	11321;193
2136	3823;9234	True;True	3849;9304	23705;237	12577;300	12577;300
443	4146;5508	True;True	4173;5548	25698;256	13604;136	13610;182
2150	5692;1087	True;True	5734;1096	35297;352	18812;188	18813;359
794	2414;3575;	True;True;	2431;3601;	15000;150	7934;7935;	7935;1177
717	4949	TRUE	4986	30550;305	16325;163	16325
805	232;3125	True;True	232;3149	1401;1402;	767;768;76	768;10223
166	6641	TRUE	6688	41100;411	21800;218	21803
2032	25;3486;64	True;True;	25;3511;65	137;138;13	63;64;65;6	67;11471;2
1666	640;3245;9	True;True;	644;3270;9	3874;3875;	2090;1059	2090;1059
889	2517;3374;	True;True;	2536;3399;	15654;156	8258;8259;	8258;1104
861	9;948;1257	True;True;	9;955;1266	60;61;62;6	23;24;3090	24;3090;41
290	1281	TRUE	1290	7841;7842;	4236	4236
1924	10163	TRUE	10243	62933;629	33397;333	33397
309	2576;7444;	True;True;	2595;7504;	16016;160	8458;2435	8458;2435
1550	1964;2657;	True;True;	1980;2677;	12178;121	6451;8743;	6451;8744;
836	3330;8989;	False;True;	3355;9057;	20666;206	10903;109	10903;292
1148	794;10167	True;True	801;10247	4840;4841;	2590;3340	2590;3340
682	6316;6842;	True;True;	6362;6890;	39146;391	20767;225	20767;225
1780	960;4091	True;True	967;4118	5836;5837;	3118;1339	3118;1339
1965	5179;1003	True;True;	5216;1011	32031;320	17157;171	17158;329
1128	602;678;75	True;True;	605;682;75	3624;3625;	1988;1989;	1989;2203;
1344	48;1935	True;True	48;1951	272;273;27	139;6360;6	139;6360
28	366;1511;6	True;True;	367;1523;6	2195;2196;	1214;5004;	1214;5004;
252	2971;1039	True;True	2995;1048	18516;645	9733;3436	9733;3436
463	779;865;13	True;True;	786;872;13	4749;4750;	2550;2551;	2552;2824;
2095	2321;4171;	True;True;	2337;4198;	14387;143	7657;7658;	7658;1370
520	8852	TRUE	8919	54490;544	28808;288	28810
474	3969	TRUE	3995	24600;246	12988;129	12988
420	141;972;18	True;True;	141;979;18	861;862;86	487;488;31	488;3164;6
70	8324;1054	True;True	8387;1062	51326;513	27112;348	27112;348
1937	451;1010;1	True;True;	452;1017;1	2735;2736;	1516;1517;	1519;3324;
2035	567;11571	True;True	569;11657	3424;3425;	1880;1881;	1881;3814
869	2052;5453;	True;True;	2068;5492;	12739;127	6783;6784;	6785;1806
810	7501	TRUE	7562	46333;463	24553;245	24554
281	674;10000	True;True	678;10078	4078;4079;	2185;3281	2185;3281
2059	162;253;74	True;True;	162;253;75	966;967;96	542;543;85	542;859;24
938	2361	TRUE	2378	14697;146	7799;7800	7799
512	620;1202;1	True;True;	623;1211;1	3737;3738;	2034;2035;	2035;3966;
559	2142;2649	True;True	2158;2669	13305;133	7089;7090;	7089;8720
679	1020;1553;	True;True;	1027;1566;	6260;6261;	3354;3355;	3358;5157;
1207	170;172;46	False;False	170;172;46	1016;1017;	565;566;56	565;576;15

1211	1303;2212;	False;False	1312;2228;	7980;7981;	4304;4305;	4305;7297;
1758	1303;2212;	False;False	1312;2228;	7980;7981;	4304;4305;	4305;7297;
1206	170;172;17	True;True;	170;172;17	1016;1017;	565;566;56	565;576;58
299	1206;3922	True;True	1215;3948	7391;7392;	3982;1285	3982;1285
1154	41;250;251	True;True;	41;250;251	229;1494;1	116;831;83	116;837;83
756	6248;9263	True;True	6291;9333	38756;387	20568;301	20568;301
1766	2664;4916	True;True	2684;4953	16594;165	8760;8761;	8761;1622
1975	440;904;27	True;True;	441;911;27	2677;2678;	1485;1486;	1485;2941;
129	2497;2643;	True;True;	2515;2663;	15518;164	8206;8706;	8206;8706;
1583	16;2134;10	True;True;	16;2150;10	97;13253;1	42;7068;70	42;7069;33
231	2338;5004;	True;True;	2354;5041;	14500;145	7697;7698;	7699;1656
161	1277;1832;	True;True;	1286;1846;	7819;7820;	4230;6027;	4230;6028;
1949	6573	TRUE	6619	40731;407	21575;215	21575
56	11608	TRUE	11694	72094;720	38300	38300
482	6367	TRUE	6413	39442;394	20907;209	20908
612	2373;1154	True;True	2390;1163	14771;147	7841;7842;	7842;3807
401	26;1953	True;True	26;1969	151;152;15	69;70;6422	69;6422
827	4731;5669	True;True	4761;5711	29118;291	15515;187	15515;187
839	11048	TRUE	11133	68597;685	36465;364	36465
629	3960	TRUE	3986	24541;245	12955;129	12955
894	754;1640;3	True;True;	761;1653;3	4586;4587;	2452;2453;	2452;5403;
1283	9408	TRUE	9479	58029;580	30648	30648
92	5766;5993;	True;True;	5808;6036;	35766;357	19071;190	19071;197
1061	1539;2960;	True;True;	1552;2984;	9468;9469;	5104;5105;	5105;9704;
402	5634;6319	True;True	5675;6365	34954;349	18643;207	18643;207
418	1628;4574	True;True	1641;4604	9968;9969;	5367;5368;	5369;1501
2117	6815	TRUE	6863	42172;421	22428	22428
1946	246	TRUE	246	1479;1480	823;824	824
121	17;4987	True;True	17;5024	98;99;100;	43;44;45;4	44;16498
1392	2065;5839;	True;True;	2081;5882;	12807;128	6816;6817;	6817;1930
2105	2495;1137	True;True	2513;1145	15506;155	8203;8204;	8204;3748
1546	184;185;18	True;True;	184;185;18	1109;1110;	621;622;62	621;622;62
421	10966	TRUE	11051	68108;681	36208;362	36209
2156	5219;5446;	True;True;	5256;5485;	32296;322	17331;173	17332;180
1622	283;338;14	True;True;	284;339;14	1720;1721;	951;952;95	955;1121;4
1623	283;338;14	False;False	284;339;14	1720;1721;	951;952;95	955;1121;4
1254	2829;8533	True;True	2850;8597	17629;176	9291;9292;	9291;2776
1665	1361;2872;	True;True;	1371;2895;	8335;1792	4508;9447;	4508;9450;
98	5210;8400;	True;True;	5247;8463;	32239;322	17289;172	17290;273
1483	8853;9336;	True;True;	8920;9406;	54495;544	28811;288	28814;304
253	3428	TRUE	3453	21259;212	11234;112	11234
208	6121;7794;	True;True;	6164;7856;	37959;379	20167;201	20168;254
1911	2893;3010;	True;True;	2917;3034;	18045;180	9500;9881;	9500;9882;
1871	7076;7190;	True;True;	7133;7249;	43734;437	23190;231	23190;235
1258	1104;1737;	True;True;	1111;1750;	6768;6769;	3641;3642;	3641;5692;
1741	8993	TRUE	9061	55431;554	29299;293	29299
704	6441	TRUE	6487	39857;398	21146;211	21148
51	2721;3998;	True;True;	2741;4024;	16934;169	8930;8931;	8930;1307
931	2362;7719;	True;True;	2379;7781;	14703;147	7801;7802;	7802;2526
1647	388;627;11	True;True;	389;631;11	2347;2348;	1299;1300;	1300;2061;

1102	4680;5142;	True;True;	4710;5179;	28790;287	15354;153	15356;170
681	6187;1123	True;True;	6230;1132	38376;383	20377;370	20377;370
2107	954;1725;2	True;True;	961;1738;2	5801;5802;	3102;5650;	3102;5650;
1952	9620	TRUE	9694	59350;593	31446;314	31446
581	5427;6894;	True;True;	5466;6943;	33595;335	17988;226	17988;226
1545	1012;5705;	True;True;	1019;5747;	6213;6214;	3328;3329;	3329;1886
522	8500	TRUE	8564	52397;523	27650;276	27651
378	4211	TRUE	4238	26107;261	13812;138	13813
1759	2218;3470;	True;True;	2234;3495;	13772;137	7314;1140	7314;1140
2113	3209;3995;	True;True;	3234;4021;	19926;199	10465;130	10465;130
1631	29;71;262;	True;True;	29;71;262;	162;410;41	74;210;211	74;214;886
1374	4969;8076;	True;True;	5006;8139;	30682;306	16400;263	16400;263
1828	2890;2908;	True;True;	2913;2932;	18019;180	9493;9545;	9493;9545;
1302	4972;7610;	True;True;	5009;7672;	30705;307	16418;164	16418;249
428	1265;2584;	True;True;	1274;2603;	7768;7769;	4196;4197;	4196;8500;
529	1377;3611;	True;True;	1387;3637;	8422;8423;	4546;1186	4546;1186
1852	958;1749;1	True;True;	965;1762;1	5822;5823;	3108;3109;	3110;5729;
90	956;7662;7	True;True;	963;7724;7	5810;5811;	3105;2508	3105;2508
2096	10142	TRUE	10222	62811;628	33328;333	33332
671	1381;2078	True;True	1391;2094	8441;8442;	4553;4554;	4553;6851
649	901;3171;3	True;True;	908;3195;3	5476;5477;	2934;2935;	2937;1037
622	4851;5808;	True;True;	4884;5851;	29933;299	16018;160	16018;192
1336	223;666;36	True;True;	223;670;36	1354;1355;	741;742;74	744;2169;1
1223	1634;2499;	True;True;	1647;2518;	10002;100	5381;5382;	5383;8216;
1823	1059;1042	True;True;	1066;1051	6490;6491;	3481;3482;	3481;3446
1813	3347	TRUE	3372	20774;207	10956	10956
1597	148;1688;4	True;True;	148;1701;4	900;901;90	508;509;55	508;5558;1
1412	166;4999;5	True;True;	166;5036;5	994;30911;	553;16548;	553;16549;
1982	1034;5487;	True;True;	1041;5527;	6329;6330;	3394;1815	3394;1815
781	5984	TRUE	6027	37081;370	19759;197	19760
1468	2192;2193;	True;True;	2208;2209;	13621;136	7237;7238;	7242;7243;
1513	6359;6585;	True;True;	6405;6631;	39398;393	20883;208	20884;216
77	1812;8995;	True;True;	1826;9063;	11177;111	5945;5946;	5946;2930
2139	5738;6572;	True;True;	5780;6618;	35580;355	18973;215	18973;215
1824	11592	TRUE	11678	71986	38227	38227
1967	224;1509;4	True;True;	224;1521;4	1361;1362;	745;746;50	746;5002;1
808	917	TRUE	924	5553;5554	2967	2967
2001	4880;1161	True;True	4913;1169	30081;300	16087;383	16087;383
430	2224	TRUE	2240	13821;138	7336;7337	7336
63	8092	TRUE	8155	49936;499	26422	26422
744	2128;3849;	True;True;	2144;3875;	13212;132	7046;7047;	7048;1265
2123	2107;4220;	True;True;	2123;4247;	13078;130	6973;6974;	6973;1385
1996	4002;7850	True;True	4028;7912	24802;248	13083;130	13084;257
2026	116;785;18	True;True;	116;792;19	678;679;68	371;372;37	372;2565;6
447	3167;8065;	True;True;	3191;8128;	19686;196	10361;103	10362;263
1082	604;2124;2	True;True;	607;2140;2	3635;3636;	1992;1993;	1992;7035;
308	8163	TRUE	8226	50417	26677	26677
943	1485;3962;	True;True;	1496;1497;	9116;9117;	4936;4937;	4946;1296
501	10279	TRUE	10361	63715;637	33878;338	33881
1809	4600;8112;	True;True;	4630;8175;	28306;283	15095;264	15095;264

1810	961;8783;8	True;True;	968;8850;8	5843;5844;	3119;2857	3119;2857
1757	8717;9092;	True;True;	8784;9160;	53729;537	28382;296	28382;296
1884	318;5235;6	True;True;	319;5272;6	1924;1925;	1067;1068;	1070;1737
1018	957;2388;2	True;True;	964;2405;2	5816;5817;	3106;3107;	3106;7877;
133	6502	TRUE	6548	40290;402	21361;213	21362
1051	1886;3496;	False;False	1901;3521;	11658;116	6193;1149	6193;1149
1104	1886;1887;	True;True;	1901;1902;	11658;116	6193;6194;	6193;6194;
256	5678;7623;	True;True;	5720;7685;	35215;352	18765;187	18772;249
312	23;183;663	True;True;	23;183;668	129;130;13	58;59;620;	58;620;217
1910	1890;6389	True;True	1905;6435	11672;116	6203;2099	6203;2099
382	825	TRUE	832	5020;5021;	2691	2691
307	4691	TRUE	4721	28864;288	15397	15397
1698	2177;4232;	True;True;	2193;4259;	13536;135	7197;7198;	7198;1387
21	10833	TRUE	10918	67249;672	35786;357	35793
2023	1057;2178;	True;True;	1064;2194;	6479;6480;	3470;3471;	3472;7199;
1920	9733	TRUE	9808	60125;601	31883	31883
157	7056	TRUE	7111	43588;435	23121;231	23123
678	7055	TRUE	7110	43584;435	23117;231	23120
919	362;2764;2	True;True;	363;2784;2	2173;2174;	1207;1208;	1208;9088;
1859	9588	TRUE	9661	59160;591	31356;313	31356
1217	2086;3079;	True;True;	2102;3103;	12924;129	6877;1010	6877;1010
2047	5572	TRUE	5612	34549;345	18422;184	18422
1660	4560;7656;	True;True;	4590;7718;	28052;280	14953;250	14953;250
171	2807	TRUE	2828	17502;175	9218;9219;	9220
236	9141	TRUE	9210	56328;563	29762	29762
1840	2951;5879;	True;True;	2975;5922;	18404;184	9680;9681;	9680;1943
728	66;1253;34	True;True;	66;1262;35	380;7705;7	194;4150;4	194;4151;1
763	780;7748	True;True	787;7810	4757;4758;	2554;2555;	2555;2536
1888	1615;8564	True;True	1628;8628	9896;9897;	5340;5341;	5340;2786
1454	2356;3013;	False;False	2372;2373;	14653;146	7767;7768;	7768;9889;
1453	2356;3013;	True;True;	2372;2373;	14653;146	7767;7768;	7768;9889;
1109	1518;1680;	True;True;	1530;1693;	9333;9334;	5029;5030;	5029;5534;
1261	10043	TRUE	10121	62122;621	32951;329	32951
1170	576;3243;4	False;True;	578;3268;4	3474;2013	1906;1058	1906;1058
1455	4156;4178;	True;True;	4183;4205;	25762;257	13644;136	13645;137
1133	4177;6759	True;True	4204;6807	25890;258	13716;137	13716;222
1499	4175;5771;	True;True;	4202;5813;	25881;258	13712;190	13712;190
1511	600;997;11	True;True;	603;1004;1	3612;3613;	1985;1986;	1986;3278;
725	9412	TRUE	9483	58059;580	30716;307	30719
2135	1519;1049	True;True	1531;1057	9338;9339;	5035;3468	5035;3468
1826	1510	TRUE	1522	9282	5003	5003
660	9637;9891	True;True	9711;9968	59481;594	31523;315	31523;324
383	4924;7027	True;True	4961;7082	30401;304	16245;230	16245;230
816	1616;1965;	True;True;	1629;1981;	9903;9904;	5342;6452;	5342;6454;
1648	205;3898;1	True;True;	205;3924;1	1233;1234;	680;681;68	682;12791;
1530	167;978;97	True;True;	167;985;98	995;996;99	554;555;55	554;3193;3
1192	980;3799;5	True;True;	987;3825;5	5982;5983;	3200;3201;	3204;1249
1570	2036;3798;	True;False;	2052;3824;	12633;126	6713;6714;	6714;1249
1160	3302;4139;	True;True;	3327;4166;	20473;204	10800;108	10805;135
1158	1529;1642;	True;True;	1541;1655;	9393;9394;	5063;5064;	5068;5408;

640	1894	TRUE	1909	11698;116	6215	6215
1863	3378	TRUE	3403	20944;209	11058	11058
832	3634;4973	True;True	3660;5010	22566;225	11954;164	11954;164
1323	1023;1042;	True;True;	1030;1049;	6281;6282;	3368;3369;	3372;3422;
712	8875	TRUE	8942	54670;546	28923	28923
246	385;4015;5	True;True;	386;4041;5	2335;2336;	1295;1314	1295;1314
268	9610	TRUE	9683	59292;592	31411;314	31413
1755	1364;4508	True;True	1374;4538	8347;8348;	4517;1479	4517;1479
705	4012;1078	True;True;	4038;1087	24861;248	13135;356	13135;356
2033	54;899;173	True;True;	54;906;174	311;312;31	151;2927;2	151;2931;5
1838	5661;8589	True;True	5703;8653	35135;351	18738;279	18738;279
1497	3071;4048;	True;True;	3095;4074;	19118;191	10075;132	10075;132
878	313	TRUE	314	1899;1900;	1049;1050;	1050
1167	796;1201;3	True;True;	803;1210;3	4845;4846;	2592;2593;	2593;3965;
278	6571	TRUE	6617	40721;407	21569;215	21570
91	1138;2482;	True;True;	1145;2500;	6960;1541	3738;8147;	3738;8148;
1588	7231;7601;	True;True;	7290;7663;	44667;446	23668;248	23668;248
1287	8247	TRUE	8310	50922;509	26929;269	26931
1683	1279;2871;	True;True;	1288;2894;	7828;7829;	4232;4233;	4232;9446;
1220	209;3046;7	True;True;	209;3070;7	1267;1268;	700;701;70	702;9994;2
1891	332	TRUE	333	2001;2002;	1107	1107
902	133;400;48	True;True;	133;401;48	799;800;80	434;435;43	437;1347;1
1077	647;701;32	True;True;	651;705;33	3910;3911;	2113;2114;	2114;2283;
1779	2024;5884	True;True	2040;5927	12552;125	6669;6670;	6669;1945
283	8821;1114	True;True	8888;1122	54304;543	28693;367	28693;367
292	2440	TRUE	2457	15145;151	8010	8010
2154	603;943;11	True;True;	606;950;11	3631;3632;	1990;1991;	1991;3070;
289	4516	TRUE	4546	27798;277	14817;148	14819
1984	1727;6642;	True;True;	1740;6689;	10594;105	5652;5653;	5652;2180
745	9867	TRUE	9944	61016	32386	32386
1598	3828;6239	True;True	3854;6282	23738;237	12589;205	12589;205
1930	481;4993	True;True	482;5030	2895;2896;	1604;1653	1604;1653
2141	405;1687;4	True;True;	406;1700;4	2451;2452;	1368;1369;	1370;5556;
1085	7130;8226;	True;True;	7189;8289;	44034;440	23343;233	23344;268
1804	2102;2274;	True;True;	2118;2290;	13048;130	6956;6957;	6959;7487;
928	4062;4968;	True;True;	4088;5005;	25180;251	13320;133	13323;163
1031	28;247;605	True;True;	28;247;608	160;161;14	72;73;825;	72;828;199
87	315;605;10	True;False;	316;608;10	1909;1910;	1055;1056;	1058;1995;
1255	3133;3697;	True;True;	3157;3723;	19463;194	10255;121	10255;121
1475	2518	TRUE	2537	15662;156	8263	8263
1832	8471	TRUE	8535	52194;521	27549;275	27549
1645	65;326;426	True;True;	65;327;427	379;1965;1	193;1090;1	193;1090;1
795	9054;1052	True;True	9122;1060	55761;557	29483;347	29483;347
823	7371	TRUE	7430	45537;455	24136;241	24136
767	2145;3060;	True;True;	2161;3084;	13319;133	7098;1003	7098;1003
987	306;316;93	True;True;	307;317;94	1858;1914;	1030;1060;	1030;1060;
2045	9034	TRUE	9102	55651;556	29427	29427
1650	1332;2784;	True;True;	1342;2805;	8168;8169;	4417;9145;	4417;9146;
1651	536;2784;4	True;False;	538;2805;4	3223;3224;	1751;1752;	1752;9146;
134	568;1621;5	True;True;	570;1634;5	3429;3430;	1884;5352;	1884;5352;

1015	586;2301;3	True;True;T	588;2317;3	3519;3520;	1931;1932;	1934;7585;
1769	0;9957	True;True	0;10034	0;1;2;3;4;6	0;1;2;3;4;3	1;32663
217	586;2300;3	False;True;	588;2316;3	3519;3520;	1931;1932;	1934;7581;
371	4606;6654	True;True	4636;6701	28338;2833	15106;1510	15106;2184
224	5786	TRUE	5828	35866;3586	19115;1911	19119
498	9130;10485	True;True	9199;10565	56264;5626	29724;3466	29724;3466
1731	991;992;23	True;True;T	998;999;23	6061;6062;	3243;3244;	3246;3248;
1548	49;2429;33	True;True;T	49;2446;34	280;281;28	140;7982;7	140;7982;1
415	1870;6897;	True;True;T	1884;6946;	11542;1154	6148;6149;	6149;22651
146	2034;2372;	True;True;T	2050;2389;	12610;1261	6702;6703;	6703;7838;
880	1525;2372;	True;False;	1537;2389;	9364;9365;	5050;5051;	5050;7838;
1818	1045;7669;	True;True;T	1052;7731;	6400;6401;	3435;3436;	3436;25106
190	577	TRUE	579	3475;3476;	1907;1908;	1907
384	5518	TRUE	5558	34231;3423	18268;1826	18269
2089	3463	TRUE	3488	21514	11379	11379
467	7411	TRUE	7471	45783;4578	24260;2426	24260
909	123;124;44	True;True;T	123;124;44	725;726;72	402;403;40	406;409;15
2103	1031;3120;	True;True;T	1038;3144;	6315;6316;	3387;3388;	3391;10216
834	5538;9238	True;True	5578;9308	34344;3434	18319;1832	18321;3008
863	90;113;450	True;True;T	90;113;451	527;528;52	279;365;36	279;366;15
1734	643;902;91	True;True;T	647;909;92	3893;3894;	2101;2102;	2101;2939;
1800	11634	TRUE	11720	72273;7227	38397;3839	38397
786	2849;6461;	True;True;T	2870;6507;	17764;1776	9365;9366;	9366;21208
2100	11273	TRUE	11358	70010;7001	37169	37169
2087	2459;3185	True;True	2477;3209	15276;1527	8086;8087;	8087;10407
1784	900;4820;6	True;True;T	907;4853;6	5469;5470;	2932;2933;	2932;15867
1687	1942;5077;	True;True;T	1958;5114;	12030;1203	6384;6385;	6385;16815
1703	1843;2223;	True;True;T	1857;2239;	11377;1137	6062;6063;	6065;7333;
1897	9240	TRUE	9310	56948;5694	30090;3009	30093
1267	1691;2546;	True;True;T	1704;2565;	10386;1038	5566;5567;	5568;8362;
194	4688	TRUE	4718	28840;2884	15377;1537	15377
165	769;1356;1	True;True;T	776;1366;1	4694;4695;	2524;2525;	2524;4500;
254	85;142;654	True;True;T	85;142;658	494;495;49	272;489;49	272;489;21
518	1109;5519	True;True	1116;5559	6799;6800;	3653;18271	3653;18271
110	1401;4188;	True;True;T	1411;4215;	8586;8587;	4642;13746	4642;13746
1978	5003;9109	True;True	5040;9178	30929;3093	16564;1656	16564;2965
668	769;2026;2	False;True;	776;2042;2	4694;4695;	2524;2525;	2524;6674;
521	10202	TRUE	10282	63168;6316	33534;3353	33535
1129	3973;10747	True;True	3999;10831	24630;2463	13012;1301	13013;3552
2006	2804;9181	True;True	2825;9250	17487;1748	9212;29901	9212;29901
78	2756;5700;	True;False;	2776;5742;	17155;1715	9064;9065;	9065;18846
1899	9236	TRUE	9306	56929	30086	30086
603	2598	TRUE	2617	16147;1614	8537;8538;	8537
2143	1166;4842	True;True	1175;4875	7146;7147;	3833;3834;	3834;15986
633	1058;2572;	True;True;T	1065;2591;	6487;6488;	3478;3479;	3478;8448;
1801	651;6732;7	True;True;T	655;6780;7	3942;3943;	2126;2127;	2126;22118
1512	872;4649;8	True;True;T	879;4679;8	5301;5302;	2848;15242	2848;15246
410	5195;8527	True;True	5232;8591	32128;3212	17216;1721	17220;2774
814	1780;1983;	True;True;T	1794;1999;	10963;1096	5844;6526;	5844;6529;
955	1374;1913;	True;True;T	1384;1928;	8399;8400;	4536;4537;	4542;6262;

1829	3141;4242;	True;True;	3165;4269;	19519;195	10282;102	10283;139
1695	5245	TRUE	5282	32474	17413	17413
0	466;11430	True;True	467;11516	2818;2819;	1560;3765	1560;3765
1202	44;64;1755	True;True;	44;64;1768	242;243;24	127;128;12	133;187;57
1919	96;833;175	True;True;	96;840;176	567;568;56	296;297;29	297;2726;5
1016	256;1181;1	True;True;	256;1190;1	1545;1546;	865;866;86	869;3883;5
2109	61;635;232	True;True;	61;639;234	358;359;36	181;2083;7	181;2083;7
1029	4112;4218;	True;True;	4139;4245;	25492;254	13468;134	13469;138
784	8019;9186;	True;True;	8082;9255;	49481;566	26192;299	26192;299
1282	5172;1043	True;True;	5209;1051	31989;319	17139;171	17139;344
298	1266;1556;	True;True;	1275;1569;	7770;7771;	4198;4199;	4199;5160;
755	329;4279	True;True	330;4306	1986;1987;	1100;1101;	1100;1403
1999	3061;5478;	True;True;	3085;5518;	19056;190	10033;100	10033;181
45	1999;2025;	True;True;	2015;2041;	12411;124	6590;6671;	6590;6671;
2067	798;10835	True;True	805;10920	4857;4858;	2596;3579	2596;3579
1628	2319;4085;	True;True;	2335;4112;	14375;143	7648;1338	7648;1338
1671	9094;9949	True;True	9162;1002	56029;560	29605;296	29609;326
1528	772;773;50	True;True;	779;780;50	4712;4713;	2537;2538;	2537;2538;
59	1247;1475	True;True	1256;1486	7674;7675;	4135;4903;	4135;4904
702	5164;8976;	True;True;	5201;9044;	31948;319	17115;292	17115;292
490	625	TRUE	629	3783;3784;	2058;2059	2059
6	5596;6682	True;True	5636;6729	34686;346	18505;185	18508;219
594	982;4440;6	True;True;	989;4469;6	6006;6007;	3215;3216;	3215;1455
538	523;1209;4	True;True;	525;1218;4	3163;3164;	1730;1731;	1731;3986;
1125	855;856;28	True;True;	862;863;28	5206;5207;	2790;2791;	2790;2794;
1126	1070;8959;	True;True;	1077;9027;	6557;6558;	3519;3520;	3521;2917
1143	510;2255;2	True;True;	511;2271;2	3091;3092;	1693;1694;	1696;7434;
759	538;633;48	True;True;	540;637;49	3235;3236;	1755;1756;	1756;2078;
1142	891;5338;6	True;True;	898;5376;6	5413;5414;	2905;1774	2905;1774
128	10910	TRUE	10995	67729;677	36016;360	36018
1090	409;1982;3	True;True;	410;1998;3	2476;2477;	1382;1383;	1385;6525;
1298	10151;116	True;True	10231;117	62859;628	33359;333	33361;384
23	3077;7210	True;True	3101;7269	19157;191	10105;236	10105;236
1144	8207;1079	True;True	8270;1087	50680;670	26812;356	26812;356
1146	543;974;38	True;True;	545;981;39	3253;3254;	1767;3168;	1767;3168;
1145	5502;8209;	True;True;	5542;8272;	34138;341	18212;182	18212;268
1912	989;4100	True;True	996;4127	6054;6055;	3239;3240;	3239;1343
651	5929;6266	True;True	5972;6311	36750;367	19613;206	19613;206
2052	7774;9840;	True;True;	7836;9917;	47986;479	25431;254	25432;322
1928	6;1011;398	True;True;	6;1018;401	38;39;40;4	16;17;3325	16;3325;13
589	1458;3946	True;True	1469;3972	8939;8940;	4838;1292	4838;1292
1820	2437	TRUE	2454	15132;151	8007	8007
1243	1950;1039	True;True	1966;1047	12095;120	6409;6410;	6411;3434
510	1638;4931;	False;True;	1651;4968;	10038;100	5400;1626	5400;1626
137	5743;6229	True;True	5785;6272	35614;356	18987;205	18987;205
2097	97;5460;93	True;True;	97;5499;94	572;573;57	299;300;18	300;18089;
187	11425	TRUE	11511	70917;709	37640;376	37640
270	9910	TRUE	9987	61295;612	32532;325	32534
1914	2018;2019;	True;True;	2034;2035;	12514;125	6647;6648;	6647;6648;
1210	6108;6672;	True;True;	6151;6719;	37864;378	20116;201	20117;219

126	4248;5942;	True;True;	4275;5985;	26313;263	13935;196	13935;196
950	570;1981;2	True;True;	572;1997;2	3440;3441;	1889;1890;	1889;6520;
1168	1549;1840;	True;True;	1562;1854;	9530;9531;	5140;5141;	5142;6054;
1028	1721;2946;	True;True;	1734;2970;	10566;105	5639;5640;	5641;9664;
1643	1990;3569;	True;True;	2006;3594;	12351;123	6552;1174	6552;1174
227	6905	TRUE	6955	42712;427	22689;226	22691
1843	516;6063;7	True;True;	517;6106;7	3123;3124;	1711;1712;	1711;1997
1291	1626;3434;	True;True;	1639;3459;	9959;9960;	5360;5361;	5362;1125
634	1716	TRUE	1729	10542;105	5631	5631
1691	2817;6680	True;True	2838;6727	17563;413	9255;2195	9255;2195
517	2189;3149;	True;True;	2205;3173;	13597;135	7225;7226;	7227;1030
687	4109	TRUE	4136	25473;254	13459;134	13460
1845	527;1819;6	True;True;	529;1833;6	3184;3185;	1735;1736;	1735;5972;
2039	1396;1874;	True;True;	1406;1888;	8552;8553;	4620;4621;	4623;6161;
1745	390;436;74	True;True;	391;437;75	2351;2352;	1302;1303;	1304;1478;
1806	4370	TRUE	4397	26905;269	14290;142	14291
1694	4915;6915;	True;True;	4952;6965;	30338;303	16220;162	16221;2272
1564	7153;1082	True;True	7212;1091	44188;441	23411;357	23411;357
549	4330	TRUE	4357	26691;266	14161;141	14162
550	4328	TRUE	4355	26684;266	14159	14159
1481	1217;2617	True;True	1226;2637	7462;7463;	4022;4023;	4025;8620
142	689;1141	True;True	693;1148	4171;4172;	2239;3743;	2239;3745
442	5016;5603	True;True	5053;5643	31011;310	16605;166	16605;1853
1246	955	TRUE	962	5805;5806;	3103;3104	3103
238	1112;1113	True;True	1119;1120	6809;6810;	3657;3658;	3660;3665
533	2713;9483	True;True	2733;9554	16895;168	8906;8907;	8907;3096
1973	6353;7425	True;True	6399;7485	39361;393	20869;242	20869;242
1770	2226;6598;	True;True;	2242;6644;	13833;138	7341;2165	7341;2165
495	4706;4736	True;True	4736;4766	28950;289	15446;154	15446;1553
579	7260	TRUE	7319	44848;448	23767	23767
1869	11010	TRUE	11095	68396;683	36364;363	36369
1849	1708;2265;	True;True;	1721;2281;	10502;105	5615;7458;	5615;7459;
1969	2655;2881;	True;True;	2675;2904;	16538;165	8739;9469;	8739;9469;
1931	1797;4176	True;True	1811;4203	11063;110	5876;5877;	5876;1371
2080	7046	TRUE	7101	43532;435	23096;230	23098
1877	11146;111	True;True	11231;112	69202;692	36783;367	36783;367
243	4605	TRUE	4635	28336;283	15104;151	15104
779	1169;2268;	True;True;	1178;2284;	7161;7162;	3837;7474;	3837;7475;
1152	749;949;12	True;True;	756;956;12	4567;4568;	2442;3092;	2442;3093;
400	1426;2152	True;True	1437;2168	8744;8745;	4739;7110	4739;7110
2065	392;1108;1	True;True;	393;1115;1	2374;2375;	1317;1318;	1317;3651;
72	10309	TRUE	10391	63942;639	34026;340	34028
1619	460;522;13	True;True;	461;524;13	2786;2787;	1541;1729;	1541;1729;
941	2594;6418	True;True	2613;6464	16132;161	8533;2109	8533;2109
1385	2294;2295;	True;True;	2310;2311;	14216;142	7558;7559;	7558;7564;
1191	1259;1598;	True;True;	1268;1611;	7733;7734;	4176;4177;	4179;5294;
1916	8830	TRUE	8897	54365;543	28731;287	28731
2016	1774;4476;	True;True;	1787;4506;	10887;108	5801;5802;	5803;1468
1890	6042;6058	True;True	6085;6101	37425;374	19916;199	19916;199
1150	2567;2568;	True;True;	2586;2587;	15956;159	8439;8440;	8439;8440;

801	7948;8375	True;True	8011;8438	49102;4910	26000;2720	26000;2726
1361	1969;2787;	True;True;T	1985;2808;	12208;1220	6468;6469;	6473;9164;
1503	2288;2558;	True;True;T	2304;2577;	14183;1418	7539;7540;	7544;8395;
1076	410;3000;6	True;True;T	411;3024;6	2491;2492;	1387;1388;	1387;9840;
601	8538	TRUE	8602	52624;5262	27770;2777	27772
636	1533;3815	True;True	1546;3841	9435;9436;	5087;5088;	5089;12554
1854	6124;6222;	True;True;T	6167;6265;	37978;3797	20176;2017	20177;2050
554	8506;9048	True;True	8570;9116	52440;5244	27679;2940	27679;2946
373	7484	TRUE	7545	46243;4624	24513	24513
234	354;2823;4	True;True;T	355;2844;4	2132;2133;	1187;1188;	1191;9279;
1378	3407;7745;	True;True;T	3432;7807;	21120;2112	11157;1115	11159;2535
1375	6918	TRUE	6968	42797;4279	22727;2272	22728
160	1245	TRUE	1254	7666;7667;	4132	4132
180	9356	TRUE	9427	57713;5771	30494;3049	30495
1870	8238	TRUE	8301	50873;5087	26905;2690	26907
75	588;2600;5	True;True;T	590;2619;5	3533;3534;	1936;1937;	1939;8549;
434	6406;1122	True;True;T	6452;1130	39657;3965	21048;2104	21048;3701
261	38;39;2898	True;True;T	38;39;2922	200;201;20	97;98;99;1	99;103;951
1536	1787;4379;	True;True;T	1801;4408;	11004;1100	5853;5854;	5853;14346
806	1602;1022	True;True;T	1615;1030	9830;9831;	5300;3363	5300;3363
898	3036;3065;	True;True;T	3060;3089;	18916;1891	9972;1004	9972;1004
237	2243;4090;	True;True;T	2259;4117;	13943;1394	7398;1339	7398;1339
448	1984;2033;	True;True;T	2000;2049;	12327;1260	6533;6701;	6533;6701;
804	3758;7547	True;True	3784;7608	23314;2331	12357;2469	12357;2469
1692	7006	TRUE	7060	43310;4331	22999	22999
624	1905	TRUE	1920	11766;1170	6243;6244	6243
1471	1567;1568;	True;True;T	1580;1581;	9620;9621;	5187;5188;	5187;5190;
1700	202;2468;4	False;False;	202;2486;4	1202;1203;	663;664;66	669;8112;1
1699	2213;2350;	False;False;	2229;2366;	13744;1374	7298;7299;	7299;7738;
1702	2328;5143;	True;True;T	2344;5180;	14433;1443	7670;1704	7670;17045
1714	4022;8302	True;True	4048;8365	24905;2490	13157;1315	13157;2705
700	585	TRUE	587	3515;3516;	1928;1929;	1930
33	2851;1045	True;True	2872;1053	17779;6485	9371;3454	9371;34545
102	5535;7566;	True;True;T	5575;7627;	34327;3432	18310;2474	18310;2474
1716	6235	TRUE	6278	38681;3868	20535;2053	20535
184	6914	TRUE	6964	42777;4277	22723	22723
14	4732;8874	True;True	4762;8941	29122;2912	15516;1551	15519;2892
1011	171;233;57	True;True;T	171;233;57	1023;1024;	569;570;57	570;774;19
967	2431;5746;	True;True;T	2448;5788;	15092;1509	7985;7986;	7986;19000
1006	1515;2383;	True;True;T	1527;2400;	9312;9313;	5024;7863;	5024;7866;
736	9250	TRUE	9320	57011;5701	30118;3011	30119
1037	273;274;41	True;True;T	273;274;27	1656;1657;	919;920;92	929;934;14
1038	273;274;41	False;False;	273;274;27	1656;1657;	919;920;92	929;934;14
1309	1722;2444;	True;True;T	1735;2461;	10572;1057	5643;5644;	5643;8025;
1977	9617;9862;	True;True;T	9691;9939;	59333;5933	31440;3230	31440;3236
1727	5394;6378;	True;True;T	5432;6424;	33403;3340	17913;1791	17913;2094
120	4087	TRUE	4114	25334;2533	13388	13388
1265	350;2417;2	True;True;T	351;2434;2	2101;2102;	1169;7950;	1169;7950;
1257	1944;3181;	True;True;T	1960;3205;	12049;1205	6393;6394;	6394;10398
1579	1507;2101;	True;True;T	1519;2117;	9266;1304	5000;6953;	5000;6955;

1272	610;661;99	True;True;	613;665;10	3677;3678;	2006;2007;	2007;2158;
1654	4326;6711	True;True	4353;6759	26677;415	14153;220	14153;220
1458	1086;1371;	True;True;	1093;1381;	6651;6652;	3580;3581;	3581;4532;
1046	1087;1371;	True;False;	1094;1381;	6659;6660;	3585;3586;	3586;4532;
2011	7088;1028	True;True	7145;1036	43792;437	23219;338	23219;338
1391	3044;5461;	True;True;	3068;5500;	18950;189	9986;9987;	9988;1809
1670	537;843;34	True;True;	539;850;34	3229;3230;	1754;2746;	1754;2748;
1395	199;2016;3	True;True;	199;2032;3	1187;1188;	657;6642;6	657;6643;1
2019	3325;4776;	True;True;	3350;4808;	20633;206	10884;108	10884;156
1441	2281;2933;	True;True;	2297;2957;	14140;141	7508;7509;	7513;9631;
1979	2281;2933;	False;False	2297;2957;	14140;141	7508;7509;	7513;9631;
2098	13;4425;69	True;True;	13;4454;70	82;83;84;8	34;35;36;1	34;14508;2
1373	537;11085	False;True	539;11170	3229;3230;	1754;3658	1754;3659
604	3152	TRUE	3176	19590;195	10309;103	10309
1390	1142;2397;	True;True;	1149;2414;	6980;6981;	3746;3747;	3746;7897;
1593	401;1007;1	True;True;	402;1014;1	2422;2423;	1349;1350;	1349;3320;
483	2944;1120	True;True	2968;1128	18356;183	9659;3695	9659;3695
1777	1922	TRUE	1937	11884;118	6300	6300
1317	3026;4120;	True;True;	3050;4147;	18859;188	9936;9937;	9936;1351
1089	6463;6742;	True;True;	6509;6790;	40000;400	21215;212	21218;221
1318	817;1006;1	True;True;	824;1013;1	4975;4976;	2672;2673;	2672;3316;
765	1207;6462;	True;False;	1216;6508;	7396;7397;	3983;2121	3983;2121
1388	6183;7315;	False;True;	6226;7374;	38339;383	20361;203	20361;239
1319	1432;8221;	True;True;	1443;8284;	8771;8772;	4755;2684	4755;2684
635	312;8222;1	True;True;	313;8285;1	1891;1892;	1043;1044;	1044;2685
1401	4940;6818;	True;True;	4977;6866;	30498;421	16295;224	16295;224
1402	563;962;11	True;True;	565;969;11	3400;3401;	1862;1863;	1865;3120;
992	5681;6331;	False;True;	5723;6377;	35233;352	18783;187	18783;208
1415	5681;6823;	True;True;	5723;6871;	35233;352	18783;187	18783;224
1380	7793	TRUE	7855	48091;480	25489;254	25490
1500	4252;5276;	True;True;	4279;5313;	26339;263	13954;139	13956;175
1352	4586;1114	True;True	4616;1123	28218;282	15047;367	15047;367
995	149;1608;3	True;True;	149;1621;3	903;904;90	510;5320;5	510;5324;1
1942	70;3555;89	True;True;	70;3580;89	405;406;40	205;206;20	207;11694;
1078	1963;5939;	True;True;	1979;5982;	12174;121	6449;6450;	6450;1966
1880	311;2752;7	True;True;	312;2772;7	1883;1884;	1040;1041;	1041;9047;
597	1105	TRUE	1112	6775;6776;	3643;3644	3644
1862	1572	TRUE	1585	9647;9648;	5206	5206
1827	4278;8035	True;True	4305;8098	26462;264	14022;140	14023;262
1203	2365;4757;	True;True;	2382;4788;	14718;147	7805;7806;	7807;1561
1240	2798;7439;	True;True;	2819;7499;	17450;174	9195;9196;	9195;2434
311	5044;8836	True;True	5081;8903	31181;311	16696;287	16696;287
1239	10982;115	True;True	11067;116	68206;682	36264;381	36264;381
244	2214;4752;	True;True;	2230;4783;	13752;137	7306;1559	7306;1559
1054	151;4526	True;True	151;4556	908;909;91	512;14849	512;14849
1138	973;8681	True;True	980;8748	5927;5928;	3165;3166;	3165;2827
1649	4339;4433;	True;True;	4366;4462;	26749;267	14196;145	14196;145
593	2122;1055	True;True;	2138;1063	13183;131	7031;7032;	7031;3485
2022	3943;4136;	True;True;	3969;4163;	24437;244	12907;129	12911;135
939	1080	TRUE	1087	6627;6628;	3568;3569;	3572

1811	7498;9811	True;True	7559;9888	46322;463[illegible]	24550;321[illegible]	24550;321[illegible]
1787	2660;9256;	True;True;[illegible]	2680;9326;	16561;165[illegible]	8751;8752;	8752;3013[illegible]
1816	2442;4036;	True;True;[illegible]	2459;4062;	15157;151[illegible]	8017;8018;	8023;1320[illegible]
2012	330;10076	True;True	331;10154	1993;1994;	1105;3307[illegible]	1105;3307[illegible]
662	264;8773;8	True;True;[illegible]	264;8840;8	1597;1598;	894;895;89	898;28552;
487	756;9739	True;True	763;9814	4600;4601;	2462;2463;	2463;3190[illegible]
1535	3177;4372;	True;True;[illegible]	3201;4401;	19755;197[illegible]	10391;143[illegible]	10391;143[illegible]
1632	8455	TRUE	8519	52103	27501	27501
962	2864;4256;	True;True;[illegible]	2887;4283;	17880;178[illegible]	9429;9430;	9432;1397[illegible]
1493	1371;2691;	False;True;	1381;2711;	8387;8388;	4532;4533;	4532;8838;
1026	2489;2588;	True;True;[illegible]	2507;2607;	15463;154[illegible]	8176;8177;	8178;8523;
1115	2690;6322;	True;True;[illegible]	2710;6368;	16749;391[illegible]	8837;2077[illegible]	8837;2077[illegible]
170	21;1480;33	True;True;[illegible]	21;1491;33	124;125;12	53;54;55;4[illegible]	53;4918;10
1384	3305;7118;	True;False;	3330;7177;	20494;204[illegible]	10815;108[illegible]	10816;233[illegible]
1014	3306;7118;	True;True;[illegible]	3331;7177;	20501;205[illegible]	10820;108[illegible]	10826;233[illegible]
2144	6128	TRUE	6171	38007;380[illegible]	20186	20186
857	45;1013;14	True;True;[illegible]	45;1020;14	256;257;25	135;136;33	135;3330;4
669	5490;5605;	True;True;[illegible]	5530;5645;	34042;340[illegible]	18164;185[illegible]	18164;185[illegible]
1367	239;301;55	True;True;[illegible]	239;302;55	1447;1829;	804;1014;1	804;1015;1
1865	87;7644;10	True;True;[illegible]	87;7706;11	509;510;51	275;25031;	275;25034;
2130	6227	TRUE	6270	38624;386[illegible]	20516;205[illegible]	20518
1620	265;423;44	True;True;[illegible]	265;424;44	1605;2577;	900;1445;1	900;1445;1
1606	1756;5569;	True;True;[illegible]	1769;5609;	10763;107[illegible]	5745;5746;	5748;1841[illegible]
1822	3203;3369;	True;True;[illegible]	3228;3394;	19894;198[illegible]	10456;110[illegible]	10456;110[illegible]
71	7771;9857;	True;True;[illegible]	7833;9934;	47973;479[illegible]	25425;254[illegible]	25425;323[illegible]
1974	3686;5230;	True;True;[illegible]	3712;5267;	22878;228[illegible]	12134;173[illegible]	12134;173[illegible]
1639	1182;3271	True;True	1191;3296	7250;7251;	3886;3887;	3889;1070[illegible]
49	6675;8747	True;True	6722;8814	41317;413[illegible]	21940;219[illegible]	21941;284[illegible]
1722	3662;4601;	True;True;[illegible]	3688;4631;	22750;283[illegible]	12054;150[illegible]	12054;150[illegible]
80	830;1415;1	True;True;[illegible]	837;1426;1	5046;5047;	2708;4705;	2708;4706;
1883	2761;4503;	True;True;[illegible]	2781;4533;	17179;171[illegible]	9073;9074;	9077;1478[illegible]
369	7327	TRUE	7386	45264;452[illegible]	23986	23986
1227	591;1930;1	True;True;[illegible]	593;1946;1	3549;3550;	1945;1946;	1948;6332;
1906	857;858;96	True;True;[illegible]	864;865;97	5217;5218;	2795;2796;	2799;2800;
1907	977;4656;9	True;False;	984;4686;9	5967;5968;	3189;3190;	3191;1526[illegible]
1300	545;2922;5	True;True;[illegible]	547;2946;5	3265;3266;	1772;1773;	1773;9586;
2069	7000	TRUE	7054	43280	22989	22989
2046	321;1282;2	True;True;[illegible]	322;1291;2	1939;1940;	1075;1076;	1075;4239;
176	4814;5006	True;True	4847;5043	29697;296[illegible]	15844;158[illegible]	15844;165[illegible]
141	1703;3715;	True;True;[illegible]	1716;3741;	10474;104[illegible]	5602;5603;	5603;1221[illegible]
2153	2941;3604;	True;True;[illegible]	2965;3630;	18333;183[illegible]	9642;9643;	9642;1184[illegible]
202	114;115;38	True;True;[illegible]	114;115;38	670;671;67	367;368;36	368;370;12
611	63;771;131	False;True;	63;778;132	363;364;36	184;185;25	184;2536;4
619	5624	TRUE	5664	34874;348[illegible]	18614	18614
2029	3332;7820	True;True	3357;7882	20676;206[illegible]	10909;256[illegible]	10909;256[illegible]
1707	5794;6415;	True;True;[illegible]	5836;6461;	35916;359[illegible]	19147;191[illegible]	19150;210[illegible]
602	1604;4189	True;True	1617;4216	9837;9838;	5302;5303;	5302;1375[illegible]
1720	5566;8893	True;True	5606;8960	34509;345[illegible]	18403;184[illegible]	18405;289[illegible]
713	724;725;70	True;True;[illegible]	731;732;71	4405;4406;	2367;2368;	2369;2375;
1728	6181	TRUE	6224	38327;383[illegible]	20357	20357

181	9800	TRUE	9877	60591;6059	32149;3215	32153
535	4107	TRUE	4134	25461;2546	13456;1345	13457
1704	699;9729;1	True;True;T	703;9804;1	4237;4238;	2276;31873	2276;31873
2134	3874;4170;	True;True;T	3900;4197;	24040;2580	12714;1370	12714;1370
2112	612;869;10	True;True;T	615;876;11	3689;3690;	2011;2012;	2012;2842;
2111	60;609;109	True;True;T	60;612;110	353;354;35	179;180;20	179;2005;3
1181	3366;4510;	True;True;T	3391;4540;	20879;2088	11012;1101	11016;1480
509	3080;5165	True;True	3104;5202	19173;1917	10108;1010	10109;1711
1062	707;721;13	True;True;T	713;728;13	4303;4304;	2322;2323;	2323;2363;
1655	217;500;13	True;True;T	217;501;13	1321;1322;	726;727;72	728;1671;4
1607	217;1084;1	False;True;	217;1091;1	1321;1322;	726;727;72	728;3578;4
258	2763	TRUE	2783	17190;1719	9082;9083;	9082
1542	5644	TRUE	5685	35010;3501	18667	18667
1833	670;3093;6	True;True;T	674;3117;6	4061;4062;	2179;10145	2179;10145
1551	514;9159;1	True;True;T	515;9228;1	3115;3116;	1706;1707;	1708;29824
46	7661	TRUE	7723	47325;4733	25078;2507	25078
769	9562	TRUE	9635	58965;5896	31246;3124	31252
760	860;7688;8	True;True;T	867;7750;8	5237;5238;	2806;2807;	2806;25165
1868	870	TRUE	877	5299	2846	2846
1204	10105	TRUE	10185	62569;6257	33203	33203
215	9215	TRUE	9285	56805;5680	30037	30037
1589	384;1341;1	True;True;T	385;1351;1	2328;2329;	1288;1289;	1290;4450;
1491	2899;4641	True;True	2923;4671	18064;1806	9516;9517;	9517;15226
1198	67;351;695	True;True;T	67;352;699	381;382;38	195;196;11	196;1173;2
2031	4166;4959;	True;True;T	4193;4996;	25838;2583	13685;1368	13686;1636
777	1606;2461;	True;True;T	1619;2479;	9843;9844;	5305;5306;	5307;8094;
778	2180;8618	True;True	2196;8684	13552;1355	7201;7202;	7203;28070
199	6870	TRUE	6918	42515	22592	22592
2108	51;2652;77	True;True;T	51;2672;78	291;292;29	142;8724;2	142;8724;2
670	1394;1589;	True;True;T	1404;1602;	8537;8538;	4610;5257;	4610;5258;
840	1392;7973	True;True	1402;8036	8523;8524;	4601;26054	4601;26054
1524	628	TRUE	632	3800;3801;	2063	2063
654	1393;9330;	True;True;T	1403;9400;	8529;8530;	4602;4603;	4608;30396
61	341;1896;7	True;True;F	342;1911;7	2048;2049;	1132;1133;	1133;6226;
1576	1385;6075;	True;False;	1395;6118;	8469;8470;	4574;4575;	4579;20024
1577	1386;6075;	True;True;T	1396;6118;	8485;8486;	4585;20023	4585;20024
104	355;1373;7	True;True;T	356;1383;7	2140;2141;	1195;1196;	1197;4535;
1575	1391;1594;	True;True;T	1401;1607;	8516;8517;	4597;4598;	4600;5280;
235	5517;9057	True;True	5557;9125	34225;3422	18266;1826	18267;2949
412	1876;10818	True;True	1891;1090	11600;1160	6175;6176;	6176;35746
296	8713	TRUE	8780	53703;5370	28371	28371
1571	4074;4778;	True;True;T	4101;4810;	25258;2525	13352;1335	13353;1569
588	2419	TRUE	2436	15031;1503	7953	7953
1462	5836;7146	True;True	5879;7205	36180;3618	19299;1930	19299;2339
1169	5546;6298;	True;True;T	5586;6344;	34400;3440	18345;1834	18345;2071
192	6724;9593;	True;True;T	6772;9666;	41612;4161	22089;2209	22090;3137
1467	1250;2493;	True;True;T	1259;2511;	7689;7690;	4142;4143;	4144;8194;
1893	396;506;76	True;True;T	397;507;76	2393;2394;	1330;1331;	1331;1684;
1417	483;3890;4	False;False	484;3916;4	2907;2908;	1609;1610;	1610;12770
1418	483;3890;4	True;True;T	484;3916;4	2907;2908;	1609;1610;	1610;12770

409	3854;5841	True;True	3880;5884	23911;2391	12663;1931	12663;1931
618	2433;4628;	True;True;	2450;4658;	15115;2847	7995;1518	7995;1518
507	197;483;38	True;False;	197;484;39	1174;1175;	648;1609;1	648;1610;1
2137	3309;7933;	True;True;	3334;7996;	20525;2052	10834;2596	10834;2596
1296	2602;6177;	True;True;	2621;6220;	16184;1618	8561;8562;	8564;20347
710	5124;6376	True;True	5161;6422	31702;3170	16989;2093	16989;2093
69	3197;4938;	True;True;	3222;4975;	19870;1987	10449;1045	10450;1628
1221	144;1767;1	True;True;	144;1780;1	877;878;87	493;494;49	495;5773;6
2	3909;4459;	True;True;	3935;4488;	24233;2423	12817;1281	12817;1460
1312	1098;1099;	True;True;	1105;1106;	6738;6739;	3621;3622;	3623;3626;
1960	2771;3385;	True;True;	2791;3410;	17247;1724	9113;1108	9113;1108
136	5885	TRUE	5928	36446;3644	19451;1945	19452
628	9300	TRUE	9370	57311;5731	30263	30263
1713	9093;1115	True;True	9161;1124	56023;5602	29602;2960	29602;3683
2027	7752;1050	True;True	7814;1059	47881;4788	25373;2537	25373;3471
83	470;2579;6	True;True;	471;2598;7	2840;2841;	1575;1576;	1576;8478;
2004	5907;7340;	True;True;	5950;7399;	36602;3660	19546;2402	19546;2402
600	7030;1046	True;True	7085;1055	43441;4344	23060;3459	23060;3459
2162	7015;1120	True;True;	7070;1129	43373;4337	23028;2302	23030;3697
20	1851;5292;	True;True;	1865;5330;	11417;1141	6087;6088;	6087;17619
1389	7459;7627;	True;True;	7520;7689;	46086;4608	24423;2442	24425;2498
596	5113;5347;	True;True;	5150;5385;	31645;3164	16954;1777	16954;1777
42	7889;9577	True;True	7951;9650	48743;4874	25828;2582	25828;3130
1294	4385;6851;	True;True;	4414;6899;	27004;2700	14359;2253	14359;2254
2151	1957;2286;	True;True;	1973;2302;	12140;1214	6429;6430;	6435;7534;
2084	1745;3033;	True;True;	1758;3057;	10713;1071	5720;5721;	5721;9964;
859	630;9481;1	True;True;	634;9552;1	3812;3813;	2065;2066;	2066;30960
656	2135;2580;	True;True;	2151;2599;	13256;1325	7070;8479;	7070;8479;
1856	1501	TRUE	1513	9230;9231;	4989	4989
851	7755	TRUE	7817	47896;4789	25382;2538	25382
1522	4565;7848;	True;True;	4595;7910;	28079;2808	14966;2570	14966;2570
532	5233	TRUE	5270	32390;3239	17376;1737	17376
698	5959;9854	True;True	6002;9931	36937;3693	19702;1970	19702;3232
1429	1306;4122	True;True	1315;4149	8001;8002;	4321;4322;	4321;13526
1430	6543;7540	True;True	6589;7601	40555;4055	21489;2467	21489;2467
1431	4233;7480;	True;True;	4260;7541;	26222;2622	13882;1388	13886;2449
1432	3174;6027;	True;True;	3198;6070;	19732;1973	10384;1038	10385;1988
672	1262;3559;	True;True;	1271;3584;	7746;7747;	4184;4185;	4185;11708
848	3158;5505;	True;True;	3182;5545;	19635;1963	10333;1822	10333;1822
260	7959;8491;	True;True;	8022;8555;	49167;4916	26032;2761	26032;2761
1410	9511;1018	True;True	9582;1026	58646;5864	31037;3103	31037;3345
541	6136	TRUE	6179	38057	20209	20209
926	118;155;19	True;True;	118;155;19	694;695;69	380;381;38	381;525;62
927	1149;1462;	True;True;	1156;1473;	7016;7017;	3768;3769;	3768;4847;
1350	4537;7804;	True;True;	4567;7866;	27915;2791	14879;2554	14879;2555
505	2802;5906	True;True	2823;5949	17473;1747	9206;9207;	9206;19545
1908	8734;9185;	True;True;	8801;9254;	53822;5382	28436;2843	28438;2991
1831	658;3915;5	True;True;	662;3941;5	3985;3986;	2149;2150;	2149;12833
627	5899	TRUE	5942	36532;3653	19497;1949	19497
547	3939;7787	True;True	3965;7849	24414;2441	12897;2547	12897;2547

1004	4065;7937;	True;True;1	4091;8000;	25195;2519	13327;1333	13327;2597
2025	8147	TRUE	8210	50295;5029	26602	26602
1501	4;704;4344	True;True;1	4;708;4371	27;28;29;3	14;2291;22	14;2291;14
274	1289;1791;	True;True;1	1298;1805;	7893;1102	4262;5861;	4262;5862;
2146	5779;1099	True;True	5821;1108	35831;3583	19104;3633	19104;3632
2106	3495;8815	True;True	3520;8882	21714;2171	11488;1148	11488;2868
812	3490;6069	True;True	3515;6112	21685;2168	11480;1148	11481;1999
2037	290;842;19	True;True;1	291;849;19	1756;1757;	977;978;97	977;2745;6
1957	248;837	True;True	248;844	1487;1488;	829;2734;2	829;2735
124	230;1347;1	True;True;1	230;1357;1	1391;1392;	759;760;44	760;4469;4
27	1695;2159;	True;True;1	1708;2175;	10403;1040	5577;5578;	5578;7129;
391	5314;7217;	True;True;1	5352;7276;	32913;3291	17677;1767	17679;2362
623	929;2985	True;True	936;3009	5643;5644;	3017;9782	3017;9782
563	649;923	True;True	653;930	3926;3927;	2123;2994	2123;2994
1599	540;2476;3	True;True;1	542;2494;3	3243;3244;	1762;1763;	1763;8136;
1659	7942;7943;	True;True;1	8005;8006;	49072;4907	25991;2599	25991;2599
1656	932;6625;9	True;True;1	939;6671;9	5659;5660;	3029;3030;	3029;21740
1556	2647;8839;	True;True;1	2667;8906;	16494;1649	8717;8718;	8718;28764
843	1955;3639;	True;True;1	1971;3665;	12128;1213	6427;1196	6427;11961
1585	117;990;50	True;True;1	117;997;51	686;687;68	379;3241;3	379;3242;1
1653	2037;3086;	True;True;1	2053;3110;	12642;1264	6719;1012	6719;10123
2132	5121;7148	True;True	5158;7207	31684;3168	16976;1697	16979;2340
991	7506;8241;	True;True;1	7567;8304;	46360;4636	24565;2456	24565;2691
701	713;2551;5	True;True;1	720;2570;5	4356;1586	2346;8379;	2346;8380;
1108	284;1127;1	True;True;1	285;1134;1	1728;6900;	958;959;37	958;3711;4
1834	797;4710;1	True;True;1	804;4740;1	4850;4851;	2594;2595;	2595;15458
459	737;1278;2	True;True;1	744;1287;2	4491;4492;	2399;4231;	2399;4231;
503	8348	TRUE	8411	51440	27169	27169
24	3813;7174;	True;True;1	3839;7234;	23652;2365	12550;2349	12550;2349
1612	731;888;88	True;True;1	738;895;89	4455;4456;	2382;2895;	2382;2902;
1749	163;1952;2	True;True;1	163;1968;2	974;975;97	544;545;54	546;6419;6
1063	922;2768;2	True;True;1	929;2788;2	5598;5599;	2992;2993;	2992;9103;
385	596	TRUE	599	3585;3586;	1971;1972;	1971
1706	3287;9521	True;True	3312;9593	20378;2037	10746;1074	10746;3107
58	3707	TRUE	3733	22999;2300	12193;1219	12193
1664	708;1092;1	True;True;1	714;1099;1	4311;4312;	2324;2325;	2326;3603;
40	309;802;96	True;True;1	310;809;97	1869;1870;	1034;1035;	1036;2603;
1839	5946	TRUE	5989	36864;3686	19673;1967	19675
1229	544;711;83	True;True;1	546;717;71	3259;3260;	1768;1769;	1771;2342;
1190	15;583;669	True;True;1	15;585;673	92;93;94;9	39;40;41;1	40;1926;21
2015	702;9558	True;True	706;9631	4253;4254;	2284;2285;	2285;31234
458	11258	TRUE	11343	69915;6991	37121;3712	37121
870	595;2645;3	True;True;1	598;2665;3	3577;3578;	1969;1970;	1970;8711;
2110	1751;2266;	True;True;1	1764;2282;	10743;1074	5731;7460;	5731;7460;
556	361;6289;6	True;True;1	362;6335;6	2171;2172;	1206;2069	1206;20699
424	1817;1947;	True;True;1	1831;1963;	11210;1121	5967;6402;	5967;6402;
1180	52;132;354	True;True;1	52;132;356	297;298;29	143;144;14	143;431;11
1340	4735;6039;	True;True;1	4765;6082;	29140;3740	15527;1990	15527;1990
2070	4519;4800;	True;True;1	4549;4833;	27810;2781	14822;1482	14827;1579
1895	3056;4868;	True;True;1	3080;4901;	19022;1902	10019;1605	10019;1605

1363	394;435;38	True;True;	395;436;39	2384;2385;	1324;1325;	1326;1477;
198	3179;6390;	True;True;	3203;6436;	19763;197	10393;103	10394;209
2166	303;2358;4	True;True;	304;2375;4	1840;1841;	1018;1019;	1020;7785;
219	3852	TRUE	3878	23901;239	12655	12655
203	4094;7488;	True;True;	4121;7549;	25375;253	13407;134	13409;245
438	9563	TRUE	9636	58981;589	31253;312	31255
1251	1975;8775	True;True	1991;8842	12263;122	6504;6505;	6504;2855
897	3498;4418;	True;True;	3523;4447;	21734;217	11503;115	11505;144
918	542;1508;3	True;True;	544;1520;3	3250;3251;	1765;1766;	1766;5001;
2124	607;5778;7	True;True;	610;5820;7	3659;3660;	1999;2000;	2000;1910
1941	5315	TRUE	5353	32919	17680	17680
101	6364;6420;	True;True;	6410;6466;	39428;394	20898;208	20898;211
1657	10616	TRUE	10701	65891;658	35089;350	35092
776	3993;8929	True;True	4019;8996	24750;247	13062;130	13062;290
847	5261;9890;	True;True;	5298;9967;	32562;325	17485;174	17485;324
852	1752;1051	True;True	1765;1060	10747;652	5732;3476	5732;3476
506	210;1631;1	True;True;	210;1644;1	1273;1274;	704;705;53	704;5372;3
807	1624;2755;	True;True;	1637;2775;	9943;9944;	5356;5357;	5357;9061;
265	269;4783	True;True	269;4815	1629;1630;	911;912;15	912;15719
225	4449;6059	True;True	4478;6102	27376;375	14582;199	14582;199
392	6428;9120	True;True	6474;9189	39790;397	21119;296	21119;296
1915	139;1293;2	True;True;	139;1302;2	842;843;84	469;470;47	474;4268;9
785	10175;108	True;True	10255;109	63007;630	33439;358	33439;358
1674	2822	TRUE	2843	17587;175	9266;9267;	9268
2003	109	TRUE	109	642;643;64	348;349;35	348
879	9334	TRUE	9404	57568;575	30411	30411
610	1779;4582	True;True	1793;4612	10952;109	5841;5842;	5843;1503
1079	2323;7533	True;True	2339;7594	14396;143	7660;7661;	7661;2464
1182	447;5100	True;True	448;5137	2717;2718;	1510;1511;	1510;1690
797	1102;1124	True;True	1109;1132	6764;6983	3639;3707	3639;3707
782	2287;9747	True;True	2303;9823	14175;141	7535;7536;	7535;3194
741	744;1926;3	True;True;	751;1941;3	4530;4531;	2421;2422;	2422;6309;
742	743;1926;3	True;False;	750;1941;3	4523;4524;	2416;2417;	2417;6309;
2142	135;159;58	True;True;	135;159;58	812;813;94	444;535;19	444;535;19
89	10942;111	True;True	11027;112	67954;679	36139;368	36139;368
1567	3074;3328;	True;True;	3098;3353;	19137;191	10088;100	10094;108
1857	63;3525;75	True;True;	63;3550;76	363;364;36	184;185;11	184;11596;
259	2581;3850;	True;True;	2600;3876;	16056;160	8480;8481;	8481;1265
762	10922	TRUE	11007	67808;678	36055;360	36062
1815	4562;7227;	True;True;	4592;7286;	28061;280	14955;236	14955;236
1966	6429	TRUE	6475	39792;397	21120	21120
1073	342;1460;2	True;True;	343;1471;2	2056;2057;	1135;4841;	1135;4843;
1479	62;942;142	True;True;	62;949;143	361;362;57	182;183;30	183;3067;4
1315	624;1157;1	True;True;	628;1165;1	3775;3776;	2049;2050;	2057;3793;
397	2619;2801;	True;True;	2639;2822;	16301;163	8625;9204;	8625;9204;
1927	1009;1183;	True;True;	1016;1192;	6196;6197;	3323;3893;	3323;3894;
1292	578;717;10	True;True;	580;724;10	3481;3482;	1911;1912;	1913;2355;
1314	490;763;10	True;True;	491;770;10	2952;2953;	1632;1633;	1634;2496;
1236	655;1569;2	True;True;	659;1582;2	3971;3972;	2144;2145;	2144;5192;
1721	3787;5166;	True;True;	3813;5203;	23492;234	12441;124	12441;171

1723	898;6349	True;True	905;6395	5457;5458;	2926;2086	2926;2086
2082	7650;9919	True;True	7712;9996	47259;472	25045;325	25045;325
455	1601;1010	True;True;	1614;1018	9824;9825;	5298;5299;	5298;3319
504	7545;8118;	True;True;	7606;8181;	46613;466	24687;246	24688;265
188	7471;7927;	True;True;	7532;7990;	46167;461	24473;244	24476;259
189	513;5295	True;True	514;5333	3114;3279	1705;1762	1705;1762
815	10357	TRUE	10440	64236;642	34205;342	34205
886	8474	TRUE	8538	52215;522	27560;275	27560
1334	11173;116	True;True	11258;117	69391;693	36870;384	36870;384
996	1296;2462;	True;True;	1305;2480;	7928;7929;	4278;4279;	4279;8099;
887	4838;1053	True;True	4871;1061	29851;298	15946;159	15946;348
1939	9308;9658;	True;True;	9378;9733;	57361;573	30281;302	30284;315
1803	571;2928;4	True;True;	573;2952;4	3444;3445;	1891;1892;	1894;9611;
10	3586;6260;	True;True;	3612;6303;	22294;222	11796;205	11796;205
1162	352;1763;2	True;True;	353;1776;2	2117;2118;	1177;1178;	1179;5763;
790	372;4555;4	True;True;	373;4585;4	2233;2234;	1237;1493	1237;1493
1987	1512;6220;	True;True;	1524;6263;	9290;9291;	5007;2049	5007;2049
2126	379;2194;2	True;True;	380;2210;2	2290;2291;	1265;1266;	1265;7244;
1682	10124;106	True;True	10204;107	62693;626	33270;332	33271;351
111	5691	TRUE	5733	35291;352	18811	18811
1772	195;6916;7	True;True;	195;6966;7	1166;1167;	645;646;22	646;22725;
1726	6956	TRUE	7008	43033	22846	22846
1131	276;289;51	True;True;	277;290;51	1685;1686;	938;939;94	940;976;17
961	1796;3331;	True;True;	1810;3356;	11055;110	5875;1090	5875;1090
608	3275	TRUE	3300	20312;203	10714;107	10715
708	7604	TRUE	7666	46977;469	24901	24901
1433	8477;8478	True;True	8541;8542	52238;522	27578;275	27579;275
2122	894;903;22	True;True;	901;910;22	5438;5485;	2911;2940;	2911;2940;
1661	1143;1155;	True;True;	1150;1163;	6987;6988;	3749;3750;	3750;3791;
150	310;337;72	True;True;	311;338;73	1876;1877;	1037;1038;	1038;1118;
846	10155	TRUE	10235	62884;628	33373	33373
1776	2968;4647;	True;True;	2992;4677;	18501;185	9723;9724;	9724;1524
1030	1239;1240;	True;True;	1248;1249;	7626;7627;	4112;4113;	4113;4124;
73	11176	TRUE	11261	69407;694	36876	36876
82	1039;6414;	True;True;	1046;6460;	6361;6362;	3409;3410;	3412;2107
460	3976	TRUE	4002	24653;246	13019	13019
652	1287;6476;	True;True;	1296;6522;	7877;7878;	4255;4256;	4255;2127
659	10102	TRUE	10182	62553;625	33196	33196
1225	89;566;482	True;True;	89;568;486	524;525;52	277;278;18	278;1878;1
93	3781	TRUE	3807	23456;234	12428;124	12430
162	2273;7143;	True;True;	2289;7202;	14098;140	7486;2339	7486;2339
386	709;2438;1	True;True;	715;2455;1	4319;4320;	2328;2329;	2330;8008;
262	5187;7770;	True;True;	5224;7832;	32086;320	17192;254	17192;254
758	3948;1149	True;True	3974;1158	24467;244	12922;379	12922;379
1496	2708;3148;	True;True;	2728;3172;	16864;168	8887;1030	8887;1030
1088	5994	TRUE	6037	37144;371	19786	19786
1578	229;1609;1	True;True;	229;1622;1	1388;1389;	758;5325;5	758;5326;5
1297	1310;2349;	True;True;	1319;2365;	8026;8027;	4335;7733;	4335;7733;
143	657	TRUE	661	3977;3978;	2147;2148	2147
429	3348;6477;	True;True;	3373;6523;	20776;207	10957;109	10958;212

1552	512;844;20	True;True;	513;851;20	3107;3108;	1704;2753;	1704;2753;
910	7;357;1093	True;True;	7;358;1100	45;46;47;4	18;19;20;2	19;1200;36
1591	11389	TRUE	11474	70705;7070	37524;3752	37525
1457	91;4190;84	True;True;	91;4217;85	530;531;53	280;281;13	280;13753;
1658	2391;9247	True;True	2408;9317	14880;1488	7882;3010	7882;3010
1407	2864;4256;	False;False	2887;4283;	17880;1788	9429;9430;	9432;1397
1224	1454;1593;	True;True;	1465;1606;	8903;8904;	4821;4822;	4821;5276;
792	2084;8640	True;True	2100;8706	12912;1291	6875;2813	6875;2813
1357	493;910;16	True;True;	494;917;16	2981;2982;	1652;1653;	1653;2952;
1151	1067;1152;	True;True;	1074;1159;	6534;6535;	3503;3504;	3504;3775;
1851	561;1164;1	True;True;	563;1173;1	3388;3389;	1858;1859;	1858;3824;
1944	2335;3933	True;True	2351;3959	14483;1448	7687;7688;	7687;1288
106	1560;1728;	True;True;	1573;1741;	9579;9580;	5165;5166;	5165;5662;
548	2402;3137;	True;True;	2419;3161;	14941;149	7911;7912;	7912;1027
523	1051;2790	True;True	1058;2811	6441;6442;	3458;9173;	3458;9174
2083	1434;8091;	True;True;	1445;8154;	8783;8784;	4757;4758;	4759;2641
206	2523;3363	True;True	2542;3388	15692;156	8280;8281;	8280;1100
271	3201;3529;	True;True;	3226;3554;	19891;198	10454;116	10454;116
1321	7464;1088	True;True;	7525;1097	46119;461	24443;244	24447;359
286	5395;8125	True;True	5433;8188	33409;334	17915;265	17915;265
454	380;7460;8	True;True;	381;7521;8	2296;2297;	1267;1268;	1269;2442
1925	1384;2972;	True;True;	1394;2996;	8461;8462;	4567;4568;	4567;9737;
2017	3146;7423;	True;True;	3170;7483;	19556;195	10294;102	10297;242
108	295;1229;3	True;True;	296;1238;3	1786;1787;	985;4084;4	985;4085;9
684	2808;4844	True;True	2829;4877	17510;175	9223;1599	9223;1599
1587	5641	TRUE	5682	34994;349	18658;186	18661
539	4953;5131	True;True	4990;5168	30577;305	16333;163	16340;170
403	4250	TRUE	4277	26317;263	13937;139	13938
1764	1234;6696;	True;True;	1243;6743;	7592;7593;	4095;4096;	4097;2199
2131	2173;5735	True;True	2189;5777	13508;135	7187;1896	7187;1896
1955	1234;2280;	False;True;	1243;2296;	7592;7593;	4095;4096;	4097;7504;
1629	1175;2914;	True;True;	1184;2938;	7204;7205;	3864;3865;	3864;9566;
1953	2137;8262	True;True	2153;8325	13271;132	7073;7074;	7078;2697
2013	2211;7087;	True;True;	2227;7144;	13731;137	7287;2321	7287;2321
1785	6107;9683	True;True	6150;9758	37861;378	20115;316	20115;316
665	6892	TRUE	6940	42626;426	22637	22637
695	9980	TRUE	10057	61701;617	32735	32735
1730	587;3821;5	True;True;	589;3847;5	3527;3528;	1935;1257	1935;1257
703	5890	TRUE	5933	36481;364	19472;194	19473
368	631;6510	True;True	635;6556	3815;3816;	2067;2068;	2073;2138
1948	3048;9407;	True;True;	3072;9478;	18977;189	10001;306	10001;306
1961	2239;7278;	True;True;	2255;7337;	13912;139	7390;2384	7390;2384
1802	2943;5217;	True;True;	2967;5254;	18348;183	9654;9655;	9658;1732
214	742;5412	True;True	749;5450	4521;4522;	2414;2415;	2414;1795
1864	3285;3513;	True;True;	3310;3538;	20366;203	10738;107	10739;115
411	441;2073;3	True;True;	442;2089;3	2682;2683;	1488;1489;	1488;6840;
1337	3848;7617;	True;True;	3874;7679;	23873;238	12647;126	12648;249
1241	1270;1430;	True;True;	1279;1441;	7784;7785;	4205;4206;	4206;4749;
1358	78;700;822	True;True;	78;704;829	453;454;45	243;244;22	244;2277;2
1100	492;5990;8	True;True;	493;6033;8	2974;2975;	1650;1651;	1650;1977

	1376	1443;2487;	True;True;	1454;2505;	8846;8847;	4795;4796;	4798;8172;
	1605	8701;9053	True;True	8768;9121	53636;536	28339;283	28341;294
	1902	3932;5561	True;True	3958;5601	24369;243	12883;183	12883;183
	1543	1577;2386;	True;True;	1590;2403;	9683;9684;	5221;7872;	5221;7872;
	484	3084;6508;	True;True;	3108;6554;	19186;191	10115;101	10116;213
	2138	2701;3652;	True;True;	2721;3678;	16816;168	8867;1201	8867;1201
	2054	3111;7014;	True;True;	3135;7069;	19355;193	10198;230	10198;230
+	978	292;307;98	True;False;	293;308;99	1768;1769;	981;1031;3	981;1031;3
	944	414;415;12	True;True;	415;416;12	2519;2520;	1398;1399;	1400;1404;
	1469	324;1242;1	False;False	325;1251;1	1957;1958;	1086;1087;	1086;4128;
	1470	307;324;89	True;True;	308;325;90	1859;1860;	1031;1086;	1031;1086;
	1110	10;1632;17	True;True;	10;1645;18	67;68;9991	25;5373;53	25;5374;58
	1748	360;1048;1	False;False	361;1055;1	2170;6412;	1205;3441;	1205;3443;
	1472	360;1048;1	True;True;	361;1055;1	2170;6412;	1205;3441;	1205;3443;
	491	360;1049;1	False;True;	361;1056;1	2170;6426;	1205;3448;	1205;3450;
	1473	360;1048;1	False;False	361;1055;1	2170;6412;	1205;3441;	1205;3443;
	954	520;682;28	True;True;	521;522;68	3142;3143;	1717;1718;	1727;2213;
	1601	520;681;27	False;True;	521;522;68	3142;3143;	1717;1718;	1727;2210;
	105	520;679;32	False;False	521;522;68	3142;3143;	1717;1718;	1727;2206;
	1474	679;1101;2	True;True;	683;1108;2	4112;4113;	2205;2206;	2206;3636;
	1950	680;2452;3	True;True;	684;2470;3	4120;1522	2209;8060;	2209;8060;
	1690	1101;2088;	False;True;	1108;2104;	6756;6757;	3634;3635;	3636;6883;
	691	6855	TRUE	6903	42417;424	22552;225	22552
	389	4833;7041;	True;True;	4866;7096;	29828;298	15928;230	15928;230
	1610	1502;6584;	True;True;	1514;6630;	9235;9236;	4990;2160	4990;2160
	1516	10332	TRUE	10414	64088;640	34126	34126
	757	6243	TRUE	6286	38725;387	20547;205	20549
	1985	2292;9860	True;True	2308;9937	14203;142	7550;7551;	7550;3235
	1360	884;9939;1	True;True;	891;10016;	5372;5373;	2882;3261	2882;3261
	43	3825;5949;	True;True;	3851;5992;	23720;237	12581;125	12584;196
	1712	1928;9394;	True;True;	1944;9465;	11931;119	6319;6320;	6319;3059
	1557	5351	TRUE	5389	33133;331	17787	17787
	986	55;621;356	True;True;	55;624;625	317;318;31	152;153;15	153;2038;1
	1554	4920;8728	True;True	4957;8795	30373;303	16235;284	16235;284
	374	2806	TRUE	2827	17496;174	9217	9217
	1737	8422	TRUE	8486	51889;518	27411;274	27415
	2090	452;781;14	True;True;	453;788;14	2743;2744;	1523;2556;	1523;2556;
	959	8048;9795;	True;True;	8111;9872;	49654;496	26279;321	26279;321
	1074	2614;3136;	True;True;	2634;3160;	16269;162	8615;8616;	8616;1026
	1347	7414;7554;	True;True;	7474;7615;	45801;458	24273;247	24273;247
	587	1506;5938;	True;True;	1518;5981;	9259;9260;	4999;1965	4999;1965
	971	1838;3645;	True;True;	1852;3671;	11351;113	6047;6048;	6051;1199
	716	3979;8940	False;True	4005;9008	24668;246	13025;291	13025;291
	79	3393;1121	True;True	3418;1129	21023;210	11103;111	11103;369
	984	3671;3822;	True;True;	3697;3848;	22791;236	12078;125	12078;125
	969	3979;1004	True;True	4005;1012	24668;246	13025;329	13025;329
	768	3090;5419;	True;True;	3114;5458;	19229;192	10138;101	10138;179
	2040	8925;1049	True;True	8992;1058	54970;549	29049;346	29049;346
	743	11104	TRUE	11189	68932;689	36651	36651
	1954	1906;6131;	True;True;	1921;6174;	11773;117	6245;2019	6245;2019

2149	367;10152	True;True	368;10232	2198;2199;	1215;3336	1215;3336
2053	2506;2686	True;True	2525;2706	15574;155	8231;8232;	8232;8820
800	335;2439;5	True;True;	336;2456;5	2019;2020;	1115;1116;	1116;8009;
787	616	TRUE	619	3714;3715;	2023;2024;	2023
53	7510;9686	True;True	7571;9761	46385;463	24577;245	24577;317
845	69;738;136	True;True;	69;745;137	397;398;39	203;204;24	203;2401;4
1881	3389;4244;	True;True;	3414;4271;	21006;210	11094;139	11094;139
2127	11639	TRUE	11725	72297;722	38408;384	38409
297	3587	TRUE	3613	22299;223	11797	11797
2104	667;2460;3	True;True;	671;2478;3	4042;4043;	2170;2171;	2174;8091;
606	11605	TRUE	11691	72078;720	38290;382	38290
614	7321	TRUE	7380	45230;452	23975	23975
1263	2237;5197	True;True	2253;5234	13900;139	7382;1722	7382;1722
1049	3160;6980;	True;True;	3184;7033;	19649;196	10338;103	10339;229
185	847;915;13	True;True;	854;922;13	5160;5161;	2758;2759;	2758;2965;
569	2368;3775	True;True	2385;3801	14739;147	7819;1240	7819;1240
1860	3346;4761	True;True	3371;4792	20768;207	10952;109	10954;156
479	2283;3544;	True;True;	2299;3569;	14150;141	7518;1166	7518;1166
1456	1194;2176;	True;True;	1203;2192;	7312;7313;	3930;3931;	3930;7196;
514	1557;1584;	True;True;	1570;1597;	9573;9733;	5162;5238;	5162;5238;
747	3198;6557	True;True	3223;6603	19873;198	10451;215	10451;215
1393	3554;6826;	True;True;	3579;6874;	22105;221	11690;116	11692;224
152	4005	TRUE	4031	24826;248	13099;131	13099
1394	300;6105;9	True;True;	301;6148;9	1824;1825;	1013;2011	1013;2011
638	2470;6139;	True;True;	2488;6182;	15343;153	8114;2021	8114;2021
1667	1115;1116;	True;True;	1122;1123;	6828;6829;	3668;3669;	3668;3669;
568	10725	TRUE	10810	66600;666	35459;354	35462
1103	299;645;64	True;True;	300;649;65	1816;1817;	1006;1007;	1011;2111;
1968	6013;6987	True;True	6056;7040	37256;372	19833;229	19833;229
16	6345	TRUE	6391	39323;393	20849;208	20851
1848	6344;8501;	True;True;	6390;8565;	39307;393	20841;208	20844;276
817	2699;3298;	True;True;	2719;3323;	16800;168	8861;8862;	8861;1079
2055	735;2447;4	True;True;	742;2464;4	4483;4484;	2397;8035;	2397;8035;
1081	698;1665;8	True;True;	702;1678;8	4232;4233;	2275;5481;	2275;5481;
1166	2608;3173;	True;True;	2627;3197;	16218;162	8583;8584;	8583;1038
1799	10627	TRUE	10712	65962;659	35131;351	35132
1835	8058	TRUE	8121	49709;497	26309	26309
304	3526;8641	True;True	3551;8707	21936;219	11600;116	11605;281
425	1861;2705;	True;True;	1875;2725;	11483;114	6121;6122;	6121;8876;
493	1590;1860;	True;True;	1603;1874;	9766;9767;	5264;5265;	5269;6117;
1562	308;2648;1	True;True;	309;2668;1	1864;1865;	1032;1033;	1033;8719;
76	5184;1149	True;True	5221;1157	32070;320	17187;378	17187;378
1998	27	TRUE	27	157;158;15	71	71
543	7155	TRUE	7214	44196;441	23416	23416
582	6613	TRUE	6659	40935;409	21700;217	21704
1896	437;11355	True;True	438;11440	2653;2654;	1480;1481;	1480;3741
1005	7016;1094	True;True;	7071;1102	43381;679	23032;361	23032;361
232	1363;2045;	True;True;	1373;2061;	8344;8345;	4515;4516;	4515;6752;
527	10050	TRUE	10128	62165;621	32975;329	32975
154	3957	TRUE	3983	24523;245	12951	12951

873	6678	TRUE	6725	41337;413	21949;219	21952
44	3831;7255;	True;True;	3857;7314;	23757;237	12595;237	12595;237
1351	907;1072;2	True;True;	914;1079;2	5510;5511;	2949;3531;	2949;3531;
1689	72	TRUE	72	418;419;42	218;219;22	221
835	2450;2478;	True;True;	2468;2496;	15218;152	8056;8138;	8056;8138;
451	6947	TRUE	6998	42970;429	22813;228	22813
1909	4890;4976;	True;True;	4923;5013;	30153;301	16116;164	16116;164
829	3731;6152	True;True	3757;6195	23156;231	12276;202	12276;202
38	6834;6950	True;True	6882;7002	42288;429	22502;228	22502;228
147	668;3627;5	True;True;	672;3653;5	4049;4050;	2176;1192	2176;1192
1812	11150	TRUE	11235	69230;692	36794;367	36796
1299	419;606;94	True;True;	420;609;95	2556;2557;	1420;1421;	1422;1997;
1742	110	TRUE	110	650;651	353	353
2062	3290;5802;	True;True;	3315;5844;	20403;204	10766;107	10768;191
153	7107;9552;	True;True;	7165;9625;	43903;439	23274;232	23275;312
2056	4439;6396;	True;True;	4468;6442;	27336;396	14557;210	14557;210
875	10716	TRUE	10801	66530;665	35414;354	35417
1668	161;6479;6	True;True;	161;6525;6	958;959;96	538;539;54	541;21283;
1951	7342;7361;	True;True;	7401;7420;	45350;453	24030;240	24030;241
2064	1362;3930	True;False	1372;3956	8336;8337;	4509;4510;	4514;1287
868	3930;6442;	True;True;	3956;6488;	24354;243	12872;128	12872;211
639	7285	TRUE	7344	45001;450	23866;238	23866
279	10903	TRUE	10988	67682;676	35988;359	35988
833	1757;7388;	True;True;	1770;7447;	10770;107	5750;5751;	5752;2418
1560	1818;6713;	True;True;	1832;6761;	11214;112	5968;5969;	5970;2204
3	964;3901;4	True;True;	971;3927;4	5855;5856;	3122;1280	3122;1280
1075	389;867;12	True;True;	390;874;12	2350;5282;	1301;2836;	1301;2838;
315	4375;1085	True;True	4404;1093	26954;269	14329;143	14329;358
1788	575	TRUE	577	3466;3467;	1901;1902;	1904
1098	2400;4199;	True;True;	2417;4226;	14927;149	7901;7902;	7902;1377
1262	1004;3594;	True;True;	1011;3620;	6161;6162;	3303;3304;	3305;1181
2114	33;918;420	True;True;	33;925;422	176;177;17	82;2968;13	82;2968;13
1783	664;4975	True;True	668;5012	4024;4025;	2166;1642	2166;1642
1136	8705	TRUE	8772	53655;536	28348;283	28348
1228	1522;3887;	True;True;	1534;3913;	9351;9352;	5042;1275	5042;1275
250	1742	TRUE	1755	10689;106	5707;5708;	5709
1093	1130;4963;	True;True;	1137;5000;	6915;6916;	3717;3718;	3717;1637
540	3322	TRUE	3347	20604;206	10870;108	10871
480	465;6162;6	True;True;	466;6205;6	2816;2817;	1559;2029	1559;2029
1216	260;879;50	True;True;	260;886;50	1573;1574;	882;883;88	883;2865;1
828	950;2228;4	True;True;	957;2244;4	5779;5780;	3095;7343;	3095;7345;
525	5908;7901;	True;True;	5951;7963;	36606;366	19547;195	19548;258
824	1236;3975;	True;True;	1245;4001;	7607;7608;	4107;4108;	4109;1301
68	4444;5941;	True;True;	4473;5984;	27355;273	14570;196	14570;196
95	2762;7396;	True;True;	2782;7455;	17186;171	9079;9080;	9081;2420
1642	5422;8678;	True;True;	5461;8745;	33575;335	17981;179	17982;282
774	1046;4521;	True;True;	1053;4551;	6402;6403;	3437;1483	3437;1483
1411	1585	TRUE	1598	9736;9737;	5239;5240;	5245
598	2651	TRUE	2671	16516;165	8723	8723
470	6540;9083	True;True	6586;9151	40532;405	21482;295	21482;295

1781	1248;9172	True;True	1257;9241	7677;7678;	4136;2986(	4136;2986(
2073	1786	TRUE	1800	11001;1100	5851;5852	5852
1023	736;1238;1	True;True;T	743;1247;1	4490;7619;	2398;4111;	2398;4111;
1022	1637;2603;	True;True;T	1650;2622;	10034;1003	5396;5397;	5397;8566;
1064	2225;3424;	False;True;	2241;3449;	13827;1382	7338;7339;	7338;1122(
2147	4412;9338	True;True	4441;9408	27175;2717	14452;3042	14452;3042
465	3512;7412	True;True	3537;7472	21838;2183	11548;242(	11548;2426
1825	2863	TRUE	2886	17876;1787	9428	9428
282	936;986;23	True;True;T	943;993;24	5691;5692;	3049;3229;	3049;3229;
866	5;5646	True;True	5;5687	33;34;35;3(	15;18672;1	15;18672
1901	473;532	True;True	474;534	2855;2856;	1583;1743;	1583;1744
1934	1285;5687;	True;True;T	1294;5729;	7868;7869;	4253;1880	4253;1880
561	6073;9852	True;True	6116;9929	37619;3762	20012;2001	20012;3232
1717	2722	TRUE	2742	16942;169	8933;8934;	8935
388	4708	TRUE	4738	28964;289(	15450;154	15450
1795	1447;3361	True;True	1458;3386	8871;8872;	4809;4810;	4810;1100(
560	1342;7964;	True;True;T	1352;8027;	8223;8224;	4452;2603	4452;2603

Oxidation (Oxidation (M) site positions

5;11971;12023;12387;35319;35381;36581

12017;12637;14780;15814;25119;30076;30758;35738

58	218
58	221
58	223
58	223
58	188
58	218
58	218

24162

3

8978;10556;18832;33432;33449

6801;21832

434;9393;9444;11433;24950

3420;33417;36558

5;17921;18351;33953

4;16727;19904

11092;13590;14775;14930;15625;17817;26691;30146

2;15040;19031;35754

25760;32276;33012;35480

441;7097;8944;22369;22513;31380;33258;38087

0;19412;20789;22803;34203

13218;19012;29685;30731

6058;10096;14116;18492;20723;22181;27990;28147;34698;37918

10543;16799;30184

53

53;28888;36811

2;25293

26

9;18153;23196;36413

55

3;36556;37806

5;19390

0;20632;35799

7

7;24404

3

5

24

17;32715
16
8237;9905;10436;14815;18901;20184;20293;21485;22120;24051;26155;29704;34983;36610;37124;37

13646
93;19399;19710;22141;25172
54
39;25129

5

16

59

51

32830
12027;13570;17897;22074;22172;33750;34064
4

4247;17478;25076
6624;6984;6991;16080;24399;26479;26985;35315
3;19551;22114;25641;30545;36550
7;12643;12657;19936;21984
286;15021;15022;32303;35044
3;14066;17344;23124;28523;37023
12004;12919;20255;25375
19686;20161;26980;33728;33733
3;26958;35032;35348;38219
11953;14650;20086;27173;35370;35783
149;11448;13370;20409;21508;29492;33312
5;32398
22850;27075;33242
12135;33537
94;32777
20762
2;24054
21767;22334
7
02
24;8130;8531;10074;10260;11408;12415;16171;17980;30985;32521

4763;19267;19947;19982;20592;23080;23587;32733;34458;36505
14255;17102;21562;21853;21864;25473;30859;35588;37136;37139

0;17517;21937;25713;35676;37063
83 32
3;32042;34346
5546;16931;18868;20095;21945;21947;23564;26904
01;15459;17747;18892;19304;19905;22998;26888
617;10921;20569;28142;28146;38372
85;4895;5689;7228;7412;9410;10902;11710;12682;13075;15330;15789;18896;19126;20540;24618;246
43
09
51 495
4103;7269;12142;12813;13382;15683;32614

02;17664;17815;18555;23946;35009
59 52
6408;9453;10652;10858;16001;18168
5307;35227
307;35227
14259;14361;15419;25675
5;17706;18572;25272;29964;33650
18361;23111;23349;27029;34424;38075
22086;33705
34;26345;28976;35340;37649
5;16274;23423;26894;27254;38186
02;32180;32186
15178;29728;34181
01
9
0;16808;20823
39;22509;35706
1;17751;26092;34248;34488
9;13988;17572;37675;38396
03;36810;38244
40;31226;37572
98;26738;27297;33015
5
4864;9007;10225;10722;14043;15434;19155;24432;28528;28937;32982;32988;33376;33437
96;29087;31471;33770
23675;27305
0455;13044;30341
17350;21711;27067;27205
38
7914;15081;24507
38052
59
3;32886;38285

2;15048;15050;15059;22974;24047;25124;25385;25391;26486;28317;29269;37134
2;14106;15067;24834;25938;27004;34682;37841
8111;11006;14179;14279;14280;14288;14901;35136;37329;38450;38451
5;17501;26063;30243;32795

14535;18081;18107;20091;22919;23805;26668;30784;30787;32202;35817;36615

3703;17520;34669
1;23210;31561;31641
54
12275;22269;25364;27366;33827;34755;36544;37535
5
02
52;53 148;196
10;28701;30607;34875;38265

53;36573

2
9;13068;19733;23758;25720

28718;36078
18
4;10536;17346;24880;25997;29541;34670;37107
76;77;78;7 44;47;153;227;283;305;325
76;77;78;7 44;47;153;227;283;305;325
78;79;80 45;48;326
2;25990;27692;29399;30008
13737;14197;18728
24825;30109
5;23551;37426

0638;35286

10467;13485;20828;21423;24064
12320
7045;9203;10204;18759;20812;34514

11
17
55;16427;18521;21429
44
45;36867

31
0;33287;34813;35369
32079
00

8096
31

3237;5207;9804;12315;15623;17843;25686;28633;33391;33698;35744;36968;38058
3618;21089;24161;25605

7
15576;35575;37569
2
0;22648;31952;36489
8758;13859;14505;16286;21417;24735;30840;34287;35863
90;19308;20288;20402;27121

35 250
844;11210;11387;12507;12783;20400;23085;26062;30649;31045;37424
5;33471
5;19536;23002;24050;25806
34;19536
5;19536;25808;35921
41;19723

0;32885
07
27226;27283;36230

25;26 72;79
35
16997;17494;26141;28476;34525
23135;29298
14924;15793
7424;8277;8850;8851;9694;12497;14637;14662;15232;15920;15923;18949;20344;31666;36360;37394
5;36360;37394
10 16
10 16
99;18364;20655;26221;26986;32224;37687
26;32742;33483;33708
9252;9606;10083;12309;16328;26939;31631;37540

02
536;1917;3014;4382;4863;5168;5334;5683;7757;8429;9187;9661;12324;13763;14348;14450;14812;14
26;1530;1917;3014;4382;5164;5683;6996;7757;8429;9289;9661;12332;13763;14347;14349;14450;148
49;22957;35199;35634

40 94
07

5;24788;31679;33474;37299

13;16146;23715
02;17363;33206
40;30393
668;2963;4229;34590
55
8910;9351;30062;34131;34332;37108

525;10293;26918;34131;36110

44;2361;3917;4259;5470;11527;12069;13348;13492;16108;17767;23378;23386;23487;25238;25735;27

10503;11847;13564;27874;27989
44 240
4967;5448;6749;12243;14569;25762;27667;27816;28797;28804
19;10452;11272;12519;12525;16919;25761;27663;35512
56;12541;13441;22506;23770;25849;27809;28532;35470;36914
7578;11267;11849;19756;20564;25212;27820;31875;31876
12246;16111;27818;27991;29859;30293;32030;34447
668;5477;7352;7358;10937;18800;26086;26707;27778;29826;32309;32607;32684
50
5424;17024;18539;20519;24039

2119;23410
4;17987;18539;19801;24039
70;29476

39
3;15006;15079;18808;31071;37337

2
50
25164;38228
28;31244
149;8568;16740;18338;27354;31315;35546
13631;17560;20140;21431;28372;36899

17378;20672;23552;27548;34234;35036
16076;18631;19350
15202;15293;17640;20630
16;34654;35633
7579;11085;14580;15296;17480;24150;24368;25480;30759;32507;34539
003;9101;9620;10352;10717;11919;15445;15476;17460;19262;20928;21290;24438;26380;26596;3271
00
19646;29748

34
58;29067

32;18493;18886;25695;29599;37514

57 51
36;37;38;3 113;145;408;478
5;18229;24193;27418;30301;35886;38282
3
0

26;34274
2420;14142;17403;21902;23250;23518;28007;31521
58;22338;28082;30909;33367;37180
2;26717;27159
4;17136;26520;30529;37041

20
38;24987;25235
50

3655;5290;6972;7149;7659;10220;12076;15417;17951;19685;19713;21876;26035;27381;28264;31533
4;32190;34407
08;25029;31204
7976;9527;10944;12349;23005;23764;25011;28055;31204;32135;37797
17415;29187;32080
93;37934
33472
;1682;5093;6653;7682;8184;10464;12316;16105;16688;17663;18477;19219;19588;20776;21176;2155
7;10380;10522;12150;12161;13131;14572;18746;34782
11990;15903;16697;24257;26695;27763;35814
3
7;23671;24949;29380;31331
12400;13325;14000;16053;16110;23034
5 352

3;25880;33401;35836;35903
3

5;35923
5
33 15

7879;20588
20232
54;17010;17954;27042;27952;29693;34051
20030;21033;21038;37927

07;29716;31547
8759
27;35613

2;28524
2;14971
26;37189
6124;9015;10978;12749;16229;17662;17704;20754;20775;22032;24278;24605;24711;25929;27462;28
0

6631;18757;23223;23893;24313;26956;28362;28786;32071;36464;38344

3

42;32240;35658

3
05
50
3;19477;29447

0;25221;25273
1292;11516;11777;13641;17394;17712;18370;18773;25973;30916;30925;32413;38420
5;32870;36834

64;7100;7441;10448;14262;15813;18104;18395;19417;19573;21519;24827;25923;28506;30198;32714
59
4;22055;26106
56;31671

102 370
05;20527;26277;26689;31404
097;14511;17180;17911;22981
6900;17499;30424;30940
607;4071;4083;4870;7952;12688;13395;16329;17168;17171;17585;17819;17967;25505;31343;32266;3
20;30849;31531
36;18751;19082;22601;26185;27141;37817
33;24584;27140;28351;37854
3;21421;27936;27937
3909;5271;12620;12979;29108;33225
60 257
7;31111
99;35256

36;28091;33065
0;36513;37249
9552;10208;12225;13024;16036;19401;27997;29341;29736;36617
7639;11344;17225;21735;29305;29511
9;14926;15864;16363;20328;27474;31831;37761
20624;20704;28041;32043
5;33078
73
06
9
49
7;1211;5627;10546;10846;21604;24310
38;16054;19991;36380;37861
28;14503;17154;18474;19198;19895;20511;23439;24215;24917;25075;25658;25799;26077;27507;295
3;22330;29429;32115;32681;33341
5;13227;13765;14552;22138;25818;27544;28189;28728;29147;29612;30358;38077

9786;26783;32126;36175
2;30741
10880;11434;21622;28683;30359;35798
7947;8835;14148;14867;26892;38384
7
59;32156
58;29832
35
24 189
21356
51;32525
41;38150
36
51
73;35317;36675
30768;37103;38392
49;19672;22231
9
4785;11881;32611
96 168
30
36

2079;17804;26190
5;13662;15409;18221;19812;24774;35170;37882
25;30142

18;22065;28981;34171;36942

6722;11890;12693;14155;17264;29111;36622
1975;2744;3753;4303;4717;9128;9334;10500;10680;13031;14390;14589;15481;16732;17229;17411;17
8936
9
59
5;34438

4
97;31656
39;12928;32317

839;26582;27025
37;35590
3;34811
218;10947;37240

4586;5860;11811;12344;20343;22180;24294;31800

7;25128;35411;38365
933;13598;19212;20867;26184;31126;34485;36732
3;19408;24753;27797;35306;36418
1;21098
30254;31810
3598;8002;13921;13925;18076;19594;22988;24007;24009;29889;29890;37152

8630;20765;33716

57;25930
12865;20341;20545
17327;21157;24789;25581;26594;27865;28510;34511;37068
2234;9489;23216;34182;34196;34197

5;31587;34431
33;35545

3910;26652;27146;29547;30776;33327
3
9
34
5;16810;22529;23579;26715;29403;29840;33490;34151
3
745;4034;13668;23566;23571;23890;26653;28452

54
09;23658;28502;36447;37400

32555;34712
93;32850
4

5
9;12252;12516;37619
75;31090

19761;21871;30222;33295;35005
52;19164;23040;29351
08;16689;18262;19288;35973

32;34832

3;12461;13787;27442;33217
48

79;32444

33
517;6646;7916;8640;9422;10903;14132;14611;18696;23310;23499;37661
8716;12203;20936;21696;22000;23466;26752;29042;31553;36888;37274
);28416
)

13463;21409;28310
32
96;27017

01;36596
27723;35674
46
12434;17741;22275
23611;36818
5;25157
34
29
49
36
1
5;13326;30297
81;3463;5442;5628;7256;9584;10153;10569;12379;13437;14143;15842;16236;16544;16546;18314;18
223
15;33084
10;32904;38209
5757;9000;14360;15124;16722;18415;19035;20952;21165;25878;25971;26553;26671;27774;28515;30
30;31688
59
12775;31392;32997
57
5726;12025;13383;23435;25586;31871;35291;36206
18;29501
46
42
20
53;25299;35737

28363
);14474;19490;21974
59;37201;38094
21
7498;8573;9688;13002;18532;19724;20784;22936;23014;26040;34315;34751;36082

34;29000

31;14753;15278;30858

5;26826;35632
7;15910;24936;25883;28970;31778;33626;35122;36411;36631;36757
13333;15045;17114;17319;18296;22449;28986;34843;35749;35969
2402;2817;9260;9265;10441;11258;12002;12356;12600;13772;14325;15133;15757;16608;16794;1709
4183;13900;13994;13995;22988;37145
53
3765;24176;32128
9;16107;35768;35808
12691;19566;24973;25526;32214;34213

0
5;22097;27444;28047

3;15134;15443;17336;22517;26012;26569;37749

25963;26733;29197
5;21250;28051;36869
71;26788
33;18434;34314

9120;9122;9798;12402;13479;13482;26368;32644;32657;37250
15617;20029;30166;30213;32320;33489
846;10222;19532;19804;32697;32723;32808;35723;38108
0;19567;26414;27885;32658;36097;38342
1;12779;13250;14755;23148;23291;26044;26891;27710;27711;28605;28759;29431;29576;30532
968;8528;12993;16199;19252;24014;24274;26045;26466;28185;33298;37638
7;10457;11723;11883;14468;15194;15781;18669;18742;24800;26982;28074;32511
3;14835;20837;23432
346;3860;4094;4241;5684;6155;8366;8917;11051;12223;12646;13784;14003;14375;15925;16284;169
59
5

33
4

1;26099
28;26187;36136
19;35535
7016;24539
5;10695;31843

19942;34868

3;33764

23507;23514

7;38250
5
3;20866;24888;29802;30504;32074;38215
3352;5451;6462;6591;10630;10961;11616;13977;14887;18362;21010;23962;24992;27728;28765;3098
16952;21347;21516;23007;24549;29192;31394;37590;37599
16574;27347
5;10307;11146;31409
9

4
4;37855
9 1
33223

57;26876

50;37625
5;32724
0 4506
06;37978
06;37978
13643

70
4501;6446;6447;7686;7848;7850;8369;10103;10106;10126;11585;16078;24674;24726;26434;28221;28
8775;9862;10951;24452;26972

07;19749;20900;21976
0;12199

51
0;23614
8;9370;21443;34480;35892
37

4;19297;19881;28595;37830
9623;24943;28788;32161;35498
20;19234;19579;27622;31715
12251;15129;19712;20840;21681;31480;34368;37185
15359;20924;26270;26730;30094;34348;37196
40;29286;29782;30403
11117;13703;14771;28563;28674;29783
11117;13703;14771;26624;28563;29783
8;6026;10591;15994;17462;17685;17848;18814;22628;25620;26811;29977;29978;31158
3815;4058;4270;5591;5600;6327;7270;7619;7712;7716;9297;10864;11684;12017;12168;12904;16168;
11;20711;28114;37458
17;37442

910;8142;8373;11485;11639;11641;12347;13448;14545;15807;17703;18179;20135;31615;32826;3321

3;15211;33282;37969
28;25651

5;21425;23687;26732;27559;32462
11288;11645
9866;11303;15880;26005;28949
23998;27602;32206;33803
79;34380
6892;7130;7364;7858;7876;8319;8324;9143;9405;9653;10713;12475;13671;17387;17636;19779;1992[illegible]
4
33239
15801;28972;35499
72;26449;29998
7778;8644;14831;17333;19459;28679

02;4319;6040;8028;8554;9040;10813;11544;12700;12861;13210;13261;15521;16382;16998;19324;21[illegible]
9473
2;18615;25758;27161;32515
96
10849;12021;17695;37791

4
95;31291
03;38340

24
6712;20345;30262
2;20345;21474;30262;33606
9790;16143;19483;20278;21314;25853;26971;27100;37835;38138
05

6138;7451;8155;11861;12201;12369;12372;13760;17660;17773;18114;20790;21233;22833;25949;341
9259;12369;12372;18114;23065;34121
1;19197;22702;25300;30277;30528
39;29512;35708;37398;38403

3
18938
3;30981;36667;38404
9421;15199;22518;31300;32630
5;14929;17244;18748;19915;28065;34706
5
8911;10558;12822;15405;15761;19200;23923;24319;32011
3;12180;13411;17089;24173;36363;38433
9925;19745;27052;27920;36716

27;19493;24121;31944;33408;37065;37810;37947
46;23497;25914;26325;29587;35504
)2;37198
8135;36384
24277;33851
12140;14493;17113;19287;22472;26091;28218;30440
3

43;23837;26247;34495;36080;36774

467;7138;7545;8580;10468;10733;12830;14051;21156;23898;25274;26860;30615;34394
5
);31406;33542
11794;13238;31376;32897
2
17203;18176;20872;20873;34806;37277
59;20040;20440;27745
1;20674;24744;26725

2;16653;35147

96
33
7517;9130;17412;26283;26400;32709;34057
;5614;17190;20435;20521;24168;26348;27335;35038;37219
3
6066;9999;12782;19767;21862;26282;31691;32021;35935
2700;5872;8510;15346;19313;21108;26251;26314;27037;27038;28391;29519
692;7436;8116;14399;14814;23680;35524;38296
098;9780;11164;13722;16436;17141;19274;34013
7600;10291;10914;15086;20376
599;10241;10916;20376
9;19237;22123;27620;28344;29481;30040;30261;37242
48;15365;16539;16556;16589;20174;23313;27457;28587;33574;35750
1;13032;14396;15353;15631;16589;17814;22963;26208;30265;35752;35933;35954
38;16560;16589;22855;23312;23889;27111
4;35465
3;17860;20698;24883;26513
9;18018;18151;21396;22092;23508;25232;25998;26150;34665;35577;36329;37037;37405;37691;3775
52;4493;4525;5113;5171;6025;6083;6443;6976;7232;8847;9730;9752;10644;12271;12690;12754;1338
5;32541;32852
9;25037
14189;14626;16085;24952;37601
36;16279;20350
5;22845;25059;26440;32743
95 1
8;948;949;1127;1375;1476;1500;1542;1631;2016;2188;2982;3214;3736;3774;3898;4431;4439;4633;46
0;1375;1483;1513;1594;2021;2543;2582;4212;4678;5546;6936;7115;10506;11251;11282;11589;12208

5;25978;26340;36739;37027

2;21085;23649;25247
3;17084;35982;36319
5;26046;26813;30516;33848

8029;18130;21171;35001
38

88;2131;4411;5100;8497;10786;12269;13697;15342;17595;21291;21302;25564;26298;26970;27064;33
13692;17595;26970;33990;37173;37174
;15153;15160;15368;17385;19799;20660;28291;33354

2;20798;24716;28094
277;10499;20748;20852;28120;35995
49
27586;38221
35
4;36052
5;16276;16551;20084;21743;22010;30978;32819
5
6015;6294;7691;14522;17737;25252;26712;27703;31242;37624

9637;9884;27104;28512;30129;35770
0

38

2;31159;31787;37433
8656;12130;12982;13798;15218;17973;20783;21141;23997;24846;32542;34618;35948
2;16307;22976;24346;29258;29340;31197;31792;34448
5845;7071;9006;9110;9243;16023;19602;19888;22486;25397;28744;28894;33047;37345
3918;4155;4826;5484;11853;14036;15505;15507;16728;17718;23292;24216;25200;30501;37972;3822
27

1;36202
9883;10835;13289;18149;22599;35426;36148;37131
9138;13754
12952;16077;28410;28702
7

2;19611;28064
23685
56;19010;20425;20677;22961

	48	92
	34	88

9531;18318;22317;25183;27841;33008
93
96;29813;33107;38051

3;36359

)4;22613;26262;28121;32318;35373
4253;23851;28301;30443;36707
716;18289;21620;31062
39;26487
9
16816;22586

5;26087;29505

35
31
3
9805;24724;25253

36
14185;22234;24287;25077;33738

96
9574;14756;15601;19285;20369;22805
9575;14758;15611;19285;20369;22805
4;27605;27606;30612
19435;19933;20560;27845;36046
7476;19933;20560;27849;27855;31223;36046
14874;15606;16909;19285;20369;22805
)4
9;25590;35485
)0

5216;6562;10841;12144;14200;22475;22495;32148;36730;36734;38361;38432
)3

3
30897;33306
16169;19819;25918;32807
664;12339;12676;17120;22264;23307;27948;34405

6 550
1366;4483;8524;8536;10350;11393;18002;18975;21603;23201;23531;23868;24080;27769;29189;3005
5930;9912;15660;17051;20444;30035;32847;34239
4297;4657;7921;9755;9916;12845;14540;15634;15648;17051;20444;23317;24650;24659;24742;24927

5694;8393;10218;11093;11393;18000;18109;20528;20628;22567;22812;23530;24636;27027;29536;30
7731;7732;8302;8331;13903;18861;22343;29273;30802;37372;38369
5285;12971;18196;18581;19283;25396;25769;26350;31729;35794;36132;38311
226;5949;6183;7299;7738;7745;8112;8309;8312;8337;8338;8619;11115;13681;13990;14114;14131;14

47 466
9916;15634;15648;17051;20444;24927;29931;32847;35978
5;29735;35406
04
0;27266;37930

12 33
0;35709
7;32755

9;14858;17726;27483
79;22665;29679;29682;31658
2;22726;22827
9;19334;19420;28243
11781;15128;19336;24609;27945;27947;28409;29760;29761
11783;15123;15257;22785;29766;36919
10064;11790;11960;15265;37571
19;37573
14374;17041;25363;26566;29842;31013;34793;36315;37456
12607;14413;29218;29997;31526;34793;36313;37453
12606;14413;29244;29997;31526;34793;37448
5181;7924;14223;29988;34788
3;11959;13151;13319;14859;15622;15679;15739;19371;20349;23430;24248;24855;27079;27083;3094[illegible]
0;18663;20800;22593;23661;24551;27506;28636;29854;30907;34484;37533;38441
1;1146;9437;9949;9954;10176;11395;12231;12760;16233;19570;19750;22836;22842;22858;22873;22[illegible]
5626;6229;7547;8852;11792;18188;19431;20614;22551;24127;27271;31483;36274;37483
7549;8692;11792;18191;19431;20610;24129;27271;31483;31888;36277;37485
5354;5722;8109;13770;13849;17497;17555;17567;20268;20270;20791;21031;22649;23088;23820;238
71;28246
020;5696;7632;10655;21117;22322;23608;23814;25211;28182;28183;29654;37993
56 193
17212;18057;20051;20052;22934;23387;23640;26164;27198;27668;27760;34053;35829
4017;11177;11183;14095;27345;30022;34280
3222;4333;10442;10552;11178;11183;14090;19775;21400;22105;32877;32907
57
79;17789;19544;23224
24

1;26475;29843

15574;25311
1

58;37465;38435
58;31042;34597
13364;17468;17506;17530;17936;27965;28378;28380
13364;17506;17530;17931;27970;28378;28380
13364;17508;17548;23887;27979
4
5;23619;23623;36356

11487;14165

5;23619;32581
31;32 60;63
31;32 60;63
31;32 60;63
31;32 60;63
1;27338;29951;38120
84 85
1649;14267;20811;32363;32889;34964;37932
11;32363;32889;34802;34964
5124;5128;9831;10217;13816;17729;17735;30322;36857;37435
5124;5128;9831;10217;13816;17729;17735;23370;30322;36857
9
5124;5136;9834;23370;30334;36839
5124;5128;9821;10217;23364;30334;36839
5124;5128;9821;10217;23364;30331
9528;18374;18885;25811;33344
8254;19707;21045;26921;29090;30961

11477;31116;31290
35
9;23045;24730;25367;31459;36401;38306
0;20338;28156;30534
09;30353;32124;34890
991;19697;22570;31870;33082;33488

5;20314;24095;24320;24485;25107;30051;31472
22595;25069;35947

7
13985;18245;20284;21286;25414;27683;29168;29684;31794;33144;33336;33353;35117
54;20512;23067;27142;29916;33320;33720;34836;35891
3;27619;35479
09
4
35
25
5
0
2305;13122;13795;14405;34842;37688;38367
75
7052;11055;12725;14016;18185;24191;26987;32707;33583;37342
3;18042;19413;32744;34953
3;23305;28602
2;28207;36304

4368;4804;15438;16014;20756;24422;25616;30059;30135;32544;33982;36534;37197
32948;37833;38345
552;4929;7627;10160;14997;15060;19224;19850;20408;20818;21368;23981;24842;27441;27712;3050

10159;29674;30009
2
5960;9712;10687;11437;11749;12190;20069;21435;27375;28214;28845;33099
6657;6781;6994;7708;8702;10211;11608;14515;16769;18692;20235;21737;22049;22148;22661;25023
5
9;27765
4;38090

7;15317;18613;18652
52;24595;26473;34831;35071;35401;36891
3740;4200;7102;7250;8776;13811;14392;15277;19349;20766;24582;28270;28486;35100;35255;35384

8727;14967;25112;29262;32423;36982
19494
4754;13724;15743;16735;19861;24020;27687;27800;28477;34244;36452
7;19163;19346;21191;22542;22866;24442;30280;31059;34283;35285
7087;10387;17971;30536;38274

091;12280;14240;14457;18305;20412;20424;20816;22324;22330;23713;24120;27308;32118;33341;35
7332;9808;16993;21388;22093;35041;36298

1;18558;26626;26635;28900;29634
15;17;18 313;341;416
4064;8986;9330;11069;16141;18558;19137;20092;28906;30974;32564;34946;35157;37336
3051;9239;12042;12053;12614;17232;18126;18560;18931;20211;25530;25665;26641;27058;28775;28
14;15 259;316
5;12101;18558;28244
32;24109;26096;28714;31900;37262;37304
10359;11906;20216;24153;37262
19 297
89;2860;6479;9570;9685;10353;12730;13189;16772;20216;24109;24153;25743;25844;26202;26423;30
60;6479;9570;9685;10353;13189;16772;20216;24109;24153;25743;25844;26423;30471;37088;37262;3
16 249
13 406
58;28346
02;30225;35625;35938
30;22546;27258;27357;32514

639;22302;24745

93;27143;33214

0
20;24304;26875;26967;28682;31809
2;12921;20082;24683;25283;27877;29878;29894;34881;37551
4;12921;13868;20082;22779;24683;25283;27166;27877;29894;34219;37551
5;31130;36518
4396;4401;4560;6151;7437;9045;9534;15115;15333;16530;17813;18469;18635;20223;21172;21360;21
987;2249;2253;3361;4386;4393;5623;6571;7673;8217;8674;8818;12450;13089;16715;19100;19203;20

57;14587
1192;19221;33279;34292;38067
19691;24876;29506
3952;7113;13028;18307;23155

9707;13475;29390;37865
16017
1851;11566;27238
0;21126
5
2
30041;33425;36460
1579;2246;4503;4682;7804;8082;8901;11550;12734;15478;16507;16892;18745;19487;19847;20410;21
14585;23726;27362

7;31801
5519;21267;22016;22262;22409;33442
7;28076;28304;30010
74;37393
1;19788;33277
3;30123
7218;25290;27386;36879
51;22910;26540;26564;27115;32442;37570
0;13727;14789;16670;21548;23746;26625;27837;29504;34775;37999
9;29851
6147;6743;10244;11698;22190;26882;27250;31885;34809;34901;36376
97;12666;15496;17153;19843;27245;28667;34901
40;17306;26245;31039;38199
7;26199;37834
5;13455;14103;15473;20356;21073;21582;24902;34547;35410;36950
73;21582;30517;34546
02;29388
06;24042;25134;28042

39;37874
5;13120;15553

3;15843;16260;20876;20880;27012;36450
30;38029
08
15373

54;23926

28394;32966
5
15
12
25569;36050

34;36163
30;18242;32204

7
3238;8434;12856;12898;14546;19402;20475;25647;33609
10973;19790;25016;35039
7881;10992;11402;13802;14061;15220;16495;21913;22978;24779;29530;31621;33684;33860;35717;3
7;21692;29227

3;18352;19711;28661;31954

9844;11465;12790;12887;12916;13814;15429;17103;18691;24875;28101;29499;29523;30964;36559
30;36719;37135
4629;32273;32277;32278;32280;32311
21 76
7
7746

11134;12412
2;12501;27019

15847;18731
58
9
32387;36717
7;26704;37935
3

14;27430
0
53;17112;19114;31951;36922

36714

94
4
464;33418;36425;38174

197;12210;12747;13711;16946;18065;21468;26662;27791;27835;28566;30853;32706;38235

39
54 517
17926;17983;19078;22034;23972;26010
56;32875

37

100 301

418;21840;24996
58;36042
20623;37658
34;26769;29137;35289;35385
99;19710;34803
34
40
30
5;15850;34450;36257;37227

1237;22491;26090
0;29703;33081
7;13307;22606;23144;26349;27106;32290;32298
62;8767;12572;24968;25739;27522

5;29907
14021;21753;25506;30145
92;30078;32165
7
14;24370;32252;33677
3
33;36090
2450;2784;2811;2812;7034;7216;9046;11137;15346;16637;16906;18150;19313;26251;27038;28383;3

21251
5
4534;29821;34510
3;29443

136;9511;12853;30296;34261;36688
38
5999;8879;9668;12801;17992;18062;19989;20308;20439;29276;29296;29455;36827
7
3;24552;27962

5
31;4995;5251;5338;5785;6190;6776;7452;8406;9596;9598;10178;10653;11868;12295;12392;12447;13

6128;6902;14495;16124;24074;27536;35167

6900;8385;10891;12769;20709;23429
72 90

70 372
8785;9819;17652;17687;18468;23546;26300
62;63;64;6! 86;137;162;329;365;627;879;941;1489;1594;1910
5
9;6951;7439;11584;11720;35608
52
3
9104;10231;10257;10570;14781;16028;17300;18894;26523;30096;32000
13983;19344
441;33494
9;20036;32621
12712;15125

5

49

12957;30072;30074;30207

35;29436
24548;37346
72
0

9;22184
0
3;624;652;662;747;761;820;913;914;2429;2451;2464;2548;3059;3524;4832;5358;7574;10179;10190;1(

53;27905
735;4954;5989;8782;10039;10728;10765;12553;14856;18793;21477;23078;24533;25222;26989;28259
735;4954;5989;8782;10039;10728;10765;12553;14856;18793;21477;23078;24533;25222;26018;26989
4
12823;19706;23980;27478;27871;29958;30442
44;36259;36782
14;31213;35809

98;26496
18764
43;30617;32105
6092;6095;6668;30082;36832;37831

0;16612;17034;29773;35979
5;30920
3711;4428;9677;10142;12358;19428;22915;25555;26518;27074;34254

44;21246;23107
54;38119
7594;19464;29422;30419

47;28481
4;22806;30733

4;24298
55;21196;21415;34198
;2641;2703;3071;3367;3650;4736;6200;6213;6878;8121;8269;10727;11760;14339;17850;18036;18120
53;28225;33202;34442;35326
10392;12598;15041;18217
25;24999;28282;32884;37413
12616;13577;16821;16897;19826;22520;24781;25467;29884;33623;38393
5;20534;20934;27725;28116;38159
6067;10897;16597;18472;20165;21739;37917
0;25219;26023;32820

7;12953;18516;30111
01;20770;21899;22274;24269;29784
1862;24217;28598;29010
37033
0;34692;36976

3536;23110;30922;34569
18402;21635;24036
7;36514

7338;11224;23895;25184;25192;27125;27133;30191
06;22655
4;33402;33445;35991
72;24822;35907

4610;17874;18400;19005;21005;21608;26583;31906

07

1;14847;15632;18485;21480;30075;35316;36649;38021
2;15695;17810;20758;21137;21457;29582;32509;33252
11
230;7082;7502;7605;8146;8872;13658;15468;19769;21666;23696;27499;27584;30130;31171;37021
32;32347
7044;9069;10200;11827;12185;12631;12632;13064;13736;17693;18108;19107;23643;24815;26024;28

41;42 65;81

39;32263

3;28609;29410;31881
01;35252
9;21397;35931;36443
8234;8451;9971;12182;13072;17876;18750;23958;26037;27136;29517;32039

4;24002;31144;35542
11494;24006;27425;31144;35542;37931
53;37193
62;27906
4

9;26592

9638;17309;22556;32024;34667

9670;12378;12565;18092;20977;33114;33482;35354

7;13876;17670;37402

57;25104

0;37463
1458;14245;23103;27323;30498;33358;36735;36737
5
9
85 76
85;86 61;136
6416;6557;10573;14568;15308;32925;34888;35206

3;13177;30941
20;27030
05
34;27775
3729;7861;9846;9935;19156;21071;22503;32486;34610;35388

3

79
52
12427;14776;19721;19737;27779;30279
35241
199;5445;12490;14008;14011;15372;18015;20645;22288;25985;26130;30156;31549
2;17673;21422;22294;26130;27065;30152;30831
0;14008;14012;16389;16797;22296;25268;26113;30156;32561;36180
31;20102;31516;34560;35626
5415;18443;20889;21494;25334;33627;36542

20
3741;4021;11323;14801;14964;16695;19669;21311;23911;25557;29069;29183;33657;35393;36606

5;17046;18473;28072;28686;35521;36692

7
40;36511
672;14125;19928;23978;28417;30522;32821;33318
49
40;13466;16620;20969;23199;32946;35518

12757;22516;24686;37031

12900;29619
37;28297;30070

13061;35412
5825;28210;28482;31575;32440;37260

623;2009;10209;11424;17072;17079;19510;24488;31387;31391;35338;36107;38188
10779;10781;14007;14566;24198;27174;30355;37117;37118
0
79

3876;4846;10181;11378;15663;26886

3;34246

43
2
13551;20470;24194;25275;30177;32861;38195
57;32688
9812;17950;17986;25336;26998;27103;27202;27757;29707
99;20714;29064;32414;38455
5;7678;8054;10795;13314;14489;16724;17879;19111;21581;22967;23670;25154;31036;36748;37128;3
3562;3765;5152;16264;17879;19111;21581;22967;23669;25943;36743;37128;38006;38308
52;23321;30589;37129

449;1571;2125;2599;2643;2659;2757;2771;2849;2876;3818;3984;4192;4412;4505;4694;4827;5758;61
33

1;13865;16590;21198;34779
3032;11939;12093;12377;14722;21202;22047;24671;25522;26550;27512;31859;32833;36510;36639

14698;15742;16250;18990;22448;25957;35571
14701;15740;16250;22440;25471;26047
17434;25611;26678;28471;34711;36471

10127;10828;11826;11829;13782;17667;23798;23799;24143;24571;25400;25642;28369;28652;28851;

10832;11826;11831;25400;28851
49

53
7846;15588;17893;19240;25602;28110;35537;35538
1119;14485;23877;25084;28258;36731;36877
1;32375
13069;14328;14658;17909;21525;21997;23856;24482
8658;21997;22609;24170;24479;29598
5;31746

09;2731;2733;2789;2910;5588;6833;6835;7644;14945;17020;18023;18261;18636;18919;20031;20223;
5;17373;18154;25860;28881;29701;31990;36252;36423
38
15;8980;9947;14005;19178;19257;36944
2966;5416;5944;7895;8948;14001;14903;16132;20247;21959;24510;28296;30520;37892

3;29194;31032;31793;34341

7
7;21813;26476;27520;30987;32729
5;19007;26048
7572;15108;19077;32064

10409;11214;11238;12298;12941;15860;16449;22282;24873;32439;32734;35311

5 330
43;5345;27219;30179;30492
1
5;19108;20546;23220;23876;28748
57
5 253

22
1
5;26589

5
9180;18147;18882;25660;27137
3;23396;24701;24706;29008;30412;32242;37053
5;27751;34759
49
15248;18347;22160;27753;33319
6283;7869;7871;17566;29722;30003;31206;32076

)4;18381;18437;20145;30218;30253

7
39;7590;9747;15249;17853;25082;25482;26755;26837;34451;35358
734;9166;9591;10502;13156;15366;16941;17277;19652;20359;23038;26759;31556;35367;35434
759;10735;20584;23137;24124
668;9750;11475;12179;13918;17131;22216;22851;22924;26039;27894;29007;31629;33480
42;21106;26197;27379;28085
14;38436
71;35519
11447;13146;25847;27680;28958;30595
1
36;32368
14152;17714;21839
5
5;14654;31371;32970;36545
40
16718;17338;31033

42;32958

50
3;20152;20678;34236
14109;17014;20181;26676;37566
9227;14204;15497;20121;22368;24668;25827;31490;32482
3;34492
7435;12936;13628;17134;19391;20643
16058;17797;22039;22573;23967;25199;38364
2;20509

11385;24393;33027
59
05
59
12721;12885;13835;14053;27028
15;32867
4
22
99;35253
046;31302;34156;34891;36948
0

3
5;23880;27690;31680
20
30587

7823;11231;36430;36432
31;25005;28464;36775

57;19882;21144;22071;24714;25798;26490;29313
43 425
61 434
9875;11725;11728;14840;18451;22668;26233;26996;32450;33788;36156;37497
2;13175;13269;13273;15241;18249;24156;24159;24677;24841;31275;34554

0;24290;28314
75 377

5
5;13960

19834;22629;29352;31299
23273
2438;6029;6237;7372;7403;7494;7816;8958;10318;10592;12448;13239;15943;17642;18564;18601;215

24;28688
59

37

3
95
3;30306;31006;32973
30

8632;22594;25417;28427;28501;30596;37439
10732;35481
5

37

9256;13351;26268
3976;6043;6697;8344;12973;21101;21374;26132;32168;32382

5220;6467;12792;24179;37447

4531;6236;15030;36390
5
25452
6039;16939

2;23807;27325;28122
54
33828;38132;38135

2
9687;36197
8914;8916;14877;20696;20697;34378
20874;29078

4
04;25047;25380
8

15570;33226
6;32857

17577;19937;35939
17;37766
5;26935;27496
;24871;29397;31270;36435;37410
;35295
;14351;19470;21967;24850;34955
7;15396;38386
19681;20752;33493;35250;36371;37113
92

	94	1
87;88;89	276;404;410	

4388;15013;16656;28623;29021;37361;37365;37611
16656;28891;34951;37361
;34738
7

46;34313

7;8	101;107	

22
00;5060;10237;13401;32716;34216
0;33884
16159;36309;36657

	55	360
	55	360

11061;22576;23047;23262;24586;25248;26263;30813;33247;36278;37593
6;34464
42;30601;30836

8391;11350;18309;19051;19112;20759;26778;29667;29903;34982
3;31812
10473;11163;20935;26934;29862;31188;35568

3282;5304;7307;7430;8890;9003;9874;10011;10700;12617;15279;16310;17083;18247;19648;19688;1
40
4840;14075;18801;21955;25520;32993;37783
4840;18467;18801;25520;32993;37783
33
0;20361;23939;32410;32949
11375;13415;14190;23701;24029;24477;28567;30026;36584
0462;11173;15573;17432;17840;21990;24314;27449;30131;31296;37632
92;16032;18379;18810;23633;30737
16609;18158;20361;22902;23525;26895;31579;31705;37167
20361;22899;23106;26895;31579;31705;36503;37167
2940;36594
1

11165;21185;27450;30603;37635
5897;6302;21213
7

0;22225;23750;25703;30562;37575
47;26847;30007;33323
4915;6020;7012;10707;11197;22410;23480;23484;24462;25886;25888;31804;32670;34914
3;35514
54;31579
9;34952
0;34963;35050
34;26901;35047;37255;37259
36685;37251
05;22458
58;26854;36453

53;23589
90
0694;28149
29066;35636
3;20838;28331;29131;33607;35772;36636
23037

36
4;20692
2;32891;35745;36571
49
25
9;22816

4
38;14904;16973;17178;21868;31100;31102;37626
1;34852
75;14272;17939;22756;27525;38205

96
9;32828
3;18426;23061;36701
7
28553
2
24;19151;24538;35748

4;16667;17916;22213
9556;20045;33103;37776
8544;18744;19160;21556;27463;35148;35665;36842
3;27285
843;12587
11;23601
11;23557;25420

880;4959;4962;8005;10018;17511;27199;27625;27632;28334;29423;32227;33146;35888
42;20105;36720
8349;34529;35937
36185

4588;35088;36362
1;18641;28075;32763;36955;37104
25;13872;14144;19434;27076
44;33748
55;36718;37056
2
33
96;24052;31507
4733;6876;8808;9281;9476;12807;14127;21262;21731;26294;28432;28505;30168;30527;32498
5;18409;26101;28630

6333;11787;12022;12926;17204;19328;27592;34125;34302
3146;15269;16291;21470;32522;32528;34871;38180
9;30583;34871
19479
101 732
8057;19944;27929
75
5;15111;30805;31335;32990;35278
3;23207
82;11478;12064;25786
361;11596;31399

10
31;29696;34470;35101
0
59
30 201

3;35135;35172
07;27767
3615;7984;12512;13359;15802;18825;25576;30542;35784;38255
609;6683;11057;13361;13428;16639;17768;30954;32471;32768;36823
04;30777;32552;37770
17
4566;5487;5493;7455;7521;9526;11336;13331;14398;15386;16640;23115;23415;24372;25177;28968;2
544;15853;18287;23871;27917;27919;30316
545;15853;18287;24350;27917;27919

5;21983;28896
4;34147

5;26908;30462

91;92;93 121;326;358
5
258;12444;13729;13857;16165;16683;19475;19922;27307;28295;31352;35220;38155
55;21907;27558
14218;20450;36552;36697;37549
0

5451;29384;29475;29888;30459;30657;32203;32219;32321;32328;32335
5292;6945;17441;28406;36695
4

5;34014;34577;37481
24558;24559
4;20686
4;25626;28248;32142
24558;24563;34267;36833
6939;9899;17451;28407;35511
98
5

98;20482;27600;30214

98
16;21664;21968;22597;24135;27834;30903;32574;35111;35210
70;36928
21139;25142;26124;29330;38170
24989;25236;25345
0;13890;15184;17395;20745;37538;38028
0;13890;14816;15184;17297;17395;38028;38114

12
27 1
2770;13890;15184;17395;25103;38028
57;32911;36709;37261
7;31559;37823
39
39;21934;38446
514;6596;8252;8253;11546;14102;15559;16117;21632;28956;30932;33749
09;24177;27433;28231;31444
4909;6428;13683;14117;17929;21181;22523;27286;27314;27315;31364
2;22784

31
19
22847;24201;28198;36872
22;25650;25993;29818;37662
92
70;36972
9;19802;28271
33;37403
70;32641;35313
05
40;25427;36244
11558;24912;27200;34204
19310
0;34078
10360;20323;28737

06;28194;34702

29
5
72
99;26988
30;33589;35283
3;14201;15228;23050
25;18630
15;34895
56

8;3402;3545;4173;5209;5538;5867;6188;9492;12367;13558;14646;17195;19929;20724;21520;23917;2
6211;33572
50;27608
5
13;30573
3;17272;23102

74

75;31096

205;15411;19125;20864;33485;37975
17033;21594;28359
24
34
92
304;10736;16777;23333;24598;29561;32262;36154

793;5574;5649;5781;5790;6641;6836;7013;7056;7065;8441;8701;13567;16988;19314;21167;21399;22
7974;8977;9364;9699;16214;22011;22340;22377;29588;30530;30555;32470;32535;33701;34886
28;30812

12400;13325;16053;16110;23034;23592;35620
92;28604;33040
0;30957
4;37026;37929
1;17356;30242;30986;31505;35357;35905;36874
6809;18295;18501;34602
3;14040;14084;17262;23154;23795;32394;33056;37399
00
11;35579;36895
19335;19593;28479;30160
430;9671;9701;10150;12722;16701;19423;19426;22853;25514;26001;27071;28692;29428;37461
3;37507
6733;7598;13027;17743;19398;22864;26804;32517;38110

93;28530
2903;8746;10180;10182;10185;13016;20992;23519;25814;27367;27370;32538;32540;33110;37501
790;6862;9425;19026;19763;24229;25935;27694;28924;29769;33395;35026;35725;36708
9171;28400;28632

70

6075;13629;13745;25007
3141;3384;6608;6610;9126;25676

73 370
78;2274;4277;4792;6070;6804;8188;13892;14571;18651;19934;20586;31912;33259
4

10959;26497;31574;31636
16261;16283;23666
9;22771;32888
10936;14229;15535;21970
659;14865;24940;33276;34741;36051
09;22158;26027
98;16244;17210;29311
57;16240;19067

12717;23337;23852;29982
97;22885
15449;18122;18170;25478;26359;27182;30468;32692;34533;37115

23;24524

4
33;32099
11570;14072;38083
3;23667;28750

00;21773;25678

59
76;34374
9
8439
10284;18846;19559;20537;26424;27162;31048

52
95
321;13506;20945;25216;30950;32956;35333
39

5
5
4
2
3
10048;22564;23881;25460;28475;28938;29030;34985
10048;22564;23881;25460;28475;28938;29030;34985
18;2904;3698;16775;25830;32108;34564;35508
49
99;12335;17918;26601;32606
24700;28199;28253;30799
3;19095;31147;35580;35852

54;32499

8865;9700;11324;14594;14792;19789;22788;23062;25637;28934;29591;29790;32578;37647;38069
743;7127;10730;11347;14038;16021;18612;19595;19597;19883;23988;25801;28864;33221;35494
5371;9225;10808;12206;14853;15407;17895;22185;25294;30905;31258;32018;34857
10745;14595;14657;16704;18884;20595;23063;28862;36705
4406;11947;20911;26191;30098;31191;32111;37945
3428;8024;8288;12307;14451;16371;17320;17716;24258;25035;31897;35965;37061
3461;5353;5849;8257;9197;9635;11637;13282;13989;14788;16016;18567;19444;25000;26127;30519;3
7729;11926;12196;12680;14448;15721;31078;35128
19;19838

5
57
3;36620
05;35565
46;31140
3

48
22959;30525;34587
07
38;36224
14622;19343;21256;31791;33808;33819
96;35574;36555;36704;36912
6707;12495;13334;14173;30174
7;15230;27914;29045;34772
7;34113;35023
7554;20941;26322;31567;37938
51

25416;26500;28843;37019
97 1
09;4146;10369;10463;10532;11879;17400;19109;23736;24790;26232;26819;31689;35661;36885
5;10990;12116;12867;13426;14987;16133;18681;26863;31708

30
7479;8955;9058;13165;18084;22732;25448;29413;30972
3915;4032;4446;7342;11968;12850;22182;25738;34465;35598
2377;6395;18148;19700;22786;23153;29237;31155

0;25753;26909;29653;30215;36025
29945
2 6936
3;32660;35780;37871

1;27862;28544;29595;32340

5911;19610;20316;21662;36958

4;23602;27212;31326
33293
24;31868;31869
15
3;27392;30288

478;13936;17156;18791;21590;21764;23095;26015;29932
31168

73;37759

6633;6640;10374;22471;29382;35623
06;4950;6209;6966;11923;14271;16056;18499;18609;18855;19320;20422;22005;22465;26771;27276;2

27641;37518
3
4;15286;17916;22213
6538;8689;11343;12852;16444;23864;25015;26388;31920
1
5301;6508;7224;8455;9091;9303;9387;10112;11484;12060;13588;18342;18837;19087;19502;20016;22
4684;9179;12345;14334;15510;15830;17830;18809;19722;22802;23139;24805;25622;30255;32702;35
5565;7208;7391;20579;21668;35011;35093
4
7247;7919;12391;14104
2;18758

3;31670;38178
3
21;13354
42;38112
31
3;28984
14425;18858;25442;37982
91;37429
973;17011;17253
1

07

3;22611;26285
5
26370
16327
4
3;24087
93
29 1

4;17757;29733

5
47;32710;38262
2;25877;28645;38015
2;27209
3
49;27698;28599
12726;12843;13233;17492;18552;18853;20885;29289;31369;31379;34570;34687;35476
41;32505
74 215
688;2689;5037;6828;10524;17519;18206;19132;22242;25149;32283;34674
3;26816;27296

14480;14778;15746;18040;26588;27035;29260;30644;33478;34839;35731;36382;36575
32
33
10206;11097;11237;15835;16040;21274;21653;23081;24497;25036;27796;37341
77;31573
3;12711;14774;18273;22248;24144;30410
27;29150;37672
220;16580;16583;18565;18985;26207;27036;28469
4254;7421;8582;8790;14082;16580;16583;16632;16633;17829;18565;18985;22797;27036;31564;3821
4130;7468;14082;16440;16580;16583;17824;18565;18985;27036;28734;31566
2920;4128;4130;7468;14082;16580;16583;18565;18985;26775;27036;28734;31566
59;6195;23213;25250

90	267
90	302
23	372
90	287
3;4;45;46	55;215;239;312
3;4;45;46	73;233;257;330
3;4	420;604
4;45;46	233;257;330
4	257
4	257

32;25594
5;31009;37623

3
0;34179;34635
33;32241;32570;34180
3;37229

49;50 41;62
14

4905;9948;12037;15709;18284;21244;26785;27435;27589
31;32524;34282;34880
7;13897;19822;21874;22875;26216;28347
13;33784
3;27289
2;19582;23358;27155;37226
07
30
76;21732;23461
57
75;22577;24564
90

3;27875;35976;36053

2

17970

06
518;4965;5351;12772;22512;23486;23973;24689;25295;29017;31368;32072;34273;34507;36973
15;32900

10706;24529;26266

4
26;29556;36349;36969;37805
4472;9758;11722;12383;25398;27168
3
24
0;16596;20542
7718;9159;16489;31695;32002
12819;30085
31
70;23541

2;32139;37409
3;37082;38024
20213;37082

2112;3695;4912;5940;12816;15502;15504;17978;18357;18840;21315;23694;23894;24011;26291;2645
47

55;36421
1;14790;15187;16106;16218;18236;35287;36437
13128;15930;17256;20733;30499;31569;34172;38401
28086;29326
3;16625;24597;37567

37
10401;11518;15613;18941;18968;19690;20768;22249;25736;28019;28785;28963;28965;29668;35610;
11629;15526;19751;20462;20477;24573;31849;35691;36381;37812
34417
97

5
41;36231
14221

48;28066
8973;14891;16051;24632;25859;31997

37327

23;16641;20751;25479;27739
54
25
5;17100;21375;35925

3089;7761;8264;8513;9977;9978;10111;11837;12277;12364;15206;18527;24541;27644;28062;28211;3

73;21365;22801;26825;34263;36167
02;33011
13;28892

21448;30011
01;24283
2
51;28985;33129;34316

2;33388
2;22267;23555;37066
0;14821;15068;17215;22154;31003;34889
3997;6078;8535;11658;13033;13563;17798;20118;22612;23039;23212;23260;24612;26083;28805;316
53

4;17886;19216;21817;21822;21880;25056;27902;30820;31757;35642;36326;36414;37001;37076;3809
8;21826;21831;21896;24448;25401;27472;31261;33726;34666;35648;35651;36330;36859;36986;3810
779;21617;23857;27464;33759;35641
2

9;18512;30560;33044;34720;35149

7;16401;23810;26154;27301;28323;32417

5;22557
6587;18792
13148
52;25897;26227;26344
7;14784;18566;30406;34412;37163
55;31054;33629;36284
4;37109
56;36943
2;27867;35736

77

[illegible]

6185;7369;8768;9306;10003;14100;19369;21865;30937;31053;31857;33831;35035;37539
9318;9319;10948;15919;16135;19742;24070;27077;27637;30980;31165
[illegible];23895;25264;29471;30191
[illegible]2
[illegible]2

7898;29002;29792

[illegible];37811
[illegible]3

[illegible]
[illegible];35667

[illegible]622;29023;29502;33409;33460;34942;36865

935;15810;17576;18511;20281;21565;22362;23018;23330;25174;25926;26984;31195;31653;34312;34
05;17583;18511;21488;21564;22359;23019;23331;23839;25921;25969;26206;31195;31653;34780;351

7720;27827;32232;35168;36822

9;34505;37492

;31639;32752;33786;36386

[illegible];25430;26465;29905;32853;35962;38061;38100

687;29028;31004;31397;33690

;34483;35275

32427;36890;37230

52;30729;32727;34907

7694;18303;18545;18953;19806;20389;20574;21025;21760;24279;24470;24490;26983;27110;30165;3(

[illegible]56;19598;21145;23053;24945;25797;27204;28068;28534;29414;31914;32577;32619;32754;33355;34[illegible]

042;30196;30724;33615;35917

4;18962;20194;20206;20208;20471;20663;21011;21104;21555;22033;22070;24590;24786;26308;2633

[illegible]1;17659;18327;18708;19680;19692;20114;20212;20469;20701;20987;21019;21136;21472;22804;232[illegible]

4;31638;32919;33382;33384;34835;36207

3588;31286;31408;37602

17354;17641;23320;24402;25456;25920;27614;29248;30045;31437;34414;34499;35607;37388;38117

0;37298

6;19967;21554;21796;24200;28356;28386;28508;30809;31113

981;22927;23882;24140;25203;27091;29441;30899;32314;35257;37910;38162

21;35305;36609

3;37889;38257
0;13403;13517;13739;13762;19276;19818;20483;20868;21348;21495;21577;22782;23355;25872;2683

38;4680;4729;5230;5232;5505;5546;6507;6930;7117;7406;7833;9633;10077;10516;11274;12390;1267
;12675;15162;15180;15414;16344;16600;18879;20340;22357;22376;22701;22715;28160;29356;29360

3994;35666;37173;37174;38316

9

0;35130

;26710;26991;28127;28527;29571;29931;32847;33191;33752;35978

230;34165;35261;37960

210;14387;16656;19629;22424;24514;26963;28996;30930;32095;34951;37361;37365;37611;38462

3

389;22908;24219;25980;34713

91;29435;29755;30769;36063;36064;37963

3;31321;35698;36434;38336

;25304;25894;27473;27486;30745;31517;32448;32633;32766;33567;33711;33986;35431;35453;35846

;35870;37334

456;36184;36292

840;29970;33121;34845;35068;35079;36647;37381

)471;37088;37262;37317;38352
37304;38352

598;21630;21631;22143;22537;24631;24784;24921;26057;26172;28776;28784;29141;32813
223;21598;22621;24493;26810;27068;28017;33085

1353;22133;22764;23048;23294;24199;25098;27443;29252;29798;30231;30456;33062;33116;34479

6174

)566;30569;32468

006;14354;16313;16323;16961;17418;17880;18003;18741;19013;19021;19128;19913;19964;20780;21

)306;10798;10799;10861;11289;11665;11668;11670;11673;11674;11678;11680;12033;12087;12113;12

;28569;30752;30754;36161;36266;36565;37437;38127
;28259;28569;30752;30754;36266;36565;37437;38127

[illegible];18408;19089;19614;19748;20431;21077;21243;21263;21611;21741;24722;25450;25677;25783;25823

585;30952;31340;34217;36404;36406

38004

06;6107;6111;6113;6291;6392;7079;7760;8315;8813;9024;10656;11432;11809;11942;11978;12464;12[illegible]

35569

21129;21197;21313;21354;21600;22131;23189;24065;24766;26328;26411;28154;28768;29153;30200;

547;21618;26470;32887

9901;21026;21728;21774;25008;26094;26362;26573;27187;28653;29241;29335;30339;31891;31955;33

29116;31443;34042;34586

4417;24816;25951;29317;32589;34020;36934;37340;37546

041;24589;24926;25772;27216;28973;29687;29808;35239;38299

33003;34035

28370;29739;34383;36041;37990

2713;23271;26701;36845
177

3

7;29005;37233;37412

36177;37813;38001

32421;32592;35839

90;33520;34037;35386;36930

3
2

780;35145
38

)246;31989;32656;33533;33996;34603;34999;35526;36166;36961;37354;37356;37622

439;35330;35407;35604;35987;36255;37183;38301

9;26491;26828;27032;29122;29579;30176;31425;31828;32106;35912;36210;37384;37455

27;23727;24018;24213;24511;25756;25765;26118;26120;26398;28484;28830;28957;29050;29545;3257

2;27154;28123;28936;29032;31308;31454;34517;34565;35193;36758;36821;36979;37096;38183

'2;13034;13435;14688;14982;16355;16600;17222;17596;17709;18873;20340;20964;21333;21588;2235
;29375;29379;30639;31115;33695;33978;34372;34461;35240;35402;36217;36263;36618

;36200;37324;38428

867;23361;23501;24327;27539;28696;28826;28929;32322;32473;34608;36140;36924;36962;38309

2120;12128;12868;12869;12882;12888;12891;12895;12931;12944;12986;13164;13180;13229;14954;14

;25924;27337;28735;31272;33575;33621;34281;34339;35207;36138;38407

[illegible]79;12667;13721;13773;13840;15703;18210;18279;18491;18972;19963;20124;20127;20190;20199;20[illegible]

30553;31822;33053;33660;33675

3871;37490;38092

[illegible]6;33002;34840;36280;36634;37195;37948;38025

7;22370;22584;22698;23653;27294;29366;29496;29711;31185;31462;31491;32249;33526;33597;3360

4958;15073;15774;16397;16677;16779;16785;16823;16825;16833;16835;16851;16853;16856;16860;16

202;20303;21051;21110;21122;21194;21510;21698;21714;21724;22867;23293;24322;25874;26178;261

5;33976;34300;34404;34787;34822;34828;35713;35879;35981;36212;36459;36788;37160;37477;3748

[illegible]866;16868;16872;16873;16874;16878;18753;19025;19027;19052;19055;19246;20057;20077;20806;2[illegible]

183;26225;26288;26336;26488;26694;26698;26773;26833;26937;27045;27530;27768;27847;28547;28

6;37487;37942;38322;38434

)986;20999;21161;21334;22656;22754;22793;23131;23132;23138;23141;23217;29345;29448;29726;32

556;29012;29128;29616;29621;29772;29883;30454;33877;35087;35104;35761;35800;35805;35835;358

[illegible]350;33897;33900;33901;33905;33908;33910;33912;33925;33929;33937;33948;33955;33961;33965;33

373;35966;36379;36572;37015

[illegible]971;34060;34068;34072;34081;34088;34096;34101;34102;34111;34208;34324;34632;34656;34766;34

785;35062;35711;35898;36030;36033;36085;36142;36143;36188;36225
