# Supplementary material for: Proteomic Studies of the Mechanism of Cytotoxicity, Induced by Palytoxin on HaCaT Cells
Source: Toxins (Basel). 2022 Apr 10;14(4):269. doi: 10.3390/toxins14040269 (PMC9031313; doi:10.3390/toxins14040269)
Supplement: Supplementary file 1 [file toxins-14-00269-s001.zip › supplementary.pdf]

Supplementary Materials: Proteomic Studies of the Mechanism of Cytotoxicity Induced by Palytoxin on HaCaT Cells

Dingyuan Cheng, Bowen Deng, Qiling Tong, Siyi Gao, Boyi Xiao, Mengxuan Zhu, Ziyu Ren, Lianghua Wang and Minjuan Sun

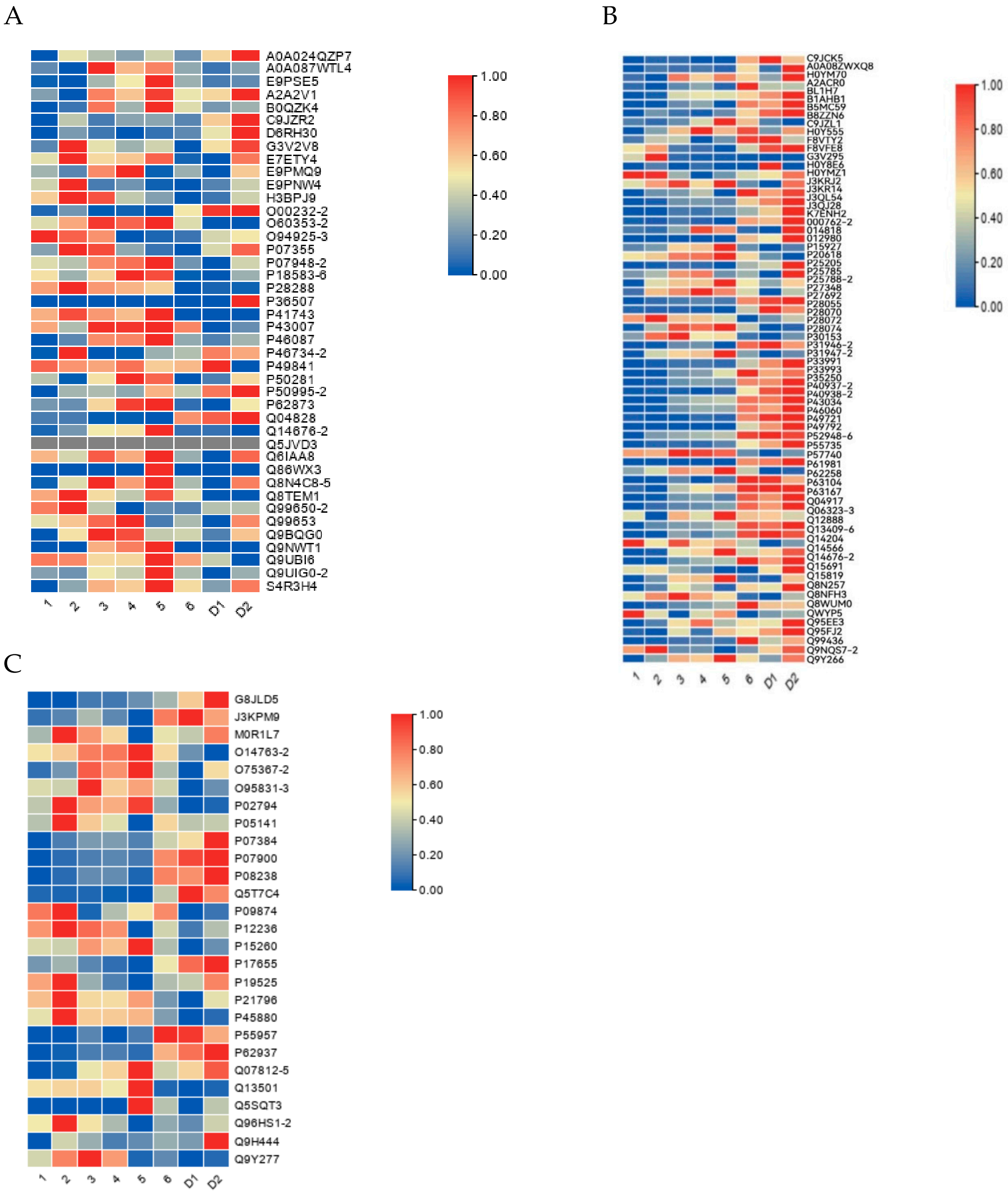

Figure S1. Cluster analysis of protein expression differences. (**A**) Cluster analysis of apoptosis-related proteins. (**B**) Cluster analysis of cell cycle-related proteins. (**C**) Cluster analysis of proteins related to cell necroptosis.

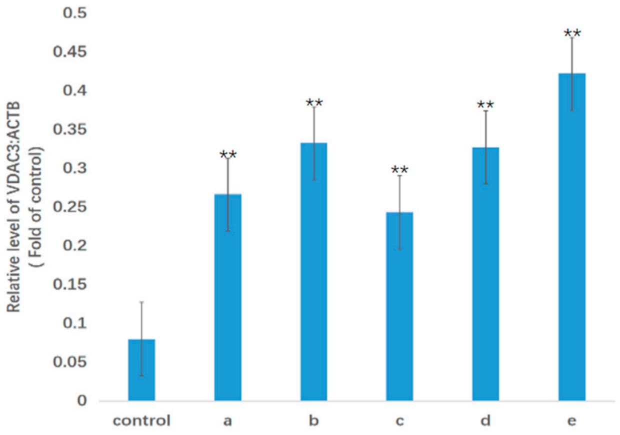

Figure S2. Gray scale analysis of Western blot. All experiments were repeated at least 3 times. Values are expressed as average ±SEM. ***P* < 0.01 vs. the control group. SPSS was used for one-way analysis of variance.

Table S1. Kinetic parameters between PTX and selected aptamer

Names	K_{on} (1/Ms)	K_{dis} (1/s)	K_d (M)	R^2
PTX-13	7.60×10^{5}	6.41×10^{-4}	8.43×10^{-10}	9.72×10^{-1}

The aptamer sequence quoted is 5′-ACCGACCGTGCTGGACTCAGGAGGAGGTGGTGGGGACTTTGCTTGTACTGGGCGCCCGGTTGAAACTATGAGCGAGCGAGCCTGGCG-3′[1].

Table S3. Proteomics related parameters.

Parameter	Value
Fixed modifications	Carbamidomethyl (C)
Include contaminants	True
PSM FDR	0.01
XPSM FDR	0.01
Protein FDR	0.01
Site FDR	0.01
Use Normalized Ratios for Occupancy	True
Min. peptide Length	7
Min. score for unmodified peptides	0
Min. score for modified peptides	40
Min. delta score for unmodified peptides	0
Min. delta score for modified peptides	6
Min. unique peptides	0
Min. razor peptides	1
Min. peptides	1
Use only unmodified peptides and	True
Modifications included in protein quantification	Oxidation (M); Acetyl (Protein N-term)
Peptides used for protein quantification	Razor
Discard unmodified counterpart peptides	True
Label min. ratio count	2

Use delta score	False
iBAQ	False
iBAQ log fit	False
Match between runs	True
Matching time window [min]	0.7
Alignment time window [min]	20
Find dependent peptides	False
Include contaminants	True
Advanced ratios	True
Second peptides	True
Stabilize large LFQ ratios	True
Separate LFQ in parameter groups	False
Require MS/MS for LFQ comparisons	True
Calculate peak properties	False
Main search max. combinations	200
Advanced site intensities	True
LFQ norm for sites and peptides	False
Write msScans table	True
Write msmsScans table	True
Write ms3Scans table	True
Write allPeptides table	True
Write mzRange table	True
Write pasefMsmsScans table	True
Write accumulatedPasefMsmsScans table	True
Max. peptide mass [Da]	4600
Min. peptide length for unspecific search	8
Max. peptide length for unspecific search	25
Razor protein FDR	True
Disable MD5	False
Max mods in site table	3
Match unidentified features	False
MS/MS tol. (FTMS)	0.1 Da
Top MS/MS peaks per Da interval. (FTMS)	12
Da interval. (FTMS)	100
MS/MS deisotoping (FTMS)	True
MS/MS deisotoping tolerance (FTMS)	10
MS/MS deisotoping tolerance unit (FTMS)	ppm
MS/MS higher charges (FTMS)	True
MS/MS water loss (FTMS)	True
MS/MS ammonia loss (FTMS)	False
MS/MS dependent losses (FTMS)	True
MS/MS recalibration (FTMS)	False
MS/MS tol. (ITMS)	0.5 Da
Top MS/MS peaks per Da interval. (ITMS)	8
Da interval. (ITMS)	100
MS/MS deisotoping (ITMS)	False
MS/MS deisotoping tolerance (ITMS)	0.15

MS/MS higher charges (ITMS)	True
MS/MS water loss (ITMS)	True
MS/MS ammonia loss (ITMS)	True
MS/MS dependent losses (ITMS)	True
MS/MS recalibration (ITMS)	False
MS/MS tol. (TOF)	40 ppm
Top MS/MS peaks per Da interval. (TOF)	10
Da interval. (TOF)	100
MS/MS deisotoping (TOF)	True
MS/MS deisotoping tolerance (TOF)	0.01
MS/MS higher charges (TOF)	True
MS/MS water loss (TOF)	True
MS/MS ammonia loss (TOF)	True
MS/MS dependent losses (TOF)	True
MS/MS recalibration (TOF)	False
MS/MS tol. (Unknown)	0.5 Da
Top MS/MS peaks per Da interval. (Unknown)	8
Da interval. (Unknown)	100
MS/MS deisotoping (Unknown)	False
MS/MS deisotoping tolerance (Unknown)	0.15
MS/MS higher charges (Unknown)	True
MS/MS water loss (Unknown)	True
MS/MS ammonia loss (Unknown)	True
MS/MS dependent losses (Unknown)	True
MS/MS recalibration (Unknown)	False

References

1. Gao, S.; Zheng, X.; Hu, B.; Sun, M.; Wu, J.; Jiao, B.; Wang, L. Enzyme-linked, aptamer-based, competitive biolayer interferometry biosensor for palytoxin. *Biosens. Bioelectron.* **2017**, *89*, 952–958.
